
JOURNAL INFORMATION
==============================
NLM Title Abbreviation: Hemasphere
Journal ID: Hemasphere
NLM Journal ID: 101740619
Journal ID: HEM3

HemaSphere

EISSN: 2572-9241
Publisher: Wiley

ARTICLE INFORMATION
==============================
PMCID: PMC13254486
DOI: 10.1002/hem3.70419
Article ID: HEM370419
Article version: 1
Subjects: Abstract Book, Abstract Book, Orals

Plenary Abstracts Session & Oral Presentations

Electronic publication date: 2026 Jun 10
Publication date: 2026 Jun
Volume: 10
Issue: Suppl 1
Electronic Location ID: e70419
Copyright: © 2026 The Author(s). HemaSphere published by John Wiley & Sons Ltd. on behalf of European Hematology Association.
License: This is an open access article under the terms of the http://creativecommons.org/licenses/by/4.0/ License, which permits use, distribution and reproduction in any medium, provided the original work is properly cited.
License URL: https://creativecommons.org/licenses/by/4.0/

Figure count: 0
Table count: 0
Page count: 570

source-schema-version-number: 2.0
cover-date: June 2026
details-of-publishers-convertor: Converter:WILEY_ML3GV2_TO_JATSPMC version:6.8.0 mode:remove_FC converted:11.06.2026

==============================

eha

HemaSphere

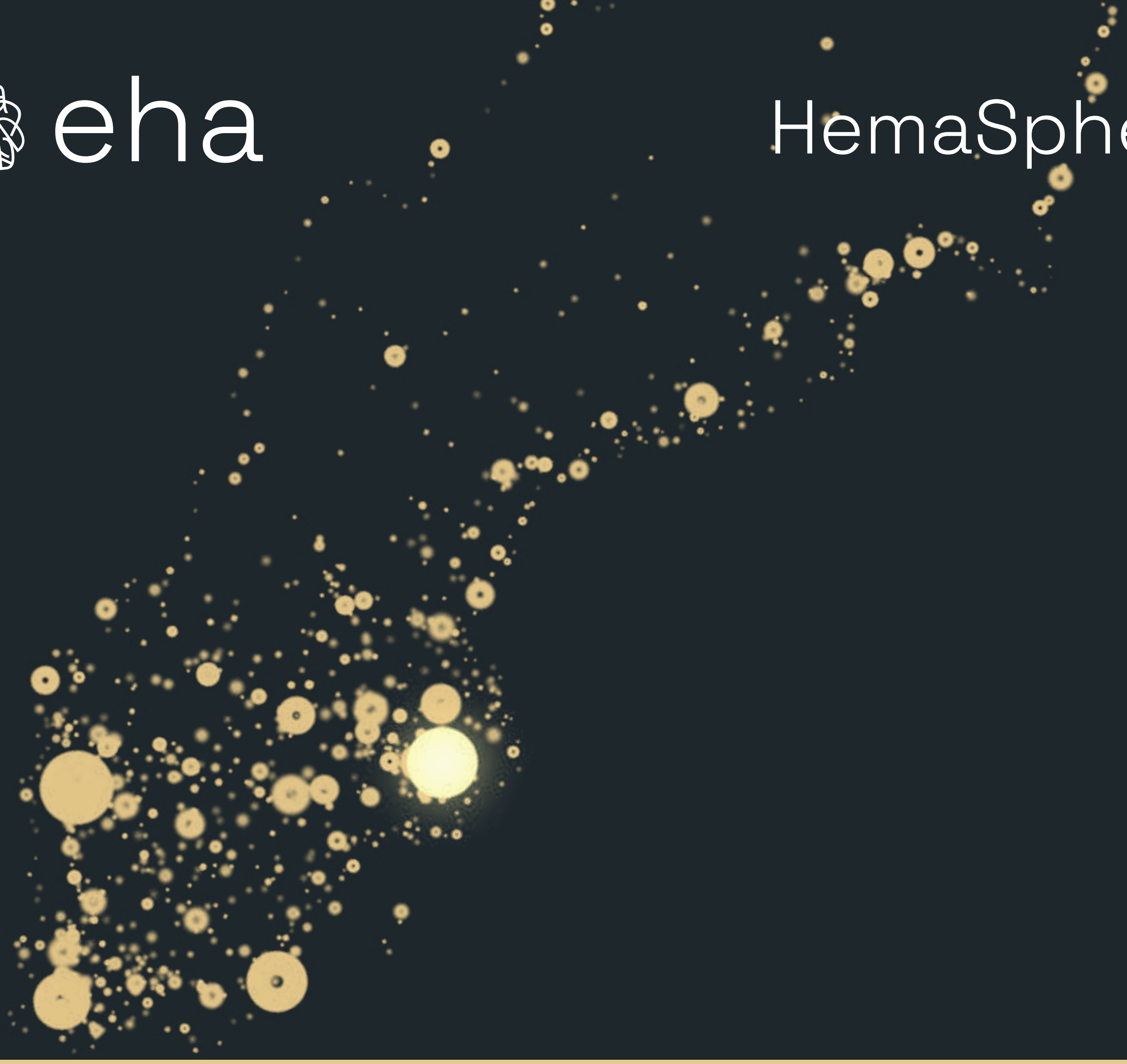

### Abstract Book

EHA2026
Congress

June 11-14
Stockholm, Sweden

Volume 10
Issue S1

www.hemaspherejournal.com

Medical Education

### Attend MM & CLL symposia at EHA2026!

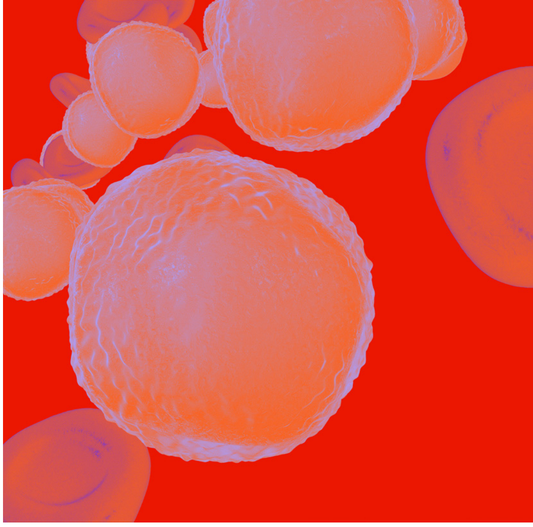

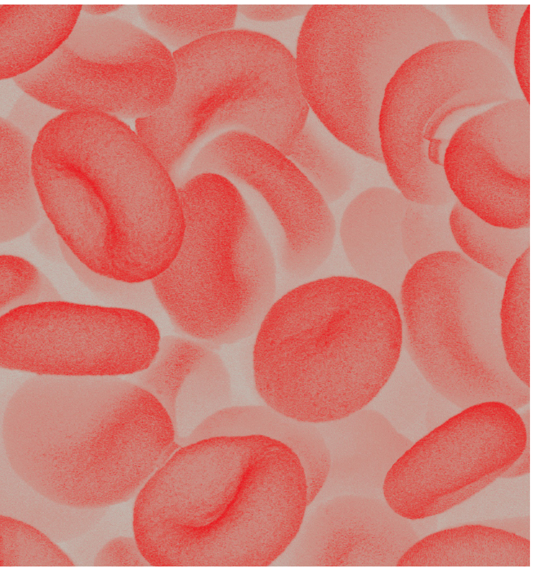

On **Thursday 11th, Friday 12th and Saturday 13th June**, follow the red thread with us throughout an exciting programme of satellite symposia supported by Johnson & Johnson and hosted by the esteemed Symposium Committee.

Prepare your burning questions for the expert panel and get ready to participate in stimulating Q&A sessions and interactive polling activities.

Thursday 11th, Friday 12th and Saturday 13th June 2026
10:00–11:30 CEST
Nobel Hall, Stockholmsmässan, Stockholm

Thursday 11th June
10:00–11:30 CEST

Weaving the red thread towards cure in newly diagnosed multiple myeloma (NDMM)

Prof. María-Victoria Mateos (Chair & Symposium Committee)
Prof. Philippe Moreau (Symposium Committee)
Prof. Paula Rodriguez-Otero
Dr Charlotte Pawlyn

Friday 12th June
10:00–11:30 CEST

Following the red thread in multiple myeloma (MM): aligning innovation with clinical practice

Prof. María-Victoria Mateos (Chair & Symposium Committee)
Dr Rakesh Popat (Symposium Committee)
Prof. Elena Zamagni
Dr Roberto Mina

Saturday 13th June
10:00-11:30 CEST

Following the red thread of evidence: guiding optimal decision-making in first-line (1L) chronic lymphocytic leukaemia (CLL)

Prof. Martin Trepel (Symposium Committee)
Dr Alessandra Tedeschi (Symposium Committee)
Dr Anna Panovská (Co-chair)
Prof. Paolo Sportoletti (Co-chair)
Dr José-Ángel Hernández-Rivas

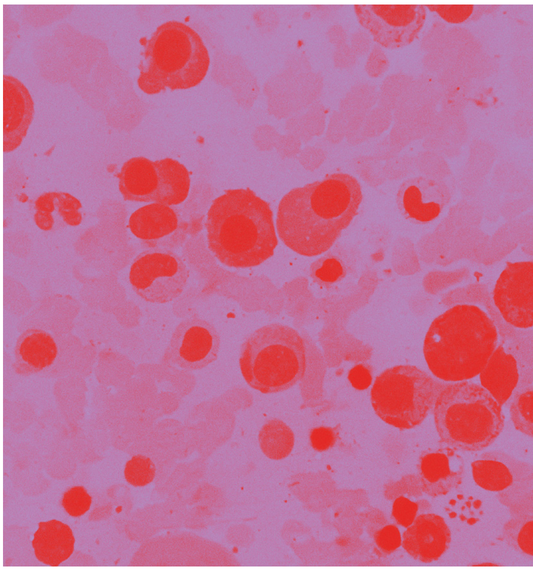

*This symposium is organised and sponsored by Johnson & Johnson and is not part of the official EHA scientific programme.*

Johnson&Johnson

Organised and supported by Janssen Pharmaceutica NV,
a Johnson & Johnson company
Janssen Pharmaceutica NV, Turnhoutseweg 30, 2340 Beerse Belgium
Copyright © 2026 Janssen Pharmaceutica NV
Job code: EM-199756 Date of prep: May 2026

31st Congress of the
European Hematology Association

EHA2026 Annual Congress Edition
June 2026

### ABSTRACT BOOK

### SECTION 01

**Author Information**

***HemaSphere*** is an open access journal powered by the European Hematology Association and dedicated to supporting hematology patient care, research, and education worldwide.

This Abstract Book of the European Hematology Association's 31st Annual Congress - EHA2026 Congress - is published as a supplement of *HemaSphere* and available online at www.hemaspherejournal.com.

Please read our author guidelines for preparing and submitting articles. Manuscripts must be submitted online via the Research Exchange submission portal.

**Article Processing Charges (APC)**

| Article types | EHA members | Non-members |
|---|---|---|
| Tier 1: Original articles, review articles, guidelines | 10%* | €2500 |
| Tier 2: Letters, perspectives | 10%* | €1500 |
| Tier 3: Editorials, comments, HemaTopics, HemaPolicy | Free | Free |
| All invited manuscripts** | Free | Free |

*2026 Promotional Discounts on APCs: 10% discount for EHA members who are lead or co-authors on an accepted submission
**APCs for all contributions on *invitation* by the Editors will be waived

**Copyright Information**
(Online) ISSN: 2572-9241
© 2026 The Author(s). *HemaSphere* published by John Wiley & Sons Ltd on behalf of European Hematology Association. This is an open access Abstract Book distributed under the Attribution-NonCommercial-NoDerivs (CC BY-NC-ND), which allows third parties to download the articles and share them with others as long as they credit the author and the Abstract Book, but they cannot change the content in any way or use them commercially.

**Rights and Permissions**
For reprints and to order copies of the Abstract Book, please email commercialreprints@wiley.com. Questions regarding permissions should be addressed to hemasphere@ehaweb.org.

**Abstract Book Citations:** Authors, Title, *HemaSphere*, 2026;10:(S1):pages.

**DOIs:**
Oral section: 10.1002/hem3.70151
Poster section: 10.1002/hem3.70152
Publication only section: 10.1002/hem3.70153

**Disclaimer**
Articles published in the journal *HemaSphere* exclusively reflect the opinions of the authors. The authors are responsible for all content in their abstracts including accuracy of the facts, statements, citing resources, etc.

### Editorial Board

### Editor in Chief
**Professor Jan Cools**
*VIB-KU Leuven Center for Cancer Biology, Belgium*

### Deputy Editor in Chief
**Professor Claire Harrison**
*Guy's and St Thomas' NHS Foundation Trust, London, United Kingdom*

### Associate Editors
Simone Ferrero, *University of Torino, Italy*
Adele Fielding, *University of York, Hull York Medical School, York, United Kingdom*
Simon Hallam, *St. Bartholomew's Hospital, Barts Health NHS Trust, London, United Kingdom*
Steffen Koschmieder, *University Medical Center RWTH, Aachen, Germany*
Riitta Lassila*, University of Helsinki, Helsinki, Finland*
Marek Mraz, *CEITEC Masaryk University and University Hospital Brno, Czech Republic*
Paula Rodríguez Otero, *Clínica Universidad de Navarra, Spain*
Jürg Schwaller, *University Hospital Basel, Switzerland*
Francesca Vinchi, *Emory University School of Medicine, Atlanta, Georgia, United States*

### Scientific Editors
Charles de Bock, *Children's Cancer Institute, Sydney, Australia*
Stephen Hibbs, *Queen Mary University of London, United Kingdom*
Mandy Lauw, *Erasmus University MC, Rotterdam, The Netherlands*
Lauren Merz, *University of Michigan, Ann Arbor, Michigan, United States*
Edeghonghon Olayemi, *Vancouver Coastal Health (Richmond Hospital Site), Richmond, British Columbia, Canada*

### Statistics Editor
Robert Hills, *University of Oxford, United Kingdom*

### Editorial Manager, European Hematology Association (EHA)
Jessica Mastrodomenico, *EHA/HemaSphere, The Hague, The Netherlands*

### Editorial Board
Frederic Baron, *University of Liège, GIGA Institute, Liège, Belgium*
Grzegorz Basak, *Medical University of Warsaw, Poland*
Andrea Biondi, *University of Milano-Bicocca, Italy*
Jonathan Bond, *University College Dublin, Ireland*
Dominique Bonnet, *The Francis Crick Institute, London, United Kingdom*
Arndt Borkhardt, *University of Düsseldorf, Germany*
Monika Brüggemann, *University Hospital Schleswig-Holstein, Kiel, Germany*
David Bryder, *Lund University, Sweden*
Ryan Cassaday, *Fred Hutchinson Cancer Center, Seattle, Washington, United States*
Giancarlo Castaman, *Azienda Ospedaliera Universitaria Careggi, Florence, Italy*
Anna Castleton, *The Christie Hospital NHS Foundation Trust, Manchester, United Kingdom*
Nicholas Chiorazzi, *Northwell Health, Manhasset, New York, United States*
Emmanuelle Clappier, *Hôpital Saint-Louis, Paris, France*
Paolo Corradini, *Università degli Studi di Milano, Italy*
Lucia De Franceschi, *University of Verona, Italy*
Kim De Keersmaecker, *University of Leuven, Belgium*

Marianne De Montalembert, *Hôpital Universitaire Necker-Enfants Malades, Paris, France*
Michel Delforge, *Leuven Cancer Institute, Leuven, Belgium*
Catherine Diefenbach, *Perlmutter Cancer Center, New York, United States*
Carlos Fernández De Larrea, *Hospital Clínic Barcelona, Spain*
Francesca Gay, *University of Turin, Italy*
Paolo Ghia, *Università Vita Salute San Raffaele, Milan, Italy*
Domenico Girelli, *Universita degli Studi di Verona, Italy*
Florian Grebien, *University of Veterinary Medicine Vienna, Austria*
Hildegard Greinix, *Medical University of Graz, Austria*
Kirsten Grønbæk, *Rigshospitalet, Copenhagen, Denmark*
Iqbal Hamza, *University of Maryland School of Medicine, Baltimore, Maryland, United States*
Florian Heidel, *University Medical Center Greifswald, Germany*
Olaf Heidenreich, *Princess Máxima Center, Utrecht, The Netherlands*
Brian Huntly, *University of Cambridge, United Kingdom*
Elaine Jaffe, N*ational Cancer Institute, Bethesda, Maryland, United States*
Mats Jerkeman, *Lund University, Sweden*
Efstathios Kastritis, *National and Kapodistrian University of Athens, Greece*
Léon Kautz, *Inserm, Toulouse, France*
David Kent, *University of York, United Kingdom*
Ulf Klein, *University of Leeds, United Kingdom*
Joost Kluiver, *Universitair Medisch Centrum Groningen, The Netherlands*
Daniela Krause, *University of Frankfurt, Germany*
Nicolaus Kröger, *University Hospital Hamburg-Eppendorf, Germany*
Steven Lane, *QIMR Berghofer Medical Research Institute, Australia*
Frank Leebeek, *Erasmus MC Cancer Institute, Rotterdam, The Netherlands*
Florent Malard, *INSERM, Saint-Antoine Hospital, Paris, France*
Maria-Victoria Mateos, *University of Salamanca, Spain*
Francesco Maura, *Memorial Sloan Kettering Cancer Center, New York, United States*
Simon Mendez-Ferrer, *University of Cambridge, United Kingdom*
Thomas Mercher, *Gustave Roussy Institute, Villejuif, France*
Martina Muckenthaler, *Heidelberg University Hospital, Heidelberg, Germany*
Cesar Nombela Arrieta, *University Hospital Zurich, Switzerland*
James O'Donnell, *Royal College of Surgeons in Ireland, Dublin, Ireland*
Kostas Pantopoulos, *McGill University, Montreal, Quebec, Canada*
Jakob Passweg, *University of Basel, Switzerland*
Heike Pfeifer, *University Hospital Frankfurt, Germany*
Marc Raab, *Heidelberg University Hospital, Heidelberg, Germany*
Marc Raaijmakers, *Erasmus Medical Center Cancer Institute, Rotterdam, The Netherlands*
Alejo Rodriguez-Fratelli, *Institute for Research in Biomedicine, Barcelona, Spain*
Richard Rosenquist, *Karolinska Institutet, Stockholm, Sweden*
Deepa Sampath, *MD Anderson Cancer Center, Houston, Texas, United States*
Rebekka Schneider, *Erasmus MC, Rotterdam, The Netherlands*
Marie Scully, *University College London Hospitals, NHS Foundation Trust, London, United Kingdom*
Kostas Stamatopoulos, *CERTH, Centre for Research and Technology Hellas, Thessaloniki, Greece*
Anna Maria Sureda Balari, *Institut Català d'Oncologia, Barcelona, Spain*
Evangelos Terpos, *National and Kapodistrian University of Athens, Greece*
Alexandre Theocharides, *University Hospital Zürich, Switzerland*
Jan Trka, *University of Prague, Czech Republic*
Karen Vanhoorelbeke, *KU Leuven, Laboratory for Thrombosis Research, Kortrijk, Belgium*
Carlo Visco, *University of Verona, Italy*
Clive Zent, *University of Rochester Medical Center, Rochester, New York, United States*

eha

### Borderless Hematology

### Join us, and let's make a difference together.

Welcome to a passionate, global network that improves patient care, advances scientific discovery, and drives innovation in hematology.

Here, every voice is heard, and every contribution matters. It's a place where we learn, inspire, and support each other, facing challenges and celebrating victories as one.

**Learn more about our community**

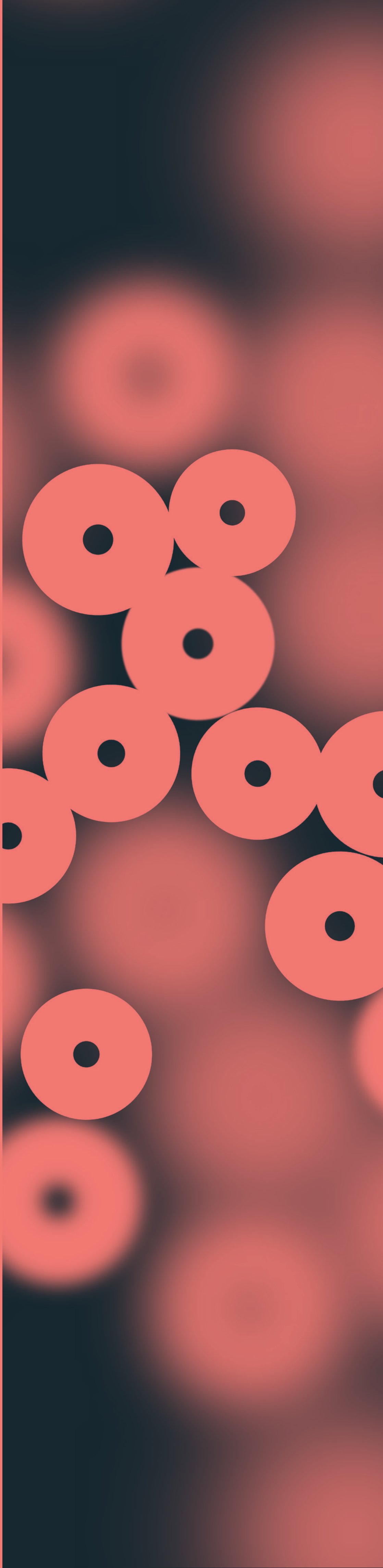

### EHA Board and Organization EHA2026 Congress

### Executive Board
K Döhner, *Germany* (President)
A Almeida, *Portugal* (Past President)
B Huntly, *United Kingdom* (President Elect)
F Leebeek, *The Netherlands* (Treasurer)
K Grønbæk, *Denmark* (Secretary)
M Alberich Jorda, *Czechia* (General member)

### Councillors
D Bonnet, *United Kingdom*
J Delgado, *Spain*
L De Franceschi, *Italy*
M Guenova, *Bulgaria*
M Hudecek, *Germany*
S Leppä, *Finland*
S Mustjoki, *Finland*
E Terpos, *Greece*
J Trka, *Czechia*

### EHA2026 Congress Scientific Program Committee
M Alberich Jorda, *Czechia* (Chair)
D Bonnet, *United Kingdom* (Chair Elect)
M Dreyling, *Germany* (Past Chair)
M Carlsten, *Sweden* (Local representative)
V Brousse, *France*
V Buxhofer-Ausch, *Austria*
A Cvejic, *Denmark*
M D'Agostino, *Italy*
K Giannopoulos, *Poland*
M Gomes da Silva, *Portugal*
J Hauer, *Germany*
D Hodson, *United Kingdom*
S Lehmann, *Sweden*
S Leppä, *Finland*
T Masszi, *Hungary*
K Meijer, *The Netherlands*
M Morado, *Spain*
C Papayannidis, *Italy*
D Rea, *France*
S Šaulytė Trakymienė, *Lithuania*
B Skopec, *Slovenia*
E Solomou, *Greece*
C So-Osman, *The Netherlands*
G Vassiliou, *United Kingdom*
MT Voso, *Italy*
G Weiss, *Austria*

### EHA2026 Congress SPC Advisory Board
L Arranz, *Norway*
Y Aydinok, *Türkiye*
C Bödör, *Hungary*
M Buschbeck, *Spain*
J Caers, *Belgium*
M Della Porta, *Italy*
M Doubek, *Czechia*
F Fioredda, *Italy*
C Fox, *United Kingdom*
S Garciaz, *France*
M Gavriatopoulou, *Greece*
E Giné, *Spain*
A Glenthøj, *Denmark*
C Halsey, *United Kingdom*
V Helgason, *United Kingdom*
C João, *Portugal*
M Martello, *Italy*
J O'Sullivan, *Ireland*
F Pirenne, *France*
C Röllig, *Germany*
S Roulland, *France*
E Sala, *Germany*
M Sobas, *Poland*
A Stathis, *Switzerland*
J Thaler, *Austria*
M Themeli, *The Netherlands*
A Theocharides, *Switzerland*
C Touzeau, *France*
E van Beers, *The Netherlands*
J Werner Hansen, *Denmark*
P Woll, *Sweden*

### EHA2026 Congress Scientific Program Committee - Observers
N Borges, *United Kingdom (Young EHA Committee)*
S Nier, *Switzerland (Patient Advocacy Committee)*

### EHA Executive Office
Koninginnegracht 12b
2514 AA The Hague
The Netherlands
Tel: +31 (0)70 345 55 63
Fax: +31 (0)70 392 36 63
E-mail: info@ehaweb.org
Website: www.ehaweb.org

**Abstract Reviewers**
EHA would like to thank the following experts for their time and efforts reviewing abstracts for this Congress

R Advani, United States
A Agathangelidis, Greece
I Age Kos, Germany
J Aguadé-Gorgorió, Spain
S Akyol, Türkiye
A Al-Ani, Iraq
A Alanya, Belgium
J Alberge, France
M Alberich Jorda, Czechia
A Alhajahjeh, Jordan
A Almeida, Portugal
M Almowaled, Saudi Arabia
A Anastasiadi, Greece
A Anastasopoulou, Greece
A Aprile, Italy
S Aras Gürcan, Türkiye
A Armatys, Poland
L Arranz, Norway
F Auer, Germany
I Aurer, Croatia
F Awan, United States
Y Aydinok, Türkiye
E Azzoni, Italy
A Bachev, Bulgaria
E Bachy, France
A Bacquelaine Veloso, Belgium
D Baggio, United Kingdom
A Balduini, Italy
A Barrett, Ireland
A Baruchel, France
K Basso, United States
B Bátai, Hungary
J Batinic, Croatia
A Bazarbachi, Lebanon
S Bea, Spain
R Beekman, Spain
F Beier, Germany
C Bellodi, Denmark
S Bertoli, France
A Bigas, Spain
M Bishton, United Kingdom
N Bizymi, Greece
S Bjelosevic, United States
J Blatny, Czechia
I Bodo, Hungary
C Bödör, Hungary
D Bogdanic, Croatia
C Bonini, Italy
D Bonnet, United Kingdom
F Borzooee, Canada
F Bosch, Spain
C Brierley, United Kingdom
S Bringhen, Italy
J Broseus, France
V Brousse, France
P Bruch, Germany
T Brümmendorf, Germany
V Bücklein, Germany
B Burak, United States
M Buschbeck, Spain
C Buske, Germany
V Buxhofer-Ausch, Austria
J Caers, Belgium
M Callanan, France
V Camus, France
A Candoni, Italy
J Carda, Portugal
B Cardoso, Portugal
M Carlsten, Sweden
M Carpenedo, Italy
M Carter, United Kingdom
F Castagnetti, Italy
H Cavé, France
G Cengiz, Türkiye
C Chabannon, France
T Chatzikonstantinou, Greece
G Cheminet, France
X Chen, China
A Chiappella, Italy
P Christoforou, Greece
G Collins, United Kingdom
A Condoluci, Switzerland
E Crisà, Italy
J Croden, Canada
N Cross, United Kingdom
N Crump, United Kingdom
M Csanadi, Hungary
S Cuartero, Spain
A Cvejic, Denmark
M D'Agostino, Italy
F Damm, Germany
A Davies, United States
S de Botton, France
L de Franceschi, Italy
S de la Iglesia, Spain
C de Larrea, Spain
P de Nully Brown, Denmark
Z DeFilipp, United States
A Degulys, Lithuania
S Delicou, Greece
M Della Porta, Italy
O Demska, Poland
T Devos, Belgium
A Di Rocco, Italy
M Dickinson, Australia
A Dimitrijevic, Denmark
M Dimopoulos, Greece
A Ditadi, Italy
M Długosz-Danecka, Poland
M Doubek, Czechia
M Dreyling, Germany
Ľ Drgoňa, Slovakia
R Duléry, France
A Duminuco, Italy
B Eichhorst, Germany
T Eipe, India

A Eiring, United States
I El Serafi, United Arab Emirates
G Enblad, Sweden
T Eyre, United Kingdom
T Facon, France
B Faganel Kotnik, Slovenia
A Fagnan, France
S Faict, Belgium
L Falchi, United States
M Farina, Italy
B Fattizzo, Italy
G Faulhaber, New Zealand
E Fermo, Italy
H Feys, Belgium
W Fibbe, The Netherlands
F Fioredda, Italy
R Fiskesund, Sweden
S Fleming, Australia
F Forconi, United Kingdom
S Forté, Canada
D Fotiou, Greece
C Fox, United Kingdom
v Frank, The Netherlands
K Freson, Belgium
E Fronkova, Czechia
S Gandhi, United Kingdom
V Garcia, Spain
S Garciaz, France
M Gavriatopoulo, Greece
E Genesca, Spain
H Ghesquières, France
A Ghiaur, Romania
K Giannopoulos, Poland
A Gimbert, Spain
E Giné, Spain
V Gjylameti Cavolli, Kosovo
S Glavey, Ireland
H Gleitz, The Netherlands
A Glenthøj, Denmark
C Glytsou, United States
S Gohil, United Kingdom
A Gołos, Poland
M Gomes da Silva, Portugal
M Gomez Alvarez, Spain
F Gonzales, Switzerland
A Gozzetti, Italy
M Griffin, United Kingdom
E Grignano, Canada
A Gullà, Italy
L Haaksma, The Netherlands
S Haebe, Germany
C Halsey, United Kingdom
E Hatjiharissi, Greece
J Hauer, Germany
O Havranek, Czechia
J Heger, Germany
S Heibl, Austria
A Heine, Austria
J Helby Petersen, Denmark
G Held, Germany
V Helgason, United Kingdom
C Herbaux, France
D Hodson, United Kingdom
I Horvat, Croatia
K Hosokawa, Japan
S Hrabovsky, Czechia
M Hultcrantz, United States
A Hundebøll Torpe, Denmark
M Hutchings, Denmark
G Iacoboni, Spain
V Iacomi, Moldova
G Illerhaus, Germany
J Infante, Portugal
I Ionita, Romania
T Ionova, Russia
M Iqbal, United States
R Irimia, Romania
S Iyengar, United Kingdom
R Jaeger, Austria
F Jardin, France
C João, Portugal
A Jonasova, Czechia
A Jurak Begonja, Croatia
W Jurczak, Poland
B Kalaja, Albania
I Kalashnikov, Finland
T Kalina, Czechia
D Karapınar, Türkiye
A Karow, Austria
I Karsten, Germany
A Kater, The Netherlands
K Keeshan, United Kingdom
W Kern, Germany
C Khadanpour, Germany
A Khan, United Kingdom
A Kittai, United States
R Klaassen, Canada
M Klimiankou, Germany
S Klobuch, The Netherlands
C Koch, Switzerland
S Koschmieder, Germany
F Köseoğlu, Türkiye
A Kramer, The Netherlands
I Krečak, Croatia
V Krishnan, Singapore
M Ku, Australia
M Kulis, Spain
K Kuo, Canada
J Kuruvilla, Canada
H Kuusanmäki, Finland
L Lanikov, Czechia
M Lauw, The Netherlands
S Lehmann, Sweden
D Lekovic, Serbia
J Lengefeld, Sweden
S Leppä, Finland
K Lewandowski, Poland
K Liang, Belgium
H Lindqvist, Sweden
P Ljungman, Sweden
C Lo Celso, United Kingdom
L Lobo de Figueiredo Pontes, Brazil
O Lohi, Finland
C Loke, United Kingdom

C Lopez, United Kingdom
Y López García, Mexico
A Lopez Guillermo, Spain
M Lordkipanidze, Canada
S Loughran, United Kingdom
M Ludvigsen, Denmark
S Luminari, Italy
M Lupo-Stanghellini, Italy
F Lussana, Italy
E Magalhaes Rego, Brazil
M Mahevas, France
A Maiques, Spain
P Malandrakis, Greece
S Manier, France
A Marin Niebla, Spain
O Marques, Portugal
M Martello, Italy
A Martín García-Sancho, Spain
I Martin-Subero, Spain
M Massaia, Italy
T Masszi, Hungary
E McCormack, Norway
H Medyouf, Germany
K Meijer, The Netherlands
M Merz, Germany
M Metzler, Germany
M Miano, Italy
M Minnema, The Netherlands
R Moia, Italy
M Morado, Spain
A Mosquera Orgueira, Spain
D Mott, United Kingdom
I Motta, Italy
C Mueller-Tidow, Germany
A Müller, Austria
A Mussetti, Spain
M Myllymäki, Finland
N Narayan, United Kingdom
R Nelles, Australia
S Nier, Switzerland
S Nier, Switzerland
M Nijland, The Netherlands
O Nnodu, Nigeria
J Nordlund, Sweden
F Onida, Italy
R Origa, Italy
M Ormhoj, Denmark
J O'Sullivan, United Kingdom
C Owen, Canada
I Pagani, Australia
L Pagano, Italy
J Panse, Germany
H Papadaki, Greece
C Papayannidis, Italy
A Pavlovsky, Argentina
E Payne, United Kingdom
Z Peric, Croatia
F Peters, Austria
A Piekarska, Poland
F Piel, United Kingdom
L Pinczes, Hungary
F Pirenne, France
M Pirosa, Switzerland
A Plesa, France
K Plevova, Czechia
I Plo, France
C Pontikoglou, Greece
R Popat, United Kingdom
K Porkka, Finland
L Pour, Czechia
I Preloznik Zupan, Slovenia
A Puissant, France
P Quarello, Italy
A Queirós, Portugal
L Quek, United Kingdom
R Rääty, Finland
M Rab, The Netherlands
J Radocha, Czechia
S Ragaini, Italy
D Rea, France
A Reinisch, Austria
M Reiterová, Czechia
R Renella, Switzerland
S Richardson, United Kingdom
L Rigacci, Italy
I Roberts, United Kingdom
P Rodriguez, Argentina
E Roldan Galvan, United Kingdom
C Röllig, Germany
A Romano, Italy
A Roque, Portugal
S Roulland, France
A Rovó, Switzerland
E Ruggiero, Italy
N Sabljic, Serbia
E Sala, United States
I Salaverria, Spain
E Salido, Spain
J Salmanton-García, Germany
V Sampath, Singapore
M Sanchez, Spain
J Sanz, Spain
A Sarmento, Portugal
B Sarni Sanches, Brazil
J Sarry, France
T Sauer, Germany
S Šaulytė Trakymienė, Lithuania
K Savage, Canada
A Schavgoulidze, France
S Scheding, Sweden
M Schemionek, Germany
C Schinke, United States
L Schmalbrock, United States
C Schneider, United States
P Schneider, United States
A Schurich, United Kingdom
V Šeda, Czechia
L Sehn, Canada
D Serruggia, Austria
C Skedgel, United Kingdom
J Skokowa, Germany
B Skopec, Slovenia
A Slomka, Poland

L Smit, The Netherlands
M Sobas, Poland
E Solomou, Greece
C So-Osman, The Netherlands
T Standal, Norway
A Stathis, Switzerland
U Steidl, United States
F Stella, Italy
M Stelljes, Germany
Y Stepanishyna, Belgium
T Stoklosa, Poland
J Stuchly, Czechia
A Sureda, Spain
M Syed, United Kingdom
M Szopa, Poland
Ł Szukalski, Poland
A Tatic, Romania
E Tausch, Germany
P te Boekhorst, The Netherlands
J Thaler, Austria
M Themeli, The Netherlands
A Theocharides, Switzerland
M Tobiasson, Sweden
D Todorieva, Bulgaria
C Tomuleasa, Romania
T Toubai, Japan
C Touzeau, France
A Trinquand, Ireland
J Trka, Czechia
C Tsioutis, Cyprus
V Turati, Sweden
A Turki, Germany
T Tzenou, Greece
A Ukmeraj, Kosovo
E van Beers, The Netherlands
M Van Duin, The Netherlands
R van Wijk, The Netherlands
G Vassiliou, United Kingdom
L Velten, Spain
P Verhamme, Belgium
K Vettenranta, Finland
A Viardot, Germany
C Visco, Italy
M von Lilienfeld-Toal, Germany
B von Tresckow, Germany
A Voskanyan, Armenia
MT Voso, Italy
E Wagner-Drouet, Germany
Y Wang, Taiwan
U Wartiovaara-Kautto, Finland
V Wauters, Argentina
T Weber, Germany
G Weiss, Austria
J Werner Hansen, Denmark
A Wierzbowska, Poland
M Wilson, United Kingdom
R Winkler, Spain
A Woelfler, Austria
P Woll, Sweden
T Wrobel, Poland
N Yasin, Malaysia
U Yilmaz, Türkiye
S Yilmaz, Türkiye
L Zamora, Spain
S Zanwar, United States
J Zaugg, Switzerland
R Zeiser, Germany
X Zhong, Sweden
J Zubicaray, Spain
A Žučenka, Lithuania
J Zuna, Czechia
S Zver, Slovenia
M Zwaan, The Netherlands eha

### Borderless Careers

### Igniting progress in hematology by enriching your professional journey

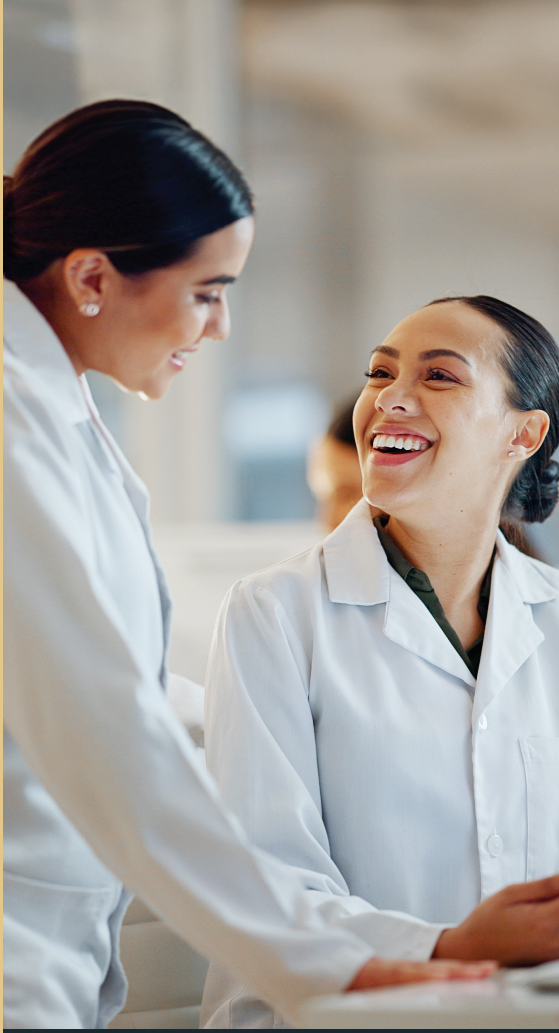

Advance your career at every stage with the European Hematology Association. Whether you’re a student, an early-career professional, or a seasoned specialist, EHA offers a vibrant community and invaluable resources to support your growth. Join us to connect with peers, access cutting-edge research, and enhance your skills through workshops and events.

Our network is not just a professional association; it is a community that nurtures growth, mentorship, and innovation in a manner that transcends geographical and intellectual borders.

To us, this is Borderless Hematology.

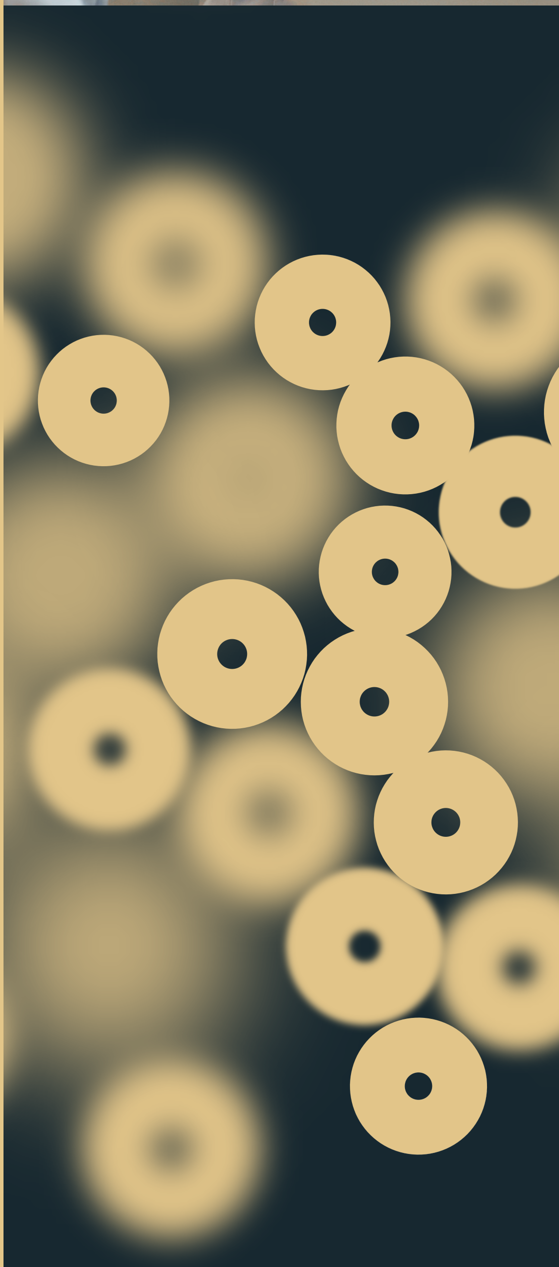

**Find out more about our career path here!**

### Words of Welcome

On behalf of the EHA Board and the Scientific Program Committee, we are pleased to present to you this year's Abstract Program for the EHA2026 Congress.

We have received a record number of 4738 abstract submissions from across the globe. After a thorough and careful review process, the Scientific Program Committee has designed a comprehensive program comprising both oral and poster presentations. We are also happy to announce an increased number of Oral Sessions to accommodate the high-quality number of submissions representing all fields of hematology. The highest scoring abstracts will be presented during the Plenary Abstract Session on Saturday, June 13 from 12:00 - 13:30 CEST, one of the highlights of the Congress program.

This year we continue with the new Congress program structure introduced last year with the scientific sessions spread out of 3,5 days (Thursday to Sunday), which also positively impacts the abstract program. This change provides you with ample opportunity to attend many of them and get updated on a broad range of topics in clinical and basic science areas.

We aim to give maximum exposure to scientific work and allow for direct interaction between the authors and the Congress delegates, so do not miss this unique opportunity to discuss and network during the Poster Sessions, which have been increased from one to two! Don't miss the opportunity to attend both on Friday and Saturday. In addition, all accepted posters can be viewed on (e)poster screens in Stockholm and on the Congress platform as of Thursday, June 11 at 09:00 CEST.

Supporting the next generation of hematologists is of the highest priority for EHA and this year we are awarding close to 300 travel grants to junior members who are presenting an abstract. In addition to the grants, EHA is again awarding Young EHA Best Abstract Awards in four categories to trainees with diverse scientific backgrounds and at different stages in their career. The names of all grant winners and awardees can be found on the next pages. EHA warmly congratulates them all.

The Late-Breaking Abstract Program is designed to provide an opportunity for investigators to submit abstracts with novel data that were not yet available at the time of the regular abstract submission deadline. Multiple abstracts of the highest scientific quality have been selected for presentation during the Late-Breaking Oral Session on Sunday, June 14 from 09:15 - 10:45 CEST, so please join us there.

All abstracts are available on the EHA Library and the Congress platform.

On behalf of the EHA Board, Scientific Program Committee & Advisory Board, all reviewers, and others involved in this year's Abstract Program, we trust you will have an interesting and inspiring congress of the European Hematology Association!

Looking forward to a lively conference in Stockholm!

Prof Meritxell Alberich Jorda
Chair Scientific Program Committee of the EHA2026 Congress

### Grant Winners

For this Congress, 300 travel grants have been awarded to junior members of EHA, based on the mean score of their abstracts as well as them being the first, submitting and presenting authors.

EHA congratulates the following grant awardees:

Abarca Rios E, Czechia
Age Kos I, Germany
Aguadé-Gorgorió J, Spain
Akhlaghi T, United States
Almabadi R, United Kingdom
Angotzi F, Italy
Appelman M, The Netherlands
Aquilina C, Italy
Argiro E, The Netherlands
Arnhardt I, Germany
Arrigo G, Italy
Bacarin F, Brazil
Baden D, Germany
Baech J, Denmark
Bähler L, Switzerland
Balantinakis A, Sweden
Barrena N, Spain
Bassani Lins De Miranda L, The Netherlands
Bataller A, United States
Becking AM, The Netherlands
Bertuglia G, Italy
Bhatnagar S, United States
Biosca C, Spain
Bock A, United States
Boldrin E, Sweden
Bonsdorff A, Finland
Booth C, United Kingdom
Boscaro E, Italy
Boudry A, France
Bouhlal J, Finland
Brieva Lopez A, Spain
Bushuieva M, Ukraine
Caduc M, Germany
Campoverde L, United States
Cantera Estefanía R, Spain
Capelletti MM, Italy
Carmody C, Ireland
Caruso M, Belgium
Cavalleri A, Italy
Ceran E, Germany
Chang YS, Taiwan
Chatzikalil E, Greece
Chatzikonstantinou T, Greece
Chen Z, United Kingdom
Cherng HJ, United States
Ciboddo M, Finland
Civettini I, Italy
Colmenero A, Spain
Consoli C, Italy
Corona L, Italy
Corsale AM, Italy
Craig R, United Kingdom
Cristóbal Vargas S, Spain
Croese K, The Netherlands
Croizier C, France
D'Addona M, Italy
Das Gupta D, Germany
de la Mata L, Spain
de Wit F, The Netherlands
Decombis S, Finland
Deyell J, United Kingdom
Doeven T, The Netherlands
Donato E, Germany
Dong M, China
Dreier T, Austria
Dulla D, Czechia
Duminuco A, Italy
Eipe T, India
Ellerington R, United Kingdom
Encinas Mayoral J, United States
Evangelidis P, Greece
Falodia R, India
Faria Zeni P, Czechia
Ferdinandus J, Germany
Fernandez-Luis S, Spain
Flight K, United Kingdom
Foo CY, United States
Foston A, United Kingdom
Galanopoulos A, Germany
Gariazzo C, Italy
Garrote-de-Barros A, Spain
Gil-Alós D, Spain
Giorgi M, United States
Globisch M, Sweden
Godard A, France
Gomez-Echarte N, Spain
Goulart H, United States
Gräßel L, Germany
Grau M, Spain
Grenier J, France
Grosu TI, Ireland
Gupta H, India
Gutierrez-Padilla C, Spain
Hamer L, United Kingdom
Hannuksela N, Finland
Herzberg F, Germany
Hoekx M, United Kingdom
Horváth R, Hungary
Htut TW, United States
Hu S, China
Hundebøll Torpe A, Denmark
Hunzeker Z, United States
Ibraheem A, United Kingdom
Idrizovic A, Spain
Jia Q, United Kingdom
Julia E, France
Kalafati E, Greece
Kamizela A, United Kingdom

Kamperman Z, The Netherlands
Kato J, Japan
Kato S, Japan
Kauer J, Germany
Kaulen LD, Germany
Ketharnathan S, Canada
Khan A, United Kingdom
Khan MA, United States
Kim R, France
Kiracı Şen B, Türkiye
Koca O, Türkiye
Koch C, Switzerland
Kolarik M, Czechia
Kolijn PM, The Netherlands
Kouroutzidou AZ, Greece
Koziel S, Germany
Kreuzbauer T, Australia
Krishnan V, United States
Laaouimir L, Belgium
Lam S, United Kingdom
Larios-Martinez KI, United Kingdom
Leonard E, Sweden
Li E, China
Li Q, China
Li S, United States
Liebaert J, Germany
Liem C, Germany
Lin F, China
Ling V, United Kingdom
Liu X, United Kingdom
Loap A, France
Löffler L, Denmark
Lui D, France
M Steiner T, Australia
MacAldaz M, Canada
MacPhee L, Canada
Maegaki M, Japan
Maeng C, Denmark
Maher N, Italy
Malik A, United Kingdom
MALJI A, India
Mandleywala K, United States
Manfreda L, Italy
Manzano-Muñoz A, Spain
Márquez-López I, Spain
Marugal MA, Spain
Marvin-Peek J, United States
Masnikov D, France
Massaar S, The Netherlands
Mato S, Spain
Mecklenbrauck R, United Kingdom
Medeiros Visentini L, Brazil
Merrion C, United Kingdom
Meskes N, The Netherlands
Mey F, Canada
Michaelou A, Czechia
Miedema SE, The Netherlands
Mikulski D, Poland
Milani M, Italy
Mönnig M, Germany
Montanaro A, Italy
Morcos MNF, Germany
Murali A, Canada
Myint PT, United States
Nasiri A, Saudi Arabia
Natani S, United Kingdom
Nikolaidou K, Greece
Nostroso A, Italy
Nyamondo K, United Kingdom
Ogłoza-Puchowska A, Poland
Ong XJ, Australia
Óskarsson JP, Iceland
Otasevic V, United States
Palmer M, United States
Palo S, Sweden
Palombella L, Switzerland
Pan M, China
Paschoudi K, Greece
Pasqualini G, Italy
Paterson J, United Kingdom
Pearce J, Germany
Pedone GL, Italy
Peek C, United States
Peng L, China
Pereira M, Portugal
Perpinello S, Italy
Perzolli A, The Netherlands
Portaluri M, Italy
Prummel K, Germany
Pula A, United States
Quero-Dotor C, Spain
Ramadani P, Canada
Ramanathan R, United States
Ramirez-Gamero A, United States
Ravattinen L, Finland
Ray G, United States
Renders S, Germany
Reuvekamp T, The Netherlands
Riccardi V, Italy
Richardson A, Iceland
Rigamonti C, Italy
RInkinen O, Finland
Rivero Equiza T, Spain
Rocha AL, Belgium
Rodriguez-Res C, Spain
Roehnert M, Germany
Rosato BE, Italy
Rosun T, United Kingdom
Roussos A, Greece
Ruiz Arenas C, Spain
Russo F, Italy
Salehi M, Switzerland
Sales L, Brazil
Sambruna E, Italy
Sánchez-Castillo A, Belgium
Scaramellini N, Italy
Schaefers C, United States
Schavgoulidze A, France
Schniederjohann C, Germany
Schönung M, Germany
Schovsbo J, Denmark
Schulze N, Germany
Seeling C, Germany
Sevim O, United Kingdom

Shah A, India
Shah P, United States
Shah R, United States
Sharif MI, Canada
Sharma s, India
Shimizu Y, Japan
Shokun N, Ukraine
Shuwaikan R, Spain
Simon F, Germany
Smits F, The Netherlands
Spadea M, Italy
Staehle H, Germany
Štefíková B, Czechia
Steinhäuser S, Germany
Stompór P, Poland
Sun R, Sweden
Swart K, The Netherlands
Szepesi G, Hungary
Tameni A, Italy
Tan M, United Kingdom
Tavakoli Shirazi P, Australia
Tavernari L, Italy
Tharmaseelan H, Germany
Toho M, Japan
Tseng TC, Taiwan
Turkulainen E, Finland
Ullahammer WM, Denmark
Urizar-Compains E, Spain
Vallvé i Oliva M, Spain
Van den Bergh S, Belgium
van der Velde J, The Netherlands
Van Trimpont M, Belgium
Vasileiou E, Germany
Vedenjuoksu J, Finland
Veltmaat N, The Netherlands
Vodicka P, Czechia
Voigt S, Germany
Wan H, Sweden
Wang S, China
Wang W, China
Wannberg F, Sweden
Wei Y, China
Weinhäuser I, The Netherlands
Wencel J, France
Wiechers L, Germany
Wikström F, Sweden
Yamamoto Y, Japan
Yami A, Italy
YANG M, Japan
Yuan Y, Australia
Zaratiegui A, Spain
Zeng A, Canada
Zhang F, China
Zhang X, China
Zhu J, Finland
Zhu J, United Kingdom
Zhu Y, United Kingdom
Žučenka A, Lithuania
Zugasti I, Spain
Zuleeg MR, Germany
Zwolsman M, The Netherlands

### Young EHA Best Abstract Awards

One of the primary missions of the European Hematology Association is to support young hematology clinicians and researchers. For the fourth year after launching in 2017, we are proud to announce the Young EHA Best Abstract Awards for 2024. These are awarded to the highest-ranking abstracts in the following four categories: Clinicians or medical students training for a PhD degree, PhD research students, postdoctoral fellows, and clinical hematology trainees. We are honored that these outstanding Young EHA trainees will be presenting during the EHA Congress - they are the future of hematology!

### CLINICIANS OR MEDICAL STUDENTS TRAINING FOR A PHD DEGREE
W Wang (China)

### POSTDOCTORAL FELLOWS
C Brieghel (Denmark)

### CLINICAL HEMATOLOGY TRAINEES
A Zhang (China)

### PHD RESEARCH STUDENTS
H Bergugnat (France)

abbvie
Join Us
as we share our commitment and passion in treating patients with hematologic malignancies
Visit the AbbVie booth B2.03 in Hall C
Don't miss our AML Product Theater on
Friday, June 12, 09:35 – 09:55
EHA Theater in Hall C
abbvie.com
ALL-ONC-260008 V1.0 Approved 3/2026

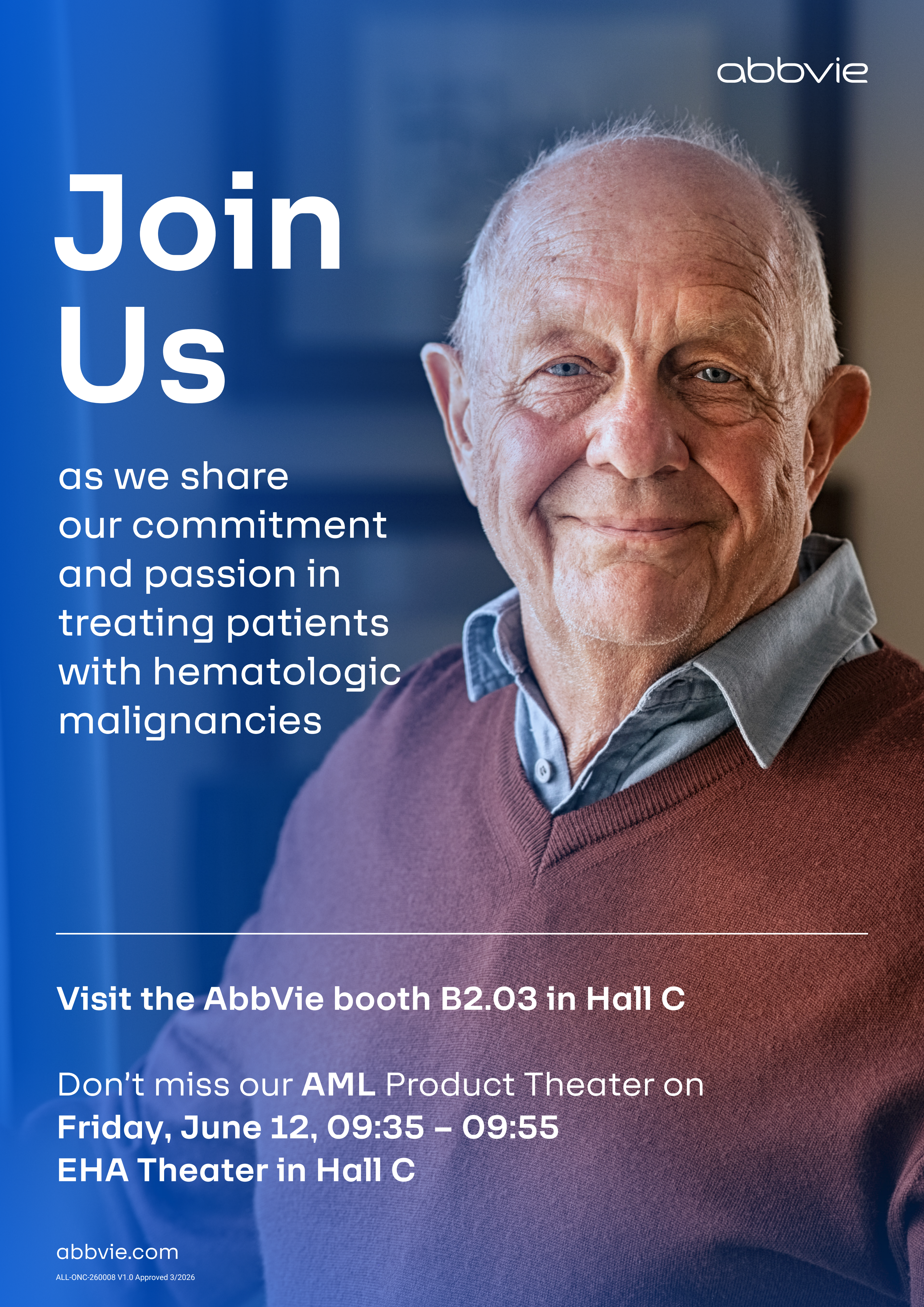

### COME SEE WHAT'S NEW IN MULTIPLE MYELOMA

Stop by the Sanofi booth to learn more sanofi

©2026 Sanofi-aventis Groupe. All rights reserved. Sanofi is a registered trademark of Sanofi or an affiliate.
MAT-GLB-2601772-v2.0-04/2026

### Have questions about multiple myeloma or myelofibrosis treatment options for your patients?

### Visit GSK Booth #E3/F3

Trademarks are owned by or licensed to the GSK group of companies.
©2026 GSK or licensor.
NP-GBL-MML-BNNR-260001 April 2026

GSK

BeOne
It's time to push the limits
of what BCL-2 inhibition can achieve
BeOne is unlocking the true power of BCL-2 inhibition with a more comprehensive development approach.
Speak with a representative at Booth G2.01
Redefine your expectations
Visit BCL2i.com
BCL-2=B-cell lymphoma-2.
BeOne Medicines is a trademark owned by BeOne Medicines I GmbH or its affiliates.
© BeOne Medicines I GmbH, 2026 All Rights Reserved. 0426-NP-PRC-077 6/2026

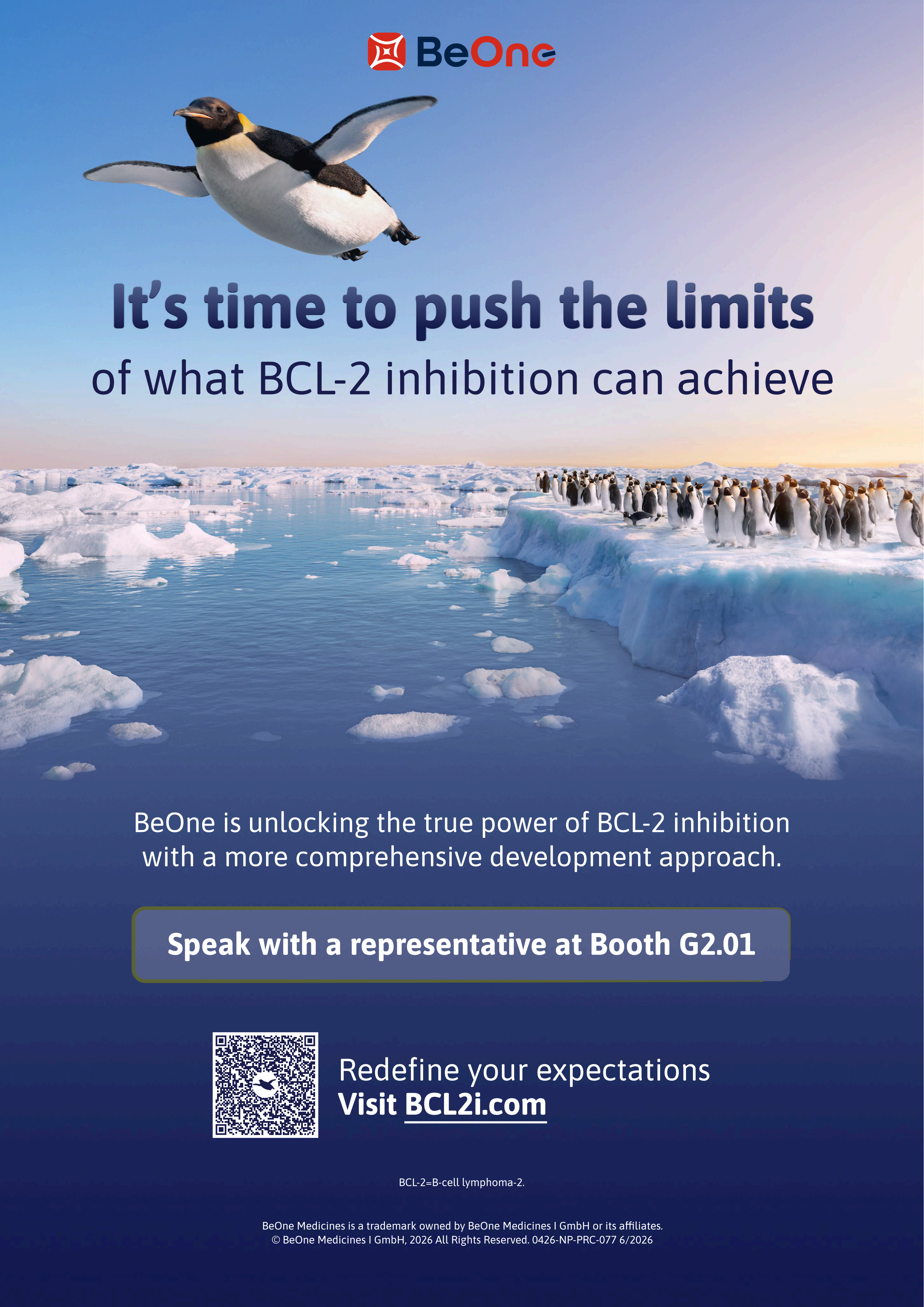

Syndax

### THE LEADER IN
### MENIN CLINICAL RESEARCH

### REVEAL ND NPM1

Revumenib Evaluation in Acute Leukemia (REVEAL-ND NPM1)

A global, actively recruiting phase 3 clinical trial investigating revumenib in combination with intensive chemotherapy for the treatment of patients aged 12 years and older with newly diagnosed AML with an *NPM1* mutation

This study is sponsored by Syndax Pharmaceuticals, Inc.

### Learn more about REVEAL-ND NPM1

**RevealTrialAML.com**

**revealsiteinquiries@syndax.com**
Please do not include any identifiable patient information in your email.
Note: Your message will be directed to a US-based email inbox.

### EVOLVE-2

A global, actively recruiting phase 3 clinical trial of revumenib in combination with azacitidine + venetoclax in adult patients with newly diagnosed *NPM1*-mutated or *KMT2A*-rearranged AML ineligible for intensive chemotherapy

This study is sponsored by HOVON, conducted by HOVON, AMLSG, UKAMLRN, ALFA, FILO, CETLAM, GIMEMA, ALLG, SAKK, Nordic AML Group, and the Beat AML Consortium, in collaboration with and funded by Syndax.

### Learn more about EVOLVE-2

**EUClinicalTrials.EU**
2024-512733-32-00

**HO177@erasmusmc.nl**
Please do not include any identifiable patient information in your email.
Note: Your message will be directed to a non-US-based email inbox.

VISIT US AT **BOOTH #A4.06**

Use of revumenib in this setting is under investigation and has not been approved by any regulatory authority worldwide for the uses under investigation.

This material is for scientific purposes only and is intended solely for healthcare practitioners.
Patient enrollment is dependent on approval by local ethics committees.

AML, acute myeloid leukemia; *KMT2A*, lysine methyltransferase 2A; *NPM1*, nucleophosmin 1.

© 2026 Syndax Pharmaceuticals, Inc. All rights reserved. The Syndax logo is a registered trademark of Syndax. All other referenced trademarks are the property of their respective owners.
MED-REV-260016 5/2026

Learn more at
**syndaxmedical.com**

### B.H.D.M

BHDM is a **global initiative confronting inequality in healthcare, research and treatment** access for Black communities affected by underfunded conditions like **Sickle Cell Disease**.
Through research, advocacy and storytelling, **we challenge a system where health innovation follows profit instead of need** — pushing to make equitable healthcare impossible to ignore.

DATA.
DIGNITY.
CHANGE.
NOT OPTIONAL.

**20+ MILLION**
people worldwide live with Sickle Cell Disease.

**<1%**
of NIH funding for SCD compared to diseases with lower mortality.

**MILLIONS**
lack access to adequate care, treatment and safe infrastructure.

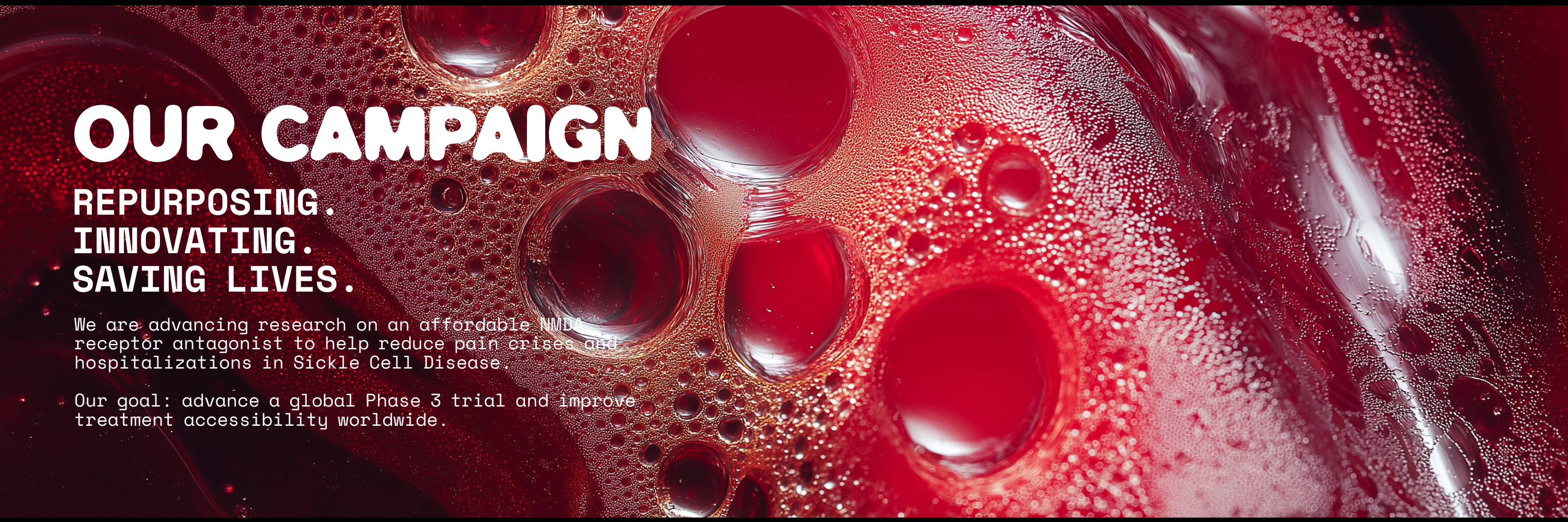

### OUR CAMPAIGN

**REPURPOSING.**
**INNOVATING.**
**SAVING LIVES.**

We are advancing research on an affordable NMDA receptor antagonist to help reduce pain crises and hospitalizations in Sickle Cell Disease.

Our goal: advance a global Phase 3 trial and improve treatment accessibility worldwide.

### JOIN the movement

Where research meets action. Join the conversation on advancing toward a Global Phase 3 Trial

### READ the research

Find the evidence recently published in **HemaSphere**, 2026, Jan 11; 10 (1): e70278

### WATCH the documentary

Discover the human stories behind the data. A film about resilience, inequity and hope.

EQUITABLE HEALTHCARE SHOULDN'T BE RADICAL

Click for more!

**SCAN NOW**
**to discover what's missing from the conversation**

### Table of Contents

### Plenary Abstracts Session

Plenary Abstracts Session S100-S104

### Oral Sessions

Advances in biology of ALL S105-S109
Advances in the treatment of lymphoblastic leukemia S110-S114
RNA and chromatin biology in AML S115-S119
Exploiting molecular vulnerabilities in AML S120-S124
Regimens involving Bcl-2 inhibition in AML S125-S129
Novel treatments in AML S130-S134
Recent advances in pediatric malignancies S135-S139
Chronic lymphocytic leukemia biology S140-S144
Prognostication and first line therapy in CLL S145-S149
Novel therapies in relapsed/refractory CLL S150-S154
Chronic myeloid leukemia - Biology & translational research S155-S159
Chronic myeloid leukemia - Clinical S160-S164
Myelodysplastic syndromes - Biology & translational research S165-S169
Myelodysplastic syndromes - Clinical S170-S174
Bone marrow failure syndromes incl. PNH - Biology & translational research S175-S179
Bone marrow failure syndromes incl. PNH - Clinical S180-S184
T cell redirected therapy in multiple myeloma S185-S189
Multiple myeloma translational studies S190-S194
Clinical trials results in multiple myeloma S195-S199
Immunotherapy in multiple myeloma S200-S204
Amyloidosis and multiple myeloma precursors S205-S209
Myeloproliferative neoplasms - Biology & translational research S210-S214
Myeloproliferative neoplasms - Clinical S215-S219
Hodgkin lymphoma (HL) S220-S224
Follicular lymphoma (FL) S225-S229
Mantle cell lymphoma (MCL) S230-S234
Prospective lymphoma trials S235-S239
Immunotherapy in lymphoma S240-S244
Other aggressive lymphomas S245-S249
Lymphoma biology S250-S254
Lymphoma translational S255-S259
Stem cell transplantation - Experimental S260-S263
Stem cell transplantation - Clinical 1 S265-S269
Stem cell transplantation - Clinical 2 S270-S274
Stem cells and their niche S275-S279
Gene therapy, cellular immunotherapy and vaccination - Biology & translational research S280-S284
Gene therapy, cellular immunotherapy and vaccination - Clinical S285-S289
Sickle Cell Disease: From drug development to real-world outcomes in Europe S290-S294

| | |
|---|---|
| Advances in therapies and insights in thalassemia and sickle cell disease | S295-S299 |
| Targeted therapies in rare red cell and metabolic disorders | S300-S304 |
| From erythropoiesis to transfusion practice | S305-S309 |
| Infections in hematology (incl. supportive care/therapy) | S310-S314 |
| Platelet disorders | S315-S319 |
| Bleeding disorders & thrombosis | S320-S324 |
| Patient-centered outcomes in hematology: Quality of life, equity and value | S325-S329 |
| Novel technologies with clinical impact in malignant hematology | S330-S334 |

### (S100) PHASE 3, RANDOMIZED STUDY OF TALQUETAMAB (TAL) PLUS DARATUMUMAB (DARA) ± POMALIDOMIDE (POM) VS DARA PLUS POM AND DEXAMETHASONE (DPD) IN RELAPSED/REFRACTORY MULTIPLE MYELOMA (RRMM): MONUMENTAL-3

**Topic:** 14. Myeloma and other monoclonal gammopathies - Clinical

Peter Voorhees*[1], Roberto Mina[2], Paula Rodríguez-Otero[3], Wenming Chen[4], María-Victoria Mateos[5], Jian Li[6], Philippe Moreau[7], Yael Cohen[8, 9], Chang-Ki Min[10], Roman Hájek[11, 12], Jing Christine Ye[13], Hila Magen[14, 15], Sam Rubinstein[16], Weijun Fu[17], Vania Hungria[18], Güldane Cengiz Seval[19], João De Holanda Farias[20], Jakub Radocha[21], Senem Maral[22], Memet Turgut[23], Youngil Koh[24], Daniel O'leary[25], Jayr Schmidt[26], Raymond Thertulien[27], Gang An[28], Jeffrey Huang[29], Sebastian Grosicki[30], Agata Tyczyńska[31], Rahul Banerjee[32], Matthew J. Pianko[33], Joaquín Martinez-Lopez[34], Paweł Steckiewicz[35], Dai Maruyama[36], Kentaro Fukushima[37], Albert Oriol[38], Jordi Lopez Pardo[39], Hartmut Goldschmidt[40], Charlotte Pawlyn[41], Aurore Perrot[42], Elena Zamagni[43], Meletios A. Dimopoulos[44], Leo Rasche[45], Jaszianne Tolbert[46], William Terry[46], Christelle Courtoux[46], Xiao Liu[47], Sandra Vasey[46], Kaitlyn Connors[48], Mariacristina Festa[49], Christoph Heuck[46], Angélique Langlois[46], Lisa O'rourke[46], Jiangxiu Zhou[46], Xiang Qin[46], Jiashen Lu[47], Jue Gong[46], Diego Vieyra[46], Meral Beksaç[50]

*[1]Atrium Health/Levine Cancer Institute, Wake Forest University School of Medicine, Charlotte, United States of America;[2]Winship Cancer Institute, Emory University, Department of Hematology and Medical Oncology, Atlanta, United States of America; [3]Cancer Center Clínica Universidad de Navarra, Pamplona, Spain; [4]Beijing Chaoyang Hospital, Capital Medical University, Beijing, China; [5]Hospital Clínico Universitario de Salamanca, Servicio de Salud de Castilla y León (SACYL), Castile and León, Spain; [6]Department of Hematology, Peking Union Medical College Hospital, Peking Union Medical College, Chinese Academy of Medical Sciences, Beijing, China; [7]Hematology Clinic, University Hospital Hôtel-Dieu, Nantes, France; [8]Tel-Aviv Sourasky (Ichilov) Medical Center, Tel Aviv, Israel; [9]Gray Faculty of Medical & Health Sciences, Tel Aviv University, Tel Aviv, Israel;[10]Seoul St. Mary's Hospital, The Catholic University of Korea, Seoul, South Korea; [11]Department of Hematooncology, University Hospital Ostrava, Ostrava, Czechia; [12]Department of Hematooncology, University of Ostrava, Ostrava, Czechia; [13]MD Anderson Cancer Center, University of Texas, Houston, United States of America; [14]Chaim Sheba Medical Center, Ramat-Gan, Israel;[15]Sackler Faculty of Medical and Health Sciences, Tel Aviv University, Tel Aviv, Israel; [16]Division of Hematology and Oncology, University of North Carolina School of Medicine, Chapel Hill, United States of America; [17]Department of Hematology, Shanghai Fourth People's Hospital, School of Medicine, Tongji University, Shanghai, China; [18]Clinica Médica São Germano, São Paulo, Brazil; [19]Ankara University Faculty of Medicine, Department of Hematology and Oncology, Ankara University, Ankara, Türkiye;[20]Liga Paranaense de Combate ao Câncer (LPCC), Curitiba, Paraná, Brazil; [21]4th Department of Internal Medicine – Hematology, University Hospital Hradec Kralove, Faculty of Medicine in Hradec Kralove, Charles University, Hradec Kralove, Czechia; [22]Medipol Mega University Hospital, Bağcılar, İstanbul, Türkiye;[23]Ondokuz Mayıs University, Samsun, Türkiye;[24]Seoul National University Hospital, Seoul National University, Jongno gu, Seoul, South Korea; [25]University of Minnesota, Minneapolis, United States of America; [26]A.C. Camargo Cancer Center, São Paolo, Brazil;[27]Novant Health Cancer Institute, Winston Salem, United States of America; [28]Institute of Hematology and Blood Diseases Hospital, Chinese Academy of Medical Sciences, Tianjin, China; [29]National Taiwan University Hospital, National Taiwan University College of Medicine, Zhongzheng District, Taipei, Taiwan; [30]Department of Cancer Prevention, Medical University of Silesia, Katowice, Poland;[31]Medical University of Gdańsk, Department of Hematology and Transplantology, University Clinical Center Gdańsk, Department of Hematology and Transplantology, Gdańsk, Poland; [32]Fred Hutchinson Cancer Center, Seattle, United States of America;[33]University of Michigan Health, Ann Arbor, United States of America; [34]Hospital Universitario 12 de Octubre, imas12/ Complutense University of Madrid/CNIO/Madrid, Madrid, Spain; [35]Holy Cross Cancer Center, Department of Hematology and Bone Marrow Transplantation, Kielce, Poland; [36]Cancer Institute Hospital, Japanese Foundation for Cancer Research (JFCR), Tokyo, Japan;[37]The University of Osaka Graduate School of Medicine, Suita, Japan; [38]Hospital Universitari Germans Trias i Pujol (HUGTiP), Institut Català de la Salut (ICS), Barcelona, Spain; [39]l'Hospital de la Santa Creu i Sant Pau, Barcelona, Catalonia, Spain;[40]Internal Medicine V, Hematology, Oncology and Rheumatology, GMMG Study Group, Heidelberg University Hospital and National Center for Tumor Diseases, Heidelberg, Germany; [41]The Institute of Cancer Research, London, UK, and The Royal Marsden NHS Foundation Trust, London, United Kingdom; [42]Universite de Toulouse, Centre Hospitalier Universitaire de Toulouse, Service Hematologie,*

**Copyright Information:** (Online) ISSN: 2572-9241
©2026 The Author(s). HemaSphere is published by John Wiley & Sons Ltd on behalf of European Hematology Association. This is an open access Abstract Book distributed under the Attribution-NonCommercial-NoDerivs (CC BY-NC-ND), which allows third parties to download the articles and share them with others as long as they credit the author and the Abstract Book, but they cannot change the content in any way or use them commercially.
**Abstract Book Citations:** Authors, Title, HemaSphere, 2026;10:(S1):pages. The abstract book DOIs can be found on the "Author Information" page.
**Disclaimer:** Articles published in the journal HemaSphere exclusively reflect the opinions of the authors. The authors are responsible for all content in their abstracts including accuracy of the facts, statements, citing resources, etc.

*IUCT Oncopole, Toulouse, France; [43]University of Bologna, Bologna, Italy; [44]National and Kapodistrian University of Athens, Athens, Greece; [45]University Hospital of Würzburg, Würzburg, Germany; [46]Johnson & Johnson, Spring House, United States of America; [47]Johnson & Johnson, Shanghai, China; [48]Johnson & Johnson, Raritan, United States of America; [49]Johnson & Johnson, Leiden, The Netherlands; [50]Istinye University, Ankara University, Ankara, Türkiye;*

**Background**

Tal (anti-GPRC5D bispecific antibody) has shown deep responses and durable efficacy in late line RRMM with a favorable infection profile. Its B-cell sparing mechanism facilitates combination with Dara and Pom, enabling potential synergistic efficacy in earlier lines where durable remissions are needed.

**Aims**

To report efficacy and safety of Tal+Dara+Pom (Tal-DP) and Tal+Dara (Tal-D) vs DPd in pts with RRMM and ≥1 prior line of therapy (LOT) from the pre-planned interim analysis of MonumenTAL-3 (NCT05455320).

**Methods**

Phase 3 study randomizing pts with RRMM and ≥1 prior LOT including lenalidomide (Len) and a proteasome inhibitor 1:1:1 to Tal-DP, Tal-D or DPd (**Figure A**). Primary endpoint was progression-free survival (PFS) by IRC for Tal-DP vs DPd and Tal-D vs DPd. Secondary endpoints included overall response, complete response or better (≥CR), minimal residual disease (MRD)-negative ≥CR ($10^{-5}$, NGS), overall survival (OS) and safety.

**Results**

Overall, 864 pts were randomized (Tal-DP, N=287; Tal-D, N=287; DPd, N=290). Baseline characteristics were balanced across arms. Median age was 64.0 yrs (range 30–88), median prior LOT was 2 (range 1–8); 11.8% were anti-CD38 (nearly all Dara) exposed; 85.1% and 93.4% were refractory to Len or last LOT, respectively; 30.9% had high risk cytogenetics. At 24.6 mo median follow-up, Tal-DP and Tal-D significantly improved PFS vs DPd (HRs [95% CI]: 0.28 [0.20–0.40], P<0.0001 and 0.33 [0.24–0.46], P<0.0001; **Figure B+C**). 24-mo PFS rates (95% CI) favored Tal-DP (81.3% [75.8–85.7]) and Tal-D (77.6% [71.7–82.5]) vs DPd (51.2% [44.8–57.1]). PFS benefit was consistent across clinically relevant subgroups. ORRs (88.2%, 88.5%, 77.6%), ≥CR rates (71.1%, 69.0%, 34.5%) and MRD-negative ≥CR rates (52.3%, 46.3%, 15.9%) were significantly higher for Tal-DP and Tal-D vs DPd, respectively. OS HRs (95% CI) vs DPd were 0.47 (0.30–0.73; P=0.0006) for Tal-DP and 0.51 (0.33–0.78; P=0.0015) for Tal-D. At data cutoff, 70.3% (Tal-DP), 69.7% (Tal-D) and 47.3% (DPd) of pts remained on study tx. AEs were manageable and consistent with expectations for each agent; overall AE rates (gr 3-4, gr 5): Tal-DP (96.7%, 1.8%), Tal-D (78.8%, 4.0%), DPd (95.8%, 4.6%). AEs leading to discontinuation (d/c) of all study tx occurred in 10.5% (Tal-DP), 8.0% (Tal-D), and 6.7% (DPd). Rates of cytopenias (any gr, gr 3-4) were: Tal-DP (91.7%, 87.7%), Tal-D (68.2%, 51.8%), and DPd (91.9%, 89.0%). Infection rates (any gr, gr 3-4, gr 5) were: Tal-DP (87.3%, 37.0%, 0.7%), Tal-D (84.3%, 27.7%, 1.5%), and DPd (83.0%, 41.0%, 1.8%). With Tal-DP and Tal-D, respectively, CRS (any gr: 67.8%; 58.4%) and ICANS (any gr: 2.9%; 1.8%) were mostly gr 1-2. Any gr GPRC5D-related AEs with Tal-DP, Tal-D and DPd, respectively, included taste changes (72.8%; 74.8%; 3.9%) and decreased weight (45.7%; 38.3%; 7.4%); most events were gr 1-2 and did not lead to Tal d/c. Ataxia/balance disorders (gr 1-2, gr 3) with Tal-DP (11.6%, 2.9%), Tal-D (10.2%, 2.2%) and DPd (0.4%, 0%) were primarily low gr (none gr ≥4); events led to Tal d/c in 4.7% (Tal-DP) and 2.2% (Tal-D).

**Copyright Information:** (Online) ISSN: 2572-9241
©2026 The Author(s). HemaSphere is published by John Wiley & Sons Ltd on behalf of European Hematology Association. This is an open access Abstract Book distributed under the Attribution-NonCommercial-NoDerivs (CC BY-NC-ND), which allows third parties to download the articles and share them with others as long as they credit the author and the Abstract Book, but they cannot change the content in any way or use them commercially.
**Abstract Book Citations:** Authors, Title, HemaSphere, 2026;10:(S1):pages. The abstract book DOIs can be found on the “Author Information” page.
**Disclaimer:** Articles published in the journal HemaSphere exclusively reflect the opinions of the authors. The authors are responsible for all content in their abstracts including accuracy of the facts, statements, citing resources, etc.

### Summary/Conclusion

Tal-DP and Tal-D demonstrated a significant and profound PFS benefit vs DPd, clinically meaningful OS improvements and gr ≥3 infection rates similar to or lower than DPd. Tal was combinable, with low rates of tx d/c. Efficacy appeared numerically better with Tal-DP vs Tal-D but with a higher gr 3-4 cytopenia rate, as expected with Pom. Tal-D with or without Pom represents a new standard of care for RRMM as early as 2L across all practice settings.

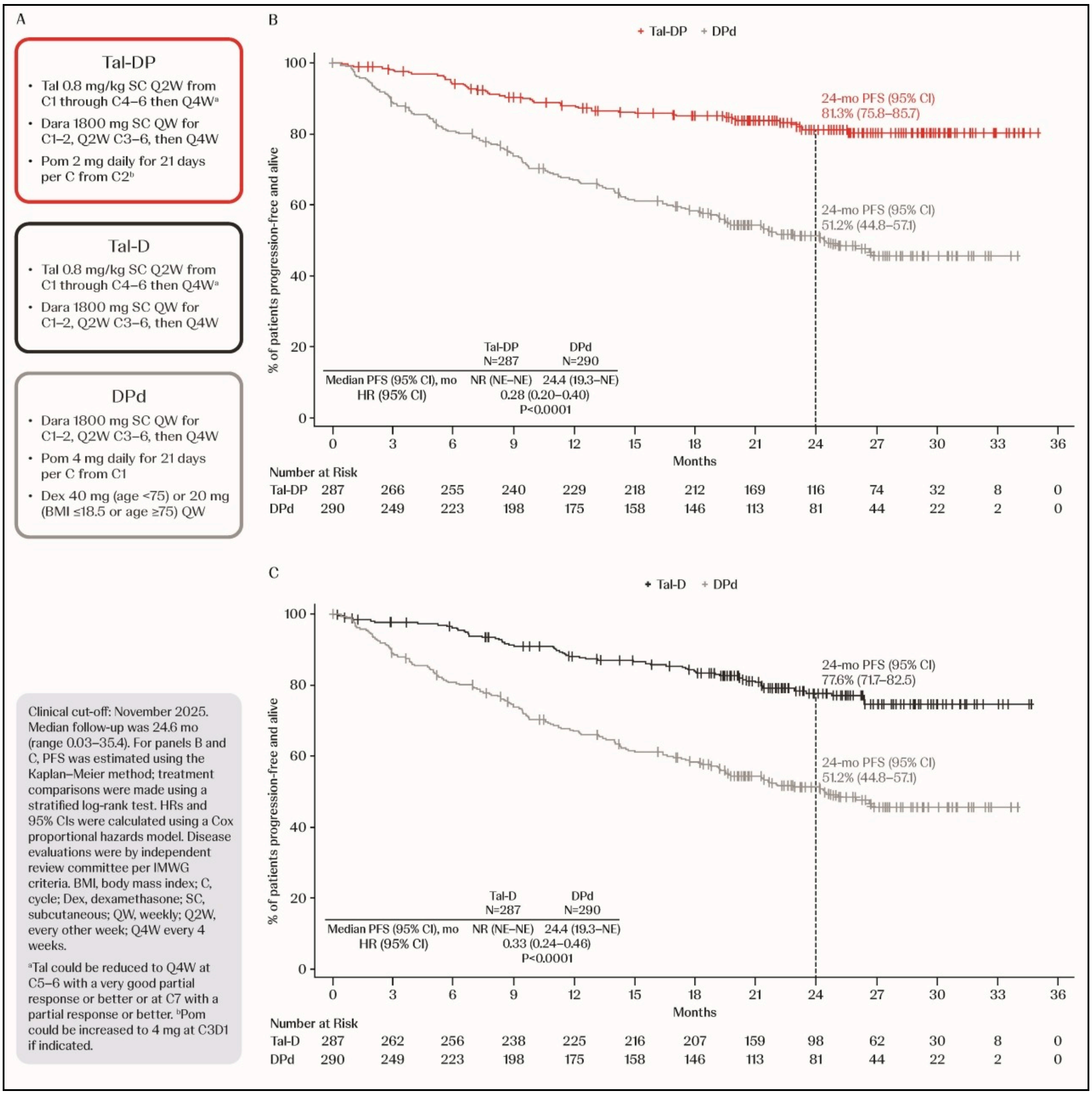

**Copyright Information:** (Online) ISSN: 2572-9241
©2026 The Author(s). HemaSphere is published by John Wiley & Sons Ltd on behalf of European Hematology Association. This is an open access Abstract Book distributed under the Attribution-NonCommercial-NoDerivs (CC BY-NC-ND), which allows third parties to download the articles and share them with others as long as they credit the author and the Abstract Book, but they cannot change the content in any way or use them commercially.
**Abstract Book Citations:** Authors, Title, HemaSphere, 2026;10:(S1):pages. The abstract book DOIs can be found on the "Author Information" page.
**Disclaimer:** Articles published in the journal HemaSphere exclusively reflect the opinions of the authors. The authors are responsible for all content in their abstracts including accuracy of the facts, statements, citing resources, etc.

### (S101) TAFASITAMAB PLUS LENALIDOMIDE AND R-CHOP FOR PATIENTS WITH PREVIOUSLY UNTREATED DIFFUSE LARGE B-CELL LYMPHOMA (DLBCL): RESULTS FROM THE PHASE 3 FRONTMIND STUDY

**Topic:** 19. Large B cell lymphomas - Clinical

Georg Lenz*[1], Marek Trněný[2], John M. Burke[3], Grzegorz Nowakowski[4], Christopher P. Fox[5], Annalisa Chiappella[6], Johannes Duell[7], Youngwoo Jeon[8], Chan Y. Cheah[9], Jason Westin[10], Joseph Z. Ye[11], Priscilla Caguioa[12], David Belada[13], Ho-Jin Shin[14], Sung Yong Oh[15], Sandy Amorim[16], Philomena Colucci[17], Sonia Ioannidis[18], Lulu Cheng[17], Umberto Vitolo[19]

*[1]University Hospital Münster, Department of Medicine A for Hematology, Oncology and Pneumology, Münster, Germany;[2]First Faculty of Medicine, Charles University, Prague, Czechia; [3]Sarah Cannon Research Institute at Rocky Mountain Cancer Centers, Aurora, CO, United States of America; [4]Division of Hematology, Mayo Clinic, Rochester, MN, United States of America;[5]School of Medicine, University of Nottingham, and Clinical Haematology, Nottingham University Hospitals NHS Trust, Nottingham, United Kingdom; [6]Fondazione IRCCS Istituto Nazionale dei Tumori, Hematology, Milano, Italy; [7]Department of Internal Medicine II, University Hospital Würzburg, Würzburg, Germany; [8]Department of Hematology, Yeouido St. Mary's Hospital, College of Medicine, The Catholic University of Korea, Seoul, South Korea; [9]Hollywood Private Hospital and University of Western Australia, Nedlands, Western Australia, Australia; [10]MD Anderson Cancer Center, Houston, TX, United States of America; [11]American Oncology Network, Vista Oncology Division, Olympia, WA, United States of America; [12]St. Luke's Medical Center, Quezon, Philippines; [13]Department of Internal Medicine-Haematology, Charles University, Hospital and Faculty of Medicine, Hradec Králové, Czechia; [14]Division of Hematology-Oncology, Department of Internal Medicine, Research Institute of Medical Science, Pusan National University Hospital, Pusan National University School of Medicine, Busan, South Korea; [15]Division of Hematology-Oncology, Department of Internal Medicine, Dong-A University College of Medicine, Busan, South Korea; [16]Department of Hematology and Cellular Therapy, Hôpital Saint Vincent de Paul, Lille, France; [17]Incyte Corporation, Wilmington, DE, United States of America; [18]Incyte Biosciences International Sàrl, Morges, Switzerland; [19]Candiolo Cancer Institute, FPO-IRCCS Candiolo, Turin, Italy;*

### Background

Approximately 40% of patients with DLBCL are not cured with first-line (1L) R-CHOP, underscoring a significant need for more effective 1L regimens. Tafasitamab (Tafa), a CD19-targeted mAb, added to lenalidomide (Len) plus R-CHOP (Tafa-Len-R-CHOP) demonstrated safety and efficacy in patients with previously untreated disease in the phase 1b First-MIND study.

### Aims frontMIND, a global, phase 3, double-blind, placebo-controlled study, compared efficacy and safety of Tafa-Len-R-CHOP vs R-CHOP in patients with high-risk, aggressive B-cell lymphomas.

### Methods

Patients aged 18-80 years with previously untreated, high-intermediate or high-risk DLBCL or HGBL (IPI 3-5, aaIPI 2-3 if ≤60 years), and ECOG PS 0-2 were randomized 1:1 to receive Tafa-Len-R-CHOP or R-CHOP. The primary endpoint was investigator-assessed PFS; secondary endpoints included EFS, OS, PET-negative CR and ORR at end of treatment (EOT), and safety; quality-of-life (QOL) was assessed as a secondary/exploratory endpoint.

**Copyright Information:** (Online) ISSN: 2572-9241
©2026 The Author(s). HemaSphere is published by John Wiley & Sons Ltd on behalf of European Hematology Association. This is an open access Abstract Book distributed under the Attribution-NonCommercial-NoDerivs (CC BY-NC-ND), which allows third parties to download the articles and share them with others as long as they credit the author and the Abstract Book, but they cannot change the content in any way or use them commercially.
**Abstract Book Citations:** Authors, Title, HemaSphere, 2026;10:(S1):pages. The abstract book DOIs can be found on the "Author Information" page.
**Disclaimer:** Articles published in the journal HemaSphere exclusively reflect the opinions of the authors. The authors are responsible for all content in their abstracts including accuracy of the facts, statements, citing resources, etc.

**Results**

At primary analysis (data cutoff Oct 20, 2025), 899 patients had been randomized to Tafa-Len-R-CHOP (n=448) or R-CHOP (n=451); baseline characteristics were similar between treatment arms. Median relative dose intensities (RDIs) for each of the six cycles were ≥99.7% and ≥80.0% for Tafa and Len, respectively, and 100% for each R-CHOP component in both treatment groups, except for vincristine (median RDI: ≥92.9% and ≥92.1% in the Tafa-Len-R-CHOP and R-CHOP arms, respectively). With a median follow-up of 35.2 months, Tafa-Len-R-CHOP led to a statistically significant improvement in PFS vs R-CHOP (hazard ratio [HR] 0.75 [95% CI: 0.59, 0.96]; *P*=0.019) in the overall population, with 24-month PFS rates of 71.1% vs 62.9%; in patients with centrally confirmed lymphoma subtypes (n=773) PFS HR was 0.68 (95% CI: 0.52, 0.88), with 24-month PFS rates of 72.7% vs 62.2%. PFS benefit was observed with Tafa-Len-R-CHOP in both activated B-cell–like (HR 0.59 [95% CI: 0.36, 0.95]) and germinal center B-cell–like (HR 0.69 [95% CI: 0.40, 1.19]) molecular cell-of-origin (COO) subtypes, per central GEP; consistent PFS benefit was observed in subgroup analyses of IPI score, age, and ECOG PS (**Table**). Tafa-Len-R-CHOP significantly improved EFS vs R-CHOP; PET-negative CR and ORR at EOT were similar between arms, and HR for OS was 0.85 (95% CI: 0.63, 1.14) (final OS analysis planned at 5 years) (**Table**). QOL outcomes improved in both treatment arms, with no significant differences observed between groups over time. Any-grade TEAEs were similar in the treatment arms (98.6% vs 97.1%); more grade ≥3 TEAEs occurred with Tafa-Len-R-CHOP vs R-CHOP (86.7% vs 76.1%). TEAEs leading to discontinuation of all study drugs occurred in 5.2% vs 5.4% of patients in the Tafa-Len-R-CHOP vs R-CHOP groups, respectively. There were more fatal TEAEs with Tafa-Len-R-CHOP vs R-CHOP (5.9% vs 3.8%) but fewer deaths overall with Tafa-Len-R-CHOP (18.5% vs 21.7%).

**Summary/Conclusion**

The primary endpoint of frontMIND was met; Tafa-Len-R-CHOP resulted in a significant 25% reduction in risk of disease progression or death compared with R-CHOP, with an 8.2% difference in 24-month PFS rate in the overall population and a 10.5% difference in patients with centrally confirmed lymphoma subtypes. TEAEs were manageable and consistent with the safety profile expected with the addition of Tafa and Len to R-CHOP. Tafa-Len-R-CHOP represents a potential new 1L standard of care for patients with high-risk DLBCL or HGBL, regardless of COO molecular subtype.

**Copyright Information:** (Online) ISSN: 2572-9241
©2026 The Author(s). HemaSphere is published by John Wiley & Sons Ltd on behalf of European Hematology Association. This is an open access Abstract Book distributed under the Attribution-NonCommercial-NoDerivs (CC BY-NC-ND), which allows third parties to download the articles and share them with others as long as they credit the author and the Abstract Book, but they cannot change the content in any way or use them commercially.
**Abstract Book Citations:** Authors, Title, HemaSphere, 2026;10:(S1):pages. The abstract book DOIs can be found on the "Author Information" page.
**Disclaimer:** Articles published in the journal HemaSphere exclusively reflect the opinions of the authors. The authors are responsible for all content in their abstracts including accuracy of the facts, statements, citing resources, etc.

**Table. Efficacy outcomes by treatment arm (investigator-assessed)**

| Variable | Tafa-Len-R-CHOP (n=448) | | R-CHOP (n=451) |
|---|---|---|---|
| ***Primary endpoint*** | | | |
| PFS, # events (%)* | 121 (27.0) | | 155 (34.4) |
| HR (95% CI) | | 0.75 (0.59, 0.96) | |
| *P* value | | 0.019 | |
| PFS rate at 24 months, % | 71.1 | | 62.9 |
| PFS rate at 36 months, % | 67.3 | | 60.7 |
| ***PFS subgroup analysis†*** | | | |
| **Cell-of-origin subtype by central GEP, HR (95% CI)** | | | |
| ABC | | 0.59 (0.36, 0.95) | |
| GCB | | 0.69 (0.40, 1.19) | |
| **IPI score, HR (95% CI)** | | | |
| IPI 3/aaIPI 2 | | 0.70 (0.50, 0.99) | |
| IPI 4-5/aaIPI 3 | | 0.82 (0.59, 1.14) | |
| **Age, HR (95% CI)** | | | |
| <65 years | | 0.77 (0.54, 1.10) | |
| ≥65 years | | 0.75 (0.55, 1.03) | |
| **ECOG PS at baseline, HR (95% CI)** | | | |
| 0-1 | | 0.79 (0.59, 1.05) | |
| 2 | | 0.71 (0.47, 1.09) | |
| ***Key secondary endpoints*** | | | |
| EFS, # events (%) | 154 (34.4) | | 191 (42.4) |
| HR (95% CI) | | 0.79 (0.64, 0.97) | |
| *P* value | | 0.026 | |
| OS, # events (%) | 82 (18.3) | | 95 (21.1) |
| HR (95% CI) | | 0.85 (0.63, 1.14) | |
| *P* value | | 0.270 | |
| ***Other secondary endpoints*** | | | |
| PET-negative CR at EOT, n (%) | 292 (65.2) | | 294 (65.2) |
| OR (95% CI) | | 0.998 (0.76, 1.31) | |
| Nominal *P* value | | 0.987 | |
| ORR at EOT, n (%) | 360 (80.4) | | 343 (76.1) |
| OR (95% CI) | | 1.290 (0.94, 1.78) | |
| Nominal *P* value | | 0.120 | |

*Disease progression or death from any cause. †Number of patients in subgroups by treatment arm (Tafa-Len-R-CHOP/R-CHOP): ABC subtype, n=84/88; GCB subtype, n=131/117; IPI 3/aaIPI 2, n=259/244; IPI 4-5/aaIPI 3, n=186/202; age <65 years, n=222/218; age ≥65 years, n=226/233: ECOG PS 0-1, n=324/326; ECOG PS 2, n=123/123.
aaIPI, age-adjusted International Prognostic Index; ABC, activated B-cell; CI, confidence interval; CR, complete response; ECOG PS, Eastern Cooperative Oncology Group performance status; EFS, event-free survival; EOT, end of treatment; GCB, germinal center B-cell; GEP, gene expression profiling; HR, hazard ratio; IPI, International Prognostic Index; OR, odds ratio; OS, overall survival; ORR,objective response rate; PET, positron emission tomography; PFS, progression-free survival.

**Copyright Information:** (Online) ISSN: 2572-9241
©2026 The Author(s). HemaSphere is published by John Wiley & Sons Ltd on behalf of European Hematology Association. This is an open access Abstract Book distributed under the Attribution-NonCommercial-NoDerivs (CC BY-NC-ND), which allows third parties to download the articles and share them with others as long as they credit the author and the Abstract Book, but they cannot change the content in any way or use them commercially.
**Abstract Book Citations:** Authors, Title, HemaSphere, 2026;10:(S1):pages. The abstract book DOIs can be found on the "Author Information" page.
**Disclaimer:** Articles published in the journal HemaSphere exclusively reflect the opinions of the authors. The authors are responsible for all content in their abstracts including accuracy of the facts, statements, citing resources, etc.

### (S102) EFFICACY AND SAFETY OF MITAPIVAT IN SICKLE CELL DISEASE: RESULTS FROM THE GLOBAL, RANDOMIZED, PHASE 3 RISE UP TRIAL

**Topic:** 27. Sickle cell disease

Biree Andemariam*[1], Kevin H.M. Kuo[2], Farjah Algahtani[3], Salam Salim Alkindi[4], Kenneth I. Ataga[5], Pablo Bartolucci[6], Andrew Campbell[7], Raffaella Colombatti[8], Alexander K. Glaros[9], Kaoutar Hafraoui[10], Modupe Idowu[11], Seethal A. Jacob[12], Clarisse Lobo[13], Obiageli Nnodu[14], Lucas Otieno[15], Wally R. Smith[16], Swee Lay Thein[17], Sarah Gheuens[18], Brooke Hensley[18], Abdulafeez Oluyadi[18], Kareem Osman[18], Asif Pathan[18], Spurthi Patil[18], Tao Song[18], Amber Yates[18], Ahmar U. Zaidi[18], Caterina Minniti[19], Miguel R. Abboud[20]

*[1]New England Sickle Cell Institute, UConn Health, Farmington, CT, United States of America;[2]Division of Hematology, Department of Medicine, University of Toronto, Toronto, ON, Canada; [3]Oncology Centre, Section of Adult Hematology/HSCT, College of Medicine, King Saud University Medical City, King Saud University, Riyadh, Saudi Arabia; [4]Department of Hematology, Sultan Qaboos University, Muscat, Oman; [5]University of Tennessee Health Science Center, Memphis, TN, United States of America; [6]Reference Center of Sickle Cell Disease, Mondor University Hospital, Créteil, France;[7]Division of Hematology, Children's National Hospital, Washington, DC, United States of America; [8]Pediatric Hematology Oncology Unit, University of Padova, Padova, Italy; [9]Central Michigan University, Children's Hospital of Michigan, Detroit, MI, United States of America; [10]Hematology, CHU Liège, Liège, Belgium; [11]McGovern Medical School, UTHealth, Houston, TX, United States of America; [12]Department of Pediatrics, Indiana University School of Medicine, Indianapolis, IN, United States of America; [13]Research Division, HEMORIO, Rio de Janeiro, Brazil; [14]Department of Hematology and Blood Transfusion, Centre of Excellence for Sickle Cell Disease Research and Training, College of Health Sciences, University of Abuja, Abuja, Nigeria; [15]Victoria Biomedical Research Institute, Kisumu, Kenya; [16]Virginia Commonwealth University, Richmond, VA, United States of America; [17]Sickle Cell Branch, The National Heart, Lung, and Blood Institute, Bethesda, MD, United States of America;[18]Agios Pharmaceuticals, Inc., Cambridge, MA, United States of America; [19]Montefiore Medical Center, Albert Einstein College of Medicine, New York, NY, United States of America; [20]Department of Pediatrics and Adolescent Medicine, American University of Beirut Medical Center, Beirut, Lebanon;*

### Background

Persistent anemia and hemolysis drive acute and chronic complications and contribute to increased mortality in sickle cell disease (SCD). Mitapivat, an oral activator of pyruvate kinase (PK), including the red blood cell–specific (PKR) and PKM2 isoforms, increases adenosine triphosphate (ATP) and decreases 2,3-diphosphoglycerate (2,3-DPG), with the potential to improve hemolytic anemia and reduce sickling.

### Aims

To evaluate the efficacy and safety of mitapivat vs placebo (PBO) in patients (pts) with SCD.

### Methods

In this global, double-blind, randomized, PBO-controlled, phase 3 trial, pts were randomized 2:1 to mitapivat 100 mg or PBO twice daily (NCT05031780). Primary endpoints were hemoglobin (Hb) response (≥1.0 g/dL increase in average Hb concentration from Week [Wk] 24 through Wk 52 compared with baseline) and annualized rate of sickle cell pain crises (SCPC). Key secondary endpoints included change in Hb concentration, markers of hemolysis, pt-reported fatigue (PROMIS Fatigue 13a T-scores), and frequency of hospitalizations for SCPC. Additionally, a post hoc analysis of pts who achieved Hb response was performed.

**Copyright Information:** (Online) ISSN: 2572-9241
©2026 The Author(s). HemaSphere is published by John Wiley & Sons Ltd on behalf of European Hematology Association. This is an open access Abstract Book distributed under the Attribution-NonCommercial-NoDerivs (CC BY-NC-ND), which allows third parties to download the articles and share them with others as long as they credit the author and the Abstract Book, but they cannot change the content in any way or use them commercially.
**Abstract Book Citations:** Authors, Title, HemaSphere, 2026;10:(S1):pages. The abstract book DOIs can be found on the "Author Information" page.
**Disclaimer:** Articles published in the journal HemaSphere exclusively reflect the opinions of the authors. The authors are responsible for all content in their abstracts including accuracy of the facts, statements, citing resources, etc.

### Results

A total of 207 pts were randomized to receive mitapivat (n=138) or PBO (n=69). The median (Q1, Q3) age was 25.0 years (21.0, 37.0); 57.5% were female. Baseline Hb (mean [±SD]) was 8.62 (±1.10) g/dL in the mitapivat group and 8.54 (±1.20) g/dL in the PBO group. In the mitapivat group, 40.6% of pts (56/138) achieved Hb response compared with 2.9% (2/69) in PBO (2-sided *P*<0.0001; **Figure**). The annualized rate (95% CI) of SCPC was 2.62 (2.29-3.01) in the mitapivat group compared with 3.05 (2.57-3.64) in PBO (2-sided *P*=0.1213). Mitapivat demonstrated significant improvements compared with placebo in average change from baseline (least-squares mean difference [95% CI]) in Hb concentration (0.74 g/dL [0.49-1.00]; 2-sided *P*<0.0001) and indirect bilirubin (-16.91 µmol/L [-21.01 to -12.81]; 2-sided *P*<0.0001) from Wk 24 through Wk 52.
Among pts who achieved Hb response, the mean (±SD) increase from baseline in Hb from Wk 24 through Wk 52 was 1.64 (±0.63) g/dL. Annualized rates (95% CIs) of SCPC were 2.20 (1.78-2.71) for Hb responders and 2.98 (2.52-3.53) for non-responders. Annualized rates (95% CIs) of hospitalizations for SCPC were 1.16 (0.87-1.55) for responders and 1.76 (1.41-2.20) for non-responders. The average change from baseline (least-squares mean [95% CI]) in PROMIS Fatigue 13a T-scores was -5.19 (-7.92 to -2.46) for responders and -2.55 (-4.94 to -0.17) for non-responders (change of -4.1 = clinically meaningful).
Serious adverse events (AEs) were reported in 20.3% (n=28) and 29.0% (n=20) of pts receiving mitapivat and PBO, respectively; 0.7% (n=1) and 0.0% (n=0) were considered treatment-related by the investigators. AEs led to treatment discontinuation in 4.3% of pts (n=6) receiving mitapivat and 2.9% (n=2) receiving PBO. No new safety signals were observed.

### Summary/Conclusion

Mitapivat treatment led to a statistically significant Hb response and improved markers of hemolysis in pts with SCD. Among those who achieved Hb response, additional clinical benefits (reductions in SCPC, associated hospitalizations, and fatigue) were observed. Mitapivat was generally well tolerated with a low discontinuation rate. These data highlight that mitapivat, through its oral administration and novel mechanism for improving hemolytic anemia, can be an effective, convenient, and disease-modifying treatment for pts with SCD.

**Figure.** Hemoglobin Response with Mitapivat and Placebo

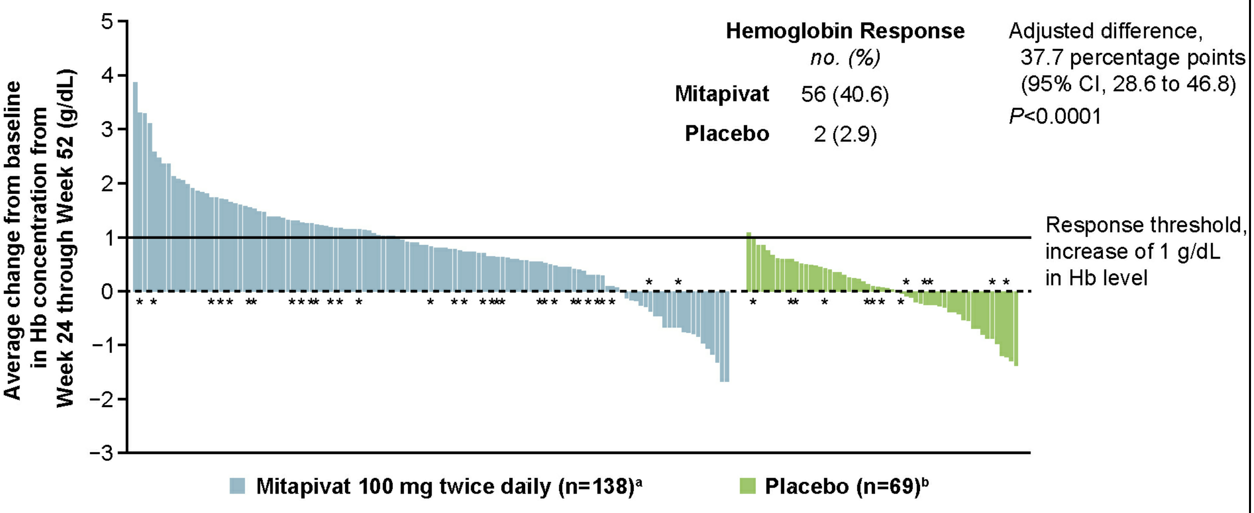

*Indicates participants with no hydroxyurea use at randomization.
[a]Includes 13 participants with missing baseline or with <2 assessments from Week 24 through Week 52, not depicted.
[b]Includes 12 participants with missing baseline or with <2 assessments from Week 24 through Week 52, not depicted.

**Copyright Information:** (Online) ISSN: 2572-9241
©2026 The Author(s). HemaSphere is published by John Wiley & Sons Ltd on behalf of European Hematology Association. This is an open access Abstract Book distributed under the Attribution-NonCommercial-NoDerivs (CC BY-NC-ND), which allows third parties to download the articles and share them with others as long as they credit the author and the Abstract Book, but they cannot change the content in any way or use them commercially.
**Abstract Book Citations:** Authors, Title, HemaSphere, 2026;10:(S1):pages. The abstract book DOIs can be found on the "Author Information" page.
**Disclaimer:** Articles published in the journal HemaSphere exclusively reflect the opinions of the authors. The authors are responsible for all content in their abstracts including accuracy of the facts, statements, citing resources, etc.

### (S103) REPLACEMENT OF HIGH DOSE COMBINATION CHEMOTHERAPY WITH BLINATUMOMAB IN NEWLY DIAGNOSED PEDIATRIC HIGH-RISK B-CELL ALL IMPROVES EFFICACY AND SAFETY IN THE RANDOMIZED PHASE 3 AIEOP-BFM ALL 2017 TRIAL

**Topic:** 02. Acute lymphoblastic leukemia - Clinical

Martin Schrappe*[1], Franco Locatelli[2], Maria Grazia Valsecchi[3], Gerhard Zugmaier[4], Michaela Vossen-Gajcy[5], Jan Stary[6], Andishe Attarbaschi[7], Anja Moricke[8], Jean-Pierre Bourquin[9], Draga Barbaric[10], Sarah Elitzur[11], Daniela Silvestri[12], Alexandra Kolenova[13], Luciano Dalla-Pozza[14], Nicole Bodmer[9], Martin Stanulla[15], Julia Alten[5], Faraz Zaman[16], Anke Bergmann[17], Giovanni Cazzaniga[18], Monika Brüggemann[19], Barbara Buldini[20], Grazia Fazio[21], Claudia Rossig[22], Lucie Sramkova[6], Rosanna Parasole[23], Patrick Hundsdörfer[24], Maria Caterina Putti[25], Arndt Borkhardt[26], Franca Fagioli[27], Laura Bettini[28], Fiona Poyer[7], Arend von Stackelberg[29], Valentino Conter[21], Martin Zimmermann[30], Carmelo Rizzari[28], Gunnar Cario[8], Andrea Biondi[18]

*[1]University Medical Center Schleswig-Holstein, Campus Kiel, Pediatric Hematology and Oncology, ALL-BFM Study Group, Kiel, Germany; [2]Catholic University of the Sacred Heart, Department of Pediatric Hematology and Oncology, IRCCS Ospedale Bambino Gesù, Rome, Italy; [3]School of Medicine and Surgery, University of Milano-Bicocca, and Tettamanti Center, Fondazione IRCCS San Gerardo dei Tintori, Monza, Monza, Italy; [4]Amgen Research (Munich GmbH), München, Germany; [5]Christian Albrechts University Kiel and University Hospital Schleswig-Holstein, Department of Pediatrics I, Pediatric Hematology/Oncology, Kiel, Germany; [6]2nd Faculty of Medicine, Charles University in Prague and Motol University Hospital, Department of Pediatric Haematology and Oncology, Prague, Czechia; [7]St. Anna Children's Hospital, Medical University of Vienna, Department of Pediatric Hematology and Oncology, Vienna, Austria; [8]Christian Albrechts University Kiel and University Hospital Schleswig-Holstein, Department of Pediatrics I, Pediatric Hematology/Oncology, ALL-BFM Study Group, Kiel, Germany; [9]Division of Oncology, Comprehensive Cancer Center Zurich, University Children Hospital Zurich, Zurich, Switzerland; [10]School of Clinical Medicine, UNSW Sydney, Kids Cancer Centre, Sydney Children's Hospital, Randwick, Randwick, Australia; [11]Schneider Children's Medical Center of Israel, Department of Hematology-Oncology, Tel Aviv, Israel; [12]Biostatistics and Clinical Epidemiology, Fondazione IRCCS San Gerardo dei Tintori, Tettamanti Center, Fondazione IRCCS San Gerardo dei Tintori, Monza, Monza, Italy; [13]National Institute of Children´s Diseases, Comenius University, Department of Pediatric Hematology and Oncology, Bratislava, Slovakia; [14]The Children's Hospital at Westmead NSW Sydney, The Cancer Centre for Children, Sydney, Australia; [15]Hannover Medical School, Hannover, Hannover, Germany; [16]Amgen Inc., Thousand Oaks, United States of America; [17]University Hospital Würzburg, Julius-Maximilians-University Würzburg, Institute for Clinical Genetics and Genomic Medicine, Würzburg, Germany; [18]School of Medicine and Surgery, University of Milano-Bicocca, Tettamanti Center, Fondazione IRCCS San Gerardo dei Tintori, Monza, Italy; [19]Christian Albrechts University Kiel and University Hospital Schleswig-Holstein, Hematology and Oncology, Section on Hematological Diagnostics, Kiel, Germany; [20]Istituto di Ricerca Pediatrica (IRP) Fondazione Città della Speranza, Pediatric Onco-Hematology, and Stem Cell Transplant and Gene Therapy Laboratory, Padova, Italy; [21]Tettamanti Center, Fondazione IRCCS San Gerardo dei Tintori, Monza, Italy; [22]University Children´s Hospital Münster, Münster, Germany; [23]Department of Oncology, Hematology and Cellular Therapy, Santobono-Pausilipon Children's Hospital, Naples, Italy; [24]Department of Pediatric Hematology and Oncology, Helios-Klinikum Berlin-Buch, Berlin, Germany;[25]Department of Women's and Child's Health, Azienda Ospedale-Università di Padova, Pediatric Hematology-Oncology Unit, Padova, Italy; [26]Haematology and Clinical Immunology, Medical Faculty, Heinrich-Heine University Düsseldorf, Department of Paediatric Oncology, Düsseldorf, Germany; [27]Regina Margherita Children's Hospital, Torino, Italy; Università di Torino, Department of Pediatric Oncology/Hematology, Torino, Italy; [28]School of Medicine and Surgery, University of Milano-Bicocca, Pediatric Hematology-Oncology Unit, Fondazione IRCCS San Gerardo dei Tintori, Monza, Italy; [29]Charité - Universitätsmedizin Berlin, Department of Pediatric Oncology and Hematology, Berlin, Germany; [30]Hannover Medical School, Department of Pediatric Hematology/Oncology, Hannover, Germany;*

**Copyright Information:** (Online) ISSN: 2572-9241
©2026 The Author(s). HemaSphere is published by John Wiley & Sons Ltd on behalf of European Hematology Association. This is an open access Abstract Book distributed under the Attribution-NonCommercial-NoDerivs (CC BY-NC-ND), which allows third parties to download the articles and share them with others as long as they credit the author and the Abstract Book, but they cannot change the content in any way or use them commercially.
**Abstract Book Citations:** Authors, Title, HemaSphere, 2026;10:(S1):pages. The abstract book DOIs can be found on the "Author Information" page.
**Disclaimer:** Articles published in the journal HemaSphere exclusively reflect the opinions of the authors. The authors are responsible for all content in their abstracts including accuracy of the facts, statements, citing resources, etc.

### Background

The bispecific T-cell engager Blinatumomab, targeting CD19 expressed on leukemic cells, may offer an option to safely reduce chemotherapy, while improving anti-leukemia efficacy in newly diagnosed pediatric patients with high-risk (HR) B-cell acute lymphoblastic leukemia (ALL).

### Aims

The primary objective of this trial was to determine whether the event-free survival (EFS) in children with HR ALL could be improved by at least 10% by replacing two highly intensive conventional chemotherapy courses (HR-2', HR-3') with two 28-day cycles of blinatumomab (plus 2 doses of intrathecal methotrexate per cycle). Secondary study objectives were: i) To assess whether life-threatening treatment-related complications and mortality could be reduced in the experimental blinatumomab arm (EA) compared with the chemotherapy control arm (CA) ii) To determine the proportion of patients with inadequate response based on minimal residual disease detection (MRD) in the two treatment arms.

### Methods

Following induction, consolidation, and one intensive chemotherapy course (HR-1`), 92.3% of 768 eligible patients were randomized to receive either the EA (n=358) or the CA (n=351) arm.

### Results

Baseline patient characteristics were balanced between the two randomized groups. A large proportion of patients presented with adverse genetic characteristics, such as hypodiploidy or *KMT2A* rearrangement, or were found to have an inadequate response to early treatment. Overall, 10% of HR patients had an indication for allogeneic hematopoietic stem cell transplantation (SCT) in first complete remission (CR1), mainly due to high end-consolidation MRD levels. A planned interim analysis (74% of target events reported, boundary of the interim analysis p-value = 0.031) revealed that the 4y-EFS was 83.0% (SE 2.5%) in the EA versus 70.3% (SE 3.1) in the CA arm (intent-to-treat analysis, p-value 0.0002), corresponding to an estimated hazard ratio (Cox model) of 0.51 (95% CI: 0.35-0.73) for EA compared to CA (2.9 years of median follow-up). The cumulative incidence of relapse (CIR) was 11.8% (SE 2.2%) in the EA (n= 31 relapses) as compared to 21.4% (SE 2.9%) in the CA (n=57). Remarkably, the rate of isolated CNS-relapse was 0.3% in the EA (n=1) while it was 2.5% in the CA (n=9). Patients still MRD-positive before randomization decreased their MRD load in 76.9% if treated with Blinatumomab, but only in 45.8% if treated with chemotherapy (p-value <0.0001). Toxicity profiles during the randomized treatment phase differed significantly between EA and CA – in favor of the EA: Clinically relevant infectious adverse reactions occurred in 69.4% of CA patients versus 22.8% in the EA (p<0.001). Neurologic adverse reactions were reported in 2.9% of the CA group and 11.7% in the EA (p<0.001). In the CA, 10% of the patients were hospitalized for severe mucositis/stomatitis versus 0.3% in the EA. Cytokine release syndrome ≥ grade 2 was rare in the EA (1.1%). Sixteen patients (4.7%) had 17 life-threatening adverse reactions in the CA versus one in the EA (as well fatal, 0.3%). Among transplanted patients, the non-relapse mortality was 16% in the CA (n=12/73) compared with 2.5% in the EA (n=2/79).

### Summary/Conclusion

For the first time, in newly diagnosed ALL, highly toxic chemotherapy elements have successfully been replaced by blinatumomab demonstrating a safer but also superior anti-leukemia efficacy of immunotherapy in prognostically unfavorable pediatric B-ALL.

**Copyright Information:** (Online) ISSN: 2572-9241
©2026 The Author(s). HemaSphere is published by John Wiley & Sons Ltd on behalf of European Hematology Association. This is an open access Abstract Book distributed under the Attribution-NonCommercial-NoDerivs (CC BY-NC-ND), which allows third parties to download the articles and share them with others as long as they credit the author and the Abstract Book, but they cannot change the content in any way or use them commercially.
**Abstract Book Citations:** Authors, Title, HemaSphere, 2026;10:(S1):pages. The abstract book DOIs can be found on the "Author Information" page.
**Disclaimer:** Articles published in the journal HemaSphere exclusively reflect the opinions of the authors. The authors are responsible for all content in their abstracts including accuracy of the facts, statements, citing resources, etc.

### (S104) CD5 INHIBITION AS A GENERALIZABLE STRATEGY TO AMPLIFY T CELL-MEDIATED ANTI-TUMOR EFFECTS OF BISPECIFIC T-CELL ENGAGERS

**Topic:** 01. Acute lymphoblastic leukemia - Biology & translational research

Weilin Wang*[1], Huanbin Zhao[2], Xu Yang[3], Qi Ji[1], Yixin Hu[1], Shuiyan Wu[1], Jiyang Yu[3], Ching-Hon Pui[4], Shaoyan Hu[1], Jun J Yang[2], Yizhen Li[1]

*[1]Children's Hospital of Soochow University, Department of Hematology and Oncology, Suzhou, China;[2]St. Jude Children's Research Hospital, Division of Pharmaceutical Sciences, Department of Pharmacy and Pharmaceutical Sciences, Memphis, United States of America; [3]St. Jude Children's Research Hospital, Department of Computational Biology, Memphis, United States of America; [4]St. Jude Children's Research Hospital, Department of Oncology, Memphis, United States of America;*

### Background

CD3-engaging T-cell engagers (TCEs), including the CD19×CD3 bispecific antibody blinatumomab, have transformed the treatment of B-cell acute lymphoblastic leukemia (B-ALL). However, responses remain heterogeneous and a substantial proportion of patients eventually relapse. While tumor-intrinsic resistance mechanisms have been extensively characterized, T cell–intrinsic determinants of TCE efficacy are poorly understood. Identifying druggable pathways that restrain T cell activation may support the design of T-cell-enhancing strategies with broad applicability across different TCE modalities.

### Aims

We sought to systematically identify T cell–intrinsic regulators of blinatumomab response, delineate the underlying mechanisms governing T cell activation, and determine whether targeting these pathways could enhance the cytotoxic effect of diverse CD3-engaging TCEs.

### Methods

We performed genome-wide and targeted CRISPR knockout screens in primary human CD8+ T cells co-cultured with B-ALL cells in the presence of blinatumomab, using cytokine expression levels as the primary functional readout. Candidate genes were validated by genetic deletion and antibody-mediated blockade. Functional consequences were evaluated using cytotoxicity assays, proliferation analysis, cytokine profiling, bulk RNA-seq, proteomics, phospho-flow cytometry, and single-cell RNA-seq. Mechanistic studies included immunoprecipitation–mass spectrometry and intracellular trafficking analyses. In vivo efficacy was assessed in xenograft models of leukemia and multiple myeloma. Additional CD3-engaging bispecific antibodies targeting CD20, BCMA, GPRC5D, DLL3, and GD2 were tested to evaluate generalizability.

**Copyright Information:** (Online) ISSN: 2572-9241
©2026 The Author(s). HemaSphere is published by John Wiley & Sons Ltd on behalf of European Hematology Association. This is an open access Abstract Book distributed under the Attribution-NonCommercial-NoDerivs (CC BY-NC-ND), which allows third parties to download the articles and share them with others as long as they credit the author and the Abstract Book, but they cannot change the content in any way or use them commercially.
**Abstract Book Citations:** Authors, Title, HemaSphere, 2026;10:(S1):pages. The abstract book DOIs can be found on the “Author Information” page.
**Disclaimer:** Articles published in the journal HemaSphere exclusively reflect the opinions of the authors. The authors are responsible for all content in their abstracts including accuracy of the facts, statements, citing resources, etc.

**Results**

The CRISPR screen identified CD5 as a negative regulator of blinatumomab-induced T cell activation. CD5 deletion or antibody blockade significantly enhanced blinatumomab-mediated cytotoxicity against B-ALL cell lines and primary blasts in vitro, and improved leukemia control and survival in vivo. CD5 inhibition augmented activation marker expression (i.e., CD25 and CD69), cytokine production (i.e., IFNγ and TNFα), and proliferation in a blinatumomab-dependent manner. Transcriptomic and proteomic profiling revealed enrichment of effector programs, including IL2–STAT5, TNFα–NF-κB, and MTORC1 signaling, accompanied by increased ERK and S6 phosphorylation. Single-cell RNA sequencing demonstrated that CD5-low CD8+ T cells preferentially undergo proliferative expansion and express effector molecules upon blinatumomab stimulation. In patient datasets, higher CD5 expression correlated with inferior response to blinatumomab. Mechanistically, antibody engagement induced CD5 internalization followed by RAB7A-dependent lysosomal degradation. Immunoprecipitation–mass spectrometry identified interactions between CD5 and proximal TCR signaling components, including LCK, ZAP70, CBLB, and UBASH3A, supporting a model in which CD5 assembles an inhibitory signalosome that restrains ERK-driven activation. Importantly, CD5 inhibition potentiated multiple CD3-engaging TCEs across hematologic and solid tumor models in vitro and in vivo.

**Summary/Conclusion**

CD5 acts as an intrinsic brake on TCE-mediated T cell activation. Genetic or antibody-mediated CD5 inhibition enhances cytotoxicity, effector-like activation, and anti-tumor efficacy across diverse CD3-engaging platforms. These findings establish CD5 as a central, druggable target to optimal TCEs efficacy.

**Copyright Information:** (Online) ISSN: 2572-9241
©2026 The Author(s). HemaSphere is published by John Wiley & Sons Ltd on behalf of European Hematology Association. This is an open access Abstract Book distributed under the Attribution-NonCommercial-NoDerivs (CC BY-NC-ND), which allows third parties to download the articles and share them with others as long as they credit the author and the Abstract Book, but they cannot change the content in any way or use them commercially.
**Abstract Book Citations:** Authors, Title, HemaSphere, 2026;10:(S1):pages. The abstract book DOIs can be found on the "Author Information" page.
**Disclaimer:** Articles published in the journal HemaSphere exclusively reflect the opinions of the authors. The authors are responsible for all content in their abstracts including accuracy of the facts, statements, citing resources, etc.

### (S105) TARGETING THE ATYPICAL PROTEIN KINASE WNK1 IN TAL1-RELATED T-CELL ACUTE LYMPHOBLASTIC LEUKEMIA

**Topic:** 01. Acute lymphoblastic leukemia - Biology & translational research

Anna Montanaro[1, 2], Gregorio Monica[1, 2], Anna D'antuono[1], Raffaella Zamponi[2, 3], Alice Andreani[2], Gabriella Sammarelli[3], Giannalisa Todaro[3], Roberta La Starza[4], Cristina Mecucci[4], Mariateresa Giaimo[1, 2], Andrea Gherli[1, 2], Federica Vento[1, 2], Bruno Lorusso[1], Elisa Simoncini[1, 2], Luca Pagliaro[1, 2], Connie Jimenez[5], Federico Quaini[1], Giovanni Roti*[1, 2, 3]

*[1]University of Parma, Department of Medicine and Surgery, Parma, Italy;[2]Translational Hematology and Chemogenomics Laboratory, University of Parma, Parma, Italy; [3]Azienda Ospedaliero-Universitaria di Parma, Hematology and BMT Unit, Parma, Italy; [4]University of Perugia, Institute of Hematology and Center for Hemato-Oncology Research, Perugia, Italy;[5]Amsterdam University Medical Centers, OncoProteomics Laboratory, Cancer Center Amsterdam, VU University, Amsterdam, The Netherlands;*

### Background

Protein kinase targeted therapies have transformed cancer treatment. However, few aberrantly activated, druggable kinases have been identified in T-cell acute lymphoblastic leukemia (T-ALL), where oncogenesis is largely transcription-driven. Therefore, strategies beyond mutational profiling, such as integrating unbiased phosphoproteomics with genomic data, may uncover novel therapeutic targets in T-ALL.

### Aims

Our study amis to identify and functionally validate kinase dependencies in T-ALL, with a specific focus on WNK1 as a therapeutic target in the TAL1-driven subgroup.

### Methods

To profile kinase dependencies in T-ALL, we intersected phosphoproteomic data from 11 T-ALL cell lines with genomic and functional data from the Cancer DepMap Project. WNK1, an atypical serine/threonine kinase, emerged as a top dependency in T-ALL cell lines, primary samples, and a *STIL::TAL1* patient-derived xenograft (PDX) model. Functional validation was achieved using the ATP-competitive WNK inhibitor WNK463 and CRISPR/Cas9 or shRNA-mediated *WNK1* knockout *in vitro* and *in vivo*.

**Copyright Information:** (Online) ISSN: 2572-9241
©2026 The Author(s). HemaSphere is published by John Wiley & Sons Ltd on behalf of European Hematology Association. This is an open access Abstract Book distributed under the Attribution-NonCommercial-NoDerivs (CC BY-NC-ND), which allows third parties to download the articles and share them with others as long as they credit the author and the Abstract Book, but they cannot change the content in any way or use them commercially.
**Abstract Book Citations:** Authors, Title, HemaSphere, 2026;10:(S1):pages. The abstract book DOIs can be found on the “Author Information” page.
**Disclaimer:** Articles published in the journal HemaSphere exclusively reflect the opinions of the authors. The authors are responsible for all content in their abstracts including accuracy of the facts, statements, citing resources, etc.

**Results**

We demonstrated that in T-ALL, WNK1 exhibits higher expression than in other tumors. Additionally, it is differentially expressed compared to lymphocytes and thymocytes, highlighting a potential clinical therapeutic index to explore. Moreover, in T-ALL patients, *WNK1* expression positively correlates ($p < 0.0001$) with *TAL1* oncogenic subgroup and consequently with *PTEN* mutations and del6q ($p < 0.05$), which are more prevalent in this context. Consistently, ectopic modulation of *TAL1* by shRNA or ORF altered WNK1 mRNA and protein levels, as well as, chromatin accessibility at the *WNK1* promoter, supporting a regulatory relationship between TAL1 and WNK1. Furthermore, PTEN protein expression inversely correlated with pWNK-S382, a marker of WNK1 activation. Accordingly, *PTEN* loss leads to upregulation of WNK1 mRNA and protein expression, suggesting a functional link between WNK1 activation and *PTEN* loss. Then, to validate WNK1 as a therapeutic target, we characterized the effect of WNK perturbation in T-ALL cell lines and primary samples upon WNK463 treatment or following *WNK1* knockout. Collectively, we demonstrated that WNK1 inhibition suppresses T-ALL viability, causing cell cycle defects, and ultimately leading to apoptosis, especially in TAL-related (TAL-R) T-ALL. Furthermore, *WNK1* repression promotes polyploidy, resulting in incomplete cell division or chromosome segregation defects. Consistently, transcriptional analysis in *WNK1* depleted cells revealed that the *WNK1* loss modulates G2/M checkpoint and cell cycle signatures, explaining the cellular phenotypes observed upon chemical inhibition. Given the WNK1 role for TAL-R survival, we established a *STIL::TAL1* PDX model and evaluated its inhibition in two contexts: drug efficacy and leukemia establishment. First, we demonstrated that WNK463 effectively reduced leukemia burden by an on-target effect, improving survival without organ toxicity in treated TAL1-driven leukemia mice compared to non-TAL1 or vehicle. Secondly, *in vivo*, inducible suppression of *WNK1* delayed leukemia engraftment and spleen infiltration compared to WNK1-competent leukemia, further prolonging survival.

**Summary/Conclusion**

Our integrative proteogenomic analysis identifies WNK1 as a novel oncogenic driver in T-ALL. These findings underscore the role of atypical proteomes in carcinogenesis and support further development of selective WNK1 inhibitors as potential targeted therapy for high-risk T-ALL patients.

**Copyright Information:** (Online) ISSN: 2572-9241
©2026 The Author(s). HemaSphere is published by John Wiley & Sons Ltd on behalf of European Hematology Association. This is an open access Abstract Book distributed under the Attribution-NonCommercial-NoDerivs (CC BY-NC-ND), which allows third parties to download the articles and share them with others as long as they credit the author and the Abstract Book, but they cannot change the content in any way or use them commercially.
**Abstract Book Citations:** Authors, Title, HemaSphere, 2026;10:(S1):pages. The abstract book DOIs can be found on the "Author Information" page.
**Disclaimer:** Articles published in the journal HemaSphere exclusively reflect the opinions of the authors. The authors are responsible for all content in their abstracts including accuracy of the facts, statements, citing resources, etc.

### (S106) ONCOGENIC MECHANISMS AND THERAPEUTIC TARGETING OF THE HIGH-RISK CDX2/UBTF B-ALL

**Topic:** 01. Acute lymphoblastic leukemia - Biology & translational research

Hugo Bergugnat*[1], Melha Benlebna[1], François Sigaux[1], Fabio Steffen[2], Marie Passet[3], Valentin Clichet[3], Rathana Kim[3], Lucie Hernandez[1], Loïc Maillard[1], Marie de Tersant[1], Lise Larcher[1], Severine Lecourt[1], Véronique Parietti[1], Véronique Lhéritier[4], Jean-Pierre Bourquin[2], Nicolas Boissel[3], Camille Lobry[1], Beat Bornhauser[2], Stephanie Gachet[1], Jean Soulier[1], Emmanuelle Clappier[1]

*[1]INSERM U1342 - Hôpital St-Louis, Paris, France;[2]Children's Hospital Zurich, Zürich, Switzerland; [3]Hôpital Saint-Louis, Paris, France; [4]Centre Hospitalier Lyon Sud, Pierre-Bénite, France;*

### Background

CDX2/UBTF B-cell acute lymphoblastic leukemia (B-ALL) is a recently described adult subtype characterized by the systematic association of two alterations: strong ectopic expression of the CDX2 gene and the presence of the chimeric fusion protein UBTF::ATXN7L3. This entity is associated with a very poor treatment response, with a 3-year cumulative relapse incidence of 75% highlighting the urgent need for novel therapeutic strategies.

### Aims

We aimed to identify effective therapeutic vulnerabilities in CDX2/UBTF B-ALL and investigate the molecular mechanisms through which CDX2 and UBTF::ATXN7L3 likely cooperate to drive leukemogenesis.

### Methods

Transcriptomic analysis was performed on 29 CDX2/UBTF B-ALL and compared to 981 other B-ALL cases. Patient-derived xenograft (PDX) models were established in NSG mice, including one luciferase-expressing model. A large-scale ex vivo pharmacologic screen was conducted using four independent CDX2/UBTF PDX models co-cultured on stromal cells. Pre-clinical experiments in NSG mice were performed with leukemic infiltration monitoring by bioluminescence imaging and bone marrow assessment by flow cytometry. Co-immunoprecipitation (co-IP) assays were performed in HEK293T transfected with CDX2 and UBTF::ATXN7L3 constructs. ATACseq and CUT&RUN experiments were performed in PDX cells to investigate chromatin accessibility and transcription factor occupancy.

### Results

Ex vivo drug screening including four distinct CDX2/UBTF PDX models identified XPO1 inhibitors among the most potent compounds in our models. Notably, CDX2/UBTF B-ALL displayed significantly greater sensitivity to XPO1 inhibition compared with a large reference dataset from other B-ALL subtypes. In vivo, eltanexor treatment induced a dramatic reduction of leukemic burden in 3 PDX models (3-log reduction in total bioluminescence flux $p<0.05$, 34% vs 1% blasts, $p < 0.05$, 57% vs 0.02% blasts, $p < 0.05$, respectively).
Interestingly, the nuclear export protein XPO1 was recently shown to be involved in chromatin binding complexes with oncoproteins that drive HOX/MEIS1 dependent-leukemogenesis. CDX2/UBTF B-ALL also harbor HOXA/MEIS expression, which prompted us to investigate a possible role of XPO1 in CDX2/UBTF leukemogenic program. By co-IP in transfected HEK293T cells we demonstrated physical interaction between both driver oncoproteins, CDX2 and UBTF::ATXN7L3, and XPO1. Furthermore, CUT&RUN and ATACseq experiments revealed shared chromatin occupancy of CDX2, UBTF, and XPO1 at regulatory regions of genes specifically expressed in this leukemia subtype.

**Copyright Information:** (Online) ISSN: 2572-9241
©2026 The Author(s). HemaSphere is published by John Wiley & Sons Ltd on behalf of European Hematology Association. This is an open access Abstract Book distributed under the Attribution-NonCommercial-NoDerivs (CC BY-NC-ND), which allows third parties to download the articles and share them with others as long as they credit the author and the Abstract Book, but they cannot change the content in any way or use them commercially.
**Abstract Book Citations:** Authors, Title, HemaSphere, 2026;10:(S1):pages. The abstract book DOIs can be found on the “Author Information” page.
**Disclaimer:** Articles published in the journal HemaSphere exclusively reflect the opinions of the authors. The authors are responsible for all content in their abstracts including accuracy of the facts, statements, citing resources, etc.

### Summary/Conclusion

Our study identifies XPO1 inhibition as a promising therapeutic strategy in chemotherapy-resistant CDX2/UBTF B-ALL. Moreover, our findings support a model in which CDX2/UBTF leukemogenesis depends on an XPO1-containing chromatin regulatory complex, thereby creating a targetable dependency. Ongoing analyses aim to clarify the transcriptional and epigenetic consequences of XPO1 inhibition and to further validate this therapeutic approach for clinical translation.

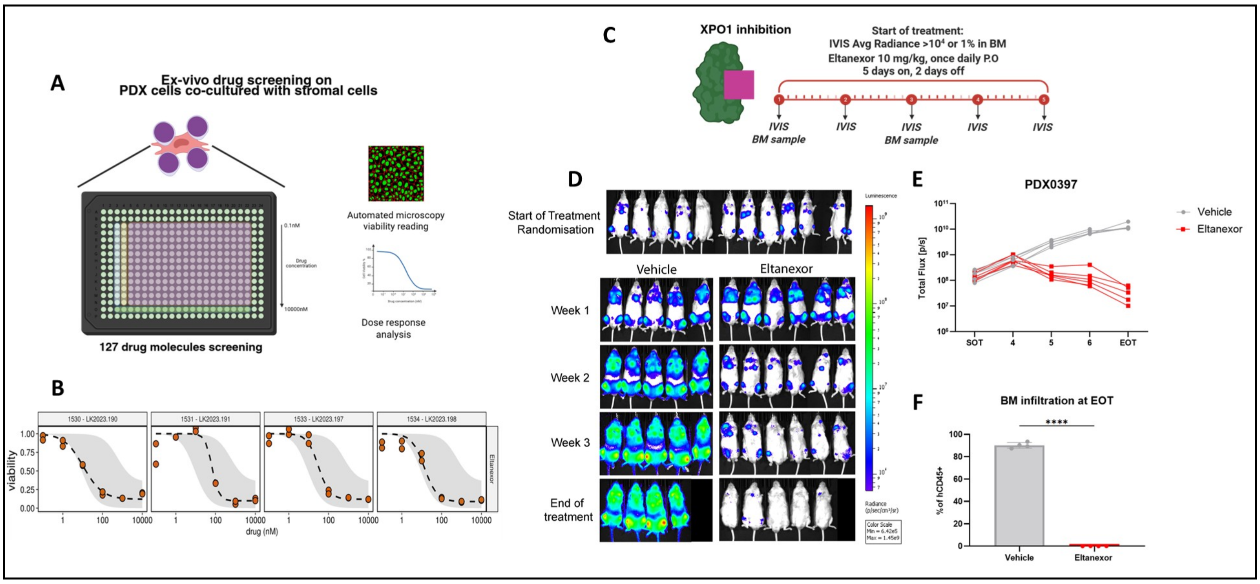

CDX2/UBTF B-ALL are sensitive to XPO1 inhibitors (A) Experimental setup of the ex-vivo drug screening (B) Dose response curves to the XPO1 inhibitor Eltanexor in 4 independent CDX2/UBTF PDX with the projection (in grey) of the 10th and 90th quantile of the cohort (C) Experimental setup of in-vivo treatment in NSG mice with 10mg/kg Eltanexor (D) Bio-luminescence images (E) Bio-luminescence total flux quantification (F) Bone marrow aspirates after 4 weeks of treatment.

**Copyright Information:** (Online) ISSN: 2572-9241
©2026 The Author(s). HemaSphere is published by John Wiley & Sons Ltd on behalf of European Hematology Association. This is an open access Abstract Book distributed under the Attribution-NonCommercial-NoDerivs (CC BY-NC-ND), which allows third parties to download the articles and share them with others as long as they credit the author and the Abstract Book, but they cannot change the content in any way or use them commercially.
**Abstract Book Citations:** Authors, Title, HemaSphere, 2026;10:(S1):pages. The abstract book DOIs can be found on the "Author Information" page.
**Disclaimer:** Articles published in the journal HemaSphere exclusively reflect the opinions of the authors. The authors are responsible for all content in their abstracts including accuracy of the facts, statements, citing resources, etc.

### (S107) ECTOPIC ACTIVATION OF FOXF1 AND FENDRR BY T(14;16)(Q32;Q24) IDENTIFIES A HIGH-RISK T-ALL/MPAL SUBTYPE.

**Topic:** 01. Acute lymphoblastic leukemia - Biology & translational research

Danika Di Giacomo*[1, 2, 3], Petri Pölönen[2], Valentina Bardelli[3], Shunsuke Kimura[2], Valentina Pierini[3], Luca Pagliaro[4], Silvia Arniani[3], Yunchao Chang[2], Qingsong Gao[2], Lindsey Montefiori[2], Yiming Wu[2], Chun Shik Park[2], William Wright[2], Federica Vento[4], Hiumei Wei[2], Caterina Matteucci[3], Shaohua Lei[2], Wojciech Rosikiewicz[2], Carlotta Nardelli[3], Anair Graciela Lema Fernandez[3], Anna Ostergaard[2], Emily Backhaus[2], Pradyuamna Baviskar[2], Marco Cerrano[5], Matteo Leoncin[6], Atsushi Manabe[7], Shinsuke Hirabayashi[7], Junko Takita[8], Daisuke Hasegawa[8], Satoshi Miyamoto[9], Antonio MacChiarulo[10], Jason Xu[11], David Teachey[12], Giovanni Roti[4], Ilaria Iacobucci[2], Roberta La Starza[3], Cristina Mecucci[3], Charles Mullighan[2]

*[1]University of Perugia, Chemistry, Biology and Biotechnology, Perugia, Italy; [2]St Jude Children's Research Hospital, Pathology, Memphis, United States of America; [3]University of Perugia, Department of Medicine, Perugia, Italy; [4]University of Parma, Department of Medicine and Surgery, Parma, Italy; [5]AOU Città della Salute e della Scienza - Presidio Molinette, Torino, Italy; [6]Hematology Unit, Ospedale dell'Angelo, Venice, Italy; [7]Hokkaido University Graduate School of Medicine, Hokkaido, Japan; [8]Graduate School of Medicine Kyoto University, Kyoto, Japan; [9]St. Luke's International Hospital, Tokyo, Japan; [10]University of Perugia, Department of Pharmaceutical Sciences, Perugia, Italy; [11]University of Pennsylvania, Philadelphia, United States of America; [12]Children's Hospital of Philadelphia, Philadelphia, United States of America;*

### Background

Despite advances in elucidating the genomic landscape of T-cell acute lymphoblastic leukemia (T-ALL) and lineage ambiguous acute leukemia, many cases still lack defining genetic hallmarks, complicating risk stratification and treatment. Notably, the discovery of recurrent genomic alterations that transcend conventional immunophenotypic boundaries supports the notion that classification based solely on immunophenotype remains limited, particularly when applied to acute leukemias of ambiguous lineage (ALAL), such as mixed-phenotype acute leukemia (MPAL), and to early T-cell precursor acute lymphoblastic leukemia (ETP-ALL) (Alexander et al. 2018; Polonen et al. 2024). Here we report a distinct genetic entity encompassing both MPAL and ETP-ALL, driven by a t(14;16)(q32;q24) translocation and aberrant expression of *FOXF1* and *FENDRR.*

### Aims

To define the molecular basis and therapeutic vulnerabilities of a novel high-risk subtype of immature T-ALL/MPAL driven by the t(14;16)(q32.2;q24) translocation.

### Methods

We retrospectively investigated 1831 T-ALL and MPAL cases by either cytogenetics or whole-genome sequencing (WGS). Somatic variants and copy number alterations were identified through whole-genome sequencing (WGS), custom next-generation sequencing, and single nucleotide polymorphism arrays (SNPa). RNA sequencing (RNA-seq) was used to define transcriptomic clustering, gene expression signatures, and pathway enrichment across 1,370 T-ALL cases. H3K27ac HiChIP and ATAC-seq were performed to examine chromatin architecture and accessibility, and single-cell DNA and RNA sequencing was performed to dissect clonal and transcriptional heterogeneity. Ex vivo drug screening using a 176-compound library identified candidate vulnerabilities, which were mechanistically validated by BH3 profiling and phospho-flow cytometry to assess apoptotic priming and pathway inhibition.

**Copyright Information:** (Online) ISSN: 2572-9241
©2026 The Author(s). HemaSphere is published by John Wiley & Sons Ltd on behalf of European Hematology Association. This is an open access Abstract Book distributed under the Attribution-NonCommercial-NoDerivs (CC BY-NC-ND), which allows third parties to download the articles and share them with others as long as they credit the author and the Abstract Book, but they cannot change the content in any way or use them commercially.
**Abstract Book Citations:** Authors, Title, HemaSphere, 2026;10:(S1):pages. The abstract book DOIs can be found on the "Author Information" page.
**Disclaimer:** Articles published in the journal HemaSphere exclusively reflect the opinions of the authors. The authors are responsible for all content in their abstracts including accuracy of the facts, statements, citing resources, etc.

### Results

We identified 13 ETP-ALL (n=9) and MPAL (n=4) cases harboring a recurrent t(14;16)(q32.2;q24) that drives ectopic *FOXF1* and *FENDRR* activation through enhancer hijacking of the *BCL11B* regulatory region. These cases exhibited a distinct gene expression profile with features of immature and myeloid potentials, consistent with developmental arrest at an early progenitor stage. Integrative analysis of ChIP-seq, H3K27ac HiChIP, and ATAC-seq demonstrated that FOXF1 occupies accessible, active regulatory regions and drives a transcriptional program that is specifically enriched in the *FOXF1/FENDRR*-ALL subtype compared to other T-ALL subtypes. Additional recurrent genomic features included bi-allelic *CDKN2A/B* deletions, *GATA3* loss-of-function mutations, and alterations in the NOTCH1 and JAK/STAT pathways. Single cell analyses suggested a model in which *FOXF1/FENDRR* deregulation is the primary oncogenic event, with *GATA3* inactivation restricting T-lineage maturation and enabling lineage plasticity. All cases showed an immature immunophenotype, with the expression of B-lymphoid markers as distinctive hallmark. All cases were refractory to front line therapies, but drug screening demonstrated synergistic effects of combined targeting of BCL-2 family proteins and the JAK/STAT pathway.

### Summary/Conclusion

The identification of *FOXF1/FENDRR* as oncogenic drivers in immature T-ALL and MPAL defines a novel high-risk leukemia subtype (*FOXF1/FENDRR*-ALL) marked by the t(14;16)(q32;q24) translocation. Its dismal prognosis calls for the implementation of tailored diagnostic strategies.

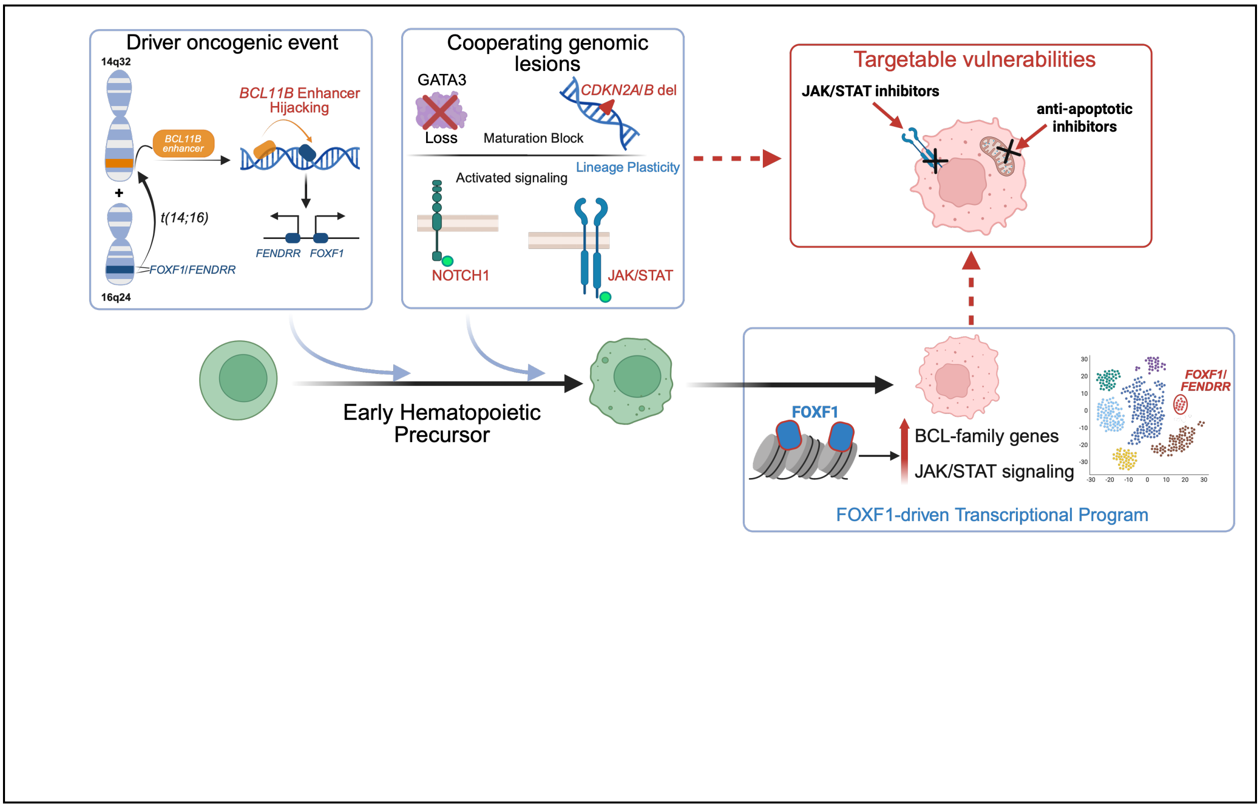

Schematic view of the genomic findings and therapeutic vulnerabilities defining the FOXF1/FENDRR-ALL subtype

**Copyright Information:** (Online) ISSN: 2572-9241
©2026 The Author(s). HemaSphere is published by John Wiley & Sons Ltd on behalf of European Hematology Association. This is an open access Abstract Book distributed under the Attribution-NonCommercial-NoDerivs (CC BY-NC-ND), which allows third parties to download the articles and share them with others as long as they credit the author and the Abstract Book, but they cannot change the content in any way or use them commercially.
**Abstract Book Citations:** Authors, Title, HemaSphere, 2026;10:(S1):pages. The abstract book DOIs can be found on the "Author Information" page.
**Disclaimer:** Articles published in the journal HemaSphere exclusively reflect the opinions of the authors. The authors are responsible for all content in their abstracts including accuracy of the facts, statements, citing resources, etc.

### (S108) NOTCHING UP LEUKEMIA-INITIATING CELLS TO ERADICATE T-ALL

**Topic:** 01. Acute lymphoblastic leukemia - Biology & translational research

Guillaume Andrieu*[1, 2], Mathieu Simonin[1, 3], Lucien Courtois[1, 2], Aurore Touzart[1, 2], El Mehdi Latiri[1, 2], Guillaume Hypolite[1, 2], Mélanie Féroul[1, 2], Guillaume Charbonnier[1, 2], Vincent Arzul[1], Estelle Balducci[1, 2], Els Verhoeyen[4, 5], Philippe Rousselot[6], Arnaud Petit[3], Hervé Dombret[7], Andre Baruchel[8], Nicolas Boissel[7], Olivier Hermine[9], Vahid Asnafi[1, 2]

*[1]Institut Necker-Enfants Malades INSERM U1151 CNRS Université de Paris, Team 'Normal and pathological lymphoid development', Paris, France; [2]Laboratoire d'Onco-Hématologie, Hôpital Necker-Enfants Malades, APHP, Paris, France; [3]Armand Trousseau Hospital, Assistance Publique-Hôpitaux de Paris, Sorbonne Université, Department of Pediatric Hematology and Oncology, Paris, France; [4]CIRI – International Center for Infectiology Research, Inserm, U1111, Université Claude Bernard Lyon 1, CNRS, UMR5308, Ecole Normale Supérieure de Lyon, Université Lyon, Lyon, France; [5]C3M, Université Côte d'Azur, INSERM, Nice, France; [6]Hemato-Oncology Unit, Centre Hospitalier de Versailles, Le Chesnay, France ; Université Versailles Saint Quentin en Yvelines, UMR1184 Université Paris-Saclay, Communauté Paris-Saclay, Versailles, France; [7]Hematology Laboratory and EA3518, University Hospital Saint-Louis, Université de Paris, Paris, France; [8]Department of Pediatric Hematology and Immunology, Assistance Publique-Hôpitaux de Paris (AP-HP), Robert Debré Hospital, University Paris Diderot, Paris, France; [9]Department of Hematology, Inserm U1163, IMAGINE Institute, Paris University, Necker Hospital, Paris, France;*

### Background

A central dogma in cancer biology posits that oncogenic activation confers selective advantages that promote tumor progression and therapy resistance. Oncogenic mutations are generally associated with poor clinical outcomes. Paradoxically, patients with NOTCH1-mutated T-cell acute lymphoblastic leukemia (T-ALL) exhibit superior treatment responses and reduced relapse compared with NOTCH1–wild-type cases, an observation that remains mechanistically unresolved. This NOTCH1 paradox challenges the prevailing view that oncogenes primarily enhance cancer cell fitness and therapy resistance. Relapse in leukemia is mainly driven by leukemia-initiating cells (LIC), a rare self-renewing population that survives chemotherapy by entering a quiescent state. However, the molecular mechanisms governing LIC dormancy and chemoresistance remain poorly defined. We postulated that NOTCH1 signaling acts as a rheostat controlling LIC cell-state dynamics and chemotherapy response, impacting disease relapse.

### Aims

To define how NOTCH1 signaling regulates LIC quiescence and chemoresistance in T-ALL and to determine whether forced activation of NOTCH1 can be exploited to eliminate LIC and prevent disease relapse.

### Methods

Primary T-ALL samples were used in an integrated approach combining patient-derived xenograft (PDX) models, single-cell transcriptomics, and functional assays. Single-cell RNA sequencing of primary samples and PDX was used to identify LIC using composite signatures. LIC quiescence was assessed in vivo by label-retention assays under induction-like chemotherapy (vincristine+dexamethasone). NOTCH1 signaling was genetically activated in NOTCH1–wild-type PDX by CRISPR-mediated mutation. Treatment effects were evaluated by flow cytometry, single-cell analyses, LIC content, monitoring of leukemia burden, relapse and survival in vivo.

**Copyright Information:** (Online) ISSN: 2572-9241
©2026 The Author(s). HemaSphere is published by John Wiley & Sons Ltd on behalf of European Hematology Association. This is an open access Abstract Book distributed under the Attribution-NonCommercial-NoDerivs (CC BY-NC-ND), which allows third parties to download the articles and share them with others as long as they credit the author and the Abstract Book, but they cannot change the content in any way or use them commercially.
**Abstract Book Citations:** Authors, Title, HemaSphere, 2026;10:(S1):pages. The abstract book DOIs can be found on the "Author Information" page.
**Disclaimer:** Articles published in the journal HemaSphere exclusively reflect the opinions of the authors. The authors are responsible for all content in their abstracts including accuracy of the facts, statements, citing resources, etc.

**Results**

Single-cell analyses demonstrated that NOTCH1 mutational status imprints distinct LIC cellular states. NOTCH1-altered T-ALL exhibited expanded LIC populations with high NOTCH1 signaling and active cell cycling, whereas NOTCH1–wild-type leukemias harbored a smaller LIC compartment with pronounced quiescence signatures. Functional in vivo modeling revealed that quiescent LIC were selectively enriched in NOTCH1–wild-type PDX under chemotherapy pressure, while LIC were depleted in NOTCH1-altered models, mirroring clinical relapse kinetics. Genetic induction of oncogenic NOTCH1 signaling in NOTCH1–wild-type LIC overrode quiescence, enforced proliferation, and restored chemosensitivity, resulting in marked LIC depletion by treatment. Importantly, activation of NOTCH1 using an identified drug combination induced robust pathway activation, triggered LIC proliferation, and synergized with standard chemotherapy to eliminate LIC and significantly prevent disease relapse in vivo.

**Summary/Conclusion**

This study establishes NOTCH1 as a rheostat controlling LIC dormancy, proliferation and chemotherapy response in T-ALL, providing a biological resolution to the longstanding NOTCH1 prognostic paradox. Beyond this, our findings support a paradigm shift in cancer biology by reinforcing that oncogenic signaling not solely enhance cancer cell fitness and therapy resistance, but can also impose enforced proliferation that exposes a critical vulnerability. By driving LIC out of protective quiescence, activation of NOTCH1 enables their eradication by chemotherapy, defining a mechanism-based strategy to eliminate therapy-resistant LIC and prevent disease relapse.

**Copyright Information:** (Online) ISSN: 2572-9241
©2026 The Author(s). HemaSphere is published by John Wiley & Sons Ltd on behalf of European Hematology Association. This is an open access Abstract Book distributed under the Attribution-NonCommercial-NoDerivs (CC BY-NC-ND), which allows third parties to download the articles and share them with others as long as they credit the author and the Abstract Book, but they cannot change the content in any way or use them commercially.
**Abstract Book Citations:** Authors, Title, HemaSphere, 2026;10:(S1):pages. The abstract book DOIs can be found on the "Author Information" page.
**Disclaimer:** Articles published in the journal HemaSphere exclusively reflect the opinions of the authors. The authors are responsible for all content in their abstracts including accuracy of the facts, statements, citing resources, etc.

### (S109) TUNING AND TARGETING CCR9 FOR THE TREATMENT OF T-ALL

**Topic:** 01. Acute lymphoblastic leukemia - Biology & translational research

Jamie Deyell*[1], Zaha Shahin[1], Yixi Zhang[1], Cliona McMahon[2], Paul Maclocia[1], Vijay Chudasama[2], Marc Mansour[1]

*[1]UCL, Cancer Institute, London, United Kingdom; [2]UCL Chemistry, London, United Kingdom;*

### Background

We previously identified CCR9 as an immunotherapy target for relapsed and refractory (r/r) T-ALL. Despite differential CCR9 expression in T-lymphoblasts, classical drawbacks of immunotherapy may limit its translational success. These include antigenic immune escape, relatively low and heterogenous CCR9 expression, and the complexities and toxicities associated with CAR-T therapies. Considering these challenges, maximising CCR9's potential requires antigen modulation strategies and alternative targeted therapies.

### Aims

1. **Define regulatory mechanisms of CCR9 in T-ALL,** potentially enabling identification of targetable pathways that modulate CCR9 expression
2. **Create a CCR9-directed antibody-drug conjugate (ADC)** as a simpler and more scalable alternative to CAR-T cells

### Methods

CRISPR screening was performed in a SUPT1 clone transduced with the genome-wide Brunello library, sorted by CCR9 expression, and analysed by MAGeCK-RRA. Quantitative flow cytometry used Quantibrite beads with PE-CCR9. ARID1A levels were assessed by western blotting. RNA- and ATAC-sequencing were analysed in-house.

An anti-CCR9 antibody was produced by gene gun rat vaccination and humanised. The ADC was generated via site-specific click-chemistry conjugation of tesirine to the anti-CCR9 antibody. In vitro killing assays used CellTiter-Glo and flow cytometry readouts. In vivo studies were carried out in immunodeficient mice using luciferised MOLT4 cells.

**Copyright Information:** (Online) ISSN: 2572-9241
©2026 The Author(s). HemaSphere is published by John Wiley & Sons Ltd on behalf of European Hematology Association. This is an open access Abstract Book distributed under the Attribution-NonCommercial-NoDerivs (CC BY-NC-ND), which allows third parties to download the articles and share them with others as long as they credit the author and the Abstract Book, but they cannot change the content in any way or use them commercially.
**Abstract Book Citations:** Authors, Title, HemaSphere, 2026;10:(S1):pages. The abstract book DOIs can be found on the "Author Information" page.
**Disclaimer:** Articles published in the journal HemaSphere exclusively reflect the opinions of the authors. The authors are responsible for all content in their abstracts including accuracy of the facts, statements, citing resources, etc.

**Results**

FACS-sorted CRISPR screening identified multiple members of the SWI/SNF complex and RUNX1/CBFB as positive regulators of CCR9 expression. In contrast, mTOR sgRNAs were enriched in CCR9-high cells, consistent with a suppressive role in controlling CCR9 expression.

Accordingly, torin1-based mTOR inhibition led to a striking increase (~2000%) in surface CCR9 expression in SUPT1 cells. These findings were recapitulated in other T-ALL cell lines, PDXs, and primary T-ALL. Torin1 did not cause clinically meaningful CCR9 induction in primary activated T-cells, indicating therapeutic selectivity.

To determine genes necessary for torin1-mediated CCR9 induction, a CCR9-based FACS CRISPR screen was executed after torin1 treatment. Significant genes included ARID1A, RUNX1, and the SWI/SNF--TF linker SMARCD1. The results indicate a tuneable SWI/SNF-SMARCD1-RUNX1 CCR9 regulatory axis.

Western blotting showed increased ARID1A protein levels with Torin1 treatment; RNA- and ATAC-seq showed increased expression of CCR9 and accessibility of the promoter after Torin1 treatment. Together, these findings support a model in which mTOR inhibition elevates ARID1A abundance post-translationally, thereby enhancing CCR9 promoter accessibility and expression.

In CCR9+ T-ALL cell lines, the CCR9-ADC demonstrated potent in vitro killing (IC50 <1nM) and log-fold selectivity for CCR9+ cells compared to isogenic CCR9KOs. These results were robust for PDX samples and primary T-ALL. Furthermore, a MOLT4-murine xenograft model showed reduced tumour burden and increased survival compared to non-targeting ADC ($p<0.0001$). Combining torin1 and the ADC yielded a significant ZIP synergy score in two-dimensional dosing.

**Summary/Conclusion**

The regulatory axis of CCR9 expression was uncovered in T-ALL. This can be modulated by use of mTOR inhibitors, increasing CCR9 via an ARID1A-RUNX1 dependent mechanism. Furthermore, we illustrate the pre-clinical efficacy of anti-CCR9 ADC for T-ALL therapy. These two discoveries synergise in vitro, suggesting a new method of tackling r/r disease.

**Copyright Information:** (Online) ISSN: 2572-9241
©2026 The Author(s). HemaSphere is published by John Wiley & Sons Ltd on behalf of European Hematology Association. This is an open access Abstract Book distributed under the Attribution-NonCommercial-NoDerivs (CC BY-NC-ND), which allows third parties to download the articles and share them with others as long as they credit the author and the Abstract Book, but they cannot change the content in any way or use them commercially.
**Abstract Book Citations:** Authors, Title, HemaSphere, 2026;10:(S1):pages. The abstract book DOIs can be found on the “Author Information” page.
**Disclaimer:** Articles published in the journal HemaSphere exclusively reflect the opinions of the authors. The authors are responsible for all content in their abstracts including accuracy of the facts, statements, citing resources, etc.

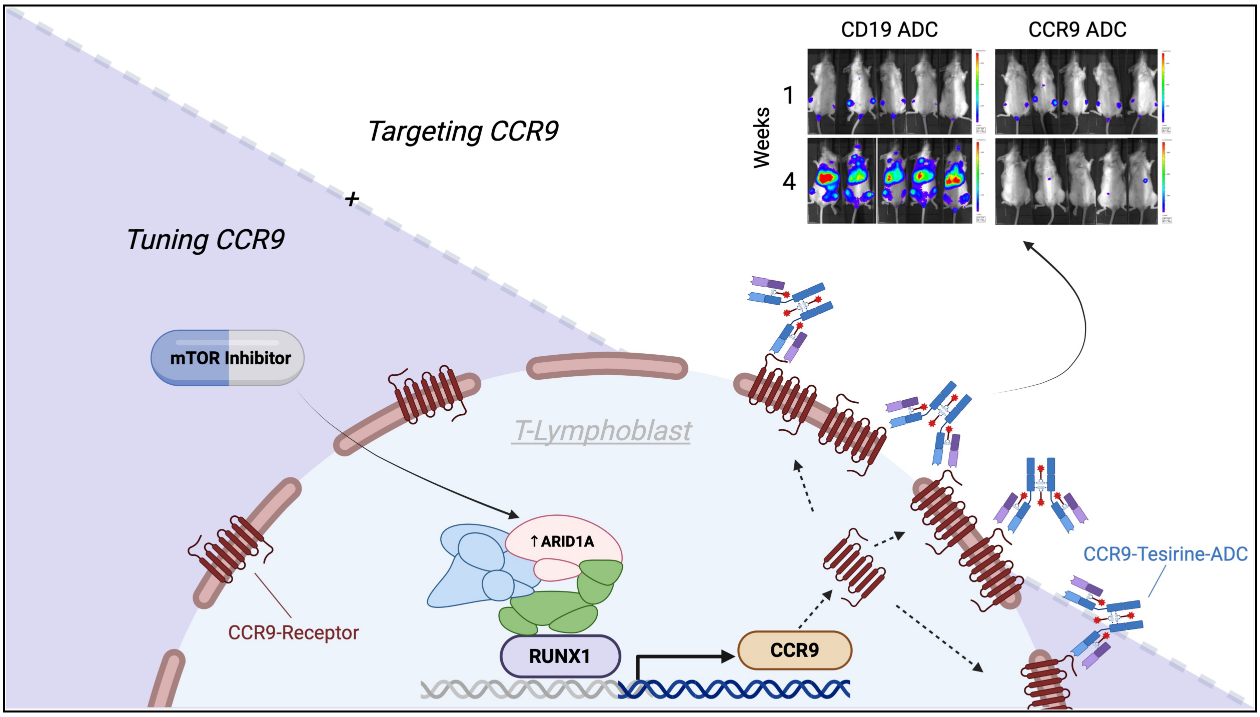

**Copyright Information:** (Online) ISSN: 2572-9241
©2026 The Author(s). HemaSphere is published by John Wiley & Sons Ltd on behalf of European Hematology Association. This is an open access Abstract Book distributed under the Attribution-NonCommercial-NoDerivs (CC BY-NC-ND), which allows third parties to download the articles and share them with others as long as they credit the author and the Abstract Book, but they cannot change the content in any way or use them commercially.
**Abstract Book Citations:** Authors, Title, HemaSphere, 2026;10:(S1):pages. The abstract book DOIs can be found on the "Author Information" page.
**Disclaimer:** Articles published in the journal HemaSphere exclusively reflect the opinions of the authors. The authors are responsible for all content in their abstracts including accuracy of the facts, statements, citing resources, etc.

### (S110) LONG-TERM OUTCOMES OF NATURALLY SELECTED CD7 CAR-T THERAPY IN RELAPSED/REFRACTORY T-LYMPHOBLASTIC LEUKEMIA/LYMPHOMA: A COHORT OF 171 PATIENTS

**Topic:** 02. Acute lymphoblastic leukemia - Clinical

Xian Zhang*[1], Jing Long[2], Junfang Yang[1], Jian Sun[3], Lin Wang[3], Peihua Lu[1]

*[1]Hebei yanda Ludaopei Hospital, Hematology, Langfang, China;[2]Beijing Ludaopei Hospital, Hematology, Beijing, China;[3]Hebei Senlang Biotechnology Co., Ltd.,, Shijiazhuang, China;*

### Background

Refractory and relapsed (R/R) T-lymphoblastic leukemia/lymphoma (T-ALL/LBL) is associated with poor clinical outcomes, whereas CD7-directed CAR-T therapy has demonstrated promising antitumor activity. In October 2020, our center initiated a clinical trial evaluating NS7 CAR-T cells (NCT04572308). Results from the first 60 enrolled patients showed high rates of remission, with bone marrow MRD negative CR or CRi achieved in 94.4% of patients.

### Aims

To report updated outcomes from an expanded and larger cohort of patients with R/R T-ALL/LBL treated with NS7 CAR-T therapy.

### Methods

NS7 CAR-T cells are naturally selected(NS) T cells without CD7 knockout or protein blockade, generated from autologous or prior donor T cells in post-allo-HSCT relapse. Patients received fludarabine (30 mg/m²) and cyclophosphamide (300 mg/m²) for 3 days before infusion. The median CAR-T dose was $1\times10^6$ cells/kg ($0.5–2\times10^6$). Consolidative allo-HSCT was allowed after day-30 evaluation. OS and PFS were estimated by the Kaplan–Meier method and compared using the log-rank test.

**Copyright Information:** (Online) ISSN: 2572-9241
©2026 The Author(s). HemaSphere is published by John Wiley & Sons Ltd on behalf of European Hematology Association. This is an open access Abstract Book distributed under the Attribution-NonCommercial-NoDerivs (CC BY-NC-ND), which allows third parties to download the articles and share them with others as long as they credit the author and the Abstract Book, but they cannot change the content in any way or use them commercially.
**Abstract Book Citations:** Authors, Title, HemaSphere, 2026;10:(S1):pages. The abstract book DOIs can be found on the "Author Information" page.
**Disclaimer:** Articles published in the journal HemaSphere exclusively reflect the opinions of the authors. The authors are responsible for all content in their abstracts including accuracy of the facts, statements, citing resources, etc.

### Results

From October 2020 to May 2025, 175 patients with R/R T-ALL/LBL enrolled in the CD7 CAR-T trial; 171 received infusion. Median follow-up was 846 days ( 62–1896). At day 30 post-infusion, 155/171 (90.6%) achieved BM CR/CRi, including 152/171 (88.8%) with MRD-negative CR/CRi. (Table1)

| | Caption |
|---|---|
| Total CR+CRi | 155/171 (90.6%),<br>MRD(-)CR 152/171 (88.8%) |
| Time from onset to progression<br>(≤1 year vs.[illegible]1 year) | 115/128 (89.8%) vs.40/43 (93.0%) |
| With history of Allo HSCT vs. without | 37/40 (92.5%) vs. 118/131 (90.1%) |
| With SIL::TAL1 fusion gene vs. without | 3/4 (75%) vs.152/167 (91.0%) |
| With TP53 mutation/17p- vs. without | 9/9 (100%) vs. 146/162 (90.1%) |

Among 155 CR/CRi pts, 2-year OS and PFS were 79% and 73.9%, respectively (Fig 1A). Consolidative allo-HSCT was performed in 137 patients (88.4%) at a median of 57 days (28–227) post–CAR-T. Whereas 18 did not undergo HSCT; among these, 8 relapsed (median 124 days; range, 60–183) and 14 died. Consolidative allo-HSCT after CART was associated with superior outcomes versus no transplantation (2-year OS: 84.4% vs. 38.1%; PFS: 80% vs. 25.9%; both p<0.001; Fig 1B–C).
Stratification by time to progression before CART (≤1 year, n=115 vs. >1 year, n=40) showed no differences in 2-year OS (81.6% vs. 77.5%, p=0.219) or PFS (75.3% vs. 69.8%, p=0.398); however, Consolidative allo-HSCT after CAR-T improved survival in both groups.
Patients with prior allo-HSCT (n=37) had inferior 2-year OS compared with those without (66.8% vs. 82.8%, p=0.008, Fig 1D), while PFS showed a similar trend (64.5% vs. 76.8%, p=0.073). In previously transplanted patients, bridging allo-HSCT (n=27 vs. 10) markedly improved 2-year OS (81.1% vs. 30%) and PFS (80.2% vs. 20%) (both p<0.001).
Among CR/CRi cases, 3 patients harbored SIL::TAL1 fusions, including 2 early deaths after relapse without bridging allo-HSCT and 1 survivor at day 249. TP53 mutations were detected in 9 patients; 8 underwent transplantation, 3 died, and 1 non-transplanted patient died at day 62. TP53-mutant cases showed numerically lower 2-year OS (55.6% vs. 80.5%, p=0.079) and PFS (55.6% vs. 75%, p=0.114) than wild type cases.

### Summary/Conclusion

This study, analyzing 171 cases, shows that CD7 CAR-T therapy at disease progression in relapsed/refractory T-ALL/LBL yields high CR rates—especially in patients with SIL::TAL1 fusion, TP53 mutation, del(17p), or progression within one year. After achieving CR, maintenance is brief; allogeneic HSCT should follow promptly to improve long-term OS and PFS.

**Copyright Information:** (Online) ISSN: 2572-9241
©2026 The Author(s). HemaSphere is published by John Wiley & Sons Ltd on behalf of European Hematology Association. This is an open access Abstract Book distributed under the Attribution-NonCommercial-NoDerivs (CC BY-NC-ND), which allows third parties to download the articles and share them with others as long as they credit the author and the Abstract Book, but they cannot change the content in any way or use them commercially.
**Abstract Book Citations:** Authors, Title, HemaSphere, 2026;10:(S1):pages. The abstract book DOIs can be found on the "Author Information" page.
**Disclaimer:** Articles published in the journal HemaSphere exclusively reflect the opinions of the authors. The authors are responsible for all content in their abstracts including accuracy of the facts, statements, citing resources, etc.

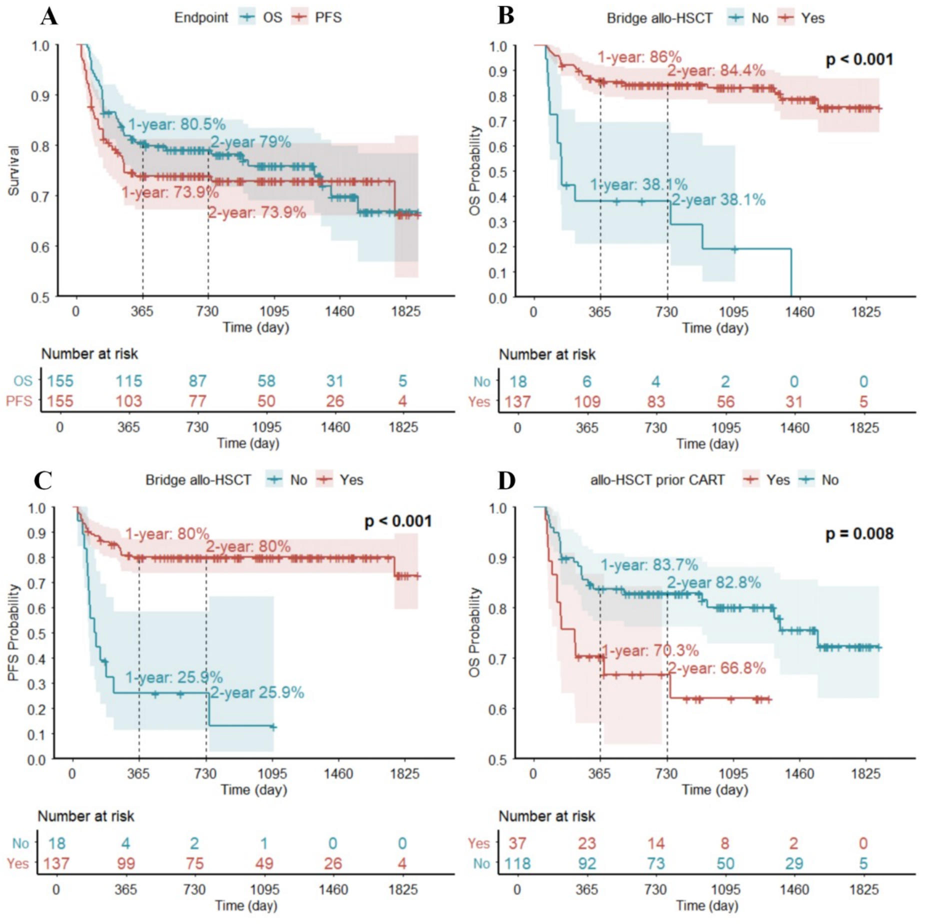

**Fig 1. Overall survival curves of the 155 CR patients and subgroups**

**Copyright Information:** (Online) ISSN: 2572-9241
©2026 The Author(s). HemaSphere is published by John Wiley & Sons Ltd on behalf of European Hematology Association. This is an open access Abstract Book distributed under the Attribution-NonCommercial-NoDerivs (CC BY-NC-ND), which allows third parties to download the articles and share them with others as long as they credit the author and the Abstract Book, but they cannot change the content in any way or use them commercially.
**Abstract Book Citations:** Authors, Title, HemaSphere, 2026;10:(S1):pages. The abstract book DOIs can be found on the “Author Information” page.
**Disclaimer:** Articles published in the journal HemaSphere exclusively reflect the opinions of the authors. The authors are responsible for all content in their abstracts including accuracy of the facts, statements, citing resources, etc.

### (S111) ANTIBODY-BASED IMMUNOTHERAPY VERSUS CELLULAR THERAPY AS BRIDGING TO ALLOGENEIC STEM CELL TRANSPLANTATION IN RELAPSED/REFRACTORY OR MRD-POSITIVE B-CELL ACUTE LYMPHOBLASTIC LEUKEMIA

**Topic:** 02. Acute lymphoblastic leukemia - Clinical

Ao-Ran Zhang*[1], Guan-Hua Hu[1], Ying-Xi Zuo[1], Ting Zhao[1], Ying Wu[1], Xian-Ying Yin[1], Shang Qiannan[1], Xiao-Dong Mo[1], Yuqian Sun[1], Yu Wang[1], Lanping Xu[1], Xiaohui Zhang[1], Xiangyu Zhao[1], Xiao-Jun Huang[1], Yi-Fei Cheng[1], Meng Lv[1]

*[1]Peking University People's Hosptial, Beijing, China;*

### Background

Blinatumomab (BLI), inotuzumab ozogamicin (INO), and CAR-T therapy have reshaped the treatment landscape of relapsed/refractory (R/R) and MRD$^+$ B-ALL by facilitating transplantation eligibility. Yet, the optimal pre-HSCT strategy and its influence on post-transplant toxicity and survival remain undefined.

### Aims

To directly compare BLI, INO, and CAR-T as pre-transplant strategies in R/R or MRD$^+$ B-ALL and to identify determinants of post-HSCT outcomes.

### Methods

A consecutive cohort of patients with R/R or MRD$^+$ B-ALL who proceeded to HSCT following antibody-based immunotherapy (BLI/INO) or CAR-T therapy was analyzed. Patients were first stratified by disease status at initiation of bridging therapy (R/R vs. MRD$^+$), and then by initial treatment strategy (immunotherapy vs. cellular). Patients who failed monotherapy and subsequently received ≥2 lines of cellular or immunotherapy were analyzed as a separate multi-line cohort.

### Results

**A total of 286 patients were included, of whom 149(52%) were MRD$^+$ and 137(48%) had active R/R disease** before immune or cellular therapy.

In the R/R cohort, cellular therapy (n=78) was associated with significantly superior post-HSCT outcomes compared with immunotherapy (n=41), including 3-year OS (90% vs. 69%, *P*=0.022), DFS (72% vs. 48%, *P*=0.040), and GRFS (63% vs. 34%, *P*=0.018). Multivariable analysis confirmed **immunotherapy as an independent adverse factor for R/R ALL** in OS (HR 3.65, *P*=0.022), DFS (HR 2.06, *P*=0.032), and GRFS (HR 2.00, *P*=0.019).

**In the MRD$^+$ cohort, OS and DFS were comparable between cellular therapy (n=49) and immunotherapy (n=79)**; however, both strategies demonstrated superior OS compared with multi-line therapy (n=21) (cellular vs. multi-line: 86% vs. 61%, *P*=0.006; immunotherapy vs. multi-line: 85% vs. 61%, *P*<0.001). Multi-line therapy was independently associated with inferior OS (HR 5.02, *P*=0.019) and DFS (HR 2.39, *P*=0.044).

In the overall cohort, persistent pre-HSCT FCM-MRD was strongly associated with inferior survival outcomes, including 3-year OS (47% vs. 86%, *P*<0.001), DFS (29% vs. 70%, *P*=0.001), GRFS (31% vs. 58%, *P*=0.050), and higher NRM (22% vs. 5%, *P*=0.013). Regarding post-HSCT complications, **mono-cellular therapy** (n=127) was associated with higher 1-year incidence of **grade II–IV aGVHD** (38% vs. 25%, *P*=0.007) and **CMV reactivation** (51% vs. 37%, *P*=0.021) compared with mono-immunotherapy (n=120);

**Copyright Information:** (Online) ISSN: 2572-9241
©2026 The Author(s). HemaSphere is published by John Wiley & Sons Ltd on behalf of European Hematology Association. This is an open access Abstract Book distributed under the Attribution-NonCommercial-NoDerivs (CC BY-NC-ND), which allows third parties to download the articles and share them with others as long as they credit the author and the Abstract Book, but they cannot change the content in any way or use them commercially.
**Abstract Book Citations:** Authors, Title, HemaSphere, 2026;10:(S1):pages. The abstract book DOIs can be found on the "Author Information" page.
**Disclaimer:** Articles published in the journal HemaSphere exclusively reflect the opinions of the authors. The authors are responsible for all content in their abstracts including accuracy of the facts, statements, citing resources, etc.

however, MVA showed that treatment modality was not independently associated with CMV or EBV reactivation or GVHD.

| | | | | | |
|---|---|---|---|---|---|
| **Median age at HSCT (Range)** | **17.5 (2-69)** | **Exposure to therapy, n(%)** | | **History of CNSL, n(%)** | 37 (13) |
| **Sex, n(%)** | | Blinatumomab | 135 (47) | **History of testicular leukemia, n(%)** | 6 (2) |
| Male | 159 (56) | CAR-T | 157 (55) | **Other EMD (excluding CNSL), n(%)** | 23 (8) |
| Female | 127 (44) | Inotuzumab | 29 (10) | **MRD status before HSCT, n(%)** | 17 (6) |
| **Diseases, n(%)** | | **Type of Therapy, n(%)** | | **TOP3 Fusion genes, n(%)** | |
| B-ALL | 275 (96) | BLI only | 109 (38) | Contains E2A | 14 (5) |
| B-LBL | 4 (1) | CAR-T only | 127 (44) | Contains MLL | 10 (4) |
| CML-LBC | 5 (2) | INO only | 11 (4) | Contains ZNF384 | 8 (3) |
| MPAL | 2 (1%) | Blinatumomab + CAR-T | 15 (5) | **TOP3 mutations, n(%)** | |
| **Ph chromosome, n(%)** | | BLI + INO | 7 (2) | IKZF1+ | 69 (24) |
| Ph+ | 69 (24) | CAR-T + INO | 5 (2) | CRLF2+ | 22 (8) |
| Ph- | 217 (76) | Blinatumomab + CAR-T + Inotuzumab | 4 (1) | PAX5+ | 20 (7) |

### Summary/Conclusion

Cellular therapy confers a survival advantage over antibody-based immunotherapy when used as bridging to HSCT in patients with active R/R B-ALL, whereas comparable outcomes are observed in MRD$^{+}$ patients. Enhanced surveillance and management of CMV reactivation and aGVHD are warranted following CAR-T–bridged transplantation.

**Copyright Information:** (Online) ISSN: 2572-9241
©2026 The Author(s). HemaSphere is published by John Wiley & Sons Ltd on behalf of European Hematology Association. This is an open access Abstract Book distributed under the Attribution-NonCommercial-NoDerivs (CC BY-NC-ND), which allows third parties to download the articles and share them with others as long as they credit the author and the Abstract Book, but they cannot change the content in any way or use them commercially.
**Abstract Book Citations:** Authors, Title, HemaSphere, 2026;10:(S1):pages. The abstract book DOIs can be found on the “Author Information” page.
**Disclaimer:** Articles published in the journal HemaSphere exclusively reflect the opinions of the authors. The authors are responsible for all content in their abstracts including accuracy of the facts, statements, citing resources, etc.

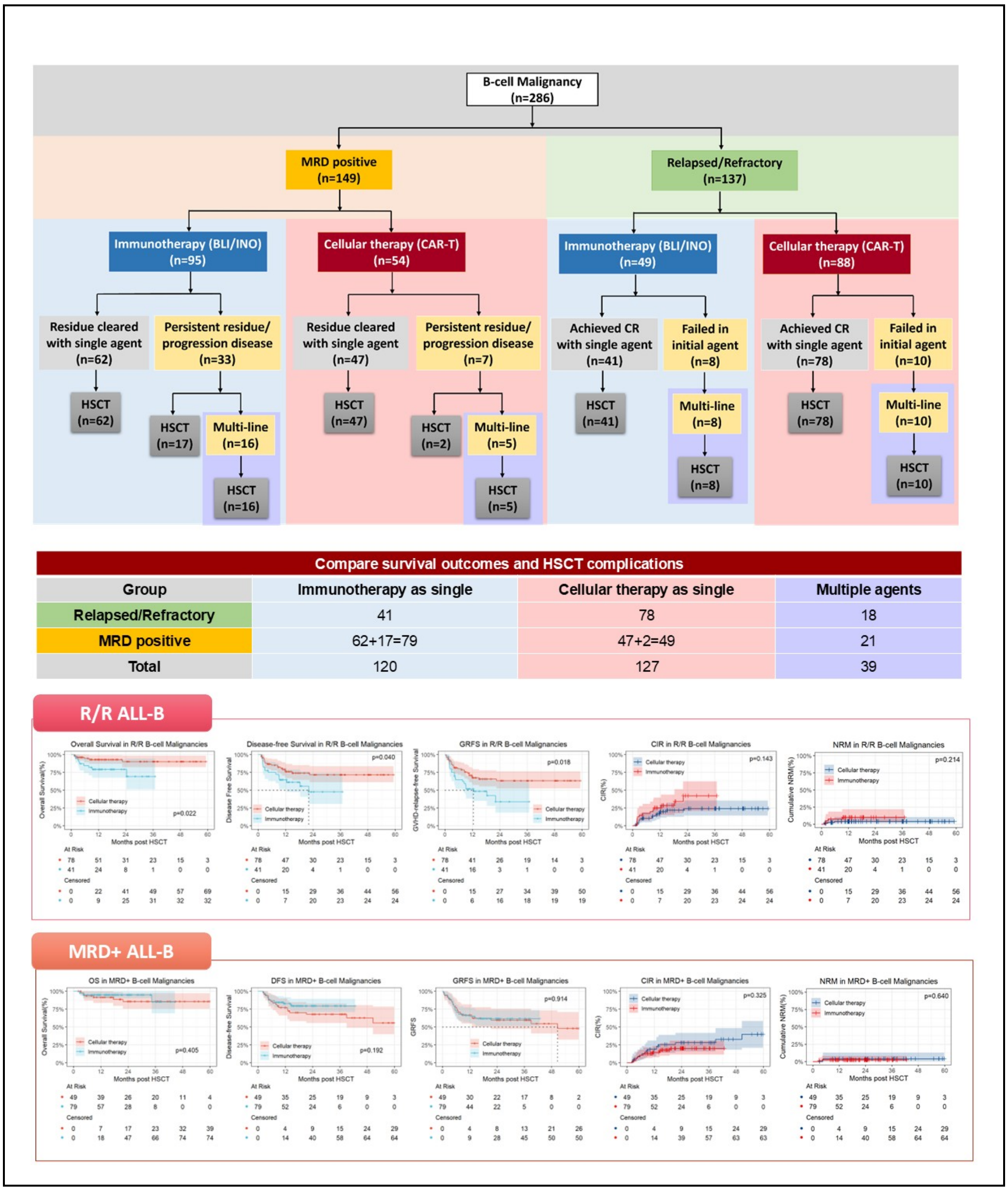

| Compare survival outcomes and HSCT complications | | | |
|---|---|---|---|
| Group | Immunotherapy as single | Cellular therapy as single | Multiple agents |
| Relapsed/Refractory | 41 | 78 | 18 |
| MRD positive | 62+17=79 | 47+2=49 | 21 |
| Total | 120 | 127 | 39 |

**Copyright Information:** (Online) ISSN: 2572-9241
©2026 The Author(s). HemaSphere is published by John Wiley & Sons Ltd on behalf of European Hematology Association. This is an open access Abstract Book distributed under the Attribution-NonCommercial-NoDerivs (CC BY-NC-ND), which allows third parties to download the articles and share them with others as long as they credit the author and the Abstract Book, but they cannot change the content in any way or use them commercially.
**Abstract Book Citations:** Authors, Title, HemaSphere, 2026;10:(S1):pages. The abstract book DOIs can be found on the "Author Information" page.
**Disclaimer:** Articles published in the journal HemaSphere exclusively reflect the opinions of the authors. The authors are responsible for all content in their abstracts including accuracy of the facts, statements, citing resources, etc.

### (S112) COMPARISON OF END INDUCTION FLOW CYTOMETRY VERSUS HIGH-THROUGHPUT SEQUENCING BASED MINIMAL RESIDUAL DISEASE IN NEWLY DIAGNOSED CHILDHOOD STANDARD RISK B ALL: A REPORT FROM COG AALL1731

**Topic:** 02. Acute lymphoblastic leukemia - Clinical

Sumit Gupta*[1], John Kairalla[2], Karen Rabin[3], Ilan Kirsch[4], Brent Wood[5], Emily Hibbits[2], Andrew Carroll[6], Lia Gore[7], Olga Militano[8], Yvonne Moyer[9], Shalini Reshmi[9], Mary Shago[1], Elizabeth Wagner[9], Tara Haworth-Wright[10], Naomi Winick[11], Faraz Zaman[12], Gerhard Zugmaier[13], Meenakshi Devidas[14], Stephen Hunger[15], David Teachey[15], Elizabeth Raetz[16], Mignon Loh[10], Rachel Rau[10]

*[1]Hospital for Sick Children, Toronto, Canada;[2]University of Florida, Gainesville, United States of America;[3]UCSF, San Francisco, United States of America; [4]Adaptive Biotechnologies, Seattle, United States of America; [5]Children's Hospital Los Angeles, Los Angeles, United States of America; [6]University of Alabama Birmingham, Birmingham, United States of America;[7]Children's Hospital Colorado, Colorado Springs, United States of America; [8]Children's Oncology Group, Monrovia, United States of America; [9]Nationwide Children's Hospital, Columbus, United States of America; [10]Seattle Childrens Hospital, Seattle, United States of America; [11]Ut Southwestern, Dallas, United States of America; [12]Amgen, Inc., Thousand Oaks, United States of America; [13]Amgen Research, München, Germany; [14]Saint Jude Children's Research Hospital, Memphis, United States of America; [15]Children's Hospital of Philadelphia, Philadelphia, United States of America; [16]NYU Langone Health, New. York City, United States of America;*

### Background

The Children's Oncology Group (COG) AALL1731 trial showed that adding blinatumomab (blina) to chemotherapy (chemo) dramatically improved disease-free survival (DFS) in NCI standard risk B-ALL at average or higher risk of relapse (Gupta, et al. NEJM 2025). On AALL1731, minimal residual disease (MRD) was determined among patients (pts) at average relapse risk (SR-AVG) by both flow cytometry (fc) and high-throughput sequencing (HTS) of immunoglobulin loci. Though retrospective data suggests pts with undetectable HTS MRD have outstanding outcomes, the role of HTS MRD is still being defined. We thus evaluated its prognostic impact, the significance of fc-HTS MRD discordance, and how this varied +/-blina.

### Aims

We aimed to: 1) Compare the DFS of SR-AVG pts with end of induction (EOI) bone marrow (BM) fcMRD <0.01% and undetectable HTS MRD non-randomly assigned to chemo to SR-AVG pts, with detectable HTS MRD randomized to chemo, and 2) Define the DFS of pts with MRD discordance (fcMRD <0.01%, but ≥0.01% by HTS) when treated with chemo +/- blina.

### Methods

AALL1731 enrolled *de novo* NCI SR (age 1-9.9 yrs with initial white blood cell count <50,000/µL) B-ALL pts, without *BCR::ABL1,* CNS3, or testicular disease. After 3-drug induction, pts were assigned to risk groups based on somatic genetics, central nervous system (CNS) status, day 8 blood fcMRD, and EOI BM fcMRD.
Patients at average relapse risk (SR-AVG) with EOI BM fcMRD <0.01% and undetectable HTS MRD were non-randomly assigned to standard-intensity chemo. All other SR-AVG patients were eligible for randomization to standard chemo +/- two cycles of blina. COG-approved labs performed fcMRD. Adaptive Biotechnologies determined clonality from diagnostic BM samples and HTS-MRD from EOI BM using the ClonoSEQ assay. Post-consolidation DFS was compared between groups using log-rank tests, with data current to Dec 2025.

**Copyright Information:** (Online) ISSN: 2572-9241
©2026 The Author(s). HemaSphere is published by John Wiley & Sons Ltd on behalf of European Hematology Association. This is an open access Abstract Book distributed under the Attribution-NonCommercial-NoDerivs (CC BY-NC-ND), which allows third parties to download the articles and share them with others as long as they credit the author and the Abstract Book, but they cannot change the content in any way or use them commercially.
**Abstract Book Citations:** Authors, Title, HemaSphere, 2026;10:(S1):pages. The abstract book DOIs can be found on the "Author Information" page.
**Disclaimer:** Articles published in the journal HemaSphere exclusively reflect the opinions of the authors. The authors are responsible for all content in their abstracts including accuracy of the facts, statements, citing resources, etc.

### Results

Of 914 SR-AVG pts with EOI fcMRD <0.01% who had EOI HTS MRD attempted, 162 (17.7%) had undetectable HTS MRD, 705 (77.1%) were detectable, 23 (2.5%) lacked a clonal Ig sequence, 15 (1.6%) had inadequate samples, and 9 (1%) had no available sample.
Median follow-up was 3.9 yrs. While overall, events were rare, the 4yr post-consolidation DFS of pts with undetectable HTS MRD (N=160) was not significantly superior to SR-AVG pts with detectable HTS MRD who received chemo alone (N=352)(94.9±2.7% vs 90.4±2.2%; p=0.37; Figure). Amongst SR-AVG pts, those with detectable HTS MRD treated with chemo + blina (N=353) had the best 4yr DFS of 95.5±1.6% (p=0.049).
The 4yr DFS of pts with MRD discordance (fcMRD <0.01% but HTS MRD ≥0.01%; N=79) was inferior to that of concordant (fcMRD and HTS MRD <0.01%; N=626) pts (85.4±5.4% vs. 93.9%±1.4%; p=0.005). However, this outcome discrepancy was 80.0% (N=33) vs. 91.5% (N=319)(p=0.02) in pts randomized to chemo, compared to 89.4% (N=46) vs. 96.4% (N=307)(p=0.05) in pts randomized to blina.

### Summary/Conclusion

Among SR-AVG patients with EOI fcMRD <0.01%, undetectable EOI HTS MRD did not confer superior DFS; these pts may thus also benefit from the addition of blina to therapy. However, pts with discordantly high HTS MRD ≥0.01% experienced inferior outcomes despite fcMRD negativity. While such pts benefited from the addition of blina, future studies should aim to further optimize treatment for this group through intensification of chemo (as is standard for fcMRD ≥0.01%) and/or optimization of immunotherapy.

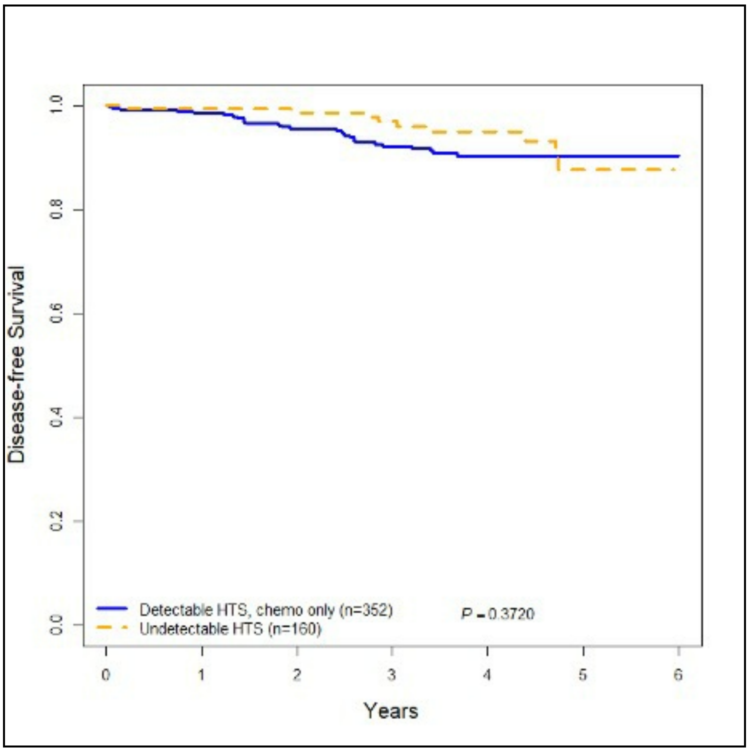

Disease-free survival of patients with undetectable end Induction bone marrow HTS MRD vs. patients with detectable HTS MRD randomized to chemotherapy only

**Copyright Information:** (Online) ISSN: 2572-9241
©2026 The Author(s). HemaSphere is published by John Wiley & Sons Ltd on behalf of European Hematology Association. This is an open access Abstract Book distributed under the Attribution-NonCommercial-NoDerivs (CC BY-NC-ND), which allows third parties to download the articles and share them with others as long as they credit the author and the Abstract Book, but they cannot change the content in any way or use them commercially.
**Abstract Book Citations:** Authors, Title, HemaSphere, 2026;10:(S1):pages. The abstract book DOIs can be found on the "Author Information" page.
**Disclaimer:** Articles published in the journal HemaSphere exclusively reflect the opinions of the authors. The authors are responsible for all content in their abstracts including accuracy of the facts, statements, citing resources, etc.

### (S113) IMPROVING EFFICACY AND SAFETY OUTCOMES IN PATIENTS (PTS) WITH RELAPSED/REFRACTORY B-CELL ACUTE LYMPHOBLASTIC LEUKEMIA IN MORPHOLOGIC REMISSION TREATED WITH OBECABTAGENE AUTOLEUCEL (OBE-CEL)

**Topic:** 02. Acute lymphoblastic leukemia - Clinical

Claire Roddie*[1], Jae H. Park[2], Karamjeet S. Sandhu[3], Bijal D. Shah[4], Eleni Tholouli[5], Paul Shaughnessy[6], Pere Barba[7], Manuel Guerreiro[8], Deborah Yallop[9], Aaron Logan[10], Mehrdad Abedi[11], Michael Bishop[12], Daniel J. Deangelo[13], Pierre Lao-Sirieix[14], Ping Wang[15], Wolfram Brugger[16], Karl Peggs[1], Elias Jabbour[17]

*[1]University College London Cancer Institute, London, United Kingdom; [2]Memorial Sloan Kettering Cancer Center, New York, United States of America; [3]City of Hope National Medical Center, Duarte, United States of America;[4]Moffitt Cancer Center, Tampa, United States of America; [5]Manchester Royal Infirmary, Manchester, United Kingdom;[6]Sarah Cannon Transplant and Cellular Therapy Program, Methodist Hospital, San Antonio, United States of America; [7]Hospital Universitari Vall d'Hebron-Universitat Autónoma de Barcelona, Barcelona, Spain; [8]Hospital Universitari i Politècnic La Fe, Valencia, Spain;[9]King's College Hospital, NHS Foundation Trust, London, United Kingdom; [10]Hematology, Blood and Marrow Transplantation, and Cellular Therapy Program, University of California San Francisco, San Francisco, United States of America; [11]University of California Davis, Davis, United States of America; [12]The David and Etta Jonas Center for Cellular Therapy, University of Chicago, Chicago, United States of America; [13]Department of Medical Oncology, Dana-Farber Cancer Institute, Boston, United States of America; [14]Autolus Therapeutics, London, United Kingdom; [15]Autolus Therapeutics, Rockville, United States of America; [16]Autolus Therapeutics, Weil am Rhein, Germany; [17]University of Texas MD Anderson Cancer Center, Houston, United States of America;*

### Background

Low disease burden (<5% bone marrow [BM] blasts) prior to chimeric antigen receptor (CAR) T-cell therapy is associated with longer survival compared with high disease burden (≥5% BM blasts) in pts with relapsed/refractory B-cell acute lymphoblastic leukemia (R/R B-ALL; Roddie et al, *NEJM* 2024). Obe-cel is an autologous, fast off-rate 4-1BB-ζ CD19-directed CAR T-cell therapy that demonstrated efficacy in adult R/R B-ALL in the Phase Ib/II FELIX study (NCT04404660). Characterization of the effect of bridging therapy (BT) and outcomes in pts based on morphologic response at the time of obe-cel infusion is required.

### Aims

To report a post-hoc analysis characterizing long-term EFS and safety in adults with R/R B-ALL in FELIX by disease burden at both screening and lymphodepletion (LD) following BT.

### Methods

All pts provided informed consent. Following leukapheresis, pts received BT at the discretion of the investigator to reduce BM blasts prior to LD. Disease burden was assessed at screening and within seven days prior to LD by local morphology. Following LD, obe-cel was administered using tumor burden-guided dosing to minimize immunotoxicities. EFS was analyzed using Kaplan-Meier methodology.

**Copyright Information:** (Online) ISSN: 2572-9241
©2026 The Author(s). HemaSphere is published by John Wiley & Sons Ltd on behalf of European Hematology Association. This is an open access Abstract Book distributed under the Attribution-NonCommercial-NoDerivs (CC BY-NC-ND), which allows third parties to download the articles and share them with others as long as they credit the author and the Abstract Book, but they cannot change the content in any way or use them commercially.
**Abstract Book Citations:** Authors, Title, HemaSphere, 2026;10:(S1):pages. The abstract book DOIs can be found on the "Author Information" page.
**Disclaimer:** Articles published in the journal HemaSphere exclusively reflect the opinions of the authors. The authors are responsible for all content in their abstracts including accuracy of the facts, statements, citing resources, etc.

**Results**

Of 127 pts treated with obe-cel, ten (7.9%) had low disease burden at both screening and LD (Low/Low [LL]), nine (7.1%) had low disease burden at screening and high disease burden at LD (Low/High [LH]), 26 (20.5%) had high disease burden at screening and low disease burden at LD (High/Low [HL]), and 82 (64.6%) had high disease burden at both screening and LD (High/High [HH]). Overall, 118/127 (92.9%) pts received BT (**Fig. A**). Most (85.4%) pts in the HH group received BT containing chemotherapy; 38.5% of pts in the HL group received BT containing inotuzumab ozogamicin. At 32.8 months' median follow-up (range 19.9–52.8), median EFS (95% CI) was not yet reached in the LL, LH, and HL groups, and was 9.0 months (5.1–12.3) in the HH group (**Fig. B**). The 24-month EFS probabilities (95% CI) were 90.0% (47.3–98.5), 66.7% (28.2–87.8), 57.6% (35.3–74.7), and 29.3% (18.3–41.1) in the LL, LH, HL, and HH groups, respectively. At last follow-up, ≥50% of pts in the LL, LH, and HL groups and 20% of pts in the HH group, were in ongoing remission without additional anti-cancer therapies, excluding tyrosine kinase inhibitors. No pts in the LH group received post obe-cel stem-cell transplant (SCT); 40.0%, 11.5%, and 13.4% of pts in the LL, HL, and HH groups received post obe-cel SCT, respectively. Grade ≥3 cytokine release syndrome (CRS) incidence was 0% in the LL, LH, and HL groups, and 3.7% in the HH group. Grade ≥3 immune effector cell-associated neurotoxicity syndrome (ICANS) incidence was 0%, 11.1%, 0% and 9.8% in the LL, LH, HL, and HH groups, respectively. Incidence of any-grade CRS and ICANS was lower in pts with low disease burden, particularly those with low disease burden at LD, compared with pts with high disease burden.

**Summary/Conclusion**

Obe-cel treatment demonstrated a therapeutic effect in pts regardless of disease burden at screening or LD. Comparable EFS and low incidence of CRS/ICANS were observed in pts who achieved low disease burden before or after BT. The greatest effect appeared to be in pts with low disease burden at both screening and LD. These data suggest that best outcomes are achieved with obe-cel when disease burden is low or can be effectively reduced. Outcomes in pts in the HH group may indicate high-risk biology or a need to further optimize their BT.

**Copyright Information:** (Online) ISSN: 2572-9241
©2026 The Author(s). HemaSphere is published by John Wiley & Sons Ltd on behalf of European Hematology Association. This is an open access Abstract Book distributed under the Attribution-NonCommercial-NoDerivs (CC BY-NC-ND), which allows third parties to download the articles and share them with others as long as they credit the author and the Abstract Book, but they cannot change the content in any way or use them commercially.
**Abstract Book Citations:** Authors, Title, HemaSphere, 2026;10:(S1):pages. The abstract book DOIs can be found on the "Author Information" page.
**Disclaimer:** Articles published in the journal HemaSphere exclusively reflect the opinions of the authors. The authors are responsible for all content in their abstracts including accuracy of the facts, statements, citing resources, etc.

**Figure A.** Patient and disease characteristics by disease burden group

| | LL (n=10) | LH (n=9) | HL (n=26) | HH (n=82) |
|---|---|---|---|---|
| **Age in years, median (range)** | 34.5 (21–73) | 43.0 (23–67) | 42.5 (21–72) | 50.5 (20–81) |
| **BM blasts, median (range)** | | | | |
| Screening | 0.5 (0–3) | 1.6 (0–3) | 53.6 (5–96) | 64.5 (6–100) |
| LD | 0.7 (0–3) | 40.0 (5–96) | 1.0 (0–5) | 65.5 (5–100) |
| **Bridging therapy received, n (%)** | 9 (90.0) | 7 (77.8) | 26 (100) | 76 (92.7) |
| Chemotherapy | 6 (60.0) | 5 (55.6) | 8 (30.8) | 61 (74.4) |
| Chemotherapy + TKI | 1 (10.0) | 0 | 4 (15.4) | 5 (6.1) |
| Chemotherapy + InO | 0 | 1 (11.1) | 4 (15.4) | 4 (4.9) |
| InO | 0 | 0 | 6 (23.1) | 3 (3.7) |
| TKI | 2 (20.0) | 1 (11.1) | 3 (11.5) | 1 (1.2) |
| Steroids | 0 | 0 | 1 (3.8) | 1 (1.2) |
| Other | 0 | 0 | 0 | 1 (1.2) |

**Figure B.** Event-free survival* by disease burden group

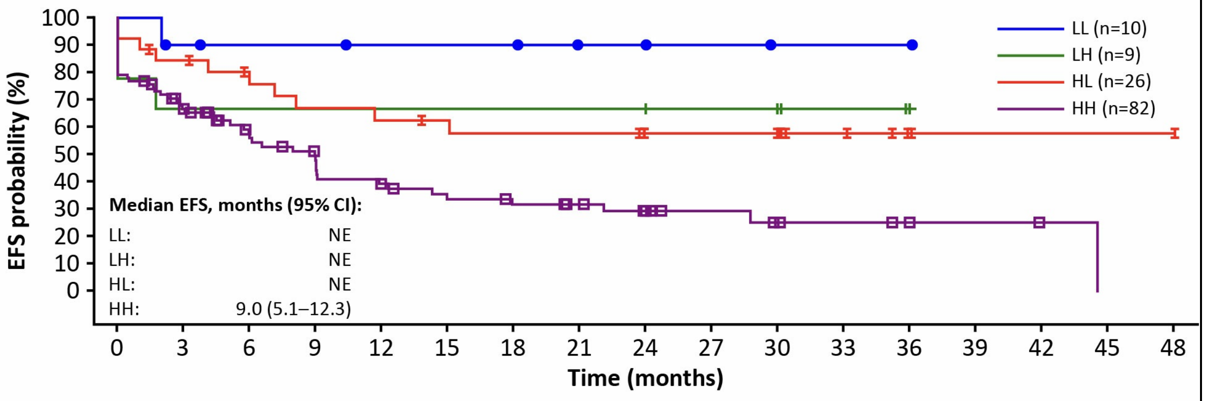

**Patients at risk**

| | 0 | 3 | 6 | 9 | 12 | 15 | 18 | 21 | 24 | 27 | 30 | 33 | 36 | 39 | 42 | 45 | 48 |
|---|---|---|---|---|---|---|---|---|---|---|---|---|---|---|---|---|---|
| LL (n=10) | 10 | 7 | 6 | 6 | 5 | 5 | 5 | 3 | 3 | 2 | 1 | 1 | 1 | 0 | 0 | 0 | 0 |
| LH (n=9) | 9 | 6 | 5 | 5 | 5 | 5 | 5 | 5 | 5 | 4 | 3 | 2 | 1 | 0 | 0 | 0 | 0 |
| HL (n=26) | 26 | 21 | 18 | 15 | 14 | 13 | 12 | 12 | 9 | 9 | 8 | 5 | 2 | 1 | 1 | 1 | 1 |
| HH (n=82) | 82 | 51 | 36 | 29 | 22 | 18 | 16 | 14 | 11 | 7 | 5 | 4 | 3 | 2 | 1 | 0 | 0 |

*Event-free survival, censoring new, non-protocol anti-cancer therapies, including stem cell transplant.
BM, bone marrow; CI, confidence interval; HH, high disease burden at screening and LD; HL, high disease burden at screening and low disease burden at LD; InO, inotuzumab ozogamicin; LD, lymphodepletion; LH, low disease burden at screening and high disease burden at LD; LL, low disease burden at screening and LD; NE, not estimable; TKI, tyrosine kinase inhibitor.

**Copyright Information:** (Online) ISSN: 2572-9241
©2026 The Author(s). HemaSphere is published by John Wiley & Sons Ltd on behalf of European Hematology Association. This is an open access Abstract Book distributed under the Attribution-NonCommercial-NoDerivs (CC BY-NC-ND), which allows third parties to download the articles and share them with others as long as they credit the author and the Abstract Book, but they cannot change the content in any way or use them commercially.
**Abstract Book Citations:** Authors, Title, HemaSphere, 2026;10:(S1):pages. The abstract book DOIs can be found on the "Author Information" page.
**Disclaimer:** Articles published in the journal HemaSphere exclusively reflect the opinions of the authors. The authors are responsible for all content in their abstracts including accuracy of the facts, statements, citing resources, etc.

### (S114) SAFETY AND EFFICACY OF UCART22 IN HEAVILY PRE-TREATED PATIENTS WITH RELAPSED OR REFRACTORY CD22+ B-CELL ACUTE LYMPHOBLASTIC LEUKEMIA (B-ALL): RESULTS OF THE PHASE 1 BALLI-01 TRIAL

**Topic:** 02. Acute lymphoblastic leukemia - Clinical

Nitin Jain*[1], Jose Carlos Cruz[2], Richard Larson[3], Gary Schiller[4], Aravind Ramakrishnan[5], Daniel J. Deangelo[6], Stephan Grupp[7], Mark Blaine Geyer[8], Patrice Chevallier[9], Marc Schwartz[10], Stephen Strickland[11], Jean-Baptiste Mear[12], Marie Balsat[13], Eunice Wang[14], Andre Baruchel[15], Luke Mountjoy[16], Narayan Lebaka[17], Carolyn Figliola[17], Subhendu Mukhopadhyay[17], Xenia Peluffo[18], Laurent Poirot[18], Robert Wasserman[17], Adrian Kilcoyne[17], Nicolas Boissel[19]

*[1]MD Anderson Cancer Center, Houston, United States of America;[2]Texas Transplant Institute, San Antonio, United States of America; [3]University of Chicago, Chicago, United States of America;[4]University of California Los Angeles, Los Angeles, United States of America; [5]South Austin Medical Center, Austin, United States of America;[6]Dana-Farber Cancer Institute, Boston, United States of America; [7]Children's Hospital of Philadelphia, Philadelphia, United States of America;[8]Memorial Sloan Kettering Cancer Center, Leukemia and Cell Therapy Services, Department of Medicine, New York, United States of America;[9]CHU de Nantes - Hotel-Dieu, Nantes, France; [10]University of Colorado - Aurora Cancer Center, Aurora, United States of America;[11]Sarah Cannon and HCA Research Institute, Nashville, United States of America; [12]CHU Rennes - Hopital Pontchaillou, Rennes, France; [13]Centre Hospitalier Lyon Sud, Département d'hématologie, Lyon, France; [14]Roswell Park Comprehensive Cancer Center, Buffalo, United States of America; [15]Hopital Robert Debre, Paris, France; [16]Sarah Cannon - Colorado Blood Cancer Institute, Denver, United States of America; [17]Cellectis Inc, New York, United States of America;[18]Cellectis, Paris, France; [19]Hopital Saint-Louis, Paris, France;*

### Background

UCART22 is an allogeneic donor derived CD22 directed CAR T cell product with TALEN® technology driven TRAC and CD52 knockout to minimize the risk of GvHD and allow for use of an anti-CD52 antibody for optimal lymphodepletion (LD). Prior data demonstrated high response rates with externally manufactured UCART22 (P1) (Boissel N EHA 2023) and Cellectis manufactured product (P2) (Jain N, ASH 2023). We now report final PhI results including all patients (pts) treated with UCART22 P2, establishing the recommended Ph2 dose (RP2D).

### Aims

Report results of a dose escalation study of UCART22 in CD22+R/R B-ALL

### Methods

The primary endpoints are safety and determination of the RP2D of UCART22. Additional endpoints are efficacy, UCART22 expansion and impact of alemtuzumab on LD. Eligibility criteria include ages 15−70y, CD22 expression ≥70%, and ≥2 prior treatments. After LD with fludarabine+cyclophosphamide (FC), +/- alemtuzumab, pts received UCART22 at 4 dose levels (1X105 to 5X106 cells/kg). A sub-study to investigate weight-based dosing of alemtuzumab was also performed.

**Copyright Information:** (Online) ISSN: 2572-9241
©2026 The Author(s). HemaSphere is published by John Wiley & Sons Ltd on behalf of European Hematology Association. This is an open access Abstract Book distributed under the Attribution-NonCommercial-NoDerivs (CC BY-NC-ND), which allows third parties to download the articles and share them with others as long as they credit the author and the Abstract Book, but they cannot change the content in any way or use them commercially.
**Abstract Book Citations:** Authors, Title, HemaSphere, 2026;10:(S1):pages. The abstract book DOIs can be found on the "Author Information" page.
**Disclaimer:** Articles published in the journal HemaSphere exclusively reflect the opinions of the authors. The authors are responsible for all content in their abstracts including accuracy of the facts, statements, citing resources, etc.

### Results

As of Feb 2026, 45 pts were enrolled (safety set), 43 received UCART22, P1=18, P2=25, (efficacy set). Median prior lines of tx (including Blina, Ino, HSCT and CAR-T) were 4 (Table 1). 53% of the pts received prior CD19 CAR T. CRS occurred in 32 pts (71%; 16 G1, 14 G2, 2 G3). ICANS occurred in 8 pts (18%; 2 G1, 4 G2, 2 G3). IEC-HS occurred in 2 pts (4%; 1 G2,1 G4). All CRS, ICANS, and IEC-HS resolved. AESIs include Infusion related reaction G4 in 1 pt, GvHD G2 in 1pt and DIC G3 in 1 pt.
Serious adverse events were reported in 31 pts (69%) including 6 (13%) bacterial infections, 6 (13%) fungal infections and 7 (16%) febrile neutropenia. Adverse event of CMV reactivation occurred in 6 pts.
In P1 cohorts, ORR (CR, CRi, CRh and MLFS) and CR/CRi were 33% and 28%, respectively. In P2 cohorts, ORR and CR/CRi were 64% and 36%, respectively.

Highest response rates were seen in the 15 pts treated with 5 x $10^6$ UCART22 cells/kg P2 (RP2D) with FC + alemtuzumab, with ORR and CR/CRi rates of 73% and 40%, respectively. Of the 6 pts who achieved a CR/CRi, 5 (83%) pts achieved MRD-ve status (<0.01%) by flow cytometry.

In the 7 pts age ≤50 y who received P2 at 5 x $10^6$ cells/kg (target Ph 2 population) with alemtuzumab (60 mg), ORR was 100% and 4/7 (57%) achieved CR/CRi (3/4 MRD-ve). All 7 pts went for a subsequent transplant.

| **UCART22 Dose** | **RP2D (P2)** | | | **All Doses** |
|---|---|---|---|---|
| | **Age 15 to 70 (n=15)** | **Age ≤ 50 (n=7) 60 mg Alemtuzumab** | **Age ≤ 50 (n=5) Lower dose Alemtuzumab** | **(n=45)** |
| **Age, median (range)** | 28(16-66) | 23(16-30) | 39(23-45) | 28(16-68) |
| **Prior tx, median (range)** | 5(2–11) | 5(4– 6) | 5 (3 –11) | 4(2 –11) |
| HSCT, n (%) | 6(40) | 3(43) | 3(60) | 22(47) |
| Inotuzumab, n (%) | 9(60) | 3 (43) | 4(80) | 25(56) |
| Blinatumomab, n (%) | 14(93) | 7(100) | 4(80) | 37(82) |
| CD19 CART, n (%) | 8(53) | 3 (42) | 4(80) | 24(53) |
| **Screening Blasts % (range)** | 67(14 –98) | 63(14-73) | 80(50-98) | 67(1-99) |
| **Efficacy** | | | | (n=43) |
| **ORR** | 11(73) | 7(100) | 3(60) | 22(51) |
| **CR/CRi** | 6(40) | 4(57) | 2(40) | 14(33) |
| **% CR/CRi MRD-ve** | 5(83) | 3(75) | 2(100) | 9(64) |

### Summary/Conclusion

Results from the BALL1-01 trial indicate that UCART22 delivers high response rates in heavily pre-treated patients enabling a majority of the targeted Ph2 patient population to proceed to transplant. UCART22 was generally well tolerated, with a favorable safety profile in comparison to approved CAR-T products. The Ph2 trial is currently recruiting (NCT#04150497).

**Copyright Information:** (Online) ISSN: 2572-9241
©2026 The Author(s). HemaSphere is published by John Wiley & Sons Ltd on behalf of European Hematology Association. This is an open access Abstract Book distributed under the Attribution-NonCommercial-NoDerivs (CC BY-NC-ND), which allows third parties to download the articles and share them with others as long as they credit the author and the Abstract Book, but they cannot change the content in any way or use them commercially.
**Abstract Book Citations:** Authors, Title, HemaSphere, 2026;10:(S1):pages. The abstract book DOIs can be found on the "Author Information" page.
**Disclaimer:** Articles published in the journal HemaSphere exclusively reflect the opinions of the authors. The authors are responsible for all content in their abstracts including accuracy of the facts, statements, citing resources, etc.

### (S115) EPIGENETIC-METABOLIC REPROGRAMMING OF MACROPHAGES BY DECITABINE REMODELS THE POST-TRANSPLANT IMMUNE MICROENVIRONMENT TO PREVENT AML RELAPSE VIA THE H3K4ME3-ACOD1-ITACONATE AXIS

**Topic:** 03. Acute myeloid leukemia - Biology & translational research

Yutong Xie*[1], Zhigang Chen[1], Chenmo Li[1], Junhong Wu[1], Chao Yang[1], Lei Gao[1, 2]

[1]*Xinqiao Hospital, Medical Center of Hematology, Chongqing, China;*[2]*Jinfeng laboratory, Chongqing, China;*

### Background

Decitabine (DAC) maintenance therapy markedly lowers relapse risk in acute myeloid leukemia (AML) post-hematopoietic stem cell transplantation (HSCT). However, post-HSCT relapse is fundamentally driven by an immunosuppressive bone marrow (BM) microenvironment, heavily orchestrated by alternatively activated macrophages. We hypothesize that DAC prevents relapse not merely through direct cytotoxicity, but by epigenetically rewiring macrophage metabolism to remodel this immunosuppressive niche and restore anti-leukemia immunity. This study investigates the H3K4me3–ACOD1–itaconate axis as the pivotal mediator of this microenvironmental reprogramming.

### Aims

To elucidate the dynamic biological alterations within the bone marrow microenvironment (BME) in patients receiving decitabine maintenance therapy, and to investigate how these microenvironmental changes correlate with clinical outcomes.

### Methods

- Clinical Cohorts: Prospective (n=30) and Retrospective (n=40) longitudinal profiling of BM macrophages pre- and post-HSCT (1, 3, 6, 12 months).
- Multi-Omics: RNA-seq, CUT&Tag, ChIP-qPCR, Untargeted Metabolomics, LC-MS/MS, 13C-Isotope Tracing.
- Microenvironment & Functional Assays: Seahorse XF analysis, Flow Cytometry. Immune crosstalk was evaluated using macrophage-T cell/AML co-culture systems.
- In Vivo: Humanized AML NSG mouse model with bioluminescence imaging and BM immune landscape profiling.

**Copyright Information:** (Online) ISSN: 2572-9241
©2026 The Author(s). HemaSphere is published by John Wiley & Sons Ltd on behalf of European Hematology Association. This is an open access Abstract Book distributed under the Attribution-NonCommercial-NoDerivs (CC BY-NC-ND), which allows third parties to download the articles and share them with others as long as they credit the author and the Abstract Book, but they cannot change the content in any way or use them commercially.
**Abstract Book Citations:** Authors, Title, HemaSphere, 2026;10:(S1):pages. The abstract book DOIs can be found on the "Author Information" page.
**Disclaimer:** Articles published in the journal HemaSphere exclusively reflect the opinions of the authors. The authors are responsible for all content in their abstracts including accuracy of the facts, statements, citing resources, etc.

### Results

Immune profiling of post-HSCT bone marrow across discovery (n=10) and validation cohorts (n=70) identified the monocyte/macrophage lineage as the primary driver of clinical outcomes. Compared to complete remission (CR), relapsed (RE) patients exhibited profound MDSC expansion (10.9-fold, P=0.0479) and progressive accumulation of immunosuppressive M2 macrophages (elevated CD163/CD206, P<0.05). Conversely, CR patients maintained robust M1 polarization (elevated CD80/iNOS, P<0.001), which independently predicted superior overall survival (495 vs 385 days; HR 0.42, 95% CI 0.21–0.84). Notably, ACOD1 (IRG1) expression showed dynamic, time-dependent changes strongly correlating with relapse risk (P=0.0001).

Mechanistically, integrated RNA-seq and CUT&Tag revealed that decitabine suppresses ACOD1 transcription by reducing H3K4me3 promoter enrichment (FC -2.8, q<0.001). Metabolomic and Seahorse profiling confirmed this epigenetic shift abolishes itaconate production, restores TCA flux, and enhances glycolytic capacity (ECAR/OCR +2.1-fold), thereby driving M2-to-M1 repolarization.

In humanized AML xenografts, decitabine suppressed bone marrow ACOD1 (-65%, P<0.01), increased the M1/M2 ratio 3.2-fold, and reduced tumor burden by 78% (P<0.001). This M1-driven anti-leukemic effect significantly prolonged median survival (58 vs 42 days, P=0.012), with pharmacological ACOD1 modulation confirming pathway dependency.

### Summary/Conclusion

We unveil a novel epigenetic-metabolic-immune crosstalk wherein Decitabine modulates the H3K4me3–ACOD1–itaconate axis to reverse M2-macrophage mediated immune tolerance. By metabolically reprogramming the post-HSCT marrow niche, Decitabine restores robust anti-leukemic immunity, establishing the ACOD1 pathway as a potent therapeutic target for relapse prophylaxis in AML.

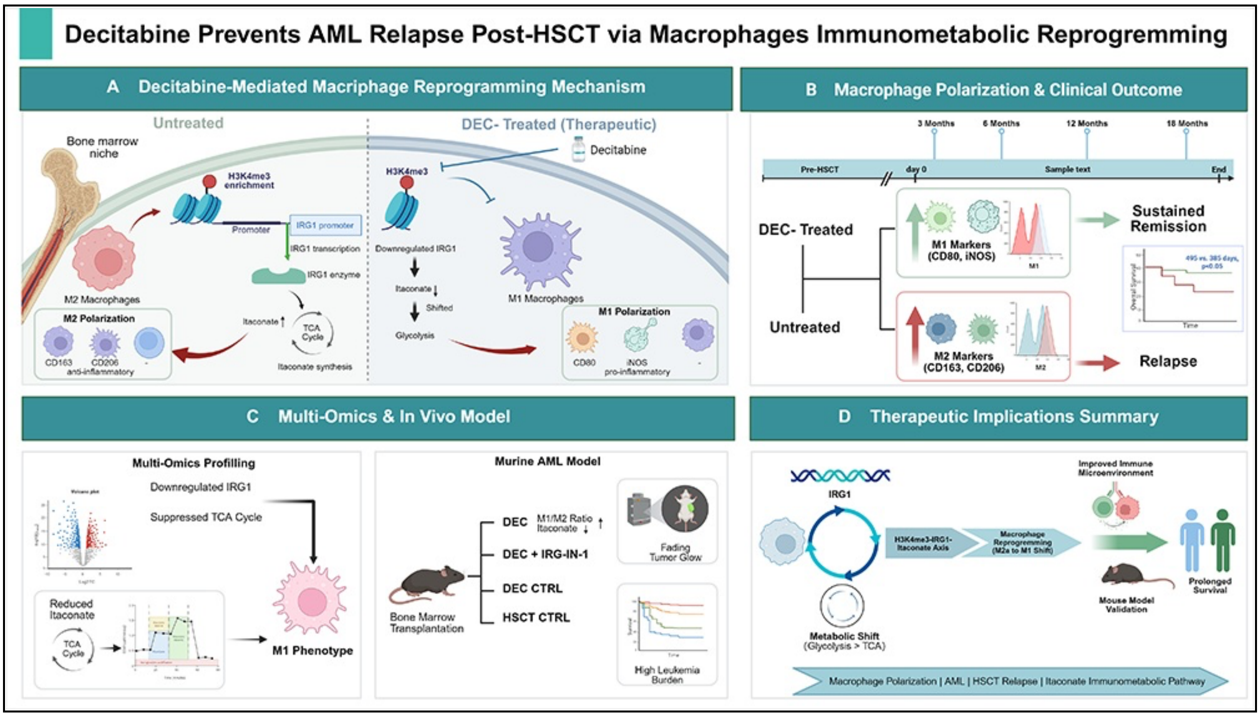

Figure1. Decitabine prevents AML relapse post-HSCT via macrophage immunometabolic reprogramming.

**Copyright Information:** (Online) ISSN: 2572-9241
©2026 The Author(s). HemaSphere is published by John Wiley & Sons Ltd on behalf of European Hematology Association. This is an open access Abstract Book distributed under the Attribution-NonCommercial-NoDerivs (CC BY-NC-ND), which allows third parties to download the articles and share them with others as long as they credit the author and the Abstract Book, but they cannot change the content in any way or use them commercially.
**Abstract Book Citations:** Authors, Title, HemaSphere, 2026;10:(S1):pages. The abstract book DOIs can be found on the "Author Information" page.
**Disclaimer:** Articles published in the journal HemaSphere exclusively reflect the opinions of the authors. The authors are responsible for all content in their abstracts including accuracy of the facts, statements, citing resources, etc.

### (S116) NPM1C REQUIRES SNORNA DEPENDENT CHROMATIN BINDING FOR LEUKEMOGENESIS

**Topic:** 03. Acute myeloid leukemia - Biology & translational research

Fengbiao Zhou*[1, 2], Christian Rohde[1, 2], Enise Ceran[1], Viral Shah[1], Haiyang Yun[3], Xiaobing Yu[1], Nesrine Aroua[4, 5], Aline Sähr[1], Jia Yan[1, 2], Maximilian Blank[1], Yi Liu[1, 2], Hannah Uckelmann[6], Margaret Goodell[7], Andreas Trumpp[4, 5], Carsten Müller-Tidow[1, 2]

*[1]Heidelberg University Hospital, Department of Medicine V, Hematology, Oncology and Rheumatology, Heidelberg, Germany; [2]European Molecule Biology Laboratory (EMBL), Molecular Medicine Partnership Unit, Heidelberg, Germany; [3]Robert Bosch Center for Tumor Diseases, Stuttgart, Germany; [4]German Cancer Research Center (DKFZ), Division of Stem Cells and Cancer, Heidelberg, Germany; [5]Heidelberg Institute of Stem Cell Technology and Experimental Medicine (HI-STEM gGmbH), Heidelberg, Germany; [6]Goethe University Frankfurt, Department of Pediatrics, Frankfurt, Germany; [7]Baylor College of Medicine, Department of Molecular and Cellular Biology, Houston, United States of America;*

### Background

Acute myeloid leukemia (AML) is a genetically heterogeneous malignancy in which recurrent somatic mutations define biologically distinct entities. NPM1 mutations represent one of the most frequent lesions in adult AML and are established diagnostic and prognostic markers. NPM1 functions as a nucleolar protein in ribosome biogenesis and rRNA processing which closely depend on NPM1 interactions with multiple RNAs. Currently, the RNA binding functions of NPM1c in leukemogenesis remain unknown.

### Aims

To determine the contribution of RNA binding to NPM1 wild-type and NPM1c-driven leukemogenesis.

### Methods

The RNA interactomes of NPM1c and wild-type NPM1 were profiled by enhanced crosslinking and immunoprecipitation (eCLIP) in OCI-AML3 (NPM1-mutant) and OCI-AML2 (wild-type) cells. Subcellular RNA sequencing defined the localization of NPM1c-bound transcripts. Putative RNA-binding residues were predicted using the machine learning–based tool RNABindRPlus followed by structural docking analysis. RNA-binding–defective NPM1c variants were generated and re-expressed in NPM1c-degron OCI-AML3 cells to assess RNA-dependent functions. Chromatin occupancy was assessed by ChIP-Seq, and transcriptional consequences were evaluated by RNA-Seq and nascent RNA-Seq. CRISPR-Cas9–mediated depletion of NPM1c-interacting RNAs was performed to determine functional dependency in vitro and in vivo.

### Results

Both NPM1 wild-type and NPM1c exhibited extensive RNA interactions. NPM1c showed enhanced binding to small nucleolar RNAs (snoRNAs) in NPM1-mutant cells. Notably, the majority of NPM1c-bound snoRNAs were enriched in the chromatin fraction. Deletion of the predicted RNA-interacting amino acid residues in NPM1c selectively impaired snoRNA association with NPM1c. In NPM1c-degron OCI-AML3 cells, re-expression of full-length NPM1c restored proliferation and blocked differentiation after endogenous NPM1c degradation. In contrast, snoRNA-binding–deficient NPM1c variants failed to rescue growth or prevent differentiation.
Mechanistically, snoRNAs facilitated chromatin recruitment of NPM1c. ChIP-seq analysis revealed that snoRNA binding was required for approximately two-thirds of NPM1c-occupied genomic regions.
Among NPM1c-interacting RNAs, SCARNA7 specifically bound NPM1c but not wild-type NPM1. SCARNA7 depletion impaired clonogenic growth and in vivo engraftment of NPM1-mutant cells. In contrast, NPM1 wild-type leukemias were not dependent on SCARNA7, indicating a selective NPM1c-associated vulnerability.

**Copyright Information:** (Online) ISSN: 2572-9241
©2026 The Author(s). HemaSphere is published by John Wiley & Sons Ltd on behalf of European Hematology Association. This is an open access Abstract Book distributed under the Attribution-NonCommercial-NoDerivs (CC BY-NC-ND), which allows third parties to download the articles and share them with others as long as they credit the author and the Abstract Book, but they cannot change the content in any way or use them commercially.
**Abstract Book Citations:** Authors, Title, HemaSphere, 2026;10:(S1):pages. The abstract book DOIs can be found on the "Author Information" page.
**Disclaimer:** Articles published in the journal HemaSphere exclusively reflect the opinions of the authors. The authors are responsible for all content in their abstracts including accuracy of the facts, statements, citing resources, etc.

### Summary/Conclusion

We uncover a mutation-driven RNA–chromatin regulatory axis in which snoRNAs function as molecular adaptors that enable oncogenic NPM1c to engage selective genomic loci and sustain leukemic transcriptional programs. RNA-dependent chromatin targeting represents a key oncogenic mechanism and defines a specific molecular vulnerability in NPM1-mutant AML.

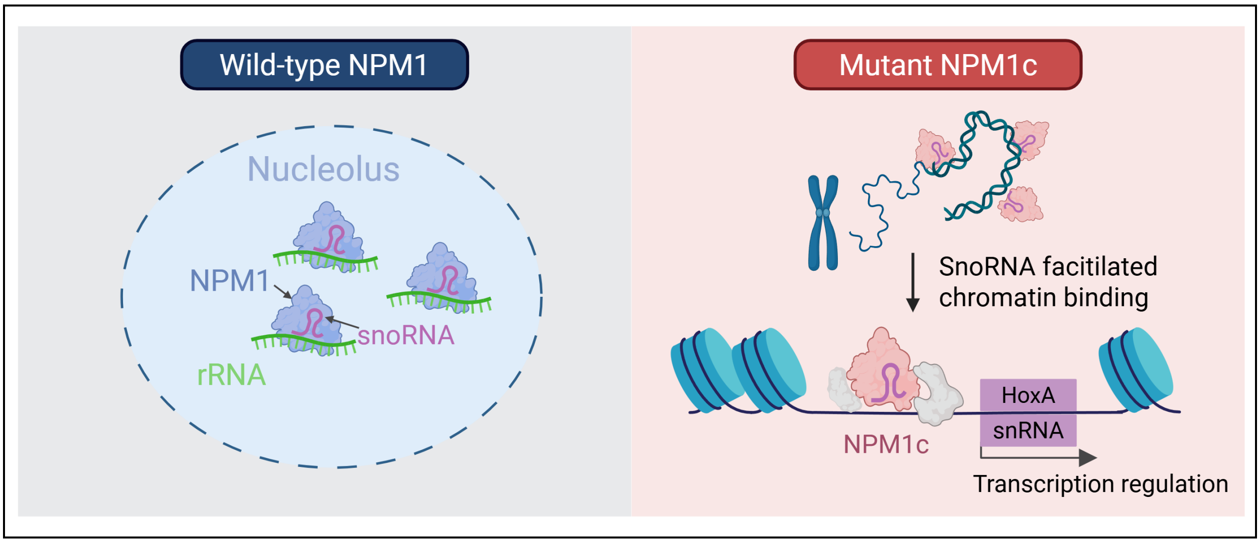

Schematic model of mutation-driven RNA-dependent chromatin targeting by NPM1c in AML.

**Copyright Information:** (Online) ISSN: 2572-9241
©2026 The Author(s). HemaSphere is published by John Wiley & Sons Ltd on behalf of European Hematology Association. This is an open access Abstract Book distributed under the Attribution-NonCommercial-NoDerivs (CC BY-NC-ND), which allows third parties to download the articles and share them with others as long as they credit the author and the Abstract Book, but they cannot change the content in any way or use them commercially.
**Abstract Book Citations:** Authors, Title, HemaSphere, 2026;10:(S1):pages. The abstract book DOIs can be found on the "Author Information" page.
**Disclaimer:** Articles published in the journal HemaSphere exclusively reflect the opinions of the authors. The authors are responsible for all content in their abstracts including accuracy of the facts, statements, citing resources, etc.

### (S117) TARGETING PCYT1A INDUCES SYNTHETIC LETHALITY IN MONOCYTIC AML BY TRIGGERING PEMT-DEPENDENT METABOLIC CATASTROPHE AND ONE-CARBON IMBALANCE

**Topic:** 03. Acute myeloid leukemia - Biology & translational research

Jakushin Nakahara*[1], Keita Yamamoto[1], Yu-Hsuan Chang[1], Tomohiro Yabushita[1], Takumi Chinen[2], Kei Ito[2], Yutaka Takeda[2], Jumpei Omi[3], Yuki Sugiura[4], Wakana Sekiguchi[1], Junken Aoki[3], Daiju Kitagawa[2], Susumu Goyama[1]

*[1]Division of Molecular Oncology, Graduate School of Frontier Sciences, The University of Tokyo, Tokyo, Japan;[2]Department of Physiological Chemistry, Graduate School of Pharmaceutical Sciences, The University of Tokyo, Tokyo, Japan; [3]Department of Health Chemistry, Graduate School of Pharmaceutical Sciences, The University of Tokyo, Tokyo, Japan; [4]Multi-Omics Platform, Center for Cancer Immunotherapy and Immunobiology, Graduate School of Medicine, Kyoto University, Kyoto, Japan;*

### Background

In recent years, the emergence of molecularly targeted agents has enhanced the treatment landscape of AML.However, some genetic aberrations such as *MLL* (*KMT2A*) rearrangements (MLL-r) are still associated with inferior outcomes. Notably, MLL-r AML frequently exhibits monocytic differentiation, a lineage feature recently linked to resistance against venetoclax-based therapies. While the recent expansion of functional genomic databases such as the DepMap has facilitated therapeutic target discovery, therapeutic vulnerabilities specific to such mutation-driven, lineage-defined subtypes remain largely unexplored.

### Aims

We aimed to identify therapeutic vulnerabilities specific to MLL-r AML using the DepMap database and to elucidate their biological efficacy and underlying mechanisms.

### Methods

To identify therapeutic targets specific to MLL-r AML, we developed a custom Python-based pipeline integrating DepMap datasets. Following *in silico* screening, we validated candidate essentiality via CRISPR/Cas9-mediated knockout. Fluorescence-based competition assays were performed both *in vitro* and *in vivo* using human AML cell lines, murine AML cells, and PDX models. Tumor burden was monitored using bioluminescence imaging. We further evaluated the impact on differentiation through morphological analysis and flow cytometry. To elucidate the underlying mechanisms, we performed integrative multi-omics analyses —including RNA-seq, lipidomics, and metabolomics—followed by Western blotting and ELISA.

**Copyright Information:** (Online) ISSN: 2572-9241
©2026 The Author(s). HemaSphere is published by John Wiley & Sons Ltd on behalf of European Hematology Association. This is an open access Abstract Book distributed under the Attribution-NonCommercial-NoDerivs (CC BY-NC-ND), which allows third parties to download the articles and share them with others as long as they credit the author and the Abstract Book, but they cannot change the content in any way or use them commercially.
**Abstract Book Citations:** Authors, Title, HemaSphere, 2026;10:(S1):pages. The abstract book DOIs can be found on the “Author Information” page.
**Disclaimer:** Articles published in the journal HemaSphere exclusively reflect the opinions of the authors. The authors are responsible for all content in their abstracts including accuracy of the facts, statements, citing resources, etc.

**Results**

Our DepMap-based screening initially identified PCYT1A, a rate-limiting enzyme of the phosphatidylcholine biosynthetic pathway, as the top-ranked specific dependency in MLL-r AML, outperforming established targets such as *MEN1*and *KMT2A* itself. We confirmed that *PCYT1A* knockout significantly suppressed growth *in vitro* and *in vivo*, and prolonged survival in xenograft models.

Notably, while characterizing this dependency, we discovered that *PCYT1A* essentiality extends beyond MLL-r AML to the broader monocytic lineage. This lineage-specific vulnerability arises from the intrinsic lack of the paralog PCYT1B in monocytic AML, creating a synthetic lethal dependency on PCYT1A. Indeed, dual depletion of both paralogs was required to suppress non-monocytic AML cell proliferation, whereas ectopic PCYT1B expression rescued *PCYT1A* knockout lethality in monocytic cells.

Mechanistically, *PCYT1A* loss induced granulo-monocytic differentiation but, surprisingly, did not alter total phosphatidylcholine levels. We attributed this to a compensatory upregulation of PEMT, confirmed by RNA-seq and Western blotting. Metabolomic profiling revealed that this rewiring triggered a hyperactivation of the methionine cycle and one-carbon metabolism, likely driven by the increased demand for methyl groups. This aberrant metabolic flux resulted in a significant and toxic accumulation of SAM. Exogenous SAM supplementation recapitulated the growth suppression and differentiation phenotypes, confirming that SAM accumulation is a primary mechanism of the anti-leukemic effect.

**Summary/Conclusion**

We identified PCYT1A as a robust synthetic lethal target, extending its relevance from MLL-r to the broader monocytic AML lineage. We elucidate a novel metabolic crosstalk where PCYT1A suppression forces PEMT-dependent rewiring, driving an excessive activation of one-carbon metabolism and leading to toxic SAM accumulation. These findings establish the PCYT1A-PEMT axis as a promising therapeutic strategy for lineage-defined, high-risk AML.

**Copyright Information:** (Online) ISSN: 2572-9241
©2026 The Author(s). HemaSphere is published by John Wiley & Sons Ltd on behalf of European Hematology Association. This is an open access Abstract Book distributed under the Attribution-NonCommercial-NoDerivs (CC BY-NC-ND), which allows third parties to download the articles and share them with others as long as they credit the author and the Abstract Book, but they cannot change the content in any way or use them commercially.
**Abstract Book Citations:** Authors, Title, HemaSphere, 2026;10:(S1):pages. The abstract book DOIs can be found on the "Author Information" page.
**Disclaimer:** Articles published in the journal HemaSphere exclusively reflect the opinions of the authors. The authors are responsible for all content in their abstracts including accuracy of the facts, statements, citing resources, etc.

### (S118) ZEB1 DEFINES A TARGETABLE MENIN-INHIBITOR ESCAPE PROGRAM IN KMT2A::MLLT3 AML

**Topic:** 03. Acute myeloid leukemia - Biology & translational research

Liam MacPhee*[1], Kathrin Krowiorz[2], Leo Escano[1], Xining Yang[1], Sarah Grasedieck[3], Diana Djaksigulova[1], Caroline Pabst[4], Linda Fogelstrand[5], Nayeli Esparza[6], Frédéric Barabé[6], Stephane Flibotte[7], Jian Huang[8], David Sanford[9], Arefeh Rouhi[1], Florian Kuchenbauer[1]

*[1]British Columbia Cancer Research Institute, Terry Fox Laboratory, Vancouver, Canada; [2]University Hospital Ulm, Internal Medicine III, Ulm, Germany; [3]University of British Columbia, Canada's Michael Smith Genome Sciences Centre, Vancouver, Canada; [4]University Hospital Heidelberg, Division of Hematology and Oncology, Heidelberg, Germany; [5]Sahlgrenska University Hospital, Department of Clinical Chemistry, Gothenburg, Sweden; [6]Laval University, Centre de Recherche du CHU de Québec, Quebec City, Canada; [7]University of British Columbia, Life Sciences Institute, Vancouver, Canada; [8]Rush University Medical Center, Department of Orthopedic Surgery, Chicago, United States of America; [9]University of British Columbia, Medicine, Vancouver, Canada;*

### Background

MENIN inhibitors have demonstrated unprecedented activity in KMT2A-rearranged acute myeloid leukemia (AML). KMT2A::MLLT3 leukemia is critically dependent on the HOXA9/MEIS1 (H9M) transcriptional axis, a central oncogenic vulnerability targeted by MENIN inhibition. However, with the emergence of clinical resistance, the transcriptional programs that support survival following H9M suppression and limit the durability of response remain poorly defined. ZEB1, a regulator of cellular plasticity and stemness that promotes aggressive, treatment-resistant phenotypes in KMT2A::MLLT3 AML, is negatively regulated by microRNA-204 (miR-204), and represents a potential mediator of resistance. However, its role in MENIN inhibitor response has not been mechanistically established.

### Aims

To determine whether ZEB1 supports transcriptional adaptation in KMT2A::MLLT3 AML under MENIN inhibition and to assess whether modulation of the miR-204/ZEB1 axis restores therapeutic vulnerability.

### Methods

We integrated bulk RNA-seq AML patient datasets with clonal murine KMT2A::MLLT3 models, human $CD34^+$ cord blood derived AML models, and genetic perturbation systems. ZEB1 was depleted by shRNA or was suppressed via miR-204 overexpression. To model resistance associated with the loss of miR-204, we generated a CRISPR/Cas9 miR-204 knockout (204-KO) model of KMT2A::MLLT3 and assessed response to MENIN inhibition. Functional effects on leukemic stemness, differentiation, and MENIN inhibitor response (revumenib/SNDX-5613) were assessed in vitro and in vivo. 5-azacytidine (5-Aza) was evaluated as a strategy for epigenetic reactivation of miR-204.

**Copyright Information:** (Online) ISSN: 2572-9241
©2026 The Author(s). HemaSphere is published by John Wiley & Sons Ltd on behalf of European Hematology Association. This is an open access Abstract Book distributed under the Attribution-NonCommercial-NoDerivs (CC BY-NC-ND), which allows third parties to download the articles and share them with others as long as they credit the author and the Abstract Book, but they cannot change the content in any way or use them commercially.
**Abstract Book Citations:** Authors, Title, HemaSphere, 2026;10:(S1):pages. The abstract book DOIs can be found on the "Author Information" page.
**Disclaimer:** Articles published in the journal HemaSphere exclusively reflect the opinions of the authors. The authors are responsible for all content in their abstracts including accuracy of the facts, statements, citing resources, etc.

**Results**

We identified a primitive leukemic subpopulation in murine KMT2A::MLLT3 AML characterized by high ZEB1 expression and suppressed H9M, consistent with an alternative survival state distinct from canonical H9M dependency. Bulk AML patient RNA-seq data confirmed an anti-correlation between ZEB1 and H9M specifically in KMT2A-rearranged AML. Genetic depletion of ZEB1 led to increased H9M expression, supporting an inverse relationship between these programs. Analysis of primary human AML samples then demonstrated suppression of miR-204, a known regulator of ZEB1. Enforced miR-204 expression reduced colony-forming capacity in both murine and human $CD34^+$ cord blood–derived KMT2A::MLLT3 AML models while sparing normal hematopoiesis. In vivo, miR-204 overexpression significantly prolonged survival in KMT2A::MLLT3 and H9M-driven AML, while promoting myeloid differentiation. RNA-seq of leukemic bone marrow confirmed repression of ZEB1 and associated EMT and chemotaxis transcriptional programs. Consistent with these findings, CRISPR-mediated 204-KO KMT2A::MLLT3 enabled ZEB1 expression and reinforced the H9M-low state. Functionally, miR-204 overexpression or ZEB1 knockdown significantly enhanced sensitivity to MENIN inhibition, whereas 204-KO cells upregulated ZEB1 under MENIN inhibitor exposure, consistent with adaptive resistance. Finally, low-dose 5-azacytidine restored miR-204 expression in human and murine KMT2A::MLLT3 models, supporting a rational combination strategy with MENIN inhibition.

**Summary/Conclusion**

We identify a previously unrecognized mechanism of MENIN inhibitor resistance in KMT2A::MLLT3 AML mediated by ZEB1, which supports H9M-independent survival. MiR-204 negatively regulates ZEB1 and can be pharmacologically reactivated with hypomethylating agents, thereby restoring sensitivity to MENIN inhibition and providing a mechanistic rationale for combination therapy.

**Copyright Information:** (Online) ISSN: 2572-9241
©2026 The Author(s). HemaSphere is published by John Wiley & Sons Ltd on behalf of European Hematology Association. This is an open access Abstract Book distributed under the Attribution-NonCommercial-NoDerivs (CC BY-NC-ND), which allows third parties to download the articles and share them with others as long as they credit the author and the Abstract Book, but they cannot change the content in any way or use them commercially.
**Abstract Book Citations:** Authors, Title, HemaSphere, 2026;10:(S1):pages. The abstract book DOIs can be found on the “Author Information” page.
**Disclaimer:** Articles published in the journal HemaSphere exclusively reflect the opinions of the authors. The authors are responsible for all content in their abstracts including accuracy of the facts, statements, citing resources, etc.

### (S119) ERG RECONFIGURES A CONTEXT-SPECIFIC LEUKAEMIC 3D ENHANCER LANDSCAPE TO DRIVE AML VIA DIRECT ENHANCER-MACHINERY REPURPOSING AND INDIRECT TRANSCRIPTION FACTOR BLOCKADE

**Topic:** 03. Acute myeloid leukemia - Biology & translational research

Cécile Lopez*[1], Eshwar Meduri[1], Kim Sharp[2], James Williamson[3], Jaana Bagri[1], George Giotopoulos[1], Gabriela Grondys-Kotarba[4], Suraj Bansal[5], Andy Zheng[5], Julie Thoms[6], John Primanda[6], John Dick[5], Paul Lehner[3], Thomas Mercher[7], James Davies[2], Brian Huntly[1]

*[1]Cambridge Stem Cell Institute, Cambridge, United Kingdom; [2]MRC Molecular Haematology Unit, Oxford, United Kingdom; [3]Cambridge Institute of Therapeutic Immunology and Infectious Disease, Cambridge, United Kingdom;[4]Cambridge Institute for Medical Research, Cambridge, United Kingdom; [5]Princess Margaret Cancer Center, Toronto, Canada;[6]University-Lowy Cancer Research Centre, Sydney, Australia; [7]Gustave Roussy Cancer Campus, Villejuif, France;*

### Background

ERG is a transcription factor (TF) with both activating and repressive functions and plays a key role in normal haematopoietic stem and progenitor cell maintenance. Aberrant *ERG* overexpression is associated with several malignancies, particularly acute myeloid leukaemia (AML), where it correlates with leukaemic stem cell (LSC) characteristics, therapy resistance and relapse. However, the molecular mechanisms through which ERG drives leukaemic initiation and maintenance remain poorly understood.

### Aims

To determine how ERG alters the regulatory landscape to control leukaemic gene expression programmes.

### Methods

We analysed 18 primary AML patient samples and six AML cell lines under native and perturbed ERG conditions (shRNA-knockdown and ERG-overexpression). Integrated transcriptomic, epigenomic (ChIP/ATAC-seq), chromatin 3D-conformation (Hi-ChIP), and protein interactome analyses (IP-MS) were performed, including integrated enhancer profiling and 3D interaction mapping (Microcapture-C).

**Copyright Information:** (Online) ISSN: 2572-9241
©2026 The Author(s). HemaSphere is published by John Wiley & Sons Ltd on behalf of European Hematology Association. This is an open access Abstract Book distributed under the Attribution-NonCommercial-NoDerivs (CC BY-NC-ND), which allows third parties to download the articles and share them with others as long as they credit the author and the Abstract Book, but they cannot change the content in any way or use them commercially.
**Abstract Book Citations:** Authors, Title, HemaSphere, 2026;10:(S1):pages. The abstract book DOIs can be found on the "Author Information" page.
**Disclaimer:** Articles published in the journal HemaSphere exclusively reflect the opinions of the authors. The authors are responsible for all content in their abstracts including accuracy of the facts, statements, citing resources, etc.

### Results

We demonstrate that ERG is essential for leukaemic cell survival and maintenance, by preventing apoptosis and differentiation, across multiple AML genotypes and baseline *ERG* expression levels. *ERG* overexpression conferred LSC-associated properties, including quiescence and therapeutic resistance, highlighting its role in leukaemia persistence and relapse.
Despite these shared cellular phenotypes, we could demonstrate that ERG regulates distinct, context-specific transcriptional programmes and different binding-cistromes across multiple AML samples. While ERG promoter occupancy established during normal haematopoiesis is preserved, it is extensively distally reprogrammed in AML to occupy leukaemia-reconfigured enhancers, thereby rewiring the enhancer landscape in a highly context-dependent manner. These ERG-bound leukaemia-reconfigured enhancers are significantly enriched for long-range chromatin interactions that activate critical genes, as demonstrated by CRISPRd. Mechanistically, ERG interacts with key enhancer-associated complexes, including KMT2D (H3K4me1-modifying), the BAF chromatin-remodelling complex, and EP300/CREBBP (H3K27ac-modifying), corroborating its role in enhancer establishment and maintenance. Functional perturbation experiments demonstrate that ERG acts as a pioneer factor, binding at previously closed chromatin, initiating enhancer formation, and promoting enhancer-promoter looping to activate leukaemia-specific transcriptional programmes.
In addition, ERG indirectly enforces differentiation blockade by functionally sequestering enhancer-associated complexes away from lineage-determining TFs (e.g. GATA1, CEBPA), thereby limiting activation of lineage-specific genes and the induction of differentiation-associated transcriptional programmes.

### Summary/Conclusion

ERG sustains leukaemia maintenance by enforcing LSC programmes and blocking differentiation. This occurs through ERG-reprogramming of the enhancer landscape and 3D genome architecture via repurposing the core enhancer machinery to establish a novel leukaemia-associated enhancer landscape, whilst decommissioning differentiation-specific enhancers (Figure). These findings identify ERG-associated enhancer complexes as a potential Achilles' heel in *ERG*-high, poor prognosis AML. In ongoing work, pharmacological targeting of ERG and/or the enhancer components shows promising *in vitro* preclinical activity, with *in vivo* studies using patient-derived xenograft models currently underway.

**Copyright Information:** (Online) ISSN: 2572-9241
©2026 The Author(s). HemaSphere is published by John Wiley & Sons Ltd on behalf of European Hematology Association. This is an open access Abstract Book distributed under the Attribution-NonCommercial-NoDerivs (CC BY-NC-ND), which allows third parties to download the articles and share them with others as long as they credit the author and the Abstract Book, but they cannot change the content in any way or use them commercially.
**Abstract Book Citations:** Authors, Title, HemaSphere, 2026;10:(S1):pages. The abstract book DOIs can be found on the "Author Information" page.
**Disclaimer:** Articles published in the journal HemaSphere exclusively reflect the opinions of the authors. The authors are responsible for all content in their abstracts including accuracy of the facts, statements, citing resources, etc.

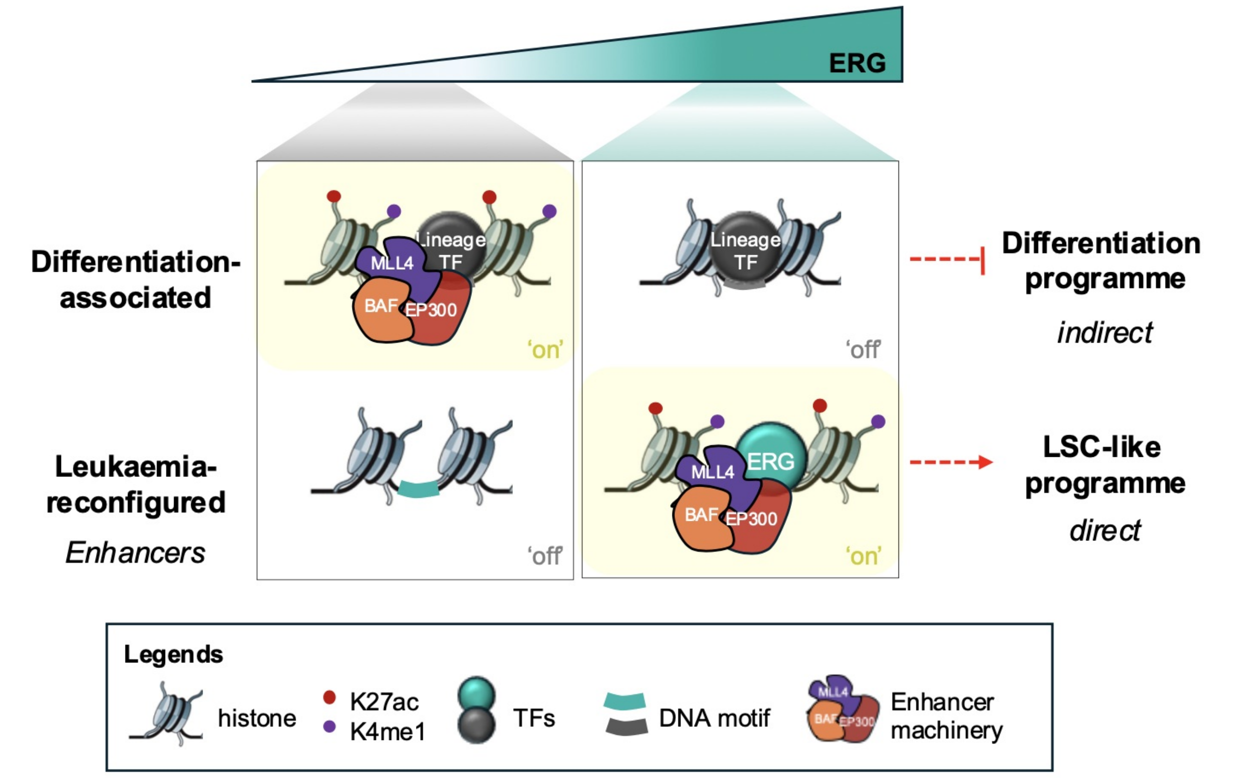

***Figure :***
ERG interacts with the enhancer-associated complexes leading to : (1) the activation of *leukaemia-reconfigured enhancers* that directly drive the LSC-like programme and (2) indirectly blocks the differentiation programmes by sequestering away the enhancer machinery from the lineage-determining TFs, resulting in the collapse of their differentiation-associated enhancers.

**Copyright Information:** (Online) ISSN: 2572-9241
©2026 The Author(s). HemaSphere is published by John Wiley & Sons Ltd on behalf of European Hematology Association. This is an open access Abstract Book distributed under the Attribution-NonCommercial-NoDerivs (CC BY-NC-ND), which allows third parties to download the articles and share them with others as long as they credit the author and the Abstract Book, but they cannot change the content in any way or use them commercially.
**Abstract Book Citations:** Authors, Title, HemaSphere, 2026;10:(S1):pages. The abstract book DOIs can be found on the "Author Information" page.
**Disclaimer:** Articles published in the journal HemaSphere exclusively reflect the opinions of the authors. The authors are responsible for all content in their abstracts including accuracy of the facts, statements, citing resources, etc.

### (S120) A CD22 ANTIGEN SHIFT OCCURS IN VENETOCLAX/AZACITIDINE RESISTANT AML AND FACILITATES TARGETED THERAPY

**Topic:** 03. Acute myeloid leukemia - Biology & translational research

Josephine Brysting*[1, 2], Julia Unglaub[2], Anne Merbach[1, 2], Binje Vick[3, 4], Zeynep Elli[2], Haizhang Chen[1, 2], Christian Rohde[1, 2], Stefanie Gryzik[2, 5], Michael Hundemer[2, 5], Sarah Richter[2], Maximilian Mönnig[2], Aino-Maija Leppä[6, 7], Alexander Waclawiczek[6, 7], Simon Renders[6, 7], Andreas Trumpp[6, 7], Rabia Shahswar[8], Michael Heuser[8, 9], Tilman Schöning[10], Caroline Pabst[1, 2], Simon Raffel[2], Tim Sauer[2], Michael Scherer[11], Judith Zaugg[1, 12, 13], Irmela Jeremias[3, 4, 14], Carsten Müller-Tidow[1, 2], Maike Janssen[1, 2]

*[1]European Molecular Biology Laboratory, Molecular Medicine Partnership Unit, Heidelberg, Germany; [2]Universitätsklinikum Heidelberg, Department of Internal Medicine V, Hematology, Oncology and Rheumatology, Heidelberg, Germany; [3]Helmholtz Zentrum München, German Research Center for Environmental Health (HMGU), Research Unit Apoptosis in Hematopoietic Stem Cells, München, Germany; [4]Partner site Munich, a partnership between DKFZ and University Hospital LMU Munich, German Cancer Consortium (DKTK), Munich, Germany; [5]MVZ Hämatologische Diagnostik, Heppenheim, Germany; [6]Deutsches Krebsforschungszentrum (DKFZ) and DKFZ-ZMBH Alliance, Heidelberg, Germany; [7]Heidelberg Institute for Stem Cell Technology and Experimental Medicine, Heidelberg, Germany; [8]Hannover Medical School, Department of Hematology, Hemostasis, Oncology, and Stem Cell Transplantation, Hannover, Germany; [9]University Hospital Halle (Saale), Department of Internal Medicine IV, Hannover, Germany; [10]Heidelberg University Hospital, Pharmacy of Heidelberg University Hospital, Heidelberg, Germany; [11]Deutsches Krebsforschungszentrum (DKFZ), Division of Cancer Epigenomics, Heidelberg, Germany; [12]University Hospital Basel, Department of Biomedicine, Basel, Switzerland; [13]European Molecular Biology Laboratory, Molecular Systems Biology Unit, Heidelberg, Germany; [14]Dr. von Hauner Children's Hospital, LMU University Hospital, Department of Pediatrics, Munich, Germany;*

### Background

Acute myeloid leukemia (AML) is a highly aggressive hematologic malignancy and treatment options remain limited, particularly in older or unfit patients. Although treatment with venetoclax (VEN) and azacitidine (AZA) has improved response rates, almost all patients relapse. Identification of resistance-associated biomarkers and targetable vulnerabilities are therefore of high clinical relevance.

### Aims

We aimed to identify novel therapeutic targets in AZA and/or VEN/AZA exposed AML by surface proteomics and by enhanced CITE-Seq of paired diagnosis-resistant specimens of VEN/AZA treated AML patients.

### Methods

We performed surface glycoproteomics for primary AML samples upon AZA exposure *in vitro* and validated candidate targets by flow cytometry. CloneTracer based single-cell RNA sequencing with extensive CITE-Seq panels was conducted on paired diagnostic and relapse samples of VEN/AZA-treated patients. CD22 expression was analyzed in a clinical cohort of 152 AML patients and correlated with risk classification and survival. Therapeutic targeting was evaluated using inotuzumab ozogamicin (InO) in CD22-positive primary samples and patient-derived xenograft (PDX) models *in vitro* and *in vivo*. Clinical activity was assessed in a patient with refractory CD22-positive AML. We treated a patient with *TP53* mutant AML relapse and prior exposure to VEN/AZA at 120 days post allogeneic stem cell transplantation. The patient was treated with two cycles of InO and three cycles of VEN/AZA. All patients provided written informed consent, and InO was administered off-label in accordance with institutional practice.

**Copyright Information:** (Online) ISSN: 2572-9241
©2026 The Author(s). HemaSphere is published by John Wiley & Sons Ltd on behalf of European Hematology Association. This is an open access Abstract Book distributed under the Attribution-NonCommercial-NoDerivs (CC BY-NC-ND), which allows third parties to download the articles and share them with others as long as they credit the author and the Abstract Book, but they cannot change the content in any way or use them commercially.
**Abstract Book Citations:** Authors, Title, HemaSphere, 2026;10:(S1):pages. The abstract book DOIs can be found on the "Author Information" page.
**Disclaimer:** Articles published in the journal HemaSphere exclusively reflect the opinions of the authors. The authors are responsible for all content in their abstracts including accuracy of the facts, statements, citing resources, etc.

### Results

Surface proteomic profiling identified CD22 as a consistently induced protein following AZA exposure. Flow cytometry confirmed aberrant CD22 expression on AML blasts, including $CD34^{+}$ progenitor populations. CloneTracer based single-cell RNA-Seq and CITE-Seq analyses revealed specific induction of CD22 surface protein expression in patients relapsed after VEN/AZA therapy. Of note, CD22 expression was observed in leukemia cell compartments including hematopoietic stem cell (HSC)/multipotent progenitors (MPP) as well as monocytes. No aberrant CD22 expression was observed in healthy progenitors of VEN/AZA relapsed AML patients. Leukemic stem cell (LSC) stemness scores were as high or even higher in CD22 expressing blast compartments as in CD22 negative counterparts.
In the clinical cohort, CD22-positive blasts were detected in a substantial proportion of patients, with increased prevalence after therapy and especially in VEN/AZA-refractory cases. CD22 positivity was enriched in European Leukemia Network (ELN) 2022 adverse-risk AML.
In primary AML specimens and PDX models, CD22 expression correlated with increased sensitivity to InO *in vitro*. *In vivo*, InO treatment of a CD22-positive PDX model resulted in reduced leukemic burden and prolonged survival compared to control mice. A patient with *TP53* mutant AML relapse and prior exposure to VEN/AZA achieved a leukemia free bone marrow state after InO therapy. The patient is in ongoing clinical remission for more than 15 months after a second allogeneic stem cell transplantation.

### Summary/Conclusion

We discovered that VEN/AZA exposure and resistance leads to aberrant CD22 expression in AML LSCs. CD22 expression increases sensitivity towards InO which we clinically utilized for a targeted therapy approach.

**Copyright Information:** (Online) ISSN: 2572-9241
©2026 The Author(s). HemaSphere is published by John Wiley & Sons Ltd on behalf of European Hematology Association. This is an open access Abstract Book distributed under the Attribution-NonCommercial-NoDerivs (CC BY-NC-ND), which allows third parties to download the articles and share them with others as long as they credit the author and the Abstract Book, but they cannot change the content in any way or use them commercially.
**Abstract Book Citations:** Authors, Title, HemaSphere, 2026;10:(S1):pages. The abstract book DOIs can be found on the “Author Information” page.
**Disclaimer:** Articles published in the journal HemaSphere exclusively reflect the opinions of the authors. The authors are responsible for all content in their abstracts including accuracy of the facts, statements, citing resources, etc.

### (S121) VENETOCLAX SENSITIVITY IN ACUTE MYELOID LEUKAEMIA WITH RUNX1 FRAMESHIFT MUTATIONS RESULTS FROM MITOCHONDRIAL ADAPTATION TO PROTEOSTATIC STRESS

**Topic:** 03. Acute myeloid leukemia - Biology & translational research

David Palmer*[1], Nicholas Jinks[2], Alice Worker[2], Mercedes Vazquez-Cantu[2], Dan Coleman[3], Sandeep Potluri[4], Kellen Gil[5], David Boocock[6], Clare Coveney[6], Jan Jacob Schuringa[7], Peter Cockerill[3], Manoj Raghavan[3], Constanze Bonifer[3], Daniel Pollyea[5], Ben Towler[8], Rhys Morgan[8], Sophie Kellaway[2]

*[1]Brighton & Sussex Medical School, Clinical Experimental Medicine, Brighton, United Kingdom;[2]University of Nottingham, Nottingham, United Kingdom; [3]University of Birmingham, Birmingham, United Kingdom; [4]University Hospital Birmingham NHS Foundation Trust DC, Birmingham, United Kingdom; [5]The University of Colorado Anschutz Medical Campus, Aurora, United States of America; [6]Nottingham Trent University (City Campus), Nottingham, United Kingdom; [7]University Of Groningen, University College, Groningen, The Netherlands; [8]University of Sussex, Brighton, United Kingdom;*

### Background

The *RUNX1* transcription factor is mutated in around 10% of cases of acute myeloid leukaemia (AML). *RUNX1* mutated (*RUNX1*mut)-AML is a heterogenous disease, associated with chemoresistance and adverse survival outcomes. Previous findings have highlighted that *RUNX1*mut-AML responds more favourably to the non-intensive regimen, venetoclax-azacitidine (ven-aza), than intensive-chemotherapy. However, a functional understanding behind this remains unclear.

### Aims

To investigate the heterogeneity of *RUNX1*mut-AML, we aimed to characterise differences in treatment responses and molecular events in a mutation-aware analysis.

### Methods

Responses to intensive-chemotherapy were re-analysed from BEAT-AML and the German-Austrian AML Study Group. Differential gene expression analysis between missense and frameshift *RUNX1*mut-AML patients were analysed using a UK-NL cohort and BEAT-AML/Leucegene. Molecular analysis including unfolded protein load was measured by TPE-MI fluorescence and Seahorse assay in OCI-AML5$^{+/RUNX1fs}$ and (CRISPR/Cas9-repaired) OCI-AML5$^{+/+}$cells (chronic, stress-adapted model). Doxycycline inducible HA-RUNX1 frameshift proteins were expressed in OCI-AML5$^{+/+}$ cells and stability (bortezomib/cycloheximide-chase) and the integrated stress response (ISR) (acute, non-adapted model) were assessed with immunoblotting. HA-immunoprecipitation followed by mass spectrometry was carried out on wild-type vs. RUNX1p.Asp212Ilefsx25. Venetoclax sensitivity was measured in cell lines and primary patient CD34+ cells by dose response assay.

**Copyright Information:** (Online) ISSN: 2572-9241
©2026 The Author(s). HemaSphere is published by John Wiley & Sons Ltd on behalf of European Hematology Association. This is an open access Abstract Book distributed under the Attribution-NonCommercial-NoDerivs (CC BY-NC-ND), which allows third parties to download the articles and share them with others as long as they credit the author and the Abstract Book, but they cannot change the content in any way or use them commercially.
**Abstract Book Citations:** Authors, Title, HemaSphere, 2026;10:(S1):pages. The abstract book DOIs can be found on the “Author Information” page.
**Disclaimer:** Articles published in the journal HemaSphere exclusively reflect the opinions of the authors. The authors are responsible for all content in their abstracts including accuracy of the facts, statements, citing resources, etc.

**Results**

*RUNX1* frameshift mutations lead to a high unfolded protein load and induce an adaptive mitochondrial gene signature. RUNX1-frameshift proteins (with the exception of those in the last exon encoding a 3' extended reading frame) are translated, but rapidly degraded via the proteosome, following failure of chaperone-mediated folding. The resulting endoplasmic reticulum stress activates the ISR and unfolded protein response (UPR). However, due to RUNX1 haploinsufficiency the UPR does not maintain proteostasis, leading to increased metabolic demand. Expression of RUNX1-frameshift proteins activate AMPK to drive upregulation of mitochondrial genome-encoded electron transport chain proteins, with a concomitant increase in cellular spare capacity. The resulting dependency on the mitochondria yields a specific sensitivity to the BCL2 inhibitor, venetoclax, known to target leukaemic stem cell dependency on oxidative phosphorylation. We see that, compared to patients with AML either without *RUNX1* mutations or with *RUNX1*-missense mutations, patient cells with *RUNX1* frameshift mutations show a complete response to physiological doses of venetoclax in vitro and a lower IC50. Supporting this, OCI-AML5$^{+/RUNX1fs}$ cells have an increased mitochondrial gene-signature and sensitivity to venetoclax than OCI-AML5$^{+/+}$ cells.

**Summary/Conclusion**

Our data demonstrates *RUNX1* frameshift mutations in AML cells (outside of the last exon) lead to proteostatic stress and the induction of a mitochondrial-adaptation response, preferentially sensitising them to venetoclax. Patients with *RUNX1*-frameshift mutations exhibit worse outcomes following intensive chemotherapy than those with missense mutations. These findings support the design of a clinical trial comparing ven-aza and intensive chemotherapy in *RUNX1*mut-AML. Importantly, we highlight here that mutation class functionally underpins heterogeneity in *RUNX1*mut AML.

**Copyright Information:** (Online) ISSN: 2572-9241
©2026 The Author(s). HemaSphere is published by John Wiley & Sons Ltd on behalf of European Hematology Association. This is an open access Abstract Book distributed under the Attribution-NonCommercial-NoDerivs (CC BY-NC-ND), which allows third parties to download the articles and share them with others as long as they credit the author and the Abstract Book, but they cannot change the content in any way or use them commercially.
**Abstract Book Citations:** Authors, Title, HemaSphere, 2026;10:(S1):pages. The abstract book DOIs can be found on the "Author Information" page.
**Disclaimer:** Articles published in the journal HemaSphere exclusively reflect the opinions of the authors. The authors are responsible for all content in their abstracts including accuracy of the facts, statements, citing resources, etc.

### (S122) ENGINEERING CLL-1 CAR-NK CELLS VIA MRNA–LNP FOR POTENT ANTITUMOR ACTIVITY AND REVERSAL OF HLA-E–MEDIATED RESISTANCE IN ACUTE MYELOID LEUKEMIA

**Topic:** 03. Acute myeloid leukemia - Biology & translational research

Zhen Jin[1], Jie Shen[1], Peng Jin[1], Yebing Liang[1], Zeyi Li[1], Rufang Xiang[1], Jinyang Zhu[1], Yunxiang Zhang[1], Hongming Zhu[1], Wei Wang[2], Xiaoyang Li[1], Junmin Li*[1]

*[1]Shanghai Institute of Hematology, State Key Laboratory of Medical Genomics, National Research Center for Translational Medicine at Shanghai, Ruijin Hospital Affiliated to Shanghai Jiao Tong University School of Medicine, SHANGHAI, China;*
*[2]Shanghai Xinpu BioTechnology Company Limited, SHANGHAI, China;*

### Background

Acute myeloid leukemia (AML) remains a highly lethal malignancy, with relapse primarily driven by resistance to chemotherapy or targeted therapies. Existing chimeric antigen receptor T cell (CAR-T) strategies are limited by toxicity and complex manufacturing, underscoring the need for transient, controllable, and safe CAR-engineering platforms that can selectively target multidrug-resistant (MDR) AML cells.

### Aims

Develop an LNP-mRNA transfection platform to generate off-the-shelf cord blood-derived CLL1-CAR NK cells, evaluate their efficacy in multidrug-resistant AML cells and animal models, and further identify blocking the NKG2A/HLA-E axis in enhancing the antitumor function of CAR-NK cells.

### Methods

We developed a lipid nanoparticle (LNP)–based mRNA delivery platform for scalable generation of C-type lectin-like molecule-1 (CLL-1) CAR-NK cells. NK phenotype, cytotoxicity, cytokine secretion, and safety were evaluated ex vivo against AML cell lines and patient-derived blasts, with in vivo efficacy tested in xenograft NSG mouse models. Mechanisms of adaptive resistance were investigated through transcriptomic profiling, modulation of the NKG2A/HLA-E axis, and functional interrogation of the JAK2–STAT1 signaling pathway.

### Results

Drug-response profiling across AML cohorts identified a multidrug-resistant subgroup marked by a distinct transcriptomic program in which CLL-1 was the only validated CAR target upregulated(Fig1A,B). mRNA–LNP transfection enabled efficient generation of primary CLL-1 CAR-NK cells with preserved phenotype and potent(Fig1C,D), antigen-specific cytotoxicity against AML cells(Fig1E-G), while sparing normal hematopoietic progenitors(data not shown). In vivo, repeated CAR-NK infusions markedly suppressed leukemia progression and prolonged survival(Fig1H,I,N). Transcriptomic analyses of tumor cells surviving CAR-NK exposure revealed inflammatory activation with progressive HLA-E upregulation, which impaired CAR-NK function(Fig1J-M). NKG2A blockade restored cytotoxicity ex vivo and enhanced leukemia clearance and survival in vivo(Fig1O,Q). Mechanistically, prolonged CAR-NK engagement or IFN-γ stimulation activated a JAK2–STAT1 axis that drove sustained HLA-E induction(Fig1P). JAK2 knockdown reduced HLA-E expression and sensitized AML cells to CAR-NK–mediated killing, whereas pharmacologic JAK2 inhibition also decreased HLA-E expression but concurrently impaired NK-cell activation(Fig1R), thereby limiting the overall therapeutic benefit.

**Copyright Information:** (Online) ISSN: 2572-9241
©2026 The Author(s). HemaSphere is published by John Wiley & Sons Ltd on behalf of European Hematology Association. This is an open access Abstract Book distributed under the Attribution-NonCommercial-NoDerivs (CC BY-NC-ND), which allows third parties to download the articles and share them with others as long as they credit the author and the Abstract Book, but they cannot change the content in any way or use them commercially.
**Abstract Book Citations:** Authors, Title, HemaSphere, 2026;10:(S1):pages. The abstract book DOIs can be found on the "Author Information" page.
**Disclaimer:** Articles published in the journal HemaSphere exclusively reflect the opinions of the authors. The authors are responsible for all content in their abstracts including accuracy of the facts, statements, citing resources, etc.

### Summary/Conclusion

Transient, non-integrating mRNA–LNP–transfected CLL-1 CAR-NK cells provide a safe and effective strategy for MDR AML. Repeated dosing enables robust antitumor activity, while adaptive resistance via NKG2A/HLA-E axis can be mitigated through checkpoint blockade. The JAK2–STAT1 pathway represents a potential upstream modulator, providing opportunities for rational combinatorial approaches to optimize CAR-NK therapy.

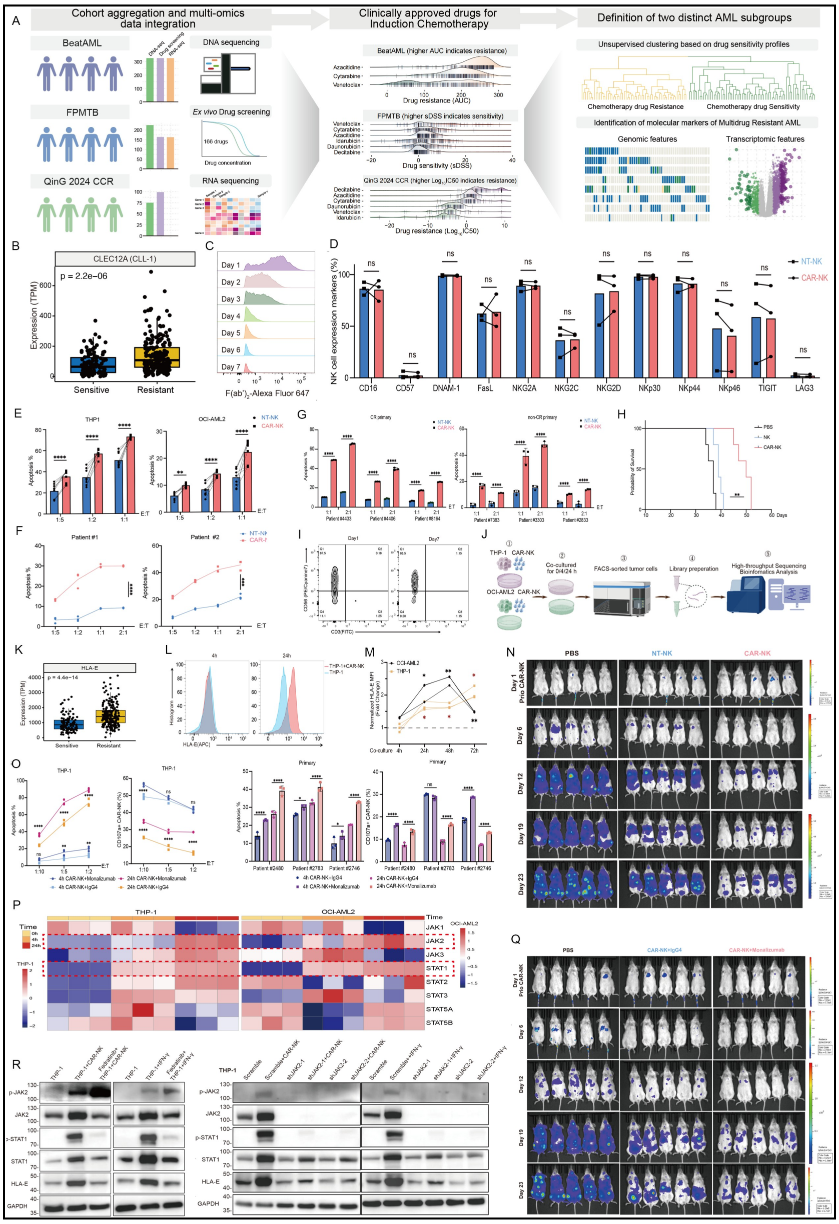

Figure1. (A) Schematic of ex vivo drug response analysis in three independent AML cohorts (FPMTB, BeatAML, and Qin et al.), focusing on key first-line agents including cytarabine, daunorubicin, venetoclax, azacitidine, idarubicin, and decitabine. (B) Comparative analysis of clinically investigated AML CAR targets revealed selective upregulation of CLL-1 in multiple drug resistant (MDR) AML. (C) The frequency of CLL-1 CAR-expressing from CB donors-derived NK cells was monitored from day 1 to day 7 post-transfection. (D) Expression of NK cell receptors after 24 h of culture. (E) Different CB-derived CLL-1 CAR-NK cells lysed CLL-1+ cell lines THP-1 and OCI-AML2 at different E:T ratios (n = 9). E:T, effector-to-target cell ratio. (F) Apoptosis of primary bone marrow (BM)-derived cells from two AML

**Copyright Information:** (Online) ISSN: 2572-9241
©2026 The Author(s). HemaSphere is published by John Wiley & Sons Ltd on behalf of European Hematology Association. This is an open access Abstract Book distributed under the Attribution-NonCommercial-NoDerivs (CC BY-NC-ND), which allows third parties to download the articles and share them with others as long as they credit the author and the Abstract Book, but they cannot change the content in any way or use them commercially.
**Abstract Book Citations:** Authors, Title, HemaSphere, 2026;10:(S1):pages. The abstract book DOIs can be found on the "Author Information" page.
**Disclaimer:** Articles published in the journal HemaSphere exclusively reflect the opinions of the authors. The authors are responsible for all content in their abstracts including accuracy of the facts, statements, citing resources, etc.

patients who were ineligible for intensive chemotherapy or Ven/Aza treatment after 4 h co-culture with NT-NK or CLL-1 CAR-NK cells at different E:T ratios. (G) Apoptosis of BM-derived cells from three AML patients who achieved complete remission (CR) or non-CR after 4 h co-culture with NT-NK or CLL-1 CAR-NK cells at different E:T ratios (n = 3). (H) Kaplan–Meier survival analysis of mice treated with PBS, NT-NK, or CLL-1 CAR-NK cells. (I) Human NK cells (hCD45+CD3-CD56+) were quantified by flow cytometry in vivo. (J) Schematic representation of RNA-seq analysis: THP-1 and OCI-AML2 cells were co-cultured with CLL-1 CAR-NK cells for 0, 4, or 24 h, and surviving tumor cells were sorted by FACS for transcriptomic profiling . (K) Differential expression of HLA-E in MDR patients versus multidrug-sensitive AML samples. (L) HLA-E surface expression on THP-1 cells cultured alone or with CAR-NK cells for 4 or 24 h. (M) Surface expression of HLA-E on “NK-resistance” THP-1 (E:T = 1:5) and “NK-sensitive” OCI-AML2 (E:T = 1:1) cells co-cultured with CLL-1 CAR-NK cells or treated with IFN-γ for 4 h, 24 h, 48 h, and 72 h . (N) BLI images of PBS-, NT-NK-, or CLL-1 CAR-NK-treated mice at days 6, 12, 19, and 23. (O) Apoptosis of THP-1 cells and percentage of CD107a+ CAR-NK cells after 4 or 24 h co-culture with CAR-NK cells in combination with IgG4 isotype control or the anti-NKG2A monoclonal antibody Monalizumab. (P) Heatmap of part of JAK-STAT family transcripts (JAK1, JAK2, JAK3, STAT1, STAT2, STAT3, STAT5A, STAT5B) in THP-1 and OCI-AML2 cells following CAR-NK co-culture. (Q) BLI of mice treated with PBS, CAR-NK cells, or CAR-NK cells in combination with monalizumab at days 6, 12, 19, and 23. (R) Western blots of JAK2–STAT1 pathway activation and HLA-E expression in THP-1 cells co-cultured with CAR-NK cell, JAK2–STAT1 pathway activation and total HLA-E expression in THP-1 cells expressing scramble control or JAK2 shRNA after 24 h of CAR-NK co-culture or IFN-γ treatment.

**Copyright Information:** (Online) ISSN: 2572-9241
©2026 The Author(s). HemaSphere is published by John Wiley & Sons Ltd on behalf of European Hematology Association. This is an open access Abstract Book distributed under the Attribution-NonCommercial-NoDerivs (CC BY-NC-ND), which allows third parties to download the articles and share them with others as long as they credit the author and the Abstract Book, but they cannot change the content in any way or use them commercially.
**Abstract Book Citations:** Authors, Title, HemaSphere, 2026;10:(S1):pages. The abstract book DOIs can be found on the “Author Information” page.
**Disclaimer:** Articles published in the journal HemaSphere exclusively reflect the opinions of the authors. The authors are responsible for all content in their abstracts including accuracy of the facts, statements, citing resources, etc.

### (S123) ENFORCING FATTY ACID SATURATION IMBALANCE ENHANCES STEAROYL-COA DESATURASE INHIBITION THERAPEUTIC EFFICACY IN ACUTE MYELOID LEUKEMIA

**Topic:** 03. Acute myeloid leukemia - Biology & translational research

Sirisha Natani*[1, 1], Sophie James[1], Alan Mitchell[1], Lori Hazlehurst[2], Melanie Lad[3], David Walter[3], Angelo D'alessandro[4], Vilma Dembitz[5], Paolo Gallipoli[1]

*[1]Barts Cancer Institute, Queen Mary University of London, Haemato-Oncology, London, United Kingdom;[2]Modulation Therapeutics, Morgantown, WV, United States of America; [3]Cancer Research UK–AstraZeneca Functional Genomics Centre, London, United Kingdom; [4]University of Colorado - Anschutz Medical Campus, Aurora, CO, United States of America;[5]University of Zagreb School of Medicine, Department of Physiology and Croatian Institute for Brain Research, Zagreb, Croatia;*

### Background

Aberrant activation of fatty acid biosynthesis (FAS) is a hallmark of many cancers including acute myeloid leukemia (AML). Sequential fatty acid (FA) synthesis, elongation and desaturation generate diverse lipid species that sustain membrane biogenesis and oncogenic signaling. Stearoyl-CoA desaturase (SCD), the rate-limiting enzyme in *de novo* FA desaturation, balances saturated FA (SFA) and monounsaturated FA (MUFA) levels to prevent SFA-induced lipotoxicity. We previously demonstrated that SCD expression is prognostic in AML and that pharmacologic SCD inhibition (SCDi) with the clinical-grade inhibitor SSI-4 (NCT06911008) induces lipotoxic cell death across AML models (Dembitz et al., Leukemia 2024). However, AML samples sensitivity varies independently of recurrent genetic lesions, suggesting metabolic compensatory pathways might drive SCDi resistance.

### Aims

To identify pathways modulating SSI-4 sensitivity and develop strategies to enhance SCDi efficacy in AML.

### Methods

Genome-wide CRISPR-Cas9 knockout and activation screens were performed under SSI-4 treatment in AML models with graded SCDi sensitivity. Candidate compensatory pathways were validated *in vitro* using viability and proliferation assays (Annexin staining/cell cycle analysis). Lipidomic profiling assessed alterations in lipid composition upon pathway targeting. Western blotting and RT-qPCR were used for protein and gene expression analysis in different experimental conditions. *In vivo* efficacy of targeting candidate compensatory pathways was evaluated in murine AML models, including for dietary interventions designed to potentiate SCDi.

**Copyright Information:** (Online) ISSN: 2572-9241
©2026 The Author(s). HemaSphere is published by John Wiley & Sons Ltd on behalf of European Hematology Association. This is an open access Abstract Book distributed under the Attribution-NonCommercial-NoDerivs (CC BY-NC-ND), which allows third parties to download the articles and share them with others as long as they credit the author and the Abstract Book, but they cannot change the content in any way or use them commercially.
**Abstract Book Citations:** Authors, Title, HemaSphere, 2026;10:(S1):pages. The abstract book DOIs can be found on the “Author Information” page.
**Disclaimer:** Articles published in the journal HemaSphere exclusively reflect the opinions of the authors. The authors are responsible for all content in their abstracts including accuracy of the facts, statements, citing resources, etc.

### Results

CRISPR screening identified fatty acid desaturase 2 (FADS2) as the strongest common mediator of resistance to SCDi. SCD and FADS2 expression were inversely correlated, and enforced FADS2 expression mitigated SSI-4–induced lipotoxicity, indicating functional redundancy. Conversely, FADS2 depletion sensitized AML cells to SCDi. Dual SCD/FADS2 targeting led to increased SFA/MUFA ratio and accumulation of ER stress–inducing lipids, including lysophospholipids and specific ceramides, accompanied by induction of ER stress effectors (CHOP, IRE1). These effects were reversed by FADS2 expression or exogenous MUFA supplementation, supporting a role for FADS2 in preserving MUFA homeostasis and protecting against SCDi-induced ER stress. As selective FADS2 inhibitors with suitable *in vivo* bioavailability are unavailable, we sought to create a metabolic desaturation bottleneck through dietary manipulation. Combination of SSI-4 with a saturated FA palmitate-enriched ketogenic diet (KDPalm) (Fig. 1a) increased the systemic and bone marrow SFA/MUFA ratio (Fig. 1b). In an SCDi-insensitive murine AML model, SSI-4 plus KDPalm significantly reduced leukemic burden and prolonged survival compared with either intervention alone (Fig. 1c-d).

### Summary/Conclusion

AML exhibits a critical dependency on FA desaturation but compensates for SCD inhibition through alternative pathways, particularly FADS2. FADS2 maintains MUFA levels thus preventing SCDi induced lipotoxic stress in AML cells. Disrupting this redundancy—genetically or via dietary modulation—creates a metabolic bottleneck that amplifies SCDi efficacy. Therapeutic strategies that enforce a critical SFA/MUFA imbalance may therefore unlock the full anti-leukemic potential of SCD inhibition.

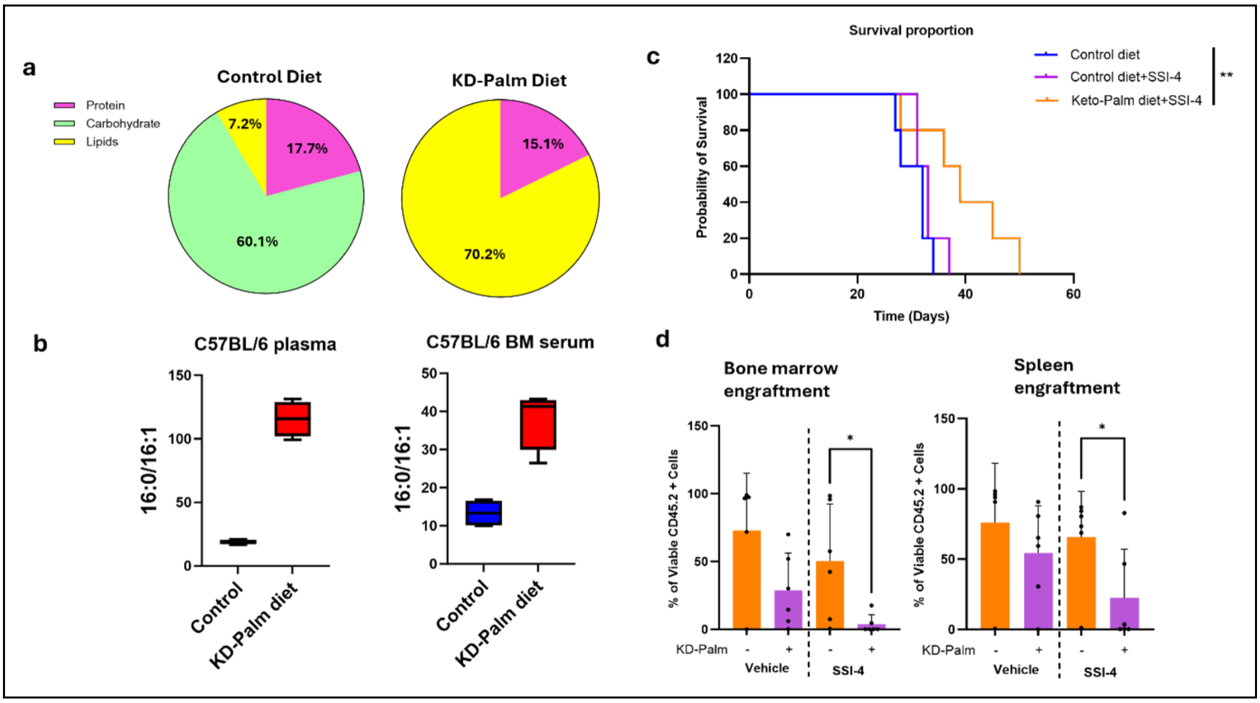

Dietary interventions can modulate SCD inhibitor sensitivity: (a) Composition of each nutrient for our control diet and ketogenic diet (KD-Palm) enriched for 16:0 (b) Changes in blood plasma and bone marrow (BM) serum of FAs 16:0/16:1 ratio following 18 days of Control or KD-Palm diet in C57BL/6.(c) Survival analysis of mice in the experimental arms (d) Bone marrow and spleen engraftment of leukaemic cells at the experimental end-point from mice in the four experimental arms. * p<0.05, ** p<0.01, **** p<0.0001

Copyright Information: (Online) ISSN: 2572-9241
©2026 The Author(s). HemaSphere is published by John Wiley & Sons Ltd on behalf of European Hematology Association. This is an open access Abstract Book distributed under the Attribution-NonCommercial-NoDerivs (CC BY-NC-ND), which allows third parties to download the articles and share them with others as long as they credit the author and the Abstract Book, but they cannot change the content in any way or use them commercially.
Abstract Book Citations: Authors, Title, HemaSphere, 2026;10:(S1):pages. The abstract book DOIs can be found on the “Author Information” page.
Disclaimer: Articles published in the journal HemaSphere exclusively reflect the opinions of the authors. The authors are responsible for all content in their abstracts including accuracy of the facts, statements, citing resources, etc.

### (S124) TRANSCRIPTIONALLY DEFINED AML CELL STATES ASSOCIATE WITH TREATMENT RESPONSE AND MICROENVIRONMENTAL REMODELING

**Topic:** 03. Acute myeloid leukemia - Biology & translational research

Nona Struyf*[1], Leonard Hartmanis[1], Lucia Rico Pizarro[1], Albin Osterroos[2], Anna Bohlin[3], Sofia Bengtzen[3], Sören Lehmann[3], Olli Kallioniemi[1, 4], Tom Erkers[1]

*[1]Karolinska Institute, Department of Oncology-Pathology, Stockholm, Sweden; [2]Uppsala University Hospital, Department of Medical Sciences, Hematology, Uppsala, Sweden; [3]Karolinska Institute, Department of Medicine, Center for Hematology and Regenerative Medicine, Stockholm, Sweden; [4]Institute for Molecular Medicine Finland, University of Helsinki, Helsinki, Finland;*

### Background

Acute myeloid leukemia (AML) is a molecularly and phenotypically heterogeneous malignancy with poor prognosis. Although therapy resistance is often attributed to leukemic stem cells (LSCs), AML cell identity and function extend beyond classical surface marker definitions. Emerging evidence suggests that leukemic cell states interact dynamically with the bone marrow microenvironment, influencing cytokine signaling, niche remodeling, and drug response. However, a functional classification of AML cell states and their clinical relevance remains incompletely defined.

### Aims

We aimed to define transcriptionally and functionally distinct AML cell states and explore how they modulate the microenvironment, influence therapy response, and associate with clinical outcome.

### Methods

Integrated scRNA-seq and CITE-seq were performed on diagnostic AML samples (n=6). Myeloid clusters were annotated using AML reference atlases and evaluated for stemness using established HSC/LSC signatures. State-specific gene signatures were projected onto bulk RNA-seq cohorts (n=448) to assess associations with ex vivo drug sensitivity (n=124), soluble proteomics (n=38), and clinical parameters. Public longitudinal single-cell datasets were used to validate findings and analyze therapy-induced shifts in cell-state composition. Cell-cell communication was inferred using CellChat and CellPhoneDB.

### Results

Clustering resolved nine conserved AML cell states across patients, including progenitor-like (cycling, stress-adapted, lymphoid-primed), stromal-like (vascular, mesenchymal), and multiple specialized monocytic or differentiated programs (antigen-presenting, cytotoxic effector, cytokine-responsive, tissue-remodeling inflammatory). CITE-seq confirmed transcript-protein concordance while revealing heterogeneity within CD34/CD38-defined populations. Stemness signatures were distributed across several cell states, indicating that LSC features are not confined to a single immunophenotypic compartment. Cell states were also defined by distinct transcriptional programs, enabling derivation of compact 20-gene signatures. Projection of state-specific signatures onto bulk RNA-seq revealed heterogeneous patient-level enrichment and distinct associations with drug sensitivity, revealing state-specific sensitivity and resistance patterns (e.g. crenolanib sensitivity in cycling progenitors). In longitudinal datasets, induction chemotherapy mainly reduced lymphoid-primed progenitors and cytotoxic effector monocytes, whereas cycling progenitor and antigen-presenting myeloid states persisted post-treatment. Venetoclax/azacitidine treatment differentially affected progenitor, stromal, and monocytic states, showing most persistence of the stress-adapted progenitors. Communication analysis predicted state-specific immune and niche signaling differences (e.g. NOTCH, THBS, ANGPT).

**Copyright Information:** (Online) ISSN: 2572-9241
©2026 The Author(s). HemaSphere is published by John Wiley & Sons Ltd on behalf of European Hematology Association. This is an open access Abstract Book distributed under the Attribution-NonCommercial-NoDerivs (CC BY-NC-ND), which allows third parties to download the articles and share them with others as long as they credit the author and the Abstract Book, but they cannot change the content in any way or use them commercially.
**Abstract Book Citations:** Authors, Title, HemaSphere, 2026;10:(S1):pages. The abstract book DOIs can be found on the "Author Information" page.
**Disclaimer:** Articles published in the journal HemaSphere exclusively reflect the opinions of the authors. The authors are responsible for all content in their abstracts including accuracy of the facts, statements, citing resources, etc.

### Summary/Conclusion

We define nine functional AML cell states that integrate lineage identity, stemness programs, and microenvironmental signaling properties. Stem-like features are embedded across multiple transcriptional states and are not adequately captured by classical surface/transcription markers alone. These states associate with drug sensitivity patterns, display treatment-specific persistence dynamics, and exhibit distinct niche-interacting programs. Functional state stratification therefore extends beyond the traditional leukemic stem cell paradigm and provides a biologically grounded framework for refining therapeutic targeting in AML.

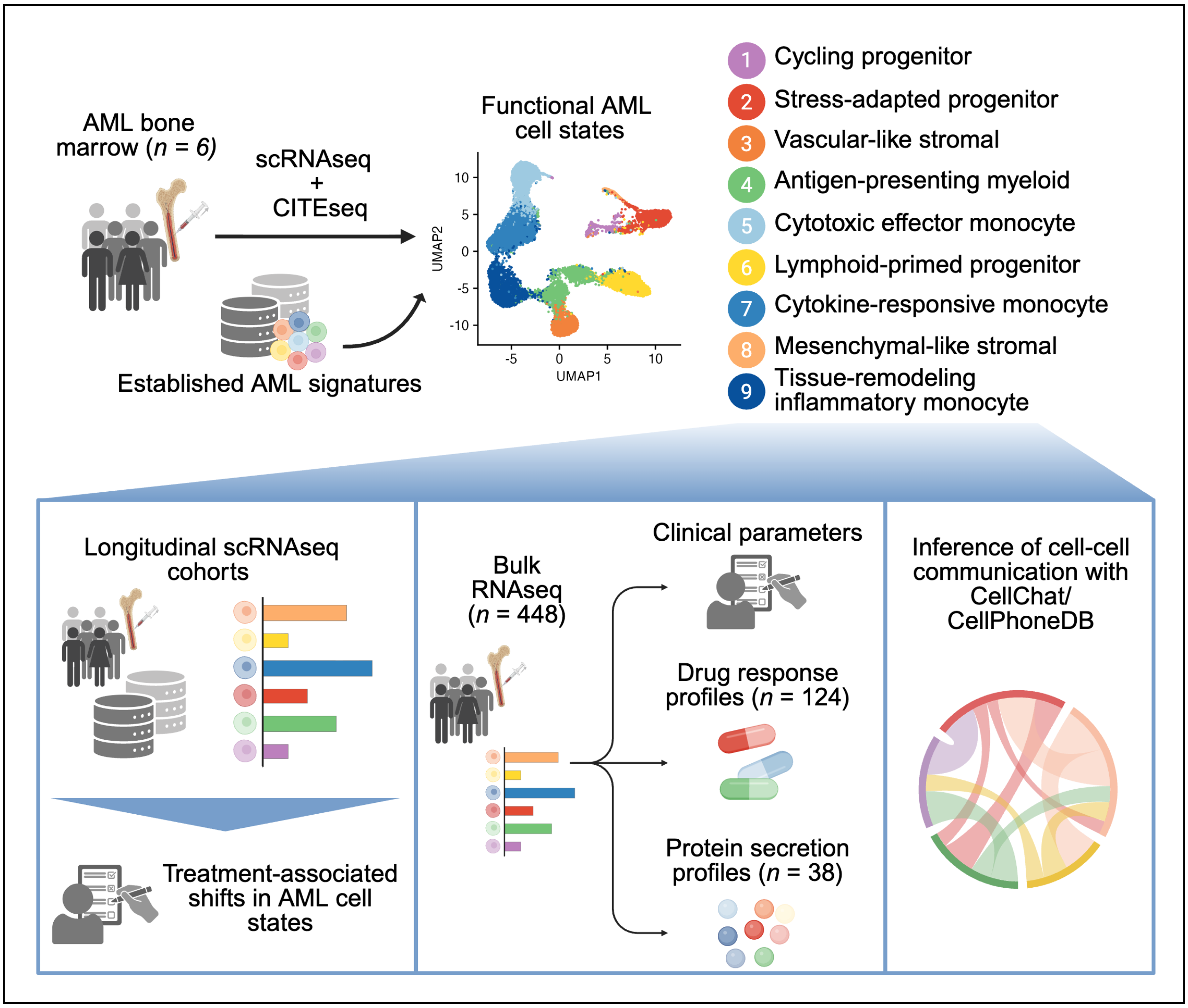

Study design and overview

Copyright Information: (Online) ISSN: 2572-9241
©2026 The Author(s). HemaSphere is published by John Wiley & Sons Ltd on behalf of European Hematology Association. This is an open access Abstract Book distributed under the Attribution-NonCommercial-NoDerivs (CC BY-NC-ND), which allows third parties to download the articles and share them with others as long as they credit the author and the Abstract Book, but they cannot change the content in any way or use them commercially.
Abstract Book Citations: Authors, Title, HemaSphere, 2026;10:(S1):pages. The abstract book DOIs can be found on the "Author Information" page.
Disclaimer: Articles published in the journal HemaSphere exclusively reflect the opinions of the authors. The authors are responsible for all content in their abstracts including accuracy of the facts, statements, citing resources, etc.

### (S125) LOW-DOSE CYTARABINE AND VENETOCLAX PLUS AZACITIDINE COMPARED WITH VENETOCLAX PLUS AZACITIDINE IN OLDER PATIENTS WITH NEWLY DIAGNOSED ACUTE MYELOID LEUKEMIA: A MULTICENTER RANDOMIZED PHASE III TRIAL

**Topic:** 04. Acute myeloid leukemia - Clinical

Xiao Han*[1], Xinlei Li[1], Mengyun Zhang[1], Ying Zhang[2], Hongju Yan[1], Zhiyuan Peng[3], Hai Yi[4], Yue Xiao[5], Kai Wan[1], Hong Liu[1], Cheng Zhang[1], Qin Wen[1], Xi Zhang[1]

*[1]Xinqiao Hospital of Army Medical University, Chongqing, China;[2]The Second Affiliated Hospital of Chongqing Medical University, chongqing, China; [3]Affiliated Hospital of Zunyi Medical University, Zunyi, China; [4]The General Hospital of Western Theater Command, Chengdu, China; [5]The Affiliated Yongchuan Hospital of Chongqing Medical University, Chongqing, China;*

### Background

The combination of venetoclax plus azacitidine (VA) regimen for older patients with acute myeloid leukemia (AML) improves remission rates and prolongs survival compared with azacitidine alone.

### Aims

We aimed to compare outcomes of low-dose cytarabine (LDAC) and VA (VAA) induction regimen with those of VA regimen in older patients with newly diagnosed AML (ND-AML).

### Methods

This is an open-label, randomized phase III trial at 6 hospital in China. Eligible patients with AML (60-90 years) were randomly assigned 1:1 to receive VAA (venetoclax 100mg on day 1, 200mg on day 2, and 400mg on days 3 to 28; azacitidine 75mg/m$^2$ on days 1 to 7; cytarabine 10mg/m$^2$ twice daily on days 1 to 7) or VA (same dose of venetoclax and azacitidine). The primary endpoint was the composite complete remission (CRc) in the intention-to-treat (ITT) population and minimal residual disease (MRD)-negative rate in the per-protocol population.

**Copyright Information:** (Online) ISSN: 2572-9241
©2026 The Author(s). HemaSphere is published by John Wiley & Sons Ltd on behalf of European Hematology Association. This is an open access Abstract Book distributed under the Attribution-NonCommercial-NoDerivs (CC BY-NC-ND), which allows third parties to download the articles and share them with others as long as they credit the author and the Abstract Book, but they cannot change the content in any way or use them commercially.
**Abstract Book Citations:** Authors, Title, HemaSphere, 2026;10:(S1):pages. The abstract book DOIs can be found on the “Author Information” page.
**Disclaimer:** Articles published in the journal HemaSphere exclusively reflect the opinions of the authors. The authors are responsible for all content in their abstracts including accuracy of the facts, statements, citing resources, etc.

### Results

From October 31, 2023, to October 20, 2025, 187 patients were randomly assigned to receive the VAA (n=93) or VA (n=94) regimen. The median age was 67 years (range, 60-85 years). The CRc was 77.4% and 60.6% (RR, 0.45; 95%CI, 0.24-0.85, p=0.013), and MRD-negative rate after CRc was 79.2% and 45.6% (RR, 0.22; 95%CI, 0.1-0.48, p<0.001), respectively (show in table). The median follow-up was 12.6 (IQR, 7.1-21.9) months after random assignment, the median overall survival (OS) was not yet reached and 13.8 months (HR, 0.6; 95%CI, 0.38-0.93, p=0.022), median EFS was 22.3 and 11.6 months (HR, 0.52; 95%CI, 0.35-0.78, p=0.002), and median time of CIR was not yet reached and 14.4 months (HR, 0.56; 95%CI, 0.32-0.99, p=0.044), respectively (show in figure). Common grade 3 or greater adverse events (AEs) were leukocytopenia (89.2% vs 78.7%, p=0.05), thrombocytopenia (69.9% vs 54.3%, p=0.028), febrile neutropenia (63.4% vs 44.7%, p=0.01), and neutropenia (62.4% vs 58.5%, p=0.59). All AEs were controllable, and there was no death within 4 weeks.

| Outcomes | All, n=187 (%) (95% CI) | VAA, n=93 (%) (95% CI) | VA, n=94 (%) (95% CI) | RR (95%CI, P) |
|---|---|---|---|---|
| ORR (CRc + PR) | 140 (74.9) (67.9-80.7) | 77 (82.8) (74.2-90.3) | 63 (67) (58.5-76.6) | 0.422 (0.212-0.841, 0.013) |
| CRc rate (CR + CRi) | 129 (69) (62.1-75.8) | 72 (77.4) (68.8-86) | 57 (60.6) (51.1-70.2) | 0.449 (0.237-0.851, 0.013) |
| Best response | | | | |
| CR | 118 (63.1) (55.6-70.6) | 66 (71) (61.3-80.6) | 52 (55.3) (45.7-64.9) | 0.506(0.277-0.927, 0.027) |
| CRi | 11 (5.9) (2.7-9.6) | 6 (6.5) (2.2-11.8) | 5 (5.3) (1.1-10.6) | - |
| PR | 11 (5.9) (2.7-9.6) | 5 (5.4) (1.1-10.8) | 6 (6.4) (2.1-11.7) | - |
| NR | 47 (25.1) (18.7-31.6) | 16 (17.2) (9.7-24.7) | 31 (33) (24.5-42.6) | - |
| MRD - Negative after CRc | 83 (64.3) (55.8-72.8) | 57 (79.2) (69.4-88.9) | 26 (45.6) (33.3-57.9) | 0.221 (0.102-0.477, <0.001) |
| Median time to assessment of response (95% Cl), d | 31 (28-34, 17-42) (30-32) | 32 (29-34, 23-39) (31-33) | 30 (28-33.25, 17-42) (29-30.5) | - |

### Summary/Conclusion

Cytarabine and venetoclax plus azacitidine regimen has higher remission rate and MRD-negative rate, and longer overall survival compared with venetoclax plus azacitidine regimen.

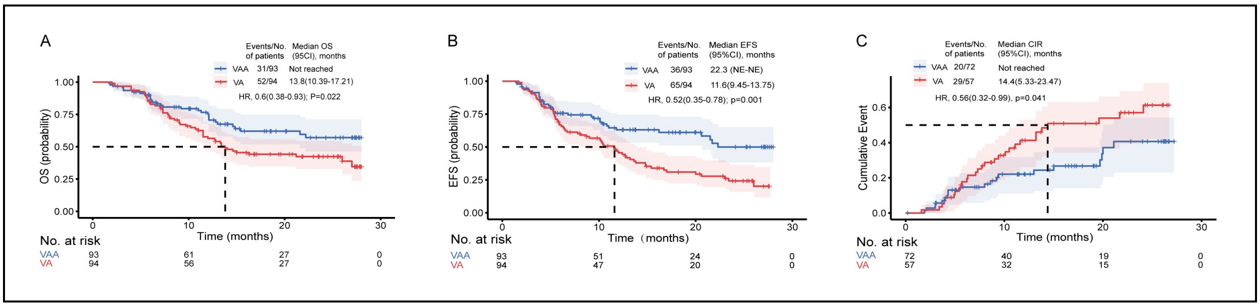

Survival analysis. (A) Overall survival in the intent-to-treat (ITT) population; (B) Event-free survival in the ITT population; (C) Cumulative incidence in the CRc population.

**Copyright Information:** (Online) ISSN: 2572-9241
©2026 The Author(s). HemaSphere is published by John Wiley & Sons Ltd on behalf of European Hematology Association. This is an open access Abstract Book distributed under the Attribution-NonCommercial-NoDerivs (CC BY-NC-ND), which allows third parties to download the articles and share them with others as long as they credit the author and the Abstract Book, but they cannot change the content in any way or use them commercially.
**Abstract Book Citations:** Authors, Title, HemaSphere, 2026;10:(S1):pages. The abstract book DOIs can be found on the "Author Information" page.
**Disclaimer:** Articles published in the journal HemaSphere exclusively reflect the opinions of the authors. The authors are responsible for all content in their abstracts including accuracy of the facts, statements, citing resources, etc.

### (S126) OPTI-AML: PROSPECTIVE RANDOMIZED PHASE 2 TRIAL OF 28 VERSUS 14 DAY SCHEDULE OF VENETOCLAX AND AZACITIDINE FOR 2 CYCLES IN NEWLY DIAGNOSED GENOMICALLY AGNOSTIC ACUTE MYELOID LEUKEMIA PATIENTS ≥60 YEARS

**Topic:** 04. Acute myeloid leukemia - Clinical

Uma Borate*[1], Ying Huang[1], Tara L. Lin[2], Shivani Handa[1], Ronan Swords[3], Curtis Lachowiez[3], Elie Traer[3], Wendy Stock[4], Olatoyosi Odenike[4], Anand Patel[4], Maria Baer[5], Vu Duong[5], Eytan Stein[6], William Blum[7], Colin Vale[7], Emily Curran[8], Yazan F. Madanat[9], Rebecca Olin[10], Gary Schiller[11], Angela Nichols[12], Lacey Williams[12], Nyla Heerema[1], Mary Johnson[13], Timothy Chen[1], Sonja Marcus[14], Molly Martycz[1], Mona Stefanos[1], Leonard Rosenberg[14], Louis Degennaro[14], Lore Gruenbaum[14], Amy Burd[14], John Byrd[15], Brian Druker[3], Ross Levine[6], Ashley Yocum[14], Alice Mims[1], Joshua Zeidner[12]

*[1]The Ohio State University, Columbus, United States of America;[2]University of Kansas Medical Center, Kansas City, United States of America; [3]Knight Cancer Institute, Oregon Health & Science University, Portland, United States of America;[4]University of Chicago Comprehensive Cancer Center, Chicago, United States of America; [5]University of Maryland Greenebaum Comprehensive Cancer Center, Baltimore, United States of America; [6]Memorial Sloan Kettering Cancer Center, New York, United States of America; [7]Winship Cancer Institute of Emory University, Atlanta, United States of America;[8]University of Cincinnati, Cincinnati, United States of America; [9]University of Texas Southwestern Medical Center, Dallas, United States of America; [10]University of California San Francisco, San Francisco, United States of America; [11]David Geffen School of Medicine at University of California Los Angeles, Los Angeles, United States of America; [12]Lineberger Comprehensive Cancer Center, University of North Carolina, Chapel Hill, United States of America; [13]Mjbiostat, Spring Lake, United States of America; [14]Blood Cancer United, Washington, United States of America; [15]UPMC Hillman Cancer Center, Pittsburgh, United States of America;*

### Background

Azacitidine (aza) and Venetoclax (ven) for older AML patients (pts) unfit for induction, based on the VIALE-A study, required frequent (53%) ven dose reductions to mitigate cycle delays (72%) due to significant cytopenias with the 28-day(D) schedule.Retrospective studies have explored shorter ven durations as novel agents are added to the aza/ven backbone and the 28D ven schedule becomes challenging, with additive myelosuppression. We report results from OPTI-AML, the first prospective randomized phase 2 trial comparing 14D versus 28D ven for 2 cycles in a genomically agnostic newly diagnosed (ND) older AML population.

### Methods

OPTI-AML (NCT03013998) with 12 academic participating centers, randomized ND AML pts ≥60y to aza (75 mg/m² D1–7)/ven D1–28 (AV28) or D1–14 (AV14) for cycles 1 (C1) and 2 (C2). Primary endpoint was complete remission (CR) rate after 2 cycles, assessed D21-28 per ELN 2017 criteria using the Cochran-Mantel-Haenszel test. Pts continued treatment per physician discretion post-C2.166 pts (83/arm) provided 80% power to detect a 10% non-inferiority margin (one-sided α=0.05), assuming a CR rate of 36% in AV28 and 45% in AV14. The upper 95% confidence interval (CI) of the difference in CR rates [CR (AV28)–CR(AV14)] had to be ≤10% to infer AV14 as non-inferior to AV28.

**Copyright Information:** (Online) ISSN: 2572-9241
©2026 The Author(s). HemaSphere is published by John Wiley & Sons Ltd on behalf of European Hematology Association. This is an open access Abstract Book distributed under the Attribution-NonCommercial-NoDerivs (CC BY-NC-ND), which allows third parties to download the articles and share them with others as long as they credit the author and the Abstract Book, but they cannot change the content in any way or use them commercially.
**Abstract Book Citations:** Authors, Title, HemaSphere, 2026;10:(S1):pages. The abstract book DOIs can be found on the "Author Information" page.
**Disclaimer:** Articles published in the journal HemaSphere exclusively reflect the opinions of the authors. The authors are responsible for all content in their abstracts including accuracy of the facts, statements, citing resources, etc.

**Results**

From 2023-2025,169 pts were randomized(83 in AV28, 86 in AV14). Baseline characteristics were balanced other than more men in AV14 (65% vs 49%; p=0.04) [Table 1a]. After 2 cycles, CR rate was 49% (AV28) vs 43% (AV14) with a 95% CI of -8% to 21% for the difference. Non-inferiority was not established as the upper limit (21%) was >10%. CR rate was 26% in both arms after C1. In subset analysis, *NPM1* & *IDH1/2* mutated pts achieved higher CR rates with AV28 (61% vs 42%) whereas CR rates were 44% in both arms for the remaining molecular subgroups [Table 1b]. Composite CR (cCR: CR/CRh/CRi) rates were 81% (AV28) vs 69% (AV14). Among responders, flow-based MRD negative (sensitivity 0.02%) rates were 78% (AV28) and 77% (AV14) post C2. Significantly fewer pts in AV28 received the full planned ven duration in C1 (68% vs 89%; p<0.01) primarily due to infection-related adverse events (AE). For C2, 67% (AV28) and 76% (AV14) received the entire ven course. In both arms, incomplete ven delivery was associated with lower CR rates (39% vs 56% in AV28; 36% vs 49% in AV14).
In pts with cCR, ANC recovery by C1D42 was higher in AV14 (77% vs 65%; p=.01), but similar by C2. Platelet recovery did not differ. Grade ≥3 treatment-emergent AEs and early mortality were similar [Table 1c]. With a median follow-up of 9.6 months, median overall survival (OS) was not reached (12 months-NR) for AV28 and 13.6 months (9.6-22.8) for AV14 (p<0.05). Multivariable analyses showed *TP53/KRAS* mutations, platelet and white blood cell count but not treatment arm were significant for their impact on OS.

**Summary/Conclusion**

The study did not show a clinically important improvement in CR rates with AV14 vs AV28 in a genomically agnostic older AML population. CR rates were higher for *NPM1* & *IDH1/2* subgroups with AV28, but small numbers limit interpretation. MRD clearance and toxicity profile were similar across arms. Our study demonstrates the challenges in delivering continuous AV28 per label, however AV14 appears to not be equivalent to AV28 for all genomic subgroups given the heterogeneous nature of AML. Development of aza/ven-based triplet regimens warrants further study to optimize ven duration in molecularly defined subsets.

**Copyright Information:** (Online) ISSN: 2572-9241
©2026 The Author(s). HemaSphere is published by John Wiley & Sons Ltd on behalf of European Hematology Association. This is an open access Abstract Book distributed under the Attribution-NonCommercial-NoDerivs (CC BY-NC-ND), which allows third parties to download the articles and share them with others as long as they credit the author and the Abstract Book, but they cannot change the content in any way or use them commercially.
**Abstract Book Citations:** Authors, Title, HemaSphere, 2026;10:(S1):pages. The abstract book DOIs can be found on the “Author Information” page.
**Disclaimer:** Articles published in the journal HemaSphere exclusively reflect the opinions of the authors. The authors are responsible for all content in their abstracts including accuracy of the facts, statements, citing resources, etc.

| Table 1a: Baseline Demographics | Overall (N=169) | AV28 (N=83) | AV14 (N=86) | p-value |
| --- | --- | --- | --- | --- |
| Age at consent, years, Median (range) | 73 (60-90) | 72 (60-88) | 73 (60-90) | 0.62 |
| Age ≥ 75 years, no. (%) | 65 (38.5) | 33 (39.8) | 32 (37.2) | 0.73 |
| Gender, no. (%) | | | | |
| Female | 72 (42.6) | 42 (50.6) | 30 (34.9) | **0.04** |
| Male | 97 (57.4) | 41 (49.4) | 56 (65.1) | |
| Race, no. (%) | | | | |
| Caucasian | 147 (89.1) | 71 (88.8) | 76 (89.4) | |
| African American | 13 (7.9) | 6 (7.5) | 7 (8.2) | 0.93 |
| Other | 5 (3.0) | 3 (3.8) | 2 (2.4) | |
| Unknown | 4 | 3 | 1 | |
| Ethnicity, no. (%) | | | | |
| Not Hispanic or Latino | 162 (98.8) | 80 (98.8) | 82 (98.8) | >0.99 |
| Hispanic or Latino | 2 (1.2) | 1 (1.2) | 1 (1.2) | |
| Unknown | 5 | 2 | 3 | |
| ECOG Performance Status, no. (%) | | | | |
| 0 | 51 (30.9) | 27 (33.3) | 24 (28.6) | |
| 1 | 80 (48.5) | 40 (49.4) | 40 (47.6) | 0.55 |
| 2 | 34 (20.6) | 14 (17.3) | 20 (23.8) | |
| Unknown | 4 | 2 | 2 | |
| **Mutations (Any VAF>0), n (%)** | **Overall (N=169)** | **AV28 (N=83)** | **AV14 (N=86)** | |
| *NPM1* | 10 (5.9) | 6 (7.2) | 4 (4.7) | 0.53 |
| *IDH2* | 29 (17.2) | 18 (21.7) | 11 (12.8) | 0.13 |
| *IDH1* | 17 (10.1) | 7 (8.4) | 10 (11.6) | 0.49 |
| *NRAS* | 13 (7.7) | 5 (6.0) | 8 (9.3) | 0.42 |
| *KRAS* | 8 (4.7) | 4 (4.8) | 4 (4.7) | >0.99 |
| *TP53* | 32 (18.9) | 13 (15.7) | 19 (22.1) | 0.29 |
| **Cytogenetics, n (%)** | **Overall (N=168)** | **AV28 (N=82)** | **AV14 (N=86)** | |
| Core Binding Factor | 8 (4.9) | 1 (1.3) | 7 (8.2) | 0.07 |
| Complex Karyotype | 50 (30.5) | 22 (27.9) | 28 (32.9) | 0.48 |
| **ELN 2024 Risk Classification** | **(N=169)** | **(N=83)** | **(N=86)** | |
| Favorable | 110 (65.1) | 58 (70.0) | 52 (60.5) | |
| Intermediate | 27 (16.0) | 12 (14.5) | 15 (17.4) | 0.42 |
| Adverse | 32 (18.9) | 13 (15.7) | 19 (22.1) | |

| Table 1b: CR Rate Per Mutation n (%) | Overall | AV28 | AV14 |
| --- | --- | --- | --- |
| *NPM1* | 6/10 (60) | 5/6 (83.3) | 1/4 (25) |
| *IDH1* | 11/17 (64.7) | 5/7 (71.4) | 6/10 (60) |
| *IDH2* | 14/29 (48.3) | 10/18 (55.6) | 4/11 (36.4) |
| *TP53* | 18/32 (56.3) | 7/13 (53.9) | 11/19 (57.9) |
| Complex karyotype | 23/50 (46) | 9/22 (41) | 14/28 (50) |
| *NPM1* or *IDH1/2* | 27/52 (52) | **17/28 (60.7)** | **10/24 (41.7)** |
| Other molecular groups (excl. *NPM1/IDH1/2*) | 51/117 (48.7) | **24/55 (43.6)** | **27/62 (43.6)** |

| Table 1c: Adverse Events | Overall (N=169) | AV28 (N=83) | AV14 (N=86) |
| --- | --- | --- | --- |
| Grade ≥3 treatment-emergent Adverse Events, n (%) | 148 (87.5) | 73 (88) | 75 (87) |
| Serious adverse events, n (%) | 64 (38) | 28 (34) | 36 (42) |
| Number of Deaths in 7 days | 1 (0.6) | 0 (0) | 1 (1.2) |
| Number of Deaths in 30 days | 7 (4.1) | 4 (4.8) | 3 (3.5) |
| Number of Deaths in 60 days | 13 (7.7) | 6 (7.2) | 7 (8.1) |
| Cause of Death | (N=68) | (N=27) | (N=41) |
| AML/Progressive disease | 42 (65.6) | 18 (72.0) | 24 (61.5) |
| Respiratory failure | 7 (10.9) | 6 (24.0) | 1 (2.6) |
| Sepsis | 7 (10.9) | 0 (0) | 7 (18.0) |
| Infection | 2 (3.1) | 1 (4.0) | 1 (2.6) |
| Bleeding | 2 (3.1) | 0 (0) | 2 (5.1) |
| Cardiac complications | 2 (3.1) | 0 (0) | 2 (5.1) |
| Acute ischemic stroke | 1 (1.6) | 0 (0) | 1 (2.6) |
| Other | 1 (1.6) | 0 (0) | 1 (2.6) |
| Unknown | 4 | 2 | 2 |

**Copyright Information:** (Online) ISSN: 2572-9241
©2026 The Author(s). HemaSphere is published by John Wiley & Sons Ltd on behalf of European Hematology Association. This is an open access Abstract Book distributed under the Attribution-NonCommercial-NoDerivs (CC BY-NC-ND), which allows third parties to download the articles and share them with others as long as they credit the author and the Abstract Book, but they cannot change the content in any way or use them commercially.
**Abstract Book Citations:** Authors, Title, HemaSphere, 2026;10:(S1):pages. The abstract book DOIs can be found on the “Author Information” page.
**Disclaimer:** Articles published in the journal HemaSphere exclusively reflect the opinions of the authors. The authors are responsible for all content in their abstracts including accuracy of the facts, statements, citing resources, etc.

### (S127) A MULTICENTER PHASE 1B/2 TRIAL OF AZACITIDINE, VENETOCLAX, AND IVOSIDENIB IN IDH1-MUTATED ACUTE MYELOID LEUKEMIA (AML)

**Topic:** 04. Acute myeloid leukemia - Clinical

Jennifer Marvin-Peek*[1], Jacqueline Garcia[2], Gautam Borthakur[1], Nicholas Short[1], Marlise Luskin[2], Curtis Lachowiez[3], Tapan Kadia[1], Abhishek Maiti[1], Lucia Masarova[1], Naval Daver[1], Sanam Loghavi[4], Koichi Takahashi[1], Ghayas Issa[1], Elie Traer[3], Ronan Swords[3], Rebecca Tidwell[5], Evan Chen[2], Rahul Vedula[2], Akriti Jain[6], Guillermo Montalban-Bravo[1], Musa Yilmaz[1], Dono Tippett[1], Dylan Wilson[2], Lydia Boer[2], Kelly Chien[1], Nitin Jain[1], Prithviraj Bose[1], Farhad Ravandi[1], Hetty Carraway[6], Guillermo Garcia-Manero[1], Hagop Kantarjian[1], Courtney Dinardo[1]

*[1]University of Texas MD Anderson Cancer Center, Leukemia, Houston, United States of America;[2]Dana-Farber Cancer Institute, Department of Medical Oncology, Boston, United States of America; [3]Oregon Health & Science University, Division of Hematology/Oncology, Portland, United States of America; [4]University of Texas MD Anderson Cancer Center, Department of Hematopathology, Houston, United States of America; [5]University of Texas MD Anderson Cancer Center, 5Department of Statistics, Houston, United States of America; [6]Taussig Cancer Institute, Cleveland Clinic, Leukemia Program, Clevelans, United States of America;*

### Background

Outcomes for patients (pts) with *IDH1*$^{mut}$ AML have improved with targeted therapies. Two doublet regimens are approved for *IDH1*$^{mut}$ AML: venetoclax (VEN) with a hypomethylating agent (HMA), and ivosidenib (IVO) plus azacitidine (AZA). Both regimens are effective, yet a substantial proportion of pts fail to respond or eventually relapse.

### Aims

We aimed to improve outcomes by combining all three agents into a “triplet” regimen. Herein, we report results from the fully enrolled newly diagnosed (ND) cohort of the multicenter phase 1b/2 study of AZA+VEN+IVO for *IDH1*$^{mut}$ AML (NCT03471260).

### Methods

Adults ≥18 years with ND *IDH1*$^{mut}$ AML not eligible for standard induction therapy and treated at the RP2D in the phase 1b or 2 cohorts were included. Pts received AZA 75 mg/m$^2$ IV on days 1-7, VEN 400mg PO on days 1-14, and IVO 500mg PO continuously. Dose attenuations in remission were permitted to minimize myelosuppression. The primary objectives were to determine the safety and overall response rate (ORR=CR+CRh+CRi+MLFS).

**Copyright Information:** (Online) ISSN: 2572-9241
©2026 The Author(s). HemaSphere is published by John Wiley & Sons Ltd on behalf of European Hematology Association. This is an open access Abstract Book distributed under the Attribution-NonCommercial-NoDerivs (CC BY-NC-ND), which allows third parties to download the articles and share them with others as long as they credit the author and the Abstract Book, but they cannot change the content in any way or use them commercially.
**Abstract Book Citations:** Authors, Title, HemaSphere, 2026;10:(S1):pages. The abstract book DOIs can be found on the “Author Information” page.
**Disclaimer:** Articles published in the journal HemaSphere exclusively reflect the opinions of the authors. The authors are responsible for all content in their abstracts including accuracy of the facts, statements, citing resources, etc.

### Results

From Nov 2019 to Oct 2025, 40 pts with ND AML at four academic centers initiated treatment (Phase 1b: 9, Phase 2: 31). The median age was 72 years (range 51-80), and 26 pts (65%) were male. AML was classified as *de novo* in 21 (53%), therapy-related in 4 (10%), and secondary to antecedent myeloid neoplasm in 15 (38%). Six (15%) pts received a prior HMA. Co-mutations included 10 with *NPM1* (25%), 3 *RAS* (8%), 2 *FLT3*-ITD (5%), and 2 *TP53* (5%). Fourteen pts (35%) had co-signaling pathway mutations. The ORR was 95% (38/40) with 93% (37/40) achieving a composite CR (CRc) (Figure 1). The median number of cycles to best response was two (range, 1-7). Measurable residual disease (MRD) negativity by flow cytometry (sensitivity <0.1%) was achieved in 91% (31/34) of MRD-evaluable CRc responders. With a median follow up of 35 months, median overall survival (OS) and duration of remission (DOR) were not reached. The 3-year OS was 79% (95% CI 64-96%). Pts with co-occurring signaling mutations had lower CRc rates (79% vs 100%, $p$=0.037) and 3-year OS (49% [95% CI 34-100%] vs 91% [95% CI 79-100%]), $p$=0.011.
Median cycle length was 35 days (range 28-79) for cycle 1 (C1) and remained 35 days for C2-12. By C12, the median treatment duration was reduced to 5 days of AZA and 7 days of VEN. One pt stopped both AZA and VEN due to persistent cytopenias, while the rest were maintained on all three agents at attenuated dosing.
To date, three pts relapsed (all with *IDH1*-negative clones).18 pts (45%) transitioned to stem cell transplant (SCT) after a median of 3 cycles (range 2-5). The 3-year DOR censored and not-censored at SCT was 82% (95% CI 68-98%) and 83% (95% CI 64-100%), respectively. Among the 22 non-transplanted pts, the median number of cycles received was 14, with 10 pts (45%) remaining on study. Reasons for discontinuation included lack of response/relapse (n=5), concurrent malignancy requiring therapy (n=2), adverse events (AEs; n=2), and pt preference (n=1).
Non-hematologic AEs occurred in 32 pts (80%), with 12 (30%) grade ≥3 AEs. Most grade ≥3 AEs were infectious (n=7, 23%), although two pts each experienced grade ≥3 QTc prolongation, tumor lysis syndrome, and differentiation syndrome, all successfully managed with supportive care and/or dose modifications.

### Summary/Conclusion

Upfront triplet therapy with AZA+VEN+IVO demonstrates high MRD negative response rates, durable remissions, and comparable safety to doublet regimens in this multicenter study for pts with ND *IDH1*$^{mut}$ AML.

© 2026 American Society of Clinical Oncology, Inc. Reused with permission. This abstract was accepted and previously presented at the 2026 ASCO Annual Meeting. All rights reserved.

**Copyright Information:** (Online) ISSN: 2572-9241
©2026 The Author(s). HemaSphere is published by John Wiley & Sons Ltd on behalf of European Hematology Association. This is an open access Abstract Book distributed under the Attribution-NonCommercial-NoDerivs (CC BY-NC-ND), which allows third parties to download the articles and share them with others as long as they credit the author and the Abstract Book, but they cannot change the content in any way or use them commercially.
**Abstract Book Citations:** Authors, Title, HemaSphere, 2026;10:(S1):pages. The abstract book DOIs can be found on the "Author Information" page.
**Disclaimer:** Articles published in the journal HemaSphere exclusively reflect the opinions of the authors. The authors are responsible for all content in their abstracts including accuracy of the facts, statements, citing resources, etc.

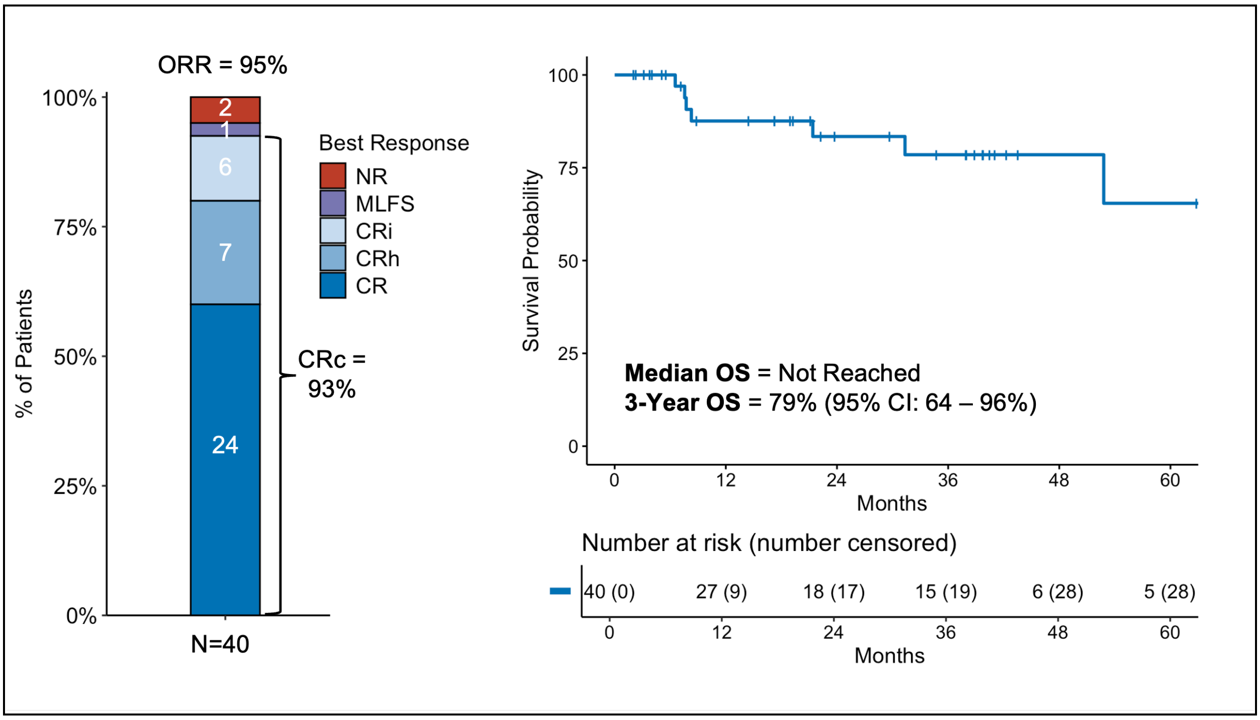

**Copyright Information:** (Online) ISSN: 2572-9241
©2026 The Author(s). HemaSphere is published by John Wiley & Sons Ltd on behalf of European Hematology Association. This is an open access Abstract Book distributed under the Attribution-NonCommercial-NoDerivs (CC BY-NC-ND), which allows third parties to download the articles and share them with others as long as they credit the author and the Abstract Book, but they cannot change the content in any way or use them commercially.
**Abstract Book Citations:** Authors, Title, HemaSphere, 2026;10:(S1):pages. The abstract book DOIs can be found on the "Author Information" page.
**Disclaimer:** Articles published in the journal HemaSphere exclusively reflect the opinions of the authors. The authors are responsible for all content in their abstracts including accuracy of the facts, statements, citing resources, etc.

### (S128) VENETOCLAX AND AZACITIDINE FOR MOLECULAR FAILURE INTERVENTION AS A BRIDGE-TO-TRANSPLANT STRATEGY FOR NPM1-MUTATED ACUTE MYELOID LEUKEMIA: THE GIMEMA AML2521 PHASE 2 TRIAL

**Topic:** 04. Acute myeloid leukemia - Clinical

Chiara Sartor*[1], Anna Candoni[2, 3], Cristina Papayannidis[1], Giovanni Marsili[4], Alfonso Piciocchi[4], Paola Fazi[4], Jacopo Nanni[5], Stefania Paolini[1], Lorenzo Brunetti[6], Luca Maurillo[7], Mario Tiribelli[3], Erika Borlenghi[8], Antonino Mulè[9], Melissa Campanelli[10], Marianna Norata[11], Roberto Cairoli[12], Emanuela Ottaviani[1], Giulia Atzeni[5], Maria Teresa Voso[7], Francesca Bonifazi[13], Enrico Maffini[13], Cristina Skert[14], Francesco Di Raimondo[15], Roberto M. Lemoli[16], Pellegrino Musto[17], Maria Paola Martelli[18], Brunangelo Falini[18], Adriano Venditti[7], Francesco Buccisano[7], Antonio Curti[1]

*[1]IRCCS Azienda Ospedaliero-Universitaria di Bologna, Istituto di Ematologia "Seràgnoli", Bologna, Italy;[2]Department of Hematology, AULSS2, Ca' Foncello Hospital, Treviso, Italy; [3]Division of Hematology and BMT, Department of Medical Area, University of Udine, Udine, Italy; [4]GIMEMA foundation, Roma, Italy; [5]Dipartimento di Scienze Mediche e Chirurgiche, Università di Bologna, Bologna, Italy; [6]Hematology Unit, Department of Clinical and Molecular Sciences, DISCLIMO, Università Politecnica delle Marche, Ancona, Italy; [7]Ematologia, Dipartimento di Biomedicina e Prevenzione, Università Tor Vergata di Roma,, Roma, Italy; [8]SC Ematologia, ASST Spedali Civili, Brescia, Italy; [9]AO Ospedali Riuniti Villa Sofia Cervello - Palermo – Uo Ematologia Ad Indirizzo Oncologico, Palermo, Italy; [10]Department of Hematology, Azienda USL - IRCCS di Reggio Emilia, Reggio Emilia, Italy; [11]IRCCS Istituto Romagnolo per lo Studio de Tuori (IRST) "Dino Amadori", Meldola, Italy;[12]ASST Grande Ospedale Metropolitano Niguarda, Milano, Italy; [13]IRCCS Azienda Ospedaliero-Universitaria di Bologna, Bologna, Italy; [14]UOC Ematologia, Ospedale dell'Angelo, Mestre, Italy; [15]AOU Policlinico Vittorio Emanuele, PO Ospedaliero "G. Rodolico" - UO Ematologia con trapianto di Midollo Osseo, Catania, Italy; [16]IRCCS AOU San Martino - Genova - UO Clinica Ematologica, Genova, Italy; [17]Dipartimento di Medicina di Precisione e Rigenerativa, Area Ionica, Università "Aldo Moro", Unità di Ematologia e Trapianto di Midollo Osseo, AOUC Policlinico, Bari, Italy; [18]Ematologia e Immunologia Clinica, Dipartimento di Medicina e Chirurgia, Università degli Studi di Perugia, e Azienda Ospedaliera 'Santa Maria della Misericordia ' di Perugia, Perugia, Italy;*

### Background

Molecular failure (MF) after 1st-line intensive chemotherapy (IC) in *NPM1*-mutated (m) acute myeloid leukemia (AML) identifies high-risk patients (pts) who benefit from allogeneic stem cell transplantation (HSCT). No therapies are approved for measurable residual disease (MRD) positive AML despite growing evidence supporting early MRD-driven intervention. Bcl-2 inhibitor venetoclax (VEN) plus azacitidine (AZA) have shown exquisite sensitivity in *NPM1*m AML, paving the way for its application in the MF setting.

### Aims

To explore the efficacy of VEN-AZA in preventing overt relapse and as a bridge-to-transplant strategy in*NPM1*m AML pts in MF after IC.

### Methods

The GIMEMA AML2521 (NCT04867928) is a phase 2 multicenter trial enrolling fit-for-transplantation *NPM1*m adult AML pts in molecular relapse/progression according to ELN2022. Inclusion criteria comprise: 1) morphological complete remission (CR) after ≥2 cycles (C) of IC; 2) centralized MRDpositivity defined as *NPM1*m copies/*ABL*1x100 ≥0.01%, assessed with qRT-PCR. Pts receive VEN400 mg days 1-28 plus AZA75 mg/m$^2$ days 1-7 (max 6 cycles). MRDnegativity (neg) is defined as*NPM1*m <0.01% *(Balsat JCO 2016)*; molecular reduction (MR) as ≥1log$_{10}$ reduction of *NPM1*m from pre-treatment levels *(Jimenez-Chillon Blood Adv 2024)*; overall response rate (ORR) as MR+MRDneg. HSCT is recommended anytime at MRDneg or after C3 according to investigator. Sample size is of 35 pts.

**Copyright Information:** (Online) ISSN: 2572-9241
©2026 The Author(s). HemaSphere is published by John Wiley & Sons Ltd on behalf of European Hematology Association. This is an open access Abstract Book distributed under the Attribution-NonCommercial-NoDerivs (CC BY-NC-ND), which allows third parties to download the articles and share them with others as long as they credit the author and the Abstract Book, but they cannot change the content in any way or use them commercially.
**Abstract Book Citations:** Authors, Title, HemaSphere, 2026;10:(S1):pages. The abstract book DOIs can be found on the "Author Information" page.
**Disclaimer:** Articles published in the journal HemaSphere exclusively reflect the opinions of the authors. The authors are responsible for all content in their abstracts including accuracy of the facts, statements, citing resources, etc.

**Results**

Overall, 47 pts have been screened, 35 treated and 34 analyzed (1 excluded for active disease). At diagnosis, AML characteristics showed concomitant *FLT3*-ITD mutation in 11 (32%) pts and *FLT3*-TKD in 3 (8.8%); ELN2022 risk was favorable, intermediate and adverse in 21 (64%), 11 (33%) and 1 (3%), respectively. Karyotype (K) analysis was normal in 25/29 (86%) pts, high-risk K were absent.
At enrolment, median age was 58 yrs [range (R), 26-72], 22 (65%) were male, all had ECOG 0. Median Hb values were 13.4 g/dL (R, 10.2-15.6), platelets 178 x$10^9$/L (R, 18-283) and leukocytes 4.8 x$10^9$/L (R, 2.9-12.6). All pts were in CR; 6 (18%) and 28 (82%) pts were in molecular progression and molecular relapse, respectively. Median *NPM1*m copies/*ABL*1x100 were 0.23% (R, 0.02-68.15), 2 pts had detectable *FLT3*-ITD.
Pts received a median of 3 cycles (R, 1-6) of VEN-AZA. Median VEN duration was 28 days during C1+C2, 18 days C3 and 14 days ≥C4; AZA was not reduced.
Concerning study endpoints, 33 (97%) pts maintained CR on study. Molecular ORR was 85% and MRDneg was achieved in 27 (79%) pts. After C1, 19 (61%) achieved a MR: median cycles to MRDneg were 2 (R, 1-3) and median time to MRDneg was 2.10 mos (R, 0.99-6.70). Reasons for AML-related end of treatment were relapse (1; 3%) and MRD progression (1; 3%) in the 2 *FLT3*-ITD pts at screening. So far 29/34 (85%) pts have been bridged to HSCT in CR or better. Median time to HSCT was 3.71 mo (R, 2.56 – 7.39). Of HSCT pts, 20 (70%) achieved MRDneg pre HSCT; 4 pts further negativized MRD post HSCT.
Median follow-up is 13 mos (R, 3-38), 1 relapsed pt has died; pts who received HSCT are in ongoing CR.
Overall, 21 adverse events (AEs) ≥G3 occurred: 17 neutropenia, 4 thrombocytopenia; 1 febrile neutropenia. Only one pt presented an AE that precluded HSCT; a G2 cardiac failure VEN-related in CR MRDneg.

**Summary/Conclusion**

This prospective MRD-driven study shows promising results for AZA-VEN in preventing disease relapse, deepening molecular responses and bridging effectively *NPM1*m AML pts in MF to alloSCT. The study has been conducted with the support of Abbvie SRL.

**Copyright Information:** (Online) ISSN: 2572-9241
©2026 The Author(s). HemaSphere is published by John Wiley & Sons Ltd on behalf of European Hematology Association. This is an open access Abstract Book distributed under the Attribution-NonCommercial-NoDerivs (CC BY-NC-ND), which allows third parties to download the articles and share them with others as long as they credit the author and the Abstract Book, but they cannot change the content in any way or use them commercially.
**Abstract Book Citations:** Authors, Title, HemaSphere, 2026;10:(S1):pages. The abstract book DOIs can be found on the "Author Information" page.
**Disclaimer:** Articles published in the journal HemaSphere exclusively reflect the opinions of the authors. The authors are responsible for all content in their abstracts including accuracy of the facts, statements, citing resources, etc.

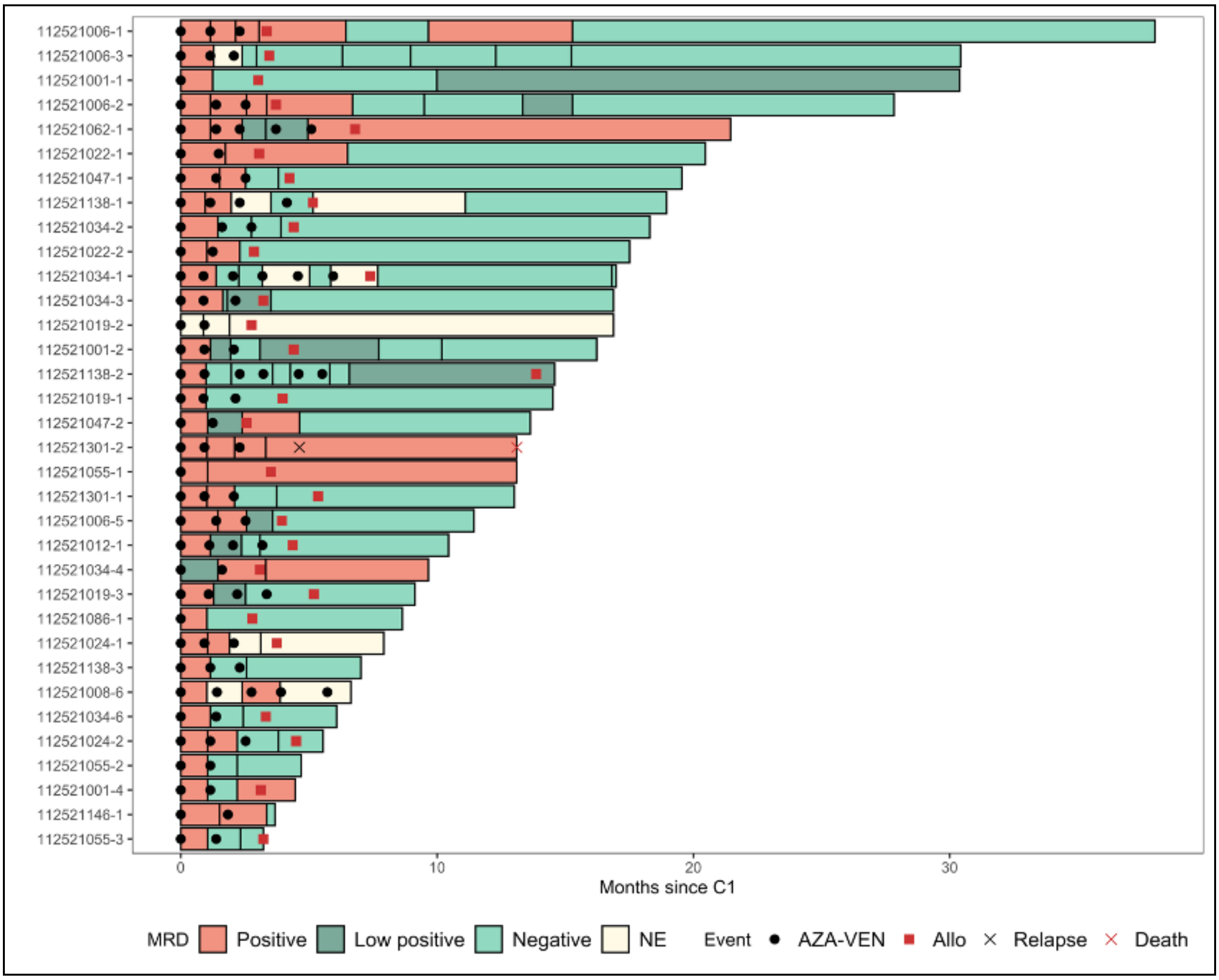

Figure: Swimmer plot of treated patients within the GIMEMA AML2521 trial

**Copyright Information:** (Online) ISSN: 2572-9241
©2026 The Author(s). HemaSphere is published by John Wiley & Sons Ltd on behalf of European Hematology Association. This is an open access Abstract Book distributed under the Attribution-NonCommercial-NoDerivs (CC BY-NC-ND), which allows third parties to download the articles and share them with others as long as they credit the author and the Abstract Book, but they cannot change the content in any way or use them commercially.
**Abstract Book Citations:** Authors, Title, HemaSphere, 2026;10:(S1):pages. The abstract book DOIs can be found on the "Author Information" page.
**Disclaimer:** Articles published in the journal HemaSphere exclusively reflect the opinions of the authors. The authors are responsible for all content in their abstracts including accuracy of the facts, statements, citing resources, etc.

### (S129) PROSPECTIVE NAMLG LD-VENEX TRIAL: AZACITIDINE IN COMBINATION WITH REDUCED DURATION OF VENETOCLAX IN PATIENTS WITH DE NOVO, SECONDARY, AND RELAPSED/REFRACTORY ACUTE MYELOID LEUKEMIA

**Topic:** 04. Acute myeloid leukemia - Clinical

Anne Louise Tølbøll Sørensen*[1, 2], Christian Landbo[1], Laura Haugsted Jedrzejowska[1], Frank Eriksson[3], Marianne Severinsen[4], Anne Stidsholt Roug[5], Claus Marcher[6], Johanna Rimpilainen[7], Vladimir Lazarevic[8], Bertil Uggla[9], Timo Siitonen[10], Anne Sophie von Krogh[11], Anna Robelius[12], Mats Olsen[13], Krister Wennerberg[2], Heikki Kuusanmäki[14], Tom Erkers[15], Jorrit Enserink[16], Sören Lehmann[17], Bo Porse[18], Kirsten Grønbæk[1], Bjørn T. Gjertsen[19], Waleed Mohammed[20], Martin Jädersten[21], Mika Kontro[22], Kim Theilgaard-Mönch[18]

*[1]Copenhagen University Hospital - Rigshospitalet, Department of Haematology, Copenhagen, Denmark;[2]University of Copenhagen, Department of Clinical Medicine, Copenhagen, Denmark; [3]University of Copenhagen, Section of Biostatistics, Copenhagen, Denmark; [4]Aalborg University Hospital, Department of Haematology, Aalborg, Denmark;[5]Aarhus University Hospital, Aarhus, Denmark; [6]Odense University Hospital, Department of Haematology, Odense, Denmark;[7]Tampere University Hospital, Department of Haematology, Tampere, Finland; [8]Skåne Univeristy Hospital, Lund, Sweden; [9]Örebro University Hospital, Örebro, Sweden; [10]Oulu University Hospital, Oulu, Finland; [11]St Olav Trondheim University Hospital, Trondheim, Sweden; [12]Uppsala University Hospital, Uppsala, Sweden; [13]University Hospital of North-Norway, Tromsø, Norway; [14]Institute for Molecular Medicine Finland, University of Helsinki, Helsinki, Finland; [15]Karolinska Institutet, Stockholm, Sweden; [16]Institute for Cancer Research, Oslo University Hospital, Oslo, Norway; [17]Karolinska Institutet, Stockholm, Stockholm, Sweden; [18]University of Copenhagen, Biotech Research & Innovation Centre (BRIC), Copenhagen, Denmark; [19]K.G. Jebsen Centre for Myeloid Blood Cancer (CMYC), Haukeland University Hospital, Bergen, Norway; [20]Stavanger University Hospital, Stavanger, Norway; [21]Karolinska University Hospital, Stockholm, Sweden; [22]Helsinki University Hospital Comprehensive Cancer Center, Helsinki, Finland;*

### Background

Combination therapy with venetoclax (VEN) and azacytidine (AZA) has become the standard treatment for newly diagnosed AML patients (pts) ineligible for intensive chemotherapy (IC). However, VEN/AZA therapy is associated with considerable myelosuppression, often requiring extended cycle intervals and dose reductions.

### Aims

To assess the efficacy and safety of reduced duration of VEN in a prospective, multicenter, phase 2 trial conducted by the Nordic AML Group.

### Methods

The LD-VenEx trial (NCT05431257) enrolled pts with de novo (dnAML), secondary (sAML), and relapsed/refractory AML (R/R AML) ineligible for IC. Written informed consent was obtained from all participating pts. Pts received 28-days treatment cycles with AZA 75 mg/m$^2$ on days 1-7 and VEN 400 mg once daily on days 1-14 upfront, which was reduced to days 1-7 after blast clearance (<5%), with VEN dose adjustment in case of concomitant azole prophylaxis. Responses were assessed on C1D29, (C2D29), C3D29, and every three cycles thereafter using ELN 2022 criteria. The primary endpoint was overall response rate (ORR =CR/CRh/CRi/MLFS/PR) and composite complete remission (CRc=CR/CRh/CRi) during the first three therapy cycles. Key secondary endpoints included overall survival (OS) and duration of response (DOR) estimated using the Kaplan-Meier method.

**Copyright Information:** (Online) ISSN: 2572-9241
©2026 The Author(s). HemaSphere is published by John Wiley & Sons Ltd on behalf of European Hematology Association. This is an open access Abstract Book distributed under the Attribution-NonCommercial-NoDerivs (CC BY-NC-ND), which allows third parties to download the articles and share them with others as long as they credit the author and the Abstract Book, but they cannot change the content in any way or use them commercially.
**Abstract Book Citations:** Authors, Title, HemaSphere, 2026;10:(S1):pages. The abstract book DOIs can be found on the "Author Information" page.
**Disclaimer:** Articles published in the journal HemaSphere exclusively reflect the opinions of the authors. The authors are responsible for all content in their abstracts including accuracy of the facts, statements, citing resources, etc.

**Results**

As of 18/12/2025 169 pts were screened, 152 pts fulfilled eligibility criteria and were treated with at least one dose of VEN and included in the safety and full analysis set. Accrual is complete, data-cut off 31/01/2026. The population included 84 dnAML, 32 sAML, and 36 R/R AML pts, of which 70% (58/83), 81% (26/32), and 50% (18/36), respectively, were classified as adverse risk according to ELN2022 criteria, whereas 28% (16/57), 12% (3/26), and 11% (4/36), respectively, were classified as adverse risk with TP53 mutations according to mPRS criteria. The ORRs were 79% (66/84) for dnAML, 78% (25/32) for sAML, and 67% (24/36) for R/R AML. The CRc rates were 68% (57/84), 50% (16/32), and 61% (22/36) for dnAML, sAML, and R/R AML, respectively. With a median potential follow-up time of 21.3 months (mo), the median OS (mOS) for dnAML, sAML, and R/R AML was 16.1 mo (95%CI 11.5-NR), 10.3 mo (95%CI 6.0-16.5), and 14.6 mo (95%CI 8.5-NR), respectively (Figure 1). Notably, mOS for NPM1 mutated dnAML was not reached (95%CI 19.4-NR), and mOS for TP53 mutated dnAML was 9.2 mo (95%CI 6.4-NR). Median DOR (mDOR) for dnAML, sAML, and R/R AML was 12.5 mo (95%CI 10.1-NR), 9.6 mo (95%CI 4.7-NR), and 7.0 mo (95%CI 5.4-18.8), respectively.The most frequent treatment emergent adverse events (TEAE) grade ≥ 3 included neutropenia (32%, 49/152), febrile neutropenia (39%, 59/152), thrombocytopenia (21%, 32/152), sepsis (15%, 23/152), and lung infection (14%, 21/152). The 30- and 60-day mortality rates were 4% (6/152) and 14% (22/152), respectively.

**Summary/Conclusion**

Efficacy of VEN/AZA treatment with shortened VEN duration appeared comparable to full-dose VEN/AZA. CRc rates and mOS for dnAML (68%, 16.1 mo) corresponded to Viale-A results (66.8%, 14.7 mo). CRc rates and mOS for sAML (50%, 10.3 mo) and R/R AML (61%, 14.6 mo) exceeded full-dose VEN/AZA in the prospective VenEx trial (sAML+R/R AML 39%, 8.4 mo) as well as several other trials and real-world evidence. TEAE grade ≥ 3 were slightly reduced compared to Viale-A results, particularly thrombocytopenia (21% vs 45%). Our data support reduced duration VEN as a feasible approach at different stages of AML.

**Copyright Information:** (Online) ISSN: 2572-9241
©2026 The Author(s). HemaSphere is published by John Wiley & Sons Ltd on behalf of European Hematology Association. This is an open access Abstract Book distributed under the Attribution-NonCommercial-NoDerivs (CC BY-NC-ND), which allows third parties to download the articles and share them with others as long as they credit the author and the Abstract Book, but they cannot change the content in any way or use them commercially.
**Abstract Book Citations:** Authors, Title, HemaSphere, 2026;10:(S1):pages. The abstract book DOIs can be found on the "Author Information" page.
**Disclaimer:** Articles published in the journal HemaSphere exclusively reflect the opinions of the authors. The authors are responsible for all content in their abstracts including accuracy of the facts, statements, citing resources, etc.

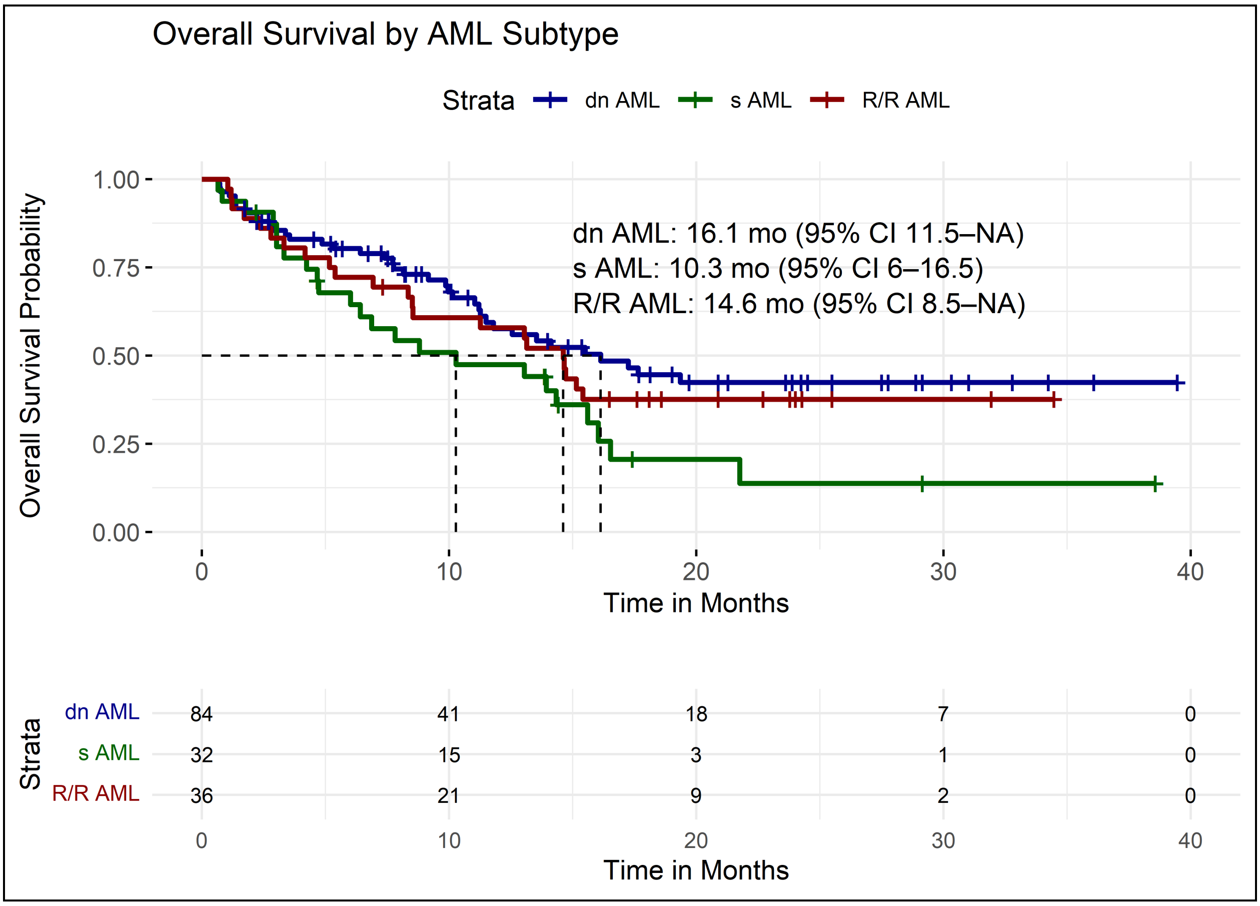

Figure 1. Overall Survival of patients stratified according to AML subtype (dnAML, sAML, R/R AML).

**Copyright Information:** (Online) ISSN: 2572-9241
©2026 The Author(s). HemaSphere is published by John Wiley & Sons Ltd on behalf of European Hematology Association. This is an open access Abstract Book distributed under the Attribution-NonCommercial-NoDerivs (CC BY-NC-ND), which allows third parties to download the articles and share them with others as long as they credit the author and the Abstract Book, but they cannot change the content in any way or use them commercially.
**Abstract Book Citations:** Authors, Title, HemaSphere, 2026;10:(S1):pages. The abstract book DOIs can be found on the "Author Information" page.
**Disclaimer:** Articles published in the journal HemaSphere exclusively reflect the opinions of the authors. The authors are responsible for all content in their abstracts including accuracy of the facts, statements, citing resources, etc.

### (S130) ZIFTOMENIB COMBINED WITH INTENSIVE INDUCTION (7+3) FOR NEWLY DIAGNOSED NPM1-M OR KMT2A-R ACUTE MYELOID LEUKEMIA (AML): LONG-TERM RESULTS FROM THE KOMET-007 TRIAL

**Topic:** 04. Acute myeloid leukemia - Clinical

Amer M. Zeidan*[1], Eunice Wang[2], Harry Erba[3], Stephen Strickland[4], Ghayas Issa[5], Suresh Balasubramanian[6], Gail J.Roboz[7], Christine McMahon[8], Marcello Rotta[9], Yazan F. Madanat[10], Keith Pratz[11], James S. Blachly[12], Tara L. Lin[13], Gary Schiller[14], Talha Badar[15], Ivana Gojo[16], Jessica K. Altman[17], James Mangan[18], Mark Juckett[19], Kiran Naqvi[20], Anjali Advani[21], Leonard C. Alsfeld[22], Jorge Cortes[23], Mohamad Khawandanah[24], Kalyan Nadiminti[25], Hongling Zhang[26], Tianle Chen[26], Valerie Santos[26], Marcie Riches[26], Daniel Corum[26], Mollie Leoni[26], Amir Fathi[27]

*[1]Yale University and Yale Comprehensive Cancer Center, New Haven, United States of America;[2]Roswell Park Comprehensive Cancer Center, Buffalo, United States of America; [3]Duke Cancer Institute, Durham, United States of America;[4]SCRI at TriStar Centennial, Nashville, United States of America; [5]Department of Leukemia, The University of Texas MD Anderson Cancer Center, Houston, United States of America; [6]Karmanos Cancer Institute, Wayne State University, Detroit, United States of America;[7]Weill Cornell Medicine and The New York Presbyterian Hospital, New York, United States of America; [8]Anschutz Medical Campus, Division of Hematology, University of Colorado School of Medicine, Aurora, United States of America; [9]Colorado Blood Cancer Center, Denver, United States of America; [10]The University of Texas Southwestern Medical Center, Dallas, United States of America; [11]Abramson Cancer Center, University of Pennsylvania, Philadelphia, United States of America; [12]The Ohio State University Comprehensive Cancer Center, Ohio, United States of America; [13]University of Kansas Medical Center, Kansas City, United States of America; [14]David Geffen School of Medicine at UCLA, Los Angeles, United States of America;[15]Mayo Clinic, Jacksonville, United States of America; [16]Sidney Kimmel Comprehensive Cancer Center, Johns Hopkins University School of Medicine, Baltimore, United States of America; [17]Robert H. Lurie Comprehensive Cancer Center, Northwestern University, Chicago, United States of America; [18]Moores Cancer Center, University of California, San Diego, United States of America; [19]Department of Hematology, University of Minnesota, Minneapolis, United States of America;[20]Chao Family Comprehensive Cancer Center, University of California Irvine Health, Orange, United States of America; [21]Cleveland Clinic, Cleveland, United States of America; [22]Ochsner MD Anderson Cancer Center, New Orleans, United States of America;[23]Georgia Cancer Center, Augusta, United States of America; [24]University of Oklahoma Health Sciences Center, Stephenson Cancer Center, Oklahoma City, United States of America; [25]Carbone Cancer Center, University of Wisconsin-Madison, Madison, United States of America; [26]Kura Oncology, Inc., San Diego, United States of America;[27]Massachusetts General Hospital, Harvard Medical School, Boston, United States of America;*

### Background

*NPM1*-m and *KMT2A*-r drive leukemogenesis in ~35–40% of AML. Ziftomenib – a potent, selective oral menin inhibitor – is U.S. FDA-approved as monotherapy for adults with relapsed/refractory *NPM1*-m AML. It has also shown clinically meaningful activity and tolerability in combination for newly diagnosed (ND) *NPM1*-m or *KMT2A*-r AML. KOMET-007 (NCT05735184) is an ongoing, multi-cohort, open-label, Ph1a/b study of ziftomenib combined with standard chemotherapies in *NPM1*-m or *KMT2A*-r AML.

### Aims

Here we report updated safety and clinical activity in patients (pts) with ND AML treated with ziftomenib 600 mg once daily (QD) in combination with standard doses of cytarabine/daunorubicin (7+3) from KOMET-007.

**Copyright Information:** (Online) ISSN: 2572-9241
©2026 The Author(s). HemaSphere is published by John Wiley & Sons Ltd on behalf of European Hematology Association. This is an open access Abstract Book distributed under the Attribution-NonCommercial-NoDerivs (CC BY-NC-ND), which allows third parties to download the articles and share them with others as long as they credit the author and the Abstract Book, but they cannot change the content in any way or use them commercially.
**Abstract Book Citations:** Authors, Title, HemaSphere, 2026;10:(S1):pages. The abstract book DOIs can be found on the “Author Information” page.
**Disclaimer:** Articles published in the journal HemaSphere exclusively reflect the opinions of the authors. The authors are responsible for all content in their abstracts including accuracy of the facts, statements, citing resources, etc.

### Methods

Adults (≥18y) with ND *NPM1*-m or *KMT2A*-r AML enrolled independently and received ziftomenib 600 mg QD starting on day 8 of induction with 7+3 and continued daily through cytarabine consolidation and post-consolidation maintenance. All pts provided written informed consent. Primary endpoints were adverse events (AEs) and complete remission (CR; ELN 2022). Key secondary endpoints included composite CR (CRc; defined as CR with full/partial/incomplete hematologic recovery), overall response rate (ORR), duration of response, and overall survival (OS).

### Results

As of 16 Jan 2026, 99 pts with ND AML (49 *NPM1*-m, 50 *KMT2A*-r) were treated with ziftomenib 600 mg plus 7+3. Median age was 53y (range 18–71), 57% were women, 69% were White, and 93% had ECOG PS 0–1. Median follow-up was 14.9 months for *NPM1*-m and 9.3 months for *KMT2A*-r. Most common (≥20%) grade (Gr)≥3 treatment-emergent AEs were similar between *NPM1*-m and *KMT2A*-r: febrile neutropenia (64%), thrombocytopenia (57%), anemia (36%), neutropenia (28%), and leukopenia (27%). 53 pts (54%) had Gr≥3 investigator-assessed ziftomenib-related AEs, most commonly (≥10%) thrombocytopenia (21%), anemia (15%), neutropenia (13%), febrile neutropenia (13%), and pruritus (10%). Three pts (3%) had Gr3 differentiation syndrome (3 *KMT2A*-r, ziftomenib-related; no Gr4), which successfully resolved with protocol-specified mitigation; 4 (4%) had Gr3 investigator-assessed QTc prolongation (1 *NPM1*-m; 3 *KMT2A*-r with 1 ziftomenib-related; no Gr4) that successfully resolved. Among all 99 response-evaluable pts, CRc rates were 96% (47/49) for *NPM1*-m and 90% (45/50) for *KMT2A*-r, with local measurable residual disease (MRD) negativity rates of 83% (39/47) and 82% (32/39), respectively (**Table**). For *NPM1*-m, 92% (36/39) reached MRD negativity by 20 wks. At data cutoff, median CRc duration was not yet reached for *NPM1*-m and 11.2 months for *KMT2A*-r; median OS was not yet reached, with OS rates of 94% for *NPM1*-m and 70% for *KMT2A*-r at 1y. During continuous ziftomenib administration in CRc responders, median times to neutrophil (≥1×$10^9$/L) and platelet (≥100×$10^9$/L) recovery were 29d and 28d for both groups, respectively. Currently, 94% of *NPM1*-m and 62% of *KMT2A*-r pts remain on study. Updated data, including central MRD, will be presented.

### Summary/Conclusion

In the ongoing KOMET-007 study, ziftomenib 600 mg QD combined with 7+3 was well tolerated with deep and durable responses in pts with ND *NPM1*-m and *KMT2A*-r AML: CRc rates were 96% and 90%, with 83% and 82% MRD negativity, respectively. Median CRc duration was not reached for *NPM1*-m. Low rates of additive myelosuppression were observed with this combination. These data further support the ongoing Ph3 registrational trial of ziftomenib in combination with intensive chemotherapy (KOMET-017; NCT07007312).

**Copyright Information:** (Online) ISSN: 2572-9241
©2026 The Author(s). HemaSphere is published by John Wiley & Sons Ltd on behalf of European Hematology Association. This is an open access Abstract Book distributed under the Attribution-NonCommercial-NoDerivs (CC BY-NC-ND), which allows third parties to download the articles and share them with others as long as they credit the author and the Abstract Book, but they cannot change the content in any way or use them commercially.
**Abstract Book Citations:** Authors, Title, HemaSphere, 2026;10:(S1):pages. The abstract book DOIs can be found on the “Author Information” page.
**Disclaimer:** Articles published in the journal HemaSphere exclusively reflect the opinions of the authors. The authors are responsible for all content in their abstracts including accuracy of the facts, statements, citing resources, etc.

**Table**

| n (%) | Newly Diagnosed AML: Ziftomenib 600 mg QD plus 7+3 | | |
|---|---|---|---|
| | *NPM1*-m N=49 | *KMT2A*-r N=50 | All Patients N=99 |
| **Median follow-up, months** | 14.9 | 9.3 | 12.8 |
| **CRc** | 47 (96) | 45 (90) | 92 (93) |
| **ORR** | 48 (98) | 46 (92) | 94 (95) |
| CR | 46 (94) | 41 (82) | 87 (88) |
| CRh | 1 (2) | 1 (2) | 2 (2) |
| CRi | 0 | 3 (6) | 3 (3) |
| MLFS | 1 (2) | 1 (2) | 2 (2) |
| PR | 0 | 0 | 0 |
| **NR** | 1 (2) | 3 (6) | 4 (4) |
| **NE** | 0 | 1 (2) | 1 (1) |
| **CR MRD negativity, n/m[a] (%)** | 38/46 (83) | 30/35 (86) | 68/81 (84) |
| **CRc MRD negativity, n/m[a] (%)** | 39/47 (83) | 32/39 (82) | 71/86 (83) |
| Median time to CR MRD negativity, weeks (range) | 6.5 (2.0–43.1) | 4.0 (2.0–12.0) | 4.9 (2.0–43.1) |
| Median time to CRc MRD negativity, weeks (range) | 6.4 (2.0–43.1) | 4.0 (2.0–12.0) | 4.7 (2.0–43.1) |

All 99 patients were response-evaluable (≥1 response assessment or had died).

[a]Number of evaluable responders tested for MRD per local assay (NGS, RT-qPCR, FISH, flow cytometry).

CRc, composite complete remission (defined as CR / CRh / CRi, complete remission will full / partial / incomplete hematologic recovery); MLFS, morphologic leukemia-free state; NE, not evaluable or not done; NR, non-response; PR, partial remission.

**Copyright Information:** (Online) ISSN: 2572-9241
©2026 The Author(s). HemaSphere is published by John Wiley & Sons Ltd on behalf of European Hematology Association. This is an open access Abstract Book distributed under the Attribution-NonCommercial-NoDerivs (CC BY-NC-ND), which allows third parties to download the articles and share them with others as long as they credit the author and the Abstract Book, but they cannot change the content in any way or use them commercially.
**Abstract Book Citations:** Authors, Title, HemaSphere, 2026;10:(S1):pages. The abstract book DOIs can be found on the “Author Information” page.
**Disclaimer:** Articles published in the journal HemaSphere exclusively reflect the opinions of the authors. The authors are responsible for all content in their abstracts including accuracy of the facts, statements, citing resources, etc.

### (S131) A PHASE II RANDOMIZED STUDY OF AK117 VERSUS PLACEBO IN COMBINATION WITH VENETOCLAX AND AZACITIDINE IN PREVIOUSLY UNTREATED ACUTE MYELOID LEUKEMIA PATIENTS INELIGIBLE FOR INTENSIVE CHEMOTHERAPY

**Topic:** 04. Acute myeloid leukemia - Clinical

Jie Jin[1], Lina Zhang[2], Qike Zhang[3], Huafeng Wang*[4], Lie Lin[5], Hao Jiang[6], Shaowei Qiu[7], Liangming MA[8], Zhichao Chen[9], Guolin Yuan[10], Zhenfang Liu[11], Jianmin Luo[12], Fei Li[13], Xin Zhou[14], Yajing Xu[15], Hua Xue[16], Jianfei Fu[17], Yi Wang[18], Xiaoyu Zhu[19], Yang Liang[20], Jishi Wang[21], Jiajia Pan[4], Guangji Zhang[7], Wenting Li[22], Baiyong Li[22], Michelle Xia[22], Jianxiang Wang[7]

*[1]The First Affiliated Hospital, Zhejiang University School of Medicine, Hangzhou, China;[2]Henan Cancer Hospital, Zhengzhou, China; [3]Gansu Provincial Hospital, Lanzhou, China; [4]The First Affiliated Hospital,Zhejiang University School of Medicine, Hangzhou, China; [5]Hainan Affiliated Hospital of Hainan Medical University, Haikou, China;[6]Peking University People's Hospital, Beijing, China; [7]Institute of Hematology & Blood Diseases Hospital, Chinese Academy of Medical Sciences & Peking Union Medical College, Tianjin, China; [8]Shanxi Bethune Hospital, Taiyuan, China; [9]Union Hospital Tongji Medical College Huazhong University of Science and Technology, Wuhan, China; [10]Xiangyang Central Hospital, Xiangyang, China; [11]The First Affiliated Hospital of Guangxi Medical University, Nanning, China; [12]The Second Hospital of Hebei Medical University, Shijiazhuang, China; [13]The First Affiliated Hospital of Nanchang University, Nanchang, China;[14]Wuxi People's Hospital, Wuxi, China; [15]Xiangya Hospital Central South University, Changsha, China; [16]Affiliated Hospital of Hebei University, Baoding, China; [17]Tongji Hospital Affiliated To Tongji University, Shanghai, China; [18]Shaanxi Provincial People's Hospital, Xi'an, China; [19]Anhui Provincial Hospital, Hefei, China; [20]Sun Yat-sen University Cancer Center, Guangzhou, China;[21]The Affiliated Hospital of Guizhou Medical University, Guiyang, China; [22]Akeso Biopharma,Inc., Zhongshan, China;*

### Background

Despite the advent of venetoclax (VEN)-based regimens, outcomes remain suboptimal for patients (pts) with previously untreated acute myeloid leukemia (AML) who are ineligible for intensive chemotherapy. AK117 is a humanized IgG4 monoclonal anti-CD47 antibody that blocks the “don’t eat me” signal on leukemia cells and enhances macrophage-mediated phagocytosis. Preclinical studies demonstrated synergistic anti-leukemic activity when CD47 blockade was combined with azacitidine (AZA) and VEN. We conducted a phase II study to evaluate the efficacy and safety of adding AK117 to the standard AZA+VEN backbone.

### Aims

This randomized, double-blind, placebo-controlled, multicenter phase II study (NCT06387420) aimed to compare the efficacy and safety of AK117 plus AZA and VEN vs placebo (PBO) plus AZA and VEN in previously untreated AML pts ineligible for intensive chemotherapy.

### Methods

Adults with previously untreated AML who were ineligible for intensive chemotherapy due to age ≥75 yrs or documented comorbidities were enrolled. Pts were randomized 1:1 to receive AK117 (20 mg/kg IV on day 1 every 2-week cycle) or matching PBO, each combined with AZA (75 mg/m² SC on days 1–7) and VEN (100 mg day 1, 200 mg day 2, 400 mg daily thereafter) in 28-day cycles. Primary endpoints were safety and composite complete remission (CRc; CR+CRh+CRi per ELN 2022 criteria). Secondary endpoints included overall response rate (ORR), MRD-negative CRc, duration of CRc, and overall survival (OS).

**Copyright Information:** (Online) ISSN: 2572-9241
©2026 The Author(s). HemaSphere is published by John Wiley & Sons Ltd on behalf of European Hematology Association. This is an open access Abstract Book distributed under the Attribution-NonCommercial-NoDerivs (CC BY-NC-ND), which allows third parties to download the articles and share them with others as long as they credit the author and the Abstract Book, but they cannot change the content in any way or use them commercially.
**Abstract Book Citations:** Authors, Title, HemaSphere, 2026;10:(S1):pages. The abstract book DOIs can be found on the “Author Information” page.
**Disclaimer:** Articles published in the journal HemaSphere exclusively reflect the opinions of the authors. The authors are responsible for all content in their abstracts including accuracy of the facts, statements, citing resources, etc.

**Results**

As of November 2025, 60 pts were randomized (AK117, n=30; PBO, n=30). Baseline demographics and disease characteristics, including age, sex, ECOG performance status, ELN 2022 risk classification, and ELN 2024 less-intensive genetic risk classification, were balanced. CRc rates were 56.7% with AK117 vs 53.3% with PBO; ORR was 80.0% vs 66.7%. A higher proportion of responders achieved MRD-negative CRc with AK117 (46.7% vs 36.7%). With a median follow-up of 8.84 months, median OS was not reached with AK117 and was 8.3 months with PBO; 6- and 9-month OS rates were 83.3% vs 73.2% and 78.7% vs 43.1%, respectively. Median duration of CRc was 10.4 vs 6.5 months. The incidence of overall treatment-emergent adverse events (TEAEs) and serious adverse events was comparable between treatment arms, and all pts experienced ≥1 TEAE. The most common TEAEs were generally consistent with those expected in the context of AML and AZA+VEN therapy and included decreased white blood cell count (83.3% vs 83.3%), neutropenia (80.0% vs 76.7%), thrombocytopenia (86.7% vs 66.7%), and hypokalemia (50.0% vs 70.0%) in the AK117 vs PBO arm, respectively. Grade ≥3 TEAEs occurred in 96.7% vs 93.3%. Permanent treatment discontinuation due to TEAEs occurred in 3 pts in the AK117 arm and 2 pts in the PBO arm. Fatal TEAEs were less frequent in the AK117 arm (16.7% vs 30.0%). Anemia, an adverse event of special interest for CD47-targeting agents, occurred in 46.7% and 50.0% of pts, with Grade ≥3 anemia reported in 43.3% of pts in both arms. Overall, no new safety signals were observed with AK117.

**Summary/Conclusion**

AK117 combined with AZA and VEN demonstrated encouraging clinical activity with a manageable safety profile in previously untreated AML pts ineligible for intensive chemotherapy, supporting further clinical development.

**Copyright Information:** (Online) ISSN: 2572-9241
©2026 The Author(s). HemaSphere is published by John Wiley & Sons Ltd on behalf of European Hematology Association. This is an open access Abstract Book distributed under the Attribution-NonCommercial-NoDerivs (CC BY-NC-ND), which allows third parties to download the articles and share them with others as long as they credit the author and the Abstract Book, but they cannot change the content in any way or use them commercially.
**Abstract Book Citations:** Authors, Title, HemaSphere, 2026;10:(S1):pages. The abstract book DOIs can be found on the "Author Information" page.
**Disclaimer:** Articles published in the journal HemaSphere exclusively reflect the opinions of the authors. The authors are responsible for all content in their abstracts including accuracy of the facts, statements, citing resources, etc.

### (S132) HEALTH-RELATED QUALITY OF LIFE OF PATIENTS WITH HIGH-RISK ACUTE PROMYELOCYTIC LEUKEMIA TREATED WITH ARSENIC TRIOXIDE. RESULTS FROM THE INTERNATIONAL RANDOMIZED PHASE III APOLLO TRIAL

**Topic:** 04. Acute myeloid leukemia - Clinical

Fabio Efficace*[1], Maria Teresa Voso[2], Pau Montesinos[3], Pierre Fenaux[4, 5], Emanuele Ammatuna[6], Céline Berthon[7], Karsten Spiekermann[8], Pierre Peterlin[9], Alwin Johannes Krämer[10], Lorenzo Santilli[1], Amelie Anota[11], Francesco Sparano[1], Jamilé Frayfer[12], Fabio Ciceri[13], Monica Bocchia[14], Erika Borlenghi[15], Jan-Henrik Mikesch[16], Ulf Schnetzke[17], Julia Pross[18], Hans Juergen Dr. Salwender[19], Stefan Wickenhauser[20], Francesco Albano[21], Mario Luppi[22], Lidwine Tick[23], Otto Visser[24], Paola Fazi[1], Lionel Ades[4, 5], Uwe Platzbecker[25]

*[1]Italian Group for Adult Hematologic Diseases (GIMEMA), Data Center and Health Outcomes Research Unit, Rome, Italy, Rome, Italy; [2]Fondazione PTV Policlinico Tor Vergata, U.O.S.D. Diagnostica Avanzata Oncoematologica, Rome, Italy;[3]Servicio de Hematologıa, Hospital Universitari i Politècnic La Fe, Valencia, Spain; [4]Hôpital Saint Louis, Service d'Hématologie Séniors, Assistance Publique hôpitaux de Paris, Paris Saint-Louis Leukemia Institute, Paris, France; [5]Université Paris Cité, Paris, France; [6]Afd. Hematologie, University Medical Center Groningen, Groningen, The Netherlands;[7]Le Centre Hospitalier Universitaire de Lille, Service des Maladies du sang, Lille, Italy; [8]University Hospital Grosshadern - Ludwig-Maximilians-University, Department of Medicine III, München, Germany; [9]Le Centre Hospitalier Universitaire de Nantes, Service d'Hématologie Clinique, Nantes, France; [10]Department of Internal Medicine 5, University Hospital Heidelberg, Heidelberg, Germany; [11]Biostatistics Unit, Direction of Clinical Research and Innovation, and French National Platform Quality of Life and Cancer, Centre Léon Bérard, Lyon, France; [12]Centre Hospitalier de Meaux, Service d'Hématologie, Meaux, France; [13]Ospedale San Raffaele S.r.l., U.O. Ematologia e Trapianto di Midollo, Milano, Italy; [14]Azienda Ospedaliera Senese, Ematologia, Siena, Italy; [15]ASST degli Spedali Civili di Brescia, Ematologia, Brescia, Italy; [16]Department of Medicine A, University Hospital Münster, Münster, Germany; [17]Department of Internal Medicine II, University Hospital Jena, Jena, Germany; [18]Department of Hematology, Oncology, Helios Klinikum Berlin-Buch, Berlin, Germany; [19]Asklepios Tumorzentrum Hamburg, AK Altona and AK St Georg, Hamburg, Germany;[20]Hematology Department, CHU de Nîmes, Nîmes, France; [21]Department of Precision and Regenerative Medicine and Ionian Area - DiMePRe-J - Azienda Ospedaliero Universitaria Consorziale Policlinico - Hematology and Stem Cell Transplantation Unit, University of Bari "Aldo Moro", Bari, Italy; [22]Hematology Unit, Azienda Ospedaliera Universitaria di Modena, Department of Medical and Surgical Sciences, University of Modena and Reggio Emilia, Modena, Italy; [23]Department of Hematology, Maxima Medical Center, Eindhoven, The Netherlands; [24]Department of Internal Medicine, Isala, Zwolle, The Netherlands;[25]University Hospital Carl Gustav Carus and TU Dresden - Faculty of Medicine, Medical Clinic 1, Dresden, Germany;*

### Background

The randomized phase III APOLLO trial compared the efficacy and toxicity of standard all-trans retinoic acid (ATRA) plus arsenic trioxide (ATO) versus ATRA plus chemotherapy (CHT) in newly diagnosed patients with high-risk acute promyelocytic leukemia (APL). The study demonstrated superior clinical efficacy in patients treated with ATRA–ATO (Platzbecker U, et al. *J Clin Oncol.* 2025;43:3160–3169).

### Aims

Given the importance of comprehensively assessing the benefit–risk profile of novel anticancer therapies, Patient-Reported Outcomes (PROs) were included as a secondary endpoint of the APOLLO trial. Here, we report the comparative PRO findings between treatment arms at the end of consolidation therapy.

**Copyright Information:** (Online) ISSN: 2572-9241
©2026 The Author(s). HemaSphere is published by John Wiley & Sons Ltd on behalf of European Hematology Association. This is an open access Abstract Book distributed under the Attribution-NonCommercial-NoDerivs (CC BY-NC-ND), which allows third parties to download the articles and share them with others as long as they credit the author and the Abstract Book, but they cannot change the content in any way or use them commercially.
**Abstract Book Citations:** Authors, Title, HemaSphere, 2026;10:(S1):pages. The abstract book DOIs can be found on the "Author Information" page.
**Disclaimer:** Articles published in the journal HemaSphere exclusively reflect the opinions of the authors. The authors are responsible for all content in their abstracts including accuracy of the facts, statements, citing resources, etc.

**Methods**

Health-related quality of life (HRQoL) and fatigue were assessed using the well-validated EORTC QLQ-C30 and FACIT-Fatigue questionnaires, respectively. These PROs were evaluated at the end of induction, after each consolidation cycle, and at month 30 after randomization. The following primary scales were prespecified in the protocol: physical functioning, cognitive functioning, fatigue, nausea and vomiting, constipation, and appetite loss. Mean HRQoL scores over time and differences between treatment arms were estimated using linear mixed-effects models.
For the present analysis, we report the estimated cross-sectional mean differences between treatment arms at the end of consolidation, corresponding to three consolidation cycles for patients treated with ATRA–CHT and four consolidation cycles for those treated with ATRA–ATO.
The clinical significance of EORTC QLQ-C30 and FACIT-Fatigue scales was assessed using previously established minimal important differences (MID) thresholds. All patients provided written informed consent.

**Results**

Overall, 133 eligible patients had received either ATRA-ATO (n = 68) or ATRA-CHT (n = 65). Of these, 119 successfully completed induction therapy (ATRA-ATO, n=62; ATRA-CHT, n=57) and were thus eligible for HRQoL analysis. Within this evaluable group, the median age was 44 years (IQR: 36-51), 47.9% were male. Overall compliance rates were: 80.7% and 72.2% after induction and after consolidation, respectively.
After induction, there were no statistically significant differences between treatment arms in any of the EORTC-QLQC30 scales.
At the end of consolidation, statistically and clinically meaningful differences favouring ATRA–ATO were observed in the following primary scales: physical functioning (Δ = 17.0; 99% CI, 4.9 to 29.1, P<0.001), fatigue (Δ = 17.6; 99% CI, 5.1 to 30.1, P<0.001), nausea and vomiting (Δ = 8.0; 99% CI, 0.7 to 15.3, P=0.005), and appetite loss (Δ = 14.5; 99% CI, 3.0 to 26.0, P=0.001). No significant differences were observed for cognitive functioning or constipation.
Results regarding fatigue differences favouring ATRA–ATO were confirmed by the FACIT-Fatigue questionnaire, with a mean difference of 6.6 points (99% CI, 1.7 to 11.5; P<0.001). This difference was clinically meaningful, exceeding twice the MID of 3 points.
Additional HRQoL differences favouring ATRA-ATO were also observed in the following secondary EORTC QLQ-C30 scales: social, role functioning and global health status/QoL.

**Summary/Conclusion**

The current findings further support the use of ATRA–ATO therapy in newly diagnosed higher-risk APL by showing improved HRQoL outcomes compared with standard ATRA–CHT at the end of consolidation. Longer-term HRQoL outcomes will be evaluated in planned longitudinal analyses

**Copyright Information:** (Online) ISSN: 2572-9241
©2026 The Author(s). HemaSphere is published by John Wiley & Sons Ltd on behalf of European Hematology Association. This is an open access Abstract Book distributed under the Attribution-NonCommercial-NoDerivs (CC BY-NC-ND), which allows third parties to download the articles and share them with others as long as they credit the author and the Abstract Book, but they cannot change the content in any way or use them commercially.
**Abstract Book Citations:** Authors, Title, HemaSphere, 2026;10:(S1):pages. The abstract book DOIs can be found on the "Author Information" page.
**Disclaimer:** Articles published in the journal HemaSphere exclusively reflect the opinions of the authors. The authors are responsible for all content in their abstracts including accuracy of the facts, statements, citing resources, etc.

### (S133) INTENSIVE CHEMOTHERAPY IN COMBINATION WITH QUIZARTINIB VERSUS MIDOSTAURIN FOR FLT3-ITD MUTATED ACUTE MYELOID LEUKEMIA: A MULTICENTER COHORT STUDY

**Topic:** 04. Acute myeloid leukemia - Clinical

Michelle Lee*[1, 2], Angela Tramontano[3], Chenyu Lin[4], Ellen Madarang[5], Joanna Cwykiel[6], Wyatt McCall[7], Stephanie Begley[8], Nayan Mainkar[9], Vincent Lok[10], Sarvarinder Gill[11], Jared Vega[12], Jeffrey Baron[12], Abdur Jabir[13], Katie Moffitt[14], Joshua Zeidner[14], Madelyn Burkart[13], Eunice Wang[12], James McCloskey[11], David Swoboda[10], Vanessa Kennedy[9], Brittany Ragon[8], Andrew Matthews[7], Alexander Perl[7], Yasmin Abaza[6], Justin Watts[5], Harry Erba[4], Andrew Brunner[1, 2], Amir Fathi[1, 2]

*[1]Massachusetts General Hospital, Department of Medical Oncology, Boston, United States of America;[2]Harvard Medical School, Boston, United States of America; [3]Dana Farber Cancer Institute, Division of Population Sciences, Boston, United States of America; [4]Duke Cancer Institute, Division of Hematologic Malignancies and Cellular Therapy, Durham, United States of America; [5]University of Miami Sylvester Comprehensive Cancer Center, Miami, United States of America;[6]Northwestern University, Robert H. Lurie Comprehensive Cancer Center, Chicago, United States of America; [7]University of Pennsylvania, Abramson Cancer Center, Philadelphia, United States of America; [8]Atrium Health Levine Cancer Institute, Charlotte, United States of America; [9]Stanford University, Department of Medicine, Stanford, United States of America; [10]University of South Florida Morsani School of Medicine, Tampa, United States of America; [11]Hackensack University Medical Center, Hackensack, United States of America; [12]Roswell Park Comprehensive Cancer Center, Buffalo, United States of America; [13]Atrium Health Wake Forest Baptist Medical Center, Winston-Salem, United States of America; [14]University of North Carolina, Lineberger Comprehensive Cancer Center, Chapel Hill, United States of America;*

### Background

For newly diagnosed (ND) *FLT3*-ITD mutant AML, midostaurin (M) or quizartinib (Q), in combination with intensive chemotherapy (IC), are established standards of care based on the CALGB 10603/RATIFY (NCT00651261) and QuANTUM-First (NCT02668653) trials, respectively. Nonetheless, retrospective and prospective comparisons of the 2 commercially available tyrosine kinase inhibitors (TKIs) are lacking to guide clinical practice.

### Aims

We conducted a retrospective, multicenter cohort study comparing treatment patterns and clinical outcomes of patients with ND *FLT3*-ITD mutant AML treated with either M or Q in the frontline with IC.

### Methods

Patients ≥18 years who received M+IC or Q+IC at one of 12 United States cancer centers were included. Patients were excluded if they did not harbor a *FLT3*-ITD mutation, received treatment on a clinical trial, or received fludarabine-based IC, CPX-351, or venetoclax combinations in the frontline. The primary outcome was 1-year overall survival (OS). Secondary analyses included 1-year event-free survival (EFS, time from treatment start to refractory disease, relapse after first remission [CR1], or death from any cause), induction response, rates of allogeneic stem cell transplantation (HCT) at CR1, rates of *FLT3* mutation clearance post-induction, and safety. Kaplan-Meier analyses were used to assess OS and EFS; log rank test and Cox proportional hazards models were used to assess differences in outcomes between groups.

**Copyright Information:** (Online) ISSN: 2572-9241
©2026 The Author(s). HemaSphere is published by John Wiley & Sons Ltd on behalf of European Hematology Association. This is an open access Abstract Book distributed under the Attribution-NonCommercial-NoDerivs (CC BY-NC-ND), which allows third parties to download the articles and share them with others as long as they credit the author and the Abstract Book, but they cannot change the content in any way or use them commercially.
**Abstract Book Citations:** Authors, Title, HemaSphere, 2026;10:(S1):pages. The abstract book DOIs can be found on the "Author Information" page.
**Disclaimer:** Articles published in the journal HemaSphere exclusively reflect the opinions of the authors. The authors are responsible for all content in their abstracts including accuracy of the facts, statements, citing resources, etc.

### Results

Between 05/2017-05/2025, 127 patients received IC+M and 88 received IC+Q. The median age of our cohort was 56 years (interquartile range [IQR]: 45, 65), 44.7% were male, and 73% were White. Most (94.9%) had de novo AML and intermediate-risk (88.8%) by EuropeanLeukemia Net classifications. The most common co-occurring mutations were *NPM1* (56.3%) and *DNMT3A* (40%); the combination of *FLT3*-ITD/*NPM1*/*DNMT3A* was more prevalent in M than Q (35.4% vs 19.3%,*p*=0.01). The median follow-up was 667 days for M and 324 days for Q. The 1-year OS was 78% for M and 93% for Q (hazard ratio [HR]=0.19, 95% confidence interval [CI]: 0.07-0.52). The 1-year EFS was 56% for M and 77% for Q (HR=0.44, 95% CI:0.25-0.77). Q increased rates of composite complete remission (CR+CRi+CRh) compared with M (85.2% vs 73.2%, *p*=0.03), but *FLT3* mutation clearance (sensitivity≥$10^{-4}$) post-induction was comparable (Q: 38.5% vs M: 40.3%, *p*=0.84). The median duration of inducition hospitalization was 32 (IQR: 28, 38) and 33 (IQR: 29, 39) days for M and Q, respectively. There were no significant differences in 30-day (M: 1.6% vs Q: 1.1%, *p*=0.79) or 60-day mortality (M: 3.9% vs Q: 2.3%, *p*=0.5). Rates of febrile neutropenia (M: 91.3% vs Q: 89.8%), culture positive infections (M: 43.3% vs Q: 38.6%), total parenteral nutrition use (M: 18.9% vs Q: 13.6%), or GCSF support (M:12.6% vs Q: 14.8%) during induction were comparable. Patients treated with M had higher intensive care unit utilization (15% vs 4.5%, *p*=0.02) and discharges to a rehabilitation center (7.1% vs 1.1%, *p*=0.04) after index hospitalization. M was more likely to be interrupted or delayed during induction than Q (25.2% vs 11.4%, *p*=0.01). On multivariable analyses, Q, *NPM1/DNMT3A/FLT3*-ITD co-mutations, and HCT at CR1 were associated with improved 1-year OS and EFS.

### Summary/Conclusion

In ND *FLT3*-ITD mutant AML, IC+Q was associated with improved outcomes over IC+M in our retrospective real-world analysis, the largest known to date.

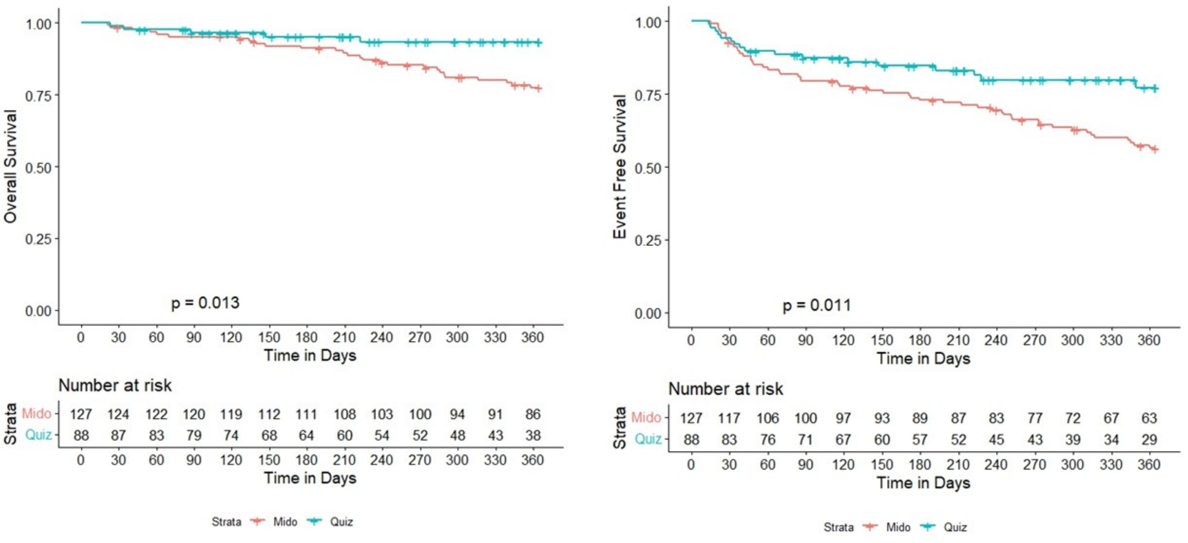

**Figure 1.** One-year overall survival (left) and event-free survival (right) for *FLT3*-ITD mutant AML patients who received frontline IC + midostaurin versus IC + quizartinib.

**Copyright Information:** (Online) ISSN: 2572-9241
©2026 The Author(s). HemaSphere is published by John Wiley & Sons Ltd on behalf of European Hematology Association. This is an open access Abstract Book distributed under the Attribution-NonCommercial-NoDerivs (CC BY-NC-ND), which allows third parties to download the articles and share them with others as long as they credit the author and the Abstract Book, but they cannot change the content in any way or use them commercially.
**Abstract Book Citations:** Authors, Title, HemaSphere, 2026;10:(S1):pages. The abstract book DOIs can be found on the “Author Information” page.
**Disclaimer:** Articles published in the journal HemaSphere exclusively reflect the opinions of the authors. The authors are responsible for all content in their abstracts including accuracy of the facts, statements, citing resources, etc.

### (S134) TUSCANY STUDY OF SAFETY AND EFFICACY OF TUSPETINIB PLUS STANDARD OF CARE VENETOCLAX AND AZACITIDINE IN STUDY PARTICIPANTS WITH NEWLY DIAGNOSED AML INELIGIBLE FOR INDUCTION CHEMOTHERAPY

**Topic:** 04. Acute myeloid leukemia - Clinical

Nikolai Podoltsev*[1], Gabriel Mannis[2], Justin Watts[3], Pankit Vachhani[4], Deepa Jeyakumar[5], Eric Tam[6], Uma Borate[7], Brian A. Jonas[8], Yuling Shi[9], Donna Haney[9], William Rice[9], Rafael Bejar[9], Naval Daver[10]

*[1]Yale University School of Medicine, New Haven, United States of America;[2]Blood Draw at Stanford Cancer Center, Palo Alto, United States of America; [3]University of Miami Miller School of Medicine, Miami, United States of America;[4]University of Alabama, Birmingham, United States of America; [5]University of California, Irvine, United States of America;[6]University of California, Los Angeles, United States of America; [7]Head And Neck Oncology In the James Cancer Hospital And Solove Research, Columbus, United States of America; [8]University of California, Sacramento, United States of America;[9]Aptose BioSciences, San Diego, United States of America; [10]MD Anderson Cancer Center, Leukemia, Houston, United States of America;*

### Background

Azacitidine (AZA) combined with venetoclax (VEN) improves response rates and overall survival in newly diagnosed AML patients. Yet, long-term outcomes remain poor in cases with adverse *FLT3-ITD*, *RAS*, or *TP53* mutations. As VEN resistance is associated with a dismal prognosis, adding a well-tolerated, broadly active agent to AZA/VEN is needed to enhance response rates, increase survival, and prevent resistance.
Tuspetinib (TUS) is a potent, once-daily oral kinase inhibitor targeting SYK, FLT3, JAK1/2, RSK1/2, and mutant KIT kinases that drive dysregulated proliferation in AML. Combination of TUS with AZA/VEN is supported by early phase safety and efficacy results in R/R AML, both as monotherapy and in combination with VEN.

### Aims

To assess the safety, tolerability, and pharmacokinetics (PK) of TUS, at four dose levels, in combination with AZA/VEN in newly diagnosed AML patients (pts) ineligible for intensive chemotherapy.

### Methods

In the TUSCANY study, TUS is administered to pts with previously untreated AML in combination with standard doses of AZA (75mg/m$^2$ daily x 7) and VEN (400mg daily x 21 or 28 days) at doses of 40-160mg daily x 28 every 28-day cycle. Safety, VEN and TUS PK, response, and measurable residual disease (MRD) data are collected.

**Copyright Information:** (Online) ISSN: 2572-9241
©2026 The Author(s). HemaSphere is published by John Wiley & Sons Ltd on behalf of European Hematology Association. This is an open access Abstract Book distributed under the Attribution-NonCommercial-NoDerivs (CC BY-NC-ND), which allows third parties to download the articles and share them with others as long as they credit the author and the Abstract Book, but they cannot change the content in any way or use them commercially.
**Abstract Book Citations:** Authors, Title, HemaSphere, 2026;10:(S1):pages. The abstract book DOIs can be found on the “Author Information” page.
**Disclaimer:** Articles published in the journal HemaSphere exclusively reflect the opinions of the authors. The authors are responsible for all content in their abstracts including accuracy of the facts, statements, citing resources, etc.

**Results**

In the prior R/R AML portion of this study, 93 pts received TUS monotherapy, and 79 pts received TUS/VEN as of February 09, 2026. No related non-hematologic SAEs, QTc prolongation, differentiation syndrome, deaths, or discontinuations were observed and no dose-limiting toxicities (DLTs) noted in the 40-160 mg dose levels.
To date, 23 pts with newly diagnosed AML, aged 63–81 years, received VEN/AZA+TUS at TUS doses of 40, 80, 120, or160 mg daily (Panel A), and enrollment is ongoing. Ten (43.5%) were male; 18 (78.2%) were FLT3-unmutated (FLT3-). The most common adverse events (>30%) were decreased platelet count (56.5%), decreased neutrophil count (47.8%), diarrhea (47.8%), decreased white blood cell count (39.1%), anemia (34.8%), nausea (34.8%), and constipation (30.4%). No DLTs were reported. PK levels of TUS (Panel B) and VEN were consistent with their use as monotherapy, indicating no meaningful drug–drug interactions.
Early results from pts treated with TUS/VEN/AZA who have become evaluable for response (n=17), show high remission rates across dose levels and molecular subgroups. Thus far, the CRc rate is 82.4%, with the best response of CR/CRh achieved in 76.5% and CR in 70.6%. CRc rates by dose are 75.0% at 40 mg, 80.0% at 80 mg, 100% at 120 mg and 80% at 160 mg. Responses were observed irrespective of FLT3 mutation status: FLT3+ pts (n=5) achieved CRc of 100%, FLT3– pts (n=12) achieved CRc of 75.0%. Among TP53 mutated/complex karyotype pts (n=4), CRc is 75.0%, compared CRc of 84.6% in pts without TP53 mutation (n=13). Both RAS mutated pts achieved a CR. MRD negativity defined as <0.1% leukemic blasts by central flow cytometry was observed in 11 pts (64.7%). Three pts received a stem cell transplant with two resuming TUS single-agent post-transplant.

**Summary/Conclusion**

Tuspetinib in combination with VEN/AZA has been well tolerated, demonstrating objective responses in a diverse group of AML pts including those with FLT3- and mutated TP53 or RAS. Early results from the VEN/AZA/TUS triplet in treatment-naïve AML indicate promising safety, tolerability, and high rates response with MRD-negative remissions. Updated results including additional pts will be presented at the meeting.

**Copyright Information:** (Online) ISSN: 2572-9241
©2026 The Author(s). HemaSphere is published by John Wiley & Sons Ltd on behalf of European Hematology Association. This is an open access Abstract Book distributed under the Attribution-NonCommercial-NoDerivs (CC BY-NC-ND), which allows third parties to download the articles and share them with others as long as they credit the author and the Abstract Book, but they cannot change the content in any way or use them commercially.
**Abstract Book Citations:** Authors, Title, HemaSphere, 2026;10:(S1):pages. The abstract book DOIs can be found on the “Author Information” page.
**Disclaimer:** Articles published in the journal HemaSphere exclusively reflect the opinions of the authors. The authors are responsible for all content in their abstracts including accuracy of the facts, statements, citing resources, etc.

### FIGURE:

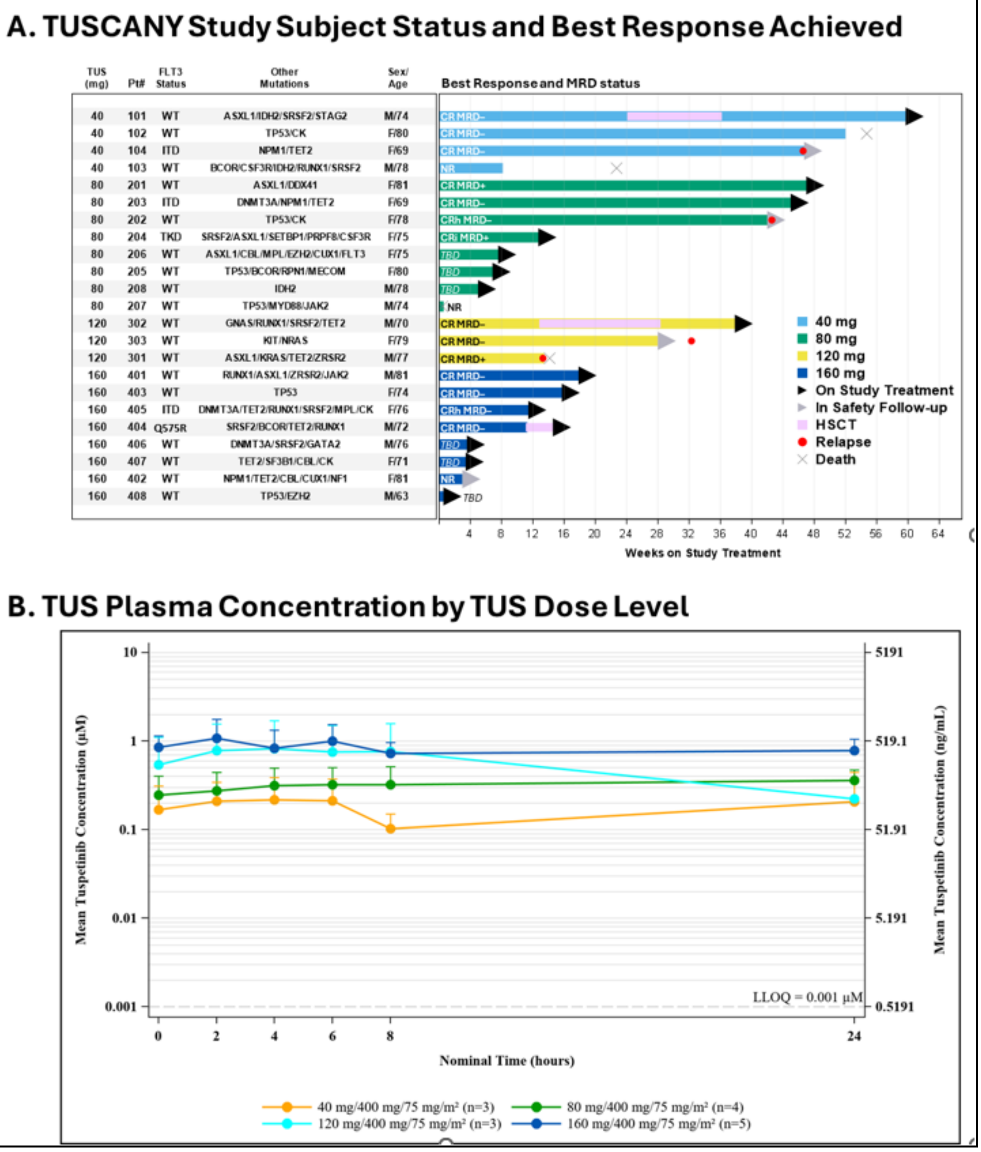

**Copyright Information:** (Online) ISSN: 2572-9241
©2026 The Author(s). HemaSphere is published by John Wiley & Sons Ltd on behalf of European Hematology Association. This is an open access Abstract Book distributed under the Attribution-NonCommercial-NoDerivs (CC BY-NC-ND), which allows third parties to download the articles and share them with others as long as they credit the author and the Abstract Book, but they cannot change the content in any way or use them commercially.
**Abstract Book Citations:** Authors, Title, HemaSphere, 2026;10:(S1):pages. The abstract book DOIs can be found on the "Author Information" page.
**Disclaimer:** Articles published in the journal HemaSphere exclusively reflect the opinions of the authors. The authors are responsible for all content in their abstracts including accuracy of the facts, statements, citing resources, etc.

### (S135) TISAGENLECLEUCEL IN PEDIATRIC AND YOUNG ADULT PATIENTS WITH HIGH-RISK B-CELL ACUTE LYMPHOBLASTIC LEUKEMIA AND MINIMAL RESIDUAL DISEASE AT THE END OF FRONTLINE CONSOLIDATION

**Topic:** 02. Acute lymphoblastic leukemia - Clinical

Shannon Maude*[1], Susana Rives[2, 3], Joerg Krueger[4], Kara Davis[5], Muna Qayed[6], Sara Ghorashian[7], Robrecht Pieters[8], Samuel John[9], André Baruchel[10], Marianne Ifversen[11], Pritesh Patel[12], Takayuki Iimori[13], Rakesh Awasthi[12], Brent Wood[14], Jennifer Willert[12], Stephan Grupp[1], Stephen Hunger[1], John Levine[15], Mignon Loh[16], Jochen Buechner[17]

*[1]Division of Oncology, Children's Hospital of Philadelphia, Department of Pediatrics, University of Pennsylvania Perelman School of Medicine, Philadelphia, PA, United States of America; [2]Pediatric Cancer Center Barcelona (PCCB), Hospital Sant Joan de Déu de Barcelona, Institut de Recerca Sant Joan de Déu, Leukemia; Pediatric Hematology Disorders, Developmental Tumors Biology Group, Barcelona, Spain; [3]Instituto de Salud Carlos III, Centro de Investigación Biomédica en Red De Enfermedades Raras (CIBERER), Madrid, Spain; [4]Division of Haematology/Oncology, Blood and Marrow Transplant/Cellular Therapy, The Hospital for Sick Children, Toronto, ON, Canada; [5]Department of Pediatrics, Hematology, Oncology, Stem Cell Transplant and Regenerative Medicine Stanford University; Center for Cancer Cell Therapy, Stanford Cancer Institute, Stanford University School of Medicine, Stanford, CA, United States of America; [6]Aflac Cancer and Blood Disorders Center, Children's Healthcare of Atlanta and Emory University, Atlanta, GA, United States of America; [7]University College London, London, United Kingdom; [8]Princess Máxima Center for Pediatric Oncology, Utrecht, The Netherlands; [9]Department of Pediatrics, UT Southwestern Medical Center, Dallas, TX, United States of America; [10]University Hospital Robert Debré (APHP) and Université Paris Cité, Paris, France; [11]Department of Pediatrics and Adolescent Medicine, Copenhagen University Hospital, Rigshospitalet, Copenhagen, Denmark; [12]Novartis Pharmaceuticals Corporation, East Hanover, NJ, United States of America; [13]Novartis Pharma K.K, Tokyo, Japan; [14]Department of Pathology and Laboratory Medicine, Children's Hospital Los Angeles, Keck School of Medicine, University of Southern California, Los Angeles, CA, United States of America; [15]BMT Clinical Research, The Tisch Cancer Institute, Icahn School of Medicine at Mount Sinai, New York, NY, United States of America; [16]Ben Towne Center for Childhood Cancer Research, Seattle Children's Research Institute; Department of Pediatrics, Seattle Children's Hospital, University of Washington, Seattle, WA, United States of America; [17]Department of Pediatric Hematology and Oncology, Oslo University Hospital, Oslo, Norway;*

### Background

In patients with B-cell acute lymphoblastic leukemia (B-ALL), persistent minimal residual disease (MRD) is associated with a high risk of relapse. In the Children's Oncology Group (COG) AALL0232 frontline trial, children and young adults with high-risk B-ALL and MRD ≥0.01% at the end of frontline consolidation (EOC) had a 5-year disease-free survival (DFS) of only 39% (Borowitz, *Blood*, 2015).

### Aims

The aim of this phase 2 trial, conducted in collaboration with the COG, was to evaluate the efficacy and safety of tisagenlecleucel, a CD19-directed chimeric antigen receptor (CAR) T cell therapy, in this very high-risk population.

**Copyright Information:** (Online) ISSN: 2572-9241
©2026 The Author(s). HemaSphere is published by John Wiley & Sons Ltd on behalf of European Hematology Association. This is an open access Abstract Book distributed under the Attribution-NonCommercial-NoDerivs (CC BY-NC-ND), which allows third parties to download the articles and share them with others as long as they credit the author and the Abstract Book, but they cannot change the content in any way or use them commercially.
**Abstract Book Citations:** Authors, Title, HemaSphere, 2026;10:(S1):pages. The abstract book DOIs can be found on the "Author Information" page.
**Disclaimer:** Articles published in the journal HemaSphere exclusively reflect the opinions of the authors. The authors are responsible for all content in their abstracts including accuracy of the facts, statements, citing resources, etc.

### Methods

CASSIOPEIA/COG AALL1721 (NCT03876769) is a global (34 US/Canada sites, 10 EU/UK sites) phase 2 study of tisagenlecleucel in patients (1-25 years old) with National Cancer Institute high-risk B-ALL and persistent MRD ≥0.01% by flow cytometry at EOC. Patients received standard lymphodepleting chemotherapy followed by a single tisagenlecleucel dose (0.2-5×$10^6$ CAR+ T cells/kg for patients ≤50 kg or 0.1-2.5×$10^8$ CAR+ T cells for patients >50 kg). Patients with B-cell recovery within 6 months and/or MRD ≥0.01% at any time could receive a reinfusion. Primary endpoints were 4-year overall survival (OS) and 5-year DFS, defined as time from first infusion to morphologic relapse, secondary malignancy, or death, without censoring for new anticancer therapy, including stem cell transplant (SCT). Secondary endpoints included DFS with censoring, MRD negative rate (MRD <0.01%) at 3 months, and safety.

### Results

The primary analysis included 121 infused patients; 1 patient was not infused due to an adverse event. Median age was 14 years (range: 1-24 years), 67% were male, 6% had trisomy 21. At data cut-off (August 20, 2025), median time from infusion to last follow-up was 38 months. Four-year OS was 85% (95% CI: 76-90%). DFS (95% CI) without censoring for new therapy was 71% (61-79%) at 3 years and 64% (53-73%) at 5 years. DFS (95% CI) with censoring was 69% (55-79%) at 3 years and 62% (46-74%) at 5 years. MRD negative rate post infusion was 95% at day 29 and 86% at 3 months. Relapse occurred in 31 patients, among which 7 relapsed after new therapy/SCT. A second infusion was given in 41 (34%) patients; 58 (48%) patients received new anticancer therapy/SCT without prior relapse. Median duration of B-cell aplasia was 5.6 months. Probability (95% CI) of ongoing remission and B-cell aplasia at 3, 6, and 12 months was 76% (67-83%), 47% (38-56%), and 31% (23-41%), respectively. Following first infusion, cytokine release syndrome (CRS) occurred in 37% (grade ≥3, 2%) and immune effector cell–associated neurotoxicity syndrome (ICANS) in 3% (grade ≥3, 1%). All 18 deaths occurred after relapse and/or new therapy/SCT.

### Summary/Conclusion

In a very high-risk population with persistent MRD in frontline therapy, who historically have low 5-year DFS, tisagenlecleucel produced durable remissions, with very low rates of severe CRS/ICANS. In this study, almost half of the patients received new anticancer therapy, including SCT, without experiencing prior relapse. Longer follow-up is needed to confirm 5-year DFS.

© 2026 American Society of Clinical Oncology, Inc. Reused with permission. This abstract was accepted and previously presented at the 2026 ASCO Annual Meeting. All rights reserved.

**Copyright Information:** (Online) ISSN: 2572-9241
©2026 The Author(s). HemaSphere is published by John Wiley & Sons Ltd on behalf of European Hematology Association. This is an open access Abstract Book distributed under the Attribution-NonCommercial-NoDerivs (CC BY-NC-ND), which allows third parties to download the articles and share them with others as long as they credit the author and the Abstract Book, but they cannot change the content in any way or use them commercially.
**Abstract Book Citations:** Authors, Title, HemaSphere, 2026;10:(S1):pages. The abstract book DOIs can be found on the "Author Information" page.
**Disclaimer:** Articles published in the journal HemaSphere exclusively reflect the opinions of the authors. The authors are responsible for all content in their abstracts including accuracy of the facts, statements, citing resources, etc.

### (S136) IMPACT OF TWO VERSUS THREE CHEMOTHERAPY CYCLES BEFORE HSCT IN MRD-NEGATIVE PEDIATRIC AML: A MULTICENTER RETROSPECTIVE STUDY

**Topic:** 04. Acute myeloid leukemia - Clinical

Lindsey Murphy*[1], Xinyi Du[2], Saro Armenian[1], Sydney Ariagno[3], Alysia Bosworth[2], Rohini Chakravarthy[4], Soohee Cho[5], Erin Doherty[6], Caitlin Elgarten[7], Vanessa Fabrizio[3], Ellen Fraint[8], Zachary Graff[9], Nishi Harwani[10], Jeffrey Huo[11, 12], Victoria Lehrmann[13], Lanie Lindenfeld[2], Amanda Lipsitt[14], Katharine Morrison[7], Megan Murphy[15], Yana Pikman[16], Troy Quigg[17], Akshay Sharma[10], Pramika Sriram[18], Heather Symons[15], Nicholas Tastet[12], Molly Thornock[2], Dominique Tlomak[9], Franziska Wachter[16], Sara Zarnegar-Lumley[19], F. Lennie Wong[2], Amanda Winters[3], Amy Keating[16]

*[1]City of Hope, Pediatrics, Duarte, United States of America;[2]City of Hope, Population Sciences, Duarte, United States of America; [3]University of Colorado, Pediatrics, Aurora, United States of America;[4]University of Chicago, Pediatrics, Chicago, United States of America; [5]University of Utah, Salt Lake City, United States of America;[6]Baylor College of Medicine, Texas Children's Hospital, Houston, United States of America; [7]Children's Hospital of Philadelphia, Division of Oncology, Philadelphia, United States of America; [8]Nemours Children's Hospital, Wilmington, United States of America;[9]Medical College of Wisconsin, Milwaukee, United States of America; [10]St. Jude Children's Research Hospital, Memphis, United States of America;[11]Wake Forest University School of Medicine, Charlotte, United States of America; [12]Atrium Health Levine Children's Hospital, Charlotte, United States of America; [13]University of Texas Health Science Center at San Antonio, San Antonio, United States of America; [14]Methodist Children's Hospital, San Antonio, United States of America; [15]Sidney Kimmel Comprehensive Cancer Center at Johns Hopkins, Baltimore, United States of America; [16]Harvard Medical School, Dana-Farber/Boston Children's Cancer and Blood Disorders Center, Boston, United States of America; [17]Corewell Health Helen DeVos Children's Hospital, Grand Rapids, United States of America; [18]University of Tennessee Health Science Center, College of Medicine, Memphis, United States of America; [19]Lurie Children's Hospital, Division of Hematology, Oncology, Neuro-Oncology, & Stem-Cell Transplant; Department of Pediatrics, Northwestern University Feinberg School of Medicine, Chicago, United States of America;*

### Background

Despite therapeutic advances, pediatric acute myeloid leukemia (AML) remains associated with substantial relapse risk and poor survival. Measurable residual disease (MRD) is a key prognostic marker and increasingly guides timing of allogeneic hematopoietic stem cell transplant (HSCT) in first complete remission (CR1). Historically, patients proceed to HSCT after three chemotherapy (chemo) cycles; however, the optimal number of cycles prior to HSCT after achieving MRD negativity and its impact on outcomes remain unclear.

### Aims

To evaluate the impact of 2 vs. 3 chemo cycles prior to HSCT and timing of MRD negativity on transplant outcomes in MRD-negative pediatric and young adult AML.

**Copyright Information:** (Online) ISSN: 2572-9241
©2026 The Author(s). HemaSphere is published by John Wiley & Sons Ltd on behalf of European Hematology Association. This is an open access Abstract Book distributed under the Attribution-NonCommercial-NoDerivs (CC BY-NC-ND), which allows third parties to download the articles and share them with others as long as they credit the author and the Abstract Book, but they cannot change the content in any way or use them commercially.
**Abstract Book Citations:** Authors, Title, HemaSphere, 2026;10:(S1):pages. The abstract book DOIs can be found on the "Author Information" page.
**Disclaimer:** Articles published in the journal HemaSphere exclusively reflect the opinions of the authors. The authors are responsible for all content in their abstracts including accuracy of the facts, statements, citing resources, etc.

### Methods

We conducted a multicenter retrospective study of AML patients diagnosed between 2012-2022 who achieved MRD negativity by the end of chemo cycle 2 and underwent HSCT in CR1. Patients were ≤25 years at time of HSCT. The effect of number of chemo cycles before HSCT (2 vs. 3) was examined in patients stratified by timing of MRD negativity (after 1 vs. 2 chemo cycles) and in the overall cohort. Outcomes included relapse, leukemia-free survival (LFS), overall survival (OS), and treatment-related mortality (TRM). Kaplan-Meier and regression models (Cox for OS/LFS, Fine-Gray for relapse, logistic for TRM) were used, adjusting for significant covariates including age, sex, race/ethnicity, performance status, cytogenetic risk, donor type, extramedullary disease (EMD) at diagnosis, year of HSCT, and cycles to achieve CR1. Unadjusted estimates are reported when no covariates remained significant after backward selection; otherwise, adjusted estimates are presented.

### Results

The cohort included 274 patients from 15 institutions; 95 (34.7%) underwent HSCT after 2 chemo cycles and 179 (65.3%) after 3 cycles. MRD negativity was achieved after cycle 1 in 149 patients and after cycle 2 in 125. Baseline characteristics were similar between groups except cycles to achieve CR1 and EMD.
Among patients achieving MRD negativity after cycle 1, OS, LFS, relapse, and TRM were not significantly different between 2 and 3 pre-HSCT chemo cycles (OS 77.9% vs. 65.2%, HR 1.59, 95% CI 0.82–3.12, p=0.17; LFS 74.3% vs. 63.4%, HR 1.57, 95% CI 0.84–2.93, p=0.16; relapse 14.5% vs. 26.7%, aHR 1.91, 95% CI 0.86–4.22, p=0.11; TRM aOR 7.25, 95% CI 0.79–66.85, p=0.08) (Figure 1a-c).
Similarly, among those MRD-negative after cycle 2, outcomes did not differ significantly (OS 74.7% vs. 62.1%, HR 1.57, 95% CI 0.75–3.30, p=0.23; LFS 59.7% vs. 54.5%, aHR 1.28, 95% CI 0.68–2.40, p=0.44; relapse 35.0% vs. 38.0%, aHR 1.21, 95% CI 0.63–2.35, p=0.57; TRM OR 0.43, 95% CI 0.03–7.06, p=0.55) (Figure 1d-f).
In the combined MRD-negative cohort, OS and LFS were higher and relapse incidence was lower among patients receiving 2 pre-HSCT chemo cycles compared with 3 cycles, though these differences were not statistically significant (OS 76.7% vs. 63.6%, HR 1.60, 95% CI 0.97–2.63, p=0.063; LFS 68.5% vs. 59.1%, HR 1.47, 95% CI 0.95–2.28, p=0.085; relapse 22.7% vs. 32.2%, aHR 1.58, 95% CI 0.96–2.59, p=0.072; TRM OR 2.18, 95% CI 0.45–10.46, p=0.33) (Figure 1g-i).

### Summary/Conclusion

In MRD-negative pediatric AML patients, receipt of a third chemo cycle prior to HSCT was not associated with improved OS, LFS, or relapse compared to proceeding after two cycles. Findings were consistent regardless of timing of achieving MRD negativity. These results challenge the paradigm that additional chemotherapy beyond MRD clearance improves HSCT outcomes for AML in CR1.

**Copyright Information:** (Online) ISSN: 2572-9241
©2026 The Author(s). HemaSphere is published by John Wiley & Sons Ltd on behalf of European Hematology Association. This is an open access Abstract Book distributed under the Attribution-NonCommercial-NoDerivs (CC BY-NC-ND), which allows third parties to download the articles and share them with others as long as they credit the author and the Abstract Book, but they cannot change the content in any way or use them commercially.
**Abstract Book Citations:** Authors, Title, HemaSphere, 2026;10:(S1):pages. The abstract book DOIs can be found on the “Author Information” page.
**Disclaimer:** Articles published in the journal HemaSphere exclusively reflect the opinions of the authors. The authors are responsible for all content in their abstracts including accuracy of the facts, statements, citing resources, etc.

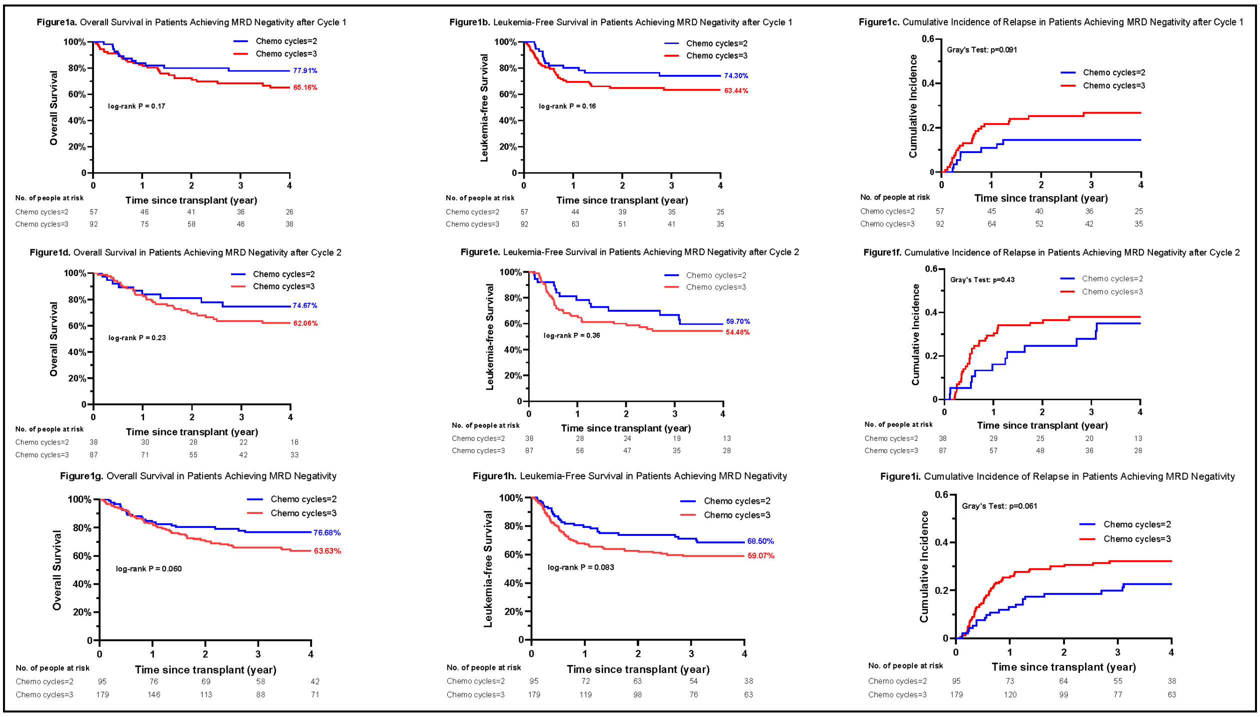

**Copyright Information:** (Online) ISSN: 2572-9241
©2026 The Author(s). HemaSphere is published by John Wiley & Sons Ltd on behalf of European Hematology Association. This is an open access Abstract Book distributed under the Attribution-NonCommercial-NoDerivs (CC BY-NC-ND), which allows third parties to download the articles and share them with others as long as they credit the author and the Abstract Book, but they cannot change the content in any way or use them commercially.
**Abstract Book Citations:** Authors, Title, HemaSphere, 2026;10:(S1):pages. The abstract book DOIs can be found on the "Author Information" page.
**Disclaimer:** Articles published in the journal HemaSphere exclusively reflect the opinions of the authors. The authors are responsible for all content in their abstracts including accuracy of the facts, statements, citing resources, etc.

### (S137) ANTHRACYCLINES-FREE TREATMENT OF 114 CHILDREN AND ADOLESCENTS WITH NEWLY DIAGNOSED ACUTE PROMYELOCYTIC LEUKEMIA (APL): EARLY FINAL RESULTS OF THE ICC-APL-02 STUDY.

**Topic:** 04. Acute myeloid leukemia - Clinical

Luisa Strocchio[1], Pietro Merli[1, 2], Maria Giuseppina Cefalo[1], Barbara Buldini[3], Carmelo Rizzari[4], Cécile Renard Loche[5], Giuseppe Menna[6], Franca Fagioli[7], Benoit Brethon[8], Lucie Sramkova[9], Maaike Luesink[10], Marco Zecca[11], Luca Lo Nigro[12], Josefine Palle[13], Simona Bianchi[14], Brigitte Nelken[15], Paul Saultier[16], Claire Pluchart[17], Anita Spirito[18], Daniela Guardo[19], Liana-Stefania Carausu[20], Justyna Kanold[21], Stéphanie Haouy[22], Fanny Rialland[23], Guy Leverger[24], Marlene Pasquet[25], Tiziana Ottone[26], Valeria Paganelli[1], Mariadomenica Divona[26], Carmelo Gurnari[26], Stéphane Ducassou[27], Anna Maria Testi[28], Maria Teresa Voso[26], Gertjan Kaspers[10], Franco Locatelli*[1, 29]

*[1]IRCCS Bambino Gesù Children's Hospital, Rome, Italy; [2]Saint Camillus International University of Health Sciences, Rome, Italy; [3]University of Padua, Padua, Italy; [4]Fondazione IRCCS San Gerardo dei Tintori, Monza, Italy; [5]Institut d'Hématologie et d'Oncologie Pédiatrique, Hospices Civils de Lyon, Lyon, France; [6]AORN Santobono- Pausilipon Hospital, Naples, Italy; [7]Regina Margherita Children's Hospital, Turin, Italy; [8]Hôpital Robert Debré, APHP, Paris, France; [9]Charles University 2nd Faculty of Medicine and UH Motol, Prague, Czechia; [10]Princess Máxima Cancer Center for Pediatric Oncology, Utrecht, The Netherlands; [11]Fondazione IRCCS Policlinico San Matteo, Pavia, Italy; [12]Azienda Ospedaliero Universitaria Policlinico-San Marco, Catania, Italy; [13]University Children's Hospital, Uppsala, Sweden; [14]IRCCS Meyer Children's Hospital, Florence, Italy; [15]CHU Lille, Lille, France; [16]Aix-Marseille University and La Timone Children's Hospital, Marseille, France; [17]CHU Reims, Reims, France; [18]Fondazione Casa Sollievo della Sofferenza, San Giovanni Rotondo, Italy; [19]IRCCS Istituto Giannina Gaslini, Genova, Italy; [20]CHU de Brest, Brest, France; [21]University Hospital of Clermont-Ferrand, Clermont-Ferrand, France; [22]University Hospital Center of Montpellier, Montpellier, France; [23]Nantes University Hospital, Nantes, France; [24]Hôpital Trousseau, Paris, France; [25]CHU Toulouse, Toulouse, France; [26]University of Rome Tor Vergata, Rome, Italy; [27]Hôpital des Enfants - Groupe Hospitalier Pellegrin, Bordeaux, France; [28]Sapienza, University of Rome, Rome, Italy; [29]University of Sacred Heart, Rome, Italy;*

### Background

The introduction of all-trans retinoic acid (ATRA) and arsenic trioxide (ATO) has radically changed the treatment of acute promyelocytic leukemia (APL), leading to the use of chemotherapy-free protocols in standard-risk (SR) adult patients.

### Aims

Within the International Consortium for Childhood (ICC) APL, we started a multicenter European pediatric trial to treat with a non-chemotherapy-based approach children with newly diagnosed SR and High Risk (HR) APL [NCT04793919].

### Methods

ICC-APL-02 is an open-label prospective, non-randomized, multicenter trial for the treatment of newly diagnosed APL children and adolescents with a risk-stratified protocol [NCT04793919] [according to the modified pediatric criteria (Testi, Blood 2018)], combining ATRA and ATO, plus gemtuzumab ozogamicin (GO on day 2 and 4 of induction) only in HR patients (see Figure 1A for treatment details).
The primary endpoint was event-free survival (EFS), where the events considered were: early death, death in CR, resistant disease and hematological relapse.

**Copyright Information:** (Online) ISSN: 2572-9241
©2026 The Author(s). HemaSphere is published by John Wiley & Sons Ltd on behalf of European Hematology Association. This is an open access Abstract Book distributed under the Attribution-NonCommercial-NoDerivs (CC BY-NC-ND), which allows third parties to download the articles and share them with others as long as they credit the author and the Abstract Book, but they cannot change the content in any way or use them commercially.
**Abstract Book Citations:** Authors, Title, HemaSphere, 2026;10:(S1):pages. The abstract book DOIs can be found on the "Author Information" page.
**Disclaimer:** Articles published in the journal HemaSphere exclusively reflect the opinions of the authors. The authors are responsible for all content in their abstracts including accuracy of the facts, statements, citing resources, etc.

### Results

From 10/2019 to 10/2025, 114 patients (76 SR and 38 HR; see Figure 1 for details) were enrolled in 52 centers across 5 countries (Italy, France, Czech Republic, Netherlands and Sweden). Median duration of induction was 38 days (range 28-60): in 38 patients (31 SR and 7 HR) treatment was temporary stopped for a median of 5 days (range 1-23), due to transient and always reversible toxicity. Forty-seven patients (41.2%) developed differentiation syndrome (DS) during induction at a median time of 10 days (range 3-31) from treatment start, which resolved in all patients. Regarding other relevant toxicities, 13 patients (11.4%) showed prolongation of the QTc interval; 3 of them required temporary ATO discontinuation. In 14 study participants (12.3%) pseudotumor cerebri was diagnosed, while 1 patient developed myocarditis following GO administration (which completely resolved without sequelae); no other drug-related side effect was reported. We recorded only 1 death during induction; in detail, one 16-year old, FLT3-ITD+ HR APL patient died of cerebral hemorrhage on day +30. All patients achieved hematological CR at the end of induction. Molecular CR was tested at the end of the third consolidation course: all evaluated patients but one reached the end-point. The only positive patient (HR) remained MRD+ also after the end of treatment; he was, thus, treated with 2 additional doses of GO, obtaining molecular remission; he then underwent autologous hematopoietic stem cell transplantation and he is actually alive and disease-free. Another patient (SR) had molecular relapse 1 year after completing the treatment protocol; he underwent a new treatment with ATRA-ATO and he is now alive with negative MRD. With a median follow-up of 27 months (range 4-75), the 2-yr overall and EFS of the whole cohort are 99.1% (95% CI 93.9-99.9) (SR 100% vs HR 97.4%, p=n.s.) and 96.9% (95% CI 90.4-99.0) (SR 98.1% vs HR 94.4%, p=n.s.), respectively. Seventeen patients are still on treatment.

### Summary/Conclusion

We reported the first large prospective pediatric trial delivering a non-chemotherapy-based treatment for both children with newly diagnosed SR and HR APL. Our data confirm that treatment with ATRA and ATO is safe and highly effective in SR patients. Notably, the addition of GO to ATR/ATO regimen in HR subjects resulted into excellent efficacy and safety profile in children with *de novo* APL.

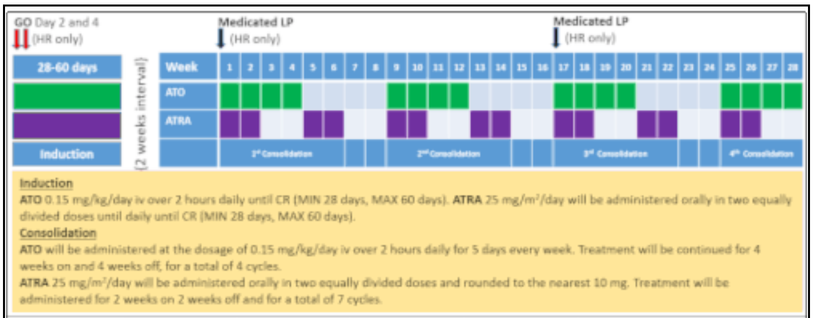

| Patients characteristics | Median age (y), range | Gender M/F | Risk SR/HR | Median WBC count ($x10^9$/l), range SR | Median WBC count ($x10^9$/l), range HR | % FLT3-ITD |
|---|---|---|---|---|---|---|
| N=114 | 13 (1-17) | M 61(53.5%) F 53(46.5%) | SR 76 (66.7%) HR 38 (33.3%) | 2.5 (0.6-9.9) | 21.2 (10.5-151.0) | 50 |

Figure 1. Trial details and patients characteristics.

**Copyright Information:** (Online) ISSN: 2572-9241
©2026 The Author(s). HemaSphere is published by John Wiley & Sons Ltd on behalf of European Hematology Association. This is an open access Abstract Book distributed under the Attribution-NonCommercial-NoDerivs (CC BY-NC-ND), which allows third parties to download the articles and share them with others as long as they credit the author and the Abstract Book, but they cannot change the content in any way or use them commercially.
**Abstract Book Citations:** Authors, Title, HemaSphere, 2026;10:(S1):pages. The abstract book DOIs can be found on the "Author Information" page.
**Disclaimer:** Articles published in the journal HemaSphere exclusively reflect the opinions of the authors. The authors are responsible for all content in their abstracts including accuracy of the facts, statements, citing resources, etc.

### (S138) LUVOMETINIB IN RECURRENT/REFRACTORY PEDIATRIC LANGERHANS CELL HISTIOCYTOSIS: A PHASE 2 TRIAL

**Topic:** 04. Acute myeloid leukemia - Clinical

Rui Zhang*[1], Rong Liu[2], Jie Yu[3], Jianpei Fang[4], Ju Gao[5], Aiguo Liu[6], Sixi Liu[7], Wei Liu[8], Xuequn Luo[9], Yongmin Tang[10], Xingli Wang[11], Zhuli Wu[11], Xin Huang[11], Zhen Wei[11], Yang Zheng[11]

*[1]Beijing Children's Hospital, Capital Medical University, Beijing, China;[2]Children's Hospital Affiliated to Capital Institute of Pediatrics, Beijing, China; [3]Children's Hospital of Chongqing Medical University, Chongqing, China;[4]Sun Yat-sen Memorial Hospital, Sun Yat-Sen University, Guangzhou, China; [5]West China Second University Hospital, Sichuan University, Sichuan, China; [6]Tongji Hospital, Tongji Medical College of Huazhong University of Science and Technology, Wuhan, China;[7]Shenzhen Children's Hospital, Shenzhen, China; [8]Henan Children's Hospital, Zhengzhou Children's Hospital,, Zhengzhou, China;[9]The First Affiliated Hospital, Sun Yat-sen University, Guangzhou, China; [10]Children's Hospital, Zhejiang University School of Medicine, Hangzhou, China; [11]Shanghai Fosun Pharmaceutical Industrial Development Co., Ltd., Shanghai, China;*

### Background

Pediatric recurrent/refractory Langerhans cell histiocytosis (LCH) represents a significant therapeutic challenge, with no approved targeted therapies despite the established role of MAPK pathway activation (e.g., BRAF V600E, MAP2K1 mutations) in its pathogenesis. Current salvage chemotherapies are associated with substantial toxicity. Luvometinib (FCN-159) is a highly potent and selective MEK1/2 inhibitor.

### Aims

This phase 2 trial evaluated the efficacy and safety of luvometinib in pediatric patients with recurrent/refractory LCH irrespective of genotype. we have reported the preliminary efficacy and safety results of this study previously. Here we report the updated data with longer follow-up.

### Methods

This was a single-arm, open-label, phase 2 trial conducted across 11 centers in China (NCT05997602). Eligible patients were aged 2-16 years with histologically confirmed, relapsed/refractory LCH after ≥1 prior systemic therapy. Patients received oral luvometinib at 5 mg/m² once daily in 28-day cycles until progression or unacceptable toxicity. The primary endpoint was the Independent Review Committee (IRC)-assessed objective response rate (ORR) per Positron Emission Tomography Response Criteria (PRC). Secondary endpoints included investigator-assessed ORR (per PRC and Histiocyte Society guidelines), disease control rate (DCR), progression-free survival (PFS), safety, and exploratory analyses of variant allele frequency (VAF) of MAPK pathway mutations in cell-free DNA (cfDNA) and health-related quality of life (HRQoL).

**Copyright Information:** (Online) ISSN: 2572-9241
©2026 The Author(s). HemaSphere is published by John Wiley & Sons Ltd on behalf of European Hematology Association. This is an open access Abstract Book distributed under the Attribution-NonCommercial-NoDerivs (CC BY-NC-ND), which allows third parties to download the articles and share them with others as long as they credit the author and the Abstract Book, but they cannot change the content in any way or use them commercially.
**Abstract Book Citations:** Authors, Title, HemaSphere, 2026;10:(S1):pages. The abstract book DOIs can be found on the "Author Information" page.
**Disclaimer:** Articles published in the journal HemaSphere exclusively reflect the opinions of the authors. The authors are responsible for all content in their abstracts including accuracy of the facts, statements, citing resources, etc.

### Results

Between September 21, 2023 and February 19, 2025, 46 patients were enrolled (median age 7.0 years; 58.7% male). As of August 19, 2025, the median follow-up was 15.1 months. In the efficacy analysis set (n=42), the IRC-assessed ORR per PRC was 90.5% (95% CI: 77.4–97.3), with 69.0% achieving complete metabolic response (CMR). In the ITT analysis set(n=46), the investigator-assessed ORR and 12-month PFS rate per Histiocyte Society Evaluations and Treatment guidelines were 97.8% (95% CI, 88.5-99.9) and 97.8% (95% CI, 85.6-99.7) respectively. Responses were observed across various genotypes: the IRC-assessed ORR per PRC was 91.7% (22/24) in patients with *BRAF* mutations, 100% (4/4) in patients with *MAP2K1* mutations, and 92.3% (12/13) in those without detectable MAPK pathway mutations. VAF of MAPK pathway mutations in cfDNA was decreased to below detection (<0.1%) in 78.9% (15/19) of evaluable patients after six cycles of treatment. Treatment-related adverse events (TRAEs) of any grade occurred in 97.8% of patients, most commonly increased blood creatine phosphokinase (52.2%) and rash (50.0%). Grade ≥3 TRAEs occurred in 8.7% of patients; no TRAE led to treatment discontinuation. Patient- and parent-reported HRQoL showed consistent improvements from baseline.

### Summary/Conclusion

Luvometinib demonstrated profound, genotype-agnostic efficacy and a manageable safety profile in recurrent/refractory pediatric LCH, supporting its potential as a novel salvage therapy.

**Copyright Information:** (Online) ISSN: 2572-9241
©2026 The Author(s). HemaSphere is published by John Wiley & Sons Ltd on behalf of European Hematology Association. This is an open access Abstract Book distributed under the Attribution-NonCommercial-NoDerivs (CC BY-NC-ND), which allows third parties to download the articles and share them with others as long as they credit the author and the Abstract Book, but they cannot change the content in any way or use them commercially.
**Abstract Book Citations:** Authors, Title, HemaSphere, 2026;10:(S1):pages. The abstract book DOIs can be found on the “Author Information” page.
**Disclaimer:** Articles published in the journal HemaSphere exclusively reflect the opinions of the authors. The authors are responsible for all content in their abstracts including accuracy of the facts, statements, citing resources, etc.

### (S139) EZH2 INACTIVATION AND HOXA9-MEDIATED REPROGRAMMING DRIVE HUMAN T-CELL LEUKEMOGENESIS AND DEFINE HIGH-RISK EARLY T-CELL PRECURSOR ACUTE LYMPHOBLASTIC LEUKEMIA (ETP-ALL)

**Topic:** 01. Acute lymphoblastic leukemia - Biology & translational research

Mattia Colucci[1], Costanzo Padovano[1], Gaja Bruno[1], Elisabetta De Santis[1], Serena Di Iasio[1], Chiara Di Nunzio[1], Angela Placentino[1], Peppino Mirabelli[2], Xuehai Wang[3], Andrew Weng[3], Vincenzo Giambra*[1]

*[1]Institute for Stem Cell Biology, Regenerative Medicine and Innovative Therapies (ISBReMIT), Fondazione IRCCS Casa Sollievo della Sofferenza, Hematopathology Unit, San Giovanni Rotondo (FG), Italy; [2]Santobono-Pausilipon Children's Hospital, Clinical and Translational Research Unit, Naples, Italy; [3]BC Cancer Research Institute, Terry Fox Laboratory, Vancouver, Canada;*

### Background

Early T-cell precursor (ETP) acute lymphoblastic leukemia (ALL) is a high-risk hematologic malignancy defined by a maturation block at the earliest stages of thymocyte development and a stem-cell-like transcriptional signature. Loss-of-function mutations in epigenetic regulators—including key components of the Polycomb Repressive Complex 2 (PRC2) such as the EZH2 histone methyltransferase—are hallmark drivers of this disease. However, the specific molecular programs and transcriptomic trajectories that orchestrate both the malignant transformation of T-cell progenitors and maintenance of established human T-ALL remain poorly understood.

### Aims

We aim to define the functional synergy between EZH2 inactivation and oncogenic drivers in human T-cell transformation. Specifically, we seek to characterize the molecular programs underlying PRC2-mutated ETP-ALL to identify high-risk subsets and novel targetable vulnerabilities.

### Methods

We optimized a novel CRISPR/Cas9-based assay to inactivate the EZH2 gene in human cord blood–derived $CD34^{+}$hematopoietic stem/progenitor cells (HSPCs). Subsequently, EZH2 knockout (KO) cells were transduced with lentiviruses encoding different T-ALL oncogenes or an empty vector control and maintained *in vitro* for 30 days in serum-free medium supplemented to support T-cell progenitor expansion. scRNA-seq and scATAC-seq analyses were performed to identify transcriptomic trajectories of human cells at pre-leukemic stages. Furthermore, leukemogenic potential was evaluated *in vivo* by transplantation into immunocompromised (NSG) mice. RNA-seq and ChIP-seq were also performed on human $EZH2^{KO}$ ETP-like ALL cell lines (LOUCY, PEER) to assess gene expression and histone modifications modulated by EZH2 loss in presence or absence of unique oncogenes. Finally, clinical relevance was assessed in a cohort of 1,335 diagnostic T-ALL cases (COG study), as well as at the single-cell level by analyzing scRNA-seq data from in-house primary T-ALL samples and a recently published cohort of 40 cases.

**Copyright Information:** (Online) ISSN: 2572-9241
©2026 The Author(s). HemaSphere is published by John Wiley & Sons Ltd on behalf of European Hematology Association. This is an open access Abstract Book distributed under the Attribution-NonCommercial-NoDerivs (CC BY-NC-ND), which allows third parties to download the articles and share them with others as long as they credit the author and the Abstract Book, but they cannot change the content in any way or use them commercially.
**Abstract Book Citations:** Authors, Title, HemaSphere, 2026;10:(S1):pages. The abstract book DOIs can be found on the “Author Information” page.
**Disclaimer:** Articles published in the journal HemaSphere exclusively reflect the opinions of the authors. The authors are responsible for all content in their abstracts including accuracy of the facts, statements, citing resources, etc.

### Results

Functional assays showed that EZH2 inactivation cooperates with HOXA9 or LYL1 overexpression to drive *in vitro* expansion of human HSPCs enriched for immature T-cell subsets, as confirmed by 26-color flow cytometry and scRNA-seq/ATAC-seq assays. Moreover, HOXA9- or LYL1-transduced EZH2$^{KO}$ HSPCs promoted aggressive T-ALL-like leukemia in NSG mice. Interestingly, PRC2 mutations were strongly associated with the HOXA subgroup and the ETP-like ALL phenotype, correlating with higher minimal residual disease (MRD) at day 30 post-therapy. Furthermore, integrated RNA-seq and ChIP-seq analyses revealed that HOXA9 expression in human EZH2$^{KO}$ ETP-like ALL cell lines induced a stem-like program enriched in chromatin remodelers (e.g., RUNX2, SATB1, ARID5B, CHD1). Notably,scRNA-seq analysis of primary samples identified distinct leukemic subsets with high HOXA9 expression and strong signature enrichment via GSVA. This signature stratified PRC2-mutated T-ALL cases, identifying a HOXA+ subgroup with inferior prognosis, and discriminated ETP-like ALL from non-ETP-like ALL cells at single-cell resolution, confirming its robustness and specificity.

### Summary/Conclusion

Collectively, these findings define a PRC2-deficient chromatin landscape where HOXA9-driven reprogramming enforces a stem-like, refractory state in ETP-like ALL. This signature stratifies high-risk patients and uncovers targetable gene functions, providing a molecular roadmap to eradicate MRD-positive clones at their origin.

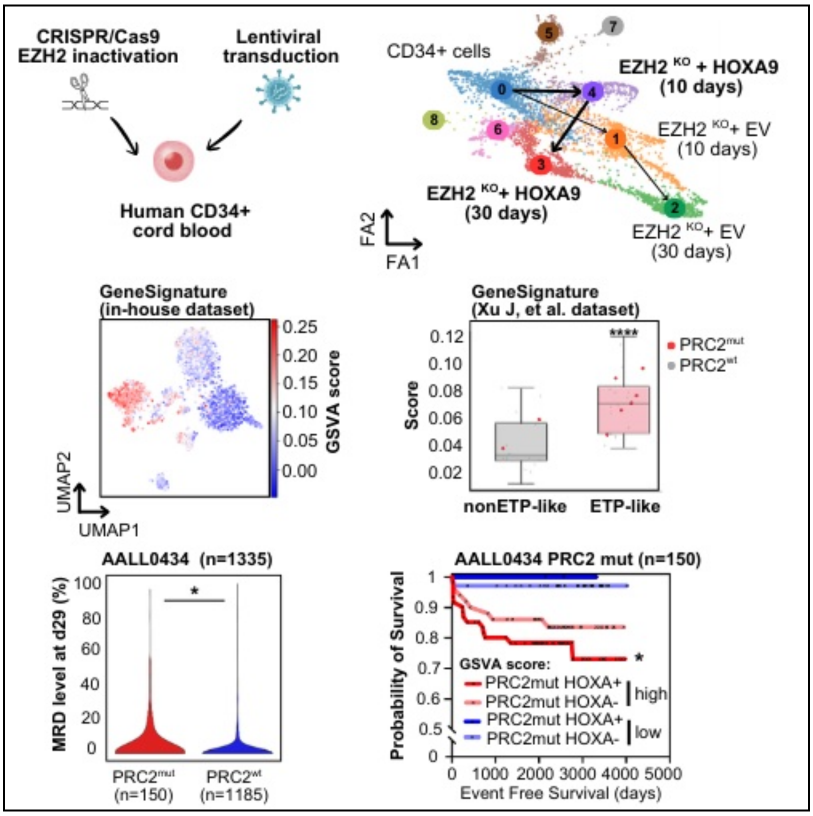

Copyright Information: (Online) ISSN: 2572-9241
©2026 The Author(s). HemaSphere is published by John Wiley & Sons Ltd on behalf of European Hematology Association. This is an open access Abstract Book distributed under the Attribution-NonCommercial-NoDerivs (CC BY-NC-ND), which allows third parties to download the articles and share them with others as long as they credit the author and the Abstract Book, but they cannot change the content in any way or use them commercially.
Abstract Book Citations: Authors, Title, HemaSphere, 2026;10:(S1):pages. The abstract book DOIs can be found on the "Author Information" page.
Disclaimer: Articles published in the journal HemaSphere exclusively reflect the opinions of the authors. The authors are responsible for all content in their abstracts including accuracy of the facts, statements, citing resources, etc.

### (S140) CLONAL TRAJECTORIES OF CHRONIC LYMPHOCYTIC LEUKEMIA AND RICHTER TRANSFORMATION OVER THE PATIENTS' LIFESPAN

**Topic:** 05. Chronic lymphocytic leukemia and related disorders - Biology & translational research

Ian Márquez-López*[1, 2, 3], Aleix Real[1, 2, 3], João M Alves[4, 5], Núria Russiñol[1], Romina Royo[6], Markus J van Roosmalen[7, 8], Heribert Playà-Albinyana[1, 3], Juan A. Piñeyroa[1, 9], Melika Bashiri[1], Silvia Ruiz-Gaspà[1], Pablo Javier Mozas Fernandez[1, 9], Julio Delgado[1, 2, 3, 9], Armando Lopez Guillermo[1, 2, 3, 9], Dolors Colomer Pujol[1, 2, 3, 9], Ruben van Boxtel[7, 8], David Posada[4, 5], Elias Campo[1, 2, 3, 9], Ferran Nadeu[1, 3]

*[1]Fundació de Recerca Clínic Barcelona-Institut d'Investigacions Biomèdiques August Pi i Sunyer (FRCB-IDIBAPS), Barcelona, Spain; [2]Universitat de Barcelona, Barcelona, Spain;[3]Centro de Investigación Biomédica en Red de Cáncer (CIBERONC), Madrid, Spain; [4]CINBIO, Universidade de Vigo, Department of Biochemistry, Genetics, and Immunology, Vigo, Spain;[5]Galicia Sur Health Research Institute (IIS Galicia Sur), SERGAS-UVIGO, Vigo, Spain; [6]Barcelona Supercomputing Center (BSC), Barcelona, Spain; [7]Princess Máxima Center for Pediatric Oncology, Utrecht, The Netherlands;[8]Oncode Institute, Utrecht, The Netherlands; [9]Hospital Clínic De Barcelona, Barcelona, Spain;*

### Background

Richter transformation (RT) is a rare but clinically challenging event in the evolution of chronic lymphocytic leukemia (CLL), characterized by the transformation of CLL into an aggressive lymphoma, commonly diffuse large B-cell lymphoma, conferring a dismal outcome. Although the molecular profiles of CLL and RT are well characterized, their evolutionary trajectories remain uncertain. Thus, a better understanding of CLL and RT longitudinal dynamics could enable earlier identification and intervention in these high-risk patients.

### Aims

Our aim was to trace CLL and RT clonal histories over the lifespan of the patients through the analysis of tumor phylogenies at single-cell whole-genome sequencing (scWGS) resolution.

### Methods

We applied primary template-directed amplification (PTA)-based scWGS to 5 patients, analyzing the genome of 349 individual cells from up to 6 sequential time points spanning up to 19 years from CLL diagnosis to RT. A bioinformatic workflow that integrates existing algorithms with an in-house PTA-artifact filtering strategy was developed to optimize the sensitivity and specificity of the variant calling. A median sensitivity of 87.7% was obtained for point mutations at a sequencing depth of ~20×, with only ~5% of cell-specific artifacts remaining.

**Copyright Information:** (Online) ISSN: 2572-9241
©2026 The Author(s). HemaSphere is published by John Wiley & Sons Ltd on behalf of European Hematology Association. This is an open access Abstract Book distributed under the Attribution-NonCommercial-NoDerivs (CC BY-NC-ND), which allows third parties to download the articles and share them with others as long as they credit the author and the Abstract Book, but they cannot change the content in any way or use them commercially.
**Abstract Book Citations:** Authors, Title, HemaSphere, 2026;10:(S1):pages. The abstract book DOIs can be found on the "Author Information" page.
**Disclaimer:** Articles published in the journal HemaSphere exclusively reflect the opinions of the authors. The authors are responsible for all content in their abstracts including accuracy of the facts, statements, citing resources, etc.

**Results**

Genomic characterization of single cells uncovered a progressive increase in mutational burden from normal B cells to CLL and from CLL to RT, as well as an extensive intratumor heterogeneity of somatic mutations and copy number alterations previously missed by bulk sequencing approaches. Reconstruction of tumor phylogenies revealed that the initial diversification of CLL occurs decades before diagnosis, generally following the acquisition of genetic drivers. However, founder driver mutations did not directly trigger CLL proliferation in all cases, suggesting that cell-extrinsic factors such as B-cell receptor stimulation or microenvironmental changes may promote CLL expansion. Notably, in 2 patients with material available at CLL diagnosis, proliferation bursts occurred ~10 years prior to clinical presentation, followed by a phase of slower growth lacking clear phylogenetic structure, consistent with weak selective constraints. In contrast, relapses after therapy exhibited clear signatures of clonal selection, including an abrupt increase in mutational burden driven by combined therapy-related and cell-intrinsic mutational processes, as well as striking intrapatient parallel genomic evolution. This was exemplified by the independent acquisition of multiple del(13q) events in 3 cases, del(9p21) in 2 cases, and 7 LOH(17p) events across 4 distinct phylogenetic clades harboring different *TP53* mutations within a single patient. Finally, RT diverged early from CLL, in some cases prior to diagnosis. Nevertheless, these clones underwent a years-long evolutionary trajectory, during which the acquisition of cell-cycle alterations and, at advanced stages, high genomic complexity facilitated their final outgrowth.

**Summary/Conclusion**

This study provides a roadmap of the origin, early diversification, and evolution of CLL and RT, with potential clinical implications for early detection and intervention, and establishes a framework for studying the evolution of other hematological malignancies.

**Copyright Information:** (Online) ISSN: 2572-9241
©2026 The Author(s). HemaSphere is published by John Wiley & Sons Ltd on behalf of European Hematology Association. This is an open access Abstract Book distributed under the Attribution-NonCommercial-NoDerivs (CC BY-NC-ND), which allows third parties to download the articles and share them with others as long as they credit the author and the Abstract Book, but they cannot change the content in any way or use them commercially.
**Abstract Book Citations:** Authors, Title, HemaSphere, 2026;10:(S1):pages. The abstract book DOIs can be found on the "Author Information" page.
**Disclaimer:** Articles published in the journal HemaSphere exclusively reflect the opinions of the authors. The authors are responsible for all content in their abstracts including accuracy of the facts, statements, citing resources, etc.

### (S141) BCR-REGULATED LNCRNA LINC00173 LIMITS RIBONUCLEOPROTEIN GRANULES (RNP) ASSEMBLY TO CONTROL MRNA TRANSLATION IN CLL

**Topic:** 05. Chronic lymphocytic leukemia and related disorders - Biology & translational research

Pedro Faria Zeni*[1, 2], Michaela Medkova[1, 2], Libuse Janska[1], Jakub Pospisil[1], Daniel Filip[1, 2], Laura Ondrisova[1, 2], Wojciech Jesionek[1], Nicolas Blavet[1], Alejandro Medaglia Mata[1], Miroslav Boudny[1, 2], Nandan Varadarajan[1], Vaclav Seda[1, 2], Ales Obrdlik[1], Amiyaranjan Das[1], Marcos González Lopez[2], Jan Krivanek[2], Thorsten Zenz[3], Gabriel Abrante Diaz[4], Susana Vinga[4], Junyan Lu[5], Marcela Hortova-Kohoutkova[6], Kristyna Kupcova[7], Alba Maiques-Diaz[8], Michael Doubek[2], Vassilios Vasileiou[9], Fotis Psomopoulos[9], Petr Nickl[10], Ondrej Havranek[7], Jan Fric[6], José Ignacio Martín-Subero[8], Gabriel Demo[1], Stepanka Vanacova[1], Jana Balounova[10], Radislav Sedlacek[10], Marek Mraz[1, 2]

*[1]CEITEC, Masaryk University, Brno, Czechia; [2]University Hospital Brno, Brno, Czechia; [3]University of Zurich, Zurich, Switzerland; [4]INESC-ID, Instituto Superior Técnico, Universidade de Lisboa, Lisbon, Portugal;[5]University of Heidelberg, Heidelberg, Germany; [6]ICRC St. Anne's University Hospital, Brno, Czechia; [7]BIOCEV, Prague, Czechia; [8]IDIBAPS, Barcelona, Spain; [9]INAB-CERTH, Thessaloniki, Greece; [10]CCP, Institute of Molecular Genetics of the Czech Academy of Sciences, Prague, Czechia;*

### Background

We and others have shown that microRNAs are embedded in the BCR signaling pathway in CLL and normal B cells, however, the role of long non-coding RNAs (lncRNAs) remains unclear.

### Aims

To identify BCR-associated lncRNAs and define their functional relevance in CLL.

### Methods

Functional studies utilized CRISPR interference, calcium influx (Fluo4), mitochondrial respiration (Seahorse), RNA pulldown/mass spectrometry, RNP granule formation assays (RNA-FISH), a novel mouse knockout (KO) model, and single-cell transcriptomic profiling for cellular phenotypic characterization.

**Copyright Information:** (Online) ISSN: 2572-9241
©2026 The Author(s). HemaSphere is published by John Wiley & Sons Ltd on behalf of European Hematology Association. This is an open access Abstract Book distributed under the Attribution-NonCommercial-NoDerivs (CC BY-NC-ND), which allows third parties to download the articles and share them with others as long as they credit the author and the Abstract Book, but they cannot change the content in any way or use them commercially.
**Abstract Book Citations:** Authors, Title, HemaSphere, 2026;10:(S1):pages. The abstract book DOIs can be found on the “Author Information” page.
**Disclaimer:** Articles published in the journal HemaSphere exclusively reflect the opinions of the authors. The authors are responsible for all content in their abstracts including accuracy of the facts, statements, citing resources, etc.

### Results

We identified 23 BCR-associated lncRNAs (Padj<0.05) by analyzing lncRNA expression in CXCR4/CD5 CLL subpopulations (n=10 pairs) and in samples obtained before and during BCR inhibitor therapy (ibrutinib n=5; idelalisib n=10). *LINC00173* was prioritized for further functional studies due to its evolutionary conservation and clinical associations. Lower *LINC00173* expression correlated with shorter OS in a chemoimmunotherapy-treated CLL cohort (n=100; *TP53* wt, P=0.04, **Fig.A**), BCR signaling score in CLL cells (n=70 vs 70; P=0.02), and its expression was higher in healthy B cells (2.1-fold; **Fig.B**). *LINC00173* expression was induced ~6-fold after BCR stimulation of primary CLL cells (anti-IgM; P=0.003) and blocked by BCR inhibitors (**Fig.C**). *LINC00173* knockdown (KD) in MEC1 cells altered the expression of 470 genes, with enrichment for oxidative phosphorylation (OXPHOS; **Fig.D**). Despite this transcriptional enrichment, KD cells showed reduced oxygen consumption (**Fig. E**). RNA pulldown identified six proteins interacting with *LINC00173* and linked to mRNA translation and ribonucleoprotein (RNP) granules (EIF4E, IGF2BP3, G3BP2, RALY, MOV10, EDF1; P<0.05). This led us to hypothesize that *LINC00173* regulates RNP granule formation. Indeed, KD cells exhibited ~3-fold increased RNP granule formation (**Fig.F,G**) and ~65% reduction in de novo protein synthesis (**Fig.H**), whereas *LINC00173* overexpression resulted in ~25% higher translation rate (**Fig.I**). Characterisation of polysome content showed that mRNAs encoding for OXPHOS and $Ca^{2+}$/BCR signaling failed to enter the ribosomal polysomes in KD cells, supporting a model in which altered RNP dynamics affects mRNA translation. Specifically, polysomes from KD cells were depleted of mRNAs for *IMPK* kinase and *INPP5B* phosphatase (**Fig.J**), both regulators of $IP_3$ turnover (a 2nd messenger regulating Ca2+ release from endoplasmic reticulum). This is likely responsible for increased $Ca^{2+}$ influx upon BCR activation in KD cells (**Fig.K**). Moreover, BCR activation induced RNP granules in primary CLL samples (2.7-fold; n=8; P=0.01, **Fig.L,M**). This indicates that the BCR-*LINC00173*-RNP granules axis is involved in CLL pathophysiology by regulating mRNA translation. To test the function of *LINC00173 in vivo*, we generated germline KO mice, which resulted in ~21% fewer bone marrow B cells, ~10% fewer splenic B cells, and a ~40% increase in B1b cells (n=10, P<0.05, **Fig.N**). scRNA profiling of WT vs. KO mouse B cells revealed reduced translation and enhanced Ig-mediated response signatures (**Fig. O**). Importantly, KO mouse B cells had higher RNP granule formation compared to WT controls (95% vs. 68%; P=0.002, **Fig.P,Q**), indicating conserved *LINC00173* function.

### Summary/Conclusion

*LINC00173* is a BCR-induced lncRNA and represents a novel regulator of mRNA translation via RNP granule formation in CLL and normal B cells.

Supported by: Czech Science Foundation (25-15368X), MH CZ-DRO (FNBr,65269705), MUNI/A/1733/2025.

**Copyright Information:** (Online) ISSN: 2572-9241
©2026 The Author(s). HemaSphere is published by John Wiley & Sons Ltd on behalf of European Hematology Association. This is an open access Abstract Book distributed under the Attribution-NonCommercial-NoDerivs (CC BY-NC-ND), which allows third parties to download the articles and share them with others as long as they credit the author and the Abstract Book, but they cannot change the content in any way or use them commercially.
**Abstract Book Citations:** Authors, Title, HemaSphere, 2026;10:(S1):pages. The abstract book DOIs can be found on the "Author Information" page.
**Disclaimer:** Articles published in the journal HemaSphere exclusively reflect the opinions of the authors. The authors are responsible for all content in their abstracts including accuracy of the facts, statements, citing resources, etc.

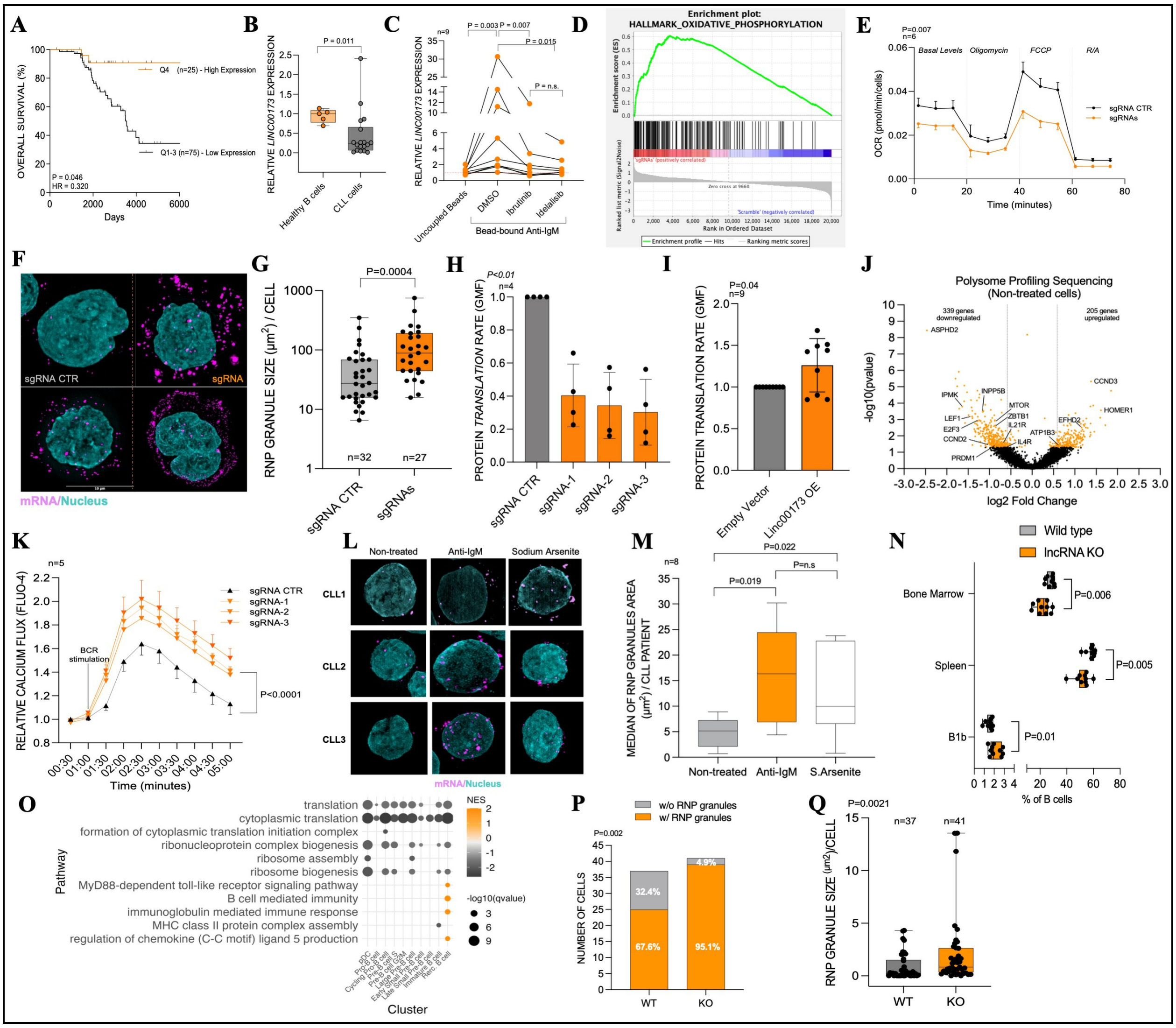

**Copyright Information:** (Online) ISSN: 2572-9241
©2026 The Author(s). HemaSphere is published by John Wiley & Sons Ltd on behalf of European Hematology Association. This is an open access Abstract Book distributed under the Attribution-NonCommercial-NoDerivs (CC BY-NC-ND), which allows third parties to download the articles and share them with others as long as they credit the author and the Abstract Book, but they cannot change the content in any way or use them commercially.
**Abstract Book Citations:** Authors, Title, HemaSphere, 2026;10:(S1):pages. The abstract book DOIs can be found on the "Author Information" page.
**Disclaimer:** Articles published in the journal HemaSphere exclusively reflect the opinions of the authors. The authors are responsible for all content in their abstracts including accuracy of the facts, statements, citing resources, etc.

### (S142) RESOLVING THE TUMOR HETEROGENEITY OF CLL SUBSET #8 AT SINGLE-CELL RESOLUTION: INSIGHTS INTO CLONAL DYNAMICS AND DISTINCT SUB-CLONAL MUTATIONAL LANDSCAPES

**Topic:** 05. Chronic lymphocytic leukemia and related disorders - Biology & translational research

Athanasios Roussos*[1, 2], Konstantinos Kardamiliotis[1], Antonios Mingos[1], Aikaterini Mallou[1], Andreas Agathangelidis[3], Michela Frenquelli[4], Riccardo Moia[5], Anton Langerak[6], Karla Plevova[7, 8], Ian Márquez-López[9, 10], Maria Satra[2], Elisavet Vlachonikola[1], Anastasia Iatrou[1], Georgios Gavriilidis[1, 11], Ferran Nadeu[9, 10], Sarka Pospisilova[7, 8], Gianluca Gaidano[5], Paolo Ghia[4, 12], Elias Campo[9, 10], Fotis Psomopoulos[1], Anastasia Chatzidimitriou[1, 13], Kostas Stamatopoulos[1, 13]

*[1]Institute of Applied Biosciences, Centre for Research and Technology Hellas, Thessaloniki, Greece;[2]Department of Public and One Health, University of Thessaly, Karditsa, Greece; [3]Department of Biology, School of Science, National and Kapodistrian University of Athens, Athens, Greece; [4]Comprehensive Cancer Center, IRCCS Ospedale San Raffaele, Milano, Italy; [5]Division of Hematology, Department of Translational Medicine, Università del Piemonte Orientale, Novara, Italy; [6]Department of Immunology, Laboratory Medical Immunology, Erasmus MC, University Medical Center Rotterdam, Rotterdam, The Netherlands; [7]Department of Internal Medicine, Hematology and Oncology, University Hospital Brno & Faculty of Medicine, Masaryk University, Brno, Czechia; [8]Institute of Medical Genetics and Genomics, University Hospital Brno & Faculty of Medicine, Masaryk University, Brno, Czechia; [9]Fundació de Recerca Clínic Barcelona-Institut d'Investigacions Biomèdiques August Pi i Sunyer (FRCB-IDIBAPS), Barcelona, Spain; [10]Centro de Investigación Biomédica en Red de Cáncer (CIBERONC), Madrid, Spain; [11]Berlin Institute of Health at Charité - Universitätsmedizin Berlin, Center of Digital Health, Berlin, Germany;[12]Medical School, Università Vita-Salute San Raffaele, Milano, Italy; [13]Department of Molecular Medicine and Surgery, Karolinska Institutet, Stockholm, Sweden;*

### Background

CLL subset #8, defined by the expression of stereotyped IGHV4-39/IGKV1(D)-39 B-cell receptor immunoglobulin (BcR IG), exhibits the highest risk of Richter Transformation (RT) amongst all CLL. This distinctive clinical course is likely driven by interactions between the malignant cells and the tumor microenvironment (TME), a claim supported by the well-known promiscuous antigen reactivity of the clonotypic BcR IG in subset #8 cases.

### Aims

We aimed to (i) delineate the malignant-TME crosstalk in CLL subset #8 at single-cell resolution, with a particular focus on bystander T cells; and (ii) uncover intraclonal diversification at the genomic and transcriptomic levels.

### Methods

We analyzed 20 samples from 11 subset #8 patients by single-cell RNA-sequencing (scRNA-seq, 10x Genomics). Five patients were studied prior to the administration of any treatment, while the rest were analyzed longitudinally, under various treatment modalities. One case progressed to RT after relapsing on BTKi and BCL2 inhibitor therapy. Seventeen longitudinal samples from 8 patients were subjected to single-cell T-cell receptor (TR) gene sequencing. Additionally, 5/11 patients were studied using single-cell DNA-sequencing (scDNA-seq, Mission Bio) with a panel examining CLL driver alterations in 35 genes. scRNA-seq data were analyzed using Seurat and scRepertoire. The ERGO-II software was employed for assessing TR-neoantigen (nAg)-MHC binding probabilities. scDNA-seq data were analyzed using the Tapestri pipeline and the Mosaic software.

**Copyright Information:** (Online) ISSN: 2572-9241
©2026 The Author(s). HemaSphere is published by John Wiley & Sons Ltd on behalf of European Hematology Association. This is an open access Abstract Book distributed under the Attribution-NonCommercial-NoDerivs (CC BY-NC-ND), which allows third parties to download the articles and share them with others as long as they credit the author and the Abstract Book, but they cannot change the content in any way or use them commercially.
**Abstract Book Citations:** Authors, Title, HemaSphere, 2026;10:(S1):pages. The abstract book DOIs can be found on the “Author Information” page.
**Disclaimer:** Articles published in the journal HemaSphere exclusively reflect the opinions of the authors. The authors are responsible for all content in their abstracts including accuracy of the facts, statements, citing resources, etc.

**Results**

Malignant B cells predominated all samples and primarily displayed a "resting fraction" phenotype ($CXCR4^{high}CD5^{low}$), suggesting priming to re-enter lymphoid tissues. A distinct subpopulation of activated CLL B cells was also identified, characterized by elevated expression of NF-κB pathway genes (*NFKB1, REL, RELA*) and co-expression of immediate-early response genes. In all patients, T cells included $CD4^{+}$, $CD8^{+}$, double negative T, MAIT and regulatory T cells. The ERGO-II software indicated a high probability that 56/240 (23.3%) T-cell clones from 4/7 patients could be nAg-specific, their TRs recognizing putative nAgs derived from the CDR3 of the clonotypic IGHV4-39 heavy chains; in 3/4 patients, the dominant T-cell clone was also nAg-specific. Most (35/56, 62.5%) nAg-specific T-cell clones persisted longitudinally, albeit with differences in clonal size over time, and were significantly enriched in the $CD8^{+}$ effector memory (TEM) and $CD4^{+}$ cytotoxic (CTL) clusters. Transcriptionally, they maintained an activated and cytotoxic profile, with elevated expression of effector molecules. However, in the RT sample, the T-cell compartment shifted toward a suppressed effector profile, with attenuated inflammation, while the malignant B cells acquired a less-differentiated profile with metabolic reprogramming. scDNA-seq of the RT case revealed that a minor subclone with co-occurring $FBXW7^{R505C}$ and $CARD11^{E122G}$ mutations identified at diagnosis expanded upon relapse to both treatments, eventually becoming the dominant subclone at RT. Concurrently, a triple mutant clone emerged, additionally harboring the $DDX3X^{C175*}$ mutation.

**Summary/Conclusion**

Intraclonal heterogeneity in CLL subset #8 is a dynamic process evolving longitudinally. Persisting expansions of putative nAg-specific T cells allude to active albeit inefficient anti-tumor immune responses.

**Copyright Information:** (Online) ISSN: 2572-9241
©2026 The Author(s). HemaSphere is published by John Wiley & Sons Ltd on behalf of European Hematology Association. This is an open access Abstract Book distributed under the Attribution-NonCommercial-NoDerivs (CC BY-NC-ND), which allows third parties to download the articles and share them with others as long as they credit the author and the Abstract Book, but they cannot change the content in any way or use them commercially.
**Abstract Book Citations:** Authors, Title, HemaSphere, 2026;10:(S1):pages. The abstract book DOIs can be found on the "Author Information" page.
**Disclaimer:** Articles published in the journal HemaSphere exclusively reflect the opinions of the authors. The authors are responsible for all content in their abstracts including accuracy of the facts, statements, citing resources, etc.

### (S143) PROSPECTIVE VALIDATION OF THE CLL-TIM MACHINE-LEARNING ALGORITHM PREDICTING INFECTION AND TREATMENT IN CLL: RESULTS FROM THE PHASE 2 CLINICAL TRIAL PREVENT-ACALL, NON-RANDOMIZED COHORT

**Topic:** 05. Chronic lymphocytic leukemia and related disorders - Biology & translational research

Rudi Agius[1, 2], Caspar Da Cunha-Bang[1, 2], Andreas Katsimigas[1, 2], Anders Osterborg[3], Jeanette Lundin[3], Magdalena Kättström[4], Richard Rosenquist[5], Robert Schou Pedersen[6], Fransien de Boer[7], Koen De Heer[8], Teresa del Peso Santos[9], Christian Brieghel[1, 2], Lisbeth Enggaard[1], Jindrich Mourek[1], Par Josefsson[1], Klaus Rostgaard[2], Christian Bjørn Poulsen[9], Arnon Philip Kater[10], Mark-David Levin[11], Carsten Niemann*[1, 2, 12]

*[1]Righospitalet, Copenhagen University Hospital, Department of Hematology, Copenhagen, Denmark;[2]Hematology branch, Danish Cancer Institute, Copenhagen, Denmark; [3]Karolinska University Hospital Solna, Stockholm, Sweden; [4]Örebro University Hospital, Section of Hematology, Department of Medicine, Örebro, Sweden; [5]Karolinska Institutet, Department of Molecular Medicine and Surgery, Karolinska Institutet, Stockholm, Sweden; [6]Gødstrup Hospital, Department of Hematology, Herning, Denmark; [7]Ikazia Hospital, Rotterdam, The Netherlands; [8]Flevoziekenhuis, Department of Internal Medicine,, Almere, The Netherlands; [9]Roskilde University Hospital, Department of Hematology, Rosklide, Denmark; [10]Cancer Center Amsterdam, Amsterdam UMC, University of Amsterdam, Department of Hematology, Amsterdam, The Netherlands; [11]Albert Schweitzer hospital, Dordrecht, Netherlands, Department of Internal Medicine, Dordrecht, The Netherlands; [12]University of Copenhagen, Department of Clinical Medicine, Copenhagen, Denmark;*

### Background

The Chronic Lymphocytic Leukemia (CLL) Treatment-Infection Model (CLL-TIM) is a machine-learning algorithm designed to identify newly diagnosed, asymptomatic patients with CLL at high risk of severe infection or CLL treatment within two years. While initially developed and validated on retrospective datasets (Agius et al., Nat Comm, 2020), this is the first report on prospective validation of a predictive score for CLL in a prospective, investigator-initiated, multicenter clinical trial. By testing CLL-TIM across diverse international hospitals in Prevent-ACaLL, this study stress-tests the model's predictive confidence and its ability to generalize across varying clinical practices.

### Aims

Based on CLL-TIM, patients were classified into four categories: **High Risk/High Confidence (HR-HC)**, **High Risk/Low Confidence (HR-LC)**, **Low Risk/Low Confidence (LR-LC)** and **Low Risk/High Confidence (LR-HC)**.
To prospectively validate the clinical utility of CLL-TIM in the **PreVent-ACaLL trial**. Specifically, we test:

1. **Generalization:** Whether the predicted High-Risk/High-Confidence (HR-HC) group demonstrates a significantly worse outcome than the Low-Risk (LR) groups.
2. **Reliability:** Whether CLL-TIM's confidence assigned to each prediction is reliable; specifically, that HR-HC predictions identify worse outcomes than High-Risk/Low-Confidence (HR-LC).

### Methods

Outcomes include CLL-TIM's composite outcome of (CTCAE) grade ≥3 infection-free / treatment-free survival (**IF-TFS**) and the exploratory outcomes of overall survival (**OS**), grade ≥3 infection-free survival (**IFS**) and treatment-free survival (**TFS**). Outcomes were calculated on the non-randomized cohort from the PreVent-ACaLL trial (NCT03868722) that were assessed according to protocol and patients were enrolled at 9 international sites.

**Copyright Information:** (Online) ISSN: 2572-9241
©2026 The Author(s). HemaSphere is published by John Wiley & Sons Ltd on behalf of European Hematology Association. This is an open access Abstract Book distributed under the Attribution-NonCommercial-NoDerivs (CC BY-NC-ND), which allows third parties to download the articles and share them with others as long as they credit the author and the Abstract Book, but they cannot change the content in any way or use them commercially.
**Abstract Book Citations:** Authors, Title, HemaSphere, 2026;10:(S1):pages. The abstract book DOIs can be found on the "Author Information" page.
**Disclaimer:** Articles published in the journal HemaSphere exclusively reflect the opinions of the authors. The authors are responsible for all content in their abstracts including accuracy of the facts, statements, citing resources, etc.

### Results

Of 574 patients assessed, 95 (17%) patients were classified as **HR-HC**, of whom 40 were included in the randomized part of the trial reported elsewhere, while 55 non-randomized patients are reported on here. **HR-LC** constituted 59 (10%) of patients, 290 (50%) were **LR-LC** and 130 (23%) were **LR-HC**.

1. **Composite (IF-TFS) (Fig 1a):**
    - **Generalization:** HR-HC (54%) vs. HR-LC (74.7%), LR-LC (90.3%), and LR-HC (95.9%). Differences between HR-HC and both LR groups were significant
    - **Reliability**: The confidence metric was validated; the HR-HC group had significantly worse outcomes than the HR-LC group (p<0.045)

1. **Survival (OS) (Fig 1b):** Though not trained on survival, HR-HC 2-year survival (88.7%) was significantly lower than LR groups (p<0.03)
2. **Infection (IF) / Treatment (TFS):** HR-HC showed significantly lower 2-year IFS (84%, p<=0.02) and TFS (56%, p <0.001) compared to LR groups

### Summary/Conclusion

We present the first prospective, international validation of a predictive algorithm (CLL-TIM) for clinical hematology, fulfilling EU ethical requirements for implementing decision support tools. Our findings confirm CLL-TIM's **generalization**: HR groups exhibited significantly higher frequency of infection and/or treatment (**IF-TFS**) than LR groups. **Reliability** was also verified; **HR-HC** patients showed significantly worse outcomes compared to **HR-LC**, confirming that CLL-TIM's confidence may be used as proxy for when a prediction may be failing due to missingness or clinical practice variance. The results indicate CLL-TIM's potential for **EHR integration** as decision support for tailoring clinical management, prioritizing infection prophylaxis and close monitoring for treatment indication in **HR-HC** patients.

**Copyright Information:** (Online) ISSN: 2572-9241
©2026 The Author(s). HemaSphere is published by John Wiley & Sons Ltd on behalf of European Hematology Association. This is an open access Abstract Book distributed under the Attribution-NonCommercial-NoDerivs (CC BY-NC-ND), which allows third parties to download the articles and share them with others as long as they credit the author and the Abstract Book, but they cannot change the content in any way or use them commercially.
**Abstract Book Citations:** Authors, Title, HemaSphere, 2026;10:(S1):pages. The abstract book DOIs can be found on the "Author Information" page.
**Disclaimer:** Articles published in the journal HemaSphere exclusively reflect the opinions of the authors. The authors are responsible for all content in their abstracts including accuracy of the facts, statements, citing resources, etc.

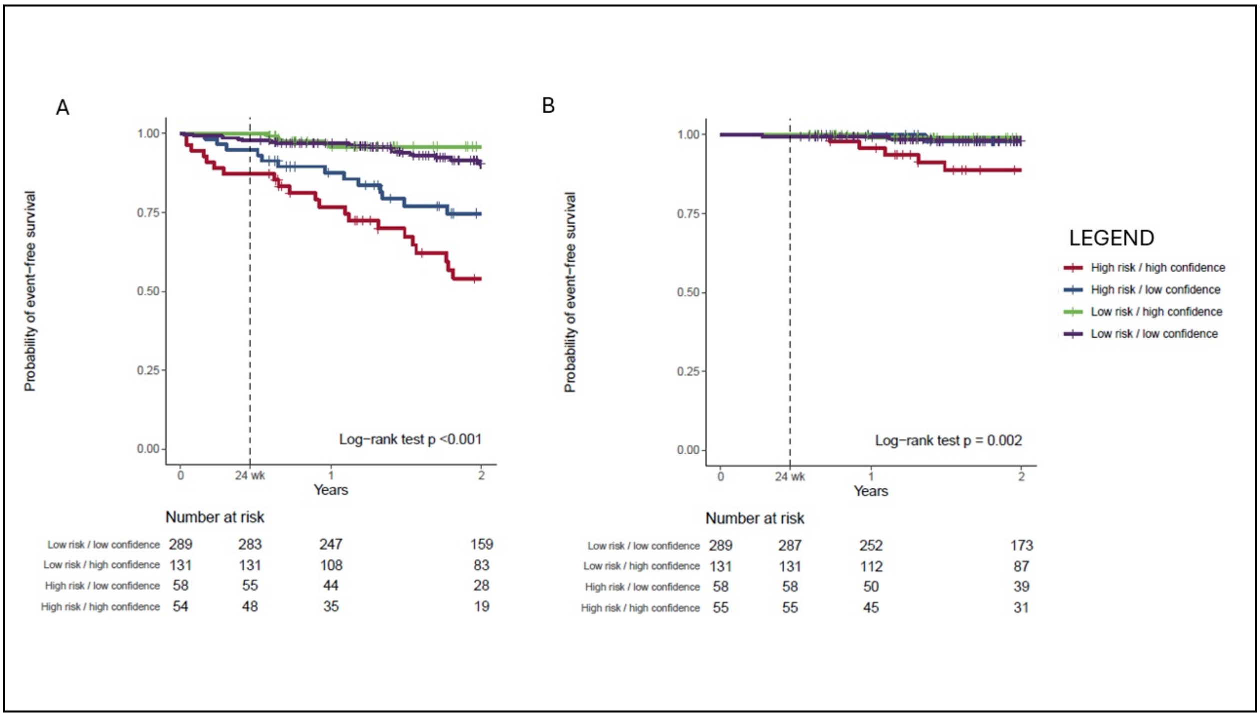

Fig. 1A) Grade 3 infection–/treatment–free survival (IF-TFS) for non–randomized patients. CLL-TIM Generalization: The HR-HC group demonstrated 54% IF-TFS at 2 years, compared to 74.7% for HR-LC, 90.3% for LR-LC and 95.9% for LR-HC. Separation of HR-HC groups vs LR groups was significant: using pairwise log-rank test, the HR-HC group vs LR-HC and LR-LC was p<0.001 for both. Separation of the HR-LC group vs LR-HC and LR-LC was p<0.001and p=0.001 respectively. CLL-TIM Reliability: Confidence metric was found to be reliable with pairwise log-rank p<0.045 between the HR-HC vs HR-LC group.Fig. 1B) Overall survival (OS) for non–randomized patients. Though not trained on survival, lower OS-rate (88.7% at 2 years) was observed for the HR-HC group as compared to the 3 other groups. Separation of HR-HC groups vs LR groups was significant: using pairwise log-rank test, the HR-HC group vs LR-HC and LR-LC was p=0.003 and p=0.002 respectively.

**Copyright Information:** (Online) ISSN: 2572-9241
©2026 The Author(s). HemaSphere is published by John Wiley & Sons Ltd on behalf of European Hematology Association. This is an open access Abstract Book distributed under the Attribution-NonCommercial-NoDerivs (CC BY-NC-ND), which allows third parties to download the articles and share them with others as long as they credit the author and the Abstract Book, but they cannot change the content in any way or use them commercially.
**Abstract Book Citations:** Authors, Title, HemaSphere, 2026;10:(S1):pages. The abstract book DOIs can be found on the “Author Information” page.
**Disclaimer:** Articles published in the journal HemaSphere exclusively reflect the opinions of the authors. The authors are responsible for all content in their abstracts including accuracy of the facts, statements, citing resources, etc.

### (S144) A RIBOCANCER CELL LINE PANEL REVEALS THAT CLL-ASSOCIATED RPS15 MUTATIONS TRANSLATIONALLY REWIRE TRANSCRIPTION THROUGH CODON-SPECIFIC TRNA ACCOMMODATION DEFECTS

**Topic:** 05. Chronic lymphocytic leukemia and related disorders - Biology & translational research

Marino Caruso*[1], Anais Astier[2], Stijn Vereecke[1], Paulo Espirito Santo[2], Coralie Capron[3], Carine Froment[4], Dana Rinaldi[2], David Cabrerizo Granados[1], Marine Leclercq[3], Naomy Pasau[1], Daniele Pepe[1], Virginie Marcel[5], Sebastien Durand[5], Clement Chapat[2], Gerben Menschaert[6], Pierre Close[3], Francesca Rapino[3], Fréderic Catez[5], Julien Marcoux[4], Célia Plisson-Chastang[2], Kim de Keersmaecker[1]

*[1]KU Leuven, Department of Oncology, Leuven, Belgium;[2]University of Toulouse, Centre de Biologie Intégrative, Toulouse, France; [3]University of Liège, GIGA institute, Liège, Belgium; [4]University of Toulouse, Institut de pharmacologie et de biologie structurale (IPBS), Toulouse, France; [5]University Claude Bernard Lyon, Institut convergence Plascan, Lyon, France;[6]Ghent University, Ghent, Belgium;*

### Background

Mutations in ribosomal protein (RP) genes are associated with oncogenesis as illustrated by deletions in RPL5, RPL11 and RPL22 in hematologic diseases such as Diamond Blackfan Anemia, T-ALL, multiple myeloma and in a variety of solid tumors. Additionally, point mutations in RPL10 and RPS15 are found in T-ALL and CLL respectively. The role of these different RP defects in oncogenesis remains incompletely understood. We showed that the RPL10 R98S mutation generates a ribosome that hypertranslates oncoproteins as BCL2, providing opportunities to target RPL10 R98S T-ALL cells with BCL2 inhibitor venetoclax. In CLL, up to 20% of relapses are characterized by missense mutations targeting the C-terminal tail of RPS15. This tail plays a key structural role in the ribosome by extending into the interface between the ribosomal tRNA accommodation (A) site and the peptidyl-transferase (P) site. However, the impact of CLL-associated RPS15 mutations on translation dysregulation remains elusive.

### Aims

We aimed to compare the effects of the most recurrent cancer-associated RP mutations on the cellular translatome (i.e. the spectrum of proteins translated by the ribosome). Furthermore, we focused on RPS15 mutations in CLL to study how these defects remodel ribosome structure and translation dynamics, and to gain deep mechanistic insights in how these mutations alter the cellular translatome and could play a role in CLL pathogenesis.

### Methods

We utilized CRISPR-Cas9 technology to engineer an isogenic RiboCancer cell line library accurately modeling the most recurrent RP defects in blood and solid cancers (Rpl5$^{+/-}$, Rpl11$^{+/-}$, Rpl22$^{+/-}$, Rpl22$^{-/-}$, Rpl10 R98S, Rps15 P131S and Rps15 H137Y). This entire library was characterized by a multi-omics translatome analysis (quantitative proteomics, Ribo-seq (sequencing of ribosome protected RNA) and total RNA-seq) as well as by RiboMethSeq (profiling of 2'-O-methylation of ribosomal RNA). Furthermore, we characterized the structure and elongation dynamics of Rps15-mutant ribosomes by cryo-electron microscopy (cryo-EM) and biochemical follow-up experiments.

**Copyright Information:** (Online) ISSN: 2572-9241
©2026 The Author(s). HemaSphere is published by John Wiley & Sons Ltd on behalf of European Hematology Association. This is an open access Abstract Book distributed under the Attribution-NonCommercial-NoDerivs (CC BY-NC-ND), which allows third parties to download the articles and share them with others as long as they credit the author and the Abstract Book, but they cannot change the content in any way or use them commercially.
**Abstract Book Citations:** Authors, Title, HemaSphere, 2026;10:(S1):pages. The abstract book DOIs can be found on the "Author Information" page.
**Disclaimer:** Articles published in the journal HemaSphere exclusively reflect the opinions of the authors. The authors are responsible for all content in their abstracts including accuracy of the facts, statements, citing resources, etc.

### Results

Within the RiboCancer panel, RP knock-out had minimal effects on translation, whereas RP point mutations induced a significant number of translation efficiency changes, affecting up to 10% of expressed genes for CLL-associated Rps15 mutations. Cryo-EM and biochemical analyses revealed that these mutations destabilize the Rps15 C-terminus and affect the translation elongation cycle dynamics by deregulating accommodation of aminoacylated tRNAs at the ribosomal A-site. Using Ribo-seq and translation reporter assays, we demonstrate that this accommodation defect selectively affects 11 codons, explaining the reduced translation efficiency of genes with high presence of these codons in Rps15 mutant cells. Notably, these translationally dysregulated genes were strongly enriched for epigenetic and transcriptional regulators such as transcription factor Runx3, resulting in downregulation of Runx3 as well as many of its target genes involved in immune regulation in RPS15-mutant cells.

### Summary/Conclusion

By developing and characterizing a unique RiboCancer cell line panel, we mapped translational rewiring driven by the most frequent somatic RP mutations. We provide unprecedented mechanistic insights into translation defects induced by CLL-associated Rps15 mutations, and reveal an intriguing translation-based rewiring of epigenetics and transcription in CLL.

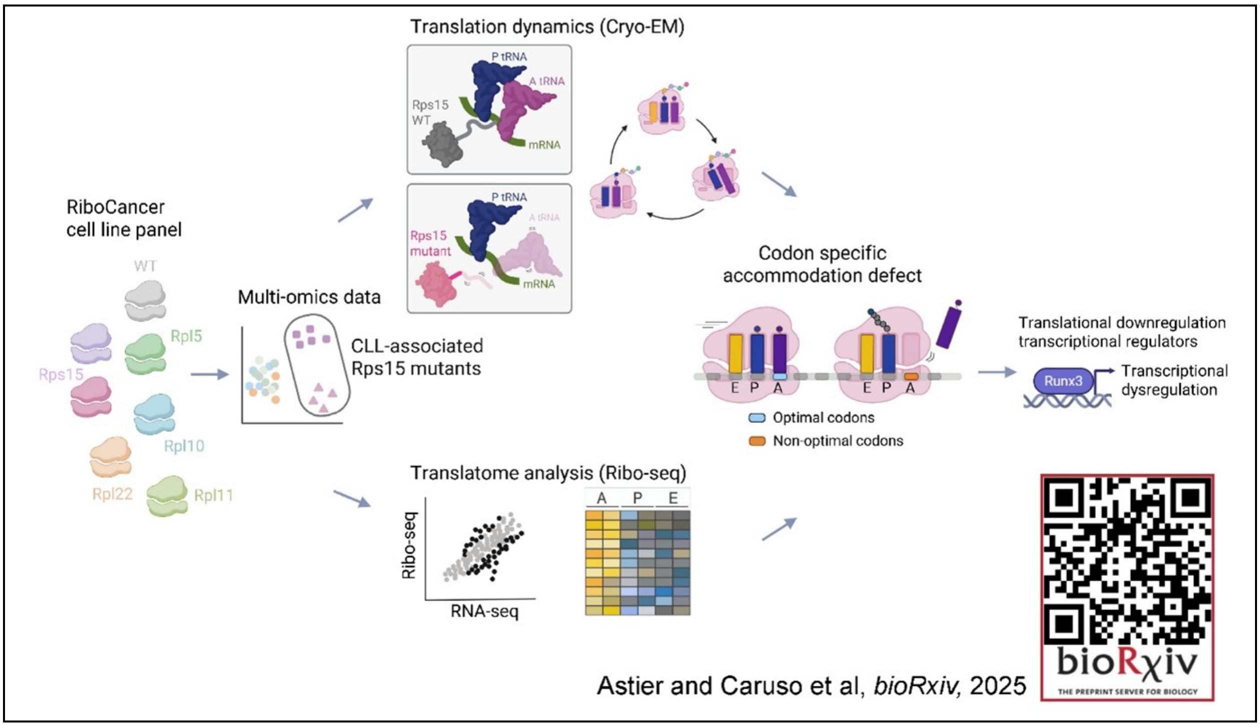

Copyright Information: (Online) ISSN: 2572-9241
©2026 The Author(s). HemaSphere is published by John Wiley & Sons Ltd on behalf of European Hematology Association. This is an open access Abstract Book distributed under the Attribution-NonCommercial-NoDerivs (CC BY-NC-ND), which allows third parties to download the articles and share them with others as long as they credit the author and the Abstract Book, but they cannot change the content in any way or use them commercially.
Abstract Book Citations: Authors, Title, HemaSphere, 2026;10:(S1):pages. The abstract book DOIs can be found on the “Author Information” page.
Disclaimer: Articles published in the journal HemaSphere exclusively reflect the opinions of the authors. The authors are responsible for all content in their abstracts including accuracy of the facts, statements, citing resources, etc.

### (S145) FIRST-LINE TREATMENT OF CLL/SLL WITH THE ALL-ORAL COMBINATION OF SONROTOCLAX AND ZANUBRUTINIB ACHIEVES UNDETECTABLE MINIMAL RESIDUAL DISEASE RATES OF >90%, INCLUDING IN PATIENTS WITH DEL(17P)/TP53

**Topic:** 06. Chronic lymphocytic leukemia and related disorders - Clinical

Chan Y. Cheah*[1, 2, 3], Stephen Opat[4], Masa Lasica[5], Peter Browett[6], Mary Anderson[7, 8], Emma Verner[9, 10], Shuo MA[11], Robert Weinkove[12, 13], Marc Hoffmann[14], Jacob Soumerai[15], Mazyar Shadman[16, 17], Talha Munir[18], Sophie Leitch[19], Raul Cordoba[20], David Westerman[21, 22], Paolo Ghia[23, 24], Binghao Wu[25], Gabriel Man[25], Yiqian Fang[25], Sheel Patel[25], Remus Vezan[25], Constantine S. Tam[26]

*[1]Sir Charles Gairdner Hospital, Nedlands, WA, Australia;[2]University of Western Australia, Crawley, WA, Australia;[3]Linear Clinical Research, Nedlands, WA, Australia; [4]Lymphoma Research Group, School of Clinical Sciences at Monash Health, Monash University,, Clayton, Australia; [5]St Vincent's Hospital Melbourne, Melbourne, Australia; [6]Auckland City Hospital, Auckland, New Zealand; [7]Royal Melbourne Hospital and Peter MacCallum Cancer Centre, Melbourne, Australia;[8]The Walter and Eliza Hall Institute, Melbourne, Australia; [9]Concord Repatriation General Hospital, Concord, Australia;[10]University of Sydney, Sydney, Australia; [11]Robert H. Lurie Comprehensive Cancer Center, Northwestern University Feinberg School of Medicine, Chicago, IL, United States of America; [12]Te Rerenga Ora Blood and Cancer Centre, Te Whatu Ora Health New Zealand Capital Coast & Hutt Valley, Wellington, New Zealand; [13]Malaghan Institute of Medical Research, Wellington, New Zealand;[14]University of Kansas Cancer Center, Kansas City, KS, United States of America; [15]Mass General Brigham Cancer Institute and Harvard Medical School, Boston, MA, United States of America; [16]Fred Hutchinson Cancer Center, Seattle, WA, United States of America; [17]University of Washington, Seattle, WA, United States of America;[18]Leeds Teaching Hospitals NHS Trust, Leeds, United Kingdom; [19]Te Whatu Ora Health New Zealand, Auckland, New Zealand;[20]Hospital Universitario Fundación Jiménez Díaz, Madrid, Spain; [21]Peter MacCallum Cancer Centre, Melbourne, Australia;[22]University of Melbourne, Melbourne, Australia; [23]Università Vita-Salute San Raffaele, Milano, Italy; [24]Comprehensive Cancer Center, IRCCS Ospedale San Raffaele, Milano, Italy; [25]BeOne Medicines, Ltd, San Carlos, CA, United States of America;[26]Alfred Hospital and Monash University, Melbourne, Australia;*

### Background

Oral, fixed-duration BTKi-BCL2i combinations are effective and convenient options for first-line treatment of CLL. However, current regimens, including those using second-generation BTKi, are limited by low uMRD rates. We hypothesize that upgrading the BCL2i component of BTKi-BCL2i with sonrotoclax, a next generation BCL2i with ~14x pharmacological potency compared with venetoclax, may lead to improved uMRD rates.

### Aims

To report updated safety and efficacy results in patients with treatment-naive CLL/SLL treated with zanubrutinib + sonrotoclax (320 mg cohort) in a phase 1/1b study (NCT04277637), with a median follow-up of 30.9 months (range, 3.1-41.9 months).

### Methods

Study treatment consisted of lead-in zanubrutinib 320 mg QD for 8-12 weeks, after which sonrotoclax was added with a ramp-up to the target dose of 320 mg QD. Patients were treated until progression, unacceptable toxicity, or protocol-defined elective discontinuation after 96 weeks of combination treatment at target dose. Primary endpoint was safety per NCI-CTCAE v5.0. Secondary endpoints included overall response rate (ORR). Exploratory endpoints included uMRD4 rates in peripheral blood per flow cytometry and next generation-sequencing (NGS)

**Copyright Information:** (Online) ISSN: 2572-9241
©2026 The Author(s). HemaSphere is published by John Wiley & Sons Ltd on behalf of European Hematology Association. This is an open access Abstract Book distributed under the Attribution-NonCommercial-NoDerivs (CC BY-NC-ND), which allows third parties to download the articles and share them with others as long as they credit the author and the Abstract Book, but they cannot change the content in any way or use them commercially.
**Abstract Book Citations:** Authors, Title, HemaSphere, 2026;10:(S1):pages. The abstract book DOIs can be found on the "Author Information" page.
**Disclaimer:** Articles published in the journal HemaSphere exclusively reflect the opinions of the authors. The authors are responsible for all content in their abstracts including accuracy of the facts, statements, citing resources, etc.

**Results**

Of the 86 patients enrolled in the sonrotoclax 320 mg cohort, 45 (52%) remained on treatment at data cut-off, and 40 (47%) discontinued sonrotoclax, most due to protocol-defined elective discontinuation (85%; n=34). The most common (≥30%) any-grade treatment-emergent adverse events (TEAEs) were neutropenia (38%), contusion (38%), COVID-19 (33%), and upper respiratory tract infection (30%). Neutropenia was the most common grade ≥3 TEAE (29%). No TLS occurred, and no AEs led to death.
In 84 efficacy-evaluable patients, the ORR was 100%, with 46 (55%) achieving complete response. Median time to response was 2.6 months (range, 1.5-9.1 months). No patients in the 320 mg cohort experienced progression with an estimated 30-month PFS of 100%.
The best uMRD4 rate assessed by flow cytometry was 99% (83/84) and 100% (10/10) in patients with *TP53*/del(17p) mutations. Median time from reaching sonrotoclax target dose to uMRD was 3.0 months (range, 2.3-27.4 months), and the best uMRD4 rates by weeks 24, 48 and 96 were 81% (69/85), 91% (63/69) and 98% (55/56), respectively. No patient with uMRD4 reverted to MRD4+. In the NGS evaluable set, best uMRD5 ($<10^{-5}$) was 86% (66/77). Updated results will be presented.

**Summary/Conclusion**

Sonrotoclax + zanubrutinib was well tolerated and achieved uMRD rates of >90%, including in patients with high-risk cytogenetics. The depth and kinetics of uMRD achieved with sonrotoclax + zanubrutinib highlights the improved potency and differentiated profile of this combination versus available combination therapies in first-line CLL. Sonrotoclax + zanubrutinib is being evaluated in two phase 3 clinical trials (NCT06073821, NCT07277231).

**Copyright Information:** (Online) ISSN: 2572-9241
©2026 The Author(s). HemaSphere is published by John Wiley & Sons Ltd on behalf of European Hematology Association. This is an open access Abstract Book distributed under the Attribution-NonCommercial-NoDerivs (CC BY-NC-ND), which allows third parties to download the articles and share them with others as long as they credit the author and the Abstract Book, but they cannot change the content in any way or use them commercially.
**Abstract Book Citations:** Authors, Title, HemaSphere, 2026;10:(S1):pages. The abstract book DOIs can be found on the “Author Information” page.
**Disclaimer:** Articles published in the journal HemaSphere exclusively reflect the opinions of the authors. The authors are responsible for all content in their abstracts including accuracy of the facts, statements, citing resources, etc.

### (S146) VENETOCLAX-OBINUTUZUMAB FOR PREVIOUSLY UNTREATED CHRONIC LYMPHOCYTIC LEUKEMIA: FINAL RESULTS OF THE RANDOMIZED CLL14 STUDY

**Topic:** 06. Chronic lymphocytic leukemia and related disorders - Clinical

Kirsten Fischer*[1], Othman Al-Sawaf[1], Sandra Robrecht[1], Adam Giza[1], Can Zhang[1], Anna-Maria Fink[1], Christof Schneider[2], Karl-Anton Kreuzer[1], Tatiana Chagorova[3], Liliya Sivcheva[4], Olga Samoilova[5], Stephen Opat[6], Carsten Niemann[7], Igor Aurer[8], Lachezar Bogdanov[9], Anthony Schwarer[10], Christian Bjørn Poulsen[11], Andrew Butler[12], Javier Loscertales Pueyo[13], Carolyn Owen[14], Emil Ankov Spasov[15], Clemens Wendtner[16], Anja Wrisch[17], Bryan Murray[17], Madlaina Breuleux[18], Yanwen Jiang[19], Hyun Yong Jin[19], Eugen Tausch[2], Anke Schilhabel[20], Matthias Ritgen[20], Stephan Stilgenbauer[2], Barbara Eichhorst[1], Michael Hallek[1]

*[1]University Hospital of Cologne, Department I of Internal Medicine; German CLL Study Group, Cologne, Germany;[2]University Hospital Ulm, Division of CLL, Department III of Internal Medicine, Ulm, Germany; [3]Penza Regional Oncology Dispensary, Penza, Russia; [4]Hristo Botev Multiprofile Hospital for Active Treatment, First Department of Internal Medicine, Vratsa, Bulgaria;[5]Regional Clinical Hospital NA Semashko, Nizhny Novgorod, Russia; [6]School of Clinical Sciences at Monash Health, Monash University, Lymphoma Research Group, Clayton, Australia; [7]Rigshospitalet, Copenhagen University Hospital, Copenhagen, Denmark; [8]University Hospital Centre Zagreb and School of Medicine, Zagreb, Croatia;[9]University Hospital Pleven, Clinic of Hematology, Pleven, Bulgaria; [10]Box Hill Hospital, Department of Haematology, Melbourne, Australia; [11]Zealand University Hospital, Roskilde, Denmark; [12]Christchurch Hospital, Department of Haematology, Christchurch, New Zealand; [13]Hospital De La Princesa, Madrid, Spain; [14]University of Calgary, Calgary, Canada; [15]UMHAT St George and Medical University Plovdiv, Clinic of Hematology, Plovdiv, Bulgaria; [16]Ludwig-Maximilians University (LMU), Medical Clinic III, Munich, Germany; [17]AbbVie Inc., Chicago, United States of America; [18]F. Hoffmann-La Roche Ltd, Basel, Switzerland; [19]Genentech Inc., San Francisco, United States of America; [20]Universitätsklinikum Schleswig-Holstein, Campus Kiel, Kiel, Germany;*

### Background

One-year fixed-duration venetoclax-obinutuzumab (Ven-Obi) is a standard-of-care for patients (pts) with previously untreated chronic lymphocytic leukemia (CLL), first introduced by the randomised CLL14 trial (Fischer K et al. 2019 NEJM). The final results of CLL14 provide new insights into long-term outcomes following Ven-Obi nearly a decade post treatment.

### Methods

Pts with previously untreated CLL and coexisting conditions were randomized 1:1 to Ven-Obi or chlorambucil-obinutuzumab (Clb-Obi). All pts are off study treatment for ≥ 8 years. The primary endpoint was investigator-assessed progression-free survival (PFS). Secondary endpoints included time to next treatment (TTNT) and overall survival (OS), rates of minimal residual disease (MRD), and safety. All reported p values are considered exploratory without adjustment for multiple testing.

**Copyright Information:** (Online) ISSN: 2572-9241
©2026 The Author(s). HemaSphere is published by John Wiley & Sons Ltd on behalf of European Hematology Association. This is an open access Abstract Book distributed under the Attribution-NonCommercial-NoDerivs (CC BY-NC-ND), which allows third parties to download the articles and share them with others as long as they credit the author and the Abstract Book, but they cannot change the content in any way or use them commercially.
**Abstract Book Citations:** Authors, Title, HemaSphere, 2026;10:(S1):pages. The abstract book DOIs can be found on the "Author Information" page.
**Disclaimer:** Articles published in the journal HemaSphere exclusively reflect the opinions of the authors. The authors are responsible for all content in their abstracts including accuracy of the facts, statements, citing resources, etc.

**Results**

Of 432 enrolled pts, 216 were randomly assigned to Ven-Obi, 216 to Clb-Obi. After a median follow-up of 110.1 months (interquartile range 99.8-113.0), the median age at the end of observation was 79 years. Progressive disease (PD) occurred in 87 pts in the Ven-Obi group compared to 144 pts in the Clb-Obi group, resulting in superior PFS for Ven-Obi (median 76.6 vs 37.9 months, HR 0.50 [95% CI 0.39-0.63], p<0.001).Time to next treatment (TTNT) was significantly prolonged with Ven-Obi compared to Clb-Obi (median, 91.9 vs. 52.5 months; HR 0.54 [0.43-0.70], p<0.001), with 54 and 111 second-line treatments initiated in the respective groups. In both groups, the most frequent second-line treatments were BTK inhibitors (53.7% and 55.0%, respectively).In the Ven-Obi group, pts with unmutated IGHV had a median PFS of 64.7 months compared to 104.9 months in pts with mutated IGHV (HR 2.74 [1.85-4.06], p<0.001), and a median TTNT of 78.6 months vs not reached (HR 2.59 [1.68-3.99], p<0.001). Pts with *TP53* mutation/deletion had a median PFS of 49.0 months compared to 79.2 months in pts without *TP53* mutation/deletion (HR 2.15 [1.36-3.40], p=0.001), and a median TTNT of 57.3 months vs 98.3 months (HR 2.35 [1.46-3.78], p<0.001). Multivariable analysis identified deletion 17p and unmutated IGHV status as independent adverse prognostic factors for PFS in the Ven-Obi group. Six years after treatment completion, 22 pts (10.2% of the intention-to-treat population) in the Ven-Obi group had uMRD (<10-4 by NGS in peripheral blood), 16 (7.4%) had low (L)-MRD (≥ 10-4 and < 10-2) and 27 (12.5%) high (H)-MRD (≥ 10-2), compared to 6 (2.8%) uMRD, 5 (2.3%) L-MRD and 16 (7.4%) H-MRD in the Clb-Obi group. Overall, 78 deaths were reported in the Ven-Obi group (14 PD related) and 92 in the Clb-Obi group (30 PD related); the median OS was not reached in the Ven-Obi and 112.7 months in the Clb-Obi group (HR 0.80 [0.59-1.09], p=0.161). Second primary malignancies excluding non-melanoma skin cancers were reported in 41 pts (19.3%) in the Ven-Obi group and 24 (11.2%) in the Clb-Obi group, including 10 (4.7%) and 8 pts (3.7%) with fatal cases. The follow-up adjusted incidence rates were 2.6 and 1.5 cases per 1000 patient-months, respectively (p=0.047). No further safety signals were observed.

**Summary/Conclusion**

After a median follow-up of 9 years, the final results of the CLL14 study confirm the long-term efficacy of one-year fixed-duration Ven-Obi in previously untreated pts with CLL and coexisting conditions, with a median progression free survival of over 6 years and a median time to next treatment approaching 8 years.

**Copyright Information:** (Online) ISSN: 2572-9241
©2026 The Author(s). HemaSphere is published by John Wiley & Sons Ltd on behalf of European Hematology Association. This is an open access Abstract Book distributed under the Attribution-NonCommercial-NoDerivs (CC BY-NC-ND), which allows third parties to download the articles and share them with others as long as they credit the author and the Abstract Book, but they cannot change the content in any way or use them commercially.
**Abstract Book Citations:** Authors, Title, HemaSphere, 2026;10:(S1):pages. The abstract book DOIs can be found on the “Author Information” page.
**Disclaimer:** Articles published in the journal HemaSphere exclusively reflect the opinions of the authors. The authors are responsible for all content in their abstracts including accuracy of the facts, statements, citing resources, etc.

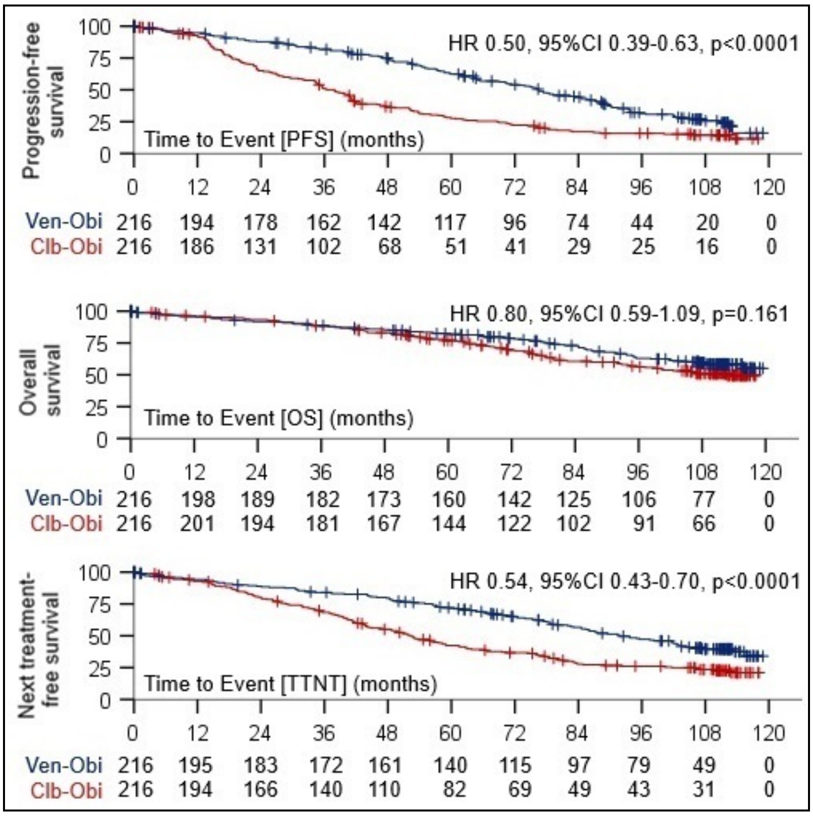

Kaplan–Meier Estimates of PFS, OS, and TTNT for Ven-Obi versus Clb-Obi. PFS, progression-free survival; OS, overall survival; TTNT, time to next treatment; HR, hazard ratio; CI, confidence interval.

**Copyright Information:** (Online) ISSN: 2572-9241
©2026 The Author(s). HemaSphere is published by John Wiley & Sons Ltd on behalf of European Hematology Association. This is an open access Abstract Book distributed under the Attribution-NonCommercial-NoDerivs (CC BY-NC-ND), which allows third parties to download the articles and share them with others as long as they credit the author and the Abstract Book, but they cannot change the content in any way or use them commercially.
**Abstract Book Citations:** Authors, Title, HemaSphere, 2026;10:(S1):pages. The abstract book DOIs can be found on the "Author Information" page.
**Disclaimer:** Articles published in the journal HemaSphere exclusively reflect the opinions of the authors. The authors are responsible for all content in their abstracts including accuracy of the facts, statements, citing resources, etc.

### (S147) OXPLORED: A MULTICENTRE UK-WIDE OBSERVATIONAL STUDY TO IDENTIFY AND PROSPECTIVELY VALIDATE PREDICTORS OF DISEASE PROGRESSION IN CHRONIC LYMPHOCYTIC LEUKAEMIA (CLL) AND MBL

**Topic:** 06. Chronic lymphocytic leukemia and related disorders - Clinical

Danmei Xu*[1, 2], Niamh Appleby[1, 2], Renata Walewska[3], Andrew Pettitt[4], Piers Patten[5], Oliver Miles[6], Helen Marr[7], Claire Hutchinson[8], Nicolas Martinez-Calle[9], Scott Marshall[10], Simon Stern[11], Toby A. Eyre[2], Murali Kesavan[2], Harriet Walter[12], Wendy Harrison[13], Charlotte Bomken[14], Yasmin Reyal[15], Savio Fernandes[16], Melinda Kormany[1], Georgeta Ciuban[1], Pauline Robbe[1], Kate Ridout[1], Timothy Coutts[17], Anthony Cutts[1], Helene Dreau[1], Dimitris Vavoulis[1, 18], Grigore Gafencu[1], Anna Schuh[1, 2, 18]

*[1]Molecular diagnostics Centre, University of Oxford, Oxford, United Kingdom;[2]Oxford University Hospitals NHS Trust, Oxford, United Kingdom; [3]University Hospitals Dorset, Bournemouth, United Kingdom;[4]The Clatterbridge Cancer Centre, Liverpool, United Kingdom; [5]King's College London, London, United Kingdom; [6]Musgrove Park Hospital, Somerset, United Kingdom; [7]Newcastle Hospitals NHS Foundation Trust, Newcastle, United Kingdom;[8]University Hospitals Plymouth NHS Trust, Plymouth, United Kingdom; [9]Nottingham City Hospital, Nottingham, United Kingdom; [10]Sunderland Royal Hospital, Sunderland, United Kingdom; [11]Epsom & St Helier Hospital NHS Trust, London, United Kingdom;[12]Leicester Royal Infirmary, Leicester, United Kingdom; [13]James Paget Hospital, Great Yarmouth, United Kingdom;[14]North Tyneside General hospital, Northumbria NHS Trust, Northumbria, United Kingdom; [15]St George's University Hospital, London, United Kingdom; [16]Russells Hall Hospital, Dudley, United Kingdom; [17]OCTO, department of Oncology, University of Oxford, Oxford, United Kingdom;[18]NIHR Oxford Biomedical Research Centre, Oxford, United Kingdom;*

### Background

Early-stage CLL and MBL are clinically heterogeneous, with variable risks of progression to symptomatic disease requiring treatment. Accurate prognostication is crucial to optimize surveillance and identify candidates for early-intervention trials. International Prognostic Score for early CLL (IPS-E) was developed using three variables: unmutated IGHV, absolute lymphocyte count >15 × 10^9/L, and presence of palpable lymphadenopathy, based on large retrospective international datasets (*Condoluci et al Blood, 2020*).

### Aims

Prospective validation of the IPS-E in early CLL and association with clinically meaningful endpoints such as infection, secondary malignancy and patient-reported quality of life.

### Methods

We prospectively enrolled 401 asymptomatic patients with Rai 0-1/Binet A CLL and high-count MBL across multiple UK centers. Baseline assessments included clinical, cytogenetic, molecular, and patient-reported quality-of-life (QoL) measures. Follow-up visits were scheduled at 24 months and either 36 or 60 months, depending on recruitment date. Patients consenting to additional procedures had longitudinal samples collected for whole genome sequencing (WGS), total RNAseq  and single cell analyses using CITE-Seq.

**Copyright Information:** (Online) ISSN: 2572-9241
©2026 The Author(s). HemaSphere is published by John Wiley & Sons Ltd on behalf of European Hematology Association. This is an open access Abstract Book distributed under the Attribution-NonCommercial-NoDerivs (CC BY-NC-ND), which allows third parties to download the articles and share them with others as long as they credit the author and the Abstract Book, but they cannot change the content in any way or use them commercially.
**Abstract Book Citations:** Authors, Title, HemaSphere, 2026;10:(S1):pages. The abstract book DOIs can be found on the "Author Information" page.
**Disclaimer:** Articles published in the journal HemaSphere exclusively reflect the opinions of the authors. The authors are responsible for all content in their abstracts including accuracy of the facts, statements, citing resources, etc.

### Results

The study is the first to prospectively validate the IPS-E in a MBL/Binet A CLL cohort (Figure 1A). Comparing patients with high versus low IPS-E confirmed the higher risk of clinical progression to requiring treatment (HR 67.1, 95% confidence interval (CI): 9.24-487, Wald test p-value: <0.001), with a similar association observed between patients with intermediate versus low IPS-E (HR 16.2, 95%CI: 2.16-121, Wald test p-value: 0.007) (Figure 1D). Moreover, at 60 months of follow-up, the Kaplan-Meier estimate of non-progression probability was 99%(95%CI: 98-100%) for IPS-E low patients, 74%(95%CI: 60-92%) for intermediate and 53% (95%CI: 43-65%) for IPS-E high (Figure 1B-C). Associations of IPS-E with WGS, T cell profiling and immunogenetics will be presented separately.

### Summary/Conclusion

This study provides prospective evidence for an accessible prognostic framework in Binet A CLL and MBL, linking molecular and patient-centred factors to disease progression. Results are expected to inform individualized surveillance and enable early-intervention trial design.

A.

| Characteristic | N = 401[1] |
|---|---|
| Age | |
| <65 yo | 160 (39.9%) |
| ≥65 yo | 241 (60.1%) |
| Gender | |
| Female | 157 (39.2%) |
| Male | 244 (60.8%) |
| Follow-up (months) | 36.097 (0.871, 74.967) |
| Palpable lymph nodes | 117 (29.2%) |
| Palpable spleen | 7 (1.7%) |
| Lymphocytes >15×10^9/L | 157 (39.2%) |
| Hb <13 g/dL (male)/<12 g/dL (female) | 52 (13.0%) |
| Platelets <150×10^9/L | 48 (12.0%) |
| B2M >3.5 mg/L | 31 (8.6%) |
| Unknown | 41 |
| IGHV status | |
| mutated IGHV | 267 (66.6%) |
| mutated IGHV subset #2 | 5 (1.2%) |
| unmutated IGHV | 129 (32.2%) |
| IPS-E classification | |
| Low | 130 (32.4%) |
| Intermediate | 159 (39.7%) |
| High | 112 (27.9%) |
| MBL/Binet A | |
| MBL | 125 (31.2%) |
| Binet A | 276 (68.8%) |

[1]n (%); Median (Min, Max)

B.

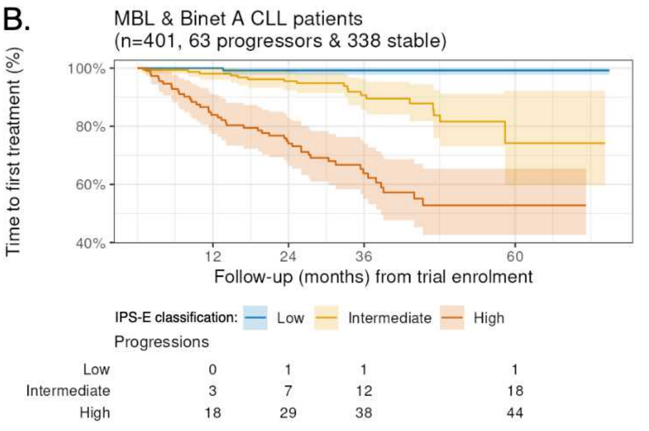

C. Kaplan-Meier estimate with 95%CI of non-progression probability

| Characteristic | 12 months | 24 months | 36 months | 60 months |
|---|---|---|---|---|
| IPS-E classification | | | | |
| Low | 100% (100%, 100%) | 99% (98%, 100%) | 99% (98%, 100%) | 99% (98%, 100%) |
| Intermediate | 98% (96%, 100%) | 96% (92%, 99%) | 91% (86%, 96%) | 74% (60%, 92%) |
| High | 84% (77%, 91%) | 74% (66%, 83%) | 64% (55%, 74%) | 53% (43%, 65%) |

D. Cox regression & Wald test results

| Characteristic | HR | 95% CI | p-value |
|---|---|---|---|
| IPS-E classification | | | |
| Low | — | — | |
| Intermediate | 16.2 | 2.16, 121 | 0.007 |
| High | 67.1 | 9.24, 487 | <0.001 |

| Characteristic | HR | 95% CI | p-value |
|---|---|---|---|
| IPS-E classification | | | |
| High | — | — | |
| Intermediate | 0.24 | 0.14, 0.42 | <0.001 |
| Low | 0.01 | 0.00, 0.11 | <0.001 |

Abbreviations: CI = Confidence Interval, HR = Hazard Ratio

Copyright Information: (Online) ISSN: 2572-9241
©2026 The Author(s). HemaSphere is published by John Wiley & Sons Ltd on behalf of European Hematology Association. This is an open access Abstract Book distributed under the Attribution-NonCommercial-NoDerivs (CC BY-NC-ND), which allows third parties to download the articles and share them with others as long as they credit the author and the Abstract Book, but they cannot change the content in any way or use them commercially.
Abstract Book Citations: Authors, Title, HemaSphere, 2026;10:(S1):pages. The abstract book DOIs can be found on the "Author Information" page.
Disclaimer: Articles published in the journal HemaSphere exclusively reflect the opinions of the authors. The authors are responsible for all content in their abstracts including accuracy of the facts, statements, citing resources, etc.

### (S148) A PHASE 2 STUDY OF FIXED-DURATION PIRTOBRUTINIB AND OBINUTUZUMAB IN PREVIOUSLY UNTREATED CLL

**Topic:** 06. Chronic lymphocytic leukemia and related disorders - Clinical

Inhye E. Ahn*[1], Svitlana Tyekucheva[2], Andrew Lipsky[3], Andres Chang[4], Jack Suher[1], Hang Phan[1], Virginia Cyr[1], Ijaabo Ali[1], Philippe Armand[1], Amy Bessnow[1], Jennifer L. Crombie[1], David Fisher[1], Cynthia Hahn[1], Eric D. Jacobsen[1], Caron Jacobson[1], Austin I. Kim[1], Ann Lacasce[1], Reid Merryman[1], Oreofe Odejide[1], Erin Parry[1], David Qualls[1], Christine Ryan[1], Aswin Sekar[1], Olivier Clerc[5], Sarah Cuddy[5], Nancy Kaddis[6], Jon Arnason[7], John Winters[8], Steven Lo[9], Jennifer R. Brown[1], Matthew Davids[1]

*[1]Dana-Farber Cancer Institute, Department of Medical Oncology, Boston, United States of America;[2]Dana-Farber Cancer Institute, Department of Data Science, Boston, United States of America; [3]Herbert Irving Comprehensive Cancer Center, Columbia University Medical Center, New York, United States of America; [4]Winship Cancer Institute of Emory University, Department of Hematology and Medical Oncology, Atlanta, United States of America; [5]Brigham and Women's Hospital, Department of Medicine, Boston, United States of America; [6]Dana-Farber Cancer Institute at South Shore Health, Weymouth, United States of America; [7]Beth Israel Deaconess Medical Center, Boston, United States of America;[8]New England Cancer Specialists, Scarborough, United States of America; [9]Bennett Cancer Center, Stamford Health, Stamford, United States of America;*

### Background

Limited prospective data exist for a fixed-duration BTK inhibitor and anti-CD20 monoclonal antibody regimen without a BCL2 inhibitor.

### Aims

We present for the first time interim efficacy and safety data from a phase 2 study of pirtobrutinib and obinutuzumab in patients (pts) with treatment-naïve (TN) CLL (NCT06333262).

### Methods

This investigator-sponsored study tested 12 cycles of pirtobrutinib (from cycle [C] 1 through 12) and delayed initiation of obinutuzumab (from C6 through 12) in TN CLL pts meeting iwCLL treatment indications. All pts stopped therapy regardless of response or minimal residual disease (MRD) status at the end of combination therapy (EoCT) after C12. The primary endpoint was the rate of complete response (CR) 3 months after EoCT. Under the optional cardiac substudy, we performed home blood pressure monitoring, 30-day ambulatory telemetry, and cardiac MRI at pretreatment and after C6.

**Copyright Information:** (Online) ISSN: 2572-9241
©2026 The Author(s). HemaSphere is published by John Wiley & Sons Ltd on behalf of European Hematology Association. This is an open access Abstract Book distributed under the Attribution-NonCommercial-NoDerivs (CC BY-NC-ND), which allows third parties to download the articles and share them with others as long as they credit the author and the Abstract Book, but they cannot change the content in any way or use them commercially.
**Abstract Book Citations:** Authors, Title, HemaSphere, 2026;10:(S1):pages. The abstract book DOIs can be found on the "Author Information" page.
**Disclaimer:** Articles published in the journal HemaSphere exclusively reflect the opinions of the authors. The authors are responsible for all content in their abstracts including accuracy of the facts, statements, citing resources, etc.

**Results**

As of February 2026, 68 pts have been enrolled from 7 collaborating sites in the United States, including 60 in an initial cohort (fully accrued) and 8 in an expansion cohort (ongoing accrual with 30 pts total planned). Median age is 65 years. 63% are male, 56% has unmutated IGHV, and 10% has deletion 17p or *TP53* mutation.
Of 60 pts in the initial cohort, 20% have completed EoCT response assessment, 52% await EoCT response assessment after completing C12, and 22% are still on active therapy. Four (7%) pts went off study (n=2 progressive disease, n=1 withdrew consent, n=1 to receive systemic therapy for pre-existing glomerulonephritis). Median duration on study was 11 months at data cutoff.
The most common treatment-emergent adverse event was bruising (30%, all grade [G] 1-2), followed by headache (22%, all G1-2). Neutropenia and thrombocytopenia occurred in 20% (5% G3-4) and 13% (5% G3-4), respectively. We observed low rates of serious infection (5% or 3 pts with G3-4) and infusion-related reaction (2% or 1 pt with G2), possibly due to the delayed initiation of obinutuzumab. No pt had febrile neutropenia. There were no treatment-related adverse events leading to early treatment discontinuation or dose reduction.
The overall response rate (ORR) after C6 was 75% in an intent-to-treat analysis of 60 pts and 78% in 58 evaluable pts with response assessment. The ORR after EoCT was 100% (25% CR) in 12 evaluable pts. The rate of undetectable MRD in blood based on flow cytometry ($<10^{-4}$) was 3% after C6 (n=1/40) and 52% after EoCT (n=13/25). Two progressed from Richter's transformation at 5 and 11 months on study. None had progressive CLL or died.
We collected detailed cardiac data on a 25 pt substudy at pretreatment and after C6. Treatment-emergent hypertension was uncommon (7%, all G1-2). One pt with a remote history of atrial fibrillation (Afib) developed symptomatic Afib on C1D16 and withdrew consent. There were no other treatment-emergent Afib or changes to the myocardium on MRI. Cardiac function, measured by left ventricular ejection fraction and global longitudinal strain, remained stable.

**Summary/Conclusion**

Our early data suggest that fixed-duration pirtobrutinib and obinutuzumab have a favorable safety profile and a high rate of response in TN CLL, including pts achieving CR and undetectable MRD at EoCT. Additional pts and longer follow-up are needed for further evaluation of the efficacy and durability of disease control after treatment cessation.

**Copyright Information:** (Online) ISSN: 2572-9241
©2026 The Author(s). HemaSphere is published by John Wiley & Sons Ltd on behalf of European Hematology Association. This is an open access Abstract Book distributed under the Attribution-NonCommercial-NoDerivs (CC BY-NC-ND), which allows third parties to download the articles and share them with others as long as they credit the author and the Abstract Book, but they cannot change the content in any way or use them commercially.
**Abstract Book Citations:** Authors, Title, HemaSphere, 2026;10:(S1):pages. The abstract book DOIs can be found on the "Author Information" page.
**Disclaimer:** Articles published in the journal HemaSphere exclusively reflect the opinions of the authors. The authors are responsible for all content in their abstracts including accuracy of the facts, statements, citing resources, etc.

### (S149) GENETIC BIOMARKERS PREDICTING SUSTAINED EFFICACY OF VENETOCLAX-BASED THERAPIES OR CIT IN CHRONIC LYMPHOCYTIC LEUKEMIA: FINAL 5-YEAR ANALYSIS OF THE GAIA/CLL13 TRIAL

**Topic:** 06. Chronic lymphocytic leukemia and related disorders - Clinical

Eugen Tausch*[1], Christof Schneider[1], Moritz Fürstenau[2], Sandra Robrecht[3], Adam Giza[3], Deyan Yosifov[1], Billy Chelliah Jebaraj[1], Christoph Brey[1], Tamar Tadmor[4], Patrick Thornton[5], Michael Gregor[6], Philipp Staber[7], Karl-Anton Kreuzer[2], Matthias Ritgen[8], Anna-Maria Fink[2], Kirsten Fischer[2], Arnon Philip Kater[9], Carsten Niemann[10], Michael Hallek[2], Barbara Eichhorst[2], Stephan Stilgenbauer[1]

*[1]Ulm University, Department of Internal Medicine III, Division of CLL, Ulm, Germany; [2]University of Cologne, Department I of Internal Medicine, Cologne, Germany; [3]German CLL Study Group, Cologne, Germany; [4]Bnai Zion medical center and the Ruth and Bruce Rappaport Faculty of Medicine, Technion,, Haifa, Israel; [5]Beaumont Hospital, Dublin, Ireland; [6]Kantonsspital, Luzern, Switzerland; [7]Saarland University Medical School, Homburg/Saar, Germany; [8]Universitaetsklinikum Schleswig-Holstein Campus Kiel, Kiel, Germany; [9]Amsterdam UMC, Amsterdam, The Netherlands; [10]Rigshospitalet, Copenhagen University Hospital, Copenhagen, Denmark;*

### Background

In CLL chemoimmunotherapy (CIT) efficacy is determined by genomic aberrations, gene mutations and IGHV mutation status. The role of these factors in time-limited venetoclax-based regimens is less well defined.

### Aims

We evaluated genetic biomarkers in the phase 3 GAIA/CLL13 trial, which compared fixed duration venetoclax combination with Rituximab (RV), Obinutuzumab (GV) or Obinutuzumab + Ibrutinib (GIV) versus CIT (FCR or BR) in fit, previously untreated CLL patients lacking del(17p) or *TP53* mutations determined by Sanger sequencing.

### Methods

Genomic aberrations were assessed by FISH, IGHV by sequencing with threshold of 98% identity. Mutations were assessed via targeted NGS for *TP53*, *NOTCH1*, *SF3B1*, *MYD88*, *KRAS*, *NRAS*, *BRAF*, *EGR2*, *NFKBIE*, *RPS15*, *XPO1*, *BIRC3*, *BTK*, *PLCG2* *and BCL2* in 913 of 926 patients (98.6%) at enrollment and in 235 of 328 CLL cases (71.6%) at progression (PD).

**Copyright Information:** (Online) ISSN: 2572-9241
©2026 The Author(s). HemaSphere is published by John Wiley & Sons Ltd on behalf of European Hematology Association. This is an open access Abstract Book distributed under the Attribution-NonCommercial-NoDerivs (CC BY-NC-ND), which allows third parties to download the articles and share them with others as long as they credit the author and the Abstract Book, but they cannot change the content in any way or use them commercially.
**Abstract Book Citations:** Authors, Title, HemaSphere, 2026;10:(S1):pages. The abstract book DOIs can be found on the “Author Information” page.
**Disclaimer:** Articles published in the journal HemaSphere exclusively reflect the opinions of the authors. The authors are responsible for all content in their abstracts including accuracy of the facts, statements, citing resources, etc.

**Results**

According to the hierarchical model, genomic aberrations occurred at the following rates at enrolment/PD: del(17p) 0%/5.7%, del(11q) 17.5%/25.3%, +12 16.3%/13.1%, del(13q) 44.6%/27.8%. Gene mutations with highest prevalence at enrolment/PD were detected in *SF3B1* 22.6%/26.0%; *NOTCH1* 20.5%/25.1%, *NFKBIE* 13.6%/10.2%, *BIRC3* 11.8%/11.5%, and *BRAF/NRAS/KRAS* 9.6%/13.2%. U-IGHV was present in 56%.
At a median follow-up of 63.8 months, univariable analysis in the pooled venetoclax treated patients (RV+GV+GIV) showed shorter PFS in the following subgroups: U-IGHV (HR2.3 [95%CI 1.74-3.05]), mutated *NOTCH1* (HR1.81 [1.36-2.39]), *BRAF/NRAS/KRAS* (HR1.72 [1.19-2.48]), *XPO1* (HR1.73 [1.19-2.53]) and *EGR2* (HR1.96 [1.27-3.01]). OS was significantly shorter for U-IGHV (HR2.08 [1.04-4.14]), +12 (HR2.08 [1.09-3.99]), mutated *BRAF/NRAS/KRAS (*HR 2.95 [1.45-5.99]), *BIRC3* (HR 2.64 [1.33-5.23]) and *NOTCH1* (HR 2.09 [1.12-3.90]). For CIT a shorter PFS/OS was observed with U-IGHV (PFS:HR 4.06 [2.53-6.52]; OS HR 3.0 [1.01-9.14]) and del(11q) (PFS: HR2.24 [1.46-3.45]; OS: HR3.92 [1.59-9.66]). *TP53* mutations missed by Sanger at screening showed a trend toward shorter PFS with CIT (HR 2.43 [0.87-6.77]), but not with RV+GV+GIV (HR1.11 [0.49-2.49]). Their prevalence increased from 2% at enrollment to 8.1% at PD (CIT 16.9%, RV+GV+GIV 4.3%).
In multivariable analysis U-IGHV (HR 2.01 [1.50-2.7]), complex karyotype (CKT [≥3 aberrations] vs. NCKT HR 1.93 [1.41-2.63], mutated *NOTCH1* (HR 1.56, [1.16-2.1]) and *EGR2* (HR 1.61 [1.03-2.52]) remained independent adverse prognostic factors for PFS for RV+GV+GIV. For CIT U-IGHV (HR 3.55 [2.20-5.75]) and CKT (HR 1.69 [1.09-2.61]) remained the only genetic independent prognostic markers.
In single-arm analysis, del(11q) patients achieved longer PFS with GV or GIV than with RV (HR 0.42 [0.24-0.74] and HR 0.16 [0.07-0.30]). Despite the superior overall PFS with GIV compared to GV no additional benefit from ibrutinib was observed in patients with mutated *BIRC3* (HR1.84 [0.74-4.59]), *BRAF/NRAS/KRAS* (HR 1.43 [.60-3.40]) and +12 (HR 1.29 [0.64-2.56]).
At PD 48 of 235 patients (20.4%) acquired new mutations (CIT 26.7%, RV+GV+GIV 17.7%, see Figure). We identified 3 acquired *BCL2* mutations in 2 patients (VAF 25%, 3%, 4%) in the RV arm, while none were found in GV/GIV. No*BTK* or *PLCG2* mutations were detected.

**Summary/Conclusion**

Beyond U-IGHV and CKT, *NOTCH1* and *EGR2* mutations were independent prognostic factors for PFS in 672 venetoclax treated CLL patients. Clonal evolution of del(17p)/TP53 mutation appeared more frequently in CIT than venetoclax; resistance mutations were rare (1%) supporting retreatment strategies.

**Copyright Information:** (Online) ISSN: 2572-9241
©2026 The Author(s). HemaSphere is published by John Wiley & Sons Ltd on behalf of European Hematology Association. This is an open access Abstract Book distributed under the Attribution-NonCommercial-NoDerivs (CC BY-NC-ND), which allows third parties to download the articles and share them with others as long as they credit the author and the Abstract Book, but they cannot change the content in any way or use them commercially.
**Abstract Book Citations:** Authors, Title, HemaSphere, 2026;10:(S1):pages. The abstract book DOIs can be found on the "Author Information" page.
**Disclaimer:** Articles published in the journal HemaSphere exclusively reflect the opinions of the authors. The authors are responsible for all content in their abstracts including accuracy of the facts, statements, citing resources, etc.

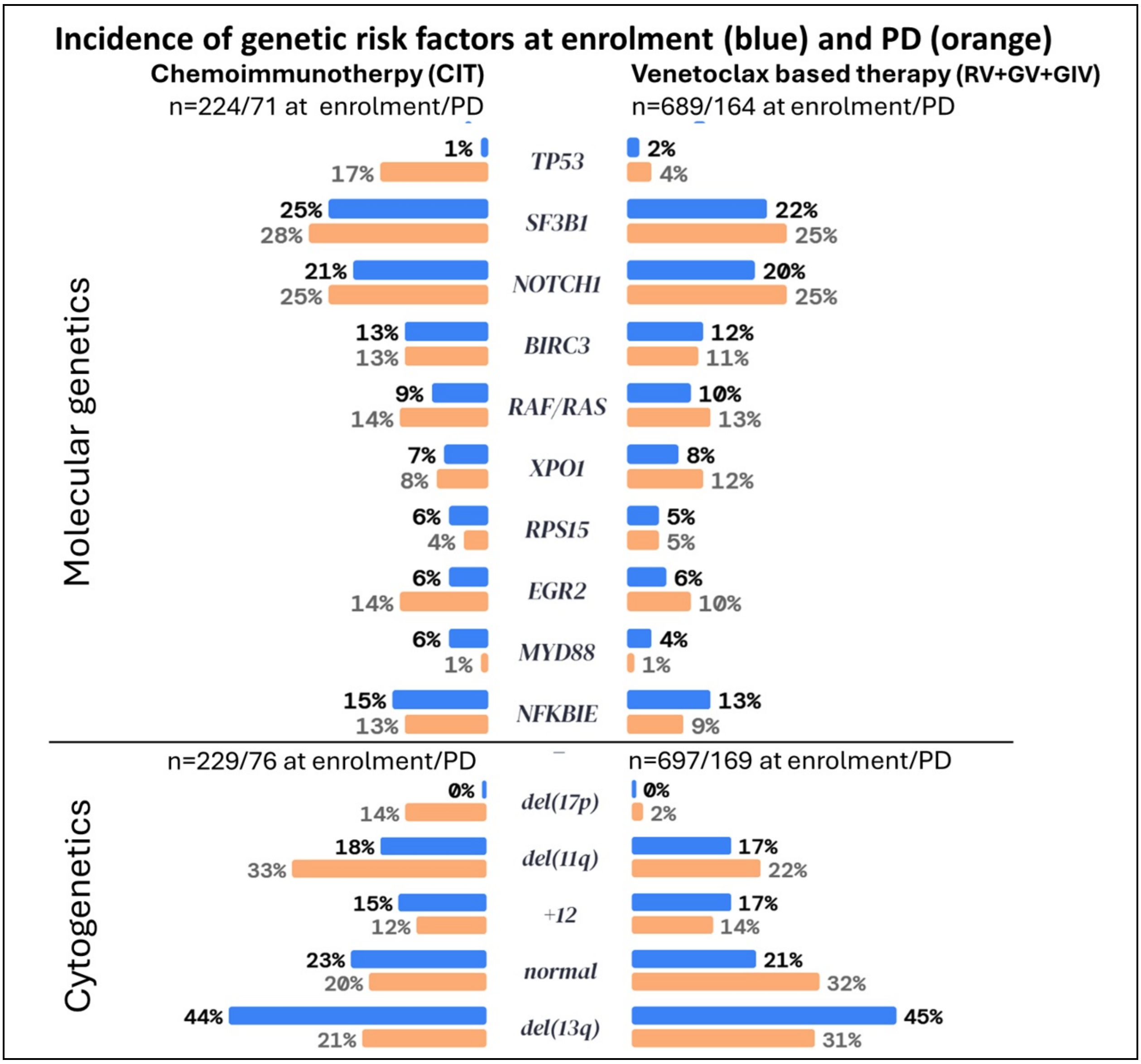

**Copyright Information:** (Online) ISSN: 2572-9241
©2026 The Author(s). HemaSphere is published by John Wiley & Sons Ltd on behalf of European Hematology Association. This is an open access Abstract Book distributed under the Attribution-NonCommercial-NoDerivs (CC BY-NC-ND), which allows third parties to download the articles and share them with others as long as they credit the author and the Abstract Book, but they cannot change the content in any way or use them commercially.
**Abstract Book Citations:** Authors, Title, HemaSphere, 2026;10:(S1):pages. The abstract book DOIs can be found on the "Author Information" page.
**Disclaimer:** Articles published in the journal HemaSphere exclusively reflect the opinions of the authors. The authors are responsible for all content in their abstracts including accuracy of the facts, statements, citing resources, etc.

### (S150) UPDATED EFFICACY AND SAFETY DATA FROM AN ONGOING PHASE 1A/B TRIAL OF THE BTK DEGRADER BEXOBRUTIDEG (NX-5948) IN PATIENTS WITH CLL ACROSS LINES OF THERAPY

**Topic:** 06. Chronic lymphocytic leukemia and related disorders - Clinical

Talha Munir*[1], Zulfa Omer[2], Sebastian Grosicki[3], Michal Kwiatek[4], Alexey Danilov[5], Nirav N Shah[6], Francesco Forconi[7], Alvaro J. Alencar[8], Mary Gleeson[9, 10], Laura Samples[11], Graham Collins[12], Jane Robertson[13], Jonathon Cohen[14], William Wierda[15], Danielle M. Brander[16], Shuo MA[17], Allison Winter[18], Krzysztof Giannopoulos[19], John C. Byrd[2], Sarah Injac[20], Wojciech Jurczak[21]

*[1]St. James's Hospital, Leeds, United Kingdom;[2]University of Cincinnati, Cincinnati, United States of America;[3]Medical University of Silesia, Katowice, Poland; [4]AidPort Hospital, Skórzewo (Poznan), Poland;[5]City of Hope National Medical Center, Duarte, United States of America; [6]Medical College of Wisconsin, Milwaukee, United States of America;[7]University Hospital Southampton NHS Trust, Southampton, United Kingdom; [8]Sylvester Comprehensive Cancer Center, University of Miami Miller School of Medicine, Miami, United States of America; [9]Guy's and St Thomas' NHS Foundation Trust, London, United Kingdom; [10]Sarah Cannon Research Institute, London, UK, London, United Kingdom; [11]Fred Hutchinson Cancer Center, Seattle, United States of America; [12]Oxford Cancer and Haematology Centre, Churchill Hospital, Oxford, United Kingdom; [13]The Christie Hospital and Manchester Cancer Research Centre, Division of Cancer Sciences, Manchester, United Kingdom; [14]Emory Winship Cancer Institute, Atlanta, United States of America; [15]MD Anderson Cancer Center, Houston, United States of America; [16]Duke Cancer Institute, Durham, United States of America; [17]Feinberg School of Medicine, Northwestern University, Chicago, United States of America; [18]Cleveland Clinic Foundation, Cleveland, United States of America; [19]Medical University of Lublin, Lublin, Poland; [20]Nurix Therapeutics, Inc., Brisbane, United States of America; [21]Maria Sklodowska-Curie National Research Institute of Oncology, Krakow, Poland;*

### Background

Although BTK inhibition is a successful treatment for CLL/SLL, the emergence of BTK inhibitor (BTKi) resistance mutations and kinase-independent signaling via the scaffolding function of BTK result in a need for alternative treatments. Bexobrutideg (NX-5948) is a highly selective, orally administered small molecule degrader that causes the removal of wild-type and mutant forms of BTK through ubiquitination by the cereblon E3 ligase complex and subsequent proteasomal degradation.

### Aims

We report new and updated efficacy and safety data with bexobrutideg in CLL/SLL from a Phase (Ph) 1a trial and select Ph1b cohorts including patients (pts) with different prior treatment profiles, in pts with relapsed/refractory (R/R) disease as well as pts in earlier lines of therapy (LoT).

### Methods

NX-5948-301 (NCT05131022) is a Ph1, first-in-human trial of bexobrutideg in advanced B-cell malignancies. In CLL/SLL, bexobrutideg is being evaluated in R/R pts in the Ph1a dose-escalation part and then in Ph1b dose-expansion cohorts that include heavily pre-treated, earlier-line and treatment-naïve pts. Safety is presented for all pts with CLL/SLL. Efficacy is presented for all Ph1a dose-escalation pts and for selected Ph1b dose-expansion cohorts treated at the recommended Ph2 dose (RP2D) of 600 mg: Cohort 1 (C1; primarily BTKi and BCL2i exposed), C5 (BTKi-exposed and BCL2i-naïve), and C15 (BTKi-naïve, including treatment-naïve pts).

**Copyright Information:** (Online) ISSN: 2572-9241
©2026 The Author(s). HemaSphere is published by John Wiley & Sons Ltd on behalf of European Hematology Association. This is an open access Abstract Book distributed under the Attribution-NonCommercial-NoDerivs (CC BY-NC-ND), which allows third parties to download the articles and share them with others as long as they credit the author and the Abstract Book, but they cannot change the content in any way or use them commercially.
**Abstract Book Citations:** Authors, Title, HemaSphere, 2026;10:(S1):pages. The abstract book DOIs can be found on the "Author Information" page.
**Disclaimer:** Articles published in the journal HemaSphere exclusively reflect the opinions of the authors. The authors are responsible for all content in their abstracts including accuracy of the facts, statements, citing resources, etc.

### Results

As of 1 Jan 2026, 142 CLL/SLL pts have been enrolled (48 to Ph1a; 42 to Ph1b C1; 19 to Ph1b C5; and 20 to Ph1b C15 with 10 treatment-naive). Ph1a and Ph1b C1 had a median 4 and 5 prior LoT respectively, while Ph1b C5 and Ph1b C15 had a median 2 and 1 prior LoT, respectively.
Bexobrutideg continues to be well tolerated in pts treated across all doses (n=142), including in 86 pts treated at 600 mg. The safety profile in Ph1a and the Ph1b cohorts was maintained over the duration of treatment and across different prior LoT with most adverse events (AEs) being grade 1/2. The most common treatment-emergent AEs included purpura/contusion, neutropenia, and petechiae.
A high objective response rate (ORR) was observed across all cohorts irrespective of prior therapies: Ph1a 83.0% (22.4 months follow-up); Ph1b C1 (BTKi/BCL2i exposed) 83.3% at 600 mg (13.1 months follow-up); Ph1b C5 (BTKi-exposed/BCL2i naïve) 86.7% (5.3 months follow-up); Ph1b C15 (BTKi-naïve including treatment naïve) 84.2% (4.9 months follow-up). The high ORRs in Ph1b C5 and Ph1b C15 are notable, given the short duration of treatment and follow-up; deepening of responses would be anticipated with longer duration of treatment. Lymph node size decreases were observed regardless of prior treatments or mutational burden at baseline (see Figure). Median progression-free survival (mPFS) was 22.1 months in Ph1a (mPFS follow-up 19.8 months).

### Summary/Conclusion

In this Ph1a/b trial, bexobrutideg demonstrated a favorable safety profile in pts with CLL/SLL with longer follow-up across doses and prior treatments. Durable responses were maintained in the Ph1a dose-escalation cohort, which has the longest follow-up to date. High response rates were observed across populations with different prior therapies, including in pts previously treated with BTKi and in BTKi-naïve pts, supporting further clinical development. Bexobrutideg is being evaluated in the Ph2 DAYBreak-201 study (NCT07221500) and a Ph3 DAYBreak-306 study investigating bexobrutideg vs pirtobrutinib is planned.

**Figure.** Lymph node reduction in select cohorts of Ph1b with different ranges of prior treatments and associated mutational burden levels at baseline

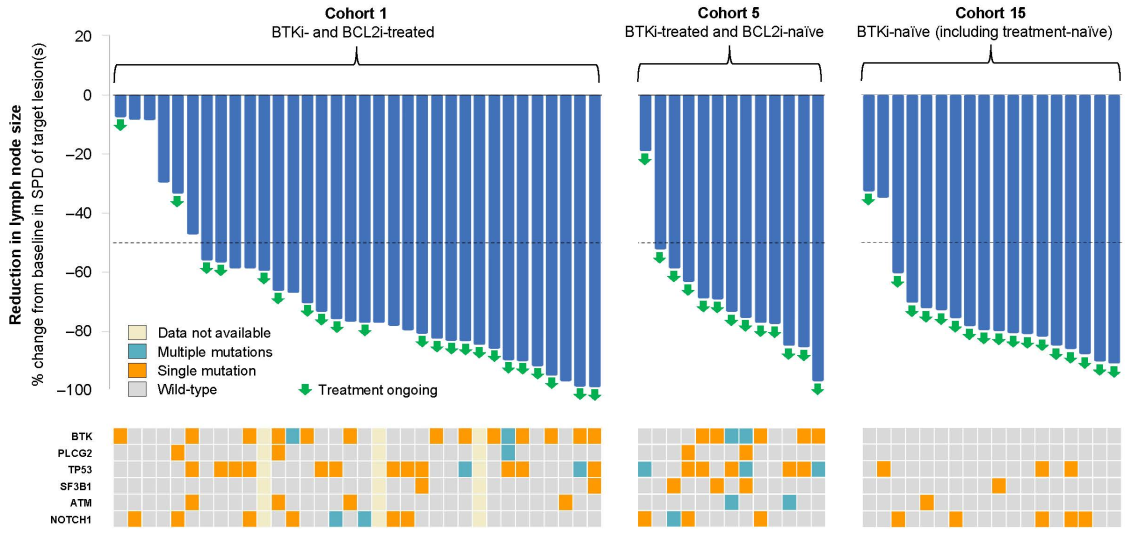

**Copyright Information:** (Online) ISSN: 2572-9241
©2026 The Author(s). HemaSphere is published by John Wiley & Sons Ltd on behalf of European Hematology Association. This is an open access Abstract Book distributed under the Attribution-NonCommercial-NoDerivs (CC BY-NC-ND), which allows third parties to download the articles and share them with others as long as they credit the author and the Abstract Book, but they cannot change the content in any way or use them commercially.
**Abstract Book Citations:** Authors, Title, HemaSphere, 2026;10:(S1):pages. The abstract book DOIs can be found on the "Author Information" page.
**Disclaimer:** Articles published in the journal HemaSphere exclusively reflect the opinions of the authors. The authors are responsible for all content in their abstracts including accuracy of the facts, statements, citing resources, etc.

### (S151) OUTCOMES OF VENETOCLAX-BASED TREATMENT IN PATIENTS WITH BRUTON TYROSINE KINASE INHIBITOR (BTKI)-TREATED CHRONIC LYMPHOCYTIC LEUKEMIA (CLL): AN INTERNATIONAL STUDY CONDUCTED BY ERIC

**Topic:** 06. Chronic lymphocytic leukemia and related disorders - Clinical

Paul Hampel[1], Thomas Chatzikonstantinou*[2], Lydia Scarfò[3, 4], Persefoni Talimtzi[5], Eva Minga[5], Maria Angelopoulou[6], Miguel Argüello-Tomás[7], Csaba Bödör[8], Francisco Bosch Albareda[9], Marcello Bradaschia[10], Sofia Chatzileontiadou[11], Caterina Chessa[12], Aline Clavert[13], Rosa Collado Nieto[14], Marta Crespo Maull[15], Antonio Cuneo[16], Zadie Davis[17], Marie Sarah Dilhuydy[18], Maria Dimou[6], Michael Doubek[19], Maria Efstathopoulou[20], Blanca Espinet Sola[21], Emmanuelle Ferrant[22], Luc Matthieu Fornecker[23], Gianluca Gaidano[24], Rocío García-Serra[14], Eva Gimeno[25], Alessandro Gozzetti[10], Romain Guieze[26], Evdoxia Chatzicharisi[11], Eleftheria Hatzimichael[27], Yair Herishanu[28], Talal Hilal[29], Luca Inchiappa[30], Saad Kenderian[1], Amber Koehler[1], Iliana Konstantinou[31], Maya Koren-Michowitz[32], Ioannis Kotsianidis[33], Andreas-Epameinondas Koumpis[27], Luca Laurenti[34], Jose Leis[29], Stephane Lepretre[35], Alexandros MacHairas[6], Anne-Sophie Michallet[36], Riccardo Moia[24], Ana Muntañola Prat[37, 38], Roberta Murru[39], Gerassimos Pangalis[20], Angel Serna Pareja[40], Ricardo D. Parrondo[41], Sarka Pavlova[42], Sarka Pospisilova[42], Anna Puiggros[43, 44], Kari Rabe[1], Sergio Ramos Cillan[45], Gian Matteo Rigolin[16], Lindsey Roeker*[1], Ilaria Romano[46], Davide Rossi[47, 48, 49], Rocio Salgado[50], Susan Schwager[1], Martin Simkovic[51, 52], Susan Slager[1], Lukáš Smolej[51, 53], Paolo Sportoletti[54, 55], Tamás László[56, 57], Emmanuelle Tchernonog[58], Anna Maria Tomasso[59, 60], Livio Trentin[61], Mazie Tsang[29], Theodoros Vassilakopoulos[6], Andrea Visentin[61], Candida Vitale[62, 63], Georgios Vrachiolias[33], Yucai Wang[1], Niki Stavrogianni[64], Anastasia Chatzidimitriou[5], Neil Kay[1], Kostas Stamatopoulos[2], Sameer Parikh[3], Paolo Ghia[3, 4], Loic Ysebaert[65]

*[1]Mayo Clinic, Rochester, MN, United States of America;[2]Institute of Applied Biosciences, Centre for Research and Technology Hellas, Thessaloniki, Greece; [3]Medical School, Università Vita-Salute San Raffaele and IRCCS Ospedale San Raffaele, Milan, Italy; [4]Comprehenzive Cancer Center, IRCCS Ospedale San Raffaele, Milan, Italy; [5]Institute of Applied Biosciences, Center for Research and Technology, Thessaloniki, Greece; [6]Department of Haematology, University of Athens, Laikon General Hospital, Athens, Greece; [7]Hematology Department, Hospital de la Santa Creu i Sant Pau, IIB-Sant Pau and José Carreras Leukemia Research Institutes, Departamento de Medicina, Universidad Autónoma de Barcelona, Barcelona, Spain; [8]MTA-SE Lendulet Molecular Oncohematology Research Group, Department of Pathology and Experimental Cancer Research, Semmelweis UniversityMTA-SE Lendulet Molecular Oncohematology Research Group, Department of Pathology and Experimental Cancer Research, Semmelweis University, Budapest, Hungary; [9]Department of Haematology, University Hospital Vall d'Hebron, Autonomous University, Barcelona, Spain; [10]Division of Hematology, University of Siena, Siena, Italy; [11]Hematology Unit, 1st Dept of Internal Medicine, AUTH, AHEPA Hospital, Thessaloniki, Greece; [12]Hematology and Stem Cell Transplantation Unit, Ospedale Oncologico A. Businco, ARNAS "G. Brotzu", Cagliari, Italy; [13]Service d'hématologie, CHU Angers, Angers, France;[14]Servicio de Hematología, Consorcio Hospital General Universitario de Valencia. Fundación de Investigación Hospital General Universitario de Valencia, Valencia, Spain; [15]Department of Medicine, Universitat Autònoma de Barcelona, Bellaterra, Spain; Servei d'Hematologia, Vall d'Hebron Hospital Universitari, Experimental Hematology, Vall d'Hebron Institute of Oncology (VHIO), Vall d'Hebron Barcelona Hospital Campus, Barcelona, Spain; [16]St. Anna University Hospital, Ferrara, Italy; [17]Molecular Pathology, UHD Royal Bournemouth Hospital, Bournemouth, United Kingdom; [18]Service d'hématologie, CHU Bordeaux, Bordeaux, France; [19]Central EuropeaInstitute of Technology (CEITEC), Masaryk University, Brno, Czechia;[20]Department of Haematology, Athens Medical Center, Athens, Greece; [21]Molecular Cytogenetics Laboratory, Pathology Department, Hospital del Mar and Translational Research on Hematological Neoplasms Group, Hospital del Mar Research Institute (IMIM), Barcelona, Spain; [22]Service d'hématologie, CHU Lyon-Sud, Lyon, France; [23]Service d'hématologie, CHU Strasbourg, Strasbourg, France;[24]Division of Hematology, Department of Translational Medicine, University of Eastern Piedmont, SCDU Ematologia, AOU Novara, Novara, Italy; [25]Hematology Service, Hospital del Mar, Barcelona, Spain;[26]Service d'hématologie, CHU Clermont-Ferrand, Clermont-Ferrand, France; [27]Department of Haematology, Faculty of Medicine, University of Ioannina, Ioannina, Greece;[28]Department of Henatology, Tel Aviv Sourasky Medical Center and Gray Faculty of Medical & Health Sciences, Tel Aviv, Israel; [29]Mayo Clinic, Phoenix, AZ, United States of America; [30]Service d'hématologie, Institut Paoli-Calmettes de Marseille, Marseille, France;[31]Laikon General Hospital, Athens, Greece; [32]Shamir Medical Center, affiliated to Gray Faculty of Medicine, Tel Aviv University, Tel Aviv, Israel; [33]Department of Hematology, University Hospital of Alexandroupolis, Democritus University of Thrace, Alexandroupolis,*

**Copyright Information:** (Online) ISSN: 2572-9241
©2026 The Author(s). HemaSphere is published by John Wiley & Sons Ltd on behalf of European Hematology Association. This is an open access Abstract Book distributed under the Attribution-NonCommercial-NoDerivs (CC BY-NC-ND), which allows third parties to download the articles and share them with others as long as they credit the author and the Abstract Book, but they cannot change the content in any way or use them commercially.
**Abstract Book Citations:** Authors, Title, HemaSphere, 2026;10:(S1):pages. The abstract book DOIs can be found on the "Author Information" page.
**Disclaimer:** Articles published in the journal HemaSphere exclusively reflect the opinions of the authors. The authors are responsible for all content in their abstracts including accuracy of the facts, statements, citing resources, etc.

Greece; [34]Università Cattolica del Sacro Cuore, Dipartimento di Diagnostica per Immagini, Radioterapia Oncologica ed Ematologia, Fondazione Policlinico Universitario Agostino Gemelli IRCCS, Rome, Italy; [35]Inserm U1245 and Department of Hematology, Centre Henri Becquerel and Normandie Univ UNIROUEN, Rouen, France; [36]Centre Léon Bérard de Lyon, Lyon, France; [37]Hematology Department, Hospital de la Santa Creu i Sant Pau, IIB-Sant Pau, Barcelona, Spain;[38]José Carreras Leukemia Research Institutes, Departamento de Medicina, Universidad Autónoma de Barcelona, Barcelona, Spain; [39]Hematology and Stem Cell Transplantation Unit, Ospedale Oncologico A. Businco, ARNAS G. Brotzu, Cagliari, Italy; [40]Department of Medicine, Universitat Autònoma de Barcelona, Barcelona, Spain;[41]Mayo Clinic, Jacksonville, FL, United States of America; [42]Department of Internal Medicine, Hematology and Oncology, University Hospital Brno and Medical Faculty, Masaryk University, Brno, Czechia; [43]Molecular Cytogenetics Laboratory, Pathology Department, Hospital del Mar, Barcelona, Spain; [44]Translational Research on Hematological Neoplasms Group, Hospital del Mar Research Institute (IMIM), Barcelona, Spain; [45]Fundación Jiménez Díaz University Hospital, Health Research Institute IIS-FJD, Madrid, Spain;[46]Oncology Institute of Southern Switzerland, Bellinzona, Switzerland; [47]Oncology Institute of Southern Switzerland (IOSI), Ente Ospedaliero Cantonale (EOC), Bellinzona, Switzerland; [48]Institute of Oncology Research (IOR), Bellinzona, Switzerland;[49]Faculty of Biomedical Sciences, Università della Svizzera Italiana (USI), Lugano, Switzerland; [50]Fundacion Jimenez Díaz University Hospital, Health Research Institute IIS-FJD, Madrid, Spain; [51]4th Department of Internal Medicine-Hematology, Faculty of Medicine in Hradec Králové, University Hospital, Hradec Králové, Czechia; [52]Charles University,, Hradec Kralove, Czechia; [53]Charles University, Hradec Králové, Czechia; [54]Institute of Hematology and Center for Hemato-Oncology Research, University of Perugia, Perugia, Italy; [55]Santa Maria della Misericordia Hospital, Perugia, Italy;[56]HCEMM-SE Molecular Oncohematology Research Group, Department of Pathology and Experimental Cancer Research, Semmelweis University, Budapest, Hungary; [57]MTA-SE Lendulet Molecular Oncohematology Research Group, Department of Pathology and Experimental Cancer Research, Semmelweis University, Budapest, Hungary; [58]Service d'Hématologie, CHU Montpellier, Montpellier, France;[59]Università Cattolica del Sacro Cuore, Dipartimento di Diagnostica per Immagini, Radioterapia Oncologica ed Ematologia, Rome, Italy; [60]Fondazione Policlinico Universitario Agostino Gemelli IRCCS, Rome, Italy; [61]Hematology and Clinical Immunology Unit, Department of Medicine, University of Padova, Padova, Italy; [62]Division of Hematology, A.O.U. Città della Salute e della Scienza di Torino, Turin, Italy; [63]Department of Molecular Biotechnology and Health Sciences, University of Turin, Turin, Italy;[64]Hematology Department and HCT Unit, G. Papanicolaou Hospital, Thessaloniki, Greece; [65]Department of Hematology, Institut Universitaire du Cancer Toulouse-Oncopole, Toulouse, France;

### Background

Venetoclax + rituximab (VenR) resulted in a median progression-free survival of 55 months in the mostly 2$^{nd}$ line (2L), post-chemotherapy setting of the MURANO clinical trial (Kater *Blood* 2025). Shorter remissions with venetoclax (Ven) treatments (tx) in patients (pts) with prior BTKi for CLL are reported. However, these studies evaluated small, heavily pre-treated cohorts.

### Aims

We aim to 1) describe the clinical outcomes of pts receiving Ven-based CLL tx after prior BTKi; and 2) identify factors associated with overall survival (OS) and time to next therapy or death (TTNTD) in these pts.

### Methods

This is a retrospective international study conducted by ERIC, the European Research Initiative on CLL. Pts who received Ven for relapsed CLL after prior BTKi were included. Pts were grouped by reason for prior BTKi discontinuation: CLL progression or non-progression. Pts who discontinued BTKi due to Richter transformation were excluded. OS was analyzed as the time from ven start until the date of death or last known to be alive. TTNTD was defined as the time from ven start until the earliest date of next CLL tx or death. OS and TTNTD were estimated by the Kaplan-Meier method. Cox proportional hazards regression models were used to assess factors associated with time-to-event outcomes.

**Copyright Information:** (Online) ISSN: 2572-9241
©2026 The Author(s). HemaSphere is published by John Wiley & Sons Ltd on behalf of European Hematology Association. This is an open access Abstract Book distributed under the Attribution-NonCommercial-NoDerivs (CC BY-NC-ND), which allows third parties to download the articles and share them with others as long as they credit the author and the Abstract Book, but they cannot change the content in any way or use them commercially.
**Abstract Book Citations:** Authors, Title, HemaSphere, 2026;10:(S1):pages. The abstract book DOIs can be found on the "Author Information" page.
**Disclaimer:** Articles published in the journal HemaSphere exclusively reflect the opinions of the authors. The authors are responsible for all content in their abstracts including accuracy of the facts, statements, citing resources, etc.

**Results**

A total of 624 pts received Ven, including: Ven monotherapy (n=278, 45%), VenR (n=230, 37%), Ven + obinutuzumab (VenO) (n=78, 13%), and other (n=38, 6%). Pt characteristics at the time of Ven start were as follows: median age 70 years (interquartile range [IQR] 63-76), 426 (68%) male, 221/341 (65%) with *TP53* disruption (del(17p) or *TP53* mutation), 284/346 (82%) with unmutated IGHV genes, and 85/183 (46%) with complex karyotype (≥3 clonal abnormalities). The median prior lines of therapy was 2 (IQR 2-4); BTKi was 1L CLL tx for 153 (25%) pts. Prior BTKi was stopped for progression in 330 (53%) pts (median duration of prior BTKi tx 42 months [IQR 24.5-58.1]) and for a reason other than CLL progression in 294 (47%) pts (median duration of prior BTKi 13 months [IQR 4.2-32]).
The median follow-up from the start of Ven-based tx was 24 months. The median TTNTD and OS for the entire cohort was 33 (95% CI: 30-36) and 46 months (95% CI 42-54), respectively. TTNTD (Figure 1A) and OS (Figure 1B) were significantly shorter in pts who stopped prior BTKi due to CLL progression compared to pts who stopped BTKi for a non-progression reason.
Among the 330 pts stopping prior BTKi for CLL progression, the median TTNTD was 32 months in pts who had received BTKi as 1L and 25 months who had received BTKi as a subsequent line tx (Figure 1C). VenO (but not VenR) was associated with better TTNTD compared to Ven monotherapy (HR 0.26; 95% CI 0.08-0.85; P=0.03) on multivariable analysis (MVA). *TP53* disruption was associated with worse TTNTD (HR 1.66; 95% CI 1.07-2.58; P=0.02). Similar trends without reaching statistical significance were seen on OS [MVA: VenO HR 0.28 (95% CI 0.07-1.17; P=0.08); *TP53* disruption HR 1.55 (95% CI 0.95-2.55; P=0.08)].

**Summary/Conclusion**

In the largest cohort of pts with BTKi-treated CLL receiving subsequent Ven-based tx pts had worse than expected outcomes, particularly those discontinuing their prior BTKi due to CLL progression (including when BTKi was 1L). These results establish an updated benchmark for outcomes following BTKi tx and provide justification for prioritizing novel tx trial strategies for pts with BTKi-resistant CLL, even in the 2L setting.

**Copyright Information:** (Online) ISSN: 2572-9241
©2026 The Author(s). HemaSphere is published by John Wiley & Sons Ltd on behalf of European Hematology Association. This is an open access Abstract Book distributed under the Attribution-NonCommercial-NoDerivs (CC BY-NC-ND), which allows third parties to download the articles and share them with others as long as they credit the author and the Abstract Book, but they cannot change the content in any way or use them commercially.
**Abstract Book Citations:** Authors, Title, HemaSphere, 2026;10:(S1):pages. The abstract book DOIs can be found on the “Author Information” page.
**Disclaimer:** Articles published in the journal HemaSphere exclusively reflect the opinions of the authors. The authors are responsible for all content in their abstracts including accuracy of the facts, statements, citing resources, etc.

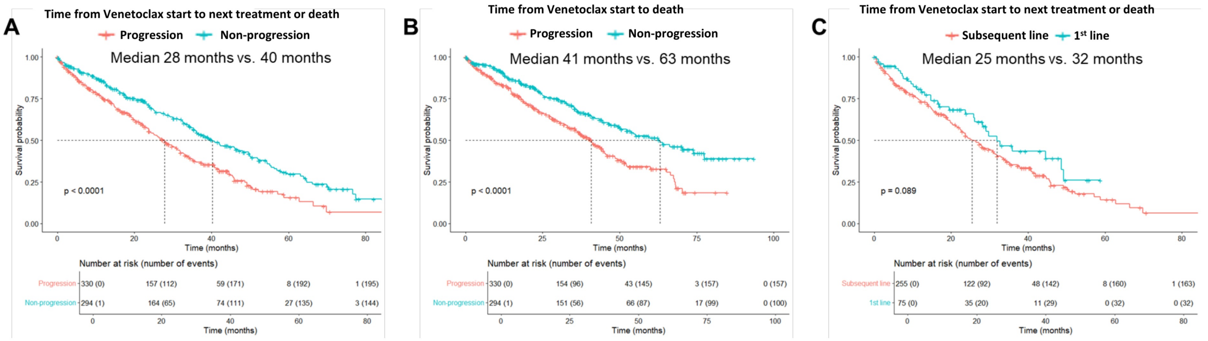

FIGURE 1 . A) Entire cohort TTNTD – patients stopping prior BTKi due to CLL progression (coral line) and patients stopping prior BTKi for a non-progression reason (teal line). B) Entire cohort (n=624) OS – patients stopping prior BTKi due to CLL progression (coral line) and patients stopping prior BTKi for a non-progression reason (teal line). C) BTKi stopped due to CLL progression subgroup (n=330) – patients treated with BTKi as 1L CLL treatment (coral line) and patients treated with BTKi for CLL in the subsequent line / relapsed setting (teal line).

**Copyright Information:** (Online) ISSN: 2572-9241
©2026 The Author(s). HemaSphere is published by John Wiley & Sons Ltd on behalf of European Hematology Association. This is an open access Abstract Book distributed under the Attribution-NonCommercial-NoDerivs (CC BY-NC-ND), which allows third parties to download the articles and share them with others as long as they credit the author and the Abstract Book, but they cannot change the content in any way or use them commercially.
**Abstract Book Citations:** Authors, Title, HemaSphere, 2026;10:(S1):pages. The abstract book DOIs can be found on the "Author Information" page.
**Disclaimer:** Articles published in the journal HemaSphere exclusively reflect the opinions of the authors. The authors are responsible for all content in their abstracts including accuracy of the facts, statements, citing resources, etc.

### (S152) BGB-16673, A BRUTON TYROSINE KINASE (BTK) DEGRADER, IN PATIENTS WITH RELAPSED/REFRACTORY (R/R) CHRONIC LYMPHOCYTIC LEUKEMIA/SMALL LYMPHOCYTIC LYMPHOMA (CLL/SLL): A PHASE 1 CADANCE-101 STUDY UPDATE

**Topic:** 06. Chronic lymphocytic leukemia and related disorders - Clinical

Stephan Stilgenbauer*[1], Ricardo D. Parrondo[2], Meghan C. Thompson[3], Anna Maria Frustaci[4], John N. Allan[5], Paolo Ghia[6, 7], Irina Mocanu[8], Damien Roos-Weil[9], Constantine S. Tam[10], Judith Trotman[11], Inhye E. Ahn[12], Lydia Scarfò[6, 7], Carlo Visco[13], Michael Choi[14], Yanan Zhang[15], Linlin Xu[15], Kunthel By[15], Shannon Fabre[15], Daniel Persky[15], Shelonitda Rose[15], John F. Seymour[16]

*[1]Ulm University, Ulm, Germany; [2]Mayo Clinic - Jacksonville, Jacksonville, FL, United States of America; [3]Memorial Sloan Kettering Cancer Center, New York, NY, United States of America; [4]ASST Grande Ospedale Metropolitano Niguarda, Milano, Italy; [5]Weill Cornell Medicine, New York, NY, United States of America; [6]Università Vita-Salute San Raffaele, Milano, Italy; [7]Comprehensive Cancer Center, IRCCS Ospedale San Raffaele, Milano, Italy; [8]The Institute of Oncology, ARENSIA Exploratory Medicine, Chisinau, Moldova; [9]Pitié-Salpêtrière Hospital, Paris, France; [10]Alfred Hospital and Monash University, Melbourne, VIC, Australia; [11]Concord Repatriation General Hospital, University of Sydney, Concord, NSW, Australia; [12]Dana-Farber Cancer Institute, Boston, MA, United States of America; [13]Universita di Verona, Verona, Italy; [14]Moores Cancer Center, University of California San Diego, La Jolla, CA, United States of America; [15]BeOne Medicines, Ltd, San Carlos, CA, United States of America; [16]Peter MacCallum Cancer Centre, Royal Melbourne Hospital, and University of Melbourne, Melbourne, VIC, Australia;*

### Background

BTK inhibitors (BTKis) are effective treatments (tx) for CLL/SLL; however, development of resistance and intolerance remain a challenge. BGB-16673 is a BTK degrader that blocks signaling by tagging BTK for degradation through the cell's proteasome pathway, leading to tumor regression. The ongoing open-label, phase 1/2 CaDAnCe-101 (BGB-16673-101; NCT05006716) study is evaluating BGB-16673 monotherapy in patients (pts) with B-cell malignancies.

### Aims

To report updated safety and efficacy results for phase 1 of the CaDAnCe-101 trial in pts with R/R CLL/SLL.

### Methods

Eligible pts had a diagnosis of R/R CLL/SLL (≥2 prior therapies), an ECOG performance status of 0-2 (0-1 in the EU), and adequate organ function. Pts in the US, EU, and Australia must have received prior tx with a covalent BTKi. BGB-16673 was administered orally once daily. The primary phase 1 study objectives were to assess safety/tolerability (NCI-CTCAE v5.0; iwCLL hematologic toxicity criteria) and to establish the maximum tolerated dose and recommended expansion dose. Secondary objectives included assessing the overall response rate (ORR) per iwCLL 2018 criteria with the partial response (PR) with lymphocytosis (PR-L) modification and per the 2014 Lugano criteria (SLL), with the first assessment after 12 weeks of tx.

**Copyright Information:** (Online) ISSN: 2572-9241
©2026 The Author(s). HemaSphere is published by John Wiley & Sons Ltd on behalf of European Hematology Association. This is an open access Abstract Book distributed under the Attribution-NonCommercial-NoDerivs (CC BY-NC-ND), which allows third parties to download the articles and share them with others as long as they credit the author and the Abstract Book, but they cannot change the content in any way or use them commercially.
**Abstract Book Citations:** Authors, Title, HemaSphere, 2026;10:(S1):pages. The abstract book DOIs can be found on the “Author Information” page.
**Disclaimer:** Articles published in the journal HemaSphere exclusively reflect the opinions of the authors. The authors are responsible for all content in their abstracts including accuracy of the facts, statements, citing resources, etc.

**Results**

As of the Dec 15, 2025, data cutoff, 67 pts with R/R CLL/SLL received BGB-16673 (50 mg, n=1; 100 mg, n=22; 200 mg, n=17; 350 mg, n=15; 500 mg, n=12). Median age was 70 (range, 47-91) years, and pts received a median of 4 prior lines of therapy (range, 2-10). Prior therapies included covalent BTKis (n=63 [94.0%]), noncovalent BTKis (n=14 [20.9%]), and BCL2 inhibitors (n=55 [82.1%]). Baseline mutations are shown (Table). Median study follow-up was 24.1 (range, 0.3-37.7) months, and 35 pts (52.2%) remained on tx at data cutoff.
Any-grade tx-emergent adverse events (TEAEs) occurred in 97.0% of the 67 pts; TEAEs in ≥25% of pts were fatigue (37.3%), contusion (32.8%), diarrhea (29.9%), and neutropenia/neutrophil count decreased (29.9%). Grade ≥3 TEAEs occurred in 61.2% of pts; those occurring in ≥5% were infections (34.3%), neutropenia/neutrophil count decreased (25.4%), and thrombocytopenia/platelet count decreased (7.5%). TEAEs led to dose reduction in 9 pts (13.4%) and tx discontinuation in 12 pts (17.9%), 4 (6.0%) of whom had tx-related TEAEs (subdural hemorrhage, peripheral swelling and ecchymosis, maculopapular rash, and disseminated aspergillosis). Among 67 pts, 5 (7.5%) had TEAEs that led to death (all due to infection; one in the context of progressive disease [PD]).
Among all 67 pts, the ORR (≥PR-L) was 85.1% (n=57), and the PR or better rate was 77.6% (n=52); the complete response (CR)/CR with incomplete marrow recovery rate (CRi) was 3.0% (n=2). At the 200-mg dose, the ORR was 94.1% (16/17), with 1 CR. Median time to first response was 2.8 (range, 2.0-19.4) months, and median duration of response was 20.7 (range, 0-27.6) months. Responses were seen in pts with high-risk features (Table); responses by BTKi/BCL2i refractoriness will be presented. The 18-month PFS rate was 65.2% (95% CI, 50.9%-76.2%); 21 pts (31.3%) had PD, and 5 (7.5%) died.

**Summary/Conclusion**

These data demonstrate that the novel BTK degrader BGB-16673 continues to have a manageable safety profile with durable responses in pts with R/R CLL/SLL, including in pts with difficult-to-treat CLL/SLL. BGB-16673 (200 mg) is being evaluated in ongoing phase 2 and 3 studies in pts with R/R CLL/SLL.

**Table. ORR (defined as PR-L or better) according to prior therapy and mutation status**

| Characteristic | Patients, n/N (%) | ORR, n/N (%) |
|---|---|---|
| **Prior therapy** | | |
| cBTKi and BCL2i | 43/67 (64.2) | 40/43 (93.0) |
| cBTKi, BCL2i, and ncBTKi | 12/67 (17.9) | 9/12 (75.0) |
| **Mutation status** | | |
| *BTK* mutation | 25/66 (37.9) | 19/25 (76.0) |
| del(17p)/*TP53* mutation | 44/67 (65.7) | 35/44 (79.5) |
| *PLCG2* mutation | 10/66 (15.2) | 9/10 (90.0) |
| IGHV unmutated | 37/48 (77.1) | 32/37 (86.5) |
| Complex karyotype (≥3 abnormalities) | 23/43 (53.5) | 17/23 (73.9) |

BCL2i, B-cell lymphoma 2 inhibitor; BTK, Bruton tyrosine kinase; cBTKi, covalent BTK inhibitor; IGHV, immunoglobulin heavy-chain variable region; ncBTKi, noncovalent BTK inhibitor; PR-L, partial response with lymphocytosis.

**Copyright Information:** (Online) ISSN: 2572-9241
©2026 The Author(s). HemaSphere is published by John Wiley & Sons Ltd on behalf of European Hematology Association. This is an open access Abstract Book distributed under the Attribution-NonCommercial-NoDerivs (CC BY-NC-ND), which allows third parties to download the articles and share them with others as long as they credit the author and the Abstract Book, but they cannot change the content in any way or use them commercially.
**Abstract Book Citations:** Authors, Title, HemaSphere, 2026;10:(S1):pages. The abstract book DOIs can be found on the “Author Information” page.
**Disclaimer:** Articles published in the journal HemaSphere exclusively reflect the opinions of the authors. The authors are responsible for all content in their abstracts including accuracy of the facts, statements, citing resources, etc.

### (S153) FIXED DURATION VENETOCLAX PLUS EPCORITAMAB SHOWS FAVORABLE TOLERABILITY AND HIGH RESPONSE RATES WITH EARLY MOLECULAR RESPONSES IN R/R CLL/SLL: INTERIM ANALYSIS OF THE RANDOMIZED HOVON 165/AETHER TRIAL

**Topic:** 06. Chronic lymphocytic leukemia and related disorders - Clinical

Arnon Philip Kater*[1], Mark-David Levin[2], Julie Dubois[1], Erik van Werkhoven[3], Tessa Groeneweg[1], Fleur Peters[1], Clemens Mellink[4], Johan Dobber[5], Ludo Evers[5], A Hollestelle[4], Mar Bellido[6], Margot Jak[7], Josien Regelink[8], Jolanda Droogendijk[9], Mels Hoogendoorn[10], Djamila Issa[11], Marten Nijziel[12], Kirsten Fischer[13], Barbara Eichhorst[13], Michael Hallek[13], Kerstin Muehle[13], Ka Lung Wu[14], Lien Deleu[15], Henrik Frederiksen[16], Caspar Da Cunha-Bang[17], Constantine S. Tam[18], Philip Thompson[19], Rebecca Valentin[20], Gregory Takacs[21], Pk Epling-Burnette[21], Edith Szafer-Glusman[21], Pegah Jafarinasabian[21], Toshiko Oki[21], Lasse Nielsen[20], Cheyenne Pierie[1], Martine Testroote[22, 23], Wida Razawy[22, 23], Sabina Kersting[24], Carsten Niemann[17]

*[1]Amsterdam Umc-Cancer Center, Hematology, Amsterdam, The Netherlands; [2]Albert Schweitzer hospital, Dordrecht, The Netherlands; [3]Erasmus University Medical Center Cancer Institute and HOVON Foundation, Hovon and Hematology, rotterdam, The Netherlands; [4]Amsterdam University Medical Centers, Cytogenetics, Amsterdam, The Netherlands; [5]Amsterdam UMC, Hematology, Amsterdam, The Netherlands; [6]UMC Groningen, Groningen, The Netherlands; [7]UMC Utrecht, Utrecht, The Netherlands; [8]Meander Medical Center, Amersfoort, The Netherlands; [9]Elisabeth Tweesteden Hospital, Tilburg, The Netherlands; [10]Frisius Medical Center, Leeuwarden, Leeuwarden, The Netherlands; [11]Jeroen Bosch Hospital, Den Bosch, The Netherlands; [12]Catharina Hospital, Eindhoven, The Netherlands; [13]University Hospital Cologne, Cologne, Germany; [14]Ziekenhuis Netwerk Antwerpen Stuivenberg, Antwerpen, Belgium; [15]AZ Delta, Roeselare, Belgium; [16]Odense University Hospital, Denmark, Odense, Denmark; [17]Rigshospitalet, Copenhagen, Denmark; [18]Alfred Health, Melbourne, Australia, Hematology, Melbourne, Australia; [19]Peter Mac Cancer center, Melbou, Australia; [20]Genmab, Copenhagen, Denmark; [21]Abbvie, Chicago, United States of America; [22]HOVON, Rotterdam, The Netherlands; [23]HOVON Foundation, Rotterdam, The Netherlands; [24]Haga Ziekenhuis, Den Haag, The Netherlands;*

### Background

Relapsed/refractory (RR) chronic lymphocytic leukemia (CLL) following targeted therapies urgently necessitates alternative strategies such as T-cell directed treatments. However, CLL-mediated T cell dysfunction severely limits CAR-T efficacy, while continuous epcoritamab (Epco, a CD3xCD20 bispecific Ab) monotherapy does show activity, but with variable response depths. Such therapies are associated with cytokine release syndrome (CRS). Venetoclax (Ven) debulking can restore T cell fitness, providing a rationale for a fixed duration combination of Ven and Epco.

### Aims

To evaluate safety and early efficacy of Ven lead in followed by Epco plus Ven in patients with RR CLL/SLL.

### Methods

HOVON 165/AETHER is an ongoing multinational investigator initiated randomized phase I/II study in patients with RR CLL/SLL after at least one prior line of therapy. Patients received Ven ramp up followed by combination therapy with Epco and Ven, randomized to 6 (arm A) or 12 (arm B) Epco cycles using step up dosing in cycle 1, with Ven for 26 cycles in both arms. Following phase I safety findings, the protocol was amended with 3 Epco step-up doses, mandatory infection prophylaxis, and dexamethasone CRS prophylaxis. The primary endpoint is uMRD4 ($<10^{-4}$) at cycle 26. This preplanned interim analysis evaluates safety after Epco priming and early efficacy including MRD kinetics and iwCLL response at cycle 9 (ClinicalTrials.gov NCT05791409).

**Copyright Information:** (Online) ISSN: 2572-9241
©2026 The Author(s). HemaSphere is published by John Wiley & Sons Ltd on behalf of European Hematology Association. This is an open access Abstract Book distributed under the Attribution-NonCommercial-NoDerivs (CC BY-NC-ND), which allows third parties to download the articles and share them with others as long as they credit the author and the Abstract Book, but they cannot change the content in any way or use them commercially.
**Abstract Book Citations:** Authors, Title, HemaSphere, 2026;10:(S1):pages. The abstract book DOIs can be found on the “Author Information” page.
**Disclaimer:** Articles published in the journal HemaSphere exclusively reflect the opinions of the authors. The authors are responsible for all content in their abstracts including accuracy of the facts, statements, citing resources, etc.

### Results

Fortyseven of 100 planned patients have initiated treatment (45 CLL, 2 SLL). Previous targeted therapy in 74% (Ven 49%, BTK inhibitor 27%, double exposed 64%). High-risk biology: TP53 aberrations in 42% and unmutated IGHV in 70%.
In phase I (n=6), grade ≥2 CRS occurred in 3 patients, ICANS in 1, and grade ≥3 infections in 2 including one fatal event. Following protocol amendment, 25 patients were evaluable for cycle 1 safety. CRS was predominantly grade 1 with no grade ≥3 CRS observed. Transient grade 3 or 4 infections occurred in 3 pts without early treatment discontinuation. Laboratory but not clinical tumor lysis syndrome occurred in 3 pts.
In central MRD analyses, uMRD4 rates were 87.5% (21/24) and 90% (18/20) at cycles 4 and 6, respectively. These results encompassed patients with high-risk biology and previous targeted therapy. Among patients with paired peripheral blood and bone marrow assessment at cycle 9 (n=12), uMRD4 occurred in 83% (10/12) and 92% (11/12), respectively.
At first response assessment (cycle 9, n=14), overall response rate was 100%, including CR/CRi in 6 (43%), consistent across high-risk subgroups. Partial responses reflected low residual disease, most often confined to a single compartment (spleen or bone marrow) rather than nodal disease.
Exploratory analyses showed no association between baseline leukocyte count and CRS, whereas baseline thrombocytopenia was associated with increased CRS risk. TP53 aberrations and prior fixed duration therapies (BTK inhibitor and/or Ven based) were not associated with reduced uMRD, while previous continuous BTK inhibitor therapy showed a trend toward lower uMRD rates.

### Summary/Conclusion

Ven debulking followed by a CLL adapted Epco plus Ven strategy resulted in improved safety and high, early uMRD rates in high risk RR CLL/SLL. Responses were consistent across high-risk subgroups despite prior targeted therapy exposure, supporting fixed duration bispecific T cell therapy in CLL.

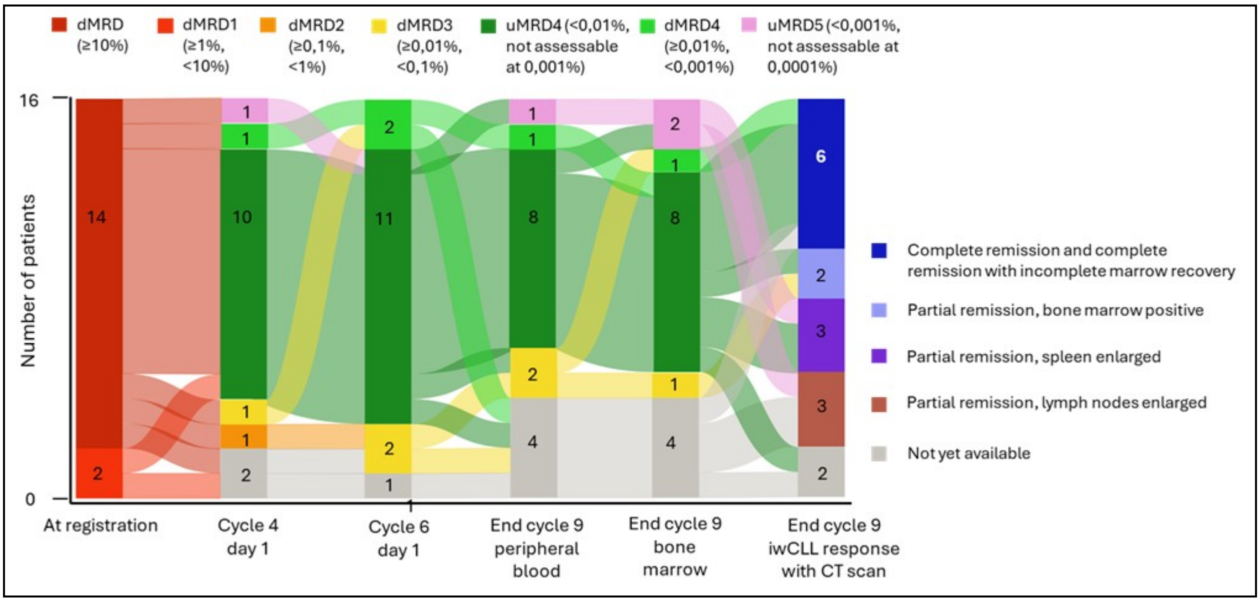

Sankey plots of MRD dynamics over time and the relationship between MRD levels and clinical response categories

**Copyright Information:** (Online) ISSN: 2572-9241
©2026 The Author(s). HemaSphere is published by John Wiley & Sons Ltd on behalf of European Hematology Association. This is an open access Abstract Book distributed under the Attribution-NonCommercial-NoDerivs (CC BY-NC-ND), which allows third parties to download the articles and share them with others as long as they credit the author and the Abstract Book, but they cannot change the content in any way or use them commercially.
**Abstract Book Citations:** Authors, Title, HemaSphere, 2026;10:(S1):pages. The abstract book DOIs can be found on the “Author Information” page.
**Disclaimer:** Articles published in the journal HemaSphere exclusively reflect the opinions of the authors. The authors are responsible for all content in their abstracts including accuracy of the facts, statements, citing resources, etc.

### (S154) DUAL TARGETED LENTIVIRAL TRANSDUCED ANTI-CD20/ANTI-CD19 (LV20.19) CAR T CELLS FOR RELAPSED, REFRACTORY CLL

**Topic:** 06. Chronic lymphocytic leukemia and related disorders - Clinical

Nirav N Shah*[1], Guru Subramanian Guru Murthy[1], Ehab Atallah[1], Sameem Abedin[1], Laura Michaelis[1], Aniko Szabo[1], Tyce Kearl[1], Gulrayz Ahmed[1], Kaitlin Annunzio[1], Anthony Stillwell[1], Daniel Bucklan[1], Bryon Johnson[1], Peiman Hematti[1], Mehdi Hamadani[1]

*[1]Medical College of Wisconsin, Milwaukee, United States of America;*

### Background

Despite advances in targeted therapies, patients (pts) with BTK/BCL2 inhibitor exposed relapsed, refractory (R/R) CLL have poor outcomes. While CD19 CAR T-cell therapy (lisocabtagene maraleucel) was approved for CLL pts exposed to BTKi/BCL2i, the complete response (CR) rate was 18% and the median PFS was 11.9 months in the pivotal phase II trial. Given sensitivity of CLL to CD20 targeting agents, we opened a cohort for R/R CLL pts within a larger ongoing trial of lentiviral anti-CD20/anti-CD19 (LV20.19) CAR T cells for R/R B-cell malignancies (NCT04186520).

### Aims

Determine the safety and efficacy of LV20.19 CAR T cells in R/R CLL

### Methods

We conducted a Phase 1/2 trial of LV20.19 CAR T cells at a fixed dose of 2.5e6 cells/kg in B-cell malignancies. CAR T cells were manufactured utilizing an adaptive 8/12-day process with the CliniMACS Prodigy® device and lymphodepletion starting during the manufacturing process to facilitate fresh infusion. CLL pts must have had two prior lines of therapy, one required to be a BTKi or BCL2i. Disease response was assessed using Hallek iwCLL criteria.

### Results

Nineteen pts with CLL received LV20.19 CAR T cells; 18 achieved target dose and were deemed evaluable. Ten pts were treated at a dose of 2.5e6 cells/kg; 8 pts were subsequently treated at a lower dose of 1e6 cells/kg due to presence of dose limiting toxicities (DLT). The median age was 65 years (34-75), and the median lines of prior therapy were 4 (range 2-7). All were BTKi/BCL2i exposed with 15 (83%) pts being refractory to both classes.

In terms of safety, 17/18 pts (94%) had CRS, only 1 patient with Grade 3 CRS. Four pts (22%) had ICANS, two (11%) pts with grade 3 and grade 4 ICANS respectively, both at the 2.5e6 cells/kg dose. The latter patient was found to have CLL in the CSF. Eight (47%) pts developed IEC-HS. Two CLL pts had grade 3 and grade 4 IEC-HS both also at the 2.5e6 cells/kg dose. Both met criteria for DLT; one died of fungemia related to immunosuppression. Consequently, the trial was amended for future CLL pts to receive a reduced dose of 1e6 cells/kg. At this lower dose, none of the 8 pts experienced high grade 3-4 CRS, ICANS, or IEC-HS. Cytokine analysis demonstrated that IEC-HS was not IL-6 driven, rather occurred in the setting of statistically significant elevations in G-CSF, IFN-gamma, IL-1ra, IL-2, IL-8, IL-9, IL-33, IL-10, TGF-a, PD-L1, and MCP-1.

In terms of clinical response, the best ORR was 89% (n=16) with CR/CRi rate of 78% (n=14). Figure 1 is swimmers plot demonstrating patient outcomes. Among 7 pts where ClonoSEQ MRD testing was done; all achieved negativity. The median PFS for all pts was 32 months. The median OS was not reached with median follow-up of 2 years for surviving pts. There was no difference in best ORR at the two dose levels. CAR T cell expansion and area under curve (AUC) was similar at both dose levels, p=0.74.

**Copyright Information:** (Online) ISSN: 2572-9241
©2026 The Author(s). HemaSphere is published by John Wiley & Sons Ltd on behalf of European Hematology Association. This is an open access Abstract Book distributed under the Attribution-NonCommercial-NoDerivs (CC BY-NC-ND), which allows third parties to download the articles and share them with others as long as they credit the author and the Abstract Book, but they cannot change the content in any way or use them commercially.
**Abstract Book Citations:** Authors, Title, HemaSphere, 2026;10:(S1):pages. The abstract book DOIs can be found on the "Author Information" page.
**Disclaimer:** Articles published in the journal HemaSphere exclusively reflect the opinions of the authors. The authors are responsible for all content in their abstracts including accuracy of the facts, statements, citing resources, etc.

### Summary/Conclusion

Dual targeted LV20.19 CAR T cell therapy resulted in high CR/CRi rate and encouraging PFS compared to currently available single targeted CARs in BTKi/BCL2i exposed CLL. Interestingly, dual targeting of CD19 and CD20 in CLL resulted in a high rate of IEC-HS, a phenomena that was IL-6 independent, and occurred at rates higher than seen with other B-cell histologies in this trial. With a lower dose of 1e6 cells/kg, responses were maintained with an improved safety profile. These data suggest dual targeting of CD20 and CD19 may improve CAR-T outcomes in R/R CLL.

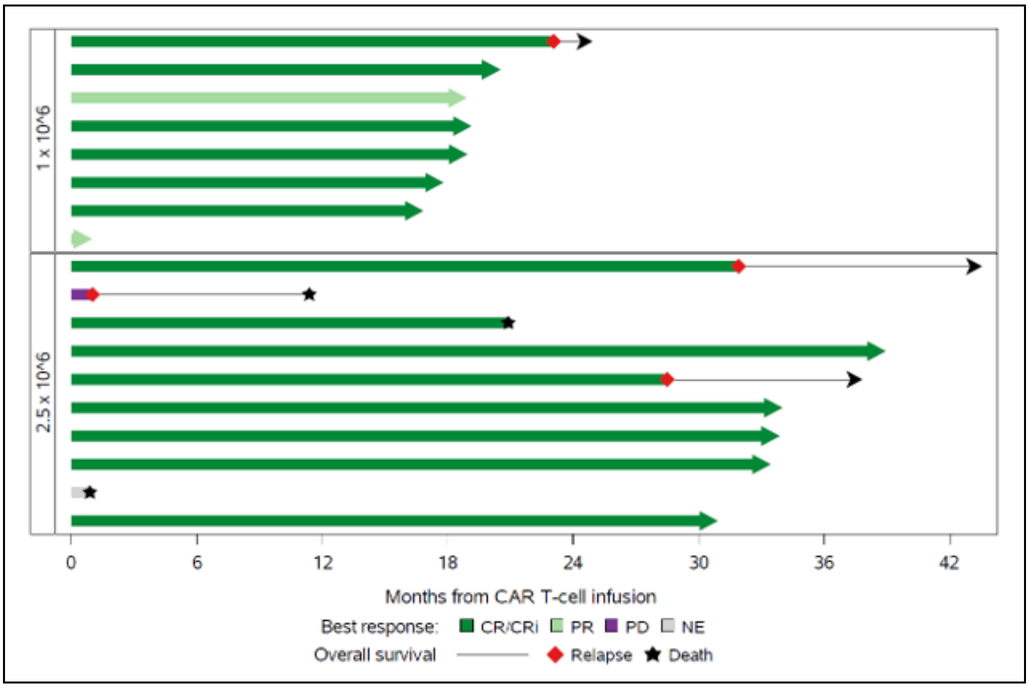

**Copyright Information:** (Online) ISSN: 2572-9241
©2026 The Author(s). HemaSphere is published by John Wiley & Sons Ltd on behalf of European Hematology Association. This is an open access Abstract Book distributed under the Attribution-NonCommercial-NoDerivs (CC BY-NC-ND), which allows third parties to download the articles and share them with others as long as they credit the author and the Abstract Book, but they cannot change the content in any way or use them commercially.
**Abstract Book Citations:** Authors, Title, HemaSphere, 2026;10:(S1):pages. The abstract book DOIs can be found on the "Author Information" page.
**Disclaimer:** Articles published in the journal HemaSphere exclusively reflect the opinions of the authors. The authors are responsible for all content in their abstracts including accuracy of the facts, statements, citing resources, etc.

### (S155) SINGLE-CELL GENETIC LINEAGE TRACING REVEALS PRE-EXISTING BLAST CRISIS-INITIATING CLONES AT CHRONIC PHASE CML DIAGNOSIS

**Topic:** 07. Chronic myeloid leukemia - Biology & translational research

Elaine Cao*[1], Vaidehi Krishnan[2], Naranie Shanmuganathan[3, 4], Xi Ren[1], Pavithra Shyamsunder[2], Verity Saunders[3], Shyam Prabhakar[1], Charles Chuah[2, 5], Tim Stuart[1], Timothy Hughes[3, 6], Susan Branford[6, 7], Jay Shin[1], Tiong Ong[2, 8]

*[1]Genome Institute of Singapore, Singapore, Singapore;[2]Duke-NUS Medical School, Cancer and Stem Cell Biology, Singapore, Singapore; [3]SAHMRI, Adelaide, Australia; [4]Royal Adelaide Hospital, Adelaide, Australia;[5]Singapore General Hospital, Singapore, Singapore; [6]University of Adelaide, School of Medicine, Adelaide, Australia;[7]SA Pathology, Adelaide, Australia; [8]Duke University, Department of Medicine, Durham, United States of America;*

### Background

Chronic phase (CP) CML can progress to the rare but highly lethal blast crisis (BC) when untreated or after TKI failure. Our published scRNA-seq atlas identified BC-like programs in CP patients who later transformed, including inflammatory signalling in rare lymphoid progenitors in lymphoid BC (LBC) and broad inflammatory activation across hematopoietic stem and progenitor cells (HSPC) in myeloid BC (MBC). However, the identity of the cell of transformation (COT) and whether it can be genetically traced at CP diagnosis as the founder of BC-initiating clones remain unclear.

### Aims

To definitively identify and genetically trace the COT in high-risk CP CML, and to define its molecular features using multi-omic single-cell (SC) analyses.

### Methods

We performed integrated SC multi-omics on 7 paired CP/BC samples (3 LBC, 4 MBC, with time to transformation [TTx] from 3-39 months [m]), combining short- and long-read scRNA-seq to infer copy number variants (CNV) and single nucleotide variants (SNV). Using Numbat and LongcallR, we reconstructed clonal lineage trees to map CP-BC evolution at SC resolution. We also integrated these data with 42 TKI-responsive CP controls to define COT features, and leveraged long-read data to identify BC-associated isoforms.

**Copyright Information:** (Online) ISSN: 2572-9241
©2026 The Author(s). HemaSphere is published by John Wiley & Sons Ltd on behalf of European Hematology Association. This is an open access Abstract Book distributed under the Attribution-NonCommercial-NoDerivs (CC BY-NC-ND), which allows third parties to download the articles and share them with others as long as they credit the author and the Abstract Book, but they cannot change the content in any way or use them commercially.
**Abstract Book Citations:** Authors, Title, HemaSphere, 2026;10:(S1):pages. The abstract book DOIs can be found on the "Author Information" page.
**Disclaimer:** Articles published in the journal HemaSphere exclusively reflect the opinions of the authors. The authors are responsible for all content in their abstracts including accuracy of the facts, statements, citing resources, etc.

**Results**

In CP diagnostic samples from patients who developed LBC, chr7 deletions (n=2, AU10 [TTx 4m], AU03 [TTx 6m]) or *IKZF1*deletions (n=1, AU166, TTx 7m) were detectable in pro-B cells and expanded to dominate at BC; in AU10, matched 7p/9p deletions were present in pro-B cells in both CP and BC (**Fig. 1A**). In AU03, the same lesions were also found in CP myeloid progenitors, implicating a rare upstream multipotent COT giving rise to both lymphoid and myeloid lesion-positive progeny. In MBC-destined patients, BC-initiating clones likewise arose from primitive compartments. AU123 (TTx 3m; Li-Fraumeni syndrome) showed rapid *TP53* LOH, with the COT mapping to a single pre-leukemic erythroid progenitor that progressed to erythroleukemic MBC with explosive, genome-wide aneuploidy within 3m. In LEU9 (TTx 17m; **Fig. 1B**) and AU02 (TTx 26m), a chr8 gain detected across multiple HSPC subsets at diagnosis implicated a multipotent COT. In AU6018 (TTx 39m), a single *U2AF1*-mutant HSC was identified as the COT (**Fig. 1C**). This ancestral clone subsequently bifurcated into two distinct subclones. Remarkably, although this patient was clinically diagnosed with MBC and lymphoid markers were only observed in CP, SC resolution analysis in BC captured both lymphoid and myeloid clones: one subclone acquired an *IKZF1* deletion, the other a *RUNX1* mutation, with both retaining the ancestral *U2AF1* mutation. U2AF1 splicing motif–signature analysis further supported this conclusion. Thus, in MBC, BC-initiating COTs commonly arose from primitive compartments (HSCs/multipotent progenitors), including clones bearing chr8 gains or *U2AF1* mutations. Notably, transcriptomic analyses revealed that most COTs, regardless of lineage, showed inflammatory pathway activation, distinguishing them from TKI-responsive controls and their own mature progeny.

**Summary/Conclusion**

Our findings show that BC CML is initiated by rare clones that are already present in CP, arising from primitive progenitors (often HSC/multipotent compartments), and seed both lymphoid and myeloid BC trajectories. Despite diverse initiating lesions, these COTs share a common inflammatory state, highlighting a potential vulnerability for intercepting transformation before clinical progression.

**Copyright Information:** (Online) ISSN: 2572-9241
©2026 The Author(s). HemaSphere is published by John Wiley & Sons Ltd on behalf of European Hematology Association. This is an open access Abstract Book distributed under the Attribution-NonCommercial-NoDerivs (CC BY-NC-ND), which allows third parties to download the articles and share them with others as long as they credit the author and the Abstract Book, but they cannot change the content in any way or use them commercially.
**Abstract Book Citations:** Authors, Title, HemaSphere, 2026;10:(S1):pages. The abstract book DOIs can be found on the "Author Information" page.
**Disclaimer:** Articles published in the journal HemaSphere exclusively reflect the opinions of the authors. The authors are responsible for all content in their abstracts including accuracy of the facts, statements, citing resources, etc.

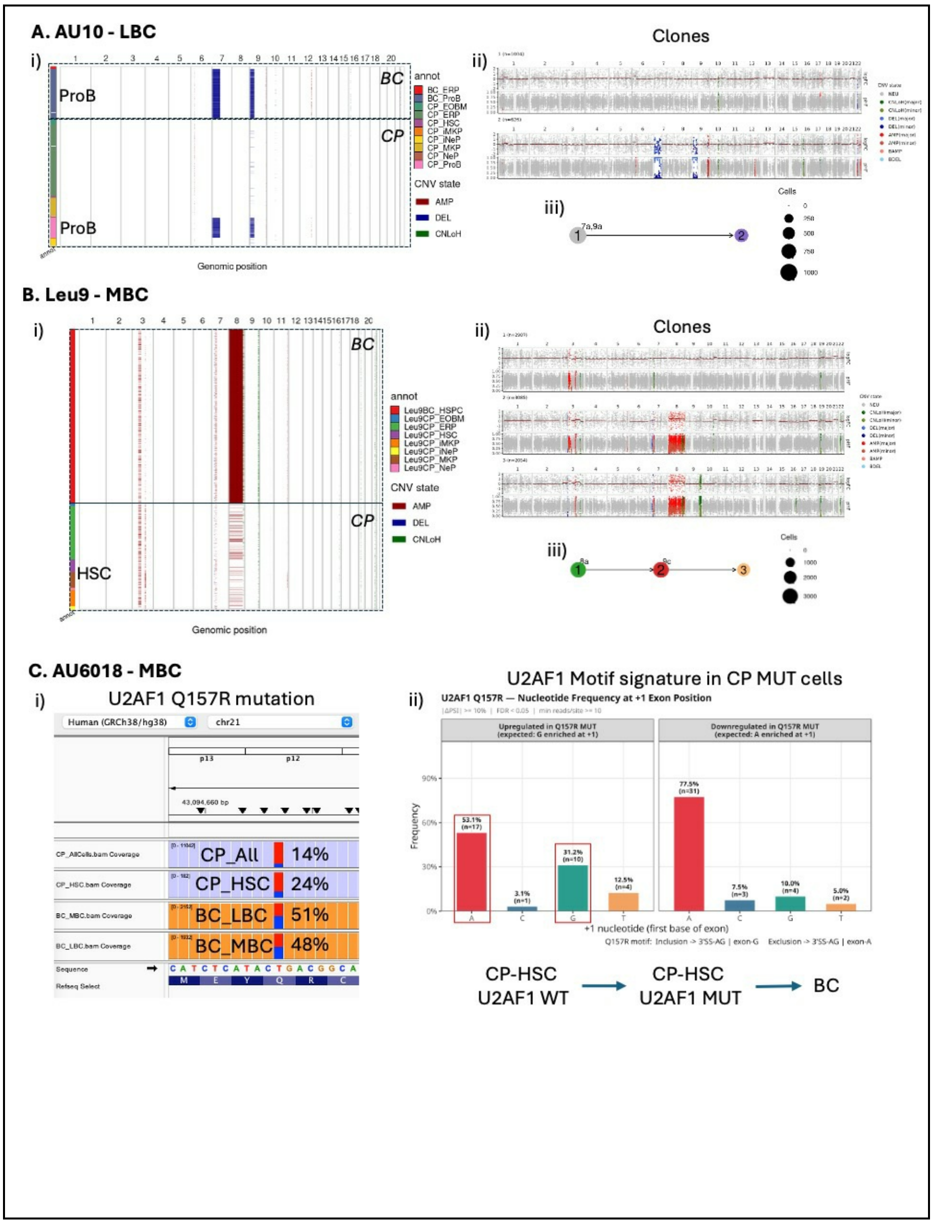

Figure 1. (A-B) Numbat CNV calling. (i) SC CNV landscape. The annotation column is colored by cell types in CP and BC samples. (ii) Pseudobulk CNV profile of each clone. Gray vertical bars represent centromeres and gap regions. (iii) Proposed clonal evolutionary lineage with genetic alterations. (A) AU10 patient; (B) Leu9 patient. (C) LongcallR mutation calling. (i) IGV view of the U2AF1 Q157R mutation in AU6018 CP and BC samples. (ii) U2AF1 splicing motif signature validation in CP cells. Bar plot showing the frequency of nucleotide at position +1 relative to the acceptor splice site AG. An enrichment of guanine (G) at this position is observed in cells with the U2AF1 Q157R mutation.

Copyright Information: (Online) ISSN: 2572-9241
©2026 The Author(s). HemaSphere is published by John Wiley & Sons Ltd on behalf of European Hematology Association. This is an open access Abstract Book distributed under the Attribution-NonCommercial-NoDerivs (CC BY-NC-ND), which allows third parties to download the articles and share them with others as long as they credit the author and the Abstract Book, but they cannot change the content in any way or use them commercially.
Abstract Book Citations: Authors, Title, HemaSphere, 2026;10:(S1):pages. The abstract book DOIs can be found on the "Author Information" page.
Disclaimer: Articles published in the journal HemaSphere exclusively reflect the opinions of the authors. The authors are responsible for all content in their abstracts including accuracy of the facts, statements, citing resources, etc.

### (S156) SINGLE-CELL TRANSCRIPTOMICS-GUIDED FLOW CYTOMETRIC ANALYSIS OF INFLAMED MEGAKARYOCYTIC AND LYMPHOID PROGENITORS AT DIAGNOSIS IDENTIFIES CHRONIC PHASE CML AT HIGH RISK FOR BLAST CRISIS TRANSFORMATION

**Topic:** 07. Chronic myeloid leukemia - Biology & translational research

Tiong Ong*[1, 2], Mengge Yu[1], Vaidehi Krishnan[1], Pavithra Shyamsunder[1], Kian Leong Lee[1], Florian Schmidt[3], Xi Ren[3], Charles Chuah[1, 4], Shyam Prabhakar[3], Verity Saunders[5], David Yeung[6], Naranie Shanmuganathan[5, 6], Agnes Yong[7, 8], Timothy Hughes[5, 9]

*[1]Duke-NUS Medical School, Cancer and Stem Cell Biology, Singapore, Singapore;[2]Duke University, Department of Medicine, Durham, United States of America; [3]Genome Institute of Singapore, Singapore, Singapore;[4]Singapore General Hospital, Singapore, Singapore; [5]SAHMRI, Adelaide, Australia; [6]Royal Adelaide Hospital, Adelaide, Australia;[7]University of Western Australia, Perth, Australia; [8]Royal Perth Hospital, Department of Haematology, Perth, Australia;[9]University of Adelaide, School of Medicine, Adelaide, Australia;*

### Background

Blast crisis (BC) remains the leading cause of CML-related mortality. While stem cell transplantation (SCT) in first chronic phase (CP1) could potentially increase survival rates among these high-risk patients, such a strategy awaits the development of robust and accurate predictors of future BC transformation.

### Aims

We set out to develop a clinically scalable multiparameter flow cytometry (MFC) assay to predict BC risk at CP diagnosis, and to determine the analytical and clinical validity of such an assay in the bone marrow and/or peripheral blood of CP patients at the time of diagnosis.

### Methods

First, we leveraged a single-cell RNA sequencing atlas of tyrosine kinase inhibitor (TKI) resistance to define transformation-linked cellular hallmarks. Using this dataset, we identified: (i) enrichment of a lineage-agnostic BC gene-expression program within lymphoid progenitors (LyP) and megakaryocyte progenitors (MKP), (ii) lineage skewing with aberrant LyP/MKP expansion, and (iii) IFNγ-STAT1-associated inflammation as features of high-risk CP patients. These features were consolidated into two standardized 8-colour MFC panels to quantify progenitor abundance and inflammatory readouts. Tube 1 (LyP inflammation) included antibodies against CD45, CD3, CD14, CD16, CD34, CD38, CD10, CD19, and IFITM1; Tube 2 (MKP inflammation) included antibodies against CD45, CD3, CD19, CD14, CD16, CD34, CD38, CD42A, and pSTAT1. Assay performance was evaluated in two independent diagnostic CP CML cohorts (total n=74). Cohort 1 (discovery; n=28; 14 BC progressions) used stringent outcome definitions to establish analytical validity and inform marker selection. Cohort 2 (independent validation; n=46; 8 BC progressions) tested performance in a more heterogeneous real-world population treated with imatinib, second-generation TKIs, or asciminib.

**Copyright Information:** (Online) ISSN: 2572-9241
©2026 The Author(s). HemaSphere is published by John Wiley & Sons Ltd on behalf of European Hematology Association. This is an open access Abstract Book distributed under the Attribution-NonCommercial-NoDerivs (CC BY-NC-ND), which allows third parties to download the articles and share them with others as long as they credit the author and the Abstract Book, but they cannot change the content in any way or use them commercially.
**Abstract Book Citations:** Authors, Title, HemaSphere, 2026;10:(S1):pages. The abstract book DOIs can be found on the "Author Information" page.
**Disclaimer:** Articles published in the journal HemaSphere exclusively reflect the opinions of the authors. The authors are responsible for all content in their abstracts including accuracy of the facts, statements, citing resources, etc.

### Results

In the outcome-enriched discovery cohort, combined assessment of LyP/MKP expansion and inflammatory activation perfectly separated CP patients who later progressed to BC from those with optimal long-term responses. In the independent validation cohort, refining the assay to quantify inflamed MKPs (pSTAT1$^{+}$ MKPs) rather than total MKP abundance maintained 100% sensitivity with 96% specificity across TKI therapies. Overall, MFC-based risk assessment outperformed Sokal score for BC prediction (AUC 0.912 vs 0.56; p<0.0001)(Figure 1). In multivariable Cox models adjusting for standard clinical factors, each biomarker remained independently associated with BC risk (all p<0.001).

### Summary/Conclusion

A diagnostic MFC assay capturing both lineage skewing and inflammatory activation within LyP/MKP compartments accurately identifies CP patients at high risk of BC transformation. Pending prospective multi-center validation and demonstration of clinical utility, this pan-TKI predictor could enable earlier risk-adapted monitoring and treatment intensification, including timely SCT evaluation in CP1.

**Figure 1: BC prediction performance (CP CML; n=62)**

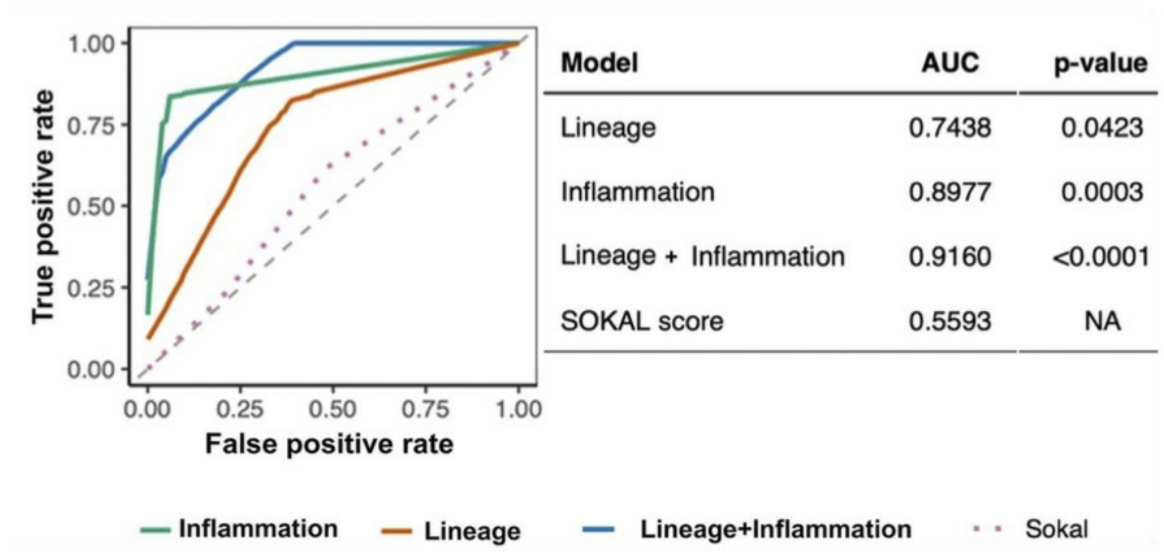

Copyright Information: (Online) ISSN: 2572-9241
©2026 The Author(s). HemaSphere is published by John Wiley & Sons Ltd on behalf of European Hematology Association. This is an open access Abstract Book distributed under the Attribution-NonCommercial-NoDerivs (CC BY-NC-ND), which allows third parties to download the articles and share them with others as long as they credit the author and the Abstract Book, but they cannot change the content in any way or use them commercially.
Abstract Book Citations: Authors, Title, HemaSphere, 2026;10:(S1):pages. The abstract book DOIs can be found on the "Author Information" page.
Disclaimer: Articles published in the journal HemaSphere exclusively reflect the opinions of the authors. The authors are responsible for all content in their abstracts including accuracy of the facts, statements, citing resources, etc.

### (S157) INFLUENCE OF NON-BCR::ABL1 SOMATIC MUTATIONS ON TYROSINE KINASE INHIBITOR (TKI) RESISTANCE IN CHRONIC MYELOID LEUKEMIA

**Topic:** 07. Chronic myeloid leukemia - Biology & translational research

Michele Ciboddo*[1, 2, 3], Aleksandr Ianevski[3, 4], Olli Dufva[1, 2, 3, 5], Hanna Lähteenmäki[1, 2, 3], Jay Klievink[1, 2, 3], Tiina Kasanen[1, 2, 3], Sara Gandolfi[1, 2, 3], Tero Aittokallio[3, 4, 6, 7], Shady Awad[1, 2, 3, 8], Satu Mustjoki[1, 2, 3]

*[1]University of Helsinki and Helsinki University Hospital, Hematology Research Unit Helsinki, Helsinki, Finland;[2]University of Helsinki, Translational Immunology Research Program, Helsinki, Finland; [3]iCAN Digital Precision Cancer Medicine Flagship, Helsinki, Finland; [4]University of Helsinki, Institute for Molecular Medicine Finland, FIMM, HiLIFE, Helsinki, Finland;[5]Wellcome Sanger Institute, Hinxton, Cambridge, United Kingdom; [6]Oslo University Hospital, Institute for Cancer Research, Department of Cancer Genetics, Oslo, Norway; [7]University of Oslo, Centre for Biostatistics and Epidemiology (OCBE), Faculty of Medicine, Oslo, Norway; [8]University of Oxford, MRC Molecular Haematology Unit, Weatherall Institute of Molecular Medicine, Radcliffe Department of Medicine and National Institute of Health Research (NIHR), Oxford, United Kingdom;*

**Background**

Chronic myeloid leukemia (CML) is a hematological malignancy driven by the BCR::ABL1 translocation. Most CML patients treated with tyrosine kinase inhibitors (TKIs) achieve deep molecular remission. Additional somatic mutations (non-BCR::ABL1) affect the clinical outcome and frequently associate with disease progression. Moreover, previous CRISPR knock-out (KO) screens have identified a group of genes, whose deletion confers a survival advantage during TKI treatment. The mechanisms by which these mutations/deletions drive TKI resistance and disease progression are still unclear.

**Aims**

1) To evaluate the transcriptional consequences of genetic perturbations in CML cells and their impact on sensitivity to TKI treatment (imatinib and asciminib). 2) To characterize the mechanisms of TKI resistance and determine whether specific somatic mutations generate novel molecular vulnerabilities.

**Methods**

We performed CRISPR-based genetic perturbations with single-cell RNA-sequencing (scRNAseq) readout (CROP-seq) in the K562 CML cell line model treated either with DMSO, imatinib or asciminib. Stable gene knock-out (KO) cell lines were generated for functional validations (qPCR, Western blotting) of CROP-seq findings for selected genes. A custom TKI-based combination drug screen was conducted to assess synergy. The project was conducted within the framework of the European Treatment and Outcome Study (EUTOS) for CML consortium.

**Copyright Information:** (Online) ISSN: 2572-9241
©2026 The Author(s). HemaSphere is published by John Wiley & Sons Ltd on behalf of European Hematology Association. This is an open access Abstract Book distributed under the Attribution-NonCommercial-NoDerivs (CC BY-NC-ND), which allows third parties to download the articles and share them with others as long as they credit the author and the Abstract Book, but they cannot change the content in any way or use them commercially.
**Abstract Book Citations:** Authors, Title, HemaSphere, 2026;10:(S1):pages. The abstract book DOIs can be found on the “Author Information” page.
**Disclaimer:** Articles published in the journal HemaSphere exclusively reflect the opinions of the authors. The authors are responsible for all content in their abstracts including accuracy of the facts, statements, citing resources, etc.

### Results

A custom CROP-Seq library was designed to target 35 genes, including common progression-associated (e.g., RUNX1, IKZF1, TP53) and TKI-resistance genes discovered by genome-wide CRISPR screens (e.g., KCTD5, APAF1, MED23). Reassuringly, some of the previously discovered TKI resistance genes (e.g., KCTD5) were enriched after the treatment with imatinib and asciminib confirming their role in TKI resistance. On the other hand, KO of frequently mutated genes in chronic phase CML such as *ASXL1, TET2* and *DNMT3A* did not induce significant resistance to either TKI in this short-term (24h) assay. Analysis of gene expression data revealed distinct perturbation-associated transcriptional profiles. Comparisons of these signatures in cells treated with either imatinib or asciminib identified shared hallmarks linked to TKI resistance including dysregulation of multiple pathways and biological processes, such as the DYRK3 pathway and proteostasis. Interestingly, CROP-seq revealed that *BCOR* mutation specifically confers resistance to asciminib but not imatinib. This finding was subsequently confirmed in individual gene-KO K562 cells. Next, we investigated whether asciminib resistance in BCOR-KO cells could be therapeutically targeted. Combination drug screening (imatinib or asciminib plus additional compounds) identified a synergistic effect between asciminib and the BCL-XL inhibitor A-1331852 specifically in BCOR-KO cells. Further proteomic and drug sensitivity testing experiments are ongoing to validate our results in primary patient samples.

### Summary/Conclusion

CROP-seq demonstrated that additional somatic mutations and TKI-resistance genes modify the transcriptional profile of CML cells and their sensitivity to imatinib and asciminib. We identified several differentially expressed pathways shared among deleterious somatic mutations and TKI-resistance genes. Notably, *BCOR* mutation was associated with resistance to asciminib but not imatinib. Moreover, BCOR-mutated CML exhibited a distinct sensitivity profile to combination treatment with asciminib and BCL-XL inhibitor.

**Copyright Information:** (Online) ISSN: 2572-9241
©2026 The Author(s). HemaSphere is published by John Wiley & Sons Ltd on behalf of European Hematology Association. This is an open access Abstract Book distributed under the Attribution-NonCommercial-NoDerivs (CC BY-NC-ND), which allows third parties to download the articles and share them with others as long as they credit the author and the Abstract Book, but they cannot change the content in any way or use them commercially.
**Abstract Book Citations:** Authors, Title, HemaSphere, 2026;10:(S1):pages. The abstract book DOIs can be found on the “Author Information” page.
**Disclaimer:** Articles published in the journal HemaSphere exclusively reflect the opinions of the authors. The authors are responsible for all content in their abstracts including accuracy of the facts, statements, citing resources, etc.

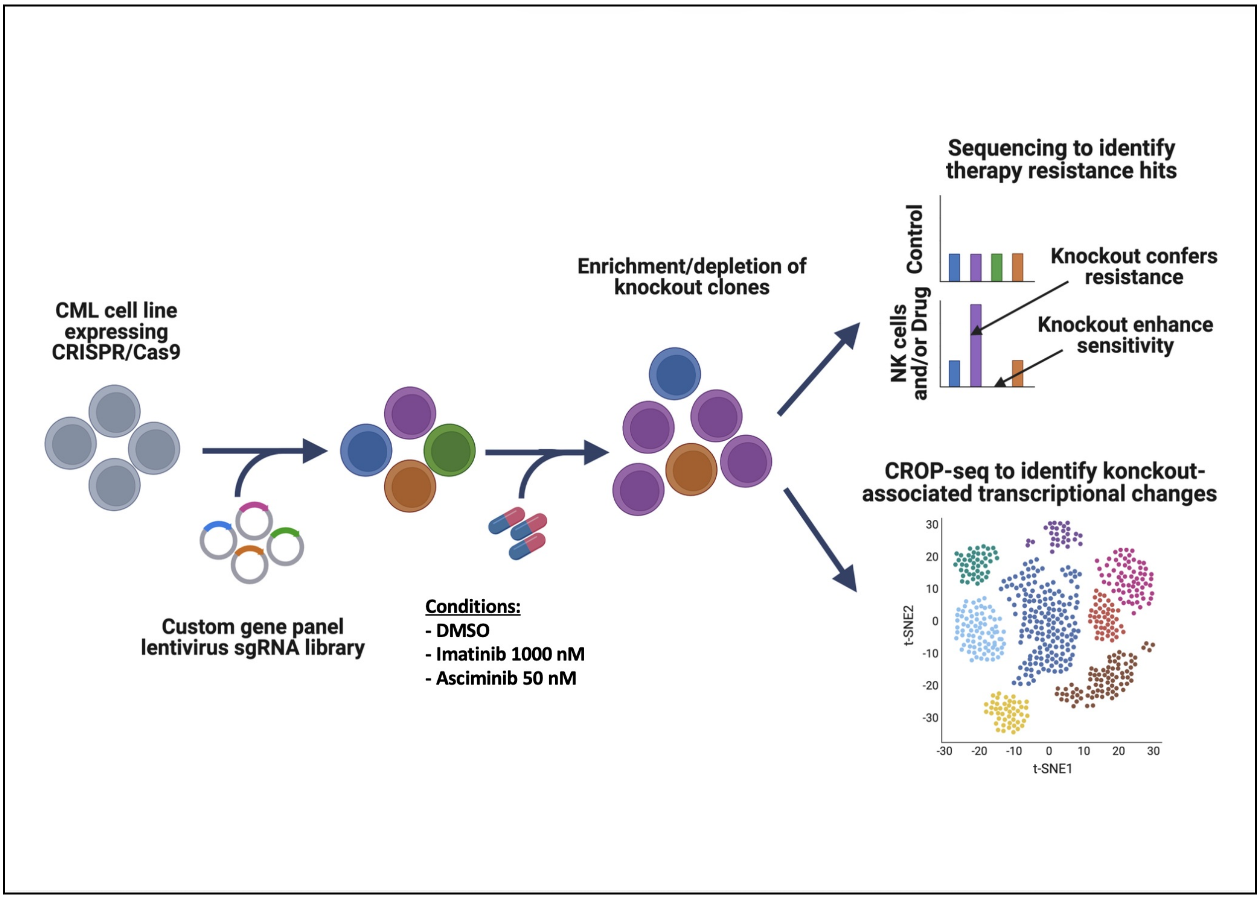

Schematic of CROP-seq experimental work-flow

**Copyright Information:** (Online) ISSN: 2572-9241
©2026 The Author(s). HemaSphere is published by John Wiley & Sons Ltd on behalf of European Hematology Association. This is an open access Abstract Book distributed under the Attribution-NonCommercial-NoDerivs (CC BY-NC-ND), which allows third parties to download the articles and share them with others as long as they credit the author and the Abstract Book, but they cannot change the content in any way or use them commercially.
**Abstract Book Citations:** Authors, Title, HemaSphere, 2026;10:(S1):pages. The abstract book DOIs can be found on the "Author Information" page.
**Disclaimer:** Articles published in the journal HemaSphere exclusively reflect the opinions of the authors. The authors are responsible for all content in their abstracts including accuracy of the facts, statements, citing resources, etc.

### (S158) MOLECULAR MECHANISM OF ZDHHC19-MEDIATED JAK1 PALMITOYLATION DRIVING TKIS RESISTANCE IN CML LEUKEMIA STEM CELLS

**Topic:** 07. Chronic myeloid leukemia - Biology & translational research

Teng Wang*[1], Siqi Luo[1], Xi Huang[1], Diguang Wen[1], Nan Zhang[1], Jianchuan Deng[1]

[1] *The second affiliated hospital of Chongqing Medical University, Department of Hematology, Chongqing, China;*

### Background

Tyrosine kinase inhibitors (TKIs) have dramatically improved the prognosis of chronic myeloid leukemia (CML) patients. However, leukemia stem cells (LSCs) persisting through BCR-ABL1-independent mechanisms remain a major cause of relapse and treatment failure. Emerging evidence implicates the bone marrow (BM) microenvironment and metabolic reprogramming in LSC survival and drug resistance. Nevertheless, the specific alterations in local fatty acid metabolism within the BM niche and their molecular mechanisms in CML LSCs are poorly understood.

### Aims

Our previous studies have found that the bone marrow of CML patients is abundant in palmitic acid and its esters. Thus, this study aims to systematically elucidate the relationship between aberrant palmitic acid (PA) metabolism in the CML BM microenvironment and LSC resistance to imatinib (IM), focusing on the molecular mechanism by which PA sustains the resistant phenotype, thereby providing a novel interventional strategy to overcome TKI resistance.

### Methods

The GC-MS was employed for metabolomic profiling of free fatty acids in BM supernatants from healthy donors and CML patients. Primary $CD34^+$ cells were isolated from CML patients using magnetic bead sorting. Combined with CML cell lines exhibiting differential IM sensitivity, we assessed the impact of a high-PA environment on IM response, cell proliferation, apoptosis, cell cycle, and colony-forming capacity in vitro. Integrated transcriptome sequencing and palmitoyl-proteomics were performed to identify resistance-related signaling pathways and key palmitoylated target proteins. Co-immunoprecipitation, Acyl-Biotin Exchange (ABE) assays, molecular docking, and in vivo xenograft models were used to validate the function of the key palmitoyltransferase in mediating resistance.

### Results

Exogenous PA significantly antagonized the inhibitory effects of IM on primary CML $CD34^+$ cells and resistant cell lines, reducing apoptosis, attenuating cell cycle arrest, and enhancing colony formation. Combining the broad-spectrum protein palmitoylation inhibitor 2-BP with IM exhibited significant synergistic anti-leukemic effects both in vitro and in vivo. Multi-omics analysis revealed that PA treatment remodeled the palmitoylation profile of CML $CD34^+$ cells and significantly enriched the IL-6/JAK/STAT3 signaling pathway, with JAK1 identified as a key hyper-palmitoylated target. Further screening identified the palmitoyltransferase ZDHHC19 as highly responsive to PA in resistant CML cells and forming a stable interaction with JAK1. Knockdown of ZDHHC19 significantly reduced JAK1 palmitoylation and STAT3 activation, enhanced IM efficacy against resistant CML $CD34^+$ cells and cell lines, and reduced tumor burden while prolonging survival in a xenograft mouse model.

**Copyright Information:** (Online) ISSN: 2572-9241
©2026 The Author(s). HemaSphere is published by John Wiley & Sons Ltd on behalf of European Hematology Association. This is an open access Abstract Book distributed under the Attribution-NonCommercial-NoDerivs (CC BY-NC-ND), which allows third parties to download the articles and share them with others as long as they credit the author and the Abstract Book, but they cannot change the content in any way or use them commercially.
**Abstract Book Citations:** Authors, Title, HemaSphere, 2026;10:(S1):pages. The abstract book DOIs can be found on the "Author Information" page.
**Disclaimer:** Articles published in the journal HemaSphere exclusively reflect the opinions of the authors. The authors are responsible for all content in their abstracts including accuracy of the facts, statements, citing resources, etc.

**Summary/Conclusion**

This study reveals that aberrant enrichment of PA in the CML BM niche is a key metabolic feature supporting LSC resistance to IM. Mechanistically, PA functions not primarily as an energy source but as a substrate for ZDHHC19-mediated JAK1 palmitoylation, sustaining persistent activation of the IL-6/JAK1/STAT3 signaling axis and promoting LSC survival and resistance. Targeting ZDHHC19 or the protein palmitoylation process effectively restores IM sensitivity in CML LSCs, offering a novel theoretical basis and potential therapeutic target for eradicating resistant leukemia stem cells and optimizing combination therapy strategies in CML.

**Copyright Information:** (Online) ISSN: 2572-9241
©2026 The Author(s). HemaSphere is published by John Wiley & Sons Ltd on behalf of European Hematology Association. This is an open access Abstract Book distributed under the Attribution-NonCommercial-NoDerivs (CC BY-NC-ND), which allows third parties to download the articles and share them with others as long as they credit the author and the Abstract Book, but they cannot change the content in any way or use them commercially.
**Abstract Book Citations:** Authors, Title, HemaSphere, 2026;10:(S1):pages. The abstract book DOIs can be found on the "Author Information" page.
**Disclaimer:** Articles published in the journal HemaSphere exclusively reflect the opinions of the authors. The authors are responsible for all content in their abstracts including accuracy of the facts, statements, citing resources, etc.

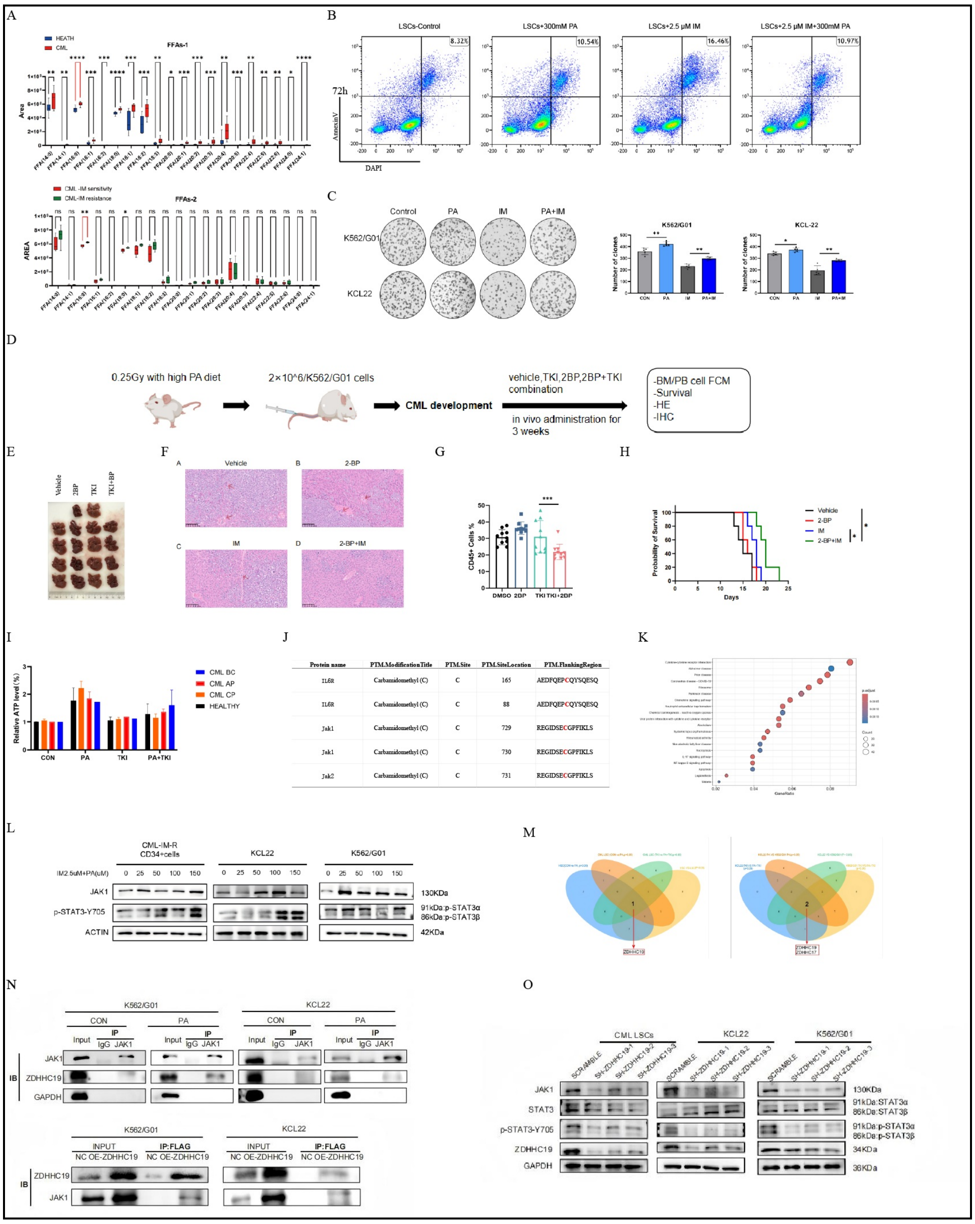

(A). PA and its esters abundant in the bone marrow microenvironment are associated with TKI resistance in CML patients. (B). PA Protects CML Leukemia Stem Cells from TKI-Induced Cytotoxicity. (C). We assessed the impact of a high-PA environment on colony-forming capacity of CML IM-resistance cell lines. (D-H). Combining the broad-spectrum protein palmitoylation inhibitor 2-BP with IM exhibited significant synergistic anti-leukemic effects in vivo. (I). PA had no significant effect on the energy metabolism (ATP levels) of hematopoietic stem/progenitor cells and LSCs, suggesting its function may extend beyond traditional metabolic support to involve more specific signaling regulatory mechanisms. (G-L). We first employed palmitoylomic analysis based on the GC-MS method in CML LSCs and identified specific palmitoylation modifications at sites C88/C165 of the IL-6 receptor (IL6R) and C729/C730/C731 of the JAK1 kinase. Combined transcriptomic sequencing and WB results showed that PA treatment significantly upregulated the expression abundance of genes related to the IL6-STAT3 signaling pathway. (M). To elucidate the mechanism of PA-mediated palmitoylation in CML leukemia stem cells, we focused on ZDHHC family palmitoyltransferases. Through bioinformatic screening combined with experimental validation, we found ZDHHC19 mRNA and protein expression levels were upregulated. (N-O). Co-IP analyse confirmed ZDHHC19 binds to JAK1 directly. By constructing a CML cell model with ZDHHC19 knockdown (shRNA efficiency >80%), we found that STAT3 Y705 phosphorylation and JAK1 protein expression were significantly reduced, suggesting that ZDHHC19 is involved in TKI resistance of CML leukemia stem cells by regulating STAT3 modification.

**Copyright Information:** (Online) ISSN: 2572-9241
©2026 The Author(s). HemaSphere is published by John Wiley & Sons Ltd on behalf of European Hematology Association. This is an open access Abstract Book distributed under the Attribution-NonCommercial-NoDerivs (CC BY-NC-ND), which allows third parties to download the articles and share them with others as long as they credit the author and the Abstract Book, but they cannot change the content in any way or use them commercially.
**Abstract Book Citations:** Authors, Title, HemaSphere, 2026;10:(S1):pages. The abstract book DOIs can be found on the "Author Information" page.
**Disclaimer:** Articles published in the journal HemaSphere exclusively reflect the opinions of the authors. The authors are responsible for all content in their abstracts including accuracy of the facts, statements, citing resources, etc.

### (S159) INTEGRATED GENOMIC AND TRANSCRIPTOMIC ANALYSES REVEAL BIOLOGICAL HETEROGENEITY OF ACCELERATED-PHASE CHRONIC MYELOID LEUKEMIA AND IDENTIFY A BLAST-PHASE-LIKE SUBGROUP

**Topic:** 07. Chronic myeloid leukemia - Biology & translational research

Xiaoshuai Zhang*[1], Haiyang Hu[2], Mei Bao[1], Qianfei Wang[2], Qian Jiang[1]

*[1]Peking University People's Hospital, Beijing, China;[2]Beijing Institute of Genomics, Chinese Academy of Sciences, China National Center for Bioinformation, Beijing, China;*

### Background

Accelerated-phase (AP) chronic myeloid leukemia (CML) is biologically heterogeneous and incompletely characterized, resulting in different clinical outcomes. This uncertainty led recent classifications to question its status as an independent disease phase.

### Aims

To characterize the biological heterogeneity of CML-AP and to identify high-risk molecular subgroup for risk stratification.

### Methods

An internal CML cohort (N=266; including newly-diagnosed CP [CP, n = 40], TKI-resistant CP [n = 38], TKI-sensitive CP [n = 8], de novo AP [n = 46], evolved AP [n = 100], de novo blast phase [BP, n = 3], evolved BP [n = 31]) and publicly available external cohorts (N = 327) with genomic (WGS/WES) and transcriptomic (bulk-RNAseq) profiling were analyzed.

### Results

De novo AP showed molecular features closer to CP than BP. Genomic analyses showed that the mutation spectrum of de novo AP closely resembled that of CP, with *ASXL1* as the most frequent mutation (21%) and low frequencies (<5%) of *EP300*, *RUNX1* and *KMT2D*. In contrast, BP was characterized by frequent *RUNX1* (50%), *ABL1* (32%) and *KMT2D* (30%) mutations. Although mutational burden in de novo AP was higher than in CP ($p$<0.001), mutation pattern remained distinct from BP. Transcriptomic analyses showed clear separation between CP and BP by principal component analysis and similarity-based clustering with de novo AP samples predominantly distributed within the CP expression space: AP with increased basophils showed high similarity to CP, while AP with increased blasts displayed partial divergence but remained distinct from BP.
Among evolved AP patients with transcriptomic data (n = 53), unsupervised analyses identified 2 subgroups: 22 (42%) clustered within the BP transcriptional space (BP-like AP), whereas 31 (58%) remained CP-like. BP-like AP demonstrated transcriptional features consistent with BP, including up-regulation of leukemic stemness and transformation-associated genes (e.g., *WT1*, *HOXA9* and *MECOM*) and down-regulation of granulocytic differentiation and inflammatory signatures. Consistently, stemness-related signatures were markedly enriched, and GSVA showed activation of cell cycle, DNA repair and hematopoietic stem cell pathways comparable to BP. Genomically, BP-like patients showed increased mutational burden and copy-number alterations with enrichment of transformation-associated mutations including *RUNX1* and *ASXL1*, and recurrent chromosomal gains and losses involving key regulators of cell cycle and DNA damage indicating increased genomic instability. These biological features were significantly-associated with clinical outcomes. Compared to CP-like AP patients, BP-like AP had significantly inferior 2-year transformation-free survival (TFS) of 35% *versus* 85% and overall survival (OS) of 28% *versus* 90% (both $p$<0.001).
To facilitate clinical application, the CP-to-BP transformation signatures were integrated using ssGSEA and further reduced to a 30-gene model (CML30) by LASSO. CML30 showed strong concordance with BP-like/CP-like classifications and predicted inferior TFS and OS (both $p$<0.001) in internal and external cohorts (C-statistics 0.85-0.91).

**Copyright Information:** (Online) ISSN: 2572-9241
©2026 The Author(s). HemaSphere is published by John Wiley & Sons Ltd on behalf of European Hematology Association. This is an open access Abstract Book distributed under the Attribution-NonCommercial-NoDerivs (CC BY-NC-ND), which allows third parties to download the articles and share them with others as long as they credit the author and the Abstract Book, but they cannot change the content in any way or use them commercially.
**Abstract Book Citations:** Authors, Title, HemaSphere, 2026;10:(S1):pages. The abstract book DOIs can be found on the "Author Information" page.
**Disclaimer:** Articles published in the journal HemaSphere exclusively reflect the opinions of the authors. The authors are responsible for all content in their abstracts including accuracy of the facts, statements, citing resources, etc.

### Summary/Conclusion

Integrated genomic and transcriptomic analyses demonstrate that CML-AP includes biologically distinct CP-like and BP-like subgroups associated with significantly different outcomes.  The CML30 score provides a practical tool for identifying high-risk patients and may support risk-adapted management in the TKI era.

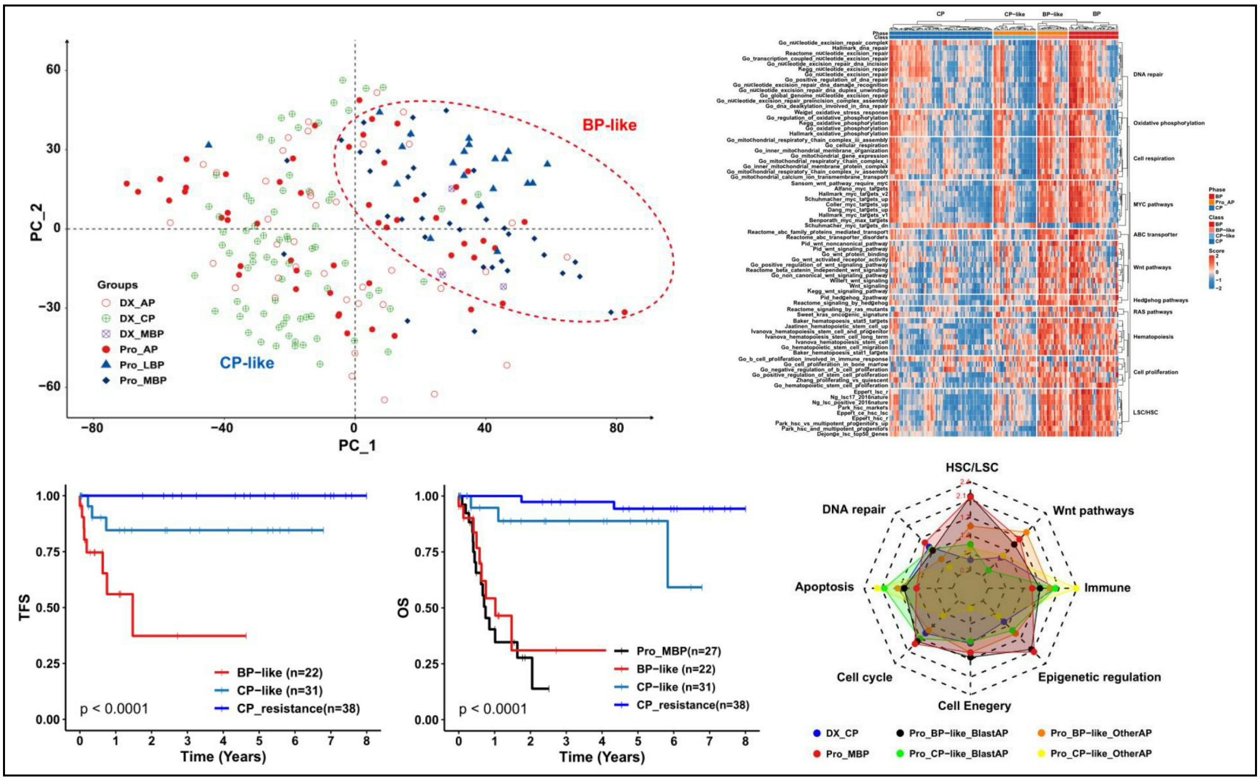

Figure

**Copyright Information:** (Online) ISSN: 2572-9241
©2026 The Author(s). HemaSphere is published by John Wiley & Sons Ltd on behalf of European Hematology Association. This is an open access Abstract Book distributed under the Attribution-NonCommercial-NoDerivs (CC BY-NC-ND), which allows third parties to download the articles and share them with others as long as they credit the author and the Abstract Book, but they cannot change the content in any way or use them commercially.
**Abstract Book Citations:** Authors, Title, HemaSphere, 2026;10:(S1):pages. The abstract book DOIs can be found on the "Author Information" page.
**Disclaimer:** Articles published in the journal HemaSphere exclusively reflect the opinions of the authors. The authors are responsible for all content in their abstracts including accuracy of the facts, statements, citing resources, etc.

### (S160) ASC4FIRST WK 144 ANALYSIS: CONTINUED SUPERIOR EFFICACY AND FAVORABLE SAFETY OF ASCIMINIB VS INVESTIGATOR-SELECTED TYROSINE KINASE INHIBITORS IN NEWLY DIAGNOSED CHRONIC PHASE CHRONIC MYELOID LEUKEMIA

**Topic:** 08. Chronic myeloid leukemia - Clinical

Timothy Hughes*[1], Naoto Takahashi[2], Richard Larson[3], Ghayas Issa[4], Felice Bombaci[5], Jianxiang Wang[6], Dw Kim[7], Dennis Kim[8], Jiří Mayer[9], Yeow Tee Goh[10], Philipp Le Coutre[11], Inho Kim[12], Gabriel Etienne[13], Kamel Malek[14], Tracey McCulloch[14], Justin Wahlstrom[15], Lillian Yau[14], Sumit Sant[16], Jorge Cortes[17], Andreas Hochhaus[18]

*[1]South Australian Health and Medical Research Institute and Adelaide University, Adelaide, SA, Australia;[2]Akita University, Akita City, Japan; [3]University of Chicago, Chicago, IL, United States of America; [4]University of Texas MD Anderson Cancer Center, Houston, TX, United States of America; [5]CML Patients Group, CML Advocates Network, Turin, Italy; [6]State Key Laboratory of Experimental Hematology, National Clinical Research Center for Blood Diseases, Institute of Hematology and Blood Diseases Hospital, Chinese Academy of Medical Sciences and Peking Union Medical College, Tianjin, China; [7]Uijeongbu Eulji Medical Center, Geumo-dong, Uijeongbu-si, South Korea; [8]Princess Margaret Cancer Centre, University Health Network, University of Toronto, Toronto, ON, Canada; [9]Hematology and Oncology, University Hospital Brno, Brno, Czechia; [10]Singapore General Hospital, Singapore, Singapore; [11]Charité-Universitätsmedizin Berlin, Berlin, Germany; [12]Seoul National University Hospital, Biomedical Research Institute, Cancer Research Institute, Seoul National University College of Medicine, Seoul, South Korea; [13]Institut Bergonié, Bordeaux, France; [14]Novartis Pharma AG, Basel, Switzerland; [15]Novartis Pharmaceuticals Corporation, East Hanover, NJ, United States of America; [16]Novartis Healthcare Private Limited, Hyderabad, India; [17]Georgia Cancer Center at Augusta University, Augusta, GA, United States of America; [18]Hematology/Oncology, Universitätsklinikum Jena, Jena, Germany;*

### Background

In the ASC4FIRST (NCT04971226) primary (wk 48) (Hochhaus A et al. *N Engl J Med.* 2024) and key secondary (wk 96) (Cortes JE et al. *Blood.* 2025) analyses, asciminib (ASC) had superior efficacy and improved safety/tolerability vs investigator-selected tyrosine kinase inhibitors (IS-TKIs: imatinib [IMA] and second generation [2G] TKIs), and a better benefit-risk profile vs 2G TKIs. ASC was approved for newly diagnosed chronic myeloid leukemia in chronic phase (CML-CP) in numerous countries.

### Aims

The ASC4FIRST primary and key secondary endpoints were major molecular response (MMR) rates at wks 48 and 96, respectively, with $ASC^{IMA}$ vs $IS\text{-}TKI^{IMA}$ and ASC vs all IS-TKIs. MMR rates at wks 48 and 96 with $ASC^{2G}$ vs $IS\text{-}TKI^{2G}$ were secondary endpoints. We present an analysis of long-term efficacy, safety, and tolerability at wk 144 (cutoff: Sep 29, 2025).

### Methods

In this multicenter, open-label, phase 3 study, patients with newly diagnosed CML-CP, who provided informed consent, were randomized 1:1 to receive ASC 80 mg once daily or an IS-TKI at label doses, stratified by ELTS risk category and prerandomization-selected TKI (IMA or a 2G TKI).
For event-free survival, an event was defined as treatment failure per ELN2020, confirmed loss of MMR in 2 consecutive tests, discontinuation due to adverse events (AEs), disease progression, or death.

Copyright Information: (Online) ISSN: 2572-9241
©2026 The Author(s). HemaSphere is published by John Wiley & Sons Ltd on behalf of European Hematology Association. This is an open access Abstract Book distributed under the Attribution-NonCommercial-NoDerivs (CC BY-NC-ND), which allows third parties to download the articles and share them with others as long as they credit the author and the Abstract Book, but they cannot change the content in any way or use them commercially.
Abstract Book Citations: Authors, Title, HemaSphere, 2026;10:(S1):pages. The abstract book DOIs can be found on the “Author Information” page.
Disclaimer: Articles published in the journal HemaSphere exclusively reflect the opinions of the authors. The authors are responsible for all content in their abstracts including accuracy of the facts, statements, citing resources, etc.

### Results

Patients were randomized to ASC (n=201; $ASC^{IMA}$, n=101; $ASC^{2G}$, n=100) or IS-TKIs (n=204; $IS\text{-}TKI^{IMA}$, n=102; $IS\text{-}TKI^{2G}$, n=102). Median follow-up was 36.2 mo with $ASC^{IMA}$ vs 35.3 mo with $IS\text{-}TKI^{IMA}$ and 39.2 mo with $ASC^{2G}$ vs 38.5 mo with $IS\text{-}TKI^{2G}$. At cutoff, more patients were ongoing treatment with $ASC^{IMA}$ vs $IS\text{-}TKI^{IMA}$ (81.2% vs 50.0%) and $ASC^{2G}$ vs $IS\text{-}TKI^{2G}$ (76.0% vs 61.8%).

MMR rates at wk 144 continued to be superior with $ASC^{IMA}$ vs $IS\text{-}TKI^{IMA}$ (79.2% vs 47.1%; treatment difference, 32.6%; unadjusted nominal $P$<.001) and higher with $ASC^{2G}$ vs $IS\text{-}TKI^{2G}$ (75.0% vs 59.8%; treatment difference, 15.2%; unadjusted nominal $P$=.01) (**Figure**).

$MR^4$ and $MR^{4.5}$ rates at wk 144 were higher with $ASC^{IMA}$ vs $IS\text{-}TKI^{IMA}$ (58% vs 33%; 44% vs 20%) and $ASC^{2G}$ vs $IS\text{-}TKI^{2G}$ (53% vs 39%; 41% vs 29%).

Long-term outcomes with $ASC^{IMA}$ vs $IS\text{-}TKI^{IMA}$ and $ASC^{2G}$ vs $IS\text{-}TKI^{2G}$ included probability of event-free survival 144 wk since randomization (83% vs 63%; 87% vs 75%), and estimated progression-free survival (97% vs 93%; 99% vs 97%) and overall survival (99% vs 96%; 99% vs 99%), 3 years since randomization.

No new *BCR::ABL1* mutations emerged with ASC after wk 96; 1 each emerged with IMA and nilotinib.

Safety/tolerability remained favorable with ASC vs IMA vs 2G TKIs including grade ≥3 AEs (49% vs 52% vs 63%), AEs leading to discontinuation (6% vs 13% vs 14%), and AEs leading to dose adjustment/interruption (37% vs 44% vs 63%); exposure-adjusted arterial occlusive event rates were 1.3, 0.5, and 1.1 cases per 100 patient-treatment years, respectively. Median exposure duration was 37.4 mo, 33.8 mo, and 37.3 mo, respectively. No deaths occurred during treatment or ≤30 d after last dose.

### Summary/Conclusion

At wk 144, ASC had consistently higher molecular response rates and improved safety/tolerability vs all comparators (IMA and 2G TKIs), with no new treatment-emergent mutations. Together with wk 48 and 96, this analysis reinforces ASC's improved benefit-risk profile and supports ASC as a treatment option for newly diagnosed CML-CP.

© 2026 American Society of Clinical Oncology, Inc. Reused with permission. This abstract was accepted and previously presented at the 2026 ASCO Annual Meeting. All rights reserved.

**Copyright Information:** (Online) ISSN: 2572-9241
©2026 The Author(s). HemaSphere is published by John Wiley & Sons Ltd on behalf of European Hematology Association. This is an open access Abstract Book distributed under the Attribution-NonCommercial-NoDerivs (CC BY-NC-ND), which allows third parties to download the articles and share them with others as long as they credit the author and the Abstract Book, but they cannot change the content in any way or use them commercially.
**Abstract Book Citations:** Authors, Title, HemaSphere, 2026;10:(S1):pages. The abstract book DOIs can be found on the "Author Information" page.
**Disclaimer:** Articles published in the journal HemaSphere exclusively reflect the opinions of the authors. The authors are responsible for all content in their abstracts including accuracy of the facts, statements, citing resources, etc.

**Figure.** MMR rates at week 144

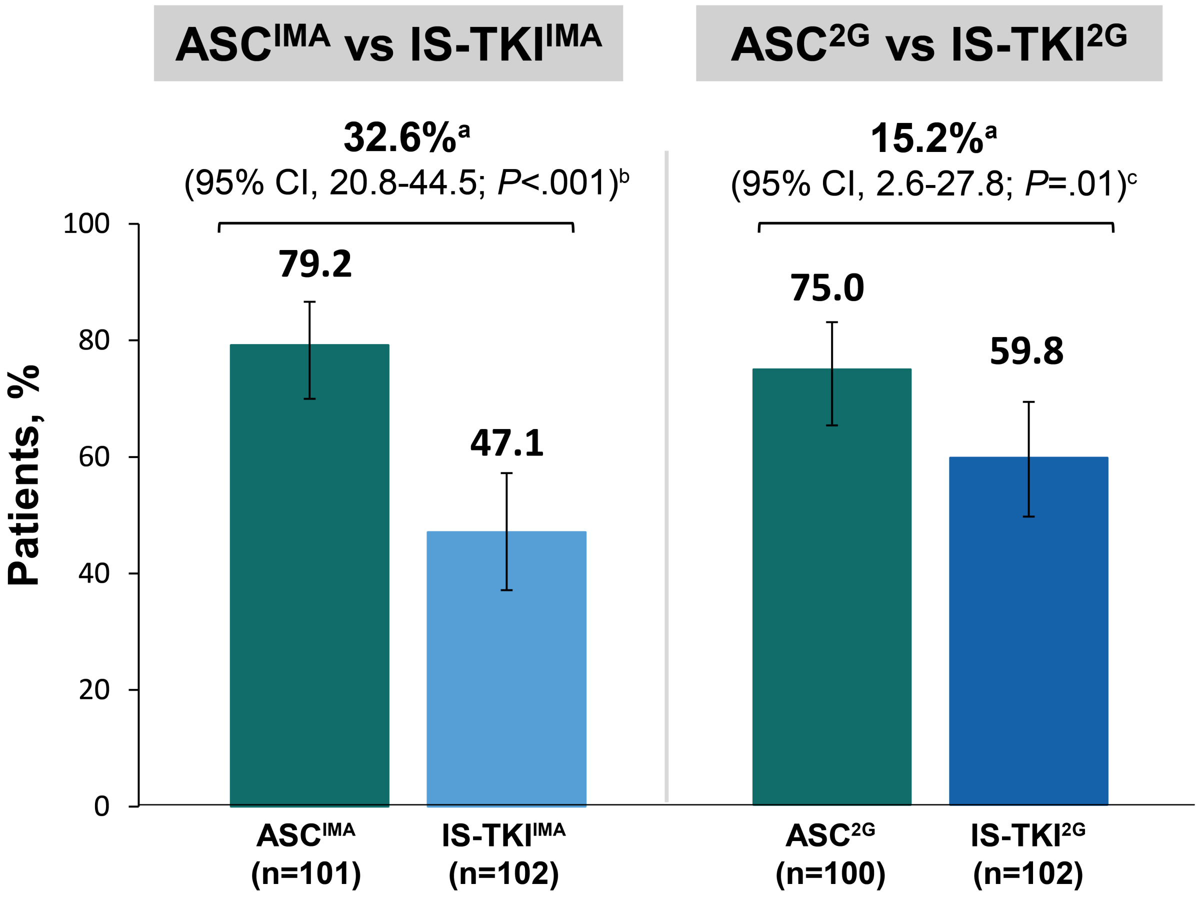

2G, second generation; ASC, asciminib; IMA, imatinib; ELTS, EUTOS Long Term Survival; EUTOS, European Treatment and Outcome Study; IRT, Interactive Response Technology; IS-TKI, investigator-selected tyrosine kinase inhibitor. Error bars represent 95% CIs. [a] The common treatment difference and its 95% CI were estimated using the Mantel-Haenszel method after stratifying for baseline ELTS risk groups (IRT data). [b] Comparisons were not α-controlled; therefore, a false positive rate ≤2.5% can not be guaranteed. [c] The study was not designed to formally confirm statistical significance for this endpoint.

**Copyright Information:** (Online) ISSN: 2572-9241
©2026 The Author(s). HemaSphere is published by John Wiley & Sons Ltd on behalf of European Hematology Association. This is an open access Abstract Book distributed under the Attribution-NonCommercial-NoDerivs (CC BY-NC-ND), which allows third parties to download the articles and share them with others as long as they credit the author and the Abstract Book, but they cannot change the content in any way or use them commercially.
**Abstract Book Citations:** Authors, Title, HemaSphere, 2026;10:(S1):pages. The abstract book DOIs can be found on the "Author Information" page.
**Disclaimer:** Articles published in the journal HemaSphere exclusively reflect the opinions of the authors. The authors are responsible for all content in their abstracts including accuracy of the facts, statements, citing resources, etc.

### (S161) PONATINIB CONSOLIDATION AND DISCONTINUATION IN CML PATIENTS AFTER FAILED FIRST TFR ATTEMPT: UPDATED TFR OUTCOMES AND CHIP MUTATION ANALYSIS FROM THE RESTOP TRIAL

**Topic:** 08. Chronic myeloid leukemia - Clinical

Almudena Zaratiegui[1], Gonzalo Carreño Gómez-Tarragona*[1], Ricardo Sanchez[1], Francisca Ferrer Marin[2], Blanca Xicoy Cirici[3], M Teresa Gómez Casares[4], Fermin Sanchez Guijo[5], Concepción Ruiz Nuño[6], Santiago Ossorio Prendes[7], Maria Raquel De Paz Arias[8], Ana Rosell Mass[9], Elvira Mora[10], Maria Alicia Senin Magan[11], Juan Carlos Hernandez Boluda[12], Rosa María Ayala Díaz[1, 13], Valentin Garcia Gutierrez[14], Joaquín Martinez-Lopez[1, 13, 15]

*[1]Hospital 12 de Octubre, Haematology and Hemotherapy, Madrid, Spain; [2]Hospital Universitario Morales Meseguer, Department of Hematology and Hemotherapy, Murcia, Spain; [3]Catalan Institute of Oncology – Badalona, Department of Hematology and Hemotherapy, Badalona, Spain; [4]Hospital Universitario de Gran Canaria Doctor Negrín, Hematology and Hemotherapy, Gran Canaria, Spain; [5]Hospital Universitario de Salamanca, Hematology and Hemotherapy, Salamanca, Spain; [6]Hospital Universitario Regional de Málaga, Hematology and Hemotherapy,, Málaga, Spain; [7]Hospital Universitario Gregorio Marañón, Hematology and Hemotherapy, Madrid, Spain; [8]Hospital Universitario La Paz, Hematology and Hemotherapy, Madrid, Spain; [9]Hospital Universitario Virgen de la Victoria, Hematology and Hemotherapy, Málaga, Spain; [10]Hospital Universitario y Politécnico La Fe, Hematology and Hemotherapy, Valencia, Spain; [11]Catalan Institute of Oncology – Hospital Duran i Reynals, Hematology and Hemotherapy, Hospitalet de Llobregat, Spain; [12]Hospital Clínico Universitario de Valencia, Hematology and Hemotherapy, Valencia, Spain; [13]Complutense University of Madrid, Madrid, Spain; [14]Hospital Universitario Ramón y Cajal, Hematology and Hemotherapy, Madrid, Spain; [15]National Cancer Research Centre (CNIO), Madrid, Spain;*

### Background

Approximately 30-50% of CML patients are eligible for TKI discontinuation, with 50-60% achieving sustained TFR, decreasing to 20-30% in second attempts. Ponatinib may help deepen molecular response before a second discontinuation. During TKI therapy, Ph- negative CHIP clones may emerge as the CML clone is suppressed, occurring in 10-15% of patients in remission.

### Aims

To evaluate MMR and MR4 maintenance during 52 weeks following ponatinib consolidation in patients who failed a prior TFR attempt, assess safety, and characterize CHIP mutation evolution and its impact on TFR outcomes and cardiovascular toxicity.

### Methods

ResToP is a phase II, multicenter, open-label, non-comparative trial enrolling chronic-phase CML patients who failed a first TKI discontinuation and regained MR4 for ≥1 year. Patients received ponatinib 15 mg/day for 2 years, followed by treatment withdrawal. Molecular response was monitored via BCR::ABL1 RT-qPCR monthly for 28 weeks, then quarterly until week 52. NGS using a 63-gene panel covering genes associated with CHIP and myeloid malignancies was performed at baseline and end of ponatinib consolidation (NCT04160546).

**Copyright Information:** (Online) ISSN: 2572-9241
©2026 The Author(s). HemaSphere is published by John Wiley & Sons Ltd on behalf of European Hematology Association. This is an open access Abstract Book distributed under the Attribution-NonCommercial-NoDerivs (CC BY-NC-ND), which allows third parties to download the articles and share them with others as long as they credit the author and the Abstract Book, but they cannot change the content in any way or use them commercially.
**Abstract Book Citations:** Authors, Title, HemaSphere, 2026;10:(S1):pages. The abstract book DOIs can be found on the “Author Information” page.
**Disclaimer:** Articles published in the journal HemaSphere exclusively reflect the opinions of the authors. The authors are responsible for all content in their abstracts including accuracy of the facts, statements, citing resources, etc.

**Results**

Of 40 enrolled patients, 34 started ponatinib consolidation; 12 discontinued early (9 treatment-related adverse events, 3 unrelated). In this updated analysis, 22 patients entered the TFR phase. With a median follow-up of 51 weeks (IQR 46.3-53) post-discontinuation, 77.3% (17/22) maintained MMR and 59.1% (13/22) maintained MR4. Only 1/4 patients with confirmed MR4 loss required TKI restart. The TFR rate, defined as MMR maintenance without TKI restart regardless of MR4 loss, was 72.7% per protocol and 47.1% per intention-to-treat. No progressions occurred.
Regarding CHIP analysis, 13 variants were detected in 9/34 patients (26.5%) initiating consolidation. All 9 patients entered the TFR phase; 3 lost confirmed MR4 (75% of all MR4 losses) without requiring TKI restart, and 1 lost MMR (20% of all MMR losses). Among patients achieving TFR, 50% (8/16) had CHIP variants. MMR loss (11.1% vs 30.8%), MR4 loss (44.4% vs 38.5%), and TFR rates (88.9% vs 61.5%) were similar between patients with and without CHIP, with no significant differences.
DNMT3A was most frequent (N=5), followed by ASXL1, KRAS, JAK2, and KIT (N=1 each), all pathogenic/likely pathogenic. SAMD9, SH2B3, PPM1D, and BCORL1 (N=1 each) were VUS. No variants emerged or disappeared during treatment; 53.8% (7/13) increased in allelic frequency (median +1.4% [IQR 0.8-1.5]), 38.5% (5/13) decreased (median -1.5% [IQR 1.4-1.6]), and 7.7% (1/13) remained stable.
Regarding safety, 76.5% (26/34) reported ponatinib-related adverse events; 19.2% (5/26) had CHIP variants. Grade ≥3 events occurred in 17.6% of patients, including 3 vascular occlusive events (stroke, MI and peripheral ischemia), none in CHIP-positive patients. Two of these had predisposing cardiovascular risk factors. The most frequent grade 1-2 toxicities were hypertension (14.7%), hepatotoxicity, arthralgia, myalgia (11.8% each). One death unrelated to therapy was reported. No association between CHIP and cardiovascular adverse events was observed.

**Summary/Conclusion**

Ponatinib consolidation achieved high TFR rates (72.7% PP, 47.1% ITT) in patients who failed prior discontinuation. Although CHIP variants were detected in 26.5% of patients, no association with TFR outcomes or cardiovascular toxicity was observed. CHIP mutations may coexist with successful TFR without impacting cardiovascular safety in the ponatinib setting.

**Copyright Information:** (Online) ISSN: 2572-9241
©2026 The Author(s). HemaSphere is published by John Wiley & Sons Ltd on behalf of European Hematology Association. This is an open access Abstract Book distributed under the Attribution-NonCommercial-NoDerivs (CC BY-NC-ND), which allows third parties to download the articles and share them with others as long as they credit the author and the Abstract Book, but they cannot change the content in any way or use them commercially.
**Abstract Book Citations:** Authors, Title, HemaSphere, 2026;10:(S1):pages. The abstract book DOIs can be found on the “Author Information” page.
**Disclaimer:** Articles published in the journal HemaSphere exclusively reflect the opinions of the authors. The authors are responsible for all content in their abstracts including accuracy of the facts, statements, citing resources, etc.

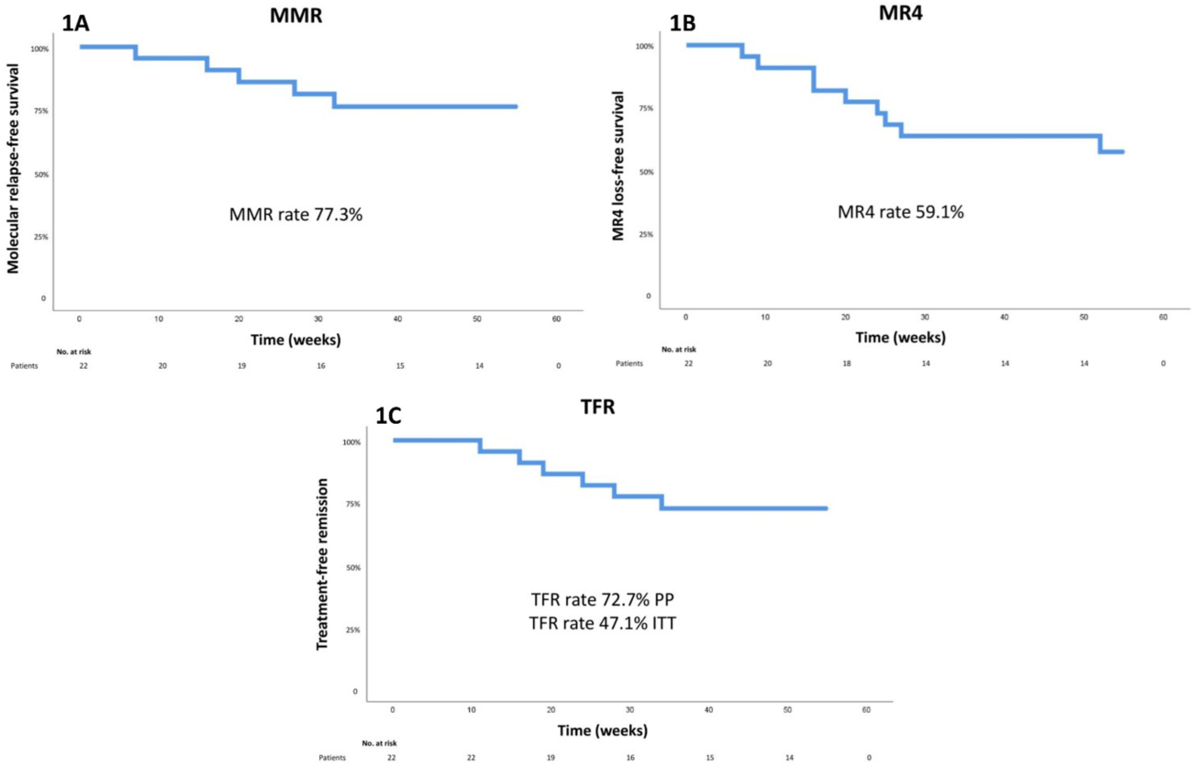

**Figure 1. Kaplan-Meier estimates of molecular response maintenance and treatment-free remission following ponatinib discontinuation.** (A) Cumulative probability of maintaining major molecular response (MMR) over 52 weeks post-discontinuation. (B) Cumulative probability of maintaining molecular response grade 4 (MR4) over 52 weeks post-discontinuation. (C) Treatment-free remission (TFR) rates analysed per protocol (PP) and intention-to-treat (ITT) populations.

**Copyright Information:** (Online) ISSN: 2572-9241
©2026 The Author(s). HemaSphere is published by John Wiley & Sons Ltd on behalf of European Hematology Association. This is an open access Abstract Book distributed under the Attribution-NonCommercial-NoDerivs (CC BY-NC-ND), which allows third parties to download the articles and share them with others as long as they credit the author and the Abstract Book, but they cannot change the content in any way or use them commercially.
**Abstract Book Citations:** Authors, Title, HemaSphere, 2026;10:(S1):pages. The abstract book DOIs can be found on the "Author Information" page.
**Disclaimer:** Articles published in the journal HemaSphere exclusively reflect the opinions of the authors. The authors are responsible for all content in their abstracts including accuracy of the facts, statements, citing resources, etc.

### (S162) HIGH RATES OF SUCCESSFUL SECOND STOP ATTEMPTS IN PATIENTS WITH CHRONIC MYELOID LEUKEMIA: RESULTS OF THE NAUT TRIAL

**Topic:** 08. Chronic myeloid leukemia - Clinical

Katharina Kohlbrenner[1], Ali Albeer[2], Jeroen Janssen[3, 4], Marcel Lorch[1], Alexander Kiani[5, 6], Andreas Burchert[7], Peter Herhaus[8], Mathias Hänel[9], Cornelius F. Waller[10], Holger Hebart[11], Tim Henrik Brümmendorf[12, 13], Christian Junghanss[14], Birgitta Vogt[15], Florian Heidel[16], Paul La Rosée[17], Haifa Kathrin Al-Ali[18], Andreas Hochhaus[19], Alice Fabarius[1], Luis Erbsland[1], Sebastian Kreil[1], Wolf-Karsten Hofmann[1], Markus Pfirrmann[2], Susanne Saussele*[1]

*[1]Heidelberg University, Medical Faculty Mannheim, Department of Hematology and Medical Oncology, Mannheim, Germany; [2]Ludwig-Maximilians-Universität München, Faculty of Medicine, Institute for Medical Information Processing, Biometry, and Epidemiology, Munich, Germany; [3]Radboud University Medical Center, Department of Hematology, Nijmegen, The Netherlands; [4]Vrije Universiteit Amsterdam, Amsterdam UMC, Cancer Center Amsterdam, Department of Hematology, Amsterdam, The Netherlands; [5]Klinikum Bayreuth, Klinik für Onkologie und Hämatologie, Bayreuth, Germany;[6]Comprehensive Center Erlangen EMN, Erlangen, Germany; [7]Marburg University, University Hospital Giessen and Marburg, Department of Hematology and Oncology, Marburg, Germany; [8]Technical University of Munich, University Hospital rechts der Isar, Department of Internal Medicine III, Munich, Germany; [9]Klinikum Chemnitz, Klinik für Innere Medizin III, Chemnitz, Germany; [10]University of Freiburg, Medical Center, Department of Medicine I, Freiburg, Germany; [11]Kliniken Ostalb, Stauferklinikum Schwäbisch Gmünd, Klinik für Innere Medizin, Schwerpunkt Hämatologie / Internistische Onkologie, Mutlangen, Germany; [12]RWTH Aachen University, Medical Faculty, Department of Hematology, Oncology, Hemostaseology and Stem Cell Transplantation, Aachen, Germany; [13]Center for Integrated Oncology - Aachen Bonn Cologne Duesseldorf (CIO-ABCD), Aachen, Germany; [14]University of Rostock, Rostock University Medical Center, Department of Internal Medicine, Clinic and Polyclinic for Hematology, Hemostaseology, Oncology, Stem Cell Therapy, and Palliative Medicine, Rostock, Germany; [15]Medizinisches Versorgungszentrum (MVZ) am Klinikum Esslingen, Onkologische Schwerpunktpraxis, Esslingen, Germany; [16]Hannover Medical School, Hematology, Hemostasis, Oncology and Stem Cell Transplantation, Hannover, Germany; [17]Schwarzwald-Baar Klinikum, Klinik für Innere Medizin II, Villingen-Schwenningen, Germany; [18]University Hospital Halle, Krukenberg Cancer Center Halle, Halle (Saale), Germany; [19]Universitätsklinikum Jena, Klinik für Innere Medizin II, Abteilung für Hämatologie und Internistische Onkologie, Jena, Germany;*

### Background

Treatment-free remission (TFR) is an established goal in the management of patients (pts) with chronic myeloid leukemia (CML). So far, data on tyrosine kinase inhibitor (TKI) discontinuation after one or more unsuccessful stop attempts are limited.

### Aims

The NAUT (Nilotinib After First or Second Unsuccessful Treatment Discontinuation) study is a multicenter prospective trial established to determine whether a second or third attempt at treatment discontinuation in pts with CML offers a realistic probability of sustained TFR.

**Copyright Information:** (Online) ISSN: 2572-9241
©2026 The Author(s). HemaSphere is published by John Wiley & Sons Ltd on behalf of European Hematology Association. This is an open access Abstract Book distributed under the Attribution-NonCommercial-NoDerivs (CC BY-NC-ND), which allows third parties to download the articles and share them with others as long as they credit the author and the Abstract Book, but they cannot change the content in any way or use them commercially.
**Abstract Book Citations:** Authors, Title, HemaSphere, 2026;10:(S1):pages. The abstract book DOIs can be found on the “Author Information” page.
**Disclaimer:** Articles published in the journal HemaSphere exclusively reflect the opinions of the authors. The authors are responsible for all content in their abstracts including accuracy of the facts, statements, citing resources, etc.

### Methods

CML pts in chronic phase ≥ 18 years were eligible after one or two prior unsuccessful stopping attempts and ≥ 1-year retreatment. After enrollment, pts received nilotinib for at least 2 years. In case of intolerability, switching to any TKI was allowed. Here we report results of the pre-planned interim analyses. The primary objective was to assess the duration of major molecular response (MMR, BCR::ABL1 ≤ 0.1 on International Scale (IS)) or better after a second or third TKI discontinuation in pts with ≥ 3 years of prior TKI treatment including ≥ 2 years of nilotinib treatment and stable $MR^4$ (BCR::ABL1 ≤ 0.01% (IS)) for at least one year and $MR^{4.5}$ (BCR::ABL1 ≤ 0.0032% (IS)) for at least 6 months. Secondary objectives included the assessment of adverse events or serious adverse events under nilotinib treatment. Hypothesis testing was performed at 12 months after TKI discontinuation for the interim analysis. The null hypothesis was defined as molecular recurrence (MRec)-free survival probability ≤ 15%. MRec-free survival was calculated from the date of second or third stop of TKI treatment until the date of loss of MMR. The study is registered as NCT02917720.

### Results

77 pts were screened and 75 pts fulfilled the inclusion/exclusion criteria. 29 (39%) pts were male, median age at diagnosis was 46 years (IQR 36-55) and at study entry 57 years (IQR 48-66). ELTS risk score of 56 eligible pts was low for 37 (66%), intermediate for 10 (18%) and high for 9 (16%) pts. 73 pts had one stop and two pts two unsuccessful stops prior to inclusion. The most recent stop attempt was 39 months (IQR 28-50) and retreatment with any TKI 27 months (IQR 18-42) in median before study entry. Median on-study TKI treatment duration was 24 months (IQR 24-27), including 24 months (IQR 24-25) nilotinib. During nilotinib treatment, 56 adverse events with grade 3 or 4 were reported for 48 pts, most commonly hypertension (n=12). No new nilotinib-related safety aspects occurred. 60 pts were eligible to be included in the TFR dataset for the interim analysis (seven pts were not screened for TFR: ongoing TKI-therapy (n=1), withdrawal of consent (n=2), and study discontinuation due to nilotinib intolerance (n=4)). In addition, 7 pts were screened for TFR but did not start an TFR attempt and one patient restarted TKI treatment without relapse. At 12 months after stopping TKI, 35/60 (58.3%) pts maintained at least MMR, whereas 25/60 (41.7%) had lost MMR. Median time to loss of MMR was 3.2 months (IQR 2.9 – 5.3) resulting in a MRec-free survival probability of 58.3% (95% CI [44.9%; 70.9%]) ($p < 0.0001$). Thus, the null-hypothesis could be rejected. Of the 25 pts with TKI restart, 24 regained at least $MR^4$; one patient was lost to follow-up.

### Summary/Conclusion

A second TKI discontinuation attempt in the NAUT trial was successful in a substantial proportion of pts and appears feasible under the given preconditions. No new safety signals were observed under nilotinib treatment.

**Copyright Information:** (Online) ISSN: 2572-9241
©2026 The Author(s). HemaSphere is published by John Wiley & Sons Ltd on behalf of European Hematology Association. This is an open access Abstract Book distributed under the Attribution-NonCommercial-NoDerivs (CC BY-NC-ND), which allows third parties to download the articles and share them with others as long as they credit the author and the Abstract Book, but they cannot change the content in any way or use them commercially.
**Abstract Book Citations:** Authors, Title, HemaSphere, 2026;10:(S1):pages. The abstract book DOIs can be found on the “Author Information” page.
**Disclaimer:** Articles published in the journal HemaSphere exclusively reflect the opinions of the authors. The authors are responsible for all content in their abstracts including accuracy of the facts, statements, citing resources, etc.

### (S163) EFFICACY AND SAFETY OF ADDING LOW DOSE TYROSINE KINASE INHIBITORS (TKIS) FOR PATIENTS WITH CHRONIC MYELOID LEUKEMIA WHO DO NOT ACHIEVE OPTIMAL RESPONSE OR A DEEP MOLECULAR REMISSION (ALERT CML)

**Topic:** 08. Chronic myeloid leukemia - Clinical

Jay Yang*[1], Michael Mauro[2], Lisa Rein[3], Srinivas Tantravahi[4], James Thompson[5], Arielle Baim[3], Sonia Moldonado-Schmidt[3], Kathryn Flynn[3], Yan Gao[3], Anthony M. Hunter[6], Jerald Radich[7], Ehab Attalah[3], Jorge Cortes[8]

*[1]Karmanos Cancer Institute, Detroit, United States of America;[2]Memorial Sloan Kettering Cancer Center, New York, United States of America; [3]Medical College of Wisconsin, Milwaukee, United States of America;[4]University of Utah, Salt Lake City, United States of America; [5]Roswell Park Cancer Center, Buffalo, United States of America;[6]Winship Cancer Institute of Emory University, Atlanta, United States of America; [7]Fred Hutchinson Cancer Center, Seattle, United States of America;[8]Georgia Cancer Center, Augusta, United States of America;*

### Background

Asciminib, an allosteric inhibitor of *BCR::ABL1*, has the potential to promote early deeper molecular response (DMR) with reduced toxicity in patients with newly diagnosed chronic phase chronic myeloid leukemia (CP-CML). Asciminib may be combined with low dose ATP-competitive TKIs (lowTKI) to further enhance the depth of molecular response in patients who fail to achieve DMR or do not meet optimal response criteria per the European LeukemiaNet (ELN).

### Aims

The primary study endpoint is the rate of MR4 or better by 12 months.

### Methods

This is an ongoing, multicenter phase 2, investigator-initiated, single-arm, open-label study (NCT05143840) within the H Jean Khoury Cure CML Consortium (HJKC3). Patients received either asciminib 40 mg orally twice daily or 80 mg once daily. Patients who did not achieve MR4.5 after 24 months on asciminib, experienced treatment failure at any time or had a warning response at 12 months (ELN 2020) were offered the addition of lowTKI (imatinib 300 mg, dasatinib 50 mg or nilotinib 300 mg daily). Patient reported outcomes were collected at multiple timepoints using NIH PROMIS and PRO-CTCAE measures. With a planned enrollment of 100 subjects, the study is powered at 86% to detect a 13% absolute improvement in the rate of MR4 or better at 12 months from 25% to 38%, estimated by the Wald test.

**Copyright Information:** (Online) ISSN: 2572-9241
©2026 The Author(s). HemaSphere is published by John Wiley & Sons Ltd on behalf of European Hematology Association. This is an open access Abstract Book distributed under the Attribution-NonCommercial-NoDerivs (CC BY-NC-ND), which allows third parties to download the articles and share them with others as long as they credit the author and the Abstract Book, but they cannot change the content in any way or use them commercially.
**Abstract Book Citations:** Authors, Title, HemaSphere, 2026;10:(S1):pages. The abstract book DOIs can be found on the "Author Information" page.
**Disclaimer:** Articles published in the journal HemaSphere exclusively reflect the opinions of the authors. The authors are responsible for all content in their abstracts including accuracy of the facts, statements, citing resources, etc.

### Results

Between April 2022 to February 2026, 75 patients were enrolled. Data cutoff was February 3, 2026. The median age was 50 years (range 21-79) and 40% were female. Sokal score was low, intermediate, high and unknown in 36%, 25%, 8% and 31% of patients, respectively. Of the 44 patients for whom NGS results were available, 16% had a variant detected: *ASXL1* (6.9%), *DDX41* (2.3%), *DNMT3A* (2.3%), *NRAS* (2.3%), *RUNX1* (2.3%). With a median follow up of 18.8 months, 67 (89%) patients remained on study; 58 (77%) on single agent and 9 (12%) on combination therapy. Reason for study discontinuation were adverse events (5), patient decision (2) and treatment failure (1). MR4 or better by 12 months (primary endpoint) and 24 months was achieved in 43% and 76% of patients, respectively. As of this analysis, 9 patients have started lowTKI: 6 (67%) for a warning response after 12 months, 2 (22%) for not achieving MR4.5 after 24 months and 1 (11%) for treatment failure. The TKI added was dasatinib 50 mg in all of them. At the time of adding lowTKI, *BCR::ABL1* was 0.1-1% in 7 (78%), and 0.01-0.0032% in 2 patients. After a median duration on combination therapy of 5.53 months (range 2.76-7.37), 5 (55%) patients had an improvement in *BCR::ABL1* levels. Both patients who added low dose TKI for not achieving MR4.5 at 24 months achieved MR4.5. Of the 7 patients with a *BCR::ABL1* 0.1-1%, 3 had improvement to a level between 0.01-0.1%. During combination therapy, one patient developed a grade 3 lung infection, one patient developed grade 2 thrombocytopenia, and all other AEs reported were grade 1. No patients have discontinued the lowTKI.

### Summary/Conclusion

The addition of lowTKI to asciminib was associated with an improved response in 55% of patients. This approach was feasible and tolerable and no patients discontinued lowTKI. Updated data will be presented at the meeting.

| Level of response | At Month 6 N=59 | At Month 12 N=46 | At Month 24 N=17 |
|---|---|---|---|
| MR3 or better | 59% | 76% | 100% |
| MR4 or better | 29% | 43% | 76% |
| MR4.5 | 15% | 28% | 35% |

Table 1: Response on single agent asciminib

**Copyright Information:** (Online) ISSN: 2572-9241
©2026 The Author(s). HemaSphere is published by John Wiley & Sons Ltd on behalf of European Hematology Association. This is an open access Abstract Book distributed under the Attribution-NonCommercial-NoDerivs (CC BY-NC-ND), which allows third parties to download the articles and share them with others as long as they credit the author and the Abstract Book, but they cannot change the content in any way or use them commercially.
**Abstract Book Citations:** Authors, Title, HemaSphere, 2026;10:(S1):pages. The abstract book DOIs can be found on the "Author Information" page.
**Disclaimer:** Articles published in the journal HemaSphere exclusively reflect the opinions of the authors. The authors are responsible for all content in their abstracts including accuracy of the facts, statements, citing resources, etc.

### (S164) ENABLE: UPDATED EFFICACY AND SAFETY RESULTS OF ELVN-001, A NOVEL SELECTIVE ATP-COMPETITIVE INHIBITOR OF BCR::ABL1, IN PATIENTS WITH PREVIOUSLY TREATED CP-CML

**Topic:** 08. Chronic myeloid leukemia - Clinical

Dennis Kim*[1], Fabian Lang[2], Koji Sasaki[3], Dragana Milojkovic[4], Dw Kim[5], Michael Mauro[6], Maria Raquel De Paz Arias[7], Susanne Saussele[8], Franck Emmanuel Nicolini[9], Nicole Blijlevens[10], Michael Heinrich[11], Naranie Shanmuganathan[12], Philipp Le Coutre[13], Sarah Altmeyer[14], Elvira Mora[15], Valerie Coiteux[16], Gabriel Etienne[17], Yingsi Yang[18], Qi Wang[18], Brianna Hoffner[18], M. Damiette Smit[18], Andreas Hochhaus[19]

*[1]Princess Margaret Cancer Centre, University Health Network, University of Toronto, Toronto, Canada;[2]Goethe University Hospital, Frankfurt, Germany; [3]The University of Texas MD Anderson Cancer Center, Houston, United States of America; [4]Hammersmith Hospital, Imperial College NHS Trust, London, United Kingdom;[5]Uijeongbu Eulji Medical Center, Uijeongbu-si, South Korea; [6]Memorial Sloan Kettering Cancer Center, New York, United States of America;[7]Hospital Universitario La Paz, Madrid, Spain; [8]Medizinische Fakultät Mannheim der Universität, Heidelberg, Germany;[9]Centre Léon Bérard & INSERM U1052 CRCL, Lyon, France; [10]Department of Hematology, Radboud University Medical Centre, Nijmegen, The Netherlands;[11]Knight Cancer Institute, Portland, United States of America; [12]Royal Adelaide Hospital, Adelaide, Australia; [13]Charité-Universitätsmedizin Berlin, Berlin, Germany; [14]Universitätsklinikum des Saarlandes, Homburg, Germany; [15]Hospital Universitario y Politecnico La Fe, Valencia, Spain; [16]Service des Maladies du Sang Hôpital Huriez - CHRU de Lille, Lille, France;[17]Institut Bergonie - Centre Regional de Lutte Contre Le Cancer de Bordeaux et Sud Ouest, Bordeaux, France; [18]Enliven Therapeutics, Boulder, United States of America; [19]Universitätsklinikum Jena, Jena, Germany;*

### Background

ELVN-001 is a potent BCR::ABL1 tyrosine kinase inhibitor (TKI). Its unique binding mode within the BCR::ABL1 ATP-binding site results in selective ABL1 inhibition with broad pre-clinical activity against resistance mutations, including T315I and emerging asciminib-resistant mutations. The ELVN-001 pharmacokinetic (PK) profile supports once daily dosing with or without food, which, in addition to low potential for drug-drug interactions (DDIs), addresses key challenges with currently available TKIs. The ENABLE study (NCT05304377) is a phase 1 study of ELVN-001 in patients (pts) with previously treated chronic phase (CP)-CML. Data from the dose escalation phase 1a previously demonstrated a favorable safety and tolerability profile with encouraging anti-CML activity. Here, we first report preliminary data from the dose expansion phase 1b of the study.

### Aims

Primary objectives are safety and tolerability of ELVN-001 in pts with CML. Secondary objectives include PK and preliminary activity in CML with/without BCR::ABL1 T315I mutation.

### Methods

Pts with previously treated CP-CML received ELVN-001 orally at doses from 10-160 mg daily. Efficacy was evaluated as major molecular response (MMR) rate by 24 weeks. Pts were evaluable for efficacy if assessed at 24-weeks, achieved MMR prior to 24-weeks (if not in MMR at baseline), maintained MMR for at least 70 days (if in MMR at baseline), or discontinued treatment with ≥ one post baseline assessment prior to 24 weeks. All pts were included for safety analyses; pts from the phase 1b cohorts with typical *BCR::ABL1* transcripts and without T315I mutation were included for the efficacy analysis.

**Copyright Information:** (Online) ISSN: 2572-9241
©2026 The Author(s). HemaSphere is published by John Wiley & Sons Ltd on behalf of European Hematology Association. This is an open access Abstract Book distributed under the Attribution-NonCommercial-NoDerivs (CC BY-NC-ND), which allows third parties to download the articles and share them with others as long as they credit the author and the Abstract Book, but they cannot change the content in any way or use them commercially.
**Abstract Book Citations:** Authors, Title, HemaSphere, 2026;10:(S1):pages. The abstract book DOIs can be found on the “Author Information” page.
**Disclaimer:** Articles published in the journal HemaSphere exclusively reflect the opinions of the authors. The authors are responsible for all content in their abstracts including accuracy of the facts, statements, citing resources, etc.

**Results**

As of 22 Dec 2025, 141 pts were enrolled (3 re-enrolled): 132/9 with typical/atypical *BCR::ABL1* transcript; 28 with BCR::ABL1 mutations (including 11 with T315I and 12 with mutations associated with resistance to asciminib). Median age was 58 y (range 19-82). The majority of pts (67%) received ≥3 prior unique TKIs, with 24% ≥5; 61% had prior asciminib, 38% prior ponatinib; 64% discontinued their last prior TKI due to lack of efficacy. Median duration of exposure was 31.7 weeks (0.1-145). The majority of pts (76%) remain on treatment, 9 (6.4%) discontinued due to adverse events, 20 (14%) due to lack of efficacy, five (3.5%) due to other reasons. The majority of TEAEs were Grade 1/2. Grade ≥3 TEAEs were reported in 46 (33%) pts, with thrombocytopenia (6%), neutropenia (6%) and lipase elevation (5%) as the most common. Dose reductions due to TEAEs occurred in <10% of pts. In the initial 80 mg QD phase 1b cohort (n=19), all pts were evaluable for efficacy by 24 weeks: 9/19 (47%) pts were in MMR, with 6/16 (38%) achieving MMR. In the randomized 60 mg and 120 mg QD phase 1b cohorts (n=41), 26 pts were evaluable for efficacy by 24 weeks, reflecting their more recent enrollment: 18/26 (69%) pts were in MMR, with 9/17 (53%) achieving MMR. All pts who received prior asciminib in phase 1b had an improved or stable response category by week 24; 14/27 (52%) pts were in MMR, with 8/21 (38%) achieving MMR. Across all phase 1b cohorts, 100% of evaluable pts in MMR at enrollment maintained or deepened their response.

**Summary/Conclusion**

With longer follow-up and more pts enrolled, ELVN-001 continues to demonstrate robust anti-CML activity despite the heavily pretreated patient population. In addition, ELVN-001 maintains favorable safety and tolerability, consistent with its selective kinase profile.

**Copyright Information:** (Online) ISSN: 2572-9241
©2026 The Author(s). HemaSphere is published by John Wiley & Sons Ltd on behalf of European Hematology Association. This is an open access Abstract Book distributed under the Attribution-NonCommercial-NoDerivs (CC BY-NC-ND), which allows third parties to download the articles and share them with others as long as they credit the author and the Abstract Book, but they cannot change the content in any way or use them commercially.
**Abstract Book Citations:** Authors, Title, HemaSphere, 2026;10:(S1):pages. The abstract book DOIs can be found on the “Author Information” page.
**Disclaimer:** Articles published in the journal HemaSphere exclusively reflect the opinions of the authors. The authors are responsible for all content in their abstracts including accuracy of the facts, statements, citing resources, etc.

### (S165) A NEW MOUSE MODEL OF JMML HIGHLIGHTS DIFFERENTIAL SUSCEPTIBILITY OF EMBRYONIC HEMATOPOIETIC STEM/PROGENITOR CELLS TO ONCOGENIC KRASG12D AND IDENTIFIES INFLAMMATION AS A TARGETABLE THERAPEUTIC AXIS

**Topic:** 09. Myelodysplastic syndromes - Biology & translational research

Emanuele Azzoni*[1], Giulia Quattrini[1], Cristiana Barone[1], Tiphanie Durfort[1], Alessandro Muratore[1], Gloria Zambelli[1], Filipa Timoteo Ferreira[1], Thea Milanesi[1], Mahdieh Naghavi Alhosseini[1], Silvia Bombelli[2], Deborah D'aliberti[1], Cristina D'orlando[1], Barbara Vergani[1], Raffaella Meneveri[1], Silvia Brunelli[1], Rocco Giovanni Piazza[1]

*[1]University of Milano-Bicocca, School of Medicine and Surgery, Milano, Italy;[2]Fondazione Human Technopole, Milan, Italy;*

### Background

Juvenile Myelomonocytic Leukemia (JMML) is a rare pediatric myelodysplastic/myeloproliferative neoplasm driven by mutations in RAS pathway genes, frequently acquired *in utero*. Developing faithful JMML models remains challenging, likely due to the importance of the developmental context in which oncogenic events occur. Allogeneic hematopoietic stem cell (HSC) transplantation remains the only curative therapy, highlighting the need to identify novel targeted approaches.

### Aims

We aimed to: i) Determine how the developmental timing and cellular context of prenatal mutation acquisition influence leukemic transformation in JMML; ii) Characterize the cellular and molecular processes underlying disease onset and progression and iii) Identify and test novel therapeutic targets.

### Methods

We recently developed a tamoxifen-inducible lineage tracing strategy able to capture and discriminate the main embryonic hematopoietic waves, including erythro-myeloid progenitors (EMPs), fetal-restricted hematopoietic stem/progenitor cells (HSPCs) and embryonic HSCs with the same mouse model (Barone et al., *Nature Cardiovascular Research*, in press). Building on this strategy, we generated a new transgenic mouse model that can selectively target Kras$^{G12D}$ to distinct subsets of HSPCs during embryonic development ( $Cdh5\text{-}CreER^{T2}::Kras^{LSL\text{-}G12D}::R26^{tdTomato}$).

**Copyright Information:** (Online) ISSN: 2572-9241
©2026 The Author(s). HemaSphere is published by John Wiley & Sons Ltd on behalf of European Hematology Association. This is an open access Abstract Book distributed under the Attribution-NonCommercial-NoDerivs (CC BY-NC-ND), which allows third parties to download the articles and share them with others as long as they credit the author and the Abstract Book, but they cannot change the content in any way or use them commercially.
**Abstract Book Citations:** Authors, Title, HemaSphere, 2026;10:(S1):pages. The abstract book DOIs can be found on the "Author Information" page.
**Disclaimer:** Articles published in the journal HemaSphere exclusively reflect the opinions of the authors. The authors are responsible for all content in their abstracts including accuracy of the facts, statements, citing resources, etc.

**Results**

Activation of $Kras^{G12D}$ in EMPs resulted in low penetrance and late onset myeloproliferative disease, indicating limited involvement in JMML. In contrast, targeting $Kras^{G12D}$ to fetal-restricted HSPCs or embryonic HSCs resulted in embryonic lethal anemia, implicating RAS signaling in fetal erythropoiesis. A reduced 4-hydroxytamoxifen activation dose enabled us to circumvent embryonic lethality by restricting $Kras^{G12D}$ to smaller subsets of HSPCs or HSCs. This led to the postnatal development of an aggressive myeloproliferative disease closely resembling JMML, more severe when $Kras^{G12D}$ was induced in HSCs. Upon mutation acquisition, E14.5 fetal liver (FL) cells already showed defining features of JMML such as GM-CSF hypersensitivity. Transcriptomic and epigenetic analyses showed a global transcriptional reprogramming in JMML fetal HSPCs with increased cycling, aberrant metabolic and inflammatory signaling and myeloid bias. To evaluate the leukemogenic potential of fetal hematopoietic cells following oncogene activation, we transplanted FL cells into adult recipients. Notably, only HSC-, but not fetal-restricted HSPCs- targeted cells could induce an aggressive JMML-like disease. In the transgenic bone marrow, we observed HSCs loss, expansion of aberrant progenitors and progressive inflammatory remodeling. Multiple evidence suggest that inflammation could be a targetable disease driver in JMML. We are testing anti-inflammatory treatments in our JMML model and show that they can ameliorate the myeloproliferative phenotype and extend survival.

**Summary/Conclusion**

Our data show that embryonic hematopoietic context dictates the leukemogenic potential of $Kras^{G12D}$ and suggest that clinical heterogeneity in JMML could stem from variability in the cell of origin. We reveal the presence of an early prenatal pre-leukemic stage in the fetal liver. Furthermore, we highlight inflammatory pathways as a potential therapeutic target in $Kras^{G12D}$ driven JMML. Our approach can be extended to other mutations and yields a valuable platform to unveil early pathogenic events in JMML, thus providing an opportunity to uncover other targetable vulnerabilities.

**Copyright Information:** (Online) ISSN: 2572-9241
©2026 The Author(s). HemaSphere is published by John Wiley & Sons Ltd on behalf of European Hematology Association. This is an open access Abstract Book distributed under the Attribution-NonCommercial-NoDerivs (CC BY-NC-ND), which allows third parties to download the articles and share them with others as long as they credit the author and the Abstract Book, but they cannot change the content in any way or use them commercially.
**Abstract Book Citations:** Authors, Title, HemaSphere, 2026;10:(S1):pages. The abstract book DOIs can be found on the “Author Information” page.
**Disclaimer:** Articles published in the journal HemaSphere exclusively reflect the opinions of the authors. The authors are responsible for all content in their abstracts including accuracy of the facts, statements, citing resources, etc.

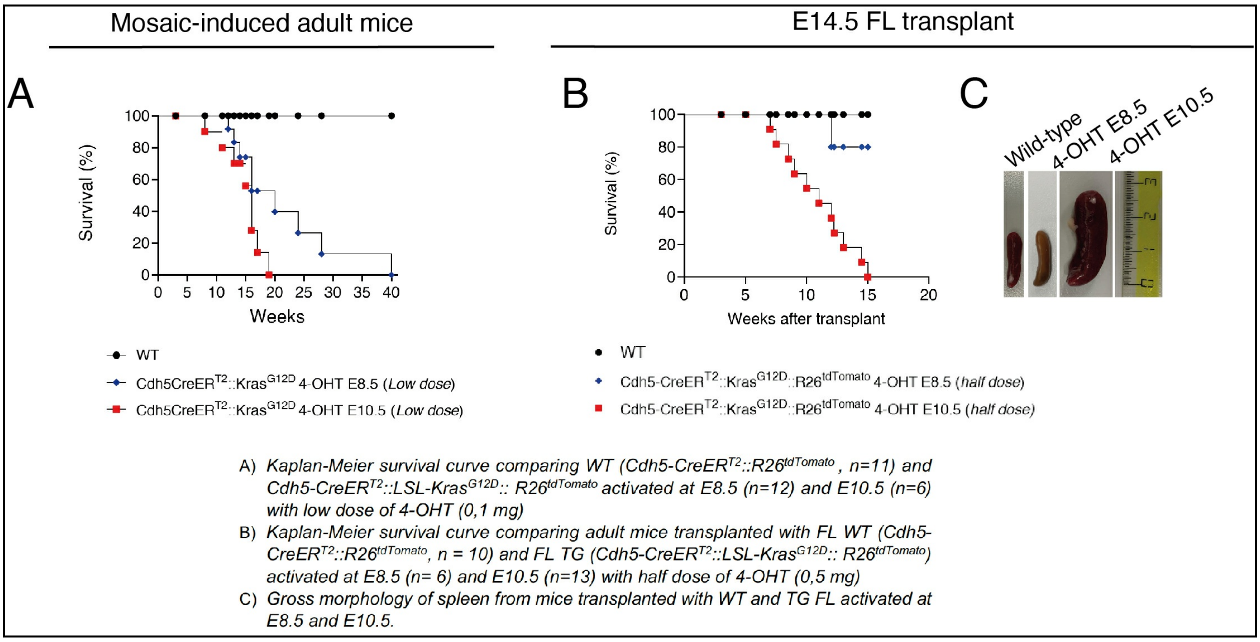

A) Kaplan-Meier survival curve comparing WT (Cdh5-CreERT2::R26tdTomato , n=11) and Cdh5-CreERT2::LSL-KrasG12D:: R26tdTomato activated at E8.5 (n=12) and E10.5 (n=6) with low dose of 4-OHT (0,1 mg) B) Kaplan-Meier survival curve comparing adult mice transplanted with FL WT (Cdh5-CreERT2::R26tdTomato, n = 10) and FL TG (Cdh5-CreERT2::LSL-KrasG12D:: R26tdTomato) activated at E8.5 (n= 6) and E10.5 (n=13) with half dose of 4-OHT (0,5 mg) C) Gross morphology of spleen from mice transplanted with WT and TG FL activated at E8.5 and E10.5.

**Copyright Information:** (Online) ISSN: 2572-9241
©2026 The Author(s). HemaSphere is published by John Wiley & Sons Ltd on behalf of European Hematology Association. This is an open access Abstract Book distributed under the Attribution-NonCommercial-NoDerivs (CC BY-NC-ND), which allows third parties to download the articles and share them with others as long as they credit the author and the Abstract Book, but they cannot change the content in any way or use them commercially.
**Abstract Book Citations:** Authors, Title, HemaSphere, 2026;10:(S1):pages. The abstract book DOIs can be found on the “Author Information” page.
**Disclaimer:** Articles published in the journal HemaSphere exclusively reflect the opinions of the authors. The authors are responsible for all content in their abstracts including accuracy of the facts, statements, citing resources, etc.

### (S166) CSNK1A1 E98V DRIVES REWIRING OF SIGNALLING AND METABOLISM IN DEL(5Q) MDS

**Topic:** 09. Myelodysplastic syndromes - Biology & translational research

Juliette Pearce*[1], Stephani Schmitz[2, 3], Sergio Martinez Høyer[2, 3, 4], Gerjanne Vroeg in de Wei[2, 3], Inge Snoeren[2, 3], Ursula Stalmann[2, 3], Stijn Fuchs[2, 3], Aurelien Dugourd[5], Giulia Franciosa[6], Eric Bindels[2], Wencke Walter[7], Claudia Meyer[8], Torsten Haferlach[7], Emiel van der Vorst[9, 10], Francesc Sole Ristol[11], Julio Saez-Rodriguez[5, 12], Andreas Linkermann[8, 13, 14], Jesper Olsen[6], Hélène Gleitz[2, 3], Rebekka Schneider-Kramann[1, 2, 3]

*[1]RWTH Uniklinik Aachen, Cell and Tumor Biology, Aachen, Germany;[2]Erasmus Medical Center, Developmental Biology, Rotterdam, The Netherlands; [3]Oncode Institute, Rotterdam, The Netherlands; [4]Karolinska Institutet, Microbiology, tumor and cell biology, Stockholm, Sweden; [5]Heidelberg University, Institute for Computational Biomedicine, Heidelberg, Germany;[6]Novo Nordisk Foundation Center for Protein Research, Copenhagen, Denmark; [7]MLL Munich Leukemia Laboratory, Munich, Germany; [8]University Medical Centre Mannheim, Department of Medicine V, Mannheim, Germany;[9]RWTH Uniklinik Aachen, Department of Internal Medicine I – Cardiology, Aachen, Germany; [10]Ludwig-Maximilians-Universität, Institute for Cardiovascular Prevention, Munich, Germany; [11]Universitat Autònoma de Barcelona, Josep Carreras Leukaemia Research Institute, Badalona, Spain; [12]European Bioinformatics Institute, Hinxton, United Kingdom; [13]Technische Universität Dresden, Dresden, Germany; [14]Albert Einstein College of Medicine, Division of Nephrology, New York, United States of America;*

**Background**

Deletion of chromosome 5q [del(5q)] is the most common cytogenetic abnormality in myelodysplastic neoplasms (MDS) and causes haploinsufficiency of several genes, including the tumor suppressor CSNK1A1. Secondary CSNK1A1 mutations, predominantly affecting the E98 hotspot, arise almost exclusively in del(5q) MDS and are associated with adverse clinical outcomes, yet their biological function remains poorly defined.

**Aims**

In our study, we aim to determine the functional consequences of recurrent CSNK1A1 E98 mutations in del(5q) MDS on kinase function, cellular signalling and differentiation, and to assess whether these changes reveal novel, clinically significant therapeutic vulnerabilities.

**Methods**

We combined conditional mouse models (Mx1-Cre;Csnk1a1fl/fl) in serial transplantation assays expressing WT or E98V Csnk1a1, alongside integrated transcriptomic, proteomic, phosphoproteomic, and kinome activity profiling of cell lines. Functional *in vitro* analyses were used to assess mitochondrial and glycolytic metabolic function, as well as iron handling and ferroptosis sensitivity. Findings were correlated with clinical and laboratory parameters from a cohort of 195 del(5q) MDS patients with or without CSNK1A1 mutations.

**Copyright Information:** (Online) ISSN: 2572-9241
©2026 The Author(s). HemaSphere is published by John Wiley & Sons Ltd on behalf of European Hematology Association. This is an open access Abstract Book distributed under the Attribution-NonCommercial-NoDerivs (CC BY-NC-ND), which allows third parties to download the articles and share them with others as long as they credit the author and the Abstract Book, but they cannot change the content in any way or use them commercially.
**Abstract Book Citations:** Authors, Title, HemaSphere, 2026;10:(S1):pages. The abstract book DOIs can be found on the "Author Information" page.
**Disclaimer:** Articles published in the journal HemaSphere exclusively reflect the opinions of the authors. The authors are responsible for all content in their abstracts including accuracy of the facts, statements, citing resources, etc.

**Results**

We observed that Csnk1a1 E98V retains essential kinase function and supports long-term hematopoiesis *in vivo*, but does not enhance clonal expansion, distinguishing it from Csnk1a1 haploinsufficiency. Instead, Csnk1a1 E98V induced broad suppression of kinase signaling networks, accompanied by coordinated repression of ribosomal gene expression, global protein translation, and cell-cycle programs. Mutant cells exhibited metabolic reprogramming characterized by reduced mitochondrial respiration, increased glycolytic flux, and metabolic inflexibility under stress. This adaptive state was accompanied by impaired megakaryopoiesis and heightened sensitivity to iron-induced toxicity and ferroptosis due to dysregulated iron handling and mitochondrial dysfunction. Clinically, CSNK1A1-mutant del(5q) MDS patients displayed thrombocytopenia, increased myeloblast burden, and lower prevalence of elevated serum iron levels, consistent with these cellular phenotypes.

**Summary/Conclusion**

Our data support a two-step model in which del(5q) Csnk1a1 haploinsufficiency drives initial clonal expansion, followed by acquisition of Csnk1a1 E98 mutations that promote stress adaptation rather than proliferation. This change-of-function rewiring establishes a more quiescent, stress tolerant state, but exposes metabolic and iron-dependent vulnerabilities, identifying potential novel therapeutic targets in del(5q) MDS with CSNK1A1 mutations.

**Copyright Information:** (Online) ISSN: 2572-9241
©2026 The Author(s). HemaSphere is published by John Wiley & Sons Ltd on behalf of European Hematology Association. This is an open access Abstract Book distributed under the Attribution-NonCommercial-NoDerivs (CC BY-NC-ND), which allows third parties to download the articles and share them with others as long as they credit the author and the Abstract Book, but they cannot change the content in any way or use them commercially.
**Abstract Book Citations:** Authors, Title, HemaSphere, 2026;10:(S1):pages. The abstract book DOIs can be found on the “Author Information” page.
**Disclaimer:** Articles published in the journal HemaSphere exclusively reflect the opinions of the authors. The authors are responsible for all content in their abstracts including accuracy of the facts, statements, citing resources, etc.

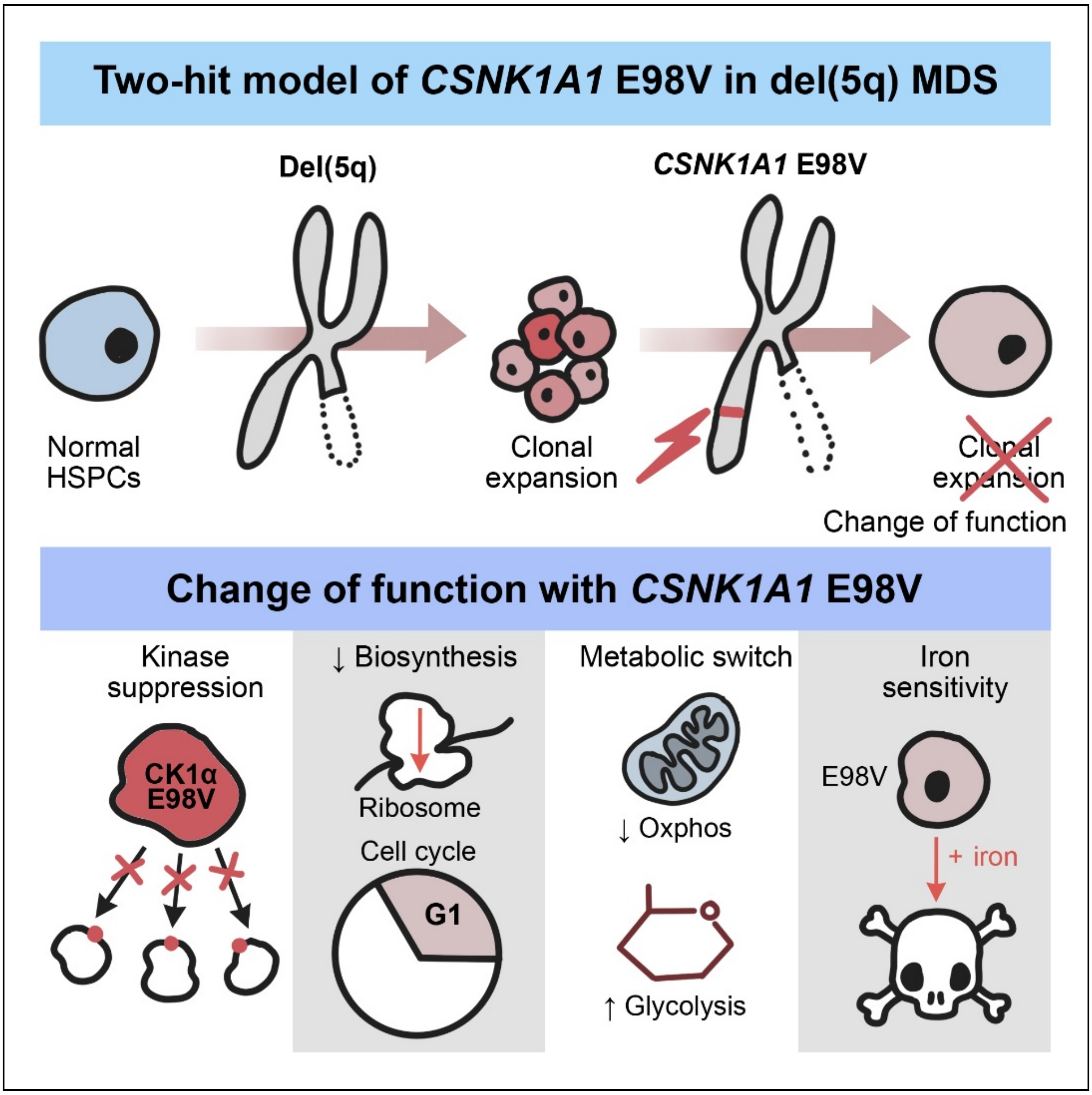

**Copyright Information:** (Online) ISSN: 2572-9241
©2026 The Author(s). HemaSphere is published by John Wiley & Sons Ltd on behalf of European Hematology Association. This is an open access Abstract Book distributed under the Attribution-NonCommercial-NoDerivs (CC BY-NC-ND), which allows third parties to download the articles and share them with others as long as they credit the author and the Abstract Book, but they cannot change the content in any way or use them commercially.
**Abstract Book Citations:** Authors, Title, HemaSphere, 2026;10:(S1):pages. The abstract book DOIs can be found on the "Author Information" page.
**Disclaimer:** Articles published in the journal HemaSphere exclusively reflect the opinions of the authors. The authors are responsible for all content in their abstracts including accuracy of the facts, statements, citing resources, etc.

### (S167) LARGE-SCALE SCREENING OF MYELOID NEOPLASTIC PRECURSOR STATES USING BLOOD CELL MORPHOLOGY

**Topic:** 09. Myelodysplastic syndromes - Biology & translational research

Oona Rinkinen*[1, 2, 3], Theerin Lanamtieng[1, 2, 3], Brankica Šimac[4, 5], Stas Tatun[1, 2, 3], Mikko Purhonen[1, 2, 3], Henri Sundquist[1, 2, 3], Nattiya Teawtrakul[6], Pisa Phiphitaporn[6], Lotta Joutsi-Korhonen[2], Sanna Siitonen[2], Anna Lempiäinen[2], Torsten Haferlach[7], Oscar Brück[1]

*[1]Hematoscope Lab Comprehensive Cancer Center, Helsinki, Finland; [2]Department of Clinical Chemistry HUS Diagnostic Center, Helsinki, Finland; [3]Department of Oncology, University of Helsinki, Helsinki, Finland; [4]Working Group for Laboratory Hematology of the Croatian Society of Medical Biochemistry and Laboratory Medicine, Zagreb, Croatia; [5]Clinical Department of Laboratory Diagnostics, Dubrava University Hospital, Zagreb, Croatia; [6]Division of Hematology, Department of Medicine, Faculty of Medicine, Khon Kaen University, Khon Kaen, Thailand; [7]MLL Munich Leukemia Laboratory, Munich, Germany;*

### Background

Acute myeloid leukemia (AML) and myelodysplastic syndromes (MDS) are preceded by pre-leukemic states characterized by mutations in myeloid driver genes and changes in complete blood counts (CBCs). Despite the routine nature of CBCs and peripheral blood smears, their potential for early screening of myeloid neoplasms has not been investigated.

### Methods

In this multi-center study funded by EHA Innovation Grant (MorphoScreen), we analyzed CBCs and corresponding peripheral blood smear images from 4,249 patients with AML (median age 64 years, 46% female), 5,818 patients with MDS (median age 71 years; 41% female), 323 patients with pre-leukemic conditions (median age 71; 46% female), and 18,625 control individuals (median age 54 years; 52% female). Controls represented patients subjected to CBC evaluation to monitor chronic diseases or diagnose acute conditions such as infections. Samples were collected from clinical centers in Finland, Germany, Thailand, and Croatia and digitized with 4 digital scanners to maximize model generalizability.
The dataset spanned over 14 million cell images, with each patient contributing hundreds to thousands of single-cell images. Each cell was represented by 384 morphological embeddings extracted using a self-supervised learning model trained on a multi-scanner and multi-center dataset.
With almost 600 million data points, we trained an Extreme Gradient Boosting model using a Cox proportional hazards objective.To support model interpretability, we used human-interpretable cell-level morphological features and sample level features to explain morphological features associated with high disease risk.

**Copyright Information:** (Online) ISSN: 2572-9241
©2026 The Author(s). HemaSphere is published by John Wiley & Sons Ltd on behalf of European Hematology Association. This is an open access Abstract Book distributed under the Attribution-NonCommercial-NoDerivs (CC BY-NC-ND), which allows third parties to download the articles and share them with others as long as they credit the author and the Abstract Book, but they cannot change the content in any way or use them commercially.
**Abstract Book Citations:** Authors, Title, HemaSphere, 2026;10:(S1):pages. The abstract book DOIs can be found on the “Author Information” page.
**Disclaimer:** Articles published in the journal HemaSphere exclusively reflect the opinions of the authors. The authors are responsible for all content in their abstracts including accuracy of the facts, statements, citing resources, etc.

### Results

We compared leukocyte subtype proportions obtained from CBC with proportions derived from image analysis for common blood cell types. Correlation coefficients ranged from $R = 0.66$ for basophils to $R = 0.85$ for eosinophils indicating reliable detection of leukocyte subpopulations from blood cell images.
The combined morphology–clinical model demonstrated robust performance in identifying preleukemic patients in the test set (AUC 0.87). Importantly, the performance was superior to models trained with only clinical (AUC 0.83) or morphology data (AUC 0.67). When decoding the morphology-clinical model, we observed a significant decrease in the median neutrophil proportion among preleukemic patients compared to controls (56.1% vs. 51.3%, $p < 0.001$). In contrast, the median monocyte proportion was significantly increased in preleukemic patients (9.6% vs. 6.4%, $p < 0.001$).
Finally, specific quantitative morphological features were associated with increased preleukemia risk. In neutrophils, nuclear hyposegmentation and increased nuclear-to-cytoplasmic (N:C) ratio were associated with preleukemia. Moreover, elevated N:C ratio, and increased nuclear roundness and cytoplasmic vacuolization in monocytes were enriched in preleukemia samples.

### Summary/Conclusion

Collectively, we show that quantitative morphological features extracted from routine peripheral blood smears improve prediction of pre-leukemic states beyond clinical features. By modeling risk at the single-cell level, our approach not only enhances predictive accuracy but also enables identification of morphological features driving progression risk. These findings highlight the potential of scalable and interpretable early detection of individuals at risk for AML and MDS.

**Copyright Information:** (Online) ISSN: 2572-9241
©2026 The Author(s). HemaSphere is published by John Wiley & Sons Ltd on behalf of European Hematology Association. This is an open access Abstract Book distributed under the Attribution-NonCommercial-NoDerivs (CC BY-NC-ND), which allows third parties to download the articles and share them with others as long as they credit the author and the Abstract Book, but they cannot change the content in any way or use them commercially.
**Abstract Book Citations:** Authors, Title, HemaSphere, 2026;10:(S1):pages. The abstract book DOIs can be found on the “Author Information” page.
**Disclaimer:** Articles published in the journal HemaSphere exclusively reflect the opinions of the authors. The authors are responsible for all content in their abstracts including accuracy of the facts, statements, citing resources, etc.

### (S168) SELECTIVE EXPANSION OF TET2-MUTANT CLONAL HEMATOPOIESIS UNDER METFORMIN IS DRIVEN BY ENHANCED MITOCHONDRIAL AND LIPID STRESS TOLERANCE

**Topic:** 09. Myelodysplastic syndromes - Biology & translational research

Silke E. Miedema*[1], Bauke de Boer[1, 2, 3], Jonas B. Salzbrunn[1], Aniek de Graaf[4], Joop Jansen[4], Maaike van Bergen[4], Pedro Aragon-Fernandez[5], Nanna Mikkelsen[6], Erwin Schoof[5], Rasmus Bak[6], Bo Porse[2, 3, 7], Dominique Sternadt[1], Avinash Dinmohamed[8, 9], Jan Jacob Schuringa[1], Gerwin Huls[1], Isabelle van Zeventer[1], Diego A Pereira-Martins[1]

*[1]Department of Hematology, University of Groningen, University Medical Center Groningen, Groningen, The Netherlands;[2]The Finsen Laboratory, Rigshospitalet, University of Copenhagen, Copenhagen, Denmark; [3]Biotech Research and Innovation Center (BRIC), University of Copenhagen, Copenhagen, Denmark; [4]Department of Laboratory Medicine, Laboratory of Hematology, Radboud University Medical Center, Nijmegen, The Netherlands; [5]Technical University of Denmark, Department of Biotechnology and Biomedicine, Lyngby, Denmark; [6]Aarhus University, Department of Biomedicine, Aarhus, Denmark;[7]University of Copenhagen, Department of Clinical Medicine, Copenhagen, Denmark; [8]Department of Research and Development, Netherlands Comprehensive Cancer Organization (IKNL), Utrecht, The Netherlands; [9]Erasmus MC, Department of Public Health, University Medical Center Rotterdam, Rotterdam, The Netherlands;*

### Background

Clonal hematopoiesis (CH) is a common, age-related premalignant condition caused by somatic mutations, most often in epigenetic regulators such as DNMT3A and TET2. It is associated with an increased risk of hematologic malignancies and cardiovascular disease. Metformin has been reported to suppress *DNMT3A* R882 mutant CH, but its impact across other CH genotypes is unclear.

### Aims

Using the population-based Lifelines cohort and functional studies, we assessed the impact of metformin on CH prevalence and longitudinal clonal dynamics.

### Methods

Error-corrected targeted NGS of 4715 individuals (347 metformin users, 4368 non-users) was performed at baseline, with 1177 individuals having serial sampling (median interval 3.58 years). *TET2* KO hematopoietic stem/progenitor cells (HSPCs) were generated using CRISPR/Cas9 for functional studies.

**Copyright Information:** (Online) ISSN: 2572-9241
©2026 The Author(s). HemaSphere is published by John Wiley & Sons Ltd on behalf of European Hematology Association. This is an open access Abstract Book distributed under the Attribution-NonCommercial-NoDerivs (CC BY-NC-ND), which allows third parties to download the articles and share them with others as long as they credit the author and the Abstract Book, but they cannot change the content in any way or use them commercially.
**Abstract Book Citations:** Authors, Title, HemaSphere, 2026;10:(S1):pages. The abstract book DOIs can be found on the “Author Information” page.
**Disclaimer:** Articles published in the journal HemaSphere exclusively reflect the opinions of the authors. The authors are responsible for all content in their abstracts including accuracy of the facts, statements, citing resources, etc.

**Results**

After adjusting for age, sex, HbA1c and BMI, we found no differences in CH prevalence, mutational spectrum or clone size between metformin users and non-users. However, growth rates for *TET2*-mutant CH clones were higher in metformin users. Increased growth for TET2-mutant CH was found only in diabetic individuals using metformin, whereas diabetes alone had no effect. To model *TET2*-CH under metformin, we used CRISPR/Cas9 to disrupt *TET2* in primary human cord blood-derived HSPCs (AAVS1-edited cells as controls). TET2-knockout (KO) HSPCs showed increased proliferation, sustained CD34 expression and less cell death across metformin doses (5–100 µM), with reduced cytoplasmic ROS and preserved mitochondrial membrane potential, indicating better tolerance to metformin-induced metabolic stress. LC-MS/MS-based lipidomic profiling revealed that *TET2* loss reprograms lipid metabolism, with enrichment of phosphatidylethanolamine (PE) and triglycerides (TG) linked to mitochondrial membranes and lipid droplets. Upon metformin, AAVS1 cells displayed marked depletion of cardiolipin (CL), which resides in the inner mitochondrial membranes. This was associated with increased TG species enriched in highly unsaturated fatty acids, and a dose-dependent rise in neutral lipid (BODIPY C16) staining. In contrast, metformin-treated *TET2* KO cells showed only mild CL depletion but robust accumulation of sphingomyelins (SM) and TG, without further increase in neutral lipid staining, consistent with a buffered, SM/TG-rich lipid state that alleviate polyunsaturated fatty acids-driven lipotoxic stress. In 1:1 co-culture competition assays (*TET2* KO $GFP^+$ vs AAVS1 $BFP^+$), metformin enhanced the clonal advantage of *TET2* KO cells. Label-free proteomics on 500 sorted $CD34^+$ TET2-KO and AAVS1 HSPCs (single and co-culture metformin-treated, 7778 proteins) showed that, relative to AAVS1, *TET2*-KO cells share a core set of upregulated proteins enriched in mitochondrial organization and lipid/amino acid metabolism. Extracellular flux analysis confirmed that *TET2*-KO HSPCs maintain high mitochondrial respiration under metformin at levels comparable to untreated AAVS1 controls.

**Summary/Conclusion**

Metformin does not uniformly suppress CH, but is associated with accelerated clonal expansion of *TET2*-mutant clones. TET2 loss establishes a mitochondrial–lipid program that confers resistance to metformin-induced metabolic stress and promotes clonal dominance. This uncovers a previously unrecognized change in lipid metabolism for TET2-mutant CH and suggests that metformin, and potentially other lipid-modulating drugs, differentially affect CH clones depending on their mutational background.

**Copyright Information:** (Online) ISSN: 2572-9241
©2026 The Author(s). HemaSphere is published by John Wiley & Sons Ltd on behalf of European Hematology Association. This is an open access Abstract Book distributed under the Attribution-NonCommercial-NoDerivs (CC BY-NC-ND), which allows third parties to download the articles and share them with others as long as they credit the author and the Abstract Book, but they cannot change the content in any way or use them commercially.
**Abstract Book Citations:** Authors, Title, HemaSphere, 2026;10:(S1):pages. The abstract book DOIs can be found on the “Author Information” page.
**Disclaimer:** Articles published in the journal HemaSphere exclusively reflect the opinions of the authors. The authors are responsible for all content in their abstracts including accuracy of the facts, statements, citing resources, etc.

### (S169) GENOMIC ANALYSIS OF PRELEUKEMIC CLONES IDENTIFIES NEW DRIVERS OF MYELOID NEOPLASMS

**Topic:** 09. Myelodysplastic syndromes - Biology & translational research

Martina Sarchi*[1, 2, 3], Xiaoyi Cheng[3, 4], Carmen Iannini[1], Sintra Stewart[3], Jason Kim[3], Claudia Fiducioso[1], Stephanie Busch[3], Nelli Aydinyan[3], Andreea Reilly[3], J Philip Creamer[3, 4], Rochelle Bergantinos[3], Jasper Hsu[3], Malgorzata Olszewska[5, 6], Anna Galli[2], Luca Malcovati[1, 2], Sioban Keel[3], David Wu[7], Derek Stirewalt[8], Janis Abkowitz[3, 9], Pamela S. Becker[10, 11], Peter van Galen[12, 13, 14], Kelley Harris[9, 15], Eirini Papapetrou[5, 6], Suleyman Gulsuner[16], Sergei Doulatov[3, 4, 9, 17]

*[1]University of Pavia, Department of Molecular Medicine, Pavia, Italy;[2]IRCCS San Matteo Hospital Foundation, Unit of Molecular Hematology & Precisione Medicine, Pavia, Italy; [3]University of Washington, Division of Hematology and Oncology, Department of Medicine, Seattle, United States of America; [4]Columbia University Medical Center, Department of Physiology and Cellular Biophysics, New York, United States of America; [5]Icahn School of Medicine at Mount Sinai, Department of Oncological Sciences and Department of Medicine, Tisch Cancer Center, New York, United States of America; [6]Icahn School of Medicine at Mount Sinai, Center for Advancement of Blood Cancer Therapies, New York, United States of America; [7]University of Washington, Department of Laboratory Medicine and Pathology, Seattle, United States of America; [8]Fred Hutchinson Cancer Center, Translational Science and Therapeutics, Seattle, United States of America; [9]University of Washington, Department of Genome Sciences, Seattle, United States of America; [10]City of Hope National Medical Center, Department of Hematology and Hematopoietic Cell Transplantation, Duarte, United States of America; [11]City of Hope Beckman Research Institute, Department of Hematologic Malignancies Translational Science, Duarte, United States of America; [12]Brigham and Women's Hospital, Division of Hematology, Boston, United States of America; [13]Broad Institute of MIT and Harvard, Boston, Boston, United States of America; [14]Harvard Medical School, Ludwig Center at Harvard, Boston, United States of America;[15]Fred Hutchinson Cancer Center, Herbold Computational Biology Program, Boston, United States of America; [16]University of Washington, Division of Medical Genetics, Department of Medicine, Seattle, United States of America; [17]University of Washington, Institute for Stem Cell and Regenerative Medicine, Institute for Stem Cell and Regenerative Medicine, United States of America;*

### Background

Myeloid neoplasms (MNs) are clonal disorders that arise from hematopoietic stem cells (HSCs) following extended preleukemic evolution. Much of our genetic understanding of preleukemic evolution comes from population studies of clonal hematopoiesis (CH). However, lifetime risk of developing MNs for individuals with CH is low. Alternatively, retrospective studies have tracked somatic mutations in single preleukemic cells in patients who developed myelodysplastic neoplasms (MDS) and acute myeloid leukemia (AML). Such retrospective studies are challenging since preleukemic cells in patients are rare. Here, we use induced pluripotent stem cell (iPSC) reprogramming to sequence genomes of preleukemic clones providing insights into drivers of preleukemic clonal evolution.

### Aims

Sequence the genomes of single clones spanning preleukemic clonal evolution and identify the mechanisms of progression from preleukemia to MDS or AML.

### Methods

We isolated single cell clones using iPSC reprogramming of $CD34^+$ cells from patients with MDS (n=8) and AML (n=10). Individual clones were staged by their driver mutation count. Using whole exome sequencing of 42 iPSC clones and 18 matched patient samples, we tracked somatic mutations during preleukemic evolution. To identify genes under selection, we applied dN/dS analysis, comparing the ratio of nonsynonymous to silent mutations. New candidate drivers were functionally tested by CRISPR/Cas9 screening in primary $CD34^+$ HSCs and progenitors (HSPCs).

**Copyright Information:** (Online) ISSN: 2572-9241
©2026 The Author(s). HemaSphere is published by John Wiley & Sons Ltd on behalf of European Hematology Association. This is an open access Abstract Book distributed under the Attribution-NonCommercial-NoDerivs (CC BY-NC-ND), which allows third parties to download the articles and share them with others as long as they credit the author and the Abstract Book, but they cannot change the content in any way or use them commercially.
**Abstract Book Citations:** Authors, Title, HemaSphere, 2026;10:(S1):pages. The abstract book DOIs can be found on the "Author Information" page.
**Disclaimer:** Articles published in the journal HemaSphere exclusively reflect the opinions of the authors. The authors are responsible for all content in their abstracts including accuracy of the facts, statements, citing resources, etc.

### Results

Genome sequencing of patient-derived clones revealed patterns of mutation acquisition during preleukemic evolution. Initiating clones with a single driver mutation harbored only 1.8 coding mutations per genome compared to HSCs without driver mutations from the same patient. Somatic mutations were acquired at a linear rate. Notably, the ratio of non-synonymous to synonymous mutations was significantly higher in more advanced clones. These data are incompatible with a model in which all somatic mutations in genes currently unrecognized as drivers are neutral, and suggest that many somatic mutations can confer fitness advantage. To identify novel drivers, we selected 19 genes with likely pathogenic variants in our dataset and performed functional screening by CRISPR/Cas9 editing of human HSPCs. Disruption of *CPSF1*, an mRNA cleavage factor, led to progressive depletion of edited cells, consistent with an essential role in human HSPCs. In contrast, knockouts of *NFE2*, *NSD1*, *RSK1*, *TTYH2*, and *CBARP* expanded HSPCs over time, nominating these as candidate driver genes. Analysis of sorted HSPCs showed enrichment of *NSD1* knockout in the immature $CD34^{+}CD133^{+}$ compartment. Consistently, *NSD1* knockdown significantly expanded $CD34^{+}CD133^{+}$ phenotypic HSCs. NSD1 histone methyltransferase activity is required for DNMT3A binding to nucleosomes and *NSD1* and *DNMT3A* mutations are mutually exclusive in MNs, providing a putative explanation for why *NSD1* loss-of-function mutations are relatively rare in MNs.

### Summary/Conclusion

Genome sequencing of preleukemic clones provides direct evidence that somatic variants in multiple genes not recognized as cancer drivers confer fitness advantage. We validated candidate drivers using functional CRISPR screening in normal human HSPCs identifying regulators of stem cell fitness. Among these, we nominate *NSD1* loss-of-function as a driver of clonal expansion, and propose that *NSD1* mutations may be relatively infrequent in MNs compared to other cancers due to epistasis with common *DNMT3A* mutations.

**Copyright Information:** (Online) ISSN: 2572-9241
©2026 The Author(s). HemaSphere is published by John Wiley & Sons Ltd on behalf of European Hematology Association. This is an open access Abstract Book distributed under the Attribution-NonCommercial-NoDerivs (CC BY-NC-ND), which allows third parties to download the articles and share them with others as long as they credit the author and the Abstract Book, but they cannot change the content in any way or use them commercially.
**Abstract Book Citations:** Authors, Title, HemaSphere, 2026;10:(S1):pages. The abstract book DOIs can be found on the “Author Information” page.
**Disclaimer:** Articles published in the journal HemaSphere exclusively reflect the opinions of the authors. The authors are responsible for all content in their abstracts including accuracy of the facts, statements, citing resources, etc.

### (S170) CPX-351 VERSUS STANDARD OF CARE AS BRIDGING TO ALLOHCT IN PATIENTS WITH HIGHER-RISK MYELODYSPLASTIC NEOPLASMS OR OLIGOBLASTIC ACUTE MYELOID LEUKEMIA: RESULTS OF THE RANDOMIZED PHASE 2 PALOMA STUDY

**Topic:** 10. Myelodysplastic syndromes - Clinical

Uwe Platzbecker*[1], Martina Crysandt[2], Martin Kaufmann[3], Andreas Rank[4], Christoph Röllig[1], Hartmut Döhner[5], Gesine Bug[6], Katharina Götze[7], Felicitas Thol[8], Tobias A.W. Holderried[9, 10], Judith Niederland[11], Mathias Hänel[12], Stefan A. Klein[13], Christian Junghanss[14], Wolfgang Bethge[15], Knut Wendelin[16], Thomas Schröder[17], Sigrid MacHherndl-Spandl[18], Ulrich Germing[19], Tim Sauer[20], Inken Hilgendorf[21], Dirk Niemann[22], Anne Sophie Platzbecker[23, 24, 25], Georg-Nikolaus Franke[26], Katja Sockel[27], Susann Leupold[28], Martin Puttrich[28], Silke Gloaguen[1], Sandra S.L. Roldan P.[29], Hubert Serve[30], Carsten Müller-Tidow[31], Klaus Metzeler[32, 33], Martin Bornhäuser[34], Wolf-Karsten Hofmann[35], Richard Schlenk[20, 29], Madlen Jentzsch[36]

*[1]University Hospital Dresden, Dresden, Germany; [2]RWTH Aachen University & Center for Integrated Oncology Aachen Bonn Cologne Düsseldorf (CIO ABCD), Department of Hematology, Oncology, Hemostaseology and Stem Cell Transplantation, Medical Faculty, Aachen, Germany; [3]Robert Bosch Krankenhaus, Stuttgart, Germany; [4]University Hospital of Ausburg, Ausburg, Germany; [5]Ulm University Hospital, Department of Internal Medicine III, Ulm, Germany; [6]Goethe University Frankfurt, University Hospital, Department of Medicine II, Frankfurt am Main, Germany; [7]TUM University Hospital, Technical University of Munich School of Medicine and Health, Munich, Germany; [8]Medizinische Hochschule Hannover, Klinik für Hämatologie, Hämostaseologie, Onkologie und Stammzelltransplantation, Hannover, Germany; [9]University of Bonn and University Hospital Bonn, Medical Clinic and Polyclinic III Internal Medicine with a focus on Oncology, Hematology, and Rheumatology, Bonn, Germany; [10]Medical University Graz, Division of Hematology, Graz, Austria; [11]Helios Hospital Berlin-Buch, Berlin, Germany; [12]Klinikum Chemnitz gGmbH, Department of Internal Medicine III, Chemnitz, Germany; [13]Universitätsmedizin Mannheim, Mannheim, Germany; [14]Universitätsmedizin Rostock, Rostock, Germany; [15]Universitätsklinikum Tübingen, Hämatologie, Onkologie, Klinische Immunologie und Rheumatologie, Tübingen, Germany; [16]Paracelsus Medizinische Privatuniversität, Klinikum Nürnberg, Klinik für Innere Medizin V, Nürnberg, Germany; [17]Universitätsklinikum Essen, Klinik für Hämatologie und Stammzelltransplantation, Essen, Germany; [18]Ordensklinikum Linz - Elisabethinen, Department of Internal Medicine I: Hematology with Stem-Cell Transplantation, Hemostaseology and Medical Oncology, Linz, Austria; [19]Heinrich Heine Universität Düsseldorf, Universitätsklinikum Düsseldorf, Klinik für Hämatologie, Onkologie und klinische Immunilogie, Düsseldorf, Germany; [20]Universitätsklinikum Heidelberg, Medizinische Klinik V, Heidelberg, Germany; [21]Universitätsklinikum Jena, Klinik für Innere Medizin II, Abteilung für Hämatologie und medizinische Onkologie, Jena, Germany; [22]Gemeinschaftsklinikum Mittelrhein, Koblenz, Germany; [23]Else Kröner Fresenius Center for Digital Health, Faculty of Medicine and University Hospital Carl Gustav Carus, Dresden University of Technology, Dresden, Germany; [24]Hasso-Plattner Institute, University of Postdam, Digital Health Cluster, Department for Digital Health, Economics & Policy, Postdam, Germany; [25]Medizinische Universität Lausitz - Carl Thiem, Cottbus, Germany; [26]Universitätsklinikum Leipzig, Leipzig, Germany; [27]University Hospital Dresden, TU Dresden, Medical Clinic I, Dresden, Germany; [28]GWT-TUD GmbH, Dresden, Germany; [29]NCT Heidelberg, Heidelberg, Germany; [30]Goethe University Hospital, Department of Medicine, Hematology/ Oncology, Frankfurt am Main, Germany; [31]Universitätsklinikum Heidelberg, Heidelberg, Germany; [32]Universitätsklinikum Leipzig, Department of Hematology, Cell Therapy, Hemostaseology, Infectious Diseases and Hemostaseology, Leipzig, Germany; [33]Comprehensive Cancer Center Central Germany, Leipzig, Germany; [34]University Hospital Dresden, Med. Klinik I, Dresden, Germany; [35]Heidelberg University, University Hospital Mannheim, Mannheim, Germany; [36]Universitätsklinikum Leipzig, Medizinische Klinik I - Hämatologie und Zelltherapie, Leipzig, Germany;*

### Background

Allogeneic hematopoietic stem cell transplantation (alloHCT) remains the only potentially curative treatment option for patients (pts) with higher-risk myelodysplastic neoplasms (HR-MDS) or oligoblastic acute myeloid leukemia (AML). Pts are commonly bridged from diagnosis to alloHCT via hypomethylating agents such as azacitidine (AZA) or with conventional induction chemotherapy (IC). CPX-351, a liposomal formulation of daunorubicin and cytarabine at a fixed ratio, has not been studied in this setting.

**Copyright Information:** (Online) ISSN: 2572-9241
©2026 The Author(s). HemaSphere is published by John Wiley & Sons Ltd on behalf of European Hematology Association. This is an open access Abstract Book distributed under the Attribution-NonCommercial-NoDerivs (CC BY-NC-ND), which allows third parties to download the articles and share them with others as long as they credit the author and the Abstract Book, but they cannot change the content in any way or use them commercially.
**Abstract Book Citations:** Authors, Title, HemaSphere, 2026;10:(S1):pages. The abstract book DOIs can be found on the "Author Information" page.
**Disclaimer:** Articles published in the journal HemaSphere exclusively reflect the opinions of the authors. The authors are responsible for all content in their abstracts including accuracy of the facts, statements, citing resources, etc.

### Aims

The PALOMA trial compares the efficacy and safety of CPX-351 and conventional care regimens (CCR) before alloHCT as first-line treatment in pts with HR-MDS or oligoblastic AML.

### Methods

In this multicenter, randomized, open-label, phase 2 study, untreated pts with HR-MDS or oligoblastic AML (≤29% blasts per central morphology) for whom alloHCT was intended in the next 6 months were randomized 1:1 to receive CPX-351 (up to 2 cycles of induction therapy with daunorubicin 44 mg/m$^2$ and cytarabine 100 mg/m$^2$ in liposomes plus optional consolidation therapy with daunorubicin 29 mg/m$^2$ and cytarabine 65 mg/m$^2$ in liposomes) or CCR (AZA or IC). The primary endpoint was event-free survival (EFS), defined as time from randomization to primary refractory disease, relapse, death from any cause, or treatment change. Secondary endpoints included overall survival (OS, time from randomization to death from any cause), the proportion of pts proceeding to alloHCT and safety. Efficacy endpoints were evaluated in the full analysis set (FAS; all randomized pts under the intention-to-treat principle and excluding pts without informative data for EFS assessment). Safety was evaluated in the safety analysis set (all enrolled pts who received any amount of study drug). EFS and OS were evaluated by two-sided stratified log-rank test stratified by age (18–60 and >60 years) and bone marrow blast count (<20% and 20–29%). EFS and OS analyses were planned hierarchically for the Sep 30, 2025, data cutoff, as well as for OS per the Lan-DeMets alpha spending method for the interim (Sep 30, 2025) and final OS analyses (Sep 30, 2026). Here we present final analysis of EFS and pre-planned interim analyses of the secondary endpoints.

### Results

At data cutoff (Sep 30, 2025), 150 pts had received CPX-351 (n=75) or CCR (IC n=52, AZA n=23). The FAS included 141 pts (CPX-351 n=67, CCR n=74). After median follow-up of 38 months, median EFS was significantly improved in the CPX-351 arm (median: not reached [NR]; 95% confidence interval [CI]: 15.3 months, NR) versus the CCR arm (median: 11.1 months; 95% CI: 4.1, 23.8, *P*=0.0263; Figure 1). Median OS was NR (95% CI: 24.6 months, NR) and 35.4 months (95% CI: 23.3, NR) among pts in the CPX-351 and CCR arms, respectively (interim analysis; not significant). Twenty-five pts had disease that was refractory to study therapy (CPX-351 n=8, CCR n=17). A total of 57 (85.1%) and 64 (86.4%) pts in the CPX-351 and CCR arms proceeded to alloHCT. The most common grade ≥3 adverse events were thrombocytopenia (65 and 55 pts in the CPX-351 and CCR arms, respectively), leukopenia (55 and 46 pts), and febrile neutropenia (44 and 35 pts). This study is registered at ClinicalTrials.gov (NCT04061239), EudraCT (2018-002430-21), and EU CT (2024-515375-35-00).

### Summary/Conclusion

PALOMA met its primary endpoint in interim analysis showing that CPX-351 resulted in improved EFS when compared to CCR among pts with HR-MDS or oligoblastic AML scheduled for alloHCT.

**Copyright Information:** (Online) ISSN: 2572-9241
©2026 The Author(s). HemaSphere is published by John Wiley & Sons Ltd on behalf of European Hematology Association. This is an open access Abstract Book distributed under the Attribution-NonCommercial-NoDerivs (CC BY-NC-ND), which allows third parties to download the articles and share them with others as long as they credit the author and the Abstract Book, but they cannot change the content in any way or use them commercially.
**Abstract Book Citations:** Authors, Title, HemaSphere, 2026;10:(S1):pages. The abstract book DOIs can be found on the “Author Information” page.
**Disclaimer:** Articles published in the journal HemaSphere exclusively reflect the opinions of the authors. The authors are responsible for all content in their abstracts including accuracy of the facts, statements, citing resources, etc.

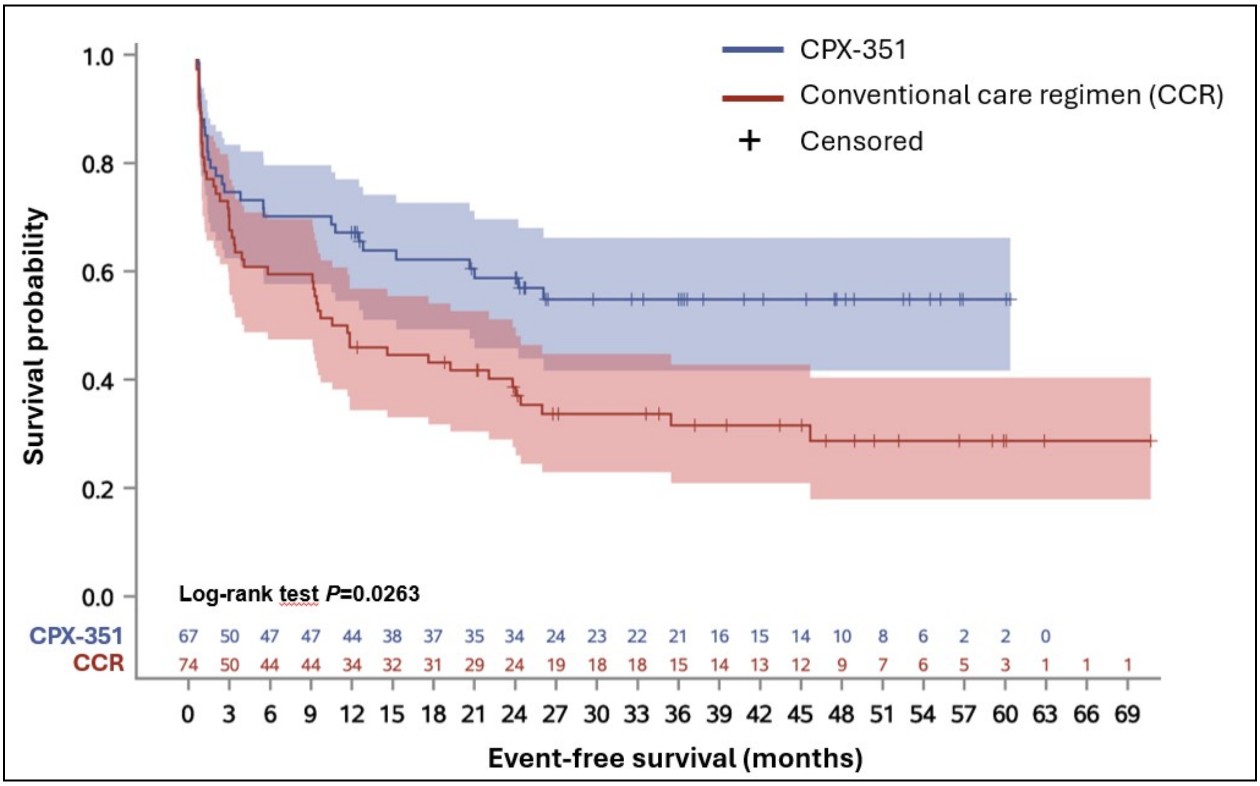

Figure 1: Event-free survival from the date of randomization in the full analysis set

**Copyright Information:** (Online) ISSN: 2572-9241
©2026 The Author(s). HemaSphere is published by John Wiley & Sons Ltd on behalf of European Hematology Association. This is an open access Abstract Book distributed under the Attribution-NonCommercial-NoDerivs (CC BY-NC-ND), which allows third parties to download the articles and share them with others as long as they credit the author and the Abstract Book, but they cannot change the content in any way or use them commercially.
**Abstract Book Citations:** Authors, Title, HemaSphere, 2026;10:(S1):pages. The abstract book DOIs can be found on the "Author Information" page.
**Disclaimer:** Articles published in the journal HemaSphere exclusively reflect the opinions of the authors. The authors are responsible for all content in their abstracts including accuracy of the facts, statements, citing resources, etc.

### (S171) EARLY VERSUS DELAYED ESA IN LOWER-RISK NON -TRANSFUSION-DEPENDENT MDS: FINAL PHASE III EPO-PRETAR RESULTS ON TRANSFUSION DEPENDENCE AND ERYTHROID RESPONSE

**Topic:** 10. Myelodysplastic syndromes - Clinical

Sophie Park*[1], Borhane Slama[2], Marius Moldovan[3], David Rombaut[4], Chloe Friedrich[4], Ismael Boussaid[4], Aurore Tourville[4], Yannick Simoni[4], Mathieu Meunier[1], Aspasia Stamatoullas Bastard[5], Maud D Aveni[6], Stephane Cheze[7], Sylvain Thepot[8], Miguel Torregrosa-Diaz[9], Sophie Dimicoli Salazar[10], Kamel Laribi[11], Reza Tabrizi[12], Anne Banos[13], Alice Garnier[14], Celia Salanoubat[15], Laurent Pascal[16], Clemence Santana[17], Ali Arar[18], Gaelle Fossard[19], Adrien Contejean[20], Ramdane Tekrane[21], Fatiha Chermat[22], Oliver Kosmider[23], Michaela Fontenay[24], Pierre Fenaux[25]

*[1]Université Grenoble Alpes, CHU Grenoble Alpes, INSERM U1209, CNRS 5309, Hematology, GRENOBLE, France;[2]CH Avignon, Hematology, Avignon, France; [3]CH Perigueux, Hematology, Perigueux, France; [4]Université Paris Cité, Institut Cochin, INSERM U1016, CNRSUMR8104; Assistance Publique-Hôpitaux de Paris Centre, Hôpital Cochin, Paris, Laboratory of Hematology, Paris, France; [5]Unicancer, Hematology, Rouen, France; [6]CHU Nancy, Hematology, Nancy, France;[7]Institut Basse Normandie, Hematology, Caen, France; [8]CHU Angers, Hematology, Angers, France;[9]CHU Poitiers, Hematology, Poitiers, France; [10]Hopital De Haut Leveque-hematologie Clinique, Hematology, Bordeaux, France; [11]Centre Hospitalier du Mans, Hematology, Le Mans, France; [12]Centre Hospitalier de Mont de Marsan, Hematology, Le Mont de Marsan, France;[13]C.H. Côte Basque, Hematology, Bayonne, France; [14]Hôpital Hôtel-Dieu, Hematology, Nantes, France; [15]Sud Francilien Hospital, Hematology, Corbeil Essones, France; [16]Groupement des Hôpitaux de l'Institut Catholique de Lille - Hôpital Saint Vincent de Paul, Hematology, Lille, France; [17]Gh De Valence, Valence, France; [18]CH Orleans, Hematology, Orleans, France; [19]Lyon University Hospital, Lyon, France, Hematology, Lyon, France; [20]CH Annecy, Hematology, Annecy, France; [21]Hopital Saint Louis, Hematology Senior, Paris, France; [22]Hopital Saint Louis, Hematology, Paris, France;[23]Assistance Publique-Hôpitaux de Paris. Centre-Université Paris Cité, Service d'hématologie biologique, Hôpital Cochin, Laboratory of Hematology, Paris, France; [24]Assistance Publique-Hôpitaux de Paris.Centre-Université Paris Cité, Service d'hématologie biologique, Hôpital Cochin, Paris France, Laboratory of Hematology, Paris, France; [25]Service d'Hématologie Séniors, Hôpital Saint-Louis, Université Paris 7, Hematology, Paris, France;*

### Background

Erythropoiesis-stimulating agents (ESAs) are the standard treatment for anemia in lower-risk myelodysplastic syndromes (MDS), yet the optimal timing of initiation remains debated. The EPO-PRETAR trial, randomized, multicenter, open-label trial, compared early versus delayed initiation of EPO ALFA in non- red blood cell (RBC) transfusion-dependent MDS patients. Interim results were presented at ASH 2024. We report here final clinical results (data cut-off: October 2025).

### Aims

To determine whether early initiation of EPO alfa in non–red blood cell (RBC) transfusion-dependent lower-risk MDS with anemia delays time to RBC transfusion dependence compared with starting EPO alfa only at the hemoglobin (Hb) threshold prompting RBC transfusion.

**Copyright Information:** (Online) ISSN: 2572-9241
©2026 The Author(s). HemaSphere is published by John Wiley & Sons Ltd on behalf of European Hematology Association. This is an open access Abstract Book distributed under the Attribution-NonCommercial-NoDerivs (CC BY-NC-ND), which allows third parties to download the articles and share them with others as long as they credit the author and the Abstract Book, but they cannot change the content in any way or use them commercially.
**Abstract Book Citations:** Authors, Title, HemaSphere, 2026;10:(S1):pages. The abstract book DOIs can be found on the "Author Information" page.
**Disclaimer:** Articles published in the journal HemaSphere exclusively reflect the opinions of the authors. The authors are responsible for all content in their abstracts including accuracy of the facts, statements, citing resources, etc.

**Methods**

In this open-label, randomized, multicenter phase III trial, non–transfusion-dependent lower-risk MDS patients with anemia were assigned to early EPO alfa (arm A: initiation at inclusion, baseline Hb 9–10.5 g/dL) or delayed EPO alfa (arm B: initiation at the center-defined Hb threshold for RBC transfusions <9 g/dL). The primary endpoint was time to RBC transfusion dependence (TD), defined as ≥2 transfusion episodes within 8 weeks after ≥12 weeks of ESA, analyzed using a cause-specific Cox model with death as competing event. Secondary endpoints included 12-week erythroid response (HI-E, IWG 2018; hemoglobin increase ≥1.5 g/dL vs nadir in the preceding 16 weeks), duration of response, overall survival (OS), progression-free survival (PFS), AML progression, safety, and quality of life (QOL; FACT-An, EQ-5D).

**Results**

Between 2018 and 2022, 118 patients were screened and 84 enrolled (43 early, 41 delayed); baseline characteristics were comparable between arms. EPO alfa was started in 41/43 (95%) arm A patients and in 33/41 (80%) arm B patients per protocol, with median time to EPO start of 0.2 months (95% CI 0.2–0.4) versus 16.2 months (95% CI 9.1–23.4), respectively. Time to TD did not differ significantly between strategies (HR 0.90; 95% CI 0.48–1.68; p=0.74); TD occurred in 21/43 (49%) arm A and 19/41 (46%) arm B patients, with 24- and 36-month TD probabilities of 26.2% and 43.4% versus 31.7% and 39.2%, respectively.
At week 12 after ESA initiation, HI-E was achieved in 35/41 (85%) arm A patients and 18/33 (55%) arm B patients (risk ratio 1.57; 95% CI 1.12–2.20; p=0.003). Median duration of erythroid response was numerically longer with early EPO (32.7 vs 24.9 months; p=0.18). OS and PFS were similar between arms: 20 deaths (47%) in arm A and 17 (41%) in arm B (HR for OS 0.84; 95% CI 0.44–1.61; p=0.60); median PFS 51.0 versus 38.0 months (HR 0.91; 95% CI 0.51–1.63; p=0.75), with 36-month PFS of 57.0% and 58.3%, respectively. AML transformation occurred in four patients per arm. Treatment was well tolerated, with no unexpected safety signals, and QOL trajectories did not differ between arms.

**Summary/Conclusion**

In lower-risk, non–transfusion-dependent MDS, early initiation of EPO alfa did not significantly delay RBC transfusion dependence or improve OS or PFS compared with delayed initiation at the transfusion Hb threshold. However, early EPO substantially increased early erythroid response rates and provided durable hematologic benefit without compromising safety or quality of life. These findings support tailoring the timing of ESA initiation to patient symptom burden and preferences, balancing the high probability of early hematologic improvement with the absence of demonstrated impact on transfusion dependence or survival.

**Copyright Information:** (Online) ISSN: 2572-9241
©2026 The Author(s). HemaSphere is published by John Wiley & Sons Ltd on behalf of European Hematology Association. This is an open access Abstract Book distributed under the Attribution-NonCommercial-NoDerivs (CC BY-NC-ND), which allows third parties to download the articles and share them with others as long as they credit the author and the Abstract Book, but they cannot change the content in any way or use them commercially.
**Abstract Book Citations:** Authors, Title, HemaSphere, 2026;10:(S1):pages. The abstract book DOIs can be found on the “Author Information” page.
**Disclaimer:** Articles published in the journal HemaSphere exclusively reflect the opinions of the authors. The authors are responsible for all content in their abstracts including accuracy of the facts, statements, citing resources, etc.

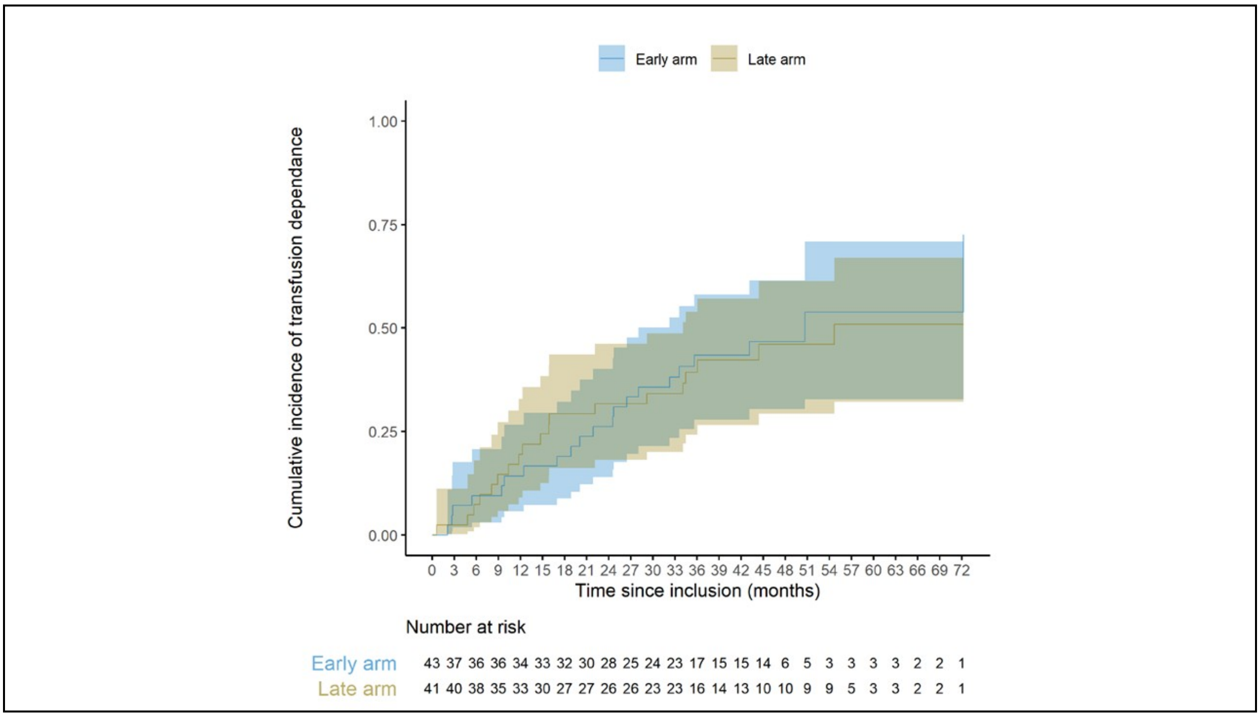

**Copyright Information:** (Online) ISSN: 2572-9241
©2026 The Author(s). HemaSphere is published by John Wiley & Sons Ltd on behalf of European Hematology Association. This is an open access Abstract Book distributed under the Attribution-NonCommercial-NoDerivs (CC BY-NC-ND), which allows third parties to download the articles and share them with others as long as they credit the author and the Abstract Book, but they cannot change the content in any way or use them commercially.
**Abstract Book Citations:** Authors, Title, HemaSphere, 2026;10:(S1):pages. The abstract book DOIs can be found on the "Author Information" page.
**Disclaimer:** Articles published in the journal HemaSphere exclusively reflect the opinions of the authors. The authors are responsible for all content in their abstracts including accuracy of the facts, statements, citing resources, etc.

### (S172) FUNCTIONAL TP53 PHENOTYPE SCORE REFINES PROGNOSTIC STRATIFICATION IN MONOALLELIC TP53-MUTATED MYELODYSPLASTIC NEOPLASMS BEYOND ALLELIC STATE

**Topic:** 10. Myelodysplastic syndromes - Clinical

Alexander Streuer*[1], Yotaro Ochi[2], Vladimir Riabov[1], Yasuhito Nannya[2, 3], Laurenz Steiner[1], Mohammed Abba[1], Georgia Metzgeroth[1], Eva Altrock[1], Felicitas Rapp[1], Verena Nowak[1], Edin Hepgülüm[4], Daniel Nowak[1], Wolf-Karsten Hofmann[1], Seishi Ogawa[2, 5, 6], Nanni Schmitt[1]

*[1]University Medical Center Mannheim, Heidelberg University, Department of Hematology and Oncology, Mannheim, Germany; [2]Kyoto University, Department of Pathology and Tumor Biology, Graduate School of Medicine, Kyoto, Japan;[3]University of Tokyo, Division of Hematopoietic Disease Control, The Institute of Medical Science, Tokyo, Japan; [4]Heidelberg University, Faculty of Biosciences, Heidelberg, Germany; [5]Kyoto University, Institute for the Advanced Study of Human Biology (WPI-ASHBi), Kyoto, Japan; [6]Kindai University, Department of Innovative Medicine, Faculty of Medicine, Osaka, Japan;*

### Background

*TP53* mutations represent one of the strongest adverse prognostic factors in myelodysplastic neoplasms (MDS). While multi-hit*TP53* (TP53multiHit) uniformly confers very poor outcomes, the prognostic relevance of monoallelic *TP53* (TP53mono) mutations remains controversial. *TP53* variants can cause loss-of-function, dominant-negative, or gain-of-function effects. Notably, survival differences reported across cohorts suggest substantial biological heterogeneity within TP53mono disease that is not captured by allelic classification alone (Bernard E et al., 2020; Weinberg OK et al., 2022).

### Aims

We therefore sought to determine whether variant-level functional annotation of individual *TP53* variants can explain this clinical heterogeneity and refine risk stratification beyond allelic state and established prognostic models.

### Methods

We analyzed 4,505 patients with MDS from two independent cohorts (IWG n=3,173; J-MDS n=1,332). TP53mono was present in 271 and TP53multiHit in 499 patients. All variants were functionally annotated using a previously published *TP53* phenotype score (PS) derived from saturation mutagenesis screens, which quantitatively capture dominant-negative and loss-of-function effects (Giacomelli AO et al., 2018). The PS reflects the functional pathogenicity of individual TP53 variants, with higher values indicating stronger dominant-negative or loss-of-function effects and lower values indicating near wild-type activity.

**Copyright Information:** (Online) ISSN: 2572-9241
©2026 The Author(s). HemaSphere is published by John Wiley & Sons Ltd on behalf of European Hematology Association. This is an open access Abstract Book distributed under the Attribution-NonCommercial-NoDerivs (CC BY-NC-ND), which allows third parties to download the articles and share them with others as long as they credit the author and the Abstract Book, but they cannot change the content in any way or use them commercially.
**Abstract Book Citations:** Authors, Title, HemaSphere, 2026;10:(S1):pages. The abstract book DOIs can be found on the “Author Information” page.
**Disclaimer:** Articles published in the journal HemaSphere exclusively reflect the opinions of the authors. The authors are responsible for all content in their abstracts including accuracy of the facts, statements, citing resources, etc.

**Results**

Median overall survival (OS) differed significantly according to *TP53* allelic state (*TP53* wild-type (TP53wt) 42.2 months; TP53mono 22.9 months; TP53multiHit 9.2 months; p<0.0001). In parallel, the frequency of complex karyotypes increased stepwise across these groups (4.1%, 32.2%, and 88.7%, respectively; p<0.001).
Patients harboring high PS variants (≥7) experienced significantly inferior OS compared with those carrying low PS variants (<7) (median 13.8 vs 39.2 months; HR 1.68, p=0.006). Consistently, high PS was also associated with increased AML transformation risk (HR 1.93, p=0.05). Especially within low-risk categories, PS identified a subgroup with markedly inferior survival (63.9 vs 23.1 months; HR 2.07, p=0.01).
Higher variant allele frequency (VAF ≥22%) was associated with shorter OS (HR 1.58, p=0.01). Notably, PS and VAF were not correlated ($R^2$=0.01), indicating that functional severity and clonal burden represent biologically distinct dimensions.
Finally, combining PS and VAF further enhanced discrimination. TP53mono patients with PS ≥7 and VAF ≥ 22% demonstrated survival comparable to TP53multiHit disease (8.8 vs 9.2 months), whereas those with PS <7 and VAF <22% exhibited outcomes similar to TP53wt cases (49.7 vs 42.2 months). Notably, this combined stratification strongly correlated with the distribution of complex karyotypes, with frequencies ranging from 12% in the PS <7 & VAF <22% subgroup to 81% in the PS ≥7 & VAF ≥22% subgroup.

**Summary/Conclusion**

Taken together, these findings demonstrate that monoallelic *TP53*-mutated MDS comprises biologically and clinically distinct subgroups. These data further indicate that *TP53* alterations should not be treated as a binary parameter of mutated versus wild-type, but rather as a spectrum defined by allelic state, functional severity, and clonal burden. Incorporation of quantitative *TP53* phenotype metrics may therefore improve individualized risk assessment, potentially identifying patients who may warrant earlier therapeutic escalation, or prioritization for transplant evaluation.

**Copyright Information:** (Online) ISSN: 2572-9241
©2026 The Author(s). HemaSphere is published by John Wiley & Sons Ltd on behalf of European Hematology Association. This is an open access Abstract Book distributed under the Attribution-NonCommercial-NoDerivs (CC BY-NC-ND), which allows third parties to download the articles and share them with others as long as they credit the author and the Abstract Book, but they cannot change the content in any way or use them commercially.
**Abstract Book Citations:** Authors, Title, HemaSphere, 2026;10:(S1):pages. The abstract book DOIs can be found on the "Author Information" page.
**Disclaimer:** Articles published in the journal HemaSphere exclusively reflect the opinions of the authors. The authors are responsible for all content in their abstracts including accuracy of the facts, statements, citing resources, etc.

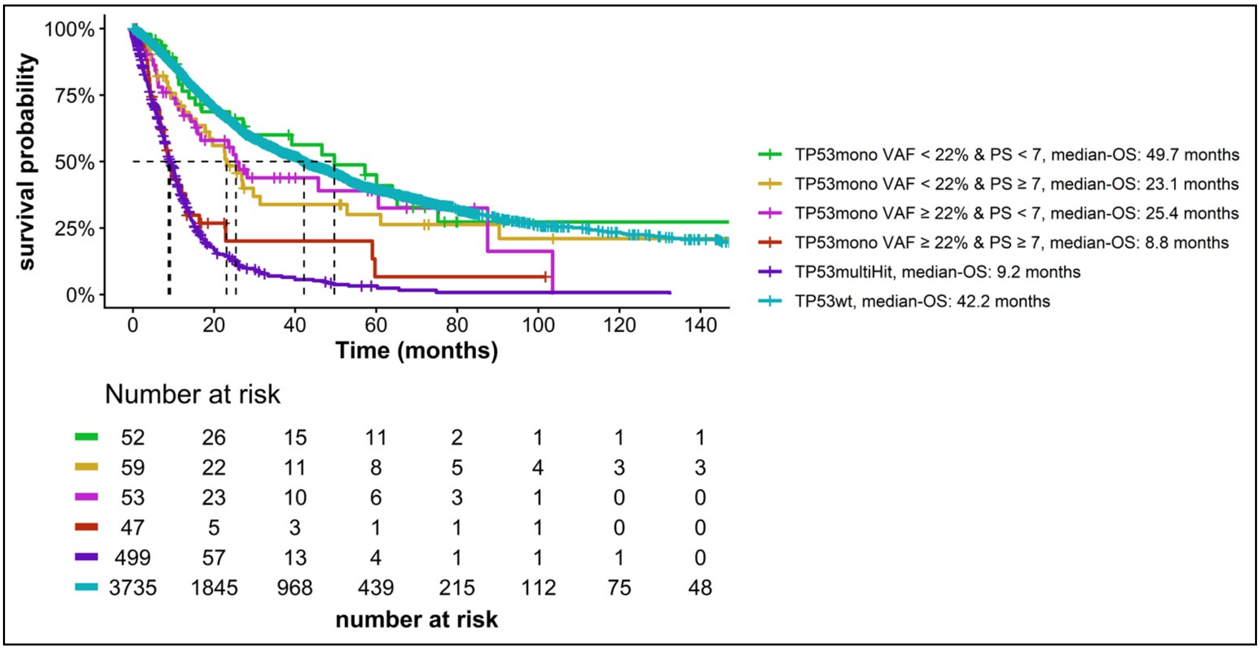

Kaplan-Meier curves for overall survival (OS) stratified by TP53 status and by combined phenotype score (PS) and variant allele frequency (VAF) groups. Monoallelic TP53 (TP53mono) cases are subdivided by PS (<7 vs ≥7) and VAF (<22% vs ≥22%). Multi-hit TP53 (TP53multiHit) and TP53 wild-type (TP53wt) are shown for comparison. Abbreviations: AML, acute myeloid leukemia; HR, hazard ratio; MDS, myelodysplastic neoplasms; OS, overall survival; PS, phenotype score; TP53mono, monoallelic TP53 mutation; TP53multiHit, multi-hit TP53 alteration; TP53wt, TP53 wild-type; VAF, variant allele frequency.

**Copyright Information:** (Online) ISSN: 2572-9241
©2026 The Author(s). HemaSphere is published by John Wiley & Sons Ltd on behalf of European Hematology Association. This is an open access Abstract Book distributed under the Attribution-NonCommercial-NoDerivs (CC BY-NC-ND), which allows third parties to download the articles and share them with others as long as they credit the author and the Abstract Book, but they cannot change the content in any way or use them commercially.
**Abstract Book Citations:** Authors, Title, HemaSphere, 2026;10:(S1):pages. The abstract book DOIs can be found on the “Author Information” page.
**Disclaimer:** Articles published in the journal HemaSphere exclusively reflect the opinions of the authors. The authors are responsible for all content in their abstracts including accuracy of the facts, statements, citing resources, etc.

### (S173) LUSPATERCEPT (LUSPA) INITIATED AT THE MAXIMUM-APPROVED DOSE IN PATIENTS (PTS) WITH LOWER-RISK MYELODYSPLASTIC SYNDROMES (LR-MDS) WHO REQUIRE TRANSFUSIONS: PRIMARY ANALYSIS FROM MAXILUS

**Topic:** 10. Myelodysplastic syndromes - Clinical

Matteo Giovanni Della Porta*[1], María Díez Campelo[2], Valeria Santini[3], Rena Buckstein[4], Lionel Adès[5], Bhavana Bhatnagar[6], Lea Sahagun[7], Yinzhi Lai[7], Dimana Mitev[8], Ali McBride[7], Krysztof Madry[9], Luke Fletcher[10], Dries Deeren[11], Thomas Cluzeau[12], David Valcárcel[13], José Torregrosa[14], Sebastian Grosicki[15], Eyck von der Hyde[16], Uwe Platzbecker[17], Sangeetha Venugopal[18], Amer M. Zeidan[19]

*[1]Cancer Center IRCCS Humanitas Research Hospital, Milan, Italy; Humanitas University, Milan, Italy; [2]University Hospital of Salamanca, Institute of Biomedical Research of Salamanca, Salamanca, Spain; [3]MDS Unit, Hematology, University of Florence, Florence, Italy; [4]Sunnybrook Health Sciences Centre, Toronto, ON, Canada; [5]Service Hématologie Séniors, Hôpital Saint-Louis (AP-HP), Paris Cité University and Paris INSERM U944, Paris, France; [6]West Virigina University Cancer Institute, Wheeling Hospital, Wheeling, WV, United States of America; [7]Bristol Myers Squibb, Princeton, NJ, United States of America;[8]Bristol Myers Squibb, Boudry, Switzerland; [9]Oncology and Internal Medicine, Medical University of Warsaw, Warsaw, Poland; [10]Willamette Valley Cancer Institute, Eugene, OR, United States of America; [11]AZ Delta Roeselare, Roeselare, Belgium; [12]Nice University Hospital, Nice, France; [13]Hospital Universitari Vall d'Hebron, Barcelona, Spain; [14]Service d'Hématologie, CHU de Poitiers, Poitiers, France; [15]Silesian Medical University, Katowice, Poland; [16]Medical Oncology, Internal Medicine, Onkologie am Raschplatz, Hannover, Germany; [17]University Hospital Leipzig, Leipzig, Germany; [18]Sylvester Comprehensive Cancer Center, University of Miami, Miami, FL, United States of America; [19]Yale University School of Medicine, New Haven, CT, United States of America;*

### Background

MAXILUS is an open-label, global, multicenter, ph 3b study (NCT06045689) evaluating LUSPA initiated at the maximum-approved dose (1.75 mg/kg) in pts with LR-MDS who are erythropoiesis-stimulating agent (ESA)–naive or –relapsed/refractory or intolerant (R/R/I) and require transfusions. In earlier analyses, LUSPA was well tolerated, with no new safety signals and efficacy consistent with its established clinical profile (Della Porta MG et al. *Lancet Haematol.* 2024; Fenaux P et al. *NEJM.* 2020).

### Aims

To report safety and efficacy outcomes of LUSPA based on primary analysis of MAXILUS after 24 wks of treatment (tx).

### Methods

Eligible pts (≥18 y; non-del[5q] LR-MDS per IPSS-R; ≥1 unit packed red blood cells [RBCs] ≤8 wks before tx; ECOG PS ≤2) were enrolled in ESA-naive or ESA-R/R/I cohorts and received SC LUSPA 1.75 mg/kg Q3W. Primary endpoint (PE) was RBC-transfusion independence (RBC-TI) ≥8 wks with concurrent mean Hb increase ≥1 g/dL (Wks 1–24). Secondary endpoints included RBC-TI ≥8 and ≥12 wks (Wks 1–24), modified erythroid hematologic improvement (mHI-E; Wks 1–24), and safety. Efficacy by subgroups was assessed.

**Copyright Information:** (Online) ISSN: 2572-9241
©2026 The Author(s). HemaSphere is published by John Wiley & Sons Ltd on behalf of European Hematology Association. This is an open access Abstract Book distributed under the Attribution-NonCommercial-NoDerivs (CC BY-NC-ND), which allows third parties to download the articles and share them with others as long as they credit the author and the Abstract Book, but they cannot change the content in any way or use them commercially.
**Abstract Book Citations:** Authors, Title, HemaSphere, 2026;10:(S1):pages. The abstract book DOIs can be found on the "Author Information" page.
**Disclaimer:** Articles published in the journal HemaSphere exclusively reflect the opinions of the authors. The authors are responsible for all content in their abstracts including accuracy of the facts, statements, citing resources, etc.

**Results**

At cutoff (Oct 1, 2025), 106 pts (53 per cohort) received ≥1 LUSPA dose; median age was 76.0 y in both cohorts. Median (IQR) time since diagnosis was 2.2 (0.3–9.2) and 24.8 (9.5–64.5) mos in ESA-naive and ESA-R/R/I cohorts, respectively. Median (IQR) follow-up (FU) was 11.1 (7.9–13.7) and 11.6 (9.0–13.9) mos; 52.8% and 56.6% had an *SF3B1* mutation; 41.5% and 37.7% had ring sideroblast–negative (RS–) status; and 77.4% and 45.3% had baseline (BL) serum erythropoietin (sEPO) ≤200 IU/L, respectively.
*ESA-naive pts:* PE was achieved in 81.1% of pts (median time to TI, 1 [range, 1–111] day); RBC-TI ≥8 wks in 83.0% and ≥12 wks in 75.5% (**Figure 1**). RBC-TI ≥12 wks was achieved in 72.7% of RS– pts and 85.7% of BL sEPO ≤200 IU/L pts. mHI-E was achieved in 86.8% of pts.
At cutoff, 69.8% of pts remained on tx, 1.9% completed, and 28.3% discontinued (11.3% from lack of efficacy). Median tx duration was 47.4 (range, 10.7–71.1) wks; 22.6% of pts had ≥1 dose reduction; 52.8% had ≥1 dose delay. Any grade tx-emergent adverse events (TEAEs) occurred in 94.3% of pts (30.2% tx-related); 20.8% led to tx interruption; grade 3/4 TEAEs occurred in 66.0% (11.3% tx-related). HR-MDS progression occurred in 3.8% of pts; none progressed to AML.
*ESA-R/R/I pts*: PE was achieved in 62.3% of pts (median time to TI, 2 [range, 1–148] days); RBC-TI ≥8 wks in 67.9% and ≥12 wks in 50.9% (**Figure 1**). RBC-TI ≥12 wks was achieved in 40.0% of RS– pts and 58.3% of BL sEPO ≤200 IU/L pts. mHI-E was achieved in 77.4% of pts.
At cutoff, 39.6% of pts remained on tx, 1.9% completed, and 58.5% discontinued (22.6% from lack of efficacy). Median tx duration was 41.0 (range, 2.1–97.1) wks; 11.3% of pts had ≥1 dose reduction; 43.4% had ≥1 dose delay. Any grade TEAEs occurred in 100% of pts (45.3% tx-related); 20.8% led to tx interruption; grade 3/4 TEAEs occurred in 54.7% (7.5% tx-related). HR-MDS progression occurred in 3.8% of pts; none progressed to AML.

**Summary/Conclusion**

With 11 mos FU, LUSPA initiated at 1.75 mg/kg led to high, sustained PE achievement in both cohorts. Sustained RBC-TI ≥12 wks occurred in pts with RS– status and low BL sEPO. RBC-TI and mHI-E were notable in both cohorts. LUSPA was well tolerated; few pts progressed to HR-MDS, in line with known rates, and none to AML. The MAXILUS primary analysis reinforces the favorable safety and efficacy profile of LUSPA at the maximum-approved dose in LR-MDS.

**Figure 1: Primary Analysis of RBC-TI ± Hb Increase ≥8 Wks and RBC-TI ≥12 Wks for LUSPA Tx in the MAXILUS Study Among the ESA-naive Cohort and the ESA-R/R/I Cohort**

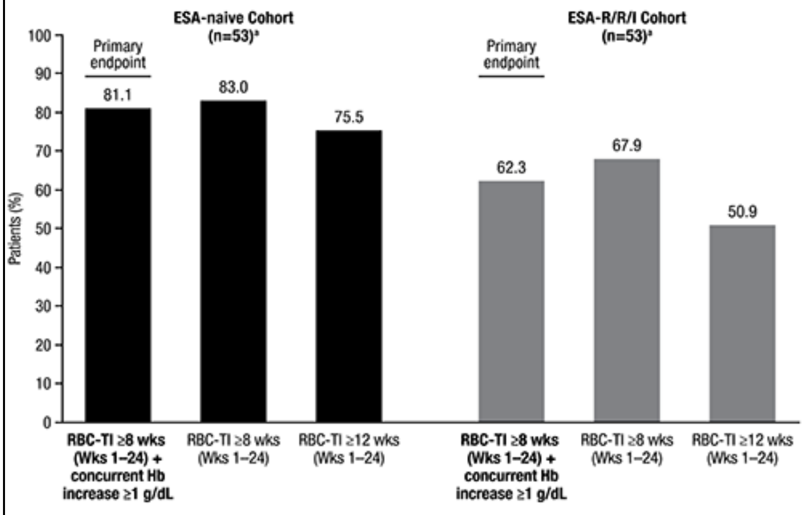

Data cutoff: October 1, 2025.
ESA, erythropoiesis-stimulating agent; Hb, hemoglobin; R/R/I, relapsed/refractory or intolerant;
RBC-TI, red blood cell-transfusion independence; wks, weeks; tx, treatment.
[a]Efficacy-evaluable population (patients who received first tx ≥24 wks prior to data cutoff).

**Copyright Information:** (Online) ISSN: 2572-9241
©2026 The Author(s). HemaSphere is published by John Wiley & Sons Ltd on behalf of European Hematology Association. This is an open access Abstract Book distributed under the Attribution-NonCommercial-NoDerivs (CC BY-NC-ND), which allows third parties to download the articles and share them with others as long as they credit the author and the Abstract Book, but they cannot change the content in any way or use them commercially.
**Abstract Book Citations:** Authors, Title, HemaSphere, 2026;10:(S1):pages. The abstract book DOIs can be found on the "Author Information" page.
**Disclaimer:** Articles published in the journal HemaSphere exclusively reflect the opinions of the authors. The authors are responsible for all content in their abstracts including accuracy of the facts, statements, citing resources, etc.

### (S174) OFIRNOFLAST, A FIRST-IN-CLASS NEK7 INHIBITOR, INDUCES ROBUST TRANSFUSION INDEPENDENCE AND MULTILINEAGE HEMATOLOGICAL IMPROVEMENT IN LOWER-RISK MYELODYSPLASTIC SYNDROME(LR-MDS): PHASE 2 RESULTS

**Topic:** 10. Myelodysplastic syndromes - Clinical

Varun Bafna*[1], Uttam Kumar Nath[2], Sandip Shah[3], Arijit Nag[4], Chandran Nair[5], Ramesh Uppada[6], Devan Bursey[7], Ajisa Muthayil Ali[7], Jeremiah Bearss[7], Alan List[7], David Bearss[7]

*[1]Star Superspeciality Clinic and Hospital, Kolhapur, India;[2]All India Institute of Medical Sciences, Rishikesh, India;[3]Hemato Oncology Clinic Vedanta Institute of Medical Sciences, Admedabad, India; [4]Tata Medical Center, Kolkata, India;[5]Malabar Cancer Centre, Kerala, India; [6]HCG Cancer Centre, Vishakhapatnam, India;[7]Halia Therapeutics, Lehi, United States of America;*

### Background

NLRP3 inflammasome hyperactivation is a central driver of ineffective hematopoiesis in MDS through caspase-1 activation, pyroptotic cell death of hematopoietic progenitor cells, and GATA-1 degradation. Ofirnoflast (HT-6184) is the first-in-class, oral, allosteric inhibitor of NEK7, a scaffold protein essential for NLRP3 inflammasome assembly. NEK7 inhibition prevents inflammasome assembly and downstream inflammatory signaling, thereby restoring multilineage hematopoiesis.

### Aims

Evaluate the safety and efficacy of ofirnoflast in ESA-refractory, lower-risk MDS.

### Methods

This open-label, single-arm, phase 2 trial enrolled ESA-refractory, intolerant, or ineligible adults with IPSS-R lower-risk MDS (score ≤4.5) and symptomatic anemia (hgb < 9 g/dL) or RBC transfusion-dependence (TD). Ofirnoflast was administered orally at 2 mg QD for up to 32 weeks, 5 days on/2 days off. The primary endpoint was hematological improvement (HI) per the IWG 2018 criteria.

**Copyright Information:** (Online) ISSN: 2572-9241
©2026 The Author(s). HemaSphere is published by John Wiley & Sons Ltd on behalf of European Hematology Association. This is an open access Abstract Book distributed under the Attribution-NonCommercial-NoDerivs (CC BY-NC-ND), which allows third parties to download the articles and share them with others as long as they credit the author and the Abstract Book, but they cannot change the content in any way or use them commercially.
**Abstract Book Citations:** Authors, Title, HemaSphere, 2026;10:(S1):pages. The abstract book DOIs can be found on the “Author Information” page.
**Disclaimer:** Articles published in the journal HemaSphere exclusively reflect the opinions of the authors. The authors are responsible for all content in their abstracts including accuracy of the facts, statements, citing resources, etc.

### Results

37 patients were treated [ages 31-86, 62% male]. 18/37 (49%) had a high transfusion burden (HTB), 5/37 (14%) had a low TB (LTB), and 14/37 (43%) were non-TD (NTD) per IWG 2018. Disease subtypes included MDS with low blasts (25/37), SF3B1 (2/37), MDS/MPN (4/37), del(5q) (3/37), and increased blasts (3/37) with a median IPSS-R score of 3.5. Median treatment duration was 32 weeks.
Ofirnoflast demonstrated a highly favorable safety profile. Treatment-related adverse events (TRAEs) occurred in 27% of patients; with only two grade ≥3 TRAEs [hypertension (n=1), leukopenia, (n=1)]. Zero treatment-related serious adverse events were reported. One patient with an initial diagnosis of MDS/MPN discontinued due to TRAE (leukocytosis).

Among the 30 evaluable patients (7 subjects were found to be ineligible or did not complete the treatment period), Ofirnoflast achieved a 67% overall HI response rate comprising 63% erythroid (HI-E), 60% platelet (HI-P), and 50% neutrophil (HI-N) responses. 55% of TD patients achieved transfusion independence (TI) for ≥8 weeks and 39% for ≥16 weeks, with a median TI duration of 28.5 weeks. Among NTD patients, 75% achieved HI-E. The median maximum hemoglobin rise in HI-E among responders was 4.2 g/dL from a baseline of 7.5 g/dL. Morphologic responses included complete remission (CR) in 9/30 (30%) and marrow CR in 1/30 (3%) patients. HI was observed across all WHO MDS subtypes and mutation backgrounds, including *SF3B1* and Del5q.

Pharmacodynamic data confirmed on-target activity, with significant reductions in TNF-alpha (mean change −5.5 pg/mL; q=0.048) and large reductions in oxidized DNA (mean change −105 pg/mL, q=0.052) after 16 weeks of treatment. Quality of life metrics notably improved, with the greatest gains in HI responders being in overall QOL-E score, fatigue and social/family well-being domains.

| Outcome | Efficacy Population |
|---|---|
| TI | 55% (10/18) |
| Median TI Duration | 28.5 weeks |
| Overall HI Rate | 67% (20/30) |
| HI-E | 63% (19/30) |
| HI-P | 60% (3/5) |
| HI-N | 50% (2/4) |

### Summary/Conclusion

Ofirnoflast demonstrated clinically meaningful, multilineage efficacy in ESA-refractory LR-MDS, with a 67% overall HI rate and 55% transfusion independence in TD patients. It also had an exceptional safety profile with no treatment-related serious adverse events. These results represent the first clinical validation that NEK7 blockade can durably suppress the NLRP3 inflammasome and restore hematopoiesis across all MDS subtypes, supporting Ofirnoflast as a transformative treatment for this high-unmet-need population.

**Copyright Information:** (Online) ISSN: 2572-9241
©2026 The Author(s). HemaSphere is published by John Wiley & Sons Ltd on behalf of European Hematology Association. This is an open access Abstract Book distributed under the Attribution-NonCommercial-NoDerivs (CC BY-NC-ND), which allows third parties to download the articles and share them with others as long as they credit the author and the Abstract Book, but they cannot change the content in any way or use them commercially.
**Abstract Book Citations:** Authors, Title, HemaSphere, 2026;10:(S1):pages. The abstract book DOIs can be found on the “Author Information” page.
**Disclaimer:** Articles published in the journal HemaSphere exclusively reflect the opinions of the authors. The authors are responsible for all content in their abstracts including accuracy of the facts, statements, citing resources, etc.

### (S175) CLONAL ARCHITECTURE AND DYNAMICS OF SOMATIC EVOLUTION IN APLASTIC ANEMIA AND PAROXYSMAL NOCTURNAL HEMOGLOBINURIA

**Topic:** 11. Bone marrow failure syndromes incl. PNH - Biology & translational research

Kudzai Nyamondo*[1, 2], Michael Spencer Chapman[1, 3], Nicholas Williams[1], Daniel Leongamornlert[1], E. Joanna Baxter[2, 4, 5], Behnaz Shojaeddin Givi[6], Matthew Mayho[1], Toni Spence[1], Matthew Holt[6, 7], Anastasia Goryanin[4, 8], Theodora Foukaneli[2, 4, 5], Duncan Brian[2, 4, 5], Richard J. Kelly[9], Darren Newton[6], Alan Warren[2, 4, 5, 8], Jyoti Nangalia[1, 2, 4, 5]

*[1]Wellcome Sanger Institute, Somatic Genomics, Hinxton, United Kingdom;[2]University of Cambridge, Cambridge Stem Cell Institute, Cambridge, United Kingdom; [3]Barts Cancer Institute, Centre for Haemato-Oncology, London, United Kingdom; [4]University of Cambridge, Department of Haematology, Cambridge, United Kingdom;[5]Cambridge University Hospitals NHS Foundation Trust, Cambridge, United Kingdom; [6]University of Leeds, Leeds Institute of Medical Research, Leeds, United Kingdom; [7]Leeds Teaching Hospitals NHS Trust, Leeds, United Kingdom;[8]University of Cambridge, Cambridge Institute of Medical Research, Cambridge, United Kingdom; [9]St. James's University Hospital, Department of Haematology, Leeds, United Kingdom;*

### Background

Aplastic anaemia (AA) is an immune-mediated bone marrow failure characterized by T-cell driven attack against haematopoietic stem and progenitor cells (HSPCs), leading to pancytopenia. Current classification of disease reflects severity of cytopenias with limited consideration of the underlying mutational landscape. Although immunosuppressive therapy (IST) is standard treatment, 30–40% of patients fail to respond, with 10–15% of responders subsequently developing myeloid malignancies. Expansion of *PIGA*-mutant clones may also occur, resulting in paroxysmal nocturnal haemoglobinuria (PNH). While somatic mutations are increasingly recognized in AA, bulk sequencing approaches cannot resolve underlying clonal dynamics or mutational processes and have limited use in clinical decision making.

### Aims

We sought to define the clonal architecture, mutation timing, and selective pressures shaping haematopoiesis in AA and PNH using whole genome single-cell phylogenetic reconstruction.

### Methods

Genome-wide somatic mutations were identified in 657 cells from 9 patients (6 AA, 3 PNH), by whole-genome sequencing of colonies (340 genomes), or primary template amplified single cells from myeloid and lymphoid cells (317 genomes). Phylogenetic reconstruction and mutational signature analysis was undertaken to infer mutation timing and underlying mutational processes. Duplex whole-exome sequencing was conducted on granulocytes from 13 patients, and genes under positive selection were identified using dN/dS analysis.

**Copyright Information:** (Online) ISSN: 2572-9241
©2026 The Author(s). HemaSphere is published by John Wiley & Sons Ltd on behalf of European Hematology Association. This is an open access Abstract Book distributed under the Attribution-NonCommercial-NoDerivs (CC BY-NC-ND), which allows third parties to download the articles and share them with others as long as they credit the author and the Abstract Book, but they cannot change the content in any way or use them commercially.
**Abstract Book Citations:** Authors, Title, HemaSphere, 2026;10:(S1):pages. The abstract book DOIs can be found on the "Author Information" page.
**Disclaimer:** Articles published in the journal HemaSphere exclusively reflect the opinions of the authors. The authors are responsible for all content in their abstracts including accuracy of the facts, statements, citing resources, etc.

### Results

**(i)** AA patients demonstrated two distinct mutational landscapes; myeloid driver predominant (MDP) and immune escape predominant (IEP). MDP patients had parallel clonal expansions with mutations in genes such as *ASXL1, U2AF1, TET2* or *BCOR.* IEP patients had multiple clones with inactivated HLA genes -either by 6p loss-of-heterozygosity (LOH) or nonsense mutations. **(ii)** Novel recurrent alterations were identified in *ERAP2* in two individuals, a gene involved in antigen processing, suggesting an additional mechanism of immune evasion. Intra-individual recurrence of mutations was high, suggesting patient specific selective landscapes. **(iii)** Our data showed that immune escape events frequently arose early in life. In one individual presenting with AA at age 8, the dominant 6p LOH clone was estimated to originate in utero (<15 weeks post-conception). **(iv)** Phylogenetic reconstruction of haematopoiesis across the haematopoietic compartment in two AA individuals showed distinct, non-overlapping clonal expansions HSPCs, CD8+ T cells and B cells. Clonal expansions of CD8+ T cells were observed to emerge in parallel in one individual with an IEP clonal landscape. **(v)** Among 3 PNH patients, both monoclonal *PIGA* expansion (2 of 3 patients) and polyclonal *PIGA* expansion (1 patient with 4 independent *PIGA* mutations) patterns were observed. *PIGA* mutations were acquired prior to age 15, though clinical onset occurred decades later.

### Summary/Conclusion

Our data represent the largest whole genome dataset in AA (>650 genomes) spanning myeloid and lymphoid genomes. We show biologically distinct AA evolutionary trajectories defined by immune escape or myeloid drivers. We identify novel *ERAP2* mutations under positive selection. Immune escape arises early and expands gradually, whereas myeloid drivers emerge later, expanding more rapidly. A revised AA classification that integrates clonal architecture could guide treatment, avoid ineffective IST, and identify those at risk of malignant transformation.

**Copyright Information:** (Online) ISSN: 2572-9241
©2026 The Author(s). HemaSphere is published by John Wiley & Sons Ltd on behalf of European Hematology Association. This is an open access Abstract Book distributed under the Attribution-NonCommercial-NoDerivs (CC BY-NC-ND), which allows third parties to download the articles and share them with others as long as they credit the author and the Abstract Book, but they cannot change the content in any way or use them commercially.
**Abstract Book Citations:** Authors, Title, HemaSphere, 2026;10:(S1):pages. The abstract book DOIs can be found on the "Author Information" page.
**Disclaimer:** Articles published in the journal HemaSphere exclusively reflect the opinions of the authors. The authors are responsible for all content in their abstracts including accuracy of the facts, statements, citing resources, etc.

### (S176) IN VIVO LENTIVIRAL GENE TRANSFER INTO HEMATOPOIETIC STEM CELLS FOR THE TREATMENT OF FANCONI ANEMIA

**Topic:** 11. Bone marrow failure syndromes incl. PNH - Biology & translational research

Michela Milani*[1], Marta Perez Rodriguez[2], Anna Fabiano[1], Luca Basso-Ricci[1], Cesare Canepari[1], Monica Volpin[1], Pamela Quaranta[1], Luca Seffin[1, 3], Fabio Russo[1], Mauro Biffi[1], Leonardo Ormoli[1], Chiara Brombin[4], Serena Scala[1], Juan Bueren[2], Paula Río[2], Alessandro Aiuti[1, 3], Alessio Cantore[1, 3], Luigi Naldini[1, 3]

*[1]San Raffaele Telethon Institute for Gene Therapy, Milan, Italy;[2]Division of Hematopoietic Innovative Therapies, Centro de Investigaciones Energéticas Medioambientales y Tecnológicas and Centro de Investigación Biomédica en Red de Enfermedades Raras (CIEMAT/CIBERER); Advanced Therapies Unit, Instituto de Investigación Sanitaria Fundación Jiménez Díaz (IIS-FJD, UAM), Madrid, Spain; [3]Vita-Salute San Raffaele University, Milan, Italy; [4]University Center for Statistics in the Biomedical Sciences, Vita-Salute San Raffaele, Milan, Italy;*

### Background

Lentiviral vector (LV)-mediated *ex vivo* gene therapy (GT) in hematopoietic stem and progenitor cells (HSPCs) fulfilled the promise of a cure for different genetic diseases. However, the collection and *ex vivo* manipulation of HSPCs, as well as the risks associated with patient conditioning and transplant, still pose challenges to broad access to HSPC-GT. These hurdles might be overcome by an *in vivo* approach.

### Methods

We studied HSPC biodistribution over time in mice using advanced multiparametric cytometric analysis. We then administered intravenously (i.v.) LV expressing a reporter marker to mice to evaluate *in vivo* gene transfer to HSPCs. We performed clonal tracking, transplantation experiments, and long-term follow-up analyses to confirm LV-mediated gene transfer in primitive long-term hematopoietic stem cells (HSCs). As a paradigmatic disease to test our strategy, we selected Fanconi anemia (FA), a rare genetic disorder belonging to the DNA repair deficiency syndromes, characterized by BM failure and cancer predisposition.

**Copyright Information:** (Online) ISSN: 2572-9241
©2026 The Author(s). HemaSphere is published by John Wiley & Sons Ltd on behalf of European Hematology Association. This is an open access Abstract Book distributed under the Attribution-NonCommercial-NoDerivs (CC BY-NC-ND), which allows third parties to download the articles and share them with others as long as they credit the author and the Abstract Book, but they cannot change the content in any way or use them commercially.
**Abstract Book Citations:** Authors, Title, HemaSphere, 2026;10:(S1):pages. The abstract book DOIs can be found on the "Author Information" page.
**Disclaimer:** Articles published in the journal HemaSphere exclusively reflect the opinions of the authors. The authors are responsible for all content in their abstracts including accuracy of the facts, statements, citing resources, etc.

**Results**

We discovered a unique window of opportunity in newborn mice, due to extensive early postnatal trafficking of HSCs to the maturing bone marrow (BM). We successfully targeted *bona fide* HSCs by i.v. administration of GFP-LV to newborn mice. We obtained stable, life-long GFP expression in up to 10% of all blood lineages, paralleled by a comparable expression in HSPCs harvested from the BM, which could engraft long-term in busulfan-conditioned mice. LV integration site analysis confirmed the common origin of different hematopoietic lineages from multiple clones. To extend the intervention window, we applied to 2-week-old mice a G-CSF/Plerixafor mobilization regimen, modelling the clinical use. We observed a sharp increase in circulating HSPCs, achieving 8% GFP-positive cells, 10-fold higher than in non-mobilized controls. We also confirmed increased GT efficiency upon HSPC mobilization in newborn mice, reaching up to 13% GFP-positive cells in PB. To increase GT efficiency, we exploited phagocytosis-shielded CD47-high LV and showed 3-fold higher gene marking in the blood and BM of treated mice. In addition, we confirmed effective LV-mediated *in vivo* gene transfer in human HSPCs in humanized newborn NBSGW mice, reaching up to 6% of GFP-positive human cells in PB and in human CD34+ cells in the BM. We then tested this strategy in FA mice and observed selective advantage of corrected cells, coupled with progressive normalization of white blood cell count. *In vivo* administration of Mitomycin C (MMC), a DNA cross-linking agent, led to a rapid expansion of corrected cells, reaching up to 100% of marking, with prompt recovery of blood parameters only in LV-treated mice. Importantly, we also measured LV-marking in other organs, including the liver and the oral mucosa, a relevant aspect for FA, since impairment of the DNA-damage repair pathway predisposes to cancer across different tissues. Notably, by applying the mobilization regimen described above, we achieved effective GT in 2-week-old and adult FA mice, with 32% and 22% of LV-marked cells, respectively, in PB 1 year after treatment.

**Summary/Conclusion**

Our work shows the efficacy and safety of *in vivo* GT to HSPCs in mice and lifelong maintenance of transgene expression. This, together with the detection of high numbers of circulating HSPCs early postnatal also in humans, lays the basis for possible clinical translation of this approach.

**Copyright Information:** (Online) ISSN: 2572-9241
©2026 The Author(s). HemaSphere is published by John Wiley & Sons Ltd on behalf of European Hematology Association. This is an open access Abstract Book distributed under the Attribution-NonCommercial-NoDerivs (CC BY-NC-ND), which allows third parties to download the articles and share them with others as long as they credit the author and the Abstract Book, but they cannot change the content in any way or use them commercially.
**Abstract Book Citations:** Authors, Title, HemaSphere, 2026;10:(S1):pages. The abstract book DOIs can be found on the “Author Information” page.
**Disclaimer:** Articles published in the journal HemaSphere exclusively reflect the opinions of the authors. The authors are responsible for all content in their abstracts including accuracy of the facts, statements, citing resources, etc.

### (S177) DISTINCT IMMUNE DIVERSITY OF CD8+ T CELLS DEFINES PEDIATRIC HEPATITIS-ASSOCIATED APLASTIC ANEMIA AND SEVERE APLASTIC ANEMIA

**Topic:** 11. Bone marrow failure syndromes incl. PNH - Biology & translational research

Cheng Xuelian[1], Ming Chen[2], Xingjie Gao[3], Xiaofan Zhu[1], Lixian Chang*[4]

[1]*Institute of Hematology and Blood Diseases Hospital, Chinese Academy of Medical Sciences and Peking Union Medical College, State Key Laboratory of Experimental Hematology, Tianjin, China;* [2]*Nanfang Hospital, Southern Medical University, Department of Hematology, GuangZhou, China;* [3]*Tianjin Medical University, School of Basic Medical Science, Tianjin, China;* [4]*Institute of Hematology and Blood Diseases Hospital, Chinese Academy of Medical Sciences and Peking Union Medical College, State Key Laboratory of Experimental Hematology, Institute of Hematology and Hospital of Blood Diseases, Tianjin, China;*

### Background

Hepatitis-associated aplastic anemia (HAAA) is a rare form of acquired aplastic anemia (AA) that occurs after hepatitis. The immune landscape of pediatric Hepatitis-associated aplastic anemia (HAAA) is poorly characterized. The specific mechanisms differentiating HAAA from AA, particularly the interaction between hepatitis and AA, are not well understood. While immune-mediated destruction of hematopoietic stem and progenitor cells (HSPCs) is a key feature, the specific roles of T-cell subsets, particularly CD8+ effector T cells, in the pathogenesis of HAAA remain incompletely understood.

### Aims

This study aimed to characterize the transcriptomic profiles and functional states of bone marrow T cells in pediatric HAAA (pedHAAA) compared to pediatric severe aplastic anemia (pedSAA) and healthy donors (pedHD). We sought to identify key immune cell subsets, dysregulated pathways, and potential therapeutic targets driving the immunopathology of HAAA.

### Methods

We analyzed single-cell RNA sequencing (scRNA-seq) data from bone marrow mononuclear cells (BMMCs) of 6 pedHAAA patients, integrated with public datasets of 6 pedHD, 5 pedSAA, and 7 adult SAA samples. Paired T-cell receptor sequencing (scTCR-seq) was performed on the pedHAAA samples. Unsupervised clustering, pseudotime trajectory analysis, cell-cell communication (CellChat), and transcription factor regulon analysis (SCENIC) were employed. Key findings were validated using flow cytometry and in vitro functional assays on $CD8^+$ T cells.

### Results

We observed a significant expansion of $CD8^+$ Effector T cells in pedHAAA (39.63%) compared to pedSAA (6.26%) and pedHD (17.67%). These cells exhibited enhanced TCR clonality and cytotoxic gene expression (LAG3, GZMB, PRF1). PedHAAA samples showed significant heterogeneity, with a subgroup ("high clonal expansion") displaying stronger cytotoxic features. Cell-cell communication analysis identified the MIF-CD74 pathway as a major mediator of T-cell activation in HAAA. Notably, we found a significant enrichment of immune exhaustion and suppression signatures in pedHAAA, with LAG3 and TGFB1 being key drivers. SCENIC analysis revealed heightened activity of transcription factors TBX21 and STAT1 in $CD8^+$ Effector T cells. In vitro experiments confirmed that the LAG3-FGL1 axis significantly suppressed $CD8^+$ T-cell proliferation in HAAA patients. Finally, pedSAA displayed a more naive T-cell phenotype compared to adult SAA, indicating that disease type (HAAA vs. SAA) has a stronger impact on the immune profile than age.

**Copyright Information:** (Online) ISSN: 2572-9241
©2026 The Author(s). HemaSphere is published by John Wiley & Sons Ltd on behalf of European Hematology Association. This is an open access Abstract Book distributed under the Attribution-NonCommercial-NoDerivs (CC BY-NC-ND), which allows third parties to download the articles and share them with others as long as they credit the author and the Abstract Book, but they cannot change the content in any way or use them commercially.
**Abstract Book Citations:** Authors, Title, HemaSphere, 2026;10:(S1):pages. The abstract book DOIs can be found on the “Author Information” page.
**Disclaimer:** Articles published in the journal HemaSphere exclusively reflect the opinions of the authors. The authors are responsible for all content in their abstracts including accuracy of the facts, statements, citing resources, etc.

**Summary/Conclusion**

Our study provides a comprehensive single-cell atlas of bone marrow T cells in pediatric HAAA. We identify a critical role for a clonally expanded, cytotoxic, and exhausted CD8+ Effector T-cell population in HAAA pathogenesis. The data highlight the LAG3-FGL1 axis as a novel mechanism of T-cell dysfunction and a promising immunotherapeutic target. The MIF-CD74 pathway and key regulons like TBX21 and STAT1 also represent potential nodes for intervention. The substantial heterogeneity among HAAA patients underscores the need for personalized approaches to immunotherapy.

**Copyright Information:** (Online) ISSN: 2572-9241
©2026 The Author(s). HemaSphere is published by John Wiley & Sons Ltd on behalf of European Hematology Association. This is an open access Abstract Book distributed under the Attribution-NonCommercial-NoDerivs (CC BY-NC-ND), which allows third parties to download the articles and share them with others as long as they credit the author and the Abstract Book, but they cannot change the content in any way or use them commercially.
**Abstract Book Citations:** Authors, Title, HemaSphere, 2026;10:(S1):pages. The abstract book DOIs can be found on the "Author Information" page.
**Disclaimer:** Articles published in the journal HemaSphere exclusively reflect the opinions of the authors. The authors are responsible for all content in their abstracts including accuracy of the facts, statements, citing resources, etc.

### (S178) LONG-TERM OUTCOMES IN PATIENTS WITH RELAPSED OR REFRACTORY SEVERE APLASTIC ANEMIA

**Topic:** 12. Bone marrow failure syndromes incl. PNH - Clinical

Fieke Hoff*[1], Luiz Bazzo Catto[1], Ruba Shalhoub[1], Olga Rios[1], Colin Wu[1], Neal Young[1], Emma Groarke[1], Bhavisha Patel[1]

[1]*National Institutes of Health, Bethesda, United States of America;*

### Background

Acquired severe aplastic anemia is treated with allogenic hematopoietic stem cell transplant (HSCT) or immunosuppressive therapy (IST), containing horse anti-thymocyte globulin (h-ATG), cyclosporin (CSA) with or without eltrombopag (EPAG), depending on age, fitness, and donor availability. Despite high initial response rates, 10-20% of patients are refractory and 30-40% of responders will eventually relapse and may require further therapy. Long-term outcomes in relapsed or refractory SAA remain poorly characterized.

### Aims

To evaluate long-term outcomes of patients with SAA who relapsed or were refractory to first line (1L) ATG-based therapy.

### Methods

Patients treated with ATG-based therapy on prospective clinical trials at the NIH Clinical Center (CC) between 1993 and 2023 who were refractory or relapsed were assessed retrospectively. All patients or their guardians provided written informed consent. OS was calculated from 1L-therapy, RFS and EFS from second line (2L) therapy. RFS and EFS were censored at time of last visit at the NIH CC. Events for EFS included subsequent relapse, requirement for next-line therapy, high risk clonal evolution (defined as myeloid neoplasm, chromosome 7 abnormalities, or complex karyotype) or death.

### Results

A total of 296 patients were included in the study; 146 were relapsed and 150 were refractory. The refractory and relapsed cohorts had similar median age (29 (range, 2-87) vs. 34 (range, 3-78) years; p=0.496) and sex distribution (female, 40% vs. 52.1%; p=0.115). Forty-three percent (n=63) and 12% (n=18) of relapsed and refractory patients, respectively, received 1L ATG, CSA plus EPAG.

In the relapsed cohort, patients received the following 2L-therapies: oral therapies, including CSA, EPAG and danazol (n=90, 61,6%), alemtuzumab (n=25, 17.1%), ATG-based IST (n=18, 12.3% (h-ATG (n=4), r-ATG (n=14)), HSCT (n=8, 5.5%), none (n=1), or unknown (n=4). At a median follow up of 8.3 years (range, 0.5-27.1), the 5-year OS was 88.0%. Patients who received oral therapy as first choice had significantly higher hemoglobin and platelet counts at time of relapse compared to those who received full IST (p<0.001), though not absolute neutrophil count. Patients receiving oral therapies had a ORR of 67.4%. At 5 years after therapy, RFS was 74.0%, EFS 38.4% and OS was 91.2%. In those who directly proceeded to a second round of IST, ORR was 67.8% for ATG-based therapy and 48.0% for alemtuzumab. At 5-years post initiation of IST, RFS was 30.8% and 44.4% (**Figure 1**), EFS was 23.7% and 26.0% and OS was 76.5% and 81.5% for patients receiving ATG-based therapies and alemtuzumab, respectively.

In the refractory cohort, 2L-therapies included oral therapies, including CSA, EPAG and danazol (n=12, 8.0%), alemtuzumab (n=43, 27.1%), ATG-based IST (n=52, 34.7%), HSCT (n=37, 24.3%), none (n=1), or unknown (n=5). At a median follow-up of 5.5 years (range, 0.2-24.5), the 5-year OS was 69.0%. ORR were 50.0%, 27.4%, and 28.6%, for oral therapies, ATG-based and alemtuzumab, respectively, and OS was 54.9%, 70.6% and 67.9%.

**Copyright Information:** (Online) ISSN: 2572-9241
©2026 The Author(s). HemaSphere is published by John Wiley & Sons Ltd on behalf of European Hematology Association. This is an open access Abstract Book distributed under the Attribution-NonCommercial-NoDerivs (CC BY-NC-ND), which allows third parties to download the articles and share them with others as long as they credit the author and the Abstract Book, but they cannot change the content in any way or use them commercially.
**Abstract Book Citations:** Authors, Title, HemaSphere, 2026;10:(S1):pages. The abstract book DOIs can be found on the "Author Information" page.
**Disclaimer:** Articles published in the journal HemaSphere exclusively reflect the opinions of the authors. The authors are responsible for all content in their abstracts including accuracy of the facts, statements, citing resources, etc.

**Summary/Conclusion**

This is the largest retrospective cohort to date evaluating long-term outcomes in patients with relapsed or refractory SAA. Despite relatively high initial ORR and long-term OS in patients with relapsed AA, responses were durable in less than half of the patients receiving alemtuzumab or ATG, requiring further AA-directed therapies or HSCT. In the refractory setting, we confirm poor response to further IST.

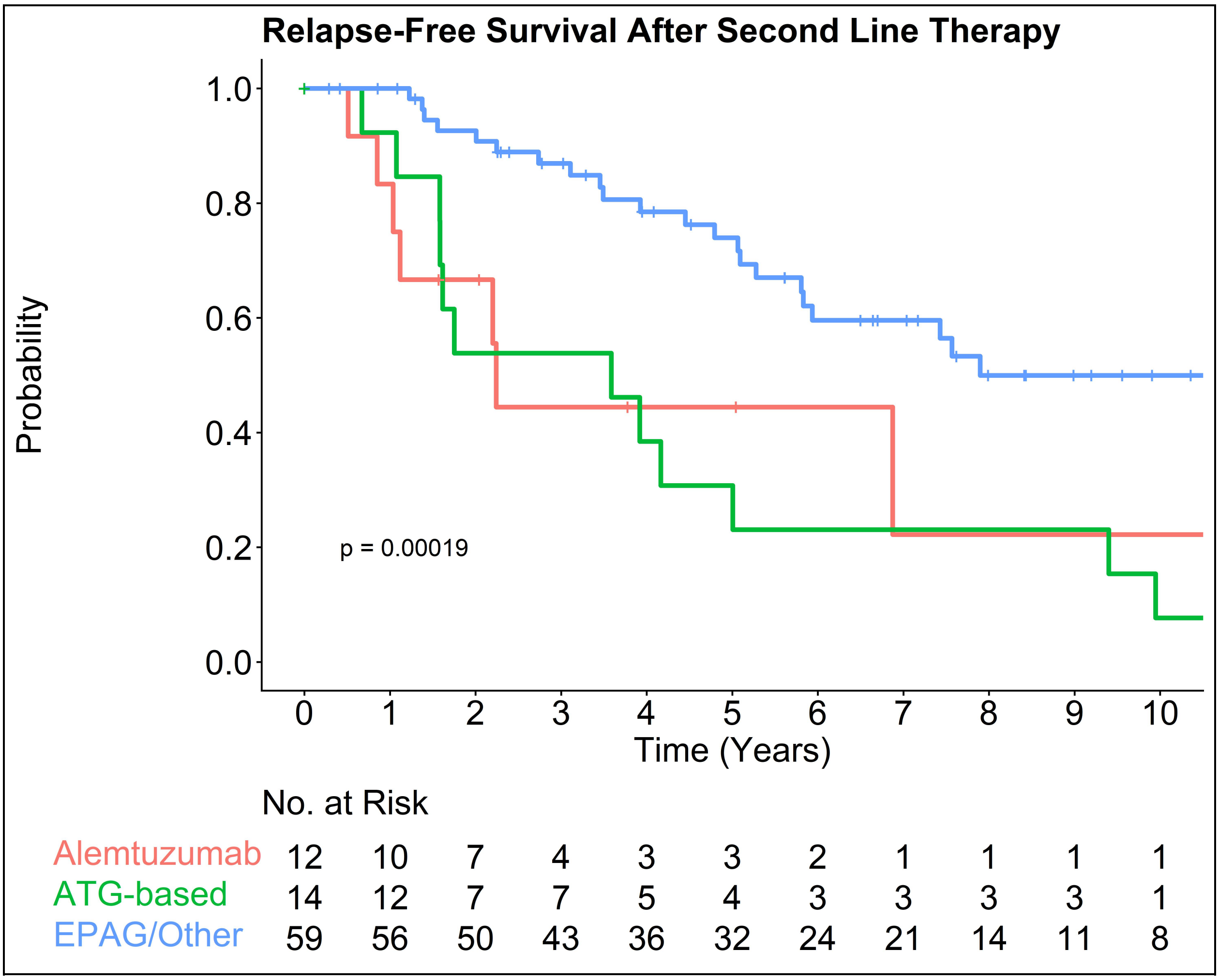

Relapse-free survival from time of second-line IST therapy in patients with relapsed AA after frontline ATG-based therapy.

**Copyright Information:** (Online) ISSN: 2572-9241
©2026 The Author(s). HemaSphere is published by John Wiley & Sons Ltd on behalf of European Hematology Association. This is an open access Abstract Book distributed under the Attribution-NonCommercial-NoDerivs (CC BY-NC-ND), which allows third parties to download the articles and share them with others as long as they credit the author and the Abstract Book, but they cannot change the content in any way or use them commercially.
**Abstract Book Citations:** Authors, Title, HemaSphere, 2026;10:(S1):pages. The abstract book DOIs can be found on the "Author Information" page.
**Disclaimer:** Articles published in the journal HemaSphere exclusively reflect the opinions of the authors. The authors are responsible for all content in their abstracts including accuracy of the facts, statements, citing resources, etc.

### (S179) EARLY BONE FRAGILITY IS ASSOCIATED WITH VASCULAR ABNORMALITIES IN TELOMERE BIOLOGY DISORDERS: FINDINGS FROM THE MULTICENTER, INTERNATIONAL INSIGHT STUDY

**Topic:** 12. Bone marrow failure syndromes incl. PNH - Clinical

Jane Koo*[1], Dawn Comstock[2], Lindsey Hornung[3], Jonathan Howell[4], Ethan Lawhorn[1], Christopher Reilly[5], Alejandro Ferrer[6], Abhishek Mangaonkar[7], Bianca Barredo[8], Jane Churpek[8], Fabian Beier[9], Linda Frazier[10], Pamela Vincent[10], Paul Szabolcs[10], Jessica Uhrich[11], Marcin Wlodarski[11], Nicholas Gloude[12], Helena Yu[12], Divya Koura[13], Tanim Jain[14], Orna Steinberg Shemer[15], Christen Ebens[16], Paul Wratkowski[16], Soundarya Avantsa[17], Emma Groarke[18], Bhavisha Patel[18], Melanie Cotter[19], Eoghan Dunlea[19], Rodrigo Calado[20], Marilia Bazzo Catto[20], Carlo Dufour[21], Francesca Fioredda[21], Erika Massaccesi[21], Katie Stevens[22], Sharon A. Savage[17], Neelam Giri[17], Kasiani C. Myers[1]

*[1]Cincinnati Children's Hospital Medical Center, Division of Bone Marrow Transplantation and Immune Deficiency, Cincinnati, United States of America; [2]Brigham and Women's Hospital, Harvard Medical School, Department of Medicine, Boston, United States of America; [3]Cincinnati Children's Hospital Medical Center, Division of Biostatistics and Epidemiology, Cincinnati, United States of America; [4]Cincinnati Children's Hospital Medical Center, Division of Endocrinology, Cincinnati, United States of America; [5]Dana Farber Cancer Institute, Division of Hematologic Neoplasia, Department of Medical Oncology, Boston, United States of America; [6]Mayo Clinic, Division of Hematology, Department of Medicine, Division of Pulmonology and Critical Care, Rochester, United States of America; [7]Mayo Clinic, Department of Hematology and Medical Oncology, Rochester, United States of America; [8]University of Wisconsin School of Medicine and Public Health & University of Wisconsin Carbone Cancer Center, Division of Hematology, Medical Oncology, and Palliative Care, Madison, United States of America; [9]RWTH Aachen University, Center for Integrated Oncology Aachen Bonn Cologne Düsseldorf (CIO ABCD), Department of Hematology, Oncology, Hemostaseology and Stem Cell Transplantation, Aachen, Germany; [10]UPMC Children's Hospital of Pittsburgh, University of Pittsburgh, Division of Blood and Marrow Transplantation and Cellular Therapies, Department of Pediatrics, Department of Immunology, Pittsburgh, United States of America; [11]St. Jude Children's Research Hospital, Department of Hematology, Memphis, United States of America; [12]Rady Children's Hospital San Diego, University of California San Diego, Division of Pediatric Hematology/Oncology, Department of Pediatrics, San Diego, United States of America; [13]University of California San Diego, Division of Blood and Marrow Transplantation, Department of Medicine, La Jolla, United States of America; [14]University of California San Diego, Division of Hematology/Oncology, Department of Medicine, La Jolla, United States of America; [15]Tel Aviv University, Felsenstein Medical Research Center, Schneider Children's Medical Center of Israel, Gray Faculty of Medical and Health Sciences, Hematology-Oncology Division, Tel Aviv, Israel; [16]University of Minnesota, Division of Blood and Marrow Transplant & Cellular Therapy, Department of Pediatrics, Minneapolis, United States of America; [17]National Cancer Institute, National Institutes of Health, Clinical Genetics Branch, Division of Cancer Epidemiology and Genetics, Bethesda, United States of America; [18]National Heart, Lung and Blood Institute, National Institutes of Health, Hematology Branch, Bethesda, United States of America; [19]Childrens Health Ireland, Temple Street, Dublin, and School of Medicine, University College Dublin, Department of Haematology, Dublin, Ireland; [20]University of São Paulo at Ribeirão Preto Medical School, Ribeirão Preto, Brazil;[21]IRCCS Istituto Giannina Gaslini, Unit of Hematology, Genoa, Italy; [22]Team Telomere Inc, New York, United States of America;*

### Background

Telomere biology disorders (TBDs) are multisystem diseases caused by germline variants in telomere maintenance genes and cause abnormally short telomeres. Skeletal complications, including reduced bone mineral density (BMD), fractures, and avascular necrosis (AVN), are increasingly recognized yet remain poorly characterized. Whether bone disease in TBDs represents a secondary consequence of systemic illness or an intrinsic disease manifestation is unknown. No TBD-specific bone health guidelines exist.

### Aims

To define the burden, timing and clinical correlates of bone disease in patients with TBDs.

**Copyright Information:** (Online) ISSN: 2572-9241
©2026 The Author(s). HemaSphere is published by John Wiley & Sons Ltd on behalf of European Hematology Association. This is an open access Abstract Book distributed under the Attribution-NonCommercial-NoDerivs (CC BY-NC-ND), which allows third parties to download the articles and share them with others as long as they credit the author and the Abstract Book, but they cannot change the content in any way or use them commercially.
**Abstract Book Citations:** Authors, Title, HemaSphere, 2026;10:(S1):pages. The abstract book DOIs can be found on the "Author Information" page.
**Disclaimer:** Articles published in the journal HemaSphere exclusively reflect the opinions of the authors. The authors are responsible for all content in their abstracts including accuracy of the facts, statements, citing resources, etc.

### Methods

We conducted a retrospective, multicenter cohort study of 400 patients with genetically and/or clinically diagnosed TBDs enrolled across 15 centers in the Clinical Care Consortium of Telomere-Associated Ailments. Fracture history, AVN, and BMD assessed by Dual-Energy X-ray Absorptiometry (DXA) were systematically reviewed. The primary endpoint was fracture incidence; secondary endpoints included BMD patterns and clinical correlates. Analyses were stratified by age (≤18 vs >18 years).

### Results

Among 400 TBD patients (63 pediatric, 337 adult), fractures occurred in 22% of pediatric and 34% of adult patients (Fig. 1A). Median age at first fracture was 5.2 years (IQR 4.0–5.8 years) in children and 51.5 years (IQR 32.5–62.8 years) in adults. Fragility fractures accounted for 13% of pediatric and 17% of adult fractures, were often recurrent (Fig. 1B) and preceded TBD diagnosis, with a median interval of –2.0 years (IQR –6.2 to 1.0 years) from diagnosis to first fracture.
AVN occurred in 11% of pediatric patients (median 9.8 years, IQR 9.6–11.2) and 13% of adults (median 27.5 years, IQR 20.8–39.4) and was strongly associated with fracture risk (36% vs 8.7%, p<0.00001). Additional vascular features were more common among patients with fractures, including gastrointestinal bleeding (34% vs 17%, p=0.008) and retinal vasculopathy (18% vs 6%, p=0.01), while pulmonary arteriovenous malformations occurred at similar rates (Fig. 1B). Inheritance pattern and lymphocyte telomere length were not associated with fracture risk; however, *CTC1* variants were enriched among patients with fragility fractures (n=4/67, 6% vs n=4/327, 1.2%, p=0.03).
As DXA screening practices across centers can be heterogenous, we evaluated variability of when first DXA was done. DXA was performed for 201 patients (25 pediatric, 176 adult), but Z- and T-scores were available for 154 patients. Median age at first DXA was 11.1 years (range 4.0–17.7) in children and 57.2 years (range 20.4–79.4) in adults. Pediatric patients had a median lumbar spine Z-score of –1.2, with 36% exhibiting Z-scores < –2 at any site. Adults had a median femoral neck T-score of –1.1, with 23% meeting criteria for osteoporosis. Low Z-scores were associated with vascular complications, AVN, prior bone marrow transplantation, skeletal deformities, autosomal recessive inheritance, and shorter lymphocyte telomere length percentiles, whereas low T-scores were not associated with clinical, vascular, or genetic features (Fig. 1C).

### Summary/Conclusion

Skeletal disease is a common and clinically significant manifestation of TBDs. Associations between low BMD, fractures, and vascular complications suggest that bone fragility in TBDs reflects a systemic disease, with vascular manifestations emerging as a prominent and underappreciated correlate. These findings support early (school age) and lifelong bone health surveillance in patients with TBDs.

**Copyright Information:** (Online) ISSN: 2572-9241
©2026 The Author(s). HemaSphere is published by John Wiley & Sons Ltd on behalf of European Hematology Association. This is an open access Abstract Book distributed under the Attribution-NonCommercial-NoDerivs (CC BY-NC-ND), which allows third parties to download the articles and share them with others as long as they credit the author and the Abstract Book, but they cannot change the content in any way or use them commercially.
**Abstract Book Citations:** Authors, Title, HemaSphere, 2026;10:(S1):pages. The abstract book DOIs can be found on the "Author Information" page.
**Disclaimer:** Articles published in the journal HemaSphere exclusively reflect the opinions of the authors. The authors are responsible for all content in their abstracts including accuracy of the facts, statements, citing resources, etc.

**Figure 1A. Cohort characteristics and TBD features**

| | Pediatric (current age) n=63 | Adult (current age) n=337 |
|---|---|---|
| **Sex** | | |
| Male | 37 (59%) | 199 (59%) |
| Female | 26 (41%) | 138 (41%) |
| **Current age (years)** | 12.2 ± 4.3 13.0 (9.0-15.7) [2.2-18.0] | 51.0 ± 16.4 54.0 (37.0-64.0) [18.8-81.4] |
| **Diagnosis age (years) [telomere testing]** | n=49 8.2 (5.1-11.0) [0.3-18.2] | n=162 50.5 (28.8-62.6) [2.0-79.0] |
| **Race** | | |
| White/Caucasian | 43/62 (69%) | 295/326 (90%) |
| Black/African American | 7/62 (11%) | 10/326 (3%) |
| Other | 12/62 (19%) | 21/326 (6%) |
| **Pathogenic variant** | | |
| ACD | 0 (0%) | 1 (0.3%) |
| CTC1 | 2 (3.2%) | 6 (1.8%) |
| DKC1 | 13 (21%) | 6 (1.8%) |
| NAF1 | 1 (1.6%) | 3 (0.9%) |
| NHP2 | 0 (0%) | 0 (0%) |
| NOP10 | 0 (0%) | 0 (0%) |
| PARN | 1 (1.6%) | 15 (4.5%) |
| POT1 | 0 (0%) | 0 (0%) |
| RPA1 | 0 (0%) | 0 (0%) |
| RTEL1 | 10 (16%) | 57 (17%) |
| STN1 | 0 (0%) | 0 (0%) |
| TERC | 3 (4.8%) | 45 (13%) |
| TERT | 16 (25%) | 99 (29%) |
| TINF2 | 9 (14%) | 5 (1.5%) |
| WRAP53 | 1 (1.6%) | 3 (0.9%) |
| ZCCHC8 | 0 (0%) | 0 (0%) |
| Other variant | 3 (4.8%) | 9 (2.7%) |
| None identified | 8 (13%) | 96 (28%) |
| **Inheritance pattern** | | |
| Autosomal recessive | 11/59 (19%) | 12/325 (4%) |
| Autosomal dominant | 32/59 (54%) | 210/325 (65%) |
| X-linked | 7/59 (12%) | 4/325 (1%) |
| None identified | 10/59 (17%) | 100/325 (31%) |
| **Telomere length testing performed** | 57/59 (97%) | 303/323 (94%) |
| **Lymphocyte telomere length (Kb, kilobases)** | n=47 5.0 ± 1.7 4.7 (3.7-6.3) [2.0-9.0] | n=231 4.2 ± 1.0 4.0 (3.6-4.6) [2.3-8.9] |
| **Percentile of Lymphocyte Telomere length** | | |
| < 1st percentile | 45/50 (90%) | 165/297 (56%) |
| 1-10th percentile | 3/50 (6%) | 127/297 (43%) |
| 10th-50th percentile | 2/50 (4%) | 5/297 (2%) |
| **History of bone marrow transplant** | 29 (46%) | 48/335 (14%) |
| Age first BMT (years) | 8.7 (5.3-10.7) n=29 | 34.0 (20.0-50.8) n=46 |
| **TBD-related disease manifestations** | | |
| **History of retinal vasculopathy or hemorrhage** | 11/59 (19%) | 14/276 (5%) |
| **History of gastrointestinal (GI) bleeding** | 12/59 (20%) | 54/278 (19%) |
| **History of pulmonary arteriovenous malformations (AVMs)** | 4/58 (7%) | 11/277 (4.0%) |
| **Skeletal manifestations** | | |
| **History of any fracture** | 14 (22%) | 115/337 (34%) |
| Age first fracture (years) | 5.2 (4.0-5.8) n=9 | 51.5 (32.5-62.8) n=84 |
| **History of fracture type** | | |
| Any fragility | 8/63 (13%) | 56/330 (17%) |
| Any traumatic | 8/63 (13%) | 59/330 (18%) |
| Both | 2/63 (3.2%) | 7/330 (2.1%) |
| **History of bone pain (not related to injury)** | 4/52 (7.7%) | 56/237 (17%) |
| Age first bone pain (years) | 10.8 (9.0-14.4) n=3 | 42.0 (24.0-53.6) n=50 |
| **History of avascular necrosis (AVN)** | 7/62 (11%) | 42/328 (13%) |
| Age first AVN (years) | 9.8 (9.6-11.2) n=5 | 27.5 (20.8-39.4) n=38 |

**Figure 1B. Clinical and vascular correlates of fragility fractures in Telomere biology disorders**

| | History of Fragility Fracture n=68 | No Known History of Fragility Fracture n=330 | P-value |
|---|---|---|---|
| **Diagnosis age (years) [telomere testing]** | n=24 56.4 (27.5-64.8) | n=182 35.7 (14.1-58.0) | 0.051 |
| **Age 1st known fragility fracture (years)** | n=50 54.5 (35.0-63.6) | NA | - |
| **Time from diagnosis [telomere testing] to 1st known fragility fracture (years)** | n=19 -2.0 (-6.2, 1.0) | | |
| **Sex** | | | 0.62 |
| Male | 42 (62%) | 193 (58%) | |
| Female | 26 (38%) | 137 (42%) | |
| **History of bone marrow transplant** | 16/67 (24%) | 60/329 (18%) | 0.29 |
| **History of avascular necrosis (AVN)** | 24/67 (36%) | 28/323 (8.7%) | **<0.0001** |
| **History of bone pain (not related to injury)** | 9/35 (26%) | 50/247 (20%) | 0.46 |
| **History of skeletal deformities** | 8/44 (18%) | 32/269 (12%) | 0.25 |
| **History of retinal vasculopathy or hemorrhage** | 8/44 (18%) | 17/284 (6%) | **0.01** |
| **History of gastrointestinal bleeding** | 15/44 (34%) | 49/286 (17%) | **0.008** |
| **History of pulmonary arteriovenous malformations (AVMs)** | 4/43 (9.3%) | 10/286 (3.5%) | 0.10 |
| **Any history of retinal vasculopathy/hemorrhage, GI bleeding, or AVMs** | 20/44 (45%) | 65/282 (23%) | **0.002** |
| **Any history of low bone mineral density (Z<-2 or T<-2)** | 20/41 (49%) | 30/147 (20%) | **0.0003** |
| **Percentile of Lymphocyte Telomere length** | | | 0.52 |
| < 1st percentile | 26/46 (57%) | 182/295 (62%) | |
| 1-10th percentile | 20/46 (43%) | 106/295 (36%) | |
| 10th-50th percentile | 0/46 (0%) | 7/295 (2%) | |
| **Inheritance pattern** | | | 0.90 |
| Autosomal recessive | 2/51 (4%) | 19/322 (6%) | |
| Autosomal dominant | 31/51 (61%) | 202/322 (63%) | |
| X-linked | 1/51 (2%) | 10/322 (3%) | |
| None identified | 17/51 (33%) | 91/322 (28%) | |
| **CTC1** | 4/67 (6%) | 4/327 (1.2%) | **0.03** |
| **TINF2** | 4/67 (6%) | 11/327 (3.4%) | 0.30 |

**Figure 1C. Low BMD Z-scores, but not T-scores, are associated with vascular and systemic complications in telomere biology disorders**

| | First DXA n=202 | Pediatric DXA n=25 | Adult DXA n=176 |
|---|---|---|---|
| **DXA age (years)** | n=201 54.8 (31.0-63.9) [4.0-79.4] | 11.1 (9.1-13.2) [4.0-17.7] | 57.2 (43.5-64.9) [20.4-79.4] |
| **DXA Z-scores** | | | |
| Left neck femur | -0.1 (-0.8, 0.6) n=116 | NA | -0.1 (-0.8, 0.6) n=116 |
| Right neck femur | -0.2 (-0.9, 0.4) n=63 | NA | -0.2 (-0.9, 0.4) n=63 |
| Lumbar spine | -0.3 (-1.3, 0.7) n=144 | -1.2 (-2.6, -0.2) n=22 | -0.2 (-1.0, 0.9) n=122 |
| Whole body | -1.1 (-2.8, -0.5) n=10 | -1.0 (-1.3, -0.4) n=7 | -2.8 (-3.3, -0.5) n=3 |
| **Low Z-score (< -2 at any site)** | 17/154 (11%) | 8/22 (36%) | 9/132 (6.8%) |
| **DXA T-scores** | | | |
| Left neck femur | -1.1 (-1.7, -0.4) n=128 | NA | -1.1 (-1.7, -0.4) n=128 |
| Right neck femur | -1.0 (-1.7, -0.3) n=72 | NA | -1.0 (-1.7, -0.3) n=72 |
| Lumbar spine | -0.5 (-1.3, 0.4) n=142 | -2.2 n=1 | -0.5 (-1.3, 0.4) n=141 |
| Whole body | -1.7 (-2.1, -0.4) n=6 | -1.3 (-2.4, -0.1) n=4 | -1.7 (-1.7, -1.6) n=2 |
| Left forearm | 0.8 (-1.2, 1.4) n=8 | NA | 0.8 (-1.2, 1.4) n=8 |
| Right forearm | 0.8 (0.2, 1.5) n=3 | NA | 0.8 (0.2, 1.5) n=3 |
| **Low T-score (< -2 at any site)** | 34/158 (22%) | 3/5 (60%) | 31/153 (20%) |
| **First DXA** | **Low Z-score (< -2 at any site) n=17** | **No Low Z-score (< -2 at any site) n=137** | **P-value** |
| **History of retinal vasculopathy or hemorrhage** | 6/15 (40%) | 7/103 (7%) | **0.002** |
| **History of gastrointestinal bleeding** | 7/15 (47%) | 29/103 (28%) | 0.23 |
| **History of pulmonary arteriovenous malformations (AVMs)** | 4/15 (27%) | 6/103 (6%) | **0.02** |
| **Any history of retinal vasculopathy/hemorrhage, GI bleeding, or AVMs** | 10/15 (67%) | 36/102 (35%) | **0.03** |
| **History of fracture** | 8 (47%) | 51 (37%) | 0.43 |
| **Inheritance pattern** | | | **0.002** |
| Autosomal recessive | 4/16 (25%) | 4/134 (3%) | |
| Autosomal dominant | 7/16 (44%) | 78/134 (58%) | |
| X-linked | 2/16 (12.5%) | 3/134 (2%) | |
| None identified | 3/16 (19%) | 49/134 (37%) | |
| **Percentile of Lymphocyte Telomere length** | | | **0.045** |
| < 1st percentile | 12/14 (86%) | 67/126 (53%) | |
| 1-10th percentile | 2/14 (14%) | 58/126 (46%) | |
| 10th-50th percentile | 0/14 (0%) | 1/126 (1%) | |
| **History of bone marrow transplant** | 10 (59%) | 21 (15%) | **0.0002** |
| **History of avascular necrosis (AVN)** | 5 (29%) | 10/133 (7.5%) | **0.015** |
| **History of bone pain (not related to injury)** | 4/13 (31%) | 15/80 (19%) | 0.46 |
| **History of skeletal deformities** | 7/15 (47%) | 15/95 (16%) | **0.01** |
| **First DXA** | **Low T-score (< -2 at any site) n=34** | **No Low T-score (< -2 at any site) n=124** | **P-value** |
| **History of retinal vasculopathy or hemorrhage** | 0/24 (0%) | 8/93 (8.6%) | 0.20 |
| **History of gastrointestinal bleeding** | 5/25 (20%) | 24/93 (26%) | 0.61 |
| **History of pulmonary arteriovenous malformations (AVMs)** | 2/24 (8.3%) | 4/95 (4.2%) | 0.60 |
| **Any history of retinal vasculopathy/hemorrhage, GI bleeding, or AVMs** | 7/24 (29%) | 31/92 (34%) | 0.67 |
| **History of fracture** | 17 (50%) | 47 (38%) | 0.20 |
| **Inheritance pattern** | | | 0.93 |
| Autosomal recessive | 0 (0%) | 2/122 (1.6%) | |
| Autosomal dominant | 19 (56%) | 70/122 (57%) | |
| X-linked | 0 (0%) | 1/122 (0.8%) | |
| None identified | 15 (44%) | 49/122 (40%) | |
| **Percentile of Lymphocyte Telomere length** | | | 1.00 |
| < 1st percentile | 16/31 (52%) | 60/115 (52%) | |
| 1-10th percentile | 15/31 (48%) | 54/115 (47%) | |
| 10th-50th percentile | 0/31 (0%) | 1/115 (1%) | |
| **History of bone marrow transplant** | 1 (3%) | 15 (12%) | 0.20 |
| **History of avascular necrosis (AVN)** | 1 (3%) | 8/120 (7%) | 0.68 |
| **History of bone pain (not related to injury)** | 3/22 (14%) | 13/67 (19%) | 0.75 |
| **History of skeletal deformities** | 7/28 (25%) | 10/78 (13%) | 0.14 |

Data presented as n (%) or median (25th-75th percentile) [min-max]. Complete data unless otherwise specified by "n=" or stated denominator. Differences between groups tested using Wilcoxon-Mann-Whitney tests for continuous variables or Chi-square and Fisher's exact tests were used.

Figure

**Copyright Information:** (Online) ISSN: 2572-9241
©2026 The Author(s). HemaSphere is published by John Wiley & Sons Ltd on behalf of European Hematology Association. This is an open access Abstract Book distributed under the Attribution-NonCommercial-NoDerivs (CC BY-NC-ND), which allows third parties to download the articles and share them with others as long as they credit the author and the Abstract Book, but they cannot change the content in any way or use them commercially.
**Abstract Book Citations:** Authors, Title, HemaSphere, 2026;10:(S1):pages. The abstract book DOIs can be found on the "Author Information" page.
**Disclaimer:** Articles published in the journal HemaSphere exclusively reflect the opinions of the authors. The authors are responsible for all content in their abstracts including accuracy of the facts, statements, citing resources, etc.

### (S180) GENOMIC LANDSCAPE AND PROGNOSTIC IMPACT OF SOMATIC MUTATIONS IN APLASTIC ANEMIA PATIENTS UNDERGOING HEMATOPOIETIC STEM CELL TRANSPLANTATION

**Topic:** 12. Bone marrow failure syndromes incl. PNH - Clinical

Lijuan Hu[1], Xiao-Di MA*[1], Zheng-Li Xu[1], Haixia Fu[1], Yun He[1], Yifei Cheng[1], Tingting Han[1], Yuan-Yuan Zhang[1], Jing-Zhi Wang[1], Xiao-Dong Mo[1], Yu Wang[1], Xiaohui Zhang[1], Lanping Xu[1], Xiaojun Huang[1]

*[1]peking university people's hospital, Beijing, China;*

### Background

Aplastic anemia (AA) is traditionally viewed as an immune-mediated bone marrow failure syndrome. However, the emergence of clonal hematopoiesis through somatic mutations is increasingly recognized as a critical factor in disease progression and clonal evolution toward myelodysplastic syndrome (MDS) or acute myeloid leukemia (AML). While hematopoietic stem cell transplantation (HSCT) remains a curative intent, the impact of the complex pre-transplant mutational landscape on post-transplant survival remains poorly defined, necessitating a robust genomic risk stratification model.

### Aims

To prospectively characterize the somatic mutational architecture in AA and validate a genomic risk signature for predicting post-HSCT outcomes.

### Methods

We prospectively enrolled 414 AA patients between 2018 and 2025. All patients underwent baseline high-resolution targeted Next-Generation Sequencing (NGS). Mutational frequencies and co-occurrence patterns were mapped. A sub-cohort of 53 patients proceeding to HSCT was monitored for survival outcomes, including Overall Survival (OS). Univariate and multivariable Cox models identified independent genomic predictors of mortality.

### Results

Among the total cohort, 85.5% patients (354/414) harboring at least one somatic mutation, including Tier I, II and III. A total of 985 mutations across 136 genes were identified. The most frequently mutated genes were KMT2D (4.67%), PIGA (4.26%), DNMT3A (3.76%), ASXL1 (3.65%), and BCOR (3.45%), with the majority being Tier III variants. Complex clonal structures were evident through significant gene co-occurrences, notably SETBP1-RUNX1 and KMT2A-NOTCH2 clusters.
In the HSCT sub-cohort, we identified a high-risk genomic signature that significantly impaired survival. Univariate analysis revealed that mutations in NPM1 (HR=51.50, p=0.0053), RARA (HR=25.50, p=0.0082), EZH2 (HR=12.44, p=0.0242), NTRK1 (HR=12.44, p=0.0242), SETBP1 (HR=12.44, p=0.0242) and RUNX1 (HR=5.85, p=0.0308) were powerful predictors of poor OS. Patients with these genes mutated were categorized into the "High-Risk Mutation" group (n=10). The "High-Risk Mutation" group exhibited a 40% OS (95%CI: 12.16-73.76%), compared to 95.34% (95%CI: 84.19-99.43%) in the non-high-risk group ($p < 0.0001$). Notably, the survival curve for high-risk patients showed an early precipitous drop within the first 500 days post-transplant. Multivariable Cox modeling confirmed the genomic signature as a superior independent prognostic factor, outperforming traditional clinical parameters such as age and disease severity.

**Copyright Information:** (Online) ISSN: 2572-9241
©2026 The Author(s). HemaSphere is published by John Wiley & Sons Ltd on behalf of European Hematology Association. This is an open access Abstract Book distributed under the Attribution-NonCommercial-NoDerivs (CC BY-NC-ND), which allows third parties to download the articles and share them with others as long as they credit the author and the Abstract Book, but they cannot change the content in any way or use them commercially.
**Abstract Book Citations:** Authors, Title, HemaSphere, 2026;10:(S1):pages. The abstract book DOIs can be found on the "Author Information" page.
**Disclaimer:** Articles published in the journal HemaSphere exclusively reflect the opinions of the authors. The authors are responsible for all content in their abstracts including accuracy of the facts, statements, citing resources, etc.

### Summary/Conclusion

Our study provides a comprehensive map of the genetic architecture in AA, demonstrating that somatic mutations are not merely bystanders but key determinants of clinical outcomes. The identification of a high-risk mutational signature allows for precise pre-transplant risk-stratification. These findings support the integration of high-resolution NGS into routine clinical evaluation to guide personalized therapeutic intensity and post-HSCT monitoring in patients with AA.

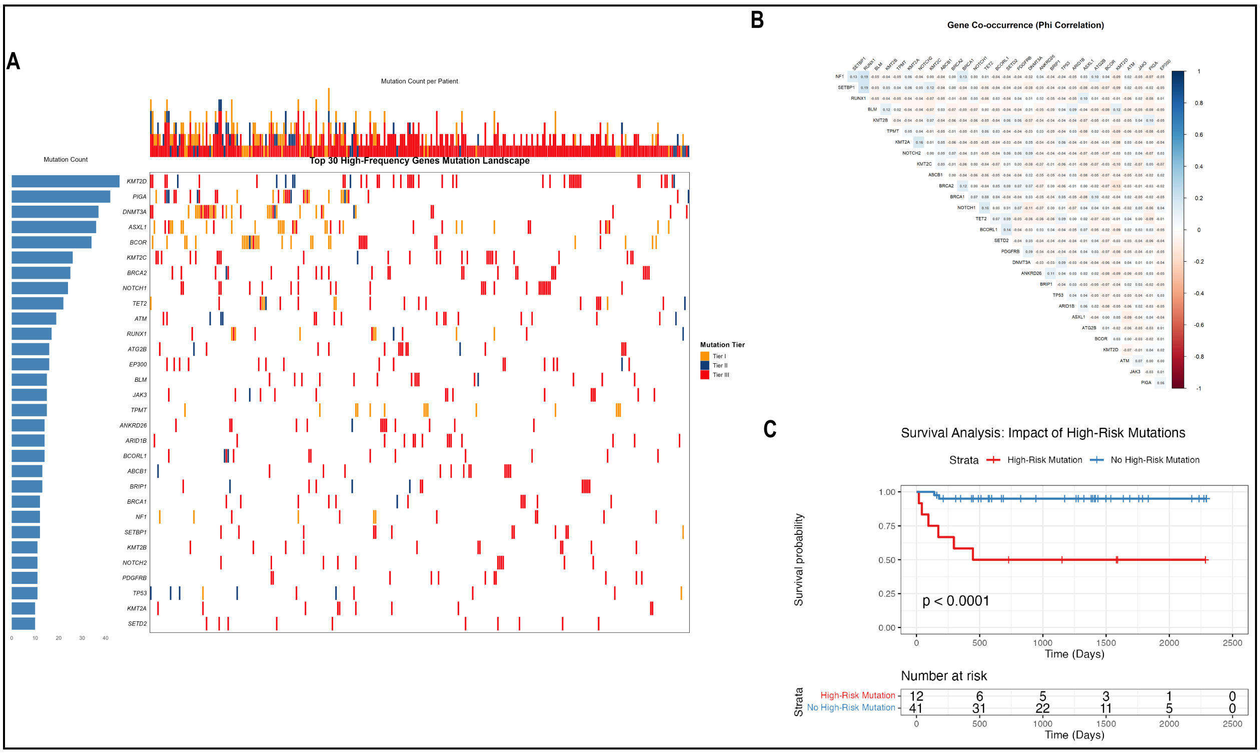

(A) Somatic mutation landscape of the top 30 high-frequency genes in the cohort. (B) Gene co-occurrence and mutual exclusivity analysis. (C) Kaplan-Meier survival analysis of the HSCT sub-cohort.

**Copyright Information:** (Online) ISSN: 2572-9241
©2026 The Author(s). HemaSphere is published by John Wiley & Sons Ltd on behalf of European Hematology Association. This is an open access Abstract Book distributed under the Attribution-NonCommercial-NoDerivs (CC BY-NC-ND), which allows third parties to download the articles and share them with others as long as they credit the author and the Abstract Book, but they cannot change the content in any way or use them commercially.
**Abstract Book Citations:** Authors, Title, HemaSphere, 2026;10:(S1):pages. The abstract book DOIs can be found on the “Author Information” page.
**Disclaimer:** Articles published in the journal HemaSphere exclusively reflect the opinions of the authors. The authors are responsible for all content in their abstracts including accuracy of the facts, statements, citing resources, etc.

### (S181) A PHASE III STUDY OF A HEAD-TO-HEAD COMPARISON OF A FACTOR B INHIBITOR HSK39297, VERSUS ECULIZUMAB IN PATIENTS WITH PAROXYSMAL NOCTURNAL HEMOGLOBINURIA NAIVE TO COMPLEMENT INHIBITION

**Topic:** 12. Bone marrow failure syndromes incl. PNH - Clinical

Yuemin Gong[1], Shunqing Wang[2], Lina Zhang[3], Yingmei Li[4], Taiwu Xiao[5], Zunmin Zhu[6], Jianda Hu[7], Juan Yang[8], Fangqiong Li[8], Hongyan Tong[9], Yawen Zhang*[10], Guangsheng He[1]

*[1]The First Affiliated Hospital with Nanjing Medical University, Nanjing, China;[2]Guangzhou First People's Hospital, Guangzhou, China; [3]Henan Cancer Hospital, Zhengzhou, China;[4]The First Affiliated Hospital of Zhengzhou University, Zhengzhou, China; [5]Liaocheng People's hospital, Liaocheng, China;[6]Henan Provincial People's Hospital, Zhengzhou, China;[7]The Second Affiliated Hospital of Fujian Medical University, Quanzhou, China; [8]Haisco Pharmaceutical Group Co, Ltd., Chengdu, China;[9]The First Affiliated Hospital, Zhejiang University School of Medicine, Hangzhou, China; [10]The First Affiliated Hospital of Nanjing Medical University, Jiangsu Province Hospital, Nanjing, Jiangsu, China, Jiangsu Province Engineering Research Center of Cell and Gene Therapy for hematologic and lymphoid Diseases, Nanjing, China;*

### Background

Paroxysmal nocturnal hemoglobinuria (PNH) is a rare hematological disorder characterized by intravascular hemolysis, bone marrow failure, and an elevated risk of life-threatening thrombosis. HSK39297, an oral complement factor B inhibitor, has been developed by Haisco for the treatment of PNH.

### Aims we present the first findings from a head-to-head comparison of a factor B inhibitor and eculizumab monotherapy in patients with PNH who were naïve to complement inhibitor therapy.

### Methods

NCT06799546 is a multicenter, randomized, open-label, active-controlled Phase III study in patients with PNH who have not previously received complement inhibitor therapy. Eligible patients with hemoglobin <10g/dL were stratified and randomized (1:1) to receive either HSK39297 200mg once daily or eculizumab for 24 weeks. Stratification was based on baseline hemoglobin level (≤7g/dL or >7g/dL) as determined by a central laboratory and history of red blood cell (RBC) transfusion within 6 months prior to randomization. The primary endpoint was the proportion of patients achieving hemoglobin ≥12 g/dL at ≥3 of 4 assessments between Week 18 and Week 24, without RBC transfusions from 2 weeks after treatment initiation. Secondary endpoints included the proportion of patients achieving a ≥2g/dL increase in hemoglobin from baseline, the proportion of patients without RBC transfusion, and changes from baseline in hemoglobin, reticulocyte count, LDH, and FACIT-Fatigue score.

**Copyright Information:** (Online) ISSN: 2572-9241
©2026 The Author(s). HemaSphere is published by John Wiley & Sons Ltd on behalf of European Hematology Association. This is an open access Abstract Book distributed under the Attribution-NonCommercial-NoDerivs (CC BY-NC-ND), which allows third parties to download the articles and share them with others as long as they credit the author and the Abstract Book, but they cannot change the content in any way or use them commercially.
**Abstract Book Citations:** Authors, Title, HemaSphere, 2026;10:(S1):pages. The abstract book DOIs can be found on the "Author Information" page.
**Disclaimer:** Articles published in the journal HemaSphere exclusively reflect the opinions of the authors. The authors are responsible for all content in their abstracts including accuracy of the facts, statements, citing resources, etc.

**Results**

A total of 73 patients with PNH were enrolled, with 37 in the HSK39297 group and 36 in the eculizumab group. The primary endpoint response rates was 59.5% (95% CI: 43.2%, 75.7%) in the HSK39297 group and 8.3% (95% CI: 2.8%, 19.4%) in the eculizumab group. The odds ratio for HSK39297 versus eculizumab was 20.3 (95% CI: 4.7, 88.0), $p < 0.0001$. The proportion of patients achieving a ≥2 g/dL increase in hemoglobin from baseline was 91.9% in the HSK39297 group and 55.6% in the eculizumab group, corresponding rates for patients not requiring RBC transfusions were 94.6% and 69.4%, respectively. A marked reduction in LDH and improvement in hemoglobin were observed in the HSK39297 group starting from Week 1 of treatment. During the Week 18 to Week 24 treatment period, the least squares mean percent change from baseline in LDH was -84.0% in the HSK39297 group and -80.4% in the eculizumab group; the least squares mean change from baseline in hemoglobin was 5.1 g/dL and 2.1 g/dL, respectively. HSK39297 demonstrated superiority over eculizumab in terms of the magnitude of hemoglobin improvement, reduction in reticulocyte count, and improvement in FACIT-Fatigue score , without the risk of extravascular hemolysis associated with C3 deposition. The incidence of TEAEs was lower with HSK39297 (75.7%) than with eculizumab (86.1%). Most TEAEs reported with HSK39297 were CTCAE Grade 1-2 in severity, with no TEAEs leading to dose reduction, treatment discontinuation, or withdrawal from the study. Drug-related TEAEs occurring in >5% of patients in the HSK39297 group included headache (18.9%), lipid disorders (10.8%), and bacterial infections (5.4%).

**Summary/Conclusion**

HSK39297 (200 mg once daily) demonstrated significantly superior efficacy compared with eculizumab in the treatment of patients with PNH, along with a favorable safety profile.

**Copyright Information:** (Online) ISSN: 2572-9241
©2026 The Author(s). HemaSphere is published by John Wiley & Sons Ltd on behalf of European Hematology Association. This is an open access Abstract Book distributed under the Attribution-NonCommercial-NoDerivs (CC BY-NC-ND), which allows third parties to download the articles and share them with others as long as they credit the author and the Abstract Book, but they cannot change the content in any way or use them commercially.
**Abstract Book Citations:** Authors, Title, HemaSphere, 2026;10:(S1):pages. The abstract book DOIs can be found on the "Author Information" page.
**Disclaimer:** Articles published in the journal HemaSphere exclusively reflect the opinions of the authors. The authors are responsible for all content in their abstracts including accuracy of the facts, statements, citing resources, etc.

### (S182) ELTROMBOPAG ADDED TO COMBINED IMMUNOSUPPRESSIVE THERAPY (IST) IN CHILDREN WITH ACQUIRED SEVERE APLASTIC ANEMIA ACCELERATES HEMATOLOGIC RECOVERY AND SHOULD BE CONTINUED IN THE SECOND COURSE OF IST

**Topic:** 12. Bone marrow failure syndromes incl. PNH - Clinical

Olga Goronkova[1], Galina Novichkova[1], Tatiana Salimova[1], Dina Baidildina[1], Olga Mironenko[1], Michail Maschan[1], Alexey Maschan*[1]

[1]*National Medical Research Center of Pediatric Hematology, Oncology and Immunology named after Dmitry Rogachev, Moscow, Russia;*

### Background

Eltrombopag (ELTR) added to first-line standard immunosuppressive therapy (IST) has demonstrated efficacy in adult patients with severe aplastic anemia (SAA), but the data in children remains controversial.

### Aims

We extended our randomized prospective multicenter trial comparing efficacy and safety of ELTR combined with IST (horse antithymocyte globulin (hATG) and cyclosporine) vs IST alone in treatment-naïve children with SAA lacking HLA-identical sibling donor, to evaluate early response and long-term outcomes in a larger patient cohort with a longer follow-up.

### Methods

Patients were randomized into ELTR+IST (initial ELTR(+)) and IST (initial ELTR(-)) groups in 1:1 ratio. In ELTR+IST group, patients received ELTR from day 1 for at least 4 months and then stopped in nonresponders and tapered during 8 weeks in patients with a response. In Part I of our trial, nonresponders at 4 months received the second course of IST with ELTR, if initial course was standard IST, or without ELTR, if initial course was ELTR+IST (crossover treatment). In Part II, all nonresponders received the second course of IST with ELTR. The endpoints for the pooled analysis of Parts I and II were 3-month overall response rate (ORR), non-response rate, second-line treatment efficacy, overall (OS) and event-free (EFS) survival, cumulative incidence (CI) of relapse and CI of clonal evolution.

### Results

Overall, 200 pediatric patients (98 in Part I; 102 in Part II) aged 2.0-17.7 years from 12 centers were included, with 99 assigned to ELTR+IST and 101 to IST. The ORR at 3 months was significantly higher in ELTR+IST group than in IST group: 56,6% (56/99) vs. 41,6% (42/101), p=0.034; the median time to overall response was 79 vs. 106 days, p=0.026. Complete response rate at 3 months was significantly higher in ELTR+IST group: 16.2% (16/99) vs. 3.9% (4/101), p=0.004. In Part I, after the crossover, 63% of initial ELTR(-) nonresponders achieved a response compared to 17% of initial ELTR(+) nonresponders (p=0.011). In Part II, the proportion of patients refractory to initial therapy was higher in the IST group than in the ELTR+IST group: 40.4% (21/52) vs. 22.0% (11/50), p=0.045. Median time to the second course of hATG was 146.5 (104-189) days. At 6 months, 54% (15/28) of initial nonresponders achieved a response: 6/8 (75%) in initial ELTR(+) and 9/20 (45%) in the initial ELTR(-) group. In total, 80.8% (80/99) and 77.2% (78/101) patients in initial ELTR+IST and IST groups, respectively, responded to one or two treatment courses (p=0.534).
With median follow-up of 4.5 (0.1-9.0) years, no significant differences in ELTR+IST and IST groups were observed in terms of 5-year OS (94% vs. 93%, p=0.827), 5-year EFS (52% vs. 47%, p =0.150), CI of relapse (24% vs 18%, p=0.456) and Cl of clonal bone marrow chromosomal abnormalities (7.5% vs 8.4%, p=0.890). No cases of myeloid malignancy developed.

**Copyright Information:** (Online) ISSN: 2572-9241
©2026 The Author(s). HemaSphere is published by John Wiley & Sons Ltd on behalf of European Hematology Association. This is an open access Abstract Book distributed under the Attribution-NonCommercial-NoDerivs (CC BY-NC-ND), which allows third parties to download the articles and share them with others as long as they credit the author and the Abstract Book, but they cannot change the content in any way or use them commercially.
**Abstract Book Citations:** Authors, Title, HemaSphere, 2026;10:(S1):pages. The abstract book DOIs can be found on the "Author Information" page.
**Disclaimer:** Articles published in the journal HemaSphere exclusively reflect the opinions of the authors. The authors are responsible for all content in their abstracts including accuracy of the facts, statements, citing resources, etc.

**Summary/Conclusion**

ELTR added to IST increased hematological response rate at 3 months in treatment-naïve pediatric patients, allowing for early identification of nonresponders and timely initiation of second-line treatment. The second course of IST without ELTR had limited efficacy among patients who received ELTR up-front. Adding ELTR to the second course of IST was equally effective in both initial ELTR(-) and ELTR(+) patients, indicating that ELTR should be continued in recipients of the second IST course. Clinicaltrials.gov #NCT03413306.

**Copyright Information:** (Online) ISSN: 2572-9241
©2026 The Author(s). HemaSphere is published by John Wiley & Sons Ltd on behalf of European Hematology Association. This is an open access Abstract Book distributed under the Attribution-NonCommercial-NoDerivs (CC BY-NC-ND), which allows third parties to download the articles and share them with others as long as they credit the author and the Abstract Book, but they cannot change the content in any way or use them commercially.
**Abstract Book Citations:** Authors, Title, HemaSphere, 2026;10:(S1):pages. The abstract book DOIs can be found on the "Author Information" page.
**Disclaimer:** Articles published in the journal HemaSphere exclusively reflect the opinions of the authors. The authors are responsible for all content in their abstracts including accuracy of the facts, statements, citing resources, etc.

### (S183) CLINICAL AND MOLECULAR FINDINGS IN 46 PATIENTS WITH DNAJC21-RELATED BONE MARROW FAILURE SYNDROME

**Topic:** 12. Bone marrow failure syndromes incl. PNH - Clinical

María Muñoz Martínez*[1, 2], Lili Kotmayer[1], Maria Solsona Gadea[3], Eva Galvez Villa[4], Guzmán López de Hontanar[5], Julián Sevilla[5], Alyssa Kennedy[1], Sara Lewis[1], Miguel R. Abboud[6], Dolly Noun[6], Akiko Shimamura[7], Annabel Cincotta[7], Sophia Polychronopoulou[8], Charikleia Kelaidi[8], Xia Qin[9], Francesco Saettini[10, 11], Albert Català[2, 3], Marcin Wlodarski[1]

*[1]St. Jude Children's Research Hospital, Bone Marrow Failure & MDS Department, Memphis, United States of America;[2]Sant Joan de Déu Children's Hospital, Oncology Department, Barcelona, Spain; [3]Sant Joan de Déu Children's Hospital, Hematology Department, Barcelona, Spain; [4]Jerez University Hospital, Hematology Department, Jerez, Spain; [5]Niño Jesús Children's Hospital, Hematology and Oncology Department, Madrid, Spain; [6]American University of Beirut, Department of Pediatrics and Adolescent Medicine, Beirut, Lebanon; [7]Dana Farber/Boston Children's Cancer and Blood Disorders Center, Harvard Medical School, Boston, United States of America; [8]Aghia Sophia Children's Hospital, Hematology Department, Athens, Greece; [9]Shanghai Children's Medical Center, Department of Hematology & Bone Marrow Transplant, Shangai, China;[10]Fondazione IRCCS San Gerardo dei Tintori, Pediatria, Monza, Italy; [11]Fondazione IRCCS San Gerardo dei Tintori, Centro Tettamanti, Monza, Italy;*

### Background

Shwachman-Diamond syndrome (SDS) is an inherited bone marrow failure disorder caused predominantly by biallelic *SBDS* variants. In recent years, *DNAJC21* emerged as a new gene associated with SDS-like phenotypes, yet the full clinical and molecular spectrum of DNAJC21-related disease remains incompletely defined.

### Aims

To characterize the largest international cohort of patients with biallelic *DNAJC21* variants to date, including clinical phenotype, molecular findings, and disease outcomes.

### Methods

We assembled 46 patients with biallelic *DNAJC21* variants from 34 unrelated families (29 identified via systematic literature search, and 17 novel cases from our consortium). Clinical and genetic data were captured in standardized format. Variant frequencies were assessed using gnomAD v4.1 population database with hg37 annotation (NM_001012339.2). Germline status was confirmed where feasible. All data were curated in an open-access online database with dynamic visualization at variant level.

**Copyright Information:** (Online) ISSN: 2572-9241
©2026 The Author(s). HemaSphere is published by John Wiley & Sons Ltd on behalf of European Hematology Association. This is an open access Abstract Book distributed under the Attribution-NonCommercial-NoDerivs (CC BY-NC-ND), which allows third parties to download the articles and share them with others as long as they credit the author and the Abstract Book, but they cannot change the content in any way or use them commercially.
**Abstract Book Citations:** Authors, Title, HemaSphere, 2026;10:(S1):pages. The abstract book DOIs can be found on the "Author Information" page.
**Disclaimer:** Articles published in the journal HemaSphere exclusively reflect the opinions of the authors. The authors are responsible for all content in their abstracts including accuracy of the facts, statements, citing resources, etc.

### Results

Of 41 patients with available demographic data, 20 (49%) were male. Median age at presentation was 1 year (range 0–8) and at diagnosis 3.7 years (0.6–30). All patients carried biallelic variants; homozygous in 38 and compound het in 8 cases.  Among 31 distinct variants (14 new), 20 (64.5%) were truncating, 9 (29%) missense, and 2 (6.5%) deletions. Sixteen variants (52%) were absent in gnomAD, 9 were ultra-rare (<0.001%) and 6 present at ≥0.001% frequency.
Hematological abnormalities occurred in 96% (44/46). Most patients presented with pancytopenia (55%, 20/36), while thrombocytopenia was the most common initial isolated cytopenia (10%) often progressing to BMF.
At diagnosis, 70% (23/33) had hypocellular marrow, 21% (7/33) were diagnosed as aplastic anemia, and 21% (7/33) showed dysplastic features. Cytogenetics was normal in 80% (28/35); trisomy 8 was the only recurrent abnormality (n=2). Extra-hematological features were present in 45/46 patients, encompassing short stature, pancreatic insufficiency, skin, dental, and ocular abnormalities, musculoskeletal anomalies, microcephaly, dysmorphism, neurological features, and genitourinary/cardiovascular malformations. Somatic variant testing (n=14) detected mutations in 64% (n=9), involving *TP53* (5), *IDH1* (2), *RUNX1* (2), *TET2* (2), *CSF3R* (1), and *PHF6* (1). One patient with biallelic *TP53* mutations progressed to myeloid malignancy while 2 patients with high-VAF *TP53* clones (15–27%) showed stable mild cytopenias without additional events after 3 years. Notably, 7 patients demonstrated spontaneous hematologic stabilization, suggesting possible somatic genetic rescue. Four patients (8.5%) developed myeloid neoplasia (2 low-blast MDS; 2 MDS/AML). Allogeneic HSCT was performed in 17 patients; median age at transplant was 2.8 years (0.8–9.3). Post-transplant survival was 64%, with 80% of deaths attributable to transplant-related toxicity. Overall, 80% of patients were alive at last follow-up.

### Summary/Conclusion

Biallelic *DNAJC21* variants cause an early-onset multisystem BMF disorder with high penetrance for hematologic abnormalities and multi-organ manifestations. Based on this cohort, prevalence of MDS/AML is relatively low (~10%) possibly due to early transplant; however, detection of clonal hematopoiesis may identify patients at higher risk of progression. Prospective collaborative cohorts are needed to better define disease evolution and optimize surveillance strategies.

**Copyright Information:** (Online) ISSN: 2572-9241
©2026 The Author(s). HemaSphere is published by John Wiley & Sons Ltd on behalf of European Hematology Association. This is an open access Abstract Book distributed under the Attribution-NonCommercial-NoDerivs (CC BY-NC-ND), which allows third parties to download the articles and share them with others as long as they credit the author and the Abstract Book, but they cannot change the content in any way or use them commercially.
**Abstract Book Citations:** Authors, Title, HemaSphere, 2026;10:(S1):pages. The abstract book DOIs can be found on the "Author Information" page.
**Disclaimer:** Articles published in the journal HemaSphere exclusively reflect the opinions of the authors. The authors are responsible for all content in their abstracts including accuracy of the facts, statements, citing resources, etc.

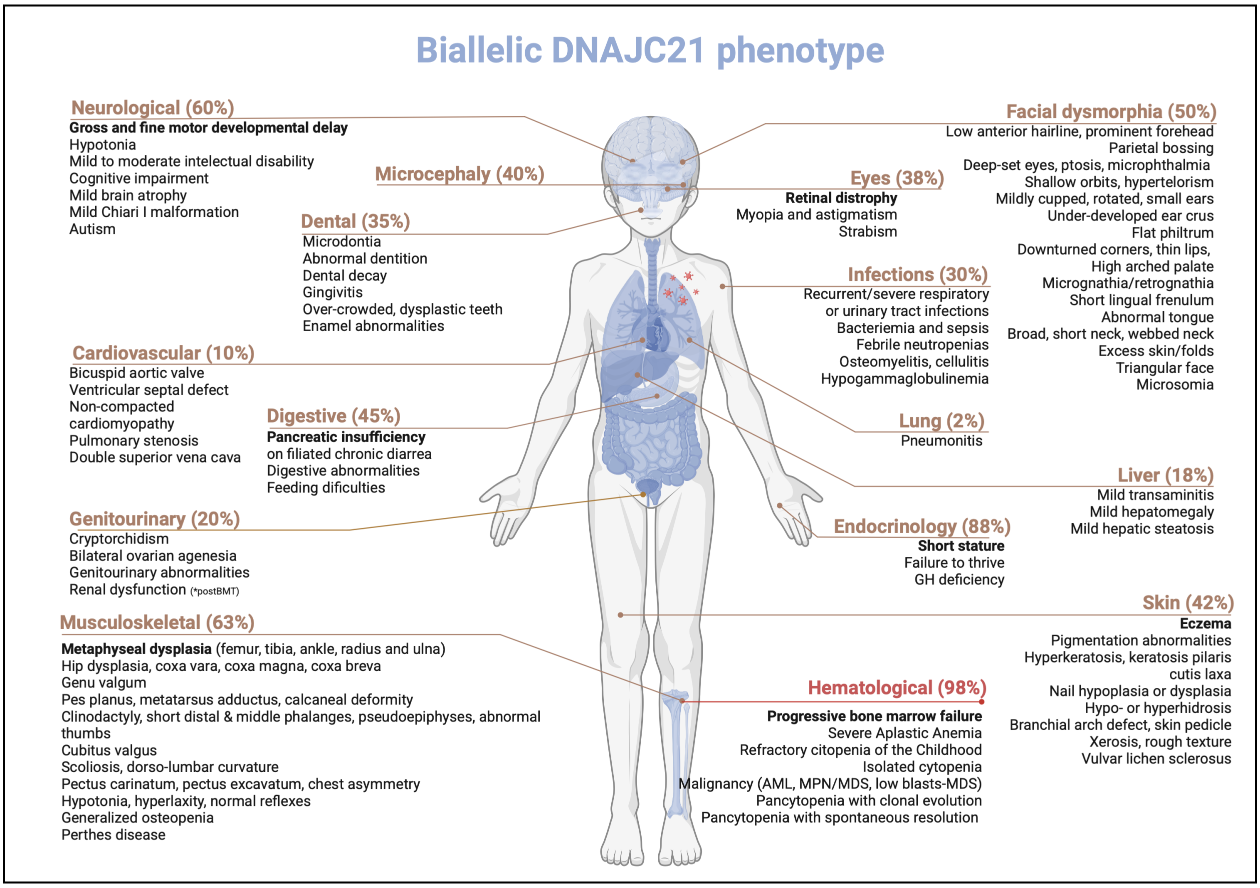

DNAJC21 hematological and extrahematological phenotype

**Copyright Information:** (Online) ISSN: 2572-9241
©2026 The Author(s). HemaSphere is published by John Wiley & Sons Ltd on behalf of European Hematology Association. This is an open access Abstract Book distributed under the Attribution-NonCommercial-NoDerivs (CC BY-NC-ND), which allows third parties to download the articles and share them with others as long as they credit the author and the Abstract Book, but they cannot change the content in any way or use them commercially.
**Abstract Book Citations:** Authors, Title, HemaSphere, 2026;10:(S1):pages. The abstract book DOIs can be found on the "Author Information" page.
**Disclaimer:** Articles published in the journal HemaSphere exclusively reflect the opinions of the authors. The authors are responsible for all content in their abstracts including accuracy of the facts, statements, citing resources, etc.

### (S184) ELTROMBOPAG PLUS CYCLOSPORINE A FOR MODERATE APLASTIC ANEMIA (EMAA), A PLACEBO-CONTROLLED, DOUBLE-BLIND, RANDOMIZED PHASE 3 TRIAL: TREATMENT MODIFICATIONS AFTER DOUBLE BLIND TREATMENT PERIOD

**Topic:** 12. Bone marrow failure syndromes incl. PNH - Clinical

Britta Höchsmann*[1, 2], Philippe Schafhausen[3], Fabian Beier[4, 5], Michael Heuser[6, 7], Jörg Westermann[8], Beatrice Drexler[9], Alexander Röth[10], Regina Herbst[11], Mathias Hänel[11], Michaela Schwarz[8], Flore Sicre de Fontbrune[12, 13], Régis Peffault de Latour[12, 13], Astrid Marx-Hofmann[1, 2], Antonia Beitzen-Heineke[3], Jens Panse[4, 5], Felicitas Thol[6], Sixten Körper[1, 2], Tim Henrik Brümmendorf[4, 5], Hubert Schrezenmeier[1, 2]

*[1]University Hospital of Ulm, Institute of Transfusion Medicine, Ulm, Germany;[2]Institute of Clinical Transfusion Medicine and Immunogenetics, German Red Cross Blood Transfusion Service and University Hospital Ulm, Ulm, Germany; [3]University Medical Center Hamburg-Eppendorf, Department of Oncology, Hematology and Bone Marrow Transplantation with Section of Pneumology, Hamburg, Germany; [4]Center for Integrated Oncology Aachen Bonn Cologne Düsseldorf, Aachen, Germany; [5]RWTH Aachen University, Department of Hematology, Oncology, Hemostaseology and Stem Cell Transplantation, Medical Faculty, Aachen, Germany; [6]Hannover Medical School, Department for Hematology, Hemostasis, Oncology and Stem Cell Transplantation, Hannover, Germany; [7]University Hospital Halle (Saale), Martin-Luther-University Halle-Wittenberg, Department of Internal Medicine IV, Halle, Germany; [8]University Medical Center, Dept. of Hematology, Oncology and Tumor Immunology, Campus Virchow Clinic, Charité, Berlin, Germany; [9]University Hospital, Hematology, Basel, Switzerland; [10]University Hospital Essen, Department of Hematology and Stem Cell Transplantation, West German Cancer Center, Essen, Germany; [11]Klinikum Chemnitz, Department of Internal Medicine III, Chemnitz, Germany; [12]French Reference Center for Aplastic Anemia and Paroxysmal Nocturnal Hemoglobinuria, Paris, France; [13]Paris Cité university, Hematology and Transplant Unit, Saint-Louis Hospital, Paris, France;*

### Background

Moderate aplastic anemia (MAA) is defined by hypoplastic bone marrow with cytopenia not meeting the SAA criteria and for which currently no uniform treatment recommendations exists. The EMAA study, a prospective, double-blind, randomized phase III study (NCT02773225; EudraCT2014-000174-19) investigating the combination therapy of EPAG (eltrombopag) plus cyclosporine (CSA) showed a significant improvement in trilineage response in the EPAG plus CSA arm 24 weeks (wks) after treatment start without safety concerns.

### Aims

Proof of efficacy and safety of first- and second-line treatment strategies with EPAG + CSA in MAA based on data of the EMAA study.

### Methods

Treatment-naïve MAA pts were randomized to placebo (n=44) or EPAG (150 mg/die) (n=41) in combination with CSA. After unblinding after 24 wks treatment depended on randomization and complete (CR), partial (PR) and no response (NR) (defined according to the protocol): in group B (placebo-arm with CR, n=2) and group D (NR in EPAG-arm, n=9) EPAG was discontinuated. EPAG was continued in group C (EPAG arm with PR/CR, n=26) and started in group A (NR/PR in placebo arm, n = 38). Group A pts and group C received study treatment until a stable response or a maximum of 48 wks. Response, relapse and failure free survival and safety were assessable in 85 pts at wk 12, 75 pts at wk 24 wk, 67 pts at wk 48 and 56 pts at wk 72.

Copyright Information: (Online) ISSN: 2572-9241
©2026 The Author(s). HemaSphere is published by John Wiley & Sons Ltd on behalf of European Hematology Association. This is an open access Abstract Book distributed under the Attribution-NonCommercial-NoDerivs (CC BY-NC-ND), which allows third parties to download the articles and share them with others as long as they credit the author and the Abstract Book, but they cannot change the content in any way or use them commercially.
Abstract Book Citations: Authors, Title, HemaSphere, 2026;10:(S1):pages. The abstract book DOIs can be found on the "Author Information" page.
Disclaimer: Articles published in the journal HemaSphere exclusively reflect the opinions of the authors. The authors are responsible for all content in their abstracts including accuracy of the facts, statements, citing resources, etc.

**Results**

Patient characteristics were balanced; compliance in the placebo-/ EPAG-arm was 88.6%/90.2%. In 5 pts (2 EPAG/3 placebo) dose was escalated to 225 mg/daily without complications. First line EPAG treatment significantly improved trilineage ORR after 12 wks (48.8% [95 % CI, 34.3; 63.5] vs. 25% ([95 % CI, 14.4; 39.6] p=0.02) and 24 wks (71.4% [95 % CI, 54.8; 83.8],in 35/41 pts) vs. 42.5% [95 % CI, 28.5; 57.8]; in 40/44pts; p = 0.011). Switch to EPAG in group A significantly improved ORR/CR from 50.7% (no CR) at wk 24 to 65.8%/28.8% at wk 48 (n=34/38pts) (p=0.009; McNemar test) and single lineage platelets response (p= 0.002). In Group C ORR/CR increased after 48 wks to 88.5%/34.6% (n=26/26pts) (p=0.617). At wk 72 in pts with known response status ORR/CR was 75 %/45.8% in group A (24/34 pts) and 89.4 % /36.8 % in group C (19/26 pts).Additionally a significant better single lineage response was shown in the EPAG-arm after 24 wks for neutrophils 58.5% [95 % CI, 43.4; 72.3] vs. 31.8 % [95 % CI, 19.9; 46.6]; p= 0.012), after 48 wks for platelets (56.1 %[95 % CI, 41; 70.1] vs. 34.1% [95 % CI, 21.8; 48.9]; p=0.034) as well as a shorter duration to neutrophil response with median 2.79 mo (IQR 2.76-3.99) vs. median 5.52 mo (IQR 2.77-8.33); p=0.025). In pts with first line EPAG cumulative incidence of trilineage response was superior to CSA only (HR 1.71; p=0.039) and median failure-free survival was numerically the longest with 99 wk [36.14; NA] vs 24.50 wk [24.00; 56.14] in group A without reaching significance between the groups in general (p=0.187, log-rank test). No significant differences of the cumulative relapse rate between placebo and EPAG arm (p = 0.726, log-rank test) as well as group A and C (p = 0.324) or safety findings were observed.

**Summary/Conclusion**

The EMAA study shows that EPAG plus CSA-combination is in first- and second-line therapy in MAA safe and efficient with significant superior cumulative response with EPAG upfront. Thus, a combination of EPAG and immunosuppression has the potential to become the standard therapy for MAA, especially in first-line therapy.

**Copyright Information:** (Online) ISSN: 2572-9241
©2026 The Author(s). HemaSphere is published by John Wiley & Sons Ltd on behalf of European Hematology Association. This is an open access Abstract Book distributed under the Attribution-NonCommercial-NoDerivs (CC BY-NC-ND), which allows third parties to download the articles and share them with others as long as they credit the author and the Abstract Book, but they cannot change the content in any way or use them commercially.
**Abstract Book Citations:** Authors, Title, HemaSphere, 2026;10:(S1):pages. The abstract book DOIs can be found on the “Author Information” page.
**Disclaimer:** Articles published in the journal HemaSphere exclusively reflect the opinions of the authors. The authors are responsible for all content in their abstracts including accuracy of the facts, statements, citing resources, etc.

### (S185) SUCCESSFUL IN VIVO CAR-T GENERATION AND MINIMAL RESIDUAL DISEASE (MRD) CLEARANCE WITH KLN-1010 ACROSS DIVERSE BASELINE T CELL PHENOTYPES IN RELAPSED/REFRACTORY MULTIPLE MYELOMA (RRMM)

**Topic:** 13. Myeloma and other monoclonal gammopathies - Biology & translational research

Andrew Spencer*[1], Simon Harrison[2, 3], Sueh-Li Lim[1, 4], Stephanie Talam[5], Hannah Pahl[6], Dharmesh Dingar[7], Scott Currence[7], Travis Quigley[7], Greg Kaufman[7], P. Joy Ho[5, 8]

*[1]Alfred Hospital-Monash University, Australian Centre for Blood Diseases, Melbourne, Australia;[2]Peter MacCallum Cancer Centre, Centre of Excellence for Cellular Immunotherapy, Melbourne, Australia; [3]University of Melbourne, Sir Peter MacCallum Department of Oncology, Parkville, Australia; [4]Alfred Hospital, Malignant Haematology, Transplantation and Cellular Therapies Service, Melbourne, Australia; [5]Royal Prince Alfred Hospital, Sydney, Australia;[6]Peter MacCallum Cancer Centre, Parkville Cancer Clinical Trials Unit, Melbourne, Australia; [7]Kelonia Therapeutics, Inc., Boston, United States of America; [8]University of Sydney, Sydney, Australia;*

### Background

KLN-1010 is an investigational off-the-shelf gene therapy that generates anti-BCMA CAR-T cells in vivo via intravenous administration of a modified lentiviral vector, eliminating the need for apheresis, ex vivo manufacturing, and lymphodepletion. A critical feasibility question for in vivo CAR-T therapy is whether successful T cell transduction and expansion can occur in heavily pretreated patients with heterogeneous baseline immune characteristics related to prior treatment-related lymphopenia and varied T cell lineages including T cell exhaustion phenotypes.

### Aims

To evaluate the feasibility and describe the early relationship between baseline T cell characteristics and in vivo CAR-T generation, expansion, and clinical outcomes in patients with RRMM treated with KLN-1010.

### Methods

Patients with RRMM, ≥3 prior lines of therapy (including a proteasome inhibitor, immunomodulatory drug, and anti-CD38 antibody), and adequate organ function were enrolled in the inMMyCAR Phase 1 dose-escalation study. Baseline peripheral blood was analyzed for absolute lymphocyte count (ALC), absolute CD3+ count, CD4/CD8 ratio, PD-1 expression, and T cell memory phenotypes. Efficacy was evaluated by International Myeloma Working Group (IMWG) response criteria.

**Copyright Information:** (Online) ISSN: 2572-9241
©2026 The Author(s). HemaSphere is published by John Wiley & Sons Ltd on behalf of European Hematology Association. This is an open access Abstract Book distributed under the Attribution-NonCommercial-NoDerivs (CC BY-NC-ND), which allows third parties to download the articles and share them with others as long as they credit the author and the Abstract Book, but they cannot change the content in any way or use them commercially.
**Abstract Book Citations:** Authors, Title, HemaSphere, 2026;10:(S1):pages. The abstract book DOIs can be found on the "Author Information" page.
**Disclaimer:** Articles published in the journal HemaSphere exclusively reflect the opinions of the authors. The authors are responsible for all content in their abstracts including accuracy of the facts, statements, citing resources, etc.

**Results**

Six patients (age 61-74 yrs, 3-5 prior lines, all triple-class exposed/refractory) demonstrated marked heterogeneity in baseline T cell parameters. Baseline ALC ranged from 0.5-2.1×$10^9$/L, absolute CD3+ counts from 369-1304 cells/μL, and CD4/CD8 ratios from 0.41-0.84 (all inverted ratios). Baseline PD-1 expression on CD8+ T cells ranged 6%-86%, indicating variable T cell exhaustion. Baseline T cell phenotypes varied substantially, with naive T cells comprising 13%-56% of CD8+ cells and 7%-31% of CD4+ cells, while memory subsets showed similar heterogeneity.

Despite baseline variability, in vivo CAR-T generation occurred in all patients. Peak vector copy number (VCN) ranged from 41,405-109,371 copies/μg genomic DNA (D15-22). Peak ALC was on D11-18 and ranged from 2.3-43.1×$10^9$/L. CAR transgene was detectable in peripheral blood at the last assessed timepoint for all patients, up to 6 months post-infusion. Baseline PD-1+ CD8+ T cell frequency did not affect CAR-T exposure by VCN area under the curve through D28. Despite T cell heterogeneity pre-treatment, all patients demonstrated memory phenotype expansion following treatment.

All patients achieved MRD-negative responses. IMWG responses deepened over time; best responses include 2 stringent complete responses and 4 very good partial responses. All patients remain in ongoing response at median follow-up of 3.5 mo.

Grade 1-2 cytokine release syndrome (CRS) occurred in 4 patients during CAR-T expansion, with no Grade ≥3 CRS. No ICANS or delayed neurotoxicity were observed. IRRs occurred in 3 patients and resolved within 6-48 hours. Cytopenias were limited, with no grade ≥3 cytopenias at or beyond D28. No serious treatment-emergent (TE) infections occurred through D84 follow-up.

**Summary/Conclusion**

KLN-1010 achieved consistent in vivo CAR-T generation, expansion, and clinical efficacy across a range of baseline T cell characteristics, including low absolute T cell counts, inverted CD4/CD8 ratios, elevated PD-1 expression, and heterogeneous memory phenotypes. A manageable safety profile, including limited cytopenias and no severe TE infections without lymphodepletion, further supports the clinical utility of this approach. Updated efficacy and safety data with extended follow-up will be presented.

© 2026 American Society of Clinical Oncology, Inc. Reused with permission. This abstract was accepted and previously presented at the 2026 ASCO Annual Meeting. All rights reserved.

**Table 1.** Selected efficacy outcomes for patients in the inMMyCAR Phase 1 trial.

| Patient | Dose level | Timepoints of MRD-negative results* | IMWG response | Duration of follow-up |
|---|---|---|---|---|
| #1 | 2.0 x $10^7$ IU/kg | Month 1, Month 3, Month 6 | **Stringent CR** | 6+ months |
| #2 | | Month 1, Month 3 | VGPR | 5+ months |
| #3 | | Month 1, Month 3 | VGPRu | 4+ months |
| #4 | 0.6 x $10^7$ IU/kg | Month 1, Month 3 | VGPR | 3+ months |
| #5 | | Month 1 | **Stringent CR** | 2+ months |
| #6 | | Month 1 | VGPRu | 2+ months |

*By next-gen flow cytometry or sequencing with sensitivity of 10[illegible] or 10[illegible].
CR, complete response; IMWG, International Myeloma Working Group; IU, infectious unit; MRD, minimal residual disease; u, unconfirmed; VGPR, very good partial response.

Copyright Information: (Online) ISSN: 2572-9241
©2026 The Author(s). HemaSphere is published by John Wiley & Sons Ltd on behalf of European Hematology Association. This is an open access Abstract Book distributed under the Attribution-NonCommercial-NoDerivs (CC BY-NC-ND), which allows third parties to download the articles and share them with others as long as they credit the author and the Abstract Book, but they cannot change the content in any way or use them commercially.
Abstract Book Citations: Authors, Title, HemaSphere, 2026;10:(S1):pages. The abstract book DOIs can be found on the “Author Information” page.
Disclaimer: Articles published in the journal HemaSphere exclusively reflect the opinions of the authors. The authors are responsible for all content in their abstracts including accuracy of the facts, statements, citing resources, etc.

### (S186) THE DYNAMIC LANDSCAPE OF IMMUNE ENVIRONMENT FOR MM PATIENTS RECEIVING T CELL REDIRECTING THERAPIES

**Topic:** 13. Myeloma and other monoclonal gammopathies - Biology & translational research

Haimeng Li*[1], Xuelin Dou[2, 3], Yang Liu[2], Nan Peng[2], Lei Wen[4], Liru Wang[5], Luyang Sun[1], Jin Lu[2]

*[1]Peking University Health Science Center, BeiJing, China;[2]Peking University People's Hospital, Beijing, China;[3]Peking University People's Hospital, BeiJing, China; [4]Peking University People's Hospital, Peking University Institute of Hematology, BeiJing, China; [5]Department of Hematology, Fu Xing Hospital, Capital Medical University, Beijing, China;*

### Background

T-cell redirecting therapies (CAR-T and TCEs) revolutionized multiple myeloma (MM) treatment, yet resistance remains a major clinical hurdle. Deciphering how these therapies differentially reshape the immune ecosystem and tumor evolution is critical.

### Aims

To determine essential differences in immune remodeling induced by CAR-T versus TCEs, and identify cellular determinants driving long-term response versus resistance.

### Methods

We performed longitudinal scRNA-seq and TCR/BCR tracking on MM patients receiving CAR-T or TCEs across four timepoints (Pre, Early, Mid, Late) to construct an immune-tumor co-evolution atlas.

**Copyright Information:** (Online) ISSN: 2572-9241
©2026 The Author(s). HemaSphere is published by John Wiley & Sons Ltd on behalf of European Hematology Association. This is an open access Abstract Book distributed under the Attribution-NonCommercial-NoDerivs (CC BY-NC-ND), which allows third parties to download the articles and share them with others as long as they credit the author and the Abstract Book, but they cannot change the content in any way or use them commercially.
**Abstract Book Citations:** Authors, Title, HemaSphere, 2026;10:(S1):pages. The abstract book DOIs can be found on the "Author Information" page.
**Disclaimer:** Articles published in the journal HemaSphere exclusively reflect the opinions of the authors. The authors are responsible for all content in their abstracts including accuracy of the facts, statements, citing resources, etc.

### Results

- **1. Distinct Remodeling & TIME Modules:**
CAR-T induced profound immune remodeling, showing broader T-cell expansion and higher TCR network hubness than TCEs (Figure 3D-F). We defined five TIME modules: Mem, Tef, IST, ISM, and Mye (Figure 2A-D). Responders polarized toward TIME-Tef/Mem (Figure 2H-I), correlating with prolonged PFS (Figure 2F-G). Non-responders were trapped in suppressive TIME-ISM/IST, with CAR-T failures facing broader myeloid suppression (Figure 2E).
- **2. Shared Efficacy Axis:**
The transition from early cytotoxic expansion (CD8+ Tef_CX3CR1) to long-term memory (CD8+ Tem_GZMK/A) drives durable remission across both therapies (Figure 3G-I, Figure 5B-E). Early Tef_CX3CR1 expansion predicts efficacy, while transitioning to memory secures maintenance. High baseline exhaustion (Tex_TOX) inhibited this axis (Figure 3O).
- **3. TCE-Specific Resistance:**
Highly cytotoxic FGFBP2+ CD8+ Tef accelerated initial TCE response (Figure 4A-G). Long-term exposure triggered an "acquired brake" via late-stage accumulation of TIGIT+ exhausted T cells (Figure 4H-L). The "TRIS" model, balancing effector expansion against TIGIT+ accumulation, accurately stratified PFS (Figure 4Q-S).
- **4. CAR-T Specific Resistance:**
CAR-T efficacy was limited by an extrinsic myeloid barrier; elevated CCR8+ CAR-Tregs predicted poor responses (Figure 5D-E). A CD16-Mono-CSF1R population with M2-like features accumulated in Non-responders (Figure 6N-P), suppressing CAR-Tef/Mem via MIF-FASLG (Figure 6S). A "CAR-T Identifier" incorporating this penalty improved outcome prediction (Figure 6T).
- **5. Shared Tumor Escape:**
Gene NMF revealed a shared S100A8/A9/A4+ inflammatory myeloma state (GM02) across patients (Figure 7A-G). Driven by 1q21 amplification (Figure 7K), this plastic clone survived immune attack and suppressed T cells via MIF-FASLG (Figure 7P-Q), serving as a shared root of relapse.

### Summary/Conclusion

While the "Tef-to-Tem" transition drives efficacy in both modalities, resistance mechanisms diverge fundamentally: TCEs are limited by intrinsic T-cell acquired exhaustion (TIGIT+), whereas CAR-T is hindered by extrinsic myeloid suppression (CD16-Mono-CSF1R). The 1q21-driven S100A8+ clone represents a shared immune evasion route. These findings provide a rationale for precision combinations—Anti-TIGIT for TCEs and CSF1R-blockade for CAR-T.

**Copyright Information:** (Online) ISSN: 2572-9241
©2026 The Author(s). HemaSphere is published by John Wiley & Sons Ltd on behalf of European Hematology Association. This is an open access Abstract Book distributed under the Attribution-NonCommercial-NoDerivs (CC BY-NC-ND), which allows third parties to download the articles and share them with others as long as they credit the author and the Abstract Book, but they cannot change the content in any way or use them commercially.
**Abstract Book Citations:** Authors, Title, HemaSphere, 2026;10:(S1):pages. The abstract book DOIs can be found on the "Author Information" page.
**Disclaimer:** Articles published in the journal HemaSphere exclusively reflect the opinions of the authors. The authors are responsible for all content in their abstracts including accuracy of the facts, statements, citing resources, etc.

**Results**

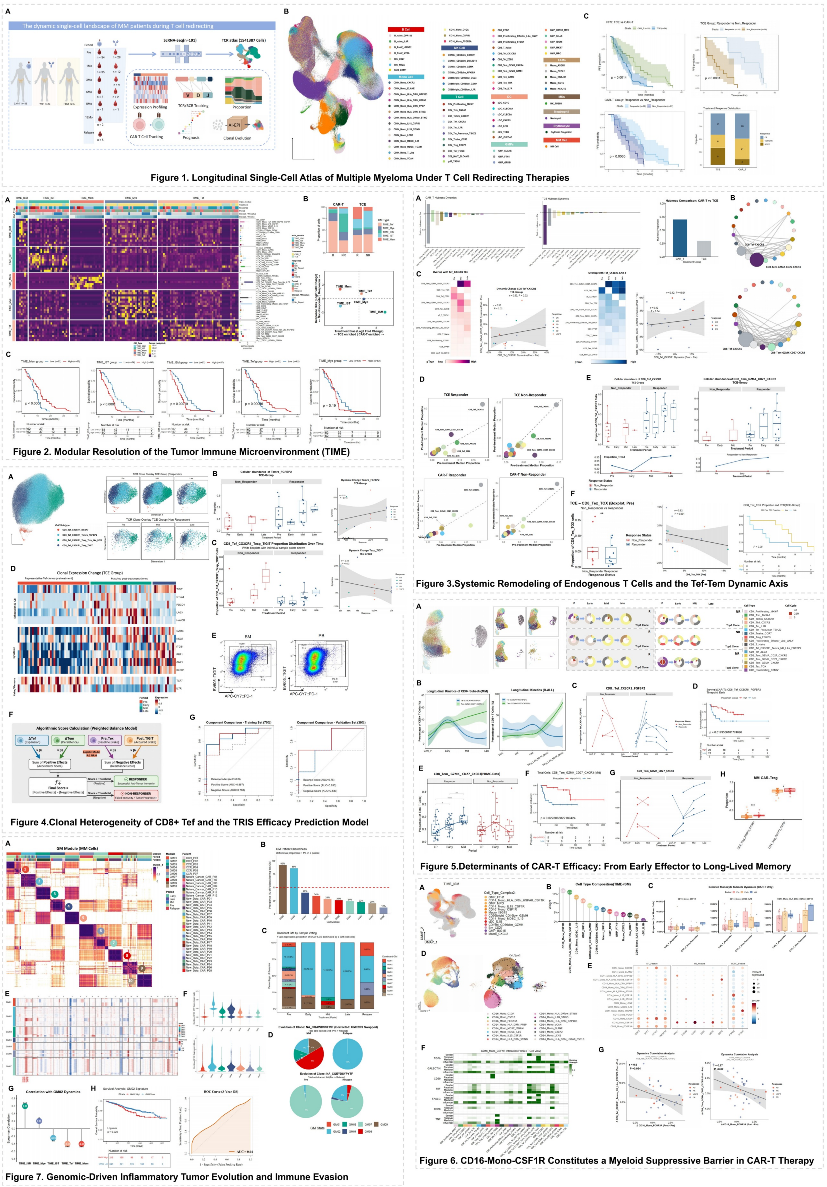

Figure 1. Longitudinal Single-Cell Atlas of Multiple Myeloma Under T Cell Redirecting Therapies

Figure 2. Modular Resolution of the Tumor Immune Microenvironment (TIME)

Figure 3.Systemic Remodeling of Endogenous T Cells and the Tef-Tem Dynamic Axis

Figure 4.Clonal Heterogeneity of CD8+ Tef and the TRIS Efficacy Prediction Model

Figure 5.Determinants of CAR-T Efficacy: From Early Effector to Long-Lived Memory

Figure 6. CD16-Mono-CSF1R Constitutes a Myeloid Suppressive Barrier in CAR-T Therapy

Figure 7. Genomic-Driven Inflammatory Tumor Evolution and Immune Evasion

**Copyright Information:** (Online) ISSN: 2572-9241
©2026 The Author(s). HemaSphere is published by John Wiley & Sons Ltd on behalf of European Hematology Association. This is an open access Abstract Book distributed under the Attribution-NonCommercial-NoDerivs (CC BY-NC-ND), which allows third parties to download the articles and share them with others as long as they credit the author and the Abstract Book, but they cannot change the content in any way or use them commercially.
**Abstract Book Citations:** Authors, Title, HemaSphere, 2026;10:(S1):pages. The abstract book DOIs can be found on the "Author Information" page.
**Disclaimer:** Articles published in the journal HemaSphere exclusively reflect the opinions of the authors. The authors are responsible for all content in their abstracts including accuracy of the facts, statements, citing resources, etc.

### (S187) AZD0120, A DUAL BCMA/CD19 CAR-T CELL THERAPY, DEMONSTRATES A NAIVE/CENTRAL MEMORY PHENOTYPE AND INDUCES A RAPID DECLINE IN SOLUBLE BCMA IN RELAPSED/REFRACTORY MULTIPLE MYELOMA

**Topic:** 13. Myeloma and other monoclonal gammopathies - Biology & translational research

Binod Dhakal*[1], Shambavi Richard[2], Patricia Cheung[3], Liang Li[4], Meiyue Hong[5], Yu-Feng Lin[6], Jennifer MA[7], Rebecca Shah[3], Scott Goldsmith[8]

*[1]Medical College of Wisconsin, Milwaukee, WI, United States of America;[2]Icahn School of Medicine at Mount Sinai, New York, NY, United States of America; [3]AstraZeneca, South San Francisco, CA, United States of America; [4]AstraZeneca, Gaithersburg, MD, United States of America; [5]AstraZeneca, Boston, MA, United States of America; [6]AstraZeneca, Waltham, MA, United States of America; [7]AstraZeneca, Santa Monica, CA, United States of America;[8]City of Hope Comprehensive Cancer Center, Duarte, CA, United States of America;*

### Background

Patients (pts) with multiple myeloma (MM) typically require multiple lines of therapy (LOT). AZD0120 is a BCMA/CD19 dual-targeting CAR-T cell therapy manufactured via a next-generation rapid process to enhance T cell fitness and accelerate pt access. Prior Ph1 data showed deep and durable responses and favorable safety with AZD0120 in newly diagnosed and relapsed/refractory MM (RRMM) (Du et al. ASCO2023, EHA2024, and ASH2025).

### Aims

To report translational results from the Ph 1b part of DURGA-1, an ongoing Ph 1b/2 trial evaluating AZD0120 in RRMM.

### Methods

Eligible pts in this open-label, single-arm, US-based, multicenter study (NCT05850234) were aged ≥18 years with RRMM (3+ prior LOT including a proteasome inhibitor, immunomodulatory drug, and anti-CD38 antibody), and documented disease progression on the most recent anti-myeloma therapy. Pts underwent lymphodepletion followed by a single infusion of AZD0120 at $1x10^5$ (dose level [DL] 1) or $3x10^5$ (DL2) cells/kg. Translational assessments included CAR-T cell product immunophenotyping, peripheral blood CAR transgene kinetics, soluble BCMA (sBCMA) quantification, flow cytometric enumeration of peripheral (CD19+) B cells, and multiplex cytokine profiling at prespecified timepoints. All pts provided informed consent.

**Copyright Information:** (Online) ISSN: 2572-9241
©2026 The Author(s). HemaSphere is published by John Wiley & Sons Ltd on behalf of European Hematology Association. This is an open access Abstract Book distributed under the Attribution-NonCommercial-NoDerivs (CC BY-NC-ND), which allows third parties to download the articles and share them with others as long as they credit the author and the Abstract Book, but they cannot change the content in any way or use them commercially.
**Abstract Book Citations:** Authors, Title, HemaSphere, 2026;10:(S1):pages. The abstract book DOIs can be found on the "Author Information" page.
**Disclaimer:** Articles published in the journal HemaSphere exclusively reflect the opinions of the authors. The authors are responsible for all content in their abstracts including accuracy of the facts, statements, citing resources, etc.

**Results**

An average of 70% of cells in evaluated CAR-T cell products displayed less differentiated (naïve/stem cell memory or central memory) phenotypes, a profile associated with favorable CAR-T outcomes. Cellular kinetic analysis from 22 evaluable pts (data cutoff [DCO] 10Dec2025) demonstrated similar characteristics across DLs, with a median Tmax for CAR-T cell expansion of 13 days (d) post-infusion. Persistence was observed in all pts up to 56 d and in 75% of pts up to 84 d. Rapid sBCMA reduction was primarily observed within 14 d post-AZD0120 infusion (1 pt at 21 d), with reduction continuing to deepen until at least end of month (M) 2, and in 1 pt up to M 12. CD19+ cell depletion was maintained across DLs within the first M and sustained up to at least end of M 2 or the latest timepoint reached. Two DL1 pts and two DL2 pts showed complete CD19+ cell recovery by M 6 and 9, respectively. Cytokine release syndrome (CRS; DCO 12Dec2025) was reported in 9/12 (75%) pts in DL1 (all Grade [Gr] 1) and 8/15 (53%) in DL2 (n=7 Gr 1; n=1 Gr 2), with no cases of Gr ≥3 CRS. No pts had peak absolute lymphocyte count (ALC) >$9x10^9$/L (median <$2x10^9$/L); ALC peak was significantly higher in pts with CRS. In cytokine analysis, IL-15 showed typical post-lymphodepletion induction and gradually returned to baseline in all pts. Similar to other CAR-Ts, IL-6, IL-8, IL-10, TNF-α, and IFN-γ increased and peaked at d 7-10 following AZD0120 infusion, with significantly higher peak levels in pts with CRS. Analysis of ferritin and C-reactive protein (CRP) showed association between baseline ferritin and CRS, but no significant association for baseline or peak CRP. Of MRD-evaluable pts, 16/17 (94%) were MRD-negative at M 1 by next-generation sequencing at a sensitivity of $10^{-5}$ (DL1 8/8; DL2 8/9).

**Summary/Conclusion**

AZD0120 exhibited a predominantly naïve/central memory phenotype and induced rapid and deep sBCMA depletion, robust CAR-T cell expansion/persistence, and sustained CD19+ depletion, all of which correlated with deep early disease control in RRMM. These findings support AZD0120 as a promising treatment for RRMM. Updated clinical data on safety and efficacy will be presented at EHA2026.

**Copyright Information:** (Online) ISSN: 2572-9241
©2026 The Author(s). HemaSphere is published by John Wiley & Sons Ltd on behalf of European Hematology Association. This is an open access Abstract Book distributed under the Attribution-NonCommercial-NoDerivs (CC BY-NC-ND), which allows third parties to download the articles and share them with others as long as they credit the author and the Abstract Book, but they cannot change the content in any way or use them commercially.
**Abstract Book Citations:** Authors, Title, HemaSphere, 2026;10:(S1):pages. The abstract book DOIs can be found on the "Author Information" page.
**Disclaimer:** Articles published in the journal HemaSphere exclusively reflect the opinions of the authors. The authors are responsible for all content in their abstracts including accuracy of the facts, statements, citing resources, etc.

### (S188) DEVELOPMENT OF BMS-986453, A DUAL-TARGETING BCMA×GPRC5D CHIMERIC ANTIGEN RECEPTOR (CAR) FOR THE TREATMENT OF MULTIPLE MYELOMA

**Topic:** 13. Myeloma and other monoclonal gammopathies - Biology & translational research

Kimberly Harrington[1], Jon C Jones[1], Yeonjoo Oh[1], Heather Striegel[1], Eric Jeffery[1], Cyr De Imus[1], Kristen Dang[1], Timothy Johnstone[2], Andrew Drake[1], Fatima Javier[2], Nels Hamacher[1], Asanga Weliwitigoda[1], Dhong Hyun Lee[1], Mike Liu[1], Jaime Jackson[1], Shari Kaiser[1], Luciano J. Costa[3], Sham Mailankody[4], Surbhi Sidana[5], Adriana Rossi[6], Doris Hansen*[7], Colin Godwin[1], Ruth Salmon[1], Douglas Jones[1]

*[1]Bristol Myers Squibb, Princeton, New Jersey, United States of America;[2]Bristol Myers Squibb, (Affiliation at the time of data collection and analysis), Princeton, New Jersey, United States of America; [3]University of Alabama at Birmingham, Birmingham, Alabama, United States of America; [4]Memorial Sloan Kettering Cancer Center, Cellular Therapy and Myeloma Services, New York, New York, United States of America; [5]Stanford University School of Medicine, Stanford, California, United States of America; [6]Icahn School of Medicine at Mount Sinai, New York, New York, United States of America;[7]Moffitt Cancer Center, Tampa, Florida, United States of America;*

### Background

T-cell redirecting therapies (TCRT) targeting BCMA or GPRC5D have demonstrated remarkable efficacy in relapsed/refractory multiple myeloma (RRMM). Both antigens are highly and independently expressed in MM making them attractive targets for CAR T cell therapy. Despite promising responses, most patients who receive TCRT eventually relapse. Loss or reduced expression of BCMA or GPRC5D after antigen-specific therapy suggests antigen heterogeneity or loss may contribute to resistance. Targeting both BCMA and GPRC5D may overcome this mechanism of resistance and lead to deeper, more durable responses.

### Aims

To design, optimize, and evaluate the activity of dual-targeting BCMA×GPRC5D CAR T cell therapy for the treatment of MM.

### Methods

To enable dual targeting, we designed a library of CAR constructs using bicistronic, linear tandem, and loop tandem formats. To identify the optimal design, we compared CAR expression, tonic signaling, *in vitro* function against single- and dual-positive target cells and affinity toward dual-expressing cells. We also evaluated *in vivo* efficacy in multiple xenograft models, including a model of antigen heterogeneity, to drive selection of BMS-986453.
BMS-986453 was further developed for clinical evaluation and is currently being studied in a first-in-human, multicenter trial (CA119-0002; NCT06153251). The initial study population includes patients with RRMM who have received ≥3 prior lines of therapy (LOTs). Baseline BCMA and GPRC5D expression levels were assessed on patient plasma cells.

**Copyright Information:** (Online) ISSN: 2572-9241
©2026 The Author(s). HemaSphere is published by John Wiley & Sons Ltd on behalf of European Hematology Association. This is an open access Abstract Book distributed under the Attribution-NonCommercial-NoDerivs (CC BY-NC-ND), which allows third parties to download the articles and share them with others as long as they credit the author and the Abstract Book, but they cannot change the content in any way or use them commercially.
**Abstract Book Citations:** Authors, Title, HemaSphere, 2026;10:(S1):pages. The abstract book DOIs can be found on the “Author Information” page.
**Disclaimer:** Articles published in the journal HemaSphere exclusively reflect the opinions of the authors. The authors are responsible for all content in their abstracts including accuracy of the facts, statements, citing resources, etc.

### Results

Both bicistronic and tandem designs showed robust CAR expression and strong activity against BCMA-positive, GPRC5D-positive, and dual-expressing cells *in vitro* and *in vivo*. However, the bicistronic approach exhibited heterogenous CAR expression due to molecular recombination. In contrast, tandem designs produced uniform expression of both binding domains and showed no unexpected recombination, with the loop tandem configuration outperforming the linear tandem and bicistronic approaches. The lead loop tandem binding domain had superior *in vivo* efficacy in a model of antigen heterogeneity (Figure 1A) and also demonstrated higher affinity toward dual-expressing cells compared to single-antigen binding domains. Based on these findings, BMS-986453 was selected for further clinical development (Figure 1B).
We confirmed both BCMA and GPRC5D are highly expressed with some patient-to-patient variability in the ongoing phase 1 multicenter trial. Initial clinical results in triple-class–exposed RRMM populations showed clinical activity, including deep responses in patients with variable antigen expression.

### Summary/Conclusion

Expression analyses in this trial demonstrated that BCMA and GPRC5D are highly expressed in patients with at least 3 prior LOTs, confirming their suitability as dual-targeting CAR antigens. The loop tandem design was superior to bicistronic or linear tandem approaches, and the resulting CAR demonstrated potent dual-antigen activity in preclinical studies. These data supported advancement of BMS-986453 into clinical development, where early phase 1 results (NCT06153251) indicate a highly functional and potent CAR capable of effectively targeting both BCMA and GPRC5D in patients.
DJ and RS contributed equally as co-senior authors.

**Figure 1. A) MM.1S antigen heterogeneity MM tumor model in NSG mice; B) BCMA×GPRC5D CAR construct with loop tandem design selected for further clinical development (BMS-986453).**

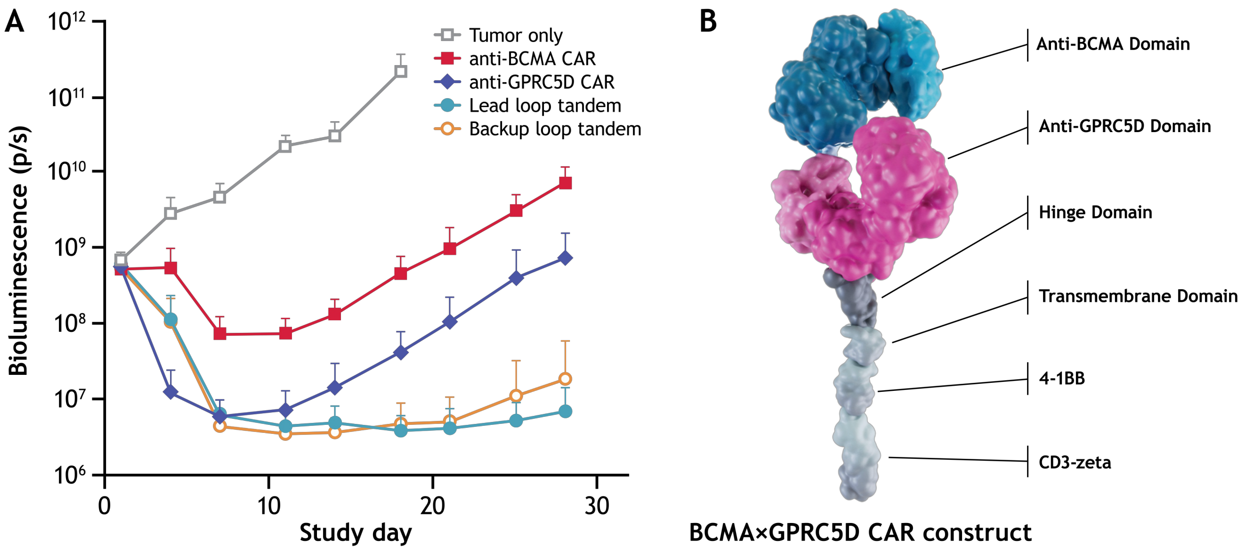

BCMA, B-cell maturation antigen; CAR, chimeric antigen receptor; GPRC5D, G protein-coupled receptor class C group 5 member D; MM, multiple myeloma; NSG, NOD scid gamma.

Figure 1

**Copyright Information:** (Online) ISSN: 2572-9241
©2026 The Author(s). HemaSphere is published by John Wiley & Sons Ltd on behalf of European Hematology Association. This is an open access Abstract Book distributed under the Attribution-NonCommercial-NoDerivs (CC BY-NC-ND), which allows third parties to download the articles and share them with others as long as they credit the author and the Abstract Book, but they cannot change the content in any way or use them commercially.
**Abstract Book Citations:** Authors, Title, HemaSphere, 2026;10:(S1):pages. The abstract book DOIs can be found on the "Author Information" page.
**Disclaimer:** Articles published in the journal HemaSphere exclusively reflect the opinions of the authors. The authors are responsible for all content in their abstracts including accuracy of the facts, statements, citing resources, etc.

### (S189) BTL-101, A FULLY HUMAN TRISPECIFIC BCMA×GPRC5D×CD3 T-CELL ENGAGER FOR THE TREATMENT OF MULTIPLE MYELOMA

**Topic:** 13. Myeloma and other monoclonal gammopathies - Biology & translational research

Wei Ran[1], Xilu Sun[1], Jiansong Yang*[1]

[1]*Biotle Inc., Hangzhou, China;*

### Background

Responses to BCMA- or GPRC5D-directed T-cell engagers (TCEs) in relapsed/refractory multiple myeloma (RRMM) can be limited by relapse and antigen escape, supporting dual-antigen targeting while controlling cytokine release to address the unmet medical need for RRMM.

### Aims

This study aimed to characterize BTL-101, a fully human trispecific BCMA×GPRC5D×CD3 T-cell engager with an affinity-optimized CD3 arm, designed to mitigate relapse and antigen escape in RRMM. Objectives were to define binding to all three targets; evaluate T cell–dependent cytotoxicity across heterogeneous/knockdown antigen expression and under soluble BCMA challenge; assess T-cell activation and cytokine release for a tempered inflammatory profile; and confirm in vivo antitumor activity and tolerability in xenograft and CD3-humanized mouse models.

### Methods

BTL-101 is a fully human BCMA×GPRC5D×CD3 trispecific antibody with an affinity-optimized CD3 arm. Binding kinetics/affinity were measured by Bio-Layer Interferometry (BLI). T cell–dependent cytotoxicity, activation markers, and cytokines were assessed in Peripheral Blood Mononuclear Cells (PBMC) or whole-blood co-cultures with myeloma cell lines (H929, MM.1S, U266), including soluble BCMA (sBCMA) challenge up to 500 ng/mL. Antitumor activity was evaluated in H929 and MM.1S cell-derived xenografts (CDX); tolerability and cytokines were assessed in CD3-humanized mice.

### Results

BTL-101 bound CD3, GPRC5D, and BCMA (KD 40.8, 2.08, and 8.66 nM) and showed faster CD3 on/off kinetics than teclistamab (KD 13.3 nM) and talquetamab (KD 81.4 nM). In myeloma co-cultures (H929, MM.1S, U266), BTL-101 mediated potent killing exceeding talquetamab+teclistamab, depleted dual- and single-antigen–positive cells (including low-to-intermediate BCMA expressers), and retained activity against BCMA- or GPRC5D-knockdown targets, consistent with efficacy under heterogeneous antigen expression. Cytotoxicity remained maximal with soluble BCMA up to 500 ng/mL, whereas teclistamab decreased by ~50%; no binding/cytotoxicity was seen on BCMA/GPRC5D-negative cells. In whole-blood NCI-H929 assays, BTL-101 achieved comparable or superior killing with CD69 activation similar to teclistamab/talquetamab, but generally required higher concentrations to drive deep activation (CD25++/CD25++CD69++) and pro-inflammatory cytokines (eg, IL-6, TNF-α), supporting a tempered cytokine/activation profile. In 48-h PBMC–H929 co-cultures (E:T=5:1), BTL-101 showed higher-potency killing with attenuated cytokines and outperformed JNJ-79635322, SIM0500, and MBS-314. In xenografts, BTL-101 improved dose-dependent tumor control versus vehicle and the talquetamab+teclistamab combination; no observable toxicity and lower cytokines were seen in CD3-humanized mice. A common light chain supports manufacturability (7–8 g/L, >98% purity, stable).

**Copyright Information:** (Online) ISSN: 2572-9241
©2026 The Author(s). HemaSphere is published by John Wiley & Sons Ltd on behalf of European Hematology Association. This is an open access Abstract Book distributed under the Attribution-NonCommercial-NoDerivs (CC BY-NC-ND), which allows third parties to download the articles and share them with others as long as they credit the author and the Abstract Book, but they cannot change the content in any way or use them commercially.
**Abstract Book Citations:** Authors, Title, HemaSphere, 2026;10:(S1):pages. The abstract book DOIs can be found on the "Author Information" page.
**Disclaimer:** Articles published in the journal HemaSphere exclusively reflect the opinions of the authors. The authors are responsible for all content in their abstracts including accuracy of the facts, statements, citing resources, etc.

**Summary/Conclusion**

BTL-101 demonstrates strong dual-antigen cytotoxicity with an attenuated cytokine profile and resistance to soluble BCMA. A Phase I/II clinical study is currently ongoing to evaluate the safety, tolerability, pharmacokinetics, and preliminary efficacy of BTL-101 in patients with relapsed/refractory multiple myeloma.

**Copyright Information:** (Online) ISSN: 2572-9241
©2026 The Author(s). HemaSphere is published by John Wiley & Sons Ltd on behalf of European Hematology Association. This is an open access Abstract Book distributed under the Attribution-NonCommercial-NoDerivs (CC BY-NC-ND), which allows third parties to download the articles and share them with others as long as they credit the author and the Abstract Book, but they cannot change the content in any way or use them commercially.
**Abstract Book Citations:** Authors, Title, HemaSphere, 2026;10:(S1):pages. The abstract book DOIs can be found on the "Author Information" page.
**Disclaimer:** Articles published in the journal HemaSphere exclusively reflect the opinions of the authors. The authors are responsible for all content in their abstracts including accuracy of the facts, statements, citing resources, etc.

### (S190) CIRCULATING TUMOR DNA COMPLEMENTS BONE MARROW GENOTYPING AND TRACKS MRD KINETICS IN MULTIPLE MYELOMA: RESULTS FROM A DSMM XVII COHORT

**Topic:** 13. Myeloma and other monoclonal gammopathies - Biology & translational research

Philipp Nakov*[1], Anke Schilhabel[1], Mouhamad Khouja[1], Nikos Darzentas[1], Constantin Foerster[2], Leander Stoeber[2], Jan Krönke[3], Monika Engelhardt[4], Claudia Baldus[1], Hermann Einsele[5], Stefan Knop[2], Monika Brüggemann[1]

*[1]University Hospital Schleswig-Holstein, Department for Hematology and Oncology, Kiel, Germany;[2]Nuremberg General Hospital, Paracelsus Medical University, Department of Hematology and Oncology, Nuremberg, Germany; [3]University Hospital of Greifswald, Department of Internal Medicine C, Greifswald, Germany; [4]Interdisciplinary Cancer Center and Comprehensive Cancer Center Freiburg, University of Freiburg, Department of Hematology and Oncology, Freiburg, Germany; [5]Würzburg University Hospital, Department of Internal Medicine II, Würzburg, Germany;*

**Background**

Molecular analysis of circulating tumor (ct)DNA from cell free (cf)DNA is considered a promising minimally invasive approach for baseline genotyping and longitudinal monitoring of measurable residual disease (MRD) in multiple myeloma (MM). While bone marrow (BM) based analyses by next generation flow cytometry (NGF) and next generation sequencing (NGS) are established gold standards for MRD assessment, clinically validated ctDNA cut-offs, algorithms, as well as the overall prognostic impact remain to be defined but would be valuable tools for future risk and intervention strategies.

**Aims**

To study a multimodal approach combining the molecular sensitivity of NGF with minimal invasive ctDNA monitoring to conclude on valid clinical standards.

**Methods**

Diagnostic and longitudinal plasma samples were genotyped (n=257) from 43 adult, treatment-naïve MM patients (pts) enrolled in the DSMM XVII trial (NCT03948035). At each time point, BM MRD was additionally assessed by NGF. Genotyping of CD138-enriched BM and cfDNA at baseline and cfDNA at follow-up was performed using the EC-NDC-panel (Univ8® Genomics, UK) to identify single-nucleotide variants (SNVs), structural variants (SVs), and immunoglobulin receptor rearrangements. Sequencing data were analysed using the ARResT/Interrogate pipeline, including UMI-aware error suppression and haplotype-based variant enrichment in somatically hypermutated loci. A panel-of-normals was applied to filter sequencing artefacts.

**Copyright Information:** (Online) ISSN: 2572-9241
©2026 The Author(s). HemaSphere is published by John Wiley & Sons Ltd on behalf of European Hematology Association. This is an open access Abstract Book distributed under the Attribution-NonCommercial-NoDerivs (CC BY-NC-ND), which allows third parties to download the articles and share them with others as long as they credit the author and the Abstract Book, but they cannot change the content in any way or use them commercially.
**Abstract Book Citations:** Authors, Title, HemaSphere, 2026;10:(S1):pages. The abstract book DOIs can be found on the “Author Information” page.
**Disclaimer:** Articles published in the journal HemaSphere exclusively reflect the opinions of the authors. The authors are responsible for all content in their abstracts including accuracy of the facts, statements, citing resources, etc.

**Results**

At baseline (paired BM and cfDNA samples, n=18 pts), SVs and recurrent mutations in *KRAS*, *NRAS*, and *TP53* were detected in 61% (SVs), 22% (*NRAS*), 17% (*KRAS*), and 11% (*TP53*) of pts, respectively. In most cases SVs and SNVs were shared across compartments (64% of pts) however, in 2 pts (11%), *NRAS* and *TP53* mutations were detectable only by ctDNA, suggesting complementary diagnostic value. Baseline total cfDNA levels ranged from 1.4–105 ng/ml (median 10.8 ng/ml) and were significantly higher in advanced R-ISS stage (II/III vs I: 27.1 vs 11.8 ng/ml; p =0.008).
A median of 64 markers (range 4-121) were available for longitudinal MRD tracking in follow-up samples, based on combined analysis of the cfDNA and BM using haplotype enrichment.
Among 227 follow-ups from n=43 pts with BM/cfDNA sample pairs, 130 were negative by both NGF and ctDNA, and 16 were positive by both methods. Discordance was frequent: 53 samples were NGF-positive/ctDNA-negative, and 28 were ctDNA-positive/NGF-negative. When correlated with NGF-defined MRD kinetics, ctDNA longitudinal trajectories were concordant with the NGF-MRD course in 28 pts (65%), supporting the ability of plasma-based monitoring to reflect MRD dynamics over time. In the remaining pts, 12 (28%) showed NGF-detected MRD persistence/recurrence that was not captured by ctDNA, consistent with reduced plasma sensitivity at very low disease levels (NGF-MRD $<1\times10^{-4}$). Three pts (7%) exhibited discordant patterns that were not evaluable without clinical correlation.

**Summary/Conclusion**

In summary, ctDNA analysis enables sensitive baseline genotyping and longitudinal MRD monitoring in MM and captures most SVs and driver mutations. In selected cases, ctDNA reveals clinically relevant alterations not detected in BM. Longitudinal ctDNA dynamics largely mirror NGF-defined MRD kinetics, supporting its value as a minimally invasive monitoring tool. Limited sensitivity at very low disease levels underscores the need for optimized algorithms and validated ctDNA thresholds.

**Copyright Information:** (Online) ISSN: 2572-9241
©2026 The Author(s). HemaSphere is published by John Wiley & Sons Ltd on behalf of European Hematology Association. This is an open access Abstract Book distributed under the Attribution-NonCommercial-NoDerivs (CC BY-NC-ND), which allows third parties to download the articles and share them with others as long as they credit the author and the Abstract Book, but they cannot change the content in any way or use them commercially.
**Abstract Book Citations:** Authors, Title, HemaSphere, 2026;10:(S1):pages. The abstract book DOIs can be found on the “Author Information” page.
**Disclaimer:** Articles published in the journal HemaSphere exclusively reflect the opinions of the authors. The authors are responsible for all content in their abstracts including accuracy of the facts, statements, citing resources, etc.

### (S191) TARGETING MYELOID IMMUNE CHECKPOINTS TO ENHANCE ANTI-MULTIPLE MYELOMA IMMUNITY

**Topic:** 13. Myeloma and other monoclonal gammopathies - Biology & translational research

Marcello Turi*[1], Pietro Folino[2], Francesca Barello[1], Tallya MacIel[2], Selma Cifric[2], Paola Rampa[1], Mattia Furlan[1], Mehmet Samur[2], Giovanni De Nola[3], Rita Starace[1], Vanessa Favasuli[1], Alessandro Salatino[2], Sofia Pasparaki[2], Carlo Campa[4], Delaney Vinaixa[2], Cole Clericuzio[2], Priya Thuran[2], Megan Johnstone[2], Francesca Bonello[1], Umberto Vitolo[1], Cirino Botta[5], Abigail Lytton-Jean[3], Eugenio Morelli[1], Nikhil Munshi[2], Kenneth Anderson[2], Annamaria Gulla[1]

*[1]Istituto di Candiolo, Candiolo, Italy;[2]Dana-Farber Cancer Institute, Medical Oncology, Boston, United States of America;[3]Koch Institute For Integrative Cancer Research at Mit, Cambridge, United States of America; [4]Italian Institute for Genomic Medicine, Candiolo, Italy; [5]University of Palermo, 6Department of Health Promotion, Mother and Child Care, Internal Medicine and Medical Specialties, Palermo, Italy;*

### Background

Cancer immunotherapies have improved outcomes in multiple myeloma (MM), yet long-term efficacy is limited by immune evasion. A key mechanism involves inhibitory phagocytosis checkpoints (IPCs), which prevent antigen-presenting cells (APC)-mediated phagocytosis of cancer cells. This pathway is relevant for the immune activity of standard anti-MM agents such as bortezomib (BTZ), as inducer of immunogenic cell death (ICD), and the monoclonal antibody daratumumab (DARA). Yet, the role of IPCs in MM remains poorly understood.

### Aims

To systematically characterize IPC axes in MM patients and define their functional and translational relevance as immunotherapeutic targets.

### Methods

Bone marrow aspirates from patients with MGUS, smoldering and newly diagnosed MM (n=34) and healthy donors (HD) (n=5) were analyzed by multiparametric flow cytometry using a 9- and 16-marker panels for MM cells and APCs, respectively, followed by a semi-automated computational pipeline. Single-cell RNA sequencing (scRNA-seq) data from 90 MM and 10 HD were interrogated. Functional validation included B2M knockout (KO) with re-expression of a LILRB1-binding-deficient mutant ($B2M^{MUT}$, harboring 16 missense mutations) in three MM cell lines. Yeast two-hybrid assays confirmed direct interaction between B2M and LILRB1, absent for $B2M^{MUT}$. Additional assays included phagocytosis following BTZ or DARA, APC maturation, LILRB1 knockdown in APCs, T cell-mediated MM cell lysis, correlative light and electron microscopy (CLEM) and transmission electron microscopy (TEM) to assess B2M internalization and microtubule dynamics. In vivo studies used a syngeneic murine model in which MM cells express a mutant B2m unable to bind Lilrb1 ortholog.

**Copyright Information:** (Online) ISSN: 2572-9241
©2026 The Author(s). HemaSphere is published by John Wiley & Sons Ltd on behalf of European Hematology Association. This is an open access Abstract Book distributed under the Attribution-NonCommercial-NoDerivs (CC BY-NC-ND), which allows third parties to download the articles and share them with others as long as they credit the author and the Abstract Book, but they cannot change the content in any way or use them commercially.
**Abstract Book Citations:** Authors, Title, HemaSphere, 2026;10:(S1):pages. The abstract book DOIs can be found on the “Author Information” page.
**Disclaimer:** Articles published in the journal HemaSphere exclusively reflect the opinions of the authors. The authors are responsible for all content in their abstracts including accuracy of the facts, statements, citing resources, etc.

### Results

The B2M/LILRB1 axis emerged as the dominantly expressed IPC. Computational analysis identified high LILRB1 expression in conventional DC1 and DC2, but not plasmacytoid DCs. scRNA-seq confirmed higher LILRB1 expression in DCs from MM patients versus HD. LILRB1 ligand, B2M, was consistently expressed in MM cells.

$B2M^{KO}$ and $B2M^{MUT}$ MM cells showed >2-fold increased phagocytosis by DCs and macrophages after BTZ or DARA ($p<0.01$). LILRB1 knockdown in APCs enhanced phagocytosis following BTZ ($p<0.05$).

As MM cells secrete soluble B2M (s-B2M), conditioned media from B2M WT, $B2M^{KO}$, and $B2M^{MUT}$ lines were analyzed. s-B2M was detected in WT supernatants, absent in KO, and present as s-$B2M^{MUT}$ in $B2M^{MUT}$ cells. Exposure of DCs to s-B2M WT, but not s-$B2M^{MUT}$, reduced phagocytosis of $B2M^{KO}$ cells after BTZ or DARA ($p<0.05$). Recombinant B2M activated LILRB1 signaling via p-SHP1.

APCs internalized s-B2M in a LILRB1-dependent manner, localizing to perinuclear vesicles near the MTOC (Microtubule Organizing Center), resulting in an increased microtubule stability (higher acetylated/tyrosinated tubulin ratio) and impaired APC maturation.

*In vivo*, mice injected with $B2m^{mut}$ 5TGM1 cells showed reduced tumor engraftment and growth versus WT, with increased $CD3^{+}$ and $CD8^{+}$ T-cell infiltration.

### Summary/Conclusion

We identify the B2M/LILRB1 axis as a dominant IPC in MM that impairs APC maturation and function via membrane-bound and soluble B2M. Disruption of this pathway enhances phagocytosis, restores T-cell activation, and reduces tumor growth in vivo. Targeting B2M/LILRB1 represents a novel strategy to restore immune competence and improve therapeutic responses in MM.

**Copyright Information:** (Online) ISSN: 2572-9241
©2026 The Author(s). HemaSphere is published by John Wiley & Sons Ltd on behalf of European Hematology Association. This is an open access Abstract Book distributed under the Attribution-NonCommercial-NoDerivs (CC BY-NC-ND), which allows third parties to download the articles and share them with others as long as they credit the author and the Abstract Book, but they cannot change the content in any way or use them commercially.
**Abstract Book Citations:** Authors, Title, HemaSphere, 2026;10:(S1):pages. The abstract book DOIs can be found on the "Author Information" page.
**Disclaimer:** Articles published in the journal HemaSphere exclusively reflect the opinions of the authors. The authors are responsible for all content in their abstracts including accuracy of the facts, statements, citing resources, etc.

### (S192) LILRB4 DRIVES IMMUNOSUPPRESSIVE MYELOID CELL FORMATION VIA THE S100A4-CCL3 AXIS TO PROMOTE IMMUNE EVASION IN MULTIPLE MYELOMA

**Topic:** 13. Myeloma and other monoclonal gammopathies - Biology & translational research

Lanting Liu[1], Xiyue Sun[1], Yijie Wang[1], Runchun Huang[1], Gang An[1], Lugui Qiu[1], Mu Hao*[1]

*[1]State Key Laboratory of Experimental Hematology, National Clinical Research Center for Blood Diseases, Haihe Laboratory of Cell Ecosystem, Institute of Hematology & Blood Diseases Hospital, Chinese Academy of Medical Sciences & Peking Union Medical College, Tianjin, China;*

### Background

Immunotherapies such as CAR-T have improved outcomes for high-risk multiple myeloma (MM) patients; however, MM remains incurable, largely due to the establishment of a tumor-driven immunosuppressive microenvironment. Our previous studies demonstrated that LILRB4 is aberrantly expressed in MM cells and promotes the accumulation of myeloid-derived suppressor cells (MDSCs) and erythroid-derived myeloid cells (EDMCs), resulting in impaired T cell–mediated anti-tumor immunity.

### Aims

To elucidate the molecular mechanisms by which tumor-intrinsic LILRB4 signaling drives immunosuppressive myeloid cell formation in MM.

### Methods

Single-cell RNA sequencing was performed on bone marrow samples from MM patients. LILRB4-overexpressing and knockout MM cell lines, as well as murine models, were established. Multi-omics approaches, including proteomics, RNA-seq, ATAC-seq, and functional assays, were applied to define downstream signaling pathways and cytokine programs.

### Results

In LILRB4-overexpressing MM cells, the phosphorylation level of downstream SHP2 was increased, accompanied by sustained activation of the NF-κB and ERK pathways (Fig.A). Notably, both the mRNA transcription level and protein expression of S100A4 were significantly upregulated in LILRB4-overexpressing MM cells (Fig.B). MMRF-COMPASS data revealed a positive correlation between S100A4 and LILRB4 expression (Fig.C). ATAC-seq results also confirmed that the level of chromatin accessibility was significantly increased following LILRB4 overexpression. The motif of AP-1, a transcription factor that regulates S100A4, was significantly enriched in the open peaks (Fig.D). Furthermore, studies have shown that S100A4 can effectively drive the expression of multiple chemokines (CCL3, GM-CSF) through transcription factors such as NF-κB, AP-1, and STAT3. Through *in vivo* and *in vitro* studies, we show that $CD45^+$ EPCs in the myeloma microenvironment lose their erythroid maturation potential and instead undergo trans-differentiation into EDMCs, driven by high levels of CCL3 secreted by $LILRB4^+$ MM cells (Fig.E). In the 5T33-MM mouse model, the LILRB4-overexpressing group exhibited increased proportions of M-MDSCs, EDMCs, and Tregs, along with a decreased proportion of $CD8^+$ T cells and aggravated anemia, compared to the EV group (Fig.F). In vivo experiments using NSG mice demonstrated that treatment with a LILRB4-targeted drug (LILRB4-PROTAC) significantly reduced the tumor burden in the mice (Fig.G).

**Copyright Information:** (Online) ISSN: 2572-9241
©2026 The Author(s). HemaSphere is published by John Wiley & Sons Ltd on behalf of European Hematology Association. This is an open access Abstract Book distributed under the Attribution-NonCommercial-NoDerivs (CC BY-NC-ND), which allows third parties to download the articles and share them with others as long as they credit the author and the Abstract Book, but they cannot change the content in any way or use them commercially.
**Abstract Book Citations:** Authors, Title, HemaSphere, 2026;10:(S1):pages. The abstract book DOIs can be found on the "Author Information" page.
**Disclaimer:** Articles published in the journal HemaSphere exclusively reflect the opinions of the authors. The authors are responsible for all content in their abstracts including accuracy of the facts, statements, citing resources, etc.

### Summary/Conclusion

These findings identify the LILRB4–S100A4–CCL3 axis as a tumor-intrinsic inflammatory circuit that programs an immunosuppressive myeloid microenvironment in MM. Targeting this pathway restores anti-tumor immunity and represents a promising therapeutic strategy to enhance immunotherapy efficacy.

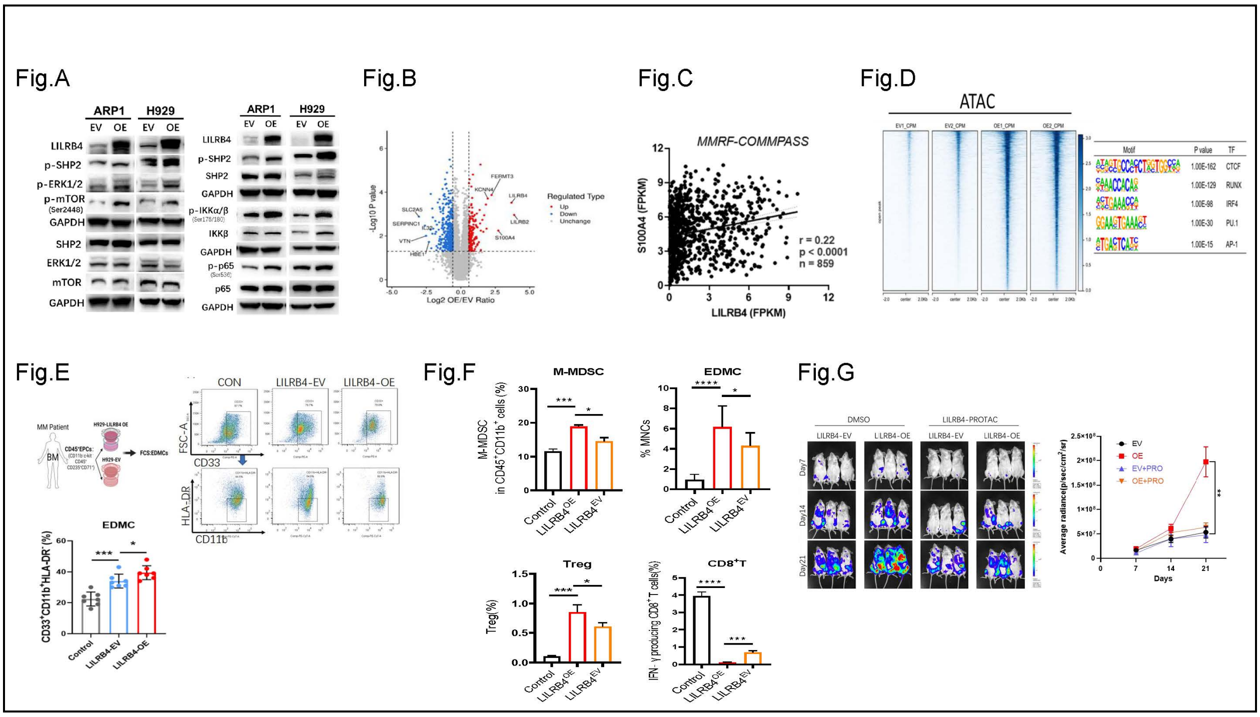

**Copyright Information:** (Online) ISSN: 2572-9241
©2026 The Author(s). HemaSphere is published by John Wiley & Sons Ltd on behalf of European Hematology Association. This is an open access Abstract Book distributed under the Attribution-NonCommercial-NoDerivs (CC BY-NC-ND), which allows third parties to download the articles and share them with others as long as they credit the author and the Abstract Book, but they cannot change the content in any way or use them commercially.
**Abstract Book Citations:** Authors, Title, HemaSphere, 2026;10:(S1):pages. The abstract book DOIs can be found on the "Author Information" page.
**Disclaimer:** Articles published in the journal HemaSphere exclusively reflect the opinions of the authors. The authors are responsible for all content in their abstracts including accuracy of the facts, statements, citing resources, etc.

### (S193) VIRMA EPITRANSCRIPTOMICALLY REGULATES CELL PROLIFERATION, APOPTOSIS, AND MITOCHONDRIAL FUNCTION VIA M6A IN MULTIPLE MYELOMA

**Topic:** 13. Myeloma and other monoclonal gammopathies - Biology & translational research

Estibaliz Urizar-Compains*[1], Naroa Barrena[2], Theodore Maximilian Nelson[3], Leire Garate[1, 4, 5], Estibaliz Miranda[1, 4], Juan Antonio Romero Torrecilla[6], Luis Vitores Valcárcel[2], Marta Kulis[4, 7], Francisco Planes[2], Maria José Blanco Prieto[6], Paula Rodríguez-Otero[1, 5], Iñaki Martín-Subero[4, 7, 8], Christopher E Mason[3, 9], Edurne San José Enériz[1, 4], Felipe Prosper[1, 4, 5], Xabier Agirre[1, 4]

*[1]Hemato-Oncology Program, Center for Applied Medical Research (CIMA), Instituto de Investigaciones Sanitarias de Navarra (IdiSNA), Cancer Center Clinica Universidad de Navarra (CCUN), Pamplona, Spain; [2]TECNUN School of Engineering, Universidad de Navarra, San Sebastián, Spain; [3]Department of Systems and Computational Biomedicine, Weill Cornell Medicine, New York, United States of America; [4]Centro de Investigación Biomédica en Red Cáncer (CIBERONC), Madrid, Spain; [5]Hematology and Cell Therapy Service. Clínica Universidad de Navarra, IdiSNA, CCUN, Pamplona, Spain;[6]Department of Pharmaceutical Sciences, Universidad de Navarra, IdiSNA, Pamplona, Spain; [7]Fundació Clínic per la Recerca Biomèdica-Institut d'Investigacions Biomèdiques August Pi i Sunyer (FCRB-IDIBAPS), Barcelona, Spain; [8]Institució catalana de Recerca i Estudis Avançats (ICREA), Barcelona, Spain; [9]The HRH Prince Alwaleed Bin Talal Bin Abdulaziz Alsaud Institute for Computational Biomedicine, Weill Cornell Medicine, New York, United States of America;*

### Background

Multiple Myeloma (MM) is a hematological neoplasm characterized by the clonal expansion of malignant plasma cells (PCs) within the bone marrow. Despite clinical advances, the majority of patients eventually experience disease relapse. Epitranscriptomic alterations, such as post-transcriptional RNA modifications, have emerged as key mechanisms in tumor development. However, their role in MM remains poorly understood.

### Aims

We investigated the role of the epitranscriptomic modification N6-methyladenosine (m6A) and its regulatory genes in the prognosis and development of MM.

### Methods

We analyzed m6A levels (ELISA) and 20 regulatory genes using RNA-seq (642 patients, CoMMPass IA21), ATAC-seq, and ChIP-seq in PCs from healthy donors and MM patients. VIRMA inhibition was performed via CRISPR-Cas9 in MM.1S and KMS-11 cell lines. Phenotypic impact was assessed through Western blot, m6A-ELISA, NF-kb-ELISA, flow cytometry, and Seahorse (mitochondrial function). Direct RNA nanopore sequencing characterized the epitranscriptome. Finally, lipid nanoparticles (LNPs) encapsulating siRNA targeting VIRMA were developed and tested *in vitro* and *in vivo* (xenograft models).

**Copyright Information:** (Online) ISSN: 2572-9241
©2026 The Author(s). HemaSphere is published by John Wiley & Sons Ltd on behalf of European Hematology Association. This is an open access Abstract Book distributed under the Attribution-NonCommercial-NoDerivs (CC BY-NC-ND), which allows third parties to download the articles and share them with others as long as they credit the author and the Abstract Book, but they cannot change the content in any way or use them commercially.
**Abstract Book Citations:** Authors, Title, HemaSphere, 2026;10:(S1):pages. The abstract book DOIs can be found on the “Author Information” page.
**Disclaimer:** Articles published in the journal HemaSphere exclusively reflect the opinions of the authors. The authors are responsible for all content in their abstracts including accuracy of the facts, statements, citing resources, etc.

**Results**

Global m6A levels were significantly elevated from premalignant stages through overt MM, accompanied by the overexpression of 18 of m6A-genes. This upregulation was epigenetically driven, evidenced by increased chromatin activation (H3K27Ac) at promoter regions. Integrative analysis identified an m6A-gene expression signature (ESMEG) that independently predicts inferior clinical outcomes beyond established genetic risk factors, whose high score correlated with proliferative pathways.
Among regulators, DepMap-Achilles identified *VIRMA* as essential in MM. VIRMA inhibition reduced cell viability and global m6A levels *in vitro*. Notably, *in vivo* studies revealed a marked impairment of MM tumor growth, demonstrating its essentiality in MM. Direct RNA sequencing confirmed a specific reduction in m6A stoichiometry affecting 1,345 sites across 1,001 genes, without affecting other modifications. Gene Ontology analysis linked these targets to cell cycle, apoptosis, and mitochondrial functions. Specifically, functional studies validated that VIRMA inhibition led to G1 cell cycle arrest, reduced NF-κB signaling, and impaired mitochondrial respiration, supporting its key role in MM cell survival.
Finally, to evaluate the potential of VIRMA as a therapeutic target, we developed LNPs encapsulating a VIRMA-targeting siRNA (LNP-siVIRMA). Treatment of MM cell lines with LNP-siVIRMA significantly inhibited MM cell proliferation *in vitro*. Importantly, *in vivo* administration led to a pronounced reduction in tumor growth, highlighting the therapeutic potential of targeting VIRMA in MM.

**Summary/Conclusion**

In summary, our findings indicate that increased m6A and upregulation of its regulatory genes are associated with poor prognosis in MM. *VIRMA* is critical for MM cell survival through regulation of the cell cycle, apoptosis, and mitochondrial function. Its inhibition via LNP-siVIRMA represents a promising epitranscriptomic-based therapeutic strategy to improve the response of MM patients.

**Copyright Information:** (Online) ISSN: 2572-9241
©2026 The Author(s). HemaSphere is published by John Wiley & Sons Ltd on behalf of European Hematology Association. This is an open access Abstract Book distributed under the Attribution-NonCommercial-NoDerivs (CC BY-NC-ND), which allows third parties to download the articles and share them with others as long as they credit the author and the Abstract Book, but they cannot change the content in any way or use them commercially.
**Abstract Book Citations:** Authors, Title, HemaSphere, 2026;10:(S1):pages. The abstract book DOIs can be found on the "Author Information" page.
**Disclaimer:** Articles published in the journal HemaSphere exclusively reflect the opinions of the authors. The authors are responsible for all content in their abstracts including accuracy of the facts, statements, citing resources, etc.

### (S194) INTEGRATED PHENOTYPIC CLASSIFICATION OF MULTIPLE MYELOMA USING MULTIMODAL SPATIAL TUMOR BURDEN AND SOLUBLE BCMA: REFINING R-ISS AND CHARACTERIZING BIOLOGICAL DISCORDANCE

**Topic:** 14. Myeloma and other monoclonal gammopathies - Clinical

Masanori Toho*[1], Rikako Tabata[1], Atsushi Uehara[1], Hajime Sakuma[1], Kentaro Narita[1], Kosuke Shima[1], Daichi Terunuma[1], Kazuki Nagao[1], Naoya Fukuda[1], Masami Takeuchi[1], Daisuke Ikeda[1, 2], Hiroyuki Takamatsu[3], Kosei Matsue[1]

*[1]Kameda Medical Center, Kamogawa, Japan; [2]Okayama University Hospital, Okayama, Japan; [3]Kanazawa University, Kanazawa, Japan;*

### Background

Current staging for multiple myeloma (MM) often overlooks the incongruence between spatial tumor distribution and biological activity. While imaging (DWI-MRI, PET-CT) captures localized masses, soluble BCMA (sBCMA) has emerged as a robust serum biomarker reflecting total viable plasma cell mass and metabolic turnover. We hypothesized that integrating spatial burden with sBCMA would resolve the "mismatch" between radiographic findings and clinical aggressiveness, providing superior disease stratification.

### Aims

To establish a novel prognostic framework by integrating multi-modal spatial parameters with sBCMA to characterize phenotypic heterogeneity in newly diagnosed MM.

### Methods

Among 352 screened patients, a core cohort of 203 with complete multi-modal datasets (TMTV, TDV, BMPC%) was analyzed. PCA was performed on this cohort to consolidate spatial parameters into a unified 'Spatial Burden Index'." Independently, sBCMA was utilized as a "Biological Activity Index." Patients were categorized into four phenotypes based on these axes: Quiescent (Low Burden/Low Activity), Macro-Dominant (High Burden/Low Activity), Bio-Dominant (Low Burden/High Activity), and Dual-High (High Burden/High Activity).

### Results

PCA confirmed the orthogonality of spatial burden and biological activity, explaining 50.3% and 22.7% of the total variance, respectively. This relationship allowed for the identification of four distinct phenotypes: Quiescent (Q, 32%), Macro-Dominant (M, 26%), Bio-Dominant (B, 16%), and Dual-High (D, 25%). First, these phenotypes showed clear prognostic separation, with the Dual-High group exhibiting the worst outcomes and the Quiescent/Macro-Dominant groups showing favorable surviva Notably, the Macro-Dominant group exhibited a significant "radiographic-biological mismatch"; despite a massive median TDV (411.0 mL), their low sBCMA/BMPC ratio (5.58) and low CTC count (0.00099) provided physical evidence of "Spatial Sequestration." In contrast, the Bio-Dominant group represented occult aggressiveness with high cellular activity (sBCMA/BMPC ratio 23.71) and CTC dissemination (0.00244), leading to frequent early relapse (35% at 24 months) despite minimal radiographic burden. Furthermore, the spatial phenotype significantly modified the prognostic impact of cytogenetic risks. In the B group, Gain1q functioned as a critical determinant of mortality (HR: 28.6, p=0.002), whereas its impact was clinically neutralized in the M group (HR: 1.22, p=0.81). Finally, multivariate analysis confirmed these phenotypes as independent predictors for OS (HR 3.12, p=0.004), outperforming traditional R-ISS categories. Our framework successfully re-classified a significant proportion of R-ISS stage II patients into distinct risk groups with divergent outcomes.

**Copyright Information:** (Online) ISSN: 2572-9241
©2026 The Author(s). HemaSphere is published by John Wiley & Sons Ltd on behalf of European Hematology Association. This is an open access Abstract Book distributed under the Attribution-NonCommercial-NoDerivs (CC BY-NC-ND), which allows third parties to download the articles and share them with others as long as they credit the author and the Abstract Book, but they cannot change the content in any way or use them commercially.
**Abstract Book Citations:** Authors, Title, HemaSphere, 2026;10:(S1):pages. The abstract book DOIs can be found on the "Author Information" page.
**Disclaimer:** Articles published in the journal HemaSphere exclusively reflect the opinions of the authors. The authors are responsible for all content in their abstracts including accuracy of the facts, statements, citing resources, etc.

**Summary/Conclusion**

Integrated assessment reveals that spatial burden does not invariably correlate with biological aggression. High biological activity defines a high-risk state characterized by systemic dissemination, even when radiographic burden is minimal. Conversely, spatial sequestration may mitigate the impact of adverse cytogenetics in bulky disease. This dual-axis framework provides a more robust biological basis for precision medicine in multiple myeloma.

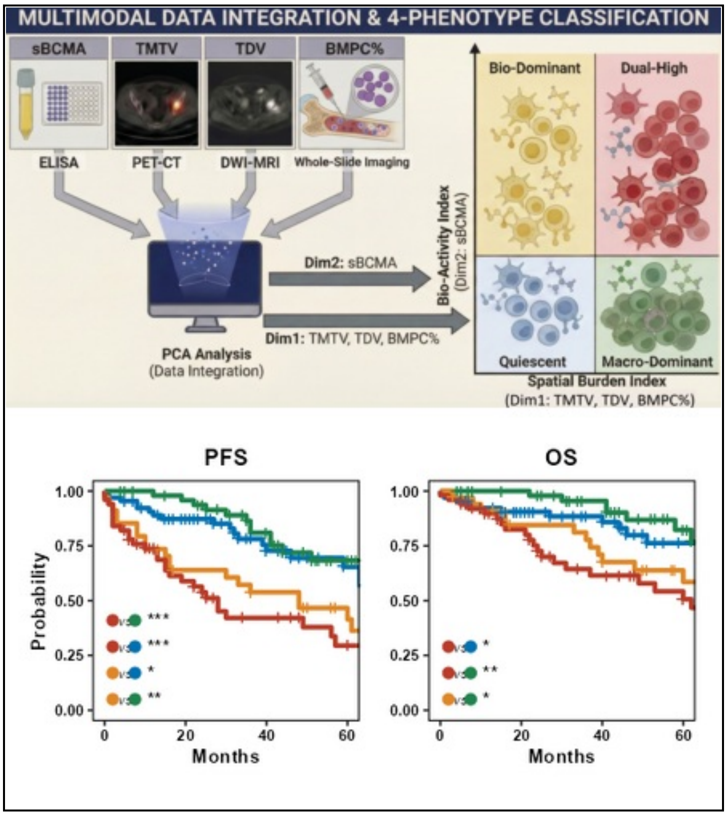

Copyright Information: (Online) ISSN: 2572-9241
©2026 The Author(s). HemaSphere is published by John Wiley & Sons Ltd on behalf of European Hematology Association. This is an open access Abstract Book distributed under the Attribution-NonCommercial-NoDerivs (CC BY-NC-ND), which allows third parties to download the articles and share them with others as long as they credit the author and the Abstract Book, but they cannot change the content in any way or use them commercially.
Abstract Book Citations: Authors, Title, HemaSphere, 2026;10:(S1):pages. The abstract book DOIs can be found on the "Author Information" page.
Disclaimer: Articles published in the journal HemaSphere exclusively reflect the opinions of the authors. The authors are responsible for all content in their abstracts including accuracy of the facts, statements, citing resources, etc.

### (S195) MAJESTEC-9: A PHASE 3 STUDY OF TECLISTAMAB MONOTHERAPY VS POMALIDOMIDE/BORTEZOMIB/DEXAMETHASONE OR CARFILZOMIB/DEXAMETHASONE (PVD/KD) IN PATIENTS (PTS) WITH RELAPSED REFRACTORY MULTIPLE MYELOMA (RRMM)

**Topic:** 14. Myeloma and other monoclonal gammopathies - Clinical

Cyrille Touzeau*[1], Roberto Mina[2, 3], Vania Hungria[4], C. Ola Landgren[5], Divaya Bhutani[6], Wenming Chen[7], Swarup Kumar[8], Chakra P. Chaulagain[9], Meletios A. Dimopoulos[10, 11], Nizar J Bahlis[12], Senem Maral[13], Niels W C J van de Donk[14], Khalid Saja[15], Raphael Teipel[16], Miki Ando[17], Dai Wang[18], Susan Wroblewski[19], Veronique Vanquickelberghe[20], Priya Shah[21], Katherine Chastain[18], Robin Carson[19], Hang Quach[22]

*[1]Department of Hematology, University Hospital of Nantes, Nantes, France; [2]Winship Cancer Institute, Emory University, Atlanta, GA, United States of America; [3]Division of Hematology, Department of Molecular Biotechnology and Health Sciences, AOU Città della Salute e della Scienza di Torino, University of Torino, Torino, Italy; [4]Clínica Médica São Germano, São Paulo, Brazil; [5]Myeloma Division, Sylvester Comprehensive Cancer Center, University of Miami, Miami, FL, United States of America; [6]Division of Hematology/Oncology, Columbia University Medical Center, New York, NY, United States of America; [7]Department of Hematology, Beijing Chao-Yang Hospital, Capital Medical University, Beijing, China; [8]Department of Hematology and Oncology, University of Connecticut Health, Farmington, CT, United States of America; [9]Department of Hematology and Oncology, Myeloma and Amyloidosis Program, Cleveland Clinic Florida, Weston, FL, United States of America; [10]Department of Clinical Therapeutics, National and Kapodistrian University of Athens, School of Medicine, Athens, Greece; [11]Department of Medicine, Korea University, Seoul, South Korea; [12]Arnie Charbonneau Cancer Research Institute, University of Calgary, Calgary, AB, Canada; [13]Department of Hematology, Faculty of Medicine, Istanbul Medipol University, Istanbul, Türkiye; [14]Department of Hematology, Amsterdam University Medical Center, Cancer Center Amsterdam Vrije Universiteit Amsterdam, Amsterdam, The Netherlands; [15]Hematology Department, Colchester General Hospital, East Suffolk and North Essex NHS Foundation Trust, Colchester, United Kingdom; [16]Department of Internal Medicine I, University Hospital Dresden, Technical University Dresden, Dresden, Germany; [17]Department of Hematology, Juntendo University School of Medicine, Tokyo, Japan; [18]Johnson & Johnson, Raritan, NJ, United States of America; [19]Johnson & Johnson, Spring House, PA, United States of America; [20]Johnson & Johnson, Beerse, Belgium; [21]Johnson & Johnson, High Wycombe, United Kingdom; [22]Clinical Haematology Service, St. Vincent's Hospital Melbourne, University of Melbourne, Melbourne, VIC, Australia;*

### Background

Triplet and quadruplet regimens have greatly improved outcomes in newly diagnosed multiple myeloma, yet a high unmet need remains for more effective, broadly accessible treatments for anti-CD38- and lenalidomide- (Len) exposed/refractory RRMM. Teclistamab (Tec) is the most widely used BCMA×CD3 BsAb in heavily pretreated RRMM, with improved outcomes in earlier lines of therapy (LOTs). In MajesTEC-3, the synergistic Tec-daratumumab combination significantly improved progression-free and overall survival (PFS/OS) as early as first relapse. MajesTEC-9 (NCT05572515) is the first phase 3 study of Tec monotherapy in pts with 1–3 prior LOTs.

### Aims

We report efficacy and safety findings from the interim analysis of MajeTEC-9 study.

**Copyright Information:** (Online) ISSN: 2572-9241
©2026 The Author(s). HemaSphere is published by John Wiley & Sons Ltd on behalf of European Hematology Association. This is an open access Abstract Book distributed under the Attribution-NonCommercial-NoDerivs (CC BY-NC-ND), which allows third parties to download the articles and share them with others as long as they credit the author and the Abstract Book, but they cannot change the content in any way or use them commercially.
**Abstract Book Citations:** Authors, Title, HemaSphere, 2026;10:(S1):pages. The abstract book DOIs can be found on the "Author Information" page.
**Disclaimer:** Articles published in the journal HemaSphere exclusively reflect the opinions of the authors. The authors are responsible for all content in their abstracts including accuracy of the facts, statements, citing resources, etc.

**Methods**

Pts with RRMM and 1–3 prior LOTs including anti-CD38 and Len were randomized 1:1 to receive 28-day cycles (C) of Tec (C1–2: 2 step-up doses then 1.5 mg/kg QW; C3–6: 3 mg/kg Q2W or Q4W depending on response; C7+: 3 mg/kg Q4W) or investigator's choice of PVd/Kd (21/28-day C). Primary endpoint was PFS by IRC; secondary endpoints included OS, complete response or better (≥CR), and safety.

**Results**

593 pts were randomized (Tec, n=296; PVd/Kd, n=297). Median (range) age: 70 (34–86) yrs; number of prior LOTs: 2 (1–3); Len refractory: 80%; anti-CD38 refractory: 85%; refractory to last LOT: 92%. With 17.3-mo median follow-up, Tec significantly improved PFS vs PVd/Kd (HR, 0.29; 95% CI, 0.23–0.38; *P*<0.0001); mPFS: NR vs 8.2 mo; 18-mo PFS rate: 69.8% vs 26.9%. OS was significantly improved with Tec vs PVd/Kd (HR, 0.60; 95% CI, 0.43–0.83; *P*=0.0020). Of 174 PVd/Kd pts who received subsequent treatment, 68.4% received BsAb or CAR-T. PFS favored Tec across all prespecified subgroups, including anti-CD38-refractory pts and Len-refractory pts. ≥CR rate was significantly higher with Tec vs PVd/Kd (65.9% vs 16.8%; OR, 10.42; 95% CI, 6.89–15.76; *P*<0.0001).
At data cutoff, 65.3% of pts remained on Tec vs 24.0% on PVd/Kd (safety set: Tec, n=291; PVd/Kd, n=283). TEAEs were similar for Tec and PVd/Kd (99.7% vs 97.9%). Grade (Gr) 3/4 TEAEs (84.9% vs 76.3%) and Gr 5 TEAEs (6.5% vs 3.5%) were higher with Tec vs PVd/Kd, noting the median treatment duration was nearly doubled with Tec (13.1 vs 7.0 mo). Treatment discontinuation due to TEAEs was lower with Tec vs PVd/Kd (10.7% vs 13.1%). Gr 3/4 infections were higher with Tec vs PVd/Kd (41.6% vs 29.0%). Any-onset Gr ≥3 infections decreased over time. CRS occurred in 66.0% of pts with Tec (Gr 1/2: 48.8%/16.5%) and ICANS in 4.1% (Gr 1/2: 2.4%/1.4%).

**Summary/Conclusion**

Tec monotherapy significantly improved PFS/OS vs standard of care (SoC) in this high unmet need population. CRS and infections were managed with established protocols. Results of phase 3 studies support Tec-based regimens as a SoC as early as second line, across all treatment settings and regardless of prior anti-CD38/Len exposure.

© 2026 American Society of Clinical Oncology, Inc. Reused with permission. This abstract was accepted and previously presented at the 2026 ASCO Annual Meeting. All rights reserved.

**Copyright Information:** (Online) ISSN: 2572-9241
©2026 The Author(s). HemaSphere is published by John Wiley & Sons Ltd on behalf of European Hematology Association. This is an open access Abstract Book distributed under the Attribution-NonCommercial-NoDerivs (CC BY-NC-ND), which allows third parties to download the articles and share them with others as long as they credit the author and the Abstract Book, but they cannot change the content in any way or use them commercially.
**Abstract Book Citations:** Authors, Title, HemaSphere, 2026;10:(S1):pages. The abstract book DOIs can be found on the "Author Information" page.
**Disclaimer:** Articles published in the journal HemaSphere exclusively reflect the opinions of the authors. The authors are responsible for all content in their abstracts including accuracy of the facts, statements, citing resources, etc.

### (S196) DARATUMUMAB PLUS BORTEZOMIB, LENALIDOMIDE, AND DEXAMETHASONE IN PATIENTS WITH NEWLY DIAGNOSED MULTIPLE MYELOMA: FINAL ANALYSIS OF TRANSPLANT-INELIGIBLE PATIENTS IN THE PHASE 3 CEPHEUS STUDY

**Topic:** 14. Myeloma and other monoclonal gammopathies - Clinical

Saad Z Usmani*[1], Thierry Facon[2], Vania Hungria[3], Nizar J Bahlis[4], Christopher Venner[5], Marc Braunstein[6], Ludek Pour[7], Josep Maria Marti Tutusaus[8], Supratik Basu[9], Yael Cohen[10], Kenshi Suzuki[11], Cyrille Hulin[12], Sebastian Grosicki[13], Meral Beksac[14], Angelo Maiolino[15], Jaclyn Stanziola[16], Lorena Lopez-Masi[16], Matteo Loi[17], Melissa Rowe[18], Sonja Zweegman[19]

*[1]Memorial Sloan Kettering Cancer Center, New York, United States of America;[2]University of Lille, CHU Lille, Service des Maladies du Sang, Lille, France; [3]Clínica Médica São Germano, São Paulo, Brazil; [4]Arnie Charbonneau Cancer Research Institute, University of Calgary, Calgary, Canada; [5]Cross Cancer Institute, University of Alberta, Edmonton, AB, Canada; BC Cancer–Vancouver Centre, University of British Columbia, Vancouver, Canada; [6]Perlmutter Cancer Center, NYU Langone Health, New York, United States of America; [7]University Hospital Brno, Brno, Czechia; [8]Hospital Universitario Mútua de Terrassa, Terrassa, Spain; [9]Royal Wolverhampton NHS Trust and University of Wolverhampton, CRN West Midlands, NIHR, Wolverhampton, United Kingdom; [10]Tel Aviv Sourasky (Ichilov) Medical Center, Tel Aviv, Israel; Gray Faculty of Medical and Health Sciences, Tel Aviv University, Tel Aviv, Israel; [11]Japanese Red Cross Medical Center, Tokyo, Japan; [12]Hôpital Haut Lévêque, University Hospital, Pessac, France; [13]School of Public Health, Medical University of Silesia, Katowice, Poland; [14]Istinye University, Ankara Liv Hospital, Ankara, Türkiye; [15]Instituto Americas de Ensino, Pesquisa e Inovação, Rio de Janeiro, Brazil; Universidade Federal do Rio de Janeiro, Rio de Janeiro, Brazil; [16]Johnson & Johnson, Raritan, United States of America; [17]Johnson & Johnson, Leiden, The Netherlands; [18]Johnson & Johnson, High Wycombe, United Kingdom; [19]Amsterdam UMC, Vrije Universiteit Amsterdam, Cancer Center Amsterdam, Amsterdam, The Netherlands;*

### Background

The phase 3 CEPHEUS study established that daratumumab plus bortezomib, lenalidomide, and dexamethasone (DVRd) improved overall minimal residual disease (MRD)-negativity (neg) rates and progression-free survival (PFS) vs VRd in patients with transplant-ineligible (TIE) or transplant-deferred newly diagnosed multiple myeloma (NDMM). The first analysis of the DVRd TIE subpopulation at a median follow-up of 58.7 months (mo) showed a complete response or better (≥CR) rate of 80.6% and overall MRD neg rate of 60.4%, with ~70% of patients alive and progression free.

### Aims

This final analysis of the CEPHEUS TIE subpopulation describes a longer median follow-up of 76.0 mo.

### Methods

The primary endpoint of overall MRD neg rate ($10^{-5}$ and ≥CR), and secondary endpoints, including investigator-assessed PFS and ≥CR rate, and sustained MRD neg (confirmed MRD neg ≥12 mo +/- 1 mo apart without MRD positivity in between and ≥CR) were assessed in the TIE population. The study was closed at the final analysis and patients were able to continue their study treatment via post-study access.

**Copyright Information:** (Online) ISSN: 2572-9241
©2026 The Author(s). HemaSphere is published by John Wiley & Sons Ltd on behalf of European Hematology Association. This is an open access Abstract Book distributed under the Attribution-NonCommercial-NoDerivs (CC BY-NC-ND), which allows third parties to download the articles and share them with others as long as they credit the author and the Abstract Book, but they cannot change the content in any way or use them commercially.
**Abstract Book Citations:** Authors, Title, HemaSphere, 2026;10:(S1):pages. The abstract book DOIs can be found on the “Author Information” page.
**Disclaimer:** Articles published in the journal HemaSphere exclusively reflect the opinions of the authors. The authors are responsible for all content in their abstracts including accuracy of the facts, statements, citing resources, etc.

**Results**

Of 395 patients enrolled, 289 patients with TIE NDMM received either DVRd (n=144) or VRd (n=145). The overall MRD neg rate at $10^{-5}$ was 61.1% for DVRd and 40.0% for VRd (odds ratio [OR] 2.35; 95% CI 1.47–3.77; *P*=0.0004) and at $10^{-6}$ was 46.5% for DVRd vs 27.6% for VRd (OR 2.27; 95% CI 1.39–3.71; *P*=0.0010). Sustained MRD neg rate at $10^{-5}$ was 49.3% for DVRd and 29.0% for VRd (OR 2.40; 95% CI 1.47–3.91; *P*=0.0005) and at $10^{-6}$ was 37.5% for DVRd and 16.6% for VRd (OR 3.01; 95% CI 1.73–5.24; *P*<0.0001). Overall ≥CR rate was 80.6% for DVRd and 61.4% for VRd (OR 2.64; 95% CI 1.54–4.51; *P*=0.0003). Median investigator-assessed PFS was not estimable (NE; 95% CI 74.81–NE) for DVRd and was 50.20 mo (95% CI 41.95–62.95) for VRd (hazard ratio [HR] 0.55; 95% CI 0.39–0.78; *P*=0.0007), with 59.3% for DVRd vs 38.3% for VRd alive and progression free at 72 mo. Overall survival favored DVRd over VRd (HR 0.84; 95% CI 0.57–1.24), particularly when censoring for COVID-19, which significantly impacted this study (HR 0.74; 95% CI 0.49–1.12). The treatment effect was consistent across most subgroups (Table).

**Summary/Conclusion**

After median follow-up of over 6 years, the final analysis of the CEPHEUS trial demonstrated that TIE patients treated with DVRd continue to have deep and durable responses compared with those on VRd, which is crucial for patients for whom transplant is not an option. These data continue to reinforce DVRd as a standard of care in the TIE NDMM population.
© 2026 American Society of Clinical Oncology, Inc. Reused with permission. This abstract was accepted and previously presented at the 2026 ASCO Annual Meeting. All rights reserved.

| | MRD neg ($10^{-5}$) rate, % | | | | Median PFS, months | | | |
|---|---|---|---|---|---|---|---|---|
| **Baseline characteristic** | **DVRd** | **VRd** | **OR** | **95% CI** | **DVRd** | **VRd** | **HR** | **95% CI** |
| ISS stage I | 66.0 | 41.7 | 2.72 | 1.20–6.17 | NE | 60.5 | 0.52 | 0.28–0.97 |
| II | 59.3 | 45.6 | 1.73 | 0.82–3.68 | NE | 49.4 | 0.50 | 0.28–0.88 |
| III | 57.5 | 30.0 | 3.16 | 1.26–7.94 | 66.4 | 43.8 | 0.66 | 0.35–1.24 |
| Cytogenetic risk High | 50.0 | 50.0 | 1.00 | 0.28–3.57 | 58.0 | 31.7 | 0.82 | 0.36–1.87 |
| Standard | 63.8 | 39.6 | 2.68 | 1.55–4.66 | NE | 61.6 | 0.58 | 0.38–0.89 |
| ECOG PS 0 | 57.7 | 45.6 | 1.63 | 0.76–3.47 | NE | 59.9 | 0.33 | 0.17–0.64 |
| ≥1 | 63.0 | 36.4 | 2.99 | 1.63–5.48 | 74.8 | 47.2 | 0.70 | 0.46–1.06 |

ECOG PS, Eastern Cooperative Oncology Group performance status; ISS, international staging system.

**Copyright Information:** (Online) ISSN: 2572-9241
©2026 The Author(s). HemaSphere is published by John Wiley & Sons Ltd on behalf of European Hematology Association. This is an open access Abstract Book distributed under the Attribution-NonCommercial-NoDerivs (CC BY-NC-ND), which allows third parties to download the articles and share them with others as long as they credit the author and the Abstract Book, but they cannot change the content in any way or use them commercially.
**Abstract Book Citations:** Authors, Title, HemaSphere, 2026;10:(S1):pages. The abstract book DOIs can be found on the “Author Information” page.
**Disclaimer:** Articles published in the journal HemaSphere exclusively reflect the opinions of the authors. The authors are responsible for all content in their abstracts including accuracy of the facts, statements, citing resources, etc.

### (S197) TECLISTAMAB (TEC) +/- LENALIDOMIDE (LEN) VERSUS LEN ALONE AS MAINTENANCE THERAPY POST-TRANSPLANT IN NEWLY DIAGNOSED MULTIPLE MYELOMA (NDMM): UPDATED SAFETY RUN-IN (SRI) RESULTS FROM EMN30/MAJESTEC-4

**Topic:** 14. Myeloma and other monoclonal gammopathies - Clinical

Niels W. C. J. van de Donk*[1], Tobias Silzle[2, 3], Ivan Špička[4], Sabrin Tahri[5], Inger S. Nijhof[6], Antonietta Pia Falcone[7], Evangelos Terpos[8], Jakub Radocha[9], Roberto Mina[10], Güldane Cengiz Seval[11], Meral Beksaç[1, 11], Cesar Rodriguez[12], Marcelo Pasquini[13], Alfred Garfall[14], Murali Janakiram[15], Michel Delforge[16], Vania Hungria[17], Donna Reece[18], Cyrille Touzeau[19], Yael Cohen[20], Kihyun Kim[21], Dominik Dytfeld[22], Jiri Minarik[23], Irene Strassl[24], Thomas Melchardt[25], Jelena Bila[26], Martin Schreder[27], Janusz Krawczyk[28], Fredrik Schjesvold[29], Caroline Cicin-Sain[3], Christoph Driessen[3], Maria Atta[30], Lugui Qiu[31], Gonzalo Garate[32], Agoston Szabo[33], Roman Hájek[34], Marc-Steffen Raab[35], Silvia Mangiacavalli[36], Cristina Maria Godinho Pires Joao[37], Hermann Einsele[38], Andrew Spencer[39], Mario Boccadoro[40], Eleni Vasalou[41], Jagoda Jasielec[42], Kayleigh Philips[43], Caline Sakabedoyan[44], Maria Krevvata[44], Rebecca Wilson[45], Pieter Sonneveld[46], Elena Zamagni[47]

*[1]Department of Hematology, Amsterdam University Medical Center, Vrije Universiteit Amsterdam, Amsterdam, The Netherlands; [2]Department of Medical Oncology and Hematology, Cantonal Hospital Graubünden, Chur, Switzerland; [3]Department of Medical Oncology and Hematology, Cantonal Hospital St. Gallen, St. Gallen, Switzerland; [4]Charles University and General Hospital, Prague, Czechia; [5]European Myeloma Network,, Rotterdam, The Netherlands; [6]Department of Internal Medicine-Hematology, St Antonius Hospital Nieuwegein, Nieuwegein, The Netherlands; [7]Istituto di Ricovero e Cura a Carattere Scientifico (IRCCS) Casa Sollievo della Sofferenza, Unità di Ematologia, San Giovanni Rotondo, Italy; [8]University of Athens, School of Medicine, Athens, Greece; [9]4th Department of Internal Medicine - Hematology, University Hospital Hradec Kralove and Faculty of Medicine in Hradec Kralove, Charles University, Hradec Kralove, Czechia; [10]Division of Hematology and Medical Oncology, Winship Cancer Institute, Emory University, Atlanta, GA, United States of America; [11]Ankara University Faculty of Medicine, Department of Hematology, Ankara, Türkiye; [12]Icahn School of Medicine at Mount Sinai, New York, NY, United States of America; [13]Center for International Blood and Marrow Transplant Research, Medical College of Wisconsin, Milwaukee, WI, United States of America; [14]Abramson Cancer Center, Perelman School of Medicine, University of Pennsylvania, Philadelphia, PA, United States of America; [15]Department of Hematology, Division of Myeloma, City of Hope National Medical Center, Los Angeles, CA, United States of America; [16]University of Leuven, Leuven, Belgium; [17]Clinica Medica São Germano, São Paulo, Brazil; [18]Princess Margaret Cancer Centre, Toronto, ON, Canada; [19]University Hospital Hôtel-Dieu, Nantes, France; [20]Department of Hematology, Tel-Aviv Sourasky (Ichilov) Medical Center, and Gray Faculty of Medical and Health Sciences, Tel Aviv University, Tel Aviv, Tel-Aviv, Israel; [21]Department of Medicine, Sungkyunkwan University School of Medicine, Samsung Medical Center, Seoul, South Korea; [22]The Department of Hematology and Bone Marrow Transplantation, Poznan University of Medical Sciences, Poznań, Poland; [23]Department of Hematooncology, University Hospital and Palacky University Olomouc, Olomouc, Czechia; [24]Division of Hematology with Stem Cell Transplantation, Hemostaseology and Medical Oncology, Department of Internal Medicine I, Ordensklinikum Linz, Linz, Austria; [25]Paracelsus Medical University, Department of Internal Medicine III With Haematology, Medical Oncology, Haemostaseology, Infectiology and Rheumatology, Salzburg, Austria; [26]Faculty of Medicine, University of Belgrade, University Clinical Centre of Serbia, Belgrade, Serbia; [27]First Department of Medicine, Center for Oncology and Hematology, Klinik Ottakring, Vienna, Austria; [28]Department of Haematology, University Hospital Galway, H91 Galway, Ireland; National University of Ireland, H91 Galway, Ireland; [29]Oslo Myeloma Center, Department of Hematology, and KG Jebsen Center for B-cell Malignancies, University of Oslo, Oslo, Norway; [30]Imperial College Healthcare NHS Trust, Hammersmith Hospital, London, United Kingdom; [31]Department of Lymphoma, Chinese Academy of Medical Sciences, Blood Diseases Hospital (Institute of Hematology), Tianjin, China; [32]Department of Hematology, Hospital Alemán, Buenos Aires, Argentina; [33]Department of Hematology, Copenhagen University Hospital Rigshospitalet, Copenhagen, Denmark; [34]Department of Hematooncology, University Hospital Ostrava and Faculty of Medicine, University of Ostrava, Ostrava, Czechia; [35]Heidelberg Myeloma Center, Department of Medicine V, University Hospital Heidelberg, Heidelberg, Germany; [36]Division of Hematology, Fondazione IRCCS Policlinico San Matteo, Pavia, Italy; [37]Department of Hematology, Champalimaud Centre for the Unknown, Lisboa, Portugal; Nova Medical School, Universidade Nova de Lisboa, Lisboa, Portugal; [38]Department of Internal Medicine II, University Hospital*

**Copyright Information:** (Online) ISSN: 2572-9241
©2026 The Author(s). HemaSphere is published by John Wiley & Sons Ltd on behalf of European Hematology Association. This is an open access Abstract Book distributed under the Attribution-NonCommercial-NoDerivs (CC BY-NC-ND), which allows third parties to download the articles and share them with others as long as they credit the author and the Abstract Book, but they cannot change the content in any way or use them commercially.
**Abstract Book Citations:** Authors, Title, HemaSphere, 2026;10:(S1):pages. The abstract book DOIs can be found on the "Author Information" page.
**Disclaimer:** Articles published in the journal HemaSphere exclusively reflect the opinions of the authors. The authors are responsible for all content in their abstracts including accuracy of the facts, statements, citing resources, etc.

*Würzburg, Würzburg, Germany; [39]Malignant Haematology and Stem Cell Transplantation Service, Alfred Health-Monash University, Melbourne, VIC, Australia; [40]European Myeloma Network, Torino, Italy; [41]Health Data Specialists, Dubline, Ireland; [42]Johnson & Johnson, Raritan, NJ, United States of America; [43]Johnson & Johnson, High Wycombe, United Kingdom; [44]Johnson & Johnson, Paris, France; [45]Johnson & Johnson, Spring House, PA, United States of America; [46]Erasmus MC Cancer Institute, Rotterdam, The Netherlands; [47]IRCCS Azienda Ospedaliero-Universitaria di Bologna, Istituto di Ematologia "Seràgnoli", and Dipartimento di Scienze Mediche e Chirurgiche, Università di Bologna, Bologna, Italy;*

**Background**

Tec is the most widely used BCMA×CD3 BsAb in heavily pretreated relapsed/refractory multiple myeloma, with improved outcomes in earlier lines of therapy. EMN30/MajesTEC-4 (NCT05243797) is a Phase 3 study evaluating Tec alone or with Len as post-ASCT maintenance in NDMM. Initial SRI results from 3 cohorts (Cohorts 1 and 2: Tec-Len; Cohort 3: Tec) showed promising early safety and efficacy with median follow-up: 21.1, 9.2, and 9.2 mo, respectively.

**Aims**

To report updated SRI safety and efficacy results with up to 3 years median follow up.

**Methods**

Eligible patients (pts) had NDMM, received 4-6 cycles (C) of a 3- or 4-drug induction therapy and ASCT ± consolidation, and achieved ≥PR. Pts received maintenance treatment for up to 26 C. Tec dosing in Cohort 1: 1.5 mg/kg QW in C1-2 (preceded by step-up doses [SUD] in C1); 3 mg/kg Q2W in C3-6; 3 mg/kg Q4W in C7+. Tec dosing in Cohorts 2/3: 1.5 mg/kg on day 8/15 in C1 (preceded by SUD); 3 mg/kg Q4W in C2+. Len dosing in Cohorts 1/2: 10 mg QD in C2-4; 15 mg in C5+, if tolerated, with the option to continue Len after C26; Tec was stopped after C13 if in ≥CR. AEs were graded by NCI-CTCAE v5.0; CRS and ICANS by ASTCT guidelines. Investigator-assessed response followed 2016 IMWG criteria. MRD negativity ($10^{-5}$) was assessed by next-generation flow.

**Results**

94 pts were enrolled (n=32, n=32, and n=30 in Cohorts 1, 2, and 3). Baseline characteristics were comparable across cohorts. At the safety data cutoff (17 Sep 2025), median follow up was 32.7, 21.2, and 21.2 mo, respectively. The median number of maintenance cycles was 26, 21, and 23; median relative Tec dose intensity was 95-99%.
Grade (Gr) 3/4 TEAEs occurred in 100%, 96.9%, and 66.7% of pts in Cohorts 1, 2, and 3; Gr 3/4 neutropenia was most common (93.8%, 78.1%, and 63.3%). Any Gr infections occurred in 96.9%, 87.5%, and 90.0% of pts, respectively; Gr 3/4 infections in 37.5%, 34.4%, and 26.7%. Hypogammaglobulinemia incidence was 96.9%, 87.5%, and 93.3% in Cohorts 1, 2, and 3; all of these pts received ≥1 dose of IgG replacement. CRS rate was 44.7% (Gr 1/2: 38.3%/6.4%); all events resolved; none led to treatment discontinuation. No ICANS occurred. Nine pts had TEAEs leading to treatment discontinuation; 2 pts in Cohort 2 had fatal TEAEs (1 due to infection, pt did not receive IgG during hospitalization). Two non-TEAE deaths occurred.
At the efficacy data cutoff (08 Dec 2025), median follow-up was 35.3, 23.7, and 23.7 mo in Cohorts 1, 2, and 3, respectively. From post-ASCT to data cutoff, ≥CR rates increased from 46.9% to 100% in Cohort 1, 40.6% to 96.9% in Cohort 2, and 60.0% to 96.7% in Cohort 3. For evaluable pts, MRD negative ≥CR rates improved from post-ASCT to 12 mo in Cohorts 1 (33.3% [7/21] to 100% [19/19]), 2 (33.3% [8/24] to 100% [23/23]), and 3 (44.0% [11/25] to 90.0% [18/20]). Estimated 24-mo PFS rate was 96.6% for Cohort 1; 18-mo PFS rates were 93.8% and 96.4% for Cohorts 2 and 3.

**Copyright Information:** (Online) ISSN: 2572-9241
©2026 The Author(s). HemaSphere is published by John Wiley & Sons Ltd on behalf of European Hematology Association. This is an open access Abstract Book distributed under the Attribution-NonCommercial-NoDerivs (CC BY-NC-ND), which allows third parties to download the articles and share them with others as long as they credit the author and the Abstract Book, but they cannot change the content in any way or use them commercially.
**Abstract Book Citations:** Authors, Title, HemaSphere, 2026;10:(S1):pages. The abstract book DOIs can be found on the "Author Information" page.
**Disclaimer:** Articles published in the journal HemaSphere exclusively reflect the opinions of the authors. The authors are responsible for all content in their abstracts including accuracy of the facts, statements, citing resources, etc.

**Summary/Conclusion**

Tec ± Len can be safely administered as maintenance therapy post-ASCT in NDMM, with monthly Tec dosing as early as C2 in responders. CRS and infections were manageable with established protocols, making this regimen suitable for use across practice settings. Tec ± Len improved ≥CR rate to nearly 100% post-ASCT in all cohorts, with notable increases in MRD negative ≥CR rates. PFS rates for SRI cohorts were promising and will be further evaluated in the randomized part of EMN30/MajesTEC-4, which is actively enrolling.

**Copyright Information:** (Online) ISSN: 2572-9241
©2026 The Author(s). HemaSphere is published by John Wiley & Sons Ltd on behalf of European Hematology Association. This is an open access Abstract Book distributed under the Attribution-NonCommercial-NoDerivs (CC BY-NC-ND), which allows third parties to download the articles and share them with others as long as they credit the author and the Abstract Book, but they cannot change the content in any way or use them commercially.
**Abstract Book Citations:** Authors, Title, HemaSphere, 2026;10:(S1):pages. The abstract book DOIs can be found on the “Author Information” page.
**Disclaimer:** Articles published in the journal HemaSphere exclusively reflect the opinions of the authors. The authors are responsible for all content in their abstracts including accuracy of the facts, statements, citing resources, etc.

### (S198) TECLISTAMAB PLUS DARATUMUMAB (TEC-DARA) IN PATIENTS (PTS) WITH RELAPSED REFRACTORY MULTIPLE MYELOMA (RRMM): ANALYSIS OF MAJESTEC-3 BASED ON CYTOGENETIC AND FUNCTIONAL RISK

**Topic:** 14. Myeloma and other monoclonal gammopathies - Clinical

Rahul Banerjee*[1, 2], Niels W C J van de Donk[3], Roberto Mina[4], María-Victoria Mateos[5], Nizar J Bahlis[6], Aurore Perrot[7], Ajay Nooka[8], Jin Lu[9], Charlotte Pawlyn[10, 11], Gaston Caeiro[12], Alain Kentos[13], Vania Hungria[14], Anne K. Mylin[15], Charlotte Toftmann Hansen[16], Raphael Teipel[17], Britta Besemer[18], Meletios A. Dimopoulos[19, 20], Elena Zamagni[21, 22], Satoshi Yoshihara[23], Kihyun Kim[24], Paulus A.F. Geerts[25], Elena Segarceanu[26], Agata Tyczyńska[27], Markus Hansson[28], Mark Grey[29], Surbhi Sidana[30], Paula Rodríguez-Otero[31], Joaquín Martinez-Lopez[32], Hamza Hashmi[33], Larry Anderson[34], Claudio Cerchione[35], Salomon Manier[36], Jesus San Miguel Izquierdo[37], Muhamed Baljević[38], Robin Carson[39], Weili Sun[40], Kristen Lantz[39], Anne Seifert[41], Deborah Briseno-Toomey[42], Lisa O'rourke[39], Maria Rubin[43], Diego Vieyra[39], Lijuan Kang[39], Hongmei Xu[44], Ricardo Attar[39], Christopher Chiu[43], Luciano J. Costa[45]

*[1]Fred Hutchinson Cancer Center, Seattle, WA, United States of America;[2]University of Washington, Seattle, WA, United States of America; [3]Amsterdam University Medical Center, Vrije Universiteit Amsterdam, Amsterdam, The Netherlands;[4]Winship Cancer Institute, Emory University, Atlanta, GA, United States of America; [5]Hospital Universitario de Salamanca, Instituto de Investigación Biomédica de Salamanca, Instituto de Biología Molecular y Celular del Cáncer (Universidad de Salamanca-Consejo Superior de Investigaciones Científicas), CIBERONC, Salamanca, Spain; [6]Arnie Charbonneau Cancer Institute, University of Calgary, Calgary, AB, Canada; [7]Universite de Toulouse, Centre Hospitalier Universitaire, Service d'Hematologie, IUCT Oncopole CRCT, Toulouse, France; [8]Emory University, Winship Cancer Institute, Atlanta, GA, United States of America; [9]Peking University People's Hospital, Peking University Institute of Hematology, National Clinical Research Center for Hematologic Disease, Beijing, China; [10]The Royal Marsden NHS Foundation Trust, London, United Kingdom;[11]The Institute of Cancer Research, London, United Kingdom; [12]Hospital Privado Centro Medico de Córdoba SA, Córdoba, Argentina; [13]Department of Hematology, Hôpital de Jolimont, Haine-Saint-Paul, Belgium;[14]Clinica São Germano, São Paulo, Brazil; [15]Department of Hematology, Rigshospitalet, Copenhagen, Denmark; [16]Department of Hematology, Odense University Hospital, Odense, Denmark; [17]Medizinische Klinik und Poliklinik I Universitätsklinikum Carl Gustav Carus an der Technischen Universität Dresden, Dresden, Germany; [18]Department of Internal Medicine II, University Tübingen, Tübingen, Germany;[19]Department of Clinical Therapeutics, National and Kapodistrian University of Athens, School of Medicine, Athens, Greece; [20]Department of Medicine, Korea University, Seoul, South Korea; [21]IRCCS Azienda Ospedaliero-Universitaria di Bologna, Istituto di Ematologia "Seràgnoli,", Bologna, Italy; [22]Dipartimento di Scienze Mediche e Chirurgiche, Università di Bologna, Bologna, Italy; [23]Department of Hematology, Hyogo Medical University Hospital, Nishinomiya, Japan;[24]Division of Hematology-Oncology, Department of Medicine, Sungkyunkwan University School of Medicine, Samsung Medical Center, Seoul, South Korea; [25]Department of Internal Medicine, Isala Klinieken, Zwolle, The Netherlands;[26]Department of Hematology, St. Antonius Hospital Nieuwegein, Nieuwegein, The Netherlands; [27]Department of Hematology and Transplantology, Medical University of Gdansk; Department of Hematology and Transplantology, University Clinical Center, Gdansk, Poland; [28]Sahlgrenska University Hospital, Göteborg, Sweden; [29]The Lancashire Haematology Centre, Blackpool Teaching Hospitals NHS Foundation Trust, Blackpool Victoria Hospital, Blackpool, United Kingdom; [30]Stanford University School of Medicine, Palo Alto, CA, United States of America; [31]Cancer Center Clínica Universidad de Navarra, University of Navarra, Pamplona, Spain;[32]Hematology Department, Hospital 12 de Octubre, i+12, Universidad Complutense, MIC, Centro Nacional de Investigaciones Oncológicas, CIBERONC, Madrid, Spain; [33]Memorial Sloan Kettering Cancer Center, New York, NY, United States of America;[34]Hematologic Malignancies and Cellular Therapy Program, Simmons Comprehensive Cancer Center, UT Southwestern Medical Center, Dallas, TX, United States of America; [35]Hematology Unit, Istituto Romagnolo per lo Studio dei Tumori "Dino Amadori"-IRST IRCCS, Via Piero Maroncelli 40, 47014 Meldola, FC, Italy; [36]Hematology, CHU Lille, University of Lille, Lille, France; [37]Cancer Center Clinica Universidad de Navarra, CIMA, IDISNA, Pamplona, Spain; [38]Vanderbilt-Ingram Cancer Center, Vanderbilt University Medical Center, Nashville, TN, United States of America; [39]Johnson & Johnson, Spring House, PA, United States of America;[40]Johnson & Johnson, Los Angeles, CA, United States of America; [41]Johnson & Johnson, High Wycombe, United Kingdom; [42]Johnson & Johnson, Yorba Linda, CA, United States of America; [43]Johnson & Johnson, Raritan, NJ, United States of America; [44]Johnson & Johnson,*

**Copyright Information:** (Online) ISSN: 2572-9241
©2026 The Author(s). HemaSphere is published by John Wiley & Sons Ltd on behalf of European Hematology Association. This is an open access Abstract Book distributed under the Attribution-NonCommercial-NoDerivs (CC BY-NC-ND), which allows third parties to download the articles and share them with others as long as they credit the author and the Abstract Book, but they cannot change the content in any way or use them commercially.
**Abstract Book Citations:** Authors, Title, HemaSphere, 2026;10:(S1):pages. The abstract book DOIs can be found on the "Author Information" page.
**Disclaimer:** Articles published in the journal HemaSphere exclusively reflect the opinions of the authors. The authors are responsible for all content in their abstracts including accuracy of the facts, statements, citing resources, etc.

*Shanghai, China; [45]Division of Hematology and Oncology, University of Alabama at Birmingham, Birmingham, AL, United States of America;*

**Background**

Tec-Dara demonstrated significant improvement in PFS (primary endpoint) and all key secondary endpoints (including ≥CR, minimal residual disease [MRD] negativity [neg], and OS) vs Dara + dexamethasone + pomalidomide or bortezomib (DPd/DVd) in the primary analysis of the phase 3 MajesTEC-3 trial (NCT05083169) in early-line RRMM. PFS/OS favored Tec-Dara across all subgroups, and the safety profile was well-managed.

**Aims**

We report in-depth, post-hoc subgroup analyses of MajesTEC-3 (PFS, MRD-neg CR rate) in pts with and without high-risk cytogenetic abnormalities (HRCAs) and functional high-risk (FHR) status.

**Methods**

Pts had 1-3 prior lines of therapy (LOT) including a proteasome inhibitor and lenalidomide (lenalidomide refractory if only 1 prior LOT) with progressive disease (PD) on/after last LOT. Pts were randomized 1:1 to Tec-Dara or DPd/DVd. Tec-Dara pts received 28-day cycles (C) of Tec (C1-2: 1.5 mg/kg QW [C1 dose preceded by approved step-up dose schedule]; C3-6: 3 mg/kg Q2W; C7+: 3 mg/kg Q4W) with Dara; steroid-sparing after C1 Day 8. Tec and Dara dosing were aligned with the approved Dara schedule. DPd/DVd were administered per approved schedules.
The prespecified definition of high risk included ≥1 of the following HRCAs: t(4;14), t(14;16), or del(17p); a revised high-risk definition also included gain(1q21) or amp(1q21) (3 and ≥4 copies, respectively). Risk subgroups reported include: standard (std.) or high (0 or ≥1 HRCAs, respectively, per prespecified definition); revised std. or high (0 or ≥1 HRCAs per revised definition); and 1 or ≥2 HRCAs (per revised definition). FHR was defined as 1 prior LOT with PD ≤18 mo post-ASCT or start of initial therapy. MRD-neg CR rate was defined as the percentage of pts in the NGS analysis set (all randomized pts except those in China [only NGF cytometry available]) who achieved both ≥CR and MRD neg ($10^{-5}$ or $10^{-6}$).

**Results**

587 pts were randomized (Tec-Dara, n=291; DPd/DVd, n=296). At 34.5 mo median follow-up, PFS favored Tec-Dara vs DPd/DVd across all cytogenetic subgroups (**Figure**). Estimated 36-mo PFS rates were higher with Tec-Dara vs DPd/DVd for both prespecified std. (87.4% vs 37.1%) and high-risk cytogenetics (77.7% vs 11.5%). Among pts with 1 prior LOT, PFS also favored Tec-Dara vs DPd/DVd both in pts with FHR (estimated 36-mo PFS rate, 77.3% vs 0%; HR, 0.22 [95% CI, 0.08-0.62]) and without FHR (89.9% vs 35.9%; HR, 0.11 [95% CI, 0.05-0.23]).

MRD-neg CR rates were higher with Tec-Dara vs DPd/DVd across all risk subgroups: std. ($10^{-5}$: 62.6% vs 24.8%; $10^{-6}$: 58.9% vs 14.7%; both P<0.0001), high (57.9% vs 9.7%; 54.7% vs 5.4%; both P<0.0001), revised std. (67.4% vs 18.8%; 67.4% vs 6.3%; both P<0.0001), revised high (57.5% vs 17.5%; 53.4% vs 11.3%; both P<0.0001), 1 HRCA (53.9% vs 21.4%; 48.3% vs 14.3%; both P<0.0001), ≥2 HRCAs (63.2% vs 11.3%; 61.4% vs 6.5%; both P<0.0001). MRD-neg CR rates were also higher with Tec-Dara in pts with FHR MM ($10^{-5}$: 42.9% vs 5.6%, P=0.0106; $10^{-6}$: 38.1% vs 0%, P=0.0040).
Additional data and high-risk subgroups will be presented.

**Copyright Information:** (Online) ISSN: 2572-9241
©2026 The Author(s). HemaSphere is published by John Wiley & Sons Ltd on behalf of European Hematology Association. This is an open access Abstract Book distributed under the Attribution-NonCommercial-NoDerivs (CC BY-NC-ND), which allows third parties to download the articles and share them with others as long as they credit the author and the Abstract Book, but they cannot change the content in any way or use them commercially.
**Abstract Book Citations:** Authors, Title, HemaSphere, 2026;10:(S1):pages. The abstract book DOIs can be found on the "Author Information" page.
**Disclaimer:** Articles published in the journal HemaSphere exclusively reflect the opinions of the authors. The authors are responsible for all content in their abstracts including accuracy of the facts, statements, citing resources, etc.

### Summary/Conclusion

Tec-Dara substantially improved PFS and MRD-neg CR rates vs DPd/DVd regardless of cytogenetic risk and FHR status. The 36-mo PFS rates of 87.4% and 89.9% in pts with std. risk and non-FHR MM, respectively, and attenuation of the adverse prognosis associated with high-risk disease support Tec-Dara as a steroid-sparing standard-of-care as early as the 2nd LOT, across all practice settings and regardless of risk status.

**Figure. (A) Subgroup analysis of PFS based on the prespecified definition for cytogenetic risk status and (B) subgroup analysis of PFS in the ITT population.**

**A.**

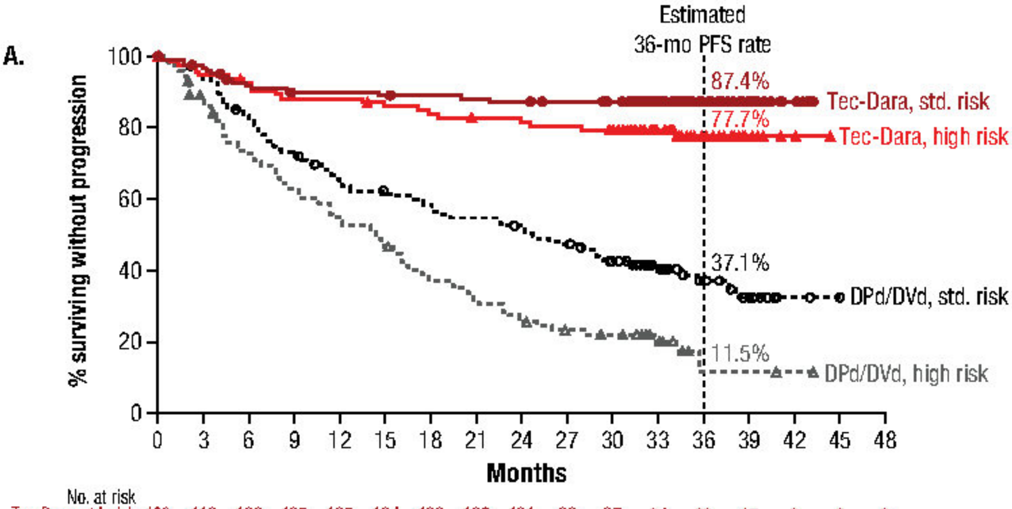

| No. at risk | | | | | | | | | | | | | | | | | |
|---|---|---|---|---|---|---|---|---|---|---|---|---|---|---|---|---|---|
| Tec-Dara, std. risk | 126 | 116 | 108 | 105 | 105 | 104 | 103 | 102 | 101 | 99 | 97 | 64 | 36 | 17 | 6 | 0 | 0 |
| DPd/DVd, std. risk | 145 | 131 | 115 | 99 | 87 | 82 | 78 | 73 | 69 | 62 | 53 | 34 | 20 | 10 | 2 | 1 | 0 |
| Tec-Dara, high risk | 104 | 89 | 86 | 82 | 82 | 79 | 77 | 75 | 74 | 73 | 71 | 47 | 31 | 10 | 3 | 0 | 0 |
| DPd/DVd, high risk | 104 | 85 | 71 | 61 | 53 | 45 | 35 | 30 | 24 | 20 | 18 | 10 | 2 | 2 | 1 | 0 | 0 |

**B.**

| | Tec-Dara | | DPd/DVd | | | |
|---|---|---|---|---|---|---|
| | Events n/N | Median PFS (mo) | Events n/N | Median PFS (mo) | HR (95% CI) | *P* value |
| Functional high risk | 5/26 | NE | 14/20 | 23.2 | 0.22 (0.08-0.62) | 0.0019 |
| Not functional high risk | 8/82 | NE | 57/94 | 20.8 | 0.11 (0.05-0.23) | <0.0001 |
| Std. risk | 15/126 | NE | 84/145 | 24.7 | 0.16 (0.19-0.27) | <0.0001 |
| High risk | 20/104 | NE | 78/104 | 14.4 | 0.15 (0.09-0.25) | <0.0001 |
| Revised std. risk | 5/52 | NE | 34/55 | 24.2 | 0.12 (0.04-0.30) | <0.0001 |
| Revised high risk | 30/169 | NE | 122/180 | 15.8 | 0.18 (0.12-0.26) | <0.0001 |
| 1 HRCA | 17/105 | NE | 69/111 | 17.9 | 0.19 (0.11-0.32) | <0.0001 |
| ≥2 HRCAs | 13/64 | NE | 53/69 | 14.4 | 0.16 (0.08-0.29) | <0.0001 |

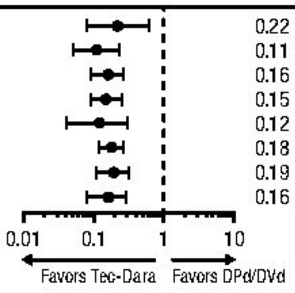

PFS, progression-free survival; ITT, intent-to-treat; Tec-Dara, teclistamab in combination with daratumumab; DPd/DVd, daratumumab with pomalidomide and dexamethasone/daratumumab with bortezomib and dexamethasone; std., standard; HR, hazard ratio; CI, confidence interval; NE, not estimable; HRCA, high-risk cytogenetic abnormality; FISH, fluorescence in situ hybridization; ASCT, autologous stem cell transplantation.
**NOTE** that cytogenetic risk was centrally assessed by FISH. Functional high-risk status included only those patients with 1 prior line of therapy and disease progression ≤18 mo after receiving ASCT or starting initial therapy.

**Copyright Information:** (Online) ISSN: 2572-9241
©2026 The Author(s). HemaSphere is published by John Wiley & Sons Ltd on behalf of European Hematology Association. This is an open access Abstract Book distributed under the Attribution-NonCommercial-NoDerivs (CC BY-NC-ND), which allows third parties to download the articles and share them with others as long as they credit the author and the Abstract Book, but they cannot change the content in any way or use them commercially.
**Abstract Book Citations:** Authors, Title, HemaSphere, 2026;10:(S1):pages. The abstract book DOIs can be found on the "Author Information" page.
**Disclaimer:** Articles published in the journal HemaSphere exclusively reflect the opinions of the authors. The authors are responsible for all content in their abstracts including accuracy of the facts, statements, citing resources, etc.

### (S199) SAFETY AND EFFICACY OF IBERDOMIDE-BASED QUADRUPLET FOR PATIENTS WITH NEWLY DIAGNOSED MULTIPLE MYELOMA: THE IDEAL PHASE I/II TRIAL RESULTS

**Topic:** 14. Myeloma and other monoclonal gammopathies - Clinical

Prashant Kapoor*[1], Wilson Gonsalves[1], Mustaqeem Siddiqui[1], Eli Muchtar[1], Taxiarchis Kourelis[1], Francis Buadi[1], Erik Asmus[1], Moritz Binder[1], Alyssa Larson[1], Samantha Erickson[1], Joselle Cook[1], Nadine Abdallah[1], Saurabh Zanwar[1], Angela Dispenzieri[1], Rahma Warsame[2], Ronald Go[1], David Dingli[1], Morie Gertz[1], Vincent Rajkumar[1], Betsy Knopf[1], Shaji Kumar[1]

*[1]Mayo Clinic, Rochester, United States of America; [2]Mayo clinic, Rochester, United States of America;*

### Background

Patients with newly diagnosed multiple myeloma (NDMM) are managed currently with an anti-CD38 monoclonal antibody, a proteasome inhibitor, an immunomodulatory drug and dexamethasone that comprise the quadruplet induction therapy. We conducted the IDEAL study (NCT05392946) to examine the safety and efficacy of a novel finite-duration approach in which iberdomide (CC-220) — an oral cereblon E3 ligase modulator (CELMoD), with a higher affinity and specificity for cereblon than lenalidomide — was substituted for lenalidomide in the traditional quadruplet induction for patients with NDMM. We present the safety and efficacy results from this trial.

### Aims

The primary aim of this clinical trial was to assess ≥ complete response (CR) rate during induction phase, utilizing a more sensitive mass-spectrometry-based assay.

### Methods

IDEAL is a Phase 1 (3+3 design)/ Phase 2 (single stage) trial in which Iberdomide, daratumumab, bortezomib & dexamethasone (Iber-DVd) were given for twelve 28-day cycles (C1-12) as induction followed by iberdomide monotherapy maintenance (C13-36). Patients with NDMM were eligible, irrespective of their eligibility for autologous stem cell transplant. In the Phase 1 portion, Dose Level (DL) 1 was the starting level, with iberdomide dosed at 1 mg/day PO, days 1-21, and the recommended phase 2 dose (RP2D) was determined. Daratumumab was given at 1800mg SQ, weekly for 2 cycles, every other week during Cycles 3-6, and every 4 weeks thereafter, bortezomib 1.3 mg/$m^2$ SQ, weekly & dexamethasone 40 mg PO, weekly, followed by iberdomide monotherapy (1 mg/day, Days, 1-21 of 28-day cycles).

**Copyright Information:** (Online) ISSN: 2572-9241
©2026 The Author(s). HemaSphere is published by John Wiley & Sons Ltd on behalf of European Hematology Association. This is an open access Abstract Book distributed under the Attribution-NonCommercial-NoDerivs (CC BY-NC-ND), which allows third parties to download the articles and share them with others as long as they credit the author and the Abstract Book, but they cannot change the content in any way or use them commercially.
**Abstract Book Citations:** Authors, Title, HemaSphere, 2026;10:(S1):pages. The abstract book DOIs can be found on the "Author Information" page.
**Disclaimer:** Articles published in the journal HemaSphere exclusively reflect the opinions of the authors. The authors are responsible for all content in their abstracts including accuracy of the facts, statements, citing resources, etc.

**Results**

Forty-seven patients were enrolled, including 9 patients in Phase 1. In the DLT assessment period (Cycle 1 during which growth factor support was not permitted), 3 dose-limiting toxicities (DLTs) were observed; two at DL1: one grade 3 febrile neutropenia and one grade 4 neutropenia; one grade 4 neutropenia occurred at DL-1. RP2D was 0.75 mg/day. This analysis focuses on 44 pts [Dose Confirmation Cohort (Phase 1) dosed at maximal tolerated dose (n=6) and Dose Expansion Cohort (n=38)]. The median age at registration was 65 (range: 36-86 ) years and 52.3% patients had high-risk disease. A median of 15 cycles (range: 3-36) had been administered by the data cutoff date. Overall response rate was 100%; ≥CR rate, 36.4% during induction & 52.3%, upon including the maintenance phase. The Euro flow-based (sensitivity $10^{-5}$) bone marrow MRD negative rate was 29.5% during the induction phase and 47.7% overall. At a median follow-up of 18.3 months, 2 fatal occurrences were noted (one death due to progressive disease during follow-up and the other on-treatment death due to COVID-19). The 12- and 18-month progression-free survival rates were 91% and 88%, respectively, while OS rates at these respective timepoints were 97% and 94%. Grade 3/4 toxicities at RP2D, occurring in ≥ 5% (total) of patients, were neutropenia (41%/16%, leading 27% of patients to require peg-filgrastim support), lymphopenia (47.7%/6.8%), infections (11.4%/2.3%), thrombocytopenia (2.3%/4.5%) and rash (7%/0%). Cases of high-grade diarrhea or fatigue were low (<5%). We will present additional updated data at the EHA Congress.

**Summary/Conclusion**

Fixed-duration therapy with Iber-DVd followed by iberdomide monotherapy is a safe and effective approach in NDMM, with 100% ORR, evidence of improvement in the depth of response and nearly one-half of patients achieving MRD negative state over time.

© 2026 American Society of Clinical Oncology, Inc. Reused with permission. This abstract was accepted and previously presented at the 2026 ASCO Annual Meeting. All rights reserved.

**Copyright Information:** (Online) ISSN: 2572-9241
©2026 The Author(s). HemaSphere is published by John Wiley & Sons Ltd on behalf of European Hematology Association. This is an open access Abstract Book distributed under the Attribution-NonCommercial-NoDerivs (CC BY-NC-ND), which allows third parties to download the articles and share them with others as long as they credit the author and the Abstract Book, but they cannot change the content in any way or use them commercially.
**Abstract Book Citations:** Authors, Title, HemaSphere, 2026;10:(S1):pages. The abstract book DOIs can be found on the "Author Information" page.
**Disclaimer:** Articles published in the journal HemaSphere exclusively reflect the opinions of the authors. The authors are responsible for all content in their abstracts including accuracy of the facts, statements, citing resources, etc.

### (S200) ARLOCABTAGENE AUTOLEUCEL, A GPRC5D-TARGETED CAR T-CELL THERAPY FOR PATIENTS WITH RELAPSED/REFRACTORY MULTIPLE MYELOMA: UPDATED PHASE 1 SAFETY AND EFFICACY RESULTS IN PATIENTS WITH 1–3 PRIOR REGIMENS

**Topic:** 14. Myeloma and other monoclonal gammopathies - Clinical

Susan Bal*[1], Myo Htut[2], Jesus Berdeja[3], Larry Anderson[4], Adriana Rossi[5], Tara Gregory[6], Luciano J. Costa[1], Hongxiang Hu[7], Ziyan Guo[7], Jinjie Chen[7], Kristina Jordahl[7], Naomey Sarkis[7], Safiyyah Ziyad[7], Wei-Ming Kao[7], Allie Kaeding[7], Mike Burgess[7], Omar Nadeem[8]

*[1]University of Alabama at Birmingham, Birmingham, Alabama, United States of America;[2]City of Hope Comprehensive Cancer Center, Duarte, California, United States of America; [3]Greco-Hainsworth Tennessee Oncology Center for Research, Nashville, Tennessee, United States of America; [4]UT Southwestern Medical Center, Myeloma, Waldenstrom's, and Amyloidosis Program, Section of Hematologic Malignancies and Cellular Therapy, Simmons Comprehensive Cancer Center, Dallas, Texas, United States of America; [5]Icahn School of Medicine at Mount Sinai, New York, New York, United States of America;[6]Colorado Blood Cancer Institute, Sarah Cannon Cancer Network, Denver, Colorado, United States of America; [7]Bristol Myers Squibb, Princeton, New Jersey, United States of America; [8]Dana-Farber Cancer Institute, Boston, Massachusetts, United States of America;*

### Background

There is an urgent need for new therapeutic options with alternative targets or mechanisms of action for patients (pts) with relapsed and refractory multiple myeloma (RRMM). Arlocabtagene autoleucel (arlo-cel; CC-95266; BMS-986393) is a chimeric antigen receptor (CAR) T cell therapy targeting GPRC5D that is being evaluated in the multicohort phase 1 trial (NCT04674813).

### Aims

To report updated safety and efficacy data for the cohort of pts with 1–3 prior lines of therapy.

### Methods

Pts had 1–3 prior anti-MM regimens, including a proteasome inhibitor and an immunomodulatory agent. Anti-CD38 therapy was not required; BCMA-directed therapies, including CAR T cells, were allowed. After screening and leukapheresis (bridging therapy optional), pts received lymphodepleting chemotherapy followed by a single infusion of arlo-cel at the recommended phase 2 dose (150×106 CAR T cells). The primary objective was safety; secondary objectives included clinical activity per IMWG Uniform Response Criteria and pharmacokinetics.

**Copyright Information:** (Online) ISSN: 2572-9241
©2026 The Author(s). HemaSphere is published by John Wiley & Sons Ltd on behalf of European Hematology Association. This is an open access Abstract Book distributed under the Attribution-NonCommercial-NoDerivs (CC BY-NC-ND), which allows third parties to download the articles and share them with others as long as they credit the author and the Abstract Book, but they cannot change the content in any way or use them commercially.
**Abstract Book Citations:** Authors, Title, HemaSphere, 2026;10:(S1):pages. The abstract book DOIs can be found on the "Author Information" page.
**Disclaimer:** Articles published in the journal HemaSphere exclusively reflect the opinions of the authors. The authors are responsible for all content in their abstracts including accuracy of the facts, statements, citing resources, etc.

**Results**

As of June 6, 2025, 31 pts had been enrolled and 100% received arlo-cel following successful manufacturing; 61% (19/31) received optional systemic bridging therapy. Median age was 62 y (range 31–78); 68% were male. Overall, 26% had high-risk cytogenetics (del[17p], t[4;14], and/or t[14;16]), 68% had 1q21 gain/amp, and 32% had extramedullary disease. Pts had a median of 2 prior regimens; 29% received 3 prior regimens.
All 31 pts had a treatment-emergent (TE) adverse event (AE); 87% had grade (G) 3/4 TEAEs. No deaths were attributed to AEs. Treatment-related AEs occurred in 97% (48% G3/4). Cytokine release syndrome occurred in 84% (all G1/2 resolved); no pts had macrophage activation syndrome/hemophagocytic lymphohistiocytosis. Immune effector cell-associated neurotoxicity syndrome occurred in 10% (all G1/2 resolved). On-target/off-tumor nail, skin, and oral TEAEs were reported in 36%, 26% and 42%, respectively. Other select neurotoxicity occurred in 6.5% of patients, including 1 event of ataxia, 1 event of dysarthria, 2 events of gait disturbance; all events were grade ≤2 and ongoing. TE infections occurred in 55% (all G1/2).
In the efficacy-evaluable population (n=24), after a median 18.3 mo follow-up (range 3.8–24.3 mo) the ORR was 96% (23/24) and CR rate was 67% (16/24); 11/23 responses were still ongoing. The 12-mo PFS rate was 74%. Of 16 pts with minimal residual disease (MRD) data, the MRD negative ($10^{-5}$ depth) CR rate was 56.3%.
Long-term follow-up data will be included in the final presentation.

**Summary/Conclusion**

A one-time administration of arlo-cel in pts with RRMM and 1–3 prior lines of therapy was well tolerated and led to a high response rate that deepened over time, with few early relapses after 18.3 mo median follow-up. The favorable benefit-risk profile for this dose and population was consistent with prior disclosures. Notably, frequency and grade of infections were lower than those reported for BCMA-targeted therapies. These data support arlo-cel as a safe and effective potential early-line treatment in RRMM, with a phase 3 trial (QUINTESSENTIAL 2; NCT06615479) underway.

**Copyright Information:** (Online) ISSN: 2572-9241
©2026 The Author(s). HemaSphere is published by John Wiley & Sons Ltd on behalf of European Hematology Association. This is an open access Abstract Book distributed under the Attribution-NonCommercial-NoDerivs (CC BY-NC-ND), which allows third parties to download the articles and share them with others as long as they credit the author and the Abstract Book, but they cannot change the content in any way or use them commercially.
**Abstract Book Citations:** Authors, Title, HemaSphere, 2026;10:(S1):pages. The abstract book DOIs can be found on the “Author Information” page.
**Disclaimer:** Articles published in the journal HemaSphere exclusively reflect the opinions of the authors. The authors are responsible for all content in their abstracts including accuracy of the facts, statements, citing resources, etc.

### (S201) CB-011, AN ALLOGENEIC ANTI-BCMA CAR-T CELL THERAPY WITH IMMUNE CLOAKING, FOR PATIENTS WITH RELAPSED/REFRACTORY MULTIPLE MYELOMA (CAMMOUFLAGE PHASE 1 TRIAL)

**Topic:** 14. Myeloma and other monoclonal gammopathies - Clinical

Binod Dhakal*[1], Adriana Rossi[2], William Clark[3], Daniel Sherbenou[4], David S. Siegel[5], Manisha Bhutani[6], Faiz Anwer[7], Sham Mailankody[8], James Essell[9], Jay Spiegel[10], Douglas Sborov[11], Donna Marcy[12], Ine-Mari Bornman[12], Chris Holland[12], Jeffrey Hellman[12], Elizabeth Garner[12], Pingping Mao[12], Eric Stawiski[12], Enrique Zudaire[12], Tonia Nesheiwat[12], Socorro Portella[12], Wellington Mendes[12], Sundar Jagannath[13], Luciano J. Costa[14]

*[1]Blood and Marrow Transplant and Cellular Therapy Program, Medical College of Wisconsin, Milwaukee, WI, United States of America; [2]Division of Hematology and Medical Oncology, Tisch Cancer Institute, Icahn School of Medicine at Mount Sinai, New York, NY, United States of America; [3]Department of Bone Marrow Transplant, VCU Massey Cancer Center, Richmond, VA, United States of America; [4]University of Colorado Anschutz Medical Campus, Division of Hematology, Department of Medicine, Aurora, CO, United States of America; [5]Division of Myeloma, John Theurer Cancer Center, Hackensack University Medical Center, Hackensack, NJ, United States of America; [6]Division of Hematology, Atrium Health Levine Cancer Institute, Wake Forest University School of Medicine, Charlotte, NC, United States of America; [7]Taussig Cancer Center, Cleveland Clinic, Cleveland, OH, United States of America; [8]Memorial Sloan Kettering Cancer Center, Department of Medicine, New York, NY, United States of America; [9]Oncology Hematology Care, Sarah Cannon Research Institute, Cincinnati, OH, United States of America; [10]Division of Transplant and Cellular Therapy, University of Miami Health System, Miami, FL, United States of America; [11]Cell Dyscrasia Program, Huntsman Cancer Institute at the University of Utah, Salt Lake City, UT, United States of America; [12]Caribou Biosciences, Inc., Berkeley, CA, United States of America; [13]Icahn School of Medicine at Mount Sinai, New York, NY, United States of America; [14]The University of Alabama, Division of Hematology and Oncology, Department of Medicine, Birmingham, AL, United States of America;*

### Background

CB-011 is an allogeneic anti-BCMA CAR-T cell therapy with immune cloaking manufactured from healthy donor T cells. It features 4 genome edits made with the CRISPR-Cas12a chRDNA genome-editing technology: (1) insertion of a BCMA-specific CAR, (2) *TRAC* gene KO to reduce risk of GvHD, (3) knockout (KO) of*B2M* to reduce T cell-mediated rejection, and (4) B2M–HLA-E fusion insertion to blunt NK cell-mediated rejection.

### Aims

CaMMouflage is a Phase 1 trial (NCT05722418) composed of 3+3 dose escalation and dose expansion to evaluate the safety and efficacy of CB-011 in patients (pts) with r/r MM and determine the recommended Phase 2 dose (RP2D).

### Methods

Pts received ≥3 prior lines of therapy (including a proteasome inhibitor, an immunomodulatory drug, and an anti-CD38 antibody). Pts received lymphodepletion (LD) for 3 days of either 300 cyclophosphamide (cy) or 500 cy (300 mg/m$^2$ or 500 mg/m$^2$ of cy, respectively) with 30 mg/m$^2$ of fludarabine followed by a single infusion of CB-011 at doses of 50 million (M), 150M, 300M, 450M, or 800M CAR-T cells.

**Copyright Information:** (Online) ISSN: 2572-9241
©2026 The Author(s). HemaSphere is published by John Wiley & Sons Ltd on behalf of European Hematology Association. This is an open access Abstract Book distributed under the Attribution-NonCommercial-NoDerivs (CC BY-NC-ND), which allows third parties to download the articles and share them with others as long as they credit the author and the Abstract Book, but they cannot change the content in any way or use them commercially.
**Abstract Book Citations:** Authors, Title, HemaSphere, 2026;10:(S1):pages. The abstract book DOIs can be found on the "Author Information" page.
**Disclaimer:** Articles published in the journal HemaSphere exclusively reflect the opinions of the authors. The authors are responsible for all content in their abstracts including accuracy of the facts, statements, citing resources, etc.

### Results

As of Sep 24, 2025, a total of 48 pts received CB-011. The median age was 68.5 years (49-84). Pts received a median of 4 prior lines of therapy (3-11), and 17% received prior BCMA-targeted therapy. 56% of pts had high-risk cytogenetics and 35% had extramedullary disease. Of the 13 pts dosed in the 300 cy LD cohort, one experienced CAR-T cell expansion and achieved an MRD-negative sCR ongoing at 21 months post dosing; therefore, the 500 cy LD regimen was implemented to optimize CAR-T cell expansion. In pts who received the 500 cy LD regimen (N=35), infections occurred in 49% of pts (14%, grade ≥3), CRS occurred in 31% of pts (3%, grade ≥3), ICANS occurred in 9% of pts (no grade ≥3), immune effector cell-associated hemophagocytic lymphohistiocytosis-like syndrome occurred in 9% of pts (3%, grade ≥3), and prolonged grade ≥3 cytopenias occurred in 33% of pts. No cases of GvHD, immune effector cell-associated enterocolitis, parkinsonism, or cranial nerve palsies were observed at any dose level. One grade 4 event of Guillain-Barré syndrome (GBS) occurred and is resolving. Three deaths occurred [pneumonia (unrelated), RSV (unrelated), ICAHT (related)], and have resulted in prophylactic measures/early intervention being implemented. For BCMA-naïve pts who received the recommended dose for expansion (RDE; 500 cy LD and 450M CAR-T cell dose) of CB-011 (N=12), the ORR was 92% and the [3] CR rate was 75% (see figure 1). At a median follow-up of 8.3 months, 7/12 (58%) pts were in a VGPR or better at ≥6 months. Of the MRD-evaluable pts, 10/11 (91%) achieved MRD negativity at $10^{-5}$ threshold. CB-011 exhibits a central memory CD8 T cell phenotype at peak of expansion. CB-011 expansion directly correlated with clinical responses and was higher in pts treated with 500 cy compared with 300 cy LD. Rapid recovery of endogenous T and NK cells was observed with endogenous B cells recovering within 6 months regardless of clinical response. Updated data will be presented.

### Summary/Conclusion

In a high-risk, heavily pretreated r/r MM pt population, CB-011 drove deep and durable responses with a manageable safety profile. Based on these results, the RDE is 450M CB-011 CAR-T cells after 500 cy LD. The dose expansion phase of CaMMouflage is enrolling BCMA-naïve and prior BCMA therapy-exposed r/r MM patients.

Figure 1: Responses observed at all CB-011 doses with selected LD

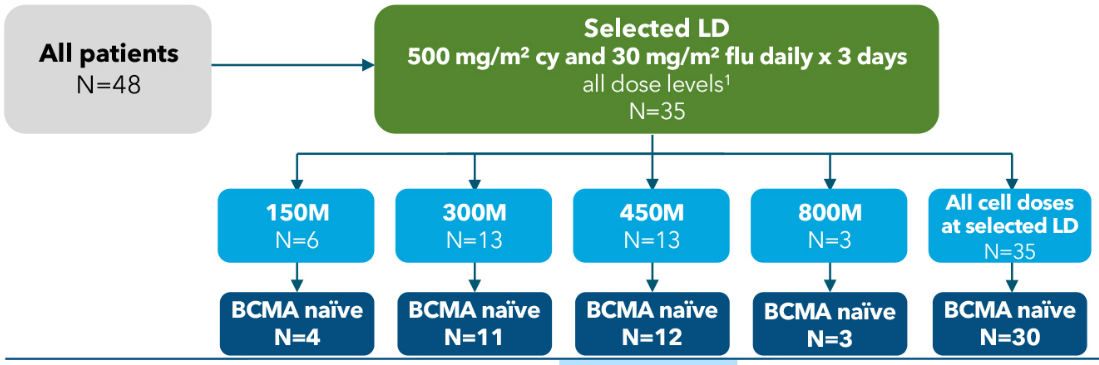

| | 150M, BCMA naïve N=4 | 300M, BCMA naïve N=11 | 450M, BCMA naïve N=12 | 800M, BCMA naïve N=3 | All cell doses at selected LD, BCMA naïve N=30 |
|---|---|---|---|---|---|
| **ORR** | 75% | 55% | **92%** | 33% | 70% |
| **CR/sCR** | 50% | 18% | **75%** | 0% | 43% |
| **≥VGPR** | 50% | 27% | **83%** | 0% | 50% |
| **MRD-negative rate[2]** | 50% (2/4) | 0% (0/3)[3] | **91% (10/11)[4]** | 0% (0/3) | 57% (12/21) |

[1]150M, 300M, 450M, or 800M CAR-T cells; [2]MRD negative at $\leq 10^{-5}$; [3]MRD available for 3 patients at the time of the data cut; [4]MRD not evaluable in one patient. Data cutoff 24Sept2025
CR: complete response; cy: cyclophosphamide; flu: fludarabine; LD: lymphodepletion; M: million; MRD: minimal residual disease; ORR: overall response rates; sCR: stringent complete response; VGPR: very good partial response

**Copyright Information:** (Online) ISSN: 2572-9241
©2026 The Author(s). HemaSphere is published by John Wiley & Sons Ltd on behalf of European Hematology Association. This is an open access Abstract Book distributed under the Attribution-NonCommercial-NoDerivs (CC BY-NC-ND), which allows third parties to download the articles and share them with others as long as they credit the author and the Abstract Book, but they cannot change the content in any way or use them commercially.
**Abstract Book Citations:** Authors, Title, HemaSphere, 2026;10:(S1):pages. The abstract book DOIs can be found on the "Author Information" page.
**Disclaimer:** Articles published in the journal HemaSphere exclusively reflect the opinions of the authors. The authors are responsible for all content in their abstracts including accuracy of the facts, statements, citing resources, etc.

### (S203) DREAMM-9 FINAL ANALYSIS: BELANTAMAB MAFODOTIN (BELAMAF), BORTEZOMIB, LENALIDOMIDE, AND DEXAMETHASONE (BVRD) FOR TRANSPLANT-INELIGIBLE (TI) NEWLY DIAGNOSED MULTIPLE MYELOMA (NDMM)

**Topic:** 14. Myeloma and other monoclonal gammopathies - Clinical

Saad Z Usmani[1], Michal Mielnik[2], Aránzazu Alonso Alonso[3, 4], Al-Ola Abdallah[5], Mamta Garg[6], Wojciech Janowski[7], Youngil Koh[8], Enrique M. Ocio*[9], Hang Quach[10], Karthik Ramasamy[11], Albert Oriol[9, 12], Paula Rodríguez-Otero[13], Irwindeep Sandhu[14], Katja C. Weisel[15], Chris Brawley[16], Miguel M. Murillo[17], Farrah Pompilus[18], Jacki Egger[19], Morrys C. Kaisermann[20], Marek Hus[2]

*[1]Memorial Sloan Kettering Cancer Center, New York, NY, United States of America;[2]Medical University of Lublin, Department of Hematooncology and Bone Marrow Transplantation, Lublin, Poland; [3]Hospital Universitario Quirónsalud Madrid, Madrid, Spain; [4]Universidad Europea de Madrid, Madrid, Spain; [5]University of Kansas Medical Center, US Myeloma Research Innovations Research Collaborative (USMIRC), Westwood, KS, United States of America; [6]Leicester Royal Infirmary, Leicester, United Kingdom; [7]Calvary Mater Newcastle, Newcastle, Australia;[8]Seoul National University Hospital, Department of Internal Medicine, Seoul, South Korea; [9]Hospital Universitario Marqués de Valdecilla (IDIVAL), Universidad de Cantabria, Santander, Spain;[10]St Vincent's Hospital Melbourne, University of Melbourne, Melbourne, VIC, Australia; [11]Oxford Translational Myeloma Centre, NDORMS, University of Oxford and Churchill Hospital, Headington, Oxford, United Kingdom; [12]Institut Català d'Oncologia and Institut Josep Carreras - Hospital Universitari Germans Trias i Pujol (HUGTP), Badalona, Spain; [13]Cancer Center Clínica Universidad de Navarra, Pamplona, Spain; [14]University of Alberta, Edmonton, AB, Canada;[15]University Medical Center of Hamburg-Eppendorf, Hamburg, Germany; [16]Development of Biostatistics for Oncology, GSK, London, United Kingdom; [17]Oncology Clinical Development, GSK, Stevenage, United Kingdom; [18]Patient-Centered Outcomes, GSK, Upper Providence, PA, United States of America; [19]Oncology Clinical Development, GSK, London, United Kingdom;[20]Oncology Clinical Development, GSK, Upper Providence, PA, United States of America;*

### Background

There is a need for new treatment options that improve outcomes in patients (pts) with TI NDMM. Belamaf-based combinations have recently been approved for relapsed/refractory multiple myeloma.

### Aims

DREAMM-9 (NCT04091126) was a phase 1 dose and schedule evaluation study of BVRd in adults with TI NDMM.

### Methods

Pts received BVRd in 1 of 8 belamaf dosing cohorts at an induction/maintenance schedule of Q3/4W (SHORT: 1.9, 1.4, 1.0 mg/kg), Q6/8W (STRETCH: 1.9, 1.4 mg/kg), Q9/12W (step-down [S/D]: 1.9 for 1 dose then 1.4 mg/kg; 1.4 for 1 dose then 1.0 mg/kg), or Q12W (1.0 mg/kg). The primary endpoint was safety. Responses, minimal residual disease negativity (MRD[-]) in complete response or better (CR+), and pt-reported outcomes (PROs) were assessed. Median dose intensities (mDIs) were calculated as mg/kg/21 days in induction and mg/kg/28 days in maintenance.

**Copyright Information:** (Online) ISSN: 2572-9241
©2026 The Author(s). HemaSphere is published by John Wiley & Sons Ltd on behalf of European Hematology Association. This is an open access Abstract Book distributed under the Attribution-NonCommercial-NoDerivs (CC BY-NC-ND), which allows third parties to download the articles and share them with others as long as they credit the author and the Abstract Book, but they cannot change the content in any way or use them commercially.
**Abstract Book Citations:** Authors, Title, HemaSphere, 2026;10:(S1):pages. The abstract book DOIs can be found on the "Author Information" page.
**Disclaimer:** Articles published in the journal HemaSphere exclusively reflect the opinions of the authors. The authors are responsible for all content in their abstracts including accuracy of the facts, statements, citing resources, etc.

### Results

As of June 2, 2025, 118 pts were enrolled (intention-to-treat population). Median duration of follow-up was 15.9–47.1 months. Induction mDI generally decreased as planned dosing intervals increased and was lowest in S/D Q9/12W or Q12W cohorts. Maintenance mDI was similar across cohorts due to dose modifications. Overall response rates were ≥83% across cohorts. Cohorts with higher induction mDI had the deepest responses (CR+/MRD[-] 75/55% for pooled SHORT+STRETCH and 59/43% for pooled Q9/12W+Q12W). Grade (Gr) ≥2 ophthalmic exam findings (OEFs; best-corrected visual acuity changes/slit lamp findings [Keratopathy and Visual Acuity scale]) were 90% for pooled SHORT+STRETCH and 69% for pooled Q9/12W+Q12W, while Gr 3/4 OEF rates were higher with higher induction mDI (79%/35%). Belamaf discontinuations due to Gr ≥3 OEFs occurred only in SHORT cohorts (n=1 each). First Gr ≥2 OEFs resolved prior to end of treatment in 83–100% across cohorts. The long-interval group (Q9/12W and Q12W dosing schedules) remained largely below the vision-related function (VRF) deterioration threshold (12.5 points per Ocular Surface Disease Index) across most timepoints, suggesting a favorable VRF profile vs the SHORT interval group.

### Summary/Conclusion

BVRd had high response rates across all cohorts (≥83%), highlighting the promising efficacy of the regimen. Regimens with higher belamaf induction mDI had the deepest responses, while those with lower induction mDI had milder OEFs. Belamaf dosing with a higher DI in the induction phase is optimal to achieve deeper responses, and a lower DI in the maintenance phase (mediated by dose modifications leading to longer schedules) improves tolerability, PROs, and maintains responses.
**Funding:** GSK (Study 209664). Drug linker technology licensed from Seagen Inc.; monoclonal antibody produced using POTELLIGENT Technology licensed from BioWa.
This abstract was previously submitted to the American Society of Clinical Oncology (ASCO) Annual Meeting, June 11–14, 2026, and is submitted on behalf of the original authors with their permission. ©2026 American Society of Clinical Oncology, Inc. Reused with permission. All rights reserved.

**Copyright Information:** (Online) ISSN: 2572-9241
©2026 The Author(s). HemaSphere is published by John Wiley & Sons Ltd on behalf of European Hematology Association. This is an open access Abstract Book distributed under the Attribution-NonCommercial-NoDerivs (CC BY-NC-ND), which allows third parties to download the articles and share them with others as long as they credit the author and the Abstract Book, but they cannot change the content in any way or use them commercially.
**Abstract Book Citations:** Authors, Title, HemaSphere, 2026;10:(S1):pages. The abstract book DOIs can be found on the "Author Information" page.
**Disclaimer:** Articles published in the journal HemaSphere exclusively reflect the opinions of the authors. The authors are responsible for all content in their abstracts including accuracy of the facts, statements, citing resources, etc.

### (S204) HIGH MRD NEGATIVITY RATES AND PROLONGED PFS WITH BELANTAMAB MAFODOTIN PLUS DARATUMUMAB, LENALIDOMIDE, AND DEXAMETHASONE IN TRANSPLANT INELIGIBLE NEWLY-DIAGNOSED MYELOMA: RESULTS OF THE BELA-DRD STUDY

**Topic:** 14. Myeloma and other monoclonal gammopathies - Clinical

Evangelos Terpos*[1], Ioannis Ntanasis-Stathopoulos[1], Maria Gavriatopoulou[1], Nikolaos Kanellias[1], Panagiotis Malandrakis[1], Eirini Solia[1], Foteini Theodorakakou[1], Yagnik Vyas[2], Magdalini Migkou[1], Evangelos Eleutherakis-Papaiakovou[1], Efstathios Kastritis[1], Meletios A. Dimopoulos[1, 3]

*[1]National and Kapodistrian University of Athens, School of Medicine, Department of Clinical Therapeutics, Athens, Greece; [2]Veeda Lifesciences, Athens, Greece; [3]Korea University, Department of Medicine, Seoul, South Korea;*

### Background

The MAIA trial established daratumumab with lenalidomide, dexamethasone (DRd) as standard therapy for transplant-ineligible (TI) newly diagnosed multiple myeloma (NDMM). Belantamab mafodotin (belamaf) showed high efficacy in relapsed/refractory disease. Quadruplet regimens improved clinical outcomes, providing a strong rationale to evaluate belamaf in combination with DRd in TI NDMM.

### Aims

The aim of this study was to assess the efficacy and safety of Bela-DRd in TI NDMM patients.

### Methods

BelaDRd is a phase 1/2, open-label study (EUCT-2021-006792-42) evaluated this combination in TI-NDMM. Part 1 (dose-finding phase) assessed the safety and tolerability of two belamaf doses (1.9/1.4 mg/kg Q8W) with DRd. This part established belamaf 1.9 mg/kg Q8W, extended to Q12W to account as the recommended phase 2 dose (RP2D). Part 2 (dose-expansion phase) evaluates the safety and efficacy of the RP2D in two cohorts Groups A and B, guided by ophthalmologist and hematologist (using Vision-Related Anamnestic,VRA tool), respectively.

**Copyright Information:** (Online) ISSN: 2572-9241
©2026 The Author(s). HemaSphere is published by John Wiley & Sons Ltd on behalf of European Hematology Association. This is an open access Abstract Book distributed under the Attribution-NonCommercial-NoDerivs (CC BY-NC-ND), which allows third parties to download the articles and share them with others as long as they credit the author and the Abstract Book, but they cannot change the content in any way or use them commercially.
**Abstract Book Citations:** Authors, Title, HemaSphere, 2026;10:(S1):pages. The abstract book DOIs can be found on the "Author Information" page.
**Disclaimer:** Articles published in the journal HemaSphere exclusively reflect the opinions of the authors. The authors are responsible for all content in their abstracts including accuracy of the facts, statements, citing resources, etc.

**Results**

In Part 1, 24 patients (median age: 73 years; male: 54.2%) entered the study;12.5% had high-risk cytogenetics (HRC). At a median follow-up of 34.3 months, 18 (75%) patients remained on treatment, while 6(25%) discontinued; one due to AE/SAE, one due to disease progression, one withdrew consent, and three died due to infections. Of 380 planned belamaf doses, 105(27.6%) doses were skipped due to OAEs. The median dose intensity (MDI) of belamaf was 0.5 mg/kg/Q4W. The overall response rate (ORR) was 91.7%; 70% of patients achieved ≥CR. MRD negativity was achieved in 13(81.3%) of evaluable patients (n=16). In Part 2 (n=12 patients; median age: 74 years),16.7% of patients had HRC. At a median follow-up of 17.9 months, 11(91.7%) patients remained on treatment; one patient (8.3%) discontinued due to PD. Of 113 planned belamaf doses 13(11.5%) doses were skipped due to OAEs. The MDI was 0.8 mg/kg/Q4W. ORR was again 91.7% (11/12 patients), while MRD negativity was achieved in 5(71.4%) of evaluable patients (n=7). The median time to ≥PR was 1.1 months for both parts. At 18 months, PFS was 91.7% (95% CI 76.4-97.2) and 87.5% (95% CI 66.1-95.8) in the overall and RP2D populations, respectively; corresponding TTP rates were 97.1% (95% CI 80.9-99.6) and 95.5% (95% CI 71.9-99.4) [Figure]. Fatal TEAEs occurred in 3 pts of cohort 1 and noneobserved in part 2. Fatigue was the most common Gr ≥3 non-ocular AE in parts 1 and 2 (13 pts [54.2%] and 6 pts [50.0%], respectively); mainly attributed to lenalidomide. Grade ≥3 BCVA decline was observed in 74(10.4%) of ocular assessments in part 1 and 4(3.9%) and 5(5.3%) in part 2 groups A and B, respectively. Grade ≥2/≥3 keratopathy occurred in 20.2%/0.6% of assessments in Part 1; in Part 2, respective rates were 6.7%/0% (Group A) and 10.6%/0% (Group B). Median ≥Grade 2 BCVA OAE/keratopathy resolution time was 1.1/1.0 months (Part 1) and 1.9/1.0 months (Part 2).

**Summary/Conclusion**

BelaDRd, with belamaf administered every 2 to 3 months, demonstrated robust clinical activity, with rapid and deep responses and high MRD negativity rates. Low frequency of ≥Grade 3 OAEs was observed and were rapidly resolved. Moreover, a similar frequency of OAEs was noted when dosing was guided by hematologist versus ophthalmologist ocular assessment. The remarkable PFS supports further evaluation of this quadruplet in a phase 3 study versus other novel quadruplet combinations in NDMM.

*Funding by GSK (NCT05280275); authors responsible for content.*

**Copyright Information:** (Online) ISSN: 2572-9241
©2026 The Author(s). HemaSphere is published by John Wiley & Sons Ltd on behalf of European Hematology Association. This is an open access Abstract Book distributed under the Attribution-NonCommercial-NoDerivs (CC BY-NC-ND), which allows third parties to download the articles and share them with others as long as they credit the author and the Abstract Book, but they cannot change the content in any way or use them commercially.
**Abstract Book Citations:** Authors, Title, HemaSphere, 2026;10:(S1):pages. The abstract book DOIs can be found on the "Author Information" page.
**Disclaimer:** Articles published in the journal HemaSphere exclusively reflect the opinions of the authors. The authors are responsible for all content in their abstracts including accuracy of the facts, statements, citing resources, etc.

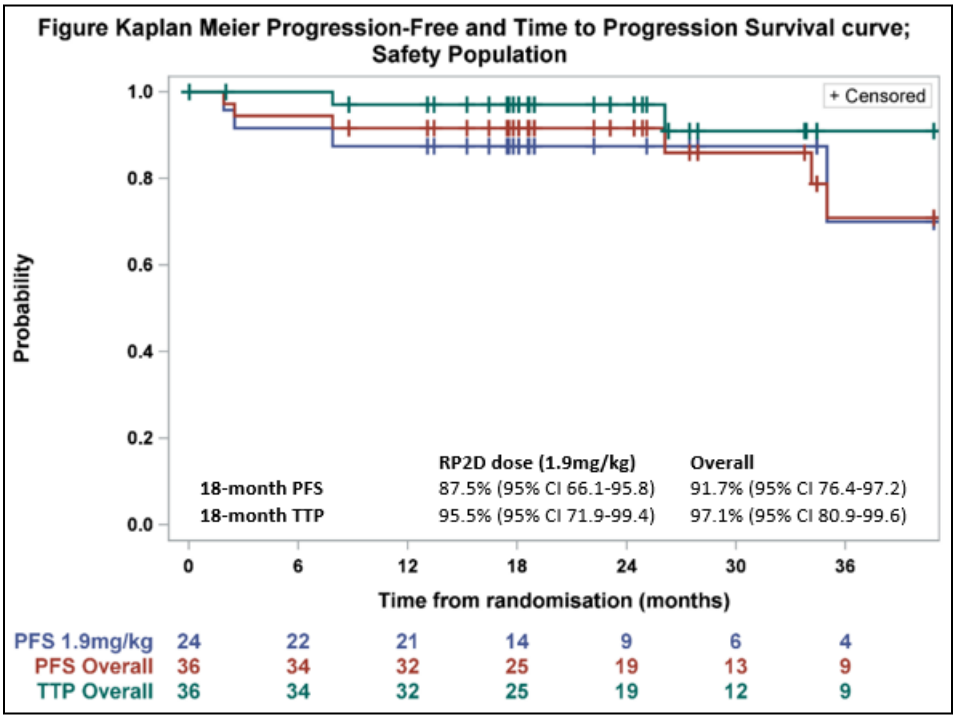

**Copyright Information:** (Online) ISSN: 2572-9241
©2026 The Author(s). HemaSphere is published by John Wiley & Sons Ltd on behalf of European Hematology Association. This is an open access Abstract Book distributed under the Attribution-NonCommercial-NoDerivs (CC BY-NC-ND), which allows third parties to download the articles and share them with others as long as they credit the author and the Abstract Book, but they cannot change the content in any way or use them commercially.
**Abstract Book Citations:** Authors, Title, HemaSphere, 2026;10:(S1):pages. The abstract book DOIs can be found on the "Author Information" page.
**Disclaimer:** Articles published in the journal HemaSphere exclusively reflect the opinions of the authors. The authors are responsible for all content in their abstracts including accuracy of the facts, statements, citing resources, etc.

### (S205) SAFETY AND EFFICACY OF ELRANATAMAB AS EARLY INTERVENTION IN PATIENTS WITH HIGH-RISK SMOLDERING MYELOMA: FIRST RESULTS FROM THE PHASE 2 ERASMM (EMN34) STUDY

**Topic:** 14. Myeloma and other monoclonal gammopathies - Clinical

Annemiek Broijl[1], Fredrik Schjesvold[2], Claudio Cerchione[3], Xavier Leleu[4], Renato Zambello[5], Carmine Liberatore[6, 7], Inger Nijhof[8], Elisa Dama[9], Thomas Chalopin[10], Pellegrino Musto[11, 12], Rossella Troia[9], Laura Paris[13], Chiara Pautasso[9], Silvia Mangiacavalli[14], Komivi Agbetsivi[15], Laure Vincent[16], Giulia Fumero[9], Elisabetta Antonioli[17], Gloria Margiotta Casaluci[18], Francesca Patriarca[19], Pieter Sonneveld[1, 20], Mario Boccadoro[9], Cyrille Touzeau*[21]

*[1]Department of Hematology, Erasmus MC Cancer Institute, Rotterdam, The Netherlands;[2]Oslo Myeloma Center, Department of Hematology, Oslo University Hospital, Oslo, Norway; [3]IRCCS Istituto Romagnolo per lo Studio dei Tumori (IRST) "Dino Amadori", Meldola, Italy; [4]Hématologie and Inserm CIC 1082, Poitiers, France; [5]Department of Medicine (DIMED), Hematology, Padua University, Padua, Italy; [6]Hematology Unit, Pescara Hospital, Pescara, Italy; [7]Department of Medicine and Aging Sciences, "G. D'Annunzio" University, Chieti, Italy; [8]Department of Internal Medicine - Hematology, St. Antonius Hospital, Nieuwegein, The Netherlands; [9]European Myeloma Network, EMN, Italy; [10]Hematology department, Hôpital Bretonneau, CHRU Tours, Tours, France; [11]Department of Precision and Regenerative Medicine and Ionian Area, "Aldo Moro" University School of Medicine, Bari, Italy; [12]Unit of Hematology and Stem Cell Transplantation, AOUC Policlinico, Bari, Italy; [13]UO Ematologia, ASST Papa Giovanni XXIII, Bergamo, Italy; [14]Division of Hematology, IRCCS Fondazione Policlinico San Matteo, Pavia, Italy; [15]Department of Internal Medicine, Centre Hospitalier de La Roche-sur-Yon, Les Oudairies 85000, La Roche-sur-Yon, France; [16]Department of Clinical Hematology, Montpellier University Hospital Center, Montpellier, France; [17]UO Ematologia, AOU Careggi, Firenze, Italy; [18]Division of Hematology, Department of Translational Medicine, Università del Piemonte Orientale/AOU Maggiore della Carità di Novara, Novara, Italy; [19]Hematologic Clinic and Transplant Center, University Hospital of Central Friuli, DMED, University of Udine, Udine, Italy; [20]European Myeloma Network, EMN, The Netherlands; [21]University Hospital of Nantes, Nantes, France;*

### Background

Early intervention with lenalidomide or daratumumab significantly delays disease progression to active disease in high-risk (HR) smoldering multiple myeloma (SMM) patients (pts). The BCMAxCD3 bispecific antibody elranatamab (Elra) demonstrated strong anti-MM activity with favorable safety profile in relapsed MM. Using T-cell engagers in the early stage of the disease, with a potentially less exhausted immune system, may lead to improved efficacy.

### Aims

Here we analyzed initial efficacy and safety results of Elra monotherapy in HR SMM pts enrolled in the phase 2 ERASMM Study (EMN34; NCT06183489).

### Methods

ERASMM is an international open-label multicenter phase 2 study of Elra as single agent in adult pts with HR SMM, defined as the presence of ≥2 Mayo 2018 "20-2-20" criteria. Treatment consisted of a fixed duration (2 years) of single-agent SC Elra with priming doses of 12 mg (C1D1) and 32 mg (C1D4), followed by 76 mg on days 8 and 15 during cycle 1, 1 and 15 during cycles 2–3, and 1 during cycles 4–24 (28-day cycle). The primary objective was the rate of complete response (CR). Safety was a key secondary endpoint. Other secondary objectives included overall response rate (ORR), measurable residual disease (MRD), progression-free survival (PFS), PFS2, time to active MM, and overall survival (OS). All patients gave written informed consent. Off-label use of drugs was involved. Pfizer provided the funding to conduct the ERASMM Study.

**Copyright Information:** (Online) ISSN: 2572-9241
©2026 The Author(s). HemaSphere is published by John Wiley & Sons Ltd on behalf of European Hematology Association. This is an open access Abstract Book distributed under the Attribution-NonCommercial-NoDerivs (CC BY-NC-ND), which allows third parties to download the articles and share them with others as long as they credit the author and the Abstract Book, but they cannot change the content in any way or use them commercially.
**Abstract Book Citations:** Authors, Title, HemaSphere, 2026;10:(S1):pages. The abstract book DOIs can be found on the "Author Information" page.
**Disclaimer:** Articles published in the journal HemaSphere exclusively reflect the opinions of the authors. The authors are responsible for all content in their abstracts including accuracy of the facts, statements, citing resources, etc.

**Results**

Fifty pts with previously untreated HR SMM were included from May 2024 to September 2025 in 17 centers collaborating with the European Myeloma Network. Median age was 65 years (range 58–71). All pts had HR SMM, including M-spike >20 g/L in 42 (84%), bone marrow plasma cells >20% in 36 (72%), or free-light chain ratio >20 in 33 (66%). As of December 4, 2025, the median follow-up was 10 months (range 2.3–18.23). Thirty-four (68%) patients experienced a cytokine release syndrome event, all grade 1–2, except for grade 3 events in 2 pts. No ICANS events were observed. Infections occurred in 25 (50%) pts, including 14% grade 3–4. Other common (≥20%) non-hematologic adverse events (AEs) were skin rash (34%), diarrhea (26%), AST/ALT increase (28%), fatigue (20%), and peripheral neuropathy (20%), mostly grade 1–2. Four patients discontinued treatment due to AEs, including AST/ALT increase (n=1), Guillain–Barré syndrome (n=1), peripheral neuropathy (n=1), and infections (n=1). The ORR was 92%, including a very good partial response or better (≥VGPR) in 82% of pts, and a complete response or better (≥CR) in 45%. The 9-month PFS and OS rates were 95% and 100%, respectively.

**Summary/Conclusion**

In HR SMM pts, single-agent Elra was highly active, with a 92% ORR, and 45% of pts achieving ≥CR. No new safety concerns related to Elra were observed. Data on safety and efficacy (response, MRD, PFS) will be updated for the meeting. These data support further investigation of Elra as a therapeutic option for HR SMM.

**Copyright Information:** (Online) ISSN: 2572-9241
©2026 The Author(s). HemaSphere is published by John Wiley & Sons Ltd on behalf of European Hematology Association. This is an open access Abstract Book distributed under the Attribution-NonCommercial-NoDerivs (CC BY-NC-ND), which allows third parties to download the articles and share them with others as long as they credit the author and the Abstract Book, but they cannot change the content in any way or use them commercially.
**Abstract Book Citations:** Authors, Title, HemaSphere, 2026;10:(S1):pages. The abstract book DOIs can be found on the "Author Information" page.
**Disclaimer:** Articles published in the journal HemaSphere exclusively reflect the opinions of the authors. The authors are responsible for all content in their abstracts including accuracy of the facts, statements, citing resources, etc.

### (S206) PHASE 3 RANDOMIZED TRIAL OF ANSELAMIMAB DEMONSTRATES IMPROVEMENT IN ALL-CAUSE MORTALITY IN Κ LIGHT-CHAIN AMYLOIDOSIS

**Topic:** 14. Myeloma and other monoclonal gammopathies - Clinical

Ashutosh Wechalekar*[1], Angela Dispenzieri[2], Vaishali Sanchorawala[3], Efstathios Kastritis[4], Divaya Bhutani[5], Giada Bianchi[6], Victor Jiminez Zepeda[7], Kenshi Suzuki[8], Suzanne Lentzsch[9], Antoine Huart[10], Alexander Carpinteiro[11], Scott Swenson[12], Zilehuma Khan[12], Julia Catini[12], Hrishikesh Kulkarni[12], Brian Meltzer[12], Michele Mercuri[12], Stephen Lake[12], Michaela Liedtke[13]

*[1]National Amyloidosis Centre and University College London Hospitals, London, United Kingdom; [2]Division of Hematology, Mayo Clinic, Rochester, United States of America; [3]Amyloidosis Center, Boston University School of Medicine, Boston Medical Center, Boston, United States of America; [4]National and Kapodistrian University of Athens, Department of Clinical Therapeutics, Athens, Greece; [5]Columbia University Medical Center, Department of Medicine, Division of Hematology, New York, United States of America; [6]Amyloidosis Program, Brigham and Women's Hospital, Dana-Farber Cancer Institute, Boston, United States of America; [7]Weill Cornell Medicine, New York, United States of America;[8]Japanese Red Cross Medical Center, Department of Hematology, Tokyo, Japan; [9]Herbert Irving Comprehensive Cancer Center, Columbia University, New York, United States of America; [10]University Hospital of Toulouse, Department of Nephrology and Organ Transplantation, Toulouse, France;[11]Institute of Molecular Biology, University of Duisburg-Essen, Essen, Germany; [12]Alexion, AstraZeneca Rare Disease, Quantitative Sciences, Boston, United States of America; [13]Stanford Amyloid Center, Stanford University School of Medicine, Palo Alto, United States of America;*

### Background

AL amyloidosis is a plasma cell dyscrasia (PCD) characterized by κ or λ Ig light chain amyloid fibrils that deposit in vital organs. Anselamimab is an investigational mAb designed to complement anti-PCD therapy through accelerated removal of amyloid fibrils.

### Aims

To investigate the efficacy and safety of anselamimab compared with placebo, in addition to anti-PCD therapy in the Cardiac Amyloid Reaching for Extended Survival (CARES) studies (NCT04512235 and NCT04504825).

### Methods

Alongside anti-PCD therapy with CyBorD (daratumumab was permitted) newly diagnosed patients with European modification of Mayo stage IIIa or IIIb AL amyloidosis were randomized 2:1 to receive anselamimab (1000 mg/m$^2$) or placebo (normal saline) IV every 7 days for 4 weeks followed by every 14 days for the remainder of the blinded study (18 months after the last participant enrolled). The primary endpoint was a hierarchical combination of time to all-cause mortality (ACM) and frequency of adjudicated cardiovascular hospitalizations (CVH) using the Finkelstein-Schoenfeld test and win-ratio in the combined study population. Subgroup analysis by involved κ or λ free light chain (iFLC) was prespecified.

**Copyright Information:** (Online) ISSN: 2572-9241
©2026 The Author(s). HemaSphere is published by John Wiley & Sons Ltd on behalf of European Hematology Association. This is an open access Abstract Book distributed under the Attribution-NonCommercial-NoDerivs (CC BY-NC-ND), which allows third parties to download the articles and share them with others as long as they credit the author and the Abstract Book, but they cannot change the content in any way or use them commercially.
**Abstract Book Citations:** Authors, Title, HemaSphere, 2026;10:(S1):pages. The abstract book DOIs can be found on the "Author Information" page.
**Disclaimer:** Articles published in the journal HemaSphere exclusively reflect the opinions of the authors. The authors are responsible for all content in their abstracts including accuracy of the facts, statements, citing resources, etc.

### Results

271 patients were randomized to receive anselamimab and 135 placebo. Baseline characteristics were similar between treatment arms. The median duration of treatment was 21.4 months and 21.1 in the anselamimab and placebo arms, respectively. Daratumumab was used in 79.3% and 83.7% of the anselamimab and placebo arms, respectively. Results of the primary efficacy analyses are shown in the **table**. Anselamimab-treated patients with κ iFLC had a 62% reduction in ACM (HR = 0.38; 95% CI, 0.17, 0.86; nominal P = 0.012; **figure**) and a 71% reduction in risk for CVH (estimated CVH rate ratio = 0.29; 95% CI, 0.10, 0.87; nominal P = 0.028). Patients with λ iFLC had a 10% reduction in risk for CVH; none in ACM. Safety data were comparable between both treatment arms. In anselamimab-treated patients, safety findings were similar between κ subgroup and full population.

**Table:** Summary of primary efficacy endpoints

| | **Overall** | | | **κ Isotype** | | | **λ Isotype** | | |
|---|---|---|---|---|---|---|---|---|---|
| **Endpoint** | **Anselamimab (N=271)** | **Placebo (N=135)** | ***P*-value** | **Anselamimab (N=48)** | **Placebo (N=24)** | ***P*-value** | **Anselamimab (N=219)** | **Placebo (N=109)** | ***P*-value** |
| ACM and CVH win ratio (95% CI) | 1.1 (0.8, 1.5) | | 0.332 | 2.06 (0.98, 4.31) | | 0.100 | 0.9 (0.6, 1.3) | | 0.848 |
| ACM: n (%) | 90 (33.2) | 52 (38.5) | | 15 (31.3) | 14 (58.3) | | 73 (33.3) | 37 (33.9) | |
| ACM: HR (95% CI) | 0.8 (0.6, 1.1) | | 0.290 | 0.38 (0.2, 0.9) | | 0.012 | 1.0 (0.7, 1.5) | | 0.647 |
| CVH: Frequency/year (95% CI) | 0.6 (0.4, 0.8) | 0.9 (0.6, 1.4) | 0.145 | 0.41 (0.2, 0.8) | 1.4 (0.6, 3.4) | 0.028 | 0.7 (0.4, 1.0) | 0.8 (0.4, 1.3) | 0.664 |
| CVH: Incidence risk ratio (95% CI) | 0.7 (0.4, 1.2) | | | 0.29 (0.1, 0.9) | | | 0.9 (0.5, 1.7) | | |

### Summary/Conclusion

Anselamimab, a κ light chain–directed anti-fibril monoclonal antibody used alongside standard of care chemotherapy, offers a first-in-class, new therapeutic option for patients with newly diagnosed κ AL amyloidosis.

© 2026 American Society of Clinical Oncology, Inc. Reused with permission. This abstract was accepted and previously presented at the 2026 ASCO Annual Meeting. All rights reserved.

**Copyright Information:** (Online) ISSN: 2572-9241
©2026 The Author(s). HemaSphere is published by John Wiley & Sons Ltd on behalf of European Hematology Association. This is an open access Abstract Book distributed under the Attribution-NonCommercial-NoDerivs (CC BY-NC-ND), which allows third parties to download the articles and share them with others as long as they credit the author and the Abstract Book, but they cannot change the content in any way or use them commercially.
**Abstract Book Citations:** Authors, Title, HemaSphere, 2026;10:(S1):pages. The abstract book DOIs can be found on the "Author Information" page.
**Disclaimer:** Articles published in the journal HemaSphere exclusively reflect the opinions of the authors. The authors are responsible for all content in their abstracts including accuracy of the facts, statements, citing resources, etc.

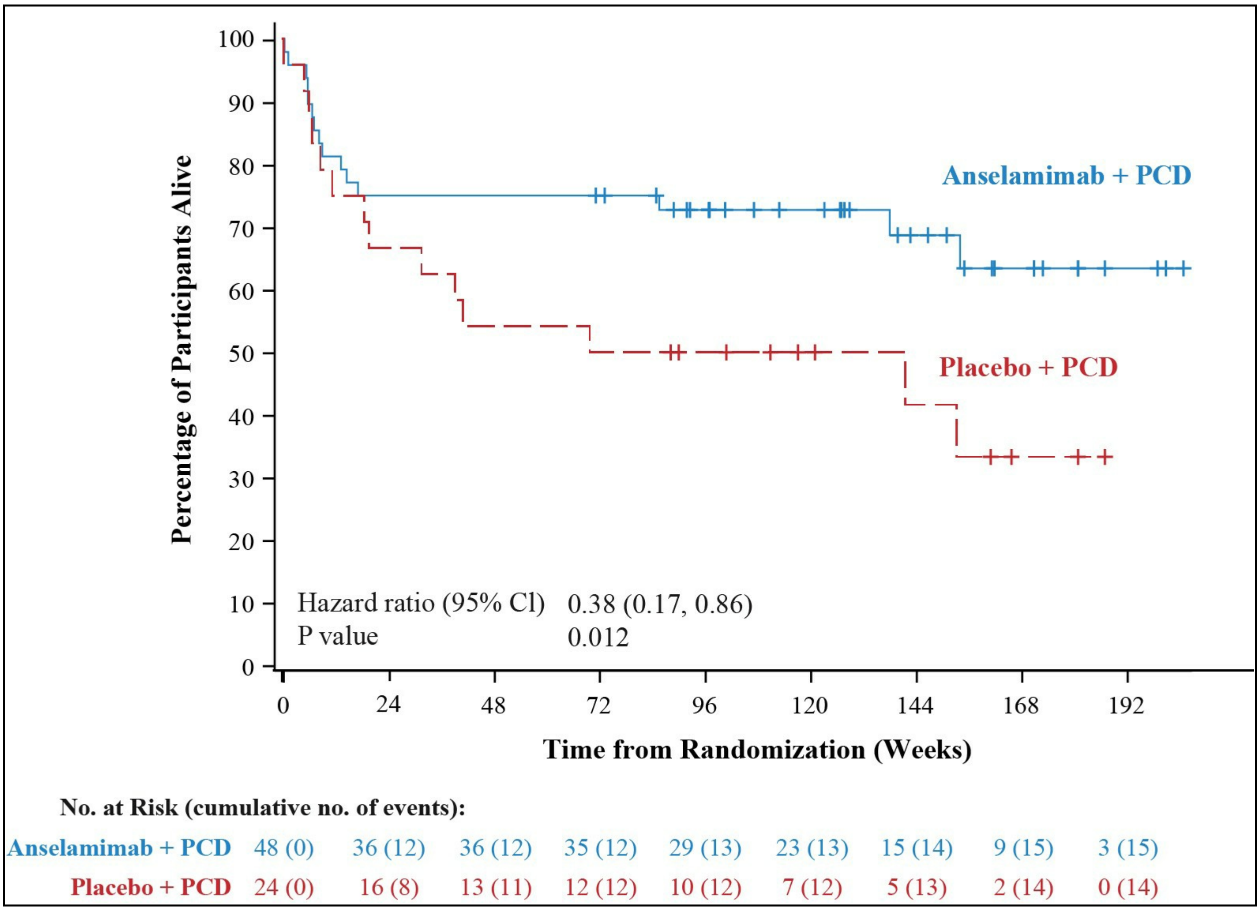

Kaplan-Meier curve of time to all-cause mortality in patients with κ isotype AL amyloidosis

**Copyright Information:** (Online) ISSN: 2572-9241
©2026 The Author(s). HemaSphere is published by John Wiley & Sons Ltd on behalf of European Hematology Association. This is an open access Abstract Book distributed under the Attribution-NonCommercial-NoDerivs (CC BY-NC-ND), which allows third parties to download the articles and share them with others as long as they credit the author and the Abstract Book, but they cannot change the content in any way or use them commercially.
**Abstract Book Citations:** Authors, Title, HemaSphere, 2026;10:(S1):pages. The abstract book DOIs can be found on the "Author Information" page.
**Disclaimer:** Articles published in the journal HemaSphere exclusively reflect the opinions of the authors. The authors are responsible for all content in their abstracts including accuracy of the facts, statements, citing resources, etc.

### (S207) AGGRESSIVE SMOLDERING CURATIVE APPROACH EVALUATING NOVEL THERAPIES (ASCENT): A PHASE 2 TRIAL OF INDUCTION, CONSOLIDATION, AND MAINTENANCE IN HIGH-RISK SMOLDERING MULTIPLE MYELOMA

**Topic:** 14. Myeloma and other monoclonal gammopathies - Clinical

Shaji Kumar*[1], Betsy Laplant[1], Ashraf Badros[2], Al-Ola Abdallah[3], Rafat Abonour[4], Erik Asmus[1], Binod Dhakal[5], Cara Rosenbaum[6], Daniel Egan[7], Manisha Bhutani[8], Andrzej Jakubowiak[9], Melissa Alsina[10]

*[1]Mayo Clinic, Rochester, United States of America; [2]University of Maryland, Baltimore, United States of America; [3]University of Kansas Medical Center, Kansas, United States of America; [4]Sylvester Cancer center, Miami, United States of America; [5]Medical College of Wisconsin, Milwaukee, United States of America; [6]AbbVie, Chicago, United States of America; [7]Swedish Center for Blood Disorders and Stem Cell Transplants, Seattle, United States of America; [8]Levine Cancer Institute, Advocate Health Wake Forest University School of Medicine, Department of Hematologic Oncology and Blood Disorders,, Charlotte, United States of America; [9]University of Chicago, Chicago, United States of America; [10]Moffitt, Miami, United States of America;*

### Background

The optimal approach for patients with smoldering multiple myeloma (SMM) at a high risk of progression remains an area of active investigation. Two phase 3 trials have demonstrated reduced risk of progression to active myeloma with lenalidomide, compared with observation alone. More recently, the phase 3 AQUILA trial demonstrated that daratumumab monotherapy reduced progression risk by 51% with improved overall survival compared to active monitoring. In addition to reducing the risk of progression, intense therapy applied at this precursor phase may potentially eradicate the malignant clone and lead to long term remissions. The ASCENT trial was designed to test this hypothesis.

### Methods

Patients with high-risk SMM by IMWG 20/2/20 staging system or a total score ≥9 on the IMWG scoring system were enrolled. Patients required adequate hematological and organ function and should have no evidence of amyloidosis. Primary endpoint was the rate of stringent complete response and secondary endpoints included MRD negativity rate and progression free survival. Treatment comprised induction (cycles 1-6), consolidation (cycles 7-12), and maintenance (cycles 13-24), with treatment limited to 24 cycles, each cycle being 28 days. Carfilzomib was given at 56 mg/m2 IV days 1, 8, 15 for cycles 1-12; lenalidomide was given at 25 mg PO daily days 1-21 for cycles 1-12 and then reduced to 10 mg; daratumumab was given at 16 mg/kg IV weekly for cycles 1-2, every other week cycles 3-6 and then once every cycle; and dexamethasone was given weekly at 40 mg for cycles 1-6 and 20 mg for cycles 7-12 and then discontinued. Disease assessments were performed by IMWG response criteria every cycle and MRD was assessed by Euroflow (sensitivity of $10^{-5}$) at end of induction, consolidation, and maintenance.

**Copyright Information:** (Online) ISSN: 2572-9241
©2026 The Author(s). HemaSphere is published by John Wiley & Sons Ltd on behalf of European Hematology Association. This is an open access Abstract Book distributed under the Attribution-NonCommercial-NoDerivs (CC BY-NC-ND), which allows third parties to download the articles and share them with others as long as they credit the author and the Abstract Book, but they cannot change the content in any way or use them commercially.
**Abstract Book Citations:** Authors, Title, HemaSphere, 2026;10:(S1):pages. The abstract book DOIs can be found on the “Author Information” page.
**Disclaimer:** Articles published in the journal HemaSphere exclusively reflect the opinions of the authors. The authors are responsible for all content in their abstracts including accuracy of the facts, statements, citing resources, etc.

### Results

Eighty-seven patients were enrolled on the trial between May 2018 and Dec 2021; median age was 64, 51% were male. At median follow-up of 52.4 (49.2-60.5) months (95%CI), 70 patients (80%) completed all 24 cycles; median number of cycles administered were 24, ranging 1 to 24. 17 patients went off study prior to 24 cycles; withdrawal by subject (3), adverse events (4), physician decision (2), progression (4), death (3), and unknown (1). Overall response rate was 97% (43 sCR/CR, 38 VGPR, 3 PR). MRD negativity was achieved in 74 patients (85%) at median of 6.6 months, 61% by end of induction, 67% by end of consolidation, and 68% at end of maintenance. 38 patients were persistently MRD negative at all time points, 5 patients became MRD positive after achieving MRD negativity. Ten patients progressed, with a median PFS not reached; estimated 5-year PFS rate is 82%. 5 patients have died; causes include 2 from Cardiac Arrest, 1 from Covid, 1 from Plasma cell leukemia/relapsed disease, and 1 Unknown. Any grade toxicity, considered possibly related to therapy, was observed in 84 patients. Grade 3 or higher hematological toxicity of any relation was seen in 22% patients and non-hematological toxicity in 70%.

### Summary/Conclusion

The quadruplet Dara-KRd combination demonstrate high rates of response similar to that observed in newly diagnosed MM, that seem to be maintained in majority of the patients. Toxicities are in line with that observed n newly diagnosed MM. MRD negativity was maintained in nearly two thirds of the patients with a medina follow up of more than 4 years. Future trials should explore such highly effective treatment approaches and the newer immunotherapies in this context.

**Copyright Information:** (Online) ISSN: 2572-9241
©2026 The Author(s). HemaSphere is published by John Wiley & Sons Ltd on behalf of European Hematology Association. This is an open access Abstract Book distributed under the Attribution-NonCommercial-NoDerivs (CC BY-NC-ND), which allows third parties to download the articles and share them with others as long as they credit the author and the Abstract Book, but they cannot change the content in any way or use them commercially.
**Abstract Book Citations:** Authors, Title, HemaSphere, 2026;10:(S1):pages. The abstract book DOIs can be found on the "Author Information" page.
**Disclaimer:** Articles published in the journal HemaSphere exclusively reflect the opinions of the authors. The authors are responsible for all content in their abstracts including accuracy of the facts, statements, citing resources, etc.

### (S208) INITIAL EFFICACY AND SAFETY DATA FROM THE PHASE 1/2 LINKER-AL2 STUDY OF LINVOSELTAMAB (LINVO) MONOTHERAPY IN PATIENTS WITH RELAPSED OR REFRACTORY (RR) SYSTEMIC LIGHT CHAIN (AL) AMYLOIDOSIS

**Topic:** 14. Myeloma and other monoclonal gammopathies - Clinical

Ashutosh Wechalekar*[1], Hans C. Lee[2], Giovanni Palladini[3], Jeffrey A. Zonder[4], Mahmoud R. Gaballa[5], Kihyun Kim[6], Henning Schade[7], M. Teresa Cibeira[8], Youngil Koh[9, 10], Javier De La Rubia Comos[11], Chang-Ki Min[12], Oluwaseun Aina[13], Benny Ren[13], Anita Boyapati[13], Anasuya Hazra[13], Shawn M. Sarkaria[13], Christopher J. Gamper[13], Umesh Chaudhari[13], Efstathios Kastritis[14, 15]

*[1]Univeristy College London Hospitals and National Amyloidosis Center, Royal Free London NHS Trust, London, United Kingdom; [2]Sarah Cannon Research Institute, Nashville, TN, United States of America;[3]Amyloidosis Research and Treatment Center, Fondazione IRCCS Policlinico San Matteo, Pavia, Italy; [4]Barbara Ann Karmanos Cancer Institute, Wayne State University School of Medicine, Detroit, MI, United States of America; [5]The University of Texas MD Anderson Cancer Center, Houston, TX, United States of America; [6]Sungkyunkwan University School of Medicine, Samsung Medical Center, Seoul, South Korea;[7]Colorado Blood Cancer Institute, Sarah Cannon Research Institute, Denver, CO, United States of America; [8]Amyloidosis and Myeloma Unit, Hospital Clínic Barcelona, IDIBAPS, Universitat de Barcelona, Barcelona, Spain; [9]Seoul National University Hospital, Seoul National University College of Medicine, Seoul, South Korea; [10]Seoul National University Cancer Institute, Seoul, South Korea; [11]Hospital Universitari i Politècnic La Fe, Universidad Católica de Valencia San Vicente Mártir, Instituto de Investigacíon Sanitaria La Fe, Valencia, Spain; [12]Catholic Hematology Hospital and Leukemia Research Institute, Seoul St. Mary's Hospital, The Catholic University of Korea, Seoul, South Korea; [13]Regeneron Pharmaceuticals, Inc., Tarrytown, NY, United States of America; [14]Alexandra Hospital, University of Athens, Athens, Greece; [15]National and Kapodistrian University of Athens, Athens, Greece;*

### Background

Systemic AL amyloidosis is a clonal plasma-cell disorder causing organ dysfunction through deposition of amyloid free light chains (FLCs). Patients (pts) are generally treated with drug combinations and there are no currently approved therapies for RR disease. Rapid hematologic and organ responses are associated with longer overall survival.

### Aims

To report initial efficacy and safety data from the Phase 1/2 LINKER-AL2 study (NCT06292780) of LINVO (human BCMA×CD3 bispecific antibody) monotherapy in RR systemic AL amyloidosis.

### Methods

Eligible adults with RR systemic AL amyloidosis who had received ≥1 prior therapy were treated with intravenous step-up doses of LINVO 5 mg (Cycle [C]0 Day [D]1) and 25 mg (C0D8), followed by 28-day cycles of 80 mg or 240 mg full doses, subcutaneously administered once-weekly in C1–C2 and then once every 4 weeks in C3–C12. The Phase 1 primary endpoint was incidence of dose-limiting toxicities (DLTs) within 28 days post-first full dose. Secondary endpoints included safety, rates of investigator-assessed hematologic complete response (hCR; by FLC ratio and absence of AL), overall response (hOR), very good partial response (hVGPR) or better, time to hCR, and assessment of organ involvement and response.

**Copyright Information:** (Online) ISSN: 2572-9241
©2026 The Author(s). HemaSphere is published by John Wiley & Sons Ltd on behalf of European Hematology Association. This is an open access Abstract Book distributed under the Attribution-NonCommercial-NoDerivs (CC BY-NC-ND), which allows third parties to download the articles and share them with others as long as they credit the author and the Abstract Book, but they cannot change the content in any way or use them commercially.
**Abstract Book Citations:** Authors, Title, HemaSphere, 2026;10:(S1):pages. The abstract book DOIs can be found on the "Author Information" page.
**Disclaimer:** Articles published in the journal HemaSphere exclusively reflect the opinions of the authors. The authors are responsible for all content in their abstracts including accuracy of the facts, statements, citing resources, etc.

### Results

As of Dec 1, 2025, 20 pts (median age, 64 years; European modified Mayo stage: II, 40%; IIIa, 35.0%) received LINVO 80 mg (n=7) or 240 mg (n=13), with a median follow-up of 5.13 months (range 1.6–9.2). Pts received a median of 1 prior line of therapy (range: 1–4); 60.0% received prior daratumumab-based therapy.

LINVO elicited a high rate of rapid and deep hematologic responses (**Table**), with mean involved FLC levels normalizing (iFLC <20 mg/L) by Day 15 (**Figure**). hCR rates were 71.4% (80 mg) and 84.6% (240 mg). As of Dec 16, 2025, of the pts with baseline cardiac and renal involvement, 4 of 8 and 8 of 10, respectively, achieved investigator-assessed, sponsor-validated organ response, most within 3 months.
No DLTs occurred. All pts had ≥1 treatment-emergent adverse event (TEAE; Grade (Gr)≥3, 55.0%). One Gr5 ventricular fibrillation event followed coronary artery stent placement in a pt with pre-existing coronary artery disease and was assessed as unrelated to LINVO. The most common TEAEs were infections (80.0%; Gr3/4, 25.0%; no Gr5), CRS (50.0%; Gr1, 35.0%; Gr2, 15.0%; 90% during step-up dosing), infusion-related reactions (30.0%; all Gr1/2), neutropenia (30.0%; Gr3/4, 25.0%), and transaminase elevations (30.0%; Gr3/4, 10.0%). One Gr 1 ICANS was reported (confusional state).

### Summary/Conclusion

In the absence of approved treatment options in the RR setting, LINVO monotherapy showed clinically meaningful activity, with high rates of hCR and organ responses, mean iFLC level normalization by Day 15, and generally manageable safety. These data support continued development of LINVO in pts with AL amyloidosis.

**Table. Investigator-assessed hematologic responses (ISA criteria)**

| **Response, n (%)** | **LINVO 80 mg (n=7)** | **LINVO 240 mg (n=13)** |
|---|---|---|
| hOR | 7 (100) | 13 (100) |
| hCR<br>Median time to hCR, weeks (range) | 5 (71.4)<br>4.3 (2.1–18.3) | 11 (84.6)<br>6.3 (1.0–12.1) |
| hVGPR | 2 (28.6) | 1 (7.7) |
| Low dFLC response* | 0 | 1 (7.7) |
| iFLC <10 mg/L<br>Median time to iFLC <10 mg/L, weeks (range) | 7 (100)<br>2.1 (2.0–10.3) | 13 (100)<br>2.7 (1.1–7.1) |
| **Median follow-up, months (range)** | 5.4 (1.6–9.2) | 4.6 (3.3–7.0) |

*If baseline dFLC ≥20 mg/L and <50 mg/L, response other than hCR reached with dFLC <10 mg/L.
dFLC, difference between iFLC and uninvolved FLC; ISA, International Society of Amyloidosis.
© 2026 American Society of Clinical Oncology, Inc. Reused with permission. This abstract was accepted and previously presented at the 2026 ASCO Annual Meeting. All rights reserved.

**Copyright Information:** (Online) ISSN: 2572-9241
©2026 The Author(s). HemaSphere is published by John Wiley & Sons Ltd on behalf of European Hematology Association. This is an open access Abstract Book distributed under the Attribution-NonCommercial-NoDerivs (CC BY-NC-ND), which allows third parties to download the articles and share them with others as long as they credit the author and the Abstract Book, but they cannot change the content in any way or use them commercially.
**Abstract Book Citations:** Authors, Title, HemaSphere, 2026;10:(S1):pages. The abstract book DOIs can be found on the "Author Information" page.
**Disclaimer:** Articles published in the journal HemaSphere exclusively reflect the opinions of the authors. The authors are responsible for all content in their abstracts including accuracy of the facts, statements, citing resources, etc.

**Figure. Mean iFLC over time by LINVO dose**

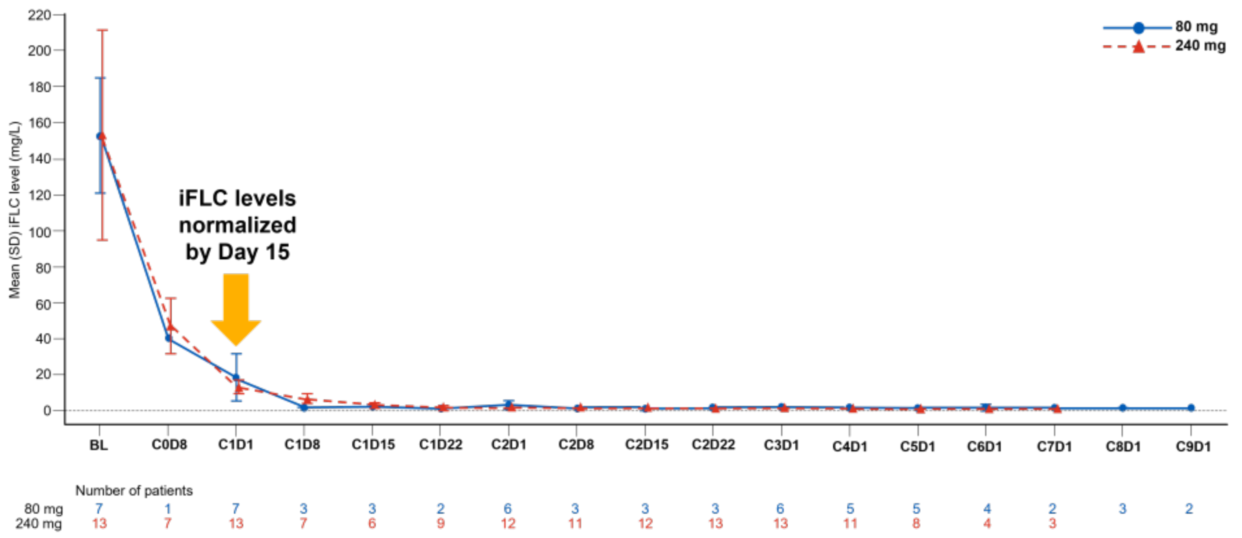

BL, baseline; C, Cycle, D, Day; iFLC, involved free light chain; LINVO, linvoseltamab; SD, standard deviation.

**Copyright Information:** (Online) ISSN: 2572-9241
©2026 The Author(s). HemaSphere is published by John Wiley & Sons Ltd on behalf of European Hematology Association. This is an open access Abstract Book distributed under the Attribution-NonCommercial-NoDerivs (CC BY-NC-ND), which allows third parties to download the articles and share them with others as long as they credit the author and the Abstract Book, but they cannot change the content in any way or use them commercially.
**Abstract Book Citations:** Authors, Title, HemaSphere, 2026;10:(S1):pages. The abstract book DOIs can be found on the “Author Information” page.
**Disclaimer:** Articles published in the journal HemaSphere exclusively reflect the opinions of the authors. The authors are responsible for all content in their abstracts including accuracy of the facts, statements, citing resources, etc.

### (S209) PHASE 1 DOSE ESCALATION SAFETY AND EFFICACY OF ETENTAMIG IN PATIENTS WITH RELAPSED OR REFRACTORY LIGHT CHAIN AMYLOIDOSIS

**Topic:** 14. Myeloma and other monoclonal gammopathies - Clinical

Efstathios Kastritis*[1], Antoine Huart[2], Rajshekhar Chakraborty[3], Arnaud Jaccard[4], Heather Landau[5], Shinsuke Iida[6], Olga Motorna[7], Tadao Ishida[8], Yawara Kawano[9], Manisha Bhutani[10], Anita D'souza[11], Andrew J Cowan[12], James E. Hoffman[13], Chetasi Talati[14], Tanya Rosenberg[14], Ross La Motte-Mohs[14], Fan Wang[14], David M Hoffman[14], Mingwei Fei[14], Emma Arriola[14], Faisal Rahman[14], Cara Rosenbaum[14], Jovian Yu[14], Shaji Kumar[15], Angela Dispenzieri[16], Giovanni Palladini[17], Vaishali Sanchorawala[18]

*[1]Department of Clinical Therapeutics, National and Kapodistrian University of Athens, School of Medicine, Athens, Greece; [2]Department of Nephrology and Organ Transplantation, University Hospital Toulouse, Toulouse, France;[3]Multiple Myeloma and Amyloidosis Program, Herbert Irving Comprehensive Cancer Center, Columbia University Irving Medical Center, New York, NY, United States of America; [4]Hematology Department, French Reference Center for AL Amyloidosis (Limoges-Poitiers), CHU Limoges, Limoges, France; [5]Department of Medicine, Memorial Sloan Kettering Cancer Center, New York, NY, United States of America; [6]Department of Hematology and Oncology, Nagoya City University Graduate School of Medical Sciences, Nagoya, Japan; [7]Department of Haematology, Eastern Health, and Eastern Clinical School, Monash University, Heidelberg, VIC, Australia; [8]Department of Hematology, Japanese Red Cross Medical Center, Tokyo, Japan;[9]Department of Hematology, Rheumatology, and Infectious Disease, Kumamoto University Graduate School of Medicine, Kumamoto, Japan; [10]Department of Hematologic Oncology and Blood Disorders, Levine Cancer Institute, Advocate Health Wake Forest University School of Medicine, Charlotte, NC, United States of America; [11]Division of Hematology/Oncology, Department of Medicine, Froedtert & Medical College of Wisconsin Cancer Center, Milwaukee, WI, United States of America; [12]University of Washington and Fred Hutchinson Cancer Center, Seattle, WA, United States of America; [13]University of Miami Health System, Miami, FL, United States of America; [14]AbbVie Inc., North Chicago, IL, United States of America; [15]Division of Hematology, Department of Internal Medicine, Mayo Clinic, Rochester, MN, United States of America; [16]Division of Hematology, Mayo Clinic, Rochester, MN, United States of America; [17]Department of Molecular Medicine, University of Pavia and Amyloidosis Research and Treatment Center, Foundation "Istituto di Ricovero e Cura a Carattere Scientifico Policlinico San Matteo,", Pavia, Italy; [18]Amyloidosis Center, Boston University School of Medicine and Boston Medical Center, Boston, MA, United States of America;*

### Background

B-cell maturation antigen (BCMA)-targeting immunotherapies represent a promising class of treatment for immunoglobulin light chain (AL) amyloidosis. Etentamig is being developed as a patient-centric, next generation differentiated BCMA x CD3 bispecific T-cell engager composed of a bivalent BCMA-binding domain with high avidity, low-affinity CD3-binding domain potentially reducing cytokine release syndrome (CRS), and a silenced Fc tail for extended half-life enabling convenient dosing from the start. Recent data from M24-209 (NCT06158854), an open-label Phase 1/2 study of etentamig monotherapy in patients (pts) with relapsed/refractory (RR) AL amyloidosis, demonstrated promising efficacy, with an 82% hematologic complete response (CR) rate and manageable safety profile with a 9% CRS rate and no immune effector cell-mediated neurotoxicity syndrome (ICANS).

### Aims

We report updated safety and efficacy results from M24-209.

**Copyright Information:** (Online) ISSN: 2572-9241
©2026 The Author(s). HemaSphere is published by John Wiley & Sons Ltd on behalf of European Hematology Association. This is an open access Abstract Book distributed under the Attribution-NonCommercial-NoDerivs (CC BY-NC-ND), which allows third parties to download the articles and share them with others as long as they credit the author and the Abstract Book, but they cannot change the content in any way or use them commercially.
**Abstract Book Citations:** Authors, Title, HemaSphere, 2026;10:(S1):pages. The abstract book DOIs can be found on the "Author Information" page.
**Disclaimer:** Articles published in the journal HemaSphere exclusively reflect the opinions of the authors. The authors are responsible for all content in their abstracts including accuracy of the facts, statements, citing resources, etc.

### Methods

The Phase 1 dose escalation (DE) portion of the study with etentamig enrolled BCMA-targeted therapy-naïve pts with RR AL amyloidosis with ≥1 prior line of therapy (LoT), including a proteasome inhibitor and anti-CD38 monoclonal antibody. Eligible pts had a difference in involved/uninvolved serum free light chain of ≥50 mg/L, and pts with high cardiac risk (Mayo stage IIIb) disease were excluded. Three dose groups were explored in DE (intravenous etentamig 20 mg, 40 mg, and 60 mg, all Q4W), with 1 step-up dose from 40 mg onward. Treatment duration is fixed for 24 months, and pts will be followed until disease progression. Primary objectives are to determine safety/tolerability, pharmacokinetics/pharmacodynamics, and recommended Phase 2 dose. Secondary objectives include efficacy as measured by hematologic response and organ response, duration of response (DoR), and time to response. Responses are investigator-assessed per consensus guidelines.

### Results

As of 22 November 2025, all doses in DE have been enrolled (N=34): 12, 11, and 11 pts have been treated with etentamig 20 mg, 40 mg, and 60 mg, respectively. Median (range) duration of follow-up in all pts was 10.1 (5.9–18.6) months; 88% of pts continued treatment. Median (range) age for pts was 70 (49–85) years. Median (range) number of prior LoT was 2 (1–8), and median (range) time from initial diagnosis was 4.5 (0–21) years. At baseline, 85% and 39% of pts had cardiac or renal involvement, respectively. Treatment-emergent adverse events were reported in 97% (53% Grade [G] ≥3) of all pts. No new CRS or ICANS events occurred compared with the previous data readout (**Figure**). Median (range) time to CRS onset and resolution was 6.9 (6.7–12.4) and 48.0 (8.8–150.0) hours, respectively. With longer safety follow-up, the manageable safety profile was maintained, with G≥3 infections in 2 (6%) pts and G≥3 neutropenia in 3 (9%) pts. Hypogammaglobulinemia occurred in 10 (29%, all G1/2) pts; all pts received ≥1 dose of IVIG. All pts achieved ≥very good partial response and 91% achieved CR. Median (range) time to CR was 1.0 (0.2–14.1) months. Median DoR has not yet been reached; no pts experienced hematologic progression. Renal responses were maintained at 54% with an increase in cardiac responses to 43% of evaluable pts. Subgroup analyses will be presented.

### Summary/Conclusion

With longer follow-up, etentamig monotherapy in pts with RR AL amyloidosis leads to 91% of CRs while maintaining its promising and manageable safety profile. These data support further investigation in the newly diagnosed AL amyloidosis setting.

**Figure.** Incidence of CRS

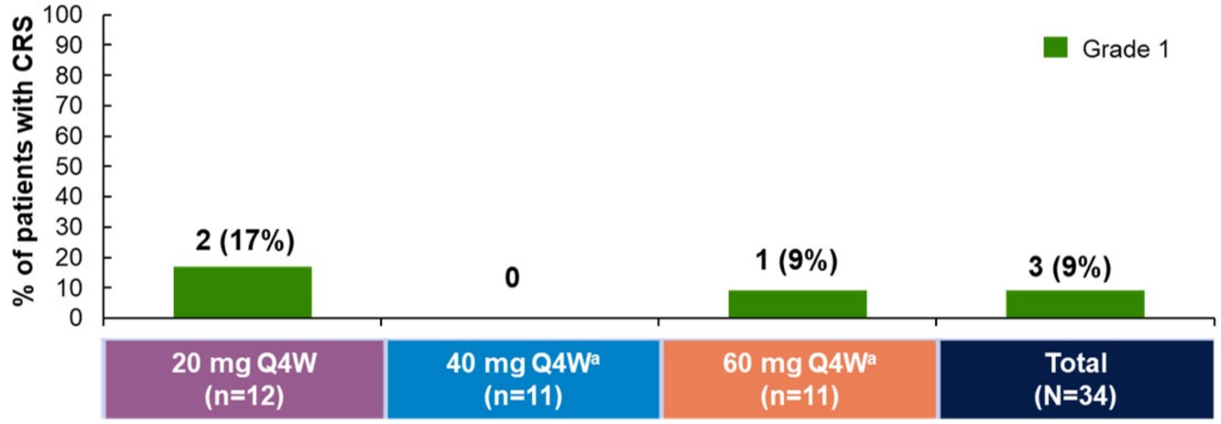

aStep-up dosing was implemented for 40 mg and 60 mg cohorts during cycle 1. Only Grade 1 CRS events were observed. CRS, cytokine release syndrome; Q4W, every 4 weeks.

**Copyright Information:** (Online) ISSN: 2572-9241
©2026 The Author(s). HemaSphere is published by John Wiley & Sons Ltd on behalf of European Hematology Association. This is an open access Abstract Book distributed under the Attribution-NonCommercial-NoDerivs (CC BY-NC-ND), which allows third parties to download the articles and share them with others as long as they credit the author and the Abstract Book, but they cannot change the content in any way or use them commercially.
**Abstract Book Citations:** Authors, Title, HemaSphere, 2026;10:(S1):pages. The abstract book DOIs can be found on the “Author Information” page.
**Disclaimer:** Articles published in the journal HemaSphere exclusively reflect the opinions of the authors. The authors are responsible for all content in their abstracts including accuracy of the facts, statements, citing resources, etc.

### (S210) DEVELOPMENT OF A G6B DIRECTED ANTIBODY DRUG CONJUGATE FOR THE TREATMENT OF MYELOPROLIFERATIVE NEOPLASMS

**Topic:** 15. Myeloproliferative neoplasms - Biology & translational research

Rafael Ioris[1], Yuqi Shen[2], Christina Simoglou Karali[2], Eileen McNeill[1], Vladimir Kiselev[1], Tung-Jui Trieu[1], Adam Mead*[2], Bethan Psaila[2], Rohit Batta[1]

*[1]Alethio Therapeutics, Abingdon, Oxford, United Kingdom; [2]Weatherall Institute of Molecular Medicine, Oxford, United Kingdom;*

### Background

Myeloproliferative neoplasms (MPN) are caused by mutations in JAK2, CALR (mutCALR) or MPL genes that activate JAK/STAT signalling and megakaryocyte-biased hematopoiesis. Clinically this manifests as essential thrombocythemia, polycythemia vera and myelofibrosis (MF). Current therapies improve blood counts and symptoms but are rarely disease-modifying, in part because they do not selectively target the mutant clone. Emerging cell-surface directed therapies, such as those against mutCALR, exemplify a new treatment class, but are applicable to only »25% of patients. There is a clear need for new therapies that target the MPN clone regardless of driver mutation.

### Aims

We sought to interrogate patient samples to identify cell-surface antigens that enable selective targeting of MPN stem/progenitor cells (HSPC) across genotypes. We determined whether targeting such antigens with an antibody-drug conjugate (ADC) could lead to selective ablation of mutant cells and offer disease-modifying potential.

### Methods

TARGET-seq[1] single-cell multi-omics was used to identify differentially expressed genes in mutant vs. wildtype HSPC[2]. Bespoke machine-learning algorithms[3] enabled prioritisation of targets suitable for ADCs. Targets were validated across a large patient cohort by orthogonal methods: flow cytometry, CyTOF, and scRNAseq. Screening of antibodies and payloads led to the design of ADCs that were tested for efficacy in disease-relevant models including cell lines, primary cells, cell-line engrafted marrow organoids[4] and xenografts.

### Results

Analysis of HSPC from MF patients with JAK2 V617F using TARGET-seq revealed 79 genes significantly overexpressed in mutant cells ($p<0.05$). Machine-learning prioritisation indicated the Ig-superfamily cell-surface receptor G6B as a potential therapeutic target. G6B expression is restricted megakaryocytes in healthy marrow but was aberrantly expressed in mutant HSPC in MPN. Flow, CyTOF and scRNAseq analysis of samples from >80 patients verified selective overexpression of G6B in patient cells, which correlated with JAK2/CALR variant allele frequency (Fig.1A).
Development of antibodies capable of G6B binding and internalisation, alongside screening of toxic payloads, led to the discovery of ALTM0230, an exatecan ADC. In HEL cells (JAK2 V617F), ALTM0230 had a cytotoxic $IC_{50}$ = 1.5 nM, that was maintained in human marrow organoids and patient-derived megakaryocyte precursors. ALTM0230 did not induce platelet cytotoxicity or platelet activation.
In HEL cell line xenografts, ALTM0230 5mg/Kg 2QWx6 was well tolerated and showed tumour growth inhibition with >2x increase in survival (Fig1B). In a splenic engrafted G6B+ AML-patient derived xenograft, ALTM0230 led to a >99% reduction of spleen and bone marrow tumour burden and normalisation of spleen weight.

**Copyright Information:** (Online) ISSN: 2572-9241
©2026 The Author(s). HemaSphere is published by John Wiley & Sons Ltd on behalf of European Hematology Association. This is an open access Abstract Book distributed under the Attribution-NonCommercial-NoDerivs (CC BY-NC-ND), which allows third parties to download the articles and share them with others as long as they credit the author and the Abstract Book, but they cannot change the content in any way or use them commercially.
**Abstract Book Citations:** Authors, Title, HemaSphere, 2026;10:(S1):pages. The abstract book DOIs can be found on the "Author Information" page.
**Disclaimer:** Articles published in the journal HemaSphere exclusively reflect the opinions of the authors. The authors are responsible for all content in their abstracts including accuracy of the facts, statements, citing resources, etc.

**Summary/Conclusion**

G6B is a unique target, differentially expressed in mutated HSPC across MPN driver mutations. Extensive validation shows that ALTM0230 causes potent and selective cytotoxicity against primary patient cells and in human marrow organoids, with improved survival and tumour burden reduction in multiple xenograft models. ADCs targeting G6B therefore have the potential to deliver disease modification to patients with MPN, irrespective of their driver mutation.

References:
1 A Rodriguez-Meira *et al.* 2019, Mol. Cell 73, 1292
2 B Psaila et al 2020, Mol. Cell 78, 477
3 V Kiselev & E Ainscow, bioRxiv, 2026.01.27.701959
4 AO Kahn *et al.* 2023 Cancer Discov. 13, 364

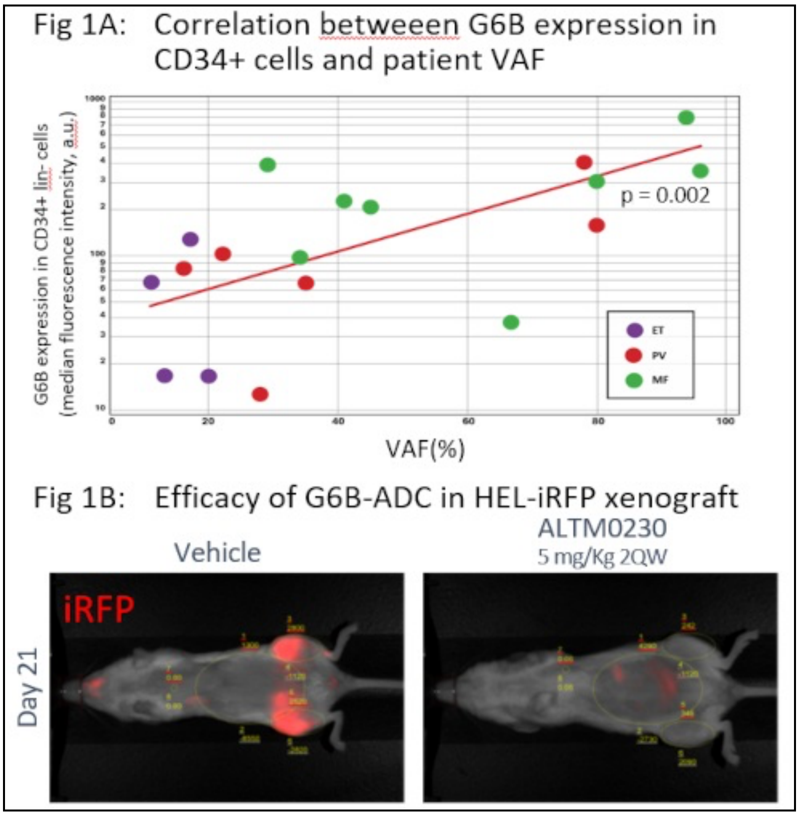

**Copyright Information:** (Online) ISSN: 2572-9241
©2026 The Author(s). HemaSphere is published by John Wiley & Sons Ltd on behalf of European Hematology Association. This is an open access Abstract Book distributed under the Attribution-NonCommercial-NoDerivs (CC BY-NC-ND), which allows third parties to download the articles and share them with others as long as they credit the author and the Abstract Book, but they cannot change the content in any way or use them commercially.
**Abstract Book Citations:** Authors, Title, HemaSphere, 2026;10:(S1):pages. The abstract book DOIs can be found on the "Author Information" page.
**Disclaimer:** Articles published in the journal HemaSphere exclusively reflect the opinions of the authors. The authors are responsible for all content in their abstracts including accuracy of the facts, statements, citing resources, etc.

### (S211) ONCOGENIC RAS EXPLOITS A FETAL-RESTRICTED CHROMATIN STATE TO INITIATE JMML VIA CEBPB ACTIVATION

**Topic:** 15. Myeloproliferative neoplasms - Biology & translational research

Giulia Orlando*[1], Sean Wen[1], Nicholas Fordham[1], Christopher Booth[1], Richard Salisbury[1], Eleni Louka[1], Christina Simoglou Karali[1], Ava Connolly[2], Orla Watson[2], Cristina Tormo Garcia[1], Nawshad Hayder[1], Natalina Elliott[1], Thomas Jackson[1], Alastair Smith[1], Joe Harman[1], Sally-Ann Clark[1], Maria Greco[1], Paola Vargas Gutierrez[1], Lai Cheng[1], Anupama Rao[3], Thomas Milne[1], Irene Roberts[1], Anindita Roy[1], Adam Mead[1]

*[1]University of Oxford, Medical Research Council (MRC) Weatherall Institute of Molecular Medicine (WIMM), Oxford, United Kingdom; [2]University of Oxford, Department of Oncology, Oxford, United Kingdom;[3]Great Ormond Street Hospital National Health Service Foundation Trust, Department of Haematology, London, United Kingdom;*

### Background

Juvenile myelomonocytic leukaemia (JMML) is a rare early-childhood myeloid neoplasm driven by activating mutations in RAS pathway genes (*NF1*, *KRAS*, *NRAS*, *PTPN11*, *CBL*). Evidence supports a prenatal acquisition of the initiating RAS-pathway lesion, yet the mechanistic basis for this developmental window of susceptibility remains unclear. As RAS mutations are almost always a secondary event in adult myeloid malignancies, we hypothesised that fetal haematopoietic stem/progenitor cells (HSPCs) possess an epigenetic landscape that is specifically susceptible to acquisition of RAS mutations.

### Aims

To define ontogeny-dependent epigenetic/transcriptional consequences of RAS activation in JMML HSPCs, identify RAS-driven transcription factor (TF) dependencies and test developmental timing of oncogenic RAS activation *in vivo*.

### Methods

We performed bulk and single-cell multi-omic profiling (paired RNA-seq and ATAC-seq) of human HSPCs spanning development and disease: fetal liver (FL, n=2), fetal bone marrow (FBM, n=2), adult bone marrow (n=3), cord blood controls (n=11), and primary JMML samples (n=12; *KRAS*/*NRAS*/*PTPN11*). Findings were functionally validated using a RAS-activated cell line (THP1), primary JMML cultures, and an inducible mouse model enabling temporally controlled activation of *KrasG12D* in HSCs during fetal (E14.5) versus postnatal (8-week) stages.

**Copyright Information:** (Online) ISSN: 2572-9241
©2026 The Author(s). HemaSphere is published by John Wiley & Sons Ltd on behalf of European Hematology Association. This is an open access Abstract Book distributed under the Attribution-NonCommercial-NoDerivs (CC BY-NC-ND), which allows third parties to download the articles and share them with others as long as they credit the author and the Abstract Book, but they cannot change the content in any way or use them commercially.
**Abstract Book Citations:** Authors, Title, HemaSphere, 2026;10:(S1):pages. The abstract book DOIs can be found on the "Author Information" page.
**Disclaimer:** Articles published in the journal HemaSphere exclusively reflect the opinions of the authors. The authors are responsible for all content in their abstracts including accuracy of the facts, statements, citing resources, etc.

**Results**

Single-cell TF activity analyses identified aberrant activation of C/EBP-family TFs (including CEBPB) together with NF-κB components, SPI1/PU.1 and STAT2 within JMML HSPC compartments. In contrast, canonical RAS-associated AP-1 (JUN/FOS) activity was limited in HSPC clusters and more evident in downstream erythroid populations, underscoring cell-type specificity. JMML leukaemic HSCs showed transcriptional similarity to FBM, yet displayed inappropriate activation of C/EBP-family programmes relative to FBM stem cells, consistent with RAS-driven escape from a fetal-restricted regulatory state. In THP1 cells, pharmacologic inhibition of RAS-pathway signalling reduced C/EBP-family TF activity and decreased phosphorylation of CEBPB, supporting a direct RAS-CEBPB regulatory link. Functionally, CEBPB knockout in primary JMML cultures suppressed a granulocyte–monocyte progenitor (GMP) transcriptional signature and reduced $CD34^+CD133^+$ HSPCs and $CD14^+/CD16^+$ monocytic output in liquid culture. *In vivo* developmental timing demonstrated that fetal (E14.5) *KrasG12D* induction produced shorter-latency, transplantable myelomonocytic leukaemia, whereas postnatal (8-week) induction more commonly resulted in T-cell leukaemias and only infrequently generated a transplantable myelomonocytic phenotype. These data indicate that developmental timing of oncogenic RAS dictates lineage output and transplantability, consistent with a fetal-specific permissive regulatory state for myelomonocytic transformation.

**Summary/Conclusion**

Together, these data support a model in which fetal chromatin state creates an ontogeny-specific vulnerability to initiating RAS-pathway mutations through limited baseline activation of CEBPB-driven myeloid stress programmes. Oncogenic RAS in fetal HSPCs can activate an aberrant CEBPB programme that promotes GMP/monocyte differentiation and JMML initiation, whereas postnatal activation more often yields alternative lineage outcomes and/or stem-cell attrition. More broadly, our findings demonstrate how developmental genome regulatory states can create stage-specific oncogenic vulnerabilities that underpin paediatric-onset cancers.

**Copyright Information:** (Online) ISSN: 2572-9241
©2026 The Author(s). HemaSphere is published by John Wiley & Sons Ltd on behalf of European Hematology Association. This is an open access Abstract Book distributed under the Attribution-NonCommercial-NoDerivs (CC BY-NC-ND), which allows third parties to download the articles and share them with others as long as they credit the author and the Abstract Book, but they cannot change the content in any way or use them commercially.
**Abstract Book Citations:** Authors, Title, HemaSphere, 2026;10:(S1):pages. The abstract book DOIs can be found on the “Author Information” page.
**Disclaimer:** Articles published in the journal HemaSphere exclusively reflect the opinions of the authors. The authors are responsible for all content in their abstracts including accuracy of the facts, statements, citing resources, etc.

### (S212) EVOLUTIONARY TRAJECTORY ANALYSIS REVEALS EARLY DETECTION OF LATENT HEMATOPOIETIC STEM CELLS FATED FOR AML IN MYELOFIBROSIS PATIENTS

**Topic:** 15. Myeloproliferative neoplasms - Biology & translational research

Jenny Ho*[1], Michelle Chan-Seng-Yue[2], Jessie Medeiros[3], Kulman Ethan[4], Alexander Murison[3], Amanda Mitchell[3], Andrea Arruda[3], James Kennedy[5], Anne Tierens[3], Dawn Maze[3], Hubert Tsui[5], Mark Minden[3], Quaid Morris[4], Vikas Gupta[3], John Dick[3]

*[1]Schulich School of Medicine & Dentistry, Western University, London, Canada;[2]Ontario Institute of Cancer Research, London, Canada; [3]University Health Network, Toronto, Canada; [4]Memorial Sloan Kettering Cancer Center, New York, United States of America; [5]Sunnybrook Health Sciences Centre, Toronto, Canada;*

### Background

In AML, we have a limited understanding of the leukemogenic steps that intervene between the formation of a leukemia cell of origin (LCO) – the first normal cell to acquire an early AML-initiating mutation – and the last pre-leukemic cell to acquire a late-appearing mutation that becomes a leukemia stem cell (LSC). Evidence suggests that LCO arise from hematopoietic stem cells (HSC)[1,2], while the origin of LSC is more obscure although predicted to be downstream progenitors[3,4]. Once final AML-driver mutations are acquired, it is generally assumed that transformation occurs rapidly and clinical diagnosis of AML quickly follows in the order of months, rather than years. The majority of AML are *de novo*, but some arise secondarily from myeloproliferative neoplasms (MPN); MPN patients with myelofibrosis (MF) are at highest risk for AML. Here, we tracked the origins of AML in serially collected MF samples of patients who transformed to AML.

### Aims

We aimed to define the hematopoietic stem and progenitor cell (HSPC) population that acquires leukemogenic mutations during the chronic phase of MF, capturing both the timing and order of acquisition.

### Methods

Nine MF patients with banked paired MPN and AML samples, including 4 with intervening MPN timepoints were used. Whole genome sequencing (WGS) of AML blasts, MPN clone, and germline control (T-cells/buccal DNA) identified driver and somatic mutations. AML-specific driver mutations were tracked in chronic phase by performing ddPCR in HSPC populations sorted from MPN timepoints. Additionally all sorted fractions were subjected to targeted sequencing (tarseq) of AML-specific (driver and passenger) mutations. Pairtree[5] analysis of tarseq results was used to create evolutionary clonal trajectories and identify putative cells of transformation.

### Results

Twenty-one AML-specific driver genes were mutated (average 5.3; range 2-8 genes mutated/patient) and 73% of these mutations were already present in chronic phase at ≥5% variant allele frequency; and were thus termed early appearing. While the remaining 27% of mutations were present at <5% and were defined as late appearing AML-driver mutations. Using ddPCR and tarseq, the late AML-driver mutations were detectable at low frequencies (0.2-5%) in one or more HSPC populations several years (1.5-3 years) before AML diagnosis. In 6 patients, late AML-driver mutations were identified within the HSC (n=5) or MPP (n=1) population. Pairtree analysis was able to establish the cell of transformation in 5 patients. In 4 patients, the transforming cell was either an HSC (n=3) or MPP (n=1); while in another patient, the cell of transformation was a LMPP/MLP.

**Copyright Information:** (Online) ISSN: 2572-9241
©2026 The Author(s). HemaSphere is published by John Wiley & Sons Ltd on behalf of European Hematology Association. This is an open access Abstract Book distributed under the Attribution-NonCommercial-NoDerivs (CC BY-NC-ND), which allows third parties to download the articles and share them with others as long as they credit the author and the Abstract Book, but they cannot change the content in any way or use them commercially.
**Abstract Book Citations:** Authors, Title, HemaSphere, 2026;10:(S1):pages. The abstract book DOIs can be found on the "Author Information" page.
**Disclaimer:** Articles published in the journal HemaSphere exclusively reflect the opinions of the authors. The authors are responsible for all content in their abstracts including accuracy of the facts, statements, citing resources, etc.

**Summary/Conclusion**

Clones that have acquired all AML-driver mutations, termed AML-fated clones, can arise years before AML diagnosis; but remain in a latent pre-leukemic phase until AML presentation. This strongly suggests that mechanisms beyond the acquisition of somatic mutations are necessary to effectively promote full leukemic transformation. We also show that AML-fated clones are primitive and derive from an HSC/MPP; and in many cases the cell that transforms into an LSC is also an HSC/MPP and not a downstream progenitor. We propose that MF-derived AML represents a case of true stem cell leukemia and perhaps this contributes to their uniquely poor clinical outcomes.

References:
1. Shlush 2014. PMID: 24522528.
2. Corces-Zimmerman 2014. PMID: 24550281.
3. Goardon 2011. PMID: 21251617.
4. Quek 2016. PMID: 27377587.
5. Wintersinger 2022. PMID: 35247865.

**Copyright Information:** (Online) ISSN: 2572-9241
©2026 The Author(s). HemaSphere is published by John Wiley & Sons Ltd on behalf of European Hematology Association. This is an open access Abstract Book distributed under the Attribution-NonCommercial-NoDerivs (CC BY-NC-ND), which allows third parties to download the articles and share them with others as long as they credit the author and the Abstract Book, but they cannot change the content in any way or use them commercially.
**Abstract Book Citations:** Authors, Title, HemaSphere, 2026;10:(S1):pages. The abstract book DOIs can be found on the "Author Information" page.
**Disclaimer:** Articles published in the journal HemaSphere exclusively reflect the opinions of the authors. The authors are responsible for all content in their abstracts including accuracy of the facts, statements, citing resources, etc.

### (S213) CD9 MARKS THE DISEASE-PROPAGATING HEMATOPOIETIC STEM CELL POPULATION IN MYELOFIBROSIS

**Topic:** 15. Myeloproliferative neoplasms - Biology & translational research

Lara Tavernari*[1, 2], Ruggiero Norfo[1, 2], Anita Neroni[1, 3], Camilla Tombari[1, 2], Sebastiano Rontauroli[1, 2], Margherita Mirabile[1, 2], Elena Genovese[1], Monica MacCaferri[4], Giulia Grisendi[5], Chiara Carretta[1, 2], Sandra Parenti[1, 2], Luca Fabbiani[5], Matteo Bertesi[1, 3], Elisa Papa[1, 3], Marica Malerba[1, 3], Leonardo Potenza[4, 5], Lorena Losi[6], Paola Guglielmelli[7, 8], Massimo Dominici[5], Mario Luppi[4, 5], Enrico Tagliafico[5, 9], Alessandro Maria Vannucchi[7, 8], Rossella Manfredini[1, 2]

*[1]Interdepartmental Centre for Stem Cells and Regenerative Medicine, University of Modena and Reggio Emilia, Modena, Italy; [2]Department of Biomedical, Metabolic and Neural Sciences, University of Modena and Reggio Emilia, Modena, Italy; [3]Department of Life Sciences, University of Modena and Reggio Emilia, Modena, Italy;[4]Department Oncology and Hematology, Hematology Unit, Modena University Hospital, Modena, Italy; [5]Department of Medical and Surgical Sciences of Children & Adults, University of Modena and Reggio Emilia, Modena, Italy; [6]Department of Life Sciences, Pathology Unit, University of Modena and Reggio Emilia, Modena, Italy; [7]CRIMM, Center Research and Innovation of Myeloproliferative Neoplasms, University of Florence, AOU Careggi, Florence, Italy; [8]Department of Experimental and Clinical Medicine, University of Florence, Florence, Italy; [9]Department of Laboratory Medicine, Diagnostic Hematology and Clinical Genomics Unit, Modena University Hospital, Modena, Italy;*

### Background

Myeloproliferative neoplasms are clonal hematopoietic disorders, among which myelofibrosis (MF) represents the most aggressive form. MF is characterized by progressive bone marrow fibrosis and a median overall survival of approximately 5 years. MF arises from a hematopoietic stem and progenitor cell (HSPC) that acquires somatic driver mutations in JAK2, CALR, or MPL, followed by additional lesions promoting clonal dominance over wild-type hematopoiesis. Current therapies, including JAK inhibitors, provide clinical benefit but fail to eradicate the malignant HSPC compartment or achieve molecular remission.

### Aims

To characterize the stemness and disease-propagating potential of $CD9^+$ HSPCs in myelofibrosis and evaluate CD9 as a marker of malignant stem cells.

### Methods

HSPCs were purified from peripheral blood samples of MF patients harboring JAK2, CALR, or MPL driver mutations and healthy donors (HDs). RNA sequencing was performed on purified HSPCs (13 MF, 5 HDs) to identify surface molecules preferentially expressed in MF. Candidate markers were subsequently validated by flow cytometry in an expanded cohort (30 MF, 15 HDs). To assess whether CD9 expression identified the neoplastic stem cell population, single-cell mutational profiling coupled with surface protein analysis was performed in 8 MF patients using the Tapestri platform (Mission Bio). CD9+ and CD9- HSPCs from MF patients and HDs were FACS-sorted and functionally characterized through clonogenic and differentiation assays *in vitro*, including long-term culture-initiating cell (LTC-IC) assays. Patient-derived xenograft (PDX) models were established by transplanting CD9+ or CD9- HSPCs into sub-lethally irradiated NSGS immunodeficient mice to evaluate their disease-propagating and repopulating capacity. Due to the limited long-term maintenance of human CD34+ cells in NSGS mice, we tested an alternative immunodeficient mouse strain, which improved *in vivo* characterization of human HSCs in MF.

**Copyright Information:** (Online) ISSN: 2572-9241
©2026 The Author(s). HemaSphere is published by John Wiley & Sons Ltd on behalf of European Hematology Association. This is an open access Abstract Book distributed under the Attribution-NonCommercial-NoDerivs (CC BY-NC-ND), which allows third parties to download the articles and share them with others as long as they credit the author and the Abstract Book, but they cannot change the content in any way or use them commercially.
**Abstract Book Citations:** Authors, Title, HemaSphere, 2026;10:(S1):pages. The abstract book DOIs can be found on the "Author Information" page.
**Disclaimer:** Articles published in the journal HemaSphere exclusively reflect the opinions of the authors. The authors are responsible for all content in their abstracts including accuracy of the facts, statements, citing resources, etc.

**Results**

Transcriptomic profiling identified CD9 among the most upregulated surface molecules in MF HSPCs compared to HDs. Flow cytometry validation confirmed a significant expansion of the CD9+ HSPC compartment in MF. Single-cell analysis showed that most driver mutation-positive HSPCs clustered within the CD9+ fraction, indicating enrichment of the malignant clone. *In vitro*, MF-derived CD9+ HSPCs displayed a more primitive phenotype and enhanced clonogenic potential. Notably, in LTC-IC assays, CD9+ MF HSPCs generated a higher number of colonies after 8 weeks of culture, supporting enrichment in primitive stem cells within this fraction. *In vivo*, MF CD9+ HSPCS exhibited higher engraftment levels across hematopoietic tissues compared to mice receiving CD9- counterparts in NSGS mice. In the newly developed immunodeficient MF-PDX model, CD9+ retained a greater number of human stem cells than their CD9- in the bone marrow confirming their enhanced stemness and disease-propagating potential.

**Summary/Conclusion**

Across MF patients with different driver mutations, CD9 marks an expanded, mutation-enriched HSPC population with enhanced clonogenic capacity and disease-propagating potential. These findings identify CD9 as a promising surface marker of MF stem/progenitor cells and highlight is potential as a therapeutic target for eradicating the malignant clone.

**Copyright Information:** (Online) ISSN: 2572-9241
©2026 The Author(s). HemaSphere is published by John Wiley & Sons Ltd on behalf of European Hematology Association. This is an open access Abstract Book distributed under the Attribution-NonCommercial-NoDerivs (CC BY-NC-ND), which allows third parties to download the articles and share them with others as long as they credit the author and the Abstract Book, but they cannot change the content in any way or use them commercially.
**Abstract Book Citations:** Authors, Title, HemaSphere, 2026;10:(S1):pages. The abstract book DOIs can be found on the "Author Information" page.
**Disclaimer:** Articles published in the journal HemaSphere exclusively reflect the opinions of the authors. The authors are responsible for all content in their abstracts including accuracy of the facts, statements, citing resources, etc.

### (S214) TRANSCRIPTIONAL SUBTYPES OF MYELOFIBROSIS ARE INDEPENDENT OF DRIVER MUTATIONS AND CAPTURE DISTINCT BIOLOGY, OUTCOMES, AND DISEASE TRAJECTORIES

**Topic:** 16. Myeloproliferative neoplasms - Clinical

Andy Zeng*[1, 2], Jessie Medeiros[1, 2], Amulya Bhagirath[1], Suraj Bansal[1], Sagar Kothari[1], Hyerin Kim[1], Andrea Arruda[1], Jaime Claudio[1], Dawn Maze[1], Hassan, Sibai[1], Mark Minden[1], Jean Wang[1, 3, 4], James Kennedy[5], John Dick[1, 2], Vikas Gupta[1]

*[1]Princess Margaret Cancer Centre, Toronto, Canada;[2]University of Toronto, Department of Molecular Genetics, Toronto, Canada; [3]University Health Network, Division of Medical Oncology and Hematology, Toronto, Canada;[4]University of Toronto, Department of Medicine, Toronto, Canada; [5]Sunnybrook Health Sciences Centre, Medical Oncology and Hematology, Toronto, Canada;*

### Background

Myelofibrosis (MF) is heterogeneous with respect to clinical presentation and outcomes. This heterogeneity cannot be fully explained by mutation profiles, suggesting that non-genetic factors, including differentiation state, microenvironment, and epigenetic factors, may contribute to disease phenotypes. We reasoned that genetic and non-genetic sources of heterogeneity may converge to produce gene expression programs detectable in peripheral blood samples, which could be utilized to define disease subtypes within MF.

### Methods

Whole-transcriptome sequencing of peripheral blood was performed in a 358-patient discovery cohort and an independent 168-patient validation cohort. Non-negative matrix factorization identified biologically relevant gene modules, which were used for feature selection and consensus clustering. Validation samples were classified using a k-nearest-neighbours classifier. Twenty MF-to-secondary AML (sAML) pairs were profiled to assess for persistence of subtype biology from MF into sAML.

### Results

Four transcriptionally and clinically distinct subtypes were identified: MF-Immune (n=116; T/B-cell programs; lowest disease burden), MF-Erythroid (n=72; erythroblast programs; anemia and multi-lineage cytopenias), MF-Myeloid (n=88; granulocyte/monocyte/dendritic cell programs; leukocytosis with left shift), and MF-MEP (n=82; megakaryocyte-erythroid progenitor programs; highest disease burden). Subtypes differed significantly in blood counts, cytopenias, peripheral blast percentage, fibrosis grade, and splenomegaly (all p<0.001).
Subtype identity was independent of canonical driver mutation status (JAK2, CALR, MPL; p=0.89). Associations with high-molecular-risk mutation burden were observed (p=3e-6), but this did not fully account for subtype identity. Five-year survival outcomes differed: 85% (MF-Immune), 58% (MF-Erythroid), 38% (MF-Myeloid), and 26% (MF-MEP) (p=3e-13, Fig. 1A). This was confirmed after adjustment for DIPSS (p=8e-3) and MIPSS70 (p=6e-4) risk scores. Importantly, subtype-defining biology, clinical correlates, and survival differences were reproduced in our independent validation cohort of 168 patients (Fig. 1B).
In a pooled competing-risks analysis, the five-year risk of transformation to AML also differed: 20% (MF-MEP), 19% (MF-Myeloid), 5% (MF-Erythroid), and 5% (MF-Immune) (p=4e-4, Fig. 1C). Relative to MF-Immune, the MF-Erythroid subtype had an elevated risk of death in MF without elevated transformation risk (Fig. 1C-D), highlighting distinct disease trajectories by MF subtype.
In 20 paired MF-to-sAML samples, antecedent MF subtype biology persisted into blast phase: MF-Myeloid-derived sAML was enriched for myelo-lymphoid progenitor states, while MF-MEP-derived sAML retained enrichment for megakaryocyte-erythroid progenitor states. Gene set enrichment analysis showed that MF-Myeloid-derived sAML harbours transcriptional features previously associated with sensitivity to azacitidine and venetoclax (p=9e-5), raising the potential for subtype-informed therapeutic selection.

**Copyright Information:** (Online) ISSN: 2572-9241
©2026 The Author(s). HemaSphere is published by John Wiley & Sons Ltd on behalf of European Hematology Association. This is an open access Abstract Book distributed under the Attribution-NonCommercial-NoDerivs (CC BY-NC-ND), which allows third parties to download the articles and share them with others as long as they credit the author and the Abstract Book, but they cannot change the content in any way or use them commercially.
**Abstract Book Citations:** Authors, Title, HemaSphere, 2026;10:(S1):pages. The abstract book DOIs can be found on the "Author Information" page.
**Disclaimer:** Articles published in the journal HemaSphere exclusively reflect the opinions of the authors. The authors are responsible for all content in their abstracts including accuracy of the facts, statements, citing resources, etc.

### Summary/Conclusion

Transcriptional subtyping identifies four biologically and clinically distinct MF subtypes independent of driver mutation status, with different prognoses and disease trajectories. sAML retained transcriptional features of the antecedent MF subtype, suggesting that disease history in chronic phase shapes biology after AML transformation. Collectively, these findings provide a framework for precision medicine in myelofibrosis.

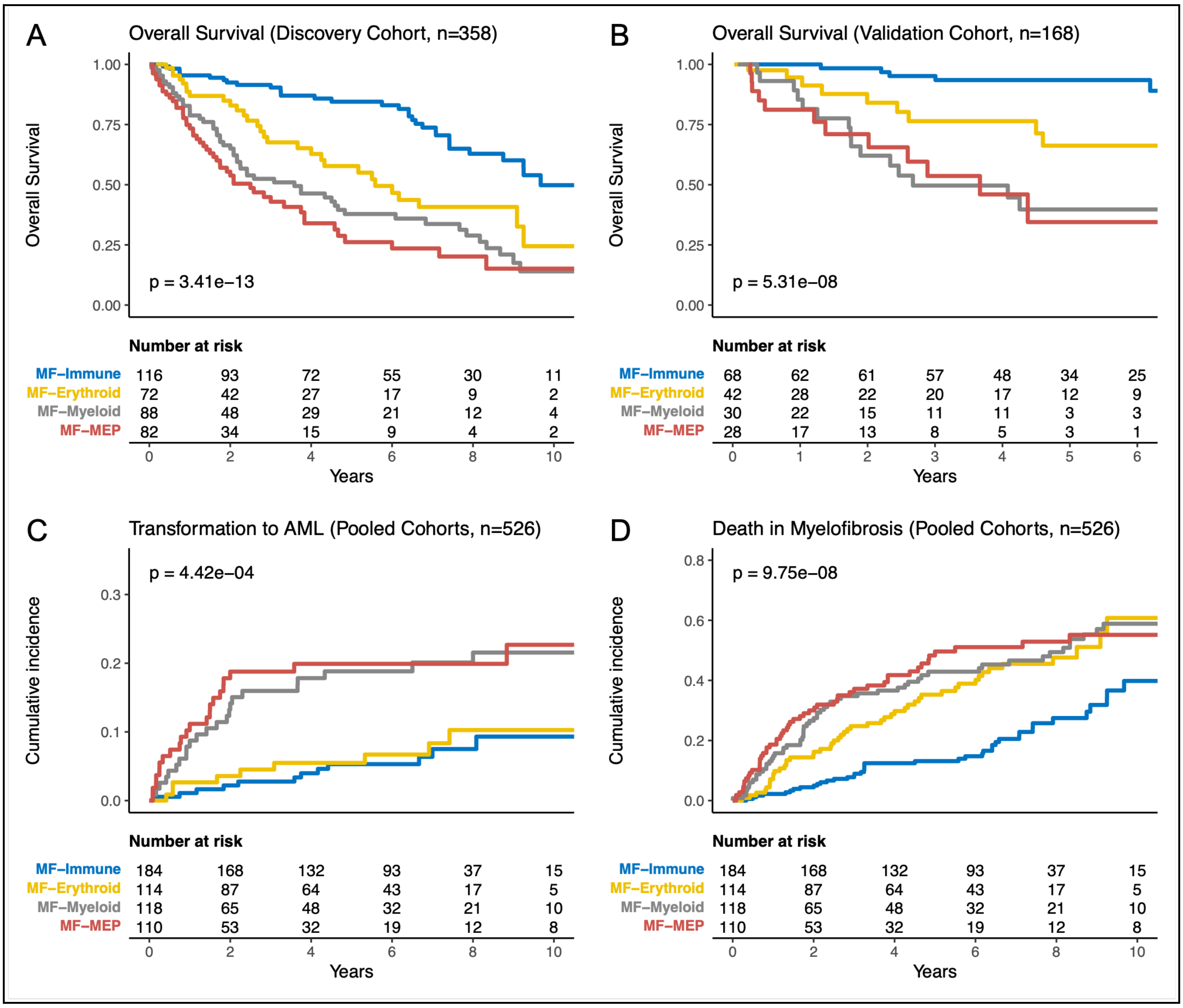

Figure 1. Distinct outcomes and disease trajectories of transcriptional subtypes in myelofibrosis. (A-B) Overall survival outcomes within the (A) discovery cohort (n=358) and the (B) validation cohort (n=168) across transcriptional subtypes. (C-D) Pooled competing risks analysis of both cohorts (n=526) comparing cumulative incidence of (C) transformation to AML versus (D) death in myelofibrosis across transcriptional subtypes.

**Copyright Information:** (Online) ISSN: 2572-9241
©2026 The Author(s). HemaSphere is published by John Wiley & Sons Ltd on behalf of European Hematology Association. This is an open access Abstract Book distributed under the Attribution-NonCommercial-NoDerivs (CC BY-NC-ND), which allows third parties to download the articles and share them with others as long as they credit the author and the Abstract Book, but they cannot change the content in any way or use them commercially.
**Abstract Book Citations:** Authors, Title, HemaSphere, 2026;10:(S1):pages. The abstract book DOIs can be found on the “Author Information” page.
**Disclaimer:** Articles published in the journal HemaSphere exclusively reflect the opinions of the authors. The authors are responsible for all content in their abstracts including accuracy of the facts, statements, citing resources, etc.

### (S215) EFFICACY AND SAFETY OF LUSPATERCEPT IN PATIENTS WITH MYELOFIBROSIS ON JANUS KINASE INHIBITORS WHO REQUIRE RED BLOOD CELL TRANSFUSIONS: PRIMARY ANALYSIS OF THE PHASE 3 INDEPENDENCE TRIAL

**Topic:** 16. Myeloproliferative neoplasms - Clinical

Francesco Passamonti*[1], Jean-Jacques Kiladjian[2], John Mascarenhas[3], Claire Harrison[4], Ruben Mesa[5], Elisabetta Abruzzese[6], Harry Gill[7], Stefan Wickenhauser[8], Je-Hwan Lee[9], Zhijian Xiao[10], Fabrizio Pane[11], Suning Chen[12], Chunting Zhao[13], Argiris Symeonidis[14], Fabian Sanabria[15], Julie Vienne Bürki[15], Ana Carolina Giuseppi[16], Jiahui Li[16], Thalia Farazi[16], Haifa Kathrin Al-Ali[17]

*[1]Università di Milano Statale, Policlinico di Milano, Ospedale Maggiore, Fondazione I.R.C.C.S. Ca Granda, Milano, Italy;[2]Hôpital Saint-Louis et Université Paris Diderot, Paris, France; [3]Icahn School of Medicine, New York, NY, United States of America;[4]Guy's and St Thomas' NHS Foundation Trust, London, United Kingdom; [5]Atrium Health Wake Forest Baptist Comprehensive Cancer Center, Winston-Salem, NC, United States of America; [6]Ospedale S. Eugenio, Division of Hematology, Roma, Italy;[7]School of Clinical Medicine, the University of Hong Kong, Hong Kong, China; [8]CHU de Nîmes, Nîmes, France; [9]Asan Medical Center, University of Ulsan College of Medicine, Seoul, South Korea; [10]National Clinical Research Center for Blood Diseases, Haihe Laboratory of Cell Ecosystem, Institute of Hematology & Blood Diseases Hospital, Chinese Academy of Medical Sciences & Peking Union Medical College, Tianjin, China; [11]Federico II University Medical School, Department of Clinical Medicine and Surgery, Naples, Italy; [12]The First Affiliated Hospital of Soochow University, Suzhou, China;[13]The Affiliated Hospital of Qingdao University, Qingdao, China; [14]University of Patras, Patras, Greece; [15]Bristol Myers Squibb, Boudry, Switzerland; [16]Bristol Myers Squibb, Princeton, NJ, United States of America; [17]Universitätsklinikum Halle (Saale), Halle, Germany;*

### Background

Patients (pts) with myelofibrosis (MF) develop progressive anemia that leads to red blood cell transfusion (RBCT) dependence—both are strong predictors of poor outcomes and reduced survival. There is a substantial unmet need for effective anemia-directed therapies, especially for pts treated with Janus kinase inhibitors (JAKi), which may worsen anemia, and for those with RBCT dependence. Luspatercept (LUSPA), a first-in-class erythroid maturation agent, demonstrated improvement in Hb levels and reduction in RBCT burden in the phase 2 ACE-536-MF-001 trial in pts with MF (NCT03194542), as a single agent or with ruxolitinib.

### Aims

To report the primary analysis of the double-blind, placebo (PBO)-controlled, phase 3 INDEPENDENCE trial (NCT04717414) evaluating efficacy and safety of LUSPA in pts with MF anemia.

### Methods

Adults with intermediate- to high-risk MF and anemia, receiving JAKi for ≥32 wks (stable dose ≥16 wks) and requiring 4–12 RBC U/12 wks, were randomized (2:1) to SC LUSPA Q3W (starting dose 1.33 mg/kg) or PBO. The primary endpoint (PE) was proportion of pts achieving RBCT independence (RBC-TI) for ≥12 consecutive wks starting within the first 24 wks (RBC-TI 12). After Wk 24 (Day 169), all pts underwent blinded disease response assessment to allow those with clinical benefit to continue on therapy. A post-hoc analysis of the PE applying Day 169 blinded investigator response criteria was conducted. Secondary endpoints included the proportion achieving RBC-TI for ≥16 consecutive wks starting within the first 24 wks (RBC-TI 16), reduction in RBCT burden ≥50% and ≥4 U/12 wks from baseline (BL) over any consecutive 12-wk period, RBC-TI 12 with mean Hb increase ≥1 g/dL from BL, and safety.

**Copyright Information:** (Online) ISSN: 2572-9241
©2026 The Author(s). HemaSphere is published by John Wiley & Sons Ltd on behalf of European Hematology Association. This is an open access Abstract Book distributed under the Attribution-NonCommercial-NoDerivs (CC BY-NC-ND), which allows third parties to download the articles and share them with others as long as they credit the author and the Abstract Book, but they cannot change the content in any way or use them commercially.
**Abstract Book Citations:** Authors, Title, HemaSphere, 2026;10:(S1):pages. The abstract book DOIs can be found on the "Author Information" page.
**Disclaimer:** Articles published in the journal HemaSphere exclusively reflect the opinions of the authors. The authors are responsible for all content in their abstracts including accuracy of the facts, statements, citing resources, etc.

### Results

As of May 26, 2025, 313 pts from North America (5.4%), Europe/Middle East/Latin America (61.0%), and Asia-Pacific (33.5%) were randomized to LUSPA (n=208) or PBO (n=105). BL characteristics were balanced between arms; however, regional differences were observed. Most pts were male (60.1%); 70.6% of pts had high RBCT burden (≥6 U/12 wks). Median (IQR) age was 73 (66–77) yrs, time since MF diagnosis was 4.0 (1.7–6.5) yrs, BL transfusion burden (TB) was 7 (5–10) U/12 wks, time since first RBCT was 17.6 (7.8–36.3) mos, and Hb level was 7.6 (6.9–8.4) g/dL. All pts were on prior JAKi therapy for a median (IQR) of 22.2 (10.6–44.3) mos. Mean (SD) treatment duration was 47.3 (45.9) wks for LUSPA and 37.1 (34.7) wks for PBO.
Pts on LUSPA achieved higher RBC-TI 12 (23.1%) compared with PBO (13.3%; *P*=0.0674; **Table**). Regional differences in PE response were observed (**Table**). Secondary endpoints, such as TB reduction and Hb increase, showed a consistent benefit in favor of LUSPA vs PBO (**Table**).
Treatment-emergent adverse events (TEAEs) occurred in 96.1% of LUSPA pts (exposure-adjusted incidence rate/100 pt-yrs [EAIR/100 PY], 747.6) and 92.4% of PBO pts (EAIR/100 PY, 574.8). Serious TEAEs occurred in 44.0% of LUSPA pts and 38.1% of PBO pts. After adjusting for exposure, serious TEAE incidence rates were not significantly different for LUSPA vs PBO (EAIR/100 PY, 64.3 vs 62.4).

### Summary/Conclusion

In pts with advanced MF-associated anemia, LUSPA vs PBO demonstrated clinically meaningful improvements in RBC-TI, TB, and Hb levels. The LUSPA safety profile was manageable and similar to that expected in advanced MF. Consistent with phase 2 results, these findings suggest LUSPA may offer a potential treatment option for pts with MF-associated anemia who have limited therapeutic options.

**Table. Primary and secondary endpoints (ITT population, N=313)**

| | LUSPA n=208 | PBO n=105 |
|---|---|---|
| **Primary endpoint** | | |
| **RBC-TI 12 main estimand: RBC-TI ≥12 consecutive wks, n (%)**[a] | 48 (23.1) | 14 (13.3) |
| CMH adjusted difference, % (95% CI)[b] | 8.27 (−0.04, 16.57) | |
| *P* value[b] | 0.0674 | |
| **Primary endpoint subgroup analysis by region** | | |
| North America, n/N (%) | 4/14 (28.6) | 0/3 (0.0) |
| Risk difference (95% CI) | 28.57 (−39.27, 59.26) | |
| Europe, Middle East, Africa, Latin America, n/N (%) | 28/127 (22.0) | 3/64 (4.7) |
| Risk difference (95% CI) | 17.36 (5.52, 26.49) | |
| Asia-Pacific, n/N (%) | 16/67 (23.9) | 11/38 (28.9) |
| Risk difference (95% CI) | −5.07 (−24.02, 12.14) | |
| **Secondary endpoints** | | |
| **RBC-TI 16: RBC-TI ≥16 consecutive wks, n (%)** | 40 (19.2) | 12 (11.4) |
| CMH adjusted difference, % (95% CI)[b] | 6.51 (−1.26, 14.28) | |
| **Reduction in RBCT burden from BL of ≥50% and ≥4 U/12 wks over any consecutive 12-wk period, n (%)** | 85 (40.9) | 24 (22.9) |
| CMH adjusted difference, % (95% CI)[b] | 17.3 (6.9, 27.7) | |
| **RBC-TI 12 and a mean Hb increase ≥1 g/dL from BL, n (%)** | 33 (15.9) | 6 (5.7) |
| CMH adjusted difference, % (95% CI)[b] | 9.3 (2.7, 15.8) | |

BL, baseline; CI, confidence interval; CMH, Cochran-Mantel-Haenszel; CRF, case report form; Hb, hemoglobin; ITT, intent-to-treat; LUSPA, luspatercept; PBO, placebo; pt, patient; RBC-TI, red blood cell-transfusion independence; wks, weeks.

[a]Response rate was calculated using number of responders divided by number of pts used in efficacy analysis.
[b]Calculated using CMH method of weighting using stratification factors per CRF and Sato for variance estimate.

**Copyright Information:** (Online) ISSN: 2572-9241
©2026 The Author(s). HemaSphere is published by John Wiley & Sons Ltd on behalf of European Hematology Association. This is an open access Abstract Book distributed under the Attribution-NonCommercial-NoDerivs (CC BY-NC-ND), which allows third parties to download the articles and share them with others as long as they credit the author and the Abstract Book, but they cannot change the content in any way or use them commercially.
**Abstract Book Citations:** Authors, Title, HemaSphere, 2026;10:(S1):pages. The abstract book DOIs can be found on the "Author Information" page.
**Disclaimer:** Articles published in the journal HemaSphere exclusively reflect the opinions of the authors. The authors are responsible for all content in their abstracts including accuracy of the facts, statements, citing resources, etc.

### (S216) MUTANT CALRETICULIN–SPECIFIC MONOCLONAL ANTIBODY, INCA033989, IS WELL TOLERATED AND ACHIEVES ROBUST SPLEEN, ANEMIA, AND MOLECULAR RESPONSES IN PATIENTS (PTS) WITH MYELOFIBROSIS (MF)

**Topic:** 16. Myeloproliferative neoplasms - Clinical

Claire Harrison*[1], Vikas Gupta[2], Haifa Kathrin Al-Ali[3], Haris Ali[4], John Mascarenhas[5], Francesco Passamonti[6], Aaron T. Gerds[7], Sanjay Mohan[8], Francesca Palandri[9], Bethan Psaila[10], Raajit K Rampal[11], William Shomali[12], David Ross[13], Vincent Ribrag[14], Terrence Bradley[15], Chi-Joan How[16], Steffen Koschmieder[17], Caroline McNamara[18], Andrew Perkins[19], Abdulraheem Yacoub[20], Jason Gotlib[12], Jyoti Nangalia[21], Chenwei Tian[22], Betty Lamothe[22], Erin Crowgey[22], Tatiana Zinger[22], Evan Braunstein[22], Tania Jain[23]

*[1]Guy's and St Thomas' NHS Foundation Trust, London, United Kingdom;[2]Princess Margaret Cancer Centre, Toronto, ON, Canada; [3]University Medicine Halle, Saale, Germany; [4]City of Hope Medical Center, Duarte, CA, United States of America; [5]Icahn School of Medicine at Mount Sinai, New York, NY, United States of America;[6]Fondazione IRCCS Ca Ganda Ospedale Maggiore, Milan, Italy; [7]Cleveland Clinic, Cleveland, OH, United States of America; [8]Vanderbilt University Medical Center, Nashville, TN, United States of America; [9]IRCCS Azienda Ospedaliero-Universitaria di Bologna, Bologna, Italy; [10]University of Oxford, Oxford, United Kingdom; [11]Memorial Sloan Kettering Cancer Center, New York, NY, United States of America;[12]Stanford Cancer Institute/Stanford University School of Medicine, Stanford, CA, United States of America; [13]Royal Adelaide Hospital, Adelaide, SA, Australia; [14]Institut Gustave Roussy, Villejuif, France; [15]University of Miami Sylvester Comprehensive Cancer Center, Division of Hematology, Miami, FL, United States of America; [16]Brigham and Women's Hospital and Dana-Farber Cancer Institute, Boston, MA, United States of America; [17]RWTH Aachen University, Faculty of Medicine, and Center for Integrated Oncology (CIO-ABCD), Aachen, Germany; [18]Royal Brisbane and Women's Hospital, Brisbane, QLD, Australia; [19]The Alfred Hospital, Melbourne, VIC, Australia; [20]The University of Kansas Cancer Center, Kansas City, KS, United States of America;[21]The Sanger Institute, Cambridge, United Kingdom; [22]Incyte Corporation, Wilmington, DE, United States of America;[23]Sidney Kimmel Comprehensive Cancer Center, Johns Hopkins University, Baltimore, MD, United States of America;*

### Background

MF is a myeloproliferative neoplasm characterized by bone marrow fibrosis, anemia, splenomegaly, and debilitating symptoms. Among pts with MF, ~25-35% harbor mutations in exon 9 of calreticulin (mut*CALR*). INCA033989 is a novel, fully human, Fc-silenced, IgG1 monoclonal antibody that selectively targets mut*CALR* in complex with thrombopoietin receptor, inhibiting oncogenic signaling and cell proliferation. INCA033989-101 (NCT05936359) and -102 (NCT06034002) are phase 1, first-in-human studies evaluating INCA033989 in pts with MF or ET.

### Aims

To evaluate safety and efficacy of INCA033989 monotherapy (MF mono) or combined with ruxolitinib (rux; MF combo).

### Methods

Enrolled pts had primary or post-ET MF with mut*CALR*, spleen volume (SV) of ≥450 mL or palpable ≥5 cm (SV <450 mL). MF mono pts were intolerant to JAK inhibitor (JAKi) treatment (tx), relapsed/resistant after ≥12 wks' tx, or ineligible for JAKi tx. MF combo pts had ongoing rux tx for ≥12 wks with a suboptimal response. Endpoints were safety and tolerability (primary); SV reduction (≥25% [SVR25]; ≥35% [SVR35]); anemia response (Tefferi. *Blood.* 2024); MPN-SAF total symptom score (TSS); reduction in mut*CALR* variant allele frequency (VAF).

**Copyright Information:** (Online) ISSN: 2572-9241
©2026 The Author(s). HemaSphere is published by John Wiley & Sons Ltd on behalf of European Hematology Association. This is an open access Abstract Book distributed under the Attribution-NonCommercial-NoDerivs (CC BY-NC-ND), which allows third parties to download the articles and share them with others as long as they credit the author and the Abstract Book, but they cannot change the content in any way or use them commercially.
**Abstract Book Citations:** Authors, Title, HemaSphere, 2026;10:(S1):pages. The abstract book DOIs can be found on the "Author Information" page.
**Disclaimer:** Articles published in the journal HemaSphere exclusively reflect the opinions of the authors. The authors are responsible for all content in their abstracts including accuracy of the facts, statements, citing resources, etc.

**Results**

As of Jan 5, 2026, doses in MF mono were 24-3500 mg; no DLTs were observed and an MTD was not reached. In total, 70 pts were enrolled; 41 (59%), 15 (21%), and 14 (20%) harbored Type 1, Type 2, or other mut*CALR*, respectively. Median (range) baseline VAF was 36% (5, 53). INCA033989 was well-tolerated with 59 (84%) pts continuing treatment; median (range) exposure was 40 wks (2, 119). Tx-emergent adverse events (TEAEs) occurred in 66 pts (94%); 21 pts (30%) had grade ≥3 TEAEs, most frequently anemia (9%) and neutropenia (7%). 7 pts (10%) had serious AEs leading to dose delay (n=1, 2500 mg); infusion interruption and dose delay (n=1, 70 mg); drug withdrawal (n=2, 250/400 mg); no dose change (n=3,100/1500 mg). 2 pts had a TEAE (AST increased; thrombocytopenia) leading to dose reduction; infusion reactions led to infusion interruption in 4 pts (6%).
Across all doses at week 24, 20/45 pts (44%) achieved SVR25; 14/45 pts (31%) achieved SVR35; and 14/38 pts (37%) achieved TSS50 (**Figure**). Of 36 pts with anemia and ≥12 wks tx, 21 (58%) had an anemia response (major, n=19 [53%]; minor, n=2 [6%]).

VAF reduction from baseline occurred in 51/56 pts (91%); exploratory analysis showed reduction in mut*CALR* $CD34^+$ cells in most pts.
In MF combo (n=20; median [range] rux dose, 40 mg daily [10, 50]), 12 (60%), 7 (35%), and 1 pt (5%) harbored Type 1, Type 2, and other mut*CALR*, respectively. Median (range) baseline VAF was 38% (30, 85). All pts experienced TEAEs; 13 (65%) had grade ≥3 TEAEs, most commonly anemia (35%); 5 (25%) had SAEs leading to dose delay (n=2; 750, 1500 mg) or drug withdrawal (n=1; 70 mg). Across all doses at week 24, 10/16 pts (63%) achieved SVR25; 6/16 pts (38%) achieved SVR35; and 4/13 pts (31%) achieved TSS50. Of 16 pts with anemia and ≥12 wks tx, 5 (31%) had an anemia response (major, n=2 [13%]; minor, n=3 [19%]). VAF reduction occurred in 18 pts (95%).

**Summary/Conclusion**

INCA033989 administered as monotherapy or with rux continues to show a favorable safety profile in pts with MF who are relapsed/resistant/intolerant to, or ineligible for, JAKi tx, or had a suboptimal response to rux. Robust spleen, symptom and anemia responses are observed and correlate with reductions in mut*CALR* cell populations. These data support the planned initiation of phase 3 trials in pts with MF.

**Copyright Information:** (Online) ISSN: 2572-9241
©2026 The Author(s). HemaSphere is published by John Wiley & Sons Ltd on behalf of European Hematology Association. This is an open access Abstract Book distributed under the Attribution-NonCommercial-NoDerivs (CC BY-NC-ND), which allows third parties to download the articles and share them with others as long as they credit the author and the Abstract Book, but they cannot change the content in any way or use them commercially.
**Abstract Book Citations:** Authors, Title, HemaSphere, 2026;10:(S1):pages. The abstract book DOIs can be found on the "Author Information" page.
**Disclaimer:** Articles published in the journal HemaSphere exclusively reflect the opinions of the authors. The authors are responsible for all content in their abstracts including accuracy of the facts, statements, citing resources, etc.

**Figure. In Patients with MF, Treatment with INCA033989 Improved Spleen Volume, Symptom Score, and Anemia Response**

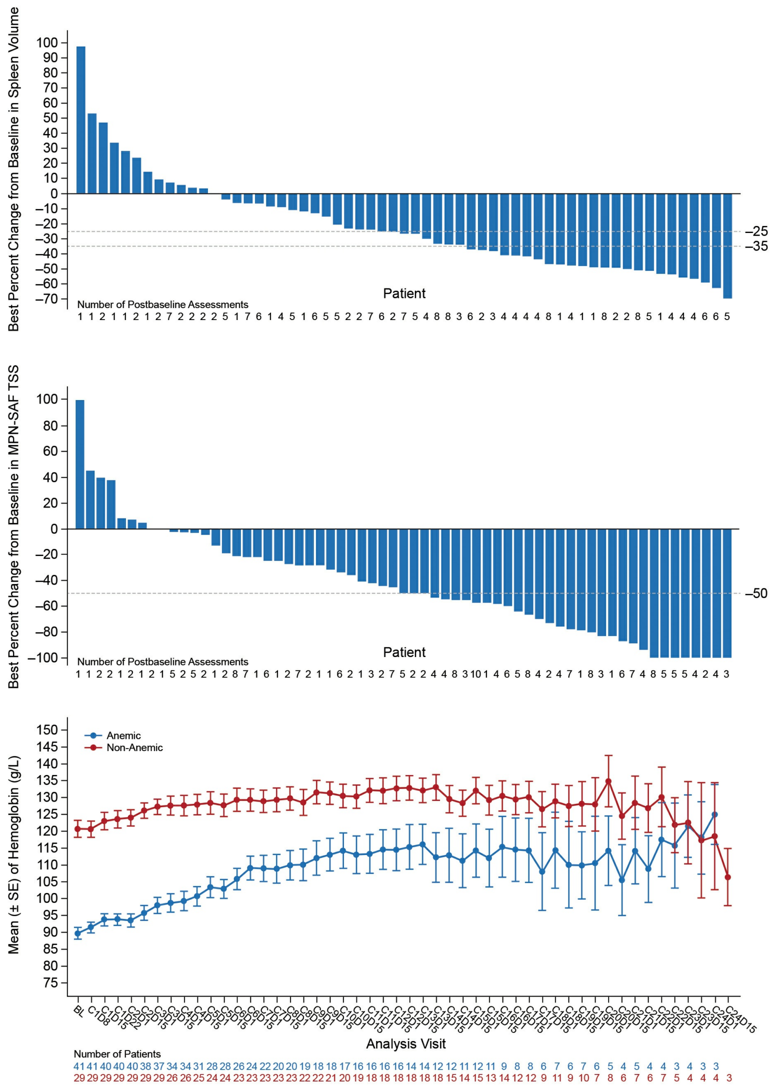

MPN-SAF, Myeloproliferative Neoplasms Symptom Assessment Form; TSS, total symptom score.

**Copyright Information:** (Online) ISSN: 2572-9241
©2026 The Author(s). HemaSphere is published by John Wiley & Sons Ltd on behalf of European Hematology Association. This is an open access Abstract Book distributed under the Attribution-NonCommercial-NoDerivs (CC BY-NC-ND), which allows third parties to download the articles and share them with others as long as they credit the author and the Abstract Book, but they cannot change the content in any way or use them commercially.
**Abstract Book Citations:** Authors, Title, HemaSphere, 2026;10:(S1):pages. The abstract book DOIs can be found on the “Author Information” page.
**Disclaimer:** Articles published in the journal HemaSphere exclusively reflect the opinions of the authors. The authors are responsible for all content in their abstracts including accuracy of the facts, statements, citing resources, etc.

### (S217) EFFICACY AND SAFETY OF BEZUCLASTINIB IN PATIENTS WITH ADVANCED SYSTEMIC MASTOCYTOSIS: PRIMARY RESULTS FROM THE APEX STUDY

**Topic:** 16. Myeloproliferative neoplasms - Clinical

Daniel J. Deangelo*[1], Deepti Radia[2], Tracy I. George[3], Isaac Goncalves[4], Khalid Shoumariyeh[5], Cristina Papayannidis[6], Pankit Vachhani[7], Tsewang Tashi[8], Anthony M. Hunter[9], Vinod Pullarkat[10], Juliana Schwaab[11], Jonathan Lambert[12], Miguel Piris-Villaespesa[13], Prithviraj Bose[14], Cecilia Arana Yi[15], Tania Jain[16], Gabriela Hobbs[17], Andreas Reiter[18], Karin Hartmann[19], Stephanie Lee[20], Marek Hus[21], Sudipto Mukherjee[22], Wolfgang Sperr[23], Stephen Fuller[24], Saskia K. Klein[25], Cristina Bulai Livideanu[26], Jens Panse[27], Olivier Hermine[28], Manish Jain[29], Minakshi Taparia[30], Ewa Wierzbicka-Hainaut[31], Kelli Williams[32], Helena Pomares[33], Gary Schiller[34], Amanda Pilla[34], Kimberly Dishman[34], Jenna Zhang[34], Lei Sun[34], Deepak Nagendra[34], Rachael Easton[34], Jessica Sachs[34], Jason Gotlib[35]

*[1]Dana-Farber Cancer Institute, Boston, United States of America; [2]Guy's and St. Thomas' NHS Foundation Trust, London, United Kingdom; [3]University of Utah, ARUP Laboratories, Salt Lake City, United States of America;[4]Royal Melbourne Hospital and Peter MacCallum Cancer Centre, Parkville, Australia; [5]University of Freiburg, Department of Hematology, Freiburg, Germany; [6]IRCCS Azienda Ospedaliero-Universitaria di Bologna, Bolgna, Italy; [7]University of Alabama at Birmingham, Birmingham, United States of America; [8]Huntsman Cancer Institute, University of Utah, Salt Lake City, United States of America;[9]Emory University School of Medicine, Atlanta, United States of America; [10]City of Hope, Duarte Cancer Center, Duarte, United States of America; [11]University Hospital Mannheim, Department of Hematology and Oncology, Mannheim, United States of America; [12]University College Hospital, University College of London Hospitals NHS Foundation Trust, Department of Haematology, London, United Kingdom; [13]Hospital Universitario Ramón y Cajal, Madrid, Spain; [14]Texas MD Anderson Cancer Center, Houston, United States of America; [15]Mayo Clinic, Division of Hematology/Oncology, Department of Internal Medicine, Phoenix, United States of America; [16]Johns Hopkins University, Sidney Kimmel Comprehensive Cancer Center, Baltimore, United States of America; [17]Massachusetts General Hospital, Division of Hematology/Oncology, Boston, United States of America;[18]University Hospital Mannheim, Heidelberg University, Department of Hematology and Oncology, Mannheim, Germany; [19]University of Basel, Basel, Switzerland; [20]St. Michael's Hospital, Toronto, Canada;[21]University of Lublin, Department of Hemato-Oncology and Bone Marrow Transplantation, Medical, Lublin, Poland; [22]Cleveland Clinic Taussig Cancer Institute, Department of Hematology and Medical Oncology, Cleveland, United States of America; [23]Medical University of Vienna, Department of Internal Medicine I, Division of Hematology and Hemostaseology, and Ludwig Boltzmann Institute for Hematology and Oncology, Vienna, Austria; [24]Sydney Medical School, Nepean Clinical School, University of Sydney, Kingswood, Australia; [25]University Medical Center Groningen, Department of Hematology, Groningen, The Netherlands; [26]Toulouse University Hospital, Dermatology Department, Toulouse, France; [27]RWTH Aachen University, Department of Hematology, Oncology, Hemostaseology and Stem Cell Transplantation, Aachen, Germany; [28]Necker Children's Hospital, Paris Centre University, Paris, France;[29]The Leeds Teaching Hospital, Leeds, United Kingdom; [30]University of Alberta, Edmonton, AB, Canada;[31]Université de Poitiers, Poitiers, France; [32]National Institutes of Health, Laboratory of Clinical Infectious Diseases, National Institute of Allergy and Infectious Diseases, Bethesda, United States of America; [33]Institut Catala d'Oncologia Hospitalet – Hospital Duran I Reynals, Barcelona, Spain;[34]Cogent Biosciences, Inc., Waltham, United States of America; [35]Stanford Cancer Institute, Stanford University School of Medicine, Division of Hematology, Department of Medicine, Stanford, United States of America;*

### Background

Advanced systemic mastocytosis (AdvSM) is a rare clonal myeloid neoplasm associated with significant disease burden and comprises aggressive systemic mastocytosis (ASM), systemic mastocytosis with associated hematologic neoplasm (SM-AHN), and mast cell leukemia (MCL). Bezuclastinib is an oral, selective type I tyrosine kinase inhibitor (TKI) with potent activity against *KIT* p.D816V, the driving mutation in ~95% of SM cases. Part 2 of the Apex study evaluated the efficacy and safety of bezuclastinib in patients with AdvSM.

**Copyright Information:** (Online) ISSN: 2572-9241
©2026 The Author(s). HemaSphere is published by John Wiley & Sons Ltd on behalf of European Hematology Association. This is an open access Abstract Book distributed under the Attribution-NonCommercial-NoDerivs (CC BY-NC-ND), which allows third parties to download the articles and share them with others as long as they credit the author and the Abstract Book, but they cannot change the content in any way or use them commercially.
**Abstract Book Citations:** Authors, Title, HemaSphere, 2026;10:(S1):pages. The abstract book DOIs can be found on the "Author Information" page.
**Disclaimer:** Articles published in the journal HemaSphere exclusively reflect the opinions of the authors. The authors are responsible for all content in their abstracts including accuracy of the facts, statements, citing resources, etc.

**Methods**

Apex is an ongoing, phase 2, open-label, registrational study evaluating bezuclastinib in patients with AdvSM. Based on tolerability, clinical activity, and pharmacokinetic data from the dose confirmation portion of the study, 150 mg daily dose of bezuclastinib was selected as the Part 2 dose. The primary endpoint was overall response rate (ORR) per modified International Working Group (mIWG)-Myeloproliferative Neoplasms Research and Treatment-European Competence Network on Mastocytosis (MRT-ECNM) response criteria. Key secondary endpoints included ORR per pure pathologic response (PPR) criteria and safety.

**Results**

Bezuclastinib 150 mg was administered in 81 patients (intention-to-treat [ITT] population) with AdvSM, including 57 patients with SM-AHN, 11 with ASM, and 13 with MCL and MCL-AHN. Sixty eight patients were evaluable for response assessment per mIWG-MRT-ECNM criteria. In the ITT population, median age (range) was 70 (43-87) years; 23 (28%) patients were female; 28 patients (35%; avapritinib, 7%; midostaurin, 30%) received prior KIT-targeted TKI therapy. Median (range) baseline bone marrow (BM) mast cell (MC) burden, serum tryptase, and whole blood *KIT* p.D816V variant allele frequency (VAF) were 35% (3-95), 191 ng/mL (23.3-1987), and 6.7% (0.0-91.4), respectively.
At the time of the primary analysis, September 19, 2025, ORR was 57% in evaluable patients per mIWG-MRT-ECNM criteria (complete response [CR/CRh], 13%; partial response [PR], 35%; clinical improvement, 9%) and 80% in the ITT population per PPR criteria (CR/CRh, 57%; PR, 24%). In evaluable patients, median time to response was 2.0 months. At the time of data cut, duration of response was not yet mature; median duration of treatment was 9.4 months. In the ITT population, bezuclastinib reduced MC burden, serum tryptase, and *KIT* p.D816V VAF by ≥50% in 89%, 89%, and 91% of patients, respectively.
Overall, treatment-related adverse events (TRAEs) occurred in 93% of patients, with a low incidence of drug-related serious AEs (6%). The most frequent TRAEs (total/grade ≥3) were hair color change (31%/0%), neutropenia (30%/24%), altered taste (28%/0%), thrombocytopenia (25%/14%), and alanine/aspartate aminotransferase increased (21%/1%). Dose reductions due to TRAEs occurred in 14.8% of patients and were mainly due to hematologic events; no other AEs led to dose reduction in >1 patient. There were no discontinuations due to TRAEs. No treatment-related deaths occurred.

**Summary/Conclusion**

Bezuclastinib demonstrated high response rates, led to deep reductions in objective markers of MC burden, and was well-tolerated, with infrequent dose reductions and no discontinuations due to TRAEs. Positive results from Apex support bezuclastinib as a potential treatment for patients with AdvSM.

**Copyright Information:** (Online) ISSN: 2572-9241
©2026 The Author(s). HemaSphere is published by John Wiley & Sons Ltd on behalf of European Hematology Association. This is an open access Abstract Book distributed under the Attribution-NonCommercial-NoDerivs (CC BY-NC-ND), which allows third parties to download the articles and share them with others as long as they credit the author and the Abstract Book, but they cannot change the content in any way or use them commercially.
**Abstract Book Citations:** Authors, Title, HemaSphere, 2026;10:(S1):pages. The abstract book DOIs can be found on the “Author Information” page.
**Disclaimer:** Articles published in the journal HemaSphere exclusively reflect the opinions of the authors. The authors are responsible for all content in their abstracts including accuracy of the facts, statements, citing resources, etc.

### (S218) RESULTS OF AJX-101, A PHASE 1 CLINICAL TRIAL OF THE TYPE II JAK2 INHIBITOR AJ1-11095, IN PATIENTS WITH MYELOFIBROSIS WHO HAVE BEEN FAILED BY A TYPE I JAK2 INHIBITOR

**Topic:** 16. Myeloproliferative neoplasms - Clinical

John Mascarenhas*[1], Uma Borate[2], Prithviraj Bose[3], John Byrd[4], Jacqueline Garcia[5], Jason Gotlib[6], Michael Grunwald[7], Gabriela Hobbs[8], Andrew Kuykendall[9], Ruben Mesa[7], Stephen Oh[10], Kristen Pettit[11], Raajit K Rampal[12], William Shomali[6], Andrew Srisuwananukorn[2], Eric Vick[13], Abdulraheem Yacoub[14], David Steensma[15]

*[1]Icahn School of medicine, New York, United States of America;[2]Ohio State University, James Cancer Center, Columbus, Ohio, United States of America; [3]MD Anderson Cancer Center, Leukemia, Houston, Texas, United States of America;[4]University of Pittsburgh, Pittsburgh, PA, United States of America; [5]Dana-Farber Cancer Institute, Adult Leukemia Program, Boston, United States of America; [6]Stanford University, Stanford, California, United States of America;[7]Atrium-Levine/Wake Forest Cancer Center, Advocate Health, Charlotte, North Carolina, United States of America; [8]Massachusetts General Hospital, Boston, Massachusetts, United States of America; [9]H. Lee Moffitt Cancer Center, Tampa, Florida, United States of America; [10]Washington University - St Louis, St Louis, Missouri, United States of America; [11]University of Michigan, Ann Arbor, Michigan, United States of America; [12]Memorial Sloan Kettering Cancer Center, New York, New York, United States of America;[13]University of Cincinnati, Cincinnati, Ohio, United States of America; [14]University of Kansas Medical Center, Kansas City, Kansas, United States of America; [15]Ajax Therapeutics, New York, United States of America;*

### Background

Aberrant JAK-STAT signaling in clonal hematopoietic stem and progenitor cells is a fundamental biologic driver of myelofibrosis (MF) and therapeutic target for approved type I JAK2 inhibitors. While these drugs provide important spleen and symptom benefit, most patients discontinue JAK2 inhibitor therapy within ~3 years of initiation due to lack of response, relapse, disease progression, or adverse events (Palandri F et al *Cancer* 2020). AJ1-11095 is a novel orally bioavailable small molecule type II JAK2 inhibitor that inhibits both JAK2 homodimerization and heterodimerization of JAK2 with JAK1 and TYK2, overcoming a common mechanism of clonal persistence and drug resistance to type I JAK2 inhibitors *in vitro* and in pre-clinical models of MPN.

### Aims

We report initial results of a first-in-human clinical trial (NCT06343805) of AJ1-11095 in patients with MF previously treated with a type I JAK2 inhibitor, but who had recurrent or persistent splenomegaly and symptoms.

### Methods

Key eligibility criteria for AJX-101 include primary MF or post-PV/post-ET MF with ≤10% marrow blasts; DIPSS intermediate-2 or high-risk disease; spleen volume ≥450cm$^3$; Myelofibrosis Symptom Assessment Form (MFSAF) version 4.0 Total Symptom Score (TSS) ≥10 or ≥2/7 symptoms scoring ≥3; relapsed/refractory after prior therapy with ≥1 type I JAK2 inhibitor(s); baseline platelet count ≥75 x 10$^9$/L; and adequate organ function. The primary study objective is to evaluate safety and establish the maximum tolerated dose (MTD) and/or recommended Phase 2 dose (RP2D) for AJ1-11095 in patients with MF. Secondary endpoints include ≥50% reduction in TSS score (TSS50), spleen volume reduction (SVR) ≥35% (SVR35), and characterization of the pharmacokinetics (PK) of AJ1-11095. Intra-patient dose escalation is permitted after 24 weeks.

**Copyright Information:** (Online) ISSN: 2572-9241
©2026 The Author(s). HemaSphere is published by John Wiley & Sons Ltd on behalf of European Hematology Association. This is an open access Abstract Book distributed under the Attribution-NonCommercial-NoDerivs (CC BY-NC-ND), which allows third parties to download the articles and share them with others as long as they credit the author and the Abstract Book, but they cannot change the content in any way or use them commercially.
**Abstract Book Citations:** Authors, Title, HemaSphere, 2026;10:(S1):pages. The abstract book DOIs can be found on the "Author Information" page.
**Disclaimer:** Articles published in the journal HemaSphere exclusively reflect the opinions of the authors. The authors are responsible for all content in their abstracts including accuracy of the facts, statements, citing resources, etc.

### Results

In the dose escalation phase, 23 patients (ages 47-87y,16M/7F; 16 JAK2 V617F, 6 CALR, 2 MPL W515K/L mutations; median 2 prior therapies (range 1-7), median 6.1y from diagnosis; all with prior ruxolitinib exposure, 8 with momelotinib) enrolled across 6 dose levels: 25 (n=3), 50 (n=3), 75 (n=6), 100 (n=6), and 125 mg (n=5) orally once daily.

As of the data cutoff, the longest follow-up was 17 months, 19/23 patients continued on therapy, no patients discontinued due to adverse events (AEs). No DLTs were observed. Most treatment-related AEs (TRAEs) related to study drug were grade 1, except for anemia and thrombocytopenia (dose dependent and related to baseline cytopenias; 19/23 patients were anemic at baseline and 10/23 were thrombocytopenic).

Of 20 patients who had reached week 12 of therapy and first on-therapy imaging, 13 (65%) had SVR35; 4 more had 25%-34% SVR (85% SVR25+). With dose escalation, 2 additional patients attained SVR35. TSS50 was achieved in cycle 1 by 17/23 (74%) and in cycle 2 by 2 subjects, with all 7 MFSAF domains improving.

PK profiling showed dose-proportionate increases in time-concentration area under the curve (AUC), consistent with pre-clinical models, and plasma concentrations exceeding SET2 IC50 for AJ1-11095 at all dose levels. All patients except 1 had reductions in driver mutation VAF within 28 days; at week 24, 4/7 treated with doses above 25 mg had VAF reductions ≥50%.

### Summary/Conclusion

The first-in-class type II JAK2 inhibitor AJ1-11095 induces symptomatic improvement, spleen volume reduction, and decrease in mutant clone size in most patients with MF previously treated with type I JAK2 inhibitors. The 75 mg QD dose was selected for initial dose expansion.

**Copyright Information:** (Online) ISSN: 2572-9241
©2026 The Author(s). HemaSphere is published by John Wiley & Sons Ltd on behalf of European Hematology Association. This is an open access Abstract Book distributed under the Attribution-NonCommercial-NoDerivs (CC BY-NC-ND), which allows third parties to download the articles and share them with others as long as they credit the author and the Abstract Book, but they cannot change the content in any way or use them commercially.
**Abstract Book Citations:** Authors, Title, HemaSphere, 2026;10:(S1):pages. The abstract book DOIs can be found on the "Author Information" page.
**Disclaimer:** Articles published in the journal HemaSphere exclusively reflect the opinions of the authors. The authors are responsible for all content in their abstracts including accuracy of the facts, statements, citing resources, etc.

### (S219) EARLY VERSUS DELAYED INITIATION OF ROPEGINTERFERON ALFA-2B IN HIGH-RISK ESSENTIAL THROMBOCYTHAEMIA: TWO-YEAR RESULTS FROM THE PHASE 3 SURPASS-ET STUDY

**Topic:** 16. Myeloproliferative neoplasms - Clinical

Harry Gill*[1], Zhijian Xiao[2], Jie Jin[3], Keita Kirito[4], Norio Komatsu[5], Albert Qin[6], Toshiaki Sato[7], Oleh Zagrijtschuk[8], Lucia Masarova[9], Ruben Mesa[10]

*[1]LKS Faculty of Medicine, the University of Hong Kong, Hong Kong, Hong Kong;[2]National Clinical Research Centre for Blood Diseases, Institute of Haematology and Blood Diseases Hospital, Chinese Academy of Medical Sciences & Peking Union Medical College, State Key Laboratory of Experimental Haematology, Tianjin, China; [3]The First Affiliated Hospital, Zhejiang University School of Medicine, Hangzhou, China; [4]University of Yamanashi, Department of Haematology and Oncology, Yamanashi, Japan; [5]PharmaEssentia Japan KK, Tokyo, Japan; [6]PharmaEssentia Corporation, Taipei, Taiwan; [7]PharmaEssentia Japan K.K, Tokyo, Japan; [8]PharmaEssentia Corporation, Burlington, United States of America;[9]MD Anderson Cancer Center, Houston, United States of America; [10]Atrium Health Wake Forest Baptist Comprehensive Cancer Center, Winston-Salem, NC, United States of America;*

### Background

In the phase 3 SURPASS-ET trial, ropeginterferon alfa-2b (P1101) demonstrated superior durable modified ELN responses at Months 9 and 12 compared with anagrelide in patients with high-risk essential thrombocythaemia (ET) and leukocytosis resistant or intolerant to hydroxyurea. After completion of Year 1, eligible patients receiving anagrelide were allowed to switch to P1101 based on predefined criteria indicating expected benefit.

### Aims

We report 2-year outcomes comparing continuous P1101 treatment (early interferon start) versus delayed initiation after 1 year of anagrelide.

### Methods

SURPASS-ET (NCT04285086) is a randomized, open-label, phase 3 study conducted at 55 sites across Asia and North America. Adults with HU intolerance or resistance, high-risk ET and leukocytosis were randomized to P1101 (250 µg Week 0, 350 µg Week 2, 500 µg from Week 4 onward, every 2 weeks) or anagrelide. Following the 12-month primary analysis period, patients in the anagrelide arm who met eligibility criteria transitioned to P1101. For the 2-year analysis, patients were evaluated in two groups: **Early P1101**: continuous treatment from baseline to Month 24, and **Delayed P1101**: initial anagrelide (Year 1) followed by switch to P1101 (Year 2). Endpoints included sustained hematologic response, molecular dynamics, safety, and progression-free survival (PFS).

**Copyright Information:** (Online) ISSN: 2572-9241
©2026 The Author(s). HemaSphere is published by John Wiley & Sons Ltd on behalf of European Hematology Association. This is an open access Abstract Book distributed under the Attribution-NonCommercial-NoDerivs (CC BY-NC-ND), which allows third parties to download the articles and share them with others as long as they credit the author and the Abstract Book, but they cannot change the content in any way or use them commercially.
**Abstract Book Citations:** Authors, Title, HemaSphere, 2026;10:(S1):pages. The abstract book DOIs can be found on the “Author Information” page.
**Disclaimer:** Articles published in the journal HemaSphere exclusively reflect the opinions of the authors. The authors are responsible for all content in their abstracts including accuracy of the facts, statements, citing resources, etc.

### Results

With approximately 24 months of follow-up, 77/91 (84.6%) patients in the Early P1101 group and 34/83 (41.0%) in the Delayed P1101 group were on P1101 at data cutoff. At Months 15, 18, 21 and 24, modified ELN responses were observed in 49.0% (24/49), 56.6% (30/53), 62.1% (36/58) and 58.6% (34/58) of Early P1101 patients versus 32.0% (8/25), 59.1% (13/22), 41.7% (10/24) and 62.5% (15/24) in the Delayed P1101 cohort, respectively. Patients switching from anagrelide demonstrated improvement in response rates after transition. Molecular responses deepened with longer interferon exposure. Partial Molecular Response at Month 24 for subjects with positive result at baseline was observed in 17/49 (34.7%) of early P1101 start patients comparing to 4/26 (15.4%) of patients with delayed P1101 treatment start. Molecular parameters remained largely stable during anagrelide treatment and began to decline only after transition to P1101. Kaplan–Meier PFS analysis (with progression events counted from deaths, progressive disease to sMF and thromboembolic events and bleedings) showed favorable outcomes in both cohorts. At 24 months, estimated PFS was 76.9% (95% CI 66.4%–84.4%) in the Early P1101 group versus 43.1% (95% CI 30.6%–55.0%) in the Delayed P1101 group. Safety during Year 2 remained consistent with the known interferon profile. Importantly, the incidence of new Grade ≥3 treatment-emergent adverse events decreased during the second year of P1101 therapy (8.8% vs 23.1% in Year 1). Patients switching from anagrelide experienced no excess toxicity following transition.

### Summary/Conclusion

Two-year results from SURPASS-ET demonstrate sustained hematologic control, progressive molecular improvement, and favorable safety with P1101. Early initiation of interferon therapy was associated with deeper molecular responses and numerically improved long-term disease control compared with delayed initiation after one year of anagrelide. These findings support consideration of earlier interferon use in high-risk ET following hydroxyurea intolerance or resistance.

**Copyright Information:** (Online) ISSN: 2572-9241
©2026 The Author(s). HemaSphere is published by John Wiley & Sons Ltd on behalf of European Hematology Association. This is an open access Abstract Book distributed under the Attribution-NonCommercial-NoDerivs (CC BY-NC-ND), which allows third parties to download the articles and share them with others as long as they credit the author and the Abstract Book, but they cannot change the content in any way or use them commercially.
**Abstract Book Citations:** Authors, Title, HemaSphere, 2026;10:(S1):pages. The abstract book DOIs can be found on the “Author Information” page.
**Disclaimer:** Articles published in the journal HemaSphere exclusively reflect the opinions of the authors. The authors are responsible for all content in their abstracts including accuracy of the facts, statements, citing resources, etc.

### (S220) COFORMULATED FAVEZELIMAB/PEMBROLIZUMAB VERSUS CHEMOTHERAPY IN PARTICIPANTS WITH RELAPSED OR REFRACTORY CLASSIC HODGKIN LYMPHOMA: RESULTS OF THE RANDOMIZED PHASE 2 KEYFORM-008 STUDY

**Topic:** 17. Hodgkin lymphoma - Clinical

Guilherme Perini*[1, 2], David Lavie[3], Paul J. Bröckelmann[4], Muhit Ozcan[5], Nathalie Johnson[6], Wonseog Kim[7], Carolina Mahuad[8], Norma Gutierrez Gutierrez[9], Senem Maral[10], Cecile Borel[11], Tae Min Kim[12], Iara Goncalves[13], Deepa Jagadeesh[14], Yasmin Karimi[15], Nina Wagner-Johnston[16], John Timmerman[17], Ryan Lynch[18], Graham Collins[19], Yulia Sidi[20], Pallavi Pillai[20], Rushdia Yusuf[20], Alex F. Herrera[21]

*[1]Instituto Américas, São Paulo, Brazil; [2]Einstein Hospital Israelita, São Paulo, Brazil; [3]Hadassah Medical Center, Jerusalem, Israel; [4]University Hospital of Cologne, Cologne, Germany; [5]Ankara University School of Medicine, Ankara, Türkiye; [6]Jewish General Hospital, Montreal, QC, Canada; [7]Samsung Medical Center, Sungkyunkwan University School of Medicine, Seoul, South Korea; [8]Hospital Alemán, Buenos Aires, Argentina; [9]Hospital Universitario de Salamanca, IBSAL, Centro de Investigación del Cáncer, Salamanca, Spain; [10]Medipol University Faculty of Medicine, Istanbul, Türkiye; [11]Institut Universitaire du Cancer Toulouse-Oncopole, Toulouse, France; [12]Seoul National University Hospital, Seoul, South Korea; [13]Fundação Pio XII-Hospital de Câncer de Barretos, São Paulo, Brazil; [14]Cleveland Clinic, Cleveland, United States of America; [15]University of Michigan, Ann Arbor, United States of America; [16]Johns Hopkins University School of Medicine, Baltimore, United States of America; [17]UCLA Medical Center, Los Angeles, United States of America; [18]Fred Hutchinson Cancer Center and University of Washington, Seattle, United States of America; [19]GenesisCare Cancer Centre, Oxford, United Kingdom; [20]Merck & Co., Inc., Rahway, United States of America; [21]City of Hope, Duarte, United States of America;*

### Background

PD-1 blockade with agents such as pembrolizumab is standard treatment (tx) for relapsed or refractory (R/R) classic Hodgkin lymphoma (cHL); however, most patients experience disease progression (PD), and optimal post–PD-1 therapy remains undefined. Favezelimab, a monoclonal antibody targeting LAG-3, plus pembrolizumab has shown promising efficacy with manageable safety in participants (pts) with anti–PD-1–refractory R/R cHL in a phase 1/2 study.

### Aims

To present data from KEYFORM-008 (NCT05508867), a randomized, open-label, phase 2 study that evaluated coformulated favezelimab/pembrolizumab vs chemotherapy in pts with anti–PD-(L)1–refractory R/R cHL.

### Methods

Eligible pts were ≥18 y with confirmed R/R cHL and ECOG PS of 0-2. Pts must have had PD per investigator ≤12 wk from last dose of anti–PD-(L)1–based therapy (received for ≥3 mo with ≥2 doses) and must have exhausted available tx options with known clinical benefit, including being ineligible for or having not responded to or progressed after autologous stem cell transplant and brentuximab vedotin. Pts were randomly assigned 1:1 to favezelimab 800 mg/pembrolizumab 200 mg IV Q3W or chemotherapy (gemcitabine 800-1200 mg/m$^2$ IV on days 1 and 8 of a 21-day cycle or bendamustine 90-120 mg/m$^2$ IV on days 1 and 2 of either a 21- or 28-day cycle). Pts who received chemotherapy with PD per Lugano criteria by BICR were eligible to cross over to favezelimab/pembrolizumab. Primary end point was PFS per Lugano criteria by investigator. Secondary end points included OS, ORR and DOR per Lugano criteria by investigator, and safety. Primary hypothesis for PFS was evaluated using a stratified log-rank test and at 5% (1-sided) alpha level. Data cutoff date was Sept 3, 2025.

**Copyright Information:** (Online) ISSN: 2572-9241
©2026 The Author(s). HemaSphere is published by John Wiley & Sons Ltd on behalf of European Hematology Association. This is an open access Abstract Book distributed under the Attribution-NonCommercial-NoDerivs (CC BY-NC-ND), which allows third parties to download the articles and share them with others as long as they credit the author and the Abstract Book, but they cannot change the content in any way or use them commercially.
**Abstract Book Citations:** Authors, Title, HemaSphere, 2026;10:(S1):pages. The abstract book DOIs can be found on the “Author Information” page.
**Disclaimer:** Articles published in the journal HemaSphere exclusively reflect the opinions of the authors. The authors are responsible for all content in their abstracts including accuracy of the facts, statements, citing resources, etc.

**Results**

Overall, 203 pts were randomly assigned to favezelimab/pembrolizumab (n=104) and chemotherapy (n=99); 52 pts in the chemotherapy arm crossed over, with 17 ongoing tx. Median follow-up was 17.6 mo (range, 7.9-34.3). Median PFS was 6.4 mo (90% CI, 5.7-8.4) with favezelimab/pembrolizumab vs 5.7 mo (90% CI, 5.6-6.2) with chemotherapy (HR, 0.757; 90% CI, 0.575-0.996; *P*=0.0462); 12-mo PFS rates were 32% vs 14%. Median OS was not reached in either arm (HR, 0.67); 12-mo OS rates were 92% vs 88%. ORR was 44.2% (95% CI, 34.5-54.3) with favezelimab/pembrolizumab vs 67.7% (95% CI, 57.5-76.7) with chemotherapy arm; CR, PR, SD and PD rates were 13.5%, 30.8%, 23.1%, and 26.0% vs 36.4%, 31.3%, 4.0%, and 25.3%, respectively. Median DOR was 11.0 mo (range, 0.0+ to 21.8+) with favezelimab/pembrolizumab vs 5.4 mo (range, 0.0+ to 27.6+) with chemotherapy; 43% vs 21% of pts had response lasting ≥12 mo. Tx-related AEs (TRAEs) occurred in 79 pts (76.0%) with favezelimab/pembrolizumab and 81 pts (83.5%) with chemotherapy. Grade 3-5 TRAEs occurred in 19 (18.3%) and 39 pts (40.2%), respectively. One tx-related death occurred in the chemotherapy arm only (sepsis).

**Summary/Conclusion**

The trial met its primary end point, showing significant PFS benefit with late separation of curves at 6 mo, although the small number of pts at risk thereafter warrants cautious interpretation. DOR was also longer. OS showed a favorable trend but remains immature. Although ORR was notably lower for favezelimab/pembrolizumab, disease control (CR+PR+SD) was similar between the two groups. KEYFORM-008 was discontinued based on the sponsor's business decision to end the entire favezelimab clinical development program and was not driven by safety or efficacy concerns.

**Copyright Information:** (Online) ISSN: 2572-9241
©2026 The Author(s). HemaSphere is published by John Wiley & Sons Ltd on behalf of European Hematology Association. This is an open access Abstract Book distributed under the Attribution-NonCommercial-NoDerivs (CC BY-NC-ND), which allows third parties to download the articles and share them with others as long as they credit the author and the Abstract Book, but they cannot change the content in any way or use them commercially.
**Abstract Book Citations:** Authors, Title, HemaSphere, 2026;10:(S1):pages. The abstract book DOIs can be found on the "Author Information" page.
**Disclaimer:** Articles published in the journal HemaSphere exclusively reflect the opinions of the authors. The authors are responsible for all content in their abstracts including accuracy of the facts, statements, citing resources, etc.

### (S221) FINAL ANALYSIS OF THE GHSG HD21 OLDER COHORT: EFFICACY, SURVIVAL AND LATE TOXICITY OF PET-GUIDED BRECADD IN OLDER PATIENTS WITH ADVANCED-STAGE CLASSIC HODGKIN LYMPHOMA

**Topic:** 17. Hodgkin lymphoma - Clinical

Justin Ferdinandus*[1, 2], Helen Kaul[1, 2], Alexander Fosså[3], Andreas Huettmann[4], Felix Keil[5, 6], Yon-Dschun Ko[7], Felicitas Hitz[8, 9], Michaela Schwarz[10], Corinna Trenker[11], Andrea Kerkhoff[12], Peter Staib[13], Kai Wille[14], Irmgard Dresel[15], Dennis Hahn[16], Bernd Hertenstein[17], Peter Moosmann[9, 18], Ulrich Jeremi Manuel Mey[9, 19], Stefan Balabanov[9, 20], Fernando Roncolato[21], Johannes Hellmuth[22], Mark Hertzberg[23, 24], Carsten Kobe[1, 2], Wolfram Klapper[2, 25], Christian Baues[2, 26], Hans T. Eich[2, 12], Stefanie Kreissl[27], Michael Fuchs[1, 2], Janina Jablonski[1, 2], Hishan Tharmaseelan[1, 2], Dennis A. Eichenauer[1, 2], Bastian von Tresckow[2, 4], Peter Borchmann[1, 2], Paul J. Bröckelmann[1, 2]

*[1]University Hospital Cologne, Cologne, Germany; [2]German Hodgkin Study Group, Cologne, Germany; [3]Oslo University Hospital, Oslo, Norway; [4]University Hospital Essen, Essen, Germany; [5]Arbeitsgemeinschaft medikamentöse Tumortherapie, Salzburg, Austria; [6]Hanusch-Krankenhaus, Vienna, Austria; [7]Johanniter Krankenhaus Bonn, Bonn, Germany; [8]Kantonsspital Sankt Gallen, Sankt Gallen, Switzerland; [9]Swiss Cancer Institute, Bern, Switzerland; [10]Charité - Universitätsmedizin Berlin, Berlin, Germany; [11]University Hospital Giessen and Marburg, Gießen, Germany; [12]University Hospital Muenster, Muenster, Germany; [13]Sankt-Antonius-Hospital, Eschweiler, Germany; [14]Johannes Wesling Medical Center Minden, University of Bochum,, Bochum, Germany; [15]Ortenau Klinikum, Offenburg, Germany; [16]Klinikum Stuttgart, Stuttgart, Germany; [17]Klinikum Bremen-Mitte, Bremen, Germany; [18]Kantonsspital Aarau, Aarau, Switzerland; [19]Kantonsspital Graubünden, Chur, Switzerland; [20]Universitätsspital Zürich, Zürich, Switzerland; [21]Gosford Hospital, Gosford, Australia;[22]University Hospital, Ludwig-Maximilians-University Muenchen (LMU), Munich, Germany; [23]Prince of Wales Hospital, Sydney, Australia;[24]The Australasian Leukaemia & Lymphoma Group, Melbourne, Australia; [25]University of Schleswig-Holstein, Campus Kiel, Kiel, Germany;[26]Marienhospital Herne, Ruhr University Bochum, Bochum, Germany; [27]Klinikum Linz Elisabethinen, Linz, Austria;*

### Background

Outcomes of older patients with advanced-stage classic Hodgkin lymphoma (AS-cHL) remain inferior compared to younger patients, partly due to limited tolerability of intensive chemotherapy. In the GHSG HD21 older cohort, the highly effective BrECADD regimen was prospectively investigated in a PET-guided strategy for patients aged 61–75 years.

### Aims

To report the final efficacy and safety of PET-guided BrECADD in older patients with newly diagnosed AS-cHL.

### Methods

This is a prospective, multicenter, single-arm phase 2 cohort within HD21 (NCT02661503) enrolling patients aged 61–75 years with AS-cHL. Treatment comprised 2 cycles of BrECADD followed by centrally reviewed PET (PET2): PET2-negative patients received a total of 4 cycles, PET2-positive patients 6 cycles; consolidative radiotherapy (30 Gy) to residual PET-positive lesions was recommended. Endpoints included complete response (CR) at end of chemotherapy, progression-free survival (PFS; time from enrolment to progression/relapse/death), overall survival (OS), time to progression (TTP; censoring deaths without prior progression), secondary malignancies, and late toxicities (data cut 01-Oct-2025).

**Copyright Information:** (Online) ISSN: 2572-9241
©2026 The Author(s). HemaSphere is published by John Wiley & Sons Ltd on behalf of European Hematology Association. This is an open access Abstract Book distributed under the Attribution-NonCommercial-NoDerivs (CC BY-NC-ND), which allows third parties to download the articles and share them with others as long as they credit the author and the Abstract Book, but they cannot change the content in any way or use them commercially.
**Abstract Book Citations:** Authors, Title, HemaSphere, 2026;10:(S1):pages. The abstract book DOIs can be found on the "Author Information" page.
**Disclaimer:** Articles published in the journal HemaSphere exclusively reflect the opinions of the authors. The authors are responsible for all content in their abstracts including accuracy of the facts, statements, citing resources, etc.

### Results

Eighty-three patients were included (median age 67 years). PET2 was centrally reviewed in 80 patients: 48/80 (60%) were PET2-negative (4 cycles recommended) and 32/80 (40%) PET2-positive (6 cycles); 71/80 (89%) received the target number of cycles. CR at end of chemotherapy was achieved in 68/83 (82%; 95%CI 72–90). With median follow-up for PFS of 34 months (95%CI 33–38), 3-year PFS was 83% (95%CI 74–93) and 3-year OS 85% (95%CI 76–94). Relapse/progression was rare (3-year event-free rate 92% [95%CI 84–99]); additional PFS events since first publication (Ferdinandus JCO 2025) were two deaths both after prior CR (27 months: secondary malignancy; 36 months: unknown cause). Secondary malignancies occurred in 12/83 patients (14%; including 1 new solid tumor); no AML/MDS was observed. One year after treatment, no grade 4 late toxicities and one grade 3 (musculoskeletal) adverse event were reported. Persistent peripheral neuropathy at last follow-up was uncommon (9 patients; 8 grade 1, 1 missing grade) and efficacy was broadly consistent across frailty groups, while persisting toxicity/organ dysfunction occurred more often among unfit/frail patients.

### Summary/Conclusion

In older patients with AS-cHL, PET-guided BrECADD is feasible and provides high long-term disease control, with very low relapse/progression rates. Severe late toxicity was rare, and no AML/MDS was observed. These results support PET-guided BrECADD as an effective first-line option for patients above 60 years with AS-cHL.

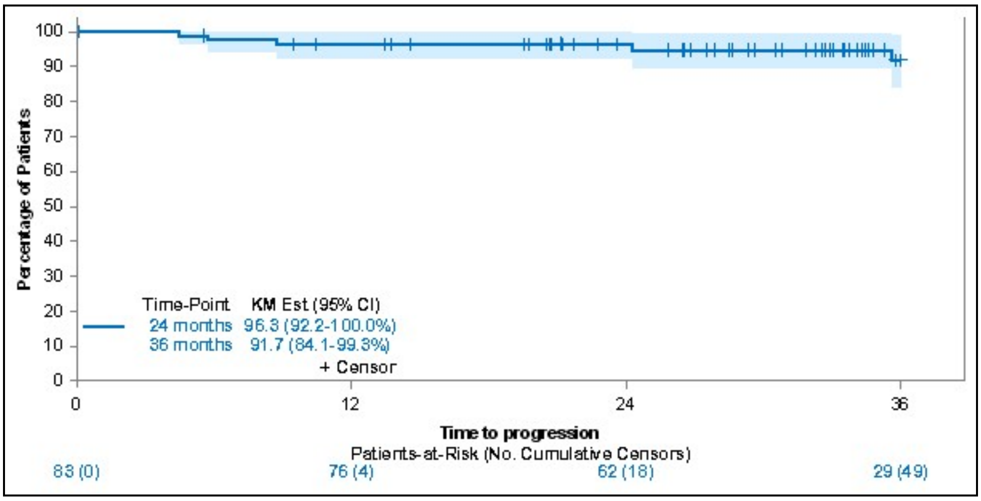

Time to progression in the HD21 older cohort

Copyright Information: (Online) ISSN: 2572-9241
©2026 The Author(s). HemaSphere is published by John Wiley & Sons Ltd on behalf of European Hematology Association. This is an open access Abstract Book distributed under the Attribution-NonCommercial-NoDerivs (CC BY-NC-ND), which allows third parties to download the articles and share them with others as long as they credit the author and the Abstract Book, but they cannot change the content in any way or use them commercially.
Abstract Book Citations: Authors, Title, HemaSphere, 2026;10:(S1):pages. The abstract book DOIs can be found on the “Author Information” page.
Disclaimer: Articles published in the journal HemaSphere exclusively reflect the opinions of the authors. The authors are responsible for all content in their abstracts including accuracy of the facts, statements, citing resources, etc.

### (S222) AVELUMAB IN THE FRONT-LINE TREATMENT OF ADVANCED CLASSICAL HODGKIN LYMPHOMA (AVENUE)-FINAL 3-YEAR RESULTS OF PHASE II STUDY

**Topic:** 17. Hodgkin lymphoma - Clinical

Aisling Barrett*[1], Amy Kirkwood[2], Laura Clifton-Hadley[2], Charlotte Tyson[2], Emma Barsoum[2], Ruth Pettengell[3], Patrick Medd[4], Pam McKay[5], Fiona Miall[6], John Radford[7], Sunil Iyengar[8], Amit Sud[8, 9], Neil Phillips[10], Cathy Burton[11], Karl Peggs[12], Arzhang Ardavan[13], Andrew Grigg[14], Sze Ting Lee[15], Andrew Scott[15], Sally Barrington[16], Eliza Hawkes[14], Graham Collins[17]

*[1]University of Oxford, Nuffield Department of Population Health, Oxford, United Kingdom;[2]University College London, CR UK & UCL Cancer Trials Centre, London, United Kingdom; [3]St. George's Healthcare NHS Trust, Haematology and Medical Oncology, United Kingdom; [4]University Hospitals Plymouth NHS Trust, Haematology, Plymouth, United Kingdom;[5]Beatson West of Scotland Cancer Centre, Haematology, Glasgow, United Kingdom; [6]University Hospitals of Leicester NHS Trust, Leicester, United Kingdom; [7]The Christie NHS Foundation Trust and University of Manchester, Medical Oncology, Manchester, United Kingdom; [8]Royal Marsden Hospital and Institute of Cancer Research, London, United Kingdom;[9]The Institute of Cancer Research, The Division of Genetics and Epidemiology, London, United Kingdom; [10]University Hospitals of North Midlands NHS Trust, Stoke-On-Trent, United Kingdom; [11]Leeds Teaching Hospitals NHS Trust, Haematology, Leeds, United Kingdom;[12]UCL Cancer Institute, London, United Kingdom; [13]University of Oxford, Physics, Oxford, United Kingdom; [14]Austin Health, Olivia Newton-John Cancer Research and Wellness Centre, Melbourne, Australia; [15]Austin Health, Department of Molecular Imaging and Therapy, Melbourne, Australia; [16]King's College London, King's College London and Guy's and St Thomas' PET Centre,, London, United Kingdom; [17]Oxford University Hospitals NHS Foundation Trust, Oxford Cancer and Haematology Centre, Oxford, United Kingdom;*

### Background

Programmed death ligand (PD-L) 1 inhibitors induce deep remissions in relapsed/ refractory classic Hodgkin lymphoma (cHL). Combination with standard chemotherapy has demonstrated high cure rates in the upfront setting. PDL1 inhibitor use remains poorly explored.

### Aims

AVENuE was a phase II non-randomised study designed to determine safety and efficacy of the PD-L1 inhibitor avelumab followed by standard chemotherapy in 16-60 year old patients with advanced stage treatment-naïve cHL. Financial support and product donation was provided as an investigator-initiated research donation from Pfizer Limited, on behalf of Pfizer-Merck Alliance.

### Methods

Patients received 2 cycles (4 doses) of avelumab with subsequent positron emission tomography (PET-1) followed by the RATHL approach. The primary end-point was overall response rate (ORR) by Lugano criteria at PET-1 (complete or partial metabolic response). Key secondary end-points included survival outcomes, toxicity and successful completion of chemotherapy. Indeterminate response at PET-1 was determined by LYRIC criteria.
A sample size of 47 was calculated using an A'hern design with 40% ORR required for demonstration of efficacy (5% 1-sided alpha, 90% power; ORR<20% not considered worthwhile). Survival end-points were assessed using Kaplan-Meier.

**Copyright Information:** (Online) ISSN: 2572-9241
©2026 The Author(s). HemaSphere is published by John Wiley & Sons Ltd on behalf of European Hematology Association. This is an open access Abstract Book distributed under the Attribution-NonCommercial-NoDerivs (CC BY-NC-ND), which allows third parties to download the articles and share them with others as long as they credit the author and the Abstract Book, but they cannot change the content in any way or use them commercially.
**Abstract Book Citations:** Authors, Title, HemaSphere, 2026;10:(S1):pages. The abstract book DOIs can be found on the "Author Information" page.
**Disclaimer:** Articles published in the journal HemaSphere exclusively reflect the opinions of the authors. The authors are responsible for all content in their abstracts including accuracy of the facts, statements, citing resources, etc.

### Results

47 eligible patients were registered with a median age of 30 years. Two ineligible patients were replaced but contributed to the safety data. 64% were male, 52% had stage IV disease and 40% had an international prognostic score of 3 or above. 44 patients fully completed avelumab; 3 did not complete both cycles (1 each due to immune-related adverse event (irAE), progressive disease (PD) and tumour flare) of whom the former 2 underwent PET-1.
The ORR in the 46 assessable patients at PET-1 was 44.7% (90% confidence interval (CI) 32.2-57.7), thus meeting the primary end-point. Following 2X ABVD, 42/47 (89.4%) achieved complete metabolic response (including the post-avelumab PD) with 5 patients subsequently escalated to BEACOPP. With a median follow-up of 45 months, 4 patients have relapsed with no deaths, leading to a 3-year progression-free survival (PFS) of 90.8% (95% CI: 77.3–96.4) and 3-year overall survival of 100%. The 3-year PFS for 21 patients with overall response at PET-1 was 100% and was 82.6% (95% CI: 50.0-93.0) in 25 patients with an indeterminate response (all of whom had progressive metabolic disease by Lugano criteria at PET-1).
59.2% of patients had an AE of any grade related to avelumab; worst grade was 3 in 14.3% and 4 in 4.1%. Avelumab-related grade 3 irAEs included colitis (n=1) and autoimmune hepatitis (n=1); both resolved and planned chemotherapy was subsequently completed. Other notable AEs considered related to avelumab included tumour flare (n=2; grade 3) and renal tubular acidosis (n=1; grade 4). 36.7% of patients developed infusion-related reactions on avelumab (all grade 1-2).
Translational analysis included radiomic and immunohistochemical analysis. Small numbers of events made definitive conclusions difficult, but an association was seen between PFS and maximum standardised uptake value on the baseline PET ($p$=0.001) with 3 of 4 relapses occurring in patients with the highest values.

### Summary/Conclusion

The excellent PFS in this study with acceptable toxicity suggests that upfront PD-L1 inhibition followed by conventional chemotherapy is a treatment strategy worth pursuing in patients with advanced cHL.

**Copyright Information:** (Online) ISSN: 2572-9241
©2026 The Author(s). HemaSphere is published by John Wiley & Sons Ltd on behalf of European Hematology Association. This is an open access Abstract Book distributed under the Attribution-NonCommercial-NoDerivs (CC BY-NC-ND), which allows third parties to download the articles and share them with others as long as they credit the author and the Abstract Book, but they cannot change the content in any way or use them commercially.
**Abstract Book Citations:** Authors, Title, HemaSphere, 2026;10:(S1):pages. The abstract book DOIs can be found on the “Author Information” page.
**Disclaimer:** Articles published in the journal HemaSphere exclusively reflect the opinions of the authors. The authors are responsible for all content in their abstracts including accuracy of the facts, statements, citing resources, etc.

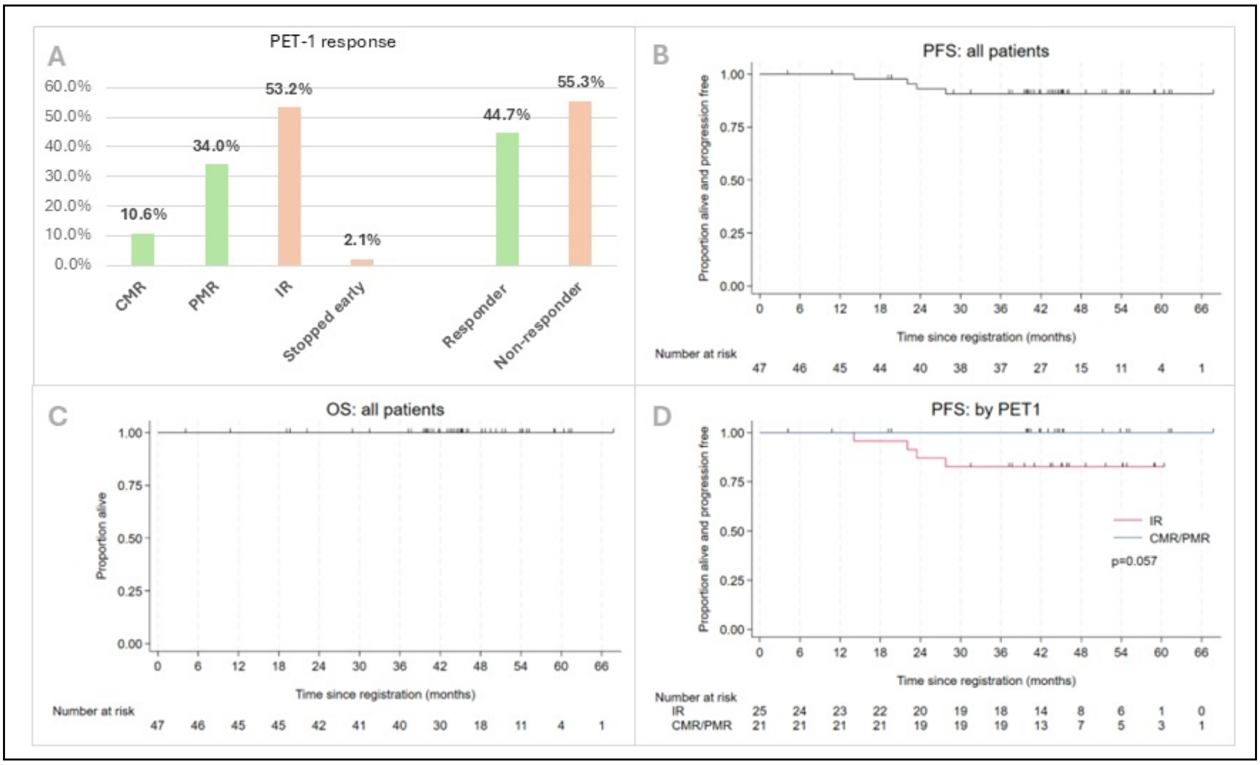

Panel A: Response by PET following 2 cycles of avelumab. Panel B: PFS of all patients following at least 3 years of follow up. Panel C: OS of all patients following at least 3 years of follow up. Panel D: PFS by response following 2 cycles of avelumab. PFS: progression-free survival. OS: overall survival. CMR: complete metabolic response. PMR: partial metabolic response. IR: indeterminate response. PET: positron emission tomography.

**Copyright Information:** (Online) ISSN: 2572-9241
©2026 The Author(s). HemaSphere is published by John Wiley & Sons Ltd on behalf of European Hematology Association. This is an open access Abstract Book distributed under the Attribution-NonCommercial-NoDerivs (CC BY-NC-ND), which allows third parties to download the articles and share them with others as long as they credit the author and the Abstract Book, but they cannot change the content in any way or use them commercially.
**Abstract Book Citations:** Authors, Title, HemaSphere, 2026;10:(S1):pages. The abstract book DOIs can be found on the "Author Information" page.
**Disclaimer:** Articles published in the journal HemaSphere exclusively reflect the opinions of the authors. The authors are responsible for all content in their abstracts including accuracy of the facts, statements, citing resources, etc.

### (S223) INTERIM PET/CT ADAPTED PD-1 INHIBITOR INCORPORATION TO AVOID HIGH-DOSE CHEMOTHERAPY AND RADIOTHERAPY IN CLASSICAL HODGKIN'S LYMPHOMA

**Topic:** 17. Hodgkin lymphoma - Clinical

Shenmiao Yang*[1], Liheng Qiu[1], Rong Wei[1, 1], Yang Liu[1], Xuelin Dou[1], Nan Peng[1], Jin Lu[1], Norbert Schmitz[1, 2]

*[1]Peking University People's Hospital, Beijing, China;[2]Department of Hematology and Oncology, University Hospital Muenster, Muenster, Germany;*

### Background

In classical Hodgkin lymphoma (cHL), results of interim PET/CT after 2 treatment cycles (iPET2) are increasingly being used to modify further therapy. Response-adapted first-line regimens combining chemotherapy with PD-1 inhibitors are currently under investigation, with promising early results.

### Aims

To investigate if pts showing partial response defined as DS 4 on iPET2, benefit from adding PD-1 inhibitor to chemotherapy by improving efficacy while avoiding the toxicities associated with high-dose therapy and ASCT or radiotherapy.

### Methods

This was a prospective, single-center, phase 2 study. cHL pts with unfavorable stage II, stage III or IV disease received ABVD followed by iPET2. Patients continued therapy according to the results of iPET2: early-stage pts with DS 1–3 received 2 more cycles of ABVD; patients with DS 4 received 2 cycles of AVD combined with PD-1 inhibitor (Sintilimab 200mg, d1, Doxorubicin 25mg/m2, d1, d15, Vinblastine 6mg/m2, d1, d15, Dacarbazine 375mg/m2, d1, d15, Q28d) followed by 4 cycles of PD-1 inhibitor. Advanced stage patients received 4 additional cycles of ABVD (DS 1-3), or four cycles of AVD combined with Sintilimab followed by 2 cycles of Sintilimab monotherapy (DS 4). Patients with DS 5 underwent salvage chemotherapy. Young, fit patients achieving at least a PR proceeded to ASCT. Radiotherapy was not mandatory for any patient on study.

**Copyright Information:** (Online) ISSN: 2572-9241
©2026 The Author(s). HemaSphere is published by John Wiley & Sons Ltd on behalf of European Hematology Association. This is an open access Abstract Book distributed under the Attribution-NonCommercial-NoDerivs (CC BY-NC-ND), which allows third parties to download the articles and share them with others as long as they credit the author and the Abstract Book, but they cannot change the content in any way or use them commercially.
**Abstract Book Citations:** Authors, Title, HemaSphere, 2026;10:(S1):pages. The abstract book DOIs can be found on the "Author Information" page.
**Disclaimer:** Articles published in the journal HemaSphere exclusively reflect the opinions of the authors. The authors are responsible for all content in their abstracts including accuracy of the facts, statements, citing resources, etc.

### Results

From January 2019 to February 2026, a total of 130 pts were enrolled, including 43 pts with unfavorable stage II disease and 87 pts with stage III/IV disease. The median age was 36.5 years (range: 17–86 yrs); 73 pts were male; 40 pts (30.8%) presented with B symptoms. iPET2 results for early-stage pts were as follows: DS 1–3 in 34 pts (79.1%), DS 4 in 8 (18.6%), and DS 5 in 1 pt (2.3%). EOTPET showed CR in 39 pts (90.7%), PR in 3 pts (7.0%), and PD in 1 pt (2.3%). iPET2 in advanced stage pts gave the following results: DS 1-2 in 64 pts (73.6%), DS 4 in 17 (19.5%), and DS 5 in 6 (6.9%). EOT evaluation showed CR in 75 (86.2%), PR in 4 (4.6%), and PD in 8 (9.2%). One-year PFS rates by iPET2 for early-stage pts were: 90.4% for DS 1–3, 100% for DS 4. With a median observation time of 26 months (range: 3-84 months) no patient relapsed after the first year. For advanced-stage pts with DS 1–3, the 1-, 3-, and 5-year PFS rates were 96.5%, 85.3%, and 80.8%, respectively. For patients with DS 4, the corresponding rates were 80.0%, 73.3%, and 73.3%. (Figure)

No cases of Grade 4 neutropenia occurred during treatment. Two patients experienced transient hyperthyroidism (increase of T3, T4), one pt developed transient proteinuria and hematuria. All were considered irAEs. All 3 pts discontinued PD-1 inhibitor and were treated with oral prednisone, resulting in resolution of symptoms and abnormal laboratory findings; they subsequently switched to alternative therapies.

Patients with DS 5 at iPET2 and pts with later PD received salvage therapy; those achieving at least PR underwent ASCT. Only 3 pts died: one pt due to PD at 9 months, one pt refused further treatment after PD and died at 9 months, one pt died in PR with salvage therapy from COVID-19 at 67 months. The 5-year OS for the advanced-stage group was 97.3%.

### Summary/Conclusion

A strategy of adding PD-1 inhibitor to standard chemotherapy in cHL pts not achieving complete metabolic remission (CMR) at iPET2 results in promising outcomes for both early-stage unfavorable and advanced-stage cHL patients. This is particularly evident in patients with DS 4 at iPET2.

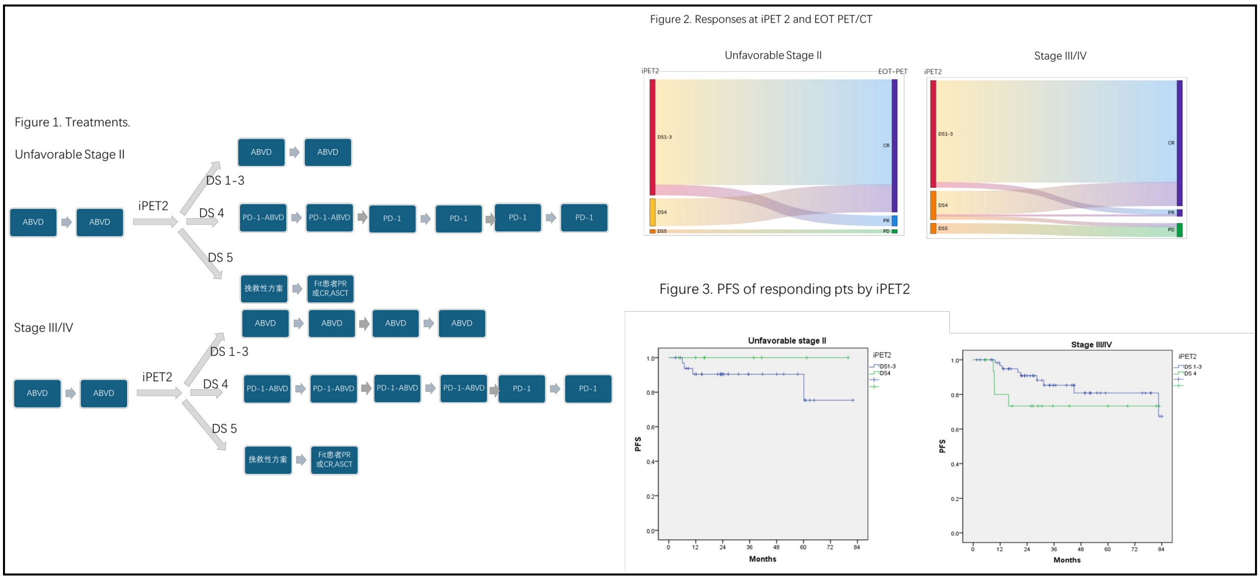

Figure: iPET2 adapted PD-1 included treatment in Hodgkin's lymphoma.

**Copyright Information:** (Online) ISSN: 2572-9241
©2026 The Author(s). HemaSphere is published by John Wiley & Sons Ltd on behalf of European Hematology Association. This is an open access Abstract Book distributed under the Attribution-NonCommercial-NoDerivs (CC BY-NC-ND), which allows third parties to download the articles and share them with others as long as they credit the author and the Abstract Book, but they cannot change the content in any way or use them commercially.
**Abstract Book Citations:** Authors, Title, HemaSphere, 2026;10:(S1):pages. The abstract book DOIs can be found on the "Author Information" page.
**Disclaimer:** Articles published in the journal HemaSphere exclusively reflect the opinions of the authors. The authors are responsible for all content in their abstracts including accuracy of the facts, statements, citing resources, etc.

### (S224) ULTRASENSITIVE CTDNA MRD BY PHASED-SEQ COMPLEMENTS PET/CT AND PREDICTS TREATMENT FAILURE IN EARLY AND ADVANCED CLASSIC HODGKIN LYMPHOMA

**Topic:** 17. Hodgkin lymphoma - Clinical

Jan Lukas Boegeholz*[1], Cedric Rossi[2], Jordan Goldstein[3], David Kurtz[3], Mohammad Shahrokh Esfahani[3], Stefan Alig[3], Andrea Garofalo[3], Chih Long Liu[3], Joe Schroers-Martin[3], Mari Olsen[3], Xiaoman Kang[3], Feng Tian[3], Marc André[4], Luc Matthieu Fornecker[5], Olivier Casasnovas[2], Maximillian Diehn[3], Herve Ghesquieres[6], Ash Alizadeh[3]

*[1]University Hospital Zurich, Zurich, Switzerland; [2]Université Bourgogne Europe, CHU Dijon Bourgogne, Dijon, France;[3]University of Stanford, Stanford, United States of America; [4]CHU UCL Namur, Namur, Belgium;[5]CHU de Strasbourg, Strasbourg, France; [6]Hôpital Lyon Sud HCL, Lyon, France;*

### Background

Response-adapted therapy in classic Hodgkin lymphoma (cHL) is guided by PET/CT imaging but is limited by suboptimal sensitivity for minimal residual disease (MRD) and high false-positive rates at interim assessments. Circulating tumor DNA (ctDNA) has demonstrated diagnostic and prognostic utility in cHL at baseline and during post-treatment surveillance (Spina et al, Blood 2018). The ultrasensitive PhasED-seq assay enables MRD detection at extremely low variant allele fractions (Kurtz et al., Nat Biotech 2021).

### Aims

We evaluated the prognostic value of MRD across different cHL disease stages.

### Methods

We analyzed newly diagnosed cHL patients treated in a prospective clinical trial (PVAB) and in two independent real-world cohorts (REALYSA, BIOLYMPH) with longitudinal follow-up (median of  4.2 years). Pretreatment plasma was used to identify phased variants for longitudinal ctDNA MRD monitoring by PhasED-seq as previously described (Kurtz et al., Nat Biotechnol 2021).

**Copyright Information:** (Online) ISSN: 2572-9241
©2026 The Author(s). HemaSphere is published by John Wiley & Sons Ltd on behalf of European Hematology Association. This is an open access Abstract Book distributed under the Attribution-NonCommercial-NoDerivs (CC BY-NC-ND), which allows third parties to download the articles and share them with others as long as they credit the author and the Abstract Book, but they cannot change the content in any way or use them commercially.
**Abstract Book Citations:** Authors, Title, HemaSphere, 2026;10:(S1):pages. The abstract book DOIs can be found on the “Author Information” page.
**Disclaimer:** Articles published in the journal HemaSphere exclusively reflect the opinions of the authors. The authors are responsible for all content in their abstracts including accuracy of the facts, statements, citing resources, etc.

### Results

We identified a total of 166 pts evaluable for ctDNA analysis. Among patients, 27% had limited-stage and 73% had advanced-stage cHL. Plasma samples were available at baseline and interim assessments (including C3D1 for n = 116 and EOT for n = 110). Frontline treatment regimens were predominantly PVAG/PVAB-based (n = 73), ABVD-based (n = 53), and BEACOPP-containing therapy (n = 30). Median ctDNA burden increased with stage (stage I: 1.55 [0.40–2.94] vs stage IV: 2.38 [1.83–3.69] log hGE/mL) but did not differ across histologic subtypes (Kruskal–Wallis p = 0.634). ctDNA correlated with TMTV (ρ = 0.34, p < 0.001). A combined risk score stratified patients into distinct prognostic groups (HR 2.22, 95% CI 1.16–4.26; p = 0.014). Importantly, phased variant yield did not differ by stage (Kruskal–Wallis p = 0.058), supporting the feasibility of MRD assessment also in early-stage disease. In limited-stage cHL, interim ctDNA positivity at C3D1 was strongly associated with inferior PFS (HR 40.0, 95% CI 4.48–357; log-rank p < 0.001). At EOT, detectable ctDNA remained highly prognostic (HR 13.5, 95% CI 2.38–76.2; log-rank p < 0.001). EOT PET positivity was also associated with markedly inferior PFS (HR 54.8, 95% CI 5.57–540; log-rank p < 0.001). At end of treatment in advanced-stage disease, detectable ctDNA strongly predicted inferior PFS (HR 5.38, 95% CI 2.74–10.6; p < 0.001), OS (HR 7.05, 95% CI 3.00–16.6; p < 0.001), and relapse risk (HR 5.76, 95% CI 2.71–12.3; p < 0.001). EOT PET positivity was also highly prognostic for inferior PFS (HR 10.8, 95% CI 5.09–23.1; p < 0.001). Strikingly, combined EOT PET/ctDNA stratification identified three distinct prognostic groups (global log-rank p < 0.001).At EOT, ctDNA positivity emerged as the strongest independent prognostic factor in multivariate analysis for relapse (HR 4.76, 95% CI 1.77–12.83; p = 0.002) and OS (HR 6.94, 95% CI 2.61–18.43; p < 0.001), whereas PET positivity independently predicted relapse (HR 8.31, 95% CI 3.17–21.74; p < 0.001) but not OS. In contrast, IPS ≥3 was not independently associated with either endpoint.

### Summary/Conclusion ctDNA-based MRD assessment during therapy and at EOT is a strong predictor of treatment failure in cHL. In limited-stage disease, ctDNA-based MRD demonstrates significant prognostic discrimination as early as after two cycles of therapy. Notably, ctDNA-based MRD provides complementary risk stratification beyond PET/CT.

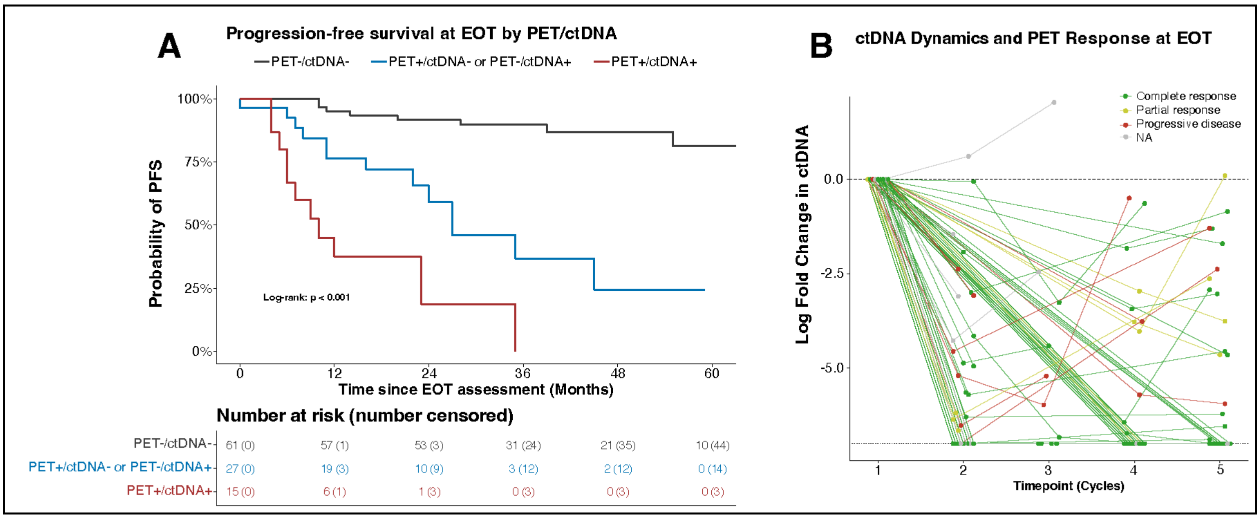

A: Progression free Survival by EOT and ctDNA MRD in patients with advanced stage cHL. B: Dynamics of ctDNA concentrations at different therapy timepoints and Response by EOT PET in colors.

**Copyright Information:** (Online) ISSN: 2572-9241
©2026 The Author(s). HemaSphere is published by John Wiley & Sons Ltd on behalf of European Hematology Association. This is an open access Abstract Book distributed under the Attribution-NonCommercial-NoDerivs (CC BY-NC-ND), which allows third parties to download the articles and share them with others as long as they credit the author and the Abstract Book, but they cannot change the content in any way or use them commercially.
**Abstract Book Citations:** Authors, Title, HemaSphere, 2026;10:(S1):pages. The abstract book DOIs can be found on the "Author Information" page.
**Disclaimer:** Articles published in the journal HemaSphere exclusively reflect the opinions of the authors. The authors are responsible for all content in their abstracts including accuracy of the facts, statements, citing resources, etc.

### (S225) MOSUNETUZUMAB FOR EARLY RELAPSE OF FOLLICULAR LYMPHOMA IN THE NORDIC COUNTRIES (MERLIN, NLG-FL6/ML43841)

**Topic:** 18. Indolent and mantle-cell non-Hodgkin lymphoma - Clinical

Marianne Brodtkorb*[1], Bjorn Wahlin[2], Micaela Hernberg[3], Mads Emil Bjørn[4], Marjukka Pollari[5], Karin Wader[6], Annikki Aromaa-Häyhä[7], Hanne Kuitunen[8], Arne Kolstad[9], Mats Hellstrom[10], Øystein Fluge[11], Ida Blok Sillesen[12], Ola Linden[13], Sara Sjöström[14], Maja Juul[15], Hedda Lerdal[16], Idun Bakke Bø[17], Martin Maisenhölder[18], Paw Jensen[19], Michael Thorsgaard[20], June Myklebust[21], Ole Christian Lingjærde[22]

*[1]Oslo University Hospital, Department of Oncology, Oslo, Norway;[2]Karolinska University Hospital, Department of Hematology, Stockholm, Sweden; [3]HUS Comprehensive Cancer Center, Clinical Trial Unit, Helsinki, Finland;[4]Rigshospitalet, Clinic of Hematology L-4241, Copenhagen, Denmark; [5]Tampere University Hospita, Department of Oncology, Tampere, Finland;[6]St Olavs Hospital, Kreftklinikken, Trondheim, Norway; [7]Kuopio University Hospital, Department of Oncology and Radiotherapy, Kuopio, Finland; [8]Oulu University Hospital, Oulu, Finland; [9]Sykehuset Innlandet HF, Kreftavdelingen og senter for lindrende behandling, Lillehammer, Norway; [10]Uppsala Academic Hospital, Department of Oncology, Uppsala, Sweden; [11]Haukeland University Hospital, Department of Oncology and Medical Physics, Bergen, Norway; [12]Aarhus University Hospital, Department of Hematology, Aarhus, Denmark; [13]Lund University Hospital, Department of Medicine, Lund, Sweden; [14]Norrlands universitetsjukhus, Cancercentrum, Umeå, Sweden; [15]Odense University Hospital, Department of Hematology, Odense, Denmark; [16]Sørlandet Hospital, Department of Medicin, Kristiansand, Norway;[17]Stavanger University Hospital, Department of Haematology, Stavanger, Norway; [18]The University Hospital of Northern Norway, Department of Medicine, Tromsø, Norway; [19]Aalborg University Hospital, Department of Hematology, Aalborg, Denmark;[20]Vejle Hospital, Department of Medicine, Vejle, Denmark; [21]Oslo University Hospital, Department of Cancer Immunology, Institute for Cancer Research, Oslo, Norway; [22]University of Oslo, Institute for Informatics, Oslo, Norway;*

### Background

Common 1$^{st}$ line treatment for patients with symptomatic follicular lymphoma (FL) is rituximab (R) alone or R combined with chemotherapy or lenalidomide. These regimens have a median survival of 18-20 years (Marcus et al NEJM 2017, Zucca et al Blood 2019). In 20-40% of patients the disease responds poorly or relapses within 24 months of initial treatment (commonly termed POD24). POD24 patients respond poorly to standard 2$^{nd}$ line immuno-chemotherapy, and five-year overall survival is approximately 50% (Casulo et al JCO 2015, Lockmer et al JCO 2019, Freeman et al Blood 2019). Mosunetuzumab is a new bispecific T-cell engaging antibody that has shown high response rates and long response duration in relapsed/refractory FL, including patients with a previous history of POD24 (Budde et al Lancet Oncol 2022, Sehn et al Blood 2024). Mosunetuzumab is approved for FL in 3$^{rd}$ and later treatment lines.

### Aims

The MERLIN trial's main objective is to investigate the efficacy of subcutaneous (SC) mosunetuzumab in 2$^{nd}$ line treatment of FL with POD24.

**Copyright Information:** (Online) ISSN: 2572-9241
©2026 The Author(s). HemaSphere is published by John Wiley & Sons Ltd on behalf of European Hematology Association. This is an open access Abstract Book distributed under the Attribution-NonCommercial-NoDerivs (CC BY-NC-ND), which allows third parties to download the articles and share them with others as long as they credit the author and the Abstract Book, but they cannot change the content in any way or use them commercially.
**Abstract Book Citations:** Authors, Title, HemaSphere, 2026;10:(S1):pages. The abstract book DOIs can be found on the "Author Information" page.
**Disclaimer:** Articles published in the journal HemaSphere exclusively reflect the opinions of the authors. The authors are responsible for all content in their abstracts including accuracy of the facts, statements, citing resources, etc.

### Methods

The MERLIN trial is a one-armed phase II multicentre trial including 80 patients diagnosed with FL without response to or with relapse/progression within 24 months of starting first line rituximab-containing treatment. Additional inclusion criteria were age ≥ 18 years, WHO performance status ≤ 2 and measurable disease. Mosunetuzumab was administered SC in three weekly step-up doses during the first cycle and one dose on day 1 of the following 21-days cycles. Patients with complete response assessed by FDG-PET-CT after 8 cycles cease to receive treatment. Patients with partial response or stable disease can receive up to 17 cycles unless progressive disease or unacceptable adverse events occur. Patients are followed for at least two years and a maximum of five years. The main trial endpoint is progression free survival following mosunetuzumab SC.

### Results

80 patients with POD24 were included between Oct 2023 and Sept 2025. Patients were aged 35-80 yrs (median 62 yrs) and the male:female ratio was 1.35. Per 1 March 2026, the median observation time was 13.8 months (4.6-29.5), the median number of administered cycles was 8 (3-17) and 66 patients were still on treatment. All patients reached response evaluation after cycle 3, and 63 patients have reached response evaluation after cycle 8. The overall and complete response rate assessed by CT after cycle 3 was 84% and 6%, respectively. The overall and complete response rate assessed by PET-CT after cycle 8 was 82% and 65%, respectively. Progression or relapse was observed in 20 patients. Three patients died. The preliminary profile of adverse events (AEs) is in line with previous reports of mosunetuzumab in FL. A total of 660 AEs have been registered, of which 602 (91%) were of grade 1-2 and 58 (9%) were of grade 3-4. There were no fatal AEs. All patients had at least one AE, 35 had at least one AE of grade 3-4. Live tumor cells and peripheral blood mononuclear cells (PBMCs) were harvested before start of treatment and after cycle 3. Single cell RNA sequencing of these samples from two patients shows rapid elimination of B-cells from tumor and blood and allows a detailed mapping of immune cell dynamics, including trafficking of various T-cell substes during treatment.

### Summary/Conclusion

Monotherapy with mosunetuzumab SC produces high response rates and is well tolerated in POD24 patients treated in 2nd line.

**Copyright Information:** (Online) ISSN: 2572-9241
©2026 The Author(s). HemaSphere is published by John Wiley & Sons Ltd on behalf of European Hematology Association. This is an open access Abstract Book distributed under the Attribution-NonCommercial-NoDerivs (CC BY-NC-ND), which allows third parties to download the articles and share them with others as long as they credit the author and the Abstract Book, but they cannot change the content in any way or use them commercially.
**Abstract Book Citations:** Authors, Title, HemaSphere, 2026;10:(S1):pages. The abstract book DOIs can be found on the “Author Information” page.
**Disclaimer:** Articles published in the journal HemaSphere exclusively reflect the opinions of the authors. The authors are responsible for all content in their abstracts including accuracy of the facts, statements, citing resources, etc.

### (S226) SAFETY AND EFFICACY OF SUBCUTANEOUS MOSUNETUZUMAB PLUS LENALIDOMIDE INDUCTION THERAPY FOLLOWED BY MOSUNETUZUMAB MAINTENANCE IN PREVIOUSLY UNTREATED FOLLICULAR LYMPHOMA

**Topic:** 18. Indolent and mantle-cell non-Hodgkin lymphoma - Clinical

Emmanuel Bachy*[1], Franck Morschhauser[2], Toby A. Eyre[3], Raul Cordoba[4], Roch Houot[5], Mark Bishton[6], Hui-Lai Zhang[7], Liqun Zou[8], Laura Galvez-Carvajal[9], Wendy Osborne[10], Catherine Thieblemont[11], Bertrand Anz[12], Andrea Knapp[13], Jason Sit[14], Naomi Chang[15], Paloma Hauser[14], Vivian Chen[14], Elicia Penuel[14], Michael C. Wei[14], Enkhtsetseg Purev[14], Krish Patel[16]

*[1]Centre Hospitalier Lyon-Sud, Lyon, France; [2]CHU de Lille, Université de Lille, Lille, France; [3]Oxford University Hospital Oxford, UK, Oxford, United Kingdom; [4]Fundación Jiménez Díaz University Hospital, Madrid, Spain; [5]CHU de Rennes, Université de Rennes, Rennes, France; [6]University of Nottingham, Nottingham, United States of America; [7]Tianjin Medical University Cancer Institute and Hospital, Tianjin, China; [8]West China Hospital, Sichuan University, Chengdu, China; [9]Regional and Virgen de la Victoria University Hospitals, Málaga, Spain; [10]Freeman Hospital Newcastle, Newcastle University, Newcastle upon Tyne, United Kingdom; [11]University Paris Cité; Assistance Publique & Hôpitaux de Paris, Hôpital Saint-Louis, Hemato-oncologie, Paris, France; [12]Tennessee Oncology, Chattanooga, TN, United States of America; [13]F. Hoffmann-La Roche Ltd, Basel, Switzerland; [14]Genentech, Inc., South San Francisco, CA, United States of America; [15]Hoffmann-La Roche Ltd, Mississauga, ON, Canada; [16]Swedish Cancer Institute, Seattle, WA, United States of America;*

### Background

In the Phase Ib/II CO41942 study (NCT04246086), mosunetuzumab (M) plus lenalidomide (Len) induction therapy showed promising results in patients (pts) with previously untreated (1L) high tumor burden follicular lymphoma (FL; Morschhauser et al. ASH 2023; Eyre et al. ICML 2025).

### Aims

The primary objective was to evaluate the efficacy and safety of M+Len induction followed by M SC maintenance in patients with newly diagnosed FL.

### Methods

Pts with 1L FL requiring treatment per GELF criteria and an ECOG PS of 0–2 were included. All patients provided informed consent. Pts received 12 cycles (Cycle [C]1, 21 days; C2–12, 28 days) of M+Len (M SC: C1 Day [D]1, 5mg; C1D8, C1D15, and C2–12 D1, 45mg; Len [oral]: D1–21 of C2–12, 20mg). Responders could receive M SC (45mg) monotherapy maintenance every 8 weeks for 9 cycles (C13–21). Hospitalization was not required. Anti-infective prophylaxis followed institutional standards.

**Copyright Information:** (Online) ISSN: 2572-9241
©2026 The Author(s). HemaSphere is published by John Wiley & Sons Ltd on behalf of European Hematology Association. This is an open access Abstract Book distributed under the Attribution-NonCommercial-NoDerivs (CC BY-NC-ND), which allows third parties to download the articles and share them with others as long as they credit the author and the Abstract Book, but they cannot change the content in any way or use them commercially.
**Abstract Book Citations:** Authors, Title, HemaSphere, 2026;10:(S1):pages. The abstract book DOIs can be found on the “Author Information” page.
**Disclaimer:** Articles published in the journal HemaSphere exclusively reflect the opinions of the authors. The authors are responsible for all content in their abstracts including accuracy of the facts, statements, citing resources, etc.

**Results**

As of September 8, 2025, 43 pts received induction therapy. The maintenance was optional for the initial 40 patients, and among 31 patients who were eligible to receive maintenance (achieved PR or CR at the end of induction), 16 pts proceeded to M maintenance at the discretion of the local investigator. Median age was 62 years (range: 28–83), 58% were male and 47% had a FLIPI score of 3/4. The median number of M doses during induction and maintenance was 14 (range: 2–27).
The best overall response rate was 90% and complete response (CR; as best response) rate was 88%. After a median follow-up of 30.6 months (range: 0-34), median duration of response (DOR), duration of CR (DOCR), progression-free survival (PFS) and overall survival (OS) were not reached. The 24-month event-free rates for DOR, DOCR, PFS and OS were 94% (95% CI: 86–100), 91% (95% CI: 81–100), 87% (95% CI: 76–98), and 97% (95% CI: 92–100), respectively. All pts reported ≥1 adverse event (AE; serious AEs [SAE], 44.2%; Grade [Gr] 3/4, 69.8%). The most common AEs were injection site reactions (ISRs; 72.1%; all Gr 1/2), neutropenia (60.5%; Gr 3/4, 48.8%), and cytokine release syndrome (CRS; 55.8%; all Gr 1/2). Among patients who experienced ISR, 37.2% experienced multiple events. All CRS events occurred in Cycles 1-2, and none during maintenance. Grade 3 infections occurred in 11.6% of pts, no patients experienced grade 4 or 5 infections. The most common infections were COVID-19 and pneumonia. No pts experienced Gr 5 AEs, febrile neutropenia, or ICANS during induction or maintenance.

**Summary/Conclusion**

M+Len induction with M maintenance showed durable responses and manageable safety, with low rates of AEs during maintenance in pts with high tumor burden 1L FL. These results highlight the promise of this chemotherapy-free regimen, although further follow-up is needed to confirm these findings. Biomarker data will be presented.

**Copyright Information:** (Online) ISSN: 2572-9241
©2026 The Author(s). HemaSphere is published by John Wiley & Sons Ltd on behalf of European Hematology Association. This is an open access Abstract Book distributed under the Attribution-NonCommercial-NoDerivs (CC BY-NC-ND), which allows third parties to download the articles and share them with others as long as they credit the author and the Abstract Book, but they cannot change the content in any way or use them commercially.
**Abstract Book Citations:** Authors, Title, HemaSphere, 2026;10:(S1):pages. The abstract book DOIs can be found on the "Author Information" page.
**Disclaimer:** Articles published in the journal HemaSphere exclusively reflect the opinions of the authors. The authors are responsible for all content in their abstracts including accuracy of the facts, statements, citing resources, etc.

### (S227) GOLCADOMIDE (GOLCA), A POTENTIAL, FIRST-IN-CLASS, ORAL CELMOD ±RITUXIMAB (R) IN PATIENTS (PTS) WITH RELAPSED/REFRACTORY (R/R) FOLLICULAR LYMPHOMA (FL): PHASE (PH) 1/2 STUDY LONG-TERM FOLLOW-UP (F/U)

**Topic:** 18. Indolent and mantle-cell non-Hodgkin lymphoma - Clinical

Daniel Morillo*[1], Jean-Marie Michot[2], Jason Westin[3], Guilherme Perini[4], Emmanuel Bachy[5], Julio Chavez[6], Javier Munoz[7], Abel Costa Neto[8], Parth Rao[9], Niloufar Marsousi[10], Gang Yang[11], Akshay Sudhindra[11], Michael Pourdehnad[12], Soraya Carrancio[13], Jessica Voetsch[14], Serena Perna[11], Judit Jørgensen[15]

*[1]START Madrid - FJD Early Phase Unit, Fundación Jiménez Díaz University Hospital, Madrid, Spain; [2]Gustave Roussy Institute of Cancer, Département d'Innovation Thérapeutique et d'Essais Précoces (DITEP), Villejuif, France; [3]The University of Texas MD Anderson Cancer Center, Houston, United States of America; [4]Hospital Israelita Albert Einstein, São Paulo, Brazil; [5]Lyon Sud Hospital, Lyon, France; [6]Mayo Clinic, Jacksonville, United States of America; [7]Mayo Clinic, Phoenix, United States of America; [8]Instituto D'Or de Pesquisa e Ensino (IDOR), São Paulo, Brazil; [9]Bristol Myers Squibb, Princeton, United States of America; [10]Bristol Myers Squibb, Boudry, Switzerland; [11]Bristol Myers Squibb, Madison, United States of America; [12]Bristol Myers Squibb, San Francisco, United States of America; [13]Bristol Myers Squibb, San Diego, United States of America; [14]Bristol Myers Squibb, Lawrence Township, United States of America; [15]Aarhus University Hospital, Department of Hematology, Aarhus, Denmark;*

### Background

While outcomes in pts with R/R FL have improved with current SOC treatments (Tx), there remains an unmet need for safe, efficacious, and more convenient chemotherapy-free treatment options (Caridà et al, *Eur J Haematol* 2025).
GOLCA is a potential, first-in-class, oral CELMoD designed for the treatment of lymphoma, with preferential distribution to lymphoid organs and enhanced activity in lymphoma cell lines. GOLCA drives the closed, active conformation of cereblon to induce rapid and deep degradation of Ikaros and Aiolos, leading to direct cell killing (agnostic of cell of origin) and immunomodulatory activity.
GOLCA + R was evaluated in heavily pretreated pts with R/R FL in the two-part, multicenter, first-in-human Ph 1/2 study (CC-99282-NHL-001; NCT03930953); GOLCA + R demonstrated promising efficacy with durable responses (overall response rate [ORR] 97% and complete response rate [CRR] 78%) and a manageable safety profile (Chavez J, et al. ASH 2025, #1006). Here, we report longer f/u in pts with R/R FL from Part B.

### Aims

To evaluate long-term safety and efficacy of oral GOLCA ± R in pts with R/R FL.

### Methods

Eligible pts had R/R FL with ≥2 prior lines of Tx (≥1 if prior anti-CD20 Tx). GOLCA was given orally, once daily at 0.2 mg or 0.4 mg (14 days on/14 days off) ± R for up to 2 years or until progressive disease (PD) or unacceptable toxicity. Primary endpoints included safety and recommended Phase 2 dose determination; secondary endpoints included efficacy and pharmacokinetics.

**Copyright Information:** (Online) ISSN: 2572-9241
©2026 The Author(s). HemaSphere is published by John Wiley & Sons Ltd on behalf of European Hematology Association. This is an open access Abstract Book distributed under the Attribution-NonCommercial-NoDerivs (CC BY-NC-ND), which allows third parties to download the articles and share them with others as long as they credit the author and the Abstract Book, but they cannot change the content in any way or use them commercially.
**Abstract Book Citations:** Authors, Title, HemaSphere, 2026;10:(S1):pages. The abstract book DOIs can be found on the "Author Information" page.
**Disclaimer:** Articles published in the journal HemaSphere exclusively reflect the opinions of the authors. The authors are responsible for all content in their abstracts including accuracy of the facts, statements, citing resources, etc.

### Results

As of September 15, 2025, 60 pts were enrolled. In 0.2 and 0.4 mg groups, median prior Tx lines were 3 and 3, 32% and 32% had prior lenalidomide (LEN), 27% and 29% had prior T-cell–redirecting Tx (CAR T and/or bispecific antibody) and 32% and 32% were refractory to last regimen. Tx is ongoing in 6 (27%) and 17 (45%) pts at 0.2 and 0.4 mg, respectively; discontinuations (d/c) were mostly due to PD.
Neutropenia, an on-target side effect of GOLCA, was the most common grade 3/4 Tx-emergent adverse event (TEAE) occurring in 59% and 68% of pts at 0.2 and 0.4 mg, followed by anemia (9% and 16%) and febrile neutropenia (9% and 8%). Median time to resolution of neutropenia was 8 days. Dose reductions occurred in 14% and 37% of pts at 0.2 and 0.4 mg, most commonly due to neutropenia (5% and 21%) and febrile neutropenia (5% and 5%). No d/c or deaths were from GOLCA-related TEAEs. Non-hematologic TEAEs were infrequent and mostly low grade.
In efficacy-evaluable pts (GOLCA 0.2 and 0.4 mg + R, n=22 and n=36), median f/u was 14.03 months (mo), ORR was 77% (CRR, 41%) at 0.2 mg, and 97% (CRR, 78%) at 0.4 mg (Fig). In the 0.4 mg group, responses were consistent in high-risk subsets including pts with prior LEN (ORR, 100%; CRR, 75%) and/or T-cell–redirecting Tx (ORR, 91%; CRR, 64%). GOLCA 0.4 mg + R demonstrated durable responses (median DOR, 9.17 mo; 91% of pts who achieved a CR remained in CR at cutoff).

### Summary/Conclusion

With longer f/u, GOLCA + R demonstrated promising efficacy with durable responses and no new safety signals. The 0.4 mg group had higher ORR and CRR than 0.2 mg, including in pts with prior LEN-based and/or T-cell–redirecting Tx, with a manageable safety profile at both doses. The results support the development of GOLCA + R as a fixed-duration, chemotherapy-free outpatient Tx in the Ph 3 GOLSEEK-4 study in 2L+ FL (NCT06911502).

Acknowledgement: BMS ChatGPT4 was used to revise existing text with human author input.

Fig. Responses (efficacy-evaluable pts)

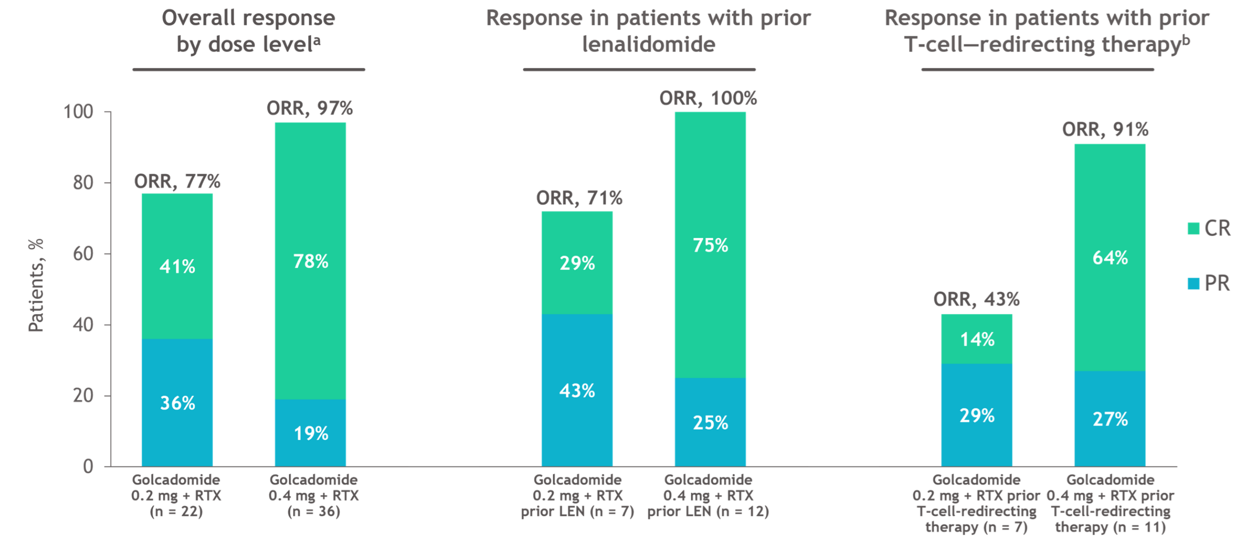

Data cutoff: 15 September 2025.
[a] Efficacy-evaluable population consisting of patients who completed ≥ 1 cycle of golcadomide (taking ≥ 75% of assigned doses) and having a baseline and ≥ 1 postbaseline tumor assessment.; [b] **CAR T and/or bispecific antibody treatment.**
CAR, chimeric antigen receptor; CR., complete response; CRR, complete response rate; LEN, lenalidomide; ORR, overall response rate; PR, partial response; RTX, rituximab.

**Copyright Information:** (Online) ISSN: 2572-9241
©2026 The Author(s). HemaSphere is published by John Wiley & Sons Ltd on behalf of European Hematology Association. This is an open access Abstract Book distributed under the Attribution-NonCommercial-NoDerivs (CC BY-NC-ND), which allows third parties to download the articles and share them with others as long as they credit the author and the Abstract Book, but they cannot change the content in any way or use them commercially.
**Abstract Book Citations:** Authors, Title, HemaSphere, 2026;10:(S1):pages. The abstract book DOIs can be found on the "Author Information" page.
**Disclaimer:** Articles published in the journal HemaSphere exclusively reflect the opinions of the authors. The authors are responsible for all content in their abstracts including accuracy of the facts, statements, citing resources, etc.

### (S228) SUROVATAMIG (AZD0486) PLUS RITUXIMAB IN PREVIOUSLY UNTREATED FOLLICULAR LYMPHOMA (FL): INITIAL SAFETY DATA FROM THE PHASE 3 SOUNDTRACK-F1 TRIAL

**Topic:** 18. Indolent and mantle-cell non-Hodgkin lymphoma - Clinical

Chan Y. Cheah*[1, 2], Monique Menzies Wojtowicz[3], Dok Hyun Yoon[4], Dai Maruyama[5], Bor-Sheng Ko[6], Daisuke Ennishi[7], Hung-Lin Liu[8], Dehui Zou[9], Rebecca Auer[10], Wonseog Kim[11], Yuqin Song[12], Lorenzo Falchi[13], Franck Morschhauser[14], Pier Luigi Zinzani[15], Francesc Garcia Pallarols[16], Safaa Ramadan[17], Stefanie Meyer[18], Xiaofei Chen[16], Mihail Obrocea[16], Ricardo Espinola[16], Tae Min Kim[19]

*[1]Department of Haematology, Sir Charles Gardiner Hospital, Nedlands, Australia;[2]Medical School, University of Western Australia, Perth, Australia; [3]Macquarie University Hospital, Macquarie University, Sydney, Australia; [4]Department of Oncology, Asan Medical Center, University of Ulsan College of Medicine, Seoul, South Korea; [5]Cancer Institute Hospital, Japanese Foundation for Cancer Research, Tokyo, Japan; [6]Department of Hematological Oncology, National Taiwan University Cancer Center, Taipei, Taiwan; [7]Center for Comprehensive Genomic Medicine, Okayama University Hospital, Okayama, Japan;[8]Chang Gung Medical Foundation, Kaohsiung, Taiwan; [9]Institute of Hematology & Blood Diseases Hospital, Chinese Academy of Medical Sciences, Tianjin, China; [10]Barts Health NHS Trust, London, United Kingdom; [11]Division of Hematology-Oncology, Department of Medicine, Samsung Medical Center, Sungkyunkwan University School of Medicine, Seoul, South Korea; [12]Key Laboratory of Carcinogenesis and Translational Research, Department of Lymphoma, Peking University Cancer Hospital & Institute, Beijing, China; [13]Lymphoma Service, Memorial Sloan Kettering Cancer Center, New York, NY, United States of America;[14]Department of Hematology, Claude Huriez Hospital, Lille University Hospital, Lille, France; [15]IRCCS Azienda Ospedaliero-Universitaria di Bologna Istituto di Ematologia "Seràgnoli" and Dipartimento di Scienze Mediche e Chirurgiche, Università di Bologna, Bologna, Italy; [16]AstraZeneca, Gaithersburg, MD, United States of America;[17]AstraZeneca, Barcelona, Spain; [18]AstraZeneca, Waltham, MA, United States of America; [19]Seoul National University Hospital, Seoul National University College of Medicine, Seoul, South Korea;*

### Background

Surovatamig, a CD19xCD3 human IgG4 bispecific T-cell engager, has yielded high and durable response rates (for ≥2.4 mg: overall response rate, 96%; complete response [CR] rate, 92%) in patients (pts) with heavily pretreated FL after up to three years of follow-up.

### Aims

Here, we report initial safety run-in (SRI) data from the surovatamig + rituximab arm of SOUNDTRACK-F1 (NCT06549595), a global, phase 3, multicenter, open-label study evaluating surovatamig + rituximab vs chemotherapy + rituximab in pts with previously untreated FL and high tumor burden.

### Methods

In the SRI part of the study, pts aged ≥18 years with treatment-naïve FL received rituximab (375 $mg/m^2$) on days (D)1, 8, 15 and 22 of cycle (C)1, then D1 of C2–7. Pts were randomized 1:1 to receive intravenous surovatamig at double step-up doses (SUD) of 0.27 mg on C1D15 and 1.0 mg on C1D22, followed by a target dose (TD) of 2.4 mg (dose level [DL] 1) or 7.2 mg (DL2) on C2D1, then 2-weekly up to C7 (28-day cycles). Responders (CR/partial response) received maintenance therapy consisting of surovatamig monotherapy every 4 weeks for 17 cycles. The primary endpoints for the SRI part were safety and tolerability, and selection of the recommended phase 3 dose (RP3D).

**Copyright Information:** (Online) ISSN: 2572-9241
©2026 The Author(s). HemaSphere is published by John Wiley & Sons Ltd on behalf of European Hematology Association. This is an open access Abstract Book distributed under the Attribution-NonCommercial-NoDerivs (CC BY-NC-ND), which allows third parties to download the articles and share them with others as long as they credit the author and the Abstract Book, but they cannot change the content in any way or use them commercially.
**Abstract Book Citations:** Authors, Title, HemaSphere, 2026;10:(S1):pages. The abstract book DOIs can be found on the "Author Information" page.
**Disclaimer:** Articles published in the journal HemaSphere exclusively reflect the opinions of the authors. The authors are responsible for all content in their abstracts including accuracy of the facts, statements, citing resources, etc.

**Results**

At data cutoff (05 Nov 25), 43 pts were enrolled (DL1, n=22; DL2, n=21). Demographics of the DL1/DL2 arms were: ≥60 years, 41%/43%; Ann Arbor stage IV, 82%/90%; high-risk Follicular Lymphoma International Prognostic Index (FLIPI), 27%/43%. Across both arms, median follow-up was 6.3 months (range 3.9–14.6), and median time on treatment was 6.0 months (range 3.7–13.4). Best overall response rate was 95% (21/22 pts) in DL1 and 100% (21/21) in DL2. CR rate was 84% (18/22) in DL1 and 85% (18/21) in DL2. Any-grade (Gr) treatment-emergent adverse events (TEAEs) occurred in 100% of pts; most common TEAEs were infusion-related reactions (DL1/DL2: 36%/52%; mostly related to rituximab), fatigue (36%/43%), myalgia (36%/38%) and decreased neutrophil count (36%/38%). Gr ≥3 TEAEs occurred in 41% and 43% of pts in DL1 and DL2; serious TEAEs occurred in 18% and 29% of pts, respectively. There were no Gr 5 TEAEs or TEAEs leading to treatment discontinuation in either arm. Infusion-related reactions to surovatamig occurred in one pt in each arm (both Gr 2). Cytokine release syndrome (CRS) occurred in 36% (DL1) and 38% (DL2) of pts, mostly during SUD and after the initial TD (all Gr 1–2; **Table**). One immune effector cell-associated neurotoxicity syndrome (ICANS) event per arm occurred during SUD (**Table**). At data cutoff, 75% of pts (12/16) in DL1 and 100% (15/15) in DL2 were minimal residual disease-negative.

**Summary/Conclusion**

Surovatamig in combination with rituximab showed deep responses and manageable safety and tolerability at both dose levels, with comparable TEAEs and no discontinuations. These results supported selection of 7.2 mg as the RP3D; the phase 3 trial is ongoing.

**Table:**

| n (%) | DL1 2.4 mg (n=22) | DL2 7.2 mg (n=21) |
|---|---|---|
| **Any CRS (any Gr)** | 8 (36) | 8 (38) |
| **CRS at SUD (all Gr 1)** | 8 (36) | 5 (24) |
| **CRS at TD (any Gr)** | 3 (14) | 5 (24) |
| **Gr 1** | 3 (14) | 4 (19) |
| **Gr 2** | 0 | 1 (5) |
| **ICANS at SUD (any Gr)** | 1 (5) | 1 (5) |
| **Gr 1** | 1 (5) | 0 |
| **Gr 3** | 0 | 1 (5) |
| **ICANS at TD (any Gr)** | 0 | 0 |

**Copyright Information:** (Online) ISSN: 2572-9241
©2026 The Author(s). HemaSphere is published by John Wiley & Sons Ltd on behalf of European Hematology Association. This is an open access Abstract Book distributed under the Attribution-NonCommercial-NoDerivs (CC BY-NC-ND), which allows third parties to download the articles and share them with others as long as they credit the author and the Abstract Book, but they cannot change the content in any way or use them commercially.
**Abstract Book Citations:** Authors, Title, HemaSphere, 2026;10:(S1):pages. The abstract book DOIs can be found on the "Author Information" page.
**Disclaimer:** Articles published in the journal HemaSphere exclusively reflect the opinions of the authors. The authors are responsible for all content in their abstracts including accuracy of the facts, statements, citing resources, etc.

### (S229) CLINICALLY RELEVANT SUBGROUP ANALYSIS FROM THE RANDOMIZED PHASE 3 EPCORE FL-1 TRIAL: TREATMENT (TX) EFFECT OF EPCORITAMAB WITH LENALIDOMIDE AND RITUXIMAB ($R^2$) IN R/R FOLLICULAR LYMPHOMA (FL)

**Topic:** 18. Indolent and mantle-cell non-Hodgkin lymphoma - Clinical

Marcel Nijland[1], Lorenzo Falchi[2], Kim Linton[3], Neta Goldschmidt[4], Benoit Tessoulin*[5], David Belada[6], Shuo MA[7], Jayr Schmidt[8], Jianzhen Shen[9], Juan Miguel Bergua Burgues[10], Caterina Patti[11], Umberto Vitolo[12], Michal Kwiatek[13], Loic Ysebaert[14], Theodoros Vassilakopoulos[15], Alexander Egle[16], Jean-Francois Larouche[17], Gauri Sunkersett[18], Mark Rocco[19], Nabanita Mukherjee[18], Rastislav Moskal[18], Yukun Li[18], Brian Elliott[20], Franck Morschhauser[21]

*[1]University Medical Center Groningen, University of Groningen, Groningen, The Netherlands; [2]Lymphoma Service, Memorial Sloan Kettering Cancer Center, New York, United States of America; [3]The Christie NHS Foundation Trust, Manchester Cancer Research Centre, and Division of Cancer Sciences, University of Manchester, Manchester, United Kingdom; [4]Hadassah-Hebrew University Medical Center, Jerusalem, Israel; [5]Centre Hospitalier Universitaire de Nantes, Nantes, France; [6]4th Department of Internal Medicine-Haematology, Charles University, Hospital and Faculty of Medicine, Hradec Králové, Czechia; [7]Feinberg School of Medicine, Chicago, United States of America; [8]A.C.Camargo Cancer Center, São Paulo, São Paulo, Brazil; [9]Fujian Medical University Union Hospital, Fuzhou, Fujian, China; [10]Hospital San Pedro de Alcántara, Cáceres, Spain; [11]Oncohematology Unit, A.O.O.R. Villa Sofia Cervello, Palermo, Italy; [12]Department of Medical Oncology, Candiolo Cancer Institute, Candiolo (Turin), Italy; [13]Aidport Clinical Trials Hospital, Skórzewo (Poznan), Poland; [14]Hematology Department, Institut Universitaire du Cancer de Toulouse-Oncopole, Toulouse, France; [15]Laikon General Hospital, National and Kapodistrian University of Athens, Athens, Greece; [16]Paracelsus Medical University, Salzburg, Austria; [17]CHU de Québec-Université Laval, Quebec City, Canada; [18]AbbVie Inc., North Chicago, United States of America; [19]AbbVie Inc., South San Francisco, United States of America; [20]Genmab US, Inc, Plainsboro, United States of America; [21]Hôpital Claude Huriez, Lille, France;*

### Background

Chemoimmunotherapy or $R^2$ are standard of care for R/R FL; however, unmet need remains for novel chemotherapy-free tx to improve outcomes across a wide range of patient (pt) subgroups. Epcoritamab (E), a subcutaneous CD3×CD20 bispecific antibody, is FDA approved as fixed-duration therapy with $R^2$ in 2L+ FL based on the Phase 3 EPCORE FL-1 trial (NCT05409066). The dual primary endpoints were met, with E+$R^2$ showing superiority vs $R^2$ in overall response rate (ORR; *P*<.0001) and progression-free survival (PFS; *P*<.0001) (Falchi *Lancet* 2026). Subgroup analyses may inform tx decisions based on pt characteristics; this analysis was performed to determine if the benefit and tolerability of E+$R^2$ extended across clinically relevant subgroups.

### Aims

Report E+$R^2$ efficacy and safety in clinically relevant subgroups from EPCORE FL-1.

### Methods

Pts ≥18 y old with CD20+ R/R FL, ECOG 0–2, and ≥1 prior therapy were randomized to E+$R^2$ or $R^2$ for ≤12 cycles (C). E was given QW in C1–3 (C1 step-up dosing) and Q4W in C4–12. Oral lenalidomide (Len) was given QD in C1–12 (Days 1–21) and intravenous rituximab QW in C1 and Q4W in C2–5. Clinically relevant subgroups were assessed, including but not limited to baseline FLIPI score (0–2 vs 3–5), disease progression ≤2 y post-frontline tx start (POD24 vs non-POD24), and the validated NHL-5 comorbidity score to assess pt fitness (low vs high+intermediate [H+I]; Gordon *JCO Clin Cancer Inform* 2024). In pts with Len dose modification, efficacy and safety were assessed by Len relative dose intensity (RDI ≤ or >70%).

**Copyright Information:** (Online) ISSN: 2572-9241
©2026 The Author(s). HemaSphere is published by John Wiley & Sons Ltd on behalf of European Hematology Association. This is an open access Abstract Book distributed under the Attribution-NonCommercial-NoDerivs (CC BY-NC-ND), which allows third parties to download the articles and share them with others as long as they credit the author and the Abstract Book, but they cannot change the content in any way or use them commercially.
**Abstract Book Citations:** Authors, Title, HemaSphere, 2026;10:(S1):pages. The abstract book DOIs can be found on the "Author Information" page.
**Disclaimer:** Articles published in the journal HemaSphere exclusively reflect the opinions of the authors. The authors are responsible for all content in their abstracts including accuracy of the facts, statements, citing resources, etc.

**Results**

As of 24May2025, 488 pts were enrolled; 243 in E+$R^2$ and 238 in $R^2$ who received ≥1 study drug dose. Median duration of E was 10.4 mo overall and was similar across FLIPI and NHL-5 subgroups. Between FLIPI subgroups, ORR was higher with E+$R^2$ vs $R^2$ (FLIPI 0–2, 96.5% vs 84.8%; FLIPI 3–5, 93.0% vs 72.6%); high complete response rates (CRR) indicated deeper responses with E+$R^2$ vs $R^2$ in both subgroups (FLIPI 0–2, 86.6% vs 62.1%; FLIPI 3–5, 77.0% vs 35.4%). PFS HRs (95% CIs) favored E+$R^2$ vs $R^2$ for FLIPI 0–2 (0.18 [0.10–0.33]) and FLIPI 3–5 (0.25 [0.15–0.42]) subgroups. In non-POD24, ORR and CRR were higher with E+$R^2$ vs $R^2$ (96.2% vs 85.4% and 85.5% vs 57.6%, respectively); PFS favored E+$R^2$ (HR 0.17 [95% CI 0.09–0.32]). Similar trend was seen in POD24. In NHL-5 subgroups, ORR and CRR favored E+$R^2$ vs $R^2$ for low (94.0% vs 75.8% and 81.3% vs 50.3%, respectively) and H+I (97.4% vs 84.4% and 85.7% vs 49.0%, respectively). PFS HRs (95% CIs) favored E+$R^2$ vs $R^2$ for the NHL-5 low (0.27 [0.17–0.42]) and H+I (0.14 [0.06–0.29]) subgroups. Additional analyses of subgroups showed clinically meaningful and consistent ORR, CRR, and PFS improvement with E+$R^2$ vs $R^2$ (**Table**).

In pts with lower Len RDI, AE rates (ie, neutropenia, infections) were higher but ORR/PFS with E+$R^2$ remained higher than with $R^2$.

The E+$R^2$ safety profile was manageable across all subgroups and was consistent with the overall trial population, with no new safety signals.

**Summary/Conclusion**

E+$R^2$ led to sustained, improved efficacy with manageable safety, regardless of baseline risk. E+$R^2$ benefit was not confined to traditionally higher-risk disease features. Clinically meaningful improvements were observed across lower-risk disease features as well as lower Len RDI, supporting E+$R^2$ as an option for a diverse $R^2$-eligible population. Collectively, the findings from EPCORE FL-1 position fixed-duration E+$R^2$ as a new benchmark standard of care for a broad population of pts with R/R FL in second-line or later tx.

**Copyright Information:** (Online) ISSN: 2572-9241
©2026 The Author(s). HemaSphere is published by John Wiley & Sons Ltd on behalf of European Hematology Association. This is an open access Abstract Book distributed under the Attribution-NonCommercial-NoDerivs (CC BY-NC-ND), which allows third parties to download the articles and share them with others as long as they credit the author and the Abstract Book, but they cannot change the content in any way or use them commercially.
**Abstract Book Citations:** Authors, Title, HemaSphere, 2026;10:(S1):pages. The abstract book DOIs can be found on the "Author Information" page.
**Disclaimer:** Articles published in the journal HemaSphere exclusively reflect the opinions of the authors. The authors are responsible for all content in their abstracts including accuracy of the facts, statements, citing resources, etc.

Table: EPCORE-FL-1 Efficacy Subgroup Analysis

| | | ORR (95% CI) | CRR (95% CI) | PFS HR (95% CI) for E+R²:R² |
|---|---|---|---|---|
| Age ≥65 | E+R² (n=88) | 94.3 (87.2–98.1) | 80.7 (70.9–88.3) | 0.24 (0.13–0.44) |
| | R² (n=106) | 80.2 (71.3–87.3) | 44.3 (34.7–54.3) | |
| Age <65 | E+R² (n=155) | 95.5 (90.9–98.2) | 83.9 (77.1–89.3) | 0.20 (0.12–0.33) |
| | R² (n=139) | 78.4 (70.6–84.9) | 54.0 (45.3–62.4) | |
| FLIPI 0–2 | E+R² (n=142) | 96.5 (92.0–98.8) | 86.6 (79.9–91.7) | 0.18 (0.10–0.33) |
| | R² (n=132) | 84.8 (77.6–90.5) | 62.1 (53.3–70.4) | |
| FLIPI 3–5 | E+R² (n=100) | 93.0 (86.1–97.1) | 77.0 (67.5–84.8) | 0.25 (0.15–0.42) |
| | R² (n=113) | 72.6 (63.4–80.5) | 35.4 (26.6–45.0) | |
| NHL-5 low | E+R² (n=166) | 94.0 (89.2–97.1) | 81.3 (74.6–86.9) | 0.27 (0.17–0.42) |
| | R² (n=149) | 75.8 (68.2–82.5) | 50.3 (42.0–58.6) | |
| NHL-5 H+I | E+R² (n=77) | 97.4 (90.9–99.7) | 85.7 (75.9–92.6) | 0.14 (0.06–0.29) |
| | R² (n=96) | 84.4 (75.5–91.0) | 49.0 (38.6–59.4) | |
| Non-POD24 | E+R² (n=131) | 96.2 (91.3–98.7) | 85.5 (78.3–91.0) | 0.17 (0.09–0.32) |
| | R² (n=144) | 85.4 (78.6–90.7) | 57.6 (49.1–65.8) | |
| POD24 | E+R² (n=106) | 95.3 (89.3–98.5) | 80.2 (71.3–87.3) | 0.22 (0.13–0.37) |
| | R² (n=93) | 69.9 (59.5–79.0) | 38.7 (28.8–49.4) | |
| 1 prior LOT | E+R² (n=145) | 95.9 (91.2–98.5) | 86.9 (80.3–91.9) | 0.20 (0.12–0.34) |
| | R² (n=141) | 80.1 (72.6–86.4) | 53.2 (44.6–61.6) | |
| ≥2 prior LOT | E+R² (n=98) | 93.9 (87.1–97.7) | 76.5 (66.9–84.5) | 0.24 (0.13–0.42) |
| | R² (n=104) | 77.9 (68.7–85.4) | 45.2 (35.4–55.3) | |
| Not double refractory | E+R² (n=152) | 95.4 (90.7–98.1) | 84.9 (78.2–90.2) | 0.19 (0.11–0.34) |
| | R² (n=154) | 85.1 (78.4–90.3) | 58.4 (50.2–66.3) | |
| Double refractory | E+R² (n=91) | 94.5 (87.6–98.2) | 79.1 (69.3–86.9) | 0.23 (0.13–0.39) |
| | R² (n=91) | 69.2 (58.7–78.5) | 35.2 (25.4–45.9) | |

Subgroups were dichotomized.
CRR, complete response rate; H+I, high and intermediate; HR, hazard ratio; LOT, line of therapy; ORR, overall response rate; PFS, progression-free survival; POD24, progression of disease ≤2 years from the date of initiation of frontline therapy.

Copyright Information: (Online) ISSN: 2572-9241
©2026 The Author(s). HemaSphere is published by John Wiley & Sons Ltd on behalf of European Hematology Association. This is an open access Abstract Book distributed under the Attribution-NonCommercial-NoDerivs (CC BY-NC-ND), which allows third parties to download the articles and share them with others as long as they credit the author and the Abstract Book, but they cannot change the content in any way or use them commercially.
Abstract Book Citations: Authors, Title, HemaSphere, 2026;10:(S1):pages. The abstract book DOIs can be found on the "Author Information" page.
Disclaimer: Articles published in the journal HemaSphere exclusively reflect the opinions of the authors. The authors are responsible for all content in their abstracts including accuracy of the facts, statements, citing resources, etc.

### (S230) CD22-DIRECTED CAR T-CELLS INDUCE HIGH RATES OF COMPLETE REMISSIONS WITH MINIMAL NEUROTOXICITY IN HIGH-RISK FOLLICULAR LYMPHOMA, MANTLE CELL LYMPHOMA, AND OTHER NON-HODGKIN LYMPHOMA SUBTYPES.

**Topic:** 18. Indolent and mantle-cell non-Hodgkin lymphoma - Clinical

Matthew J. Frank*[1], Neha Agarwal[1], Hrishi Srinagesh[1], Anne Marijn Kramer[1], Bita Sahaf[1], Yi-Jiun Su[1], Kendall Kozy[1], Latesha Jackson[1], Annie K. Brown[1], Kimi F. Denoble[1], Sharon Mavroukakis[1], Francisco J. Cortina[1], Kristen Cunanan[1], Sally Arai[1], Laura Johnston[1], Robert Lowsky[1], Everett Meyer[1], Robert Negrin[1], Andrew R. Rezvani[1], Judith Shizuru[1], Lekha Mikkilineni[1], Hany Elmariah[1], Parveen Shiraz[1], Surbhi Sidana[1], Wen-Kai Weng[1], Vanessa Kennedy[1], Lori Muffly[1], Sushma Bharadwaj[1], Melody Smith[1], Saurabh Dahiya[1], Steven Feldman[1], Crystal MacKall[1], David Miklos[1]

*[1]Stanford University, Stanford, United States of America;*

### Background

Patients with Large B-cell Lymphoma (LBCL), Mantle Cell Lymphoma (MCL), and Follicular Lymphoma (FL) who progress after CD19 CAR T cell therapy (CAR19) have poor outcomes.  CD22 is frequently expressed on B-cell lymphomas.  Previously, we treated 38 LBCL patients who progressed after CAR19 with an autologous CD22-targeting CAR T-cell therapy (CAR22) resulting in a 68% overall response rate (ORR) and 53% complete response rate (CRR), a median overall survival (OS) of 25.7 months, without at any grade 3+ CRS, ICANS, or IECHS at a dose of 1 million CAR+/kg.  Here, we report the results of using CAR22 at this dose for the treatment of MCL, FL, and other non-Hodgkin Lymphoma (NHL) subtypes, including Marginal Zone Lymphoma (MZL), Burkitt's Lymphoma (BL), Lymphoplasmacytic Lymphoma (LPL), and LBCL.

### Aims

The primary aims of this single-center Ph1b trial (NCT06340737) are to assess the ability to manufacture CAR22 successfully and to determine the initial safety of the CAR22 in FL, MCL, and other NHL subtypes. Key secondary aims included ORR, CRR, progression-free survival, and OS. Exploratory aims include CAR expansion, MCL MRD monitoring by Clono-Seq, and the impact of tumor CD22 expression on response and relapse.

### Methods

Eligibility criteria required adequate organ function, expression of CD22 on tumor cells by flow or IHC, and 2 lines of prior therapy (except POD24 FL and 1 line for BL was allowed). Prior CAR19 was allowed if <=5% of circulating CD3+ cells expressed CAR19. CAR22 was manufactured via the same process as previously reported (Frank et. al. Lancet 2024) and was infused after standard Flu/Cy.

**Copyright Information:** (Online) ISSN: 2572-9241
©2026 The Author(s). HemaSphere is published by John Wiley & Sons Ltd on behalf of European Hematology Association. This is an open access Abstract Book distributed under the Attribution-NonCommercial-NoDerivs (CC BY-NC-ND), which allows third parties to download the articles and share them with others as long as they credit the author and the Abstract Book, but they cannot change the content in any way or use them commercially.
**Abstract Book Citations:** Authors, Title, HemaSphere, 2026;10:(S1):pages. The abstract book DOIs can be found on the "Author Information" page.
**Disclaimer:** Articles published in the journal HemaSphere exclusively reflect the opinions of the authors. The authors are responsible for all content in their abstracts including accuracy of the facts, statements, citing resources, etc.

**Results**

Between April 2024 and January 2026, 24 patients have been treated (FL: n = 10, MCL: n = 8, MZL=2, BL=1, LPL=1, and LBCL=2). The median age overall is 65 (36-78), and for FL, MCL, and other NHL subtypes was 68 (36-77), 73 (62-78), and 63 (38-66), respectively. 16 (67%) patients were males (by FL, MCL, and other NHL was 60%, 75%, and 50%, respectively). These patients had high-risk features. Patients with FL were POD24 (n=5) or had prior CAR19 (n=3) and received a median of 3 (range 1-8) prior lines of therapy (LOT). Patients with MCL had either TP53 abnormalities or complex karyotype (n=5), or had prior CAR19 (n=7), and received a median of 4 (3-9) LOT. Patients with other NHL subtypes had received a median of 6 (3-15) prior LOT and 3 received a prior CAR19 (BL=1, LBCL=2).
With a median follow-up of 9.9 mo (range 2.6-22.0 mo), the best ORR and CRR were 22 of 24 (92%) and 20 of 24 (83%), respectively. The best ORR and CRR by subtype:FL 100%/100%, MCL 100%/88%, MZL 50%/50%, BL 0%/0%, LPL 100%/0%, and LBCL 100%/100%. MRD became undetectable in 7 of 8 MCL patients.
No grade 3+ CRS, ICANS, or IEC-HS has been observed. Overall rates of Grade (Gr) 1 or 2 CRS, ICANS, and IEC-HS is (n=18) 75% (Gr1:25%, Gr2:50%), (n=2) 8% (Gr 1:8%), (n=8) 33% (Gr1:4%, Gr2: 29%), respectively; CRS, ICANS, and IEC-HS rates by FL, MCL, and other NHL is CRS: 80%, 75%, 66%; ICANS: 10%,13%, 0%; and IEC-HS: 20%, 50%, and 33%, respectively.
7 patients have progressed after initially responding (see figure); 5 had a biopsy after CAR22, 4 had a reduction or loss of tumor CD22 expression (FL: 2 of 2; MCL: 2 of 3).
CAR22 expansion peaked between D+14 and D+21 and was not significantly different between FL, MCL, and other NHL patients (median peak: 432 vs. 38 vs. 178 cells/uL), see figure.

**Summary/Conclusion**

CAR22 induces a high CR rate without grade 3+ CRS, ICANS, or IECHS in FL, MCL, and other NHL subtypes.

**Copyright Information:** (Online) ISSN: 2572-9241
©2026 The Author(s). HemaSphere is published by John Wiley & Sons Ltd on behalf of European Hematology Association. This is an open access Abstract Book distributed under the Attribution-NonCommercial-NoDerivs (CC BY-NC-ND), which allows third parties to download the articles and share them with others as long as they credit the author and the Abstract Book, but they cannot change the content in any way or use them commercially.
**Abstract Book Citations:** Authors, Title, HemaSphere, 2026;10:(S1):pages. The abstract book DOIs can be found on the "Author Information" page.
**Disclaimer:** Articles published in the journal HemaSphere exclusively reflect the opinions of the authors. The authors are responsible for all content in their abstracts including accuracy of the facts, statements, citing resources, etc.

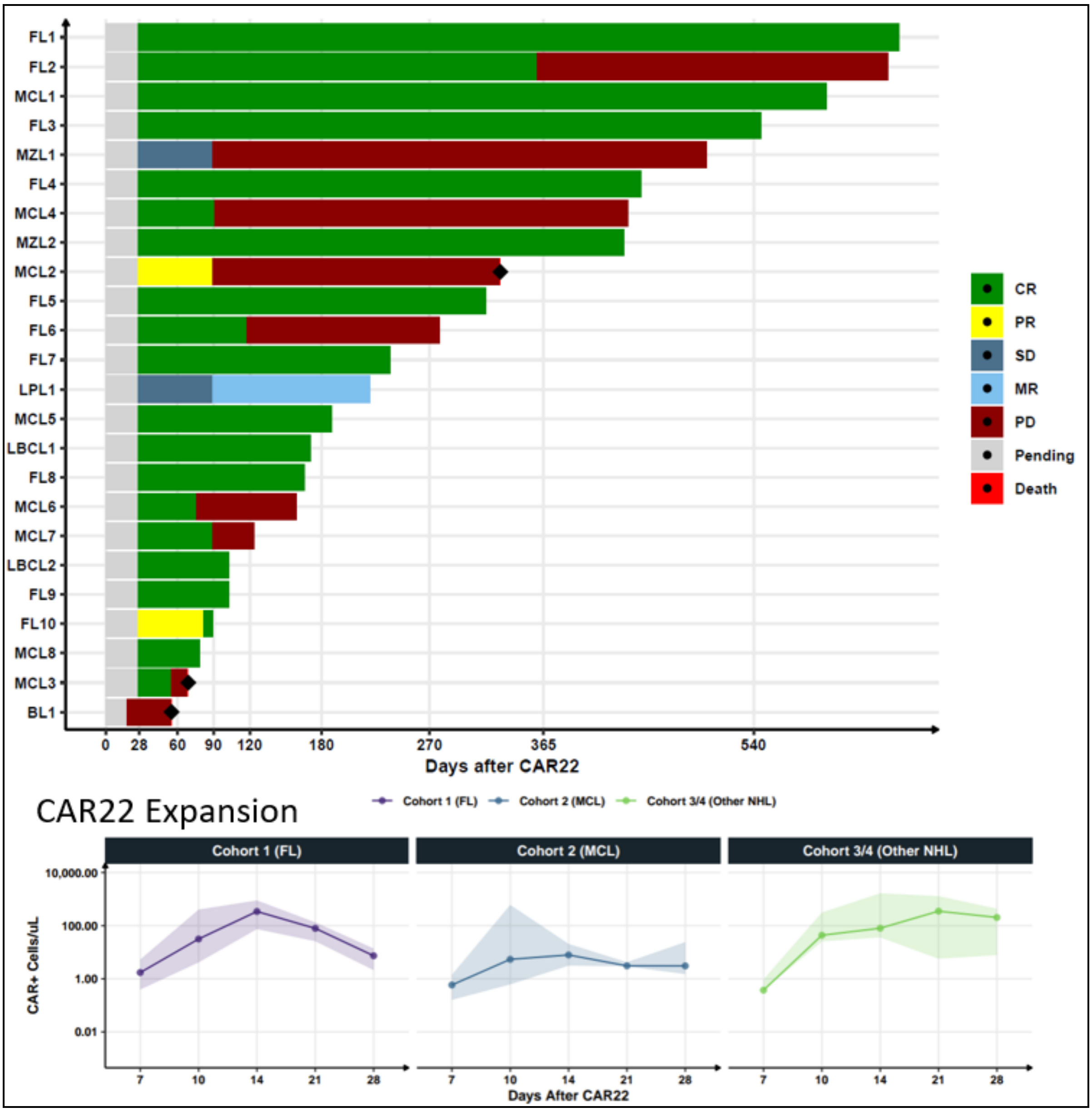

**Copyright Information:** (Online) ISSN: 2572-9241
©2026 The Author(s). HemaSphere is published by John Wiley & Sons Ltd on behalf of European Hematology Association. This is an open access Abstract Book distributed under the Attribution-NonCommercial-NoDerivs (CC BY-NC-ND), which allows third parties to download the articles and share them with others as long as they credit the author and the Abstract Book, but they cannot change the content in any way or use them commercially.
**Abstract Book Citations:** Authors, Title, HemaSphere, 2026;10:(S1):pages. The abstract book DOIs can be found on the "Author Information" page.
**Disclaimer:** Articles published in the journal HemaSphere exclusively reflect the opinions of the authors. The authors are responsible for all content in their abstracts including accuracy of the facts, statements, citing resources, etc.

### (S231) CLINICAL IMPACT OF GERMLINE SINGLE-NUCLEOTIDE POLYMORPHISMS IN MANTLE CELL LYMPHOMA: INSIGHTS FROM THE TRIANGLE TRIAL BY THE MULTIPLY CONSORTIUM

**Topic:** 18. Indolent and mantle-cell non-Hodgkin lymphoma - Clinical

Bashar Al Deeban*[1], Riccardo Moia[1], Katja Gutmair[2], Elisa Genuardi[3], Mouhamad Khouja[4], Jana Nabki[1], Camilla Sasso[3], Rita Tavarozzi[5], Simone Ragaini[3], Amir Karani[1], Philipp Nakov[4], Stephan Stilgenbauer[6], Maria Chiara Tisi[7], Andrea Kerkhoff[8], Luca Arcaini[9], Christian Schmidt[10], Silvia Bea[11], Martin Dreyling[10], Christiane Pott[4], Eva Hoster[2], Gianluca Gaidano[1], Simone Ferrero[3], Marco Ladetto[1, 5]

*[1]Division of Hematology, Department of Translational Medicine, Università del Piemonte Orientale, Novara, Italy, Novara, Italy; [2]Institute for Medical Information Processing, Biometry, and Epidemiology (IBE), LMU University, Munich, Germany, Munich, Germany; [3]Hematology Division, Department of Molecular Biotechnology and Health Sciences, University of Torino,Turin, Italy, Turin, Italy; [4]Second Medical Department, University Hospital Schleswig-Holstein, Kiel, Germany, Kiel, Germany;[5]SCDU Ematologia, Azienda Ospedaliera Santi Antonio e Biagio e Cesare Arrigo, Alessandria, Italy, Alessandria, Italy; [6]Department of Internal Medicine III, Ulm University, Ulm, Germany, Ulm, Germany; [7]Hematology Unit, San Bortolo Hospital, AULSS 8 Berica, Vicenza, Italy, Vicenza, Italy; [8]Department for Medicine A, Hematology, Oncology, Hemostaseology and Pneumology, University Hospital Muenster, 48149 Muenster, Germany, Muenster, Germany; [9]Department of Molecular Medicine, University of Pavia & Division of Hematology, Fondazione IRCCS Policlinico San Matteo, Pavia, Italy, Pavia, Italy; [10]Department of Medicine III, University Hospital, Ludwig-Maximilian-University, Department of Medicine III, Munich, Germany, Munich, Germany; [11]Institut d'Investigacions Biomèdiques August Pi i Sunyer (IDIBAPS), Pathology Department, Hospital Clínic de Barcelona, Barcelona, Spain, Barcelona, Spain;*

### Background

Germline single-nucleotide polymorphisms (SNPs) may influence therapy responses and toxicity in B-cell malignancies and the impact of SNPs on mantle cell lymphoma (MCL) patients treated with ibrutinib-containing regimens remains largely unexplored.

### Aims

The phase 3 TRIANGLE trial, randomizing newly diagnosed fit MCL patients to arm I (IR-CHOP/R-DHAP + ibrutinib maintenance), arm A+I (IR-CHOP/R-DHAP + ASCT + ibrutinib maintenance) or arm A (R-CHOP/R-DHAP + ASCT) with optional rituximab maintenance, was exploited to characterize the clinical impact of germline SNPs in terms of failure-free survival (FFS). This study was funded by Blood Cancer United (LLS No. MCL 7005-24).

### Methods

Genomic DNA from peripheral blood was analyzed by CAPP-Seq targeting 183 SNPs across 74 genes implicated in different pathways, including drug metabolism. SNP associations with FFS were assessed using SNP-wise log-rank tests (α=0.05) with Bonferroni-Holm correction. Additionally, penalized elastic net Cox regression was applied for SNP selection. SNPs identified by either approach were subsequently evaluated in multivariable Cox regression.

**Copyright Information:** (Online) ISSN: 2572-9241
©2026 The Author(s). HemaSphere is published by John Wiley & Sons Ltd on behalf of European Hematology Association. This is an open access Abstract Book distributed under the Attribution-NonCommercial-NoDerivs (CC BY-NC-ND), which allows third parties to download the articles and share them with others as long as they credit the author and the Abstract Book, but they cannot change the content in any way or use them commercially.
**Abstract Book Citations:** Authors, Title, HemaSphere, 2026;10:(S1):pages. The abstract book DOIs can be found on the "Author Information" page.
**Disclaimer:** Articles published in the journal HemaSphere exclusively reflect the opinions of the authors. The authors are responsible for all content in their abstracts including accuracy of the facts, statements, citing resources, etc.

### Results

A total of 515 patients out of the 870 enrolled in TRIANGLE trial were analyzed. This subsample was comparable to the TRIANGLE population for baseline characteristics (median age 57 years [IQR 27-65], low-risk MIPI 59%, blastoid/pleomorphic histology 12% and Ki67 ≥30% 26%), FFS and overall survival (OS). In the combined ibrutinib-containing arms (A+I and I; N=351), no SNP was significant in the log-rank test. Elastic net identified *NAT2*-rs1799930-G/A (prevalence 45%) as associated with prolonged FFS. After adjustment for p53 immunohistochemistry, MIPI, cytology (blastoid/pleomorphic vs. classical/small cell) and Ki-67, the *NAT2* SNP remained independently associated with improved FFS (HR 0.55, 95% CI 0.35-0.86, p=0.009) (Fig. 1A). Carriers of the *NAT2* SNP had a 4-year FFS and OS of 90.2% and 95.2% compared to 76.5% and 86.5% for other patients (p=0.002) (Fig. 1B,C). In Arm A, the *TP53*-rs1625895-T/C SNP (prevalence 94%) associated with longer FFS in the log-rank test. Elastic net additionally selected *ABCC2*-rs1885301-A/G (prevalence 82%), *ABCC2*-rs3740066-C/T (prevalence 57%), *ABCC1*-rs35587-T/C (prevalence 57%), *ABCG2*-rs2622604-T/C (prevalence 96%), and *TP53*-rs1625895-T/C (prevalence 94%). After multivariable adjustment (MIPI, Ki-67, cytology, p53), the *ABCC1*-rs35587-T/C SNP retained independent prognostic value for improved FFS (HR 0.41, 95% CI 0.22-0.78; p=0.006) (Fig. 1D). Carriers of the *ABCC1*-rs35587-T/C SNP had a 4-year FFS and OS of 78.2% and 86.6% compared to 61.4% and 77.0% for other patients (p=0.003) (Fig. 1E,F). The *ABCC2*-rs3740066-C/T SNP showed a trend toward shorter FFS, whereas the *ABCC2*-rs1885301-A/G, *ABCG2*-rs2622604-T/C, and *TP53*-rs1625895-T/C SNPs showed trends toward longer FFS.

### Summary/Conclusion

This study represents the largest analysis to date evaluating the clinical impact of multiple SNPs in MCL, particularly in the context of first-line ibrutinib-containing therapy. *NAT2* encodes an enzyme involved in the ibrutinib acetylation, and the rs1799930 G/A SNP reduces *NAT2* enzymatic activity, potentially increasing ibrutinib exposure and explaining the improved FFS observed exclusively in ibrutinib-treated patients. Furthermore, ibrutinib appears to overcome the impact of the *ABCC1* rs35587 T/C SNP, which is involved in the metabolism of several chemotherapeutic agents, including platinum compounds, doxorubicin, and vincristine.

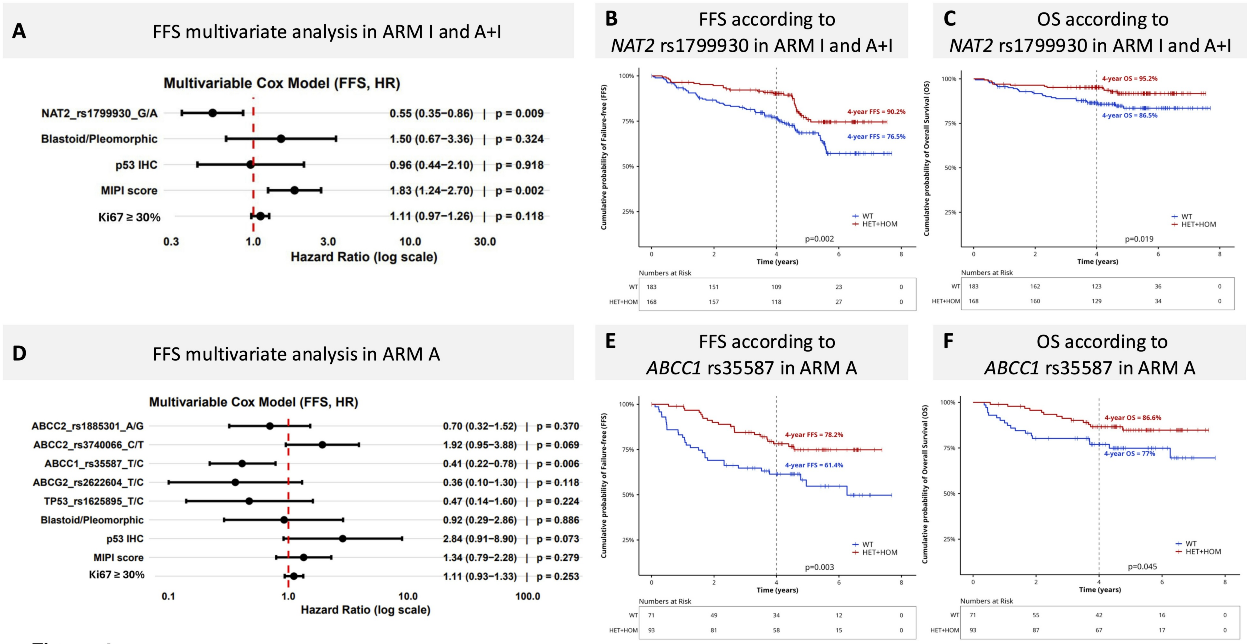

**Figure 1**

**Copyright Information:** (Online) ISSN: 2572-9241
©2026 The Author(s). HemaSphere is published by John Wiley & Sons Ltd on behalf of European Hematology Association. This is an open access Abstract Book distributed under the Attribution-NonCommercial-NoDerivs (CC BY-NC-ND), which allows third parties to download the articles and share them with others as long as they credit the author and the Abstract Book, but they cannot change the content in any way or use them commercially.
**Abstract Book Citations:** Authors, Title, HemaSphere, 2026;10:(S1):pages. The abstract book DOIs can be found on the "Author Information" page.
**Disclaimer:** Articles published in the journal HemaSphere exclusively reflect the opinions of the authors. The authors are responsible for all content in their abstracts including accuracy of the facts, statements, citing resources, etc.

### (S232) FIXED-DURATION GLOFITAMAB MONOTHERAPY IN RELAPSED OR REFRACTORY MANTLE CELL LYMPHOMA WITH OR WITHOUT PRIOR BRUTON'S TYROSINE KINASE INHIBITOR EXPOSURE: UPDATED OUTCOMES WITH 3.5 YEARS OF FOLLOW-UP

**Topic:** 18. Indolent and mantle-cell non-Hodgkin lymphoma - Clinical

Yasmin Karimi*[1], Martin Hutchings[2], Tycel Phillips[3], Marek Trněný[4], Michael Dickinson[5], Fritz C. Offner[6], Franck Morschhauser[7], Emmanuel Bachy[8], Nancy Bartlett[9], Joshua Brody[10], Jan Zaucha[11], Tomasz Wrobel[12], Lidia Gil[13], Vanessa Breton[14], Raluca Negricea[15], Martine Joanna Kallemeijn[16], Aurelien Berthier[16], Gila Sellam[16], Linda Lundberg[16], Carmelo Carlo-Stella[17]

*[1]Division of Hematology/Oncology, University of Michigan, Ann Arbor, MI, United States of America;[2]Rigshospitalet and University of Copenhagen, Copenhagen, Denmark; [3]City of Hope National Medical Center, Duarte, CA, United States of America; [4]First Faculty of Medicine, Charles University and the General Hospital, Prague, Czechia;[5]Peter MacCallum Cancer Centre, Royal Melbourne Hospital and The University of Melbourne, Melbourne, Australia; [6]Department of Hematology Universitair Ziekenhuis, Gent, Belgium; [7]CHU Lille, Service des Maladies du Sang, Lille, France;[8]Centre Hospitalier Lyon Sud, Lyon, France; [9]Siteman Cancer Center, Washington University School of Medicine, St. Louis, MO, United States of America;[10]Tisch Cancer Institute, New York, NY, United States of America; [11]Medical University of Gdańsk, Gdańsk, Poland; [12]Wroclaw Medical University, Wroclaw, Poland; [13]Department of Hematology and Hematopoietic Cell Transplantation, Medical University, Poznan, Poland; [14]Hoffmann-La Roche Ltd, Mississauga, ON, Canada; [15]Roche Products Ltd, Welwyn Garden City, United Kingdom; [16]F. Hoffmann-La Roche Ltd, Basel, Switzerland; [17]Humanitas University and IRCCS Humanitas Research Hospital, Milan, Italy;*

### Background

Patients with advanced relapsed or refractory (R/R) mantle cell lymphoma (MCL) have poor outcomes, especially patients with high-risk features or those progressing after Bruton's tyrosine kinase inhibitor-based (BTKi) therapies. Glofitamab is a CD20xCD3 2:1 bispecific antibody that redirects T cells to eliminate malignant B cells. A Phase 1/2 trial (NCT03075696) evaluated glofitamab monotherapy, given as an investigational regimen with step-up dosing after obinutuzumab pretreatment (Gpt), in patients with R/R MCL. Prior analyses showed high response rates and manageable safety among these patients, regardless of prior BTKi exposure (Phillips, et al. ASCO 2024).

### Aims

Here, we report longer follow-up data from this MCL cohort of the Phase 1/2 trial.

### Methods

Patients with R/R MCL after ≥1 prior line of systemic therapy received Gpt (single 1000mg dose or 2000mg split over 2 days, as needed) on cycle (C)1 day (D)1. Glofitamab step-up-dosing was given on C1D8 (2.5mg) and C1D15 (10mg), followed by the target dose of 16 or 30mg every 3 weeks on D1 of C2–12. Efficacy endpoints included investigator-assessed complete response (CR) rate, overall response rate (ORR), duration of CR (DoCR), duration of response (DoR), progression free survival (PFS), and overall survival (OS). All patients provided informed consent.

**Copyright Information:** (Online) ISSN: 2572-9241
©2026 The Author(s). HemaSphere is published by John Wiley & Sons Ltd on behalf of European Hematology Association. This is an open access Abstract Book distributed under the Attribution-NonCommercial-NoDerivs (CC BY-NC-ND), which allows third parties to download the articles and share them with others as long as they credit the author and the Abstract Book, but they cannot change the content in any way or use them commercially.
**Abstract Book Citations:** Authors, Title, HemaSphere, 2026;10:(S1):pages. The abstract book DOIs can be found on the “Author Information” page.
**Disclaimer:** Articles published in the journal HemaSphere exclusively reflect the opinions of the authors. The authors are responsible for all content in their abstracts including accuracy of the facts, statements, citing resources, etc.

**Results**

A total of 61 patients with R/R MCL were enrolled (Gpt: 1000mg, n=17; 2000mg, n=44); 60 patients received treatment. The median number of prior lines of therapy was 2 (range: 1–5), median age was 72.0 years (range: 41–86), 86.9% of patients had Ann Arbor stage III/IV, and 26.3% had a simplified MCL International Prognostic Index score of ≥6. Patients had high-risk disease features such as Ki-67 proliferation index ≥30% (62.3%), blastoid/pleomorphic variants (9.8%), and *TP53* mutation (19.7%). The median number of glofitamab cycles received was 12 (range: 1–13). At the data cut-off of September 8, 2025, the median OS follow-up was 41.5 months (95% confidence interval [CI]: 37.4–48.7), and the ORR and CR rate were 82% and 77%, respectively. The median DoCR was 40.8 months (95% CI: 14.1–not estimable [NE]), and 48.9% of patients had ongoing CRs at the data cut-off. Estimated 33-month DoCR and DoR rates were 50.5% and 47.4%, respectively. Median (95% CI) PFS was 18 months (11.3–42.8) and OS was not reached (26.9–NE).
For patients who received prior BTKi therapy (n=34 [55.7%]), the ORR and CR rate were 73.5% and 70.6%, respectively. Median (95% CI) DoCR was 15.4 months (8.3–NE), PFS was 11.3 months (5.1–32.6), and OS was 29.9 months (11.3–NE). Estimated 33-month DoCR and DoR rates were 42.7% and 41.0%, respectively.
No new safety signals were observed. Cytokine release syndrome remained the most common adverse event (all Grade, n=42/60, 70%; Grade 1–2, 58.3%; Grade 3–4, 11.6%; no Grade 5 events), with lower rates reported in the 2000mg Gpt cohort (n=28/44, 63.6%) compared with the 1000mg Gpt cohort (n=14/16, 87.5%).

**Summary/Conclusion**

Updated data on fixed-duration glofitamab monotherapy in heavily pretreated patients with R/R MCL demonstrate robust efficacy and manageable safety, including in BTKi-exposed patients. Glofitamab monotherapy is a suitable treatment for patients with R/R MCL in need of rapid disease control, and is under investigation in the Phase 3 GLOBRYTE trial.

© 2026 American Society of Clinical Oncology, Inc. Reused with permission. This abstract was accepted and previously presented at the 2026 ASCO Annual Meeting. All rights reserved.

**Copyright Information:** (Online) ISSN: 2572-9241
©2026 The Author(s). HemaSphere is published by John Wiley & Sons Ltd on behalf of European Hematology Association. This is an open access Abstract Book distributed under the Attribution-NonCommercial-NoDerivs (CC BY-NC-ND), which allows third parties to download the articles and share them with others as long as they credit the author and the Abstract Book, but they cannot change the content in any way or use them commercially.
**Abstract Book Citations:** Authors, Title, HemaSphere, 2026;10:(S1):pages. The abstract book DOIs can be found on the “Author Information” page.
**Disclaimer:** Articles published in the journal HemaSphere exclusively reflect the opinions of the authors. The authors are responsible for all content in their abstracts including accuracy of the facts, statements, citing resources, etc.

### (S233) GENOMIC PREDICTORS OF OUTCOME IN MANTLE CELL LYMPHOMA: TP53 AND ADDITIONAL ALTERATIONS IN THE TRIANGLE TRIAL BY THE MULTIPLY CONSORTIUM

**Topic:** 21. Lymphoma biology & translational research

Marta Grau*[1, 2], Ferran Nadeu[1, 2], Anna Montaner[1], Guillem Clot[1, 2, 3], Melika Bashiri[1], Ian Márquez-López[1, 2], Gerard Frigola[1, 4], Cristina Lopez[1, 2, 3, 4], Sarah Reinke[5], Wolfram Klapper[5], Sara Ek[6], Sofia Klint[6], Vittorio Stefoni[7], Valentina Tabanelli[8], Simone Ragaini[9], Katja Gutmair[10], Eva Hoster[10], Simone Ferrero[9], Elias Campo[1, 2, 3, 4], Eva Gine[1, 2, 3, 11], Marco Ladetto[12], Martin Dreyling[13], Silvia Bea[1, 2, 3, 4]

*[1]Fundació de Recerca Clínic Barcelona-Institut d'Investigacions Biomèdiques August Pi i Sunyer (FRCB-IDIBAPS), Barcelona, Spain; [2]Centro de Investigación Biomédica en Red de Cáncer (CIBERONC), Madrid, Spain;[3]Universitat de Barcelona, Barcelona, Spain; [4]Hematopathology Unit, Department of Pathology, Hospital Clínic de Barcelona, Barcelona, Spain;[5]University Hospital Schleswig-Holstein, Department of Pathology, Hematopathology Section, Kiel, Germany; [6]Department of Immunotechnology, Lund University, Lund, Sweden; [7]Institute of Hematology "L. e A. Seràgnoli" University of Bologna, Bologna, Italy; [8]IRCCS Istituto Europeo di Oncologia, Ematopatologia, Milan, Italy; [9]Department of Molecular Biotechnologies and Health Sciences, University of Torino, Torino, Italy; [10]Institute for Medical Information Processing, Biometry, and Epidemiology, Ludwig Maximilian University of Munich, Munich, Germany; [11]Hematology Department, Hospital Clínic de Barcelona, Barcelona, Spain; [12]Dipartimento di Medicina Traslazionale, Università del Piemonte Orientale and SCDU Ematologia AOU Santi Antonio e Biagio e Cesare Arrigo Alessandria, Alessandria, Italy; [13]Department of Medicine III, LMU University Hospital, Munich, Germany;*

### Background

The randomized TRIANGLE trial established an Ibrutinib-containing, ASCT-free therapy (arm I) as the new standard of care for younger, previously untreated patients with mantle cell lymphoma (MCL). However, the impact of genetic alterations on outcomes in MCL patients receiving first-line Ibrutinib is still unexplored.

### Aims

The TRIANGLE trial provides an optimal framework to investigate the prognostic and predictive relevance of genetic alterations.

### Methods

Mutations and gains/losses were analyzed on available tumor DNA from TRIANGLE using a custom targeted sequencing panel. Samples were categorized by arms: A (standard treatment with ASCT, n=105), A+I (standard ASCT treatment with Ibrutinib, n=107) and I (n=117). SOX11, Ki67, and p53 expression were evaluated by immunohistochemistry (IHC). The impact of genetic alterations was assessed using log-rank tests, arm-specific Cox proportional hazards models and a likelihood ratio test to evaluate the statistical interaction between *TP53* status and treatment arm.

**Copyright Information:** (Online) ISSN: 2572-9241
©2026 The Author(s). HemaSphere is published by John Wiley & Sons Ltd on behalf of European Hematology Association. This is an open access Abstract Book distributed under the Attribution-NonCommercial-NoDerivs (CC BY-NC-ND), which allows third parties to download the articles and share them with others as long as they credit the author and the Abstract Book, but they cannot change the content in any way or use them commercially.
**Abstract Book Citations:** Authors, Title, HemaSphere, 2026;10:(S1):pages. The abstract book DOIs can be found on the "Author Information" page.
**Disclaimer:** Articles published in the journal HemaSphere exclusively reflect the opinions of the authors. The authors are responsible for all content in their abstracts including accuracy of the facts, statements, citing resources, etc.

**Results**

A total of 329 patients from the 870 enrolled in the TRIANGLE trial were analyzed. Baseline characteristics were: median age 57 years, 11% high-risk MIPI, 17% blastoid/pleomorphic morphology, 35% Ki67≥30%, and 12% p53 overexpressed (>50%). Based on the L-MCL16 (NanoString) signature and/or SOX11 IHC, 86% tumors were classified as cMCL, 9% as nnMCL, and 5% remained unclassified. Mutations were identified in 205/329 (62%) patients. The most frequent alterations included mutations of *TP53* (22%), *NSD2* (11%), *SMARCA4* (11%), *NOTCH1* (11%); 8q24(*MYC)* (13%) gains/amplifications, and losses at 9p21(*CDKN2A/B)* (15%), 17p13(*TP53)* (9%), and 13q14(*RB1)* (8%). All cases with *TP53* loss also harbored *TP53* mutation. No remarkable differences in clinicopathological variables or the mentioned genetic alterations were observed across treatment arms. Notably, 89% (32/36) of tumors with p53 overexpression carried *TP53* mutations, although only 52% (32/61) of *TP53*-mutated tumors showed p53 overexpression. In the whole cohort, *TP53*-mutated tumors (n=72) exhibit an inferior FFS (**Fig. 1A**). When considering the three treatment arms, patients with *TP53*-mutated MCL exhibited shorter FFS, more prominently in the non-Ibrutinib arm (A) (HR 5.3, 95% CI 2.6-10.9) (**Fig. 1B**). Of note, the addition of Ibrutinib seemed to improve the outcome for *TP53*-mutated patients, showing a different effect of *TP53* across the three treatment groups ($P$=0.028). Interestingly, an inferior OS was observed in patients with *TP53*-mutated tumors which had not received Ibrutinib, with a median OS of 3.7 years in arm A, while it has not been reached in A+I and I arms. Other common genetic alterations associated with shorter OS in arm A were *CDKN2A/B* deletions (HR 2.6, 95% CI 1.05-6.6), *NOTCH1/2* mutations (HR 3.1, 95% CI 1.1-8.5), and *MYC* gains (HR 3.6, 95% CI 1.2-10.7).

**Summary/Conclusion**

Our preliminary analysis of the TRIANGLE trial shows that *TP53* mutations are strong predictors of inferior FFS, outperforming p53 IHC, which identified only half (52%) of the *TP53*-mutated cases. The addition of Ibrutinib partially abrogated the poor outcome of *TP53*-mutated patients. In addition, Ibrutinib seems to reduce the adverse prognostic impact of other high-risk genetic lesions, including *CDKN2A/B* deletions, *NOTCH1/2* mutations and *MYC* gains. Genetic profiling of additional patients from the TRIANGLE trial is underway and may provide robust evidence to support routine *TP53* mutational testing in all MCL patients prior to treatment selection.

**Copyright Information:** (Online) ISSN: 2572-9241
©2026 The Author(s). HemaSphere is published by John Wiley & Sons Ltd on behalf of European Hematology Association. This is an open access Abstract Book distributed under the Attribution-NonCommercial-NoDerivs (CC BY-NC-ND), which allows third parties to download the articles and share them with others as long as they credit the author and the Abstract Book, but they cannot change the content in any way or use them commercially.
**Abstract Book Citations:** Authors, Title, HemaSphere, 2026;10:(S1):pages. The abstract book DOIs can be found on the "Author Information" page.
**Disclaimer:** Articles published in the journal HemaSphere exclusively reflect the opinions of the authors. The authors are responsible for all content in their abstracts including accuracy of the facts, statements, citing resources, etc.

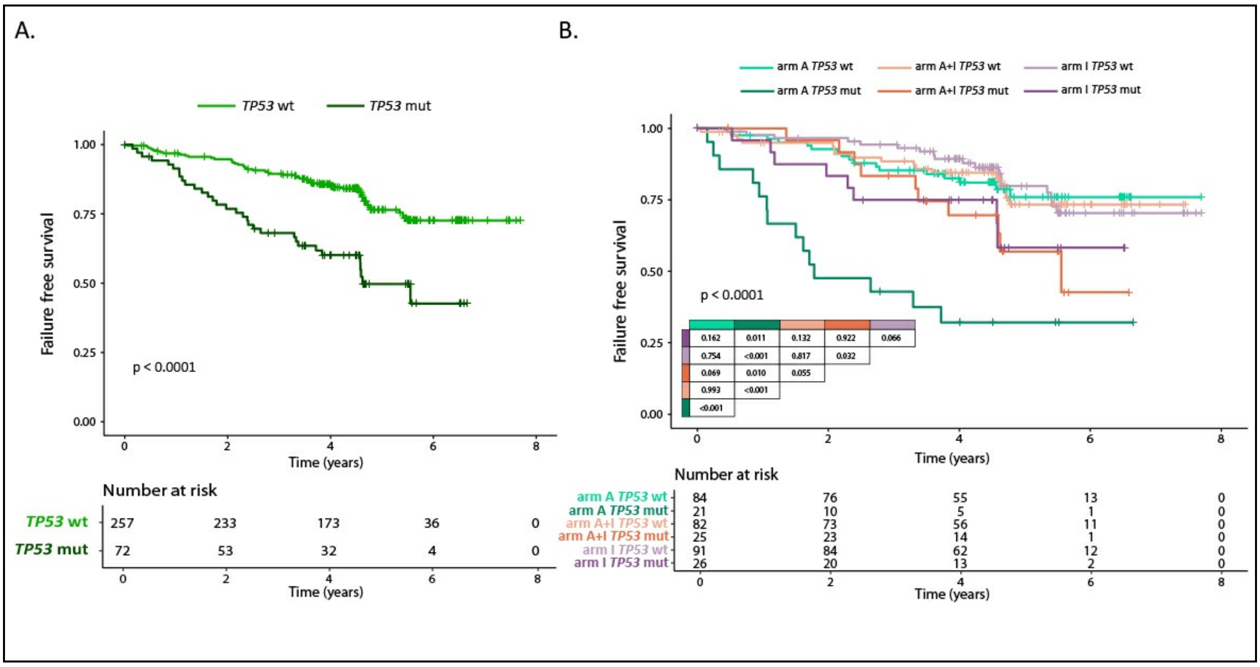

Failure-free survival (FFS) curves from MCL TRIANGLE patients stratified by TP53 mutational status are shown for the whole cohort (A) and separately within each of the three treatment arms (B). All pairwise comparisons are displayed within the plot area. P values were calculated using the log-rank test.

**Copyright Information:** (Online) ISSN: 2572-9241
©2026 The Author(s). HemaSphere is published by John Wiley & Sons Ltd on behalf of European Hematology Association. This is an open access Abstract Book distributed under the Attribution-NonCommercial-NoDerivs (CC BY-NC-ND), which allows third parties to download the articles and share them with others as long as they credit the author and the Abstract Book, but they cannot change the content in any way or use them commercially.
**Abstract Book Citations:** Authors, Title, HemaSphere, 2026;10:(S1):pages. The abstract book DOIs can be found on the "Author Information" page.
**Disclaimer:** Articles published in the journal HemaSphere exclusively reflect the opinions of the authors. The authors are responsible for all content in their abstracts including accuracy of the facts, statements, citing resources, etc.

### (S234) TUMOR CELL EXPRESSION OF SOX11 AND CYCLIN D1 DIFFERENTLY IMPACT THE SPATIAL TUMOR IMMUNE CELL LANDSCAPE IN MANTLE CELL LYMPHOMA- A TRANSLATIONAL NORDIC MCL2/3 STUDY

**Topic:** 21. Lymphoma biology & translational research

Colin Lamont[1, 2], Daniel Nilsson[3, 4], Kanutteh Huse[1, 2, 5], Ivana Spasevska[1, 2, 5], Lina Olsson[3, 4], Arne Kolstad[6], Anna Porwit[7], Mats Jerkeman[8], June Myklebust*[1, 2, 5], Sara Ek[3, 4, 9]

*[1]Oslo University Hospital, Department of Cancer Immunology, Institute for Cancer Research, Oslo, Norway;[2]University of Oslo, KG Jebsen Centre for B-cell Malignancies, Institute of Clinical Medicine, Oslo, Norway; [3]Lund University, Department of Immunotechnology, Lund, Sweden; [4]Lund University, CREATE Health translational cancer center, Lund, Sweden;[5]University of Oslo, Precision Immunotherapy Alliance, Oslo, Norway; [6]Innlandet Hospital Trust, Division Gjøvik-Lillehammer, Department of Oncology, Gjøvik/Lillehammer, Norway; [7]Lund University, Division of Oncology and Pathology, Department of Clinical Sciences, Lund, Sweden; [8]Lund University Hospital, Department of Oncology, Lund, Sweden;[9]Lund University, Science for Life Laboratory, Lund, Sweden;*

### Background

Mantle cell lymphoma (MCL) is a heterogeneous B-cell lymphoma with frequent relapses. Intensified first-line chemoimmunotherapy with cytarabine followed by autologous stem cell transplantation (ASCT) improves outcomes in younger patients, yet high-risk disease (high Ki67/MIPI-B and TP53 aberrations) remains difficult to control. The tumor microenvironment (TME) contributes to this heterogeneity as immune-depleted, macrophage-rich MCL have been linked to adverse prognosis and resistance. However, the spatial organization of TME and associated transcriptional programs in clinically annotated cohorts are incompletely defined.

### Aims

To integrate spatial proteomics with compartment-resolved transcriptomics to map TME architecture in MCL and relate it to tumor biology and outcome.

### Methods

We included TMA sections containing 135 diagnostic tissue cores from 98 patients treated on Nordic Lymphoma Group trials MCL2/3 with long term follow-up clinical data. Imaging mass cytometry (IMC, Hyperion) used a 31-marker panel covering tumor, T-cell, myeloid, endothelial and checkpoint proteins. GeoMx™ DSP profiled 1,811 transcripts (Cancer Transcriptomic Atlas) in 190 ROIs (~2 ROIs/patient), segmented into CD20+ tumor, CD8+ cytotoxic T cells, and CD3+/CD8− helper T cells.

### Results

Fourteen distinct cell types/subsets were identifed in IMC data and unsupervised analyses of these cell type frequencies identified three recurrent TME clusters: tumor-rich, M2-like macrophage-rich, and T-cell-rich. Patients with M2-rich TME had inferior progression-free survival and had tumor cells with lower SOX11 and higher Ki67 and cyclin D1 protein expression. Transcriptomics analysis of tumor cell segments revealed that SOX11-low tumors (enriched in the M2-rich cluster) had upregulated proliferation and cell cycle programs (e.g., Cyclin D1/Ki67 signatures), in contrast to SOX11+ tumor cells characterized by B-cell differentiation/activation program and immune-regulatory genes. Exhaustion-associated markers (*LAG3*, *TIGIT*) were detected in T cells within M2-rich regions, yet accompanied by higher activation marker expression relative to the other clusters. Canonical correlation analysis highlighted the inverse relationship between cyclin D1 and SOX11, and SOX11 as the strongest tumor cell feature linked to neighborhood composition: SOX11 expression correlated positively with dendritic cells and PD-1+ CD8 T cells and negatively with PD-1− CD4 and CD8 T cells and macrophage abundance.

**Copyright Information:** (Online) ISSN: 2572-9241
©2026 The Author(s). HemaSphere is published by John Wiley & Sons Ltd on behalf of European Hematology Association. This is an open access Abstract Book distributed under the Attribution-NonCommercial-NoDerivs (CC BY-NC-ND), which allows third parties to download the articles and share them with others as long as they credit the author and the Abstract Book, but they cannot change the content in any way or use them commercially.
**Abstract Book Citations:** Authors, Title, HemaSphere, 2026;10:(S1):pages. The abstract book DOIs can be found on the "Author Information" page.
**Disclaimer:** Articles published in the journal HemaSphere exclusively reflect the opinions of the authors. The authors are responsible for all content in their abstracts including accuracy of the facts, statements, citing resources, etc.

**Summary/Conclusion**

MCL exhibits distinct spatial TIME architectures with prognostic relevance. A macrophage-dominant, SOX11-low state couples increased tumor proliferation with altered T-cell phenotypes and inferior outcome. SOX11 emerges as a central axis, connecting tumor-intrinsic programs to immune neighborhood structure, supporting its use for risk stratification and for guiding microenvironment-targeted strategies (e.g., macrophage reprogramming and checkpoint/exhaustion-pathway combinations) in high-risk MCL.

First two and last two authorships respectively are shared.

**Copyright Information:** (Online) ISSN: 2572-9241
©2026 The Author(s). HemaSphere is published by John Wiley & Sons Ltd on behalf of European Hematology Association. This is an open access Abstract Book distributed under the Attribution-NonCommercial-NoDerivs (CC BY-NC-ND), which allows third parties to download the articles and share them with others as long as they credit the author and the Abstract Book, but they cannot change the content in any way or use them commercially.
**Abstract Book Citations:** Authors, Title, HemaSphere, 2026;10:(S1):pages. The abstract book DOIs can be found on the “Author Information” page.
**Disclaimer:** Articles published in the journal HemaSphere exclusively reflect the opinions of the authors. The authors are responsible for all content in their abstracts including accuracy of the facts, statements, citing resources, etc.

### (S235) RESULTS FROM EPCORE DLBCL-1: RANDOMIZED PHASE 3 STUDY OF EPCORITAMAB (EPCOR) VS INVESTIGATOR'S CHOICE CHEMOIMMUNOTHERAPY (CIT) IN PATIENTS WITH RELAPSED/REFRACTORY LARGE B-CELL LYMPHOMA (R/R LBCL)

**Topic:** 19. Large B cell lymphomas - Clinical

Christopher P. Fox*[1, 2], Luca Inchiappa[3], Burhan Ferhanoglu[4], Sherida Woei-A-Jin[5], Takayuki Ikezoe[6], Tai-Chung Huang[7], Juan Miguel Bergua Burgues[8], Jae-Yong Kwak[9], Alexander Fosså[10], Alessandra Tucci[11], Esther Hian Li Chan[12, 13], Tamas Masszi[14], Michael Roost Clausen[15], Martin Hutchings[16], Pieternella Lugtenburg[17], Gilles Salles[18], Pier Luigi Zinzani[19, 20], Mihaela Popa McKiver[21], Liwei Wang[22], Avani Badani[22], Chris Zhang[22], Signe Diness Vindeløv[23], Wojciech Jurczak[24]

*[1]Nottingham University Hospitals NHS Trust, Nottingham, United Kingdom;[2]School of Medicine, University of Nottingham, Nottingham, United Kingdom; [3]Service d'Onco-hématologie, Centre Antoine Lacassagne, Nice, France; [4]Division of Hematology, Department of Internal Medicine, School of Medicine, Koç University, Istanbul, Türkiye; [5]Department of General Medical Oncology, University Hospitals Leuven, Leuven, Belgium; [6]Department of Hematology, Fukushima Medical University, Fukushima, Japan; [7]Division of Hematology, Department of Internal Medicine, National Taiwan University Hospital, Taipei, Taiwan; [8]Servicio De Hematologia Y Hemoterapia, Hospital San Pedro de Alcántara, Cáceres, Spain;[9]Department of Internal Medicine, Jeonbuk Stem Cell Transplantation Program, Jeonbuk National University Medical School & Hospital, Jeonju, South Korea; [10]Department of Oncology, Oslo University Hospital, Oslo, Norway; [11]ASST Spedali Civili-Ematologia, Brescia, Italy; [12]NUS Centre for Cancer Research (N2CR), National University of Singapore, Singapore, Singapore;[13]Department of Haematology-Oncology, National University Health System, Singapore, Singapore; [14]Department of Internal Medicine and Hematology, Semmelweis University, Budapest, Hungary; [15]Vejle Hospital, Vejle, Denmark; [16]Rigshospitalet and University of Copenhagen, Copenhagen, Denmark; [17]Department of Hematology, Erasmus MC Cancer Institute, University Medical Center Rotterdam, Rotterdam, The Netherlands; [18]Lymphoma Service, Memorial Sloan Kettering Cancer Center, New York, NY, United States of America; [19]Istituto Di Ematologia, Universitaria di Bologna, Bologna, Italy;[20]Institute of Hematology "Seràgnoli," University of Bologna, Bologna, Italy; [21]AbbVie, North Chicago, IL, United States of America;[22]Genmab, Plainsboro, NJ, United States of America; [23]Genmab, Copenhagen, Denmark; [24]Maria Skłodowska-Curie National Research Institute of Oncology, Kraków, Poland;*

### Background

Epcor is a CD3×CD20 bispecific antibody (bsAb) approved as monotherapy for R/R DLBCL after ≥ 2 prior lines of systemic treatment (pLOT). The phase 3 EPCORE DLBCL-1 trial (EudraCT No. 2020-003016-27) evaluated epcor monotherapy vs investigator's choice CIT (rituximab + gemcitabine/oxaliplatin [R-GemOx] or bendamustine + rituximab [BR]) in R/R LBCL.

### Aims

To report primary results from EPCORE DLBCL-1.

### Methods

Eligible patients (pts) had CD20+ R/R LBCL, were ASCT-ineligible or relapsed after ASCT, and had ≥ 1 systemic pLOT (1 pLOT capped at 25%). Pts were randomized 1:1 to epcor monotherapy or CIT. Epcor was given in 28-d cycles (2 step-up doses then 48-mg full doses QW in C1–3, Q2W in C4–9, Q4W in C10+ until disease progression or unacceptable toxicity). R-GemOx was given as standard Q2W for four 28-d cycles and BR Q3W for six 21-d cycles. Dual primary endpoints were PFS (Lugano criteria; IRC) and OS.

**Copyright Information:** (Online) ISSN: 2572-9241
©2026 The Author(s). HemaSphere is published by John Wiley & Sons Ltd on behalf of European Hematology Association. This is an open access Abstract Book distributed under the Attribution-NonCommercial-NoDerivs (CC BY-NC-ND), which allows third parties to download the articles and share them with others as long as they credit the author and the Abstract Book, but they cannot change the content in any way or use them commercially.
**Abstract Book Citations:** Authors, Title, HemaSphere, 2026;10:(S1):pages. The abstract book DOIs can be found on the "Author Information" page.
**Disclaimer:** Articles published in the journal HemaSphere exclusively reflect the opinions of the authors. The authors are responsible for all content in their abstracts including accuracy of the facts, statements, citing resources, etc.

**Results**

483 pts (epcor 241; CIT 242 [R-GemOx 174; BR 68]) were randomized; 67% were randomized in 2022 or later, coinciding with the COVID-19 Omicron period. Median follow-up was 43.2 mo (epcor) and 42.3 mo (CIT). Median age was 71 y (range, 28–89); 53% of pts were from North America/Western Europe; 73% had ≥ 2 pLOT, and 34% had ≥ 3 pLOT; 69% were refractory to last line of treatment (tx); 15% had prior SCT, and 11% had prior CAR-T.
A statistically significant improvement in PFS was demonstrated with epcor vs CIT (HR, 0.74 [95% CI, 0.60–0.92]; $P$=0.0059; 24-mo PFS: 30% vs 13%). Furthermore, clinically meaningful higher complete response rate (CRR: 38% vs 26%; nominal $P$ value 0.0032), longer durability of response (median DOR 37 vs 6 mo; DOCR NR vs 11 mo) and longer TTNT (7 vs 4 mo; nominal $P$ value <0.0001) were observed for epcor vs CIT. ORR was comparable (51% vs 48%). OS was not significantly different (HR, 0.96 [95% CI, 0.77–1.20]; 24-mo OS: 38% vs 33%). Notably, subsequent tx with bsAb, CAR-T, or SCT was received by 26% in the CIT arm vs 6% in the epcor arm. After post-hoc adjustments for these subsequent tx and COVID-19–related deaths, the HR for OS favored epcor (adjusted HR, 0.76 [95% CI, 0.59–0.99]). In pts with 1 pLOT, trends favoring epcor were observed (PFS HR, 0.67 [95% CI, 0.44–1.04], OS HR, 0.80 [95% CI, 0.51–1.25], CRR: 45% vs 38%).
Mean tx duration was longer for epcor vs CIT (11 vs 2 mo); 31% of pts received ≥ 12 cycles of epcor. Higher rates of grade 3–4 infections (30% vs 12%) and any-grade COVID-19 (36% vs 11%) were reported in the epcor arm; exposure-adjusted event rates were 4.3 vs 4.9 (grade 3–4 infections) and 4.4 vs 3.4 (any-grade COVID-19) per 100 pt-mo. Grade 5 TEAEs occurred in 17% vs 6% (exposure-adjusted, 1.5 vs 1.8 per 100 pt-mo) and were largely attributable to grade 5 COVID-19 (9% vs 2%). CRS was reported in 53% (grade 3, 3%) in the epcor arm and ICANS in 4% (grade 3–4, 1%).

**Summary/Conclusion**

EPCORE DLBCL-1 is the largest randomized phase 3 trial in R/R LBCL and the first to demonstrate a statistically significant improvement in PFS with a CD3×CD20 bsAb monotherapy vs combination CIT. Furthermore, compared with CIT, epcor resulted in clinically meaningful improvements in CRR, DOR, DOCR, and less frequent use of subsequent tx, demonstrating deep and durable responses. OS was not significantly different, being confounded by COVID-19 mortality and use of novel subsequent tx. Observed AEs were consistent with the established epcor safety profile and the expected profile for a CD3×CD20 bsAb.

**Copyright Information:** (Online) ISSN: 2572-9241
©2026 The Author(s). HemaSphere is published by John Wiley & Sons Ltd on behalf of European Hematology Association. This is an open access Abstract Book distributed under the Attribution-NonCommercial-NoDerivs (CC BY-NC-ND), which allows third parties to download the articles and share them with others as long as they credit the author and the Abstract Book, but they cannot change the content in any way or use them commercially.
**Abstract Book Citations:** Authors, Title, HemaSphere, 2026;10:(S1):pages. The abstract book DOIs can be found on the "Author Information" page.
**Disclaimer:** Articles published in the journal HemaSphere exclusively reflect the opinions of the authors. The authors are responsible for all content in their abstracts including accuracy of the facts, statements, citing resources, etc.

### (S236) VISPA-CEL, AN ALLOGENEIC ANTI-CD19 CAR-T CELL THERAPY WITH A PD-1 KNOCKOUT, IN PATIENTS WITH RELAPSED/REFRACTORY B CELL NON-HODGKIN LYMPHOMA (ANTLER PHASE 1 CLINICAL TRIAL)

**Topic:** 19. Large B cell lymphomas - Clinical

Stephen Schuster*[1], Houston Holmes[2], James Essell[3], Ayad Hamdan[4], Boyu Hu[5], Elizabeth Brem[6], Rushang Patel[7], Sunita Nasta[8], Costas K. Yannakou[9], Mohamad Cherry[10], Don Stevens[11], Umar Farooq[12], Kenneth Micklethwaite[13], Vamsi Kota[14], Abraham Kanate[15], Tatyana Feldman[16], Melhem Solh[17], David Lavie[18], Ben Carnley[19], Muhammad Husnain[20], Cesar Gentille[21], Fevzi Yalniz[22], Gary Simmons[23], Donna Marcy[24], Erin Mullane[24], Jim Peck[24], Chris Holland[24], Ashraf Garrett[24], Govind Shah[24], Pingping Mao[24], Eric Stawiski[24], Enrique Zudaire[24], Tonia Nesheiwat[24], Socorro Portella[24], Loretta Nastoupil[25], Susan O'brien[26], Mehdi Hamadani[27]

*[1]Abramson Cancer Center, University of Pennsylvania, Philadelphia, PA, United States of America;[2]Texas Oncology, Dallas, TX, United States of America; [3]Oncology Hematology Care, Sarah Cannon Research Institute, Cincinnati, OH, United States of America; [4]University of California San Diego, Department of Medicine, Division of Hematology and Cellular Therapy, La Jolla, CA, United States of America; [5]Division of Hematology and Hematologic Malignancies, University of Utah Huntsman Cancer Institute, Salt Lake City, UT, United States of America; [6]Division of Hematology/Oncology, University of California, Irvine Health, Orange, CA, United States of America; [7]AdventHealth, Orlando, FL, United States of America;[8]Division of Hematology Oncology, Abramson Cancer Center, University of Pennsylvania, Philadelphia, PA, United States of America; [9]Epworth HealthCare and The University of Melbourne, Melbourne, Australia; [10]Atlantic Health System, Morristown, NJ, United States of America;[11]Norton Cancer Institute, Louisville, KY, United States of America; [12]University of Iowa Hospitals and Clinics, Iowa City, IA, United States of America; [13]Westmead Hospital and Blood Transplant and Cell Therapies Laboratory, NSW Health Pathology - ICPMR Westmead, Sydney, Australia; [14]Augusta University - Georgia Cancer Center, Augusta, GA, United States of America; [15]HonorHealth Cancer Transplant Institute, Scottsdale, AZ, United States of America; [16]John Theurer Cancer Center at Hackensack Meridian Health, Hackensack Meridian Health School of Medicine, Hackensack, NJ, United States of America; [17]Northside Hospital Cancer Institute, Atlanta, GA, United States of America; [18]Hadassah Medical Center, Jerusalem, Israel; [19]Royal Perth Hospital and Cell and Tissue Therapies WA, Perth, Australia;[20]Division of Hematology/Oncology, University of Arizona, Tucson, AZ, United States of America; [21]UAMS Winthrop P. Rockefeller Cancer Institute, Little Rock, AR, United States of America; [22]Division of Hematology and Cellular Therapy, University of Kentucky Markey Cancer Center, Lexington, KY, United States of America; [23]Virginia Oncology Associates, Norfolk, VA, United States of America;[24]Caribou Biosciences, Inc., Berkeley, CA, United States of America; [25]CommonSpirit Oncology Mercy, Durango, CO, United States of America;[26]Chao Family Comprehensive Cancer Center, University of California Irvine Medical Center, Division of Hematology/Oncology, Department of Medicine, Orange, CA, United States of America; [27]Division of Hematology & Oncology, Medical College of Wisconsin, Milwaukee, WI, United States of America;*

### Background

Vispacabtagene regedleucel (vispa-cel, formerly CB-010) is an allogeneic anti-CD19 CAR-T cell therapy manufactured from healthy donor T cells. The Cas9 chRDNA genome-editing technology is used for three genome edits: (1) KO of *TRAC* to reduce risk of GvHD, (2) insertion of a CD19-specific CAR (FMC63 scFv, 4-1BB), and (3) KO of PD-1 to prevent premature CAR-T cell exhaustion and potentially enhance antitumor activity.

### Aims

ANTLER is a Phase 1 trial (NCT04637763) with 3+3 dose escalation and expansion to evaluate the safety and efficacy of vispa-cel in patients (pts) with r/r B-NHL and determine the RP2D.

**Copyright Information:** (Online) ISSN: 2572-9241
©2026 The Author(s). HemaSphere is published by John Wiley & Sons Ltd on behalf of European Hematology Association. This is an open access Abstract Book distributed under the Attribution-NonCommercial-NoDerivs (CC BY-NC-ND), which allows third parties to download the articles and share them with others as long as they credit the author and the Abstract Book, but they cannot change the content in any way or use them commercially.
**Abstract Book Citations:** Authors, Title, HemaSphere, 2026;10:(S1):pages. The abstract book DOIs can be found on the "Author Information" page.
**Disclaimer:** Articles published in the journal HemaSphere exclusively reflect the opinions of the authors. The authors are responsible for all content in their abstracts including accuracy of the facts, statements, citing resources, etc.

### Methods

All pts received lymphodepletion of sequential cyclophosphamide (60 mg/kg/day x 2 days) then fludarabine (25 mg/m$^2$/day x 5 days) followed by a single infusion of vispa-cel. Three vispa-cel doses (40 million [M], 80M, and 120M cells) were evaluated in dose escalation and dose expansion. 80M cells was selected as the RP2D. Subsequently, a 2L LBCL confirmatory cohort prospectively evaluated donor-recipient matching ≥4 HLA alleles. A retrospective analysis was performed and identified LBCL pts treated with vispa-cel manufactured from a young donor (<30 yrs) and ≥2 matched HLA alleles, reported as the optimized cohort.

### Results

As of Sep 2025, 84 pts received vispa-cel including 67 2L LBCL pts. The median age was 66 years (20-86). Of the 2L LBCL pts, 40 pts (60%) had an aaIPI score of ≥2. Vispa-cel was generally well tolerated and no GvHD was observed. CRS occurred in 55% of pts (1% Gr ≥3), ICANS occurred in 14% of pts (4% Gr ≥3), infections occurred in 51% of pts (25% Gr ≥3), Gr ≥3 prolonged cytopenias occurred in 28% of pts, and immune effector cell-associated hemophagocytic lymphohistiocytosis-like syndrome occurred in 2% of pts (all Gr≥3). By day 60, the neutropenia and thrombocytopenia recovery to Gr ≤2 was 95% and 86%, respectively. Five patients died due to AEs following vispa-cel. 1 related (IEC-HS at day 25); 1 possibly related (bladder perforation at day 172); 3 deaths unrelated to vispa-cel per investigator were acute respiratory failure/pneumothorax, ARDS, and HHV6 encephalitis. In the 2L confirmatory cohort (N=22), ORR was 82% and CR rate was 64%. Median PFS and DOR were not reached (NR) with a 12-month PFS of 51%. Donor age (<30 yrs) and ≥2 matched HLA alleles were identified as covariates associated with improved PFS. In the optimized cohort (N=35), ORR was 86% and CR rate was 63%. The median PFS and DOR were NR with a 12-month PFS of 53%. In contrast, the median PFS in pts treated with vispa-cel from older donor (≥30 yrs) T cells and with <2 HLA alleles matched was 2.5 months (Figure 1). Peak expansion occurred days 7-10 with persistence up to 30 days post-infusion. Endogenous T and NK cells recovered rapidly in peripheral blood (~4 weeks) post-infusion, with B cell recovery in all pts by ~300 days. Updated clinical data with translational analyses from the optimized cohort will be presented.

### Summary/Conclusion

Vispa-cel is an allogeneic CAR-T cell therapy that results in deep and durable responses on par with approved autologous CAR-T cell therapies in 2L LBCL pts with a generally well-tolerated safety profile supporting outpatient administration. Improved PFS was observed in pts treated with vispa-cel manufactured from young donors (<30 yrs) and ≥2 matched HLA alleles. A randomized controlled pivotal trial in 2L LBCL pts who are ineligible for both autologous CAR-T and transplant is being planned.

**Copyright Information:** (Online) ISSN: 2572-9241
©2026 The Author(s). HemaSphere is published by John Wiley & Sons Ltd on behalf of European Hematology Association. This is an open access Abstract Book distributed under the Attribution-NonCommercial-NoDerivs (CC BY-NC-ND), which allows third parties to download the articles and share them with others as long as they credit the author and the Abstract Book, but they cannot change the content in any way or use them commercially.
**Abstract Book Citations:** Authors, Title, HemaSphere, 2026;10:(S1):pages. The abstract book DOIs can be found on the "Author Information" page.
**Disclaimer:** Articles published in the journal HemaSphere exclusively reflect the opinions of the authors. The authors are responsible for all content in their abstracts including accuracy of the facts, statements, citing resources, etc.

Figure 1: Partial HLA matching and young donors lead to durable outcomes with vispa-cel in LBCL patients

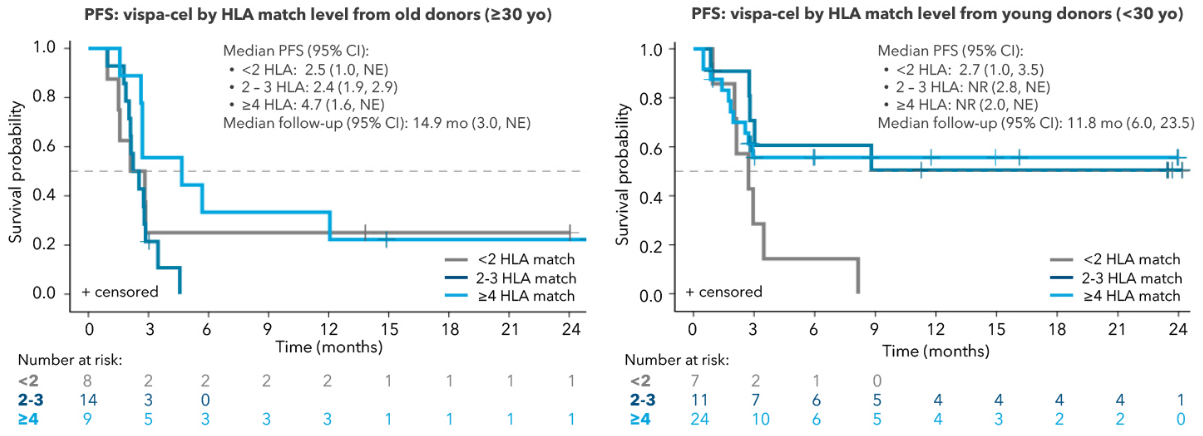

**Copyright Information:** (Online) ISSN: 2572-9241
©2026 The Author(s). HemaSphere is published by John Wiley & Sons Ltd on behalf of European Hematology Association. This is an open access Abstract Book distributed under the Attribution-NonCommercial-NoDerivs (CC BY-NC-ND), which allows third parties to download the articles and share them with others as long as they credit the author and the Abstract Book, but they cannot change the content in any way or use them commercially.
**Abstract Book Citations:** Authors, Title, HemaSphere, 2026;10:(S1):pages. The abstract book DOIs can be found on the "Author Information" page.
**Disclaimer:** Articles published in the journal HemaSphere exclusively reflect the opinions of the authors. The authors are responsible for all content in their abstracts including accuracy of the facts, statements, citing resources, etc.

### (S237) POLATUZUMAB VEDOTIN PLUS RITUXIMAB, IFOSFAMIDE, CARBOPLATIN, ETOPOSIDE (POLA-R-ICE) VS R-ICE AS SECOND LINE TREATMENT IN LARGE B CELL LYMPHOMA (LBCL). FINAL ANALYSIS OF A RANDOMIZED PHASE 3 STUDY

**Topic:** 19. Large B cell lymphomas - Clinical

Bertram Glass*[1], Anna Ossami Saidy[1, 2], Ralph Michael[3], Frank Kroschinsky[4], Maria Fatima De La Cruz Vicente[5], Andreas Viardot[6], Andrea Kerkhoff[7], Pau Abrisqueta[8], Christopher P. Fox[9, 10], Philipp Staber[11], Sonja Heibl[12], Anna Polowinski[13], Tracey Mason[14], Santiago Montes Moreno[15], Bettina Altmann[3], Richard Greil[16], Andreas Rosenwald[17], Dirk Hellwig[18], Marita Ziepert[3], Andrew Davies[19], Alejandro Martín García-Sancho[20]

*[1]Helios Klinikum Berlin-Buch, Berlin, Germany; [2]MSB Medical School Berlin, Department of Human Medicine,, Berlin, Germany; [3]Institute for Medical Informatics, Statistics, and Epidemiology, University of Leipzig, Leipzig, Germany;[4]University Hospital Carl Gustav Carus, Technical University Dresden, Medical Department I, Dresden, Germany; [5]Hospital Universitario Virgen del Rocío, Instituto de Biomedicina de Sevilla (IBIS)/CSIC/Universidad de Sevilla, Sevilla, Spain; [6]Department of Internal Medicine III, University Hospital of Ulm, Ulm, Germany; [7]Department for Medicine A, Hematology, Oncology, Hemostaseology and Pneumology, University Hospital Muenster, Muenster, Germany; [8]Hospital Vall d'Hebron, Barcelona, Spain; [9]University of Nottingham, Nottingham, United Kingdom; [10]Nottingham University Hospitals NHS Trust, UK, Nottingham, United Kingdom; [11]Division of Hematology and Hemostaseology, Department of Medicine I, Medical University of Vienna, Vienna, Austria; [12]Klinikum Wels-Grieskirchen, Wels, Austria; [13]GWT-TUD GmbH, Dresden, Germany; [14]Southampton Clinical Trials Unit, University of Southampton, Southampton, United Kingdom; [15]Translational Hematopathology Laboratory and Anatomic Pathology Service, Hospital Universitario Marqués de Valdecilla/IDIVAL, Universidad de Cantabria, Santander, Spain; [16]University Clinic of PMU Salzburg, Salzburg, Austria; [17]Institute of Pathology, University of Würzburg, Würzburg, Germany; [18]University Hospital Regensburg, Department of Nuclear Medicine, Regensburg, Germany; [19]University of Southampton, Southampton General Hospital, Southampton, United Kingdom; [20]Hospital Universitario de Salamanca, IBSAL, CIBERONC, Universidad de Salamanca, Salamanca, Spain;*

### Background

R-ICE is one of the standard induction regimens prior to autologous stem cell transplantation (ASCT) in the treatment of patients (pts) with relapsed and refractory (r/r) LBCL after first line therapy and is also used as bridging therapy prior to CAR T-cell therapy (CART) in this situation. However, further improvement of induction and bridging regimens is needed. Polatuzumab vedotin is a CD-79b directed antibody-drug conjugate with proven efficacy in r/r LBCL.

### Aims

This open label, multinational, randomized phase III clinical study compared safety and efficacy of polatuzumab vedotin plus R-ICE (Pola-R-ICE; arm E) with R-ICE (arm S).

### Methods

Key inclusion criteria were diagnosis of first relapse or progression of LBCL, ECOG PS ≤ 2 and adequate organ function. Pts in arm E received polatuzumab vedotin 1.8 mg/kg at d1, in addition to SOC R-ICE. Pts went to consolidation therapy by ASCT or CART by decision of the investigator outside the clinical study. The primary endpoint was event-free survival (EFS). Key secondary endpoints included progression-free survival (PFS) and overall survival (OS). The threshold to reject the null hypothesis was a hazard ratio (HR) of 0.663 with a power of 80% and alpha 5% (two-sided).

**Copyright Information:** (Online) ISSN: 2572-9241
©2026 The Author(s). HemaSphere is published by John Wiley & Sons Ltd on behalf of European Hematology Association. This is an open access Abstract Book distributed under the Attribution-NonCommercial-NoDerivs (CC BY-NC-ND), which allows third parties to download the articles and share them with others as long as they credit the author and the Abstract Book, but they cannot change the content in any way or use them commercially.
**Abstract Book Citations:** Authors, Title, HemaSphere, 2026;10:(S1):pages. The abstract book DOIs can be found on the "Author Information" page.
**Disclaimer:** Articles published in the journal HemaSphere exclusively reflect the opinions of the authors. The authors are responsible for all content in their abstracts including accuracy of the facts, statements, citing resources, etc.

**Results**

From December 2021 to December 2024, a total of 296 pts were randomized, 148 in each arm. One patient in each arm received no study therapy and was excluded from the analysis. All relevant baseline characteristics were well balanced. Characteristics for the complete cohort included: mean age of 57 years, ECOG > 1 in 7%, primary refractory disease in 42%, bulky disease in 22%, elevated LDH in 55%, histology of DLBCL (all subtypes) in 77%, with non-GCB by Hans-classifier in 111 pts. Both arms had limited severe toxicity with only one case of treatment related mortality per arm (arm E: ischemic colitis, arm S: cardiorespiratory arrest). There were more infections grade ≥ 3 in arm E (23 vs 12 pts, p = 0.048). Other toxicities were equally distributed. With a median observation time of 24.6 months, there was no statistically significant difference between Pola-R-ICE and R-ICE at 2 years in EFS (26% vs 30%), PFS (47% vs 41%) and OS (75% vs 64%). However, subgroup analyses showed significantly better PFS and OS after Pola-R-ICE compared to R-ICE at 2 years in pts with histologic subtype DLBCL (PFS 52% vs 42%, p = 0.040, OS 77% vs 61%, p = 0.033), relapsed disease (PFS 66% vs 46%, p = 0.004, OS 87% vs 71%, p = 0.015), bulky disease (PFS 45% vs 23%, p = 0.006, OS 76% vs 39%, p = 0.011) and high LDH (PFS 44% vs 31%, p = 0.034, OS 67% vs 49%, p = 0.036). PFS was significantly better in pts with non-GCB subtype (54% vs 34%, p = 0.040). Multivariate analysis confirmed treatment results for EFS (HR 1.0, p = 0.901) and showed primary refractory disease as the most important adverse prognostic factor for EFS, PFS and OS (HR 2.9; 3.0; 3.2, p< .0001). Other significant adverse prognostic factors for EFS were high LDH (HR 1.5, p = 0.005) and ECOG > 1 (HR 1.8, p = 0.032). Additionally, age > 60 years was an adverse prognostic factor for OS (HR 1.7, p = 0.031).

**Summary/Conclusion**

This study could not detect a benefit of applying Pola-R-ICE over R-ICE as second line treatment for pts with LBCL. However, pts with DLBCL, relapsed disease (in contrast to those with primary refractory disease) and those with high tumour burden (bulky disease, high LDH) may clinically benefit from Pola-R-ICE instead of R-ICE when given prior to consolidative therapy with ASCT or CART.

**Copyright Information:** (Online) ISSN: 2572-9241
©2026 The Author(s). HemaSphere is published by John Wiley & Sons Ltd on behalf of European Hematology Association. This is an open access Abstract Book distributed under the Attribution-NonCommercial-NoDerivs (CC BY-NC-ND), which allows third parties to download the articles and share them with others as long as they credit the author and the Abstract Book, but they cannot change the content in any way or use them commercially.
**Abstract Book Citations:** Authors, Title, HemaSphere, 2026;10:(S1):pages. The abstract book DOIs can be found on the “Author Information” page.
**Disclaimer:** Articles published in the journal HemaSphere exclusively reflect the opinions of the authors. The authors are responsible for all content in their abstracts including accuracy of the facts, statements, citing resources, etc.

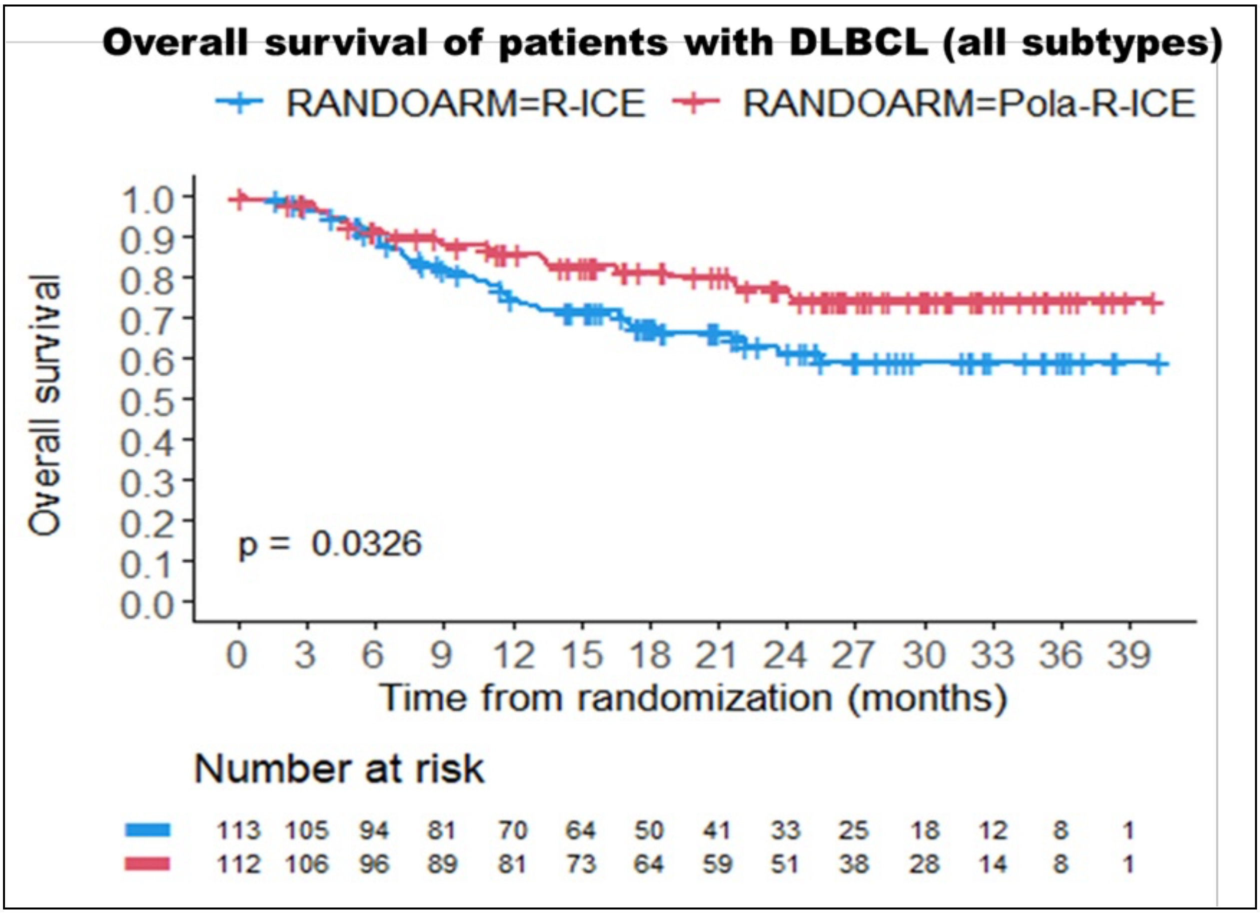

**Copyright Information:** (Online) ISSN: 2572-9241
©2026 The Author(s). HemaSphere is published by John Wiley & Sons Ltd on behalf of European Hematology Association. This is an open access Abstract Book distributed under the Attribution-NonCommercial-NoDerivs (CC BY-NC-ND), which allows third parties to download the articles and share them with others as long as they credit the author and the Abstract Book, but they cannot change the content in any way or use them commercially.
**Abstract Book Citations:** Authors, Title, HemaSphere, 2026;10:(S1):pages. The abstract book DOIs can be found on the "Author Information" page.
**Disclaimer:** Articles published in the journal HemaSphere exclusively reflect the opinions of the authors. The authors are responsible for all content in their abstracts including accuracy of the facts, statements, citing resources, etc.

### (S238) SAFETY DATA AND INITIAL POOLED EFFICACY DATA FROM THE NORDIC LYMPHOMA GROUP PHASE 3 POLAR BEAR TRIAL IN ELDERLY OR FRAIL PATIENTS WITH DIFFUSE LARGE CELL LYMPHOMA - R-POLA-MINI-CHP VS. R-MINI-CHOP

**Topic:** 19. Large B cell lymphomas - Clinical

Mats Jerkeman*[1], Andrés José María Ferreri[2], Peter Brown[3], Matthew Ku[4], Julian Hamfjord[5], Elisa Lucchini[6], Alessandro Morotti[6], Alessandro Severino[7], Judit Jørgensen[8], Carlo Visco[9], Karen Juul-Jensen[10], Maurilio Ponzoni[2], Lene Sjø[3], Mats Ehinger[1], Penny McKelvie[4], Klaus Beiske[5], Marja-Liisa Karjalainen-Lindsberg[11], Emilie Skou[8], Sunita Palanisamy[12], Sara Ekberg[13], Sirpa Leppä[11]

*[1]Lund University, Lund, Sweden; [2]IRCCS San Raffaele Scientific Institute, Milano, Italy; [3]Rigshospitalet, Copenhagen, Denmark; [4]St Vincent's Hospital and University of Melbourne, Melbourne, Australia; [5]Norwegian Radium Hospital, Oslo, Norway; [6]University of Turin, Torino, Italy; [7]Universitaria Federico, Napoli, Italy; [8]Aarhus University Hospital, Aarhus, Denmark; [9]University of Verona, Verona, Italy; [10]Odense University Hospital, Odense, Denmark; [11]Helsinki University Hospital, Helsinki, Finland; [12]Australasian Leukaemia & Lymphoma Group, Melbourne, Australia; [13]Red Door Analytics AB, Stockholm, Sweden;*

### Background

For patients with diffuse large B-cell lymphoma (DLBCL), overall survival (OS) has substantially improved during the last decade. An exception is the group of patients above 80 years of age, a group that also constitutes an increasing proportion of DLBCL patients due to a demographic shift, with a progressively aging population. Effective and safe treatment for this population is an obvious unmet medical need.

### Aims

In this randomized phase 3 trial (NCT04332822), we evaluated tolerability and efficacy of Pola-R-mini-CHP as first line treatment for elderly/frail patients with DLBCL, compared to the current treatment standard, R-mini-CHOP. Here, we present data on safety from the 300 patients in this trial, as well as efficacy pooled across treatment arms, including subgroups.

### Methods

Patients with newly diagnosed DLBCL, >80 years, or 75-80 years and frail, according to a simplified Comprehensive Geriatric Assessment, were randomized 1:1 to either the standard treatment for this population, R-miniCHOP (Arm A), or an experimental regimen, Pola-R-miniCHP (Arm B), where vincristine is substituted by polatuzumab vedotin, 1.8 mg/kg, day 1, both for 6 cycles. The sample size is based on a target HR for PFS of 0.64, and the total number of events necessary to achieve at least 80% power with type I error $\alpha$=0.05, is 158. In this report, the highest grade of adverse event for each category, according to CTCAE v 5, was reported. The data cut-off was 31-AUG-2025.

**Copyright Information:** (Online) ISSN: 2572-9241
©2026 The Author(s). HemaSphere is published by John Wiley & Sons Ltd on behalf of European Hematology Association. This is an open access Abstract Book distributed under the Attribution-NonCommercial-NoDerivs (CC BY-NC-ND), which allows third parties to download the articles and share them with others as long as they credit the author and the Abstract Book, but they cannot change the content in any way or use them commercially.
**Abstract Book Citations:** Authors, Title, HemaSphere, 2026;10:(S1):pages. The abstract book DOIs can be found on the "Author Information" page.
**Disclaimer:** Articles published in the journal HemaSphere exclusively reflect the opinions of the authors. The authors are responsible for all content in their abstracts including accuracy of the facts, statements, citing resources, etc.

**Results**

Between 08/2020 and 06/2025, enrollment was completed, with 300 patients from 55 sites in Sweden, Norway, Denmark, Finland, Italy, Australia and New Zealand, 150 in each arm. 48 patients (16%) were <80 years, and enrolled due to frailty. The median age was 82 years. 25 (8.3%) presented with performance status 3. The median follow-up is currently 14.8 months.
At the time of analysis, there were 85 events, of which 35 deaths were the first occurring event. The pooled 2-year progression-free survival (PFS) was 67% (95% CI 60-73). Age (<80, 80-90 or ≥90 years) or comorbidity (CIRS-G <10 or ≥10) had no impact on PFS. Pooled OS at 2 years was 76% (95% CI 70-81).
In total, grade ≥3 events occurred in 45 patients (30%) in Arm A and 54 patients (36%) in Arm B. There was no difference in terms of hematological toxicity between the treatment groups. In terms of infections, there were 11 grade ≥3 events in Arm A (7.3%), vs 19 in Arm B (12.7%). Gastrointestinal toxicity grade >2, was recorded in 9 Arm-B pts, (6%), and 5 Arm-A pts (3.3 %). Peripheral neuropathy was usually of grade 1-2 (10.7% in arm A and 17.3% in arm B), with only two episodes of grade ≥3 in arm B. There were 10 grade 5 adverse events, 2 in Arm A (infection: 1; other: 1) and 8 in Arm B (respiratory: 4; infection: 2; cardiovascular: 1; CNS: 1; gastrointestinal: 1).

**Summary/Conclusion**

Rituximab/anthracycline-based chemotherapy remains the standard of care for elderly and frail patients with DLBCL, demonstrating excellent outcomes with acceptable tolerability. Compared to historical data with R-mini-CHOP (Peyrade 2011), showing 2 year PFS of 47% and OS of 59%—our observed pooled 2-year PFS of 67% and OS of 76% are highly encouraging. Infectious complications, gastrointestinal toxicity, and peripheral neuropathy were generally manageable, and substitution of vincristine with polatuzumab vedotin in the R-mini-CHOP did not result in a significant increase in either the incidence or severity of toxicity.

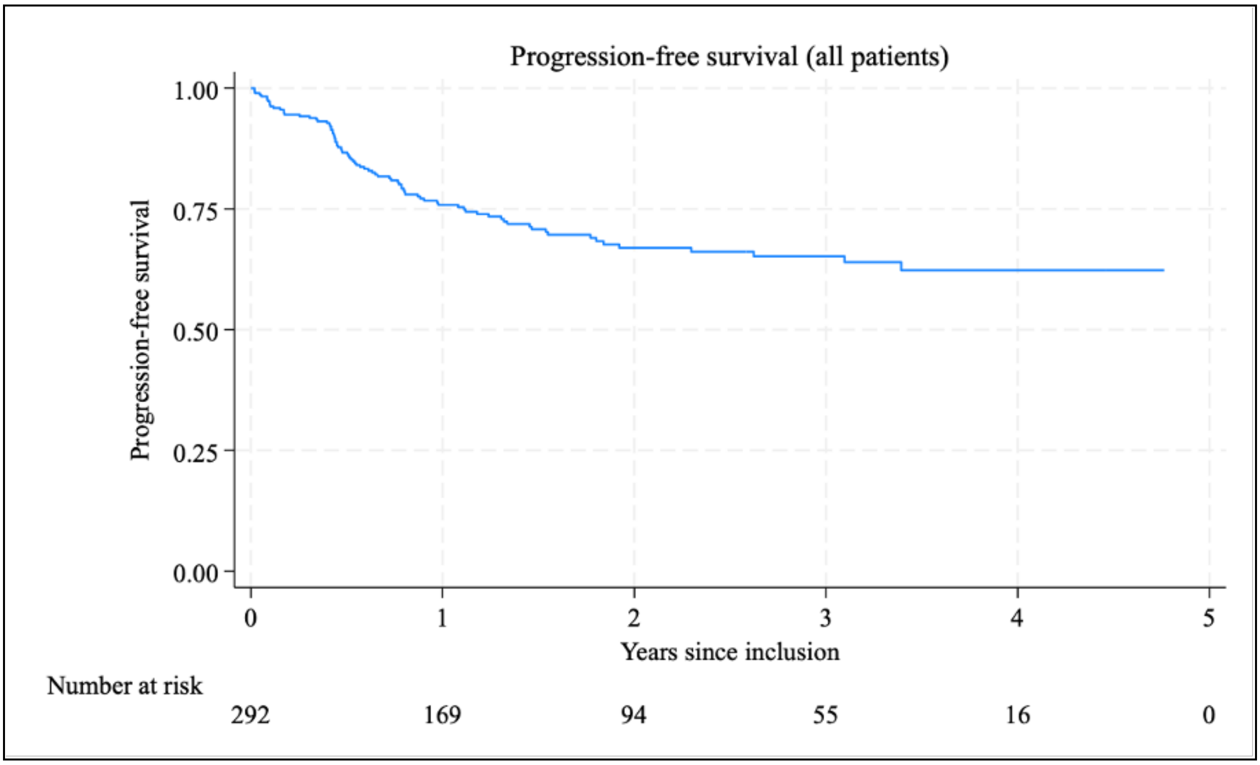

**Copyright Information:** (Online) ISSN: 2572-9241
©2026 The Author(s). HemaSphere is published by John Wiley & Sons Ltd on behalf of European Hematology Association. This is an open access Abstract Book distributed under the Attribution-NonCommercial-NoDerivs (CC BY-NC-ND), which allows third parties to download the articles and share them with others as long as they credit the author and the Abstract Book, but they cannot change the content in any way or use them commercially.
**Abstract Book Citations:** Authors, Title, HemaSphere, 2026;10:(S1):pages. The abstract book DOIs can be found on the "Author Information" page.
**Disclaimer:** Articles published in the journal HemaSphere exclusively reflect the opinions of the authors. The authors are responsible for all content in their abstracts including accuracy of the facts, statements, citing resources, etc.

### (S239) GOLCADOMIDE (GOLCA), A POTENTIAL, FIRST-IN-CLASS ORAL CELMOD + POLA-RCHP IN PATIENTS (PTS) WITH NEWLY DIAGNOSED AGGRESSIVE B-CELL LYMPHOMA (A-BCL): SAFETY AND 12-MONTH EFFICACY FROM A PHASE 1B STUDY

**Topic:** 19. Large B cell lymphomas - Clinical

Maria Bouzani*[1], Marc Hoffmann[2], Theodoros Vassilakopoulos[3], Grzegorz Nowakowski[4], Dagmara Zimowska-Curyło[5], Muhamad Alhaj Moustafa[6], Alejandro Martín García-Sancho[7], Juan-Manuel Sancho Cia[8], Wojciech Jurczak[9], Arpankumar Patel[10], Floriane Boucaud[11], Argyrios Gkasiamis[11], Mark Kaplan[12], Arnaud Amzallag[13], Matthew E. Stokes[14], Tanvi Jindia[15], Serena Perna[12], Michal Kwiatek[16]

*[1]Evaggelismos General Hospital, Athens, Greece; [2]The University of Kansas Cancer Center, Kansas City, United States of America; [3]National and Kapodistrian University of Athens, Laikon General Hospital, Athens, Greece; [4]Mayo Clinic, Rochester, United States of America; [5]Krakow University Hospital, Krakow, Poland; [6]Mayo Clinic, Jacksonville, United States of America; [7]Hospital Universitario de Salamanca, IBSAL, CIBERONC, University of Salamanca, Salamanca, Spain; [8]Germans Trias i Pujol University Hospital, ICO-IJC-Badalona, Spain; [9]MSC National Research Institute of Oncology, Krakow, Poland; [10]Bristol Myers Squibb, Princeton, United States of America; [11]Bristol Myers Squibb, Boudry, Switzerland; [12]Bristol Myers Squibb, Madison, United States of America; [13]Bristol Myers Squibb, Cambridge, United States of America; [14]Bristol Myers Squibb, Summit, United States of America; [15]Bristol Myers Squibb, Hyderabad, Telangana, India; [16]Poznan University of Medical Sciences, Poznan, Poland;*

### Background

Approximately 40% of pts with diffuse large B-cell lymphoma (DLBCL) will relapse or have refractory disease following first-line (1L) treatment (Tx) with standard of care (SOC) chemoimmunotherapy regimens R-CHOP or Pola-RCHP. High risk (HR) populations include those with International Prognostic Index (IPI) score 3–5 and IPI 1–2 with either ≥1 lesion ≥7 cm or baseline LDH >1.3×ULN (Maurer et al, ASH 2023, #4512).
GOLCA is a potential, first-in-class, oral CELMoD designed for the Tx of lymphoma, with preferential distribution to lymphoid organs and enhanced activity in lymphoma cell lines. GOLCA drives the closed, active conformation of cereblon to induce rapid and deep degradation of Ikaros and Aiolos, leading to direct cell killing (agnostic of cell of origin [COO]) and immunomodulatory activity.
CC-220-DLBCL-001 (NCT04884035) is an ongoing Phase (Ph) 1b, open-label, multicenter trial to assess safety and preliminary efficacy of GOLCA + R-CHOP and GOLCA + Pola-RCHP in pts with newly diagnosed a-BCL. GOLCA + R-CHOP demonstrated a manageable safety profile and encouraging efficacy with high end of Tx (EOT) complete metabolic response (CMR; 88%), minimal residual disease (MRD ≥90%) negativity rates and 24-month (mo) PFS (79%) (Nowakowski et al, ASH 2025, 476) and is being evaluated in the Ph 3 study GOLSEEK-1 (NCT06356129).

### Aims

To evaluate safety and 12-month efficacy for GOLCA + Pola-RCHP in 1L a-BCL.

### Methods

Pts aged ≥18 y with ECOG PS ≤2, IPI 0–5 (Part 2A) or 2–5 (Part 2B), and newly diagnosed a-BCL with measurable tumorous disease were included. Pts were treated with GOLCA + Pola-RCHP for six 21-day (D) cycles or until disease progression/unacceptable toxicity/study withdrawal. In dose escalation, GOLCA was dosed at 0.2 and 0.4 mg D1–7. In dose expansion, pts were randomized 1:1 to Pola-RCHP + GOLCA 0.2 or 0.4 mg. Primary endpoint was safety; efficacy was a secondary endpoint.

**Copyright Information:** (Online) ISSN: 2572-9241
©2026 The Author(s). HemaSphere is published by John Wiley & Sons Ltd on behalf of European Hematology Association. This is an open access Abstract Book distributed under the Attribution-NonCommercial-NoDerivs (CC BY-NC-ND), which allows third parties to download the articles and share them with others as long as they credit the author and the Abstract Book, but they cannot change the content in any way or use them commercially.
**Abstract Book Citations:** Authors, Title, HemaSphere, 2026;10:(S1):pages. The abstract book DOIs can be found on the "Author Information" page.
**Disclaimer:** Articles published in the journal HemaSphere exclusively reflect the opinions of the authors. The authors are responsible for all content in their abstracts including accuracy of the facts, statements, citing resources, etc.

**Results**

At data cutoff (DCU), 57 pts were treated with GOLCA + Pola-RCHP (0.2 mg, n=28; 0.4 mg, n=29). Median age was 63 y. Most pts had HR disease (86%) and DLBCL not otherwise specified histology (84%). COO was germinal center B cell (GCB) in 47% of pts and non-GCB in 39% of pts by Hans algorithm. Forty-seven pts (83%) completed Tx and 52 (91%) entered the follow-up (f/u) period.
Grade 3/4 Tx-emergent adverse events occurred in 95% of pts, most frequently neutropenia (86%; an on-target side effect of GOLCA) and anemia (39%). Median relative dose intensity for GOLCA and Pola-RCHP components was >95%.
At 15.5 mo median f/u, EOT CMR rate was 69% (n=18) at 0.2 mg, 82% (n=23) at 0.4 mg (all 23 pts were alive and progression-free at DCU; fig), and similar across COO subtypes. EOT CMR was consistent in HR pts (65% [n=15] at 0.2 mg, 83% [n=20] at 0.4 mg). The 12-mo DOR and PFS rates were 92% and 88% at 0.2 mg, and 96% and 96% at 0.4 mg.
In pts with both pre-Tx and EOT ctDNA analysis, 65% and 83% at 0.2 and 0.4 mg achieved MRD negativity (undetectable MRD by PhasED-Seq) at EOT.

**Summary/Conclusion**

GOLCA + Pola-RCHP demonstrated a predictable and manageable safety profile, and addition of GOLCA did not compromise median dose intensity of Pola-RCHP. GOLCA 0.4 mg + Pola-RCHP resulted in a high rate of durable CMRs, high rate of MRD negativity, and a promising 12-mo PFS rate. These data continue to show that GOLCA + SOC regimens R-CHOP and Pola-RCHP has potential to improve outcomes in 1L HR a-BCL.

Acknowledgement: BMS ChatGPT4 used to revise text with human input.

© 2026 American Society of Clinical Oncology, Inc. Reused with permission. This abstract was accepted and previously presented at the 2026 ASCO Annual Meeting. All rights reserved.

**Copyright Information:** (Online) ISSN: 2572-9241
©2026 The Author(s). HemaSphere is published by John Wiley & Sons Ltd on behalf of European Hematology Association. This is an open access Abstract Book distributed under the Attribution-NonCommercial-NoDerivs (CC BY-NC-ND), which allows third parties to download the articles and share them with others as long as they credit the author and the Abstract Book, but they cannot change the content in any way or use them commercially.
**Abstract Book Citations:** Authors, Title, HemaSphere, 2026;10:(S1):pages. The abstract book DOIs can be found on the "Author Information" page.
**Disclaimer:** Articles published in the journal HemaSphere exclusively reflect the opinions of the authors. The authors are responsible for all content in their abstracts including accuracy of the facts, statements, citing resources, etc.

Figure. Treatment duration and response (safety population)

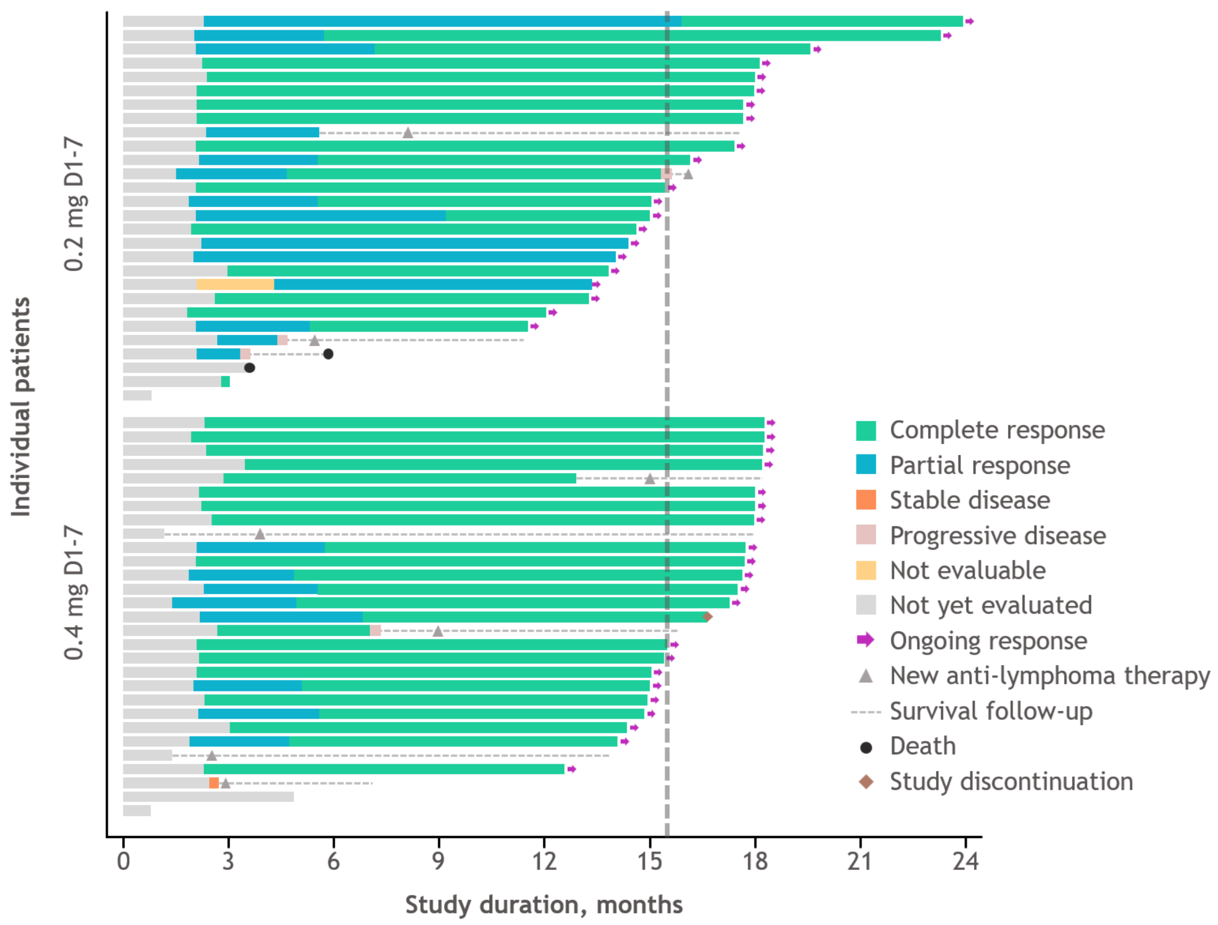

**Copyright Information:** (Online) ISSN: 2572-9241
©2026 The Author(s). HemaSphere is published by John Wiley & Sons Ltd on behalf of European Hematology Association. This is an open access Abstract Book distributed under the Attribution-NonCommercial-NoDerivs (CC BY-NC-ND), which allows third parties to download the articles and share them with others as long as they credit the author and the Abstract Book, but they cannot change the content in any way or use them commercially.
**Abstract Book Citations:** Authors, Title, HemaSphere, 2026;10:(S1):pages. The abstract book DOIs can be found on the "Author Information" page.
**Disclaimer:** Articles published in the journal HemaSphere exclusively reflect the opinions of the authors. The authors are responsible for all content in their abstracts including accuracy of the facts, statements, citing resources, etc.

### (S240) REAL WORLD EXPERIENCE OF GLOFITAMAB AND EPCORITAMAB FOR LARGE B CELL LYMPHOMA IN THE UK: EXTENDED ANALYSIS OF DURATION OF REMISSION AND FACTORS AFFECTING RESPONSE IN A COHORT OF 324 PATIENTS

**Topic:** 19. Large B cell lymphomas - Clinical

William Townsend*[1, 2], Euan Haynes[3], William Wilson[2], James Paterson[2], Kushani Ediriwickrema[2, 4], Diva Baggio[2], Tobias Menne[3], Dima El-Sharkawi[5], Li Yuan Chan[5], Sunil Iyengar[5], Sridhar Chaganti[6], Angus Broom[7], Sanne Lugthart[8], Jack Easton[9], Sudarshan Gurung[10], Jeff Smith[11], Andrew Davies[12], Joanna Fawcett[3], Mathew Wells[11], Hwai Jing Hiew[13], David Foldes[14], Mary Owen[15], George Follows[4], Anna Santarsieri[4], Matthew R. Wilson[16], Pam McKay[16], Robert Osborn[16], David Tucker[17], Christina Thiebauld[17], Christopher Fox[18], Mary Gleeson[19], Jeff Lam[19], Laura Oldham[11], Kim Linton[20, 21], Lisa Jeffers[20], Andrea Kuhnl[22], Graham Collins[23], Nimish Shah[24], Rory McCulloch[25], Mahina Baloch[25], David Lewis[26], Kyaw Maw[27], Desmond Ojie[10], Susanna Mathew[28], Rebecca Auer[29], Jane Norman[30], Vismay Deshani[30], Victoria Stables[31], John Willan[32], Matthew Ahearne[33], Laura Dunning[34], Philippa Kelsey[35], Catherine Cox[36], Gavin Preston[37], Sarah Lawless[38], Caroline Besley[39], Dominic Culligan[37], Janine Qasim[23], Abigail Martin[26], Henry Crosland[40], Wendy Osborne[41]

*[1]University College London Hospitals, Haematology, London, United Kingdom; [2]University College London CRUK Cancer Trials Centre, London, United Kingdom; [3]Newcastle Upon Tyne NHS Foundation Trust, Newcastle Upon Tyne, United Kingdom; [4]Cambridge University Hospitals, Cambridge, United Kingdom; [5]The Royal Marsden Hospital, Sutton, United Kingdom; [6]University Hospitals Birmingham, Birmingham, United Kingdom; [7]Edinburgh Cancer Centre, Edinburgh, United Kingdom; [8]University Hospitals Bristol And Weston NHS Foundation Trust, Bristol, United Kingdom;[9]Bristol Haematology and Oncology Centre, Bristol, United Kingdom; [10]Medway Maritime NHS Trust, Medway, United Kingdom; [11]Clatterbridge Cancer Centre, Wirral, United Kingdom; [12]Southampton Experimental Cancer Medicines Centre, Southampton, United Kingdom; [13]University Hospital Southampton NHS Trust, Southampton, United Kingdom; [14]Imperial College Healthcare NHS Trust, London, United Kingdom; [15]Leeds Cancer Centre, Leeds, United Kingdom; [16]Beatson West of Scotland Cancer Centre, Glasgow, United Kingdom; [17]Royal Cornwall Hospital, Truro, United Kingdom;[18]Nottingham University Hospitals NHS Trust City Hospital, Nottingham, United Kingdom; [19]Guy's and St Thomas' NHS Foundation Trust, London, United Kingdom;[20]The Christie NHS Hospital Foundation Trust, Manchester, United Kingdom; [21]The University of Manchester, Division of Cancer Sciences,, Manchester, United Kingdom; [22]King's College Hospital, London, United Kingdom; [23]Oxford Cancer and Haematology Centre, Oxford, United Kingdom; [24]Norfolk and Norwich University Hospital, Norwich, United Kingdom;[25]Gloucestershire Hospitals NHS Foundation Trust, Gloucester, United Kingdom; [26]University Hospitals Plymouth NHS Trust, Plymouth, United Kingdom; [27]James Paget Hospital, Great Yarmouth, United Kingdom; [28]South Tyneside and Sunderland NHS Trust, Sunderland, United Kingdom; [29]Barts Health NHS Trust St Bartholomew's Hospital, London, United Kingdom; [30]Manchester University NHS Foundation Trust, Manchester, United Kingdom; [31]Maidstone and Tunbridge Wells NHS Trust, Kent, United Kingdom; [32]Frimley Health NHS Trust, Frimley, United Kingdom; [33]University Hospitals of Leicester NHS Trust, Leicester, United Kingdom;[34]South Tees Hospitals NHS Trust, Middlesbrough, United Kingdom; [35]Sheffield Teaching Hospitals NHS Trust, Sheffield, United Kingdom;[36]St Georges Hospital, London, United Kingdom; [37]Aberdeen Royal Infirmary, Aberdeen, United Kingdom;[38]Belfast City Hospital, Belfast, United Kingdom; [39]University Hospitals Bristol NHS Foundation Trust, Bristol, United Kingdom;[40]University Hospitals Birmingham NHS Foundation Trust, Birmingham, United Kingdom; [41]The Newcastle upon Tyne Hospitals NHS Foundation Trust and Newcastle University, Newcastle Upon Tyne, United Kingdom;*

### Background

Glofitamab (glofit) and epcoritamab (epcor) are CD3-20 bispecific antibodies (BsAb) licensed for relapsed/refractory large B cell lymphoma (RR LBCL), real-world data can help understand safety, responses and optimal patient selection.

**Copyright Information:** (Online) ISSN: 2572-9241
©2026 The Author(s). HemaSphere is published by John Wiley & Sons Ltd on behalf of European Hematology Association. This is an open access Abstract Book distributed under the Attribution-NonCommercial-NoDerivs (CC BY-NC-ND), which allows third parties to download the articles and share them with others as long as they credit the author and the Abstract Book, but they cannot change the content in any way or use them commercially.
**Abstract Book Citations:** Authors, Title, HemaSphere, 2026;10:(S1):pages. The abstract book DOIs can be found on the "Author Information" page.
**Disclaimer:** Articles published in the journal HemaSphere exclusively reflect the opinions of the authors. The authors are responsible for all content in their abstracts including accuracy of the facts, statements, citing resources, etc.

**Aims**

The aim of this extended analysis is to better understand risk factors impacting response to BsAb and to determine long term outcomes for responding patients.

**Methods**

A retrospective analysis of anonymised consecutive patient data from 34 UK centres. Patients commenced glofit or epcor November 2023 to May 2025. Extended follow-up for responding patients was collected until February 2026.
Response assessments were locally reported. Toxicities were retrospectively graded according to CTCAE v5.0, CRS and ICANS were graded against ASTCT.
All patients commencing BsAb are included in safety and efficacy evaluable populations. Baseline data, safety and response rates were compared using chi-squared and Wilcoxon rank-sum tests. Duration of response (DoR) and duration of complete response (DoCR) were measured from date of best response. Progression-free survival (PFS) and overall survival (OS) were measured from date of first dose. DoR, DoCR, PFS and OS were assessed using Kaplan-Meier survival analysis. Multivariable analyses were performed to evaluate predictors of PFS and CR rate using Cox regression and logistic regression.

**Results**

This high-risk cohort comprised 332 patients (219 glofit, 113 epcor); there were clear differences in demographics for patients receiving either BsAB with the Epcor cohort having a higher proportion of ECOG PS 2 (40% vs 17%). Patients were heavily pre-treated and were enriched for high risk features; 152 (50%) had received prior CAR-T; 179 (55%) had primary refractory disease; 81 (25%) ECOG ≥2; 232 (78%) were retrospectively assessed as ineligible for the pivotal trials. Of 324 treated patients, 28% had CRS (grade 1/2 in 82/90). ORR and CR rate (CRR) were 43% and 24% respectively, while for patients meeting trial eligibility criteria, the CRR was 43%. At a median follow-up of 10.0 months (IQR 5.3-15.0), median progression-free survival (PFS) was 3.1 months (95% confidence interval [CI], 2.5-4.2), median overall survival (OS) was 6.9 months (95% CI, 4.9-10.8), outcomes for trial eligible patients were superior. A high proportion of patients did not start cycle 2 (16% glofit and 47% epcor), these patients had 6-month OS of 4% (95% CI 1-11%). Median DoCR follow up was 12 months; of 76 patients with CR, 7 have progressed, 5 have died without progression reported, the median DoCR is not reached and 1 year DoCR is 88% (95% CI 76-94) with similar DoCR for Glofit and Epcor.
Prior line of treatment refractoriness (HR 2.89, 95% CI 1.73-4.81, p=0.007), elevated LDH (HR 2.62, 95% CI 1.74-3.93. p=0.001), bendamustine exposure within 6 months (HR 1.62, 95% CI 1.14-2.30, p=0.007) and ECOG 1 (HR 2.70, 95% CI 1.52-4.79, p=0.001) or 2 (HR 6.49, 95% CI 3.47-12.01, p<0.001) were associated with inferior PFS.

**Summary/Conclusion**

This analysis is the largest real world data series on BsAbs to date. Our data demonstrates that both glofitamab and epcoritamab are effective treatment options in RR LBCL with updated data confirming durable complete remissions comparable to trial data in this high risk population. Some patients are unable to commence the 2$^{nd}$ cycle of treatment and have very poor outcomes. Identified risk factors for poor response should be used to improve patient selection and guide prognosis.

**Copyright Information:** (Online) ISSN: 2572-9241
©2026 The Author(s). HemaSphere is published by John Wiley & Sons Ltd on behalf of European Hematology Association. This is an open access Abstract Book distributed under the Attribution-NonCommercial-NoDerivs (CC BY-NC-ND), which allows third parties to download the articles and share them with others as long as they credit the author and the Abstract Book, but they cannot change the content in any way or use them commercially.
**Abstract Book Citations:** Authors, Title, HemaSphere, 2026;10:(S1):pages. The abstract book DOIs can be found on the "Author Information" page.
**Disclaimer:** Articles published in the journal HemaSphere exclusively reflect the opinions of the authors. The authors are responsible for all content in their abstracts including accuracy of the facts, statements, citing resources, etc.

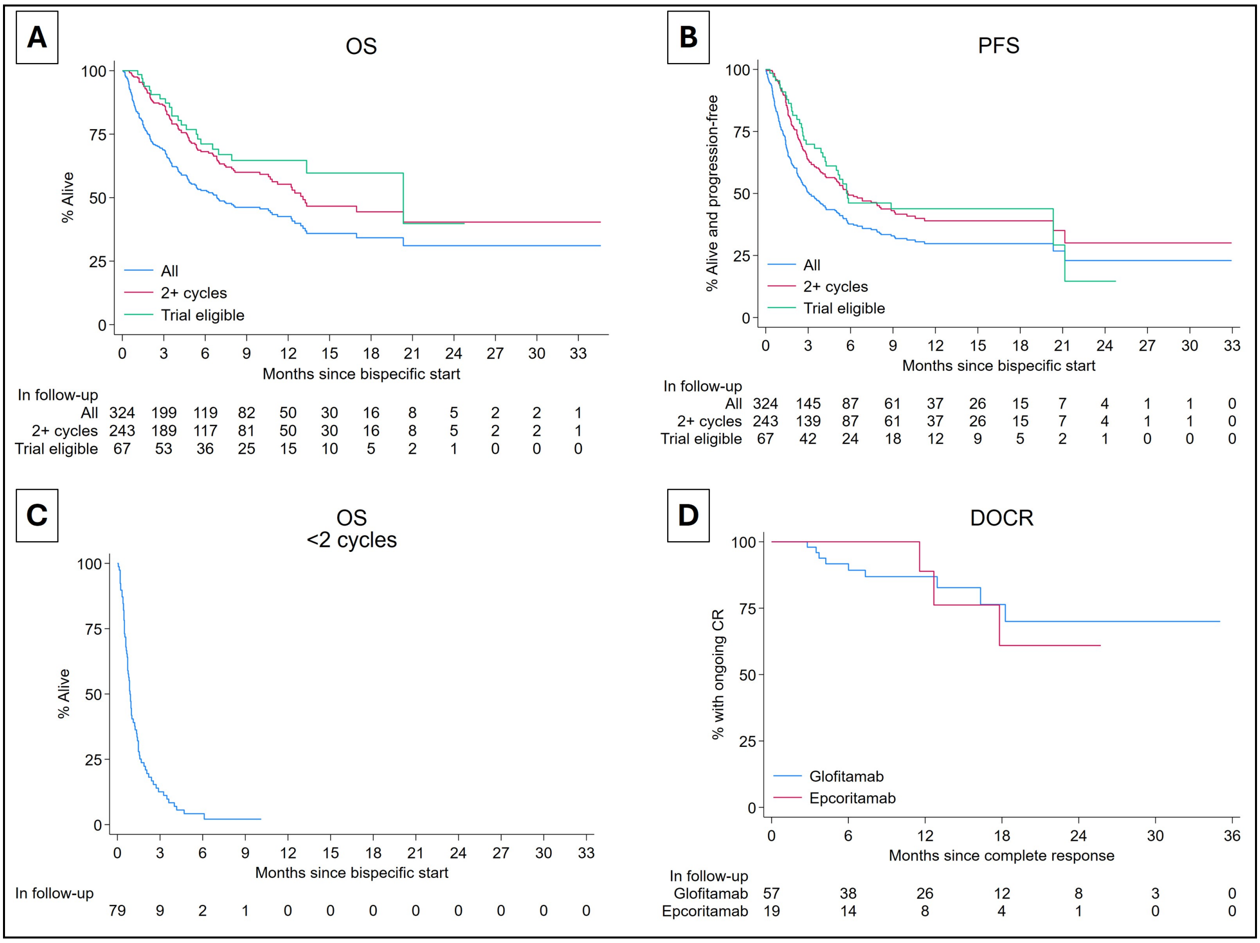

Figure legend: A and B OS (A)and PFS (B) for all patents, trial eligible patients and those receiving 2 or more cycles. C OS for patients who did not commence 2nd cycle of treatment. D DoCR

**Copyright Information:** (Online) ISSN: 2572-9241
©2026 The Author(s). HemaSphere is published by John Wiley & Sons Ltd on behalf of European Hematology Association. This is an open access Abstract Book distributed under the Attribution-NonCommercial-NoDerivs (CC BY-NC-ND), which allows third parties to download the articles and share them with others as long as they credit the author and the Abstract Book, but they cannot change the content in any way or use them commercially.
**Abstract Book Citations:** Authors, Title, HemaSphere, 2026;10:(S1):pages. The abstract book DOIs can be found on the "Author Information" page.
**Disclaimer:** Articles published in the journal HemaSphere exclusively reflect the opinions of the authors. The authors are responsible for all content in their abstracts including accuracy of the facts, statements, citing resources, etc.

### (S241) COMPREHENSIVE ANALYSIS OF BRIDGING TO CAR T-CELL THERAPY IN LARGE B-CELL LYMPHOMA WITH CONVENTIONAL TREATMENT OR CD3XCD20 BISPECIFIC ANTIBODIES

**Topic:** 19. Large B cell lymphomas - Clinical

Marie Anne-Catherine Neumann[1, 2, 3], Hanna Katharina Zieger[4], Ruth Fluemann*[1, 2, 5], Noëlle Sieg[1, 2, 3, 5], Anna Lütz[1, 5], Julia K. Schleifenbaum[1, 2], Sebastian Theobald[5], Martin Thelen[6, 7], Conrad-Amadeus Voltin[8], Justin Ferdinandus[1, 2], Marcel Teichert[4], Keven Hoerster[4], Vanessa Ridder[4], Evgenii Shumilov[9], Julia Katharina Scholz[10], Henriette Huber[11, 12], Rebecca Wurm-Kuczera *[13], Farina Eigendorff[14], Florian Eisele[15], Giulia Magno[16, 17], Philipp Schommers[1], Max Szameitat[1], Jessica Schneider[1], Silvia Seghezzi[3], David Stahl[1, 2], Paul J. Bröckelmann[1, 2, 18, 19], Daniel Bachurski[1, 2], Nadine Kutsch[1, 2], Philipp Gödel[1, 2], Joern Meinel[20], Johannes Rosenbrock[21], Ismini Halmer[1], Moritz Reese[1, 2, 19], Moritz Graef[1, 2, 19], Hans C. Reinhardt[4], Georg Lenz[9], Fabian Müller[10], Björn Chapuy[13], Ulf Schnetzke[14], Max Topp[15], Veit Bücklein[16], Dennis A. Eichenauer[1], Sven Borchmann[1, 2, 18], Jan-Michel Heger[1, 2], Peter Borchmann[1, 2, 18], Ron Jachimowicz[1, 2, 19], Bastian von Tresckow[4]

*[1]Department I of Internal Medicine, Center for Integrated Oncology Aachen Bonn Cologne Düsseldorf, University of Cologne, Medical Faculty and University Hospital Cologne, Cologne, Germany; [2]Cologne Lymphoma Working Group (CLWG), Cologne, Germany; [3]University of Cologne, Faculty of Medicine and University Hospital Cologne, Institute for Metabolomics in Ageing, Cluster of Excellence Cellular Stress Responses in Aging-associated Diseases (CECAD), Cologne, Germany; [4]Department of Hematology and Stem Cell Transplantation, West German Cancer Center and German Cancer consortium (DKTK partner site Essen), University Hospital Essen, Essen, Germany; [5]Institute for Molecular Immunology, University of Cologne, Cologne, Germany; [6]Department of General, Visceral, Thoracic, and Transplantation Surgery, University of Cologne, Faculty of Medicine and University Hospital Cologne, Cologne, Germany; [7]Center for Molecular Medicine Cologne, University of Cologne, Faculty of Medicine and University Hospital Cologne, Cologne, Germany; [8]Department of Nuclear Medicine, Faculty of Medicine and University Hospital Cologne, University of Cologne, Cologne, Germany; [9]Department of Medicine A, Hematology, Oncology and Pneumology, University Hospital Muenster, Muenster, Germany; [10]Department of Medicine 5 - Haematology and Oncology University Hospital Erlangen, Erlangen, Germany; [11]Städtisches Klinikum Karlsruhe, Karlsruhe, Germany; [12]Department of Internal Medicine III, Division of CLL, Ulm University, Ulm, Germany; [13]Department of Hematology, Oncology and Cancer Immunology Charité Charité - Universitätsmedizin Berlin Campus Benjamin Franklin, Berlin, Germany; [14]Klinik für Innere Medizin II, Abteilung Hämatologie und Internistische Onkologie, Universitätsklinikum Jena, Jena, Germany; [15]Universitätsklinikum Würzburg, Medizinische Klinik und Poliklinik II, Würzburg, Germany; [16]LMU University Hospital, Department of Medicine III, Hematology/Oncology, Munich, Germany; [17]German Cancer Consortium (DKTK), Partner Site Munich, Munich, Germany; [18]German Hodgkin Study Group (GHSG), Cologne, Germany; [19]Max Planck Institute for Biology of Ageing, Cologne, Germany; [20]Institute of Pathology, University of Cologne, Faculty of Medicine and University Hospital Cologne, Cologne, Germany; [21]Department of Radiation Oncology and Cyberknife Center, Faculty of Medicine and University Hospital Cologne, University of Cologne, Cologne, Germany;*

### Background

Bispecific antibodies (bABs) are used as holding or bridging prior to CAR T-cell treatment. However, the impact of this strategy on CAR T-cell safety and efficacy remains unclear. While T-cell redirecting therapies may adversely affect T-cell fitness, other preclinical data suggest synergistic effects.

### Aims

This study aimed to evaluate safety and efficacy of bAB-based holding/bridging with epcoritamab or glofitamab compared to conventional chemotherapy/no bridging prior CAR T-cell therapy in relapsed/refractory large B-cell lymphoma (r/r LBCL), including an analysis of immune- and CAR T-cell subsets.

**Copyright Information:** (Online) ISSN: 2572-9241
©2026 The Author(s). HemaSphere is published by John Wiley & Sons Ltd on behalf of European Hematology Association. This is an open access Abstract Book distributed under the Attribution-NonCommercial-NoDerivs (CC BY-NC-ND), which allows third parties to download the articles and share them with others as long as they credit the author and the Abstract Book, but they cannot change the content in any way or use them commercially.
**Abstract Book Citations:** Authors, Title, HemaSphere, 2026;10:(S1):pages. The abstract book DOIs can be found on the "Author Information" page.
**Disclaimer:** Articles published in the journal HemaSphere exclusively reflect the opinions of the authors. The authors are responsible for all content in their abstracts including accuracy of the facts, statements, citing resources, etc.

### Methods

We assessed patient characteristics, prior lines of treatment, progression-free (PFS) and overall survival (OS), and safety outcomes of bAb-based and conventional holding/bridging and subsequent CAR T-cell therapy, including but not limited to cytokine release syndrome (CRS) and immune effector cell-associated neurotoxicity syndrome (ICANS). In a subset of patients, detailed flow-cytometric analysis of peripheral blood mononuclear cells (PBMCs) before and after bridging and after CAR T-cell infusion was performed. CAR-copies were assessed via PCR.

### Results

A total of 112 patients with r/r LBCL from nine German centers were included, comprising 67 patients with bAbs-holding/bridging prior to CAR T-cell therapy (BisCAR, 47% female, median age at CAR T-cell infusion 66 years [range 25-85]), and 45 patients who received no or conventional bridging prior to CAR T-cell therapy (ConCAR, 33% female, median age 60 years [range 24-84]). Patients in the BisCAR cohort had a significantly lower risk of severe cytopenia during holding/bridging (3.0% vs. 17.8% cytopenia grade III-IV; p=0.0082), whereas post CAR T-cell therapy toxicity rates and management were comparable (CRS 71.5% vs 73%, p=1.0, ICANS 26.9% vs 28.9%, p=0.832) between cohorts. Treatment-related mortality was 3.0% in the BisCAR cohort and 8.9% in the ConCAR cohort.
Overall response rates (ORR) after bridging and at month 1 following CAR T-cell therapy trended higher in BisCAR versus ConCAR (70.1% vs 61.1% and 80.6% vs 72.1%, respectively), without reaching statistical significance (Fig. 1A).
Glofitamab-based bridging was associated with transient immune activation and proliferation, resulting in enhanced CAR T-cell activation without signs of terminal exhaustion: BisCAR exhibited higher $CD25^+$ CAR T-cell mean fluorescence intensity (MFI) compared with ConCAR (p=0.023), while $PD\text{-}1^+TIGIT^+$ CAR T-cell frequencies were comparable between groups (p=0.350) (Fig. 1B). Early CAR T-cell activation correlated with both response and toxicity.

### Summary/Conclusion

Our data shows that bAB-based bridging prior to CAR T-cell therapy is feasible, safe and effective in r/r LBCL. Furthermore, bAb-based bridging demonstrated superior efficacy with a favorable toxicity profile compared with conventional approaches. Interestingly, our analyses show glofitamab-induced transient immune-cell activation without evidence of negative or detrimental effects on CAR T-cell function and expansion. In summary, our findings support bAb bridging as a promising strategy.

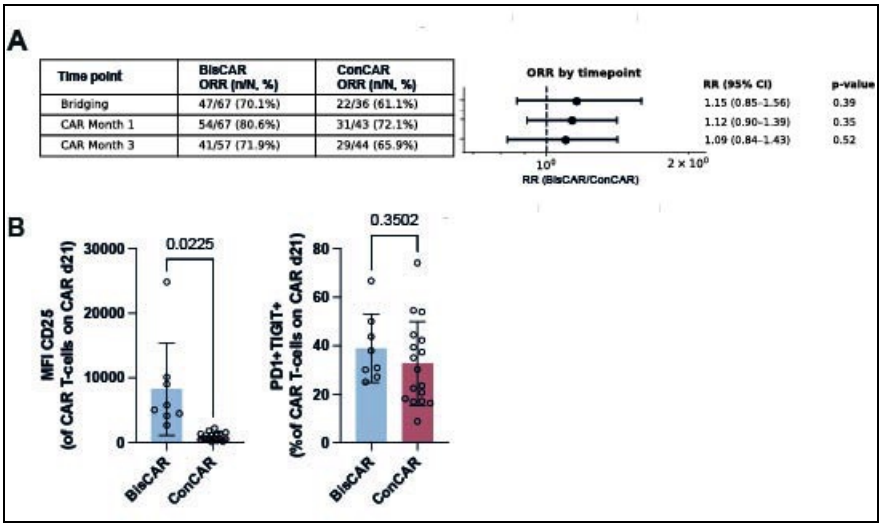

Copyright Information: (Online) ISSN: 2572-9241
©2026 The Author(s). HemaSphere is published by John Wiley & Sons Ltd on behalf of European Hematology Association. This is an open access Abstract Book distributed under the Attribution-NonCommercial-NoDerivs (CC BY-NC-ND), which allows third parties to download the articles and share them with others as long as they credit the author and the Abstract Book, but they cannot change the content in any way or use them commercially.
Abstract Book Citations: Authors, Title, HemaSphere, 2026;10:(S1):pages. The abstract book DOIs can be found on the "Author Information" page.
Disclaimer: Articles published in the journal HemaSphere exclusively reflect the opinions of the authors. The authors are responsible for all content in their abstracts including accuracy of the facts, statements, citing resources, etc.

### (S243) FAVORABLE REAL-WORLD OUTCOMES AFTER CD19 CAR T CELL TREATMENT IN RELAPSED/REFRACTORY HIGH-GRADE B-CELL LYMPHOMAS (HGBL): AN EBMT LWP ANALYSIS

**Topic:** 19. Large B cell lymphomas - Clinical

Igor Age Kos*[1], Maud Ngoya[2], Michael Northend[3], Robin Sanderson[4], Ibrahim Yakoub-Agha[5], Sridhar Chaganti[6], Ron Ram[7], Mi Kwon[8, 9, 10], Anne Sirvent[11], Christof Scheid[12], Urban Novak[13], Peter Dreger[14], Edouard Forcade[15], Didier Blaise[16], Eleni Tholouli[17], Linde Morsink[18], Francesca Bonifazi[19], Igor Wolfgang Blau[20], Rajesh Alajangi[21], Pere Barba[22], Matthew Collin[23], Anne Huynh[24], Philipp Staber[1], Bertram Glass[25], Catherine Thieblemont[26], Anna Ossami Saidy[25], Rémy Dulery[27], Lorenz Thurner[1], Ali Bazarbachi[28]

*[1]University of Saarland, Hematology, Oncology, Rheumatology and Clinical Immunology, Homburg, Germany; [2]European Society for Blood and Marrow Transplantation, Paris, France; [3]University College London Hospital, London, United Kingdom; [4]Kings College Hospital London, London, United Kingdom; [5]CHU de Lille, Lille, France; [6]Birmingham Centre for Cellular Therapy and Transplat (BCCTT), Birmingham, United Kingdom; [7]Tel Aviv Sourasky Medical Center, Tel Aviv, Israel; [8]Department of Hematology, Gregorio Marañón General University Hospital., Madrid, Spain; [9]Faculty of Medicine, Complitense University of Madrid, Madrid, Spain; [10]Gregorio Marañón Health Research Institute, Madrid, Spain; [11]CHU Montpellier, Montpellier, France; [12]University of Cologne, Cologne, Germany; [13]Medical Oncology, Inselspital, Bern University Hospital and University of Bern, Bern, Switzerland; [14]University of Heidelberg, Heidelberg, Germany; [15]CHU Bordeaux, Bordeaux, France; [16]Institut Paoli Calmettes, Marseille, France; [17]Manchester Royal Infirmary, Manchester, United Kingdom; [18]University Medical Center Groningen (UMCG), Groningen, The Netherlands; [19]Bologna University, S.Orsola-Malpighi Hospital, Bologna, Italy; [20]Medizinische Klinik M. S. Hamatologie, Onkologie und Tumorimmunologie, University Hospital Charité, Berlin, Germany; [21]University Hospitals Bristol and Weston NHSFT, Bristol, United Kingdom; [22]Vall Hebron University Hospital, Barcelona, Spain; [23]RVI Newcastle, Newcastle upon Tyne, United Kingdom; [24]CHU - Institute Universitaire du Cancer Toulouse, Toulouse, France; [25]Department of Haematology and Cell Therapy, Helios Clinic Berlin-Buch, Berlin, Germany; [26]Assistance Publique & Hôpitaux de Paris (APHP), Hôpital Saint-Louis, Hémato-oncologie, Université de Paris, Paris, France; [27]Sorbonne University, Department of Clinical Hematology and Cellular Therapy, Hôpital Saint-Antoine, Assistance Publique – Hôpitaux de Paris (AP-HP),, Paris, France; [28]Department of Internal Medicine, American University of Beirut, Beirut, Lebanon;*

### Background

High-grade B cell lymphoma (HGBL) not otherwise specified (NOS) and HGBL with MYC and BCL2 rearrangements (HGBL-MYC/BCL2) are considered distinct entities in the WHO 5th edition classification. Both represent difficult-to-treat subtypes, particularly in the relapsed/refractory (R/R) setting. As the standardized diagnostic category “HGBL with MYC and BCL2 rearrangements” is relatively new, precise outcome data remain limited, as many reports rely on broader definitions. As these entities can also be underrepresented in pivotal trials, defining real-world, entity-specific outcomes after CD19 CAR T cell treatment is essential.

### Aims

Describe real-world (RW), entity-specific outcomes after CD19 CAR T-cell therapy in HGBL-NOS and HGBL-MYC/BCL2 and compare them with the outcomes of a comprehensive diffuse large B cell lymphoma (DLBCL)-NOS EBMT registry cohort.

### Methods

Adult patients (pts) diagnosed with HGBL-NOS or HGBL-MYC/BCL2, or DLBCL-NOS who received CD19 CAR T therapy between 2018 and 2023 and were reported to the EBMT registry were included.

**Copyright Information:** (Online) ISSN: 2572-9241
©2026 The Author(s). HemaSphere is published by John Wiley & Sons Ltd on behalf of European Hematology Association. This is an open access Abstract Book distributed under the Attribution-NonCommercial-NoDerivs (CC BY-NC-ND), which allows third parties to download the articles and share them with others as long as they credit the author and the Abstract Book, but they cannot change the content in any way or use them commercially.
**Abstract Book Citations:** Authors, Title, HemaSphere, 2026;10:(S1):pages. The abstract book DOIs can be found on the “Author Information” page.
**Disclaimer:** Articles published in the journal HemaSphere exclusively reflect the opinions of the authors. The authors are responsible for all content in their abstracts including accuracy of the facts, statements, citing resources, etc.

**Results**

In total 5298 pts were included: 5066 DLBCL-NOS, 177 HGBL-MYC/BCL2, and 55 HGBL-NOS. Median age was 62.6 years (y) and did not differ between groups (p=0.51). Median time from diagnosis to CAR T was shorter in both HGBL cohorts (10.4 months (mo) for MYC/BCL2; 10.9 mo for NOS) compared with DLBCL-NOS (15.3 mo; p<0.001). Treatment within 10 months of diagnosis was more frequent in HGBL (46.9% MYC/BCL2; 40.0% HGBL-NOS) than in DLBCL-NOS (25.4%). Median treatment year was 2022 for DLBCL-NOS and HGBL-NOS, and 2023 for HGBL-MYC/BCL2. Stage IV disease at treatment was more frequent in HGBL-MYC/BCL2 (74.1%) than in DLBCL-NOS (58.7%) and HGBL-NOS (54.2%) (p=0.0048). Overall, 67.5% had R/R disease at CAR T infusion; 9.6% were in CR and 22.9% in PR, without significant differences between groups. Most patients received Axi-cel (66.4%) followed by Tisa-cel (29.3%). Prior autologous transplantation was more common in DLBCL-NOS (21.5%) than in HGBL-MYC/BCL2 (7.0%) and HGBL-NOS (10.0%) (p=0.0001). With a median follow-up of 24 mo for DLBCL-NOS and 21.6 mo for both HGBL cohorts, 2y PFS was 42.8% (95% CI 41.1–44.4) for DLBCL-NOS, 42.7% (95% CI 33–52) for HGBL-MYC/BCL2, and 46.0% (95% CI 31–59.8) for HGBL-NOS, while 2y OS was 51.9% (95% CI 50.2–53.5), 49.0% (95% CI 39.2–58.1), and 51.0% (95% CI 34.9–65.1), respectively. At 2 years, the overall cumulative relapse incidence was 45.1% (95% CI 43.5–46.7), and the 2y non-relapse mortality was 12.1% (95% CI 11.1–13.3), with no significant differences between cohorts.

**Summary/Conclusion**

In this large EBMT RW cohort, pts with HGBL-NOS and HGBL-MYC/BCL2 achieved promising PFS, despite a high-risk clinical profile at infusion. The results indicate that CD19 CAR T cell therapy may mitigate the adverse biology of HGBL in the R/R setting. Despite its limitations, including the retrospective design, absence of central pathology review, and potential under-reporting of MYC/BCL2-rearranged cases, these findings provide contemporary, entity-specific benchmarks and support CD19 CAR T as a very relevant therapeutic option for R/R HGBL, informing treatment sequencing and future study design.

**Copyright Information:** (Online) ISSN: 2572-9241
©2026 The Author(s). HemaSphere is published by John Wiley & Sons Ltd on behalf of European Hematology Association. This is an open access Abstract Book distributed under the Attribution-NonCommercial-NoDerivs (CC BY-NC-ND), which allows third parties to download the articles and share them with others as long as they credit the author and the Abstract Book, but they cannot change the content in any way or use them commercially.
**Abstract Book Citations:** Authors, Title, HemaSphere, 2026;10:(S1):pages. The abstract book DOIs can be found on the “Author Information” page.
**Disclaimer:** Articles published in the journal HemaSphere exclusively reflect the opinions of the authors. The authors are responsible for all content in their abstracts including accuracy of the facts, statements, citing resources, etc.

### (S244) TUMOUR MICROENVIRONMENT ARCHETYPES THAT SUPPORT ANTI-TUMOUR T-CELL IMMUNITY AND CD4+ T-CELL CYTOTOXIC DIFFERENTIATION UNDERPIN RESPONSE TO GLOFITAMAB IN DIFFUSE LARGE B-CELL LYMPHOMA

**Topic:** 21. Lymphoma biology & translational research

Thiago M Steiner*[1, 2], Bao Le[1], Sean Harrop[1], Stefanie Navaratnam[1], Faiza Zuairia Tabassum[1], Ruiqing Zhu[1], Lucas Tobar[1], Sean MacDonald[1], Lei Shong Lau[1], Tristan Molden-Hauer[1], Marina Bacac[3], Vivien R. Sutton[1, 2], Joseph A. Trapani[1, 2], Niko Thio[1, 2], Adrian Minson[1, 2], Michael Dickinson[1, 2], Paul Neeson[1, 2]

*[1]Peter MacCallum Cancer Centre, Melbourne, Australia; [2]University of Melbourne, Sir Peter MacCallum Department of Oncology, Melbourne, Australia; [3]Roche Innovation Center Zurich, Roche Pharma Research and Early Development, Schlieren, Switzerland;*

### Background

Glofitamab, CD20×CD3 T-cell engager (TCE), redirects T cells to kill tumour cells. We showed that combining glofitamab with standard first-line (1L) chemotherapy (RCHOP/Pola-R-CHP) in DLBCL produced a historically high rate of complete remissions, which were durable in an ultra-high-burden population [COALITION-study (NCT04914741/Minson et al., JCO-2025)]. Despite overall treatment success, some patients relapsed or were refractory (10%). Understanding the mechanisms underlying resistance will inform ongoing 1L TCE development and may stem from tumour intrinsic, microenvironmental (TME) factors, or T-cell-intrinsic dysfunction.

### Aims

To elucidate the TME and T-cell-intrinsic mechanisms that limit therapeutic response to glofitamab in first-line DLBCL.

### Methods

Baseline and progression tumour biopsies from COALITION trial patients were analysed using bulk RNA seq and mIHC. In parallel, we performed scRNA-seq and spectral flow cytometry on longitudinal COALITION blood samples collected pre- and post-TCE treatment. To further interrogate observations from patient samples, we established in vitro co-cultures comprising DLBCL tumour cells, donor- or COALITION patient-derived T cells, and glofitamab. Co-cultured cells were subsequently profiled using scRNA-seq, flow cytometry, spatial proteomics (Pixelgen), and live-cell imaging

**Copyright Information:** (Online) ISSN: 2572-9241
©2026 The Author(s). HemaSphere is published by John Wiley & Sons Ltd on behalf of European Hematology Association. This is an open access Abstract Book distributed under the Attribution-NonCommercial-NoDerivs (CC BY-NC-ND), which allows third parties to download the articles and share them with others as long as they credit the author and the Abstract Book, but they cannot change the content in any way or use them commercially.
**Abstract Book Citations:** Authors, Title, HemaSphere, 2026;10:(S1):pages. The abstract book DOIs can be found on the “Author Information” page.
**Disclaimer:** Articles published in the journal HemaSphere exclusively reflect the opinions of the authors. The authors are responsible for all content in their abstracts including accuracy of the facts, statements, citing resources, etc.

**Results**

Our results revealed that TME of responders contained optimal signalling for T-cell responses (IFNγ and IL-2-STAT5) when compared to non-responders (NR), progressive disease or relapse ≤6 months post-treatment. Responders, independently of polatuzumab addition, showed increased tissue remodelling and enriched archetypes containing CD4/CD8 T-cells–tumour interactions. scRNA-seq and flow cytometry on longitudinal blood samples demonstrated that T-cells expanded robustly following glofitamab therapy. Interestingly, two populations of proliferating CD4-T cells emerged, containing upregulated expression of cytokine-related genes (IL2, IFNγ), consistent with a helper phenotype. One cluster displayed a cytotoxic (CTL) signature (*PRF1, GZMB, TBX21, RUNX3, EOMES, NKG7).*
Surprisingly, in vitro co-culture data indicate that CD4 T cells contribute to TCE-mediated responses by both supporting CD8 T-cell activity and acquiring cytotoxic function. Upon engagement, CD4 T cells upregulated canonical cytotoxic markers (GzmB, Runx3, Eomes, T-bet) and killed tumour cells via a perforin-GzmB pathway. Notably, the killing capacity of both naïve and antigen-experienced CD4 T cells was comparable to that of CD8 T cells, and these CD4-derived CTLs maintained a cytotoxic phenotype for at least seven days.
Cell interactome analysis using single-cell data from co-cultured cells, Pixelgen and blocking antibodies revealed the contribution of MHCII, adhesion molecules (ICAM1 and CD58) and IL2 for efficient CD4-CTL differentiation when CD20 expression on the tumour cells was low.

Bulk RNA-seq on COALITION samples showed that MHCII, CD2, ICOS and CD6 were already downregulated in the TME of NR at baseline. Moreover, expression of these molecules was further downregulated in NR at progression, suggesting a potential role in immune escape.

**Summary/Conclusion**

Overall, our results indicate that a pre-treatment TME that supports productive T-cell engagement with tumour cells, and differentiation of CD4-CTLs, underpins successful responses to glofitamab. We also identify downregulation of CD20, alongside adhesion molecules, and co-stimulatory pathways as potential immune-escape mechanisms.

**Copyright Information:** (Online) ISSN: 2572-9241
©2026 The Author(s). HemaSphere is published by John Wiley & Sons Ltd on behalf of European Hematology Association. This is an open access Abstract Book distributed under the Attribution-NonCommercial-NoDerivs (CC BY-NC-ND), which allows third parties to download the articles and share them with others as long as they credit the author and the Abstract Book, but they cannot change the content in any way or use them commercially.
**Abstract Book Citations:** Authors, Title, HemaSphere, 2026;10:(S1):pages. The abstract book DOIs can be found on the "Author Information" page.
**Disclaimer:** Articles published in the journal HemaSphere exclusively reflect the opinions of the authors. The authors are responsible for all content in their abstracts including accuracy of the facts, statements, citing resources, etc.

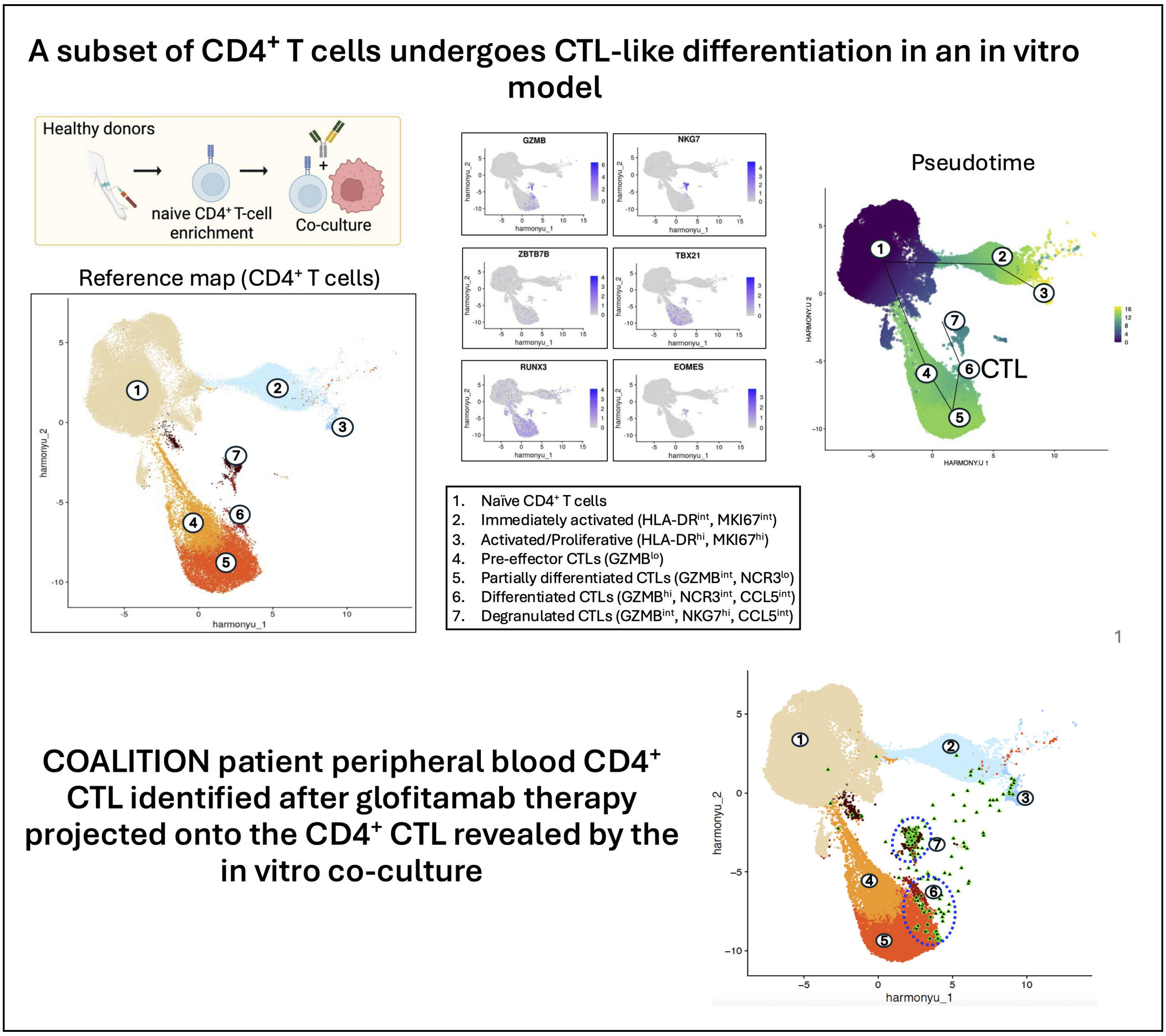

Top: Naïve CD4+ T cells were co-cultured with the DLBCL cell line MD901 in the presence of glofitamab. After 24 hours, scRNA-seq was performed on naïve CD4+ T cells alone or from the co-cultures. UMAP and pseudotime analyses demonstrate the differentiation trajectory of naïve CD4+ T cells toward a cytotoxic phenotype. Feature plots confirm the upregulation of genes associated with CD4+ T-cell cytotoxicity. Bottom: Cytotoxic CD4+ T cells identified in COALITION patients after glofitamab therapy (green triangles) were projected onto the co-culture UMAP. The projection shows that patient-derived CTLs cluster with the differentiated and degranulated CTLs from the co-culture assay.

**Copyright Information:** (Online) ISSN: 2572-9241
©2026 The Author(s). HemaSphere is published by John Wiley & Sons Ltd on behalf of European Hematology Association. This is an open access Abstract Book distributed under the Attribution-NonCommercial-NoDerivs (CC BY-NC-ND), which allows third parties to download the articles and share them with others as long as they credit the author and the Abstract Book, but they cannot change the content in any way or use them commercially.
**Abstract Book Citations:** Authors, Title, HemaSphere, 2026;10:(S1):pages. The abstract book DOIs can be found on the “Author Information” page.
**Disclaimer:** Articles published in the journal HemaSphere exclusively reflect the opinions of the authors. The authors are responsible for all content in their abstracts including accuracy of the facts, statements, citing resources, etc.

### (S245) VT-EBV-N AS POST-REMISSION THERAPY SIGNIFICANTLY IMPROVES DISEASE-FREE SURVIVAL IN EBV-POSITIVE EXTRANODAL NK/T-CELL LYMPHOMA: A RANDOMIZED, DOUBLE-BLIND PHASE 2 TRIAL

**Topic:** 20. Other aggressive lymphomas

Youngwoo Jeon[1], Seok Goo Cho[2], Deok-Hwan Yang*[3]

*[1]Yeouido ST. Mary Hospital, Catholic Hematology Hospital, Catholic University of Korea, Seoul, South Korea;[2]Seoul ST. Mary hosptial, Catholic Hematology Hospital, Catholic University of Korea, Seoul, South Korea; [3]Chonnam National University Medical School, Department of Hematology-Oncology, Hwasun, South Korea;*

### Background

Extranodal NK/T-cell lymphoma (ENKL) is an aggressive Epstein–Barr virus (EBV)-associated lymphoma with a substantial risk of relapse despite achieving complete remission (CR) after intensive therapy. No standard post-remission treatment has been established. VT-EBV-N is an autologous EBV-specific cytotoxic T lymphocyte therapy designed to eliminate residual EBV-infected malignant cells.

### Aims

To evaluate the efficacy and safety of VT-EBV-N as post-remission therapy in patients with EBV-positive ENKL in complete remission.

### Methods

This randomized, double-blind, placebo-controlled phase 2 trial enrolled patients with EBV-positive ENKL who achieved complete remission within 6 months prior to enrollment and met protocol-defined eligibility criteria. Patients were randomized (1:1) to receive VT-EBV-N or autologous peripheral blood mononuclear cells (PBMC) as control. Treatment was administered intravenously once weekly for 4 weeks, followed by a 4-week rest period, and then once weekly for an additional 4 weeks (total of 8 doses).
The primary endpoint was 2-year disease-free survival (DFS). Secondary endpoints included overall survival (OS) and safety. Efficacy was analyzed in the full analysis set. The study was conducted in accordance with the Declaration of Helsinki, and all patients provided written informed consent.

### Results

A total of 50 patients were randomized, and 46 were included in the full analysis set (VT-EBV-N, n=21; control, n=25). Baseline demographic and clinical characteristics were well balanced between groups. At 2 years, VT-EBV-N demonstrated a statistically significant improvement in DFS compared with control (95.0% vs 77.6%; stratified log-rank p=0.0347). DFS events occurred in 1/21 (4.8%) patients in the VT-EBV-N group and 8/25 (32.0%) in the control group.
No deaths occurred in the VT-EBV-N group, whereas 4 deaths (16.0%) were observed in the control group (p=0.0580).
VT-EBV-N was generally well tolerated. Grade ≥3 adverse events occurred in 2/21 (9.5%) patients in the VT-EBV-N group and 4/25 (16.0%) in the control group. No treatment-related deaths were reported.

### Summary/Conclusion

In this randomized phase 2 trial, VT-EBV-N significantly improved 2-year disease-free survival with a favorable safety profile in patients with EBV-positive ENKL in complete remission. These findings provide randomized clinical evidence supporting VT-EBV-N as an effective post-remission strategy in this rare and aggressive lymphoma.

**Copyright Information:** (Online) ISSN: 2572-9241
©2026 The Author(s). HemaSphere is published by John Wiley & Sons Ltd on behalf of European Hematology Association. This is an open access Abstract Book distributed under the Attribution-NonCommercial-NoDerivs (CC BY-NC-ND), which allows third parties to download the articles and share them with others as long as they credit the author and the Abstract Book, but they cannot change the content in any way or use them commercially.
**Abstract Book Citations:** Authors, Title, HemaSphere, 2026;10:(S1):pages. The abstract book DOIs can be found on the "Author Information" page.
**Disclaimer:** Articles published in the journal HemaSphere exclusively reflect the opinions of the authors. The authors are responsible for all content in their abstracts including accuracy of the facts, statements, citing resources, etc.

### (S246) A PHASE I STUDY OF AUTOLOGOUS DUAL-EPITOPE NANOBODY-BASED ANTI-CD5 CAR T CELLS IN RELAPSED/REFRACTORY T-ALL/LBL AND PTCL/CTCL

**Topic:** 20. Other aggressive lymphomas

Ming-Kun Wang*[1], Meng Lv[1], Lin Wang[2], Na Kuang[2], Ting Zhao[1], Ying Wu[1], Xuying Pei[1], Xiao-Dong Mo[1], Yuqian Sun[1], Xiaohui Zhang[1], Xiao-Jun Huang[1], Xiangyu Zhao[1]

*[1]Peking University People's Hosptial, Beijing, China; [2]Hebei Senlang Biotechnology Co, Ltd, Shijiazhuang, China;*

### Background

Patients with relapsed or refractory T-lymphoblastic leukemia/lymphoma(T-ALL/LBL) and peripheral T-cell lymphoma (PTCL) have dismal outcomes. We previously demonstrated that a naturally selected anti-CD7 CAR-T platform can overcome fratricide without gene editing or protein blockade (Blood 2022; 2025). CD5 is a pan–T-cell antigen, particularly in cutaneous T-cell lymphoma (CTCL); however, effective CD5-directed cellular therapies remain limited.

### Aims

This first-in-human phase I study (NCT06874946) evaluated the safety, preliminary efficacy, and recommended phase II dose (RP2D) of autologous CD5-directed CAR-T cells (SL105) in patients with R/R T-ALL/PTCL.

### Methods

Autologous CD5-directed CAR-T cells incorporating a tandem camelid-derived dual-epitope nanobody were generated using a natural selection platform to mitigate fratricide. Following fludarabine/cyclophosphamide lymphodepletion, patients were treated in a 3+3 dose-escalation phase I design (0.5, 1.0, and 2.0 $\times 10^6$ cells/kg). Single-cell RNA sequencing of peripheral blood at 1–3 months post-infusion was performed to characterize immune reconstitution and its association with viral complications.

### Results

As of March 1, 2026, 22 patients had received SL105 and completed the 28-day DLT assessment. The median age was 37 years (range, 16–57), and 22.7% had prior HSCT. Diagnoses included T-ALL/LBL (n=11) and PTCL (n=11), including mycosis fungoides/Sézary syndrome (n=3) and other CTCL (n=2). Median follow-up was 6 months (range, 1–11). No dose-limiting toxicities were observed, and the RP2D was determined as 2.0 $\times 10^6$ CAR-T cells/kg. Median time to peak CAR-T expansion was 14 days by qPCR, with a median peak level of $6.3 \times 10^4$ copies/μg.
Grade 1 CRS occurred in 50% and grade 2 CRS in 22.7%; one patient experienced grade 1 ICANS. The best overall response rate (ORR) was 81.8%, including CRc/CMR in 54.5% and PR of extramedullary disease in 27.3%. At the RP2D level, ORR was 100%, with CR/CMR in 62.6%. Three patients with PR of extramedullary disease at M1 remain under follow-up, with ongoing responses. All responding patients with bone marrow involvement achieved MRD negativity by flow cytometry. All four non-responders exhibited low CAR-T expansion (peak <500 copies/μg). Five responders proceeded to consolidative allo-HSCT. EBV viremia or clinically diagnosed EBV-PTLD occurred in 9 patients (41%), and CMV viremia in 6 patients (27.3%).

Single-cell RNA sequencing of peripheral blood (M1–M3) from patients with viral infections revealed a time-dependent expansion of cytotoxic $CD56^{dim}$ $CD16^{hi}$ GZMH+ NK cells exclusively in the EBV-only group, whereas NK expansion was limited in CMV-only or EBV+CMV co-infection. Expanded NK cells showed stress-related transcriptional signatures without exhaustion features, suggesting virus-specific functional remodeling during T-cell reconstitution following CD5 CAR-T therapy.

**Copyright Information:** (Online) ISSN: 2572-9241
©2026 The Author(s). HemaSphere is published by John Wiley & Sons Ltd on behalf of European Hematology Association. This is an open access Abstract Book distributed under the Attribution-NonCommercial-NoDerivs (CC BY-NC-ND), which allows third parties to download the articles and share them with others as long as they credit the author and the Abstract Book, but they cannot change the content in any way or use them commercially.
**Abstract Book Citations:** Authors, Title, HemaSphere, 2026;10:(S1):pages. The abstract book DOIs can be found on the “Author Information” page.
**Disclaimer:** Articles published in the journal HemaSphere exclusively reflect the opinions of the authors. The authors are responsible for all content in their abstracts including accuracy of the facts, statements, citing resources, etc.

### Summary/Conclusion

SL105, a first-in-human dual-epitope nanobody CD5-directed CAR-T therapy, demonstrated a favorable safety profile and promising antitumor activity in R/R T-ALL/PTCL, including cutaneous T-cell lymphoma such as mycosis fungoides, a population with limited effective treatment options. The RP2D has been established, supporting further evaluation in an ongoing phase II study.

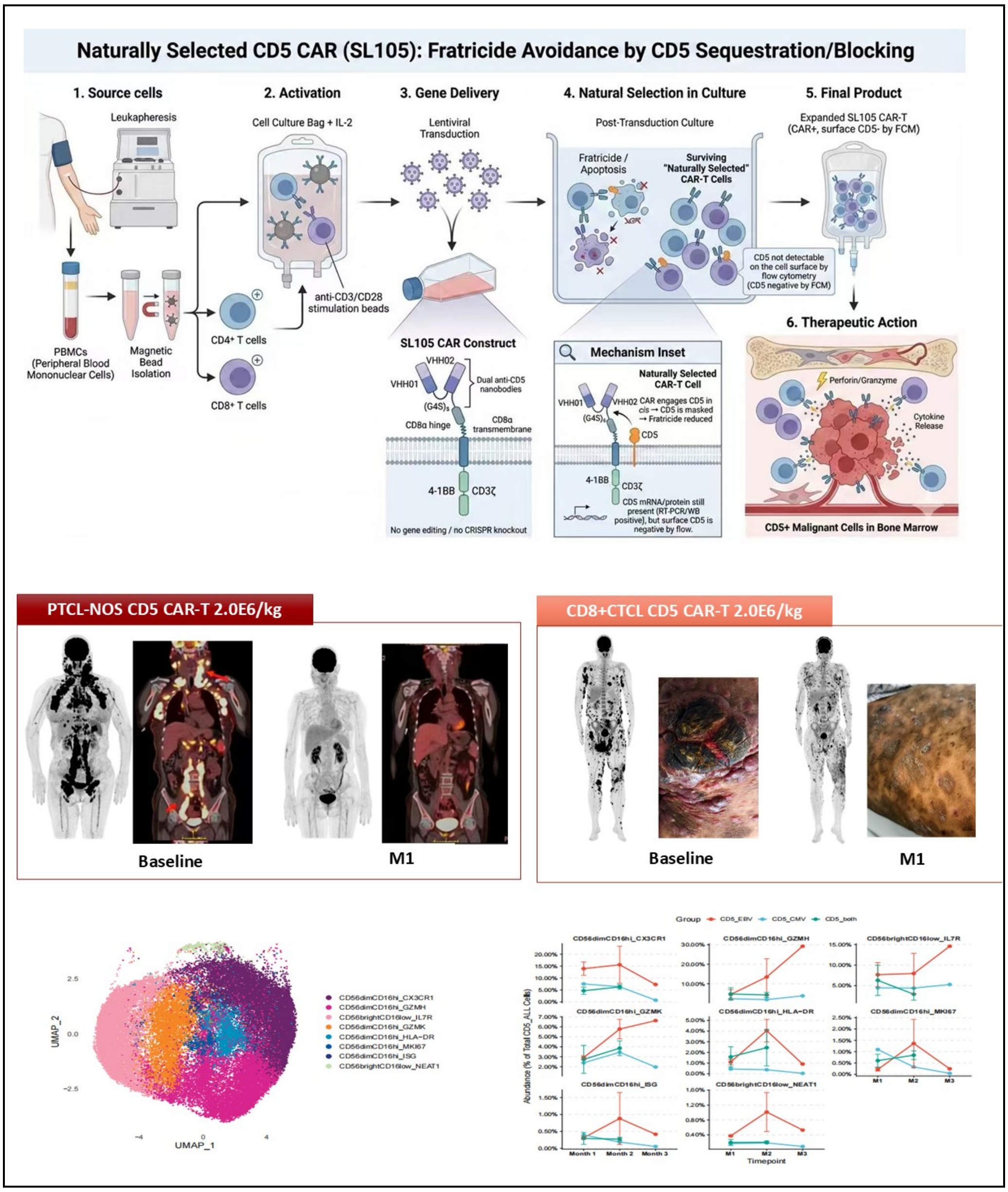

**Copyright Information:** (Online) ISSN: 2572-9241
©2026 The Author(s). HemaSphere is published by John Wiley & Sons Ltd on behalf of European Hematology Association. This is an open access Abstract Book distributed under the Attribution-NonCommercial-NoDerivs (CC BY-NC-ND), which allows third parties to download the articles and share them with others as long as they credit the author and the Abstract Book, but they cannot change the content in any way or use them commercially.
**Abstract Book Citations:** Authors, Title, HemaSphere, 2026;10:(S1):pages. The abstract book DOIs can be found on the "Author Information" page.
**Disclaimer:** Articles published in the journal HemaSphere exclusively reflect the opinions of the authors. The authors are responsible for all content in their abstracts including accuracy of the facts, statements, citing resources, etc.

### (S247) CLINICAL, GENETIC, AND IMMUNE MICROENVIRONMENTAL LANDSCAPES OF PRIMARY BREAST DIFFUSE LARGE B-CELL LYMPHOMA: A COMPARATIVE STUDY WITH NODAL AND IMMUNE-PRIVILEGED SITE DLBCL

**Topic:** 20. Other aggressive lymphomas

Junlei Jia*[1], Zheng Song[1], Yidan Zhang[1], Zechao Hu[1], Xianhuo Wang[1], Hui-Lai Zhang[1]

[1] *Tianjin Medical University Cancer Institute and Hospital, Department of Lymphoma, Tianjin, China;*

### Background

Diffuse large B-cell lymphoma (DLBCL) is an aggressive, heterogeneous malignancy arising in nodal or extranodal sites. Among these, primary breast DLBCL (PB-DLBCL) represents a distinct entity characterized by unique clinicopathological features and an increased risk of central nervous system recurrence. However, systematic studies are currently lacking due to its rarity.

### Aims

This study conducted an integrated analysis to elucidate the distinct biological characteristics of PB-DLBCL and delineate its similarities and differences compared to nodal and IP-DLBCL.

### Methods

This single-center study enrolled 703 DLBCL patients (134 PB, 363 nodal, and 206 IP), excluding those with secondary extranodal involvement. Baseline clinical characteristics and survival data were retrospectively collected. Inverse probability of treatment weighting (IPTW) was applied to mitigate potential confounders. We performed DNA (n=223; 63 PB, 127 nodal, 33 IP) and RNA sequencing (n=121; 58 PB, 48 nodal, 15 IP) on the DLBCL cohort. The LymphGen algorithm was applied to subcategorize patients based on their genetic alteration profiles. Microenvironmental signatures were identified using the Lymphoma Microenvironment (LME) classification. A novel immortalized PB-DLBCL cell line was established and is intended for subsequent mechanistic research and evaluation of therapeutic strategies.

### Results

Among all 134 patients with PB-DLBCL, the median age at diagnosis was 57 years (range, 21-89 years). seventy-one cases (53.0%) were considered non-GCB phenotype and 35 cases (26.1%) GCB. The bilateral breast involvement occurred in 6 patients (4.4%), and ki-67 >80% was observed in 85 (63.4%) patients. Following IPTW adjustment, IP-DLBCL exhibited the poorest survival outcomes. Notably, PB-DLBCL showed superior weighted PFS initially (<8 years) but worse late-phase outcomes compared to nodal DLBCL.

In terms of the mutational landscape, the most prevalent driver mutations in PB-DLBCL were *PIM1* (63%), followed by *SPEN* (46%), *NOTCH2* (35%), *CD79B* (33%), *CREBBP* (33%), *DNMT3A* (27%) and *MYD88* (24%). Compared to nodal DLBCL, PB-DLBCL and IP-DLBCL showed enriched BCR/NF-κB pathway mutations, reflecting their non-GCB predominance. Notably, mutations in epigenetic regulators and NOTCH2 signaling pathway were significantly more frequent in PB-DLBCL. The mutation rates of *TP53* in nodal, PB, and IP-DLBCL were 29%, 14% and 9%, respectively. Conversely, *PIM1* mutation frequencies were 34%, 59% and 73%, respectively. Among patients classifiable by the LymphGen algorithm, PB-DLBCL was predominantly comprised of the MCD (48.6%) and EZB (34.3%) subtypes. IP-DLBCL was largely categorized as the MCD subtype (79.2%), whereas nodal DLBCL displayed a more diverse profile, with a relatively balanced distribution of BN2, EZB, MCD and ST2 subtypes. Survival analysis revealed *PTPRK, MAP2K1* or *DNMT3A* mutations were significantly associated with shorter PFS in PB-DLBCL.

LME classification revealed that the depleted subtype was more common (34.5% vs 29.2%), while the germinal center-like subtype was less frequent (5.2% vs 12.5%) in PB-DLBCL compared to nodal DLBCL, suggesting different microenvironmental composition that warrant further mechanistic study.

**Copyright Information:** (Online) ISSN: 2572-9241
©2026 The Author(s). HemaSphere is published by John Wiley & Sons Ltd on behalf of European Hematology Association. This is an open access Abstract Book distributed under the Attribution-NonCommercial-NoDerivs (CC BY-NC-ND), which allows third parties to download the articles and share them with others as long as they credit the author and the Abstract Book, but they cannot change the content in any way or use them commercially.
**Abstract Book Citations:** Authors, Title, HemaSphere, 2026;10:(S1):pages. The abstract book DOIs can be found on the "Author Information" page.
**Disclaimer:** Articles published in the journal HemaSphere exclusively reflect the opinions of the authors. The authors are responsible for all content in their abstracts including accuracy of the facts, statements, citing resources, etc.

### Summary/Conclusion

Overall, our comprehensive analysis defines PB-DLBCL as a distinct biological entity, distinguishable from nodal and IP-DLBCL. The prevalence of specific molecular subtypes and recurrent epigenetic dysregulation highlight the potential for novel precision therapeutic strategies.

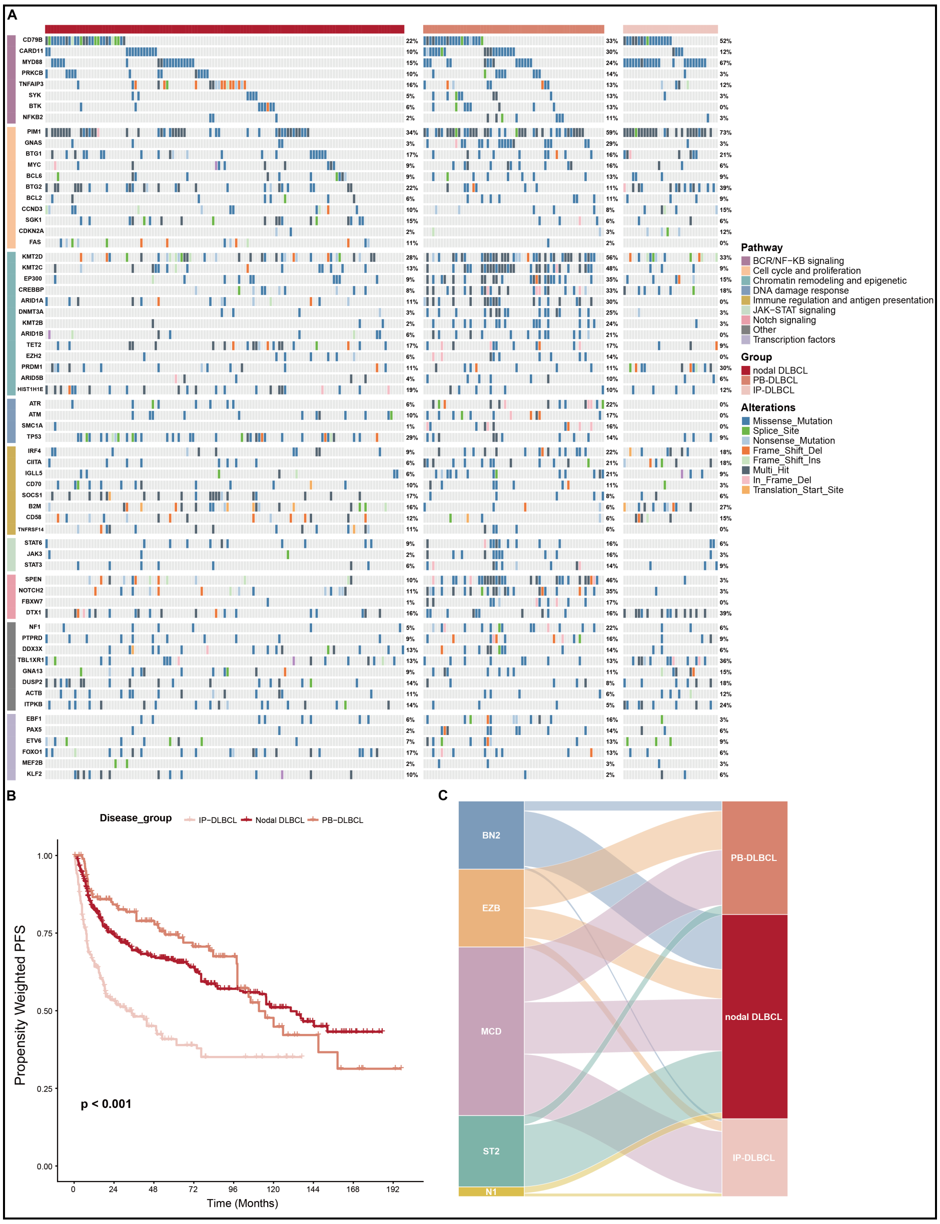

Figure1. Genomic and clinical comparisons among nodal DLBCL, PB-DLBCL and IP-DLBCL cohorts. (A) Oncoplot displaying the mutational landscape across the three cohorts. (B) Kaplan-Meier curves showing weighted PFS after IPTW adjustment. (C) Sankey diagram illustrating distribution of the three DLBCL entities across LymphGen categories.

**Copyright Information:** (Online) ISSN: 2572-9241
©2026 The Author(s). HemaSphere is published by John Wiley & Sons Ltd on behalf of European Hematology Association. This is an open access Abstract Book distributed under the Attribution-NonCommercial-NoDerivs (CC BY-NC-ND), which allows third parties to download the articles and share them with others as long as they credit the author and the Abstract Book, but they cannot change the content in any way or use them commercially.
**Abstract Book Citations:** Authors, Title, HemaSphere, 2026;10:(S1):pages. The abstract book DOIs can be found on the "Author Information" page.
**Disclaimer:** Articles published in the journal HemaSphere exclusively reflect the opinions of the authors. The authors are responsible for all content in their abstracts including accuracy of the facts, statements, citing resources, etc.

### (S248) BRENTUXIMAB VEDOTIN PLUS CHP PLUS ETOPOSIDE (CHEPA) IN NEWLY DIAGNOSED CD30- POSITIVE PERIPHERAL T-CELL LYMPHOMAS WITH INTEGRATED CTDNA ANALYSIS: PRIMARY RESULTS OF THE PHASE 2 CHEPA TRIAL.

**Topic:** 20. Other aggressive lymphomas

Marek Trněný*[1], Jozef Michalka[2], Pavel Klener[1], David Belada[3], Heidi Mociková[4], Ales Obr[5], Katerina Steinerova[6], Juraj Ďuraš[7], Katerina Benesova[1], Andrea Janíková[2], Diana Malarikova[1], Alice Sykorova[3], Vaclav Ptacnik[1], Jan Galko[1], Vít Campr[8], Takeshi Sugio[9], Troy Noordenbos[9], Jurik Mutter[9], Ash Alizadeh[9], Magdalena Klanova[1]

*[1]Charles University General Hospital, Praha, Czechia; [2]FN Brno Bohunice, Brno, Czechia; [3]FN Hradec Kralove, Hradec Kralove, Czechia; [4]FN Kralovske Vinohrady, Praha, Czechia; [5]FN Olomouc, Olomouc, Czechia; [6]FN Plzen, Plzen, Czechia; [7]FN Ostrava, Ostrava, Czechia; [8]FN Motol, Praha, Czechia; [9]Stanford Cancer Center, Stanford, United States of America;*

### Background

Background: Peripheral T-cell lymphomas (PTCLs) are aggressive malignancies associated with poor outcomes. While adding brentuximab vedotin (BV) to CHP has improved results in CD30+ PTCL, relapses remain common. Given the suggested benefit of etoposide (CHOEP) in younger patients, the prospective multicenter single-arm Phase 2 CHEPA study (NCT05006664) was conducted by CLSG to evaluate the efficacy and safety of CHEPA (BV-CHEP) induction combined with circulating tumor DNA (ctDNA) analysis.

### Aims

Given the suggested benefit of etoposide (CHOEP) in younger patients, the prospective multicenter single-arm Phase 2 CHEPA study (NCT05006664) was conducted by CLSG to evaluate the efficacy and safety of CHEPA (BV-CHEP) induction combined with circulating tumor DNA (ctDNA) analysis.

### Methods

Methods: Patients with previously untreated CD30+ PTCL eligible for autologous stem cell transplantation (ASCT) were enrolled. Treatment consisted of 6 cycles of CHEPA (D1: BV 1.8 mg/kg, cyclophosphamide 750 mg/m², doxorubicin 50 mg/m²; D1–3: etoposide 100 mg/m²; D1–5: prednisone 100 mg/d) every 21 days. The primary endpoint was the complete metabolic response (CMR) rate at the end of treatment (EOT). Secondary endpoints included safety, PFS, OS, ORR, and exploratory ctDNA analysis. Plasma cfDNA was profiled using CAPP-Seq (259 genes) and clonotypic VDJ sequencing at all TCR loci via SABER.

**Copyright Information:** (Online) ISSN: 2572-9241
©2026 The Author(s). HemaSphere is published by John Wiley & Sons Ltd on behalf of European Hematology Association. This is an open access Abstract Book distributed under the Attribution-NonCommercial-NoDerivs (CC BY-NC-ND), which allows third parties to download the articles and share them with others as long as they credit the author and the Abstract Book, but they cannot change the content in any way or use them commercially.
**Abstract Book Citations:** Authors, Title, HemaSphere, 2026;10:(S1):pages. The abstract book DOIs can be found on the “Author Information” page.
**Disclaimer:** Articles published in the journal HemaSphere exclusively reflect the opinions of the authors. The authors are responsible for all content in their abstracts including accuracy of the facts, statements, citing resources, etc.

### Results

Results: Between 05/2022 and 09/2025, 40 patients were screened; 33 met eligibility criteria and initiated therapy. The cohort included 14 (42%) ALCL and 19 (58%) non-ALCL cases (median age 57 years, range 26–69). Baseline characteristics showed a predominance of males (67%), advanced-stage disease (79%), elevated LDH (58%), and bone marrow involvement (30%); all pts had an ECOG PS of 0–1. There was similar mutation burden in ctDNA found in ALCL as non-ALCL group with most frequent mutations of *TET2, DNMT3A, RHOA and STAT3* in non-ALCL. All 33 pts completed 6 cycles of CHEPA. At EOT, 25/33 (76%) achieved CMR, meeting the primary endpoint. The ORR was 91% for the entire cohort (CMR: 86% for ALCL, 68% for non-ALCL). ASCT was preplanned in 21/33 pts and has been performed to date in 14 pts. At a median follow-up of 19 months, the 18-month PFS and OS probabilities were 75% and 92%, resp. Specifically, PFS was 100% for ALCL vs. 59% for non-ALCL, while OS was 100% vs. 87%. Age and bone marrow involvement were the only baseline variables significantly associated with CMR. Baseline ctDNA burden was significantly correlated with total metabolic tumor volume ($r=0.68$, $p<0.001$), LDH level ($r=0.48$, $p=0.03$), and PIT ($p<0.05$). Grade 3–4 adverse events occurred in 82% of pts, primarily hematological: neutropenia (79%), thrombocytopenia (18%), febrile neutropenia (15%), and anemia (15%). No grade 3–4 peripheral neuropathy or grade 5 events were reported

### Summary/Conclusion

Conclusions: The CHEPA study met its primary endpoint with a 76% CMR rate. Despite limited follow-up and high non-ALCL prevalence of our cohort, PFS and OS results are promising. Toxicity was manageable with no treatment-related deaths even though 33% of patients were >60 years old, supporting the safety of adding etoposide to the BV-CHP backbone. Further investigation of outcomes based on EOT MRD by ctDNA is ongoing.

**Copyright Information:** (Online) ISSN: 2572-9241
©2026 The Author(s). HemaSphere is published by John Wiley & Sons Ltd on behalf of European Hematology Association. This is an open access Abstract Book distributed under the Attribution-NonCommercial-NoDerivs (CC BY-NC-ND), which allows third parties to download the articles and share them with others as long as they credit the author and the Abstract Book, but they cannot change the content in any way or use them commercially.
**Abstract Book Citations:** Authors, Title, HemaSphere, 2026;10:(S1):pages. The abstract book DOIs can be found on the “Author Information” page.
**Disclaimer:** Articles published in the journal HemaSphere exclusively reflect the opinions of the authors. The authors are responsible for all content in their abstracts including accuracy of the facts, statements, citing resources, etc.

### (S249) HIGH-DOSE METHOTREXATE DOES NOT REDUCE RISK OF CNS RELAPSE IN ULTRA HIGH-RISK LARGE B-CELL LYMPHOMA: AN INTERNATIONAL RETROSPECTIVE ANALYSIS OF 1,923 PATIENTS

**Topic:** 19. Large B cell lymphomas - Clinical

Matthew R. Wilson*[1], Katharine Lewis[2], Amy Kirkwood[3], Kate Cwynarski[4], Chan Y. Cheah[2], Tarec El-Galaly[5], Diego Villa[6], Kerry Savage[6], Paola Ghione[7], Sabela Bobillo Varela[7], Karin Ekstrom Smedby[8], Sara Harrysson[8], Marek Trněný[9], Magdalena Klanova[9], Robert Puckrin[10], Christopher P. Fox[11, 12], Mark Bishton[11, 12], Gita Thanarajasingam[13], Nicole Wong Doo[14], Greg Hapgood[15], Carole Soussain[16], Sylvain Choquet[17], Michael Dickinson[18], Dipti Talaulikar[19], Sanjay de Mel[20], Gavin Preston[21], Matthew Ahearne[22], Eliza Hawkes[23], Elisabeth Schorb[24], Aline Clavert[25], Matthew Ku[26], Anna Guidetti[27], Mayur Narkhede[28], Teresa Calimeri[29], Eric Durot[30], Jeff Smith[31], Loïc Renaud[32], Pam McKay[1], Toby A. Eyre[33]

*[1]Beatson West of Scotland Cancer Centre, Glasgow, United Kingdom;[2]Sir Charles Gairdner Hospital, Perth, Australia;[3]University College London, London, United Kingdom; [4]University College London Hospital, London, United Kingdom; [5]Aalborg University Hospital, Aalborg, Denmark; [6]BC Cancer - Vancouver Cancer Centre, Vancouver, Canada; [7]Memorial Sloan Kettering Cancer Center, New York, United States of America; [8]Karolinska Institutet, Stockholm, Sweden; [9]Charles University, General University Hospital, Prague, Czechia; [10]University of Calgary, Calgary, Canada; [11]School of Medicine, University of Nottingham, Nottingham, United Kingdom; [12]Nottingham University Hospitals NHS Trust, Nottingham, United Kingdom; [13]Mayo Clinic, Rochester, Minnesota, United States of America; [14]Concord Clinical School, University of Sydney, Sydney, Australia;[15]Princess Alexandra Hospital, Brisbane, Australia; [16]Institut Curie, site de Saint-Cloud, Paris, France;[17]Sorbonne Université, Hôpital de la Pitié-Salpêtrière, APHP, Paris, France; [18]Peter MacCallum Cancer Centre, Melbourne, Australia; [19]Canberra Health Services, Canberra, Australia; [20]National University Cancer Institute, Singapore, Singapore;[21]Aberdeen Royal Infirmary, Aberdeen, United Kingdom; [22]University Hospitals of Leicester, Leicester, United Kingdom;[23]Olivia Newton-John Cancer Centre at Austin Health, Melbourne, Australia; [24]Medical Center, Faculty of Medicine, University of Freiburg, Freiburg, Germany;[25]Maladie du sang CHU ANGERS, Angers, France; [26]St Vincent's Hospital and University of Melbourne, Melbourne, Australia;[27]Instituo Nazionale dei Tumori, Milan, Italy; [28]University of Alabama, Birmingham, United States of America;[29]Fondazione IRCCS Istituto Nazionale Tumori, Milan, Italy; [30]CHU de Reims, Reims, France;[31]Clatterbridge Cancer Centre, Liverpool, United Kingdom;[32]AP-HP, Hôpital Saint-Louis, Paris, France; [33]Oxford University Hospitals NHS Trust, Oxford, United Kingdom;*

**Background**

The role of high-dose methotrexate (HDMTX) as CNS prophylaxis in high-risk large B-cell lymphoma (LBCL) remains controversial. There are no large prospective RCTs supporting its use. Two recent retrospective studies suggested minimal impact on CNS relapse (CNSr). However, guidelines continue to recommend consideration of HDMTX in pts with 'ultra high-risk' (UHR) features due to a lack of robust data in these subgroups, where CNSr risk is 10-25%.

**Aims**

To analyse the impact of HDMTX on LBCL pts considered UHR for CNSr.

**Copyright Information:** (Online) ISSN: 2572-9241
©2026 The Author(s). HemaSphere is published by John Wiley & Sons Ltd on behalf of European Hematology Association. This is an open access Abstract Book distributed under the Attribution-NonCommercial-NoDerivs (CC BY-NC-ND), which allows third parties to download the articles and share them with others as long as they credit the author and the Abstract Book, but they cannot change the content in any way or use them commercially.
**Abstract Book Citations:** Authors, Title, HemaSphere, 2026;10:(S1):pages. The abstract book DOIs can be found on the "Author Information" page.
**Disclaimer:** Articles published in the journal HemaSphere exclusively reflect the opinions of the authors. The authors are responsible for all content in their abstracts including accuracy of the facts, statements, citing resources, etc.

### Methods

This multicentre, retrospective analysis combined data from 56 sites who contributed to the aforementioned studies (*Wilson et al, Blood 2022; Lewis et al, JCO 2023*). All pts received curative intent chemo-immunotherapy. *Wilson et al* recruited LBCL pts who had received HDMTX, regardless of risk. *Lewis et al* recruited pts with ≥1 of these high-risk criteria: CNS-IPI 4-6; high grade B-cell lymphoma with MYC+BCL2+/-BCL6 rearrangements; testicular/breast/renal/adrenal extranodal (EN) involvement.
For this analysis, pts were deemed UHR if they met ≥1 of the following: CNS-IPI 5-6; testicular, renal/adrenal or breast involvement; ≥3 EN sites. Time to CNSr was measured from diagnosis (or landmark) until first event (CNSr) or competing event (systemic relapse, stable disease at EOT or death); pts not experiencing an event were censored at last follow up. Groups were compared using competing risks survival analysis (Fine and Grey). Time to isolated CNSr was also analysed with synchronous events included as competing risks. A landmark analysis (LMA) was performed for all pts who had no event at 6 months.

### Results

Analyses were restricted to 1923 pts meeting criteria for UHR (no HDMTX n=1051, HDMTX n=872). Median age was 66y (range 18-86) and follow-up 54.4 months (IQR 31.9-85.2). HDMTX treated pts had more frequent involvement of high-risk EN sites vs no HDMTX (renal/adrenal 35.4% vs 30.1% (p=0.014), testicular 27.1% vs 17.0% (p<0.0001), ≥3 EN sites 52.6% vs 43.7% (p=0.0001)). Conversely, fewer HDMTX pts had CNS-IPI 5-6 (31.4% vs 48.0%, p<0.0001). Intrathecal prophylaxis was given in 32.4% no HDMTX pts vs 50.3% HDMTX (p<0.0001). In HDMTX pts, 73% received cumulative dose of ≥6g/m2.
In all UHR pts, there was no significant difference in 3yr CNSr rate between no HDMTX vs HDMTX pts, including on multivariable analysis adjusting for baseline characteristics (9.3% vs 8.1%, adjusted HR 1.10 (95%CI 0.80-1.53)). Analyses restricted to isolated CNSr also confirmed no difference (5.9% vs 5.7%, adjusted HR 1.01 (95% CI 0.68-1.50)).
In the LMA (n=1529; no HDMTX n=782, HDMTX n=773), 3yr overall survival was 79.7% (95% CI 76.6–82.4) vs 83.8% (95% CI 80.5–86.6). No difference in 3yr CNSr rate was seen in the UHR group (6.7% vs 6.6%, adjusted HR 0.89 (95%CI 0.58-1.35)). No significant reduction in CNSr was seen in any high-risk subgroup (fig 1c). A non-statistically significant reduction in any CNSr was observed in pts with testicular involvement (9.4% vs 6.8%, HR 1.54 (95%CI 0.78-3.03)), however the difference was not seen in isolated CNSr (6.1% vs 6.3%, HR 1.08 (95%CI 0.51-2.31)).

### Summary/Conclusion

This is the largest international comparative dataset of UHR LBCL pts treated with or without HDMTX prophylaxis, showing no significant reduction in CNSr with HDMTX in all UHR pts or in any individual high-risk subgroup. Despite inherent limitations, the data suggest HDMTX prophylaxis has no benefit and future studies should focus on alternative approaches to reduce risk in UHR pts.

**Copyright Information:** (Online) ISSN: 2572-9241
©2026 The Author(s). HemaSphere is published by John Wiley & Sons Ltd on behalf of European Hematology Association. This is an open access Abstract Book distributed under the Attribution-NonCommercial-NoDerivs (CC BY-NC-ND), which allows third parties to download the articles and share them with others as long as they credit the author and the Abstract Book, but they cannot change the content in any way or use them commercially.
**Abstract Book Citations:** Authors, Title, HemaSphere, 2026;10:(S1):pages. The abstract book DOIs can be found on the "Author Information" page.
**Disclaimer:** Articles published in the journal HemaSphere exclusively reflect the opinions of the authors. The authors are responsible for all content in their abstracts including accuracy of the facts, statements, citing resources, etc.

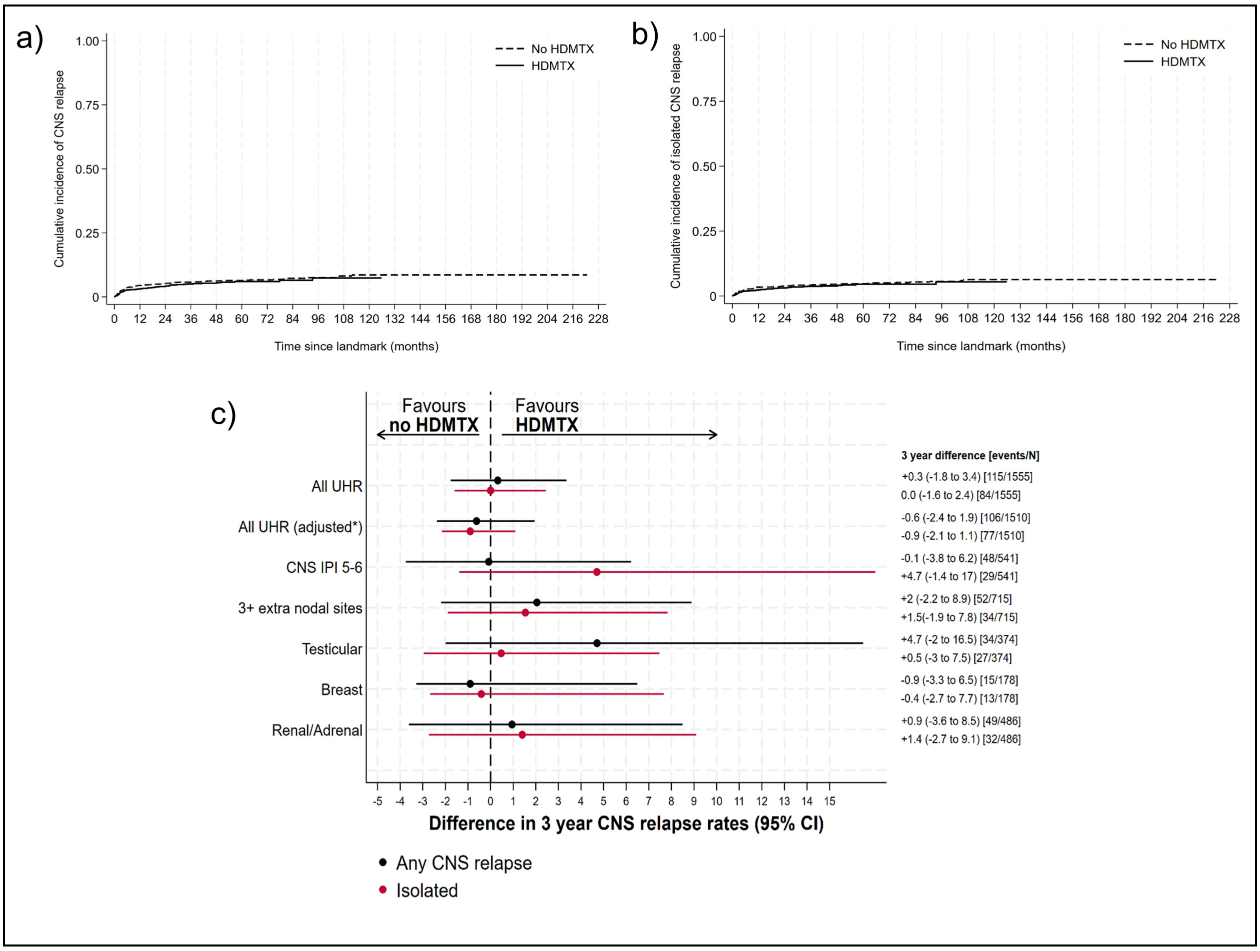

Figure 1: a) Cumulative incidence of any CNS relapse for landmark cohort by receipt of HDMTX, b) Cumulative incidence of isolated CNS relapse for landmark cohort by receipt of HDMTX, c) difference in 3yr CNS relapse rates for individual subgroups in landmark cohort *adjusted for age, sex, stage, LDH, extranodal sites 2+, ECOG 2+, renal/adrenal involvement, use of IT

**Copyright Information:** (Online) ISSN: 2572-9241
©2026 The Author(s). HemaSphere is published by John Wiley & Sons Ltd on behalf of European Hematology Association. This is an open access Abstract Book distributed under the Attribution-NonCommercial-NoDerivs (CC BY-NC-ND), which allows third parties to download the articles and share them with others as long as they credit the author and the Abstract Book, but they cannot change the content in any way or use them commercially.
**Abstract Book Citations:** Authors, Title, HemaSphere, 2026;10:(S1):pages. The abstract book DOIs can be found on the "Author Information" page.
**Disclaimer:** Articles published in the journal HemaSphere exclusively reflect the opinions of the authors. The authors are responsible for all content in their abstracts including accuracy of the facts, statements, citing resources, etc.

### (S250) BRAIN-LYM: DEFINING IMMUNE-MOLECULAR PHENOTYPES IN PRIMARY CNS LYMPHOMA

**Topic:** 21. Lymphoma biology & translational research

Fleur de Groot*[1], Tim Dekker[1], Lorraine de Haan[1], Suzanne Blommers[1], Romée Jansen[1], Joost Scheffers[1], Rosanne van der Post[1], Stefan Böhringer[1], Jeanette Doorduijn[2], King Lam[2], Arjan Diepstra[3], Liane Te Boome[4], Valeska Terpstra[4], Lara Bohmer[5], Josée Zijlstra[6], Lianne Koens[6], Eduardus F.M. Posthuma[7], Marc Durian[8], Jeroen Stavast[8], Marjolein Van der Poel[9], Myrurgia Abdul Hamid[9], Wendy Stevens[10], Josephus van Rooij[11], Margot Jak[11], Angelika Mühlebner[11], Michiel van den Brand[10], Okke de Weerdt[12], Aart Beeker[13], Otto Visser[14], Nynke Tjeerdsma[14], Tjeerd Snijders[15], Sherida Woei-A-Jin[16], Thomas Tousseyn[16], Marie José Kersten[6], Jacoline Bromberg[2], Patty Jansen[1], Marcel Nijland[3], Ruben de Groen[1], Joost Vermaat[1]

*[1]Leiden University Medical Center, Hematology, Leiden, The Netherlands; [2]Erasmus MC, Hematology, Rotterdam, The Netherlands; [3]UMCG, Pathology, Groningen, The Netherlands; [4]Haaglanden MC, Hematology, Den Haag, The Netherlands; [5]Haga ziekenhuis, Hematologi, Den Haag, The Netherlands; [6]Amsterdam UMCs, Hematology, Amsterdam, The Netherlands; [7]Reinier de Graaf Gasthuis, Hematology, Delft, The Netherlands; [8]ETZ Tilburg, Hematology, Tilbirg, The Netherlands; [9]Maastricht UMC, Hematology, Maastricht, The Netherlands; [10]Radboud UMC, Hematology, Nijmegen, The Netherlands; [11]UMC Utrecht, Neurology, Utrecht, The Netherlands; [12]Antonius ziekenhuis, Hematology, Nieuwegein, The Netherlands; [13]Spaarne Gasthuis, Hematology, Haarlem, The Netherlands; [14]Isala ziekenhuis, Hematology, Zwolle, The Netherlands; [15]MST, Hematology, Enschede, The Netherlands; [16]UZ Leuven, Hematology, Leuven, Belgium;*

### Background

Primary central nervous system lymphoma (PCNSL) is a distinct, immune-privileged diffuse large B-cell lymphoma with high relapse rates despite high-dose methotrexate (HD-MTX)–based polychemotherapy. While immune-directed treatments, like CAR T-cells and bispecific antibodies are increasingly explored, the immune-molecular architecture of PCNSL and its relation to therapy response remain poorly defined (PMID: 33664848, 36402300). Defining clinically relevant immune-molecular subgroups is essential to enable rational implementation of immunotherapy.

### Aims

To perform a nationwide integrated in-depth DNA and RNA analysis of PCNSL tumors, linking immune-molecular profiles to treatment response to identify subgroups with potential therapeutic implications.

### Methods

Patients with histologically confirmed PCNSL (2000–2022) treated with ≥1 cycle HD-MTX-based chemotherapy were included. DNA and RNA were extracted from FFPE. Gene-expression profiling using the BLYM-777 panel (biology-driven 777-gene lymphoma panel, PMID: 35454765) assessed tumor-intrinsic and microenvironment signatures. Targeted next-generation sequencing was performed for mutational profiling.

**Copyright Information:** (Online) ISSN: 2572-9241
©2026 The Author(s). HemaSphere is published by John Wiley & Sons Ltd on behalf of European Hematology Association. This is an open access Abstract Book distributed under the Attribution-NonCommercial-NoDerivs (CC BY-NC-ND), which allows third parties to download the articles and share them with others as long as they credit the author and the Abstract Book, but they cannot change the content in any way or use them commercially.
**Abstract Book Citations:** Authors, Title, HemaSphere, 2026;10:(S1):pages. The abstract book DOIs can be found on the "Author Information" page.
**Disclaimer:** Articles published in the journal HemaSphere exclusively reflect the opinions of the authors. The authors are responsible for all content in their abstracts including accuracy of the facts, statements, citing resources, etc.

**Results**

In total 259 histologically confirmed PCNSL patients were treated with HD-MTX-based polychemotherapy. 109 patients were chemorefractory with progression or relapse < 12 months from diagnosis, 115 patients were chemosensitive without progression or relapse, 35 patients relapsed > 12 months after diagnosis. The clinical response groups showed clear OS differences (p< 0.0001, Figure A).

For GEP in 259 patients no batch effects were detected by principal component analysis. Unsupervised clustering identified three distinct groups (Figure B). Next, GEP data was analyzed using the microenvironment characterization proposed by Kotlov et al. (PMID: 33541860, Figure C). Cluster 1 shows low expression of T regulatory cell, T follicular helper cells, T cell traffic, and NK cells. Cluster 2 distinguishes itself by low MHC-I and -II expression. Cluster 3 has high expression of macrophages, T cell traffic, T follicular helper cells, tumor infiltrating lymphocytes, NK cells and low B cells and cell proliferation. So, the unsupervised clusters were classified according to their immune characteristics: cluster 1 as immune-low, cluster 2 as immune-evasive, and cluster 3 as immune-rich. Distribution of response categories across clusters suggested enrichment of chemorefractory cases within the immune-low subgroup (exploratory analysis). The immune-evasive (MHC-low) phenotype suggests antigen-presentation escape that may underlie early treatment failure, supporting early immunotherapy strategies.

tNGS was performed in 194 PCNSLs. A median of 12 mutations (range 1–53) per patient was identified. The most frequently mutated genes, including *MYD88* (67%), *PIM1* (54%), *CD79B* (43%), and *TBL1XR1* (32%), are associated with the NFκB pathway (PMID: 26314080).

**Summary/Conclusion**

To our knowledge, this is one of the largest nationwide integrated DNA/RNA analyses of HD-MTX treated PCNSL linking immune-molecular profiles to standardized clinical response categories. According to Kotlov et al., GEP data identified three subgroups with distinct immune characteristics: immune-low, immune-evasive and immune-rich. These findings provide a biological framework for immune-based patient stratification in future PCNSL trials.

**Copyright Information:** (Online) ISSN: 2572-9241
©2026 The Author(s). HemaSphere is published by John Wiley & Sons Ltd on behalf of European Hematology Association. This is an open access Abstract Book distributed under the Attribution-NonCommercial-NoDerivs (CC BY-NC-ND), which allows third parties to download the articles and share them with others as long as they credit the author and the Abstract Book, but they cannot change the content in any way or use them commercially.
**Abstract Book Citations:** Authors, Title, HemaSphere, 2026;10:(S1):pages. The abstract book DOIs can be found on the "Author Information" page.
**Disclaimer:** Articles published in the journal HemaSphere exclusively reflect the opinions of the authors. The authors are responsible for all content in their abstracts including accuracy of the facts, statements, citing resources, etc.

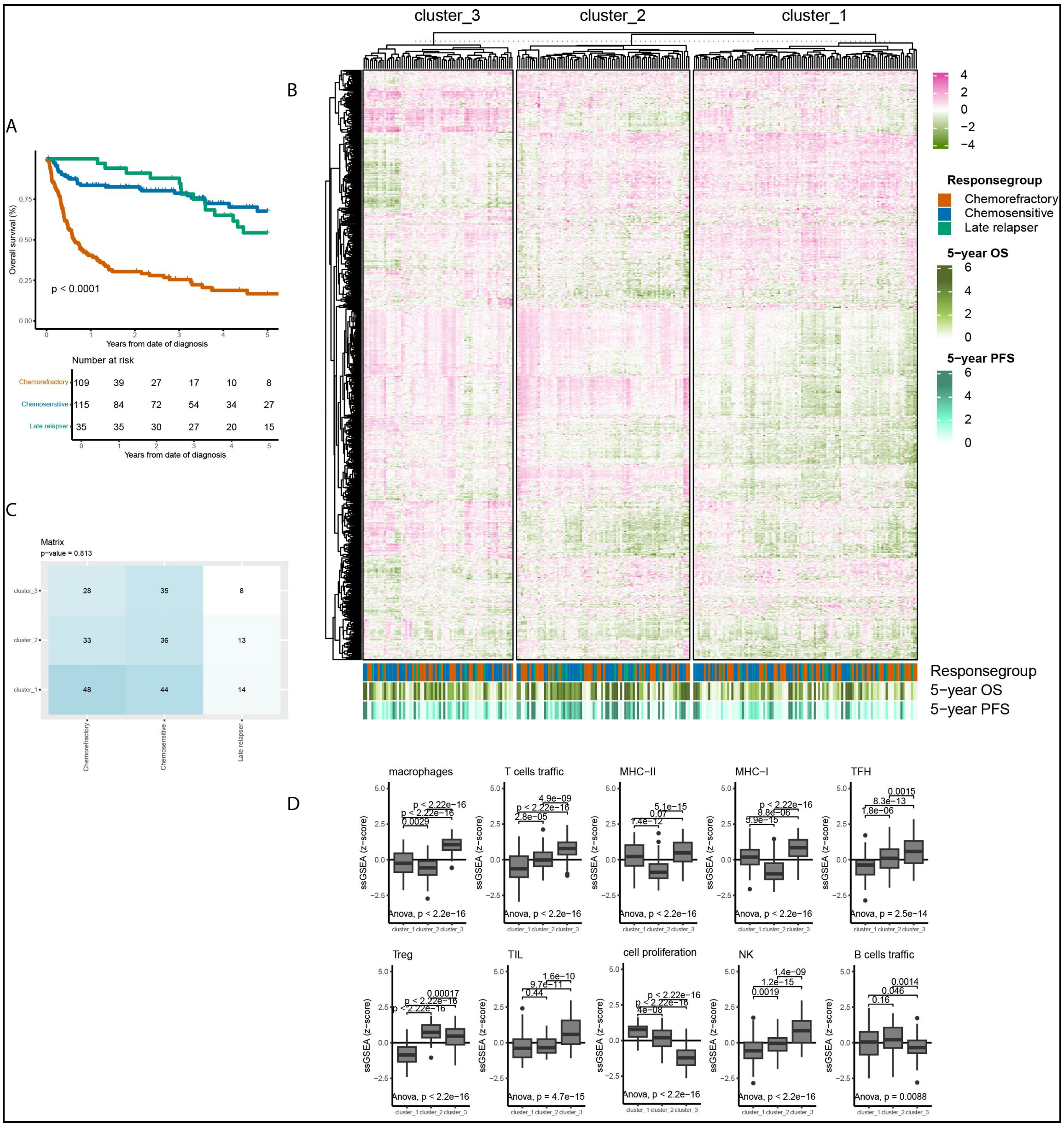

A) Overall survival curve, B) GEP heatmap depicting three distinct clusters, C) Incidence matrix, D) Kotlov et al. microenviroment characterization

**Copyright Information:** (Online) ISSN: 2572-9241
©2026 The Author(s). HemaSphere is published by John Wiley & Sons Ltd on behalf of European Hematology Association. This is an open access Abstract Book distributed under the Attribution-NonCommercial-NoDerivs (CC BY-NC-ND), which allows third parties to download the articles and share them with others as long as they credit the author and the Abstract Book, but they cannot change the content in any way or use them commercially.
**Abstract Book Citations:** Authors, Title, HemaSphere, 2026;10:(S1):pages. The abstract book DOIs can be found on the "Author Information" page.
**Disclaimer:** Articles published in the journal HemaSphere exclusively reflect the opinions of the authors. The authors are responsible for all content in their abstracts including accuracy of the facts, statements, citing resources, etc.

### (S251) INTEGRATIVE PROTEOMICS IDENTIFIES DISTINCT SUBGROUPS AND ACTIONABLE SIGNALING PATHWAYS IN RICHTER TRANSFORMATION

**Topic:** 21. Lymphoma biology & translational research

Sébastien Hergalant[1], Romain Piucco[1], Ghislain Fievet[1], Romain Morizot[2], Anne Quinquenel[3], Reiner Siebert[4], Romain Guieze[5], Christine Carapito[6], Luc-Matthieu Fornecker[7], Pierre Feugier[2], Julien Broseus*[2]

*[1]INSERM U1256, Nancy, France; [2]Hospital Center Régional And University de Nancy Hospital Central, Nancy, France; [3]University Hospital of Reims, Reims, France; [4]University of Ulm, Ulm, Germany; [5]University Hospital of Clermont-Ferrand, Clermont-Ferrand, France; [6]CNRS, Strasbourg, France; [7]University Hospital of Strasbourg, Strasbourg, France;*

### Background

Richter transformation (RT) is the development of an aggressive lymphoma, mostly diffuse large B-cell lymphoma (DLBCL) in patients with chronic lymphocytic leukemia (CLL), associated with marked chemoresistance and a median overall survival below 12 months. The majority of RT cases (~80-90%) arise from pre-existing CLL clones, whereas a minority develop from an unrelated clone. The genetic and transcriptomic landscapes of RT have been characterized [1-4], revealing high mutational burden and recurrent alterations in key oncogenic pathways, including PI3K/AKT, B-cell receptor signaling, MYC, NF-KB and MAPK pathways, and cell cycle regulation.
Proteins are the primary functional effectors of the cell, shaping malignant phenotypes beyond genetic aberrations or RNA expression. The proteomic landscape underlying RT aggressiveness, heterogeneity, and potential therapeutic vulnerabilities remains largely unexplored. Comprehensive profiling of the RT proteome is therefore essential to elucidate oncogenic mechanisms and identify actionable therapeutic targets.

### Aims

The objectives of this study were to delineate the proteomic architecture of RT, define biologically and clinically relevant subgroups, and identify protein networks driving oncogenicity, chemoresistance, and patient outcomes through integrated multi-omic analyses. In addition, the study aimed to identify distinctive biomarkers between RT and *de novo* DLBCL.

### Methods

Large-scale, quantitative proteomic profiling of fresh tumour biopsies from 21 RT, 27 de novo DLBCL, and 7 transformed follicular lymphomas was performed using LC–MS/MS. Proteomes were reconstructed using a custom computational pipeline and integrated with matched whole-transcriptome and whole-exome sequencing data, as well as copy number alterations and DNA methylation profiles. Associations with recurrent genetic alterations, key biological processes, and clinical outcomes were assessed. Both unsupervised and supervised analyses were applied to identify RT-specific proteomic signatures, functional pathways, molecular subgroups, and druggable protein networks.

**Copyright Information:** (Online) ISSN: 2572-9241
©2026 The Author(s). HemaSphere is published by John Wiley & Sons Ltd on behalf of European Hematology Association. This is an open access Abstract Book distributed under the Attribution-NonCommercial-NoDerivs (CC BY-NC-ND), which allows third parties to download the articles and share them with others as long as they credit the author and the Abstract Book, but they cannot change the content in any way or use them commercially.
**Abstract Book Citations:** Authors, Title, HemaSphere, 2026;10:(S1):pages. The abstract book DOIs can be found on the "Author Information" page.
**Disclaimer:** Articles published in the journal HemaSphere exclusively reflect the opinions of the authors. The authors are responsible for all content in their abstracts including accuracy of the facts, statements, citing resources, etc.

**Results**

The RT proteome differs from *de novo* DLBCL, with enrichment in proteins involved in immunological synapse organisation (CD44, ITGB2), signal transduction (PRKCB, PTPN6), metabolic reprogramming (CPT1A, HADHA), and cell cycle regulation (ATM, PRKDC). Two RT subgroups with divergent immunogenic and proliferative programs were identified, associated with *SF3B1* and *NOTCH1* mutations, MYC-driven transcriptional activity, and altered B-cell transcription factor networks.
Cox proportional hazards modelling and selective analysis of bimodal missing non at random proteins identified complementary abundance- and absence-based signatures associated with chemoresistance and survival, converging on BCR signalling, JAK–STAT activation, TP53-associated apoptotic dysfunction, oxidative phosphorylation, and fatty acid metabolism. Downstream BCR components, including PKC isoforms and PTPRC, emerged as actionable targets.

**Summary/Conclusion**

This proteome-centered integrative study identifies a specific RT proteomic architecture, capturing disease heterogeneity beyond genomic and transcriptomic alterations. RT-specific proteomic subgroups and potentially druggable signaling networks highlight downstream BCR signaling and metabolic pathways as key drivers of aggressiveness and therapeutic resistance, providing a framework for proteome-informed stratification in RT.

**Copyright Information:** (Online) ISSN: 2572-9241
©2026 The Author(s). HemaSphere is published by John Wiley & Sons Ltd on behalf of European Hematology Association. This is an open access Abstract Book distributed under the Attribution-NonCommercial-NoDerivs (CC BY-NC-ND), which allows third parties to download the articles and share them with others as long as they credit the author and the Abstract Book, but they cannot change the content in any way or use them commercially.
**Abstract Book Citations:** Authors, Title, HemaSphere, 2026;10:(S1):pages. The abstract book DOIs can be found on the “Author Information” page.
**Disclaimer:** Articles published in the journal HemaSphere exclusively reflect the opinions of the authors. The authors are responsible for all content in their abstracts including accuracy of the facts, statements, citing resources, etc.

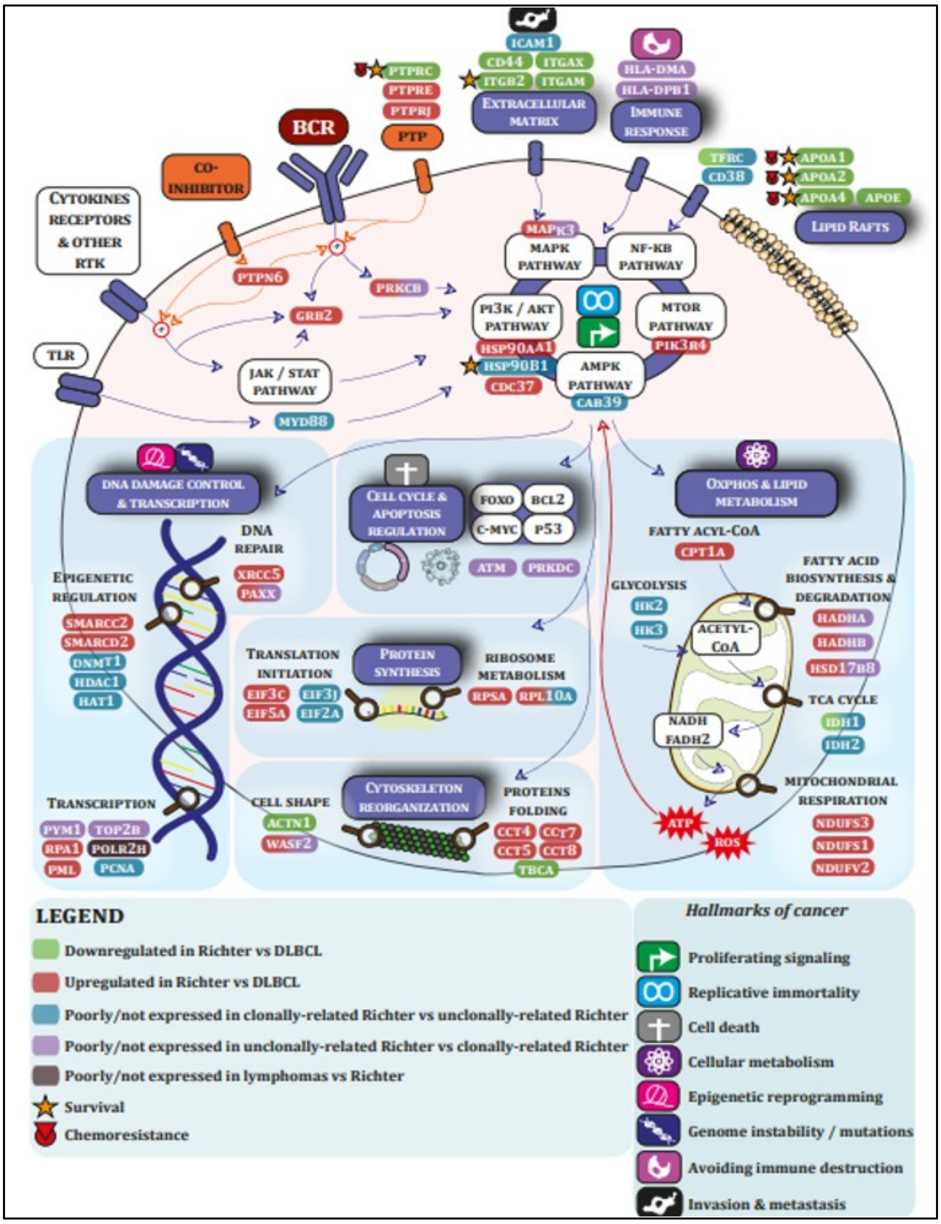

**Copyright Information:** (Online) ISSN: 2572-9241
©2026 The Author(s). HemaSphere is published by John Wiley & Sons Ltd on behalf of European Hematology Association. This is an open access Abstract Book distributed under the Attribution-NonCommercial-NoDerivs (CC BY-NC-ND), which allows third parties to download the articles and share them with others as long as they credit the author and the Abstract Book, but they cannot change the content in any way or use them commercially.
**Abstract Book Citations:** Authors, Title, HemaSphere, 2026;10:(S1):pages. The abstract book DOIs can be found on the "Author Information" page.
**Disclaimer:** Articles published in the journal HemaSphere exclusively reflect the opinions of the authors. The authors are responsible for all content in their abstracts including accuracy of the facts, statements, citing resources, etc.

### (S252) CD20:CD3 T-CELL BISPECIFIC ANTIBODIES REPROGRAM THE TUMOR MICROENVIRONMENT AND SYNERGIZE WITH LYMPHOMA-TARGETED 4-1BB CO-STIMULATION IN PRECLINICAL DLBCL MODELS

**Topic:** 21. Lymphoma biology & translational research

Sara C Grijalba[1, 2], Marcos Garcia-Lacarte[1, 2], Javier Melchor[1, 2], Adrián Arnaiz-Leché[1, 2], Blanca Berrozpe[1, 2], Sylvia Herter[3], Marina Bacac[3], Pablo Umana[3], Christian Klein[3], Maria Amann[3], Koorosh Korfi[3], Fabien Gava[4], Loic Ysebaert[5], Laurent Camille[5], Miguel Ángel Canales Albendea[2, 6], Carlos Grande García[2, 6], Jose A. Martinez-Climent[2, 7, 8], Christine Bezombes[4], Francisco Javier Novo Villaverde[1, 2], Sergio Roa*[1, 2, 7]

*[1]University of Navarra (UNAV), Department of Biochemistry and Genetics, Pamplona, Spain;[2]Cancer Center Clínica Universidad de Navarra (CCUN), Pamplona, Spain; [3]Roche Innovation Center Zurich, Schlieren, Switzerland; [4]Cancer Research Center of Toulouse (CRCT), Toulouse, France; [5]Toulouse University Hospital (CHU Toulouse), IUCT-Oncopole, Toulouse, France; [6]Clínica Universidad de Navarra (CUN), Pamplona, Spain; [7]Centro de Investigación Biomédica en Red de Cáncer (CIBERONC), Instituto de Salud Carlos III, Madrid, Spain; [8]Cima Universidad de Navarra (CIMA), Pamplona, Spain;*

### Background

Diffuse large B-cell lymphoma (DLBCL) is the most common aggressive B-cell lymphoma, and 30-40% of patients develop relapsed/refractory (R/R) disease. Conventional anti-CD20 antibodies (e.g. rituximab, obinutuzumab) primarily rely on Fc-mediated effector mechanisms such as ADCC/ADP, whereas CD20:CD3 T-cell engaging bispecific antibodies (TCB; e.g. mosunetuzumab, glofitamab) directly recruit cytotoxic T cells and offer an off-the-shelf therapeutic strategy. However, limited durability and immune escape highlight the need for rational combinations that reinforce T-cell priming, persistence, and long-term surveillance beyond tumor debulking.

### Aims

To define immune mechanisms underlying durable lymphoma control with CD20:CD3 TCB and evaluate whether CD19-targeted 4-1BB co-stimulation enhances response depth and durability in preclinical models.

### Methods

We generated a genetically engineered mouse model (GEMM) of high-risk DLBCL humanized for CD20 (pBIC20 mice), preserving tumor-immune co-evolution and exhibiting late genetic relapses despite deep debulking after CD20+ cell clearance. Mice were treated with a surrogate hCD20:mCD3 TCB alone or combined with lymphoma-targeted 4-1BB co-stimulation (mCD19:m4-1BB or mCD19:m4-1BBL). Immune remodeling was assessed by multiparameter flow cytometry, bulk and single-cell transcriptomics, and TCR repertoire reconstruction. Core immune programs were cross-validated in DLBCL patient-derived lymphoma spheroids (DLBCL-PDLS) treated with hCD20:hCD3 bispecific therapy (glofitamab) and hCD19:h4-1BBL (englumafusp alfa).

**Copyright Information:** (Online) ISSN: 2572-9241
©2026 The Author(s). HemaSphere is published by John Wiley & Sons Ltd on behalf of European Hematology Association. This is an open access Abstract Book distributed under the Attribution-NonCommercial-NoDerivs (CC BY-NC-ND), which allows third parties to download the articles and share them with others as long as they credit the author and the Abstract Book, but they cannot change the content in any way or use them commercially.
**Abstract Book Citations:** Authors, Title, HemaSphere, 2026;10:(S1):pages. The abstract book DOIs can be found on the "Author Information" page.
**Disclaimer:** Articles published in the journal HemaSphere exclusively reflect the opinions of the authors. The authors are responsible for all content in their abstracts including accuracy of the facts, statements, citing resources, etc.

**Results**

TCB therapy achieved robust tumor control while preserving circulating T-cell numbers and induced immune remodeling distinct from Fc-mediated anti-CD20 depletion. Consistent with direct CD3 engagement, TCB treatment was associated with sustained TCR activation (signal 1) and maintained intratumoral activated CD8+ T cells. TCBs upregulated co-stimulatory receptors, including 4-1BB, and prolonged exposure further promoted effector and central memory differentiation and drove clonal focusing consistent with expansion of tumor antigen–experienced CD8+ populations with memory features. In DLBCL-PDLS, TCB exposure similarly induced CD8+ activation, increased 4-1BB expression, and enhanced cytotoxic programs, supporting translational relevance of sustained signal 1-driven remodeling.

Lymphoma-targeted CD19:4-1BB co-stimulation (signal 2) was ineffective as monotherapy and did not enhance Fc-mediated anti-CD20 therapy, consistent with the requirement for productive TCR signal 1 priming, which was not sustained after CD20+ cell depletion. In contrast, combining 4-1BB co-stimulation with TCB augmented TCB-driven activation, demonstrating synergistic interplay between signal 1 and signal 2 that deepened and prolonged tumor control, improved survival, expanded central memory, enhanced IFNγ effector activity, and reduced immunosuppressive and dysfunctional compartments, including Tregs and exhausted CD8+ states.

**Summary/Conclusion**

Beyond debulking, CD20:CD3 TCBs function as immune-reprogramming agents that sustain tumor-specific CD8+ activation, promote memory differentiation, and reinforce long-term immunologic surveillance in DLBCL. Spatially coordinated 4-1BB co-stimulation potentiates these effects, deepening and prolonging tumor control while mitigating immunosuppressive states. Together, these findings provide a mechanistic rationale for CD20:CD3 × CD19:4-1BB/L combinations to enhance response durability and prolong relapse-free survival.

**Copyright Information:** (Online) ISSN: 2572-9241
©2026 The Author(s). HemaSphere is published by John Wiley & Sons Ltd on behalf of European Hematology Association. This is an open access Abstract Book distributed under the Attribution-NonCommercial-NoDerivs (CC BY-NC-ND), which allows third parties to download the articles and share them with others as long as they credit the author and the Abstract Book, but they cannot change the content in any way or use them commercially.
**Abstract Book Citations:** Authors, Title, HemaSphere, 2026;10:(S1):pages. The abstract book DOIs can be found on the “Author Information” page.
**Disclaimer:** Articles published in the journal HemaSphere exclusively reflect the opinions of the authors. The authors are responsible for all content in their abstracts including accuracy of the facts, statements, citing resources, etc.

### (S253) TUMOR MICROENVIRONMENT WITH VASCULATURE-DRIVEN T-CELL EXHAUSTION IN ANGIOIMMUNOBLASTIC T-CELL LYMPHOMA REVEALED BY SINGLE-CELL SPATIAL MULTI-OMICS

**Topic:** 21. Lymphoma biology & translational research

Yuki Shimizu*[1], Manabu Fujisawa[2, 3], Yoshiaki Abe[3], Yasunori Ota[4], Sakurako Suma[3, 5], Junko Zenkoh[6], Akinori Kanai[6], Atsushi Uehara[5, 7], Ryota Matsuoka[8], Nobuhiko Nakamura[9], Shuku Sato[10], Shinichi Teshima[11], Naoya Nakamura[12], Koji Kato[13], Takeshi Iwasaki[14], Kennosuke Karube[15], Kazuyuki Shimada[16], Kenichi Makishima[5], Yasuhito Suehara[3, 8], Keiichiro Hattori[3, 8], Tatsuhiro Sakamoto[3, 8], Ayako Suzuki[6], Hiroko Bando-Tamura[17], Daisuke Kaji[18], Yoshihito Otsuka[19], Daisuke Matsubara[8], Miwako Kakiuchi[20], Kosei Matsue[7], Shumpei Ishikawa[20], Yutaka Suzuki[6], Christian Steidl[2, 21, 22], Mamiko Sakata-Yanagimoto[1, 3, 5, 22]

*[1]Comprehensive Human Biosciences, University of Tsukuba, Department of Hematology, Tsukuba, Japan;[2]British Columbia Cancer, Centre for Lymphoid Cancer, Vancouver, Canada; [3]Institute of Medicine, University of Tsukuba, Department of Hematology, Tsukuba, Japan; [4]Toranomon Hospital, Department of Pathology, Tokyo, Japan;[5]University of Tsukuba Hospital, Department of Hematology, Tsukuba, Japan; [6]Graduate School of Frontier Sciences, The University of Tokyo, Department of Computational Biology and Medical Sciences, Kashiwa, Japan; [7]Kameda Medical Center, Division of Hematology/Oncology, Department of Internal Medicine, Kamogawa, Japan; [8]Institute of Medicine, University of Tsukuba, Department of Pathology, Tsukuba, Japan; [9]Gifu University Hospital, Department of Hematology and Infectious Disease, Gifu, Japan; [10]Shonan Kamakura General Hospital, Department of Hematology, Kamakura, Japan; [11]Shonan Kamakura General Hospital, Department of Pathology, Kamakura, Japan; [12]Tokai University School of Medicine, Department of Pathology, Isehara, Japan; [13]Kyushu University Graduate School of Medical Science, Department of Medicine and Biosystemic Science, Fukuoka, Japan; [14]Graduate School of Medical Sciences, Kyushu University Graduate School of Medical Sciences, Department of Anatomic Pathology, Fukuoka, Japan; [15]Graduate School of Medicine, Nagoya University, Department of Pathology and Laboratory Medicine, Nagoya, Japan; [16]Nagoya University Graduate School of Medicine, Department of Hematology and Oncology, Nagoya, Japan; [17]Institute of Medicine, University of Tsukuba, Department of Breast and Endocrine Surgery, Tsukuba, Japan;[18]Toranomon Hospital, Department of Hematology, Tokyo, Japan; [19]Kameda Medical Center, Department of Clinical Laboratory, Kamogawa, Japan; [20]Graduate School of Medicine, The University of Tokyo, Department of Preventive Medicine, Tokyo, Japan;[21]University of British Columbia, Department of Pathology and Laboratory Medicine, Vancouver, Canada; [22]Transborder Medical Research Center, University of Tsukuba, Division of Advanced Hemato-Oncology, Tsukuba, Japan;*

### Background

Angioimmunoblastic T cell lymphoma (AITL) is a subtype of peripheral T-cell lymphoma characterized by the proliferation of diverse stromal cell populations, including blood endothelial cells (BECs) and follicular dendritic cells (FDCs). At EHA2025, we reported that stromal cells in AITL can be grouped into 30 distinct phenotypic clusters, including transitional BECs from capillary BECs to activated high endothelial venules (aHEVs), referred to as C-aHEVs. However, the functional relationship of this stromal cell population with other tumor microenvironment compartments, as well as its clinical significance, remains to be elucidated.

### Aims

We aimed to investigate the pathophysiological and clinical impact of stromal heterogeneity within the AITL tumor microenvironment (TME) using single-cell RNA sequencing (scRNA-seq), spatial transcriptomics (ST), and multiplex digital spatial profiling (MDSP).

**Copyright Information:** (Online) ISSN: 2572-9241
©2026 The Author(s). HemaSphere is published by John Wiley & Sons Ltd on behalf of European Hematology Association. This is an open access Abstract Book distributed under the Attribution-NonCommercial-NoDerivs (CC BY-NC-ND), which allows third parties to download the articles and share them with others as long as they credit the author and the Abstract Book, but they cannot change the content in any way or use them commercially.
**Abstract Book Citations:** Authors, Title, HemaSphere, 2026;10:(S1):pages. The abstract book DOIs can be found on the “Author Information” page.
**Disclaimer:** Articles published in the journal HemaSphere exclusively reflect the opinions of the authors. The authors are responsible for all content in their abstracts including accuracy of the facts, statements, citing resources, etc.

### Methods

Non-hematopoietic cells from 10 AITL and 5 metastasis free lymph nodes were sorted using magnetic cell sorting and fluorescence-activated cell sorting, followed by scRNA-seq. We designed two types of gene panels for ST using the 10X Xenium in situ platform: 1) A 298-gene panel to detect the cellular composition of the TME; 2) A 5,000-gene plus add-on gene panel to evaluate the enrichment of specific transcriptional signatures in the TME. For validation, an antibody panel composed of 40 markers was used for MDSP using the PhenoCycler Fusion system. ST was performed on 51 formalin-fixed paraffin-embedded (FFPE) samples, and MDSP was performed on 128 FFPE samples from AITL patients.

### Results scRNA-seq data of 49,363 stromal cells identified 30 distinct subclusters including 10 BEC subclusters. Notably, C-aHEVs were divided into two subclusters: C-aHEV1, which highly expressed a capillary signature, and C-aHEV2, which highly expressed an HEV signature specific to AITL.
The ST analysis, utilizing both gene panels (median cell density: 16,750 and 18,537 cells/mm², respectively), revealed an intricate cellular landscape comprising tumor cells alongside diverse immune subsets—including exhausted T cells ($T_{exh}$), five B-cell subclusters, and three myeloid subclusters—as well as 10 BEC and 6 non-endothelial cell subclusters. Neighborhood analysis identified three tumor-supportive niches: germinal center B cell-enriched, T cell-enriched, and C-aHEV-FDC-enriched. Specifically, C-aHEV1 localized in the closest proximity to tumor cells among all BECs, forming a unique niche with FDCs and $T_{exh}$ (C-aHEV-FDC-enriched niche). Other HEV subtypes, including C-aHEV2, aHEV, and homeostatic HEV were also located closer to tumor cells than other BECs. MDSP, which detected a median cell density of 13,491 cells/mm², identifying tumor cells, 6 BEC subclusters, FDCs, and 10 immune subsets including $T_{exh}$. MDSP results also recapitulated the localization of C-aHEV1 around tumor cells and enrichment of $T_{exh}$ within the C-aHEV-FDC-enriched niche, further supporting the ST results. Finally, analysis of a previously published microarray dataset with survival data revealed that patients with high C-aHEV signature score had significantly shorter overall survival.

### Summary/Conclusion

Using scRNA-seq and spatial multi-omics data, we identified dynamic alterations in BEC composition in the AITL TME, including an increase in C-aHEV2 and tBEC. C-aHEV1 represents a tumor-proximal BEC subcluster that forms a C-aHEV-FDC-enriched niche associated with T cell exhaustion.

**Copyright Information:** (Online) ISSN: 2572-9241
©2026 The Author(s). HemaSphere is published by John Wiley & Sons Ltd on behalf of European Hematology Association. This is an open access Abstract Book distributed under the Attribution-NonCommercial-NoDerivs (CC BY-NC-ND), which allows third parties to download the articles and share them with others as long as they credit the author and the Abstract Book, but they cannot change the content in any way or use them commercially.
**Abstract Book Citations:** Authors, Title, HemaSphere, 2026;10:(S1):pages. The abstract book DOIs can be found on the “Author Information” page.
**Disclaimer:** Articles published in the journal HemaSphere exclusively reflect the opinions of the authors. The authors are responsible for all content in their abstracts including accuracy of the facts, statements, citing resources, etc.

### (S254) HMGA1 STABILIZES MYC PROTEIN TO DRIVE ONCOGENIC TRANSCRIPTIONAL PROGRAMS IN TRANSFORMED FOLLICULAR LYMPHOMA (FL)

**Topic:** 21. Lymphoma biology & translational research

Androniki Michaelou*[1, 2], Daniel Filip[1, 2], Václav Šeda[1, 2], Josef Veceřa[1, 2], Pedro Faria Zeni[1, 2], Miroslav Boudny[1, 2], Kryštof Hlaváč[1, 2], Kvetoslava Matulova[1], Charles Berka[3], David Salek[1], Michael Doubek[1], Leos Kren[1], Marek Mraz[1, 2]

*[1]University Hospital Brno, Brno, Czechia; [2]CEITEC Masaryk University, Brno, Czechia; [3]Palacky University Olomouc, Olomouc, Czechia;*

### Background

FL transformation (tFL) is a clinically aggressive event often driven by increased MYC activity. While *MYC* genetic alterations are present in ~one third of tFL cases, we hypothesised that other transcription factors (TFs) can contribute to MYC activity gain.

### Aims

Identify TFs contributing to MYC-driven tFL.

### Methods

RNA profiling and immunohistochemistry (IHC) in paired FL-tFL samples. Functional studies using CRISPR KO, co-immunoprecipitation (co-IP), and proteasome inhibition.

**Copyright Information:** (Online) ISSN: 2572-9241
©2026 The Author(s). HemaSphere is published by John Wiley & Sons Ltd on behalf of European Hematology Association. This is an open access Abstract Book distributed under the Attribution-NonCommercial-NoDerivs (CC BY-NC-ND), which allows third parties to download the articles and share them with others as long as they credit the author and the Abstract Book, but they cannot change the content in any way or use them commercially.
**Abstract Book Citations:** Authors, Title, HemaSphere, 2026;10:(S1):pages. The abstract book DOIs can be found on the “Author Information” page.
**Disclaimer:** Articles published in the journal HemaSphere exclusively reflect the opinions of the authors. The authors are responsible for all content in their abstracts including accuracy of the facts, statements, citing resources, etc.

### Results

RNA profiling in 11 paired FL-tFL samples identified 1,075 differentially expressed mRNAs. *In silico* regulon enrichment analysis (Viper tool) identified MYC and HMGA1 (a chromatin architectural protein) among the most overexpressed TFs in tFL, together with a highly enriched activation signature (**FIG.A**). Notably, higher *HMGA1* levels correlated with shorter OS in FL (n=129; HR=1.8, p=0.019) and in *de novo* DLBCL (n=415; HR=1.9, p<0.01; **FIG.B**). IHC in 6 FL-tFL pairs revealed concurrent induction of HMGA1 and MYC (fold-change [FC]=2.4 and 2.1, respectively, p<0.05; **FIG.C**). *HMGA1* upregulation was also observed in *de novo* DLBCL (n=15) compared with FL (n=56; FC=1.8, p<0.01). In malignant B cells, *HMGA1* and *MYC* expression was induced by microenvironmental stimuli *in vitro*, such as BCR activation (anti-IgM) or T cell factors (IL-4/CD40L/IL-21 combination; n=4, p<0.01), suggesting that HMGA1 plays a role in B cell activation. However, MYC itself is not regulating HMGA1 levels as *MYC* silencing via siRNA (SuDHL4, DOHH2; n=3) or overexpression (Karpas422; n=3) did not alter *HMGA1* mRNA or protein levels. Notably, *HMGA1* knockout (KO) via CRISPR reduced malignant B cell proliferation by ~30% (**FIG.D**) and increased spontaneous apoptosis (from 3.4% to 11.1%, p<0.05; **FIG.E**). *HMGA1* KO in the SuDHL4 cell line affected the expression of 327 mRNAs and markedly downregulated MYC transcriptional activity (GSEA; FDR<0.01). Notably, *HMGA1* KO reduced MYC protein levels by ~60% across 3 MYC-dependent lymphoma cell lines (SuDHL4, Karpas422, DOHH2) and 2 CLL cell lines (HG3, MEC1), without affecting *MYC* mRNA levels (**FIG.F-G** and not shown). Conversely, *HMGA1* overexpression increased MYC protein levels (MEC1; n=4, FC=1.83, p=0.03), but not *MYC* mRNA levels. Moreover, *HMGA1* re-expression in HMGA1 KO cells rescued MYC levels (SuDHL4). Based on these data, we hypothesised that HMGA1 regulates MYC post-translationally. Indeed, co-IP assays revealed that HMGA1 physically interacts with MYC (**FIG.H**) in a DNA-dependent manner (propidium iodide assay), consistent with *in silico* structural predictions (AlphaFold 3). Importantly, *HMGA1* KO shortened MYC half-life by ~35% after inhibition of *de novo* protein synthesis with cycloheximide (n=3; **FIG.I**). Mechanistically, HMGA1–MYC interaction protected MYC from proteasomal degradation, as proteasome inhibition with MG-132 in *HMGA1* KO cells restored MYC levels (n=3; **FIG.J**), while *HMGA1* KO increased MYC polyubiquitination (a mark for degradation, **FIG.K**). Blocking GSK3β (laduviglusib) in *HMGA1* KO cells also rescued MYC levels (n=5, **FIG.L**), suggesting that the enhanced MYC ubiquitination upon *HMGA1* depletion is GSK3β-regulated.

### Summary/Conclusion

HMGA1 overexpression in tFL stabilises MYC levels by preventing its proteasomal degradation via the GSK3β pathway (**FIG.M**). Overall, this represents a novel mechanism leading to MYC gain in tFL.

Supported by the Ministry of Health of the Czech Rep. (NU22-03-00117, NW24-03-00369), Czech Science Foundation (25-15368X), MH CZ-DRO (FNBr,65269705), MUNI/A/1733/2025.

**Copyright Information:** (Online) ISSN: 2572-9241
©2026 The Author(s). HemaSphere is published by John Wiley & Sons Ltd on behalf of European Hematology Association. This is an open access Abstract Book distributed under the Attribution-NonCommercial-NoDerivs (CC BY-NC-ND), which allows third parties to download the articles and share them with others as long as they credit the author and the Abstract Book, but they cannot change the content in any way or use them commercially.
**Abstract Book Citations:** Authors, Title, HemaSphere, 2026;10:(S1):pages. The abstract book DOIs can be found on the "Author Information" page.
**Disclaimer:** Articles published in the journal HemaSphere exclusively reflect the opinions of the authors. The authors are responsible for all content in their abstracts including accuracy of the facts, statements, citing resources, etc.

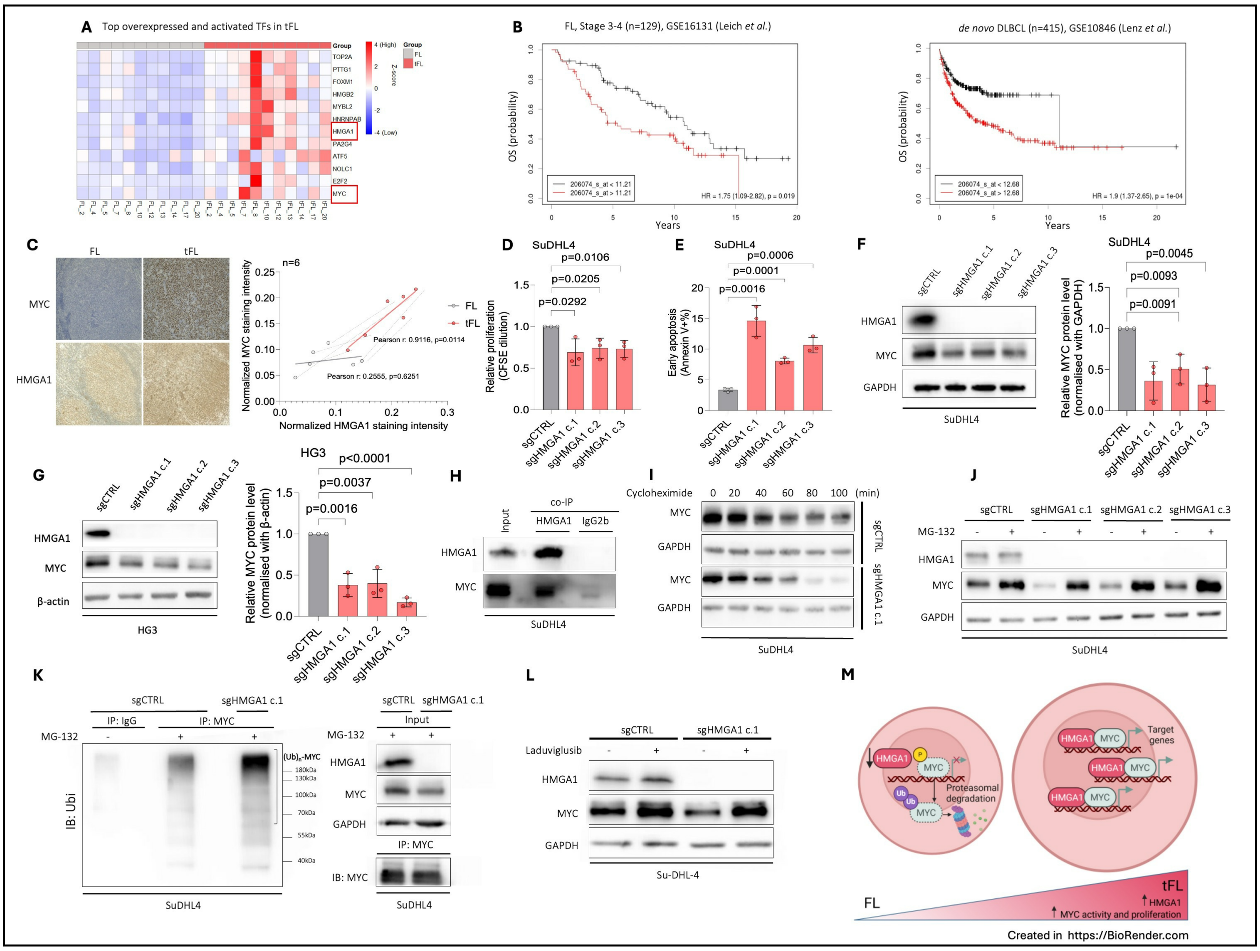

**Copyright Information:** (Online) ISSN: 2572-9241
©2026 The Author(s). HemaSphere is published by John Wiley & Sons Ltd on behalf of European Hematology Association. This is an open access Abstract Book distributed under the Attribution-NonCommercial-NoDerivs (CC BY-NC-ND), which allows third parties to download the articles and share them with others as long as they credit the author and the Abstract Book, but they cannot change the content in any way or use them commercially.
**Abstract Book Citations:** Authors, Title, HemaSphere, 2026;10:(S1):pages. The abstract book DOIs can be found on the "Author Information" page.
**Disclaimer:** Articles published in the journal HemaSphere exclusively reflect the opinions of the authors. The authors are responsible for all content in their abstracts including accuracy of the facts, statements, citing resources, etc.

### (S255) DYNAMICS AND CLINICAL IMPACT OF CLONAL HEMATOPOIESIS IN DIFFUSE LARGE B-CELL LYMPHOMA THROUGH MULTI-COMPARTMENT GENOMIC PROFILING

**Topic:** 21. Lymphoma biology & translational research

Nawar Maher*[1], Samir Mouhssine[1], Mohammad Almasri[1], Chiara Cosentino[1], Luca Cividini[1], Riccardo Bruna[1], Rita Tavarozzi[1], Riccardo Dondolin[1], Matteo Bellia[1], Maria Chiara La Licata[1], Jana Nabki[1], Giovanni Manfredi Assanto[2], Bashar Al Deeban[1], Joseph Ghanej[1], Francesca Maiellaro[1], Monica Leutner[3], Annalisa Andorno[4], Francesca Mercalli[4], Abdurraouf Mokhtar Saleh Mahmoud[1], Ndeye Marie Diop[1], Clara Deambrogi[1], Silvia Rasi[1], Alice Di Rocco[2], Ilaria Del Giudice[2], Maurizio Martelli[2], Robin Foà[2], Renzo Boldorini[4], Marco Ladetto[1], Gianluca Gaidano[1], Riccardo Moia[1]

*[1]Division of Hematology, Department of Translational Medicine, Università del Piemonte Orientale, Alessandria and Novara, Italy; [2]Division of Hematology, Department of Translational and Precision Medicine, 'Sapienza' University, Rome, Italy;[3]Division of Pathology, Azienda Sanitaria Locale del VCO, Verbania, Italy; [4]Division of Pathology, Department of Health Sciences, Università del Piemonte Orientale, Novara, Italy;*

### Background

Clonal hematopoiesis (CH) in diffuse large B-cell lymphoma (DLBCL) has been insufficiently characterized across compartments and over time, limiting understanding of its biological and clinical relevance.

### Aims

*i*) Characterize CH from the myeloid compartment and plasma cfDNA; *ii*) evaluate the clinical impact of CH in newly diagnosed DLBCL and its association with disease molecular subtypes; *iii*) assess the clonal dynamics of CH at baseline and at end of treatment (EOT); *iv*) determine the prevalence and clinical significance of tumor infiltrating (TI)-CH in DLBCL.

### Methods

We analyzed 453 samples from 157 newly diagnosed DLBCL patients treated with R-CHOP–like regimens using CAPP-Seq–based NGS targeting 28 CH-related genes. Samples included peripheral blood (PB) Ficoll isolated granulocytes (n=151), serial plasma cfDNA [n=107 baseline, n=47 at cycle (C) 3, and n=92 at EOT], and lymph node biopsies (n=56). Clonal fitness was estimated from weighted regression on logit-transformed VAF (Fig. 1E). DLBclass clustering was available for 102 patients.

**Copyright Information:** (Online) ISSN: 2572-9241
©2026 The Author(s). HemaSphere is published by John Wiley & Sons Ltd on behalf of European Hematology Association. This is an open access Abstract Book distributed under the Attribution-NonCommercial-NoDerivs (CC BY-NC-ND), which allows third parties to download the articles and share them with others as long as they credit the author and the Abstract Book, but they cannot change the content in any way or use them commercially.
**Abstract Book Citations:** Authors, Title, HemaSphere, 2026;10:(S1):pages. The abstract book DOIs can be found on the "Author Information" page.
**Disclaimer:** Articles published in the journal HemaSphere exclusively reflect the opinions of the authors. The authors are responsible for all content in their abstracts including accuracy of the facts, statements, citing resources, etc.

**Results**

Median age of the cohort was 67 years. CH was detected in the granulocyte compartment in 37.7% of patients, with *DNMT3A* (20.5%) and *TET2* (14.6%) as the most frequently mutated genes (Fig. 1A). Among CH$^{+}$ patients, *DNMT3A* mutations were significantly enriched in the C3 molecular subtype of DLBCL (*p*=0.005) (Fig. 1B). Both *DNMT3A* and *TET2* mutations significantly associated with an increased risk of second primary malignancies (5-year CIF: 7.1 vs 16.3, *P*=0.03; 7.3 vs 19.4, *p*<0.001). In plasma, cfDNA sequencing detected 78 of 80 CH granulocyte mutations (97.5% sensitivity), with strong VAF correlation between compartments (r=0.89, *p*<0.001) (Fig. 1C). The most common mutations were *DNMT3A* (22.4%) and *TET2* (15.0%). In PB, CH$^{+}$ patients demonstrated marginally reduced PFS (55.8% vs. 64.1%) and OS (69.8% vs. 72.4%) relative to CH cases, without however reaching statistical significance (*p*=0.547 and *P*=0.466, respectively). At EOT, the prevalence of CH increased from 34.7% at baseline to 50.5% (*p*<0.001). The median VAF also rose significantly, from 1.44% to 2.44% ( *p*<0.001). Notably, mutations of *PPM1D* were mostly undetectable before treatment but emerged following chemoimmunotherapy (CIT), increasing from 1.1% baseline cases to 16.3% EOT cases and showing the highest inferred clonal fitness (Fig. 1D). All *PPM1D* mutations were already detectable after C3, with higher clonal fitness from baseline to C3 compared to C3 to EOT, suggesting an early CIT-driven bottleneck effect ( *p*=0.03). In tissue biopsies, at least one TI-CH mutation occurred in 42.9% of patients, most commonly involving *DNMT3A* (21.4%, VAF range 0.4–27.0) and *TET2* (16.1%, VAF range 0.4–27.4), almost mutually exclusive. At a median follow-up of 69 months, TI- *DNMT3A*$^{+}$ patients had a significantly inferior PFS (33.3% vs 70.9%, *p*=0.009) and OS (41.7% vs 83.7%, *p*<0.001) compared to TI-*DNMT3A*$^{-}$ cases. This adverse impact remained significant after adjusting for the international prognostic index (Fig. 1E).

**Summary/Conclusion**

Multi-compartment profiling shows that CH in DLBCL is frequent and detectable in multiple compartments, enriched in specific molecular subtypes, and associated with an increased risk of second primary malignancies. R-CHOP–based therapy promotes expansion of CH clones, including early emergence of *PPM1D* mutations. While CH in PB did not significantly affect survival, TI-CH *DNMT3A* mutations emerged as an independent predictor of inferior PFS and OS in DLBCL treated with CIT.

**Copyright Information:** (Online) ISSN: 2572-9241
©2026 The Author(s). HemaSphere is published by John Wiley & Sons Ltd on behalf of European Hematology Association. This is an open access Abstract Book distributed under the Attribution-NonCommercial-NoDerivs (CC BY-NC-ND), which allows third parties to download the articles and share them with others as long as they credit the author and the Abstract Book, but they cannot change the content in any way or use them commercially.
**Abstract Book Citations:** Authors, Title, HemaSphere, 2026;10:(S1):pages. The abstract book DOIs can be found on the “Author Information” page.
**Disclaimer:** Articles published in the journal HemaSphere exclusively reflect the opinions of the authors. The authors are responsible for all content in their abstracts including accuracy of the facts, statements, citing resources, etc.

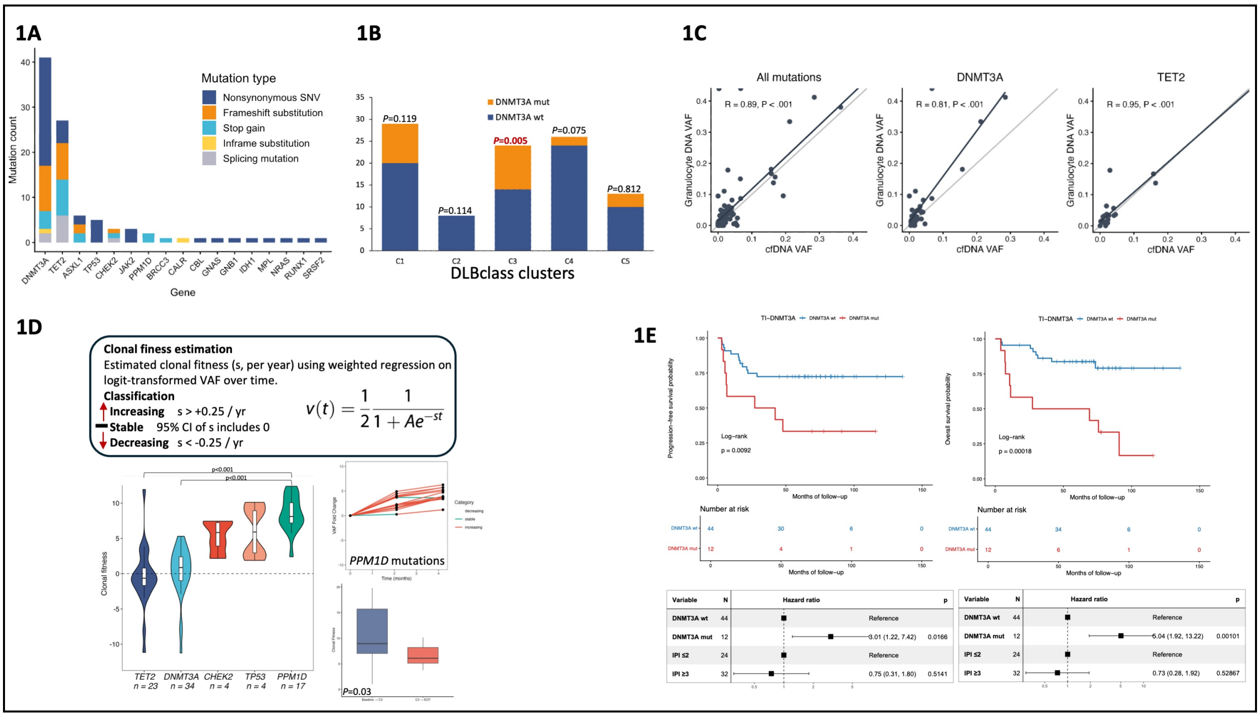

1A: Number and spectrum of clonal hematopoiesis (CH) mutations in the granulocyte compartment; 1B: Distribution of DNMT3A-mutated versus wild-type cases across DLBCL molecular clusters (C1–C5); 1C: Comparison of CH mutations detected in granulocytes and cfDNA; 1D: Clonal fitness analysis demonstrating gene-specific expansion dynamics of CH mutations over time; 1E: Kaplan–Meier survival curves and multivariable Cox regression analysis according to tumor-infiltrating DNMT3A mutation status.

**Copyright Information:** (Online) ISSN: 2572-9241
©2026 The Author(s). HemaSphere is published by John Wiley & Sons Ltd on behalf of European Hematology Association. This is an open access Abstract Book distributed under the Attribution-NonCommercial-NoDerivs (CC BY-NC-ND), which allows third parties to download the articles and share them with others as long as they credit the author and the Abstract Book, but they cannot change the content in any way or use them commercially.
**Abstract Book Citations:** Authors, Title, HemaSphere, 2026;10:(S1):pages. The abstract book DOIs can be found on the "Author Information" page.
**Disclaimer:** Articles published in the journal HemaSphere exclusively reflect the opinions of the authors. The authors are responsible for all content in their abstracts including accuracy of the facts, statements, citing resources, etc.

### (S256) CIRCULATING TUMOR DNA REVEALS LYMPHOMA-ASSOCIATED MUTATIONS LONG BEFORE CLINICAL DIAGNOSIS

**Topic:** 21. Lymphoma biology & translational research

Nick Veltmaat*[1, 2], Yujie Zhong[1, 2], Isabelle van Zeventer[1], Joost Kluiver[2, 3], Arjan Diepstra[2, 3], Anke van den Berg[2, 3], Wouter Plattel[1, 2], Marcel Nijland[1, 2]

*[1]University Medical Center Groningen, Hematology, Groningen, The Netherlands; [2]University Medical Center Groningen, Lymphoma Research Groningen, Groningen, The Netherlands; [3]University Medical Center Groningen, Pathology & Medical Biology, Groningen, The Netherlands;*

### Background

The interval between early lymphoma-driving events and clinical presentation is poorly defined. Diffuse large B-cell lymphoma (DLBCL) and classic Hodgkin lymphoma (cHL) arise from germinal-center (GC) B cells and carry recurrent genetic alterations. Incidental findings from noninvasive prenatal testing and population biobanks have identified tumor-specific somatic variants in plasma prior to cHL diagnosis. In cHL, serum thymus and activation-regulated chemokine (sTARC), a validated biomarker, is elevated long before symptom onset. Aberrant somatic hypermutation produces clustered in-cis substitutions, known as multi-nucleotide variants (MNVs), which frequently affect super-enhancers (SE) and other key B-cell regulating loci early in lymphomagenesis. MNVs provide a distinct mutational footprint that may enable highly sensitive tumor detection in cfDNA in pre-diagnostic plasma. We hypothesize that cfDNA analysis can capture these oncogenic events and map the temporal trajectory of lymphomagenesis.

### Aims

By integrating single nucleotide variant (SNV) and MNV calling, we aim to identify early molecular aberrations preceding clinical diagnosis of DLBCL and cHL.

### Methods

We analyzed pre-diagnostic plasma from the Dutch Lifelines biobank in a retrospective case-cohort of 33 DLBCL and 13 cHL patients, including matched diagnostic tumor tissue samples and white blood cells for 41 cases. Targeted sequencing of 115 genes (~391 kbp), including SEs of 14 genes was performed. SNV analysis was restricted to DLBCL, as tumor cell content in cHL tissue is too low to reliably call SNVs. MNVs were called using our in-house developed MNVista tool.

### Results

Overall, pre-diagnostic ctDNA was detected in 31/46 (67%) cases. The median pre-diagnostic plasma sample was drawn 21 months prior to diagnosis (DLBCL range = 1–106 months, cHL range = 3–55 months). Tumor-informed pre-diagnostic detection of SNVs indicated presence of ctDNA in 13/33 DLBCL patients (39%). MNVs were detected in pre-diagnostic ctDNA in 20/33 (61%) DLBCL and 9/13 (69%) cHL patients. Both MNVs and SNVs were identified in 11/33 DLBCL cases, while only MNVs were identified in 9/33 and only SNVs in 2/33 cases. The earliest pre-diagnostic time point with detectable ctDNA was 106 months) and 55 months before diagnosis of DLBCL and cHL, respectively (Figure 1A). In cHL, pre-diagnostic ctDNA levels (hGE/mL) from MNVs correlated strongly with sTARC ($r = 0.86$, $p<0.001$). Pre-treatment plasma samples that were ctDNA-positive were collected significantly closer to diagnosis (median 17, range 1–106 months) than ctDNA-negative samples (median 33, range 50–3 months) (Figure 1B). In line with these observations, ctDNA levels significantly correlated with sampling timepoint (Figure 1C). Assessment of the ctDNA levels over time indicated that tumor cells of DLBCL and cHL expanded at a similar rate.

**Copyright Information:** (Online) ISSN: 2572-9241
©2026 The Author(s). HemaSphere is published by John Wiley & Sons Ltd on behalf of European Hematology Association. This is an open access Abstract Book distributed under the Attribution-NonCommercial-NoDerivs (CC BY-NC-ND), which allows third parties to download the articles and share them with others as long as they credit the author and the Abstract Book, but they cannot change the content in any way or use them commercially.
**Abstract Book Citations:** Authors, Title, HemaSphere, 2026;10:(S1):pages. The abstract book DOIs can be found on the "Author Information" page.
**Disclaimer:** Articles published in the journal HemaSphere exclusively reflect the opinions of the authors. The authors are responsible for all content in their abstracts including accuracy of the facts, statements, citing resources, etc.

### Summary/Conclusion

Summary/Conclusion: ctDNA can be detected years prior to B-cell lymphoma diagnosis. In total, 31/46 cases had detectable ctDNA in pre-diagnostic plasma samples. Our data show that MNV-approaches are sensitive and can be used for pre-diagnostic ctDNA detection in a tumor-informed setting. They provide novel insight in the rise of early molecular aberrations in DLBCL and cHL and support the use of ctDNA as liquid biopsy to aid in early diagnosis of patients suspected of lymphoma.

**Copyright Information:** (Online) ISSN: 2572-9241
©2026 The Author(s). HemaSphere is published by John Wiley & Sons Ltd on behalf of European Hematology Association. This is an open access Abstract Book distributed under the Attribution-NonCommercial-NoDerivs (CC BY-NC-ND), which allows third parties to download the articles and share them with others as long as they credit the author and the Abstract Book, but they cannot change the content in any way or use them commercially.
**Abstract Book Citations:** Authors, Title, HemaSphere, 2026;10:(S1):pages. The abstract book DOIs can be found on the "Author Information" page.
**Disclaimer:** Articles published in the journal HemaSphere exclusively reflect the opinions of the authors. The authors are responsible for all content in their abstracts including accuracy of the facts, statements, citing resources, etc.

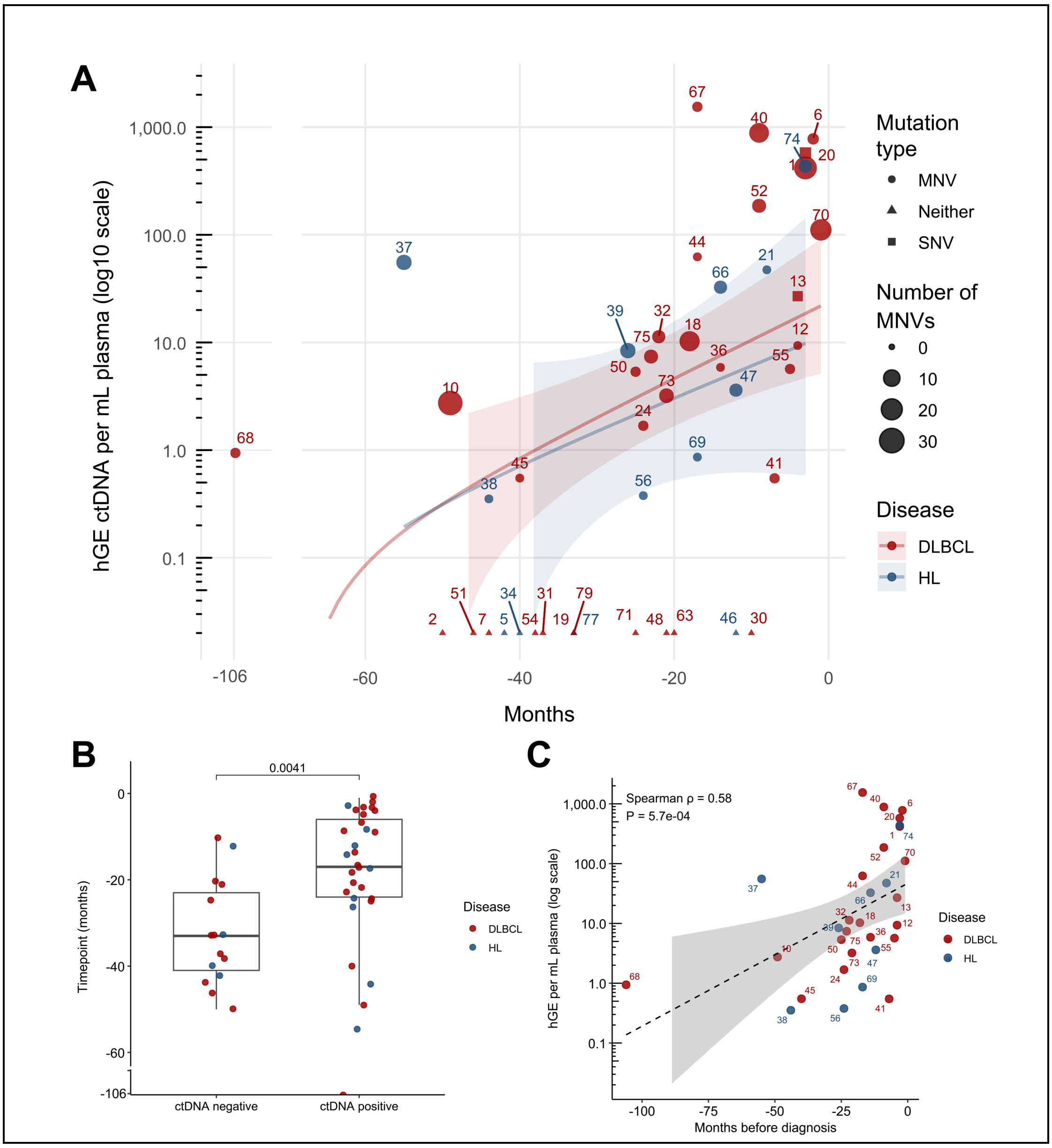

Figure 1: Pre-diagnostic circulating tumor DNA (ctDNA) detection in diffuse large B-cell lymphoma (DLBCL) and Hodgkin lymphoma (HL) patients. A) Haploid genome equivalents (hGE) of circulating tumor DNA (ctDNA) per mL plasma by analyzing multi-nucleotide variants (MNVs) over time per disease in both diffuse large B-cell lymphoma (DLBCL) and Hodgkin lymphoma (HL) patients. Each point represents an individual pre-diagnosis patient sample (patient number shown next to each point). The point size is proportional to the number of MNVs detected in the sample. X-axis: timepoint of the pre-treatment plasma sample in months before diagnosis. Y-axis: hGE per mL plasma (log10 scale). Solid lines represent per-disease fitted trends (predicted hGE) obtained from linear regression on the log scale, accompanied by shaded ribbons which represent the 95% confidence intervals. An x-axis break between −100 and −70 was introduced to improve readability. B) Association between ctDNA status and timepoint relative to diagnosis. Box-and-jitter plot showing the distribution of pre-treatment plasma sample timepoints (months) in patients with detectable circulating tumor DNA (ctDNA positive) versus those without detectable ctDNA (ctDNA negative). Each dot represents an individual patient sample. Boxes indicate the interquartile range (IQR), horizontal lines represent the median, and whiskers extend to 1.5× IQR. Statistical comparison between groups was performed using a Wilcoxon rank-sum test (two-sided), with the p-value shown above the plot (overall ctDNA negative vs ctDNA positive p = 0.0041). C) Scatterplot depicting the correlation (Spearman) between plasma sampling timepoint and ctDNA level in hGE/mL plasma. In all panels, disease type is depicted by color (DLBCL in red, cHL in blue).

**Copyright Information:** (Online) ISSN: 2572-9241
©2026 The Author(s). HemaSphere is published by John Wiley & Sons Ltd on behalf of European Hematology Association. This is an open access Abstract Book distributed under the Attribution-NonCommercial-NoDerivs (CC BY-NC-ND), which allows third parties to download the articles and share them with others as long as they credit the author and the Abstract Book, but they cannot change the content in any way or use them commercially.
**Abstract Book Citations:** Authors, Title, HemaSphere, 2026;10:(S1):pages. The abstract book DOIs can be found on the "Author Information" page.
**Disclaimer:** Articles published in the journal HemaSphere exclusively reflect the opinions of the authors. The authors are responsible for all content in their abstracts including accuracy of the facts, statements, citing resources, etc.

### (S257) DIAGNOSING PRIMARY CENTRAL NERVOUS SYSTEM LYMPHOMA THROUGH MOLECULAR AND IMMUNE BIOMARKERS IN CEREBROSPINAL FLUID: A MULTICENTRE PROSPECTIVE COHORT STUDY (PRICELUS)

**Topic:** 19. Large B cell lymphomas - Clinical

Josephus van Rooij*[1, 2], Peter Wessels[2], Johanna Niers[3], Jacoline Bromberg[4], Johanna Gijtenbeek[5], Rick van Haastert[2], Henk Ruven[2], Bob Meek[2], Marcel Verbeek[5], Nienke Solleveld-Westerink[1], Wendy de Leng[1], Angelika Mühlebner[1], Anton Langerak[4], Sharon Berendsen[1], Edward Knol[1], Hanna van Alkemade[2], Tatjana Seute[1], Monique Minnema[1], Stefan Nierkens[6], Tom Snijders[1]

*[1]Universitair Medisch Centrum Utrecht, Utrecht, The Netherlands;[2]St Antonius Hospital, Utrecht, The Netherlands;[3]Vrije Universiteit Amsterdam, Amsterdam, The Netherlands; [4]Erasmus University Medical Center, Rotterdam, The Netherlands; [5]Radboud University Medical Center, Nijmegen, The Netherlands;[6]Prinses Máxima Centrum Voor Kinderoncologie, Utrecht, The Netherlands;*

### Background

Primary central nervous system lymphoma (PCNSL) is typically diagnosed by brain biopsy, a procedure with risks and diagnostic delay. Emerging cerebrospinal fluid (CSF) biomarkers, including the MYD88 L265P mutation in CSF (MYD88-CSF) and several cytokines, may improve diagnostic accuracy, but evidence is mostly derived from retrospective, non-consecutive cohorts. We conducted a nationwide, multicentre, prospective cohort study to validate the diagnostic utility of multiple CSF biomarkers in patients with suspected PCNSL.

### Aims

To prospectively validate the diagnostic performance of CSF biomarkers in suspected PCNSL and to construct a high-specificity diagnostic algorithm, with additional exploratory analyses of prognostic value, repeated lumbar puncture, and radiological features.

### Methods

Patients with suspected PCNSL who underwent CSF analysis were prospectively enrolled across five hospitals in the Netherlands between 2021 and 2025. Analyses focused on MYD88-CSF and cytokines with reported diagnostic value. Multivariable and LASSO regression were used to construct a diagnostic panel, followed by decision tree analysis to evaluate diagnostic performance. Exploratory analyses assessed associations between novel biomarkers and clinical outcomes. We also evaluated the diagnostic yield of repeated lumbar puncture and examined the relationship between radiological characteristics and CSF biomarker levels.

**Copyright Information:** (Online) ISSN: 2572-9241
©2026 The Author(s). HemaSphere is published by John Wiley & Sons Ltd on behalf of European Hematology Association. This is an open access Abstract Book distributed under the Attribution-NonCommercial-NoDerivs (CC BY-NC-ND), which allows third parties to download the articles and share them with others as long as they credit the author and the Abstract Book, but they cannot change the content in any way or use them commercially.
**Abstract Book Citations:** Authors, Title, HemaSphere, 2026;10:(S1):pages. The abstract book DOIs can be found on the "Author Information" page.
**Disclaimer:** Articles published in the journal HemaSphere exclusively reflect the opinions of the authors. The authors are responsible for all content in their abstracts including accuracy of the facts, statements, citing resources, etc.

### Results

Of 255 enrolled patients, 50 (20%) were diagnosed with CNS lymphoma (CNSL). In univariable analyses, six cytokines showed significant diagnostic value (area under the curve 0.67-0.96). MYD88-CSF was detected in 56% of CNSL cases, with 99% specificity. Three non-CNSL patients tested positive for MYD88-CSF and were diagnosed with lymphoplasmacytic lymphoma (concurrent with glioblastoma), light-chain amyloidosis–associated neuropathy, and IgM MGUS–associated CIDP. In the multivariable logistic regression model, MYD88-CSF, IL10, and CXCL13 remained independently associated with CNSL diagnosis. The combination of MYD88-CSF with elevated IL10 and/or CXCL13 achieved 100% specificity (sensitivity 54%). A negative result for all three biomarkers provided a negative predictive value of 99.4%. Fifty-six patients were classified as indeterminate, as they did not meet PRICELUS positivity criteria (MYD88-CSF combined with IL10 and/or CXCL13), despite at least one positive marker. Of these, 20 (36%) had CNSL. In our cohort, 45% (13/29) of CNSL biopsies could potentially have been avoided based on positive CSF markers, whereas 55% (16/29) yielded indeterminate (n=15) or negative (n=1) results. In a subgroup analysis restricted to patients with intracerebral mass lesions on neuroimaging, results were similar. The added diagnostic value of repeated lumbar puncture was limited. No clear association was observed between radiological characteristics, including tumour contact with the ventricular system, and CSF biomarker levels. Analysis of the prognostic value of the CSF biomarkers is ongoing and will be presented.

### Summary/Conclusion

In this prospective, multicentre cohort of patients with suspected PCNSL, we validated the high diagnostic value of MYD88-CSF, IL10, and CXCL13. In selected clinical scenarios, combined CSF biomarker testing may be sufficient for a diagnosis, without the need for brain biopsy, although results should be interpreted within the full clinical context. For patients not fulfilling the CSF biomarker threshold diagnostic brain biopsy remains necessary.

**Copyright Information:** (Online) ISSN: 2572-9241
©2026 The Author(s). HemaSphere is published by John Wiley & Sons Ltd on behalf of European Hematology Association. This is an open access Abstract Book distributed under the Attribution-NonCommercial-NoDerivs (CC BY-NC-ND), which allows third parties to download the articles and share them with others as long as they credit the author and the Abstract Book, but they cannot change the content in any way or use them commercially.
**Abstract Book Citations:** Authors, Title, HemaSphere, 2026;10:(S1):pages. The abstract book DOIs can be found on the "Author Information" page.
**Disclaimer:** Articles published in the journal HemaSphere exclusively reflect the opinions of the authors. The authors are responsible for all content in their abstracts including accuracy of the facts, statements, citing resources, etc.

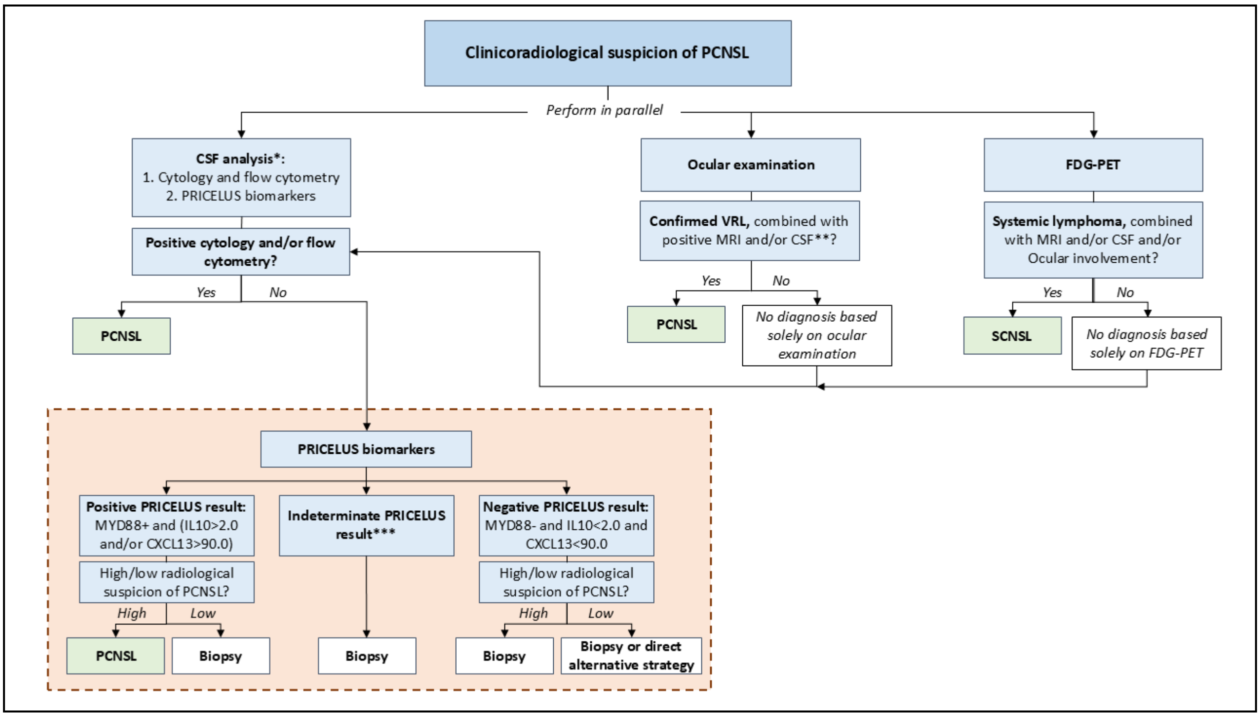

Diagnostic workflow incorporating the PRICELUS algorithm for suspected PCNSL. * CSF analysis consists of cytology, flow cytometry, and PRICELUS biomarker assessment performed in parallel. ** Diagnosis of PCNSL established upon confirmation of vitreoretinal lymphoma in ocular tissue in patients with MRI features typical of PCNSL, or CSF findings consistent with monoclonal B-cell expansion by immunophenotyping and/or malignant cells on cytology; role of PRICELUS biomarkers unknown. *** Indeterminate PRICELUS result: positive MYD88 with IL10 ≤2·0 and CXCL13 ≤90·0, or IL10 >2·0 and/or CXCL13 >90·0 without MYD88 positivity.

**Copyright Information:** (Online) ISSN: 2572-9241
©2026 The Author(s). HemaSphere is published by John Wiley & Sons Ltd on behalf of European Hematology Association. This is an open access Abstract Book distributed under the Attribution-NonCommercial-NoDerivs (CC BY-NC-ND), which allows third parties to download the articles and share them with others as long as they credit the author and the Abstract Book, but they cannot change the content in any way or use them commercially.
**Abstract Book Citations:** Authors, Title, HemaSphere, 2026;10:(S1):pages. The abstract book DOIs can be found on the "Author Information" page.
**Disclaimer:** Articles published in the journal HemaSphere exclusively reflect the opinions of the authors. The authors are responsible for all content in their abstracts including accuracy of the facts, statements, citing resources, etc.

### (S258) INTEGRATED CLINICAL AND GENETIC SCORING REFINES PROGNOSIS IN DARK ZONE SIGNATURE-POSITIVE (DZSIGPOS) DIFFUSE LARGE B-CELL LYMPHOMA: THE DARK ZONE PROGNOSTIC INDEX (DZPI)

**Topic:** 19. Large B cell lymphomas - Clinical

Jinhua Liang*[1], Guozhong Xi[1], Kai-Xin Du[1], Chunyu Shang[1], Yi-Fan Wu[1], Li Wang[1], Jianyong Li[1], Wei Xu[1]

[1]*the First Affiliated Hospital of Nanjing Medical University, Jiangsu Province Hospital, Nanjing, China;*

### Background

The dark zone signature (DZsig) stems from transcriptional differences between high-grade B-cell lymphomas with MYC and BCL2 rearrangements and other germinal centre B-cell-like (GCB) diffuse large B-cell lymphomas (DLBCL). Around 20% of GCB-DLBCLs are DZsig$^{pos}$ (Ennishi et al., J Clin Oncol, 2019; Alduaij et al., Blood, 2023). DZsig$^{pos}$ cases with TP53 abnormalities show poor survival, whereas those without exhibit favourable outcomes, reflecting prognostic heterogeneity. DZsig is considered a refinement of cell-of-origin (COO) classification; however, the drivers of its heterogeneous prognosis remain unclear.

### Aims

To develop and validate a prognostic scoring system for risk-stratifying DZsig$^{pos}$-DLBCL.

### Methods

By calculating the 104 gene DZsig score for RNAseq data, DZsig$^{pos}$ cases were identified from an integrated public cohort (n=238) (Chapuy B et al., REMoDL-B, Lacy S et al., NCI, BCC cohorts) and our centre's cohort (n=21). Excluding BCC, the remaining public cohorts served as the training set (n=114). Immune infiltration gene sets were sourced from Tumuluru et al., Blood, 2025.

### Results

DLBCL90 expression across four refined COO subtypes showed distinct DZsig$^{pos}$ profiles, confirming classification accuracy **(Fig. 1A)**. DZsig$^{pos}$ cases were enriched for the EZB LymphGen subtype **(Fig. 1B)** and for the Depleted subtype within Kotlov LME **(Fig. 1C)**. Patients with activated B-cell-like (ABC) and DZsig$^{pos}$ subtypes showed significantly poorer prognosis **(Fig. 1D–E)**.
Univariable analysis in the training set found prognosis varied by IPI score, its components, and specific gene mutations **(Fig. 1F–H)**.
A multivariate Cox model integrated these factors **(Fig. 1I)**. Scores were assigned: IPI (0=Low, 1=Intermediate, 2=High) +1 for TP53/MYC mutation each. Scores 0-1 (low-risk, n=59) and 2-4 (high-risk, n=55) defined DZPI categories. The DZPI demonstrated the strongest discriminative power in receiver operating characteristic (ROC) analysis **(Fig. 1J)**. There was a significant difference in overall survival (OS) between the categories **(Fig. 1K)**, replicated in both the BCC and our centre's cohort **(Fig. 1L)**.
We subsequently focused on elucidating mechanistic distinctions between DZPI risk categories. Mutations in BCL2 and IL4R occurred with significantly greater frequency in the high-risk category **(Fig. 1M)**. Transcriptomic profiling revealed upregulation of pathways related to tumour and metabolism, concurrent with downregulation of immune-associated pathways **(Fig. 1N)**. Immune cell infiltration analysis indicated significantly reduced enrichment scores for multiple immune cell subsets in the high-risk category **(Fig. 1O)**.
To explore the interplay between the immune microenvironment, DZPI stratification, and prognostic implications, the immune microenvironment of DZsig$^{pos}$ cases underwent reclustering **(Fig. 1P)**. High-risk category had more cold cluster cases **(Fig. 1Q)**. Patients designated as high-risk by DZPI and concurrently assigned to the cold immune cluster experienced the poorest prognosis **(Fig. 1R–S)**.

**Copyright Information:** (Online) ISSN: 2572-9241
©2026 The Author(s). HemaSphere is published by John Wiley & Sons Ltd on behalf of European Hematology Association. This is an open access Abstract Book distributed under the Attribution-NonCommercial-NoDerivs (CC BY-NC-ND), which allows third parties to download the articles and share them with others as long as they credit the author and the Abstract Book, but they cannot change the content in any way or use them commercially.
**Abstract Book Citations:** Authors, Title, HemaSphere, 2026;10:(S1):pages. The abstract book DOIs can be found on the "Author Information" page.
**Disclaimer:** Articles published in the journal HemaSphere exclusively reflect the opinions of the authors. The authors are responsible for all content in their abstracts including accuracy of the facts, statements, citing resources, etc.

### Summary/Conclusion

By integrating clinical and genetic factors, the DZPI stratifies DZsig$^{pos}$-DLBCL into prognostic categories validated in a public cohort and our centre, with the high-risk category associated with a cold immune cluster and inferior survival to inform personalised therapy.

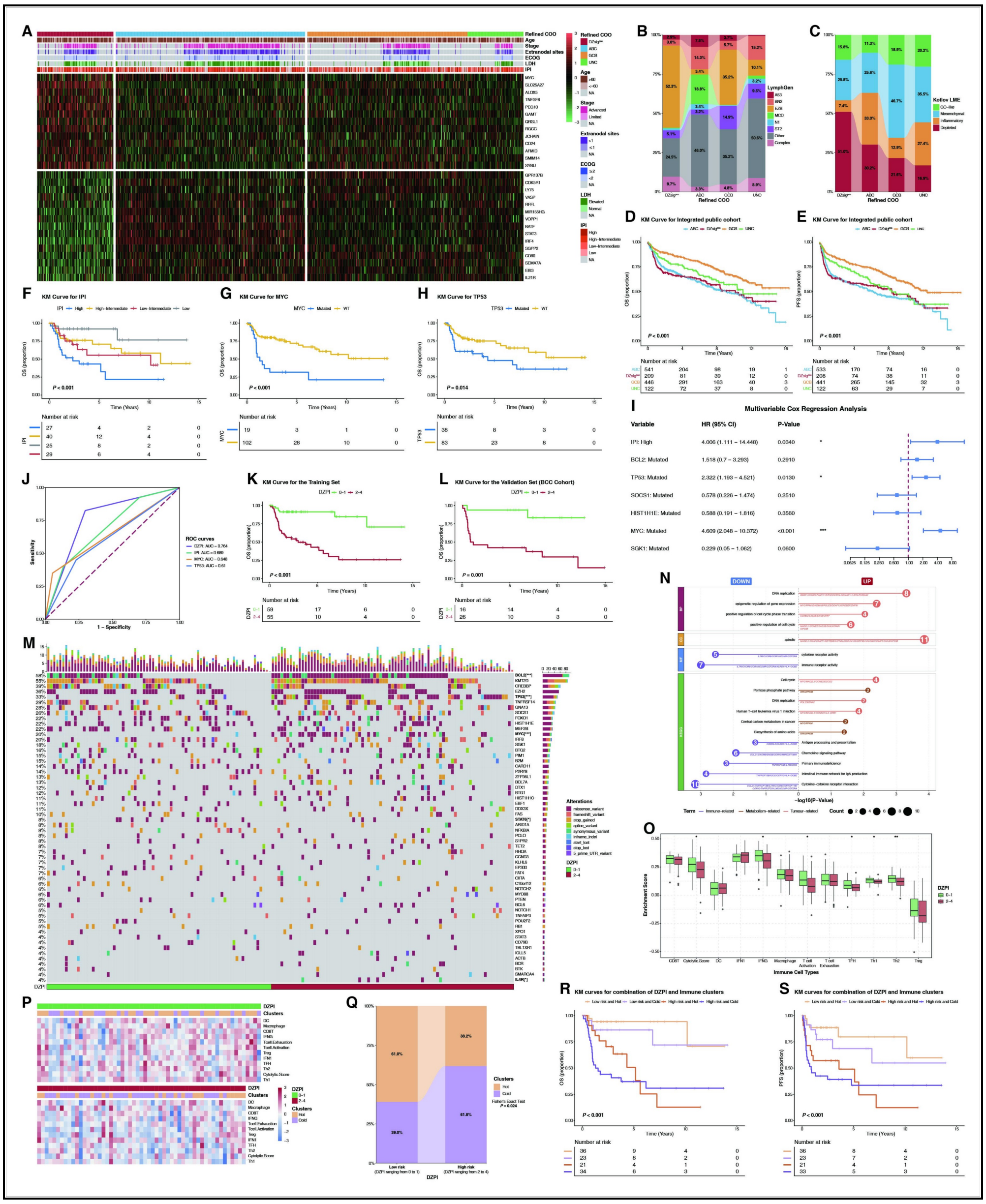

**Copyright Information:** (Online) ISSN: 2572-9241
©2026 The Author(s). HemaSphere is published by John Wiley & Sons Ltd on behalf of European Hematology Association. This is an open access Abstract Book distributed under the Attribution-NonCommercial-NoDerivs (CC BY-NC-ND), which allows third parties to download the articles and share them with others as long as they credit the author and the Abstract Book, but they cannot change the content in any way or use them commercially.
**Abstract Book Citations:** Authors, Title, HemaSphere, 2026;10:(S1):pages. The abstract book DOIs can be found on the "Author Information" page.
**Disclaimer:** Articles published in the journal HemaSphere exclusively reflect the opinions of the authors. The authors are responsible for all content in their abstracts including accuracy of the facts, statements, citing resources, etc.

### (S259) MOLECULAR SURVEILLANCE OF AGGRESSIVE LARGE B-CELL LYMPHOMA USING CIRCULATING TUMOR DNA DUPLEXES

**Topic:** 19. Large B cell lymphomas - Clinical

Sirpa Leppä*[1], Leo Meriranta[1], Maare Arffman[1], Judit Jørgensen[2], Kristina Drott[3], Øystein Fluge[4], Sirkku Jyrkkiö[5], Peter Brown[6], Harald Holte[7]

*[1]University of Helsinki and Helsinki University Hospital, HELSINKI, Finland;[2]Aarhus University Hospital, Aarhus, Denmark; [3]Skane University Hospital, Lund, Sweden; [4]Haukeland University Hospital, Bergen, Norway; [5]Turku University Hospital, Turku, Finland; [6]Rigshospitalet, Copenhagen, Denmark; [7]Oslo University Hospital and KG Jebsen Center for B-cell Malignancies, Oslo, Norway;*

### Background

Treatment failure occurs in one-third of patients with aggressive large B-cell lymphoma (LBCL), and current tools are insufficient for determining remission. Liquid biopsies using plasma circulating tumor DNA (ctDNA) complement diagnostics, predict treatment response and survival, and are expected to transform treatment decision-making.

### Aims

We aimed to assess the added clinical benefit of longitudinal ctDNA monitoring by duplex sequencing in plasma samples from patients with primary high-risk LBCL.

### Methods

We used a targeted duplex sequencing-based ctDNA assay on 447 longitudinal plasma cfDNA samples to analyze measurable residual disease (MRD) from 123 patients with primary LBCL at various time points during treatment and follow-up as part of the Nordic LBC-06 phase II trial (Leppä et al., 2025). The patients were young (aged 18-64 years), had clinically high-risk LBCL, and were treated with dose-intensified immunochemotherapy.  The lowest ctDNA fractions detected were in the parts-per-million (ppm) range, and the median limit of detection was $1.69*10^{-5}$, with 26% of the samples having a limit of detection in the ppm range.

**Copyright Information:** (Online) ISSN: 2572-9241
©2026 The Author(s). HemaSphere is published by John Wiley & Sons Ltd on behalf of European Hematology Association. This is an open access Abstract Book distributed under the Attribution-NonCommercial-NoDerivs (CC BY-NC-ND), which allows third parties to download the articles and share them with others as long as they credit the author and the Abstract Book, but they cannot change the content in any way or use them commercially.
**Abstract Book Citations:** Authors, Title, HemaSphere, 2026;10:(S1):pages. The abstract book DOIs can be found on the "Author Information" page.
**Disclaimer:** Articles published in the journal HemaSphere exclusively reflect the opinions of the authors. The authors are responsible for all content in their abstracts including accuracy of the facts, statements, citing resources, etc.

### Results

During a median follow-up of 60.4 months, 22 patients experienced progression, including 17 relapses and 13 deaths, translating to 82% progression free survival (PFS) and 90% overall survival (OS) at 5 years. Pretreatment ctDNA levels declined in all evaluable patients (Fig 1A), with 67% (75/112) reaching molecular remission after two treatment cycles. $MRD^{CYC2}$ was prognostic (5-year OS, 97% vs 75%, p<0.0001; Fig 1B); however, 68% (25/37) of patients with $MRD^{CYC2+}$ did not relapse. Major molecular response (MMR, defined as ≥2.5 log reduction in ctDNA) was achieved by 85% of patients (94/111). After four treatment cycles, an additional 14 (52%) of the 27 evaluable $MRD^{CYC2+}$ patients became MRD-negative, and, as with $MRD^{CYC2+}$, elevated ctDNA levels among those with $MRD^{CYC4+}$ predicted refractory disease and poor survival (Fig 1C). At the end of therapy (EOT), another seven (54%) of the 13 patients with $MRD^{CYC4+}$ achieved MRD negativity. Assessment of $MRD^{EOT}$ also clarified most false radiological response evaluations. Among all evaluable patients, $MRD^{EOT}$ identified a significant difference in outcomes (5-year OS, 96% vs 30%, p < 0.0001; Fig 1D). During follow-up, all evaluable patients in clinical remission while being $MRD^{EOT+}$ remained MRD+ in subsequent plasma samples, and six (86%) of seven relapsing patients with $MRD^{EOT-}$ became MRD+ before clinical progression was identified. Follow-up provided lead time before progression in 92% (11/12) of patients (median, 6 months).

### Summary/Conclusion

Longitudinal plasma ctDNA detection during treatment and follow-up with duplex sequencing identifies refractory disease, predicts survival, determines therapy responses, and provides lead time before clinical progression. Overall, the results support incorporating molecular remission into LBCL response criteria to complement imaging and provide a dynamic molecular tool to guide clinical decision-making.

**Copyright Information:** (Online) ISSN: 2572-9241
©2026 The Author(s). HemaSphere is published by John Wiley & Sons Ltd on behalf of European Hematology Association. This is an open access Abstract Book distributed under the Attribution-NonCommercial-NoDerivs (CC BY-NC-ND), which allows third parties to download the articles and share them with others as long as they credit the author and the Abstract Book, but they cannot change the content in any way or use them commercially.
**Abstract Book Citations:** Authors, Title, HemaSphere, 2026;10:(S1):pages. The abstract book DOIs can be found on the "Author Information" page.
**Disclaimer:** Articles published in the journal HemaSphere exclusively reflect the opinions of the authors. The authors are responsible for all content in their abstracts including accuracy of the facts, statements, citing resources, etc.

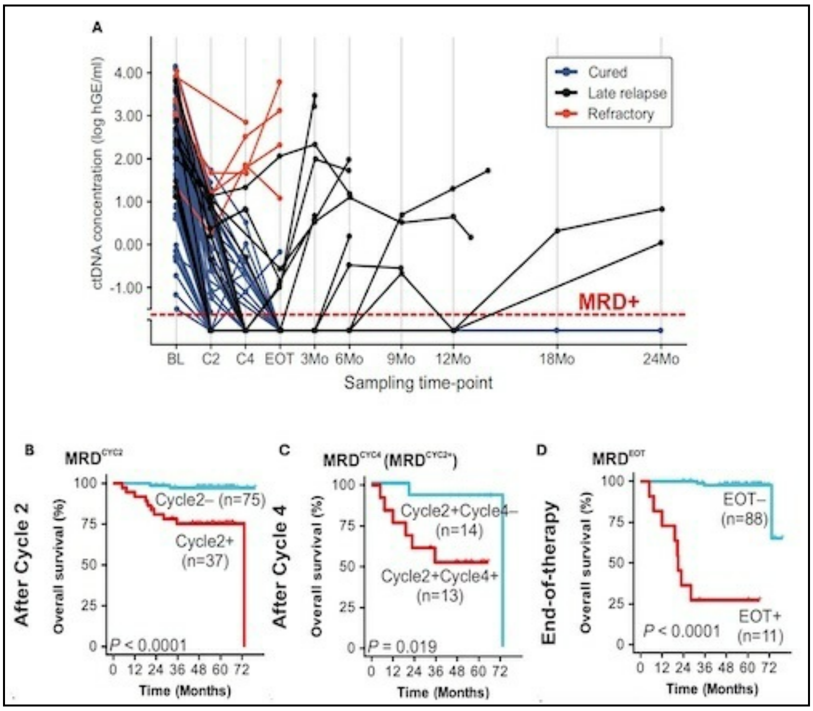

Figure 1. Concentration, clearance, and prognostic significance of circulating tumor DNA (ctDNA) in serial samples collected during immunochemotherapy and follow-up. A. Dendrogram of ctDNA detection and levels per patient during treatment and surveillance. Patients with ctDNA detected are shown; B. Kaplan-Meier (KM) survival estimate for overall survival (OS) according to presence of measurable residual disease (MRD) after two cycles of therapy; C. KM survival estimate for OS among evaluable MRD-CYC2+ patients according to MRD after four cycles (n=27). D. KM survival estimate for OS according to EOT MRD.

**Copyright Information:** (Online) ISSN: 2572-9241
©2026 The Author(s). HemaSphere is published by John Wiley & Sons Ltd on behalf of European Hematology Association. This is an open access Abstract Book distributed under the Attribution-NonCommercial-NoDerivs (CC BY-NC-ND), which allows third parties to download the articles and share them with others as long as they credit the author and the Abstract Book, but they cannot change the content in any way or use them commercially.
**Abstract Book Citations:** Authors, Title, HemaSphere, 2026;10:(S1):pages. The abstract book DOIs can be found on the "Author Information" page.
**Disclaimer:** Articles published in the journal HemaSphere exclusively reflect the opinions of the authors. The authors are responsible for all content in their abstracts including accuracy of the facts, statements, citing resources, etc.

### (S260) INTEGRATED METABOLIC AND EPIGENETIC PROFILING UNCOVERS DISTINCT IMMUNE-ESCAPE PROGRAMS AND TARGETABLE VULNERABILITIES IN POST-TRANSPLANT AML RELAPSE

**Topic:** 22. Stem cell transplantation - Experimental

Annalisa Tameni*[1], Maria Chiara Di Bonito[1, 2], Gabriella Santuccio[1], Francesca Tortorelli[1], Emanuele Rondelli[1], Angela Gentile[1], Vittorio Niccolucci[1, 2], Fatemeh Kordevani[1, 2], Simone Cardaci[3], Beatrice Biferali[4], Raffaella Di Micco[5], Dejan Lazarevic[2], Giovanni Tonon[2, 6], Davide Gabellini[4], David Sumpton[7], Alejandro Huerta Uribe[7], Marco Punta[1, 2], Cristina Toffalori[1], Fabio Ciceri[6, 8], Luca Vago[1, 6, 8]

*[1]Unit of Immunogenetics, Leukemia Genomics and Immunobiology, IRCCS San Raffaele Scientific Institute, Milan, Italy, Milano, Italy; [2]Center for Omics Sciences, IRCCS San Raffaele Scientific Institute, Milan, Italy, Milano, Italy; [3]Cancer metabolism Unit, IRCCS San Raffaele Scientific Institute, Milan, Italy, Milano, Italy; [4]Division of Genetics and Cell Biology, Gene Expression Regulation Unit, San Raffaele Scientific Institute, Milano, Italy; [5]San Raffaele Telethon Institute for Gene Therapy (SR-TIGET), IRCCS San Raffaele Scientific Institute, Milan, Italy, Milano, Italy; [6]Vita-Salute San Raffaele University, Milan, Italy, Milano, Italy; [7]University of Glasgow, Glasgow, United Kingdom, Glasgow, United Kingdom; [8]Hematology and Bone Marrow Transplantation Unit, IRCCS San Raffaele Scientific Institute, Milan, Italy, Milano, Italy;*

### Background

Relapse of acute myeloid leukemia (AML) after allogeneic hematopoietic cell transplantation (allo-HCT) frequently arises from immune-evasion mechanisms that enable leukemic blasts to escape graft-versus-leukemia activity. In particular, we described two mutually-exclusive non-genomic modalities, downregulation of HLA class II molecules and upregulation of inhibitory T cell ligands, that together account for up to 60% of relapses *(*Toffalori et al., *Nat Med*, 2019).

### Aims

To investigate whether these two major immune-escape modalities are driven by distinct epigenetic and metabolic programs, and to uncover potential actionable vulnerabilities.

### Methods

We analyzed paired samples at diagnosis and post-transplant relapse from 16 patients. After AML blasts enrichment, we perform bulk RNA- and ATAC-seq, LC–MS metabolomics of intracellular (endometabolome) and extracellular metabolites (esometabolome). To further define metabolic function, we applied Mitotracker and SCENITH assays (Argüello et al., *Cell Metab* 2020).

**Copyright Information:** (Online) ISSN: 2572-9241
©2026 The Author(s). HemaSphere is published by John Wiley & Sons Ltd on behalf of European Hematology Association. This is an open access Abstract Book distributed under the Attribution-NonCommercial-NoDerivs (CC BY-NC-ND), which allows third parties to download the articles and share them with others as long as they credit the author and the Abstract Book, but they cannot change the content in any way or use them commercially.
**Abstract Book Citations:** Authors, Title, HemaSphere, 2026;10:(S1):pages. The abstract book DOIs can be found on the "Author Information" page.
**Disclaimer:** Articles published in the journal HemaSphere exclusively reflect the opinions of the authors. The authors are responsible for all content in their abstracts including accuracy of the facts, statements, citing resources, etc.

### Results

Gene expression profiling segregated the two relapse phenotypes: downregulation of HLA class II expression (downHLA, n=7) and upregulation of T-cell inhibitory ligands (upInhib, n=9). We found that most metabolic changes were relapse modality-specific, with upInhib cases showing substantial biological alterations in hypoxia (p=0.01), regulation of reactive oxygen/nitrogen species (p=0.03) and aromatic amino acid (AAAs) metabolism (p=0.01). Consistently, ATAC-sequencing analysis revealed increased chromatin accessibility at loci involved in response to oxidative stress (p=0.0015), amino acid (p=0.0014) and organic acid (p=0.0004) metabolism. Coherently, endometabolome analysis showed increased intracellular levels of AAs—including tryptophan (FC=1.48, p=0.03), phenylalanine (FC=1.55, p=0.03), and tyrosine (FC=1.70, p=0.03)—as well as citrulline (FC=2.22, p=0.015) and arginine metabolites (FC=1.44, p=0.046), all of which serve as substrates/intermediates to sustain pro-oxidative metabolism. We also detected significant increase in intracellular 5-oxoproline (FC=1.71, p=0.046), a key intermediate of the γ-glutamyl cycle indicating a shift toward a pro-oxidative state. In addition, SCENITH profiling revealed a trend to increased mitochondrial dependence in upInhib relapses, indicating a preferential engagement of oxidative metabolism, and MitoTracker analysis showed elevated mitochondrial function and intracellular ROS production in 2/4 upInhib patients. Finally, through exometabolome analysis, we detected a significant increase of lactic acid (LA) at relapse for upInhib relapses (p=0.01), while no changes were observed for their downHLA counterpart. Besides its known role as immunosuppressive oncometabolite in the tumor microenvironment (TME), LA has been described to be a direct epigenetic regulator (Zhang et al, *Nature,* 2019). To identify differentially lactylated genes, we performed CUT&Tag profiling of the H4K12la modification in one case of UpInhib relapse, observing enriched lactylation at the promoter regions of PD-L1 and CD86.

### Summary/Conclusion

Taken together, our data delineate a comprehensive landscape of metabolic alterations underlying AML relapse, revealing novel relapse-specific metabolic vulnerabilities that may be therapeutically exploited to design novel complementary strategies for upInhib cases, where direct checkpoint inhibition has largely failed to improve patient outcomes.

**Copyright Information:** (Online) ISSN: 2572-9241
©2026 The Author(s). HemaSphere is published by John Wiley & Sons Ltd on behalf of European Hematology Association. This is an open access Abstract Book distributed under the Attribution-NonCommercial-NoDerivs (CC BY-NC-ND), which allows third parties to download the articles and share them with others as long as they credit the author and the Abstract Book, but they cannot change the content in any way or use them commercially.
**Abstract Book Citations:** Authors, Title, HemaSphere, 2026;10:(S1):pages. The abstract book DOIs can be found on the "Author Information" page.
**Disclaimer:** Articles published in the journal HemaSphere exclusively reflect the opinions of the authors. The authors are responsible for all content in their abstracts including accuracy of the facts, statements, citing resources, etc.

### (S261) INTEGRATIVE MULTIMODAL PROFILING OF THE MICROBIOME-IMMUNE AXIS IN AUTOLOGOUS STEM CELL RECIPIENTS

**Topic:** 22. Stem cell transplantation - Experimental

Sabrina Becker*[1], Julius Enssle[1, 2, 3, 4], Anne Wilke[1, 3, 4], Sylvia Pfundt[1], Nico Zeltner[1], Ana Jankovic[1], Sebastian Wolf[1, 2, 3, 4], Thomas Wichelhaus[1, 5], Tilman Schultze[1, 5], Christina Muhs[6], Islam Alshamleh[6], Juliane Willenborg[1], Henri Mark[1], Volkhardt Kempf[1, 5], Katharina Pütz[1], Björn Häupl[1, 2, 3], Josefine Jakob[1, 2], Maria Vehreschild[1, 7], Florian Büttner[1, 2, 3, 4, 8], Hubert Serve[1, 2, 3, 4], Harald Schwalbe[6], Thomas Oellerich[1, 2, 3, 4], Björn Steffen[1, 3, 4], Sebastian Scheich[1, 2, 3, 4]

*[1]Goethe University Frankfurt, University Hospital, Frankfurt am Main, Germany; [2]Frankfurt Cancer Institute, Goethe University, Frankfurt am Main, Germany; [3]German Cancer Consortium (DKTK), Partner Site Frankfurt/Mainz, Frankfurt am Main, Germany; [4]University Cancer Center (UCT) Frankfurt, Frankfurt am Main, Germany; [5]Institute for Medical Microbiology and Infection Control, University Hospital, Frankfurt, Germany; [6]Institute for Organic Chemistry and Chemical Biology, Center for Biomolecular Magnetic Resonance (BMRZ), Goethe University, Frankfurt am Main, Germany; [7]Goethe University Frankfurt, University Hospital, Department of Internal Medicine II, Infectious Diseases, Frankfurt, Germany; [8]Department of Informatics, Goethe University, Frankfurt, Germany;*

### Background

Autologous stem cell transplantation (ASCT) is an established consolidative therapy for lymphoid malignancies, yet it induces severe immunosuppression and gut microbiome disruption, rendering patients highly susceptible to infections as a major source of clinical complications. While preclinical data suggest bidirectional crosstalk between the gut microbiome and host immunity, human longitudinal multi-omics studies in this context remain scarce.

### Aims

We aimed to characterize the microbiome-immune axis during ASCT using an integrative multi-omics modelling approach.

### Methods

In this prospective, longitudinal study, 253 patients with lymphoid malignancies (Multiple Myeloma, n=141; lymphoma, n=109; other, n=3) undergoing ASCT at University Hospital Frankfurt were enrolled. Paired stool and peripheral blood samples were collected at up to five timepoints from pre-transplant to day 100 post-ASCT. Gut microbiome composition was profiled via 16S-rRNA amplicon sequencing (906 samples), immune reconstitution by flow cytometry (33 cellular subsets, 215 samples), and functional molecular signatures by NMR-based metabolomics and mass spectrometry-based metaproteomics.

**Copyright Information:** (Online) ISSN: 2572-9241
©2026 The Author(s). HemaSphere is published by John Wiley & Sons Ltd on behalf of European Hematology Association. This is an open access Abstract Book distributed under the Attribution-NonCommercial-NoDerivs (CC BY-NC-ND), which allows third parties to download the articles and share them with others as long as they credit the author and the Abstract Book, but they cannot change the content in any way or use them commercially.
**Abstract Book Citations:** Authors, Title, HemaSphere, 2026;10:(S1):pages. The abstract book DOIs can be found on the "Author Information" page.
**Disclaimer:** Articles published in the journal HemaSphere exclusively reflect the opinions of the authors. The authors are responsible for all content in their abstracts including accuracy of the facts, statements, citing resources, etc.

### Results

Microbiome analysis revealed a steep decline in alpha-diversity reaching its nadir between days 10-20 post-ASCT, accompanied by expansion of Enterococcaceae, with partial recovery after engraftment. Immune profiling demonstrated global composition shifts across T and B cell compartments over time. To uncover novel biology in both data-layers, we applied a multi-omics factor analysis framework (MEFISTO) in which the resulting latent embedding of each sample is modelled under the influence of a Gaussian process accounting for the temporal dependency. This recapitulated known biology: dimensionality reduction confirmed sampling timepoint and microbial alpha-diversity gradients, rather than disease entity, as key drivers of variance across both data layers. We assessed each latent factor's (LF) association with patient outcome and identified LF9 significantly associated with inferior overall survival, independently of entity. LF9 was defined by high Enterobacteriaceae as a positive driver and Prevotellaceae as the dominant negative contributor, with high Prevotellaceae or low Enterobacteriaceae abundance individually displaying significant survival benefits. High LF9 scores tracked with reduced frequencies of T helper, activated cytotoxic, and naïve T regulatory cells, and this immune phenotype was driven by the combined LF9 microbiome profile rather than a singular bacterial family. Stool metabolomics corroborated these findings: an Enterococcaceae-dominated dysbiotic sample cluster showed elevated lactate and formate levels, while an LF9-low cluster enriched for Prevotellaceae was characterized by high short chain fatty acid concentrations. Fecal metaproteomics validated the LF9 signature, with differential bacterial protein expression reflecting the underlying Prevotellaceae and Enterobacteriaceae abundance patterns.

### Summary/Conclusion

Multi-modal profiling identified a clinically relevant microbiome-immune signature during ASCT, linking specific microbial signatures to immune reconstitution and patient survival, and providing a foundation for microbiome-informed risk stratification in ASCT recipients.

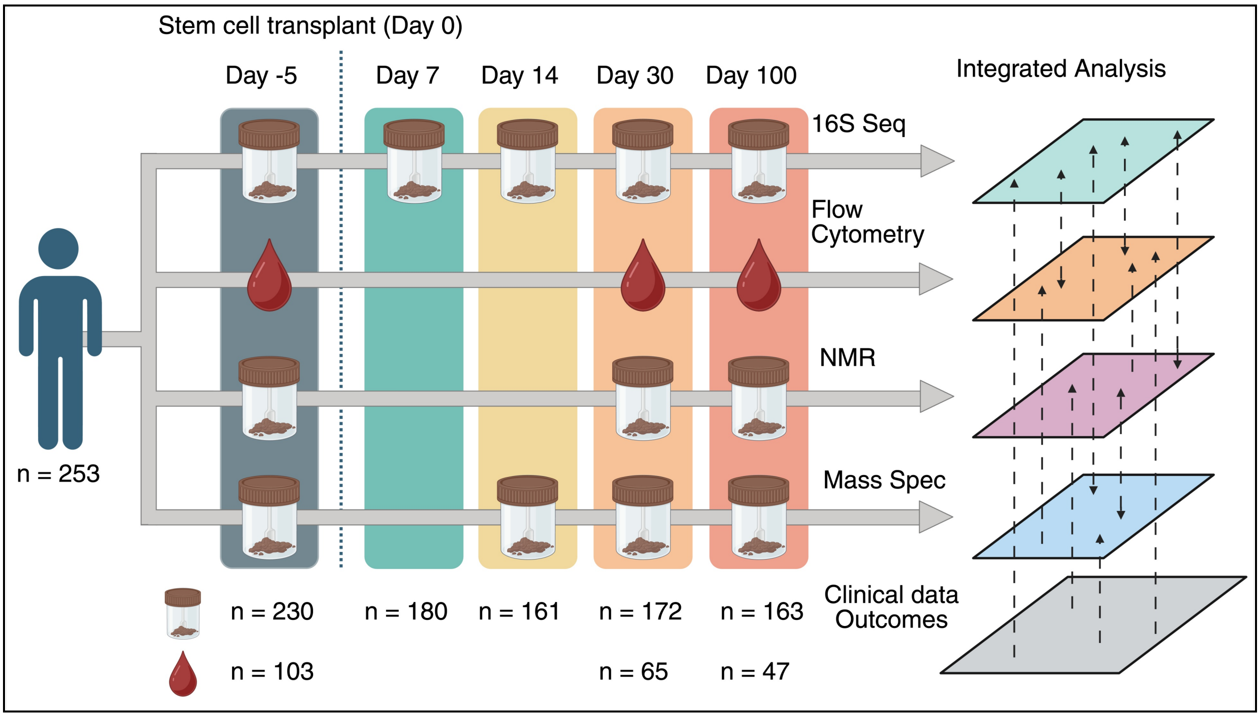

Figure 1: Study Overview. Created with BioRender.com

**Copyright Information:** (Online) ISSN: 2572-9241
©2026 The Author(s). HemaSphere is published by John Wiley & Sons Ltd on behalf of European Hematology Association. This is an open access Abstract Book distributed under the Attribution-NonCommercial-NoDerivs (CC BY-NC-ND), which allows third parties to download the articles and share them with others as long as they credit the author and the Abstract Book, but they cannot change the content in any way or use them commercially.
**Abstract Book Citations:** Authors, Title, HemaSphere, 2026;10:(S1):pages. The abstract book DOIs can be found on the "Author Information" page.
**Disclaimer:** Articles published in the journal HemaSphere exclusively reflect the opinions of the authors. The authors are responsible for all content in their abstracts including accuracy of the facts, statements, citing resources, etc.

### (S262) 7-KETOCHOLESTEROL REPROGRAMS T-CELL LINEAGE METABOLISM TO AMELIORATE STEROID-REFRACTORY ACUTE GVHD

**Topic:** 22. Stem cell transplantation - Experimental

Qi Wang[1, 2], Rui Wang[1], Yuxuan He[1], Yuqing Liu[1], Xi Zhang[1], Qingxiao Song*[1]

[1]*Xinqiao hospital of Army Medical University, Chongqing, China;*[2]*General hospital of Central Theater Command of PLA, Wuhan, China;*

### Background

Steroid-resistant acute graft-versus-host disease (SR-aGVHD) remains a major clinical challenge following allogeneic hematopoietic stem cell transplantation (allo-HSCT), and its underlying mechanisms are poorly understood. Our previous studies suggest dysregulation of T-cell lineage differentiation in the pathogenesis of SR-aGVHD.

### Aims

In this study, we investigate cholesterol metabolic reprogramming that regulate T-cell lineage transdifferenciation and evaluate their potential to reverse steroid resistance in aGVHD.

### Methods

Steroid-sensitive (SS) and steroid-resistant (SR) acute GVHD (aGVHD) models were established. Metabolomic profiling was performed to compare the metabolic landscapes of SS and SR T cells. Histopathological changes in GVHD target organs were assessed by H&E staining. Flow cytometry was used to evaluate T-cell lineage differentiation both in vivo and in vitro. Underlying mechanisms were further investigated using chromatin accessibility assays (ATAC-seq) and CUT&Tag profiling.

### Results

Steroid-sensitive (SS) and steroid-resistant (SR) aGVHD models were successfully established using distinct donor strains. Metabolomic profiling revealed a marked reduction of 7-ketocholesterol (7-KC) in T cells from SR-aGVHD models, whereas donor T cells from SS-aGVHD models exhibited significant enrichment of 7-KC. In vivo, supplementation with 7-KC restored glucocorticoid (GC) sensitivity in SR-aGVHD models, leading to significantly improved survival and ameliorated liver damage. In vitro, combined treatment with 7-KC and dexamethasone (DEX) robustly promoted transdifferentiation of Th17 cells into regulatory T cells (Tregs). Mechanistically, ATAC-seq analysis demonstrated that 7-KC combined with DEX reduced chromatin accessibility at Th17-associated loci (Il17, Il22, and Rorc), while increasing accessibility at the Foxp3 promoter. Consistently, immunofluorescence staining showed that 7-KC enhanced glucocorticoid receptor (GR) nuclear translocation. CUT&Tag profiling further revealed strengthened GR binding at the Foxp3 promoter in the presence of 7-KC.

### Summary/Conclusion

7-KC is a pivotal metabolic regulator of glucocorticoid (GC) sensitivity in aGVHD, with its deficiency on donor T cells potentially contributing to GC resistance. The combination of 7-KC and GC therapy reverses SR-aGVHD by enhancing nuclear GR accumulation and promoting GR binding to the Foxp3 promoter, ultimately facilitating Treg generation. This study provides novel insights into the metabolic and epigenetic mechanisms underlying GC resistance and highlights the potential of 7-KC as a biomarker or therapeutic target for predicting and managing SR-aGVHD.

**Copyright Information:** (Online) ISSN: 2572-9241
©2026 The Author(s). HemaSphere is published by John Wiley & Sons Ltd on behalf of European Hematology Association. This is an open access Abstract Book distributed under the Attribution-NonCommercial-NoDerivs (CC BY-NC-ND), which allows third parties to download the articles and share them with others as long as they credit the author and the Abstract Book, but they cannot change the content in any way or use them commercially.
**Abstract Book Citations:** Authors, Title, HemaSphere, 2026;10:(S1):pages. The abstract book DOIs can be found on the “Author Information” page.
**Disclaimer:** Articles published in the journal HemaSphere exclusively reflect the opinions of the authors. The authors are responsible for all content in their abstracts including accuracy of the facts, statements, citing resources, etc.

### (S263) TRIM5 DRIVES T CELL PATHOGENICITY IN GRAFT-VERSUS-HOST DISEASE THROUGH SLC25A35 UBIQUITINATION

**Topic:** 22. Stem cell transplantation - Experimental

Qiao Cheng*[1], Jun Gao[1], Yafen Han[1], Yang Xu[1], Depei Wu[1]

*[1]the First Affiliated Hospital of Soochow University, Suzhou, China;*

### Background

Dysregulation of the ubiquitin-proteasome system has been implicated in numerous human diseases, and several deubiquitinases have been shown to contribute to the pathogenesis of graft-versus-host disease (GVHD). However, the role of E3 ubiquitin ligases, which directly control substrate ubiquitination, remains largely unexplored in GVHD. Here, we uncover a previously unrecognized role for TRIM5 in T cell–mediated GVHD and suggest that targeting E3 ligase pathways may represent a potential therapeutic strategy for aGVHD.

### Aims

Aim 1: To determine the expression levels of TRIM5 in allogeneic T cells and assess whether TRIM5 expression correlates with the severity of graft-versus-host disease (GVHD) in patients.
Aim 2: To evaluate whether targeting TRIM5 can suppress allogeneic T cell responses and attenuate GVHD.
Aim 3: To elucidate the molecular mechanisms by which TRIM5 regulates T cell activation, differentiation, and effector function in GVHD.

### Methods

**Patients and sample collection.** The study includes one hundred and fifty-four patients who received HSCT in the First Affiliated Hospital of Soochow University from 2018 to 2022. Peripheral blood samples were collected in patients 14 days after HSCT.

**Mice.** C57BL/6 (B6, H2K$^b$)⊠BALB/c (H2K$^d$) mice (aged 6 - 8 weeks) were purchased from SLAC Laboratory Animal Center. TRIM5 knockout (KO) mice were generated by GemPharmatech Co., Ltd. MHC-mismatched (B6 to BALB/c) model was used to establish GVHD.

**Single cell RNA sequencing (scRNA seq).** T cells isolated from wild type (WT) or TRIM5-KO mice along with BM-TCD cells isolated from WT mice were transferred into lethally irradiated BALB/c recipients. On day 10 after BMT, spleen cells were harvested to perform scRNA seq at CapitalBio Corporation Company (Beijing, China).

**Proteomics.** CD4$^+$ T cells isolated from WT or TRIM5-KO mice were stimulated with anti-CD3/CD28 antibodies for 3 days, and then cells were performed by Proteomics at PTMBIO company.

**Silencing of SLC25A35 by CRISPR-CAS9.** T cells isolated from WT B6 mice were stimulated with anti-CD3/CD28 for 24 h, and then replaced with complete RPMI 1640 medium containing IL-2 for 2-3 days. Lonza reagent was used to transfect T cells with RNP containing vector or SLC25A35 KO.

**Copyright Information:** (Online) ISSN: 2572-9241
©2026 The Author(s). HemaSphere is published by John Wiley & Sons Ltd on behalf of European Hematology Association. This is an open access Abstract Book distributed under the Attribution-NonCommercial-NoDerivs (CC BY-NC-ND), which allows third parties to download the articles and share them with others as long as they credit the author and the Abstract Book, but they cannot change the content in any way or use them commercially.
**Abstract Book Citations:** Authors, Title, HemaSphere, 2026;10:(S1):pages. The abstract book DOIs can be found on the “Author Information” page.
**Disclaimer:** Articles published in the journal HemaSphere exclusively reflect the opinions of the authors. The authors are responsible for all content in their abstracts including accuracy of the facts, statements, citing resources, etc.

**Results**

We identified TRIM5, a member of the tripartite motif (TRIM) family, as being markedly upregulated in allogeneic $CD4^+$ T cells and significantly associated with GVHD incidence and overall survival in patients after HSCT. Genetic ablation of TRIM5 in donor $CD4^+$ T cells attenuates GVHD severity by suppressing Th1 and Th17 differentiation. Furthermore, TRIM5 deficiency impairs mitochondrial oxidative phosphorylation in activated $CD4^+$ T cells, accompanied by loss of mitochondrial membrane potential and reduced mitochondrial superoxide production. Mechanically, TRIM5 interacts with and ubiquitinates SLC25A35, promoting its proteasomal degradation in an E3 ligase-dependent manner. Silencing SLC25A35 enhances allogeneic responses of donor $CD4^+$ T cells in GVHD mice. Notably, SLC25A35 silencing fails to restore cytokine production in TRIM5-deficient donor $CD4^+$ T cells, indicating that SLC25A35 is a critical downstream effector of TRIM5.

**Summary/Conclusion**

TRIM5 functions as an E3 ubiquitin ligase for SLC25A35, promoting its ubiquitination, enhancing oxidative phosphorylation in $CD4^+$ T cells, and thereby exacerbating allogeneic T cell responses and GVHD.

**Copyright Information:** (Online) ISSN: 2572-9241
©2026 The Author(s). HemaSphere is published by John Wiley & Sons Ltd on behalf of European Hematology Association. This is an open access Abstract Book distributed under the Attribution-NonCommercial-NoDerivs (CC BY-NC-ND), which allows third parties to download the articles and share them with others as long as they credit the author and the Abstract Book, but they cannot change the content in any way or use them commercially.
**Abstract Book Citations:** Authors, Title, HemaSphere, 2026;10:(S1):pages. The abstract book DOIs can be found on the "Author Information" page.
**Disclaimer:** Articles published in the journal HemaSphere exclusively reflect the opinions of the authors. The authors are responsible for all content in their abstracts including accuracy of the facts, statements, citing resources, etc.

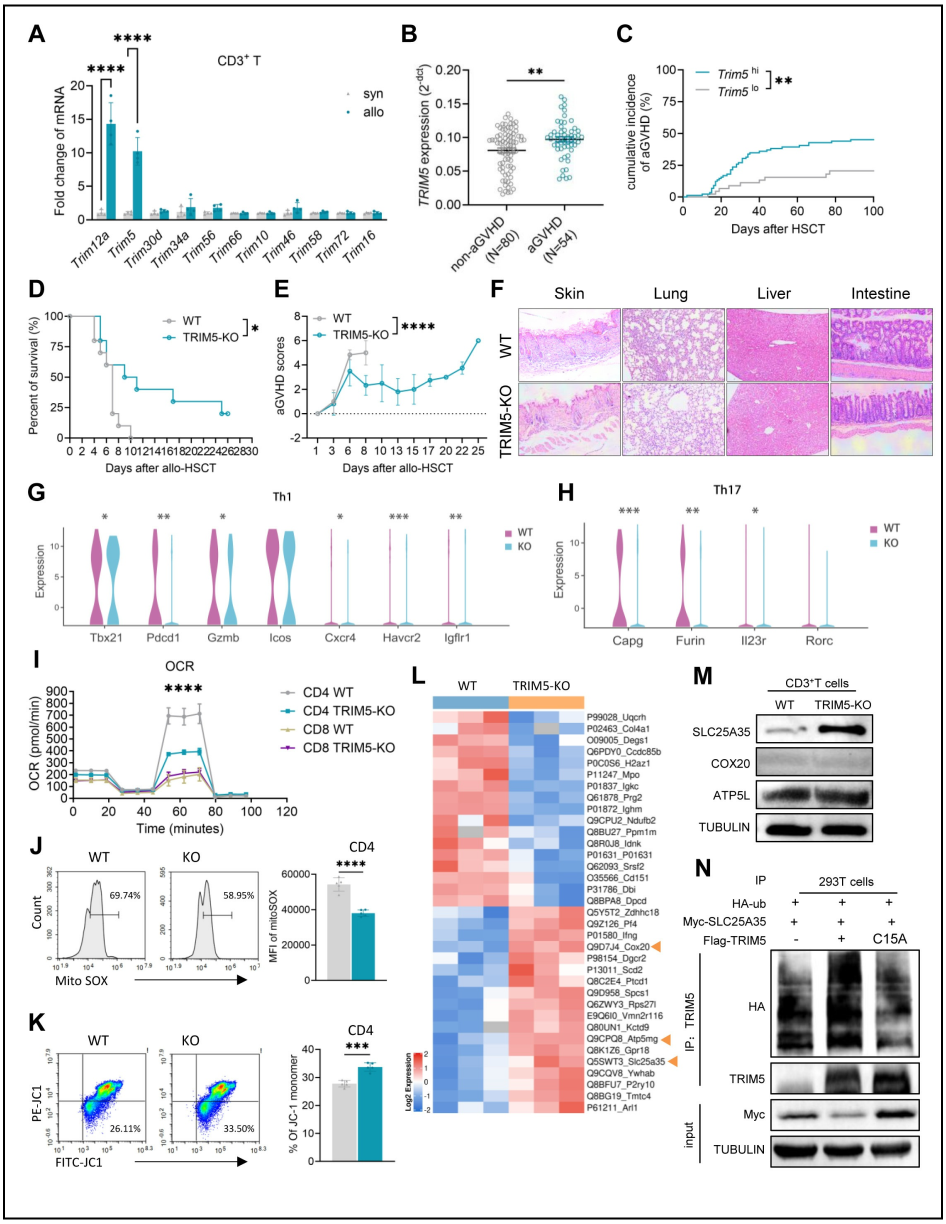

**Copyright Information:** (Online) ISSN: 2572-9241
©2026 The Author(s). HemaSphere is published by John Wiley & Sons Ltd on behalf of European Hematology Association. This is an open access Abstract Book distributed under the Attribution-NonCommercial-NoDerivs (CC BY-NC-ND), which allows third parties to download the articles and share them with others as long as they credit the author and the Abstract Book, but they cannot change the content in any way or use them commercially.
**Abstract Book Citations:** Authors, Title, HemaSphere, 2026;10:(S1):pages. The abstract book DOIs can be found on the "Author Information" page.
**Disclaimer:** Articles published in the journal HemaSphere exclusively reflect the opinions of the authors. The authors are responsible for all content in their abstracts including accuracy of the facts, statements, citing resources, etc.

### (S265) DEVELOPMENT AND VALIDATION OF THE FAC-HCT SCALE (FRAILTY, AGE AND COMORBIDITY) TO PREDICT OVERALL SURVIVAL AFTER ALLOGENEIC HEMATOPOIETIC CELL TRANSPLANTATION

**Topic:** 23. Stem cell transplantation - Clinical

Mónica Baile-González[1], Tommy Alfaro-Moya[2], Mikhail Vlasov[3], Victor Navarro[3], Ivan Pasic[2], Marina Acera-Gómez[1], Maria Laura Fox[4], María del Mar Pérez-Artigas[4], Ana Santamaria[5], María del Carmen Quintela-González[5], Andrés Sanchez Salinas[6], Joaquina Salmerón-Camacho[6], Verónica Illana-Álvaro[7], Zahra Abdallahi-Lefdil[7], Javier Cornago Navascués[8], Laura Pardo Gambarte[8], Sara Fernandez-Luis[9], Leddy Patricia Vega[9], Sara Villar-Fernandez[10], Patricia Beorlegui-Murillo[10], Albert Esquirol Sanfeliu[11], Isabel Izquierdo-García[12], Alberto Mussetti[13], María Esperanza Lavilla Rubira[14], Javier López[15], Pascual Balsalobre[16], Leyre Bento[17], Auro Viswabandya[2], Fotios Michelis[2], Jonas Mattsson[2], Shabbir Alibhai[2], Montserrat Rovira Tarrats[18], Dennis Kim[2], Anna Sureda[19], Rajat Kumar[2], Maria Queralt Salas*[18]

*[1]Complejo Asistencial Universitario de Salamanca / IBSAL, Hematology, Salamanca, Spain;[2]Princess Margaret Cancer Center, Toronto, Canada; [3]Vall d'Hebron Instituto de Oncología (VHIO), Barcelona, Spain;[4]Hospital Universitari Vall d'Hebron, Barcelona, Spain; [5]Hospital Álvaro Cunqueiro, Vigo, Spain;[6]H.C.U. Virgen de la Arrixaca. IMIB Pascual Parrilla, Murcia, Spain; [7]Hospital Universitario de la Princesa, Madrid, Spain;[8]Hospital Universitario Fundación Jiménez Díaz, Madrid, Spain;[9]Hospital Universitario Marqués de Valdecilla - IDIVAL, Santander, Spain; [10]Clinica Universidad de Navarra, Pamplona, Spain; [11]Hospital de la Santa Creu i Sant Pau, Barcelona, Spain; [12]Hospital Universitario Miguel Servet, Zaragoza, Spain; [13]Institut Català d'Oncologia - Hospital Duran i Reynals, L'Hospitalet del LLobregat,, Barcelona, Spain; [14]Hospital Universitario Lucus Augusti, Lugo, Spain; [15]Hospital Universitario de Alicante, Alicante, Spain;[16]Grupo Español de Trasplante de Progenitores Hematopoyéticos y Terapia Celular, Madrid, Spain; [17]Hospital Universitario Son Spases, Palma de Mallorca, Spain; [18]Hospital Clinic de Barcelona, Barcelona, Spain; [19]Institut Català d'Oncologia - Hospital Duran i Reynals, L'Hospitalet del LLobregat, Barcelona, Spain;*

### Background

Allogeneic hematopoietic cell transplantation (allo-HCT) is a curative therapy for high-risk hematologic disorders but carries substantial morbidity and mortality. As allo-HCT evolves, candidates become older and heterogeneous, emphasizing the need for tools to predict overall survival (OS). Traditional pre-transplant assessments do not fully capture biological vulnerability, whereas frailty—a multidimensional measure of physiologic reserve—is being recognized as a key component of patient´s evaluation.

### Aims

The Spanish Group for HCT (GETH-TC) and the HCT Program at Princess Margaret Cancer Centre (PMCC) initiated a collaborative project to develop the FAC-HCT scale, a composite predictive model integrating age, comorbidity, and frailty for OS prediction.

### Methods

This study included retrospective data from 1,075 adults who underwent allo-HCT at PMCC (2018–2024, n=734) and 15 GETH-TC centers (2022–2024, n=341). All patients participated in a prior study evaluating frailty using the HCT Frailty Scale (HCT-FS). The primary endpoint was OS. Age was recorded in years, comorbidity burden using the HCT-CI, and frailty using the HCT-FS. The HCT-FS comprises eight variables generating a frailty index (0–10.5), classifying patients as fit, prefrail, or frail. The impact of each variable on OS was evaluated using LASSO penalized regression to determine the appropriate inclusion format (continuous vs. categorical). Clinically meaningful cut-offs were identified using the maximally selected log-rank test. The points assigned to each variable , were obtained with the formula *[(HRx-1/(HRFrailty-1)]*

**Copyright Information:** (Online) ISSN: 2572-9241
©2026 The Author(s). HemaSphere is published by John Wiley & Sons Ltd on behalf of European Hematology Association. This is an open access Abstract Book distributed under the Attribution-NonCommercial-NoDerivs (CC BY-NC-ND), which allows third parties to download the articles and share them with others as long as they credit the author and the Abstract Book, but they cannot change the content in any way or use them commercially.
**Abstract Book Citations:** Authors, Title, HemaSphere, 2026;10:(S1):pages. The abstract book DOIs can be found on the "Author Information" page.
**Disclaimer:** Articles published in the journal HemaSphere exclusively reflect the opinions of the authors. The authors are responsible for all content in their abstracts including accuracy of the facts, statements, citing resources, etc.

### Results

The cohort was divided into an experimental cohort (EC; PMCC) and a validation cohort (VC; GETH-TC), with different baseline characteristics (**Table 1**). The scale was designed using data from the EC. Age and HCT-CI were included as categorical variables, while frailty was included as continuous variable using the frailty index. The FAC-HCT scoring system assigned was: <40 years (0 points), 40–65 (+3), >65 (+5); HCT-CI <3 (0), ≥3 (+2); plus the frailty index, and then, patients were classified as low (0–5), intermediate (5.5–10), or high risk (>10) using those specified cut-offs. (**Figure 1**).
In the EC, patients were stratified into low- (44,5%), intermediate- (44,5%), and high-risk (10,5%) groups, with 2-year OS rates of 78.8%, 63.5%, and 44.4%, respectively (LRT P=3e-09), demonstrating appropriate discrimination. Complementary in the VC, patients were similarly classified (39,5%, 51,5% and 9% for each risk group), with 2-year OS of 75.7%, 61.2%, and 36.5% for low-, intermediate-, and high-risk groups (LRT P=0.002), confirming reproducibility and external validity **(Figure 1).**

| | **PMMC n=734/%** | **GETH-TC n=341/%** | **p-value** |
|---|---|---|---|
| **Median age, years** | 59 | 55 | 0.01 |
| < 40 | 145/20 | 68/20 | |
| 40-65 | 397/54 | 217/63,5 | |
| >65 | 192/26 | 56/16,5 | 0.02 |
| **Male** | 409/56 | 226/66.5 | 0.002 |
| **Dx** | | | |
| AML | 370/50.5 | 128/37.5 | |
| MDS | 134/18,5 | 71/21 | |
| ALL | 68/9 | 45/13 | |
| NPM | 66/9 | 25/7,5 | 0.009 |
| LPD | 40/5,5 | 45/13 | |
| Other | 56/7,5 | 27/8 | |
| **HCT-CI** | | | |
| ≥3 | 252/34.5 | 102/30 | 0.47 |
| **HCT-FS** | | | |
| Fit | 267/36.5 | 91/26,5 | |
| Prefrail | 373/50.5 | 206/60.5 | 0.02 |
| Frail | 94/13 | 44/13 | |
| **Transplant** | | | |
| RIC | 494/67.5 | 179/52.5 | 0.001 |
| PTCY | 626/85 | 194/57 | 0.001 |
| MSD | 160/22 | 109/32 | |
| MUD | 383/52 | 110/32 | 0.19 |
| 9/10 MMUD | 75/10 | 37/11 | |
| Haplo | 116/16 | 85/25 | |

### Summary/Conclusion

FAC-HCT stratified allo-HCT patients into three independent risk groups with progressively worse OS. Its applicability was consistent across two cohorts with different baseline characteristics, supporting its robustness and potential generalizability.

**Copyright Information:** (Online) ISSN: 2572-9241
©2026 The Author(s). HemaSphere is published by John Wiley & Sons Ltd on behalf of European Hematology Association. This is an open access Abstract Book distributed under the Attribution-NonCommercial-NoDerivs (CC BY-NC-ND), which allows third parties to download the articles and share them with others as long as they credit the author and the Abstract Book, but they cannot change the content in any way or use them commercially.
**Abstract Book Citations:** Authors, Title, HemaSphere, 2026;10:(S1):pages. The abstract book DOIs can be found on the "Author Information" page.
**Disclaimer:** Articles published in the journal HemaSphere exclusively reflect the opinions of the authors. The authors are responsible for all content in their abstracts including accuracy of the facts, statements, citing resources, etc.

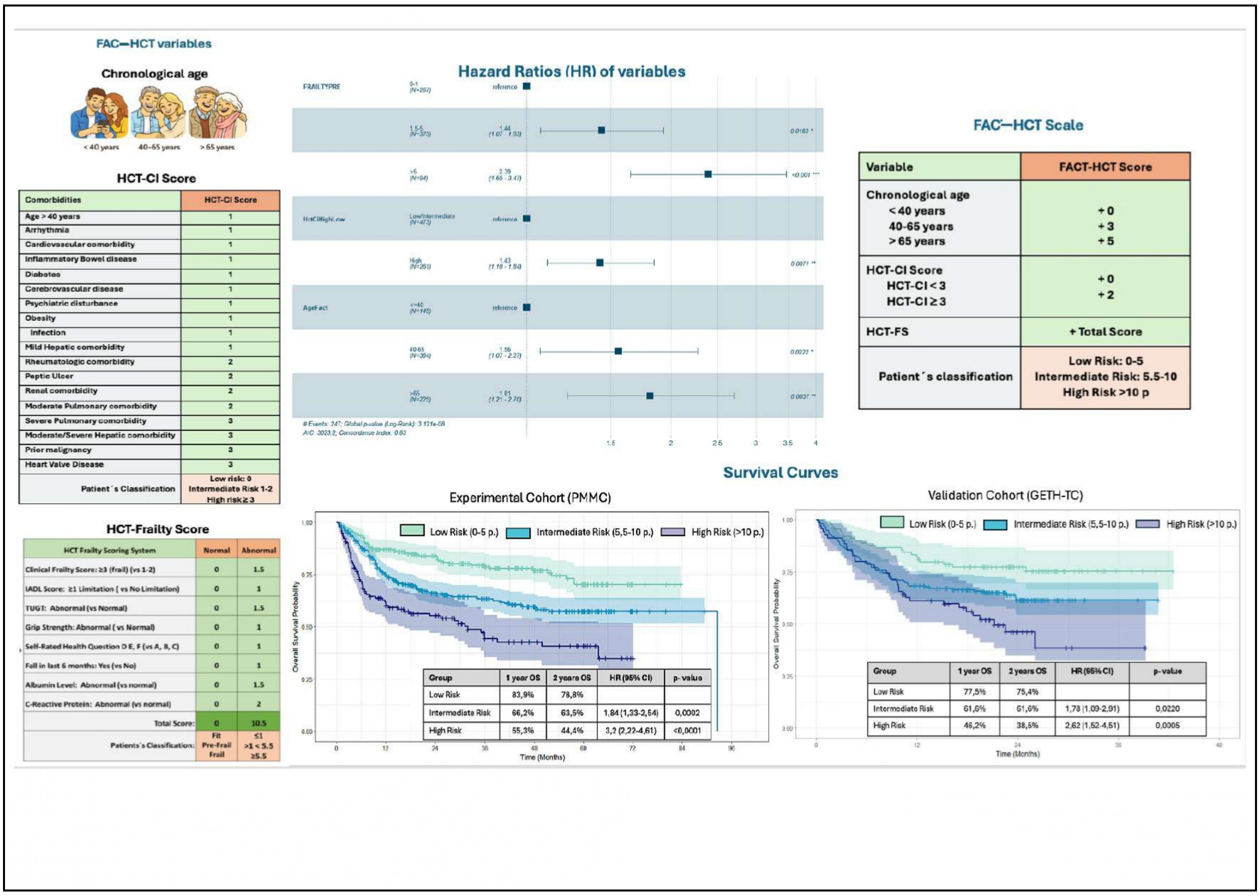

**Copyright Information:** (Online) ISSN: 2572-9241
©2026 The Author(s). HemaSphere is published by John Wiley & Sons Ltd on behalf of European Hematology Association. This is an open access Abstract Book distributed under the Attribution-NonCommercial-NoDerivs (CC BY-NC-ND), which allows third parties to download the articles and share them with others as long as they credit the author and the Abstract Book, but they cannot change the content in any way or use them commercially.
**Abstract Book Citations:** Authors, Title, HemaSphere, 2026;10:(S1):pages. The abstract book DOIs can be found on the “Author Information” page.
**Disclaimer:** Articles published in the journal HemaSphere exclusively reflect the opinions of the authors. The authors are responsible for all content in their abstracts including accuracy of the facts, statements, citing resources, etc.

### (S266) ABC: AN ENTIRELY CALCINEURIN AND M-TOR INHIBITOR-FREE SHORT DURATION GVHD PREVENTION REGIMEN

**Topic:** 23. Stem cell transplantation - Clinical

Stephanie Boisclair[1, 2], Jaime Andres Londono[1, 2], Kelli Cole[2], Omar Marzouk[2], Angie Fleury[2], Farzaneh Mashayekhi[2], Mariam Daghestani[2], Saud Khalaf[2], Ruthee Lu Bayer[1, 2], Douglas Gladstone[1, 2], Maher Abdul-Hay[3], A Samer Al-Homsi*[1, 2]

*[1]Donald and Barbara Zucker School of Medicine at Hofstra University, Hempstead, United States of America;[2]Northwell Cancer Institute, New Hyde Park, United States of America; [3]NYU Langone Health and Grossman School of Medicine, New York, United States of America;*

### Background

An ideal graft-versus-host disease (GvHD) prevention regimen following allogeneic hematopoietic stem cell transplantation (AHSCT) should be effective, non-toxic and cost effective. Shortened regimens permit earlier introduction of maintenance strategies. Building on our prior work combining post-transplant cyclophosphamide (PTC) with bortezomib (Bor), we hypothesized that adding abatacept (Aba) would provide enhanced GvHD prevention regimen meeting these prerequisites.

### Aims

To report the results of the ongoing ABC phase I–II clinical trial (NCT06681922).

### Methods

Patients (pts) undergoing peripheral blood AHSCT for hematological malignancy were enrolled into 2 cohorts: matched (related or unrelated) or mismatched (7 out of 8 HLA loci) donor cohorts. Conditioning was either, fludarabine (Flu) and busulfan (Bu) (2 days) or Flu, Bu (3 days) and thiotepa. Unrelated donor recipients received r-ATG, 5 mg/Kg total dose, split over days -4 to -2. GvHD prophylaxis included PTC (50 mg/Kg; days +3 and +4), Bor (1.3 mg/m²; days 0 and +3) and Aba 10mg/Kg. During phase I, Aba was escalated in a 3+3 design: 1 dose on day +5, 2 doses on days +5 & +14 and 3 doses on days +5, +14 & +28. No dose limiting toxicity occurred and the 3-dose regimen was adopted. Following the first 25 pts, the protocol was amended, decreasing PTC to 37.5 mg/Kg/dose. Survival was estimated by Kaplan–Meier analysis. Estimated cumulative incidence (ECI) was calculated using Gray’s competing risk model.

| | | PTC 50mg/Kg | PTC 37.5mg/Kg |
|---|---|---|---|
| Age (yr) | Median (range) | 59 (24-68) | 60 (27-76) |
| Sex | Male | 13 | 23 |
| Disease | AML | 16 | 17 |
| | MDS | 5 | 5 |
| | | | |

**Copyright Information:** (Online) ISSN: 2572-9241
©2026 The Author(s). HemaSphere is published by John Wiley & Sons Ltd on behalf of European Hematology Association. This is an open access Abstract Book distributed under the Attribution-NonCommercial-NoDerivs (CC BY-NC-ND), which allows third parties to download the articles and share them with others as long as they credit the author and the Abstract Book, but they cannot change the content in any way or use them commercially.
**Abstract Book Citations:** Authors, Title, HemaSphere, 2026;10:(S1):pages. The abstract book DOIs can be found on the “Author Information” page.
**Disclaimer:** Articles published in the journal HemaSphere exclusively reflect the opinions of the authors. The authors are responsible for all content in their abstracts including accuracy of the facts, statements, citing resources, etc.

| | | | |
|---|---|---|---|
| | NHL | 4 | 4 |
| | Others | 0 | 9 |
| Disease Risk Index | Low | 0 | 2 |
| | Intermediate | 16 | 19 |
| | High or Very High | 9 | 14 |
| Disease Status | CR1 | 18 | 22 |
| | CR2+ | 3 | 5 |
| | Active Disease | 4 | 8 |
| Donor | MRD | 9 | 1 |
| | MUD | 13 | 19 |
| | MMUD (7 out of 8) | 3 | 15 |
| Conditioning regimen | r-ATG Flu Bu TT | 5 | 14 |
| | r-ATG Flu Bu | 11 | 20 |
| | Flu Bu TT | 6 | 1 |
| | Flu Bu | 3 | 0 |

**Copyright Information:** (Online) ISSN: 2572-9241
©2026 The Author(s). HemaSphere is published by John Wiley & Sons Ltd on behalf of European Hematology Association. This is an open access Abstract Book distributed under the Attribution-NonCommercial-NoDerivs (CC BY-NC-ND), which allows third parties to download the articles and share them with others as long as they credit the author and the Abstract Book, but they cannot change the content in any way or use them commercially.
**Abstract Book Citations:** Authors, Title, HemaSphere, 2026;10:(S1):pages. The abstract book DOIs can be found on the "Author Information" page.
**Disclaimer:** Articles published in the journal HemaSphere exclusively reflect the opinions of the authors. The authors are responsible for all content in their abstracts including accuracy of the facts, statements, citing resources, etc.

### Results

Enrollment has been completed for the matched cohort (42 pts). 18 out of 32 planned pts have been enrolled in the mismatched donor cohort (total 60 pts). Median follow-up was 16.2 months. Pt characteristics are summarized in the table. Neutrophil and platelet engraftment occurred earlier in reduced dose PTC group (15 vs 17 days, p=0.003 and 22 vs 25.5 days, p = 0.04, respectively), with 1 pt in each group not achieving platelet engraftment. No primary or secondary graft failure was observed. ECI of grade II-IV and grade III-IV acute GvHD was 11.8% and 0% in reduced PTC group versus 4% and 0% (p = 0.29) in full-dose PTC group. Moderate to severe chronic GvHD occurred in 9.5% (2.3 -22.8) and 12% (2.9 – 28.1) of reduced- and full-dose groups, respectively (p=0.88). 1-year ECI of relapse and treatment-related mortality was 18.3% (9.2 – 29.7) and 3.5% (0.64– 10.8%), respectively, with comparable outcomes between PTC dose groups. 1-year estimated overall survival, progression-free survival and GvHD- and relapse-free survival are shown in the figure, with no significant difference between PTC dose groups. Rates of CMV, EBV and adenovirus reactivation requiring treatment were 31.6%, 16.6% and 3.3%, respectively. $CD3^+$, $CD4^+$, $CD8^+$, $CD19^+$ or $CD16^+CD56^+$ cell recovery at day +30, 100 and 180 were comparable between the 2 PTC groups.

### Summary/Conclusion

ABC is a novel entirely calcineurin and m-TOR inhibitor-free GvHD prevention regimen that is completed in 4 weeks. The regimen is effective and well tolerated. Reducing the dose PTC in ABC accelerates engraftment and maintains low rates of acute and chronic GvHD without apparent impact on immune reconstitution.

**Figure 1.** Survival Outcomes

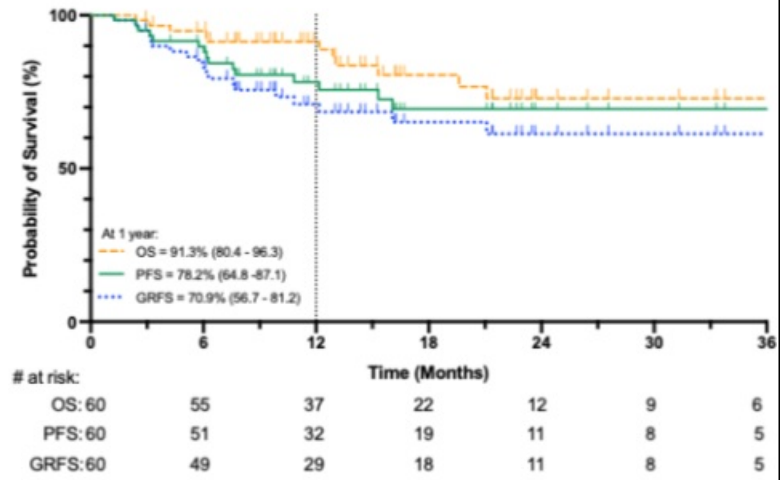

**Copyright Information:** (Online) ISSN: 2572-9241
©2026 The Author(s). HemaSphere is published by John Wiley & Sons Ltd on behalf of European Hematology Association. This is an open access Abstract Book distributed under the Attribution-NonCommercial-NoDerivs (CC BY-NC-ND), which allows third parties to download the articles and share them with others as long as they credit the author and the Abstract Book, but they cannot change the content in any way or use them commercially.
**Abstract Book Citations:** Authors, Title, HemaSphere, 2026;10:(S1):pages. The abstract book DOIs can be found on the "Author Information" page.
**Disclaimer:** Articles published in the journal HemaSphere exclusively reflect the opinions of the authors. The authors are responsible for all content in their abstracts including accuracy of the facts, statements, citing resources, etc.

### (S267) FBEM (FLUDARABINE, BUSULFAN, ETOPOSIDE, AND MELPHALAN) CONDITIONING REGIMEN FOR PATIENTS WITH LYMPHOID MALIGNANCIES UNDERGOING ALLO-HSCT: A PROSPECTIVE PHASE II STUDY

**Topic:** 23. Stem cell transplantation - Clinical

Mengmeng Pan*[1], Chuanhe Jiang[1], Jieling Jiang[1], Shi-wei Jin[1], Zengkai Pan[1], Ling Wang[1], Jin Wang[1], Xiaoxia Hu[1], Jian-qing Mi[1], Jiong Hu[1], Weiping Zhang[1]

[1]*Shanghai Institute of Hematology, State Key Laboratory of Medical Genomics, National Research Center for Translational Medicine at Shanghai, Ruijin Hospital, Shanghai jiao Tong University School of Medicine, Shanghai, China;*

### Background

Intensified FBEM (Flu-Bu-Et-Mel) conditioning offers potent anti-tumor activity for lymphoid malignancies but is limited by severe mucositis and infections. While post-transplant cyclophosphamide (PTCY) is standard for GVHD prophylaxis, the efficacy of combining it with post-engraftment basiliximab or ATG in mitigating regimen-related toxicities remains to be optimized.

### Aims

To evaluate the safety and efficacy of the intensified FBEM regimen in lymphoid malignancies and to identify the optimal PTCy-based GVHD prophylaxis strategy (incorporating post-engraftment basiliximab or ATG) for reducing regimen-related toxicities and relapse.

### Methods

In this single-center, prospective phase II study (NCT04897139), 203 patients with lymphoid malignancies undergoing allo-HSCT (Nov 2020–Nov 2025) were enrolled. including Ph+ ALL (n=99, 48.8%), Ph- ALL (n=76, 37.4%), and T-ALL/LBL (n=22, 10.8%). Notably, 70% of patients received haplo-identical transplants. FBEM conditioning consisted of fludarabine (150 mg/m²), busulfan (9.6 mg/kg), melphalan (100 mg/m²), and etoposide (primarily 400–600 mg/m²; range 200–800 mg/m²). GVHD prophylaxis regimens included: ATG alone (n=25); ATG+PTCy (n=19); PTCy+post-engraftment basiliximab (n=99); and PTCy+post-engraftment ATG (n=59). Survival outcomes (OS, LFS, and GRFS) were estimated using the Kaplan-Meier method. Cumulative incidence rates of relapse (CIR), NRM, and GVHD were calculated accounting for competing risks. Multivariate analyses for toxicities and survival predictors were performed using logistic regression and Cox proportional hazards models, respectively.

### Results

With a median follow-up of 25.1 months (range, 0.2–63.4), the estimated 2-year OS and LFS were 91.0% and 83.2%, respectively. The 1-year NRM was remarkably low at 2.6%, and the 2-year CIR was 13.5% (95% CI: 8.3%–18.8%). The median time to neutrophil and platelet engraftment was 14 and 13 days, respectively. The incidence of grade 3–4 aGVHD was 5.5%, while moderate-to-severe cGVHD was 17.0%. Notably, intensified etoposide ( 600 mg/m$^2$, n=76) did not increase regimen-related toxicities.
With a median follow-up of 25.1 months, the estimated 2-year OS and LFS were 91.0% and 83.2%, respectively. The 1-year NRM was 2.6%, and 2-year CIR was 13.5%. Median neutrophil and platelet engraftment occurred at 14 and 13 days. Grade 3–4 aGVHD was 5.5% and moderate-to-severe cGVHD was 17.0%. Intensified etoposide (600 mg/m², n=76) did not increase toxicities. Multivariate analysis identified the prophylaxis regimen as the sole independent predictor for severe complications. Compared to ATG monotherapy, PTCy + post-engraftment basiliximab significantly reduced the risk of severe oral mucositis (OR=0.117; P=0.01) and severe infection (OR=0.046; P<0.001). PTCy + post-engraftment ATG also reduced infection risk (OR=0.164; P=0.002). Notably, PTCy combined with post-engraftment basiliximab or ATG significantly lowered EBV reactivation within 180 days post-transplant (P<0.05). Survival and cumulative incidence (considering competing risks) were analyzed.

**Copyright Information:** (Online) ISSN: 2572-9241
©2026 The Author(s). HemaSphere is published by John Wiley & Sons Ltd on behalf of European Hematology Association. This is an open access Abstract Book distributed under the Attribution-NonCommercial-NoDerivs (CC BY-NC-ND), which allows third parties to download the articles and share them with others as long as they credit the author and the Abstract Book, but they cannot change the content in any way or use them commercially.
**Abstract Book Citations:** Authors, Title, HemaSphere, 2026;10:(S1):pages. The abstract book DOIs can be found on the “Author Information” page.
**Disclaimer:** Articles published in the journal HemaSphere exclusively reflect the opinions of the authors. The authors are responsible for all content in their abstracts including accuracy of the facts, statements, citing resources, etc.

### Summary/Conclusion

FBEM conditioning is highly effective for lymphoid malignancies, achieving 91.0% OS with minimal NRM, even in 70% haplo-identical transplants. Integrating PTCy with post-engraftment basiliximab or ATG remarkably improves safety by mitigating severe oral mucositis and infections. Pre-transplant MRD remains the strongest predictor of survival.

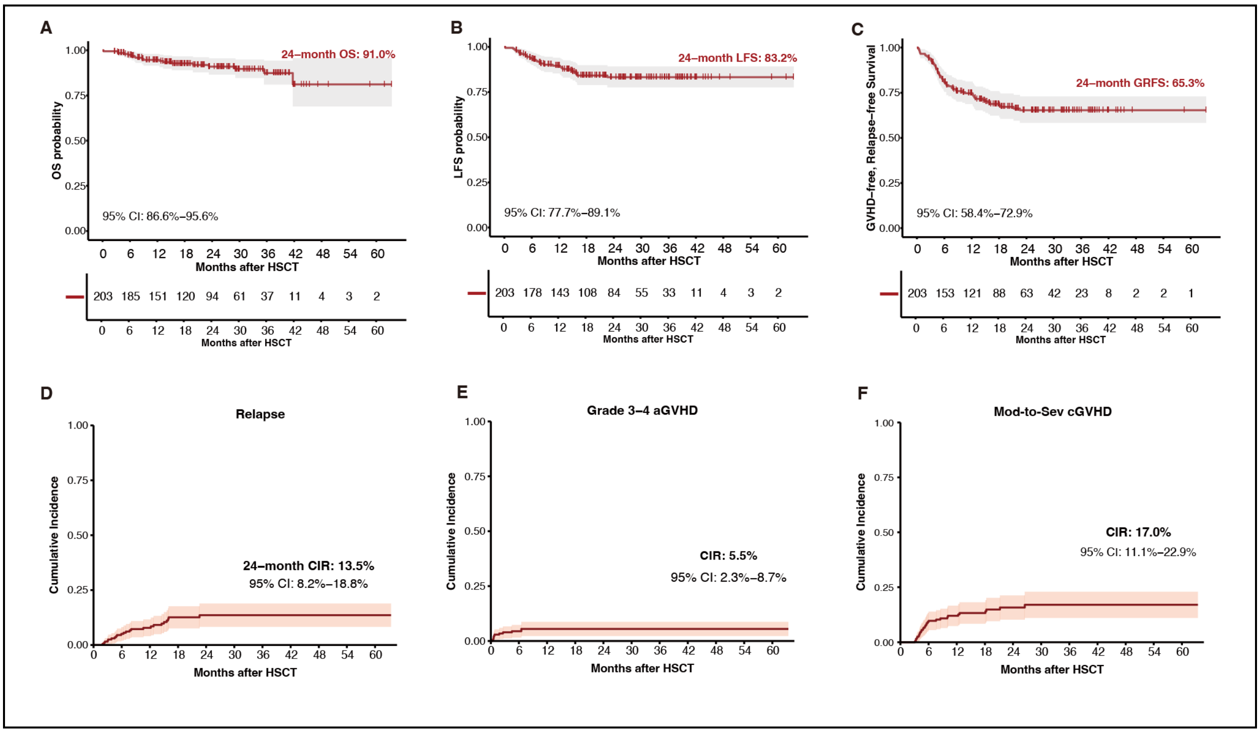

**Copyright Information:** (Online) ISSN: 2572-9241
©2026 The Author(s). HemaSphere is published by John Wiley & Sons Ltd on behalf of European Hematology Association. This is an open access Abstract Book distributed under the Attribution-NonCommercial-NoDerivs (CC BY-NC-ND), which allows third parties to download the articles and share them with others as long as they credit the author and the Abstract Book, but they cannot change the content in any way or use them commercially.
**Abstract Book Citations:** Authors, Title, HemaSphere, 2026;10:(S1):pages. The abstract book DOIs can be found on the "Author Information" page.
**Disclaimer:** Articles published in the journal HemaSphere exclusively reflect the opinions of the authors. The authors are responsible for all content in their abstracts including accuracy of the facts, statements, citing resources, etc.

### (S268) POST-TRANSPLANT MOLECULAR SURVEILLANCE REVEALS DIVERGENT CLINICAL TRAJECTORIES OF DONOR-DERIVED CLONAL HEMATOPOIESIS

**Topic:** 23. Stem cell transplantation - Clinical

Todd Knepper*[1], Hammad Tashkandi[1], Mohammad Hussaini[1], Asmita Mishra[1], Aaron Bossler[1], Rory M. Shallis[1], Onyee Chan[1], David Sallman[1], Jeffrey Lancet[1], Rami S. Komrokji[1], Eric Padron[1], Zhuoer Xie[1]

*[1]H. Lee Moffitt Cancer Center & Research Institute, Tampa, FL, United States of America;*

### Background

Allogeneic stem cell transplantation (allo-HCT) is a potentially curative therapy for myeloid neoplasms, but donor-derived clonal hematopoiesis (ddCH) has emerged as a significant post-transplant phenomenon. Recent evidence suggests that donor *DNMT3A*-CH may augment graft-versus-leukemia activity through altered inflammatory cytokine signaling, whereas rare clones harboring *TP53* or splicing factors mutations carry an elevated risk of progression to donor cell leukemia. The clinical significance of detecting these mutations in recipients during routine molecular surveillance remains a critical area of investigation.

### Aims

To identify allo-HCT recipients with suspected ddCH and evaluate associations between donor-derived mutations, relapse, survival, and the development of donor-derived myeloid neoplasms (ddMN).

### Methods

We reviewed 822 patients with myeloid malignancies who underwent allo-HCT at a single center with next-generation sequencing (NGS, 98-gene panel) reported between 2023 and 2026. NGS was performed on bone marrow samples at standard intervals (day 30, 90, 180, 365, and annually). Suspected ddCH was defined as the emergence of new somatic mutations post-HCT in the setting of ~100% donor CD33 chimerism without morphologic evidence of myeloid neoplasm by AML flow minimal residual disease panel or pre-transplant recipient mutations. In cases where a post-transplant myeloid neoplasm emerged from these new donor-derived mutations rather than the original recipient clone, ddMN was suspected. To ensure clinical rigor, each case was reviewed and discussed at a multidisciplinary precision medicine tumor board involving hematologists, molecular pathologists, and precision medicine faculty.

**Copyright Information:** (Online) ISSN: 2572-9241
©2026 The Author(s). HemaSphere is published by John Wiley & Sons Ltd on behalf of European Hematology Association. This is an open access Abstract Book distributed under the Attribution-NonCommercial-NoDerivs (CC BY-NC-ND), which allows third parties to download the articles and share them with others as long as they credit the author and the Abstract Book, but they cannot change the content in any way or use them commercially.
**Abstract Book Citations:** Authors, Title, HemaSphere, 2026;10:(S1):pages. The abstract book DOIs can be found on the “Author Information” page.
**Disclaimer:** Articles published in the journal HemaSphere exclusively reflect the opinions of the authors. The authors are responsible for all content in their abstracts including accuracy of the facts, statements, citing resources, etc.

### Results

Suspected ddCH was identified in 30 patients (3.6%) and was first detected at a median of 10.7 (range 1-64) months post-HCT. ddCH was enriched in matched related donor (MRD) transplants (16%) compared with matched unrelated (1.4%) or haploidentical (2.5%) grafts (p < 0.001). Donor age differed between MRD (n=15), median age 60 years and non-MRD donors (MUD + haploidentical; n=10), median age = 40 years (p<0.001).
The most frequently observed mutations were *DNMT3A* (63%), *ASXL1* (13%), and *TP53* (10%), with 67% of patients having a single mutated gene. The median VAF of the largest clone at first detection was 5.3% (IQR 3.5% - 15.6%).
With a median follow-up of 3.0 years, 23 patients (77%) were alive and 3 (10%) had died. Four (13%) were censored due to stale follow-up (>8 months). Two-year overall survival was 93%.
Among those with *DNMT3A*-mutated ddCH, 65% (13/19) remained relapse-free, compared with 27% (3/11) of those with suspected ddCH without *DNMT3A* (relative risk 2.51, p=0.057).
Four (13%) patients developed a suspected ddMN at a median time of 13 months post HCT. The median interval from ddCH detection to ddMN was 7.5 months. Mutations in *TP53* or splicing-factor genes (*SF3B1* or *SRSF2*) were present in 100% (n=4) ddMN cases vs. 7.7% of those without ddMN (p < 0.05). Only one ddMN carried *DNMT3A* (co-occurring with *SF3B1* and *ASXL1*).

### Summary/Conclusion

Suspected ddCH was seen in a small subset (3.6%) of allo-HCT recipients and was enriched in MRD grafts.*DNMT3A*-mutated ddCH was associated with higher relapse-free rates, whereas ddMN arose exclusively in cases with *TP53* or splicing-factor mutations. These findings support a distinction between donor-derived *DNMT3A*-mutated CH and high-risk ddMN-associated clones and highlight the importance of integrated molecular surveillance after allo-HCT.

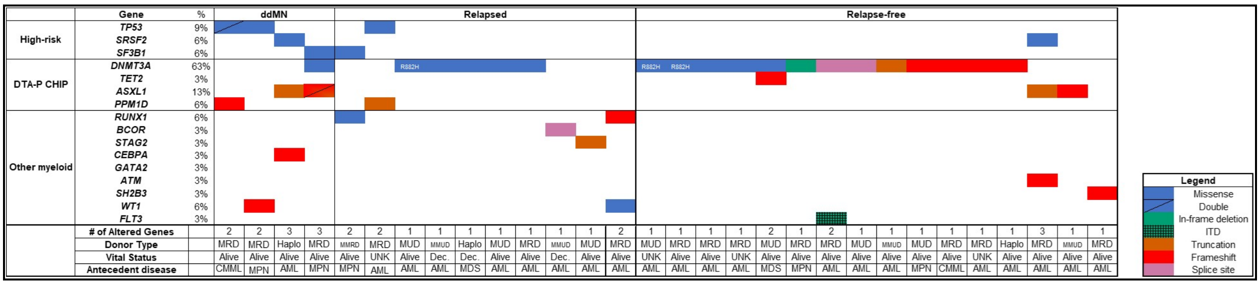

Oncoprint of suspected donor-derived mutations with annotated mutation type, donor type, number of altered genes, vital status, and antecedent disease, grouped by ddMN, relapse, and relapse-free outcomes.

**Copyright Information:** (Online) ISSN: 2572-9241
©2026 The Author(s). HemaSphere is published by John Wiley & Sons Ltd on behalf of European Hematology Association. This is an open access Abstract Book distributed under the Attribution-NonCommercial-NoDerivs (CC BY-NC-ND), which allows third parties to download the articles and share them with others as long as they credit the author and the Abstract Book, but they cannot change the content in any way or use them commercially.
**Abstract Book Citations:** Authors, Title, HemaSphere, 2026;10:(S1):pages. The abstract book DOIs can be found on the "Author Information" page.
**Disclaimer:** Articles published in the journal HemaSphere exclusively reflect the opinions of the authors. The authors are responsible for all content in their abstracts including accuracy of the facts, statements, citing resources, etc.

### (S269) DONOR AGE DOES NOT AFFECT OUTCOMES OF HAPLOIDENTICAL TRANSPLANTATION FOR HEMATOLOGIC MALIGNANCIES: A STUDY BASED ON THE CHINESE BONE MARROW TRANSPLANTATION REGISTRY GROUP (CBMTRG)

**Topic:** 23. Stem cell transplantation - Clinical

Jia Liu[1], Haixia Fu[2], Li Xuan[3], Yao Sun[4], Jia Chen[5], Xiaoli Liang[1], Jing Liu[2], Congcong Wang[3], Shengye Lu[2], Lanping Xu[2], Xiaoqi Wang[1], Ruihao Huang[1], Xuechun Wang*[1], Depei Wu[5], Dai-Hong Liu[4], Qifa Liu[3], Xiao-Jun Huang[2], Xi Zhang[1]

*[1]Medical Center of Hematology, Xinqiao Hospital of Army Medical University,, Chongqing, China;[2]ChinaPeking University People's Hospital, Peking University Institute of Hematology, Beijing, China; [3]Department of Hematology,Nanfang Hospital, Southern Medical University, Guangzhou, China; [4]Chinese PLA General Hospital, Department of Hematology in the Fifth Medical Center of PLA General Hospital, Beijing, China; [5]Department of Hematology, the First Affiliated Hospital of Soochow University, Suzhou, China;*

### Background

Allogeneic hematopoietic stem cell transplantation (allo-HSCT) is a crucial treatment for high-risk hematologic malignancies. Haploidentical HSCT (haplo-HSCT) has become the predominant modality in China and is increasingly used worldwide as an alternative donor source. The impact of donor age on outcomes remains controversial and may vary by transplantation type and disease population. However, large-scale real-world evidence specifically in the setting of haplo-HSCT for hematologic malignancies is still lacking.

### Aims

To evaluate the impact of donor age on outcomes in patients with hematologic malignancies undergoing haplo-HSCT and to inform donor selection strategies.

### Methods

This retrospective study enrolled patients with hematologic malignancies who underwent haplo-HSCT at five centers between 2018 and 2023, with data from the Chinese Bone Marrow Transplantation Registry Group (CBMTRG). Inclusion criteria required donor age ≤35 years or ≥50 years. Propensity score matching (1:1) balanced ten key covariates. Outcomes included overall survival (OS), disease-free survival (DFS), non-relapse mortality (NRM), cumulative incidence of relapse (CIR), and acute/chronic graft-versus-host disease (aGVHD/cGVHD). Multivariable Cox regression and subgroup analyses by recipient age were performed.

### Results

Of 979 eligible patients, 260 were included in each group after matching. No significant differences were observed between young (<30 years) and older (>50 years) donor groups in OS (P=0.617), DFS (P=0.824), NRM (P=0.96), CIR (P=0.970), grade II-IV aGVHD (P=0.190), or cGVHD (P=0.530)(Figure 1). Multivariable analysis confirmed donor age was not an independent prognostic factor for OS (HR=1.07, P=0.748) or CIR (HR=0.99, P=0.960). Recipient age, donor-recipient sex combination, HCT-CI score, and pre-transplantation disease status significantly influenced outcomes. Subgroup analyses revealed that donor age did not significantly affect the aforementioned transplantation outcomes in either younger or older recipient subgroups (P > 0.05).

### Summary/Conclusion

Donor age has no significant impact on transplantation outcomes in patients with malignant hematologic diseases undergoing haplo-HSCT, whereas recipient-related factors such as age and disease status are more critical.

**Copyright Information:** (Online) ISSN: 2572-9241
©2026 The Author(s). HemaSphere is published by John Wiley & Sons Ltd on behalf of European Hematology Association. This is an open access Abstract Book distributed under the Attribution-NonCommercial-NoDerivs (CC BY-NC-ND), which allows third parties to download the articles and share them with others as long as they credit the author and the Abstract Book, but they cannot change the content in any way or use them commercially.
**Abstract Book Citations:** Authors, Title, HemaSphere, 2026;10:(S1):pages. The abstract book DOIs can be found on the "Author Information" page.
**Disclaimer:** Articles published in the journal HemaSphere exclusively reflect the opinions of the authors. The authors are responsible for all content in their abstracts including accuracy of the facts, statements, citing resources, etc.

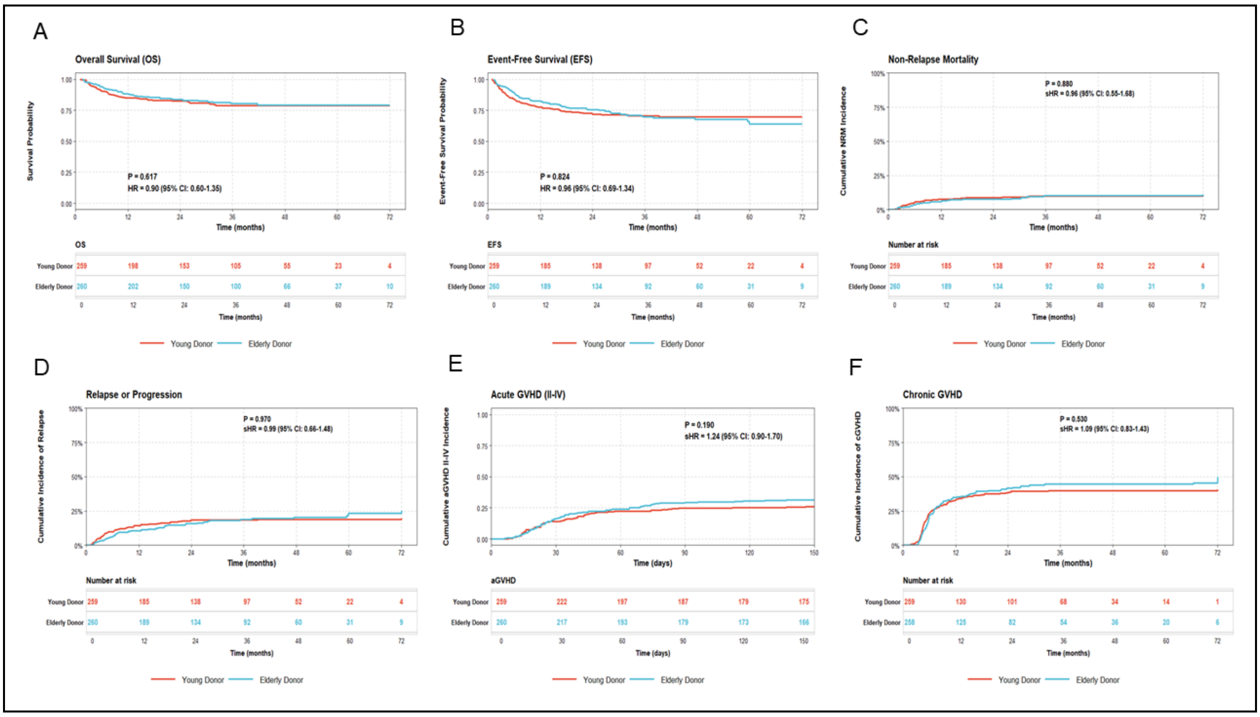

Figure 1. Comparison of transplantation outcomes between young donors (≤35 years) and older donors (≥50 years).(A) Overall survival (OS, P=0.617). (B) Event-free survival (EFS, P=0.824). (C) Non-relapse mortality (NRM, P=0.96). (D) Cumulative incidence of relapse (CIR, P=0.970). (E) Grade II–IV acute graft-versus-host disease (aGVHD, P=0.190). (F) Chronic graft-versus-host disease (cGVHD, P=0.530).

**Copyright Information:** (Online) ISSN: 2572-9241
©2026 The Author(s). HemaSphere is published by John Wiley & Sons Ltd on behalf of European Hematology Association. This is an open access Abstract Book distributed under the Attribution-NonCommercial-NoDerivs (CC BY-NC-ND), which allows third parties to download the articles and share them with others as long as they credit the author and the Abstract Book, but they cannot change the content in any way or use them commercially.
**Abstract Book Citations:** Authors, Title, HemaSphere, 2026;10:(S1):pages. The abstract book DOIs can be found on the "Author Information" page.
**Disclaimer:** Articles published in the journal HemaSphere exclusively reflect the opinions of the authors. The authors are responsible for all content in their abstracts including accuracy of the facts, statements, citing resources, etc.

### (S270) POST-TRANSPLANT DYNAMICS OF DTA MUTATIONS AND THEIR ASSOCIATION WITH MOLECULAR RELAPSE AFTER ALLOGENEIC HEMATOPOIETIC CELL TRANSPLANTATION

**Topic:** 23. Stem cell transplantation - Clinical

Bargavi Rasiah[1], Sarusan Kathirchelvan[1], Joerg Halter[1], Michael Bader[1], Jakob Passweg[1], Darius Juskevicius[1], Corinne Widmer*[1]

*[1]University Hospital Basel and University of Basel, Division of Hematology, Basel, Switzerland;*

### Background

Mutations in DNMT3A, TET2, and ASXL1 (DTA) are characteristic of clonal hematopoiesis of indeterminate potential and frequently detected in hematologic malignancies. In acute myeloid leukemia (AML), DTA mutations are generally considered unsuitable markers for measurable residual disease (MRD) outside the transplant setting. Their biological behavior and clinical relevance after allogeneic hematopoietic cell transplantation (allo HCT) remain still insufficiently defined.

### Aims

The aim was to investigate the prevalence, kinetics, and potential prognostic impact of DTA mutations after allo HCT and to analyze their relationship with non-DTA molecular MRD status, variant allele frequency (VAF), and overall survival (OS).

### Methods

We retrospectively analyzed 743 adult patients undergoing allo HCT between 2018–2024 at a single transplantation center with available next-generation sequencing (NGS) data before and after transplantation. DTA positive mutation profiles were identified in 232 patients (31%). Molecular MRD was defined by detection of ≥1 non-DTA mutation. Patients were categorized according to their post-transplant DTA and MRD status. Longitudinal VAF dynamics, timing of mutation reappearance, donor chimerism, and OS were assessed.

### Results

Post-transplant DTA positivity (DTA+ post) was observed in 116 of the 232 DTA patients (50%). DTA mutations reappeared early, 57% within the first month, 79% by three months, and 95% within one year. DNMT3A and TET2 showed comparable recurrence kinetics, whereas ASXL1 re-emerged more gradually. MRD positivity after allo HCT was significantly more frequent in the DTA+ post group. In most cases (74%), DTA re-detection occurred simultaneously with non-DTA MRD positivity. In approximately 20%, DTA mutations preceded MRD detection (Figure 1).
Isolated DTA positivity post-transplant (7%) was usually transient, characterized by very low VAF and frequently associated with incomplete donor chimerism, resolving without relapse in most cases. Persistent isolated DTA positivity without relapse was rare (<1%) and occurred in cases with microchimerism. Donor-derived DTA mutations were identified in 25 patients (3.4% of the total cohort). Isolated donor-derived clones, confirmed by full donor chimerism, showed stable or slowly increasing VAF over years within the observation period.
Detection of DTA mutations after allo HCT was associated with significantly inferior OS in AML patients. However, when stratified by combined DTA/MRD categories, isolated DTA positivity did not confer worse survival. Moreover, DTA status alone did not add prognostic impact beyond established disease-specific risk factors.

**Copyright Information:** (Online) ISSN: 2572-9241
©2026 The Author(s). HemaSphere is published by John Wiley & Sons Ltd on behalf of European Hematology Association. This is an open access Abstract Book distributed under the Attribution-NonCommercial-NoDerivs (CC BY-NC-ND), which allows third parties to download the articles and share them with others as long as they credit the author and the Abstract Book, but they cannot change the content in any way or use them commercially.
**Abstract Book Citations:** Authors, Title, HemaSphere, 2026;10:(S1):pages. The abstract book DOIs can be found on the “Author Information” page.
**Disclaimer:** Articles published in the journal HemaSphere exclusively reflect the opinions of the authors. The authors are responsible for all content in their abstracts including accuracy of the facts, statements, citing resources, etc.

**Summary/Conclusion**

DTA mutations frequently reappear early after allo-HCT and, in the majority of cases, parallel molecular relapse. While isolated post-transplant DTA positivity is often transient and clinically limited, re-detection in association with MRD identifies patients with inferior survival. These findings support a context-dependent interpretation of DTA mutations and their integration into post-transplant MRD surveillance rather than their exclusion from relapse monitoring.

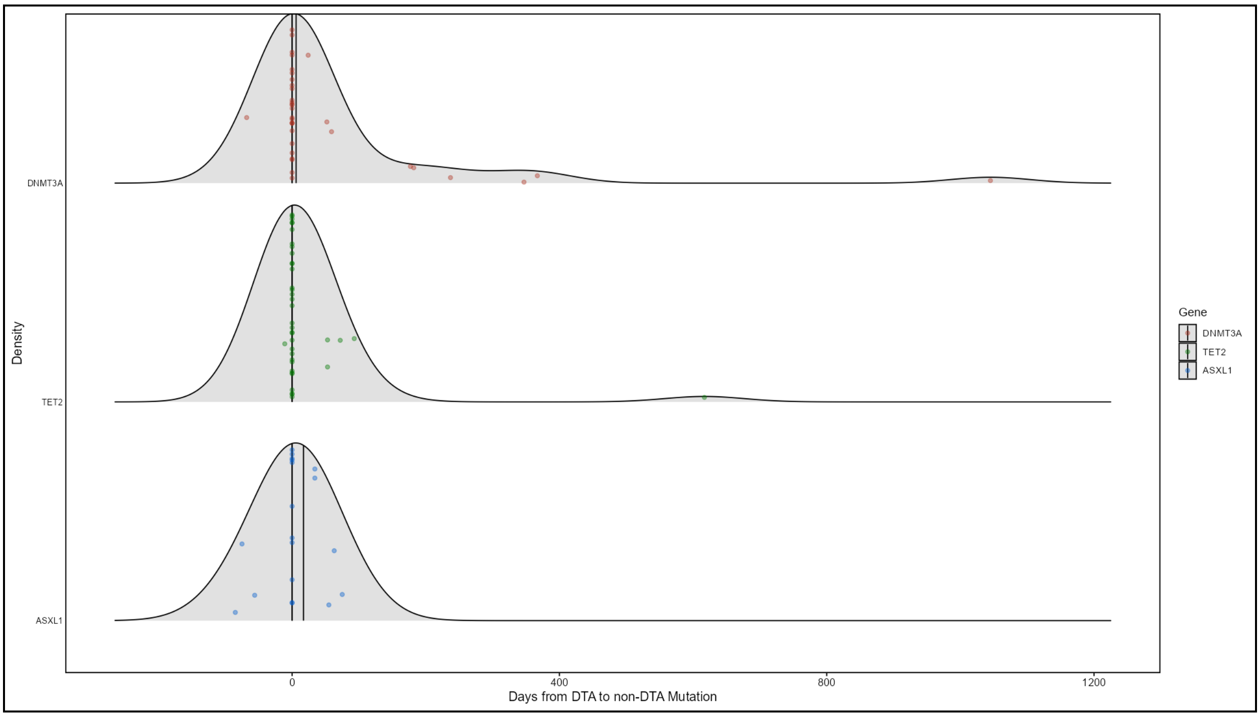

Figure 1: Days between detection of DTA mutations and reemergence of other molecular MRD markers after allo HCT. Density plots show the time interval (days) between DTA detection (DNMT3A, TET2, ASXL1) and non-DTA MRD positivity. Day 0 indicates simultaneous detection; positive values indicate DTA preceding MRD.

**Copyright Information:** (Online) ISSN: 2572-9241
©2026 The Author(s). HemaSphere is published by John Wiley & Sons Ltd on behalf of European Hematology Association. This is an open access Abstract Book distributed under the Attribution-NonCommercial-NoDerivs (CC BY-NC-ND), which allows third parties to download the articles and share them with others as long as they credit the author and the Abstract Book, but they cannot change the content in any way or use them commercially.
**Abstract Book Citations:** Authors, Title, HemaSphere, 2026;10:(S1):pages. The abstract book DOIs can be found on the “Author Information” page.
**Disclaimer:** Articles published in the journal HemaSphere exclusively reflect the opinions of the authors. The authors are responsible for all content in their abstracts including accuracy of the facts, statements, citing resources, etc.

### (S271) REFINING PROGNOSTIC STRATIFICATION FOR SAML POST-ALLO-HSCT: VALIDATION OF ELN 2022 AND PREDOMINANT ROLE OF MDS-RELATED CYTOGENETICS.

**Topic:** 23. Stem cell transplantation - Clinical

Nan Yang*[1], Shan Jiang[1], Erlie Jiang[1], Aiming Pang[1]

*[1]State Key Laboratory of Experimental Hematology, National Clinical Research Center for Blood Diseases, Haihe Laboratory of Cell Ecosystem, Institute of Hematology & Blood Diseases Hospital, Chinese Academy of Medical Sciences & Peking Union Medical College, Tianjin Institutes of Health Science, Tianjin, China, Tianjin, China;*

### Background

Secondary AML (sAML), arising from prior hematologic diseases or therapy, has poorer outcomes than de novo AML. While allo-HSCT is the primary cure, post-transplant relapse remains the leading cause of failure. The prognostic utility of ELN 2022 in this setting requires optimization, specifically regarding the relative significance of MDS-related gene mutations (MR-GM) versus cytogenetics (MR-CG).

### Aims

To characterize the comprehensive clinical features and molecular/cytogenetic landscape of sAML patients; 2) To evaluate the prognostic utility of **the** standard ELN 2022 risk stratification; 3) To assess **the refined ELN 2022 framework for allo-HSCT**, which incorporates an "Adverse-Plus" group (defined by *TP53* mutation, complex karyotype, *MECOM* rearrangement, and 17p-) to identify highest-risk candidates; 4) To elucidate whether MR-CG or MR-GM serves as the predominant driver of post-transplant outcomes under MAC intensity.

### Methods

We retrospectively analyzed 126 adult patients with sAML who underwent first allo-HSCT following MAC (97.6% busulfan-based) between 2017 and 2023. ELN 2022 was evaluated alongside a refined system that segregated an **"Adverse-Plus"** category based on high-risk genetic features. Survival endpoints, including overall survival (OS), leukemia-free survival (LFS), and MRD-free survival (MFS), were estimated using Kaplan-Meier methods. Cumulative incidence of relapse (CIR) and non-relapse mortality (NRM) were assessed using Fine-Gray competitive risk models. Multivariate Cox regression analysis was performed to identify independent predictors of outcome.

**Copyright Information:** (Online) ISSN: 2572-9241
©2026 The Author(s). HemaSphere is published by John Wiley & Sons Ltd on behalf of European Hematology Association. This is an open access Abstract Book distributed under the Attribution-NonCommercial-NoDerivs (CC BY-NC-ND), which allows third parties to download the articles and share them with others as long as they credit the author and the Abstract Book, but they cannot change the content in any way or use them commercially.
**Abstract Book Citations:** Authors, Title, HemaSphere, 2026;10:(S1):pages. The abstract book DOIs can be found on the "Author Information" page.
**Disclaimer:** Articles published in the journal HemaSphere exclusively reflect the opinions of the authors. The authors are responsible for all content in their abstracts including accuracy of the facts, statements, citing resources, etc.

**Results**

1. Genetics: Frequent mutations included U2AF1 (21%), RUNX1 (21%), and ASXL1 (19%). High VAF in BCOR/STAG2 (0.40–0.45) suggested early clones, while ZRSR2 (0.20) represented subclonal evolution, reflecting significant clonal complexity.
2. ELN 2022 vs. Allo-HCT refined ELN 2022: ELN 2022 significantly distinguished OS and LFS between favorable and adverse groups (P<0.05) but showed limited efficacy in predicting relapse (Global P=0.156). The refined system successfully identified the Adverse-Plus group with significantly inferior 3-year OS (43.0%), LFS (32.7%), and MFS (30.2%) compared to other risk categories (P<0.05). Multivariate analysis confirmed Adverse-Plus as a robust independent risk factor for OS (HR 3.15, P=0.033), LFS (HR 3.65, P=0.008), and CIR (HR 5.26, P=0.036), using the Intermediate group as the reference.
3. MR-CG Dominance: A key finding was the superior prognostic weight of MR-CG over MR-GM. MR-CG+ patients (e.g., complex karyotype) had markedly worse OS (P=0.011) and MFS (P<0.001). Conversely, MR-GM alone (without MR-CG) showed no significant survival difference compared to MR-GM-negative patients, identifying MR-CG as the primary driver of failure despite intensive MAC.
4. Clinical Factors: Age >50, non-remission at transplant, and pre-transplant MRD+ (HR 6.31, P=0.020) also independently predicted failure.

**Summary/Conclusion**

The allo-HSCT-refined ELN 2022 system, incorporating the Adverse-Plus subgroup, provides superior prognostic discrimination for sAML patients following MAC allo-HSCT. Our study underscores that MDS-related cytogenetics (MR-CG) is the predominant driver of post-transplant survival and relapse, carrying significantly more prognostic weight than molecular mutations alone. These findings advocate for a cytogenetics-centered approach to risk assessment and tailored post-transplant maintenance strategies.

**Copyright Information:** (Online) ISSN: 2572-9241
©2026 The Author(s). HemaSphere is published by John Wiley & Sons Ltd on behalf of European Hematology Association. This is an open access Abstract Book distributed under the Attribution-NonCommercial-NoDerivs (CC BY-NC-ND), which allows third parties to download the articles and share them with others as long as they credit the author and the Abstract Book, but they cannot change the content in any way or use them commercially.
**Abstract Book Citations:** Authors, Title, HemaSphere, 2026;10:(S1):pages. The abstract book DOIs can be found on the “Author Information” page.
**Disclaimer:** Articles published in the journal HemaSphere exclusively reflect the opinions of the authors. The authors are responsible for all content in their abstracts including accuracy of the facts, statements, citing resources, etc.

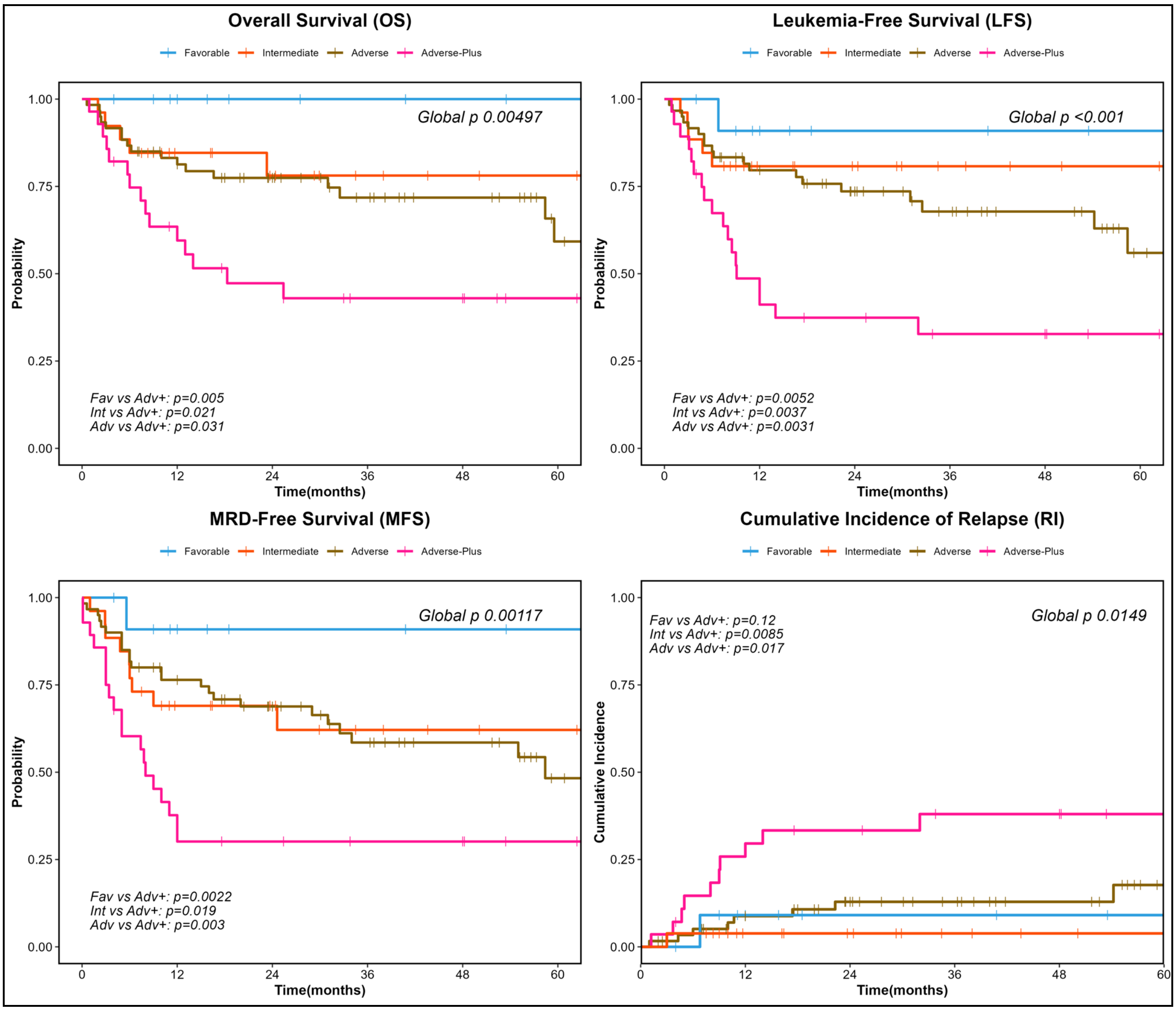

**Copyright Information:** (Online) ISSN: 2572-9241
©2026 The Author(s). HemaSphere is published by John Wiley & Sons Ltd on behalf of European Hematology Association. This is an open access Abstract Book distributed under the Attribution-NonCommercial-NoDerivs (CC BY-NC-ND), which allows third parties to download the articles and share them with others as long as they credit the author and the Abstract Book, but they cannot change the content in any way or use them commercially.
**Abstract Book Citations:** Authors, Title, HemaSphere, 2026;10:(S1):pages. The abstract book DOIs can be found on the "Author Information" page.
**Disclaimer:** Articles published in the journal HemaSphere exclusively reflect the opinions of the authors. The authors are responsible for all content in their abstracts including accuracy of the facts, statements, citing resources, etc.

### (S272) LACK OF PROGNOSTIC IMPACT OF FLT3-ITD ON ALLOGENEIC TRANSPLANTATION OUTCOME IN AML WITH INV (16) (P13.1; Q22.1) OR T (16;16) (P13.1; Q22.1) ;(CBFB::MYH11): A STUDY ON BEHALF OF THE ALWP OF THE EBMT

**Topic:** 23. Stem cell transplantation - Clinical

Arnon Nagler*[1], Jordi Esteve Reyner[2], Jacques-Emmanuel Galimard[3], Depei Wu[4], Jurjen Versluis[5], Anne Huynh[6], Edouard Forcade[7], Tobias Gedde-Dahl[8], Cristina Castilla Llorente[9], Peter von Dem Borne[10], Régis Peffault de Latour[11], Hélène Labussiere Wallet[12], Alessandro Rambaldi[13], Erlie Jiang[14], Maija Itala-Remes[15], Eric Deconinck[16], Francesca Kinsella[17], Urpu Salmenniemi[18], Jaime Sanz[19], Bruno Lioure[20], Ibrahim Yakoub-Agha[21], Bipin Savani[22], Ali Bazarbachi[23], Eolia Brissot[24], Mohamad Mohty[25], Fabio Ciceri[26]

*[1]Division of Hematology, Sheba Medical Center, Tel Hashomer, Israel;[2]Hematology Department, Institute of Cancer & Blood Disorders (ICAMS), IDIBAPS, Hospital Clinic, Barcelona, Spain, Barcelona, Spain; [3]EBMT Paris Study Unit; Department of Haematology, Saint Antoine Hospital; INSERM UMR 938, Sorbonne University, Paris, France; [4]First Affiliated Hospital of Soochow University, Suzhou, China; [5]Erasmus MC Cancer חInstitute, Rotterdam, The Netherlands; [6]6CHU - Institut Universitaire du Cancer Toulouse, Toulouse, France; [7]CHU Bordeaux, Hôpital Haut-Lévêque, Bordeaux, France;[8]Oslo University Hospital, Rikshospitalet, Oslo, Norway; [9]Gustave Roussy Cancer Campus, Villejuif, France; [10]Leiden University Hospital, Leiden, The Netherlands; [11]Saint-Louis Hospital, BMT Unit, Paris, France; [12]Centre Hospitalier Lyon Sud, Pierre Bénite, France, Lyon, France; [13]ASST Papa Giovanni XXIII, Bergamo, Italy, Bergamo, Italy; [14]nstitute of Hematology, Chinese Academy of Medical Sciences, Tianjin, China; [15]Turku University Hospital, Turku, Finland; [16]Hopital Jean Minjoz, Besançon, France; [17]Birmingham Centre for Cellular Therapy and Transplant (BCCTT), Birmingham, United Kingdom; [18]HUCH Comprehensive Cancer Center, Helsinki, Finland; [19]Departament de Medicina Universitat de Valencia, CIBERONC, Instituto Carlos III, Madrid, Spain;[20]ICANS - Institut de cancerologie Strasbourg Europe, Strasbourg, France; [21]CHU de Lille, Univ Lille, INSERM U1286, infinite, 59000, Lille, France; [22]Department of Hematology/Oncology, Department of Medicine, Vanderbilt University Medical Center, Nashville, United States of America; [23]Hematology-Oncology Division, Department of Internal Medicine, American University of Beirut Medical Center, Beirut, Lebanon; [24]Service d'hématologie clinique et thérapie cellulaire, Hôpital Saint-Antoine, APHP, Paris, France;[25]Sorbonne University, Department of Haematology, Saint Antoine Hospital; INSERM UMR 938, Paris, France; [26]Ospedale San Raffaele, Haematology and BMT, Milano, Italy;*

### Background

AML with inv (16) (p13.1; q22.1) or t (16;16) (p13.1; q22.1), resulting in the *CBFB::MYH11* fusion gene (AML *CBFB::MYH11*), is classified as a favorable-risk AML by the ELN 2022, irrespective of the presence of frequent secondary signaling co-mutations. Recently, the HARMONY Alliance developed and validated a novel unsupervised genomic classification of AML from a large cohort of patients (pts) (n=5,244), identifying 17 molecular classes with distinct clinical outcomes (Turki A., HemaSphere 2025, S148; Blood 2025; 146,1: 451). In this model, AML *CBFB::MYH11* pts were segregated by *FLT3*- ITD status, with *FLT3*-ITD conferring inferior prognosis. However, the impact of *FLT3*-ITD in the setting of allogeneic transplantation (HSCT) in AML *CBFB::MYH11* remains unclear.

### Aims

This study aimed to assess relapse incidence (RI) and other outcome variables in pts with CBFB::MYH11 AML who underwent HSCT between 2000 and 2024, while in CR, according to FLT3-ITD co-mutation in a registry-based real-life setting.

**Copyright Information:** (Online) ISSN: 2572-9241
©2026 The Author(s). HemaSphere is published by John Wiley & Sons Ltd on behalf of European Hematology Association. This is an open access Abstract Book distributed under the Attribution-NonCommercial-NoDerivs (CC BY-NC-ND), which allows third parties to download the articles and share them with others as long as they credit the author and the Abstract Book, but they cannot change the content in any way or use them commercially.
**Abstract Book Citations:** Authors, Title, HemaSphere, 2026;10:(S1):pages. The abstract book DOIs can be found on the "Author Information" page.
**Disclaimer:** Articles published in the journal HemaSphere exclusively reflect the opinions of the authors. The authors are responsible for all content in their abstracts including accuracy of the facts, statements, citing resources, etc.

### Methods

The Kaplan-Meier estimator was used to evaluate LFS and OS, while RI and NRM were calculated using the cumulative incidence estimator. The impact of *FLT3*-ITD was evaluated using a matched-pair analysis (MPA), with a maximum of 3 matches per case, matching on age, year of HSCT, patient sex, use of TBI, conditioning intensity, donor type, disease status, and female to male combination.

### Results

702 pts with AML *CBFB::MYH11* were included, 164 harbored *FLT3*-ITD (23.4%; median age 43.3 years (y), IQR 32.3–54.8) and 538 did not (76.6%; median age 42.1 y, IQR 30.6–50.9). For the MPA, 580 pts were included: 162 AML *CBFB::MYH11*/*FLT3*-ITD$^{+}$ and 418 AML *CBFB::MYH11*/*FLT3*-ITD$^{-}$. Median follow-up was 3.3 y (95% CI 2.4–4.1) and 3.8 y (95% CI 3.0–4.4), respectively. The main differences between cohorts were disease status, with a higher proportion of pts transplanted in CR1 among AML *CBFB::MYH11*/*FLT3*-ITD$^{+}$ pts (61.6% vs. 45.2%, p<0.001), and conditioning intensity, with more frequent use of myeloablative conditioning in AML *CBFB::MYH11*/*FLT3*-ITD$^{+}$ (69.5% vs. 60.0%, p=0.03). A numerically higher proportion of MRD-negativity at HSCT was observed in AML *CBFB::MYH11*/*FLT3*-ITD$^{+}$ (61.4% vs. 51.5%, p=0.08). No other significant differences were observed between cohorts, including patient sex, KPS ≥90, donor type, CMV, use of PTCy or *ATG*.

Outcomes after HSCT were not different between both groups (AML CBFB::MYH11/FLT3$^{+}$ vs. CBFB::MYH11/FLT3$^{-}$), with similar 2-year RI (15.3% vs. 18.9%, HR 1.00, 95% CI 0.67–1.50, p = 0.99), NRM (14.6% vs. 13.9%, HR 0.93, 95% CI 0.57–1.52, p = 0.78), LFS (70.2% vs. 67.3%, HR 0.97, 95% CI 0.77–1.34, p = 0.84), and OS (74.1% vs. 76.1%, HR 1.04, 95% CI 0.72–1.52, p = 0.83) (Figure 1A). The incidence of GVHD was also similar between groups, with non-different incidence of acute GVHD at day 100 (grade II–IV: 32.3% vs. 27.4%, HR 1.14, 95% CI 0.83–1.56, p = 0.41; grade III–IV: 9.8% vs. 11.3%, HR 0.79, 95% CI 0.44–1.40, p = 0.42) and chronic GVHD (total: 47.2% vs. 38.6%, HR 1.17, 95% CI 0.85–1.61, p = 0.33; extensive/severe: 16.5% vs. 13.0%, HR 1.14, 95% CI 0.67–1.95, p = 0.63) (Figure 1B).

### Summary/Conclusion

In this retrospective analysis of pts with AML CBFB::MYH11 undergoing HSCT in CR, the presence of FLT3-ITD co-mutation did not adversely impact transplant outcomes. Notably, no increase in RI was observed in AML CBFB::MYH11/FLT3$^{+}$ pts, suggesting that HSCT may overcome the adverse prognostic effect associated with this mutational event.

**Copyright Information:** (Online) ISSN: 2572-9241
©2026 The Author(s). HemaSphere is published by John Wiley & Sons Ltd on behalf of European Hematology Association. This is an open access Abstract Book distributed under the Attribution-NonCommercial-NoDerivs (CC BY-NC-ND), which allows third parties to download the articles and share them with others as long as they credit the author and the Abstract Book, but they cannot change the content in any way or use them commercially.
**Abstract Book Citations:** Authors, Title, HemaSphere, 2026;10:(S1):pages. The abstract book DOIs can be found on the “Author Information” page.
**Disclaimer:** Articles published in the journal HemaSphere exclusively reflect the opinions of the authors. The authors are responsible for all content in their abstracts including accuracy of the facts, statements, citing resources, etc.

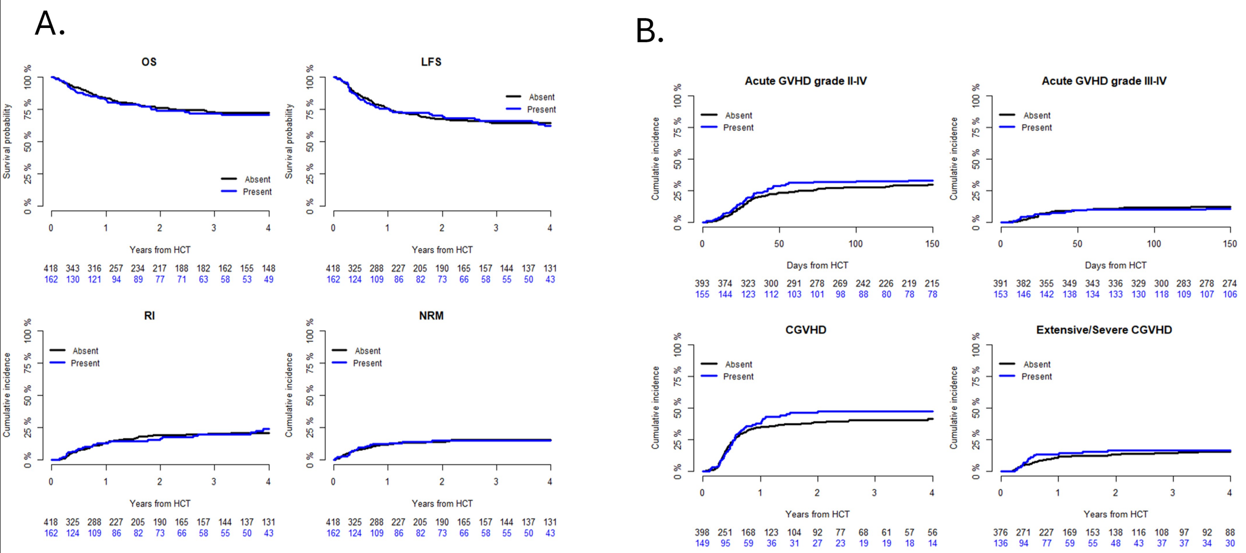

**A.** Transplantation outcomes in *inv*(16)/t(16;16) AML patients with FLT3 mutation (Present) versus *inv*(16)/t(16;16) AML patients without FLT3 mutation (Absent): overall survival (OS), leukemia-free survival (LFS), relapse incidence (RI), and non-relapse mortality (NRM). B. Graft-versus-host disease (GVHD) in *inv*(16)/t(16;16) AML patients with FLT3 mutation (Present) versus *inv*(16)/t(16;16) AML patients without FLT3 mutation (Absent): acute GVHD grades II–IV and III–IV; chronic GVHD total (cGVHD) and extensive/severe (extensive/severe cGVHD).

**Arnon Nagler[1]*, Jordi Esteve[2]*** *equal contribution

**Copyright Information:** (Online) ISSN: 2572-9241
©2026 The Author(s). HemaSphere is published by John Wiley & Sons Ltd on behalf of European Hematology Association. This is an open access Abstract Book distributed under the Attribution-NonCommercial-NoDerivs (CC BY-NC-ND), which allows third parties to download the articles and share them with others as long as they credit the author and the Abstract Book, but they cannot change the content in any way or use them commercially.
**Abstract Book Citations:** Authors, Title, HemaSphere, 2026;10:(S1):pages. The abstract book DOIs can be found on the "Author Information" page.
**Disclaimer:** Articles published in the journal HemaSphere exclusively reflect the opinions of the authors. The authors are responsible for all content in their abstracts including accuracy of the facts, statements, citing resources, etc.

### (S273) ALLOGENEIC HSCT FOR TP53-ABERRANT MYELOID NEOPLASMS: REAL-WORLD OUTCOMES FROM A SCANDINAVIAN MULTICENTRE COHORT

**Topic:** 23. Stem cell transplantation - Clinical

Andreas T Björklund[1], Henrik Tor Anderson Tvedt[2], Anne Stidsholt Roug[3], Mikael Lisak[4], Daniel Tuyet Kristensen[3], Emma Bergfelt Lennmyr[5], Lars Klingen Gjærde[6], Pål Tore Bentsen[7], Jörg Cammenga[8], Anders Eivind Myhre*[2]

*[1]Karolinska University Hospital, Department of Cell Therapy and Allogeneic Stem Cell Transplantation, Stocholm, Sweden;[2]Oslo University Hospital, Department of Haematology, Oslo, Norway; [3]Aarhus University Hospital, Department of Haematology, Aarhus, Denmark; [4]Sahlgrenska University Hospital, Department of Haematology, Gothenburg, Sweden;[5]Uppsala University Hospital, Department of Haematology, Uppsala, Sweden; [6]Copenhagen University Hospital, Rigshospitalet, Department of Haematology, Copenhagen, Denmark; [7]Haukeland University Hospital, Department of Medicine, Bergen, Norway;[8]Skåne University Hospital, Department of Haematology, Lund, Sweden;*

### Background

TP53-mutated myeloid neoplasms carry a dismal prognosis, and the benefit of allogeneic haematopoietic stem cell transplantation (allo-HSCT) remains controversial due to the high risk of post-transplant relapse, with long-term survival frequently reported at approximately 20%. Improvements in transplant outcomes overall have not clearly translated into better survival for TP53-mutated disease. While performance status, comorbidity and multi-hit disease consistently impact survival, the influence of conditioning and T-cell depletion remains less well defined. Scandinavian transplant centres apply broadly similar strategies for patient selection and transplant procedures, providing a harmonised real-world setting for benchmarking outcomes in this very high-risk population.

### Aims

To describe real-world outcomes after allo-HSCT in myeloid neoplasms with TP53 aberrations and identify clinical and molecular factors associated with long-term survival.

### Methods

We assembled a multicentre cohort of adult patients with TP53-aberrant myeloid neoplasms undergoing allo-HSCT at eight Scandinavian transplant centres between 2009 and 2025. Overall survival (OS) was estimated by Kaplan–Meier methodology; relapse and non-relapse mortality (NRM) were analysed using competing-risk methods. Associations with OS were explored using Cox regression, including Karnofsky performance status (KPS), comorbidity index (HCT-CI), and TP53 allelic status (biallelic/multi-hit per ICC 2022 criteria).

**Copyright Information:** (Online) ISSN: 2572-9241
©2026 The Author(s). HemaSphere is published by John Wiley & Sons Ltd on behalf of European Hematology Association. This is an open access Abstract Book distributed under the Attribution-NonCommercial-NoDerivs (CC BY-NC-ND), which allows third parties to download the articles and share them with others as long as they credit the author and the Abstract Book, but they cannot change the content in any way or use them commercially.
**Abstract Book Citations:** Authors, Title, HemaSphere, 2026;10:(S1):pages. The abstract book DOIs can be found on the “Author Information” page.
**Disclaimer:** Articles published in the journal HemaSphere exclusively reflect the opinions of the authors. The authors are responsible for all content in their abstracts including accuracy of the facts, statements, citing resources, etc.

### Results

A total of 169 first transplants were identified: 45% myelodysplastic syndrome (MDS), 51% acute myeloid leukaemia (AML), and 4% myeloproliferative neoplasm (MPN). Median age at transplant was 62 years (range 20–74). Treosulfan-based conditioning was used in 69% of transplants; 54% received myeloablative conditioning and 46% reduced-intensity or non-myeloablative conditioning. Unrelated donors accounted for 85% of grafts and peripheral blood stem cells were used in 95%. Anti-thymocyte globulin was administered in 73%.

In MDS-LB/IB1, 3-year OS was 44.3% (95% CI 32.8–55.2) with a cumulative incidence of relapse (CIR) of 55.1% (95% CI 43.4–65.4). In AML, 3-year OS was 33.1% (95% CI 23.2–43.3) and CIR was 59.7% (95% CI 48.5–69.3) (figure 1a).

Patients with KPS >80 had an estimated 3-year OS of 49.3%, compared with 30.2% for KPS ≤80. Biallelic TP53 was associated with inferior survival (HR 4.05, 95% CI 1.76–9.32; p=0.001) (figure 1b), reaching statistical significance only in the MDS subgroup. A low blast count at diagnosis was associated with improved survival (HR 0.50, 95% CI 0.29–0.88; p=0.02).

No clear associations were observed for age, HCT-CI, donor type, conditioning intensity or ATG. NRM did not differ significantly between MDS and AML (figure 1c).

### Summary/Conclusion

In this multicentre Scandinavian real-world cohort of 169 patients with TP53-aberrant myeloid neoplasms, 3-year survival rates exceeded those historically reported after allo-HSCT. Relapse remains the dominant cause of treatment failure, while patient fitness, biallelic TP53 status and blast burden at diagnosis identified patients with particularly poor outcomes, supporting careful candidate selection and refined molecular risk stratification.

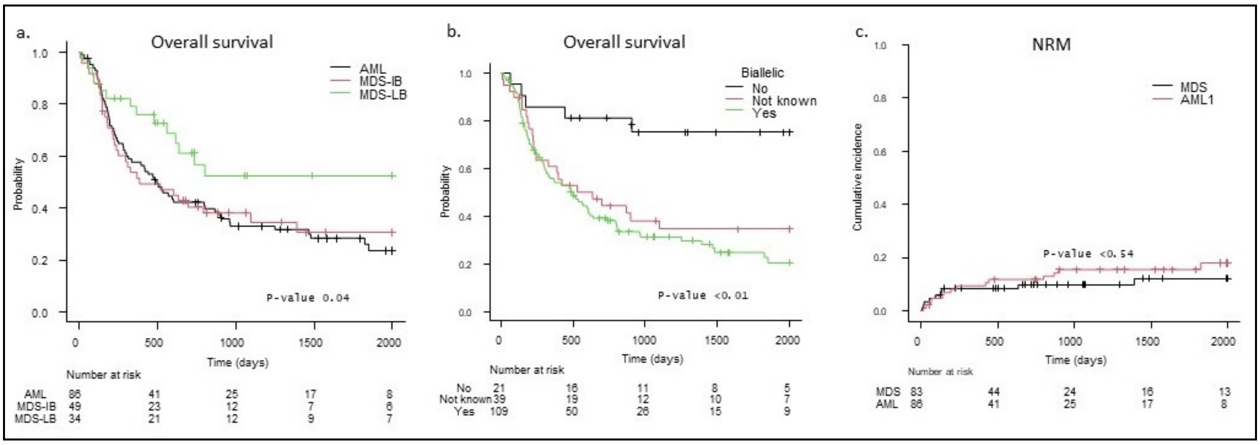

Figure 1. Outcomes after allo-HSCT in TP53-aberrant AML and MDS (n=169). (A) Kaplan–Meier estimates of OS stratified by diagnosis at transplant (AML, MDS-IB, MDS-LB). (B) Cumulative incidence of relapse stratified by TP53 biallelic (multi-hit) status (Yes/No/Not known). (C) Cumulative incidence of competing events, showing relapse and non-relapse mortality (NRM) after allo-HSCT. Time is shown in days post-transplant; numbers at risk are displayed below each panel.

**Copyright Information:** (Online) ISSN: 2572-9241
©2026 The Author(s). HemaSphere is published by John Wiley & Sons Ltd on behalf of European Hematology Association. This is an open access Abstract Book distributed under the Attribution-NonCommercial-NoDerivs (CC BY-NC-ND), which allows third parties to download the articles and share them with others as long as they credit the author and the Abstract Book, but they cannot change the content in any way or use them commercially.
**Abstract Book Citations:** Authors, Title, HemaSphere, 2026;10:(S1):pages. The abstract book DOIs can be found on the "Author Information" page.
**Disclaimer:** Articles published in the journal HemaSphere exclusively reflect the opinions of the authors. The authors are responsible for all content in their abstracts including accuracy of the facts, statements, citing resources, etc.

### (S274) A GENETIC–CLINICAL PROGNOSTIC ALGORITHM FOR RISK STRATIFICATION IN PATIENTS WITH RELAPSED/REFRACTORY ACUTE MYELOID LEUKAEMIA UNDERGOING ALLOGENEIC HAEMATOPOIETIC STEM CELL TRANSPLANTATION

**Topic:** 23. Stem cell transplantation - Clinical

Yue Jin*[1], Zhuo-Yu An[1], Peng Zhao[1], Haixia Fu[1], Ying Lu[2], Dingming Wan[3], Xi Zhang[4], Fang Zhou[5], Nainong Li[6], Li Liu[7], Pengcheng He[8], Shifeng Lou[9], Fei Li[10], Lu Wang[10], Han Zhu[11], Ruize Chen[11], Junjie Cao[2], Li Li[3], Xiao Hu[9], Li Li[4], Qianqian Xiao[5], Xufei Huang[6], Xueqian Yan[7], Xiaoning Wang[8], Yunya Luo[12], Mingyang Deng[12], Qiusha Huang[1], Li-Ping Yang[1], Chencong Wang[1], Fengxue Zhu[1], Xiaodong Mo[1], Xiang-Yu Zhao[1], Yu Wang[1], Yingjun Chang[1], Lanping Xu[1], Xiaojun Huang[1], Xiaohui Zhang[1]

*[1]Peking University People's Hospital, Peking University Institute of Hematology, National Clinical Research Center for Hematologic Disease, Beijing Key Laboratory of Cell and Gene Therapy for Hematologic Malignancies, Peking University, Beijing, China., Beijing, China; [2]Department of Hematology, The Affiliated People's Hospital of Ningbo University., Ningbo, China; [3]The First Affiliated Hospital of Zhengzhou University, Zhengzhou, China;[4]Medical Center of Hematology, Xinqiao Hospital of Army Medical University State Key Laboratory of Trauma and Chemical Poisoning Chongqing Key Laboratory of Hematology and Microenvironment., Chongqing, China; [5]Hematology Department, The 960th Hospital of The People's Liberation Army (PLA) Joint Logistics Support Force, Jinan, China; [6]Fujian Institute of Hematology,Fujian Provincial Key Laboratory on Hematology,Fujian Medical University Union Hospital, Fuzhou, China; [7]The Second Affiliated Hospital of Air Force Medical University, Xi'an, China; [8]Department of hematology, the first affiliated hospital of xi'an jiaotong university, Xi'an, China; [9]Department of Hematology, The Second Affiliated Hospital of Chongqing Medical University; Chongqing Medical University., Chongqing, China; [10]Senior Department of Hematology, The Fifth Medical Center of Chinese People's Liberation Army(PLA) General Hospital., Beijing, China; [11]Department of Hematology, Jiangsu Province Hospital, The First Affiliated Hospital of Nanjing Medical University., Nanjing, China; [12]The second xiangya hospital of central south university, Changsha, China;*

### Background

The prognosis of patients diagnosed with relapsed/refractory acute myeloid leukaemia (R/R AML) remains poor, and allogeneic haematopoietic stem cell transplantation (allo-HSCT) is a curative strategy. However, the prognosis of patients who relapse after HSCT is dismal, and non-relapse mortality (NRM) post-transplantation remains a significant concern. Although multiple risk factors for AML have been identified, an integrated algorithm incorporating both genetic and clinical features to inform clinical decision-making in the HSCT setting for R/R AML patients is lacking.

### Aims

The aims of this study were to investigate the clinical and genetic factors that are associated with the prognosis of patients with R/R AML and to develop a novel prognostic algorithm for R/R AML patients who are undergoing allo-HSCT.

**Copyright Information:** (Online) ISSN: 2572-9241
©2026 The Author(s). HemaSphere is published by John Wiley & Sons Ltd on behalf of European Hematology Association. This is an open access Abstract Book distributed under the Attribution-NonCommercial-NoDerivs (CC BY-NC-ND), which allows third parties to download the articles and share them with others as long as they credit the author and the Abstract Book, but they cannot change the content in any way or use them commercially.
**Abstract Book Citations:** Authors, Title, HemaSphere, 2026;10:(S1):pages. The abstract book DOIs can be found on the "Author Information" page.
**Disclaimer:** Articles published in the journal HemaSphere exclusively reflect the opinions of the authors. The authors are responsible for all content in their abstracts including accuracy of the facts, statements, citing resources, etc.

**Methods**

Data from patients who met the diagnostic criteria for R/R AML and had received allo-HSCT from 2018 to 2023 at Peking University People's Hospital were reviewed and included in the derivation group. Patients treated at 11 other HSCT centres in China were included in the validation group. Variables with a P value < 0.1 in univariable analyses were subsequently incorporated into a multivariable Cox proportional hazards regression model. A prognostic algorithm was constructed based on the final multivariable model, and risk points for the scoring system were assigned according to hazard ratios (HRs). The discriminative capacity of the algorithm was assessed using receiver operating characteristic (ROC) curves. Model validation was assessed through internal bootstrap validation and further evaluated through external validation in an independent cohort. Model calibration was evaluated in the internal and external validation cohorts. Clinical utility was assessed using decision curve analysis (DCA).

**Results**

In the current study, 301 R/R AML patients who underwent allo-HSCT were included in the derivation group. A total of 297 R/R AML patients were included in the external validation group. A comprehensive prognostic algorithm combining clinical factors and genetic abnormalities, including disease status before HSCT, patient and donor age, FLT3-ITD mutation, TP53 mutation, KMT2A rearrangement (KMT2Ar), and complex karyotypes, was developed for R/R AML patients treated with allo-HSCT. Patients were stratified into four risk groups: low-risk, intermediate-low-risk, intermediate-high-risk, and high-risk. In the internal validation cohort, the 1-, 2-, and 3-year area under the curve (AUC) values of overall survival (OS) were 0.825, 0.816, and 0.818, respectively. In the external validation cohort, the 1-, 2-, and 3-year AUC values of OS were 0.725, 0.756, and 0.789, respectively. The calibration plots indicated that the estimated and actual mortality risks were highly consistent in both the internal and external validation cohorts. DCA demonstrated that the novel algorithm consistently provided a greater net benefit than the ELN 2022 classification. In the derivation and validation cohorts, this algorithm exhibited superior utility across a wide threshold range.

**Summary/Conclusion**

This algorithm could refine risk stratification in R/R AML patients undergoing allo-HSCT and potentially inform combination therapy strategies and post-transplantation monitoring.

**Copyright Information:** (Online) ISSN: 2572-9241
©2026 The Author(s). HemaSphere is published by John Wiley & Sons Ltd on behalf of European Hematology Association. This is an open access Abstract Book distributed under the Attribution-NonCommercial-NoDerivs (CC BY-NC-ND), which allows third parties to download the articles and share them with others as long as they credit the author and the Abstract Book, but they cannot change the content in any way or use them commercially.
**Abstract Book Citations:** Authors, Title, HemaSphere, 2026;10:(S1):pages. The abstract book DOIs can be found on the "Author Information" page.
**Disclaimer:** Articles published in the journal HemaSphere exclusively reflect the opinions of the authors. The authors are responsible for all content in their abstracts including accuracy of the facts, statements, citing resources, etc.

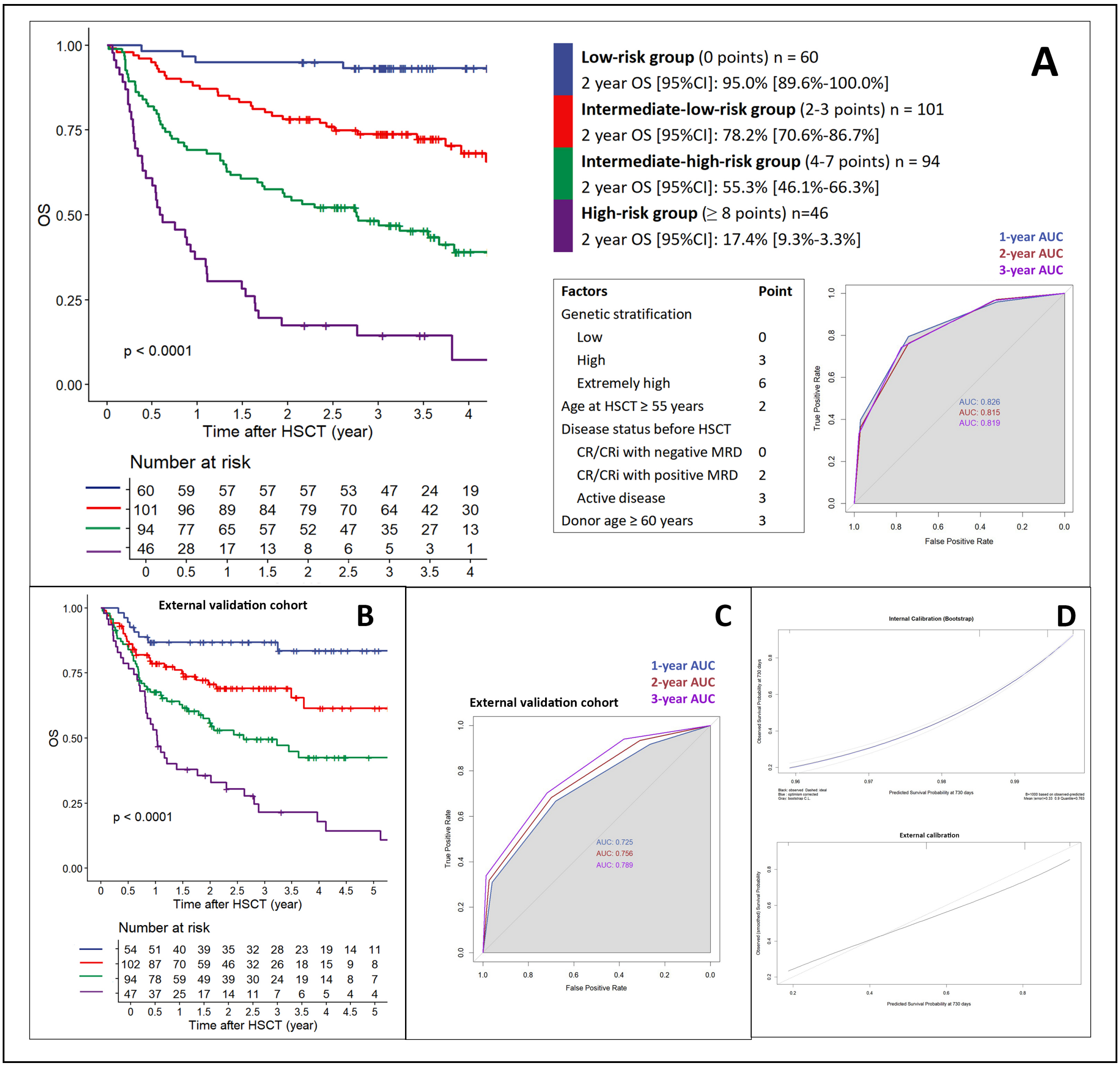

**Copyright Information:** (Online) ISSN: 2572-9241
©2026 The Author(s). HemaSphere is published by John Wiley & Sons Ltd on behalf of European Hematology Association. This is an open access Abstract Book distributed under the Attribution-NonCommercial-NoDerivs (CC BY-NC-ND), which allows third parties to download the articles and share them with others as long as they credit the author and the Abstract Book, but they cannot change the content in any way or use them commercially.
**Abstract Book Citations:** Authors, Title, HemaSphere, 2026;10:(S1):pages. The abstract book DOIs can be found on the "Author Information" page.
**Disclaimer:** Articles published in the journal HemaSphere exclusively reflect the opinions of the authors. The authors are responsible for all content in their abstracts including accuracy of the facts, statements, citing resources, etc.

### (S275) ULTRA-SENSITIVE METABOLOMICS UNCOVERS L-CARNITINE AS A CRITICAL REGULATOR OF HUMAN HEMATOPOIETIC STEM CELL REGENERATION

**Topic:** 24. Hematopoiesis, stem cells and microenvironment

Honglin Duan*[1], Bohong Wang[2], Yawei Zheng[1], Erlie Jiang[1], Tao Cheng[1], Zeping Hu[2], Hui Cheng[1]

[1]*Institute of Hematology & Blood Diseases Hospital, Chinese Academy of Medical Sciences & Peking Union Medical College, Tianjin, China;* [2]*School of Pharmaceutical Sciences, Tsinghua University, Beijing, China;*

### Background

Understanding how metabolism governs human hematopoietic stem cell (HSC) function is critical for advancing regenerative therapies. However, direct metabolic profiling of these rare cells has been hindered by their scarcity and the technical limitations of conventional methods. While studies in murine models have identified key metabolic regulators, species differences limit their translational relevance. A comprehensive metabolic atlas of human hematopoiesis, essential for identifying targetable pathways to enhance HSC function in clinical settings like transplantation, remains largely uncharted.

### Aims

This study aims to systematically map the metabolic landscape of human hematopoiesis using an ultra-sensitive platform, and to identify key metabolic pathways regulating HSC regenerative capacity. Specifically, we sought to uncover targetable metabolic features of human hematopoietic stem and progenitor cells (HSPCs) and evaluate their functional relevance in both homeostasis and bone marrow failure conditions.

### Methods

We applied an optimized ultra-sensitive mass spectrometry-based metabolomics platform to profile 13 immunophenotypically defined hematopoietic cell types sorted from the bone marrow of 18 healthy donors, using as few as ~10,000 cells per sample. Functional validation employed genetic knockdown, L-carnitine supplementation, colony-forming unit assays, and xenotransplantation models using human cord blood or patient-derived HSPCs. Multi-omics analyses (RNA-seq, ATAC-seq, CUT&Tag) and metabolic assays were integrated to dissect underlying mechanisms.

### Results

We generated a foundational metabolomic atlas, revealing conserved metabolic hallmarks of primitive HSPCs and highlighting lineage-specific rewiring of key bioenergetic and biosynthetic pathways during differentiation. Notably, we identified L-carnitine-driven fatty acid oxidation (FAO) as a key metabolic feature supporting HSPC function. Mechanistically, L-carnitine activates the PPARA-TFEB signaling axis, promoting mitochondrial metabolism and lysosomal autophagy. Genetic inhibition of the L-carnitine transporter OCTN2 impaired FAO, suppressed TFEB activity, and compromised HSPC regeneration both in vitro and in vivo. Conversely, L-carnitine supplementation enhanced FAO, boosted mitochondrial function, and improved the regenerative capacity of healthy donor HSPCs. Crucially, L-carnitine treatment also restored metabolic integrity and function in HSPCs derived from patients with aplastic anemia.

**Copyright Information:** (Online) ISSN: 2572-9241
©2026 The Author(s). HemaSphere is published by John Wiley & Sons Ltd on behalf of European Hematology Association. This is an open access Abstract Book distributed under the Attribution-NonCommercial-NoDerivs (CC BY-NC-ND), which allows third parties to download the articles and share them with others as long as they credit the author and the Abstract Book, but they cannot change the content in any way or use them commercially.
**Abstract Book Citations:** Authors, Title, HemaSphere, 2026;10:(S1):pages. The abstract book DOIs can be found on the “Author Information” page.
**Disclaimer:** Articles published in the journal HemaSphere exclusively reflect the opinions of the authors. The authors are responsible for all content in their abstracts including accuracy of the facts, statements, citing resources, etc.

**Summary/Conclusion**

This study provides a comprehensive metabolomic reference for human hematopoiesis and reveals L-carnitine-driven FAO, mediated through the PPARA-TFEB axis, as a critical regulator of human HSPC regeneration. The findings identify a therapeutically targetable metabolic circuit with potential to enhance stem cell-based therapies, not only under homeostasis but also in aplastic anemia condition.

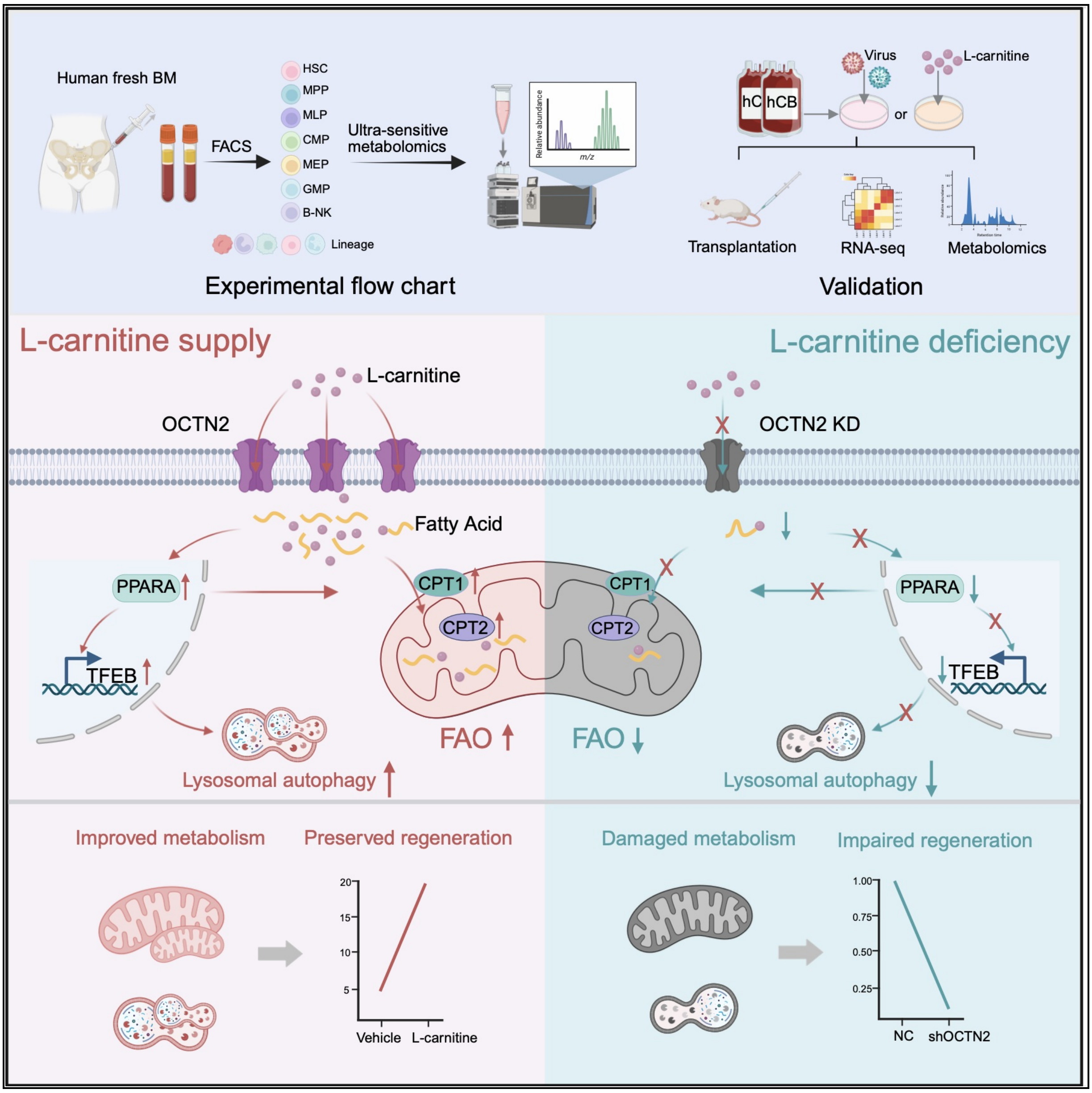

**Copyright Information:** (Online) ISSN: 2572-9241
©2026 The Author(s). HemaSphere is published by John Wiley & Sons Ltd on behalf of European Hematology Association. This is an open access Abstract Book distributed under the Attribution-NonCommercial-NoDerivs (CC BY-NC-ND), which allows third parties to download the articles and share them with others as long as they credit the author and the Abstract Book, but they cannot change the content in any way or use them commercially.
**Abstract Book Citations:** Authors, Title, HemaSphere, 2026;10:(S1):pages. The abstract book DOIs can be found on the "Author Information" page.
**Disclaimer:** Articles published in the journal HemaSphere exclusively reflect the opinions of the authors. The authors are responsible for all content in their abstracts including accuracy of the facts, statements, citing resources, etc.

### (S276) A TIME-RESOLVED SINGLE-NUCLEUS MULTIOME ATLAS OF HUMAN FETAL LIVER HEMATOPOIESIS

**Topic:** 24. Hematopoiesis, stem cells and microenvironment

Yiwei Niu*[1], Mervin Fansler[1], Weiyan Chen[1], Lisha Yuan[1], George Hunt[1], Ulf Tiemann[1], Pernille Rainer[1], Rebecca Moller[1], Ana Boavida[1], Xiaoling He[2, 3], Roger Barker[2, 3], Ana Cvejic[1]

*[1]University of Copenhagen, Biotech Research & Innovation Centre (BRIC), Copenhagen, Denmark; [2]Cambridge Stem Cell Institute, University of Cambridge, Cambridge, United Kingdom; [3]University of Cambridge, John van Geest Centre for Brain Repair, Department of Clinical Neurosciences, Cambridge, United Kingdom;*

**Background**

The fetal liver is the primary site of hematopoiesis during early human development. However, due to the limited tissue access, small sample sizes, sparse time-points, and reliance on transcriptomic data alone the timing and regulatory mechanisms of hematopoietic stem cell (HSC) functional maturation and early blood formation remain incompletely understood.

**Aims**

To define the cellular, transcriptional, and epigenetic changes underlying human fetal liver hematopoiesis over development.

**Methods**

We generated a high-resolution, time-resolved single-nucleus multiome atlas of human fetal liver spanning 5–18 post-conception weeks (PCW). In total, 308,021 nuclei from 53 donors were profiled using paired chromatin accessibility and transcriptomic modalities. We integrated cell-state annotation, cell-cycle inference, gene regulatory network analysis, and DNA methylation profiling to characterize developmental dynamics.

**Results**

Our analysis revealed that HSCs colonized the fetal liver by 5 PCW but initially lacked differentiation competence. Early hematopoiesis was instead sustained by HSC-independent progenitors until their exhaustion around 8 PCW. Between 5 and 18 PCW, HSCs progressively acquired differentiation competence and transitioned from highly proliferative to quiescent states. Quiescent HSCs exhibited a distinct transcriptional and epigenetic signature (e.g., *MECOM*, *HLF*, *PRDM16*, *MLLT3*) and cell cycle readiness factors including *CDK6*, enabling stemness maintenance while remaining poised for cell-cycle re-entry. Mechanistically, we identified a close coupling between cell-cycle dynamics and fate competence in HSCs. The acquisition of differentiation competence coincided with the progressive G1 phase lengthening, creating a permissive window for lineage priming through selective accessibility of lineage-associated transcription factor motifs. Over developmental time, regulatory networks underwent sustained and stage-specific rewiring, characterized by increasing diversity and complexity over development. Consistent with these changes, DNA methylation changes were associated with the repression of cell-cycle and early developmental genes, as well as the activation of antigen presentation pathways.

**Summary/Conclusion**

This study uncovered a dynamic, multi-layered regulatory landscape underlying human fetal liver hematopoiesis and provided a comprehensive atlas that serves as a framework for understanding early blood development, HSC maturation, and regulatory network remodeling. The resulting resource also offers an important reference for investigating the developmental origins of pediatric leukemias and for informing efforts to engineer functional HSCs for therapeutic applications.

**Copyright Information:** (Online) ISSN: 2572-9241
©2026 The Author(s). HemaSphere is published by John Wiley & Sons Ltd on behalf of European Hematology Association. This is an open access Abstract Book distributed under the Attribution-NonCommercial-NoDerivs (CC BY-NC-ND), which allows third parties to download the articles and share them with others as long as they credit the author and the Abstract Book, but they cannot change the content in any way or use them commercially.
**Abstract Book Citations:** Authors, Title, HemaSphere, 2026;10:(S1):pages. The abstract book DOIs can be found on the "Author Information" page.
**Disclaimer:** Articles published in the journal HemaSphere exclusively reflect the opinions of the authors. The authors are responsible for all content in their abstracts including accuracy of the facts, statements, citing resources, etc.

### (S277) THE LANDSCAPE OF SOMATIC RETROTRANSPOSON MUTATIONS IN HSCS, AND A NOVEL JAK2 SIGNALLING PATHWAY TO HNRNPA1 THAT REPRESSES RETROTRANSPOSON ACTIVITY

**Topic:** 24. Hematopoiesis, stem cells and microenvironment

Stephen Loughran*[1], Jeremy Deuel[1, 2], Andrejs Makarovs[1], Sheidasadat Goosheh[1], Sophie Webster[3], George Winder[1], Hisham Mohammed[4, 5], Jason Carroll[5], Michael Spencer Chapman[3], Chiraag Kapadia[3], Peter Campbell[3], Elisa Laurenti[1], Constanze Schneider[6], Thomas Oellerich[6], Jyoti Nangalia[3], Tony Green[1]

*[1]University of Cambridge, Cambridge Stem Cell Institute, Cambridge, United Kingdom; [2]University Hospital Zurich, Department of Medical Oncology and Haematology, Zurich, United Kingdom; [3]Wellcome Sanger Institute, Hinxton, United Kingdom; [4]Oregon Health & Science University, Knight Cancer Institute, Portland, United States of America; [5]University of Cambridge, CRUK Cambridge Institute, Cambridge, United Kingdom; [6]University Hospital Frankfurt, Department of Medicine 2, Frankfurt, Germany;*

### Background

Retrotransposon (RT) expression must be tightly controlled, particularly in long-lived multipotent stem cells, to prevent deleterious consequences including insertional mutations. Little is known about retrotransposon expression and mobility in haematopoietic stem cells (HSCs) or how these processes might be dynamically regulated.

### Aims

We aimed to detect whether insertional mutations derived from RTs contribute to the somatic mutation landscape of human and mouse HSCs. We also determined whether RTs are dynamically regulated during cytokine-induced HSC cell cycling, when the genome is vulnerable to the insertion of new copies of retrotransposon DNA.

### Methods

We developed PEAR-TREE, a new tool to detect somatic RT mutations in whole genome sequences from single-HSC-derived colonies.

RT RNA was quantified in single cell RNA-seq where thrombopoietin (THPO) or JAK2 signalling was activated in HSCs.

Proteins phosphorylated downstream of THPO and JAK2 were identified using immunoprecipitation of JAK2 or phosphotyrosine followed by quantiative SILAC mass spectrometry.

HNRNPA1 phosphomutations were generated using CRISPR/Cas9 gene editing.

RT mutation rates in polyclonal populations were quantified using a LINE1-GFP reporter plasmid, and PARTRIDGE, a new technique using capture hybridisation to detect mutations caused by intracisternal A particle endogenous retroviruses (IAP ERVs).

**Copyright Information:** (Online) ISSN: 2572-9241
©2026 The Author(s). HemaSphere is published by John Wiley & Sons Ltd on behalf of European Hematology Association. This is an open access Abstract Book distributed under the Attribution-NonCommercial-NoDerivs (CC BY-NC-ND), which allows third parties to download the articles and share them with others as long as they credit the author and the Abstract Book, but they cannot change the content in any way or use them commercially.
**Abstract Book Citations:** Authors, Title, HemaSphere, 2026;10:(S1):pages. The abstract book DOIs can be found on the "Author Information" page.
**Disclaimer:** Articles published in the journal HemaSphere exclusively reflect the opinions of the authors. The authors are responsible for all content in their abstracts including accuracy of the facts, statements, citing resources, etc.

**Results**

We provide the first evidence that RTs are active in HSCs, observing 460 somatic mutations derived from retrotransposons within the human HSC population across a lifetime (95% confidence interval 126-1456). The mouse HSC population acquires 4610 mutations over their shorter lifetime.

Mouse HSCs and human colonic stem cells had similar RT mutation rates, both much higher than the human HSC rate, indicating unexpected species- and tissue-specific differences in RT regulation. Further, we observed surprising RT class-specific regulation between tissues, with LINEs being more repressed relative to SINEs in HSCs than in colonic stem cells.

We explored whether RTs are dynamically regulated during cytokine-induced HSC cycling, and detected a diverse range of LINE, SINE and endogenous retrovirus (ERV) mRNAs downregulated by THPO treatment in human cells and mouse HSCs. We observed similar RT downregulation in HSCs carrying mutations that hyperactivate THPO/JAK2 signalling from mouse models and human MPN patients. RT mutation rate was quantified in 10 MPN patients, none were detected in the JAK2 V617F clone, a significantly lower RT mutation rate than the JAK2 wildtype HSCs from the same patients, confirming that JAK2 represses RT activity.

The RNA-binding protein HNRNPA1 is phosphorylated by JAK2 downstream of THPO treatment. Analysis of HSCs from phophomutant mice indicated that HNRNPA1 phosphophorylation accounted for at least one third of THPO/JAK2-mediated RT mRNA repression. HNRNPA1 phosphorylation also reduced mutations caused by a LINE-1 reporter in a human cell line, and by IAP ERVs in mouse bone marrow.

**Summary/Conclusion**

Here we provide the first evidence that RTs are actively contributing to the mutational landscape of HSCs.

We also identify a novel THPO/JAK2/HNRNPA1 pathway that represses RT activity. Importantly this provides a dynamic mechanism that integrates RT repression with cytokine-induced proliferation; protecting the HSC genome while it is most vulnerable to RT mutations.

**Copyright Information:** (Online) ISSN: 2572-9241
©2026 The Author(s). HemaSphere is published by John Wiley & Sons Ltd on behalf of European Hematology Association. This is an open access Abstract Book distributed under the Attribution-NonCommercial-NoDerivs (CC BY-NC-ND), which allows third parties to download the articles and share them with others as long as they credit the author and the Abstract Book, but they cannot change the content in any way or use them commercially.
**Abstract Book Citations:** Authors, Title, HemaSphere, 2026;10:(S1):pages. The abstract book DOIs can be found on the "Author Information" page.
**Disclaimer:** Articles published in the journal HemaSphere exclusively reflect the opinions of the authors. The authors are responsible for all content in their abstracts including accuracy of the facts, statements, citing resources, etc.

### (S278) MITOCHONDRIAL DNA MOSAICISM AND CLONALITY IN HUMAN HEMATOPOIETIC AGING AND REGENERATION

**Topic:** 24. Hematopoiesis, stem cells and microenvironment

Lena Nitsch[1, 2, 3], Pauline Kautz[1, 2, 4], Ambre Giguelay*[1, 2], Janet Liebold[1, 2], Marie Cotta[1, 2], Julia Kühlwein[1, 2, 3], Kim Kremser[1, 2], Yajie Yin[5, 6], Jacob Gutierrez[7], Coral Fustero-Torre[1, 2, 8], Lena Oevermann[9], Livius Penter[8], Mareike Frick[8], Frederik Damm[8], Ansuman Satpathy[5, 6], Caleb Lareau[7], Leif Ludwig[1, 2]

*[1]Max-Delbrück-Center for Molecular Medicine in the Helmholtz Association (MDC), Institute for Medical Systems Biology (BIMSB), Berlin, Germany; [2]Berlin Institute of Health at Charité, Charité - Universitätsmedizin Berlin, Berlin, Germany;[3]Freie Universität Berlin, Berlin, Germany; [4]Technische Universität Berlin, Berlin, Germany;[5]Stanford University School of Medicine, Center for Cancer Cell Therapy, Stanford Cancer Institute, Standford, United States of America; [6]Parker Institute for Cancer Immunotherapy, San Francisco, United States of America; [7]Memorial Sloan Kettering Cancer Center, Computational and Systems Biology Program, New York, United States of America; [8]Charité-Universitätsmedizin Berlin, Department of Hematology, Oncology, and Tumor Immunology, Berlin, Germany; [9]Charité - Universitätsmedizin Berlin, Department of Pediatric Oncology and Hematology, Berlin, Germany;*

### Background

The tracking of somatic nuclear DNA mutations has enabled reconstructing clonal dynamics and high-resolution phylogenies to detect alterations in hematopoietic clonal architecture in human aging and disease. However, current approaches remain limited by their relatively low throughput and compatibility to attain additional information about cellular state. More recently, somatic mitochondrial DNA (mtDNA) mutations have emerged as natural clonal barcodes that enable significantly more scalable profiling of large cell numbers in conjunction with single-cell genomic profiles, opening new avenues for clonal tracing in humans.

### Aims

Here, we first define the mutational dynamics of somatic mtDNA variation in hematopoietic cells and subsequently use this foundation to develop a more quantitative understanding of the clonal architecture underlying human hematopoiesis during aging and regeneration.

### Methods

We performed mtscATAC-seq on collectively 94 bone marrow and peripheral blood samples, including (i) a healthy aging cohort spanning 0 to 74 years; (ii) longitudinal samples collected over a period up to three years from mid-aged healthy donors; and (iii) leukemic patients following hematopoietic stem and progenitor cells transplantation. Across these samples, we successfully co-captured chromatin accessibility profiles and mtDNA mutations profiles of >1.25 million single cells.

**Copyright Information:** (Online) ISSN: 2572-9241
©2026 The Author(s). HemaSphere is published by John Wiley & Sons Ltd on behalf of European Hematology Association. This is an open access Abstract Book distributed under the Attribution-NonCommercial-NoDerivs (CC BY-NC-ND), which allows third parties to download the articles and share them with others as long as they credit the author and the Abstract Book, but they cannot change the content in any way or use them commercially.
**Abstract Book Citations:** Authors, Title, HemaSphere, 2026;10:(S1):pages. The abstract book DOIs can be found on the "Author Information" page.
**Disclaimer:** Articles published in the journal HemaSphere exclusively reflect the opinions of the authors. The authors are responsible for all content in their abstracts including accuracy of the facts, statements, citing resources, etc.

**Results**

We observed an age-associated increase in mitochondrial genetic variation, confidently detecting several thousand somatic mtDNA mutations per individual. Importantly, cord blood was relatively depleted of mutations, suggesting that somatic mtDNA events mostly arise after the hematopoietic stem cell pool has been established. Most of the mtDNA mutations were present at low pseudo bulk allele frequencies (<1%), and occurred independently of each other, thereby marking independent clones. This pattern is consistent with ongoing mitochondrial genetic drift and provides insight into its dynamics across hematopoiesis. In the context of longitudinal sampling, we reveal largely stable clonal activities under homeostatic conditions, whereas a more dynamic clonal landscape emerges during hematopoietic regeneration, with evidence of clonal expansion events and decreased diversity. Importantly, even clones with low cellular fractions were repeatedly detected across time points, showing the robustness and sensitivity of our approach. Notably, most evidence surrounding a pronounced lineage bias of hematopoietic clones under physiological conditions could be attributed to immune cell expansions, rather than indicating intrinsic heterogeneity at the level of stem cells. In contrast, a clear and progressive myeloid bias emerged primarily at an advanced age and in individuals with clonal hematopoiesis.

**Summary/Conclusion**

Overall, our results align with the respective biological contexts and demonstrate that mtDNA mutations sensitively capture context-dependent variation in human clonality at unprecedented cellular scale. To our knowledge, it constitutes one of the first and most comprehensive longitudinally resolved evaluations of clonal dynamics at single-cell resolution in humans and paves the way for high-throughput and clinically applicable monitoring of clonal architecture and evolution using mtDNA variation.

**Copyright Information:** (Online) ISSN: 2572-9241
©2026 The Author(s). HemaSphere is published by John Wiley & Sons Ltd on behalf of European Hematology Association. This is an open access Abstract Book distributed under the Attribution-NonCommercial-NoDerivs (CC BY-NC-ND), which allows third parties to download the articles and share them with others as long as they credit the author and the Abstract Book, but they cannot change the content in any way or use them commercially.
**Abstract Book Citations:** Authors, Title, HemaSphere, 2026;10:(S1):pages. The abstract book DOIs can be found on the “Author Information” page.
**Disclaimer:** Articles published in the journal HemaSphere exclusively reflect the opinions of the authors. The authors are responsible for all content in their abstracts including accuracy of the facts, statements, citing resources, etc.

### (S279) MAPPING HEMATOPOIETIC STEM CELL NICHE INTERACTIONS AT SINGLE-CELL AND CLONAL RESOLUTION IDENTIFIES ALTERED CROSSTALK AND NOVEL TARGETS: Β-THALASSEMIA AS A CASE STUDY OF INHERITED BLOOD DISORDERS

**Topic:** 24. Hematopoiesis, stem cells and microenvironment

Annamaria Aprile*[1], Stefano Beretta[1], Trine Kristiansen[2, 3, 4], Mariangela Storto[1], Martina Scalera[1], Silvia Sighinolfi[1], Erica Fogliazza[1], Christina Mayerhofer[2, 3, 4], Foivos Chatzidimitriou[5], Ivan Merelli[1, 6], David Scadden[2, 3, 4], Giuliana Ferrari[1, 7]

*[1]San Raffaele-Telethon Institute for Gene Therapy (SR-Tiget), IRCCS San Raffaele Scientific Institute, Milan, Italy;[2]Center for Regenerative Medicine, Massachusetts General Hospital, Boston, United States of America; [3]Harvard Stem Cell Institute, Cambridge, United States of America; [4]Department of Stem Cell and Regenerative Biology, Harvard University, Cambridge, United States of America; [5]Center for Systems Biology, Massachusetts General Hospital Research Institute and Department of Genetics, Harvard Medical School, Boston, United States of America; [6]Institute for Biomedical Technologies, National Research Council, Segrate, Italy; [7]Vita-Salute San Raffaele University, Milan, Italy;*

### Background

The bone marrow (BM) stromal microenvironment finely regulates hematopoietic stem cells (HSC), however its alteration in blood disorders remains understudied. Unraveling the deregulated BM interactions in disease context can be relevant to improve curative transplantation and gene therapy. The genetic anemia β-thalassemia (Bthal) represents a paradigmatic model of chronic HSC and BM niche perturbation, as we recently demostrated in mice and patients (*Aprile et al., Blood 2020 & Sci Transl Med 2023; Crippa et al., JCI 2019; Lidonnici et al., Nat Comm 2025*).

### Aims

We constructed a comprehensive single-cell and clonal map of HSC-stromal niche interactions to elucidate mechanisms of Bthal niche dysregulation and identify potential therapeutic targets to restore niche function during disease progression and therapy.

### Methods

We used sc-RNAseq and CARLIN *in vivo* lineage tracing to explore the transcriptome and clonal architecture of BM hematopoietic stem and progenitor cells (HSPC) and stroma from Bthal $Hbb^{th3/+}$ (*th3*) mice. We defined ligand-receptor networks by CellChat and stromal subsets were quantified by flow cytometry. α-catulin $^{GFP/+}$ *th3* HSC-reporter strain was generated to validate the spatial organization of HSC and stroma. We tested the efficacy of stromal niche targeting by *in vivo* FGF23 inhibition (*Aprile et al., Sci Transl Med 2023*).

**Copyright Information:** (Online) ISSN: 2572-9241
©2026 The Author(s). HemaSphere is published by John Wiley & Sons Ltd on behalf of European Hematology Association. This is an open access Abstract Book distributed under the Attribution-NonCommercial-NoDerivs (CC BY-NC-ND), which allows third parties to download the articles and share them with others as long as they credit the author and the Abstract Book, but they cannot change the content in any way or use them commercially.
**Abstract Book Citations:** Authors, Title, HemaSphere, 2026;10:(S1):pages. The abstract book DOIs can be found on the "Author Information" page.
**Disclaimer:** Articles published in the journal HemaSphere exclusively reflect the opinions of the authors. The authors are responsible for all content in their abstracts including accuracy of the facts, statements, citing resources, etc.

**Results**

sc-RNAseq of *th3* HSPC highlighted significant alterations within primitive HSC, characterized by downregulation of stemness genes (*Jun*, *Runx1*, *Meis1*) and upregulation of proliferation-associated programs, assessed at clonal level as a higher proportion of active clones (40 *th3* vs 22% wt).
The BM stroma counterpart displayed instead downregulated pathways related to cell-cycle activation and metabolism. We observed reduced frequencies of LeptinR$^+$ mesenchymal stromal cells (L-MSC) and sinusoidal endothelial cells (sEC), as confirmed by immunophenotype analysis, with decreased expression of niche factors (*Spp1*, *Bglap*, *Cdh2*).
The analysis of matched HSPC and stromal cells enabled the construction of interactome maps, revealing globally dampened molecular crosstalk. L-MSC and sEC ligand-receptor interactions with HSC were the most reduced. Shared downregulated interactions included *Spp1-Itga4/Itgb1* and *Fn1-Cd44*, while population-specific ones were *Kitl-Kit* (L-MSC) and *Tgfb1-Tgfbr1/2* (sEC). These alterations identified candidate targets to restore a balanced niche crosstalk, supported by ongoing imaging, spatial transcriptomics and functional assays.
Niche targeting by FGF23 inhibition increased the number of HSC quiescent clones, L-MSC frequency and HSC-sEC interactions, ameliorating BM dysfunction.

**Summary/Conclusion**

This study provides the first integrated single-cell and clonal-resolution atlas of HSC and niche alterations in an inherited blood disorder. By combining gold-standard techniques, we uncovered novel alterations of HSC-niche interactions in Bthal, particularly involving L-MSC and sEC. These findings refine our understanding of pathophysiology and highlight potential targets to therapeutically restore the BM microenvironment. This conceptual and experimental framework is readily applicable to other blood disorders characterized by HSC or niche impairment.

**Copyright Information:** (Online) ISSN: 2572-9241
©2026 The Author(s). HemaSphere is published by John Wiley & Sons Ltd on behalf of European Hematology Association. This is an open access Abstract Book distributed under the Attribution-NonCommercial-NoDerivs (CC BY-NC-ND), which allows third parties to download the articles and share them with others as long as they credit the author and the Abstract Book, but they cannot change the content in any way or use them commercially.
**Abstract Book Citations:** Authors, Title, HemaSphere, 2026;10:(S1):pages. The abstract book DOIs can be found on the "Author Information" page.
**Disclaimer:** Articles published in the journal HemaSphere exclusively reflect the opinions of the authors. The authors are responsible for all content in their abstracts including accuracy of the facts, statements, citing resources, etc.

### (S280) MULTIPLEX PRIME EDITING ENABLES SAFE, DRUG-CONTROLLABLE, AND IMMUNOSUPPRESSION-RESISTANT T-CELL THERAPIES

**Topic:** 25. Gene therapy, cellular immunotherapy and vaccination - Biology & translational research

Thomas Lew*[1, 2], Esther Bandala Sanchez[1], Kerry Ramsay[1], Adrienne Hilton[1], Christine White[1], Naiara Garcia Bediaga[1], Connie Li Wai Suen[1], Shalin Niak[1], Carrie van der Weyden[2], Lucy Fox[2], David Ritchie[2], Gaurav Sutrave[3], Kenneth Micklethwaite[3], David Gottlieb[3], Samantha Chan[1], Andrew Roberts[1, 2], Charlotte Slade[1], Miles Horton[1]

*[1]Walter and Eliza Hall Institute of Medical Research,, Melbourne, Australia;[2]Peter MacCallum Cancer Centre & the Royal Melbourne Hospital, Melbourne, Australia; [3]University of Sydney and Westmead Hospital, Westmead, Australia;*

### Background

Current cell and gene therapies lack practical mechanisms to selectively promote or suppress therapeutic cells *in vivo*. This challenge is particularly acute in gene therapy for immune dysregulation syndromes and antigen-specific or chimeric antigen receptor (CAR) T-cell therapy in transplant recipients, where immunosuppression may inhibit both pathogenic and therapeutic cells. While gene editing could address this limitation, existing approaches have significant drawbacks: lentiviral transduction risks insertional oncogenesis, CRISPR/Cas9 introduces genotoxic double-strand breaks, and base editing is restricted to transition mutations. In contrast, prime editing enables precise and versatile modifications without double-strand breaks.

### Aims

To develop a multiplex prime-editing platform that: (1) corrects pathogenic variants in primary human T-cells; (2) installs resistance mutations to commonly used immunosuppressants, converting standard drugs into tools for *in vivo* selection and safety control; and (3) demonstrates broad applicability across T-cell therapies.

### Methods

Primary T-cells from healthy donors and patients with *HAVCR2*-driven subcutaneous panniculitis-like T-cell lymphoma (SPTCL) were prime edited using optimized pegRNAs. Editing efficiency was quantified by targeted sequencing. Off-target effects were evaluated using CIRCLE-seq, transcriptional profiling, immunophenotyping, and TCR sequencing. Drug-resistance variants were introduced into the targets of tacrolimus (*FKBP1A*), methotrexate (*DHRF*) and mycophenolate (*IMPDH2*). Functional validation was performed *in vitro* and in NSG xenografts models under immunosuppression. The platform was extended to CD19 CAR and CMV-specific virus-specific T-cells (VSTs).

**Copyright Information:** (Online) ISSN: 2572-9241
©2026 The Author(s). HemaSphere is published by John Wiley & Sons Ltd on behalf of European Hematology Association. This is an open access Abstract Book distributed under the Attribution-NonCommercial-NoDerivs (CC BY-NC-ND), which allows third parties to download the articles and share them with others as long as they credit the author and the Abstract Book, but they cannot change the content in any way or use them commercially.
**Abstract Book Citations:** Authors, Title, HemaSphere, 2026;10:(S1):pages. The abstract book DOIs can be found on the "Author Information" page.
**Disclaimer:** Articles published in the journal HemaSphere exclusively reflect the opinions of the authors. The authors are responsible for all content in their abstracts including accuracy of the facts, statements, citing resources, etc.

**Results**

Prime editing corrected multiple inborn error of immunity (IEI)-associated loci with 8–52% intended edits (mean 22%) minimal indels (mean 1.8%), and approximately 30-fold higher edit-to-indel ratios compared to Cas9/HDR. In SPTCL patient T-cells (n=3), optimized editing corrected 18.7–35.8% (mean 29.8%) of *HAVCR2* alleles and restored TIM-3 surface expression. Genome-wide analyses revealed no detectable exonic off-target editing and no significant alterations in immunophenotype, transcriptional profile, or clonal diversity.

Drug-resistance variants conferred resistance to tacrolimus, methotrexate, or mycophenolate while preserving sensitivity to alternative agents, providing an intrinsic safety mechanism. *FKBP1A*-edited T-cells maintained proliferation and IFN-γ production under tacrolimus exposure. Multiplex editing of *HAVCR2* and *FKBP1A* enabled robust *in vivo* enrichment: corrected *HAVCR2* alleles increased from approximately 10% at infusion to 76.8% in tacrolimus-treated mice, with no enrichment in vehicle-treated controls. Conversely, sensitivity of *FKBP1A*-edited cells to cyclosporin *in vivo* was comparable to unedited cells, confirming bidirectional drug control.

Tacrolimus-resistant CD19 CAR and CMV-specific T-cells exhibited preserved effector function under immunosuppression, demonstrating broad applicability of the platform.

**Summary/Conclusion**

Multiplex prime editing enables precise correction of pathogenic T-cell variants and programmable resistance to widely used immunosuppressants without detectable off-target effects. This approach transforms standard immunosuppressants into clinically familiar tools for *in vivo* selection and control of therapeutic T-cells, supporting translation toward first-in-human clinical evaluation.

**Copyright Information:** (Online) ISSN: 2572-9241
©2026 The Author(s). HemaSphere is published by John Wiley & Sons Ltd on behalf of European Hematology Association. This is an open access Abstract Book distributed under the Attribution-NonCommercial-NoDerivs (CC BY-NC-ND), which allows third parties to download the articles and share them with others as long as they credit the author and the Abstract Book, but they cannot change the content in any way or use them commercially.
**Abstract Book Citations:** Authors, Title, HemaSphere, 2026;10:(S1):pages. The abstract book DOIs can be found on the "Author Information" page.
**Disclaimer:** Articles published in the journal HemaSphere exclusively reflect the opinions of the authors. The authors are responsible for all content in their abstracts including accuracy of the facts, statements, citing resources, etc.

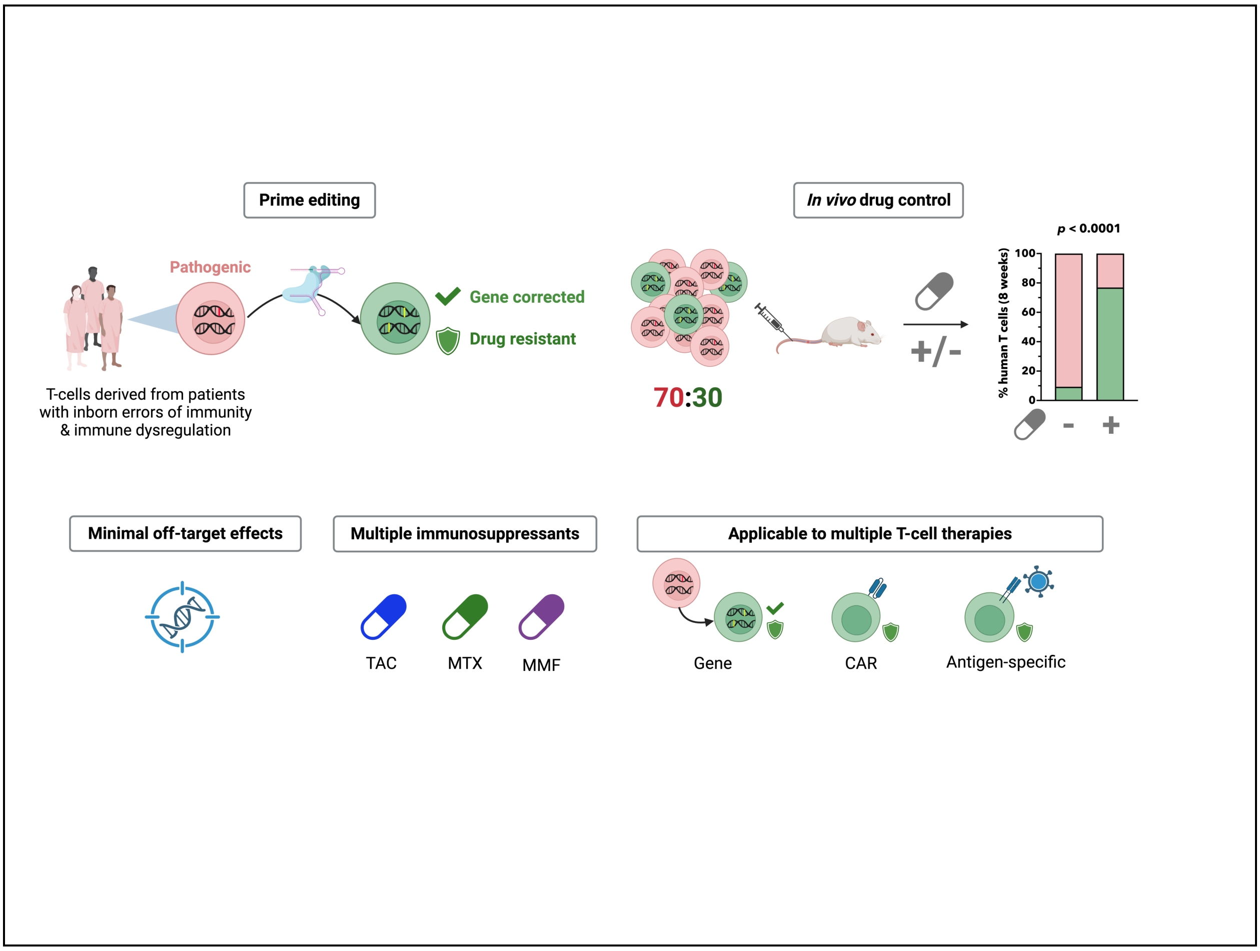

Copyright Information: (Online) ISSN: 2572-9241
©2026 The Author(s). HemaSphere is published by John Wiley & Sons Ltd on behalf of European Hematology Association. This is an open access Abstract Book distributed under the Attribution-NonCommercial-NoDerivs (CC BY-NC-ND), which allows third parties to download the articles and share them with others as long as they credit the author and the Abstract Book, but they cannot change the content in any way or use them commercially.
Abstract Book Citations: Authors, Title, HemaSphere, 2026;10:(S1):pages. The abstract book DOIs can be found on the "Author Information" page.
Disclaimer: Articles published in the journal HemaSphere exclusively reflect the opinions of the authors. The authors are responsible for all content in their abstracts including accuracy of the facts, statements, citing resources, etc.

### (S281) REWIRING TUMOR-NK CELL CROSSTALK IN HEMATOLOGICAL MALIGNANCIES THROUGH MULTIMODAL DRUG SCREENING

**Topic:** 25. Gene therapy, cellular immunotherapy and vaccination - Biology & translational research

Jonas Bouhlal*[1, 2, 3], Emmi Jokinen[1, 2, 3], Petra Nygren[1, 2, 3], Diogo Dias[1, 2, 3, 4], Aleksandr Ianevski[4], Jay Klievink[1, 2, 3], Hanna Lähteenmäki[1, 2, 3], Salome Decombis[1, 2, 3], Hanna Duàn[1, 2, 3], Emmi Järvelä[5], Emma Saarinen[4], Anna Näätänen[4], Konstantin Matjusinski[1, 2, 3], Tiina Kasanen[1, 2, 3], Tiina Hannunen[4], Laura Turunen[4], Mikko Myllymäki[1, 2, 3], Essi Laajala[1, 2, 3], Diana Schenkwein[6], Seppo Ylä-Herttuala[6, 7], Dean A. Lee[8], Matti Korhonen[5], Helka Göös[5], Tero Aittokallio[3, 4, 9, 10], Maija Hollmén[11], Constantine Mitsiades[12, 13, 14, 15], Sara Gandolfi[1, 2, 3], Olli Dufva[1, 2, 3, 16], Satu Mustjoki[1, 2, 3]

*[1]Hematology Research Unit Helsinki, University of Helsinki and Helsinki University Hospital, Helsinki, Finland;[2]Translational Immunology Research Program, University of Helsinki, Helsinki, Finland; [3]iCAN Digital Precision Cancer Medicine Flagship, Helsinki, Finland; [4]Institute for Molecular Medicine Finland, FIMM, University of Helsinki, HiLIFE, Helsinki, Finland;[5]Finnish Red Cross Blood Service, Helsinki, Finland; [6]A.I. Virtanen Institute for Molecular Sciences, University of Eastern Finland, Kuopio, Finland; [7]Gene Therapy Unit, Kuopio University Hospital, Kuopio, Finland; [8]Nationwide Children's Hospital, Division of Hematology, Oncology, and BMT, Columbus, OH, United States of America; [9]Institute for Cancer Research, Department of Cancer Genetics, Oslo University Hospital, Oslo, Norway; [10]Centre for Biostatistics and Epidemiology (OCBE), Faculty of Medicine, University of Oslo, Oslo, Norway; [11]MediCity Research Laboratory, Department of Microbiology and Immunology, University of Turku, Turku, Finland; [12]Department of Medical Oncology, Dana-Farber Cancer Institute, Harvard Medical School, Boston, MA, United States of America; [13]Broad Institute of MIT and Harvard, Cambridge MA, United States of America; [14]Department of Medicine, Harvard Medical School, Boston MA, United States of America; [15]Ludwig Center, Harvard Medical School, Boston MA, United States of America; [16]Wellcome Sanger Institute, Hinxton, Cambridge, United Kingdom;*

### Background

Natural Killer (NK) cells are key mediators of innate immune surveillance and promising effectors in cancer immunotherapy, particularly in hematological malignancies. Despite their potential as an off-the-shelf therapy, clinical responses remain variable due to tumor resistance and functional limitations of NK cells. NK cell cytotoxicity is shaped by NK cell signaling networks and tumor-intrinsic programs, both of which can potentially be targeted pharmacologically. However, systematic pharmacologic strategies to enhance NK cell activity and overcome tumor resistance remain lacking.

### Aims

In this study, we aimed to improve NK cell-based immunotherapies in hematological malignancies by systematically identifying druggable pathways that enhance NK cell function and sensitize cancer cells, and by defining mechanisms through which pharmacologic agents reprogram the cancer-NK cell interface.

### Methods

We performed high-throughput drug sensitivity and resistance testing (DSRT) using 527 oncology drugs in co-cultures of feeder-expanded donor NK cells and 10 hematological cancer cell lines (AML n = 5; CML n = 2; ALL/DLBCL/MM n = 1 each). Hit compounds were validated across three additional NK donors and in CD19-targeted CAR-NK cells, and further characterized by single-cell RNA sequencing (scRNA-seq) and cytokine profiling to dissect immune- and tumor-intrinsic responses. Genome-wide CRISPR-Cas9 screening was used to identify genetic dependencies in cancer cells underlying synergistic interactions. Top drug candidates were tested in primary AML and ALL samples to assess translational potential.

**Copyright Information:** (Online) ISSN: 2572-9241
©2026 The Author(s). HemaSphere is published by John Wiley & Sons Ltd on behalf of European Hematology Association. This is an open access Abstract Book distributed under the Attribution-NonCommercial-NoDerivs (CC BY-NC-ND), which allows third parties to download the articles and share them with others as long as they credit the author and the Abstract Book, but they cannot change the content in any way or use them commercially.
**Abstract Book Citations:** Authors, Title, HemaSphere, 2026;10:(S1):pages. The abstract book DOIs can be found on the "Author Information" page.
**Disclaimer:** Articles published in the journal HemaSphere exclusively reflect the opinions of the authors. The authors are responsible for all content in their abstracts including accuracy of the facts, statements, citing resources, etc.

### Results

Overall, our approach robustly identified compounds that enhanced or suppressed NK cell cytotoxicity across hematological malignancies *in vitro*. To systematically dissect the underlying mechanisms, we constructed a single-cell perturbation atlas comprising 240 drug-treated conditions across NK cells, tumor cells, and their co-cultures, paired with extensive cytokine profiling (> 5000 measurements). Integrative analysis of this dataset identified protein kinase C (PKC) activation using bryostatin 1 as a key mechanism, simultaneously increasing NK cell effector functions and tumor susceptibility to NK cell killing. In NK cells, PKC activation induced transcriptional programs associated with cytoskeletal remodeling and immune synapse formation, resulting in strengthened degranulation and release of cytokines such as IFN-γ and TNF-α. On the other hand, cancer cells were influenced by PKC activation by upregulation of NK-activating ligands and interferon- and TNF-associated signaling pathways, promoting immune recognition and sensitivity. Genome-wide CRISPR screening in THP-1 cells identified PKC-δ as a critical mediator of tumor-intrinsic responses to NK-bryostatin 1 combination. Importantly, PKC activation strengthened responses and overcame resistance to NK cells in primary AML and ALL samples, and potentiated CD19 CAR-NK cell activity, highlighting its translational relevance.

### Summary/Conclusion

In conclusion, our study establishes a systematic multimodal framework to reveal how oncology drugs reprogram the cancer-NK cell interface. Among others, we identify PKC signaling as an actionable pathway that simultaneously enhances NK cell effector function and increases tumor susceptibility to immune-mediated killing. These findings provide a rationale for combining PKC activation with NK cell-based therapies and offer a strategy to overcome resistance to NK cells in hematological malignancies.

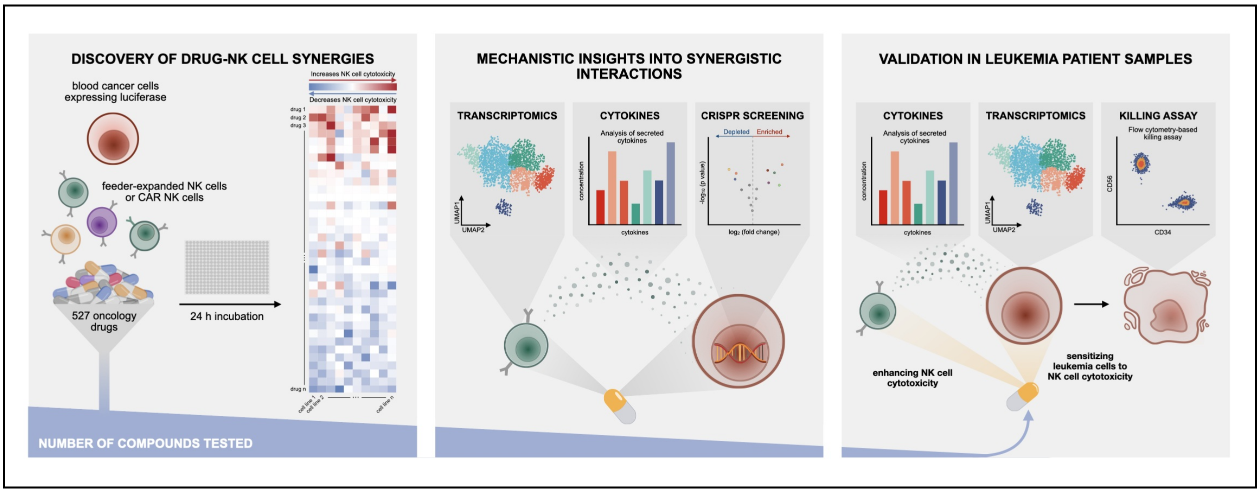

Overview of the workflow to identify NK cytotoxicity-modulating compounds and for deciphering underlying biological mechanisms.

**Copyright Information:** (Online) ISSN: 2572-9241
©2026 The Author(s). HemaSphere is published by John Wiley & Sons Ltd on behalf of European Hematology Association. This is an open access Abstract Book distributed under the Attribution-NonCommercial-NoDerivs (CC BY-NC-ND), which allows third parties to download the articles and share them with others as long as they credit the author and the Abstract Book, but they cannot change the content in any way or use them commercially.
**Abstract Book Citations:** Authors, Title, HemaSphere, 2026;10:(S1):pages. The abstract book DOIs can be found on the "Author Information" page.
**Disclaimer:** Articles published in the journal HemaSphere exclusively reflect the opinions of the authors. The authors are responsible for all content in their abstracts including accuracy of the facts, statements, citing resources, etc.

### (S282) SENESCENCE AND INFLAMMATION ARE UNINTENDED ADVERSE CONSEQUENCES OF CRISPR-CAS9/AAV6-MEDIATED GENE EDITING IN HEMATOPOIETIC STEM CELLS

**Topic:** 25. Gene therapy, cellular immunotherapy and vaccination - Biology & translational research

Anastasia Conti*[1], Kety Giannetti[1], Federico Midena[1, 2], Stefano Beretta[1], Nicolò Gualandi[1], Rosaria De Marco[1, 2], Edoardo Carsana[1], Angelica Varesi[3], Teresa Tavella[1], Laura Alessandrini[1, 2], Parinaz Zarghamian[4], Alessandra Weber[1], Samuele Ferrari[1], Chiara Brombin[5], Diego Gilioli[1, 2], Lucrezia Della Volpe[1, 2], Stephanie Xie[3], Ivan Merelli[1, 6], Toni Cathomen[4], Luigi Naldini[1, 2], Raffaella Di Micco[1]

*[1]San Raffaele Telethon Institute for Gene Therapy, IRCCS San Raffaele Scientific Institute, Milan, Italy;[2]Vita-Salute San Raffaele University, Milan, Italy; [3]Princess Margaret Cancer Centre, University Health Network, Toronto, Canada;[4]Institute for Transfusion Medicine and Gene Therapy, Medical Center – University of Freiburg, Freiburg, Germany; [5]University Center for Statistics in the Biomedical Sciences, Vita-Salute San Raffaele University, Milan, Italy; [6]National Research Council, Institute for Biomedical Technologies, Segrate, Italy;*

### Background

Gene editing (GE) using homology-directed repair (HDR) in hematopoietic stem and progenitor cells (HSPCs) holds promise for correcting mutations in inherited genetic disorders. However, CRISPR-Cas9/AAV6 engineering triggers cellular responses that impair the long-term repopulating potential of HDR-edited HSPCs, hindering clinical translation. We previously showed that an acute p53-mediated DNA damage response (DDR) limits HSPC proliferation and yield post-GE. Protracted DDR signaling promotes cellular senescence—wherein viable cells cease proliferating and activate inflammatory programs. Yet, whether GE induces durable, long-term functional deficits in HSPCs remains unclear.

### Aims

The overarching goals of this project are to uncover the long-term impacts of CRISPR/Cas9 GE on HSPCs and to develop strategies counteracting edited HSPC dysfunction, enabling more efficient gene and cell therapies.

### Methods

GE was performed in human cord blood (CB)- and mobilized peripheral blood (mPB)-derived HSPCs using AAV6 with an HDR donor template carrying GFP or dNGFR reporter genes for edited cell detection. To assess long-term repopulating potential, cells were transplanted into sublethally irradiated NSG mice, with human engraftment monitored in peripheral blood over time and in hematopoietic organs at endpoint via flow cytometry. Senescence was evaluated post-GE by flow cytometry for senescence markers and RNA-seq for inflammatory/senescence-associated gene signatures. Clonal tracking of individual HDR-edited HSPCs used our barcoding strategy, while genotoxicity was assessed via ultra-deep whole-exome sequencing (WES), micronuclei assays, large deletion detection, and CAST-seq for translocations.

**Copyright Information:** (Online) ISSN: 2572-9241
©2026 The Author(s). HemaSphere is published by John Wiley & Sons Ltd on behalf of European Hematology Association. This is an open access Abstract Book distributed under the Attribution-NonCommercial-NoDerivs (CC BY-NC-ND), which allows third parties to download the articles and share them with others as long as they credit the author and the Abstract Book, but they cannot change the content in any way or use them commercially.
**Abstract Book Citations:** Authors, Title, HemaSphere, 2026;10:(S1):pages. The abstract book DOIs can be found on the "Author Information" page.
**Disclaimer:** Articles published in the journal HemaSphere exclusively reflect the opinions of the authors. The authors are responsible for all content in their abstracts including accuracy of the facts, statements, citing resources, etc.

**Results**

Our study uncovers a durable senescence-like response in genetically engineered HSPCs triggered by p53 and interleukin (IL)-1/nuclear factor κB (NF-κB) activation, which restricts graft size and clonal diversity in long-term transplantation assays. We show that transient p53 inhibition or blocking inflammatory pathways mitigates senescence-associated responses, improving the repopulating capacity of edited HSPCs. Importantly, we identify treatment with Anakinra, an IL-1 signaling antagonist, as a promising strategy to enhance polyclonal output in HDR-edited cells while minimizing genotoxicity risks associated with the editing procedure.

**Summary/Conclusion**

Our study uncovers a durable senescence-like response in genetically-engineered HSPCs triggered by p53 and IL-1/NF-κB activation, which restricts graft size and clonal diversity in long-term transplantation assays. Overall, our findings present strategies to overcome key hurdles in HDR-based HSPC gene therapies, providing a framework for enhancing their efficacy and safety in clinical applications.

**Copyright Information:** (Online) ISSN: 2572-9241
©2026 The Author(s). HemaSphere is published by John Wiley & Sons Ltd on behalf of European Hematology Association. This is an open access Abstract Book distributed under the Attribution-NonCommercial-NoDerivs (CC BY-NC-ND), which allows third parties to download the articles and share them with others as long as they credit the author and the Abstract Book, but they cannot change the content in any way or use them commercially.
**Abstract Book Citations:** Authors, Title, HemaSphere, 2026;10:(S1):pages. The abstract book DOIs can be found on the "Author Information" page.
**Disclaimer:** Articles published in the journal HemaSphere exclusively reflect the opinions of the authors. The authors are responsible for all content in their abstracts including accuracy of the facts, statements, citing resources, etc.

### (S283) INTEGRATED MULTI-OMIC PROFILING IDENTIFIES GENOMIC SUBTYPES ASSOCIATED WITH DIFFERENTIAL OUTCOMES AFTER CAR-T THERAPY IN LARGE B-CELL LYMPHOMA

**Topic:** 25. Gene therapy, cellular immunotherapy and vaccination - Biology & translational research

Silvia Escribano Serrat*[1, 2, 3], Sandeep S. Raj[1, 2], Aaron Gillmor[1, 2], Sigrun Einarsdottir[1, 2], Marina Gomez-Llobell[1, 2, 4], Evgenia Alekseeva[5], Nazar Aryutyunian[5], Alexander Nesmelov[5], Andrey Suponin[5], Pavel Zemskiy[5], Dmitrii Snitkin[5], Gunjan Shah[1, 2, 6], M. Lia Palomba[2, 6, 7], Connie Lee Batlevi[7], Craig Sauter[1, 2, 6], Miguel-Angel Perales[1, 2, 6], Marcel van den Brink[8], Gilles Salles[6, 7], Roni Shouval[1, 2, 6]

*[1]Memorial Sloan Kettering Cancer Center, Adult Bone Marrow Transplant Service, Department of Medicine, New York, United States of America; [2]Memorial Sloan Kettering Cancer Center, Cellular Therapy Service, Department of Medicine, New York, United States of America; [3]Universitat Autònoma de Barcelona, Department of Medicine, Barcelona, Spain;[4]Complutense University of Madrid, Department of Medicine, Madrid, Spain; [5]BostonGene Corporation, Waltham, United States of America; [6]Weill Cornell Medical College, Department of Medicine, New York, United States of America;[7]Memorial Sloan Kettering Cancer Center, Lymphoma Service, Department of Medicine, New york, United States of America; [8]City of Hope National Medical Center, Hematologic Malignancies Program, Duarte, United States of America;*

### Background

Outcomes after CD19-directed CAR-T cell therapy in large B-cell lymphoma (LBCL) remain heterogeneous and are not fully explained by established clinical prognostic models. Tumor genomic architecture and microenvironmental states may influence CAR-T efficacy, but their integrated impact in real-world cohorts remains incompletely defined. We performed a multi-omic analysis to identify biological determinants of clinical variability after CAR-T therapy.

### Aims

To define tumor-intrinsic genomic and transcriptomic features associated with resistance to CAR-T cell therapy in LBCL.

### Methods

We conducted a retrospective cohort study of patients with LBCL treated with commercial CAR-T therapy between 2016 and 2022. Pre-CAR-T tumor specimens underwent whole-exome sequencing (WES) and RNA sequencing (RNA-seq), followed by integrated genomic and transcriptomic analyses using a standardized multi-omic computational pipeline (BostonGene, Waltham, MA). Genomic subtypes were assigned using the established LymphGen framework ( *Wright et al., Cancer cell, 2020*) and Lymphly, a hierarchical genomic classification algorithm developed to improve molecular stratification of LBCL. The tumor microenvironment (TME), and double hit signature (DHIT) were classified according to *Kotlov et al.* (*Cancer Discovery, 2021*), dark zone (DZsig) signature in accordance with *Harris et al.* (*Leukemia Lymphoma*, 2025). Associations between molecular features and progression-free survival (PFS), 6-month PFS (PFS6), and complete response (CR) were evaluated. Subgroups with fewer than 10 cases were excluded from statistical comparisons but retained for visualization.

**Copyright Information:** (Online) ISSN: 2572-9241
©2026 The Author(s). HemaSphere is published by John Wiley & Sons Ltd on behalf of European Hematology Association. This is an open access Abstract Book distributed under the Attribution-NonCommercial-NoDerivs (CC BY-NC-ND), which allows third parties to download the articles and share them with others as long as they credit the author and the Abstract Book, but they cannot change the content in any way or use them commercially.
**Abstract Book Citations:** Authors, Title, HemaSphere, 2026;10:(S1):pages. The abstract book DOIs can be found on the “Author Information” page.
**Disclaimer:** Articles published in the journal HemaSphere exclusively reflect the opinions of the authors. The authors are responsible for all content in their abstracts including accuracy of the facts, statements, citing resources, etc.

**Results**

Among 112 patients with available molecular data, median follow-up was 66 months (IQR 46-73). CAR-T products included axicabtagene ciloleucel (49%), tisagenlecleucel (38%), and lisocabtagene maraleucel (13%). Fifty-four percent of tumors were germinal center B-cell type, 46% activated B-cell type, and 17% were DHIT-positive.
WES data were available in 99 patients. BostonGene's Lymphly classifier assigned genomic subtypes to a larger proportion of evaluable cases compared with LymphGen and demonstrated improved outcome discrimination (**Fig. 1A**). Inferior outcomes were primarily driven by the BN2 subtype, which was associated with worse PFS (16%, 95% CI 6-46, p=0.017), lower PFS6 (p=0.009), and reduced CR rates (p=0.041) compared with other evaluable subgroups (**Fig. 1B**). In multivariable Cox models adjusting for relevant clinical covariates, BN2 remained independently associated with inferior PFS and PFS6 (both p<0.001).
RNA-seq data were available for 55 patients. RNA-defined TME subtypes and DHIT status were not significantly associated with PFS in univariable analyses. The inflammatory TME subtype demonstrated numerically inferior outcomes, with a 1-year PFS of 19% (95% CI 7-52) compared with 36% and 41% in the mesenchymal and depleted subtypes, respectively, whereas DHIT-positive cases showed numerically favorable 1-year PFS (46% vs 31%) (**Fig. 1C**). Within the GCB subgroup, the Dzsig showed similar PFS between DZsig-positive and DZsig-negative cases (**Fig 1D**); formal statistical testing was not performed due to limited sample size.

**Summary/Conclusion**

Multi-omic profiling identifies biologically defined high-risk subgroups after CAR-T therapy, highlighting BN2 as a genomically driven resistance phenotype. Genomic classification may refine patient selection and enable mechanism-based therapeutic strategies in LBCL.

**Copyright Information:** (Online) ISSN: 2572-9241
©2026 The Author(s). HemaSphere is published by John Wiley & Sons Ltd on behalf of European Hematology Association. This is an open access Abstract Book distributed under the Attribution-NonCommercial-NoDerivs (CC BY-NC-ND), which allows third parties to download the articles and share them with others as long as they credit the author and the Abstract Book, but they cannot change the content in any way or use them commercially.
**Abstract Book Citations:** Authors, Title, HemaSphere, 2026;10:(S1):pages. The abstract book DOIs can be found on the "Author Information" page.
**Disclaimer:** Articles published in the journal HemaSphere exclusively reflect the opinions of the authors. The authors are responsible for all content in their abstracts including accuracy of the facts, statements, citing resources, etc.

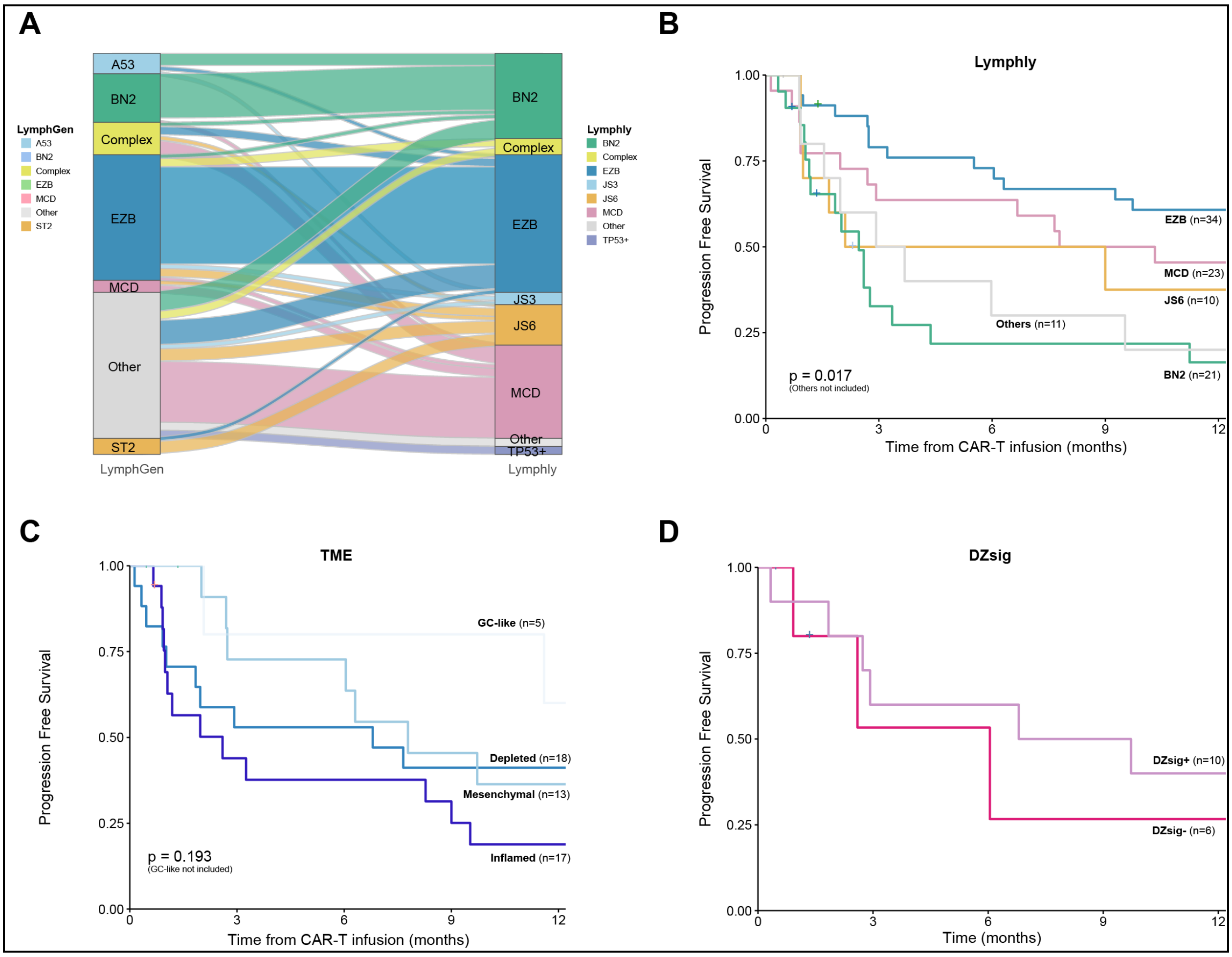

**Copyright Information:** (Online) ISSN: 2572-9241
©2026 The Author(s). HemaSphere is published by John Wiley & Sons Ltd on behalf of European Hematology Association. This is an open access Abstract Book distributed under the Attribution-NonCommercial-NoDerivs (CC BY-NC-ND), which allows third parties to download the articles and share them with others as long as they credit the author and the Abstract Book, but they cannot change the content in any way or use them commercially.
**Abstract Book Citations:** Authors, Title, HemaSphere, 2026;10:(S1):pages. The abstract book DOIs can be found on the "Author Information" page.
**Disclaimer:** Articles published in the journal HemaSphere exclusively reflect the opinions of the authors. The authors are responsible for all content in their abstracts including accuracy of the facts, statements, citing resources, etc.

### (S284) BRANCHED-CHAIN AMINO ACID BIOAVAILABILITY DURING CD19 CAR T-CELL EXPANSION ASSOCIATES WITH FAVORABLE T-CELL STATES AND CLINICAL RESPONSE IN LBCL

**Topic:** 25. Gene therapy, cellular immunotherapy and vaccination - Biology & translational research

Sylvain Lamure*[1, 2], Emilie Agostinho[2], Sthéphanie Badiou[3], Caroline Mollevi[3], Mohammad Salma[2], Axelle Nkodia[4], Roch Houot[5], Emmanuel Bachy[6], Pierre Bories[7], Cristina Castilla Llorente[8], Bertrand Nadel[9], Jean-Paul Cristol[3], Christine Ménétrier-Caux[1], Cyril Degletagne[1], Eric Soler[2], Naomi Taylor[10], Valérie Zimmermann[2], Valerie Dardalhon[2], Guillaume Cartron[2, 3]

*[1]Centre Léon Bérard, Hématologie, Lyon, France; [2]Institut de Génétique Moléculaire de Montpellier, Montpellier, France;[3]CHU de Montpellier, Montpellier, France; [4]Centre de Recherche en Cancérologie de Lyon, Lyon, France;[5]CHU de Rennes, Rennes, France; [6]Hospices Civiles de Lyon, Lyon, France; [7]IUCT Oncopole, Toulouse, France; [8]Institut Gustave Roussy, Villejuif, France; [9]CALYM, Paris, France; [10]National Cancer Institute, Bethesda, United States of America;*

### Background

The metabolic environment critically regulates T-cell activation, differentiation, and effector function. Distinct T-cell subsets exhibit specific metabolic programs: effector T cells are predominantly glycolytic or even lipogenic, whereas regulatory T-cells ($T_{REG}$) rely more heavily on oxidative and lipid metabolic pathways. Nutrient deprivation impairs effector function and promotes dysfunctional states. The host metabolic milieu may therefore represent an underappreciated determinant of CAR T-cell expansion and anti-tumor efficacy.

### Aims

We sought to determine whether dynamic changes in plasma nutrient availability following CD19 CAR T-cell infusion associate with T-cell subsets and clinical outcomes in patients with large B-cell lymphoma (LBCL).

### Methods

Plasma and cryopreserved peripheral blood mononuclear cells were collected at baseline (D0) and day 7 (D7) following infusion of axicabtagene ciloleucel (axi-cel) or tisagenlecleucel in LBCL patients from the CeVi_Collection. Glucose, lactate, and insulin were quantified on the Roche Cobas 8000 platform. Amino acid concentrations were measured by ultra-performance liquid chromatography-tandem mass spectrometry (Waters Xevo-TQD), and plasma fatty acid composition was assessed by gas liquid chromatography with flame-ionization detection. Unsupervised hierarchical clustering of D0-D7 variations was used to define metabolic profiles. Single cell RNA-Seq was performed on sorted D7 CAR+ and CAR- T-cells (10x Genomics) to characterize T cell subsets and cellular states.

**Copyright Information:** (Online) ISSN: 2572-9241
©2026 The Author(s). HemaSphere is published by John Wiley & Sons Ltd on behalf of European Hematology Association. This is an open access Abstract Book distributed under the Attribution-NonCommercial-NoDerivs (CC BY-NC-ND), which allows third parties to download the articles and share them with others as long as they credit the author and the Abstract Book, but they cannot change the content in any way or use them commercially.
**Abstract Book Citations:** Authors, Title, HemaSphere, 2026;10:(S1):pages. The abstract book DOIs can be found on the "Author Information" page.
**Disclaimer:** Articles published in the journal HemaSphere exclusively reflect the opinions of the authors. The authors are responsible for all content in their abstracts including accuracy of the facts, statements, citing resources, etc.

**Results**

Sixty-six patients were analyzed (70% Axi-cel). Low baseline nutrients levels were associated with higher metabolic tumor volume (p=0.03). Clustering based on D0-D7 nutrient dynamics identified three major categories (Figure A):
Profile A (n=20): global increases in amino acids, glucose, insulin and lactate
Profile B (n=8): increased fractioned fatty acids with decreased amino acids
Profile C (n=38): generalized nutrient decline
Profiles were independent of tumor subtype, CAR T-product and nutritional assessment. Patients with profile A and B exhibit higher level of CD4 and CD8 CAR T-cells compared to patients with profile C (p=0.04 and 0.02).
Among all metabolites, increases in the branched-chain amino acids valine and isoleucine were significantly associated with complete response and prolonged survival (Figure B).
Single-cell RNA sequencing was performed in 14 patients. Five patients with profile A (characterized by increased valine and isoleucine) exhibited balanced proportions of $CAR^-$ and $CAR^+$ CD4 central memory ($CD4_{CM}$) and CD8 effector memory ($CD8_{EM}$) cells, with a predominance of activated CD4 T-cells. In contrast, 4 with profile B (characterized for increased saturated fatty acids), and 5 with profile C (characterized by decreased valine, isoleucine and saturated fatty acids) showed reduced $CD4_{CM}$ frequency and enrichment of $T_{REG}$ (CAR- and CAR+), along with fewer activated CD4 T-cells; profile C additionally exhibited increased activated CD8 T-cells (Figure C and D).

**Summary/Conclusion**

Dynamic changes in plasma nutrient availability following CD19 CAR T-cell therapy define distinct metabolic trajectories that associate with T-cell differentiation states and clinical outcome in LBCL. In particular, increased branched-chain amino acid bioavailability correlates with favorable CAR T-cell phenotypes and therapeutic response. These findings support the development of metabolic or nutritional interventions to optimize CAR T-cell expansion and anti-tumor efficacy.

**Copyright Information:** (Online) ISSN: 2572-9241
©2026 The Author(s). HemaSphere is published by John Wiley & Sons Ltd on behalf of European Hematology Association. This is an open access Abstract Book distributed under the Attribution-NonCommercial-NoDerivs (CC BY-NC-ND), which allows third parties to download the articles and share them with others as long as they credit the author and the Abstract Book, but they cannot change the content in any way or use them commercially.
**Abstract Book Citations:** Authors, Title, HemaSphere, 2026;10:(S1):pages. The abstract book DOIs can be found on the "Author Information" page.
**Disclaimer:** Articles published in the journal HemaSphere exclusively reflect the opinions of the authors. The authors are responsible for all content in their abstracts including accuracy of the facts, statements, citing resources, etc.

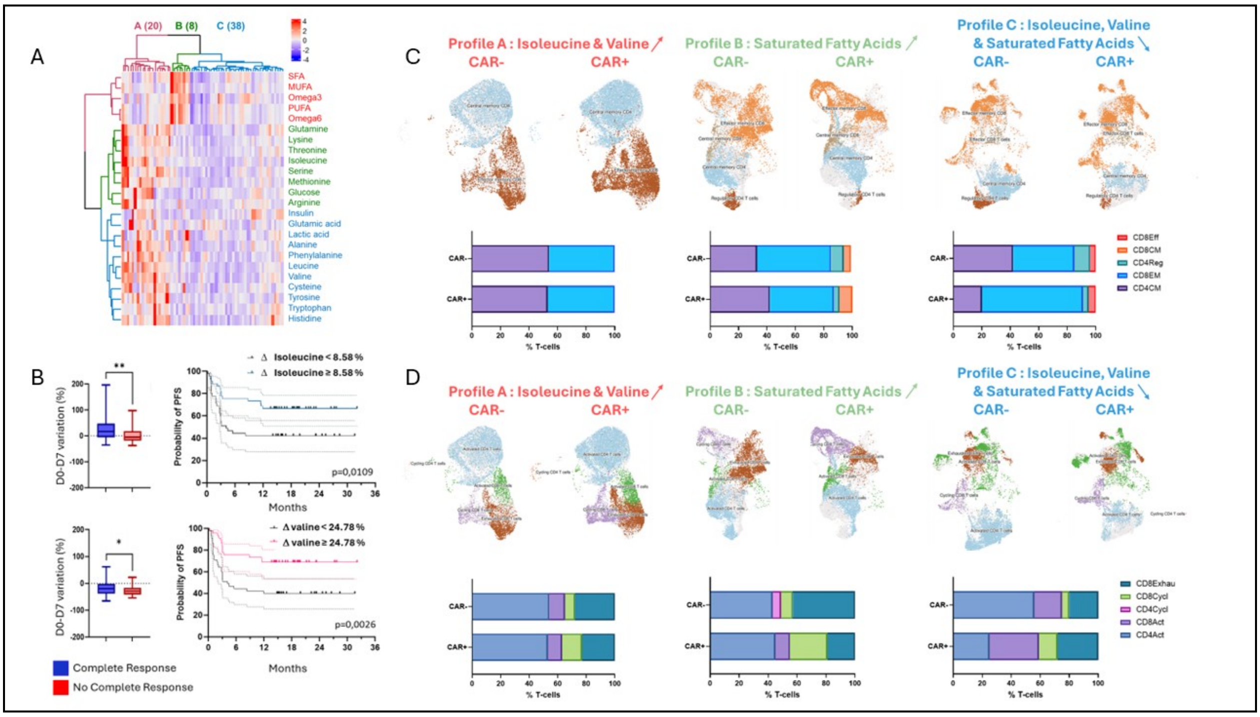

Figure: Metabolic trajectories during CD19 CAR T-cell therapy associate with T-cell phenotypes and clinical outcome in large B-cell lymphoma. (A) Twenty-four plasma nutrients were quantified at D0 and D7 in 66 patients; unsupervised clustering of D0-D7 variation defined three metabolic profiles. (B) Patients with increased valine and isoleucine levels were compared to others for response rate and progression-free survival. (C) Single-cell RNA sequencing of D7 CAR- and CAR+ T-cells from representative patients across group A, B and C were used to quantify T-cell subsets and activation/exhaustion states. Statistical significance was assessed Wilcoxon-Mann-Whitney and log-rank tests (*p<0.05, **p<0.01).

**Copyright Information:** (Online) ISSN: 2572-9241
©2026 The Author(s). HemaSphere is published by John Wiley & Sons Ltd on behalf of European Hematology Association. This is an open access Abstract Book distributed under the Attribution-NonCommercial-NoDerivs (CC BY-NC-ND), which allows third parties to download the articles and share them with others as long as they credit the author and the Abstract Book, but they cannot change the content in any way or use them commercially.
**Abstract Book Citations:** Authors, Title, HemaSphere, 2026;10:(S1):pages. The abstract book DOIs can be found on the "Author Information" page.
**Disclaimer:** Articles published in the journal HemaSphere exclusively reflect the opinions of the authors. The authors are responsible for all content in their abstracts including accuracy of the facts, statements, citing resources, etc.

### (S285) OL-101, A BCMA/GPRC5D DUAL-TARGETING CAR-T, INDUCES DEEP RESPONSES IN HEAVILY PRETREATED ADVANCED MYELOMA, PARTICULARLY IN PATIENTS POST BCMA- OR GPRC5D-TARGETED THERAPY OR WITH EXTRAMEDULLARY DISEASE

**Topic:** 26. Gene therapy, cellular immunotherapy and vaccination - Clinical

Mingming Zhang*[1], Yongxian Hu[1], Yajing Zhang[2], Chengcheng Fu[3], Shan Fu[1], Jingjing Feng[1], Guoai Su[2], Jingjing Shang[3], Dan Song[4], Hongmei Wang[4], Kaiyi Fan[4], Weimin Li[4], He Huang[1]

*[1]Bone Marrow Transplantation Center, the First Affiliated Hospital, and Liangzhu Laboratory, Zhejiang University School of Medicine, Hangzhou, China; [2]Innovative Medical Center for Oncology & Immunology, Beijing GoBroad Boren Hospital, Beijing, China; [3]Hematology Department and BMT center, First Affiliated Hospital of Soochow University, Suzhou, China; [4]Overland Biotherapeutics (SH) Co. Ltd, Shanghai, China;*

### Background

Patients with R/R MM who have progressed after BCMA- or GPRC5D-directed therapies represent an increasing population with profound therapeutic resistance and limited subsequent options. Prognosis is particularly poor in patients with EMD, where responses to existing CAR-T therapies are often suboptimal. OL-101 is an autologous CAR-T incorporating VHH binders targeting both BCMA and GPRC5D, designed to mitigate either antigen escape or deletion, and enhance depth and durability of response in high-risk, heavily pretreated R/R MM, especially those post BCMA- or GPRC5D-targeted therapy with or without EMD. Here we report preliminary results from an ongoing first-in-human phase I study (NCT06644118).

### Aims

To evaluate the safety, efficacy, and cellular kinetics of OL-101 and to define the Recommended Phase II Dose (RP2D).

### Methods

Patients with R/R MM after ≥3 prior lines of therapy (including a proteasome inhibitor, immunomodulatory agent, and anti-CD38 antibody) were enrolled. Following lymphodepletion, patients received a single infusion of OL-101 at 1 of 3 dose levels (DL1: $1.0\times10^6$, DL2: $2.0\times10^6$, DL3: $1.5\times10^6$ CAR-T cells/kg) in a modified 3+3 escalation design. DL3 was evaluated after a dose-limiting toxicity observed at DL2.

**Copyright Information:** (Online) ISSN: 2572-9241
©2026 The Author(s). HemaSphere is published by John Wiley & Sons Ltd on behalf of European Hematology Association. This is an open access Abstract Book distributed under the Attribution-NonCommercial-NoDerivs (CC BY-NC-ND), which allows third parties to download the articles and share them with others as long as they credit the author and the Abstract Book, but they cannot change the content in any way or use them commercially.
**Abstract Book Citations:** Authors, Title, HemaSphere, 2026;10:(S1):pages. The abstract book DOIs can be found on the "Author Information" page.
**Disclaimer:** Articles published in the journal HemaSphere exclusively reflect the opinions of the authors. The authors are responsible for all content in their abstracts including accuracy of the facts, statements, citing resources, etc.

**Results**

As of 06 Feb 2026, 15 patients were dosed (median follow-up 7.2 months). All patients were triple-class exposed with a median of 5 prior lines. High-risk features were common, including high-risk cytogenetics in 6 patients (40.0%), EMD in 7 patients (46.7%) and prior exposure to BCMA- and/or GPRC5D-directed therapies in 8 patients (53.3%).
All patients were evaluable for safety. CRS occurred in all cases (Grade 1-2: 73.3%, Grade ≥3: 26.7%, no Grade 5). One patient experienced Grade 1 ICANS (ICE score of 7 for 1 day). Other common Grade ≥3 Treatment-Related Adverse Events (TRAEs) were primarily hematological toxicities, including Grade 4 neutropenia in 66.7% and Grade 4 thrombocytopenia in 53.3% of patients.
Among 13 efficacy-evaluable patients, the overall response rate (ORR) was 100%, with 11 patients (84.6%) achieving very good partial response (VGPR) or better, including 9 (69.2%) stringent complete response (sCR). Notably, deep responses were observed in difficult-to-treat subgroups. Among patients with EMD, 5/6 (83.3%) achieved VGPR or better, including 4/6 (66.7%) with sCR. Similarly, among patients previously treated with BCMA- and/or GPRC5D-directed therapies, 4/6 (66.7%) achieved VGPR or better, including 3/6 (50.0%) with sCR. Median time to first response was 28 days. Robust CAR-T expansion was observed across all dose levels, with peak expansion between Days 10-21 post-infusion. All patients achieved minimal residual disease (MRD) negativity (at $10^{-5}$ sensitivity by next-generation flow). Baseline expression of BCMA and GPRC5D was confirmed in all patients and was not associated with depth of response. Targeted sequencing of BCMA (TNFRSF17) and GPRC5D genes in pre- and post-infusion bone marrow samples did not identify pathogenic variants.

**Summary/Conclusion**

OL-101 demonstrates a manageable safety profile and highly compelling preliminary efficacy in heavily pretreated, high-risk R/R MM populations. The achievement of universal, deep MRD-negative responses in patients with prior BCMA- or GPRC5D-directed therapy and in those with EMD supports dual-targeting CAR-T strategies to overcome antigen escape and therapeutic resistance.

**Copyright Information:** (Online) ISSN: 2572-9241
©2026 The Author(s). HemaSphere is published by John Wiley & Sons Ltd on behalf of European Hematology Association. This is an open access Abstract Book distributed under the Attribution-NonCommercial-NoDerivs (CC BY-NC-ND), which allows third parties to download the articles and share them with others as long as they credit the author and the Abstract Book, but they cannot change the content in any way or use them commercially.
**Abstract Book Citations:** Authors, Title, HemaSphere, 2026;10:(S1):pages. The abstract book DOIs can be found on the “Author Information” page.
**Disclaimer:** Articles published in the journal HemaSphere exclusively reflect the opinions of the authors. The authors are responsible for all content in their abstracts including accuracy of the facts, statements, citing resources, etc.

Figure. Waterfall plot of best SPD% change from baseline for 6 patients with EMD

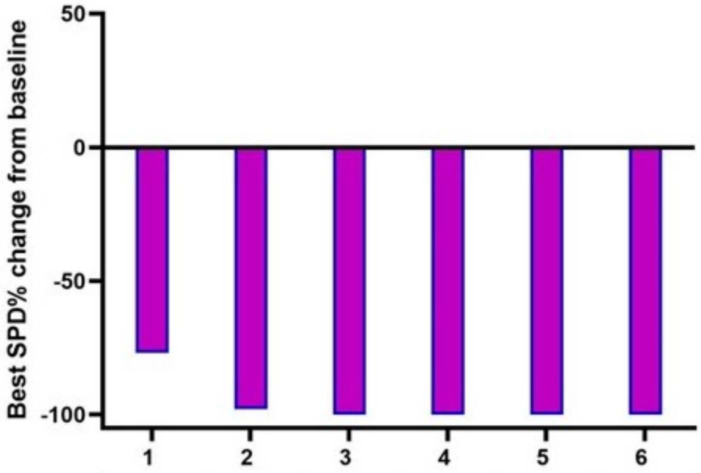

| | 1 | 2 | 3 | 4 | 5 | 6 |
|---|---|---|---|---|---|---|
| SPD at baseline ($mm^2$) | 1656 | 3122 | 1369 | 832 | 550 | 180 |
| Post BCMA- or GPRC5D-Targeted Therapy | Yes | Yes | No | Yes | Yes | No |

Note. Each bar represents an individual patient. SPD = sum of product of diameters.

Copyright Information: (Online) ISSN: 2572-9241
©2026 The Author(s). HemaSphere is published by John Wiley & Sons Ltd on behalf of European Hematology Association. This is an open access Abstract Book distributed under the Attribution-NonCommercial-NoDerivs (CC BY-NC-ND), which allows third parties to download the articles and share them with others as long as they credit the author and the Abstract Book, but they cannot change the content in any way or use them commercially.
Abstract Book Citations: Authors, Title, HemaSphere, 2026;10:(S1):pages. The abstract book DOIs can be found on the "Author Information" page.
Disclaimer: Articles published in the journal HemaSphere exclusively reflect the opinions of the authors. The authors are responsible for all content in their abstracts including accuracy of the facts, statements, citing resources, etc.

### (S286) CD19/CD22 BISPECIFIC CAR-T CELL THERAPY FOR RELAPSED/REFRACTORY LARGE B-CELL LYMPHOMA AND MECHANISTIC INVESTIGATION: A PROSPECTIVE, SINGLE-ARM, SINGLE-CENTER, PHASE 2 CLINICAL TRIAL

**Topic:** 26. Gene therapy, cellular immunotherapy and vaccination - Clinical

Xindi Liu[1], He-Nan Wang[1], Jia Cong[1], Liang Wang*[1]

*[1]Beijing Tongren Hospital, Capital Medical University, Beijing, China;*

### Background

Chimeric antigen receptor T-cells (CAR-T) targeting CD19 have shown promising benefits to patients of relapsed or refractory large B-cell lymphoma (R/R LBCL), but only one-third patients got durable remission and the resistance mechanisms were complicated. CAR-T cells targeting CD22 were found to be effective in patients who failed previous anti-CD19 CAR-T cell therapy.

### Aims

In this prospective study, we aimed to evaluate the efficacy and safety of CD19/CD22 bispecific CAR-T cell therapy (CAR2219) in patients with R/R LBCL, and to explore the underlying mechanisms associated with therapeutic response using single-cell sequencing and in vitro experiments.

### Methods

Till February 10, 2026, this ongoing phase 2 clinical trial have enrolled 73 patients with R/R LBCL, and all were evaluable for efficacy and safety profiles. Bridging therapy was allowed. A 3-day lymphodepletion (cyclophosphamide: 250 mg/m2/d; fludarabine: 25 mg/m2/d) was followed by an intravenous dose of CAR2219 (2×10e6/kg). For patients who responded to CAR2219 therapy, maintenance therapy using PD-1 inhibitors, XPO1 inhibitors, BTK inhibitors, or HDAC inhibitors were also allowed. The primary endpoint was best overall response rate (ORR) as of 3 months post-CAR-T cells infusion. The secondary endpoints included best complete response rate (CRR), progression-free survival (PFS), overall survival (OS), and adverse events (AE). Meanwhile, single-cell RNA sequencing was performed on CAR-T cell products collected from 14 patients; clustering and functional analyses were conducted after stratification by PFS with a cutoff of 6 months, and double-stranded RNA (dsRNA) levels in CAR-T cell products were detected by immunofluorescence.

### Results

All enrolled patients had undergone a median of three (range 3-8) prior lines of therapy. The median IPI score was 4 (range 1-5) at the time of CAR-T cell therapy. *TP53* mutation was positive in 22 of 24 patients. CAR2219 was successfully manufactured and infused to all 73 patients. With a median follow-up of 10.43 months after CAR-T cells infusion, the best ORR was 95.9%, including a CRR of 56.2%. The median PFS and OS were not reached. Estimated 24-month PFS and OS rates were 68.5% [95%CI: 57.2-79.8%] and 82.3% [73.5-91.1%], respectively. The most common AEs included neutropenia (93.2%), thrombocytopenia (76.7%), and anemia (76.7%). Cytokine release syndrome (CRS) occurred in 83.6% of patients, with 2 cases of grade 3. Immune effector cell-associated neurotoxicity syndrome (ICANS) occurred in 5.5% of patients, including 2 cases of grade 3 and 1 case of grade 4. Tocilizumab and corticosteroids were effective in managing CRS and ICANS, and no treatment-related deaths were reported. Single-cell RNA sequencing analysis revealed that the proportions of $CD4^+$ T cell subsets associated with memory and cytotoxicity were significantly higher in patients with longer PFS. Pathway scoring of these two subsets demonstrated marked activation of the DDX58 pathway and interferon pathways. In parallel, in vitro immunofluorescence assays showed significantly higher dsRNA expression in CAR-T cell products from patients with longer PFS.

**Copyright Information:** (Online) ISSN: 2572-9241
©2026 The Author(s). HemaSphere is published by John Wiley & Sons Ltd on behalf of European Hematology Association. This is an open access Abstract Book distributed under the Attribution-NonCommercial-NoDerivs (CC BY-NC-ND), which allows third parties to download the articles and share them with others as long as they credit the author and the Abstract Book, but they cannot change the content in any way or use them commercially.
**Abstract Book Citations:** Authors, Title, HemaSphere, 2026;10:(S1):pages. The abstract book DOIs can be found on the “Author Information” page.
**Disclaimer:** Articles published in the journal HemaSphere exclusively reflect the opinions of the authors. The authors are responsible for all content in their abstracts including accuracy of the facts, statements, citing resources, etc.

**Summary/Conclusion**

CD19/CD22 bispecific CAR-T cell therapy demonstrated encouraging efficacy and a favorable safety profile in patients with R/R LBCL. Enrichment of specific $CD4^{+}$ T cell subsets, activation of the interferon pathway, and activation of viral mimicry may be closely associated with the efficacy of this therapy. (ClinicalTrials.gov Identifier: NCT06081478)

**Copyright Information:** (Online) ISSN: 2572-9241
©2026 The Author(s). HemaSphere is published by John Wiley & Sons Ltd on behalf of European Hematology Association. This is an open access Abstract Book distributed under the Attribution-NonCommercial-NoDerivs (CC BY-NC-ND), which allows third parties to download the articles and share them with others as long as they credit the author and the Abstract Book, but they cannot change the content in any way or use them commercially.
**Abstract Book Citations:** Authors, Title, HemaSphere, 2026;10:(S1):pages. The abstract book DOIs can be found on the "Author Information" page.
**Disclaimer:** Articles published in the journal HemaSphere exclusively reflect the opinions of the authors. The authors are responsible for all content in their abstracts including accuracy of the facts, statements, citing resources, etc.

### (S287) DURABLE RESPONSES AND FAVORABLE SAFETY OF BAFF-R CAR T-CELLS (PMB-CT01) IN PATIENTS WITH RELAPSED/ REFRACTORY B-CELL LYMPHOMAS WITH PRIOR CD19-DIRECTED THERAPY FAILURE OR CD19-NEGATIVE DISEASE

**Topic:** 26. Gene therapy, cellular immunotherapy and vaccination - Clinical

L. Elizabeth Budde*[1], Marissa Del Real[1], Alan MacIas[1], Joo Song[1], Hazel (Ting-Ying) Cheng[2], Swetha Thiruvengadam[1], John Baird[1], Alex F. Herrera[1], Geoffrey Shouse[1], Nilanjan Ghosh[3], Marie Hu[4], Matthew Schwede[5], Matthew J. Frank[6], Forat Lutfi[7], Michael Wang[8], Steven T Rosen[1], Lu Chen[1], Jennifer Davis[1], Baishakhi Barva[1], Jamie Wagner[1], Zhenyuan Dong[1], Anmol Kandel[1], Xiuli Wang[1], Soung-Chul Cha[1], Brian Sworder[1], Deshaun Noakes[2], Sandrine Puverel[2], Stephen Forman[1], Larry Kwak[2]

*[1]City of Hope National Medical Center, Duarte, United States of America;[2]PeproMene Bio, Inc., Irvine, United States of America; [3]Atrium Health Levine Cancer Institute, Charlotte, United States of America;[4]University of Minnesota, Minneapolis, United States of America; [5]Swedish Cancer Institute, Seattle, United States of America; [6]Stanford Medicine, Stanford, United States of America; [7]University of Kansas Medical Center, Kansas City, United States of America;[8]MD Anderson Cancer Center, Houston, United States of America;*

### Background

CD19-directed CAR T-cell therapies have improved outcomes for patients with relapsed/refractory (r/r) B-cell non-Hodgkin lymphoma (B-NHL), yet relapse remains frequent and is commonly associated with loss or downregulation of CD19. Patients who relapse after or are ineligible for CD19 CAR T-cell therapy have limited therapeutic options and poor outcomes. The B-cell activating factor receptor (BAFF-R) is a key regulator of B-cell function that is broadly expressed across B-cell lymphoma subtypes and retained in CD19-negative disease. Because BAFF-R is required for B-cell survival, targeting this receptor may reduce the likelihood of therapeutic escape through antigen downregulation compared with CD19 or CD20. These properties make BAFF-R a compelling alternative CAR T-cell target. PMB-CT01 is a novel autologous BAFF-R-targeted CAR T-cell therapy currently being evaluated in a phase 1 study in patients with B-cell lymphomas.

### Aims

To assess the safety and preliminary efficacy of PMB-CT01 and to establish the recommended phase 2 dose (RP2D) following completion of the dose-escalation portion of the study.

### Methods

This multicenter phase 1 study (NCT05370430) enrolled adult patients with r/r B-NHL. Following standard lymphodepletion, patients received a single infusion of BAFF-R CAR T-cells at either $50x10^6$ (dose level 1, DL1) or $200x10^6$ (dose level 2, DL2) CAR-positive T-cells. The primary objective was safety, including dose-limiting toxicities (DLTs) and determination of the RP2D. Secondary objectives included assessment of anti-tumor activity and survival outcomes. Cytokine release syndrome (CRS) and immune effector cell-associated neurotoxicity syndrome (ICANS) were graded according to ASTCT consensus criteria, and responses were evaluated per Lugano 2014 criteria.

**Copyright Information:** (Online) ISSN: 2572-9241
©2026 The Author(s). HemaSphere is published by John Wiley & Sons Ltd on behalf of European Hematology Association. This is an open access Abstract Book distributed under the Attribution-NonCommercial-NoDerivs (CC BY-NC-ND), which allows third parties to download the articles and share them with others as long as they credit the author and the Abstract Book, but they cannot change the content in any way or use them commercially.
**Abstract Book Citations:** Authors, Title, HemaSphere, 2026;10:(S1):pages. The abstract book DOIs can be found on the "Author Information" page.
**Disclaimer:** Articles published in the journal HemaSphere exclusively reflect the opinions of the authors. The authors are responsible for all content in their abstracts including accuracy of the facts, statements, citing resources, etc.

**Results**

Nine patients were treated between September 2022 and August 2025, completing the dose-escalation portion of the study, with 3 patients treated at DL1 and 6 patients treated at DL2. The median age was 62 years (range 41-75), and patients had received a median of 3 prior therapies (range, 1-10), including CD19 CAR T-cells (n=6) and a T-cell engager (n=3). Histologies included mantle cell lymphoma (MCL; n=6), large B-cell lymphoma (LBCL; n=1), follicular lymphoma (FL; n=1), and marginal zone lymphoma (n=1). No DLTs were observed at either dose level. CRS occurred in 7 patients (78%), all grade 1, with no grade ≥2 events. Two patients experienced grade 1 ICANS, both resolving without corticosteroids. Seven patients (78%) achieved complete responses, including patients with prior CD19 CAR T-cell therapy or with CD19/CD20-negative disease; two patients had stable disease. Robust CAR T-cell expansion was observed in all responders. All responding MCL patients (n=4) were MRD-negative. At a median follow-up of 22 months (range, 3-36), all patients were alive and all complete responses were ongoing without relapse. Based on these findings, DL2 ($200 \times 10^6$ CAR-positive cells) was selected as the RP2D.

**Summary/Conclusion**

Completion of the dose-escalation portion of this phase 1 trial demonstrated a favorable safety profile and durable complete responses with BAFF-R-targeted CAR T-cell therapy in patients with r/r B-NHL, including those with prior CD19 CAR T-cell failure or CD19-negative disease. These results supported the selection of the RP2D and initiation of disease-specific expansion cohorts in MCL, LBCL, and FL. Enrollment in these cohorts is ongoing, and additional clinical and correlative analyses are underway.

| | **Total (N=9)** |
|---|---|
| **Best Overall Response** | |
| Complete Response (CR) | 7 (78%) |
| Partial Response (PR) | 0 |
| Stable Disease (SD) | 2 (22%) |
| Progressive Disease (PD) | 0 |
| **Mantle Cell Lymphoma Subgroup** | **(n=6)** |
| CR | 4 (67%) |
| MRD-negative * | 4/4 (100%) |
| SD | 2 (33%) |

** MRD status assessed in patients who achieved CR.*

**Copyright Information:** (Online) ISSN: 2572-9241
©2026 The Author(s). HemaSphere is published by John Wiley & Sons Ltd on behalf of European Hematology Association. This is an open access Abstract Book distributed under the Attribution-NonCommercial-NoDerivs (CC BY-NC-ND), which allows third parties to download the articles and share them with others as long as they credit the author and the Abstract Book, but they cannot change the content in any way or use them commercially.
**Abstract Book Citations:** Authors, Title, HemaSphere, 2026;10:(S1):pages. The abstract book DOIs can be found on the "Author Information" page.
**Disclaimer:** Articles published in the journal HemaSphere exclusively reflect the opinions of the authors. The authors are responsible for all content in their abstracts including accuracy of the facts, statements, citing resources, etc.

### (S288) DURABLE CLINICAL BENEFITS WITH RM001 (AUTOLOGOUS HBG1/2 PROMOTER-MODIFIED CD34+ HEMATOPOIETIC STEM AND PROGENITOR CELLS) FOR TRANSFUSION-DEPENDENT β-THALASSEMIA

**Topic:** 26. Gene therapy, cellular immunotherapy and vaccination - Clinical

Rongrong Liu[1], Li Wang[2], Hui Xu*[3], Jianpei Fang[4], Sixi Liu[5], Xiaolin Yin[2], Junbin Liang[3], Xinyu Li[6], Yue Li[5], Gaohui Yang[1], Yaoyun Li[2], Yali Zhou[2], Lin Cheng[3], Yongrong Lai[1], Junjiu Huang[7], Xinhua Zhang[2]

*[1]The First Affiliated Hospital of Guangxi Medical University, Nanning, China;[2]923rd Hospital of the People's Liberation Army, Nanning, China; [3]Reforgene Medicine, Guangzhou, China; [4]Sun Yat-Sen Memorial Hospital of Sun Yat-Sen, Guangzhou, China; [5]Shenzhen Children's Hospital, Shenzhen, China; [6]Sun Yat-Sen Memorial Hospital of Sun Yat-Sen University, Guangzhou, China; [7]Sun Yat-sen University, Guangzhou, China;*

### Background

RM001 is an investigational gene editing therapy for β-hemoglobinopathies that reactivates fetal hemoglobin (HbF) through *ex vivo* CRISPR-Cas9 editing of the BCL11A binding site in the promoters of γ-globin gene (*HBG1/2*) in autologous CD34+ hematopoietic stem and progenitor cells (HSPCs). We report long-term efficacy and safety for participants with TDT in the early clinical studies.

### Aims

Both an investigator-initiated trial (IIT; ChiCTR2100053406 and ChiCTR2100052858, n=7) and a Phase I trial (ChiCTR2300069244, n=12) were conducted to evaluate the safety and efficacy of RM001 in patients with transfusion-dependent β-thalassemia (TDT).

### Methods

Eligible participants (aged 6 to 35 years) had a history of packed red blood cell (pRBC) transfusions ≥100 mL/kg/year or ≥10 units/year within the 2 years prior to screening. The primary efficacy endpoint was transfusion independence (TI12), defined as the proportion of patients maintaining a weighted average hemoglobin ≥9 g/dL without pRBC transfusion for ≥12 consecutive months. TI12 assessment began 60 days after the last transfusion. Patients who complete the 24-month trial will be transitioned to a long-term follow-up study.

**Copyright Information:** (Online) ISSN: 2572-9241
©2026 The Author(s). HemaSphere is published by John Wiley & Sons Ltd on behalf of European Hematology Association. This is an open access Abstract Book distributed under the Attribution-NonCommercial-NoDerivs (CC BY-NC-ND), which allows third parties to download the articles and share them with others as long as they credit the author and the Abstract Book, but they cannot change the content in any way or use them commercially.
**Abstract Book Citations:** Authors, Title, HemaSphere, 2026;10:(S1):pages. The abstract book DOIs can be found on the "Author Information" page.
**Disclaimer:** Articles published in the journal HemaSphere exclusively reflect the opinions of the authors. The authors are responsible for all content in their abstracts including accuracy of the facts, statements, citing resources, etc.

**Results**

As of January 25, 2026, 19 patients (mean age, 15.9 years; 5 patients aged ≥18 years, 7 aged ≥12 to <18 years, and 7 aged ≥6 to <12 years) had received RM001, with a median follow-up of 32.8 months (range, 25.4 to 50.9). Before enrollment, patients received a mean of 56.9 units/year (range, 35.3 to 106.3) of pRBC transfusions. Genotypes included $\beta^0/\beta^0$ in 13 patients (68.4%), $\beta^0/\beta^+$ in 5 patients (26.3%), and $\beta^+/\beta^+$ in 1 patient (5.3%). All patients achieved neutrophil engraftment (median, 15 days) and platelet engraftment (median, 21 days). All 19 patients (100%) discontinued transfusions and remained transfusion-free for ≥22 months (range, 22.6 to 48.4). Patients stopped transfusions at a median of 22 days (range, 10 to 94) after RM001 infusion, with stable hemoglobin levels ≥9 g/dL at a median of 31 days (range, 14 to 127).
All patients had ≥25 months of follow-up after RM001 infusion, with all (100%) achieving TI12 within 2 years post-treatment, with mean total hemoglobin levels of 10.8 g/dL at month 3, 11.6 g/dL at month 6, 11.8 g/dL at month 12, and 12.3 g/dL at month 24, and corresponding fetal hemoglobin (HbF) levels of 9.2, 11.4, 11.6, and 12.1 g/dL. HBG1/2 allele editing proportions remained stable in patient's bone marrow over time.
All patients (100%) have finished 24-month follow-up and enrolled in a long-term study. The first 5 patients have been followed over 4 years and their Hb levels maintained stably above 10 g/dL.
No treatment-related serious adverse events were reported. All adverse events resolved completely, with no deaths, treatment discontinuations, or malignancies observed.

**Summary/Conclusion**

The data from 19 TDT patients infused with RM001 demonstrated clinically meaningful and sustained increases in total Hb and HbF, leading to transfusion independence in all subjects. The safety profile of RM001 was excellent and no product-related serious adverse events were reported during the study. These results indicate that RM001 has potential to cure TDT with one-time treatment.

**Copyright Information:** (Online) ISSN: 2572-9241
©2026 The Author(s). HemaSphere is published by John Wiley & Sons Ltd on behalf of European Hematology Association. This is an open access Abstract Book distributed under the Attribution-NonCommercial-NoDerivs (CC BY-NC-ND), which allows third parties to download the articles and share them with others as long as they credit the author and the Abstract Book, but they cannot change the content in any way or use them commercially.
**Abstract Book Citations:** Authors, Title, HemaSphere, 2026;10:(S1):pages. The abstract book DOIs can be found on the “Author Information” page.
**Disclaimer:** Articles published in the journal HemaSphere exclusively reflect the opinions of the authors. The authors are responsible for all content in their abstracts including accuracy of the facts, statements, citing resources, etc.

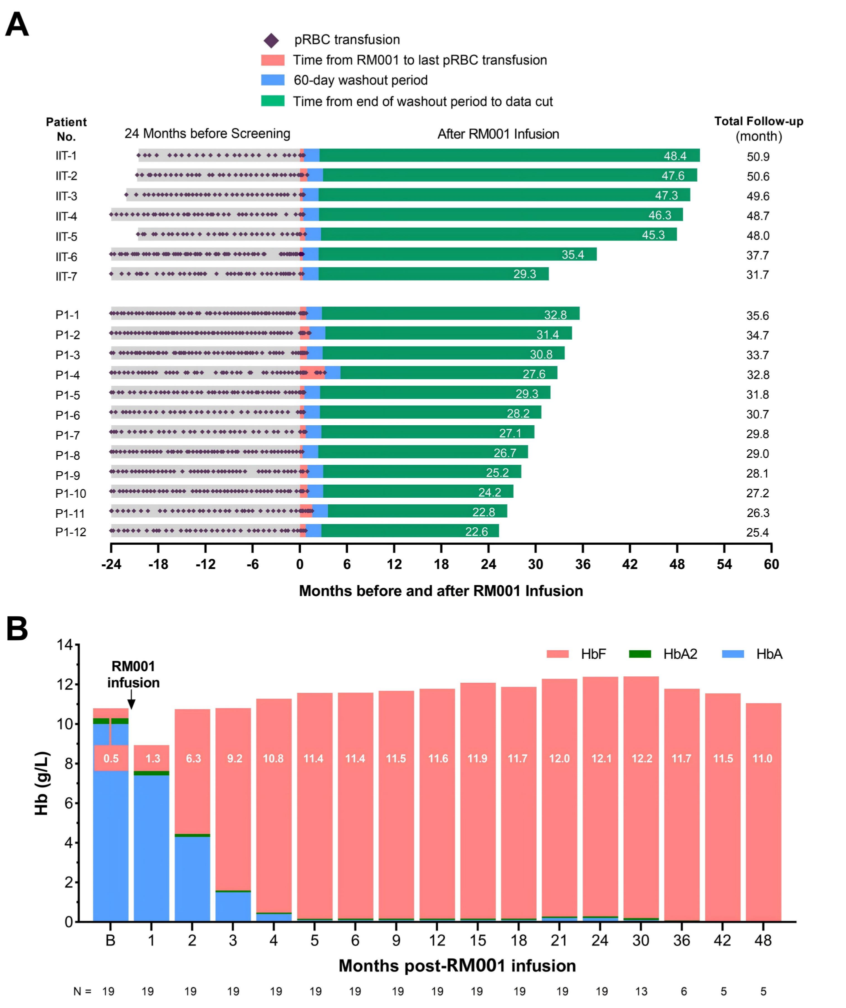

A. Prior transfusion frequency and time to transfusion-free status; B.Proportions of HbF, HbA, and HbA2 hemoglobin in peripheral blood.

**Copyright Information:** (Online) ISSN: 2572-9241
©2026 The Author(s). HemaSphere is published by John Wiley & Sons Ltd on behalf of European Hematology Association. This is an open access Abstract Book distributed under the Attribution-NonCommercial-NoDerivs (CC BY-NC-ND), which allows third parties to download the articles and share them with others as long as they credit the author and the Abstract Book, but they cannot change the content in any way or use them commercially.
**Abstract Book Citations:** Authors, Title, HemaSphere, 2026;10:(S1):pages. The abstract book DOIs can be found on the "Author Information" page.
**Disclaimer:** Articles published in the journal HemaSphere exclusively reflect the opinions of the authors. The authors are responsible for all content in their abstracts including accuracy of the facts, statements, citing resources, etc.

### (S289) PRE-TRANSPLANT BLINATUMOMAB AND CAR-T SHAPE IMMUNE RECONSTITUTION DYNAMICS IN CHILDREN

**Topic:** 23. Stem cell transplantation - Clinical

Silvia Nucera*[1], Violante Gustuti[1, 2], Anita Toso[2], Dorothea Bauer[3], Marco Maria Sindoni[2], Anita Lawitschka[3], Giuseppe Gaipa[4], Adriana Balduzzi[1, 2]

*[1]Pediatric Department, Fondazione IRCCS San Gerardo dei Tintori, Monza, Italy; [2]University of Milano Bicocca, School of Medicine and Surgery, Monza, Italy; [3]St. Anna Children's Hospital, Medical University Vienna and Children's Cancer Research Institute, Vienna, Austria; [4]Tettamanti Center, Fondazione IRCCS San Gerardo dei Tintori, Monza, Italy;*

### Background

Immune reconstitution (IR) after allogeneic hematopoietic cell transplant (HCT) is one of the main determinants of patients' outcomes. Post-HCT IR is influenced by many pre- and post-HCT treatments, especially those acting on the immune system (e.g. GvHD prophylaxis). Anti-CD19 therapies, including Blinatumomab and CAR-T cells are increasingly used in acute lymphoblastic leukemia (ALL) patients, and often precede HCT. However, whether the use of these therapies canimpact post-HCT IR is still unknown.

### Aims

Evaluate whether pre-HCT exposure to Blinatumomab or CAR-T cells affects post-HCTIR kinetics.

### Methods

We retrospectively evaluated 133 children who underwent HCT for ALL between 2011 and 2025 at Fondazione IRCCS San Gerardo dei Tintori. We evaluated IR in the first 12 months post-HCT as assessed for diagnostic routine monitoring (6-colors TBNK, BD). On a subset of these patients (n=29) we evaluated the composition of CD8+ compartment by using EuroFlow PERISCOPE Cytotoxic antibody panel. The analysis was performed according to EuroFlow guidelines.

### Results

Patients who received Blinatumomab (n=24) display a different kinetic of B cell reconstitution, with higher numbers of B cells, which becomes evident after 6 months (Fig1A). At later timepoints (11-12 months), these patients also shows higher CD4+ T cell counts (Fig1B).
Conversely, patients who received CAR-T (n=18) displayed a more rapid CD3+ (Fig1C ) and CD8+ T cell reconstitution in the first 3 months (Fig1C), while no difference was observed for CD4+ T and NK cells (Fig1D-E). To better characterize whether the expansion of CD8+ T cells is associated with a change in their phenotype, we exploited PERISCOPE cytotoxic antibody panel, which analyzes in detail the CD8+ compartment. As shown in Fig1E, the expansion of CD8+ T cells is confirmed with this panel, both at month 1 and 3. The overall composition of CD8+ compartment (Fig1F) is only mildly affected, with an expansion of central memory (CM) T cells and contraction of transitional memory (TM) in the first month, while the difference is already lost at 3 months. Interestingly, patients with prior CAR-T had a lower incidence of acute GvHD (Fig1G) but the timing of onset was comparable in the three groups. On the other hand, patients who received prior Blinatumomab had a lower incidence of chronic GvHD (Fig1H).

**Copyright Information:** (Online) ISSN: 2572-9241
©2026 The Author(s). HemaSphere is published by John Wiley & Sons Ltd on behalf of European Hematology Association. This is an open access Abstract Book distributed under the Attribution-NonCommercial-NoDerivs (CC BY-NC-ND), which allows third parties to download the articles and share them with others as long as they credit the author and the Abstract Book, but they cannot change the content in any way or use them commercially.
**Abstract Book Citations:** Authors, Title, HemaSphere, 2026;10:(S1):pages. The abstract book DOIs can be found on the "Author Information" page.
**Disclaimer:** Articles published in the journal HemaSphere exclusively reflect the opinions of the authors. The authors are responsible for all content in their abstracts including accuracy of the facts, statements, citing resources, etc.

**Summary/Conclusion**

We hypothesize that selective B cell depletion by pre-HCT Blinatumomab may facilitate post-HCT B cell expansion and physiological maturation, as suggested by the lower incidence of cGvHD. The improved CD4+ T cell recovery in Blinatumomab group may fit both with B cell recovery, as these subsets were shown to be interconnected, and with reduced cGvHD and immune suppressive burden. On the other hand, in patients who receive anti-CD19 CAR-T cells, we observed no effect on B cells. Instead, early expansion of CD8+ T cells may be a consequence of prior lymphodepletion. Importantly, in our cohort, most patients received their first HCT (124/133). As shown in the figure, patients who received prior CAR-T had a lower incidence of aGvHD. Whether this is related to different kinetics in immune reconstitution needs to be assessed with ad hoc prospective studies. This is a preliminary monocentric study and multicentric validation is ongoing. However, these data suggest that CAR-T and Blinatumomab should be listed among the pre-transplant treatments that impact on post-HCT IR.

**Copyright Information:** (Online) ISSN: 2572-9241
©2026 The Author(s). HemaSphere is published by John Wiley & Sons Ltd on behalf of European Hematology Association. This is an open access Abstract Book distributed under the Attribution-NonCommercial-NoDerivs (CC BY-NC-ND), which allows third parties to download the articles and share them with others as long as they credit the author and the Abstract Book, but they cannot change the content in any way or use them commercially.
**Abstract Book Citations:** Authors, Title, HemaSphere, 2026;10:(S1):pages. The abstract book DOIs can be found on the “Author Information” page.
**Disclaimer:** Articles published in the journal HemaSphere exclusively reflect the opinions of the authors. The authors are responsible for all content in their abstracts including accuracy of the facts, statements, citing resources, etc.

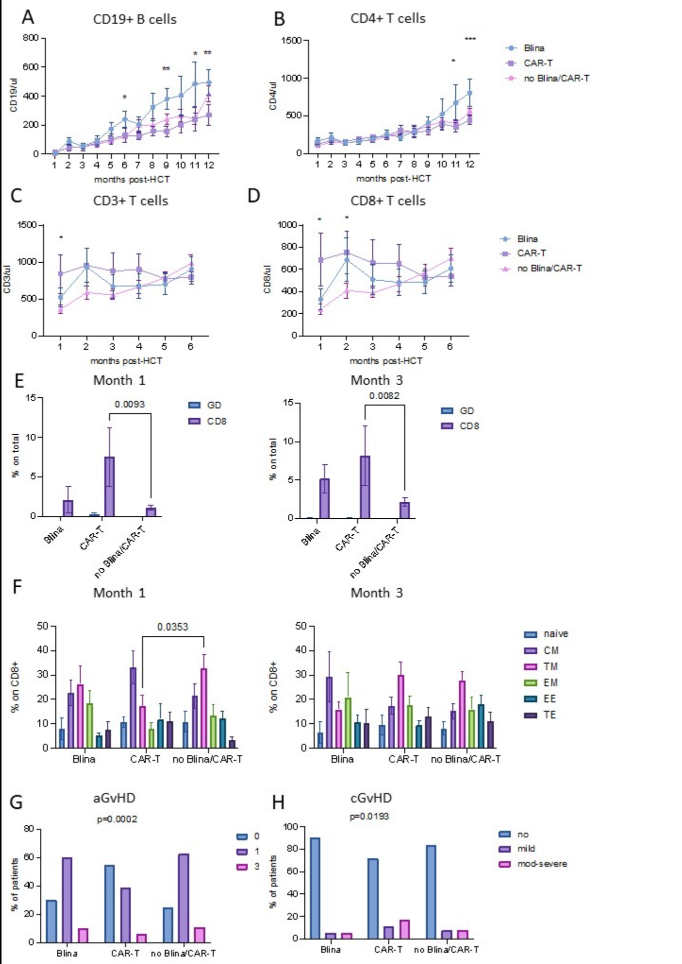

(A) B cell reconstitution in the first 12 months. Mixed effect analysis. m6 Blina vs no Blina/CAR-T p= 0,0432; m9 Blina vs CAR-T p=0.0088 and Blina vs no Blina/CAR-T p=0.0289, m11 Blina vs no Blina/CAR-T p=0.0208, m12 Blina vs CAR-T p=0.006. (B) CD4 T cell reconstitution in the first 12 months. Mixed effect analysis. m11 Blina vs CAR-T p= 0.02, Blina vs no Blina/CAR-T p=0.02, m12 Blina vs CAR-T p= 0.0004, Blina vs no Blina/CAR-T p=0.002. (C) CD3 T cell reconstitution in the first 6 months. Mixed effect analysis. m1 CAR-T vs no Blina/CAR-T p=0.029. (D) CD8 T cell reconstitution in the first 6 months. Mixed effect analysis. m1 CAR-T vs no Blina/CAR-T p=0.0354, m2 CAR-T vs no Blina/CAR-T p=0.0122, m2 CAR-T vs no Blina/CAR-T p=0.0386. (E) CD8 and gamma delta (GD) T cells at month 1 and 3 analyzed with Periscope panel. 2 way ANOVA with Tukey's multiple comparison. (F) Composition of CD8 compartment analyzed with Periscope. 2 way ANOVA with Tukey's multiple comparison. (G) Percentage of patients with acute GvHD divided by grading. Chi square test. (H) Percentage of patients with chronic GvHD divided by grading. Chi square test.

**Copyright Information:** (Online) ISSN: 2572-9241
©2026 The Author(s). HemaSphere is published by John Wiley & Sons Ltd on behalf of European Hematology Association. This is an open access Abstract Book distributed under the Attribution-NonCommercial-NoDerivs (CC BY-NC-ND), which allows third parties to download the articles and share them with others as long as they credit the author and the Abstract Book, but they cannot change the content in any way or use them commercially.
**Abstract Book Citations:** Authors, Title, HemaSphere, 2026;10:(S1):pages. The abstract book DOIs can be found on the "Author Information" page.
**Disclaimer:** Articles published in the journal HemaSphere exclusively reflect the opinions of the authors. The authors are responsible for all content in their abstracts including accuracy of the facts, statements, citing resources, etc.

### (S290) PHASE 1/2 HIBISCUS KIDS STUDY OF ETAVOPIVAT IN PEDIATRIC PATIENTS WITH SICKLE CELL DISEASE: SAFETY AND EFFICACY FINDINGS FROM THE COMPLETE FIRST COHORT

**Topic:** 27. Sickle cell disease

Bernhards Ogutu*[1, 2], Miguel R. Abboud[3], Subarna Chakravorty[4], Iheanyi Okpala[5], Pavithra Dhayakar[6], E. Leila Jerome Clay[7], Kaming Lo[8], Adlette Inati[9, 10]

*[1]KEMRI Kondele Children's Hospital, Kisumu, Kenya; [2]CREATES, Strathmore University, Nairobi, Kenya; [3]American University of Beirut, Beirut, Lebanon; [4]King's College Hospital, London, United Kingdom; [5]University of Nigeria, Nsukka, Nigeria; [6]Novo Nordisk, Bangalore, India; [7]Novo Nordisk Inc., Princeton, NJ, United States of America; [8]Novo Nordisk Inc., Lexington, MA, United States of America; [9]University of Balamand, Faculty of Medicine and Medical Sciences, Koura, Lebanon; [10]NINI Hospital, Tripoli, Lebanon;*

### Background

Etavopivat is an orally bioavailable, small-molecule activator of the pyruvate kinase red blood cell (RBC) (PKR) isozyme in development for treatment of sickle cell disease (SCD). It lowers 2,3-diphosphoglycerate and increases adenosine triphosphate levels, enhancing hemoglobin (Hb)-oxygen affinity, raising Hb levels, and supporting RBC health. HIBISCUS Kids (NCT06198712) is an ongoing global phase 1/2 open-label study to evaluate pharmacokinetics (PK), safety and efficacy of etavopivat in children and adolescents with SCD aged 6 months to <18 years. Each subsequent age-based cohort will initiate treatment after a 12-week (wk) safety and PK review of the prior cohort.

### Aims

To report the 24-wk safety and efficacy findings of etavopivat from the fully recruited cohort 1, aged
12–<18 years.

### Methods

All participants received oral etavopivat 400 mg once daily for 24 wks. Eligible participants provided informed consent, had Hb ≥5.5–<10.5 g/dL at screening, and severe SCD, defined as at least one of the following in the previous year: 2–15 vaso-occlusive crises (VOCs), SCD-related hospitalization, urinary albumin:creatinine ratio >100 mg/g (2 measures), or conditional Transcranial Doppler (TCD).
A concomitant stable dose of hydroxyurea, crizanlizumab, or L-glutamine was permitted.
Primary endpoints included incidence of treatment-emergent adverse events (TEAEs), serious AEs (SAEs), and etavopivat discontinuations, interruptions, or dose reductions over 24 wks. Efficacy endpoints included Hb response rate (>1.0 g/dL increase from baseline [BL]) at wks 12 and 24, annualized VOC rate and number of VOCs over 24 wks, and change from BL in Hb, PROMIS Fatigue T-score, and hemolysis markers at wks 12 and 24. Time-averaged mean of the maximum velocity (TAMMV) by TCD and 6-minute walk test (6MWT) were also assessed.

**Copyright Information:** (Online) ISSN: 2572-9241
©2026 The Author(s). HemaSphere is published by John Wiley & Sons Ltd on behalf of European Hematology Association. This is an open access Abstract Book distributed under the Attribution-NonCommercial-NoDerivs (CC BY-NC-ND), which allows third parties to download the articles and share them with others as long as they credit the author and the Abstract Book, but they cannot change the content in any way or use them commercially.
**Abstract Book Citations:** Authors, Title, HemaSphere, 2026;10:(S1):pages. The abstract book DOIs can be found on the "Author Information" page.
**Disclaimer:** Articles published in the journal HemaSphere exclusively reflect the opinions of the authors. The authors are responsible for all content in their abstracts including accuracy of the facts, statements, citing resources, etc.

### Results

25 adolescent participants (15 female, 20 Black/African American, 23 HbSS, and 2 HbS$\beta^{0}$-thalassemia genotype) were enrolled in cohort 1. Mean (standard deviation [SD]) age was 14.1 (1.48) years and mean (SD) BL Hb was 7.74 (1.07) g/dL.
Most TEAEs (114/119 events) were mild/moderate in severity. The most common TEAEs were sickle cell pain crisis (10/25), increased appetite (5/25), headache (4/25), and tonsillitis (4/25). One SAE (cholestasis with history of cholelithiasis) required dose interruption, event resolved after cholecystectomy.
No deaths were reported.
Early (from wk 2) and sustained improvements in Hb levels from BL were achieved with etavopivat and accompanied by reductions in hemolysis biomarkers (**Table**). 16 (64%) participants achieved Hb response at wk 24. At 24 wks, annualized VOC rate was 2.2, mean (SD) number of VOCs was 1.0 (1.57) per participant. At wk 24, mean TAMMVs with imaging and non-imaging TCDs were similar to BL, and 5/16 participants showed improvement (≥15 cm/s reduction) with non-imaging TCDs. Mean (SD) change from BL in PROMIS Fatigue T-scores was −0.71 (8.04; n=21) at wk 12 and 0.64 (9.52; n=17) at wk 24. All participants completed the 6MWT, with no clear trend observed: mean (SD) change from BL was 0.17 m (75.75) at wk 24.

### Summary/Conclusion

This first dedicated paediatric SCD trial of a PKR activator shows etavopivat's safety profile remains consistent with prior observations and what is expected with the underlying disease, while demonstrating favorable Hb and hemolysis marker changes in adolescents (cohort 1). The study will continue to evaluate etavopivat's safety, tolerability and potential benefits in children from six months of age.

**Copyright Information:** (Online) ISSN: 2572-9241
©2026 The Author(s). HemaSphere is published by John Wiley & Sons Ltd on behalf of European Hematology Association. This is an open access Abstract Book distributed under the Attribution-NonCommercial-NoDerivs (CC BY-NC-ND), which allows third parties to download the articles and share them with others as long as they credit the author and the Abstract Book, but they cannot change the content in any way or use them commercially.
**Abstract Book Citations:** Authors, Title, HemaSphere, 2026;10:(S1):pages. The abstract book DOIs can be found on the “Author Information” page.
**Disclaimer:** Articles published in the journal HemaSphere exclusively reflect the opinions of the authors. The authors are responsible for all content in their abstracts including accuracy of the facts, statements, citing resources, etc.

**Table: Change from baseline in hemoglobin and hemolysis markers at week 12 and week 24.**

| Parameter | BL (n=25) | Week 12 (n=25) | | Week 24 (n=25) | |
|---|---|---|---|---|---|
| | Mean (SD) | Mean (SD) | Mean change from BL (SD) | Mean (SD) | Mean change from BL (SD) |
| Hb (g/dL) | 7.74 (1.069) | 9.25 (1.216) | 1.51 (0.698) | 9.10 (1.109) | 1.37 (0.744) |
| Indirect bilirubin (μmol/L) | 49.91 (31.5644) | 29.20 (20.7804) | −20.71 (34.4270) | 29.47 (13.7392) | −20.44 (24.7675) |
| LDH (IU/L) | 526.87 (171.848) | 389.12 (133.278) | −137.75 (121.666) | 406.37 (165.920) | −120.50 (166.202) |
| ARC ($10^9$/L) | 230.28 (98.607) | 199.25 (90.359) | −31.02 (97.622) | 175.81 (97.387) | −54.47 (75.138) |

BL was defined as the average of screening and day 1 measurement prior to the first dose of etavopivat. Hb at week 12 is the average between week 12 and week 8. Hb at week 24 is the average between week 24 and week 20. ARC, absolute reticulocyte count; BL, baseline; Hb, hemoglobin; LDH, lactate dehydrogenase; SD, standard deviation.

**Copyright Information:** (Online) ISSN: 2572-9241
©2026 The Author(s). HemaSphere is published by John Wiley & Sons Ltd on behalf of European Hematology Association. This is an open access Abstract Book distributed under the Attribution-NonCommercial-NoDerivs (CC BY-NC-ND), which allows third parties to download the articles and share them with others as long as they credit the author and the Abstract Book, but they cannot change the content in any way or use them commercially.
**Abstract Book Citations:** Authors, Title, HemaSphere, 2026;10:(S1):pages. The abstract book DOIs can be found on the “Author Information” page.
**Disclaimer:** Articles published in the journal HemaSphere exclusively reflect the opinions of the authors. The authors are responsible for all content in their abstracts including accuracy of the facts, statements, citing resources, etc.

### (S291) RESULTS OF THE PHASE 1B PIONEER STUDY: SAFETY AND EFFICACY OF POCIREDIR IN ADULTS WITH SEVERE SICKLE CELL DISEASE AND HYDROXYUREA INTOLERANCE OR UNRESPONSIVENESS

**Topic:** 27. Sickle cell disease

Sheinei Alan[1], Johnny Mahlangu[2], Michael Birrer[3], Marshall Patrick Stagg[4], Debra Ferman[5], Rita Lemming[6], Caterina Minniti[7], Thomas Winkler[6], Adeyemi Adenola[6], Livingstone Dogara[8], Foluke Fasola[9], John Jiang[6], Blessing Ojika[10], Modupe Idowu*[11]

*[1]UVA School of Medicine, Inova Campus, Fairfax, VA, USA and Inova Adult Sickle Cell Program, Inova Health System, Fairfax, VA, USA, Fairfax, United States of America; [2]University of the Witwatersrand and NHLS, Johannesburg, South Africa, Johannesburg, South Africa; [3]University of Arkansas for Medical Sciences, Little Rock, AR, USA, Little Rock, United States of America; [4]Franciscan Missionaries of Our Lady Health System, Baton Rouge, LA, USA, Baton Rouge, United States of America; [5]New York City Health + Hospitals/Queens, Jamaica, NY, Jamaica, United States of America;[6]Fulcrum Therapeutics, Cambridge, MA, USA, Cambridge, United States of America; [7]Albert Einstein College of Medicine, Bronx, NY, USA, Bronx, United States of America; [8]Kaduna State University, and Barau Dikko Teaching Hospital, Kaduna, Nigeria, Kaduna, Nigeria; [9]Department of Haematology, College of Medicine, University of Ibadan, University College Hospital, Ibadan, Nigeria, Ibadan, Nigeria; [10]National Hospital Abuja, Abuja, Nigeria, Abuja, Nigeria;[11]Division of Hematology, Department of Internal Medicine, UTHealth McGovern Medical School, Houston, TX, USA, Houston, United States of America;*

### Background

Hemoglobin S polymerization, the primary driver of sickle cell disease (SCD), is inhibited by increased fetal hemoglobin (HbF), reducing anemia and vaso-occlusive crises (VOCs) and improving clinical outcomes. Pociredir is a novel, once-daily, oral small molecule that increases HbF expression by downregulating the repressor protein BCL11A. Preliminary 12-week data demonstrated HbF induction and hematologic improvement in severe SCD.

### Aims

To report safety and efficacy results for cohorts 3b (12 mg) and 4 (20 mg) from the phase 1b PIONEER (NCT05169580) dose-escalation study of pociredir.

### Methods

Adults with severe SCD (≥4 and ≤10 VOCs over 12 months, ≥2 and ≤5 VOCs over 6 months, or documented SCD-related organ damage) who were ineligible for or unresponsive to hydroxyurea were enrolled in 2 dose cohorts (3b [12 mg] and 4 [20 mg]) and treated for 12 weeks with a 4-week posttreatment safety follow-up. All patients (pts) provided written informed consent and the study was approved by each participating center's ethics committee. Primary endpoints were safety and tolerability. Secondary and exploratory endpoints included HbF induction; markers of anemia, hemolysis, and erythropoiesis; and % of F cells. Within-cohort changes from baseline (CFBs) were evaluated using paired *t*-tests without multiplicity adjustment.

**Copyright Information:** (Online) ISSN: 2572-9241
©2026 The Author(s). HemaSphere is published by John Wiley & Sons Ltd on behalf of European Hematology Association. This is an open access Abstract Book distributed under the Attribution-NonCommercial-NoDerivs (CC BY-NC-ND), which allows third parties to download the articles and share them with others as long as they credit the author and the Abstract Book, but they cannot change the content in any way or use them commercially.
**Abstract Book Citations:** Authors, Title, HemaSphere, 2026;10:(S1):pages. The abstract book DOIs can be found on the "Author Information" page.
**Disclaimer:** Articles published in the journal HemaSphere exclusively reflect the opinions of the authors. The authors are responsible for all content in their abstracts including accuracy of the facts, statements, citing resources, etc.

**Results**

Sixteen pts received 12 mg (56% female; mean age, 34.3 years) and 13 pts received 20 mg (83% female; mean age, 32.3 years; pharmacodynamic analysis set excludes 1 pt who discontinued study after 1 dose). All evaluable pts completed 12 weeks of treatment. Pociredir was generally well tolerated, with no dose-limiting toxicities, discontinuations due to treatment-related adverse events (TRAEs), or treatment-related serious AEs. TRAEs (including diarrhea, nausea, headache, paresthesia, catarrh, rhinorrhea, reticulocytopenia, upper respiratory tract infection, insomnia, and rash) were reported for 3 pts in each cohort. HbF increased from baseline to week 12 in all pts (12 mg: mean increase, 8.6% [$P$<0.0001]; 20 mg: mean increase, 12.2% [$P$<0.0001]) (**Figure 1A**). At week 12, ≥20% absolute HbF was achieved by 44% (7/16) pts in the 12-mg cohort and 58% (7/12) pts in the 20-mg cohort (**Figures 1B and 1C**). Hemoglobin (in nontransfused pts) increased significantly from baseline to week 12 in both cohorts (12 mg: mean CFB 0.9 g/dL [$P$=0.001]; 20 mg: mean CFB 1.1 g/dL [$P$=0.0001]). Hemolysis/erythropoiesis markers showed significant improvement from baseline to week 12 in both cohorts. In the 12-mg cohort, mean CFBs for lactate dehydrogenase, indirect bilirubin, absolute reticulocyte count, and red cell distribution width were −171.5 IU/L ($P$=0.0001), −20.9 µmol/L ($P$=0.0005), −121.8 × $10^9$/L ($P$=0.002), and −5.6% ($P$<0.0001), respectively. In the 20-mg cohort, mean CFBs for these markers were −206.8 IU/L ($P$=0.008), −26.4 µmol/L ($P$=0.029), −164.0 × $10^9$/L ($P$=0.023), and −5.5% ($P$<0.0001), respectively. The F-cell percentage at week 12 reached 67.8% in the 12-mg cohort and 63.3% in the 20-mg cohort.

**Summary/Conclusion**

This is the first full data report from pts treated with once-daily pociredir for 12 weeks at 12 or 20 mg. Pociredir was generally well tolerated and demonstrated clinically meaningful, dose-dependent HbF induction, increased total hemoglobin, and improvements in markers of hemolysis and erythropoiesis. These findings support further clinical development of pociredir as a novel, oral HbF inducer in patients with SCD.

**Copyright Information:** (Online) ISSN: 2572-9241
©2026 The Author(s). HemaSphere is published by John Wiley & Sons Ltd on behalf of European Hematology Association. This is an open access Abstract Book distributed under the Attribution-NonCommercial-NoDerivs (CC BY-NC-ND), which allows third parties to download the articles and share them with others as long as they credit the author and the Abstract Book, but they cannot change the content in any way or use them commercially.
**Abstract Book Citations:** Authors, Title, HemaSphere, 2026;10:(S1):pages. The abstract book DOIs can be found on the "Author Information" page.
**Disclaimer:** Articles published in the journal HemaSphere exclusively reflect the opinions of the authors. The authors are responsible for all content in their abstracts including accuracy of the facts, statements, citing resources, etc.

**Figure**. Changes in HbF after pociredir treatment. **(A)** Mean absolute % HbF change from baseline. **(B)** Baseline % HbF and CFB % HbF in cohort 3b (12 mg). **(C)** Baseline % HbF and CFB % HbF in cohort 4 (20 mg). *Patients with multiple transfusions during the treatment period. No patients in cohort 4 received transfusions during the treatment period.

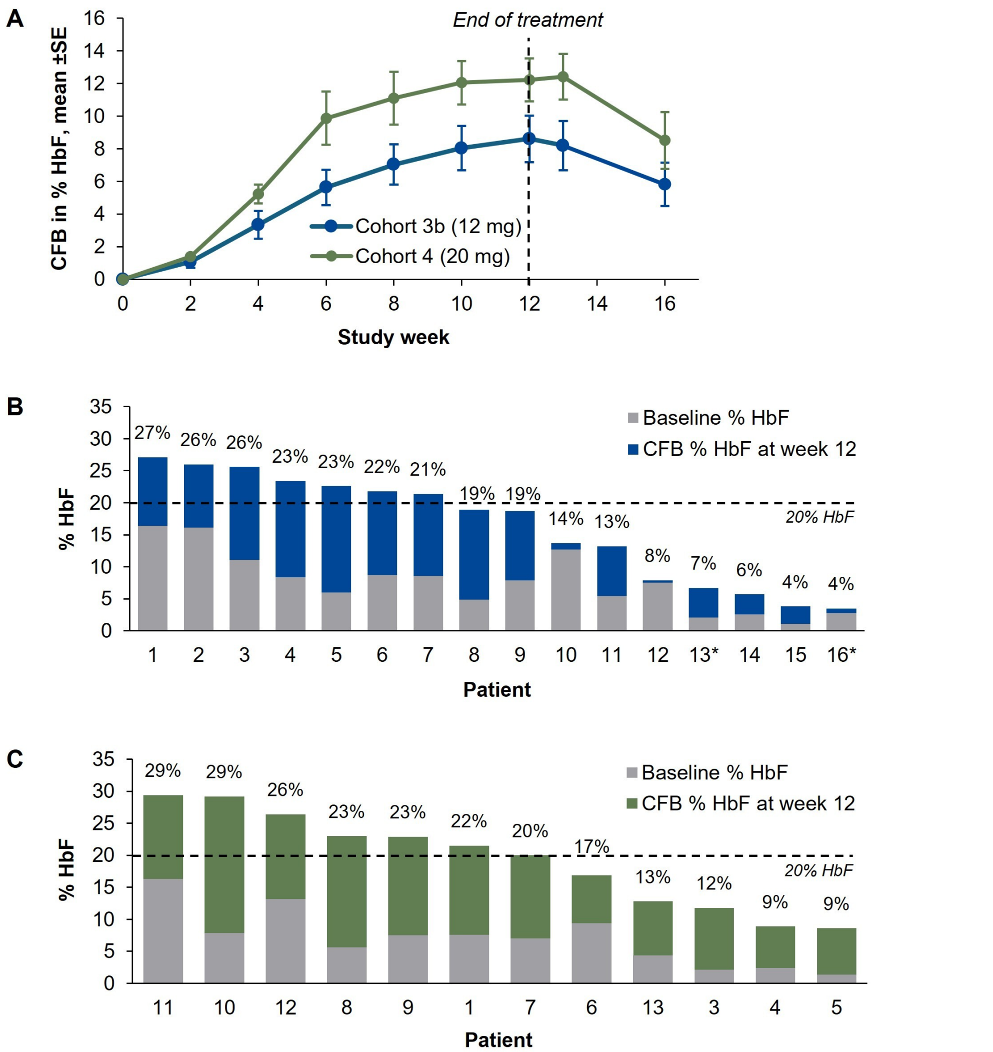

**Copyright Information:** (Online) ISSN: 2572-9241
©2026 The Author(s). HemaSphere is published by John Wiley & Sons Ltd on behalf of European Hematology Association. This is an open access Abstract Book distributed under the Attribution-NonCommercial-NoDerivs (CC BY-NC-ND), which allows third parties to download the articles and share them with others as long as they credit the author and the Abstract Book, but they cannot change the content in any way or use them commercially.
**Abstract Book Citations:** Authors, Title, HemaSphere, 2026;10:(S1):pages. The abstract book DOIs can be found on the "Author Information" page.
**Disclaimer:** Articles published in the journal HemaSphere exclusively reflect the opinions of the authors. The authors are responsible for all content in their abstracts including accuracy of the facts, statements, citing resources, etc.

### (S292) SICKLE CELL DISEASE IN EUROPE: A CROSS-BORDER REAL-WORLD DATA ANALYSIS FROM THE RADEEP REGISTRY

**Topic:** 27. Sickle cell disease

Anna Collado Gimbert*[1, 2], Sara Isabel Reidel[2, 3], Raffaella Colombatti[4], Eduard J. van Beers[5], Paola Bianchi[6], Stella Tamana[7], Angelo Loris Brunetta[8], Dore Peereboom[9], Giovanna Russo[10, 11], Daniela Cuzzubbo[11], Elena Cela[12], José Manuel Marco Sánchez[12], Sarah Wambacq[13], Laurence Dedeken[14], Mariane de Montalembert[15], Karin Fijnvandraat[16, 17], Bart Biemond[16], Frédéric Galactéros[18], Pablo Bartolucci[18, 19], Andreas Glenthøj[20], Marianne Hoffmann[20], Antonis Kattamis[21, 22], Ilona Binenbaum[21, 22], Soterola Christou[23], Irene Savvidou[23], Maria Pilar Nicolás[24], Iñigo de Miguel Beriain[24, 25], Alexis Rodriguez Gallego[2], Angela Menárguez López[1], Maximo Tartaglia[2], Nuria Torquet[2], Roberta Trapanese[4], Marleen Ausloos[13], Betzabel Cajiao Garcia[5], Sandra Manceau[15], Kian Haseli[20], Efthymia Panteli[22, 26], Elisabetta Mezzalira[4], Vincent Malcor Deydier De Pierrefeu[18], Anna Minaidou[7], Petros Kountouris[7], Beatrice Gulbis[13], Maria del Mar Mañú Pereira[2]

*[1]Hospital Universitari Vall d'Hebron, Barcelona, Spain; [2]Vall d'Hebron Institut de Recerca, Barcelona, Spain; [3]Universitat Politècnica de Catalunya - BarcelonaTech, Barcelona, Spain; [4]Azienda Ospedale - Università di Padova, Padova, Italy; [5]University Medical Center Utrecht, Utrecht, The Netherlands; [6]Fondazione IRCCS Ca' Granda, Ospedale Maggiore Policlinico, Milan, Italy; [7]The Cyprus Institute of Neurology & Genetics, Nicosia, Cyprus; [8]Thalassaemia International Federation, Genoa, Italy; [9]Stichting Zeldzame Bloedziekten, Westervoort, The Netherlands; [10]University of Catania, Catania, Italy; [11]Azienda Ospedaliero Universitaria Policlinico "G.Rodolico - San Marco", Catania, Italy; [12]Hospital General Universitario Gregorio Marañón, Madrid, Spain; [13]Hôpital Universitaire de Bruxelles - Université Libre de Bruxelles, Brussels, Belgium; [14]Hôpital Universitaire des Enfants Reine Fabiola, Brussels, Belgium; [15]Assistance Publique - Hôpitaux de Paris - Hôpital Necker Enfants Malades - Université Paris-Cité, Paris, France; [16]Amsterdam UMC, University of Amsterdam, Amsterdam, The Netherlands; [17]Sanquin Research, Amsterdam, Spain; [18]Assistance Publique - Hôpitaux de Paris - Hôpital Henri Mondor, Paris, France; [19]Institut Mondor de Recherche Biomédicale, Paris, France; [20]Copenhagen University Hospital - Rigshospitalet, Copenhagen, Denmark; [21]National and Kapodistrian University of Athens, Athens, Greece; [22]Aghia Sophia Children's Hospital, Athens, Greece; [23]Archbishop Makarios III Hospital, Nicosia, Cyprus; [24]University of the Basque Country, Vizcaya, Spain; [25]Ikerbasque, Basque Foundation for Science, Bilbao, Spain; [26]National & Kapodistrian University of Athens, Athens, Greece;*

### Background

Advances in understanding the pathophysiology and clinical heterogeneity of sickle cell disease (SCD) have led to new therapies and patient stratification. However, lack of cross-border standardized data and limited integration into health policy planning hinders personalized care.

### Aims

To describe the largest cross-border European cohort of SCD patients recorded in the RADeep Registry, endorsed by European Hematology Association, from 01.12.2021–30.11.2025. The aim is to characterize disease burden, acute events, organ damage, treatment patterns, and healthcare utilization.

### Methods

Prospective longitudinal patient-level data were collected from RADeep-participating EU centers. Patients with prior curative stem cell transplantation or gene therapy were excluded. Analyses were stratified by age (pediatric <18 years vs adult), subdiagnosis (SCA vs SC), genotype ($\beta^S\beta^S$, $\beta^S\beta^o$, $\beta^S\beta^C$, $\beta^S\beta^+$) and hydroxyurea (HU) use.

**Copyright Information:** (Online) ISSN: 2572-9241
©2026 The Author(s). HemaSphere is published by John Wiley & Sons Ltd on behalf of European Hematology Association. This is an open access Abstract Book distributed under the Attribution-NonCommercial-NoDerivs (CC BY-NC-ND), which allows third parties to download the articles and share them with others as long as they credit the author and the Abstract Book, but they cannot change the content in any way or use them commercially.
**Abstract Book Citations:** Authors, Title, HemaSphere, 2026;10:(S1):pages. The abstract book DOIs can be found on the "Author Information" page.
**Disclaimer:** Articles published in the journal HemaSphere exclusively reflect the opinions of the authors. The authors are responsible for all content in their abstracts including accuracy of the facts, statements, citing resources, etc.

**Results**

A total of 3985 patients were included across 94 centers in 8 countries (Belgium, Cyprus, Denmark, France, Greece, Italy, The Netherlands and Spain); 51.3% were female, and median age was 14 years (60.8% pediatric), though only 48.3% diagnosed via neonatal screening. Subdiagnoses distribution was 71% SCA, 16% SC, 7.3% Sβthalassemia and 1.8% for others. Confirmatory genotypes were available only for 32.2%.
Two or more acute pain vaso-occlusive crises (VOC) were observed in 8.4% of patients (12.8% adults vs 5.6% pediatric). When using broader vaso-occusive events (VOE) definition (VOC, acute chest syndrome, priapism and splenic or hepatic sequestration), ≥2 were reported in 11% (15.9% adults vs 7.8% pediatric), with higher rates in SCA vs SC (12.6% vs 6.1%) in all age groups, and in patients receiving HU (13.1% vs 5.8%).
At least one chronic organ damage manifestation was reported in 65.9% of adults (SCA 69%, SC 64.9%) and 18.9% of pediatric patients (SCA 20.4%, SC 15.3%). HU-treated cohort reported higher rates across all age groups and subdiagnoses.
In adults, microalbuminuria was reported in 12.5% (15.9% SCA, 5% SC) and renal insufficiency, in 5.5% (7% SCA, 0.83% SC). None was substantially reported in pediatrics.
Acute and chronic complications were higher in SCA compared to $\beta^S\beta^S$ confirmed genotype, suggesting that SCA subdiagnosis includes a heterogeneous subgroup ($\beta^S\beta^o$, or $\beta^S/\delta\beta^o$ thalassemia) with different clinical phenotypes.
HU was prescribed to 71.9% of patients (84.1% SCA vs 26.2% SC), with no age cohort differences in SCA. However, in SC, higher rates were reported for adult vs pediatric patients (30.5% vs 23.6%).

**Summary/Conclusion**

Although β-globin genotype is the main genetic modifier of disease severity, it is available in only one third of patients.
The SC cohort exhibits substantial chronic morbidity, sometimes surpassing SCA, with increasing HU use with age, supporting the hypothesis of a moderate pediatric phenotype that progresses to greater severity over time and has historically been underestimated.
The low proportion meeting the ≥2 VOC/VOE threshold suggests that commonly used clinical trial eligibility criteria may not reflect real-world SCD population, potentially limiting access to emerging therapies.
RADeep provides a harmonized platform for data-driven research in SCD and supports generation of regulatory-grade real-world evidence and standardized cohort identification for advanced clinical research.

**Copyright Information:** (Online) ISSN: 2572-9241
©2026 The Author(s). HemaSphere is published by John Wiley & Sons Ltd on behalf of European Hematology Association. This is an open access Abstract Book distributed under the Attribution-NonCommercial-NoDerivs (CC BY-NC-ND), which allows third parties to download the articles and share them with others as long as they credit the author and the Abstract Book, but they cannot change the content in any way or use them commercially.
**Abstract Book Citations:** Authors, Title, HemaSphere, 2026;10:(S1):pages. The abstract book DOIs can be found on the "Author Information" page.
**Disclaimer:** Articles published in the journal HemaSphere exclusively reflect the opinions of the authors. The authors are responsible for all content in their abstracts including accuracy of the facts, statements, citing resources, etc.

### (S293) IN HUMANIZED SICKLE CELL MICE, COLCHICINE PROTECTS AGAINST THE PROGRESSION OF SICKLE CELL CARDIOMYOPATHY

**Topic:** 27. Sickle cell disease

Iana Iatcenko[1], Enrica Federti*[1], Alessandra Ghigo[2], Jacopo Ceolan[1], Rebecca Priolo[2], Antonio Recchiuti[3], Immacolata Andolfo[4], Achille Iolascon[4], Alessandro Mattè[1], Richard Pozzetto Huot[1], Veronica Riccardi[1], Simone Villaboni[1], Emanuela Tolosano[2], Elisa Gremese[1], Manuela Stella[5], Gian Luca Forni[6], Lucia De Franceschi[1]

*[1]University of Verona, Verona, Italy; [2]University of Torino, Torino, Italy; [3]"G. d'Annunzio" University of Chieti – Pescara, Chieti, Italy; [4]Università degli studi di Napoli "Federico II", Napoli, Italy;[5]University of Genova, Genova, Italy; [6]For Anemia Foundation, Genova, Italy;*

**Background**

Sickle cell disease (SCD) is a globally distributed hereditary red cell disorder with still high mortality. Growing evidence indicate that sickle cell related cardiovascular disease contributes to the early death of adults with SCD. Recently, in humanized mouse model for SCD, we reported that chronic mild inflammation lays a key role in the pathogenesis of sickle cell cardiomyopathy.

**Aims**

To evaluate the role of inflammaging in the development of sickle cell cardiomyopathy in humanized SS mice and assess the protective effect of Colchicine (CLC).

**Methods**

Experiments were carried out using 3 and 8 months-old sex and aged matched healthy [Hbatm1(HBA)Tow Hbbtm3(HBG1, HBB)Tow] and sickle cell [Hbatm1(HBA)Tow Hbbtm2(HBG1,HBB*)Tow] mice. Colchicine (0.1 mg/kg daily) or vehicle (water) were administrated for 4 months by gavage, starting at 3-4 months of age. We also evaluated a subgroup of animals treated with hydroxyurea (HU) at the dosage of 50 mg/Kg/d for 3 months.

**Results**

Here, we show that humanized SCD mice developed an age-dependent cardiomyopathy characterized by (i) increased circulating Th17 lymphocytes, Th17 heart infiltration associated with increased plasma IL-17; (ii) collagen deposition and activation of both platelet derived growth factor-B (PDGF-B) and transforming growth factor-b1 (TGF-b1) canonical pathways; (iii) overactivation of heart NF-kB associated with up-regulation of markers of inflammatory vasculopathy. These data support the role of inflammaging in the natural history of sickle cell related cardiomyopathy. As a proof of concept, we used colchicine (CLC) that acts as anti-inflammatory drug with immunomodulatory effects against inflammatory vasculopathy and cardiovascular diseases. In humanized SCD mice, we demonstrated the protective role of low-dose CLC treatment against (i) chronic inflammation with the reduction in both circulating and heart lymphocytes Th17infiltration; (ii) overactivation of NF-kB pathway; and (iii) pathologic heart remodeling with down-regulation of PDGF-B and TGF-b1 pathways, preserving myocardial performance. Of note, hydroxyurea treatment reduces the expression of PDGF-B without change in collage deposition or IL-17 hear infiltration.

**Summary/Conclusion**

Taken together our data revealed the novel role of inflammaging in the development of sickle cell cardiomyopathy. CLC might be proposed as possible new therapeutic tool to treat early stage of sickle cell cardiomyopathy.

**Copyright Information:** (Online) ISSN: 2572-9241
©2026 The Author(s). HemaSphere is published by John Wiley & Sons Ltd on behalf of European Hematology Association. This is an open access Abstract Book distributed under the Attribution-NonCommercial-NoDerivs (CC BY-NC-ND), which allows third parties to download the articles and share them with others as long as they credit the author and the Abstract Book, but they cannot change the content in any way or use them commercially.
**Abstract Book Citations:** Authors, Title, HemaSphere, 2026;10:(S1):pages. The abstract book DOIs can be found on the "Author Information" page.
**Disclaimer:** Articles published in the journal HemaSphere exclusively reflect the opinions of the authors. The authors are responsible for all content in their abstracts including accuracy of the facts, statements, citing resources, etc.

### (S294) A PLASMA–PLACENTAL ATLAS OF SICKLE CELL PREGNANCY REVEALS ENDOTHELIAL, COAGULATION, AND COMPLEMENT DYSREGULATION

**Topic:** 27. Sickle cell disease

Alexandra Boye-Doe*[1], Valentina Restrepo[1], Mohammed Ali[1], Jennifer Afranie-Sakyi[1], Molly McAdow[1], Layla Van Doren[1], Anish Sharda[1]

[1] *Yale University School of Medicine, New Haven, United States of America;*

### Background

Pregnancy in sickle cell disease (SCD) is high-risk, with maternal morbidity–mortality rates up to 20-fold higher than the general population. The pathophysiology of SCD culminates in endothelial injury, inflammation and hypercoagulability, which are amplified in pregnant persons with SCD (PP-SCD) as evidenced by abnormal placental pathology and adverse pregnancy outcomes (APO). Complement activation may also contribute to SCD pregnancy; anecdotal reports suggest potential utility of anti-complement therapy in severe cases. Yet, the vascular-inflammatory milieu of PP-SCD remains poorly defined, the characterization of which may enable diagnostic and therapeutic biomarker discovery.

### Aims

To generate a proteo-transcriptomic atlas of PP-SCD and identify candidate biomarkers and functional pathways that distinguish PP-SCD from healthy pregnancy and non-pregnant SCD.

### Methods

Serial plasma was collected each trimester and postpartum from PP-SCD (n=6; 5 HbSS, 1 HbSC), with age/genotype-matched non-pregnant SCD controls not in crisis (nPP-SCD; n=8) and pregnant persons with normal hemoglobin (PP-N). Plasma proteomics was performed by SomaScan Select 3.7K assay (~4,000 proteins). Complement proteins were orthogonally validated by ELISA (C3, C3a, C4, C4a, C5a, C5b-9, CFH). Placental scRNA-seq (10x Chromium 3′ v5) was performed in 3 uncomplicated PP-SCD and compared with 2 PP-N. Differential abundance/expression and pathway enrichment used multiple-testing correction (FDR<0.1); features were prioritized at $|\log2FC| \geq 0.58$ and $p<0.05$.

### Results

Comparing PP-SCD vs PP-N, 595 (early pregnancy) and 114 (late pregnancy) plasma proteins were differentially abundant. Enriched pathways included endothelial activation, coagulation, complement, hypoxia, angiogenesis, and innate immune signaling. Trajectory analysis (early→late change) revealed aberrant temporal patterns in endothelial markers (ge.g FLT1, TIE1, vWF, TFPI, E-selectin) and coagulation/complement proteins (e.g. ficolins, CFP, F9, F11), indicating progressive vascular inflammation across gestation. ELISA validation showed upstream complement consumption (decreased C3 and C4) together with marked elevation of anaphylotoxin C4a and terminal complement complex C5b-9 in PP-SCD versus PP-N, with pregnancy-associated increases and postpartum attenuation. Relative to nPP-SCD, PP-SCD exhibited 336 differentially abundant proteins, implicating pregnancy-specific amplification of coagulation–inflammation programs beyond basal SCD. Placental scRNA-seq showed broadly preserved cellular composition in uncomplicated PP-SCD versus uncomplicated PP-N, yet cluster-resolved analyses identified endothelial hyperactivation in SCD (increased vWF and FLT1 with reduced PAPPA) and an activated Hofbauer macrophage transcriptional program, consistent with inflammatory vascular stress despite clinically uncomp

**Copyright Information:** (Online) ISSN: 2572-9241
©2026 The Author(s). HemaSphere is published by John Wiley & Sons Ltd on behalf of European Hematology Association. This is an open access Abstract Book distributed under the Attribution-NonCommercial-NoDerivs (CC BY-NC-ND), which allows third parties to download the articles and share them with others as long as they credit the author and the Abstract Book, but they cannot change the content in any way or use them commercially.
**Abstract Book Citations:** Authors, Title, HemaSphere, 2026;10:(S1):pages. The abstract book DOIs can be found on the "Author Information" page.
**Disclaimer:** Articles published in the journal HemaSphere exclusively reflect the opinions of the authors. The authors are responsible for all content in their abstracts including accuracy of the facts, statements, citing resources, etc.

### Summary/Conclusion

Longitudinal multi-omics profiling defines PP-SCD as a state of dynamic systemic vascular-inflammatory dysregulation spanning endothelial, coagulation, immune and complement pathways. This integrated plasma–placental atlas identifies potentially actionable biomarkers to improve risk stratification and mitigate adverse pregnancy outcomes in SCD.

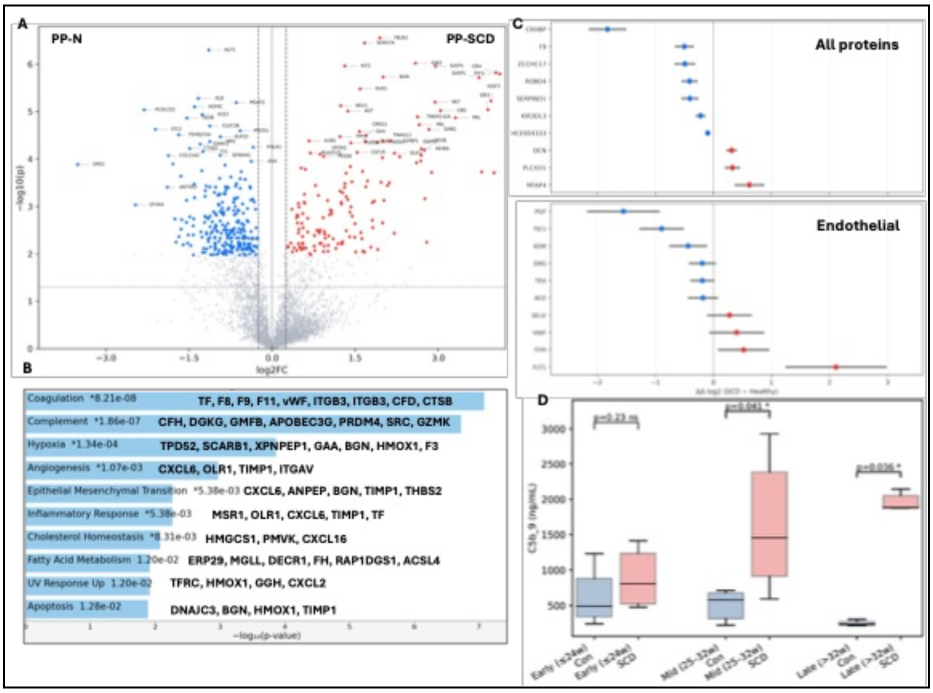

Vascular-inflammation and complement activation in SCD pregnancy. (A) Volcano plot of late-pregnancy plasma proteome differences (PP-SCD vs PP-N). (B) Pathway enrichment of differentially abundant proteins from (A) showing predominant activation of complement, coagulation, hypoxia and angiogenesis related pathways in SCD pregnancy. (C) Forest plots of early→late pregnancy trajectory differences between PP-SCD and PP-N (all proteins (top); endothelial-associated subset (bottom). (D) C5b-9 ELISA demonstrating a significant longitudinal rise in terminal complement complex in PP-SCD with advancing pregnancy.

**Copyright Information:** (Online) ISSN: 2572-9241
©2026 The Author(s). HemaSphere is published by John Wiley & Sons Ltd on behalf of European Hematology Association. This is an open access Abstract Book distributed under the Attribution-NonCommercial-NoDerivs (CC BY-NC-ND), which allows third parties to download the articles and share them with others as long as they credit the author and the Abstract Book, but they cannot change the content in any way or use them commercially.
**Abstract Book Citations:** Authors, Title, HemaSphere, 2026;10:(S1):pages. The abstract book DOIs can be found on the "Author Information" page.
**Disclaimer:** Articles published in the journal HemaSphere exclusively reflect the opinions of the authors. The authors are responsible for all content in their abstracts including accuracy of the facts, statements, citing resources, etc.

### (S295) EFFICACY AND SAFETY OF ORAL TETRAHYDROURIDINE-DECITABINE (NDEC) IN ADULTS WITH SICKLE CELL DISEASE: RESULTS FROM THE PHASE 2, GLOBAL, RANDOMIZED, PARALLEL-GROUP ASCENT1 TRIAL

**Topic:** 27. Sickle cell disease

Biree Andemariam*[1], Priyanka Samal[2], Dharmesh Vaghasiya[3], Modupe Idowu[4], Pernille Højlund Jacobsen[5], Christian Jakob Carlsson[5], Raffaella Colombatti[6]

*[1]University of Connecticut Health, Farmington, Connecticut, United States of America;[2]Institute of Medical Sciences and SUM Hospital, Siksha O Anusandhan University, K-8, Kalinga Nagar, Bhubaneswar, Odisha, India; [3]BCI-Blood and Cancer Institute, Surat, Gujarat, India; [4]McGovern Medical School, UTHealth, Houston, Texas, United States of America;[5]Novo Nordisk A/S, Copenhagen, Denmark; [6]University of Padova, Padova, Italy;*

### Background

NDec is an oral modified-release, fixed combination of tetrahydrouridine (THU) and decitabine designed to treat sickle cell disease (SCD). Decitabine directly increases fetal hemoglobin (HbF) by depleting DNA methyltransferase 1 and reactivating HbF gene expression, while THU inhibits cytidine deaminase to prevent rapid decitabine degradation.

### Aims

ASCENT1 (NCT05405114) was a global phase 2 trial evaluating the efficacy and safety of once-weekly (QW) or weekly on two consecutive days (W2CD) NDec vs placebo (PBO) in adults with SCD.

### Methods

Eligible participants (ppts) gave consent, were aged ≥18 years, had any SCD genotype, Hb 5.0–10.5 g/dL, and 2–10 vaso-occlusive crises (VOCs) in the year prior. Ppts with platelets $>800\times10^9$/L or absolute neutrophil count $\leq1.5\times10^9$/L were excluded. Chronic transfusion therapy, hematopoietic growth factors, recent voxelotor, crizanlizumab, or L-glutamine use were prohibited. Based on hydroxyurea (HU) use at screening, ppts not treated with HU for ≥12 weeks (wks) entered the HU-inactive block; ppts currently treated with stable dose HU entered the exploratory HU-active block. Ppts were randomized to 48 wks of double-blind NDec QW or W2CD (for both, decitabine: 0.16–0.25 mg/kg; THU: 8–12.5 mg/kg), and either PBO (HU-inactive) or open-label HU (HU-active). HU-active ppts randomized to NDec had a 4-wk HU washout.
Primary endpoint was change in Hb from baseline (BL) to wk 24 in HU-inactive ppts. Secondary endpoints (not powered to assess statistical significance) included change in %HbF, %F-cells and hemolysis markers from BL to wk 24, and number of VOCs and transfusions through wk 48. Safety was assessed over 52 wks.

**Copyright Information:** (Online) ISSN: 2572-9241
©2026 The Author(s). HemaSphere is published by John Wiley & Sons Ltd on behalf of European Hematology Association. This is an open access Abstract Book distributed under the Attribution-NonCommercial-NoDerivs (CC BY-NC-ND), which allows third parties to download the articles and share them with others as long as they credit the author and the Abstract Book, but they cannot change the content in any way or use them commercially.
**Abstract Book Citations:** Authors, Title, HemaSphere, 2026;10:(S1):pages. The abstract book DOIs can be found on the “Author Information” page.
**Disclaimer:** Articles published in the journal HemaSphere exclusively reflect the opinions of the authors. The authors are responsible for all content in their abstracts including accuracy of the facts, statements, citing resources, etc.

**Results**

Of 60 HU-inactive ppts (19 NDec QW; 20 NDec W2CD; 21 PBO), 55% were male, 62% from India, 83% HbSS genotype, and 85% 2–5 VOCs in the year prior. Mean (SD) age was 31 (10) years; mean (SD) BL Hb was 8.89 (1.16) g/dL. Several ppts discontinued treatment early (3 NDec QW; 8 NDec W2CD; 2 PBO).
Estimated mean change (95% CI) in Hb from BL to wk 24 was 0.37 g/dL (–0.02, 0.76) with NDec QW, 0.97 g/dL (0.60, 1.34) with NDec W2CD, and 0.00 g/dL (–0.35, 0.35) with PBO. NDec W2CD resulted in significant increases from BL in Hb, %HbF and %F-cells vs PBO at wk 24 (**Figure**)—sustained or increased through wk 48. Despite higher BL %HbF in India vs rest of the world (mean [SD] 19.57% [8.45; n=37] vs 8.21% [4.69; n=20]), there was a clinically relevant increase in mean %HbF from BL in both regions with NDec W2CD.
Reductions in lactate dehydrogenase and absolute reticulocyte count were observed with NDec W2CD at wks 24 and 48. A lower estimated annual VOC rate was observed with NDec W2CD vs PBO (0.84 vs 1.13); estimated rate ratio (95% CI) 0.74 (0.33, 1.69). In total, 13 ppts required transfusions, with fewer transfusions in NDec arms (4 NDec QW; 7 NDec W2CD; 29 PBO).
Treatment-emergent adverse events (AEs) were more frequent with NDec W2CD vs NDec QW or PBO, with similar serious AE rates to PBO. Several ppts experienced neutropenia (n: 1 NDec QW; 9 NDec W2CD; 1 PBO) and thrombocytosis (n: 5 NDec W2CD); most events were detected via routine laboratory tests; all recovered after treatment pause/discontinuation.
In the exploratory HU-active block (9 NDec QW; 10 NDec W2CD; 11 HU), a numerically greater estimated change from BL (pre-washout) in %HbF was observed with NDec W2CD vs HU at wks 24 and 48.

**Summary/Conclusion**

ASCENT1 demonstrated proof of concept for NDec, showing safety and efficacy profiles for W2CD dosing that support progression to phase 3.

**Copyright Information:** (Online) ISSN: 2572-9241
©2026 The Author(s). HemaSphere is published by John Wiley & Sons Ltd on behalf of European Hematology Association. This is an open access Abstract Book distributed under the Attribution-NonCommercial-NoDerivs (CC BY-NC-ND), which allows third parties to download the articles and share them with others as long as they credit the author and the Abstract Book, but they cannot change the content in any way or use them commercially.
**Abstract Book Citations:** Authors, Title, HemaSphere, 2026;10:(S1):pages. The abstract book DOIs can be found on the "Author Information" page.
**Disclaimer:** Articles published in the journal HemaSphere exclusively reflect the opinions of the authors. The authors are responsible for all content in their abstracts including accuracy of the facts, statements, citing resources, etc.

**Figure.** Key efficacy outcomes at week 24 in hydroxyurea-inactive participants

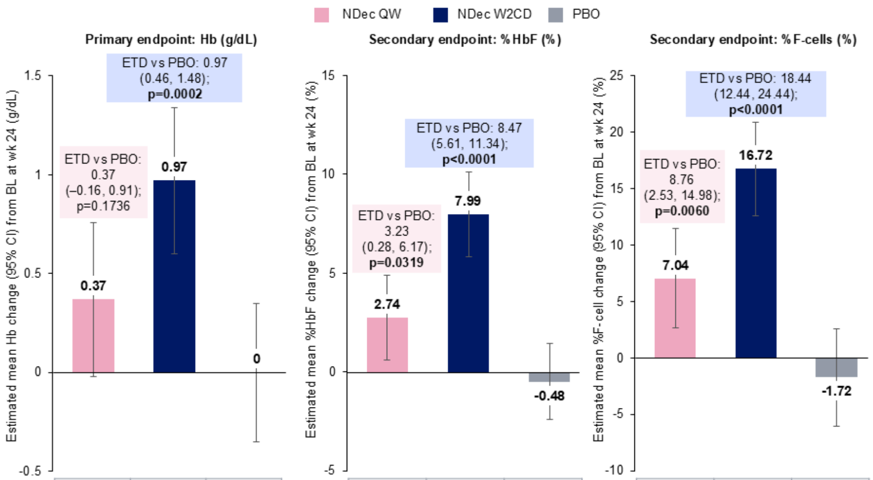

| | Hb NDec QW | Hb NDec W2CD | Hb PBO | %HbF NDec QW | %HbF NDec W2CD | %HbF PBO | %F-cells NDec QW | %F-cells NDec W2CD | %F-cells PBO |
|---|---|---|---|---|---|---|---|---|---|
| N = | 19 | 20 | 21 | 18 | 18 | 21 | 19 | 20 | 18 |
| Mean* (SD): BL | 9.24 (0.95) | 8.87 (1.10) | 8.59 (1.35) | 17.56 (9.96) | 15.14 (10.51) | 14.27 (7.03) | 53.33 (21.69) | 49.38 (22.52) | 54.96 (18.36) |
| Wk 24 | 9.44 (0.59) | 9.82 (0.99) | 8.64 (1.42) | 18.87 (7.86) | 22.65 (8.15) | 13.80 (7.20) | 61.52 (17.33) | 67.64 (15.63) | 52.03 (17.80) |

*Observed mean. Bolded p-values indicate p<0.05. Error bars represented 95% CI of the estimated mean change. N=number of participants contributing to analysis. Mean changes in total Hb, %HbF, and %F-cells were estimated from a mixed model for repeated measurements with treatment, number of vaso-occlusive crises in the previous year, region and sex nested within visit as fixed factors and baseline level of the parameter as a covariate. BL, baseline; CI, confidence interval; ETD, estimated treatment difference; F-cells, HbF-enriched red blood cells; Hb, hemoglobin; HbF, fetal hemoglobin; HU, hydroxyurea; PBO, placebo; QW, once-weekly; SD, standard deviation; W2CD, weekly on two consecutive days; wk, week.

**Copyright Information:** (Online) ISSN: 2572-9241
©2026 The Author(s). HemaSphere is published by John Wiley & Sons Ltd on behalf of European Hematology Association. This is an open access Abstract Book distributed under the Attribution-NonCommercial-NoDerivs (CC BY-NC-ND), which allows third parties to download the articles and share them with others as long as they credit the author and the Abstract Book, but they cannot change the content in any way or use them commercially.
**Abstract Book Citations:** Authors, Title, HemaSphere, 2026;10:(S1):pages. The abstract book DOIs can be found on the “Author Information” page.
**Disclaimer:** Articles published in the journal HemaSphere exclusively reflect the opinions of the authors. The authors are responsible for all content in their abstracts including accuracy of the facts, statements, citing resources, etc.

### (S296) LONG-TERM HEMOGLOBIN IMPROVEMENTS IN NON–TRANSFUSION-DEPENDENT ALPHA- OR BETA-THALASSEMIA: RESULTS FROM THE OPEN-LABEL EXTENSION OF THE ONGOING PHASE 3 ENERGIZE TRIAL

**Topic:** 28. Thalassemias

Hanny Al-Samkari*[1], Lauren Czapla[2], Jeremie H. Estepp[2], Keely Gilroy[2], Shihao Shen[2], Kevin H. M. Kuo[3], Ali T. Taher[4]

*[1]Division of Classical Hematology, Mass General Brigham Cancer Institute, Harvard Medical School, Boston, United States of America; [2]Agios Pharmaceuticals, Inc., Cambridge, United States of America; [3]Division of Hematology, Department of Medicine, University of Toronto, Toronto, Canada; [4]Division of Hematology and Oncology, Department of Internal Medicine, American University of Beirut Medical Center, Beirut, Lebanon;*

### Background

In thalassemia, chronic hemolytic anemia is associated with increased risk of morbidity and mortality; sustained improvement in hemoglobin (Hb) is essential to reducing these risks. Mitapivat, a first-in-class oral allosteric activator of pyruvate kinase (activating the red blood cell–specific PKR isoform and PKM2 isoform), is approved to be used in adults with α- or β-thalassemia, regardless of transfusion needs, in the US and Saudi Arabia. In the international, randomized, placebo-controlled, phase 3 ENERGIZE trial in non–transfusion-dependent (NTD) α- or β-thalassemia (NCT04770753; Eudra CT: 2021-000211-23), 42.3% of patients (pts) receiving mitapivat achieved the primary endpoint of Hb response (≥1.0 g/dL increase in average Hb over weeks [wks] 12-24 vs baseline [BL]) during the 24-wk double-blind period (DBP) vs 1.6% receiving placebo.

### Aims

To assess longer-term Hb outcomes with mitapivat during the ongoing open-label extension (OLE) in ENERGIZE, including among pts who did not achieve Hb response during the DBP.

### Methods

In the DBP of the ENERGIZE trial, adults aged ≥18 y with NTD α- or β-thalassemia and BL Hb ≤10.0 g/dL were randomized 2:1 to mitapivat 100 mg or placebo twice daily. Pts who completed the 24-wk DBP could enter the OLE, in which all pts received mitapivat (M/M, mitapivat in the DBP and OLE; P/M, placebo in the DBP and mitapivat in the OLE). Hb was assessed every 4 wks up to Wk 36 and every 12 wks thereafter.
This post-hoc analysis includes data through the 10 July 2024 cutoff. Duration of Hb response in DBP Hb responders was defined as number of wks from the earliest date after first dose with Hb ≥1 g/dL from BL through the first date with Hb change <1 g/dL from BL after the last DBP assessment, or censored at last assessment if no Hb change <1 g/dL occurred after the DBP. OLE Hb response was defined as ≥1.0 g/dL increase from BL in average Hb during any 2 consecutive study visits in the OLE. Mean change from BL in Hb over time was assessed.

**Copyright Information:** (Online) ISSN: 2572-9241
©2026 The Author(s). HemaSphere is published by John Wiley & Sons Ltd on behalf of European Hematology Association. This is an open access Abstract Book distributed under the Attribution-NonCommercial-NoDerivs (CC BY-NC-ND), which allows third parties to download the articles and share them with others as long as they credit the author and the Abstract Book, but they cannot change the content in any way or use them commercially.
**Abstract Book Citations:** Authors, Title, HemaSphere, 2026;10:(S1):pages. The abstract book DOIs can be found on the "Author Information" page.
**Disclaimer:** Articles published in the journal HemaSphere exclusively reflect the opinions of the authors. The authors are responsible for all content in their abstracts including accuracy of the facts, statements, citing resources, etc.

### Results

Of the 192 pts who received ≥1 dose of mitapivat or placebo in the ENERGIZE DBP, 182 pts enrolled in the OLE, of whom 121 received mitapivat (93.8% of mitapivat arm) and 61 received placebo (96.8% of placebo arm) in the DBP. Median (range) duration of exposure in the M/M and P/M groups was 71.0 (24.1-127.3) and 44.1 (5.4-105.0) wks, respectively; 116 (95.9%) M/M pts and 26 (42.6%) P/M pts had >48 wks of mitapivat exposure.

Among M/M pts, the mean Hb increase achieved during the DBP was sustained during the OLE; P/M pts showed a similar pattern of Hb increase in the OLE (**Fig**). Of the 55 pts treated with mitapivat who achieved the primary endpoint in the DBP, mean (SD) duration of Hb response was 17.9 (3.6) wks in the DBP and 43.6 (24.2) wks in the DBP+OLE, with ongoing responses up to 93.3 wks as of the cutoff. During the OLE, a total of 72/121 (59.5%) M/M pts had an OLE Hb response, including 23/68 (33.8%) who did not achieve a Hb response during the DBP. In the P/M group, 30/61 (49.2%) achieved an OLE Hb response.

### Summary/Conclusion

Long-term mitapivat treatment resulted in sustained, clinically meaningful Hb improvements in M/M pts receiving mitapivat for up to 127.3 wks. Hb improved in a similar pattern in P/M pts after switching to mitapivat, supporting a consistent treatment effect. An OLE Hb response was observed in ~60% of M/M pts, including ~1/3 of those who did not achieve a Hb response in the DBP, suggesting that some pts may benefit from continued mitapivat treatment regardless of initial Hb response.

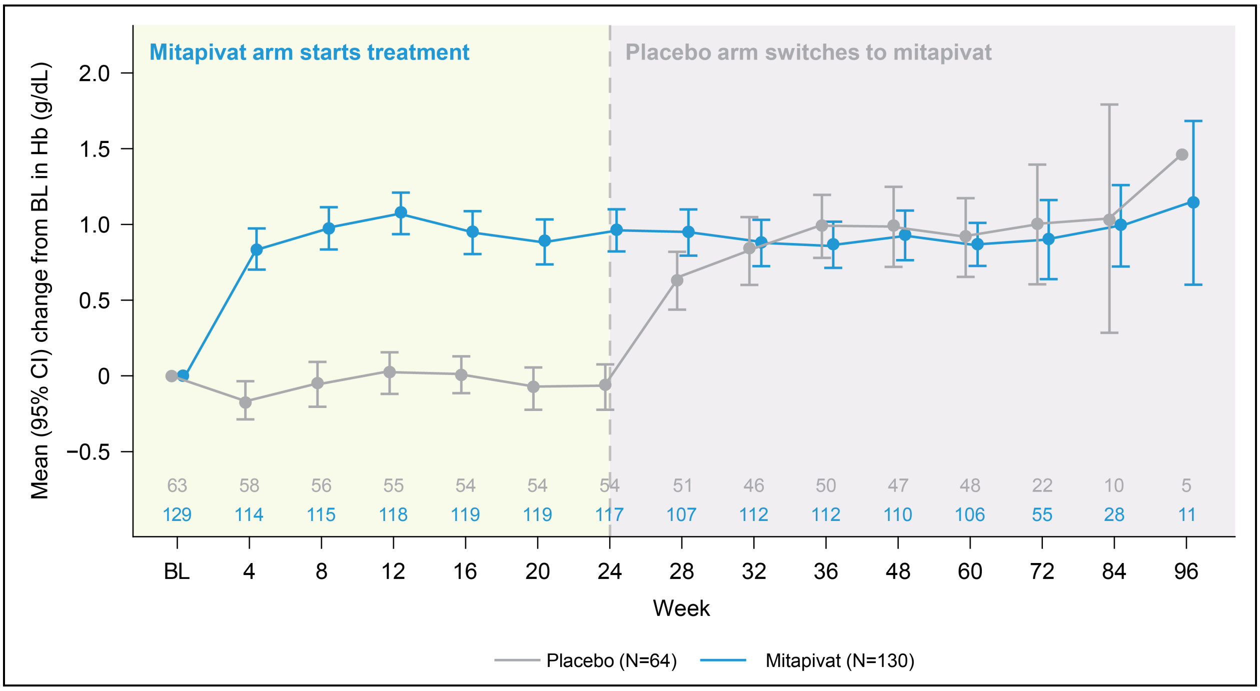

Fig. Change from baseline in Hb over time by treatment arm in the ENERGIZE DBP and OLE. BL, baseline; CI, confidence interval; DBP, double-blind period; Hb, hemoglobin; OLE, open-label extension

**Copyright Information:** (Online) ISSN: 2572-9241
©2026 The Author(s). HemaSphere is published by John Wiley & Sons Ltd on behalf of European Hematology Association. This is an open access Abstract Book distributed under the Attribution-NonCommercial-NoDerivs (CC BY-NC-ND), which allows third parties to download the articles and share them with others as long as they credit the author and the Abstract Book, but they cannot change the content in any way or use them commercially.
**Abstract Book Citations:** Authors, Title, HemaSphere, 2026;10:(S1):pages. The abstract book DOIs can be found on the “Author Information” page.
**Disclaimer:** Articles published in the journal HemaSphere exclusively reflect the opinions of the authors. The authors are responsible for all content in their abstracts including accuracy of the facts, statements, citing resources, etc.

### (S297) ETAVOPIVAT IMPROVES HEMOGLOBIN LEVELS IN ADULTS AND ADOLESCENTS WITH NON-TRANSFUSION-DEPENDENT ALPHA- OR BETA-THALASSEMIA: RESULTS FROM THE OPEN-LABEL PHASE 2 GLADIOLUS STUDY

**Topic:** 28. Thalassemias

Sujit Sheth*[1], Kevin H. M. Kuo[2, 3], Theodosia A. Kalfa[4], D. Mark Layton[5], Perla Eleftheriou[6], Anastasios Giakoumis[7], Olga Polyanskaya[8], Navid Nedjatian[9], Ashutosh Lal[10]

*[1]Weill Cornell Medicine, New York, New York, United States of America;[2]University of Toronto, Toronto, Ontario, Canada;[3]Scarborough Health Network Research Institute, Toronto, Ontario, Canada;[4]Cincinnati Children's Hospital Medical Center and University of Cincinnati College of Medicine, Cincinnati, OH, United States of America; [5]Hammersmith Hospital, Imperial College Healthcare NHS Trust, London, United Kingdom; [6]University College London Hospital, London, United Kingdom; [7]Novo Nordisk A/S, Bagsvaerd, Denmark; [8]Novo Nordisk Inc., Lexington, MA, United States of America;[9]Novo Nordisk Health Care AG, Zurich, Switzerland; [10]UCSF School of Medicine, San Francisco, CA, United States of America;*

### Background

People with non-transfusion-dependent thalassemia (NTDT) experience progressive morbidity from chronic anemia driven by ineffective erythropoiesis and hemolysis, leading to iron overload, organ damage, fatigue, and reduced quality of life. Etavopivat is an investigational, once-daily (QD), red blood cell (RBC) pyruvate kinase activator under evaluation as a disease-modifying therapy in people with sickle cell disease and thalassemia.

### Aims

Evaluate safety and clinical activity of etavopivat during the phase 2, multicenter, open-label gladiolus study (NCT04987489) in participants (ppts) with thalassemia not receiving chronic RBC transfusions (cohort C).

### Methods

Eligible cohort C ppts (aged 12–65 years) with thalassemia gave informed consent, had baseline (BL) hemoglobin (Hb) ≤10 g/dL, and did not have RBC transfusions within 8 weeks of enrollment, ≥4 RBC units or a transfusion-free period ≤35 days in the prior 6 months. Ppts received oral etavopivat 400 mg QD for the main (48-week [wk]) and extension period until rollover to FLORAL (NCT06609226) or discontinuation. The primary endpoint was Hb response rate (increase ≥1.0 g/dL from BL) at wk 12. Secondary endpoints included Hb response rates at wks 24 and 48, changes from BL in Hb and serum ferritin at wks 12, 24, and 48, and liver iron concentration at wk 48. An exploratory endpoint assessed PROMIS Fatigue Scale 7a (≥18 years) and 10a (<18 years). Additional analyses evaluated markers of hemolysis (absolute reticulocyte count, indirect bilirubin, lactate dehydrogenase), ineffective erythropoiesis (erythropoietin, erythroferrone, soluble transferrin receptor), and iron metabolism (hepcidin). Pharmacodynamic markers adenosine triphosphate (ATP) and 2,3-diphosphoglycerate (2,3-DPG) were analyzed. Safety was assessed over the main and extension period + 28 days.

**Copyright Information:** (Online) ISSN: 2572-9241
©2026 The Author(s). HemaSphere is published by John Wiley & Sons Ltd on behalf of European Hematology Association. This is an open access Abstract Book distributed under the Attribution-NonCommercial-NoDerivs (CC BY-NC-ND), which allows third parties to download the articles and share them with others as long as they credit the author and the Abstract Book, but they cannot change the content in any way or use them commercially.
**Abstract Book Citations:** Authors, Title, HemaSphere, 2026;10:(S1):pages. The abstract book DOIs can be found on the "Author Information" page.
**Disclaimer:** Articles published in the journal HemaSphere exclusively reflect the opinions of the authors. The authors are responsible for all content in their abstracts including accuracy of the facts, statements, citing resources, etc.

**Results**

Twenty ppts (15 female, 16 adults, 11 β-thalassemia [5 Hb E β-thalassemia], 9 Hb H thalassemia, 3 splenectomized) enrolled in cohort C. Mean (SD) age was 31.4 (14.77) years and BL Hb was 7.66 (0.929) g/dL. Four ppts discontinued etavopivat early.
Hb response was achieved in 50% of ppts (n/N=10/20) at wk 12, 39% at wk 24 (7/18), and 38% at wk 48 (6/16). Hb levels increased from wk 2 and were sustained through wk 48 (least squares mean changes [SE] from BL at wks 12, 24, and 48 were 1.12 [0.158] g/dL, 0.88 [0.166] g/dL, 0.91 [0.176] g/dL, respectively; all $p<0.0001$). Markers of ineffective erythropoiesis, hemolysis, and iron metabolism improved over time (**Table**). Early and sustained changes in ATP and 2,3-DPG supported etavopivat's mechanism of action. At BL, PROMIS Fatigue scores were worse than the general population (mean [SD] adults: 58.41 [9.158]; adolescents: 55.50 [12.633]) and improved in adults over the study; no change was observed in adolescents.
Etavopivat was well tolerated; safety findings were consistent with the underlying disease, with no new safety concerns. Most treatment-emergent adverse events (TEAEs) were mild/moderate; one TEAE (elevated alanine aminotransferase [ALT]) led to treatment discontinuation. Six serious AEs were reported; none considered drug related. Three ppts experienced Grade 2 (n=2) or 3 (n=1) ALT elevations; three experienced Grade 2 aspartate aminotransferase elevations.

**Summary/Conclusion**

These results from the gladiolus study demonstrated proof of concept for etavopivat, indicating favorable efficacy in people with NTDT, and no new safety findings. These findings support further investigation of etavopivat in thalassemia.

**Copyright Information:** (Online) ISSN: 2572-9241
©2026 The Author(s). HemaSphere is published by John Wiley & Sons Ltd on behalf of European Hematology Association. This is an open access Abstract Book distributed under the Attribution-NonCommercial-NoDerivs (CC BY-NC-ND), which allows third parties to download the articles and share them with others as long as they credit the author and the Abstract Book, but they cannot change the content in any way or use them commercially.
**Abstract Book Citations:** Authors, Title, HemaSphere, 2026;10:(S1):pages. The abstract book DOIs can be found on the "Author Information" page.
**Disclaimer:** Articles published in the journal HemaSphere exclusively reflect the opinions of the authors. The authors are responsible for all content in their abstracts including accuracy of the facts, statements, citing resources, etc.

**Table:** Summary of endpoints and analyses for cohort C of the gladiolus study.

| | **Week 12** (n=20) | **Week 24** (n=18) | **Week 48** (n=16) |
|---|---|---|---|
| **Primary endpoint** | | | |
| Ppts achieving Hb ≥1.0 g/dL increase at week 12, % (95% CI) | 50 (27.2, 72.8), p<0.0001 | - | - |
| **Secondary endpoints** | | | |
| Ppts achieving Hb ≥1.0 g/dL increase at week 24, % (95% CI) | - | 39 (17.3, 64.3), p=0.0012 | - |
| Ppts achieving Hb ≥1.0 g/dL increase at week 48, % (95% CI) | - | - | 38 (15.2, 64.6), p=0.0033 |
| CFB in Hb (g/dL) | 1.12 (0.158), p<0.0001 | 0.88 (0.166), p<0.0001 | 0.91 (0.176), p<0.0001 |
| CFB in serum ferritin (pmol/L) | 113.7 (72.25), p=0.12 | 18.6 (76.40), p=0.81 | –56.4 (77.14), p=0.47 |
| **Exploratory endpoints** | | | |
| CFB in PROMIS Fatigue score in adult ppts (n=16) | –5.57 (1.646) | –4.52 (1.718) | –5.45 (1.791) |
| CFB in PROMIS Fatigue score in adolescent ppts (n=4) | –6.31 (4.326) | –8.75 (5.023) | –3.89 (4.318) |
| **Pharmacodynamic endpoints** | | | |
| CFB in ATP (μg/mL), mean (SD) | 131.0 (56.91) | 119.6 (61.55) | 86.8 (71.46) |
| CFB in 2,3-DPG (μg/mL), mean (SD) | –112.9 (99.24) | –187.8 (175.08) | –179.0 (112.32) |
| **Additional analyses** | | | |
| Markers of hemolysis | | | |
| CFB in ARC ($10^9$/L) | –17.86 (20.985), p=0.40 | –36.37 (22.168), p=0.10 | –37.16 (24.439), p=0.13 |
| CFB in indirect bilirubin (μmol/L) | –4.93 (2.714), p=0.072 | –5.48 (2.825), p=0.055 | –3.14 (3.009), p=0.30 |
| CFB in LDH (IU/L) | –63.69 (25.331), p=0.013 | –81.16 (27.203), p=0.0034 | –75.88 (28.465), p=0.0088 |
| Markers of ineffective erythropoiesis | | | |
| CFB in erythropoietin (mIU/mL) | –60.13 (18.180), p=0.0021 | –40.69 (19.488), p=0.043 | –37.39 (20.366), p=0.074 |
| CFB in erythroferrone (ng/mL) | –8.367 (2.5757), p=0.0023 | –8.981 (2.7417), p=0.0022 | –12.829 (3.3267), p=0.0004 |
| CFB in sTfR (mg/L) | –1.255 (0.3195), p=0.0016 | –1.720 (0.2919), p<0.0001 | –2.316 (0.5781), p=0.0039 |
| Markers of iron metabolism | | | |
| CFB in hepcidin (ng/mL) | 3.41 (3.333), p=0.31 | 5.16 (3.536), p=0.15 | –0.39 (4.293), p=0.93 |

Table shows LS mean CFB (SE) unless otherwise indicated. BL was defined as the arithmetic mean of the Screening and Day 1 (pre-dose) results for all parameters, except for pharmacodynamic, ineffective erythropoiesis, and iron metabolism markers, where BL was defined as the most recent non-missing measurement prior to the first administration of the study drug. LS mean, SE, 95% CI, and p-value are based on an MMRM with CFB as the response and fixed covariates for BL value and nominal visit and a random effect for ppt. LS means reported for each visit are model-adjusted estimates based on the MMRM fitted to all available post-BL data up to the end of the extension treatment period. The variance–covariance matrix used an autoregressive structure of order 1 for parameter estimation, except for sTfR which used an unstructured structure. The proportion of ppts with an Hb response was compared with a 10% null rate using a one-sided exact binomial test ($\alpha$ = 0.025). P-values were not calculated for pharmacodynamic endpoints. 2,3-DPG, 2,3-diphosphoglycerate; ARC, absolute reticulocyte count; ATP, adenosine triphosphate; BL, baseline; CFB, change from baseline; CI, confidence interval; Hb, hemoglobin; LDH, lactate dehydrogenase; LS, least squares; MMRM, mixed model repeated measures; ppts, participants; SD, standard deviation; SE, standard error; sTfR, soluble transferrin receptor.

**Copyright Information:** (Online) ISSN: 2572-9241
©2026 The Author(s). HemaSphere is published by John Wiley & Sons Ltd on behalf of European Hematology Association. This is an open access Abstract Book distributed under the Attribution-NonCommercial-NoDerivs (CC BY-NC-ND), which allows third parties to download the articles and share them with others as long as they credit the author and the Abstract Book, but they cannot change the content in any way or use them commercially.
**Abstract Book Citations:** Authors, Title, HemaSphere, 2026;10:(S1):pages. The abstract book DOIs can be found on the “Author Information” page.
**Disclaimer:** Articles published in the journal HemaSphere exclusively reflect the opinions of the authors. The authors are responsible for all content in their abstracts including accuracy of the facts, statements, citing resources, etc.

### (S298) ATHEROSCLEROTIC RISK ASSESSMENT IN THE AGING BETA-THALASSEMIA POPULATION

**Topic:** 28. Thalassemias

Natalia Scaramellini*[1], Simona Stella[2], Cecilia Vismara[3], Lorena Duca[1], Francesca Granata[1], Valeria Di Stefano[1], Elena Cassinerio[1], Giovanna Graziadei[1], Matteo Colleoni[1, 3], Daniele Lello Panzieri[1, 3], Diletta Maira[1], Valentina Brancaleoni*[1], Simona Leoni[1, 4], Irene Motta[1, 4]

*[1]SC Medicina ad Indirizzo Metabolico, Fondazione IRCCS Ca' Granda Ospedale Maggiore Policlinico, Milano, Italy; [2]Occupational Health Unit,Fondazione IRCCS Ca' Granda Ospedale Maggiore Policlinico, Milano, Italy; [3]Università degli Studi di Milano, Milano, Italy; [4]Dipartimento di Scienze Cliniche e di Comunità, Dipartimento di Eccellenza 2023-2027, Università degli studi di Milano, Milano, Italy;*

### Background

In recent decades, survival among thalassemia patients has improved dramatically, leading to the emergence of age-related comorbidities. Heart disease remains the leading cause of death in these patients; however, the burden of atherosclerotic cardiovascular disease (ASCVD), the leading cause of death in the general population, remains unknown in this population

### Aims

This study aimed to estimate the prevalence of ASCVD and explore potential risk factors for ASCVD in patients with beta-thalassemia

### Methods

We collected clinical, laboratory, and radiological data from patients with beta-thalassemia regularly followed at our tertiary care centre in Milan, Italy, between January 2024 and June 2025

**Copyright Information:** (Online) ISSN: 2572-9241
©2026 The Author(s). HemaSphere is published by John Wiley & Sons Ltd on behalf of European Hematology Association. This is an open access Abstract Book distributed under the Attribution-NonCommercial-NoDerivs (CC BY-NC-ND), which allows third parties to download the articles and share them with others as long as they credit the author and the Abstract Book, but they cannot change the content in any way or use them commercially.
**Abstract Book Citations:** Authors, Title, HemaSphere, 2026;10:(S1):pages. The abstract book DOIs can be found on the "Author Information" page.
**Disclaimer:** Articles published in the journal HemaSphere exclusively reflect the opinions of the authors. The authors are responsible for all content in their abstracts including accuracy of the facts, statements, citing resources, etc.

**Results**

We enrolled 235 patients (median age 49; 58% female), including 153 transfusion-dependent thalassemia and 82 non-transfusion-dependent thalassemia. The median hemoglobin (Hb) level was 96 g/L, and the median serum ferritin was 571 µg/L. 103 patients were splenectomized. The prevalence of ASCVD was 15.7% (37/235, 95% CI 11.1-20.3%), and patients with ASCVD were older (Figure 1a). 32 patients had plaques in major arteries (carotid, aortic, and femoral), 4 had a brain ischemic lesion, and only 1 had experienced a myocardial infarction. Carotid Doppler ultrasound evaluation showed that intima-media thickness (IMT) increased with age. Interestingly, all our patients, including those with carotid plaques, have an IMT below 1 mm. When evaluating Hb levels, iron-related parameters, and transfusions, we found no differences between patients with and without ASCVD.  Also, no differences were observed between TDT and NTDT.
Considering known ASCVD risk factors, we observed an increased risk of developing ASCVD in those with alcohol intake (evaluated as more than 2 unit per week)(OR 3.57, 1.14-11.2, p 0.029), smoking history by 3.19 (OR 3.19, 1.20-8.50, p 0.020), glucose intolerance (OR 3.19, 1.04-0.76, p 0.041), and diabetes (OR 3.89, 1.34-11.34, p 0.013). Blood pressure was generally within the normal range; however, for each 1 mmHg increase in BP, the risk of ASCVD increases.
Overall, median levels of total cholesterol, high-density lipoproteins, low-density lipoproteins, and oxidized low-density lipoproteins—key factors in atherosclerosis—were all within the normal range, with only a limited proportion of subjects with levels above the upper limit of normality (Figure 1b). When comparing patients with and without ASCVD, there were no significant differences in lipoprotein levels. Similarly, no differences were observed in endothelial activation markers (VCAM-1, ICAM-1, p-selectin, e-selectin), although the profile is consistent with activated endothelium.
When considering disease-specific factors, such as iron overload, ferritin, and markers of ineffective erythropoiesis, we found no association with ASCVD

**Summary/Conclusion**

This is the first comprehensive study of ASCVD in aging patients with thalassemia. The role of known risk factors for ASCVD and the peculiar lipid profile highlight the need for special considerations: i) ASCVD and lifestyle factors should be carefully investigated; ii) developing disease-specific risk factors is crucial, since those from the European Society of Cardiology are not applicable because only a small proportion of patients meets the minimum cholesterol level for risk assessment; iii) metabolic perturbations in cholesterol synthesis and usage need to be explored, given the emerging link between adipose tissue and glucose metabolism with erythropoiesis

**Copyright Information:** (Online) ISSN: 2572-9241
©2026 The Author(s). HemaSphere is published by John Wiley & Sons Ltd on behalf of European Hematology Association. This is an open access Abstract Book distributed under the Attribution-NonCommercial-NoDerivs (CC BY-NC-ND), which allows third parties to download the articles and share them with others as long as they credit the author and the Abstract Book, but they cannot change the content in any way or use them commercially.
**Abstract Book Citations:** Authors, Title, HemaSphere, 2026;10:(S1):pages. The abstract book DOIs can be found on the “Author Information” page.
**Disclaimer:** Articles published in the journal HemaSphere exclusively reflect the opinions of the authors. The authors are responsible for all content in their abstracts including accuracy of the facts, statements, citing resources, etc.

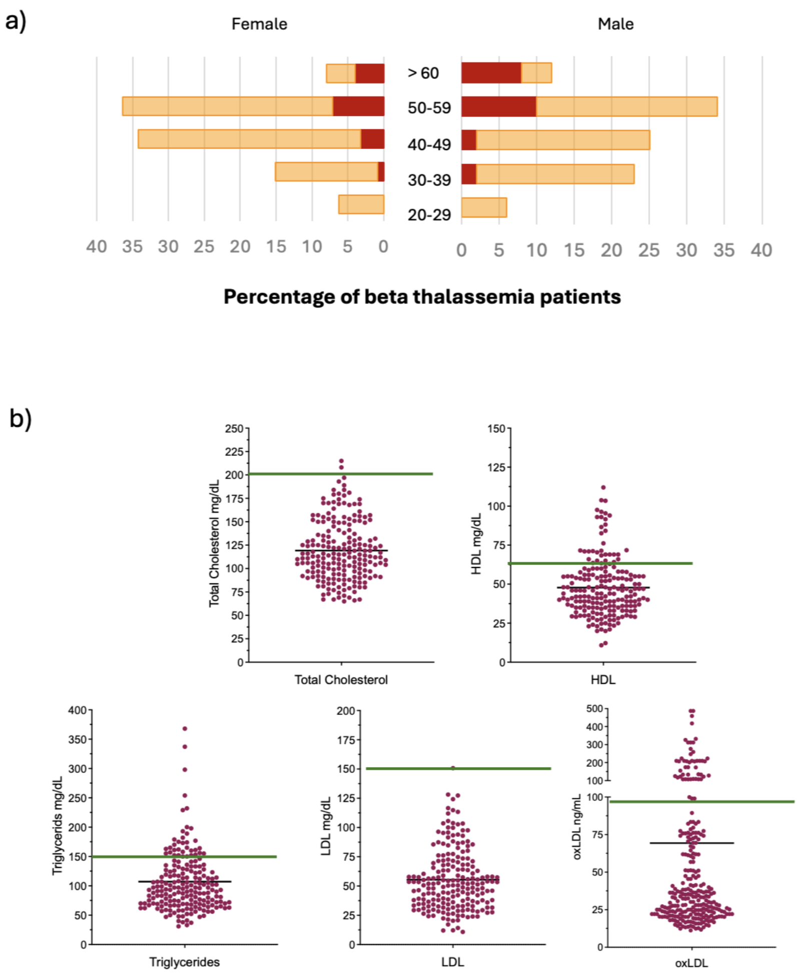

Fig 1
a) ASCVD prevalence in beta thalassemia patients, stratified by gender.
In red patients with ASCVD, in orange patients without ASCVD.
b) Lipid profile of beta thalassemia patients. HDL: high-density lipoproteins; LDL: low-density lipoproteins; oxLDL: oxidized low-density lipoproteins

**Copyright Information:** (Online) ISSN: 2572-9241
©2026 The Author(s). HemaSphere is published by John Wiley & Sons Ltd on behalf of European Hematology Association. This is an open access Abstract Book distributed under the Attribution-NonCommercial-NoDerivs (CC BY-NC-ND), which allows third parties to download the articles and share them with others as long as they credit the author and the Abstract Book, but they cannot change the content in any way or use them commercially.
**Abstract Book Citations:** Authors, Title, HemaSphere, 2026;10:(S1):pages. The abstract book DOIs can be found on the "Author Information" page.
**Disclaimer:** Articles published in the journal HemaSphere exclusively reflect the opinions of the authors. The authors are responsible for all content in their abstracts including accuracy of the facts, statements, citing resources, etc.

### (S299) ETAVOPIVAT REDUCES TRANSFUSION REQUIREMENTS IN ADULTS AND ADOLESCENTS WITH TRANSFUSION-DEPENDENT ALPHA- OR BETA-THALASSEMIA: RESULTS FROM THE OPEN-LABEL PHASE 2 GLADIOLUS STUDY

**Topic:** 28. Thalassemias

Ashutosh Lal*[1], Saranya Veluswamy[2], Melanie Kirby-Allen[3], Yves Dominique Pastore[4], Janet L. Kwiatkowski[5], E. Leila Jerome Clay[6], Olga Polyanskaya[7], Navid Nedjatian[8], Ali T. Taher[9]

*[1]UCSF School of Medicine, San Francisco, CA, United States of America;[2]Keck School of Medicine of USC, Children's Hospital Los Angeles, Los Angeles, CA, United States of America; [3]Hospital for Sick Children, Toronto, Canada;[4]CHU Sainte-Justine, Montréal, Canada; [5]Children's Hospital of Philadelphia, and Perelman School of Medicine, University of Pennsylvania, Philadelphia, PA, United States of America; [6]Novo Nordisk Inc., Princeton, NJ, United States of America;[7]Novo Nordisk Inc., Lexington, MA, United States of America; [8]Novo Nordisk Health Care AG, Zurich, Switzerland; [9]American University of Beirut Medical Center, Beirut, Lebanon;*

### Background

Transfusion-dependent thalassemia (TDT) imposes a lifelong burden on quality of life. Etavopivat is an investigational once-daily (QD), red blood cell (RBC) pyruvate kinase activator under evaluation as a disease-modifying therapy to improve RBC health in people with sickle cell disease and thalassemia.

### Aims

Evaluate safety and clinical activity of etavopivat during the phase 2, multicenter, open-label gladiolus study (NCT04987489) in participants with thalassemia on chronic RBC transfusions (cohort B).

### Methods

Eligible cohort B participants (aged 12–65) provided informed consent and had chronically transfused thalassemia (≥6 RBC units in the 24 weeks [wks] before first dose with no transfusion-free period >35 days). Participants received transfusions every 3–5 wks, with a target pre-transfusion hemoglobin (Hb) of ~9–10 g/dL. Oral etavopivat 400 mg QD was started ≤24 hours post-transfusion and continued for the main (48-wk) and extension period until rollover to FLORAL (NCT06609226) or discontinuation. If pre-transfusion Hb increased ≥1.0 g/dL vs baseline (BL), transfusions could be delayed by 1 wk or RBC units reduced at investigator discretion.
Primary and secondary erythroid response endpoints were proportion of participants achieving ≥20% or ≥33% reduction in RBC transfusion units over any continuous 12-wk interval during the 48 wks vs BL. Other secondary endpoints included relative reductions in RBC transfusions, change in serum ferritin at wks 12, 24, and 48, and liver iron concentration (LIC) at wk 48 vs BL. Exploratory endpoints included PROMIS Fatigue 7a (≥18 years) and 10a (<18 years). Pharmacodynamic markers (adenosine triphosphate [ATP] and 2,3-diphosphoglycerate [2,3-DPG]) were analyzed. Safety was assessed over the main and extension period + 28 days.

**Copyright Information:** (Online) ISSN: 2572-9241
©2026 The Author(s). HemaSphere is published by John Wiley & Sons Ltd on behalf of European Hematology Association. This is an open access Abstract Book distributed under the Attribution-NonCommercial-NoDerivs (CC BY-NC-ND), which allows third parties to download the articles and share them with others as long as they credit the author and the Abstract Book, but they cannot change the content in any way or use them commercially.
**Abstract Book Citations:** Authors, Title, HemaSphere, 2026;10:(S1):pages. The abstract book DOIs can be found on the "Author Information" page.
**Disclaimer:** Articles published in the journal HemaSphere exclusively reflect the opinions of the authors. The authors are responsible for all content in their abstracts including accuracy of the facts, statements, citing resources, etc.

**Results**

Twenty participants (12 male, 10 adolescents, 1 α-thalassemia) enrolled in cohort B. Mean (SD) age was 21.5 (9.57) years and mean (SD) RBC units transfused in the 24 wks before study start was 19.25 (4.778). Two participants discontinued etavopivat; one before 12 wks (ineligible for efficacy analysis).
Overall, 89% of participants (n/N=17/19) achieved a ≥20% reduction, and 79% of participants (15/19) achieved a ≥33% reduction in RBC units transfused over any continuous 12-wk period vs BL (**Figure**). Mean (SD) relative reduction from BL in RBC transfusion burden at wk 12 was 15.3% (20.97); wk 24: 11.9% (18.11); wk 48: 10.9% (15.77). Pre-transfusion Hb initially increased with etavopivat but returned to BL as transfusions were reduced per protocol. No significant changes from BL were observed in serum ferritin, LIC, or PROMIS Fatigue. Early and sustained changes in ATP and 2,3-DPG supported etavopivat's mechanism of action. Etavopivat was well tolerated; safety findings were consistent with the underlying disease, with no new safety concerns. Most treatment-emergent adverse events (TEAEs) were mild/moderate; one TEAE (bone pain) led to discontinuation. One serious allergic transfusion reaction was reported (considered not drug related). Three participants showed Grade 2 (n=2) or 3 (n=1) alanine aminotransferase elevations; one showed Grade 3 aspartate aminotransferase elevation.

**Summary/Conclusion**

Etavopivat reduced transfusion requirements in participants with TDT, providing proof of concept and demonstrating favorable efficacy, with no new safety concerns. These findings support further investigation of etavopivat in thalassemia.

**Copyright Information:** (Online) ISSN: 2572-9241
©2026 The Author(s). HemaSphere is published by John Wiley & Sons Ltd on behalf of European Hematology Association. This is an open access Abstract Book distributed under the Attribution-NonCommercial-NoDerivs (CC BY-NC-ND), which allows third parties to download the articles and share them with others as long as they credit the author and the Abstract Book, but they cannot change the content in any way or use them commercially.
**Abstract Book Citations:** Authors, Title, HemaSphere, 2026;10:(S1):pages. The abstract book DOIs can be found on the "Author Information" page.
**Disclaimer:** Articles published in the journal HemaSphere exclusively reflect the opinions of the authors. The authors are responsible for all content in their abstracts including accuracy of the facts, statements, citing resources, etc.

**Figure:** The proportion of participants achieving the primary and secondary erythroid response endpoints.

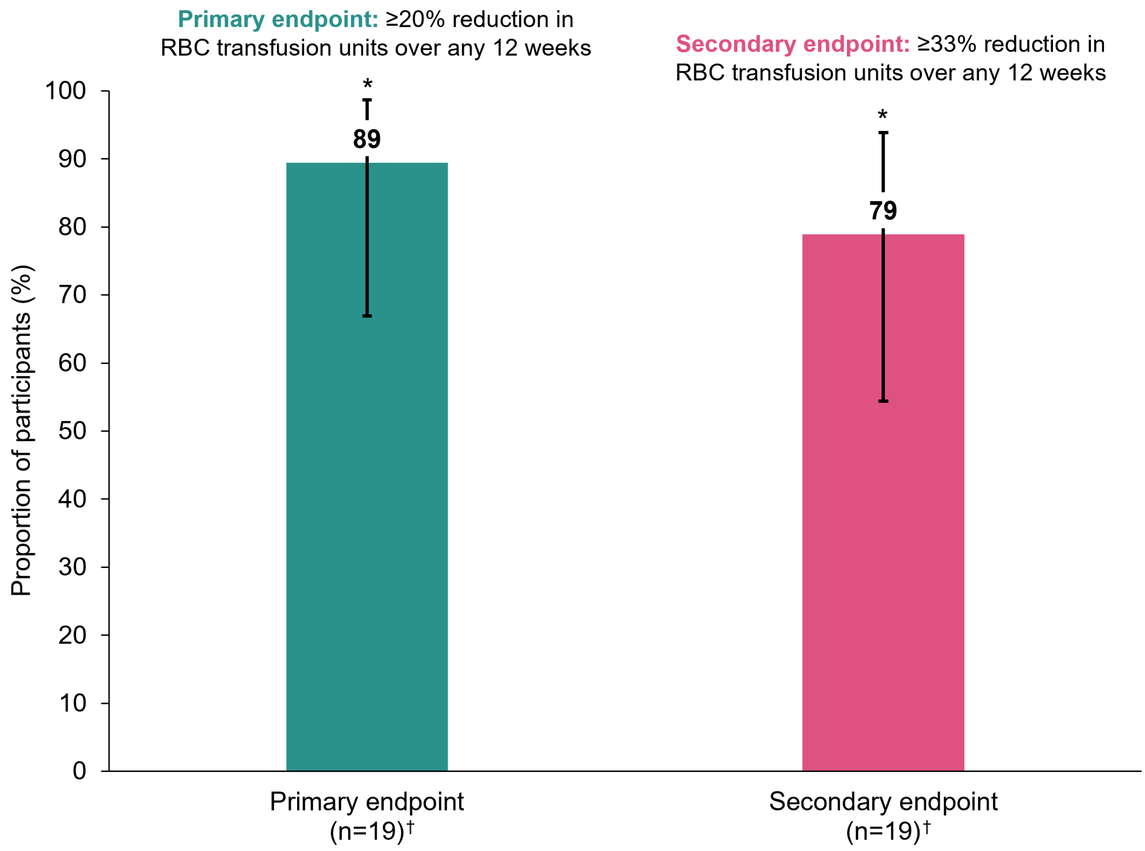

*p<0.0001 for both endpoints using an exact binomial test, testing a null response rate of ≤20% and using a one-sided significance level of 0.025. †One participant was withdrawn before week 12 and did not have 84 days of treatment in the study (ineligible for efficacy analysis). Error bars represent exact 95% confidence intervals for binomial proportions, calculated using the Clopper–Pearson method by inverting the exact binomial test. Responders were defined as any participant with ≥20% or ≥33% reduction from baseline in RBC units transfused during at least one qualifying 12-week (84-day) interval in the primary treatment period (i.e., Days 2 to 85, 3 to 86, ..., 253 to end of primary treatment period), where baseline was defined as the total number of RBC units transfused from Study Day –83 to Study Day 1. RBC, red blood cell.

**Copyright Information:** (Online) ISSN: 2572-9241
©2026 The Author(s). HemaSphere is published by John Wiley & Sons Ltd on behalf of European Hematology Association. This is an open access Abstract Book distributed under the Attribution-NonCommercial-NoDerivs (CC BY-NC-ND), which allows third parties to download the articles and share them with others as long as they credit the author and the Abstract Book, but they cannot change the content in any way or use them commercially.
**Abstract Book Citations:** Authors, Title, HemaSphere, 2026;10:(S1):pages. The abstract book DOIs can be found on the "Author Information" page.
**Disclaimer:** Articles published in the journal HemaSphere exclusively reflect the opinions of the authors. The authors are responsible for all content in their abstracts including accuracy of the facts, statements, citing resources, etc.

### (S300) NIPOCALIMAB FOR WARM AUTOIMMUNE HEMOLYTIC ANEMIA: RESULTS FROM THE PHASE 2/3 RANDOMIZED DOUBLE-BLIND ENERGY STUDY

**Topic:** 29. Enzymopathies, membranopathies and other red blood cell disorders

Bruno Fattizzo*[1, 2], Irina Murakhovskaya[3], Yasutaka Ueda[4], Douglas Schlichting[5], Kristen Sweet[5], Michael Zelasky[5], Jagriti Craig[5], Jocelyn Leu[5], Leona Ling[6], Sheryl Pease[7], Cathye Shu[5]

*[1]Hematology Unit, Fondazione IRCCS Ca' Granda Ospedale Maggiore Policlinico, Milan, Italy; [2]Department of Oncology and Hemato-Oncology, University of Milan, Milan, Italy; [3]Albert Einstein College of Medicine/Montefiore Medical Center, Bronx, NY, United States of America; [4]The University of Osaka, Suita, Osaka, Japan;[5]Johnson & Johnson, Spring House, PA, United States of America; [6]Johnson & Johnson, Cambridge, MA, United States of America; [7]Johnson & Johnson, Raritan, NJ, United States of America;*

### Background

Warm autoimmune hemolytic anemia (wAIHA) is a rare, life-threatening disease characterized by autoantibody-mediated premature destruction of red blood cells. No treatments are currently approved for wAIHA. Nipocalimab is an immunoselective neonatal Fc receptor blocker that reduces circulating IgG levels, including autoantibodies.

### Aims

The phase 2/3, 24-week, double-blind ENERGY trial (ClinicalTrials.gov identifier: NCT04119050) evaluated the efficacy and safety of intravenous nipocalimab in wAIHA.

### Methods

Adults with wAIHA (hemoglobin [Hgb] <10 g/dL, laboratory evidence of hemolysis, positive direct antiglobulin test) who received ≥1 prior therapy and consented to participate were randomized 1:1:1 to nipocalimab 30 mg/kg q4w, 15 mg/kg q2w, or placebo. Concomitant stable corticosteroids or immunosuppressants were allowed. Endpoints included durable Hgb response (primary; Hgb ≥10 g/dL and ≥2 g/dL increase from baseline at ≥3 consecutive visits [≥28 days] starting by Week 16 [W16], without rescue therapy or new/increased steroids/immunosuppressants), mean change from baseline in FACIT-Fatigue at W24, and % reduction in corticosteroid dose at W24 (mandatory taper for Hgb responders only). Multiplicity was controlled via an equal weight analysis with one-sided $\alpha$=0.02499; *P*-values were one-sided. Other endpoints included measures of hemolysis and safety.

**Copyright Information:** (Online) ISSN: 2572-9241
©2026 The Author(s). HemaSphere is published by John Wiley & Sons Ltd on behalf of European Hematology Association. This is an open access Abstract Book distributed under the Attribution-NonCommercial-NoDerivs (CC BY-NC-ND), which allows third parties to download the articles and share them with others as long as they credit the author and the Abstract Book, but they cannot change the content in any way or use them commercially.
**Abstract Book Citations:** Authors, Title, HemaSphere, 2026;10:(S1):pages. The abstract book DOIs can be found on the "Author Information" page.
**Disclaimer:** Articles published in the journal HemaSphere exclusively reflect the opinions of the authors. The authors are responsible for all content in their abstracts including accuracy of the facts, statements, citing resources, etc.

**Results**

A total of 115 participants were randomized to nipocalimab 30 mg/kg q4w (n=38), 15 mg/kg q2w (n=38) or placebo (n=39); at baseline, median (IQR) time from diagnosis was 2.7 (1.7-5.0), 3.7 (1.7-6.8) and 1.9 (1.4-5.9) years and concomitant corticosteroids were used by 89%, 86% and 87%, respectively. Median (IQR) age was 57 (41-72); 46% of participants were White, 55% were female, and 87% had primary wAIHA.
Durable Hgb response was achieved by 24% (9/38) on nipocalimab 30 mg/kg q4w ($P$=0.015) and 21% (8/38) on 15 mg/kg q2w ($P$=0.044) versus 8% (3/39) on placebo. Mean Hgb increased by 1.1 g/dL at W1 on nipocalimab 30 mg/kg q4w and 0.7 g/dL on 15 mg/kg q2w versus –0.1 g/dL on placebo (**Figure**). Hgb level ≥10 g/dL and increase of ≥2 g/dL at ≥1 visit was achieved by 61% (23/38) on nipocalimab 30 mg/kg q4w (nominal $P$<0.001) and 42% (16/38) on 15 mg/kg q2w (nominal $P$=0.004) versus 15% (6/39) on placebo.
FACIT-Fatigue improvement was observed from W2 to W24; at W24 (key secondary endpoint), mean (SD) improvement was 3.4 (7.3) for nipocalimab 30 mg/kg q4w (nominal $P$=0.007) and 1.2 (6.1) for 15 mg/kg q2w (nominal $P$=0.267) versus 0.6 (3.4) for placebo. The mean (SD) reduction in corticosteroid dose at W24 was 15% (28) for nipocalimab 30 mg/kg q4w (nominal $P$=0.039) and 14% (30) for 15 mg/kg q2w (nominal $P$=0.055) versus 4% (16) for placebo.
Safety findings were consistent with nipocalimab's known safety profile and wAIHA-associated risks. The proportion of patients with ≥1 adverse event (AE) was similar across groups (81%-92%); common AEs (≥10% of patients on nipocalimab) included wAIHA, fatigue, diarrhea, asthenia, dizziness, headache, peripheral edema and pyrexia. Grade ≥3 infections were less frequent with nipocalimab 30 mg/kg q4w (5%) and 15 mg/kg q2w (8%) versus placebo (13%). Two deaths, both deemed not related to study drug, occurred in the nipocalimab 15 mg/kg q2w group.

**Summary/Conclusion**

In wAIHA, nipocalimab 30 mg/kg q4w demonstrated rapid and durable efficacy in achieving the strict primary Hgb response with improvements in fatigue observed as early as W2, which was maintained over 24 weeks, with a favorable safety profile.

**Copyright Information:** (Online) ISSN: 2572-9241
©2026 The Author(s). HemaSphere is published by John Wiley & Sons Ltd on behalf of European Hematology Association. This is an open access Abstract Book distributed under the Attribution-NonCommercial-NoDerivs (CC BY-NC-ND), which allows third parties to download the articles and share them with others as long as they credit the author and the Abstract Book, but they cannot change the content in any way or use them commercially.
**Abstract Book Citations:** Authors, Title, HemaSphere, 2026;10:(S1):pages. The abstract book DOIs can be found on the "Author Information" page.
**Disclaimer:** Articles published in the journal HemaSphere exclusively reflect the opinions of the authors. The authors are responsible for all content in their abstracts including accuracy of the facts, statements, citing resources, etc.

**Figure. (A) Mean Change From Baseline in Hgb and (B) Mean Hgb by Visit Through W24**

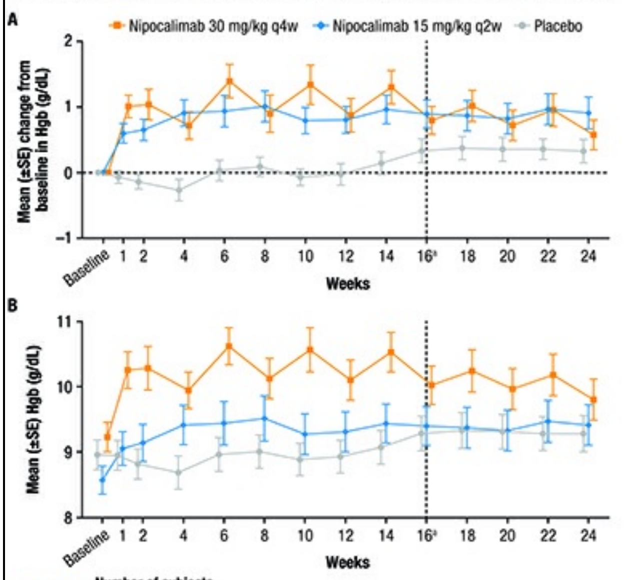

Number of subjects

| | | | | | | | | | | | | | | |
|---|---|---|---|---|---|---|---|---|---|---|---|---|---|---|
| Nipocalimab 30 mg/kg q4w | 38 | 37 | 37 | 38 | 38 | 38 | 38 | 38 | 38 | 38 | 38 | 37 | 38 | 38 |
| Nipocalimab 15 mg/kg q2w | 38 | 34 | 36 | 37 | 37 | 37 | 36 | 37 | 36 | 37 | 37 | 37 | 37 | 37 |
| Placebo | 39 | 35 | 39 | 38 | 38 | 38 | 39 | 39 | 38 | 38 | 39 | 39 | 38 | 39 |

Hgb, hemoglobin; q2w, every 2 weeks; q4w, every 4 weeks; W, week.
[a]To meet the primary endpoint, the first of 3 consecutive visits satisfying the Hgb response definition needed to occur by W16. The primary endpoint analysis was based on a composite strategy where participants with intercurrent events (including any use of rescue therapies or initiation or dose increase of protocol-specified standard-of-care background therapy) were considered nonresponders, with no change from baseline.

**Copyright Information:** (Online) ISSN: 2572-9241
©2026 The Author(s). HemaSphere is published by John Wiley & Sons Ltd on behalf of European Hematology Association. This is an open access Abstract Book distributed under the Attribution-NonCommercial-NoDerivs (CC BY-NC-ND), which allows third parties to download the articles and share them with others as long as they credit the author and the Abstract Book, but they cannot change the content in any way or use them commercially.
**Abstract Book Citations:** Authors, Title, HemaSphere, 2026;10:(S1):pages. The abstract book DOIs can be found on the "Author Information" page.
**Disclaimer:** Articles published in the journal HemaSphere exclusively reflect the opinions of the authors. The authors are responsible for all content in their abstracts including accuracy of the facts, statements, citing resources, etc.

### (S301) A RANDOMIZED, DOUBLE-BLIND, PLACEBO-CONTROLLED PHASE 3 STUDY OF ESLIM-02 FOR EFFICACY AND SAFETY OF SOVLEPLENIB (HMPL-523) IN PATIENTS WITH WARM AUTOIMMUNE HEMOLYTIC ANEMIA IN CHINA

**Topic:** 29. Enzymopathies, membranopathies and other red blood cell disorders

Fengkui Zhang[1], Bing Han*[2], Liansheng Zhang[3], Xin Zhao[1], Miao Chen[2], Zhihua Zhang[4], Hu Zhou[5], Yan Li[6], Yingmei Li[7], Na Gao[8], Guangsheng He[9], Hongyan Tong[10], Xinquan Liang[11], Jing Sun[12], Chenghao Jin[13], Haiping Yang[14], Guolin Yuan[15], Honghua He[16], Lie Lin[17], Fei Li[18], Yi Wang[19], Songfu Jiang[20], Xiaoxia Chu[21], Tiejun Gong[22], Lijuan Li[3], Xiaoming Li[23], Xin Li[24], Yajing Xu[25], Mei Hong[26], Xin Du[27], Zeping Zhou[28], Qingyi Wang[29], Yunfeng Cheng[30], Wenqian Li[31], Yanjuan Lin[32], Zhimei Wu[33], Hong Liu[34], Xinrong Zhan[35], Xuliang Shen[36], Jun Wang[37], Hua Xue[38], Lei Lei[39], Luguang MA[39], Qian Xu[39], Hongyan Yin[39], Songhua Fan[39], Guangxiu Dai[39]

*[1]National Clinical Research Center for Blood Diseases, Institute of Hematology and Blood Diseases Hospital, Chinese Academy of Medical Sciences, Tianjin, China; [2]State Key Laboratory for Complex, Severe, and Rare Diseases, Peking Union Medical College Hospital, Beijing, China; [3]The Second Hospital of Lanzhou University, Lanzhou, China; [4]Affiliated Hospital of Chengde Medical University, Chengde, China; [5]Henan Cancer Hospital/ The Affiliated Cancer Hospital of Zhengzhou University, Zhengzhou, China; [6]People's Hospital of Xinjiang Uygur Autonomous Region, Urumqi, China; [7]The First Affiliated Hospital of Zhengzhou University, Zhengzhou, China; [8]Binzhou Medical University Hospital, Binzhou, China; [9]The First Affiliated Hospital of Nanjing Medical University, Jiangsu Province Hospital, Nanjing, China; [10]The First Affiliated Hospital, Zhejiang University School of Medicine, Hangzhou, China; [11]Chenzhou First People's Hospital, Chenzhou, China; [12]Nanfang Hospital, Southern Medical University, Guangzhou, China; [13]Jiangxi Provincial People's Hospital, Nanchang, China; [14]The First Affiliated Hospital, and College of Clinical Medicine of Henan University of Science and Technology, Luoyang, China; [15]Xiangyang Central Hospital, Xiangyang, China; [16]Affiliated Hospital of Guangdong Medical University, Zhanjiang, China; [17]Hainan General Hospital, Haikou, China; [18]Jiangxi Clinical Research Center for Hematologic Disease, Jiangxi Provincial Key Laboratory of Hematological Diseases, The First Affiliated Hospital, Jiangxi Medical College, Nanchang, China; [19]Shaanxi Provincial People's Hospital, Xi'an, China; [20]The First Affiliated Hospital of Wenzhou Medical University, Wenzhou, China; [21]Yantai Yuhuangding Hospital, Yantai, China; [22]Harbin the Frist Hospital, Harbin, China; [23]The Affiliated Hospital of Southwest Medical University, Luzhou, China; [24]The Third Xiangya Hospital of Central South University, Changsha, China; [25]Xiangya Hospital of Central South University, Changsha, China; [26]Union Hospital, Tongji Medical College, Huazhong University of Science and Technology, Wuhan, China; [27]Shenzhen Second People's Hospital, Shenzhen, China; [28]Second Affiliated Hospital of Kunming Medical University, Kunming, China; [29]The Fifth Affiliated Hospital of Anhui Medical University, Fuyang, China; [30]Jinshan Hospital of Fudan University, Shanghai, China; [31]Qinghai Provincial People's Hospital, Xining, China; [32]Fujian Medical University Union Hospital, Fuzhou, China; [33]The First Affiliated Hospital of Guilin Medical University, Guilin, China; [34]Affiliated Hospital of Nantong University, Nantong, China; [35]Xinxiang Central Hospital, Xinxiang, China; [36]Heping Hospital Affiliated to Changzhi Medical College, Changzhi, China; [37]The First People's Hospital of Changde City, Changde, China; [38]Affiliated Hospital of Hebei University, Baoding, China; [39]HUTCHMED, Shanghai, China;*

### Background

Sovleplenib, a novel spleen tyrosine kinase (SYK) inhibitor, has showed promising hemoglobin (Hb) response benefits in Chinese patients (pts) with warm autoimmune hemolytic anemia (wAIHA) in the phase 2 part (Zhao X. et al. Lancet Haematol 2025) of the phase 2/3 study (NCT05535933).

### Aims

The double-blind randomized, placebo-controlled phase 3 ESLIM-02 study aimed to evaluate the efficacy and safety of sovleplenib in wAIHA pts in China.

**Copyright Information:** (Online) ISSN: 2572-9241
©2026 The Author(s). HemaSphere is published by John Wiley & Sons Ltd on behalf of European Hematology Association. This is an open access Abstract Book distributed under the Attribution-NonCommercial-NoDerivs (CC BY-NC-ND), which allows third parties to download the articles and share them with others as long as they credit the author and the Abstract Book, but they cannot change the content in any way or use them commercially.
**Abstract Book Citations:** Authors, Title, HemaSphere, 2026;10:(S1):pages. The abstract book DOIs can be found on the "Author Information" page.
**Disclaimer:** Articles published in the journal HemaSphere exclusively reflect the opinions of the authors. The authors are responsible for all content in their abstracts including accuracy of the facts, statements, citing resources, etc.

### Methods

Eligible pts were those who aged 18–75 yrs with primary or secondary wAIHA, insufficient response to previous glucocorticoid treatment, ECOG PS 0–2, Hb <100 g/L and positive direct antiglobulin test at baseline.
Pts were randomized 1:1 to receive sovleplenib or placebo, 300 mg once daily for 24 weeks (wks). Upon completion of 24-wk double-blind treatment and 4-wk safety follow-up, or completing 20-wk double-blind treatment without any response (Hb <100 g/L or with an increase of < 20 g/L from baseline at all visits within 20 wks), eligible pts could enter an open-label extension phase to receive sovleplenib.
The primary endpoint was durable response rate (Hb ≥100 g/L with an increase of ≥20 g/L from baseline in 3 consecutive available evaluations with an interval of at least 7 days, not affected by rescue therapy) during weeks 5–24 of double-blind period.

### Results

From 22 Feb 2024 to 10 Jun 2025, 90 pts (44 vs 46) were randomized to receive sovleplenib or placebo. Baseline demographics and disease characteristics were generally balanced between two groups. The median prior line of anti-wAIHA treatments was both 3.0, and 36% vs 41% of pts have received rituximab.
The durable response rate during weeks 5–24 was significantly higher with sovleplenib than with placebo (66% vs 15%,$p$<0.0001). Consistent results were observed in all sensitivity analyses, and all subgroup analyses supported the findings of the primary endpoint. In pts received prior rituximab, durable response rate also favored sovleplenib over placebo (69% vs 16%, $p$=0.0022).
During the 24-wk double-blind treatment period, compared with placebo, the overall response rate (Hb ≥100 g/L with an increase of ≥20 g/L from baseline, not affected by rescue therapy) was significantly increased (70% vs 22%, $p$<0.0001), the use of protocol-defined rescue therapy was significantly reduced (16% vs 54%, $p$=0.0001) with sovleplenib, and fewer pts received red blood cell transfusion (11% vs 43%). Median time to response was 3.1 vs 6.3 wks, with a median cumulative duration of response of 16.1 vs 6.1 wks among overall responders. An improvement in hemolytic markers was observed with sovleplenib compared with placebo, showing an alleviation of active hemolysis.
The median treatment duration was 24 vs 20 wks with sovleplenib vs placebo, with 33 (75%) vs 11 (24%) pts receiving treatment for ≥24 wks. Grade ≥3 treatment-emergent adverse events (TEAEs) were reported in 19 (43%) pts with sovleplenib and 27 (59%) pts with placebo. The most common (≥10%) Grade ≥3 TEAEs were warm autoimmune hemolytic anemia (18% vs 43%) and upper respiratory tract infection (2% vs 11%). No TEAE-related discontinuations or deaths occurred with sovleplenib.

### Summary/Conclusion

Sovleplenib demonstrated significantly and clinically meaningful durable Hb response with a favorable safety profile. Sovleplenib could be a breakthrough therapy for pts with wAIHA who have insufficient response to previous glucocorticoid treatment.

**Copyright Information:** (Online) ISSN: 2572-9241
©2026 The Author(s). HemaSphere is published by John Wiley & Sons Ltd on behalf of European Hematology Association. This is an open access Abstract Book distributed under the Attribution-NonCommercial-NoDerivs (CC BY-NC-ND), which allows third parties to download the articles and share them with others as long as they credit the author and the Abstract Book, but they cannot change the content in any way or use them commercially.
**Abstract Book Citations:** Authors, Title, HemaSphere, 2026;10:(S1):pages. The abstract book DOIs can be found on the “Author Information” page.
**Disclaimer:** Articles published in the journal HemaSphere exclusively reflect the opinions of the authors. The authors are responsible for all content in their abstracts including accuracy of the facts, statements, citing resources, etc.

### (S302) REAL-WORLD EXPERIENCE OF MITAPIVAT IN PYRUVATE KINASE DEFICIENCY

**Topic:** 29. Enzymopathies, membranopathies and other red blood cell disorders

Giacinto Luca Pedone*[1, 2], Paolo Ricchi[3], Michela Ribersani[4], Francesco Arcioni[5], Teresa Ceglie[6], Filippo Mazzi[7], Antonella Sau[8], Maria Pia MD[9], Caterina Giovanna Valentini[10], Anna Paola Marcello[1], Paola Bianchi[1], Marta Bortolotti[1], Francesco Passamonti[1], Wilma Barcellini[1], Bruno Fattizzo[1, 11]

*[1]Fondazione IRCCS Ca' Granda Ospedale Maggiore Policlinico, Hematology Unit, Milan, Italy; [2]University of Milan, Department of Pathophysiology and Transplantation, Milan, Italy; [3]AORN Cardarelli, UOSD Malattie Rare Del Globulo Rosso, Napoli, Italy; [4]Policlinico Umberto I University Hospital, Hematology Unit, Milan, Italy; [5]Perugia Hospital, Pediatric Onco-Hematology Unit With Bone Marrow Transplantation, Perugia, Italy; [6]San Luigi Gonzaga Hospital, SSD Microcitemie e Malattie Rare Ematologiche, Orbassano, Italy; [7]Azienda Ospedaliera Universitaria Integrata Verona, Medicina Generale B, Verona, Italy; [8]Pescara Hospital, Pescara, Italy, Hematology Unit, Pescara, Italy; [9]A. Perrino Hospital, Brindisi, Italy, Hematology Unit, Brindisi, Italy; [10]Fondazione Policlinico Universitario A. Gemelli IRCCS, Dipartimento di Scienze di Laboratorio ed Ematologiche, Rome, Italy; [11]University of Milan, Department of Oncology and Hemato-oncology, Milan, Italy;*

**Background**

Pyruvate kinase deficiency (PKD) is a rare congenital hemolytic anemia characterized by chronic hemolysis, ineffective erythropoiesis, and serious long-term complications. Mitapivat, an oral allosteric activator of PK, is the first disease-modifying therapy approved for PKD. Its efficacy was proven in the DRIVE-PK, ACTIVATE, and ACTIVATE-T trials, but data in heterogeneous populations with complex comorbidities or rare genotypes remain limited.

**Aims**

This study reports the first multicenter real-world analysis of mitapivat in adult patients with PKD treated in Italy.

**Methods**

A retrospective multicenter analysis was conducted across 9 Italian hematology centers (compassionate use or post-trial access). Adult transfusion-dependent (TD) and non-transfusion-dependent (NTD) patients received mitapivat titrated from 5 mg BID to 20 mg or 50 mg BID. Response was defined as sustained hemoglobin (Hb) increase ≥1.5 g/dL from baseline at ≥2 assessments or as ≥33% reduction in red blood cell transfusion burden. Hemolytic markers, iron metabolism, and safety profile were evaluated.

**Copyright Information:** (Online) ISSN: 2572-9241
©2026 The Author(s). HemaSphere is published by John Wiley & Sons Ltd on behalf of European Hematology Association. This is an open access Abstract Book distributed under the Attribution-NonCommercial-NoDerivs (CC BY-NC-ND), which allows third parties to download the articles and share them with others as long as they credit the author and the Abstract Book, but they cannot change the content in any way or use them commercially.
**Abstract Book Citations:** Authors, Title, HemaSphere, 2026;10:(S1):pages. The abstract book DOIs can be found on the "Author Information" page.
**Disclaimer:** Articles published in the journal HemaSphere exclusively reflect the opinions of the authors. The authors are responsible for all content in their abstracts including accuracy of the facts, statements, citing resources, etc.

**Results**

Thirty adults were included (16 males, 14 females), median age 45 years. Ten (33%) were TD; 20 (67%) had prior splenectomy. Genotypes were biallelic missense (65%), missense/non-missense (12%), or biallelic non-missense (23%).
After a median follow-up of 19 months, the overall response rate was 67% (20/30). Among responders, 18 (60%) achieved the Hb endpoint. In NTD responders, the median Hb improved significantly from 9.45 g/dL (IQR 8.6–10.3) at baseline to 11.1 g/dL (IQR 10.4–12.7) post-treatment ($p<0.0001$), with a median individual sustained Hb increase of 2.2 g/dL. In the TD cohort (n=10), 7 patients (70%) achieved a significant reduction in transfusion burden, with 6 (60%) achieving complete transfusion independence. Hemolysis parameters improved: median reticulocytes decreased significantly from 229 to 133×$10^9$/L ($p<0.001$), LDH from 233 to 201 U/L ($p=0.013$), and indirect bilirubin from 2.6 mg/dL to 0.82 mg/dL ($p<0.0001$). Haptoglobin increased from a median of 10 mg/dL to 38 (16–90) mg/dL.
Notably, responses occurred within 3 months of therapy in 14 (78%) cases, although most patients (21, 70%) were titrated to the maximum dose of 50 mg BID in an attempt to achieve normal Hb values, which were obtained in 8 (40%) cases. Response rates were 82% in patients with biallelic missense mutations, 33% in those with missense/non-missense genotypes, and 17% in those with biallelic non-missense mutations ($p=0.005$). Conversely, baseline peripheral blood PK enzymatic activity did not differ significantly between responders and non-responders ($p=0.44$). Efficacy was independent of prior splenectomy ($p=0.7$). The safety profile was consistent with clinical trials, with manageable lipid (LDL and total cholesterol) increases observed in 20% of patients. Two unintended pregnancies occurred during treatment, which required interruption.

**Summary/Conclusion**

Mitapivat showed high effectiveness and acceptable safety in real-world Italian adults with PKD, in real-world practice, offering a functional cure for a significant proportion of TD patients and significantly ameliorating anemia in NTD ones. Genotype was the main determinant of response. Further analyses on genotype-phenotype correlations and long-term iron remodeling are ongoing.

**Copyright Information:** (Online) ISSN: 2572-9241
©2026 The Author(s). HemaSphere is published by John Wiley & Sons Ltd on behalf of European Hematology Association. This is an open access Abstract Book distributed under the Attribution-NonCommercial-NoDerivs (CC BY-NC-ND), which allows third parties to download the articles and share them with others as long as they credit the author and the Abstract Book, but they cannot change the content in any way or use them commercially.
**Abstract Book Citations:** Authors, Title, HemaSphere, 2026;10:(S1):pages. The abstract book DOIs can be found on the "Author Information" page.
**Disclaimer:** Articles published in the journal HemaSphere exclusively reflect the opinions of the authors. The authors are responsible for all content in their abstracts including accuracy of the facts, statements, citing resources, etc.

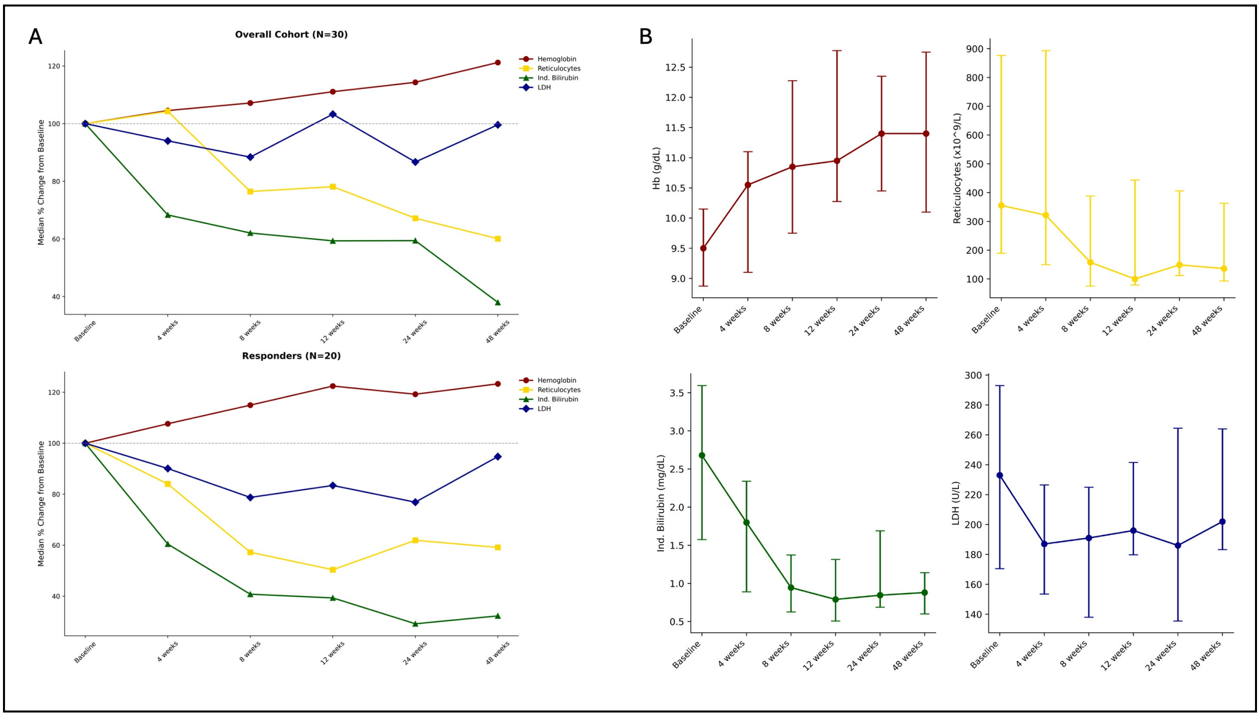

Figure 1 - A: Median percentage change from baseline of hematologic parameters in the overall cohort and in responders to mitapivat. B: Median and IQR trends of hematologic parameters in responders to mitapivat.

**Copyright Information:** (Online) ISSN: 2572-9241
©2026 The Author(s). HemaSphere is published by John Wiley & Sons Ltd on behalf of European Hematology Association. This is an open access Abstract Book distributed under the Attribution-NonCommercial-NoDerivs (CC BY-NC-ND), which allows third parties to download the articles and share them with others as long as they credit the author and the Abstract Book, but they cannot change the content in any way or use them commercially.
**Abstract Book Citations:** Authors, Title, HemaSphere, 2026;10:(S1):pages. The abstract book DOIs can be found on the "Author Information" page.
**Disclaimer:** Articles published in the journal HemaSphere exclusively reflect the opinions of the authors. The authors are responsible for all content in their abstracts including accuracy of the facts, statements, citing resources, etc.

### (S303) VENGLUSTAT DEMONSTRATES SUPERIOR NEUROLOGIC EFFICACY WHILE MAINTAINING SYSTEMIC CONTROL IN GAUCHER DISEASE TYPE 3: RESULTS FROM THE PHASE 3 LEAP2MONO TRIAL

**Topic:** 29. Enzymopathies, membranopathies and other red blood cell disorders

Eugen Mengel[1], Irene Motta*[2], Fatma Bulut[3], Cheryl Rockman-Greenberg[4], Anais Brassier[5], Zhengqing Qiu[6], Dmitry Cherkasov[7], Kristina An Haack[8], Gabriela Perichon[9], Atef Zaher[10], Qi Zhang[11], Pramod Mistry[12]

*[1]Institute of Clinical Science for Lysosomal Storage Disorders, SphinCS GmbH, Hochheim, Germany;[2]Unit of Medicine and Metabolic Disease, Fondazione IRCCS Ca' Granda Ospedale Maggiore Policlinico, 20122 Milan, Italy; [3]Cukurova University, Pediatric Metabolism and Nutrition Department, Adana, Türkiye; [4]Department of Pediatrics and Child Health, Rady Faculty of Health Sciences, University of Manitoba, Winnipeg, MB, Canada; [5]Reference Center for Inherited Metabolic Diseases,Necker—Enfants Malades University Hospital, Assistance Publique—Hôpitaux de Paris (AP-HP), Paris- Cité University, Paris, France; [6]Peking Union Medical College Hospital, Beijing, China; [7]Sanofi, Chilly-Mazarin, Île-de-France, France; [8]Sanofi, Gentilly, Île-de-France, France; [9]Sanofi, Cambridge, MA, United States of America; [10]Sanofi, Toronto, ON, Canada; [11]Sanofi, Morristown, NJ, United States of America; [12]Yale University School of Medicine, New Haven, CT, United States of America;*

### Background

Type 3 Gaucher disease (GD3) is a rare, chronic neuronopathic lysosomal disease characterized by multisystem involvement and progressive neurodegeneration. Enzyme replacement therapy (ERT) improves hematologic, visceral, and bone manifestations but has minimal central nervous system (CNS) penetration, leading to progressive neurological disease. Venglustat (VEN), an oral, brain-penetrant glucosylceramide synthase inhibitor, has shown potential to treat CNS manifestations of GD3.

### Aims

To evaluate efficacy and safety of oral VEN vs intravenous imiglucerase (IMI) in pediatricand adult patients with GD3.

### Methods

LEAP2MONO (NCT05222906), a Phase 3, randomized, double-blind, double-dummy, active-comparator, multinational, multicenter trial comparing oral VEN to intravenous IMI in pediatrics (≥12 to <18 years) and adults with GD3 stable on long-term ERT (≥3 years) and met predefined therapeutic goals. Eligibility criteria included Scale for Assessment and Rating of Ataxia (SARA) score ≥1 and presence of gaze palsy with slow/absent horizontal saccades. Participants (pts, n=43) were randomized 1:1 to receive 52 weeks (Wk) of either VEN (15mg adults; 12/15mg adolescents) with placebo infusions, or IMI at pre-trial dose with placebo tablets. The primary objective was to evaluate neurologic efficacy over 52 Wk. Due to heterogeneity of disease presentation there is no single measure that captures GD3 neurologic disease, therefore primary endpoint was prespecified as a global test of multiple endpoints (Prespecified O'Brien ordinary least square [OLS] test) of motor function (assessed by mSARA) and cognition (assessed by Repeatable Battery for the Assessment of Neuropsychological Status [RBANS]). Secondary and exploratory systemic endpoints included % changes in spleen/liver volume, change in hemoglobin level and platelet count, biomarker plasma/CSF levels, and bone disease measured by bone marrow burden (BMB) total score, bone marrow density (BMD) Z-scores, and bone pain. Key secondary safety endpoints included treatment-emergent adverse events (AE), serious AEs, and AEs of special interest.

**Copyright Information:** (Online) ISSN: 2572-9241
©2026 The Author(s). HemaSphere is published by John Wiley & Sons Ltd on behalf of European Hematology Association. This is an open access Abstract Book distributed under the Attribution-NonCommercial-NoDerivs (CC BY-NC-ND), which allows third parties to download the articles and share them with others as long as they credit the author and the Abstract Book, but they cannot change the content in any way or use them commercially.
**Abstract Book Citations:** Authors, Title, HemaSphere, 2026;10:(S1):pages. The abstract book DOIs can be found on the "Author Information" page.
**Disclaimer:** Articles published in the journal HemaSphere exclusively reflect the opinions of the authors. The authors are responsible for all content in their abstracts including accuracy of the facts, statements, citing resources, etc.

**Results**

Enrollment was completed with 43 pts (VEN, n=21; IMI, n=22). Baseline demographic and clinical characteristics were comparable between groups, and all met predefined systemic therapeutic goals. VEN demonstrated statistically significant superiority in neurologic benefit at Wk 52 vs IMI based on the global O'Brien test of mSARA and RBANS scores (OLS statistic 2.59; one-sided p=0.007). Cognitive scores improved with VEN and declined with IMI (LS mean difference 8.32; nominal one-sided p=0.016), while motor outcomes showed directional improvement favoring VEN. Systemic disease control was maintained, with non-inferiority achieved for spleen/liver volume, and hemoglobin, except for platelet count. Pts treated with VEN demonstrated nominally significant reductions in plasma and CSF biomarkers vs IMI. Changes from baseline to Wk 52 in BMB total and BMD Z-scores across all assessed skeletal sites were similar between both arms and no pts reported bone pain in VEN. Safety profile was acceptable, with comparable AE incidence between arms, no new safety signals, and no need for rescue IMI during the 52-wk randomized period in the VEN arm.

**Summary/Conclusion**

LEAP2MONO is the first trial evaluating the brain-penetrant glucosylceramide synthase inhibitor VEN, in pediatric and adult pts with GD3. The trial demonstrates that VEN controls both CNS and systemic manifestations of GD3.

**Copyright Information:** (Online) ISSN: 2572-9241
©2026 The Author(s). HemaSphere is published by John Wiley & Sons Ltd on behalf of European Hematology Association. This is an open access Abstract Book distributed under the Attribution-NonCommercial-NoDerivs (CC BY-NC-ND), which allows third parties to download the articles and share them with others as long as they credit the author and the Abstract Book, but they cannot change the content in any way or use them commercially.
**Abstract Book Citations:** Authors, Title, HemaSphere, 2026;10:(S1):pages. The abstract book DOIs can be found on the "Author Information" page.
**Disclaimer:** Articles published in the journal HemaSphere exclusively reflect the opinions of the authors. The authors are responsible for all content in their abstracts including accuracy of the facts, statements, citing resources, etc.

### (S304) EFFECTS OF MITAPIVAT ON IRON BURDEN AND SPLEEN SIZE IN ERYTHROCYTE MEMBRANOPATHIES AND CONGENITAL DYSERYTHROPOIETIC ANEMIA TYPE II: 56-WEEK FOLLOW-UP RESULTS FROM THE SATISFY STUDY

**Topic:** 29. Enzymopathies, membranopathies and other red blood cell disorders

Thomas Doeven*[1], Eduard J. van Beers[1], Richard van Wijk[2], Joline Saes[1], Jesper Brix Petersen[3], Niels Vejlstrup[4], Jens Helby[3], Adeline Gladieux[5], Fatiha Chermat[5], Pierre Fenaux[5], Kevin H.M. Kuo[6], Andreas Glenthøj[3, 5]

*[1]University Medical Center Utrecht, Utrecht University, Center for Benign Hematology, Thrombosis and Hemostasis, van Creveldkliniek, Utrecht, The Netherlands; [2]University Medical Center Utrecht, Utrecht University, Central Diagnostic Laboratory, Utrecht, The Netherlands; [3]Copenhagen University Hospital, Rigshospitalet, Danish Red Blood Cell Center, Department of Hematology, Copenhagen, Denmark; [4]Copenhagen University Hospital, Rigshospitalet, Department of Cardiology, Copenhagen, Denmark; [5]ERN-EuroBloodNet, Paris, France; [6]University of Toronto, Division of Hematology, Toronto, Canada;*

### Background

Hereditary spherocytosis (HS), hereditary xerocytosis (HX) and congenital dyserythropoietic anemia type II (CDA II) are inherited hemolytic anemias. Secondary iron overload, a frequent complication, increases morbidity. Mitapivat, an oral pyruvate kinase activator, has shown beneficial effects on hemolysis and ineffective erythropoiesis across several hereditary anemias. Mitapivat also improved iron homeostasis in patients with pyruvate kinase deficiency and reduced iron overload and spleen size in HS mice.

### Aims

To evaluate the long-term effects of mitapivat on hemoglobin (Hb), liver iron content (LIC), and spleen size in patients with HS, HX, and CDA II.

### Methods

This multicenter, single-arm, phase 2 study (NCT05935202) enrolled 24 patients in Denmark and the Netherlands. Following 8 weeks of dose escalation, participants received mitapivat 100mg twice daily during two 24-week fixed dose periods (FDP1 and FDP2). FDP1 results have been reported previously, demonstrating a favorable safety profile with improvements in Hb, hemolytic markers and patient-reported outcomes, particularly in patients with HS. Hb response was defined as an increase of ≥1.0 g/dL from baseline, sustained at ≥2 consecutive visits. LIC was calculated from the T2* MRI using Garbowksi et al.'s method (LIC = 31.94 × $(T2^*)^{-1.014}$), and spleen volume using the standard prolate ellipsoid formula (0.52 x length x width x depth) from manual MRI measurements.

**Copyright Information:** (Online) ISSN: 2572-9241
©2026 The Author(s). HemaSphere is published by John Wiley & Sons Ltd on behalf of European Hematology Association. This is an open access Abstract Book distributed under the Attribution-NonCommercial-NoDerivs (CC BY-NC-ND), which allows third parties to download the articles and share them with others as long as they credit the author and the Abstract Book, but they cannot change the content in any way or use them commercially.
**Abstract Book Citations:** Authors, Title, HemaSphere, 2026;10:(S1):pages. The abstract book DOIs can be found on the "Author Information" page.
**Disclaimer:** Articles published in the journal HemaSphere exclusively reflect the opinions of the authors. The authors are responsible for all content in their abstracts including accuracy of the facts, statements, citing resources, etc.

### Results

Twenty-four participants were enrolled; median age was 42.5 years, and 54% were females. FDP1 was completed by 23 participants (15 HS, 4 CDA II, 4 HX) and FDP2 by 21 participants (15 HS, 4 CDA II, 2 HX). Sustained Hb improvements were observed, with an average increase from baseline of 1.1 g/dL (95% CI, 0.7 to 1.5, p<0.001) during FDP1, and 1.1 g/dL (95% CI, 0.6 to 1.5, p<0.001) during FDP2. Hb response was achieved by 13 of 23 (57%) participants during FDP1, and 10 of 21 (48%) participants during FDP2, the latter all with HS. Mean (SD) reticulocytes decreased from 265 (168) $x10^9$/L at baseline to 175 (111) $x10^9$/L at week 56, p<0.001, and total bilirubin from 3.5 (2.2) mg/dL to 2.1 (1.0) mg/dL, p<0.001. LDH and haptoglobin remained unchanged. Four participants receiving iron chelation or phlebotomies were excluded from the LIC and iron markers analysis. Median LIC decreased from 3.2 mg/g dry weight (95% CI, 2.2-3.8) at baseline to 2.7 mg/g dry weight (95% CI, 2.2-3.4) at week 56, p=0.29. Mean (SD) ferritin concentration decreased significantly from 396 µg/L (292) at baseline to 262 µg/L (170) at week 56, p<0.001, whilst transferrin saturation remained unchanged. In a subgroup of Hb responders, median LIC decreased significantly from 3.2 mg/g dry weight (95% CI, 1.7-4.2) at baseline to 2.6 mg/g dry weight (95% CI, 1.6-3.4), p=0.009. Spleen size and volume did not change in the full cohort or in the Hb responders subgroup. Mean (SD) length in centimeters was 15.9 (2.4) at baseline and 16.0 (2.5) at week 56, p=0.56; width was 12.5 (1.3) and 12.4 (1.5), p=0.40; and depth was 5.7 (1.1) and 5.7 (0.9), p=0.85. Lastly, mean (SD) spleen volume at baseline was 603 $cm^3$ (224) and 598 $cm^3$ (198) at week 56, p=0.79.

### Summary/Conclusion

Mitapivat treatment resulted in sustained Hb improvements, particularly in HS, along with reductions in ferritin concentration and, among responders, liver iron content, while spleen size remained unchanged. Further investigation in populations with higher iron burden and extended follow-up is warranted.

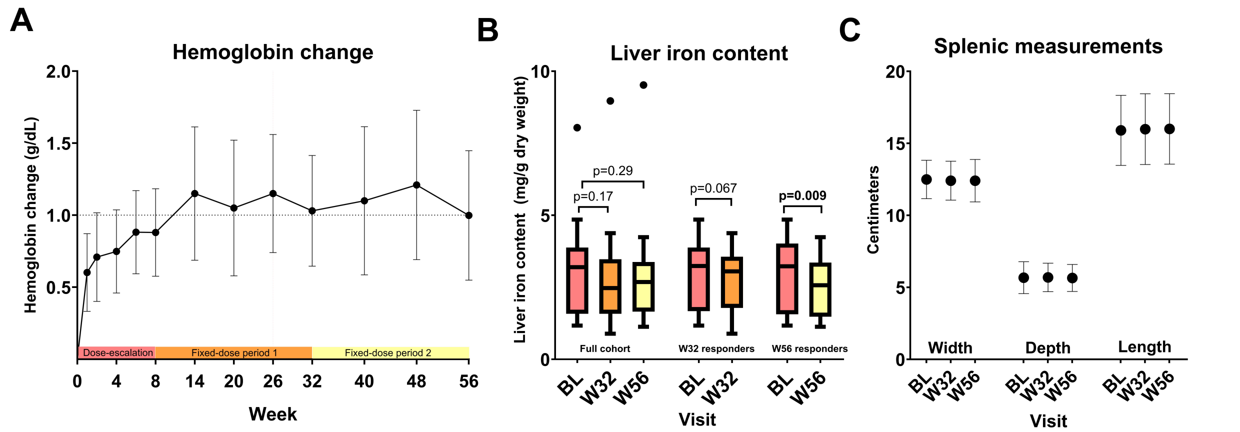

A) Mean hemoglobin change over time. Error bars represent the 95% confidence interval. The dotted line indicates a hemoglobin increase of 1.0 g/dL. B) Distribution of liver iron content by study visit in all participants that completed fixed dose period 2, excluding those receiving iron chelation or phlebotomies (n=18), and in the W32 responders (n=12) and W56 responders (n=10) subroups. Boxplots using Tukey's method, with p-values calculated using the exact paired Wilcoxon signed-rank test. C) Mean splenic measurements (length, depth, width) by study visit (n=20). Error bars represent the standard deviation. Abbrevations: BL: Baseline, W: Week

Copyright Information: (Online) ISSN: 2572-9241
©2026 The Author(s). HemaSphere is published by John Wiley & Sons Ltd on behalf of European Hematology Association. This is an open access Abstract Book distributed under the Attribution-NonCommercial-NoDerivs (CC BY-NC-ND), which allows third parties to download the articles and share them with others as long as they credit the author and the Abstract Book, but they cannot change the content in any way or use them commercially.
Abstract Book Citations: Authors, Title, HemaSphere, 2026;10:(S1):pages. The abstract book DOIs can be found on the "Author Information" page.
Disclaimer: Articles published in the journal HemaSphere exclusively reflect the opinions of the authors. The authors are responsible for all content in their abstracts including accuracy of the facts, statements, citing resources, etc.

### (S305) PAIRED PROTEOMIC ANALYSIS OF IN VIVO AND EX VIVO HUMAN ERYTHROID DIFFERENTIATION IDENTIFIES KEY ELEMENTS OF STRESS ERYTHROPOIESIS

**Topic:** 30. Iron metabolism, deficiency and overload

Christina Perraki[1], Kovi Shrung[2], Sadaf Fazeli[1], Yaroslav Kaminskiy[2], Ann-Charlotte Björklund[1], Indira Barbosa[1], Mikaela Hillberg Widfeldt[1], Janne Lehtiö[2], Eva Hellström Lindberg[1, 3], Teresa Mortera Blanco[1], Rui Branca[2], Pedro Moura*[1]

*[1]Karolinska Institutet, Center for Hematology and Regenerative Medicine (HERM), Department of Medicine Huddinge (MedH), Stockholm, Sweden; [2]Karolinska Institutet, Department of Oncology and Pathology, SciLifeLab, Stockholm, Sweden; [3]Karolinska University Hospital, Division of Hematology, Department of Medicine, Stockholm, Sweden;*

### Background

To generate a mature red blood cell, the committed erythroid progenitor cell and later erythroblast (EB) must undergo several phenotypical shifts such as hemoglobinization and surface content remodeling. This has been mainly understood through study of immunophenotypically-defined cell states. Coupled with *in vitro* culture, proteomics has proven key to understand the extensive proteome remodeling that underlies this process. However, due to the large number of cells required for conventional proteomics methods, little is known about human steady-state erythropoiesis, or how culture-induced stress erythropoiesis may shape our knowledge of erythroid maturation.

### Aims

In this study, we aimed to investigate the proteome of surface marker-defined erythroid cell compartments during steady-state and culture stress-induced differentiation in order to resolve the proteomic impact of stress erythropoiesis and define the steady-state human erythroid proteome.

### Methods

We investigated a cohort of 11 healthy bone marrow (BM) donors with paired cryopreserved BM mononuclear cell (MNC) samples. The first sample from each pair was thawed, magnetically-enriched for $CD34^{+}$ cells and cultured *in vitro* with defined, serum-free media for 14 days. At D14, the second sample was thawed, and erythroid cell subsets were purified from both samples through fluorescence-assisted cell sorting (FACS) based on CD71, CD105 and Glycophorin A (Yan H *et al.*, AJH 2021). In total, 130 samples were analyzed encompassing 8 erythroid cell subsets (EP1, EP2, EP3+4, ProEB, Early BasoEB, Late BasoEB, PolyEB, OrthoEB). For mass spectrometric analysis, we utilized our own newly-developed technology for low-cell total proteomics (pauciproteomics), achieving a total proteome coverage of ~9200 quantified proteins.

**Copyright Information:** (Online) ISSN: 2572-9241
©2026 The Author(s). HemaSphere is published by John Wiley & Sons Ltd on behalf of European Hematology Association. This is an open access Abstract Book distributed under the Attribution-NonCommercial-NoDerivs (CC BY-NC-ND), which allows third parties to download the articles and share them with others as long as they credit the author and the Abstract Book, but they cannot change the content in any way or use them commercially.
**Abstract Book Citations:** Authors, Title, HemaSphere, 2026;10:(S1):pages. The abstract book DOIs can be found on the “Author Information” page.
**Disclaimer:** Articles published in the journal HemaSphere exclusively reflect the opinions of the authors. The authors are responsible for all content in their abstracts including accuracy of the facts, statements, citing resources, etc.

**Results**

Although paired BM and cultured EB samples were purified by identical immunophenotype gating, cultured EB were significantly more varied in their morphological differentiation state (P < 0.01). This did not impair the intended proteomic analysis along differentiation, which resolved four common patterns of protein expression: rapid decrease of non-erythroid proteins (*n* = 699), early and late bimodal proteins (*n* = 836 and 412, respectively) and rapid increase of erythroid-associated proteins (*n* = 459). Remarkably, despite the above, unsupervised dimensional reduction separated the two groups by sample source. Most erythroid cytoskeleton and surface proteins were stably expressed (e.g. Band 3, Spectrins, Ankyrins). However, cultured cells highly upregulated embryoid/fetal globin chains (Zeta and Gamma 1+2, mean $Log_2$ Fold Change [$mLog_2FC$] = 3.58, max. adjusted P-value [$P_{adj}$] = $1.65 \times 10^{-13}$) and were significantly deficient in all carbonic anhydrases (CA1, CA2 and CA3, $mLog_2FC$ = -2.91, max. $P_{adj}$ = $1.01 \times 10^{-16}$). The major culture-enriched pathways included the specific expression of cytokines GDF15 and erythroferrone, both absent in BM cells; an extreme metabolic restructuring, in particular of cholesterol and fatty acid metabolism genes; and the remodeling of several kinase-driven signaling pathways, e.g. STAT3 and BTK.

**Summary/Conclusion**

This first proteomic analysis of steady-state human erythropoiesis reveals across all differentiation states that culture-induced stress remodels the erythroid proteome. The pathways identified here both validate decades of model studies and define new elements of stress erythropoiesis, offering key insights into the molecular underpinnings of erythroid development.

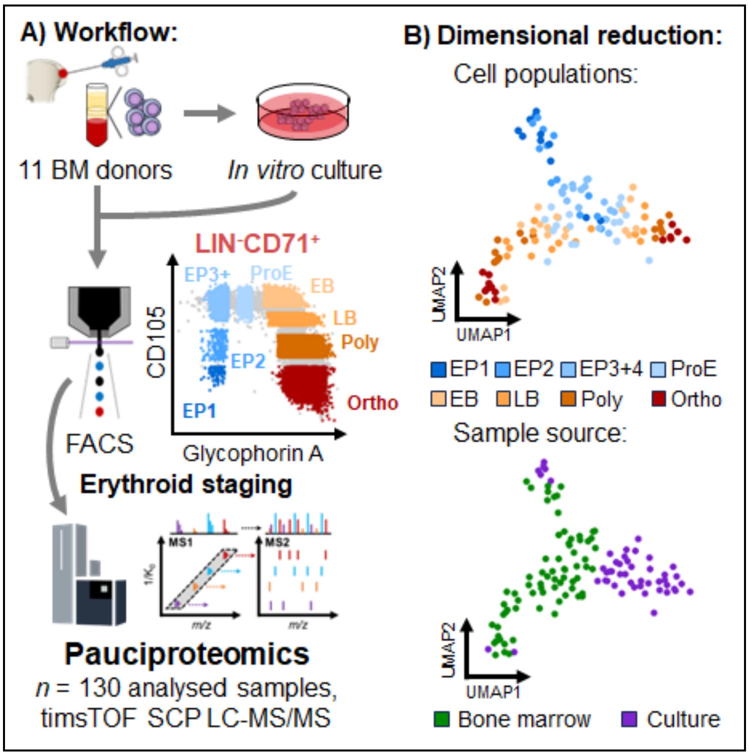

A) Study workflow, B) Dimensional reduction overlay of cell population and sample source data

**Copyright Information:** (Online) ISSN: 2572-9241
©2026 The Author(s). HemaSphere is published by John Wiley & Sons Ltd on behalf of European Hematology Association. This is an open access Abstract Book distributed under the Attribution-NonCommercial-NoDerivs (CC BY-NC-ND), which allows third parties to download the articles and share them with others as long as they credit the author and the Abstract Book, but they cannot change the content in any way or use them commercially.
**Abstract Book Citations:** Authors, Title, HemaSphere, 2026;10:(S1):pages. The abstract book DOIs can be found on the "Author Information" page.
**Disclaimer:** Articles published in the journal HemaSphere exclusively reflect the opinions of the authors. The authors are responsible for all content in their abstracts including accuracy of the facts, statements, citing resources, etc.

### (S306) TITLE: RALLY-MF: INITIAL EFFICACY OF A PHASE 2 STUDY OF DISC-0974, AN ANTI-HEMOJUVELIN ANTIBODY, TO TREAT ANEMIA IN MYELOFIBROSIS

**Topic:** 30. Iron metabolism, deficiency and overload

Naseema Gangat*[1], Ayalew Tefferi[1], Elizabeth Hexner[2], Moshe Talpaz[3], Kristen Pettit[3], Aaron T. Gerds[4], Akriti Jain[4], Laura Michaelis[5], James M Foran[6], Sima Bhatt[7], Akshay Buch[7], Olivia Pelletier[7], Will Savage[7], Prioty Islam[8], Raajit K Rampal[8], Prithviraj Bose[9], Amy Zhou[10], John Mascarenhas[11], Ronan Swords[12], Anna B. Halpern[13, 14]

*[1]Mayo Clinic, Rochester, United States of America; [2]University of Pennsylvania, Philadelphia, United States of America; [3]University of Michigan, Ann Arbor, United States of America; [4]Cleveland Clinic, Cleaveland, United States of America; [5]Medical College of Wisconsin, Milwaukee, United States of America; [6]Mayo Clinic, Jacksonville, United States of America; [7]Disc Medicine, Watertown, United States of America; [8]Memorial Sloan Kettering Cancer Center, New York, United States of America; [9]MD Anderson Cancer Center, Houston, United States of America; [10]Washington University, St. Louis, United States of America; [11]Mount-Sinai Hospital, New York, United States of America; [12]Oregon Health and Science University, Portland, United States of America; [13]Fred Hutchinson Cancer Center, Seattle, United States of America; [14]University of Washington, Seattle, United States of America;*

### Background

Hepcidin, a central regulator of iron homeostasis, is pathologically elevated in patients with myelofibrosis (MF) and anemia. Chronic hepcidin elevation limits iron availability for erythropoiesis and contributes to anemia onset and severity. DISC-0974 is an investigational monoclonal antibody that blocks hemojuvelin, a co-receptor in the bone morphogenetic protein-signaling pathway driving hepcidin expression.

### Aims

Evaluate the efficacy (primary) and safety and pharmacodynamics (secondary) of DISC-0974 in anemia of MF.

### Methods

RALLY-MF (NCT05320198) is an ongoing US Phase 2 open-label study in adults (≥18 years) with primary or secondary MF and hemoglobin (Hgb) <10 g/dL or red blood cell (RBC) transfusion requirement. Participants are categorized based on the number of RBC units transfused in the 84 days prior to screening: non-transfusion dependent (nTD; 0 units, Hgb <10 g/dL); low transfusion burden (TD low; 1-2 units); high transfusion burden (TD high; 3-12 units). Stable dose of concomitant Janus kinase inhibitor (JAKi) is allowed. DISC-0974 is administered subcutaneously at 50 mg monthly for up to 6 doses (treatment period) followed by an optional continuation period. The primary endpoint (major hematologic response) is cohort specific: transfusion independence for 16 weeks (TD low) or 12 weeks (TD high), or a mean Hgb increase of ≥1.5 g/dL from baseline for ≥12 weeks without transfusions (nTD). Minor response is a 50% reduction in transfusion requirement from baseline (TD) or mean Hgb increase of ≥1 g/dL from baseline for ≥12 weeks without transfusions (nTD). Data are summarized using descriptive statistics pooling Phase 1b participants treated at 50 mg (n=12).

**Copyright Information:** (Online) ISSN: 2572-9241
©2026 The Author(s). HemaSphere is published by John Wiley & Sons Ltd on behalf of European Hematology Association. This is an open access Abstract Book distributed under the Attribution-NonCommercial-NoDerivs (CC BY-NC-ND), which allows third parties to download the articles and share them with others as long as they credit the author and the Abstract Book, but they cannot change the content in any way or use them commercially.
**Abstract Book Citations:** Authors, Title, HemaSphere, 2026;10:(S1):pages. The abstract book DOIs can be found on the "Author Information" page.
**Disclaimer:** Articles published in the journal HemaSphere exclusively reflect the opinions of the authors. The authors are responsible for all content in their abstracts including accuracy of the facts, statements, citing resources, etc.

**Results**

At data cut (05 Jan 2026), the analysis set included 54 participants (nTD n=32 [59%], TD low n=12 [22%], TD high n=10 [19%]). Thirty-one participants (57%) completed treatment, with 74% (23/31) entering the continuation period; 6 participants (11%) withdrew early. There were no early withdrawals due to adverse events. Forty-eight percent (26/54) received concomitant JAKi therapy, mostly momelotinib (54%) and ruxolitinib (38%). DISC-0974 led to early, sustained hepcidin reduction (mean -87% on Day 15) and serum iron mobilization (mean 150% increase on Day 15). Meaningful and early hematologic responses were preceded by increased absolute reticulocyte counts (mean 110% on Day 22). Major response among evaluable participants (follow-up through Day 113): 54% (15/28) of nTD, 67% (6/9) of TD low, and 50% (3/6) of TD high participants. Overall response (major + minor) across cohorts was 70% with similar response regardless of concomitant JAKi therapy: 71% (15/21) not on JAKi; 68% (15/22) on JAKi, including 78% (7/9) on ruxolitinib and 64% (7/11) on momelotinib. Forty-eight participants (89%) experienced ≥1 treatment-emergent adverse event (TEAE), with 11 participants experiencing a TEAE considered related to DISC-0974. There were no related serious TEAEs, TEAEs leading to death, or SUSARs (suspected unexpected serious adverse reactions).

**Summary/Conclusion**

Treatment with DISC-0974 resulted in hematologic responses in most patients with MF and anemia, irrespective of concomitant JAKi therapy. DISC-0974 was well tolerated and demonstrated a favorable safety profile. Updated data, including patient-reported outcomes and long-term follow-up, will be presented. These findings validate potent hepcidin reduction as a promising approach to treating anemia in MF.

© 2026 American Society of Clinical Oncology, Inc. Reused with permission. This abstract was accepted and previously presented at the 2026 ASCO Annual Meeting. All rights reserved.

**Copyright Information:** (Online) ISSN: 2572-9241
©2026 The Author(s). HemaSphere is published by John Wiley & Sons Ltd on behalf of European Hematology Association. This is an open access Abstract Book distributed under the Attribution-NonCommercial-NoDerivs (CC BY-NC-ND), which allows third parties to download the articles and share them with others as long as they credit the author and the Abstract Book, but they cannot change the content in any way or use them commercially.
**Abstract Book Citations:** Authors, Title, HemaSphere, 2026;10:(S1):pages. The abstract book DOIs can be found on the “Author Information” page.
**Disclaimer:** Articles published in the journal HemaSphere exclusively reflect the opinions of the authors. The authors are responsible for all content in their abstracts including accuracy of the facts, statements, citing resources, etc.

### (S307) DIGITAL CAPABILITIES AND TRANSFUSION PRACTICE IN EUROPEAN HOSPITALS: EMERGING FINDINGS FROM THE PAN-EUROPEANTRANSFUSION RESEARCH INFRASTRUCTURE (PETRA) COLLABORATIVE

**Topic:** 32. Transfusion medicine

Hayley G Evans*[1], Mikko Arvas[2], Minna Ilmakunnas[3], Allameddine Allameddine[4], Isis Alonso[5], Denise Borg Aquilina[6], Ines Bojanic[7], Francesco Buccisano[8], Louise Ørnskov Drechsler[9], Christian Erikstrup[9], Niall Flavin[10], Frederic Garban[11], Jose Antonio García-Erce[12], Joan R Grífols[13], Katie Hands[14], Riin Kullaste[15], Ursula La Rocca[16], Martina Lukic[17], Toni Masi Roig[13], Anna Millan Alvarez[13], Claudio Pellegrino[18], Mirela Raos[19], Mafalda Ribeirinho[20], Linda Schoenborn[21], Cynthia So-Osman[22], Amanda Stewart[14], Thomas Thiele[21], Esa Turkulainen[23], Matea Vinkovic[24], Allison Waters[25], Suzanne Fustolo-Gunnink[26], Katja K van den Hurk[27], Simon J Stanworth[1]

*[1]University of Oxford, NIHR Blood and Transplant Research Unit in Data Driven Transfusion Practice, Nuffield Division of Clinical Laboratory Sciences, Radcliffe Department of Medicine, Oxford, United Kingdom; [2]Finnish Red Cross Blood Service, Research and Development, Helsinki, Finland; [3]Helsinki University Hospital, Helsinki, Finland; [4]Northern Ireland Blood Transfusion Service, Northern Ireland, United Kingdom; [5]Portuguese Institute of Blood and Transplantation,, Lisboa, Portugal; [6]Malta National Blood Transfusion Service, Msida, Malta; [7]University Hospital Centre Zagreb, Zagreb, Croatia; [8]University of Rome Tor Vergata, Rome, Italy; [9]Aarhus University Hospital, Aarhus, Denmark; [10]National Haemovigilance Office, Dublin, Ireland; [11]University of Grenoble Alpes, Grenoble, France; [12]Banco de Sangre y Tejidos de Navarra, Servicio Navarro de Salud, Pamplona, Spain;[13]Banc de Sang i Teixits, Blood Bank, Catalonia, Spain; [14]Scottish National Blood Transfusion Service, Scotland, United Kingdom; [15]North Estonia Medical Centre Foundation (PERH), Tallinn, Estonia; [16]Italian National Blood Center, National Institute of Health, Rome, Italy; [17]Croatian Institute of Transfusion Medicine, Clinical Hospital Merkur,, Zagreb, Croatia;[18]Policlinico Universitario Agostino Gemelli IRCCS, Rome, Italy; [19]University Clinical Hospital Centre Zagreb, Zagreb, Croatia;[20]Portuguese Institute of Blood and Transplantation, Lisboa, Portugal; [21]University Hospital Greifswald, Institute for Transfusion Medicine, Greifswald, Germany; [22]Sanquin, Amsterdam and Erasmus Medical Centre, Rotterdam, The Netherlands;[23]Finnish Red Cross Blood Service, Helsinki, Finland; [24]Croatian Institute of Transfusion Medicine, Research and Development, Zagreb, Croatia;[25]Irish Blood Transfusion Service, UCD School of Public Health, Physiotherapy and Sports Science, University College Dublin, Dublin, Ireland; [26]Public and Occupational Health, Amsterdam UMC & Amsterdam Public Health Research Institute, Amsterdam, The Netherlands; [27]Public and Occupational Health, Amsterdam UMC &Amsterdam Public Health Research Institute, Amsterdam, The Netherlands;*

### Background

The ability to benchmark and address variation in donation and transfusion practice across multiple sites is constrained by fragmented digital systems, inconsistent data standards, and limited donor-recipient linkage. The Pan-European Transfusion Research InfrAstructure (PETRA) collaborative was formed to describe the challenges of digital capabilities for transfusion analytics and to test whether a harmonised, multinational core dataset can be implemented to support pan-European donation and transfusion research, benchmarking and Patient Blood Management (PBM).

### Aims

To characterise digital capabilities for transfusion analytics across European hospitals and blood services, and to evaluate the feasibility of implementing a harmonised multinational core dataset to enable pan-European benchmarking, donation transfusion linkage, and support for Patient Blood Management (PBM).

**Copyright Information:** (Online) ISSN: 2572-9241
©2026 The Author(s). HemaSphere is published by John Wiley & Sons Ltd on behalf of European Hematology Association. This is an open access Abstract Book distributed under the Attribution-NonCommercial-NoDerivs (CC BY-NC-ND), which allows third parties to download the articles and share them with others as long as they credit the author and the Abstract Book, but they cannot change the content in any way or use them commercially.
**Abstract Book Citations:** Authors, Title, HemaSphere, 2026;10:(S1):pages. The abstract book DOIs can be found on the “Author Information” page.
**Disclaimer:** Articles published in the journal HemaSphere exclusively reflect the opinions of the authors. The authors are responsible for all content in their abstracts including accuracy of the facts, statements, citing resources, etc.

### Methods

We conducted a multi-centre study across hospitals and blood services recruited via EBA/EHA networks. Sites completed structured surveys and data flow diagrams describing Electronic Health Record (EHR), Laboratory Information Management System (LIMS), transfusion platforms, and linkage pathways. Over 12 months we developed a consensus core dataset via monthly working-group meetings and a workshop. Participating sites returned aggregate data for a recent complete calendar year. Analyses were descriptive and focused on data availability, extractability and linkage capability.

### Results

Nineteen sites from 15 countries completed digital capability surveys. Feasibility testing produced 32 datasets (30 hospitals and 2 national sources) across 13 countries, comprising information on 597,151 red blood cell (RBC) units.Transfusion (95%), laboratory (84%), and clinical data (79%) were widely available across sites, whereas access to outcome (63%), treatment (58%), and surgical data (58%) was more variable. Barriers to transfusion benchmarking included limited IT resources and poor interoperability (both 84%), data errors or missing information (68%), limited clinical resources (63%), processing capability gaps (47%), format differences (42%), regulatory or governance barriers (42%), delays in data access (32%), and ethical considerations (16%). As a result, variables requiring cross-system linkage, such as pre-transfusion haemoglobin, anaemia at admission, and peri-operative metrics, were inconsistently extractable, and many sites were unable to reliably link transfusions to clinical context or upstream blood supplier and donor data.

### Summary/Conclusion

Scalable, pan-European transfusion benchmarking is feasible but currently hampered by uneven interoperability, governance, and analytic capacity. Benchmarking capabilities vary widely, ranging from activity-based metrics to, less commonly, clinically meaningful analyses. The findings and consensus dataset provide a pragmatic foundation for staged expansion. Unlocking routine, clinically meaningful transfusion research and benchmarking will require targeted investment in interoperability, governance, and automated data extraction. The next step is the coordinated development and adoption of standardised European transfusion performance metrics to harmonise practice and enable clinically meaningful benchmarking in support of PBM.

**Copyright Information:** (Online) ISSN: 2572-9241
©2026 The Author(s). HemaSphere is published by John Wiley & Sons Ltd on behalf of European Hematology Association. This is an open access Abstract Book distributed under the Attribution-NonCommercial-NoDerivs (CC BY-NC-ND), which allows third parties to download the articles and share them with others as long as they credit the author and the Abstract Book, but they cannot change the content in any way or use them commercially.
**Abstract Book Citations:** Authors, Title, HemaSphere, 2026;10:(S1):pages. The abstract book DOIs can be found on the "Author Information" page.
**Disclaimer:** Articles published in the journal HemaSphere exclusively reflect the opinions of the authors. The authors are responsible for all content in their abstracts including accuracy of the facts, statements, citing resources, etc.

### (S308) THE COOL SIDE OF PLATELETS: EMERGENCE OF UNIQUE PLATELET SUBPOPULATIONS WITH SPECIFIC FUNCTIONAL PROPERTIES IN COLD-STORED PLATELET CONCENTRATES

**Topic:** 32. Transfusion medicine

Xavier Delabranche*[1, 2], Stephanie Magnenat[1], Nathalie Brouard[1], Clarisse Mouriaux[1], Floriane Pissenem-Rudwill[1], Adeline Galvanin[1], Hervé Isola[1], Pierre Mangin[1], Beatrice Hechler[1]

*[1]Etablissement Français du Sang - Grand Est, Inserm UMR-S1255, Strasbourg, France;[2]Strasbourg University Hospital, Department of Anesthesia and Intensive Care, Strasbourg, France;*

### Background

Current platelet transfusion requirements are evolving rapidly, with a significant increase in the need for therapeutic platelet transfusions compared to prophylactic use. In this context, cold-stored platelets have gained interest owing to their potentially advantageous characteristics, including the possible emergence of specific platelet subpopulations with particular functional properties.

### Aims

To assess the emergence of various platelet subpopulations and related functional properties in pathogen-reduced buffy-coat (BC) platelet concentrates (PCs) stored at +22°C or +4°C for up to 21 days.

### Methods

A pool-and-split strategy was used to obtain double-dose BC-PCs collected into PAS-III/plasma (55/45%) treated with amotosalen-UVA (INTERCEPT™ Blood System, Cerus Inc.) for pathogen reduction and stored at +22°C with constant agitation or at +4°C without agitation. Various platelet subpopulations were identified using a combination of platelet markers (P-selectin (Ps) and phosphatidylserine exposure (A), mitochondrial transmembrane potential $\Delta\psi m$ (T), PAC-1 binding (Pc) for activated GPIIbIIIa) analyzed by multicolor flow cytometry, confocal imaging on immobilized fibrinogen, and thrombus formation on a collagen-coated surface under flow (1500 $s^{-1}$).

**Copyright Information:** (Online) ISSN: 2572-9241
©2026 The Author(s). HemaSphere is published by John Wiley & Sons Ltd on behalf of European Hematology Association. This is an open access Abstract Book distributed under the Attribution-NonCommercial-NoDerivs (CC BY-NC-ND), which allows third parties to download the articles and share them with others as long as they credit the author and the Abstract Book, but they cannot change the content in any way or use them commercially.
**Abstract Book Citations:** Authors, Title, HemaSphere, 2026;10:(S1):pages. The abstract book DOIs can be found on the "Author Information" page.
**Disclaimer:** Articles published in the journal HemaSphere exclusively reflect the opinions of the authors. The authors are responsible for all content in their abstracts including accuracy of the facts, statements, citing resources, etc.

**Results**

During storage, the combination of the four markers allowed identification of six known platelet subpopulations: activated ($T^+A^-Ps^+Pc^-$), aggregatory ($T^+A^-Ps^+Pc^+$), procoagulant ($T^-A^+Ps^+Pc^-$), apoptotic ($T^-A^+Ps^-Pc^-$) and senescent ($T^-A^-Ps^{\pm}Pc^{\pm}$). Interestingly, we identified two novel PhtdSer-exposing ($A^+$) platelet subpopulations that displayed also activated αIIbβ3 ($Pc^+$), with either polarized ($T^+$) or depolarized ($T^-$) Δψm, all being P-selectin positive ($Ps^+$), designated Intermediate 1 (Int1, $T^+A^+Ps^+Pc^+$) and 2 (Int2, $T^-A^+Ps^+Pc^+$). Int1 and 2 appeared spontaneously in cold-stored PCs only, as of day 7, amounting to 7±2% and 15±3%, respectively. After strong stimulation with TRAP-6/CVX, both new populations were evidenced as soon as day 1, amounting to 26±5% and 36±8%, respectively, suggesting that they might also be generated *in vivo*. Indeed, both new populations emerged in fresh whole blood stimulated with TRAP-6/CVX, amounting to 23±2% (Int1) and 18±2% (Int2) after 2 min and then progressively decreased to (9±1%) and (1±1%) after 1 hour. Int1 platelets underwent robust spreading on immobilized fibrinogen, generating filopodia and lamellipodia, in accordance with functional αIIbβ3, together with PhtdSer exposure, either via surface-bound extracellular vesicles or through their own membrane remodeling. These latter features were also observed for Int2 platelets though without lamellipodia. These various subpopulations efficiently contributed to thrombus formation on a collagen-coated surface under flow, with Int1, Int2 and aggregatory platelets localized within the thrombus, while procoagulant and apoptotic platelets found surrounding the thrombus (**Figure**). Both contributed to the increased platelet aggregation and procoagulant activity observed upon stimulation of cold- versus room temperature-stored PCs.

**Summary/Conclusion**

These results identify two novel platelet subpopulations, with pathophysiological implications for both transfusion medicine in hemorrhagic patients as well as hemostasis and thrombosis.

**Copyright Information:** (Online) ISSN: 2572-9241
©2026 The Author(s). HemaSphere is published by John Wiley & Sons Ltd on behalf of European Hematology Association. This is an open access Abstract Book distributed under the Attribution-NonCommercial-NoDerivs (CC BY-NC-ND), which allows third parties to download the articles and share them with others as long as they credit the author and the Abstract Book, but they cannot change the content in any way or use them commercially.
**Abstract Book Citations:** Authors, Title, HemaSphere, 2026;10:(S1):pages. The abstract book DOIs can be found on the "Author Information" page.
**Disclaimer:** Articles published in the journal HemaSphere exclusively reflect the opinions of the authors. The authors are responsible for all content in their abstracts including accuracy of the facts, statements, citing resources, etc.

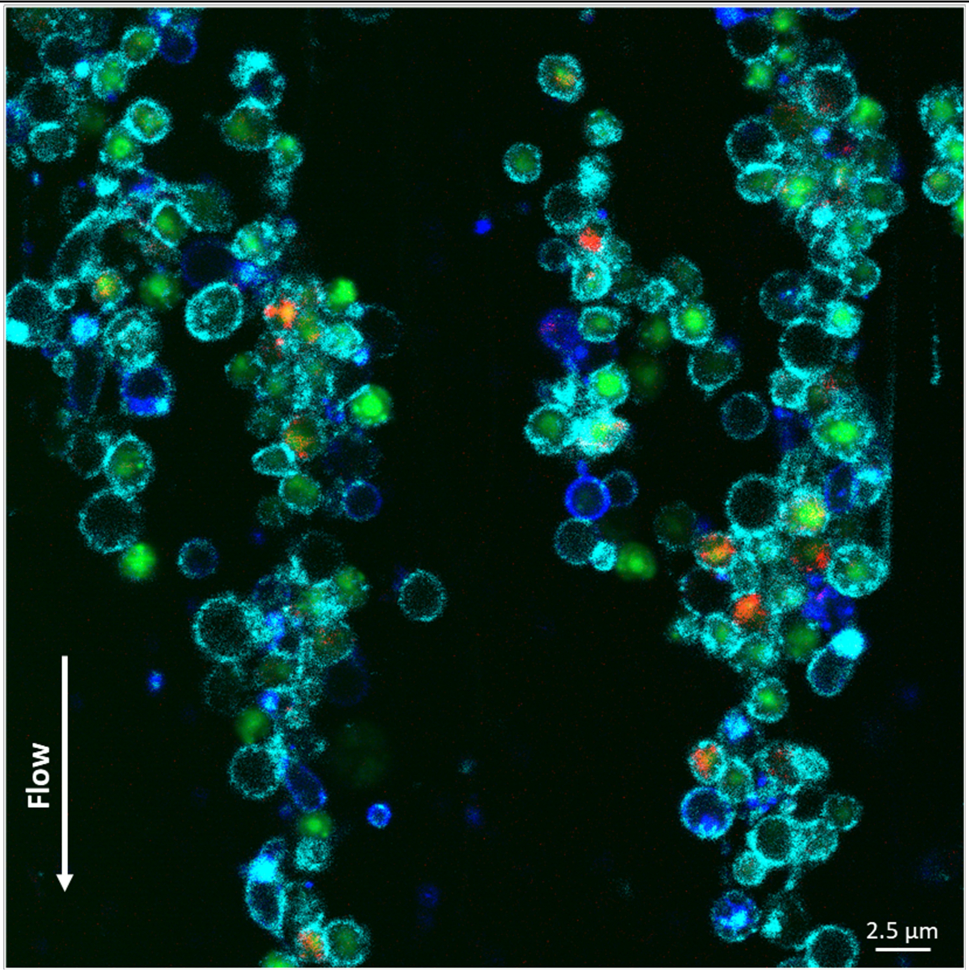

**Figure: Thrombus formation on collagen under flow.**
Cold-stored BC-PCs (day 14) were reconstituted with 50% (v/v) packed washed red blood cells (RBCs) containing hirudin (100 U/mL) and perfused over collagen at 1500 $s^{-1}$ for 2 min. Analysis of platelet thrombus content by confocal microscopy with the 4 fluorescent markers revealed the presence of various subpopulations. TMRM (green), P-selectin (cyan), PAC-1 (red), Annexin V (dark blue).

**Copyright Information:** (Online) ISSN: 2572-9241
©2026 The Author(s). HemaSphere is published by John Wiley & Sons Ltd on behalf of European Hematology Association. This is an open access Abstract Book distributed under the Attribution-NonCommercial-NoDerivs (CC BY-NC-ND), which allows third parties to download the articles and share them with others as long as they credit the author and the Abstract Book, but they cannot change the content in any way or use them commercially.
**Abstract Book Citations:** Authors, Title, HemaSphere, 2026;10:(S1):pages. The abstract book DOIs can be found on the "Author Information" page.
**Disclaimer:** Articles published in the journal HemaSphere exclusively reflect the opinions of the authors. The authors are responsible for all content in their abstracts including accuracy of the facts, statements, citing resources, etc.

### (S309) BEYOND PATIENT FACTORS: THE SUBSTANTIAL IMPACT OF SURGEON AND PROCEDURE VARIABILITY ON BLOOD TRANSFUSION RISK

**Topic:** 32. Transfusion medicine

Esa Turkulainen*[1], Mikko Arvas[1], Miika Koskinen[2], Minna Ilmakunnas[3]

*[1]Finnish Red Cross Blood Service, Research and Development, Helsinki, Finland;[2]Helsinki University Hospital, Diagnostic Center, Helsinki, Finland; [3]Helsinki University Hospital, Helsinki, Finland;*

### Background

Despite decades of development in statistical and machine learning models, accurate prediction of perioperative red blood cell (RBC) transfusion remains challenging. Most existing models rely predominantly on patient-level characteristics and preoperative laboratory values. However, inter-surgeon variability, an established but insufficiently quantified source of variation in surgical practice, has largely been excluded from transfusion risk modeling. This omission may contribute substantially to the limited predictive performance observed in prior studies.

### Aims

We sought to quantify the relative contribution of the operating surgeon and procedure type to perioperative red blood cell (RBC) transfusion risk and to assess whether incorporating these factors improves predictive performance.

### Methods

We retrospectively analyzed 283,224 elective surgical procedures and 16,507 associated RBC transfusion events at HUS Helsinki University Hospital (Finland) between 2021 and 2025. The primary outcome was perioperative RBC transfusion (during procedure and up to 1 week after). We fitted hierarchical logistic regression models including patient-level covariates (age, sex, BMI, estimated blood volume, latest preoperative Hb, and ASA class) and random effects for operating surgeon and procedure type. Odds ratios (ORs) and variance components were extracted to compare effect sizes across predictor domains. Model discrimination was evaluated using area under the precision-recall curve (AUPRC) and receiver operating characteristic curve (AUC), comparing models with and without surgeon and procedure effects.

### Results

Both operating surgeon and procedure type demonstrated substantial effect sizes in our preliminary analyses, yielding Median Odds Ratios of 3.78 and 2.95, respectively (**Figure 1**). Together, they accounted for 49.6% of the unobserved variance in the outcome (Intraclass Correlation Coefficients: surgeon = 29.8%, procedure = 19.8%). The inclusion of these random effects significantly improved model fit over fixed effects alone (Likelihood Ratio Test $\chi^2 = 16002$, $p < 0.001$). Furthermore, models incorporating surgeon and procedure data significantly outperformed the fixed-effects baseline in predictive discrimination (AUPRC: 0.42 vs. 0.14; AUC: 0.87 vs. 0.67, DeLong's test $p < 0.001$). The variability attributable to surgeon-level effects remained highly significant even after comprehensive adjustment for fixed patient factors and procedure type (Nested LRT $\chi^2 = 4204$, $p < 0.001$). Further development of the fixed-effects model and the addition of other relevant patient-level predictors are expected to affect these preliminary results.

**Copyright Information:** (Online) ISSN: 2572-9241
©2026 The Author(s). HemaSphere is published by John Wiley & Sons Ltd on behalf of European Hematology Association. This is an open access Abstract Book distributed under the Attribution-NonCommercial-NoDerivs (CC BY-NC-ND), which allows third parties to download the articles and share them with others as long as they credit the author and the Abstract Book, but they cannot change the content in any way or use them commercially.
**Abstract Book Citations:** Authors, Title, HemaSphere, 2026;10:(S1):pages. The abstract book DOIs can be found on the "Author Information" page.
**Disclaimer:** Articles published in the journal HemaSphere exclusively reflect the opinions of the authors. The authors are responsible for all content in their abstracts including accuracy of the facts, statements, citing resources, etc.

### Summary/Conclusion

Operating surgeon appears to be a major determinant of perioperative transfusion risk, even within the same procedural category. Our findings identify a critical blind spot in current transfusion risk estimation protocols and suggest that provider-level effects should be explicitly incorporated into predictive models. Accounting for surgeon-related variability may improve transfusion forecasting, resource allocation, and standardization of perioperative blood management practices.

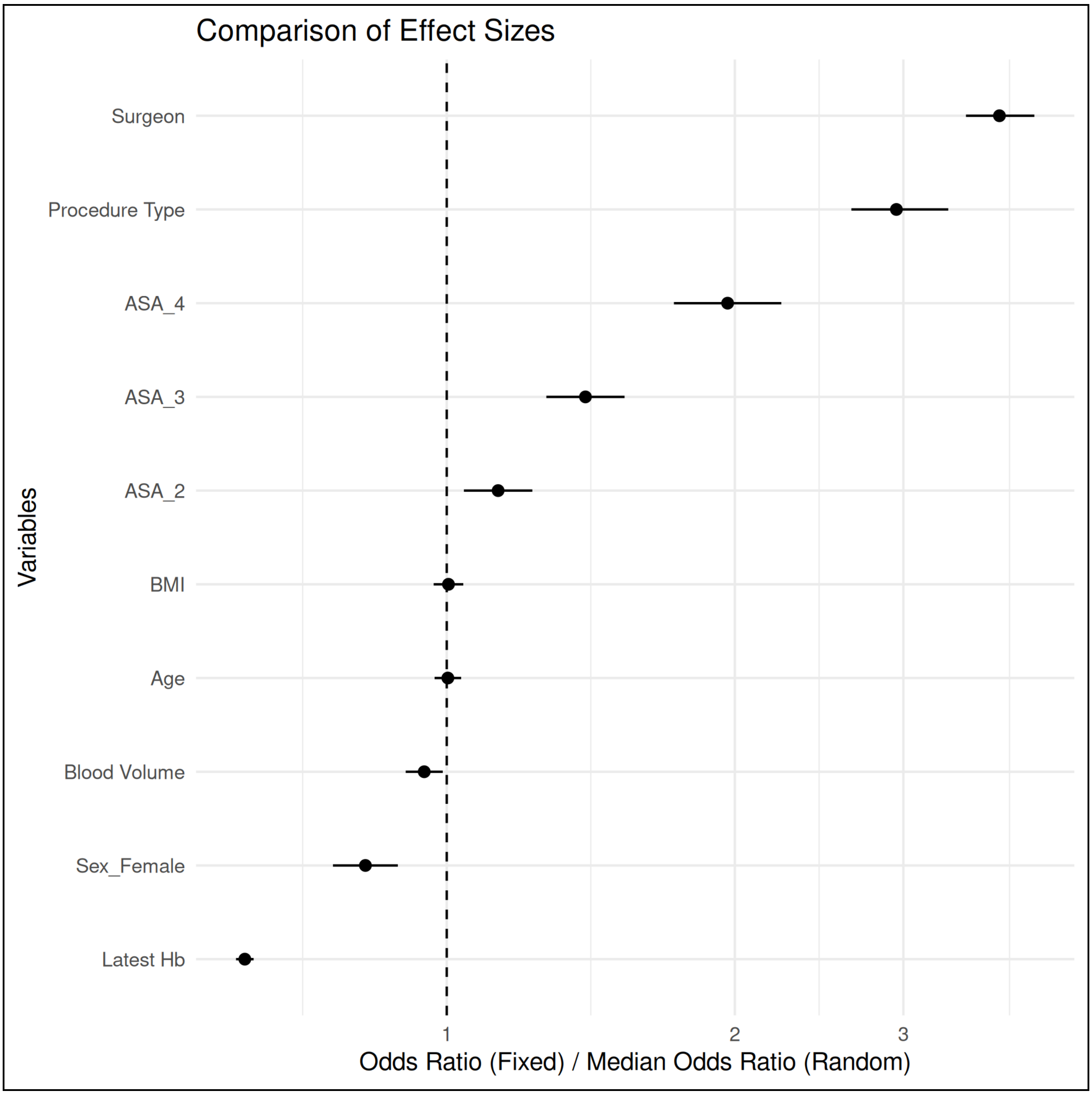

**Copyright Information:** (Online) ISSN: 2572-9241
©2026 The Author(s). HemaSphere is published by John Wiley & Sons Ltd on behalf of European Hematology Association. This is an open access Abstract Book distributed under the Attribution-NonCommercial-NoDerivs (CC BY-NC-ND), which allows third parties to download the articles and share them with others as long as they credit the author and the Abstract Book, but they cannot change the content in any way or use them commercially.
**Abstract Book Citations:** Authors, Title, HemaSphere, 2026;10:(S1):pages. The abstract book DOIs can be found on the "Author Information" page.
**Disclaimer:** Articles published in the journal HemaSphere exclusively reflect the opinions of the authors. The authors are responsible for all content in their abstracts including accuracy of the facts, statements, citing resources, etc.

### (S310) TEMPORAL TRENDS IN INFECTION-RELATED PROPORTIONAL MORTALITY IN LYMPHOMA: A DANISH POPULATION-BASED STUDY

**Topic:** 31. Infections in hematology (incl. supportive care/therapy)

William Mølgaard Ullahammer*[1], Nickolaj Risbo[2, 3], Eyal Oren[2, 4], Henrik Sørensen[2, 3], Tarec El-Galaly[1, 2, 3, 5]

*[1]Aarhus University Hospital, Department of Hematology, Aarhus, Denmark;[2]Aarhus University and Aarhus University Hospital, Department of Clinical Epidemiology, Center for Population Medicine, Aarhus, Denmark; [3]Aarhus University, Department of Clinical Medicine, Aarhus, Denmark; [4]San Diego State University, Division of Epidemiology and Biostatistics, School of Public Health, San Diego, California, United States of America; [5]Aarhus University Hospital, Department of Molecular Medicine, Aarhus, Denmark;*

### Background

Lymphomas are heterogeneous cancers characterized by substantial variations in outcomes across histological subtypes. Over the past two decades, both treatment strategies and supportive care have undergone notable changes, which may have influenced the extent to which infections contribute to cause of death among lymphomas over time.

### Aims

To examine temporal trends in proportional mortality from infections in patients with lymphoma, both overall and stratified by histological subtype and treatment.

### Methods

This proportional mortality study included adult Danish patients (≥ 18 years) with diffuse large B-cell lymphoma (DLBCL), follicular lymphoma (FL), peripheral T-cell lymphoma (PTCL), and Hodgkin's lymphoma (HL) diagnosed in the period 2001-2020 and registered in the Danish National Lymphoma Registry (LYFO). The primary endpoint was proportional mortality from infections evaluated in restricted 5-year intervals. Mortality fully or partially attributed to infections was identified based on the presence of infections listed on death certificates. Secondary analyses included trends over time by age, first-line treatment, performance status, and lymphoma subtype.

### Results

A total of 15,849 patients were included; 8,294 (52.3%) with DLBCL, 3,987 (25.2%) with FL, 2,516 (15.9%) with HL, and 1,052 (6.6%) with PTCL. The median age for the full cohort was 65 years but varied over time from 61 years in 2001-2005 to 68 years in 2016-2020. The male:female ratio was 1.2 (54.6% males). Across the full study period, infections were recorded on death certificates in 30% of all patients. Proportional mortality related to infections was 25.6% (95% CI: 22.9-28.6) in 2001-2005, 33.8% (95% CI: 30.8-37) in 2006-2010, 33.6% (95% CI: 30.8-36.6) in 2011-2015, and 26.7% (95% CI: 24-29.6) in 2016-2020. Patients with PTCL had the highest proportional mortality from infections (36.9%, 95% CI: 32.5–41.4), followed by HL (33.4%, 95% CI: 28.3–39.0), FL (29.3%, 95% CI: 25.2–33.8), and DLBCL (28.5%, 95% CI: 26.8–30.3). The study population showed an increase in proportional mortality from 2001–2005 to 2006–2010, including analyses where patients were restricted to receiving standard first-line therapy. Proportional mortality subsequently decreased from 2011–2015 to 2016–2020 among patients treated with standard first-line therapy.

**Copyright Information:** (Online) ISSN: 2572-9241
©2026 The Author(s). HemaSphere is published by John Wiley & Sons Ltd on behalf of European Hematology Association. This is an open access Abstract Book distributed under the Attribution-NonCommercial-NoDerivs (CC BY-NC-ND), which allows third parties to download the articles and share them with others as long as they credit the author and the Abstract Book, but they cannot change the content in any way or use them commercially.
**Abstract Book Citations:** Authors, Title, HemaSphere, 2026;10:(S1):pages. The abstract book DOIs can be found on the "Author Information" page.
**Disclaimer:** Articles published in the journal HemaSphere exclusively reflect the opinions of the authors. The authors are responsible for all content in their abstracts including accuracy of the facts, statements, citing resources, etc.

**Summary/Conclusion**

A rising trend of infection-related deaths was observed among patients with DLBCL, HL, PTCL, and FL from 2001-2005 to 2006-2010. This pattern was also observed among those receiving standard first-line treatment for DLBCL, HL, and FL. In the most recent calendar period (2016-2020), infection-related mortality declined, which may reflect improved supportive care measures such as wider use of granulocyte-colony stimulating factor and antimicrobial prophylaxis. The observed stability in proportional mortality related to infections over time, despite a substantial increase in the median age of patients and more frequent use of curative chemotherapy in older patient groups, is consistent with improvements in supportive care.

**Copyright Information:** (Online) ISSN: 2572-9241
©2026 The Author(s). HemaSphere is published by John Wiley & Sons Ltd on behalf of European Hematology Association. This is an open access Abstract Book distributed under the Attribution-NonCommercial-NoDerivs (CC BY-NC-ND), which allows third parties to download the articles and share them with others as long as they credit the author and the Abstract Book, but they cannot change the content in any way or use them commercially.
**Abstract Book Citations:** Authors, Title, HemaSphere, 2026;10:(S1):pages. The abstract book DOIs can be found on the "Author Information" page.
**Disclaimer:** Articles published in the journal HemaSphere exclusively reflect the opinions of the authors. The authors are responsible for all content in their abstracts including accuracy of the facts, statements, citing resources, etc.

### (S311) HIGH-DOSE VERSUS STANDARD-DOSE INACTIVATED INFLUENZA VACCINE IN OLDER ADULTS WITH HEMATOLOGICAL MALIGNANCIES: SUBGROUP ANALYSIS FROM THE DANFLU-2 RANDOMIZED TRIAL

**Topic:** 31. Infections in hematology (incl. supportive care/therapy)

Christian Brieghel*[1, 2, 3], Niklas Johansen[4, 5], Anne Marie Jensen[4, 5], Daniel Modin[4, 5], Kira Janstrup[5], Maj Henningsen[5], Matthew Loiacono[6, 7], Rebecca Harris[8], Ashley Huang[9], Kaveh Hosseini[4, 5], Carsten Larsen[10, 11, 12], Lykke Larsen[13], Lothar Wiese[14], Michael Dalager-Pedersen[15, 16], Brian Claggett[17], Katja Bartholdy[4, 5], Katrine Bernholm[4, 5], Julie Borchsenius[4, 5], Filip Davidovski[4, 5], Lise Davodian[4, 5], Maria Dons[4, 5], Lisa Duus[4, 5], Caroline Espersen[4, 5], Frederik Fussing[4, 5], Nino Landler[4, 5], Adam Langhoff[4, 5], Mats Lassen[4, 5], Anne Nielsen[4, 5], Camilla Ottosen[4, 5], Morten Sengeløv[4, 5], Kristoffer Skaarup[4, 5], Scott Solomon[17], Martin Landray[18], Gunnar Gislasson[4, 19, 20], Lars Køber[19, 21], Pradeesh Sivapalan[19, 22], Cyril Martel[23], Jens Jensen[19, 22], Mads Eiberg[2], Jakob Werner Hansen[1], Agoston Szabo[1, 24], Caspar Da Cunha-Bang[1, 4], Carsten Niemann[1, 2, 19], Tor Biering-Sørensen[4, 5, 21, 25]

*[1]Copenhagen University Hospital - Rigshospitalet, Hematology, Copenhagen, Denmark; [2]Danish Cancer Insititute, Hematology, Copenhagen, Denmark; [3]Zealand University Hospital - Roskilde, Department of Hematology, Roskilde, Denmark; [4]Copenhagen University Hospital–Herlev and Gentofte, Department of Cardiology, Copenhagen, Denmark; [5]University of Copenhagen, Center for Translational Cardiology and Pragmatic Randomized Trials, Department of Biomedical Sci-ences, Faculty of Health and Medical Sciences, Copenhagen, Denmark; [6]Sanofi, Swiftwater; PA, United States of America; [7]University of Delaware, Department of Epidemiology, Newark, Delaware, United States of America; [8]Sanofi, Singapore, Singapore; [9]Sanofi, Lyon, France; [10]Aarhus University Hospital, Department of Clinical Medicine, Aarhus, Denmark; [11]European LifeCare Group, Danske Lægers Vaccinations Service, Søborg, Denmark; [12]Aarhus University Hospital, Department of Infectious Diseases, Aarhus, Denmark; [13]Odense University Hospital, Department of Infectious Diseases, Odense, Denmark; [14]Zealand University Hospital - Roskilde, Department of Infectious Diseases, Roskilde, Denmark; [15]Aalborg University Hospital, Department of Infectious Diseases, Aalborg, Denmark; [16]Aalborg University, Department of Clinical Medicine, Aalborg, Denmark; [17]Brigham and Women's Hospital, Harvard Medical School, Cardiovascular Division, Boston, MA, United States of America; [18]University of Oxford, Nuffield Department of Population Health, Oxford, United Kingdom; [19]University of Copenhagen, Department of Clinical Medicine, Copenhagen, Denmark; [20]Danish Heart Foundation, Copenhagen, Denmark; [21]Copenhagen University Hospital - Rigshospitalet, Department of Cardiology, Copenhagen, Denmark; [22]Copenhagen University Hospital–Herlev and Gentofte, Respiratory Medicine Section, Department of Medicine, Copenhagen, Denmark; [23]Statens Serum Institut, Epidemiological Infectious Disease Preparedness, Copenhagen, Denmark; [24]Vejle Hospital, Department of Hematology, Vejle, Denmark; [25]Steno Diabetes Center Copenhagen, Herlev, Denmark;*

### Background

Patients with hematological malignancies (HM) are at high risk of infections and have suboptimal vaccine response. However, it remains unclear whether high-dose (HD-IIV) vs standard-dose inactivated influenza vaccine (SD-IIV) may improve clinical outcomes in patients with HM.

### Aims

To investigate the relative vaccine effectiveness (rVE) of HD-IIV vs SD-IIV against hospitalizations in participants from DANFLU-2 with HM with or without antineoplastic treatment (ATx) within the year prior to vaccination.

**Copyright Information:** (Online) ISSN: 2572-9241
©2026 The Author(s). HemaSphere is published by John Wiley & Sons Ltd on behalf of European Hematology Association. This is an open access Abstract Book distributed under the Attribution-NonCommercial-NoDerivs (CC BY-NC-ND), which allows third parties to download the articles and share them with others as long as they credit the author and the Abstract Book, but they cannot change the content in any way or use them commercially.
**Abstract Book Citations:** Authors, Title, HemaSphere, 2026;10:(S1):pages. The abstract book DOIs can be found on the "Author Information" page.
**Disclaimer:** Articles published in the journal HemaSphere exclusively reflect the opinions of the authors. The authors are responsible for all content in their abstracts including accuracy of the facts, statements, citing resources, etc.

### Methods

DANFLU-2 (NCT05517174) was a pragmatic, registry-based, active-controlled, 1:1 randomized trial of a single dose of HD-IIV vs SD-IIV in individuals ≥65 years during the 2022/2023, 2023/2024, and 2024/2025 influenza seasons in Denmark. We evaluated the rVE of HD-IIV vs SD-IIV against hospitalization for 1) influenza or pneumonia (primary endpoint), 2) influenza, and 3) cardio-respiratory hospitalizations in participants with and without HM diagnoses (i.e. ICD10 C81-C96, D46, D47.4) within 1 year prior to randomization.

### Results

In the overall population, 166,218 (2561 with HM) and 166,220 (2584 with HM) participants were randomized to receive HD-IIV and SD-IIV, respectively. HM included lymphoma (n=2345; 45.6%), chronic lymphocytic leukemia (CLL: n=1217; 23.7%), plasma cell dyscrasia (PCD: n=858; 16.7%), myeloid neoplasia (MN: n=717; 13.9%), and acute lymphoblastic leukemia (n=8; 0.2%): 738 (28.9%) and 710 (27.5%) had received ATx in the HD-IIV and SD-IIV group, respectively.
The HM population (n=5145) was older (mean 75.3 vs 73.7 years) and more comorbid compared with participants without HM and had a significant higher incidence of 1) hospitalization for pneumonia or influenza (3.5% vs 0.7%), 2) hospitalization for influenza alone (0.5% vs 0.08%), and 3) cardio-respiratory hospitalization (6.1% vs 2.3%; P<0.001; Figure).
HD-IIV vs SD-IIV did not significantly lower the risk of hospitalization for pneumonia or influenza in the overall population, with similar results regardless of HM status (HM: rVE -3.2 [95%CI -40.0;23.9] vs no HM: rVE 6.6 [95%CI -1.7;14.2]; p=0.52). Without evidence of differential effects according to HM status for influenza (HM: rVE 36.9 [95%CI -47.8;74.4] vs no HM: rVE 44.2 [95%CI 27.4;57.3]; p=0.77) and cardio-respiratory hospitalization (HM: rVE -7.5 [95% CI -35.2;14.4] vs no HM: rVE 6.2 [95%CI 1.8;10.4]; p=0.22), results were consistent with the effect for influenza hospitalization in the overall population.
Participants with HM in the HD-IIV group, who received ATx <1 year prior to randomization, showed higher event rates of hospitalizations for influenza or pneumonia (n=48; 6.5% vs n=31; 4.4%) and cardio-respiratory hospitalizations (n=79; 10.7% vs n=44; 6.2%), but not for influenza alone (n=8; 1.1% vs n=8; 1.1%) as compared to the SD-IIV group (Figure). This difference was seen in participants with lymphoma, CLL, PCD, and MN, respectively. Types of ATx were well-balanced between the vaccination groups.

### Summary/Conclusion

In this subgroup analysis of the largest randomized influenza vaccine trial, HD-IIV showed consistent benefit over SD-IIV against hospitalization for influenza regardless of a history of HM. Higher event rates among participants with HM potentially increases the absolute benefit from vaccination in HM. Even so, for participants with HM receiving antineoplastic treatment, higher rates of pneumonia or influenza and cardio-respiratory hospitalizations were seen in the HD-IIV group as compared with the SD-IIV group.

**Copyright Information:** (Online) ISSN: 2572-9241
©2026 The Author(s). HemaSphere is published by John Wiley & Sons Ltd on behalf of European Hematology Association. This is an open access Abstract Book distributed under the Attribution-NonCommercial-NoDerivs (CC BY-NC-ND), which allows third parties to download the articles and share them with others as long as they credit the author and the Abstract Book, but they cannot change the content in any way or use them commercially.
**Abstract Book Citations:** Authors, Title, HemaSphere, 2026;10:(S1):pages. The abstract book DOIs can be found on the "Author Information" page.
**Disclaimer:** Articles published in the journal HemaSphere exclusively reflect the opinions of the authors. The authors are responsible for all content in their abstracts including accuracy of the facts, statements, citing resources, etc.

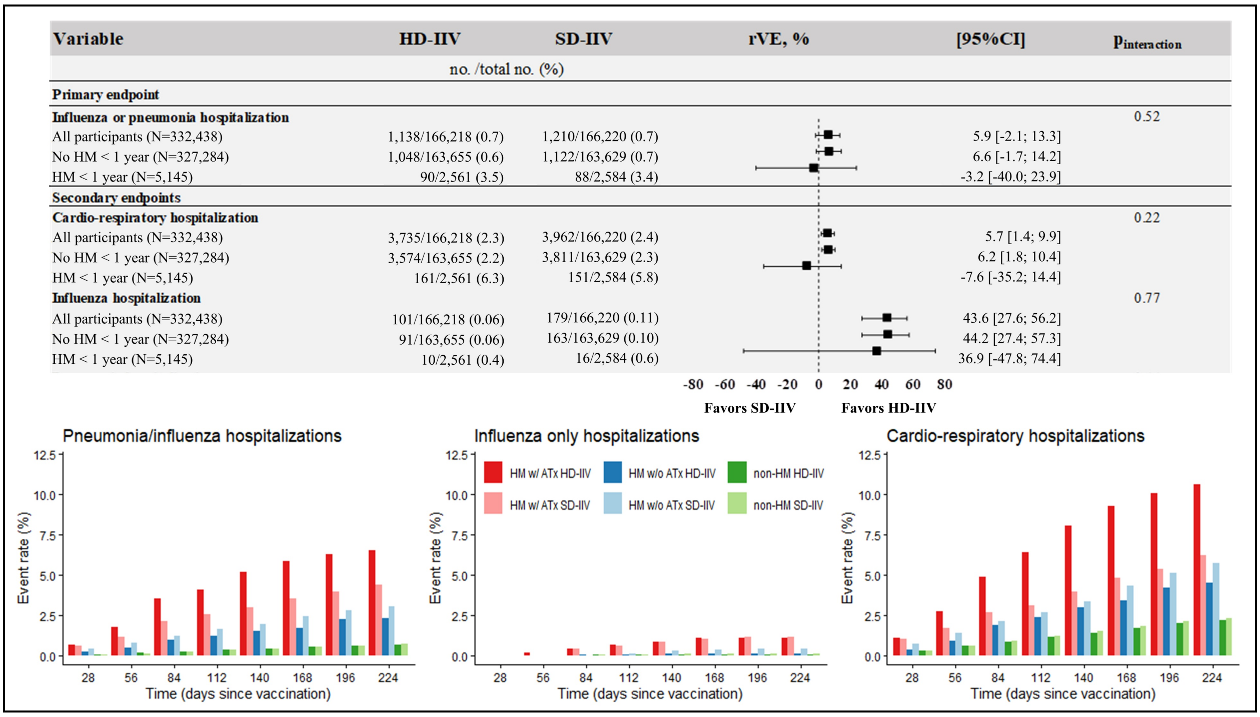

| Variable | HD-IIV | SD-IIV | rVE, % | [95%CI] | $P_{interaction}$ |
|---|---|---|---|---|---|
| | no. /total no. (%) | | | | |
| **Primary endpoint** | | | | | |
| **Influenza or pneumonia hospitalization** | | | | | 0.52 |
| All participants (N=332,438) | 1,138/166,218 (0.7) | 1,210/166,220 (0.7) | | 5.9 [-2.1; 13.3] | |
| No HM < 1 year (N=327,284) | 1,048/163,655 (0.6) | 1,122/163,629 (0.7) | | 6.6 [-1.7; 14.2] | |
| HM < 1 year (N=5,145) | 90/2,561 (3.5) | 88/2,584 (3.4) | | -3.2 [-40.0; 23.9] | |
| **Secondary endpoints** | | | | | |
| **Cardio-respiratory hospitalization** | | | | | 0.22 |
| All participants (N=332,438) | 3,735/166,218 (2.3) | 3,962/166,220 (2.4) | | 5.7 [1.4; 9.9] | |
| No HM < 1 year (N=327,284) | 3,574/163,655 (2.2) | 3,811/163,629 (2.3) | | 6.2 [1.8; 10.4] | |
| HM < 1 year (N=5,145) | 161/2,561 (6.3) | 151/2,584 (5.8) | | -7.6 [-35.2; 14.4] | |
| **Influenza hospitalization** | | | | | 0.77 |
| All participants (N=332,438) | 101/166,218 (0.06) | 179/166,220 (0.11) | | 43.6 [27.6; 56.2] | |
| No HM < 1 year (N=327,284) | 91/163,655 (0.06) | 163/163,629 (0.10) | | 44.2 [27.4; 57.3] | |
| HM < 1 year (N=5,145) | 10/2,561 (0.4) | 16/2,584 (0.6) | | 36.9 [-47.8; 74.4] | |

**Copyright Information:** (Online) ISSN: 2572-9241
©2026 The Author(s). HemaSphere is published by John Wiley & Sons Ltd on behalf of European Hematology Association. This is an open access Abstract Book distributed under the Attribution-NonCommercial-NoDerivs (CC BY-NC-ND), which allows third parties to download the articles and share them with others as long as they credit the author and the Abstract Book, but they cannot change the content in any way or use them commercially.
**Abstract Book Citations:** Authors, Title, HemaSphere, 2026;10:(S1):pages. The abstract book DOIs can be found on the "Author Information" page.
**Disclaimer:** Articles published in the journal HemaSphere exclusively reflect the opinions of the authors. The authors are responsible for all content in their abstracts including accuracy of the facts, statements, citing resources, etc.

### (S312) SEROPROTECTION AGAINST VACCINE-PREVENTABLE INFECTIONS IN CAR T-CELL RECIPIENTS: LOWER BASELINE TITERS IN BCMA- VERSUS CD19-CAR T PATIENTS PERSIST AFTER THERAPY

**Topic:** 31. Infections in hematology (incl. supportive care/therapy)

Giulia Magno*[1, 2], Elif Apak[3], Savanna Süß[1, 4], Giulia Rappa[1, 4], Patricia M. Späth[3], Sophia Stock[1, 2, 5], Alessandra Holzem[1, 2], Wajma Shahbaz[1], Linus Kruk[1], Florentien Kolbig[1], Tobias Tix[1], Stephan Baumann[6, 7], Anna Purcarea[6, 7], Louise Cremer[8], Sibylle Mellinghoff[8], Carolina Berger[9], Boris Fehse[9], Francis Ayuk[9], Judith Hecker[6, 7], Florian Bassermann[6, 7], Sebastian Theurich[1, 2], Michael von Bergwelt[1, 2], Kai Rejeski[1, 2, 10], Oliver Keppler[3], Marion Subklewe[1, 2, 4], Veit Bücklein[1, 2]

*[1]Department of Medicine III, University Hospital LMU Munich, Munich, Germany; [2]German Cancer Consortium (DKTK), Partner Site Munich, Munich, Germany; [3]Max von Pettenkofer-Institute and Gene Center, Virology, National Reference Center for Retroviruses, Faculty of Medicine, Ludwig-Maximilians-University Munich, Munich, Germany; [4]Laboratory for Translational Cancer Immunology, Gene Center LMU Munich, Munich, Germany; [5]Institute of Clinical Pharmacology, LMU University Hospital, LMU Munich, Munich, Germany; [6]Department of Medicine III, Technical University of Munich (TUM) Klinikum rechts der Isar, School of Medicine and Health, Munich, Germany; [7]TranslaTUM, Center for Translational Cancer Research, Technical University of Munich (TUM), Munich, Germany; [8]Department I of Internal Medicine, Centre for Integrated Oncology Aachen Bonn Cologne Duesseldorf (CIO ABCD), Faculty of Medicine and University Hospital Cologne, University of Cologne, Cologne, Germany; [9]Department of Stem Cell Transplantation, University Medical Center Hamburg-Eppendorf, Hamburg, Germany; [10]Adult BMT and Cellular Therapy Service, Department of Medicine, Memorial Sloan Kettering Cancer Center, New York, United States of America;*

### Background

CAR T-cell therapy has significantly improved outcomes in B-cell and plasma cell malignancies but is frequently associated with prolonged immune dysfunction and sustained infection risk. Vaccination is central to infection prevention; however, current vaccine recommendations for CAR T-cell recipients are largely extrapolated from other immunocompromised populations. Emerging data suggest differential seroprotection following CD19-directed versus BCMA-directed CAR T-cell therapy, yet knowledge of pre and post CART vaccine titers remain unknown.

### Aims

To investigate the longitudinal changes in pathogen-specific humoral immunity after CAR T-cell therapy.

### Methods

We performed a retrospective, observational study of 273 patients with a B-cell or plasma cell malignancy who were treated with commercial BCMA-directed (n = 84) or CD19-directed (n = 189) CAR T cells between January 2019 and August 2025 at four German academic centers. IgG antibody titers against vaccine-preventable infections – including mumps, measles, rubella, varicella zoster virus (VZV), hepatitis A, hepatitis B, SARS-CoV-2, Influenza A and B and respiratory syncytial virus (RSV) – were assessed before CAR T-cell infusion, 3-6 months and starting from 12 months after treatment. As per institutional practices, patients did not receive MMR-V or hepatitis vaccinations post-treatment, whereas a considerable proportion were vaccinated against SARS-CoV-2.

**Copyright Information:** (Online) ISSN: 2572-9241
©2026 The Author(s). HemaSphere is published by John Wiley & Sons Ltd on behalf of European Hematology Association. This is an open access Abstract Book distributed under the Attribution-NonCommercial-NoDerivs (CC BY-NC-ND), which allows third parties to download the articles and share them with others as long as they credit the author and the Abstract Book, but they cannot change the content in any way or use them commercially.
**Abstract Book Citations:** Authors, Title, HemaSphere, 2026;10:(S1):pages. The abstract book DOIs can be found on the "Author Information" page.
**Disclaimer:** Articles published in the journal HemaSphere exclusively reflect the opinions of the authors. The authors are responsible for all content in their abstracts including accuracy of the facts, statements, citing resources, etc.

**Results**

Overall, patient characteristics in the BCMA and CD19 treated cohorts were largely comparable, with no significant differences in age, sex, number of prior therapy lines, and baseline IgG levels. Prior hematopoietic cell transplantation and the use of IVIG replacement therapy following CAR T-cell treatment were more frequent in the BCMA-treated cohort.
Across all time points, BCMA-CAR T-cell recipients presented with lower antibody titers compared with CD19-CAR T-cell recipients for mumps, measles, rubella, VZV, influenza A, influenza B, and RSV (**Figure 1**). No significant difference was detected regarding hepatitis A, hepatitis B or SARS-CoV-2.
Importantly, among patients with available paired pre- and post-treatment samples (n=197), only 1-6% demonstrated a loss of seropositivity following CAR-T therapy depending on the pathogen, indicating that pre-existing humoral immunity is generally maintained following CAR T-cell treatment, irrespective of CAR-T target antigen.
When assessing immune reconstitution parameters at the 3-6 months timepoint, higher IgG levels where significantly associated with seropositivity, whereas B-cell counts, CD4 T-cell counts, and IgA levels did not differ significantly between seropositive and seronegative patients.

**Summary/Conclusion**

To our knowledge, this is the largest CAR T patient cohort with longitudinal assessments of vaccine-specific antibody titers, including assessments prior to therapy, so far. BCMA-CAR T-cell recipients display significantly lower seroprotection rates compared with CD19-CAR T-cell recipients, both prior to and following treatment. CAR-T cell therapy itself is of limited influence on the seroprotection rates. Our findings underscore the need for personalized vaccination strategies before and after CAR T-cell therapy.

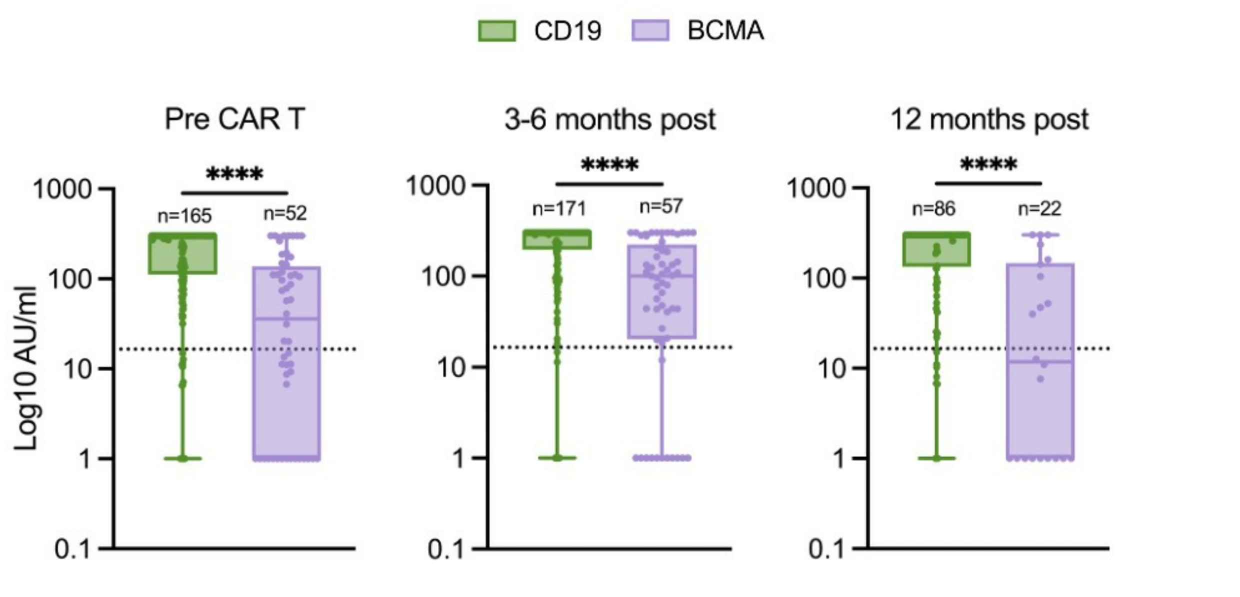

Figure 1 – Antibody titers against measles prior to and following CAR T-cell treatment

**Copyright Information:** (Online) ISSN: 2572-9241
©2026 The Author(s). HemaSphere is published by John Wiley & Sons Ltd on behalf of European Hematology Association. This is an open access Abstract Book distributed under the Attribution-NonCommercial-NoDerivs (CC BY-NC-ND), which allows third parties to download the articles and share them with others as long as they credit the author and the Abstract Book, but they cannot change the content in any way or use them commercially.
**Abstract Book Citations:** Authors, Title, HemaSphere, 2026;10:(S1):pages. The abstract book DOIs can be found on the "Author Information" page.
**Disclaimer:** Articles published in the journal HemaSphere exclusively reflect the opinions of the authors. The authors are responsible for all content in their abstracts including accuracy of the facts, statements, citing resources, etc.

### (S313) PRIMARY PROPHYLAXIS WITH IMMUNOGLOBULIN-REPLACEMENT THERAPY IMPROVES INFECTION-FREE AND OVERALL SURVIVAL IN PATIENTS WITH MULTIPLE MYELOMA TREATED WITH B-CELL MATURATION ANTIGEN BISPECIFIC ANTIBODIES

**Topic:** 31. Infections in hematology (incl. supportive care/therapy)

Antoine Azar*[1], Rahul Rajkumar[2], Jiani Zhou[3], Elisabeth Calderón-Gómez[4], Jonathan Lilley[2], Igor Rossa-Ramm[4], Yu-Shan Lee[2], Xuan Zhang[2], James Jordan[3], Elisabet Viayna[4]

*[1]Johns Hopkins University School of Medicine, Division of Allergy and Clinical Immunology, Baltimore, United States of America; [2]Boston Strategic Partners, Inc., Boston, United States of America; [3]Grifols Shared Services North America (SSNA), Durham, United States of America; [4]Grifols S.A., Barcelona, Spain;*

### Background

Multiple myeloma (MM) is one of the most common hematological malignancies and remains incurable despite an expanding treatment armamentarium. Bispecific antibodies (BsAbs) targeting B-cell maturation antigen (BCMA) improve overall and progression-free survival but are associated with an increased risk of severe infections that may hinder clinical management. Immunoglobulin-replacement therapy (IgRT) reduces infection risk, although the optimal timing of initiation remains uncertain.

### Aims

To assess the effectiveness of primary IgRT prophylaxis in patients with MM initiating BCMA BsAbs therapy.

### Methods

A retrospective, real-world evidence study was conducted using data from TriNetX's Dataworks, a large U.S. electronic health record database. Adults diagnosed with MM initiating treatment with BCMA BsAbs between Jun-21 and Jun-25 who had a minimum of 6 months of observable history prior to index were included. Index was defined as the first recorded BCMA BsAbs administration. Patients with a history of primary immunodeficiency, human immunodeficiency virus infection, solid organ transplant or rejection, or other hematological malignancies prior to index were excluded. Patients with severe infections within 7 days prior to index or those treated with IgRT within 28 days prior to index were also excluded. Patients initiating IgRT within 60 days of index in the absence of a severe infection were considered exposed to primary prophylaxis; the rest were unexposed. Patients were followed from index through 18 months, death, loss-to-follow-up, or end of data availability, whichever occurred first. Severe infection was defined as either an event meeting the U.S. Food and Drug Administration (FDA) Sentinel-validated serious infection algorithm or an infection resulting in inpatient hospitalization. Severe infection-free survival (IFS), defined as time to first severe infection or death, and overall survival (OS) were assessed. IgRT was modeled as time-dependent exposure in survival analyses. IFS and OS were analyzed using the Simon–Makuch method with Mantel–Byar comparison, and hazard ratios (HR) were estimated using Cox proportional hazards models.

**Copyright Information:** (Online) ISSN: 2572-9241
©2026 The Author(s). HemaSphere is published by John Wiley & Sons Ltd on behalf of European Hematology Association. This is an open access Abstract Book distributed under the Attribution-NonCommercial-NoDerivs (CC BY-NC-ND), which allows third parties to download the articles and share them with others as long as they credit the author and the Abstract Book, but they cannot change the content in any way or use them commercially.
**Abstract Book Citations:** Authors, Title, HemaSphere, 2026;10:(S1):pages. The abstract book DOIs can be found on the "Author Information" page.
**Disclaimer:** Articles published in the journal HemaSphere exclusively reflect the opinions of the authors. The authors are responsible for all content in their abstracts including accuracy of the facts, statements, citing resources, etc.

**Results**

Of the 965 patients included in the analysis, 225 (23.3%) received primary IgRT prophylaxis, and 740 (76.7%) did not. Mean (SD) age was 69.8 (9.7) vs 69.6 (9.8) years, and 54.7% and 53.6% were male among those exposed and unexposed to primary IgRT prophylaxis, respectively. Most common pre-index therapies were immunomodulatory imide drugs (72.9% vs 65.7%), proteasome inhibitors (63.6% vs 48.6%), and anti-CD38 monoclonal antibodies (51.6% vs 38.5%); 40.4% vs 39.2% had undergone hematopoietic stem cell transplantation. Primary IgRT prophylaxis was associated with improved IFS (Mantel–Byar p=0.013) (Figure 1A), as well as a reduced hazard of infection or death (HR 0.71, 95% CI 0.54–0.93). Median IFS was 17.6 months in patients treated with primary IgRT prophylaxis compared to 6.8 months in those who were not; 12- and 18-month IFS were 53.0% vs 41.6% and 46.7% vs 37.0%, respectively. Patients exposed to primary IgRT prophylaxis also experienced longer OS (Mantel–Byar p=0.002) (Figure 1B), with a HR of 0.39 (95% CI 0.21–0.71). Twelve- and 18-month OS were 93.9% vs 78.8% and 90.1% vs 77.0%, respectively.

**Summary/Conclusion**

Among patients with MM initiating BCMA BsAbs, primary IgRT prophylaxis was associated with significantly improved severe infection-free and overall survival.

**Copyright Information:** (Online) ISSN: 2572-9241
©2026 The Author(s). HemaSphere is published by John Wiley & Sons Ltd on behalf of European Hematology Association. This is an open access Abstract Book distributed under the Attribution-NonCommercial-NoDerivs (CC BY-NC-ND), which allows third parties to download the articles and share them with others as long as they credit the author and the Abstract Book, but they cannot change the content in any way or use them commercially.
**Abstract Book Citations:** Authors, Title, HemaSphere, 2026;10:(S1):pages. The abstract book DOIs can be found on the "Author Information" page.
**Disclaimer:** Articles published in the journal HemaSphere exclusively reflect the opinions of the authors. The authors are responsible for all content in their abstracts including accuracy of the facts, statements, citing resources, etc.

**Figure 1.** Infection-free survival (A) and overall survival (B) among patients with multiple myeloma initiating B-cell maturation antigen (BCMA)–targeting bispecific antibodies (BsAbs), comparing primary prophylaxis with immunoglobulin replacement therapy (IgRT) (defined as initiation within 60 days of BsAbs start and in the absence of a severe infection) versus no primary IgRT prophylaxis. Survival estimates were generated using time-varying (Simon–Makuch) methods.

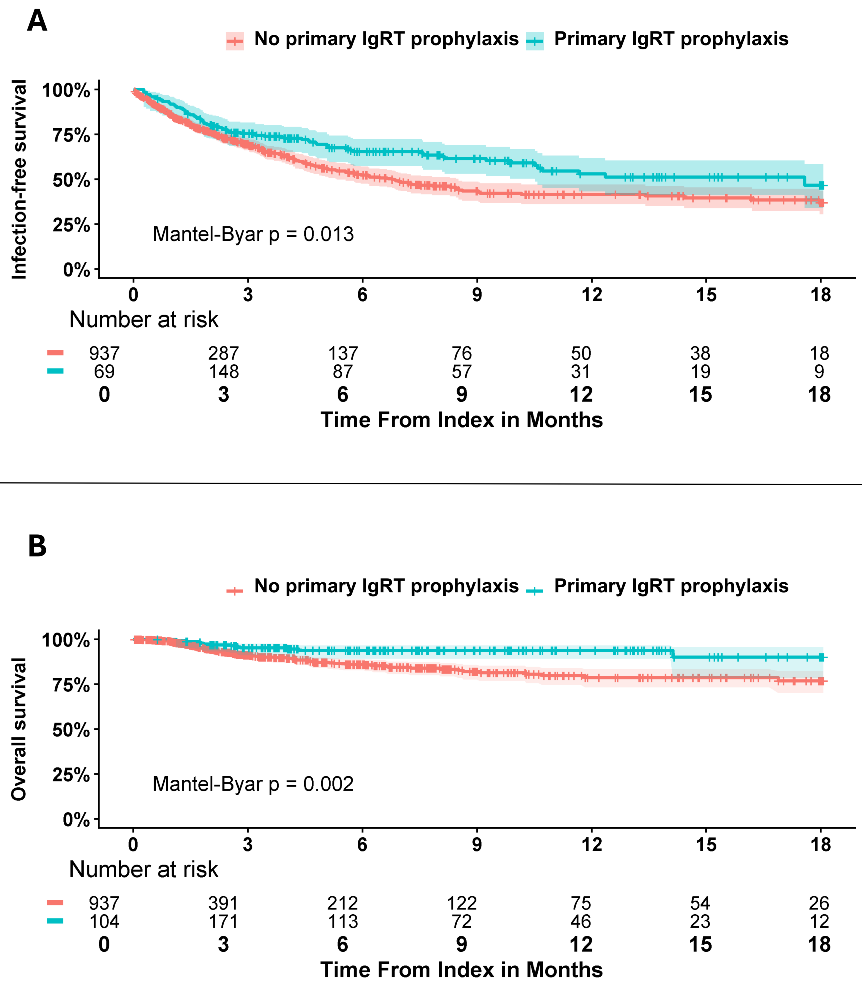

**Copyright Information:** (Online) ISSN: 2572-9241
©2026 The Author(s). HemaSphere is published by John Wiley & Sons Ltd on behalf of European Hematology Association. This is an open access Abstract Book distributed under the Attribution-NonCommercial-NoDerivs (CC BY-NC-ND), which allows third parties to download the articles and share them with others as long as they credit the author and the Abstract Book, but they cannot change the content in any way or use them commercially.
**Abstract Book Citations:** Authors, Title, HemaSphere, 2026;10:(S1):pages. The abstract book DOIs can be found on the "Author Information" page.
**Disclaimer:** Articles published in the journal HemaSphere exclusively reflect the opinions of the authors. The authors are responsible for all content in their abstracts including accuracy of the facts, statements, citing resources, etc.

### (S314) EARLY PROPHYLACTIC RITUXIMAB REDUCED THE INCIDENCE OF EBV INFECTION AND PTLD IN ALLOGENEIC HEMATOPOIETIC STEM CELL TRANSPLANTATION: A MULTICENTER STUDY

**Topic:** 23. Stem cell transplantation - Clinical

Ao Zhang*[1], Zilu Zhang[1], Zhuohui Man[2], Shanyu Zhang[3], Chuanhe Jiang[1], Luxiang Wang[1], Jiayu Huang[1], Zengkai Pan[1], Haiyang Lu[1], Chen Chen[4], Ren Lin[3], Yang Cao[2], Xiaoxia Hu[1]

*[1]State Key Laboratory of Medical Genomics, Shanghai Institute of Hematology, National Research Center for Translational Medicine, Shanghai Rui Jin Hospital, Shanghai Jiao Tong University School of Medicine, Shanghai, China; [2]Department of Hematology, Tongji Hospital, Tongji Medical College, Huazhong University of Science and Technology, Wuhan, China; [3]Department of Hematology, Nanfang Hospital, Southern Medical University, Clinical Medical Research Center of Hematological Diseases of Guangdong Province, Guangzhou, China; [4]GoBroad Healthcare Group, Beijing, China;*

### Background

Anti-thymocyte globulin (ATG) significantly reduces graft-versus-host disease (GvHD) after allogeneic hematopoietic cell transplantation (allo-HCT), but has associated with an increased risk of Epstein-Barr virus (EBV) infection and post-transplant lymphoproliferative disorder (PTLD). Current preemptive rituximab strategies with or without chemotherapy resulted in 40-60% efficacy, and still approximately 50% patients need alternative therapy, including Epstein-Barr Virus-Specific T cells (EBV-VST). Therefore, prophylactic strategies are of significant clinical interest.

### Aims

To evaluate the efficacy and safety of an early prophylactic rituximab strategy (day -15 to +30) on EBV infection, PTLD, and immune reconstitution in patients undergoing ATG-based allo-HCT.

### Methods

Between April 2021 and October 2025, 1127 allo-HCT recipients were consecutively screened in three transplant centers. The inclusion criteria included: 1) age ≥14 years; 2) undergoing their first allo-HCT; 3) receiving an ATG-based conditioning regimen; and 4) no prior history of EBV-associated hemophagocytic lymphohistiocytosis or lymphoma. Based on these criteria, 714 eligible patients were enrolled in accordance with the Declaration of Helsinki. According to whether the patient received ≥ one dose of 375 $mg/m^2$ rituximab between day -15 and +30, the patients were divided into two groups: R+ group (n = 280, 39.22%) and R- group (n = 434, 60.78%). Inverse probability of treatment weighting (IPTW) balanced baseline characteristics, while competing risk and multivariable regressions evaluated endpoints.

**Copyright Information:** (Online) ISSN: 2572-9241
©2026 The Author(s). HemaSphere is published by John Wiley & Sons Ltd on behalf of European Hematology Association. This is an open access Abstract Book distributed under the Attribution-NonCommercial-NoDerivs (CC BY-NC-ND), which allows third parties to download the articles and share them with others as long as they credit the author and the Abstract Book, but they cannot change the content in any way or use them commercially.
**Abstract Book Citations:** Authors, Title, HemaSphere, 2026;10:(S1):pages. The abstract book DOIs can be found on the "Author Information" page.
**Disclaimer:** Articles published in the journal HemaSphere exclusively reflect the opinions of the authors. The authors are responsible for all content in their abstracts including accuracy of the facts, statements, citing resources, etc.

### Results

The median age was 43 and 41 years for the R+ and R- groups, respectively. The majority of patients underwent haploidentical transplantation (86.8% *vs.* 80.4%) and had an EBV D+R+ serostatus (92.9% *vs.* 76.0%). After IPTW adjustment, the two groups achieved balance across all baseline variables. In the IPTW-weighted cohort, the 180-day cumulative incidence of EBV reactivation was significantly lower in the R+ group (5.8% *vs.* 37.7%, P < 0.001; Figure 1A). Consistently, the 180-day cumulative incidence of PTLD was also significantly reduced in the R+ group (1.3% *vs.* 5.1%, P = 0.035; Figure 1B). Multivariable analysis confirmed prophylactic rituximab as an independent protective factor for both EBV infection (HR=0.167, 95% *CI*: 0.090–0.310, P<0.001) and PTLD (HR=0.203, 95% *CI*: 0.065–0.632, P=0.006). The R+ group exhibited a significantly lower 100-day cumulative incidence of total acute GvHD (27.1% *vs.* 37.5%, P=0.036). Long-term survival outcomes (2-year overall survival, relapse-free survival, GvHD-free and relapse-free survival) and hematologic reconstitution did not differ significantly between the groups. Notably, the proportion of patients with infection-related non-relapse mortality was significantly lower in the R+ cohort (25.79% *vs.* 53.27%, P=0.030). Longitudinal immune reconstitution revealed that while B-cell recovery was expectedly delayed, T-cell (CD4+ and CD8+) and NK-cell trajectories were not compromised. Importantly, the R+ group demonstrated a significantly higher proportion of achieving good immune reconstitution after 4.5 months (P = 0.016).

### Summary/Conclusion

Early prophylactic rituximab is a safe and effective strategy for preventing EBV infection and PTLD in ATG-based allo-HCT, reducing early aGvHD and infection-related mortality without compromising long-term survival and T-cell immune reconstitution.

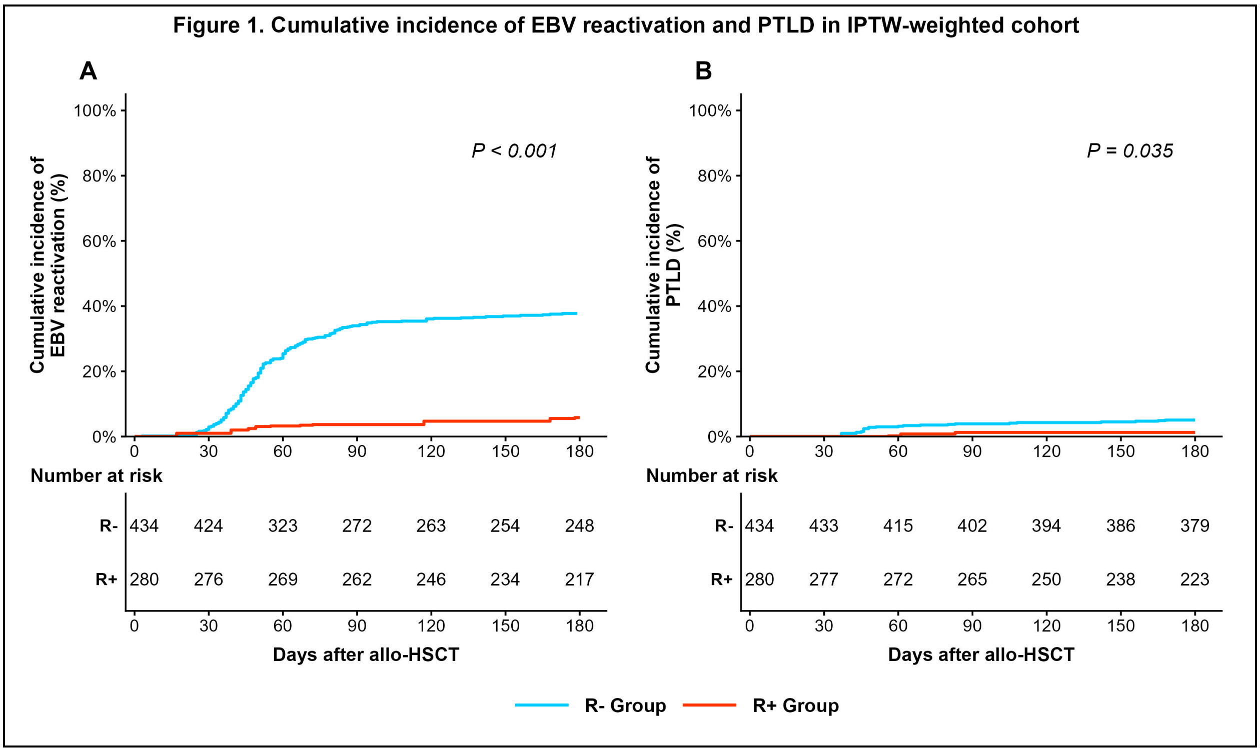

Figure 1. Cumulative incidence of EBV reactivation and PTLD in the IPTW-weighted cohort. (A) Cumulative incidence of EBV reactivation. (B) Cumulative incidence of PTLD. allo-HSCT, allogeneic hematopoietic stem cell transplantation; EBV, Epstein-Barr virus; PTLD, post-transplant lymphoproliferative disorder; IPTW, inverse probability of treatment weighting.

**Copyright Information:** (Online) ISSN: 2572-9241
©2026 The Author(s). HemaSphere is published by John Wiley & Sons Ltd on behalf of European Hematology Association. This is an open access Abstract Book distributed under the Attribution-NonCommercial-NoDerivs (CC BY-NC-ND), which allows third parties to download the articles and share them with others as long as they credit the author and the Abstract Book, but they cannot change the content in any way or use them commercially.
**Abstract Book Citations:** Authors, Title, HemaSphere, 2026;10:(S1):pages. The abstract book DOIs can be found on the "Author Information" page.
**Disclaimer:** Articles published in the journal HemaSphere exclusively reflect the opinions of the authors. The authors are responsible for all content in their abstracts including accuracy of the facts, statements, citing resources, etc.

### (S315) PHASE 3 LUNA3 FINAL EFFICACY AND SAFETY RESULTS FOR RILZABRUTINIB IN THE LONG-TERM EXTENSION PERIOD IN ADULTS WITH PERSISTENT/CHRONIC IMMUNE THROMBOCYTOPENIA (ITP)

**Topic:** 33. Platelet disorders

David J. Kuter*[1], Lei Zhang[2], Waleed Ghanima[3], Yoshitaka Miyakawa[4], David Gómez Almaguer[5], Sylvain Audia[6], Wojciech Homenda[7], Chuen Wen Tan[8], Matias Cordoba[9], Marek Wardecki[10], Larissa Mege[11], Qingcong Yuan[9], Ahmed Daak[9], Howard Liebmann[12]

*[1]Hematology Division, Massachusetts General Hospital, Harvard Medical School, Boston, United States of America;[2]Institute of Hematology and Blood Diseases Hospital, Chinese Academy of Medical Sciences and Peking Union Medical College, Tianjin, China; [3]Østfold Hospital Trust, Grålum, Norway and Institute of Clinical Medicine, University of Oslo, Oslo, Norway;[4]Department of Hematology, Saitama Medical University, Saitama, Japan; [5]Hospital Universitario Dr. José Eleuterio González, Monterrey, Mexico; [6]CHU Dijon Bourgogne – Hospital Francois Mitterrand, Dijon, France;[7]Regional Hospital, Slupsk and Institute of Health Sciences, Pomeranian University of Slupsk, Slupsk, Poland; [8]Department of Hematology, Singapore General Hospital, Singapore, Singapore; [9]Sanofi, Cambridge, United States of America; [10]Sanofi, Warsaw, Poland; [11]Sanofi, Lyon, France; [12]University of Southern California, Los Angeles, United States of America;*

### Background

Rilzabrutinib, an oral Bruton tyrosine kinase inhibitor acting through multi-immune modulation, has shown rapid and durable platelet responses, improved physical fatigue and bleeding scores, and favorable safety in the phase 2 LUNA2 study and the double-blind (DB; vs placebo) and open-label (OL) periods of the phase 3 LUNA3 study (NCT04562766, 2023-509401-71). Eligible patients (pts) could then participate in the long-term extension (LTE) period

### Aims

Evaluate the efficacy and safety of rilzabrutinib in adults with persistent/chronic ITP in the phase 3 LUNA3 LTE.

### Methods

LTE-eligible pts required platelet counts of ≥50x$10^9$/L or ≥30x$10^9$/L and doubled from baseline at ≥50% of visits without rescue therapy during the last 8 weeks of the 28-week OL period. Oral rilzabrutinib 400 mg bid was given in the LTE until the last patient was treated in the LTE period for 12 months (study conclusion). Allowed stable baseline concomitant corticosteroids (CS) and/or thrombopoietin-receptor agonists (TPO-RA) could be dose-reduced and/or discontinued.

**Copyright Information:** (Online) ISSN: 2572-9241
©2026 The Author(s). HemaSphere is published by John Wiley & Sons Ltd on behalf of European Hematology Association. This is an open access Abstract Book distributed under the Attribution-NonCommercial-NoDerivs (CC BY-NC-ND), which allows third parties to download the articles and share them with others as long as they credit the author and the Abstract Book, but they cannot change the content in any way or use them commercially.
**Abstract Book Citations:** Authors, Title, HemaSphere, 2026;10:(S1):pages. The abstract book DOIs can be found on the "Author Information" page.
**Disclaimer:** Articles published in the journal HemaSphere exclusively reflect the opinions of the authors. The authors are responsible for all content in their abstracts including accuracy of the facts, statements, citing resources, etc.

**Results**

Of 69 eligible pts entering the LTE on rilzabrutinib (original randomized DB arm: n=48 rilzabrutinib, n=21 placebo), 52 (75%) pts completed the LTE period and 17 (25%) prematurely
discontinued the LTE period. At baseline, median age was 52 years (range, 18-80), 72% were female, median duration of ITP was 9.6 years (range, 0.3-42.7), median number of unique prior therapies was 4 (range, 1-9; 26% splenectomy), and median DB baseline platelet count was $18x10^9$/L.
Median platelet count at LTE entry (week 53) was $113x10^9$/L (range, $20\text{-}877x10^9$/L), and median platelet counts ranged from $83\text{-}139x10^9$/L for the first 2 years of the LTE (Fig 1A). Platelet counts $\geq 50x10^9$/L or between $30\text{-}50x10^9$/L and doubled from baseline were maintained for a mean of 80% (SD, 33%) weeks during the LTE period. Complete response (platelet count $\geq 100x10^9$/L on 2 consecutive visits) was achieved in 41 (59%) LTE pts. Based on mean changes from baseline (CFB), improvements in physical fatigue (increased per ITP-PAQ item 10, Fig 1B) and ITP Bleeding scores (IBLS, CFB ranged from –0.11 to –0.24) were maintained during the LTE.
For the entire study (DB+OL+LTE), concomitant ITP medication was given in 47/69 (68%) pts. As of LTE end, study discontinuation, or last rilzabrutinib dose, 12/47 (26%) pts had discontinued concomitant medication (CS and/or TPO-RA) and remained on rilzabrutinib monotherapy, 17/34 (50%) pts decreased CS dose >50% from baseline, and 12/27 (44%) reduced their dose from baseline or stopped TPO-RA.
In the LTE period, median duration of rilzabrutinib was 599 days (range, 31-1254), with 99% compliance; 16 (23%) pts received rescue medication. In the LTE period, 15 (22%) pts had treatment-related adverse events (per the investigators); most common were mainly mild: 5 (7%) pts with nausea and 3 (4%) diarrhea. One patient had grade 3 cellulitis (serious adverse event) that resolved with rilzabrutinib dose unchanged. There was no death, thromboembolic event, nor related grade ≥2 bleeding or gastrointestinal events.

**Summary/Conclusion**

Extended rilzabrutinib treatment during the LTE period resulted in sustained platelet responses and maintained improvements in physical fatigue and bleeding scores. A high percentage of pts reduced or discontinued concomitant ITP therapy. Rilzabrutinib continues to be well tolerated, with a favorable safety profile in adults with persistent/chronic ITP.

**Copyright Information:** (Online) ISSN: 2572-9241
©2026 The Author(s). HemaSphere is published by John Wiley & Sons Ltd on behalf of European Hematology Association. This is an open access Abstract Book distributed under the Attribution-NonCommercial-NoDerivs (CC BY-NC-ND), which allows third parties to download the articles and share them with others as long as they credit the author and the Abstract Book, but they cannot change the content in any way or use them commercially.
**Abstract Book Citations:** Authors, Title, HemaSphere, 2026;10:(S1):pages. The abstract book DOIs can be found on the “Author Information” page.
**Disclaimer:** Articles published in the journal HemaSphere exclusively reflect the opinions of the authors. The authors are responsible for all content in their abstracts including accuracy of the facts, statements, citing resources, etc.

**Figure 1. LUNA3 Long-Term Extension Period: Rilzabrutinib Improved Effects on Platelet Count (A) and Physical Fatigue (B; Higher Score=Less Fatigue)**

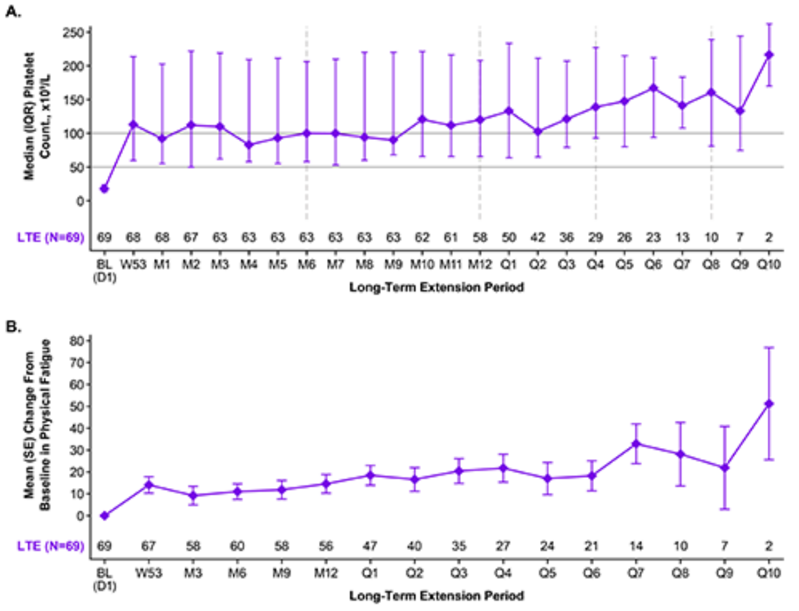

**Copyright Information:** (Online) ISSN: 2572-9241
©2026 The Author(s). HemaSphere is published by John Wiley & Sons Ltd on behalf of European Hematology Association. This is an open access Abstract Book distributed under the Attribution-NonCommercial-NoDerivs (CC BY-NC-ND), which allows third parties to download the articles and share them with others as long as they credit the author and the Abstract Book, but they cannot change the content in any way or use them commercially.
**Abstract Book Citations:** Authors, Title, HemaSphere, 2026;10:(S1):pages. The abstract book DOIs can be found on the "Author Information" page.
**Disclaimer:** Articles published in the journal HemaSphere exclusively reflect the opinions of the authors. The authors are responsible for all content in their abstracts including accuracy of the facts, statements, citing resources, etc.

### (S316) TIME TO PLATELET COUNT RESPONSE AFTER INTRAVENOUS EFGARTIGIMOD IN ADULTS WITH PRIMARY IMMUNE THROMBOCYTOPENIA IN A PHASE 3 CLINICAL TRIAL (ADVANCE IV) AND ITS OPEN-LABEL EXTENSION STUDY (ADVANCE IV+)

**Topic:** 33. Platelet disorders

Monica Carpenedo*[1], Catherine Broome[2], Blanca Sanchez-Gonzalez[3], Vickie McDonald[4], Yoshitaka Miyakawa[5], Kaichi Nishiwaki[6], Francesco Zaja[7], Hanny Al-Samkari[8], Filip Matthijssens[9], Damien Chimits[9], Lan Lan[9], Jaume Ayguasanosa[9], Kristof De Beuf[9], Francesco Rodeghiero[10], Marc Michel[11], Adrian Newland[12]

*[1]ASST Grande Ospedale Metropolitano Niguarda, Department of Hematology Oncology and Molecular Medicine, Milan, Italy; [2]Lombardi Cancer Center, Division of Hematology, Georgetown, United States of America;[3]Hospital del Mar, Department of Hematology, Barcelona, Spain; [4]Barts Health NHS Trust, Department of Clinical Haematology, London, United Kingdom; [5]Saitama Medical University Hospital, Department of Hematology, Saitama, Japan;[6]The Jikei University Kashiwa Hospital, Division of Clinical Oncology/Hematology, Department of Internal Medicine, Chiba, Japan; [7]University of Trieste, Department of Medical, Surgical and Health Sciences, Trieste, Italy; [8]Massachusetts General Hospital, Hematology Division, Boston, United States of America; [9]argenx, Ghent, Belgium; [10]S Bortolo Hospital, Haematology Project Foundation, Department of Haematology, Vicenza, Italy; [11]Henri Mondor University Hospital, Assistance Publique–Hôpitaux de Paris, Department of Internal Medicine, National Reference Center for Immune Cytopenias, Créteil, France; [12]Queen Mary University of London, Centre for Haematology, Barts and the London School of Medicine & Dentistry, London, United Kingdom;*

### Background

In primary immune thrombocytopenia (ITP), immunoglobulin G (IgG) anti-platelet (PLT) autoantibodies contribute to PLT clearance and impair PLT production. Efgartigimod (EFG), a human IgG1 Fc fragment, engineered to prevent recycling of IgG, reduces IgG levels, including pathogenic IgG autoantibodies.

### Aims

To evaluate the time to achieve a platelet count response after IV efgartigimod in adults with ITP.

### Methods

ADVANCE IV, a phase 3, placebo (PBO)-controlled trial (NCT04188379) evaluated EFG IV in adults with persistent/chronic ITP. Participants with an average of 2 screening PLT counts of $<30\times10^9$/L were randomized 2:1 to weekly (wk 1-24) or biweekly (wk 5-24, dependent on PLT count) EFG IV 10 mg/kg or PBO IV for 24 weeks. Participants who completed ADVANCE IV were eligible for the open-label extension study (OLE; ADVANCE IV+) of weekly or biweekly EFG IV (10 mg/kg) here analyzed at a 2-year cut-off. PLT counts were measured weekly. This analysis evaluates time to response in ADVANCE IV, and the pooled participants receiving EFG in ADVANCE IV and/or ADVANCE IV+. Time to achieve 2 consecutive PLT counts $\geq50\times10^9$/L was evaluated as a secondary endpoint in ADVANCE IV and as a post hoc analysis in the pooled block. Time to 2 consecutive PLT counts $\geq100\times10^9$/L was also assessed. Additional PLT count assessments, change from baseline and Kaplan-Meier (KM) analyses were performed post hoc.

**Copyright Information:** (Online) ISSN: 2572-9241
©2026 The Author(s). HemaSphere is published by John Wiley & Sons Ltd on behalf of European Hematology Association. This is an open access Abstract Book distributed under the Attribution-NonCommercial-NoDerivs (CC BY-NC-ND), which allows third parties to download the articles and share them with others as long as they credit the author and the Abstract Book, but they cannot change the content in any way or use them commercially.
**Abstract Book Citations:** Authors, Title, HemaSphere, 2026;10:(S1):pages. The abstract book DOIs can be found on the “Author Information” page.
**Disclaimer:** Articles published in the journal HemaSphere exclusively reflect the opinions of the authors. The authors are responsible for all content in their abstracts including accuracy of the facts, statements, citing resources, etc.

**Results**

Overall, 131 participants were randomized (86 EFG, 45 PBO); 124 participants comprised the pooled block. Least-squares mean estimate changes from baseline in PLT counts were significantly different in for EFG vs PBO starting at day 7 ($22.2\times10^9$/L vs –$0.9\times10^9$/L; *P*=0.0293) and remained significant at most study time points. Based on KM analysis, the proportion of participants with 2 consecutive PLT counts ≥$50\times10^9$/L by day 42 was 31.8% for EFG vs 2.2% for PBO and 35.0% in the pooled block. The proportion of participants with 2 consecutive PLT counts ≥$100\times10^9$/L by day 42 was 16.6% for EFG vs 2.4% for PBO and 19.7% in the pooled block. EFG was well tolerated; most adverse events were mild/moderate.

**Summary/Conclusion**

In ADVANCE IV and the OLE, EFG demonstrated a rapid PLT count increase, as early as 7 days post-EFG. In addition, significantly more sustained responses were observed with EFG versus PBO.

**Copyright Information:** (Online) ISSN: 2572-9241
©2026 The Author(s). HemaSphere is published by John Wiley & Sons Ltd on behalf of European Hematology Association. This is an open access Abstract Book distributed under the Attribution-NonCommercial-NoDerivs (CC BY-NC-ND), which allows third parties to download the articles and share them with others as long as they credit the author and the Abstract Book, but they cannot change the content in any way or use them commercially.
**Abstract Book Citations:** Authors, Title, HemaSphere, 2026;10:(S1):pages. The abstract book DOIs can be found on the "Author Information" page.
**Disclaimer:** Articles published in the journal HemaSphere exclusively reflect the opinions of the authors. The authors are responsible for all content in their abstracts including accuracy of the facts, statements, citing resources, etc.

### (S318) IMPACT OF PROTOCOL-MANDATED DOSE HOLDS ON PLATELET RESPONSE DURABILITY IN PEDIATRIC ITP PATIENTS TREATED WITH AVATROMBOPAG: AVA-301 POST-HOC ANALYSIS

**Topic:** 33. Platelet disorders

Rachael F. Grace[1, 2], Göksel Göksel Leblebisatan2[3], Yesim Aydinok[4], Şule Ünal Cangül[5], John John Grainger[6], Amanda Grimes*[7], Michele Lambert[8], Jessica Zhang[9], Brian Jamieson[9], Chelsea Bernheisel[9], Michael Vredenburg[9], Faizan Sattar[9]

*[1]Dana-Farber/Boston Children's Cancer and Blood Disorders Center, Boston, United States of America;[2]Harvard Medical School, Boston, United States of America; [3]Çukurova University Medical Faculty, Pediatric Hematology, Adana, Türkiye;[4]Ege University School of Medicine, Pediatric Hematology and Oncology, Izmir, Türkiye; [5]Hacettepe University Faculty of Medicine, Pediatric Hematology, Ankara, Türkiye; [6]Royal Manchester Children's Hospital, Department of Haematology, Manchester, United Kingdom; [7]Texas Children's Hospital, Pediatric Hematology and Oncology, Houston, United States of America;[8]Children's Hospital of Philadelphia, Pediatric Hematology and Oncology, Philadelphia, United States of America; [9]Sobi, Inc, Morrisville, United States of America;*

### Background

In the Phase 3b AVA-PED-301 core study, platelet response to avatrombopag (AVA) (PR; ≥2 consecutive platelet counts ≥50×10⁹/L without rescue therapy) was significantly higher compared with placebo (81.5% vs 0%; p<0.0001), despite 73.3% of patients having failed a prior thrombopoietin receptor agonist. Durable platelet response (DR; ≥6 of 8 weekly counts ≥50×10⁹/L in the final 8 weeks) was also higher with AVA (27.8% vs 0%; p=0.0077). Per the study protocol, treatment was held for platelet counts >250×10⁹/L, which is substantially lower than the >400×10⁹/L threshold referenced in the AVA prescribing information, potentially affecting response and durability assessments.

### Aims

To evaluate the incidence, characteristics, and clinical impact of protocol-mandated dose holds during AVA treatment in pediatric ITP.

### Methods

This post-hoc analysis utilized data from the 12-week randomized, double-blind, core phase study of AVA for the treatment of pediatric ITP (AVA n=54; placebo n=21). Dose holds attributable to platelet counts >250×10⁹/L were assessed by frequency, the platelet levels prompting interruption, subsequent platelet declines, and association with DR. Per protocol, AVA was not restarted until platelet counts decreased to ≤100×10⁹/L, at which point therapy resumed one dose level lower.

**Copyright Information:** (Online) ISSN: 2572-9241
©2026 The Author(s). HemaSphere is published by John Wiley & Sons Ltd on behalf of European Hematology Association. This is an open access Abstract Book distributed under the Attribution-NonCommercial-NoDerivs (CC BY-NC-ND), which allows third parties to download the articles and share them with others as long as they credit the author and the Abstract Book, but they cannot change the content in any way or use them commercially.
**Abstract Book Citations:** Authors, Title, HemaSphere, 2026;10:(S1):pages. The abstract book DOIs can be found on the "Author Information" page.
**Disclaimer:** Articles published in the journal HemaSphere exclusively reflect the opinions of the authors. The authors are responsible for all content in their abstracts including accuracy of the facts, statements, citing resources, etc.

**Results**

Overall, 61.1% (33/54) of AVA-treated patients experienced ≥1 dose hold, compared with 4.8% (1/21) of placebo-treated patients. Among the 33 AVA patients with dose holds, 10 achieved a DR and 23 were non-durable responders. Durable responders had fewer dose holds in the final 10 weeks (median 0.8 vs 1.6 per patient), and none experienced multiple dose holds, compared with 56.5% (13/23) of non-durable responders who had >1 dose hold. Following dose holds, rebound thrombocytopenia (PC <50×10⁹/L for ≥15 days) occurred in 45.5% (15/33) of patients (40.0% [4/10] of durable and 47.8% [11/23] of non-durable responders). Median nadir PCs post-dose hold was lower in non-durable than in durable responders (11.5×10⁹/L vs 20.0×10⁹/L).
Among the 23 non-durable responders who accounted for 40 dose holds, 11 had holds triggered solely by platelet counts of 250–400×10⁹/L, 5 by counts >400×10⁹/L (platelet range: 415–1,723 ×10⁹/L), and 7 by counts spanning both ranges. Overall, 62.5% (25/40) of dose holds occurred at platelet levels of 250–400×10⁹/L, which would generally prompt dose reduction rather than interruption in real-world practice. Additionally, 91.3% (21/23) of patients experienced dose cycling (titrating the reduced restart dose of AVA to the pre-hold dose), with a median of 14 days between restart and dose re-escalation.

**Summary/Conclusion**

In the Phase 3b AVA-PED-301 trial, protocol-mandated dose holds at platelet counts >250×10⁹/L significantly impacted whether patients met criteria for durable response. Because AVA could not be restarted until platelet counts fell to ≤100×10⁹/L and was resumed at a lower dose, dose holds frequently produced rebound thrombocytopenia, led to dose cycling, and resulted in loss of durable response. Most dose holds occurred at platelet ranges (250–400×10⁹/L) that would not require interruption under approved dosing recommendations, suggesting that protocol criteria likely underestimated the true durability of response achievable with AVA. Alignment of dose hold criteria with real-world practice may enable more accurate evaluation of AVA's clinical efficacy.

**Copyright Information:** (Online) ISSN: 2572-9241
©2026 The Author(s). HemaSphere is published by John Wiley & Sons Ltd on behalf of European Hematology Association. This is an open access Abstract Book distributed under the Attribution-NonCommercial-NoDerivs (CC BY-NC-ND), which allows third parties to download the articles and share them with others as long as they credit the author and the Abstract Book, but they cannot change the content in any way or use them commercially.
**Abstract Book Citations:** Authors, Title, HemaSphere, 2026;10:(S1):pages. The abstract book DOIs can be found on the "Author Information" page.
**Disclaimer:** Articles published in the journal HemaSphere exclusively reflect the opinions of the authors. The authors are responsible for all content in their abstracts including accuracy of the facts, statements, citing resources, etc.

**Table: Dose hold characteristics in avatrombopag-treated patients with durable and non-durable platelet responses and ≥1 dose hold during the core phase**

| | Durable platelet response (n=10) | Non-durable platelet response (n=23) |
|---|---|---|
| **Dose holds, n** | | |
| Core phase (event count) | 13 | 40 |
| Last 10 weeks of the core phase | 8 | 36 |
| **Dose holds per patient, n** | | |
| Core phase | 1.3 | 1.7 |
| Last 10 weeks of the core phase | 0.8 | 1.6 |
| **Patients with multiple dose holds, n (%)** | | |
| Core phase | 3 (30.0) | 13 (56.5) |
| Last 10 weeks of the core phase | 0 | 12 (52.2) |
| **Patients with post-dose hold PC <50×10$^9$/L for ≥15 days, n (%)** | | |
| Last 10 weeks of the core phase | 4 (40.0) | 11 (47.8) |
| **Time spent with post-dose hold PC <50×10$^9$/L** | | |
| Last 10 weeks of the core phase, n | 4 | 11 |
| Median (Q1, Q3), days | 19.5 (18.0, 21.3) | 25.0 (21.0, 38.0) |
| Mean (SD), days | 19.8 (2.1) | 29.0 (9.9) |
| **Post-dose hold nadir PC, × 10$^9$/L** | | |
| Median (Q1, Q3) | 20.0 (14.0, 25.0) | 11.5 (5.0, 23.5) |
| Mean (SD) | 20.6 (9.4) | 27.3 (54.8) |

**Copyright Information:** (Online) ISSN: 2572-9241
©2026 The Author(s). HemaSphere is published by John Wiley & Sons Ltd on behalf of European Hematology Association. This is an open access Abstract Book distributed under the Attribution-NonCommercial-NoDerivs (CC BY-NC-ND), which allows third parties to download the articles and share them with others as long as they credit the author and the Abstract Book, but they cannot change the content in any way or use them commercially.
**Abstract Book Citations:** Authors, Title, HemaSphere, 2026;10:(S1):pages. The abstract book DOIs can be found on the “Author Information” page.
**Disclaimer:** Articles published in the journal HemaSphere exclusively reflect the opinions of the authors. The authors are responsible for all content in their abstracts including accuracy of the facts, statements, citing resources, etc.

### (S319) EARLY T-CELL RECEPTOR REPERTOIRE FEATURES PREDICT LONG-TERM OUTCOME IN PAEDIATRIC IMMUNE THROMBOCYTOPAENIA

**Topic:** 33. Platelet disorders

Amna Malik*[1], Michelle Tan[1], Alicia Au[1], Eleanor Watt[2], Rocel Saputil[1], Alice Hart[1], Nehal Joshi[1], Stuart Adams[2], Bing Zhang[3], Nichola Cooper[1]

*[1]Imperial, Immunology and Inflammation, London, United Kingdom;[2]Great Ormond Street Hospital for Children, Specialist Integrated Haematology and Malignancy Diagnostic Service–Haematology, London, United Kingdom; [3]Stanford University, Department of Pathology, Stanford, United States of America;*

### Background

Immune thrombocytopaenia (ITP) is an acquired autoimmune bleeding disorder characterised by platelet destruction. Most children follow a self-limiting course, but approximately one-third develop persistent disease. Clinical presentation alone does not reliably predict long-term outcome. Dysregulated T-cell responses have been implicated in ITP pathogenesis, and in adult chronic ITP, we have reported oligoclonal expansion of cytotoxic $CD8^{+}$ T cells. However, whether such repertoire features are present in children at diagnosis and relate to long-term clinical outcome remains unclear.

### Aims

To investigate whether the TCR repertoire at diagnosis can identify children with ITP who will achieve sustained remission, and to define the clonal and transcriptional features that distinguish outcome groups.

### Methods

We studied 52 paediatric patients with primary ITP (23 newly diagnosed and 25 chronic) and 18 age-matched healthy controls from London and Stanford. Bulk TCRβ sequencing (Illumina MiSeq) was performed at diagnosis. Bulk repertoire data were integrated with longitudinal sampling, and single-cell RNA and paired TCR sequencing (10x Genomics) was performed in a subset of children with chronic ITP. Expanded clonotypes were defined as TCR clones comprising >1% of the total TCRβ repertoire. Remission was defined as a sustained platelet count $\geq 100 \times 10^9$/L for ≥12 months without treatment.

**Copyright Information:** (Online) ISSN: 2572-9241
©2026 The Author(s). HemaSphere is published by John Wiley & Sons Ltd on behalf of European Hematology Association. This is an open access Abstract Book distributed under the Attribution-NonCommercial-NoDerivs (CC BY-NC-ND), which allows third parties to download the articles and share them with others as long as they credit the author and the Abstract Book, but they cannot change the content in any way or use them commercially.
**Abstract Book Citations:** Authors, Title, HemaSphere, 2026;10:(S1):pages. The abstract book DOIs can be found on the “Author Information” page.
**Disclaimer:** Articles published in the journal HemaSphere exclusively reflect the opinions of the authors. The authors are responsible for all content in their abstracts including accuracy of the facts, statements, citing resources, etc.

### Results

Expanded clonotypes were present in both healthy controls and ITP patients but were more prominent in ITP. T-cell clonal expansions were detected at diagnosis, and repertoire metrics were comparable between newly diagnosed and chronic patients, indicating that this clonal architecture is established at disease onset and maintained over time. Clonal expansions predicted long-term outcome: children without expanded clonotypes at diagnosis uniformly achieved sustained remission, whereas the presence of expanded clonotypes was associated with delayed or absent remission ($p=0.019$). GLIPH2 motif-based analysis at diagnosis identified more TCR similarity groups per patient among those who subsequently achieved remission, with enrichment of virus-associated clonotypes within these groups. This raises the possibility that remission-associated expansions are driven by viral antigens, whereas those in non-remission patients may reflect self-antigen–driven responses.
Longitudinal tracking demonstrated persistence of expanded clonotypes over multiple years in non-remission patients, while remission patients exhibited contraction or loss of expanded clonotypes.
Single-cell analysis in patients with chronic ITP localised expanded clonotypes predominantly to CD8 TEMRA and CD8 $GZMK^+$ compartments. Among these patients, those who had not achieved remission harboured clones in more advanced differentiation states with significantly higher inflammatory and metabolic pathway activity. Cell–cell communication analysis identified MIF signalling as the dominant interaction axis, with twofold higher pathway strength in refractory disease, linking CD4 central memory T cells and $CD14^+$ monocytes with B cells and CD8 effector subsets.

### Summary/Conclusion

These data support that clonal T-cell expansion at diagnosis is associated with long-term outcome in paediatric ITP, and that the persistence, transcriptional phenotype, and intercellular signalling of expanded clones distinguish refractory from remitting disease. These findings support the potential utility of TCR repertoire profiling as a prognostic tool at diagnosis.

**Copyright Information:** (Online) ISSN: 2572-9241
©2026 The Author(s). HemaSphere is published by John Wiley & Sons Ltd on behalf of European Hematology Association. This is an open access Abstract Book distributed under the Attribution-NonCommercial-NoDerivs (CC BY-NC-ND), which allows third parties to download the articles and share them with others as long as they credit the author and the Abstract Book, but they cannot change the content in any way or use them commercially.
**Abstract Book Citations:** Authors, Title, HemaSphere, 2026;10:(S1):pages. The abstract book DOIs can be found on the "Author Information" page.
**Disclaimer:** Articles published in the journal HemaSphere exclusively reflect the opinions of the authors. The authors are responsible for all content in their abstracts including accuracy of the facts, statements, citing resources, etc.

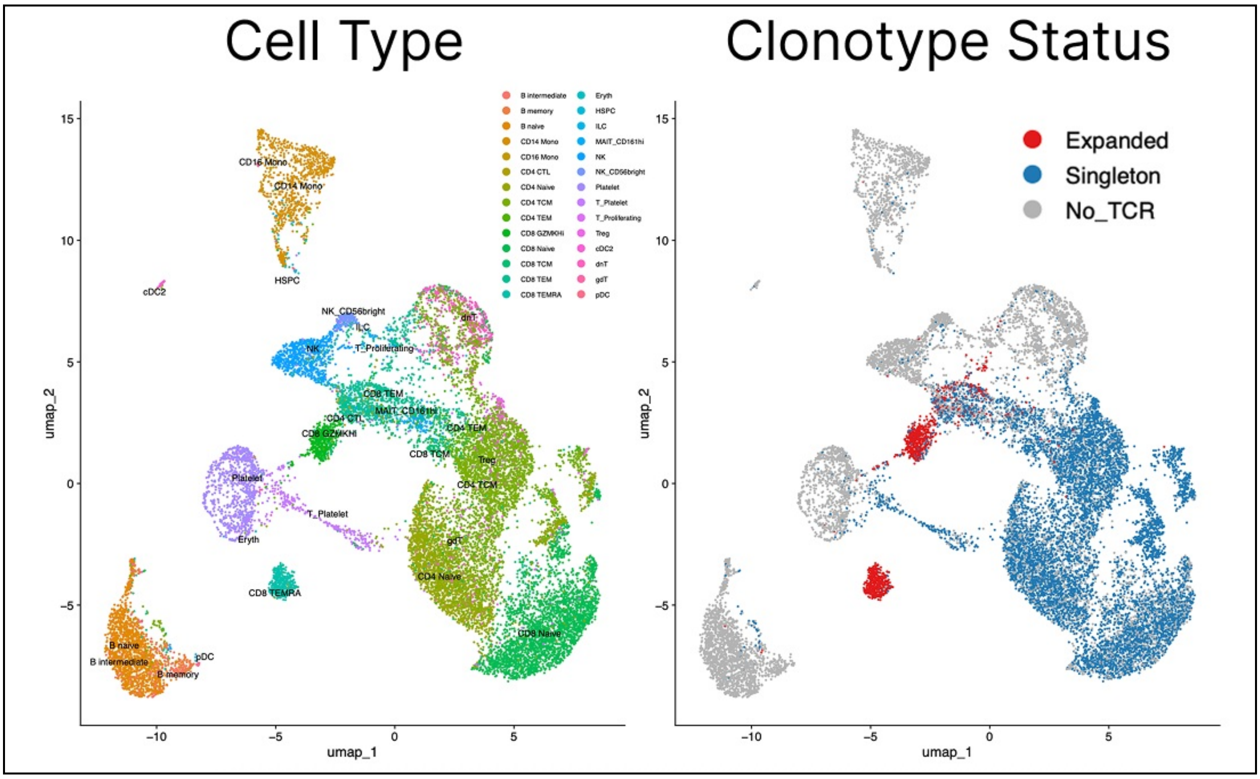

Single-cell phenotype of expanded T-cell clones in a non-remission patient. (A) UMAP visualisation of major immune populations annotated by marker gene expression. (B) Distribution of expanded clonotypes across cell types, showing localisation predominantly to CD8 TEMRA and CD8 GZMK⁺ compartments

**Copyright Information:** (Online) ISSN: 2572-9241
©2026 The Author(s). HemaSphere is published by John Wiley & Sons Ltd on behalf of European Hematology Association. This is an open access Abstract Book distributed under the Attribution-NonCommercial-NoDerivs (CC BY-NC-ND), which allows third parties to download the articles and share them with others as long as they credit the author and the Abstract Book, but they cannot change the content in any way or use them commercially.
**Abstract Book Citations:** Authors, Title, HemaSphere, 2026;10:(S1):pages. The abstract book DOIs can be found on the "Author Information" page.
**Disclaimer:** Articles published in the journal HemaSphere exclusively reflect the opinions of the authors. The authors are responsible for all content in their abstracts including accuracy of the facts, statements, citing resources, etc.

### (S320) UPFRONT CYDRI IS MORE EFFECTIVE FOR THE TREATMENT OF ACQUIRED HEMOPHILIA A THAN TRADITIONAL ORAL IMMUNOSUPPRESSION

**Topic:** 34. Bleeding disorders (congenital and acquired)

Róbert Horváth[1], Ambrus Kaposi[2], Jeroen Eikenboom[3], Roger Schutgens[4], Imre Bodó*[5, 6]

*[1]Semmelweis University, Department of Laboratory Medicine, Budapest, Hungary;[2]ELTE, Faculty of Informatics, Budapest, Hungary; [3]Department of Internal Medicine, Division of Thrombosis and Hemostasis, Leiden, The Netherlands;[4]Department of Hematology, UMC Utrecht, Utrecht, The Netherlands; [5]Semmelweis University, Department of Internal Medicine and Hematology, Budapest, Hungary; [6]Emory University, Department of Hematology and Medical Oncology, Atlanta, United States of America;*

### Background

Acquired hemophilia A (AHA) is a rare, life-threatening autoimmune bleeding disorder with substantial morbidity and mortality. Eradication of the factor VIII inhibitor requires immunosuppression, but the optimal regimen remains undefined. Current international guidelines recommend oral corticosteroids as first-line therapy, with or without oral cyclophosphamide, while additional agents are reserved for steroid failure. Prolonged steroid exposure is highly toxic in this frail population; therefore, alternative strategies are urgently needed.

### Aims

To present the first direct *in silico* comparison of the efficacy and safety of traditional oral sequential immunosuppression and a novel upfront intravenous pulse-dose regimen (Cyclophosphamide, Dexamethasone and Rituximab, CyDRi) in AHA.

### Methods

Raw data from two comparable AHA cohorts differing primarily in immunosuppressive strategy were analyzed. The **KWARK** cohort (*Am J Hematol* 2021;96:51–59) included Dutch patients diagnosed between 1992 and 2018 (n=143) treated with traditional immunosuppression (prednisolon monotherapy; or prednisolon combined with cyclophosphamide or rituximab) eight patients were excluded due to missing essential data (n=135). The **CyDRi** cohort comprised 59 Hungarian and Polish patients diagnosed between 2009 and 2025 and treated with upfront CyDRi; 32 patients have been previously reported (*Blood* 2022;140:1983–1992). Both cohorts had access to identical bypassing agents.
To enhance comparability, inverse probability of treatment weighting (IPTW), a form of *in silico* randomization for time-to-event analyses, was applied using eight baseline variables: age, sex, idiopathic AHA, postpartum AHA, time from first symptom to diagnosis, baseline FVIII activity, baseline inhibitor titer, and severe bleeding at presentation.
Primary endpoints were overall survival (OS) and time to complete remission (TTCR). Complete remission (CR) was defined as FVIII >70 IU/dL off immunosuppression. Secondary endpoints included 2-year survival and CR rates, bleeding-related mortality, treatment-related mortality, and toxicity.

**Copyright Information:** (Online) ISSN: 2572-9241
©2026 The Author(s). HemaSphere is published by John Wiley & Sons Ltd on behalf of European Hematology Association. This is an open access Abstract Book distributed under the Attribution-NonCommercial-NoDerivs (CC BY-NC-ND), which allows third parties to download the articles and share them with others as long as they credit the author and the Abstract Book, but they cannot change the content in any way or use them commercially.
**Abstract Book Citations:** Authors, Title, HemaSphere, 2026;10:(S1):pages. The abstract book DOIs can be found on the "Author Information" page.
**Disclaimer:** Articles published in the journal HemaSphere exclusively reflect the opinions of the authors. The authors are responsible for all content in their abstracts including accuracy of the facts, statements, citing resources, etc.

**Results**

Baseline characteristics were comparable. Mean age was 67.6 vs 71.2 years, and female sex 48.1% vs 52.5% in the KWARK and CyDRi cohorts, respectively. Median baseline FVIII activity (2 vs 1 IU/dL) and inhibitor titers (20 vs 25.5 BU) were similar; residual imbalances were addressed by IPTW. Median follow-up was 17.5 vs 26.1 months.
Kaplan–Meier analyses demonstrated highly significant differences in both OS and TTCR. Two-year overall survival was markedly higher with CyDRi (**93.2% vs 68.9%**), and TTCR was significantly shorter. Results were unchanged when CyDRi was compared with KWARK patients treated after October 2009. KWARK patients diagnosed before and after October 2009 showed similar 2-year mortality (26.2% vs 33.3%), supporting that differences were attributable to immunosuppressive strategy. Two-year CR rates were **54.8% and 91.5%**, respectively.

**Summary/Conclusion**

This first direct *in silico* comparison demonstrates that upfront intravenous pulse-dose CyDRi immunosuppression is substantially superior to traditional oral sequential prednisone-based regimens for acquired hemophilia A. Despite continued guideline endorsement of oral steroid-based therapy, CyDRi achieved faster remissions and significantly improved survival. These findings may be practice-changing and warrant urgent consideration by the international AHA community.

**Copyright Information:** (Online) ISSN: 2572-9241
©2026 The Author(s). HemaSphere is published by John Wiley & Sons Ltd on behalf of European Hematology Association. This is an open access Abstract Book distributed under the Attribution-NonCommercial-NoDerivs (CC BY-NC-ND), which allows third parties to download the articles and share them with others as long as they credit the author and the Abstract Book, but they cannot change the content in any way or use them commercially.
**Abstract Book Citations:** Authors, Title, HemaSphere, 2026;10:(S1):pages. The abstract book DOIs can be found on the “Author Information” page.
**Disclaimer:** Articles published in the journal HemaSphere exclusively reflect the opinions of the authors. The authors are responsible for all content in their abstracts including accuracy of the facts, statements, citing resources, etc.

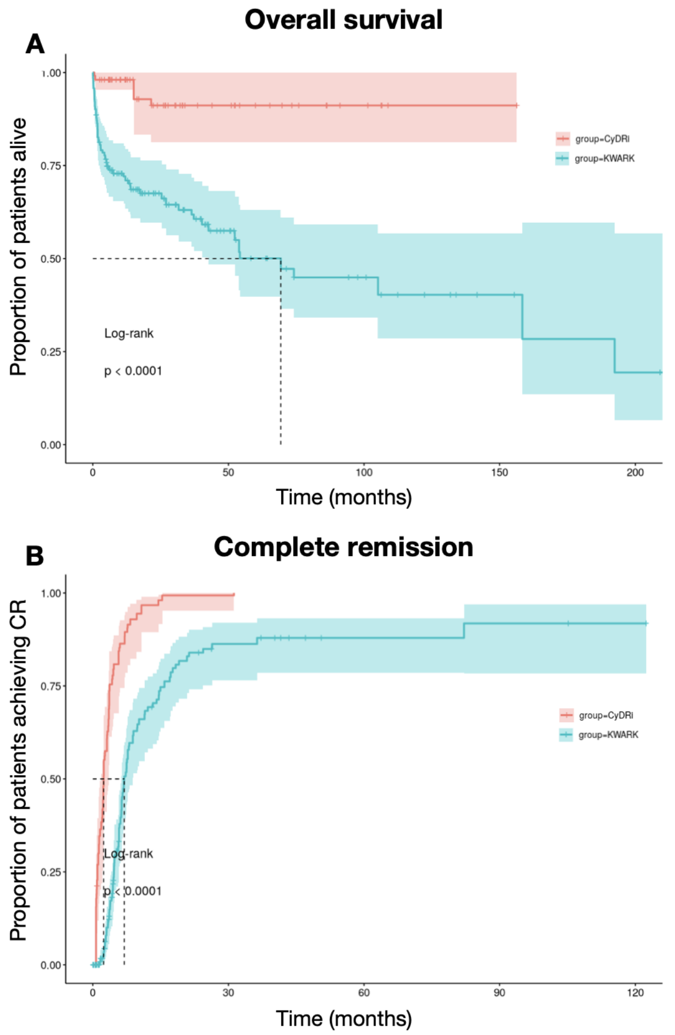

**Overall survival** (**A**) and **Time to complete remission** with initial immunosuppression (**B**). Kaplan-Meier curves for the KWARK (n=135, light green) and CyDRi (n=59, red) cohorts. Median follow-up was **17.5** (IQR: 4.7-43.0) months for the KWARK cohort, and **26.1** (IQR: 10.2-52.3) for the CyDRi cohort.

**Copyright Information:** (Online) ISSN: 2572-9241
©2026 The Author(s). HemaSphere is published by John Wiley & Sons Ltd on behalf of European Hematology Association. This is an open access Abstract Book distributed under the Attribution-NonCommercial-NoDerivs (CC BY-NC-ND), which allows third parties to download the articles and share them with others as long as they credit the author and the Abstract Book, but they cannot change the content in any way or use them commercially.
**Abstract Book Citations:** Authors, Title, HemaSphere, 2026;10:(S1):pages. The abstract book DOIs can be found on the "Author Information" page.
**Disclaimer:** Articles published in the journal HemaSphere exclusively reflect the opinions of the authors. The authors are responsible for all content in their abstracts including accuracy of the facts, statements, citing resources, etc.

### (S321) DYSREGULATED CELLULAR CROSSTALK BETWEEN CD4+ T AND B CELLS IN ACQUIRED HEMOPHILIA A REVEALED BY SINGLE-CELL ANALYSIS

**Topic:** 34. Bleeding disorders (congenital and acquired)

Enhao Li*[1, 2], Haoyang Wu[1, 2], Jinzeng Wang[1], Yilun Xue[1], Chihao Zhang[1], Wenman Wu[1, 2], Xuefeng Wang[1, 2], Jing Dai[1, 2]

*[1]Ruijin Hospital, Shanghai Jiao Tong University School of Medicine, Department of Laboratory Medicine, Shanghai, China;*
*[2]College of Health Science and Technology, Shanghai Jiao Tong University School of Medicine, Shanghai, China;*

### Background

Acquired hemophilia A (AHA) is an autoimmune bleeding disorder characterized by the development of neutralizing autoantibodies against coagulation factor VIII (FVIII), leading to a severe deficiency in FVIII activity. Its therapeutic strategy primarily consists of hemostatic management and immunosuppressive therapy. The efficacy of immunosuppressive therapy varies widely among patients. Hence, defining the immune profile at onset is thus critical to understanding the pathogenesis of AHA.

### Aims

We aim to identify the pathogenic immune signature at disease onset by uncovering aberrant shifts in cell subsets and their functional status, thereby elucidating the critical crosstalk that contributes to the development of AHA.

### Methods

Single-cell RNA sequencing was performed on PBMCs from 9 newly diagnosed, treatment-naïve AHA patients and 5 healthy controls. Raw data were pre-processed with CellRanger, followed by downstream analyses using “Seurat” and “CellChat” R packages in R 4.4.1.

### Results

Integrated single-cell analysis of 124,715 QC-passed cells identified 15 initial clusters (Fig. 1A). Subsequent lineage-focused sub-clustering resolved 19 T/NK subsets from 72,385 cells (Fig. 1B) and pinpointed disease-specific CD4$^+$ T and B cell subsets driving pathological anti-FVIII autoimmunity (Fig.1C/J). In patients with AHA, naïve CD4$^+$ T cells (CD4 T_1) were diminished, while activated (CD4 T_7 & CD4 T _9), effector (CD4 T_10), and memory (CD4 T_11) T cell subsets were expanded, indicating systemic immune hyperactivation and a loss of naïve cell reserve (Fig.1D-H). Differential analysis revealed divergent transcriptional profiles across CD4$^+$ T cell subsets. Pan-subset upregulation of activation markers (CD69, FOS, JUN, NFKBIA, NFKBIZ) was observed, consistent with a heightened activation state (Fig.1I). Collectively, these quantitative and molecular shifts define a comprehensively activated CD4$^+$ T cell state in patients with AHA. Compared to controls, patients with AHA exhibit a reshaping of the B cell repertoire, marked by the expansion of hyperactivated, homeostatic/IgE memory, and pre-plasma cell clusters (6,7,8,9) and the contraction of plasmablast and naïve subsets (3,11,12), indicating a pathogenic bias toward memory phenotypes and a disruption in terminal differentiation (Fig.1K-Q). Multiple B cell subsets in patients with AHA showed upregulation of activation markers such as CD69, CD83, and NF-κB-related genes, consistent with elevated B cell activity. In contrast, two key costimulatory molecules for B cell activation, HLA-DQA2 and CD81, were broadly downregulated, pointing to a defect in the CD4$^+$ T cell-dependent physiological B cell activation program in patients with AHA. Cell-cell interaction analysis quantified the interaction strength between each CD4$^+$ T and B cell subset across groups (Fig. 1S). Notably, B cell subsets exerted a stronger influence on CD4$^+$ T cell subsets than the reverse in patients with AHA, positioning B cells as potential key drivers in the pathological crosstalk (Fig. 1T).

**Copyright Information:** (Online) ISSN: 2572-9241
©2026 The Author(s). HemaSphere is published by John Wiley & Sons Ltd on behalf of European Hematology Association. This is an open access Abstract Book distributed under the Attribution-NonCommercial-NoDerivs (CC BY-NC-ND), which allows third parties to download the articles and share them with others as long as they credit the author and the Abstract Book, but they cannot change the content in any way or use them commercially.
**Abstract Book Citations:** Authors, Title, HemaSphere, 2026;10:(S1):pages. The abstract book DOIs can be found on the “Author Information” page.
**Disclaimer:** Articles published in the journal HemaSphere exclusively reflect the opinions of the authors. The authors are responsible for all content in their abstracts including accuracy of the facts, statements, citing resources, etc.

**Summary/Conclusion**

Our study revealed a systemic disruption in the homeostasis of B cells and their helper $CD4^+$ T cells, the two essential cellular components of humoral immunity. Thus, the reshaped humoral immune landscape in patients with AHA presents a crucial new focus for deciphering AHA pathogenesis and developing potential targeted therapies.

**Copyright Information:** (Online) ISSN: 2572-9241
©2026 The Author(s). HemaSphere is published by John Wiley & Sons Ltd on behalf of European Hematology Association. This is an open access Abstract Book distributed under the Attribution-NonCommercial-NoDerivs (CC BY-NC-ND), which allows third parties to download the articles and share them with others as long as they credit the author and the Abstract Book, but they cannot change the content in any way or use them commercially.
**Abstract Book Citations:** Authors, Title, HemaSphere, 2026;10:(S1):pages. The abstract book DOIs can be found on the "Author Information" page.
**Disclaimer:** Articles published in the journal HemaSphere exclusively reflect the opinions of the authors. The authors are responsible for all content in their abstracts including accuracy of the facts, statements, citing resources, etc.

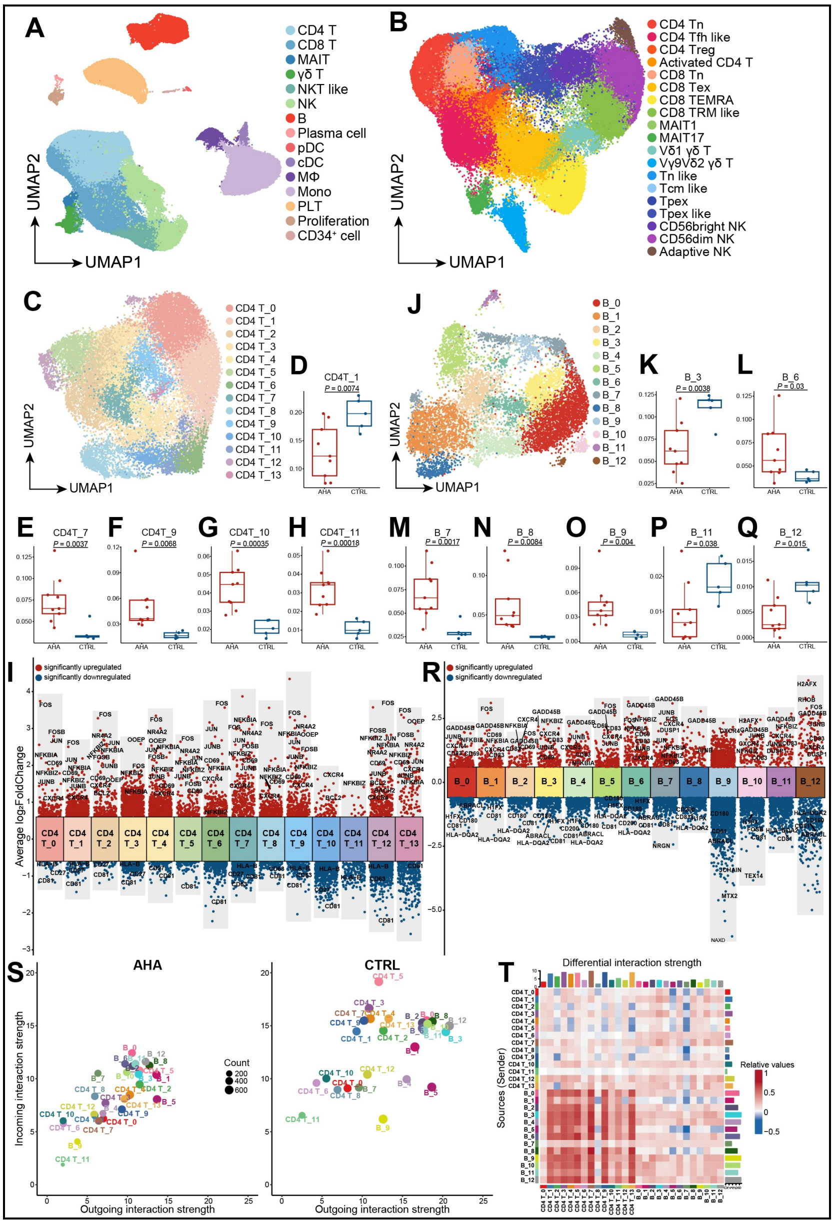

Figure 1. scRNA-seq profiling reveals systemic immune cell alterations in patients with AHA. (A) Cellular composition and clustering of PBMCs visualized through uniform manifold approximation and projection (UMAP). (B) The re-clustering of T and NK cells isolated from PBMC clusters. (C) Further sub-clustering of CD4+ T cells isolated from the T/NK subsets. (D-H) Significantly differential abundance of CD4+ T cell subsets between patients with AHA and healthy controls (CTRL). (I) The combined volcano plot showed the differentially expressed genes (DEGs) of each CD4+ T cell subset between the two groups. (J) Further sub-clustering of B cells isolated from the PBMC clusters. (K-Q) Significantly differential abundance of B cell subsets between patients with AHA and controls. (R) The combined volcano plot showed the differentially expressed genes (DEGs) of each B cell subset between the two groups. (S) The two-dimension plot showed the interaction strength of each CD4+ T and B cell subset in each group. (T) The differential strength of the interactions of CD4+ T with B cell subsets in AHA group comparing to CTRL visualized through the heatmap.

**Copyright Information:** (Online) ISSN: 2572-9241
©2026 The Author(s). HemaSphere is published by John Wiley & Sons Ltd on behalf of European Hematology Association. This is an open access Abstract Book distributed under the Attribution-NonCommercial-NoDerivs (CC BY-NC-ND), which allows third parties to download the articles and share them with others as long as they credit the author and the Abstract Book, but they cannot change the content in any way or use them commercially.
**Abstract Book Citations:** Authors, Title, HemaSphere, 2026;10:(S1):pages. The abstract book DOIs can be found on the “Author Information” page.
**Disclaimer:** Articles published in the journal HemaSphere exclusively reflect the opinions of the authors. The authors are responsible for all content in their abstracts including accuracy of the facts, statements, citing resources, etc.

### (S322) HEAVY MENSTRUAL BLEEDING IN MILD-TO-MODERATE BLEEDING DISORDERS: INVESTIGATING PREVALENCE AND RISK-FACTORS USING MACHINE LEARNING

**Topic:** 34. Bleeding disorders (congenital and acquired)

Tim Dreier*[1], Dino Mehic[1], Justin Oosterlee[1], Cihan Ay[1], Ingrid Pabinger[1], Johanna Gebhart[1]

[1]*Medical University Vienna, Department of Medicine I, Vienna, Austria;*

### Background

Mild-to-moderate bleeding disorders (MBD), including bleeding disorder of unknown cause (BDUC), platelet function defects (PFD), von Willebrand disease (VWD), and coagulation factor deficiencies (CFD), often manifest as life-long mucocutaneous bleeding, easy bruising and bleeding complications after haemostatic challenges. However, heavy menstrual bleeding (HMB) is also highly prevalent among female MBD patients. Limited data on the prevalence and risk-factors of HMB in heterogenous MBD patient cohorts is currently available.

### Aims

We aimed to comprehensively analyse the prevalence of HMB across different MBD diagnoses and to identify laboratory features associated with HMB.

### Methods

Female MBD patients were selected from the Vienna Bleeding Biobank, a prospective, single-centre cohort study that recruits individuals with a life-long, clinically relevant bleeding tendency who have not undergone prior haemostatic evaluation. Upon enrolment, all patients undergo a standardised haemostatic screening for VWD, PFD, and relevant CFD. Patients who test negative for all assessed conditions are classified as BDUC. The individual bleeding severity was quantified using the ISTH-BAT.
Pairwise comparisons were conducted using the Fisher exact test, corrected according to Bonferroni-Holm. TabPFN v2.5, a transformer-based pre-trained neural network optimised for small tabular data, was used to screen a panel of routine haemostatic and biochemical laboratory parameters measured at study inclusion (n=54), as well as selected patient characteristics (age, blood group (O vs. non-O), weight, and height) for their ability to predict HMB. Model performance was evaluated by the out-of-fold area under the receiver operating characteristic curve (AUC) using 10-fold cross-validation repeated 5 times, with bootstrapped 95% confidence intervals (CI, n=2000).

### Results

885 female MBD patients were analysed, with a median ISTH-BAT score of 6 [CI 4-9]. Of these, 558 (63%) were classified as BDUC, 252 (29%) had PFD, 64 (7%) had VWD, and the remaining 11 (1%) had CFD. BDUC patients showed the lowest prevalence of HMB in 357/558 (64%), while PFD patients reported HMB significantly more often in comparison to BDUC in 186/252 (74%, pfisher=0.036). 48 of 64 VWD patients (75%) and 9 of 11 CFD patients (82%) also reported HMB, however without statistical significance in comparison to BDUC or any other diagnosis. These findings remained consistent in a sensitivity analysis restricted to patients of menstrual age (<55 years, n=720) to account for potential recall bias.
The TabPFN model achieved a statistically significant but poor discriminative ability with an AUC of 0.60 (CI: 0.56–0.64, per-fold SD: 0.053). Model diagnostics confirmed limited overall predictive performance but adequate calibration and no evidence of overfitting (Brier score: 0.214 [0.20–0.23], ECE: 0.042, log loss: 0.618; calibration intercept: −0.220, slope: 1.204). Again, this was reproducible in a sensitivity analysis including female patients of menstrual age only (AUC 0.618, CI: 0.574–0.661) and BDUC patients only (AUC 0.58, CI: 0.54–0.63).

**Copyright Information:** (Online) ISSN: 2572-9241
©2026 The Author(s). HemaSphere is published by John Wiley & Sons Ltd on behalf of European Hematology Association. This is an open access Abstract Book distributed under the Attribution-NonCommercial-NoDerivs (CC BY-NC-ND), which allows third parties to download the articles and share them with others as long as they credit the author and the Abstract Book, but they cannot change the content in any way or use them commercially.
**Abstract Book Citations:** Authors, Title, HemaSphere, 2026;10:(S1):pages. The abstract book DOIs can be found on the "Author Information" page.
**Disclaimer:** Articles published in the journal HemaSphere exclusively reflect the opinions of the authors. The authors are responsible for all content in their abstracts including accuracy of the facts, statements, citing resources, etc.

**Summary/Conclusion**

The overall similar prevalence of HMB across BDUC, VWD, and CFD suggests that HMB is largely independent of the underlying haemostatic diagnosis, which may explain the limited predictive value of laboratory parameters. The higher prevalence in PFD might highlight role of platelet function in endometrial haemostasis.

**Copyright Information:** (Online) ISSN: 2572-9241
©2026 The Author(s). HemaSphere is published by John Wiley & Sons Ltd on behalf of European Hematology Association. This is an open access Abstract Book distributed under the Attribution-NonCommercial-NoDerivs (CC BY-NC-ND), which allows third parties to download the articles and share them with others as long as they credit the author and the Abstract Book, but they cannot change the content in any way or use them commercially.
**Abstract Book Citations:** Authors, Title, HemaSphere, 2026;10:(S1):pages. The abstract book DOIs can be found on the "Author Information" page.
**Disclaimer:** Articles published in the journal HemaSphere exclusively reflect the opinions of the authors. The authors are responsible for all content in their abstracts including accuracy of the facts, statements, citing resources, etc.

### (S323) DEVELOPMENT OF A CATALYTICALLY INACTIVE BIVALENT FVII-FC FUSION PROTEIN FOR SELECTIVE MODULATION OF TF–FVIIA INITIATION

**Topic:** 34. Bleeding disorders (congenital and acquired)

Nan Zhang[1], Xi Quan[1], Jian-Chuan Deng[1], Shifeng Lou*[1]

[1] *The Second Affiliated Hospital of Chongqing Medical University, Department of hematology, Chongqing, China;*

### Background

The tissue factor (TF)–factor VIIa (FVIIa) complex serves as the primary initiator of coagulation and plays a critical role in thromboinflammatory disorders, including sepsis, malignancy-associated coagulopathy, and disseminated intravascular coagulation (DIC). Current anticoagulant strategies predominantly target downstream proteases, which may compromise physiological hemostasis and increase bleeding risk. A selective approach that modulates TF-driven initiation without directly inhibiting catalytic coagulation activity remains an unmet need.

### Aims

To develop and functionally characterize a catalytically inactive, bivalent FVII-based fusion protein designed to selectively engage the TF–FVIIa initiation complex.

### Methods

A rationally engineered FVII(1–211)-Fc fusion construct was generated by removing the entire protease domain while preserving structural regions responsible for TF interaction. An engineered IgG1 Fc domain was incorporated to enable dimerization and enhance molecular stability while minimizing effector function. Recombinant proteins were expressed in mammalian cells and purified by protein A chromatography. Structural integrity was assessed by electrophoretic and size-exclusion analyses. TF binding capacity and coagulation-related activity were evaluated using TF-based functional assays, FX activation assays, thrombin generation assays, and plasma clotting measurements under TF stimulation.

### Results

The fusion protein was successfully expressed and purified as a stable, predominantly dimeric species with minimal aggregation. Structural analyses confirmed appropriate assembly and molecular stability. The construct retained functional TF-binding capability while demonstrating complete loss of catalytic activity toward factor X, consistent with elimination of the protease domain. In competitive settings, FVII(1–211)-Fc dose-dependently reduced TF–FVIIa-mediated FX activation and attenuated downstream thrombin generation under TF-driven conditions. Importantly, baseline clotting parameters were minimally affected in the absence of excessive TF stimulation, indicating selective modulation of the initiation phase rather than global anticoagulation. These findings were reproducible across independent preparations.

### Summary/Conclusion

We report the development of a catalytically inactive, bivalent FVII-Fc fusion protein that selectively targets the TF–FVIIa initiation complex while lacking intrinsic proteolytic activity. This strategy introduces a novel approach for modulating TF-driven coagulation activation with a mechanistically selective profile distinct from conventional anticoagulants, supporting further translational investigation in TF-associated thromboinflammatory conditions.

**Copyright Information:** (Online) ISSN: 2572-9241
©2026 The Author(s). HemaSphere is published by John Wiley & Sons Ltd on behalf of European Hematology Association. This is an open access Abstract Book distributed under the Attribution-NonCommercial-NoDerivs (CC BY-NC-ND), which allows third parties to download the articles and share them with others as long as they credit the author and the Abstract Book, but they cannot change the content in any way or use them commercially.
**Abstract Book Citations:** Authors, Title, HemaSphere, 2026;10:(S1):pages. The abstract book DOIs can be found on the "Author Information" page.
**Disclaimer:** Articles published in the journal HemaSphere exclusively reflect the opinions of the authors. The authors are responsible for all content in their abstracts including accuracy of the facts, statements, citing resources, etc.

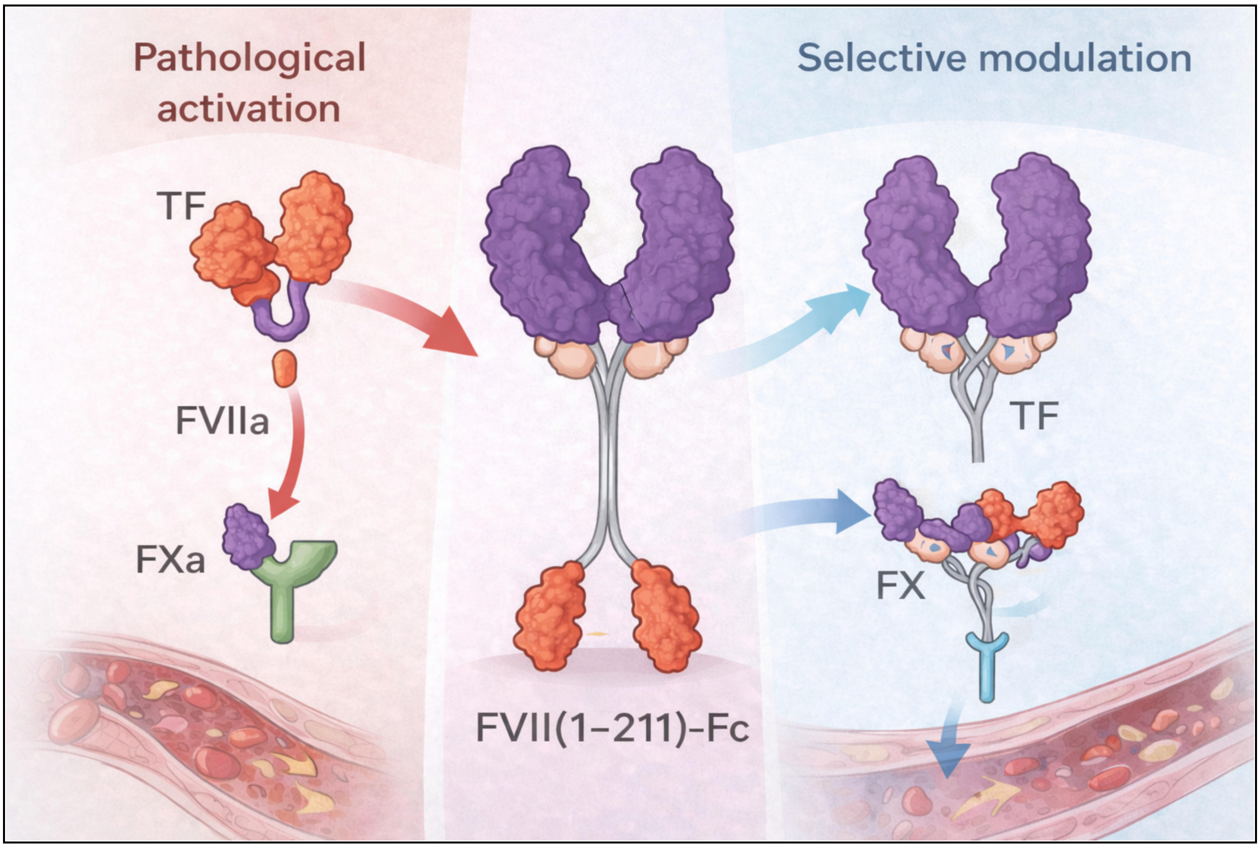

Schematic illustration of TF-driven coagulation initiation and selective modulation by an engineered FVII(1–211)-Fc fusion protein. Left (Pathological activation): Tissue factor (TF) binds endogenous activated factor VII (FVIIa) to form the TF–FVIIa complex, which activates factor X (FX → FXa), initiating downstream thrombin generation and coagulation amplification. Center: The bivalent FVII(1–211)-Fc fusion protein retains TF-binding domains but lacks the protease domain, rendering it catalytically inactive. Right (Selective modulation): By competitively occupying TF, FVII(1–211)-Fc reduces TF–FVIIa–mediated FX activation under TF-stimulated conditions, attenuating excessive initiation while preserving basal hemostasis.

**Copyright Information:** (Online) ISSN: 2572-9241
©2026 The Author(s). HemaSphere is published by John Wiley & Sons Ltd on behalf of European Hematology Association. This is an open access Abstract Book distributed under the Attribution-NonCommercial-NoDerivs (CC BY-NC-ND), which allows third parties to download the articles and share them with others as long as they credit the author and the Abstract Book, but they cannot change the content in any way or use them commercially.
**Abstract Book Citations:** Authors, Title, HemaSphere, 2026;10:(S1):pages. The abstract book DOIs can be found on the "Author Information" page.
**Disclaimer:** Articles published in the journal HemaSphere exclusively reflect the opinions of the authors. The authors are responsible for all content in their abstracts including accuracy of the facts, statements, citing resources, etc.

### (S324) MORTALITY TRENDS OF HEMATOLOGICAL MALIGNANCIES AND THOSE COMPLICATED BY PULMONARY EMBOLISM: A NATIONWIDE POPULATION-BASED STUDY

**Topic:** 35. Thrombosis and vascular biology

Xi Quan*[1], Zhiming Luo[1], Nan Zhang[1], Shifeng Lou[1]

[1] *The Second Affiliated Hospital of Chongqing Medical University, Department of Hematology, Chongqing, China;*

### Background

Pulmonary embolism (PE) is a common and fatal clinical condition, for which timely treatment may be life-saving. The incidence of acute PE in the general population of North America is estimated to be 70 to 110 per 100,000 individuals. PE represents the most severe clinical manifestation of VTE and may lead to serious adverse events, such as heart failure, respiratory failure, and sudden death. Cancer is an established cause of VTE and exacerbates the factors contributing to VTE formation: venous stasis, endothelial injury, and hypercoagulability (Virchow's triad). Due to the hypercoagulable state associated with cancer, PE is a major cause of mortality in cancer patients, and VTE (including PE) is the second leading cause of death in cancer patients. Multiple studies have reported a significantly increased risk of VTE in cancer patients compared with the general population. However, most of the literature has focused on solid tumors and has not included hematological malignancies.

### Aims

The aim of this study was to analyze the mortality trends of hematological malignancies and those complicated by pulmonary embolism (PE) in the United States from 1999 to 2023. We also explores the mortality changes across multiple dimensions, including gender, age, region, urban-rural differences, and race.

### Methods

Using death certificate data from the Centers for Disease Control's Wide-ranging Online Data for Epidemiologic Research (CDC WONDER) database, we analyzed the age-adjusted mortality rates (AAMR) of hematological malignancies and hematological malignancies with PE among individuals aged 25 and above in the United States from 1999 to 2023. We assessed mortality trends across different genders, age groups, regions, urban-rural differences, and racial groups by calculating the average annual percentage change (AAPC) and annual percentage change (APC).

### Results

From 1999 to 2023, the AAMR of hematological malignancies among individuals aged 25 and above in the United States showed a steady decline, with an AAPC of -1.75 (95% confidence interval (CI): -1.81 to -1.69), reflecting the positive achievements in the diagnosis, treatment, and management of hematological malignancies. However, the mortality rate of hematological malignancies with PE showed a distinctly different upward trend. The mortality rate increased with a low slope from 1999 to 2017 (APC 0.75, 95% CI 0.17–1.33), followed by a significant acceleration from 2017 to 2023 (APC 3.02, 95% CI 0.51–5.58), indicating a sharp increase in the mortality burden during these six years. Mortality rates for hematological malignancies with PE increased across gender, age, region, urban-rural differences, and race, particularly among males, the Western region, and individuals aged 75 and above. Higher mortality rates were also observed in non-metropolitan areas and among non-Hispanic Black individuals.

**Copyright Information:** (Online) ISSN: 2572-9241
©2026 The Author(s). HemaSphere is published by John Wiley & Sons Ltd on behalf of European Hematology Association. This is an open access Abstract Book distributed under the Attribution-NonCommercial-NoDerivs (CC BY-NC-ND), which allows third parties to download the articles and share them with others as long as they credit the author and the Abstract Book, but they cannot change the content in any way or use them commercially.
**Abstract Book Citations:** Authors, Title, HemaSphere, 2026;10:(S1):pages. The abstract book DOIs can be found on the "Author Information" page.
**Disclaimer:** Articles published in the journal HemaSphere exclusively reflect the opinions of the authors. The authors are responsible for all content in their abstracts including accuracy of the facts, statements, citing resources, etc.

**Summary/Conclusion**

Despite a significant decline in the overall mortality rate of patients with hematological malignancies over the past 24 years, the mortality rate of patients with hematological malignancies complicated by PE has shown an upward trend, with significant differences across different genders, age groups, regions, urban-rural differences, and race. Future research and public health policies need to focus on these differences and develop targeted intervention strategies to reduce the mortality rate of PE in such patients and optimize prognosis.

**Copyright Information:** (Online) ISSN: 2572-9241
©2026 The Author(s). HemaSphere is published by John Wiley & Sons Ltd on behalf of European Hematology Association. This is an open access Abstract Book distributed under the Attribution-NonCommercial-NoDerivs (CC BY-NC-ND), which allows third parties to download the articles and share them with others as long as they credit the author and the Abstract Book, but they cannot change the content in any way or use them commercially.
**Abstract Book Citations:** Authors, Title, HemaSphere, 2026;10:(S1):pages. The abstract book DOIs can be found on the "Author Information" page.
**Disclaimer:** Articles published in the journal HemaSphere exclusively reflect the opinions of the authors. The authors are responsible for all content in their abstracts including accuracy of the facts, statements, citing resources, etc.

### (S325) ASSESSMENT OF NIPOCALIMAB EFFECTS ON FATIGUE IN WARM AUTOIMMUNE HEMOLYTIC ANEMIA: RESULTS FROM THE DOUBLE-BLIND PHASE 2/3 ENERGY TRIAL

**Topic:** 36. Quality of life, ethics, supportive and palliative care

Irina Murakhovskaya*[1], Bruno Fattizzo[2, 3], Yasutaka Ueda[4], Kayla Scippa[5], Michael Zelasky[6], Cathye Shu[6], Sheryl Pease[5]

*[1]Albert Einstein College of Medicine/Montefiore Medical Center, Bronx, NY, United States of America;[2]Hematology Unit, Fondazione IRCCS Ca' Granda Ospedale Maggiore Policlinico, Milan, Italy; [3]Department of Oncology and Hemato-Oncology, University of Milan, Milan, Italy; [4]The University of Osaka, Suita, Osaka, Japan;[5]Johnson & Johnson, Raritan, NJ, United States of America; [6]Johnson & Johnson, Spring House, PA, United States of America;*

### Background

Warm autoimmune hemolytic anemia (wAIHA) is a rare, life-threatening disease characterized by autoantibody-mediated premature destruction of red blood cells (hemolysis). Patients with wAIHA frequently report fatigue and consider it to be a debilitating symptom associated with their diagnosis. There is currently a high unmet need for an effective treatment to address patients' fatigue and other symptoms impacting health-related quality of life.

### Aims

ENERGY trial (ClinicalTrials.gov identifier: NCT04119050) data were analyzed to assess the impact of intravenous (IV) nipocalimab on fatigue in patients with wAIHA.

### Methods

ENERGY is a phase 2/3 trial evaluating the efficacy and safety of IV nipocalimab in wAIHA. Adults who received ≥1 prior therapy and consented to participate were randomized 1:1:1 to nipocalimab 30 mg/kg q4w, 15 mg/kg q2w, or placebo in the 24-week double-blind period. The primary endpoint was durable hemoglobin (Hgb) response (Hgb ≥10 g/dL and a Hgb increase of ≥2 g/dL for ≥3 consecutive visits [≥28 days], at or before Week 16 [W16]) without the need for rescue therapy. Fatigue was quantified by FACIT-Fatigue (key secondary endpoint), a content-valid patient-reported outcome measure that evaluates wAIHA-related fatigue (Pease S, et al. *J Patient Rep Outcomes*. 2025;9(1):97) and single item Patient Global Impressions of Severity and Change (PGIS and PGIC) measures for fatigue. PGIS and PGIC data were used to determine the meaningful change threshold (MCT) for FACIT-Fatigue using anchor-based analysis. Correlation between Hgb and FACIT-Fatigue was examined. All analyses were prespecified.

**Copyright Information:** (Online) ISSN: 2572-9241
©2026 The Author(s). HemaSphere is published by John Wiley & Sons Ltd on behalf of European Hematology Association. This is an open access Abstract Book distributed under the Attribution-NonCommercial-NoDerivs (CC BY-NC-ND), which allows third parties to download the articles and share them with others as long as they credit the author and the Abstract Book, but they cannot change the content in any way or use them commercially.
**Abstract Book Citations:** Authors, Title, HemaSphere, 2026;10:(S1):pages. The abstract book DOIs can be found on the "Author Information" page.
**Disclaimer:** Articles published in the journal HemaSphere exclusively reflect the opinions of the authors. The authors are responsible for all content in their abstracts including accuracy of the facts, statements, citing resources, etc.

**Results**

Participants were randomized to nipocalimab 30 mg/kg q4w (n=38), 15 mg/kg q2w (n=38) or placebo (n=39). Nipocalimab-treated participants reported rapid improvements in fatigue by W2 (first timepoint of assessment on treatment), measured by mean change from baseline in FACIT-Fatigue total score (**Figure A**). Participants in the nipocalimab 30 mg/kg q4w group maintained improvements through W24. At W24, mean (SD) changes in FACIT-Fatigue total scores were 3.4 (7.3), 1.2 (6.1), and 0.6 (3.4) points for the nipocalimab 30 mg/kg q4w, 15 mg/kg q2w, and placebo groups, respectively; a positive correlation (r=0.33) was observed between change from baseline in Hgb and FACIT-Fatigue. At W24, the nipocalimab 30 mg/kg q4w group had greater numerical mean improvements on the FACIT-Fatigue Experience (1.5) and Impact subscales (1.9) compared to placebo (0.2 and 0.4, respectively).
The MCT in FACIT-Fatigue was determined to be 6 points; at W24, the MCT was achieved by 27%, 19%, and 5% of participants in the nipocalimab 30 mg/kg q4w, 15 mg/kg q2w, and placebo groups, respectively.
A numerically greater percentage of nipocalimab-treated participants had improved PGIS fatigue severity (≥1-point) at W16, 20 and 24 compared to placebo (**Figure B**). The percentage of participants reporting their fatigue on the PGIC to be "much better" at W24 compared to baseline was numerically greater in the nipocalimab-treated groups (38%, 12% and 9% for 30 mg/kg q4w, 15 mg/kg q2w, and placebo, respectively).

**Summary/Conclusion**

In the ENERGY trial, participants treated with nipocalimab reported early and prolonged improvements in fatigue, with the most consistent benefit observed with nipocalimab 30 mg/kg q4w. Fatigue improvement was supported by global patient assessments and showed a modest positive correlation with hemoglobin recovery, highlighting a patient-relevant benefit beyond correction of anemia.

**Figure. (A) Mean Change From Baseline in FACIT-Fatigue[a] Total Scores by Visit[b] and (B) Participants With PGIS Improvement ≥1 Point From Baseline**

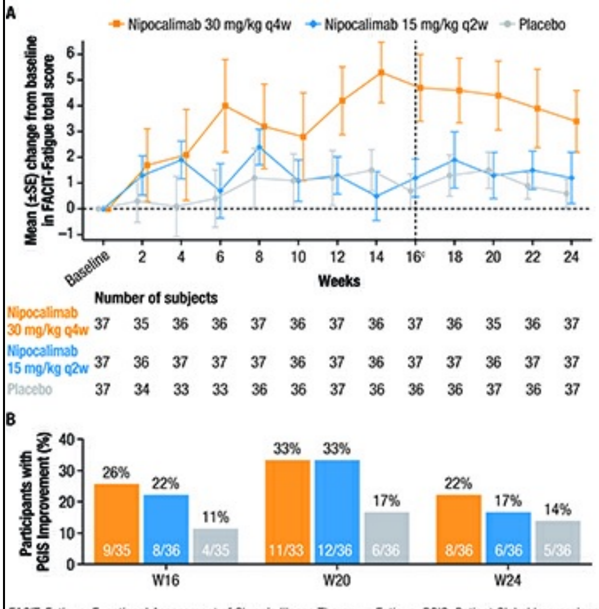

FACIT-Fatigue, Functional Assessment of Chronic Illness Therapy – Fatigue; PGIS, Patient Global Impressions of Severity; q2w, every 2 weeks; q4w, every 4 weeks; W, week.
[a]FACIT-Fatigue scale total score range is 0-52 with higher scores indicating less fatigue.
[b]Analysis was based on a composite strategy where participants with intercurrent events (including any use of rescue therapies or initiation or dose increase of protocol-specified standard-of-care background therapy) were considered nonresponders, with no change from baseline.
[c]To meet the primary endpoint, the first visit satisfying the hemoglobin response criteria needed to occur by W16.

**Copyright Information:** (Online) ISSN: 2572-9241
©2026 The Author(s). HemaSphere is published by John Wiley & Sons Ltd on behalf of European Hematology Association. This is an open access Abstract Book distributed under the Attribution-NonCommercial-NoDerivs (CC BY-NC-ND), which allows third parties to download the articles and share them with others as long as they credit the author and the Abstract Book, but they cannot change the content in any way or use them commercially.
**Abstract Book Citations:** Authors, Title, HemaSphere, 2026;10:(S1):pages. The abstract book DOIs can be found on the "Author Information" page.
**Disclaimer:** Articles published in the journal HemaSphere exclusively reflect the opinions of the authors. The authors are responsible for all content in their abstracts including accuracy of the facts, statements, citing resources, etc.

### (S326) MAPPING RESEARCH AND EQUITY ACROSS EUROPE: FINDINGS FROM THE EUROPEAN ATLAS ON CLINICAL TRIALS IN CANCER AND HAEMATOLOGY (EUROACT)

**Topic:** 36. Quality of life, ethics, supportive and palliative care

Lis Puga*[1], Rachel Gales[2], Attila Imre[3], Merce Cases Escute[1], Maria Piggin[4], Susanna Leto di Priolo[5], Monica Racovita[6], Lora Ruth Wogu[7], Jan Geissler[1], Dalma Hosszú[3], Ákos Józwiák[3], Tamás Ágh[3], Janos Pitter[3], Ananda Plate[1]

*[1]European Patient Advocacy Institute, Riemerling, Germany; [2]VHL Europa, Vlaardingen, The Netherlands; [3]Syreon Research Institute, Budapest, Hungary; [4]PNH Global Alliance, Tiel, The Netherlands; [5]T.U.T.O.R rare thoracic tumours, Milan, Italy; [6]Myeloma Patients Europe, Brussels, Belgium; [7]European Sickle Cell Federation-ESCF, Dublin, Ireland;*

### Background

The European Atlas on Clinical Trials in Cancer and Haematology (EuroACT) analyses the European clinical trial landscape, focusing on geographical distribution, patient-reported outcome measure (PROM) integration, and publication of PROM-related data across malignant and non-malignant haematological diseases.

### Aims

To map trial distribution, PROM integration, and publication gaps across European haematology.

### Methods

A harmonised dataset was constructed from ClinicalTrials.gov (NCT), EudraCT, and CTIS, including observational and interventional phase II–IV trials ongoing or completed by February 2025 with ≥1 European site. Publications were identified via PubMed.

### Results

In total, EuroACT analysed 24,797 trials; fifteen haematological conditions were represented in the database, comprising 6,809 trials (5,204 interventional, 1,605 observational) across the malignant and non-malignant diseases and disorders. Within this haematology subset, 69.5% trials were registered in a single registry, and 5.3% were registered across all three registries.
Geographically, 69.2% of trials were conducted in Western Europe and 47.7% in Southern Europe, compared with 25.3% in Central and Eastern Europe and 17.6% in Northern Europe.
PROMs were utilised in 19.4% (n=1,009) of interventional and 13.0% (n=209) of observational studies. PROM use was lower in paediatric trials (OR 0.48, 95% CI 0.29–0.75; p=0.0020). Among completed trials including PROMs, only one (~0.14%) reported PROM-related data in its publication abstract.
Trial volume was highest in lymphomas (2,832), myeloproliferative neoplasms (2,576), acute leukaemia (1,259), myeloma (986), and myelodysplastic syndromes (624). Lower numbers were observed in chronic lymphocytic leukaemia (520), sickle cell disease (476), haemophilia (449), chronic myeloid leukaemia (361), thalassaemia (321), Waldenström's macroglobulinaemia (293), idiopathic thrombocytopenic purpura (179), AL amyloidosis (64), paroxysmal nocturnal haemoglobinuria (59), and haemochromatosis (56).

**Copyright Information:** (Online) ISSN: 2572-9241
©2026 The Author(s). HemaSphere is published by John Wiley & Sons Ltd on behalf of European Hematology Association. This is an open access Abstract Book distributed under the Attribution-NonCommercial-NoDerivs (CC BY-NC-ND), which allows third parties to download the articles and share them with others as long as they credit the author and the Abstract Book, but they cannot change the content in any way or use them commercially.
**Abstract Book Citations:** Authors, Title, HemaSphere, 2026;10:(S1):pages. The abstract book DOIs can be found on the "Author Information" page.
**Disclaimer:** Articles published in the journal HemaSphere exclusively reflect the opinions of the authors. The authors are responsible for all content in their abstracts including accuracy of the facts, statements, citing resources, etc.

**Summary/Conclusion**

Substantial regional disparities and variability in research volume across haematological diseases persist in Europe. PROM integration and reporting remain limited. EuroACT provides a structured evidence base to support more equitable, transparent, and patient-centred haematology research.

**Copyright Information:** (Online) ISSN: 2572-9241
©2026 The Author(s). HemaSphere is published by John Wiley & Sons Ltd on behalf of European Hematology Association. This is an open access Abstract Book distributed under the Attribution-NonCommercial-NoDerivs (CC BY-NC-ND), which allows third parties to download the articles and share them with others as long as they credit the author and the Abstract Book, but they cannot change the content in any way or use them commercially.
**Abstract Book Citations:** Authors, Title, HemaSphere, 2026;10:(S1):pages. The abstract book DOIs can be found on the "Author Information" page.
**Disclaimer:** Articles published in the journal HemaSphere exclusively reflect the opinions of the authors. The authors are responsible for all content in their abstracts including accuracy of the facts, statements, citing resources, etc.

### (S327) PROGNOSTIC IMPORTANCE OF PRE-TREATMENT HEALTH-RELATED QUALITY OF LIFE AND FITNESS INDICATORS IN OLDER ADULTS WITH ACUTE MYELOID LEUKEMIA. ANALYSIS FROM THE INTERNATIONAL EORTC AML 21 PHASE 3 TRIAL

**Topic:** 36. Quality of life, ethics, supportive and palliative care

Fabio Efficace*[1], Michal Kicinski[2], Daniela Krepper[1, 3], Sylvain Chantepie[4], Walter Van der Velden[5], Richard Noppeney[6], Laimonas Griškevičius[7], Andreas Neubauer[8], Martina Crysandt[9], Radovan Vrhovac[10], Mario Luppi[11], Stephan Fuhrmann[12], Ernesta Audisio[13], Anna Candoni[14], Ollivier Legrand[15], Robin Foà[16], Gianluca Gaidano[17], Eduardus F.M. Posthuma[18], Mels Hoogendoorn[19], Marco Vignetti[1], Corneel Coens[2], Stefan Suciu[2], Stéphanie Antunes[2], Frédéric Baron[20], Pierre Wijermans[21], Adriano Venditti[22], Gerwin Huls[23], Michael Lübbert[24]

*[1]Italian Group for Adult Hematologic Diseases (GIMEMA), Data Center and Health Outcomes Research Unit, Rome, Italy, Rome, Italy; [2]EORTC Headquarters, Brussels, Belgium; [3]University Hospital of Psychiatry II, Medical University of Innsbruck, Innsbruck, Austria; [4]Department of Hematology, Centre Hospitalo-Universitaire de Caen, Caen, France;[5]Department of Hematology, Radboud University, Nijmegen, The Netherlands; [6]Klinik für Hämatologie und Stammzelltransplantation, University Hospital Essen, Essen, Germany; [7]Department of Hematology, Oncology and Transfusion Medicine Center, Vilnius University Hospital Santaros Klinikos, Vilnius University, Vilnius, Lithuania; [8]Department of Internal Medicine, Hematology, Oncology and Immunology, Philipps University Marburg and University Hospital Gießen and Marburg, Campus Marburg, Marburg, Germany; [9]Department of Hematology, Oncology, Hemostasiology and Stem Cell Transplantation, Medical Clinic IV, University Hospital RWTH Aachen, Aachen, Germany; [10]Department of Haematology, University Hospital Centre Zagreb, Zagreb, Croatia; [11]Dipartimento di Scienze Mediche e Chirurgiche Materno-Infantili e dell'Adulto, University of Modena and Reggio Emilia, Azienda Ospedaliera Universitaria, Modena, Italy; [12]Department of Hematology and Oncology, HELIOS Hospital Berlin-Buch, Kiel, Germany; [13]Department of Hematology, Azienda Ospedaliera Città della Salute e della Scienza di Torino - Ospedale Molinette, Torino, Italy; [14]Clinica Ematologica Azienda Sanitaria Universitaria Integrata di Udine, Udine, Italy;[15]Service d'Hématologie Clinique et de Thérapie cellulaire, Hôpital Saint Antoine, APHP, Paris, France; [16]Ematologia, Dipartimento di Medicina Traslazionale e di Precisione, "Sapienza" Università di Roma, Rome, Italy; [17]Division of Hematology, Department of Translational Medicine, Università del Piemonte Orientale and Azienda Ospedaliero-Universitaria Maggiore della Carità, Novara, Italy; [18]Department of Internal Medicine, Reinier de Graaf Hospital, Delft, The Netherlands;[19]Department of Hematology, Medical Center Leeuwarden, Leeuwarden, The Netherlands; [20]University and CHU of Liège, Liège, Belgium;[21]Department of Hematology, Haga Teaching Hospital, The Hague, The Netherlands; [22]Hematology Department of Biomedicine and Prevention, University of Rome "Tor Vergata", Rome, Italy; [23]University Medical Center Groningen, Groningen, The Netherlands; [24]Department of Hematology, Oncology and Stem Cell Transplantation, Faculty of Medicine, University Medical Center Freiburg, Freiburg, Germany;*

**Background**

The fitness of patients with acute myeloid leukemia (AML) at clinical presentation is critical for successful treatment. However, assessment of fitness and identification of prognostic factors remains challenging. Limited data are available on the role of health-related quality of life (HRQoL).

**Aims**

To investigate the prognostic value of baseline HRQoL, beyond other fitness indicators in older adults with AML. Prognostic factor analyses were performed for overall survival (OS) and complete remission (CR)/CR with incomplete hematological recovery (CRi) probability.

**Copyright Information:** (Online) ISSN: 2572-9241
©2026 The Author(s). HemaSphere is published by John Wiley & Sons Ltd on behalf of European Hematology Association. This is an open access Abstract Book distributed under the Attribution-NonCommercial-NoDerivs (CC BY-NC-ND), which allows third parties to download the articles and share them with others as long as they credit the author and the Abstract Book, but they cannot change the content in any way or use them commercially.
**Abstract Book Citations:** Authors, Title, HemaSphere, 2026;10:(S1):pages. The abstract book DOIs can be found on the "Author Information" page.
**Disclaimer:** Articles published in the journal HemaSphere exclusively reflect the opinions of the authors. The authors are responsible for all content in their abstracts including accuracy of the facts, statements, citing resources, etc.

### Methods

The EORTC AML21 study is a randomized phase 3 trial comparing 10-day decitabine versus intensive chemotherapy in older adults with AML (Lübbert M, et al., Lancet Haematol. 2023,11:e879-e889). At baseline (i.e., pre-treatment), HRQoL was assessed with validated questionnaires (EORTC QLQ-C30, QLQ-ELD14). The following scales were a priori selected: physical functioning (PF), role functioning (RF), global health status/QoL, fatigue (FA), and burden of illness (BI). The prognostic value of baseline indicators was assessed using Cox proportional hazards models. Hazard ratios (HRs) were reported, expressed per 10-point increase on the 0-100 scale. Multivariable analyses were adjusted for established prognostic factors in AML, including age, sex, white blood cell count at diagnosis, and the ELN 2022 risk category. Sensitivity analyses for HRQOL scales were further adjusted for the following frailty indicators: ECOG performance status, short physical performance battery (SPPB) score, and Sorror index. The predictive value of baseline indicators for differential treatment effect was evaluated using Cox models incorporating the treatment arm, the covariate of interest, and their interaction.

### Results

Overall, 606 patients were randomized. Of these, 57.2% were male, and 33.7% were aged ≥70 years. Based on the ELN 2022 risk classification, 19% of patients were classified at favorable, and 63% at adverse risk. None of the baseline characteristics showed a statistically significant association with the effect of the randomized treatment on overall OS (p-values >0.05).
In contrast with BI (p>0.05), FA, PF, RF, and global health status/QoL showed strong associations with OS in univariable analyses (p<0.001). In the multivariable analyses, the adjusted HRs were: 1.053 (95% CI: 1.017–1.091, p=0.0038) for FA, 0.925 (0.885–0.966, p=0.0005) for PF, 0.955 (0.926–0.985, p=0.0033) for RF, and 0.929 (0.891–0.969, p=0.0006) for global health status/QoL. Sensitivity analyses, further adjusting for frailty indicators, revealed that PF, RF, and global health status/QoL remained independently associated with OS, with HRs of 0.949 (95% CI: 0.901-0.998, p=0.0433), 0.962 (0.930-0.996, p=0.0296), and 0.940 (0.897-0.985, p=0.0096), respectively. These latter HRs translate into approximately a 4-6% decrease in the instantaneous rate of death for every 10-point increase (better score) in the PF, RF, and global health status/QoL scales of the EORTC QLQ-C30, respectively. None of the HRQoL scales showed an indication of prognostic importance for CR/CRi probability.

### Summary/Conclusion

Patient-reported HRQoL data provide independent prognostic information for OS beyond well-established laboratory and clinical data in older adults with AML. Our findings support the assessment of HRQoL in the routine diagnostic work-up practice and lay the groundwork for developing a more patient-centric assessment of fitness in AML.

**Copyright Information:** (Online) ISSN: 2572-9241
©2026 The Author(s). HemaSphere is published by John Wiley & Sons Ltd on behalf of European Hematology Association. This is an open access Abstract Book distributed under the Attribution-NonCommercial-NoDerivs (CC BY-NC-ND), which allows third parties to download the articles and share them with others as long as they credit the author and the Abstract Book, but they cannot change the content in any way or use them commercially.
**Abstract Book Citations:** Authors, Title, HemaSphere, 2026;10:(S1):pages. The abstract book DOIs can be found on the “Author Information” page.
**Disclaimer:** Articles published in the journal HemaSphere exclusively reflect the opinions of the authors. The authors are responsible for all content in their abstracts including accuracy of the facts, statements, citing resources, etc.

### (S328) HEALTH-RELATED QUALITY OF LIFE FOR LONG-TERM SURVIVORS OF CLASSICAL HODGKIN LYMPHOMA WITH AND WITHOUT RELAPSE

**Topic:** 36. Quality of life, ethics, supportive and palliative care

Joachim Baech*[1], Sissel Johanne Godtfredsen[1, 2], Paw Jensen[2, 3], Peter Martin Hjørnet Kamper[4], Jacob Haaber Christensen[5], Martin Hutchings[6, 7], Mikkel Porsborg Andersen[8, 9, 10], Christian Torp-Pedersen[8, 11], Kristian Hay Kragholm[1, 2], Tarec El-Galaly[4, 5, 12, 13, 14]

*[1]Aalborg University Hospital, Department of Cardiology, Aalborg, Denmark;[2]Aalborg University, Department of Clinical Medicine, Aalborg, Denmark; [3]Aalborg University Hospital, Department of Hematology, Aalborg, Denmark;[4]Aarhus University Hospital, Department of Hematology, Aarhus, Denmark; [5]Odense University Hospital, Department of Hematology, Odense, Denmark; [6]Copenhagen University Hospital Rigshospitalet, Department of Hematology, Copenhagen, Denmark;[7]University of Copenhagen, Department of Clinical Medicine, Copenhagen, Denmark; [8]Steno, Copenhagen University Hospital, Diabetes Center Copenhagen, Copenhagen, Denmark; [9]The Prehospital Center, Region Zealand, Naestved, Denmark;[10]Aalborg University, Department of Health Science and Technology, Aalborg, Denmark; [11]University of Copenhagen, Department of Public Health, Copenhagen, Denmark; [12]Aarhus University Hospital, Department of Molecular Medicine, Aarhus, Denmark; [13]Aarhus University Hospital, Department of Clinical Epidemiology, Aarhus, Denmark; [14]Karolinska Institutet, Department of Medicine Solna, Clinical Epidemiology Division, Stockholm, Sweden;*

### Background

Most patients with classical Hodgkin lymphoma (cHL) are cured and the 5-year overall survival now exceeds 95% for younger patients even in the advanced disease stage setting, leading to more long-term survivors. However, around 10% of patients with cHL relapse within the first five years. Fit and younger patients with relapse receive intensive salvage chemotherapy with high-dose therapy followed by autologous stem cell transplantation (HDT/ASCT). Following HDT/ASCT, 50% of patients achieve durable remissions. In order to improve survivorship care, there is a need to understand how relapse impacts patient-reported outcomes like health-related quality of life.

### Aims

To assess and compare the long-term symptom burden for cHL survivors with and without relapse in the form of functional and symptom domains from the European Organization for Research and Treatment of Cancer Quality of Life Core-30 (QLQ-C30).

### Methods

In this Danish nationwide cross-sectional survey, all adult patients diagnosed with cHL between 2000–2022 in the Danish Lymphoma Registry were invited to answer the QLQ-C30 questionnaire in the period from March to May 2025. Patients were stratified by prior relapse. The QLQ-C30 is a cancer-specific multidimensional questionnaire with a one-week look-back period, where higher scores for the symptom domains indicate higher symptom burden on a linear scale of 0-100. The scores were described using means with 95% confidence intervals (95% CI) and adjusted mean differences using linear regression models adjusted for sex, age (restricted cubic splines with three degrees of freedom), and time since last disease activity (diagnosis or relapse). Thresholds for minimal clinically important differences were considered small for adjusted mean differences of 5-10 points, moderate for 11-20 points, and large for >20.

**Copyright Information:** (Online) ISSN: 2572-9241
©2026 The Author(s). HemaSphere is published by John Wiley & Sons Ltd on behalf of European Hematology Association. This is an open access Abstract Book distributed under the Attribution-NonCommercial-NoDerivs (CC BY-NC-ND), which allows third parties to download the articles and share them with others as long as they credit the author and the Abstract Book, but they cannot change the content in any way or use them commercially.
**Abstract Book Citations:** Authors, Title, HemaSphere, 2026;10:(S1):pages. The abstract book DOIs can be found on the "Author Information" page.
**Disclaimer:** Articles published in the journal HemaSphere exclusively reflect the opinions of the authors. The authors are responsible for all content in their abstracts including accuracy of the facts, statements, citing resources, etc.

**Results**

A total of 2,312 patients with cHL (2,069 without relapse and 243 with a history of relapse) were invited to participate. Response rates were similar between the groups, resulting in 872 (37.7%) responders (780 without relapse, 92 with relapse). The median time from last disease activity was similar (no relapse: 12.8, relapse: 12.3 years) as was median age (55 versus 56.5). Among patients with relapse, 44 (47.8%) were treated with HDT/ASCT.
Overall, patients with a history of relapse had a lower (i.e., worse) adjusted mean score for global health (-3.33 [95% CI: -7.75–1.08]), physical functioning (-2.16 [95% CI: -5.58–1.55]), and cognitive functioning (-1.91 [95% CI: -6.80–2.98]), although differences were not statistically significant or clinically important. In addition, patients with a history of relapse reported a higher symptom burden across all symptom domains (Figure 1). The largest adjusted mean differences (relapse versus no relapse) were observed for fatigue (8.40 [95% CI: 3.06–13.73]) and diarrhea (5.88 [95% CI: 1.58–10.18]), corresponding to small but statistically significant differences, and dyspnea (5.06 [95% CI: -0.03–10.15]), which was borderline significant.

**Summary/Conclusion**

Long-term global health, physical functioning, and cognitive functioning were similar in cHL survivors with relapse versus those without relapse. However, relapse survivors reported a consistently higher symptom burden, most notably small differences for fatigue and diarrhea, highlighting a need for symptom-focused survivorship care after relapse therapy even a decade after diagnosis or last relapse.

**Copyright Information:** (Online) ISSN: 2572-9241
©2026 The Author(s). HemaSphere is published by John Wiley & Sons Ltd on behalf of European Hematology Association. This is an open access Abstract Book distributed under the Attribution-NonCommercial-NoDerivs (CC BY-NC-ND), which allows third parties to download the articles and share them with others as long as they credit the author and the Abstract Book, but they cannot change the content in any way or use them commercially.
**Abstract Book Citations:** Authors, Title, HemaSphere, 2026;10:(S1):pages. The abstract book DOIs can be found on the "Author Information" page.
**Disclaimer:** Articles published in the journal HemaSphere exclusively reflect the opinions of the authors. The authors are responsible for all content in their abstracts including accuracy of the facts, statements, citing resources, etc.

| | Relapse mean score (95% CI) | No relapse mean score (95% CI) | Adj. mean diff. (95% CI) |
|---|---|---|---|
| **Fatigue** | | | |
| Relapse vs. no relapse | 34.18 (28.54-39.82) | 26.20 (24.49-27.91) | 8.40 (3.06; 13.73) |
| **Nausea and vomiting** | | | |
| Relapse vs. no relapse | 4.35 (2.37-6.32) | 3.67 (2.90-4.44) | 0.93 (-1.47; 3.34) |
| **Pain** | | | |
| Relapse vs. no relapse | 19.38 (14.26-24.51) | 15.26 (13.69-16.82) | 4.18 (-0.65; 9.02) |
| **Dyspnea** | | | |
| Relapse vs. no relapse | 21.77 (16.00-27.53) | 14.62 (12.97-16.26) | 5.06 (-0.03; 10.15) |
| **Insomina** | | | |
| Relapse vs. no relapse | 25 (19.07-30.93) | 24.66 (22.63-26.68) | 1.54 (-4.66; 7.74) |
| **Appetite loss** | | | |
| Relapse vs. no relapse | 9.42 (4.58-14.26) | 6.97 (5.63-8.3) | 2.26 (-1.96; 6.48) |
| **Constipation** | | | |
| Relapse vs. no relapse | 11.96 (7.55-16.37) | 8.90 (7.49-10.30) | 3.32 (-1.06; 7.71) |
| **Diarrhea** | | | |
| Relapse vs. no relapse | 13.27 (8.30-18.23) | 7.99 (6.65-9.33) | 5.88 (1.58; 10.18) |
| **Financial difficulties** | | | |
| Relapse vs. no relapse | 9.86 (6.00-13.73) | 8.20 (6.78-9.62) | 2.96 (-1.49; 7.41) |

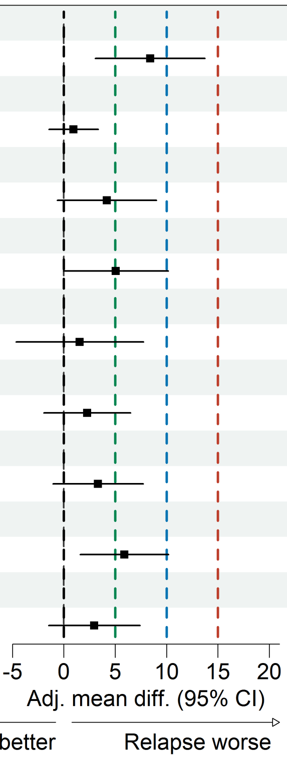

Figure 1. QLQ-C30 symptom subscale score for 872 patients with Hodgkin lymphoma with (92) or without relapse (780).

**Copyright Information:** (Online) ISSN: 2572-9241
©2026 The Author(s). HemaSphere is published by John Wiley & Sons Ltd on behalf of European Hematology Association. This is an open access Abstract Book distributed under the Attribution-NonCommercial-NoDerivs (CC BY-NC-ND), which allows third parties to download the articles and share them with others as long as they credit the author and the Abstract Book, but they cannot change the content in any way or use them commercially.
**Abstract Book Citations:** Authors, Title, HemaSphere, 2026;10:(S1):pages. The abstract book DOIs can be found on the "Author Information" page.
**Disclaimer:** Articles published in the journal HemaSphere exclusively reflect the opinions of the authors. The authors are responsible for all content in their abstracts including accuracy of the facts, statements, citing resources, etc.

### (S329) COST-EFFECTIVENESS AND BUDGET IMPACT OF GENE THERAPY FOR ELIGIBLE PATIENTS WITH SICKLE CELL DISEASE IN THE NETHERLANDS

**Topic:** 37. Health economics

Melanie Bruinooge*[1, 2], Anita Rijneveld[3], Charlotte van Tuijn[4], Erfan Nur[4], Eduard J. van Beers[5], Alexander Mohseny[6], Marjon Cnossen[1], Carin Uyl-de Groot[2]

*[1]Erasmus MC Sophia Children's Hospital, University Medical Centre Rotterdam, Department of Pediatric Hematology and Oncology, Rotterdam, The Netherlands; [2]Erasmus School of Health Policy and Management, Erasmus University Rotterdam, Rotterdam, The Netherlands; [3]Erasmus MC, University Medical Centre Rotterdam, Department of Hematology, Rotterdam, The Netherlands; [4]Amsterdam University Medical Center, Department of Hematology, Amsterdam, The Netherlands;[5]Center for Benign Hematology, Thrombosis and Hemostasis, Van Creveldkliniek University Medical Center Utrecht, Utrecht, The Netherlands; [6]Leiden University Medical Center, Department of Pediatric Hematology and Stem Cell Transplantation, Leiden, The Netherlands;*

### Background

Gene therapy offers promising benefits for patients with sickle cell disease (SCD) but also raises questions regarding affordability.

### Aims

This study aimed to evaluate the cost-effectiveness and budget impact of gene therapy compared to comprehensive care in patients with severe SCD genotypes (HbSS or HbS$\beta^0$/$\beta^+$) aged ≥12 years with severe disease-related symptoms.

### Methods

We conducted a cost-effectiveness analysis (CEA) using a Markov state-transition model from a Dutch societal perspective over a lifetime horizon. Efficacy and safety data for gene therapy were sourced from the HGB-206 (NCT02140554) and HGB-210 (NCT03499818) clinical trials. Additional data on clinical outcomes, quality of life (QoL), and costs were obtained from the HGB-206 and HGB-210 trial, and scientific literature. Budget impact analysis (BIA) was performed from a healthcare perspective, covering a five-year time horizon. Uncertainty in the CEA and BIA were assessed through probabilistic sensitivity and scenario analysis.

### Results

After applying discounting and half-cycle correction, gene therapy yielded 11.70 additional life years, and 13.29 additional quality-adjusted life years (QALYs) compared to comprehensive care. The deterministic incremental cost-effectiveness ratio (ICER) was €123,384 per QALY gained; the probabilistic ICER was €124,972 per QALY gained. Results were sensitive to drug acquisition costs, survival rates, and utility of patients with no or mild vaso-occlusive events in the gene therapy group, and the method used to calculate productivity losses (human capital versus friction period). The budget impact of treating eligible patients with gene therapy over 5 years was projected to be approximately €71 million.

**Copyright Information:** (Online) ISSN: 2572-9241
©2026 The Author(s). HemaSphere is published by John Wiley & Sons Ltd on behalf of European Hematology Association. This is an open access Abstract Book distributed under the Attribution-NonCommercial-NoDerivs (CC BY-NC-ND), which allows third parties to download the articles and share them with others as long as they credit the author and the Abstract Book, but they cannot change the content in any way or use them commercially.
**Abstract Book Citations:** Authors, Title, HemaSphere, 2026;10:(S1):pages. The abstract book DOIs can be found on the "Author Information" page.
**Disclaimer:** Articles published in the journal HemaSphere exclusively reflect the opinions of the authors. The authors are responsible for all content in their abstracts including accuracy of the facts, statements, citing resources, etc.

**Summary/Conclusion**

Gene therapy substantially improves survival and QoL for patients with severe SCD compared to comprehensive care but at a high cost. At the current cost-effectiveness threshold of €80,000 per QALY in the Netherlands, it is not considered cost-effective. Reducing the market price of gene therapy is essential to make it more economically viable and accessible.

**Copyright Information:** (Online) ISSN: 2572-9241
©2026 The Author(s). HemaSphere is published by John Wiley & Sons Ltd on behalf of European Hematology Association. This is an open access Abstract Book distributed under the Attribution-NonCommercial-NoDerivs (CC BY-NC-ND), which allows third parties to download the articles and share them with others as long as they credit the author and the Abstract Book, but they cannot change the content in any way or use them commercially.
**Abstract Book Citations:** Authors, Title, HemaSphere, 2026;10:(S1):pages. The abstract book DOIs can be found on the "Author Information" page.
**Disclaimer:** Articles published in the journal HemaSphere exclusively reflect the opinions of the authors. The authors are responsible for all content in their abstracts including accuracy of the facts, statements, citing resources, etc.

### (S330) FISRT-IN-HUMAN OF ALPACA-DERIVED NANOBODY-BASED BISPECIFIC EPITOPE CD5 CAR-T CELLS FOR RELAPSED OR REFRACTORY T-CELL ACUTE LYMPHOBLASTIC LEUKEMIA

**Topic:** 01. Acute lymphoblastic leukemia - Biology & translational research

Jing Pan[1, 2], Yuxuan Song*[1, 3], Ang Zhang[4], Su Li[5], Xiaoxia Hu[6]

*[1]State Key Laboratory of Experimental Hematology, Department of Hemato-Oncology and Immunotherapy, Beijing Gobroad Hospital, Beijing, China; [2]State Key Laboratory of Experimental Hematology, National Clinical Research Center for Blood Diseases, Haihe Laboratory of Cell Ecosystem, Institute of Hematology & Blood Diseases Hospital, Chinese Academy of Medical Sciences & Peking Union Medical College, Tianjin, China; [3]School of Basic Medicine and Clinical Pharmacy, China Pharmaceutical University, Nanjing, China; [4]Department of Comprehensive Basic Experiment, The Ninth Medical Center of Chinese PLA General Hospital, Beijing, China; [5]Department of Hematology, Shanghai Liquan Hospital, Shanghai, China;[6]State Key Laboratory of Medical Genomics, Shanghai Institute of Hematology, National Research Center for Translational Medicine, Shanghai Ruijin Hospital, Shanghai Jiao Tong University School of Medicine, Shanghai, China;*

### Background

CD5 is expressed on the tumor cells of several hematological malignancies. We have designed humanized CD5 CAR-T (hCD5 CAR-T) cell for patients with relapsed or refractory T-cell acute lymphoblastic leukemia (r/r T-ALL), showing high remission efficacy (*Pan et al. Nat Med 2025*). However, its delayed peak expansion relative to other CAR-T therapies contributed to infections and tumor progression. Therefore, we developed a novel alpaca-derived nanobody-based bispecific epitope CD5 CAR (NbCD5 CAR) vector to accelerat expansion and improve safety and efficacy. Here we present its safety and efficacy of preclinical trial and a phase 1/2 trial of NbCD5 CAR-T in r/r T-ALL.

### Aims

We aim to accelerate CAR-T cells expansion, improving efficacy and safety in r/r T-ALL by screening novel NbCD5 CAR vector.

### Methods

We constructed an alpaca-derived nanobody library and screened it for CD5-specific nanobodies to engineer NbCD5 CAR-T cells. In contrast to scFv-based hCD5 CAR-T(*Pan et al. Nat Med 2025*), NbCD5 CAR-T uses a 15 kDa alpaca VHH domai for simpler structure and superior penetration. We assessed antigen-specific activation and in vitro cytotoxicity of CAR-T cells. In vivo efficacy was evaluated in Jurkat-engrafted NSG mice by flow cytometry and bioluminescent imaging. Ultimately, the efficacy and safety of NbCD5 CAR-T were evaluated in a multicenter phase 1/2 trial of r/r T-ALL (NCT07070323). The trial used BOIN1/2 design with initial dose $1\times10^6$ (±20%) CAR-T cells/kg and low-dose $5\times10^5$ (±20%) cells/kg. The primary endpoints was safety and efficacy secondary.

**Copyright Information:** (Online) ISSN: 2572-9241
©2026 The Author(s). HemaSphere is published by John Wiley & Sons Ltd on behalf of European Hematology Association. This is an open access Abstract Book distributed under the Attribution-NonCommercial-NoDerivs (CC BY-NC-ND), which allows third parties to download the articles and share them with others as long as they credit the author and the Abstract Book, but they cannot change the content in any way or use them commercially.
**Abstract Book Citations:** Authors, Title, HemaSphere, 2026;10:(S1):pages. The abstract book DOIs can be found on the "Author Information" page.
**Disclaimer:** Articles published in the journal HemaSphere exclusively reflect the opinions of the authors. The authors are responsible for all content in their abstracts including accuracy of the facts, statements, citing resources, etc.

**Results**

Flow cytometric CD107a degranulation assay confirmed that all CD5-targeting CAR-T cells (excluding controls) were specifically activated by $CD5^+$ target cells, with NbCD5 CAR-T demonstrating superior antigen-dependent activation compared to hCD5 CAR-T.

In vitro cytotoxicity assays, NbCD5 CAR-T cells showed potent, dose-dependent cytotoxicity specifically against $CD5^+$ target cells, significantly outperforming controls; minimal killing activity against $CD5^-$ K562 cells confirmed their antigen specificity.

In Jurkat-engrafted NSG mice, NbCD5 CAR-T on day 7 eradicated tumors and ensured long-term survival, outperforming Mock-T and hCD5 CAR-T.

We then evaluated NbCD5 CAR-T in a clinical trial for r/r T-ALL. The trial enrolled 6 patients (4 were refractory to prior CD7 CAR-T therapy). Three patients who were relapsed post-transplant received CAR-T cells derived from preivous transplant donors (Cohort B) and 3 patients were relapsed after chemotherpies and received CAR-T cells derived from new HLA-matched donors (Cohort C). All patients received $1.0\times10^6$/kg NbCD5 CAR-T cells. Adverse events within 30 days included: Grade 1 CRS (6/6), Grade 2 ICANS (1/6), and Grade 1~2 GVHD (2/6). All patients achieved complete remission by day 30. In a follow-up time of 90 days, 5 patients bridged to allo SCT, 4 patients remained $MRD^-$; 2 patients relapsed: 1 patient CD5dim relapsed at day 88, 1 patient $CD5^-$ relapsed at day 45 and died 6 days later. The peak expansion of CAR-T cells at day 10 (range: 7~21).

**Summary/Conclusion**

The nanobody-based CD5 CAR we modified demonstrated excellent antigen specificity, cytotoxicity and efficacy in preliminaryin vitro and in vivo studies. Furthermore, we got the safety and efficacy profiles of NbCD5 CAR-T in r/r T-ALL. Our trial is still ongoing, it requires further investigation of long-term efficacy, safety and immune reconstitution.

**Copyright Information:** (Online) ISSN: 2572-9241
©2026 The Author(s). HemaSphere is published by John Wiley & Sons Ltd on behalf of European Hematology Association. This is an open access Abstract Book distributed under the Attribution-NonCommercial-NoDerivs (CC BY-NC-ND), which allows third parties to download the articles and share them with others as long as they credit the author and the Abstract Book, but they cannot change the content in any way or use them commercially.
**Abstract Book Citations:** Authors, Title, HemaSphere, 2026;10:(S1):pages. The abstract book DOIs can be found on the "Author Information" page.
**Disclaimer:** Articles published in the journal HemaSphere exclusively reflect the opinions of the authors. The authors are responsible for all content in their abstracts including accuracy of the facts, statements, citing resources, etc.

### (S331) DECIPHERING THE EPIGENETIC LANDSCAPE OF MATURE T-CELL LYMPHOMAS AND LEUKEMIAS: INTEGRATING ATAC-SEQ AND MACHINE LEARNING FOR PRECISION DIAGNOSTICS

**Topic:** 38. Novel technologies, techniques and digital analytical tools in hematology

Edith Julia*[1], Amel Chebel[2], Sarah Sebai[2], David Armisen[3], Sylvain Mareschal[4], Jonathan Lefebvre[3], Camille Golfier[5], Camille Lours[6], Marie Donzel[7], Fanny Drieux[8], Aurelie Verney[3], Edoardo Missiaglia[9], Vimel Rattina[9], Sylvain Carras[10], Ambroise Marçais[11], Morgane Cheminant[11], Laurent Martin[12], Luc Xerri[13], Pierre Sujobert[14], Marco Herling[15], Claire Bardel[4], Gilles Salles[16], Laurent Camille[17], Catherine Chassagne-Clement[18], Lucile Baseggio[14], Alexandra Traverse-Glehen[19], Philippe Gaulard[20], Laurence de Leval[21], Laurent Genestier[22], Emmanuel Bachy[1]

*[1]Department of Hematology, Hospices Civils de Lyon and Université Claude Bernard Lyon 1, Lyon, France;[2]Université Claude Bernard Lyon 1, Lyon, France; [3]CIRI, Inserm U1111, CNRS UMR5308, ENS de Lyon, Université Lyon 1, Lyon, France;[4]Hospices Civils de Lyon, Plateforme ngs-hcl, cellule bioinformatique, Lyon, France; [5]Hospices Civils de Lyon, Hematology, Lyon, France; [6]Hospices Civils de Lyon, Service d'hématologie biologique, Centre de biolologie et pathologie est, Bron, France;[7]Hospices Civils de Lyon, Service d'anatomopathologie, Lyon, France; [8]Centre Henri Becquerel, LYON, France; [9]Institute of Pathology, Lausanne University Hospital, Lausanne, Switzerland; [10]Clinique d'Hématologie, C.H.U. Grenoble, La Tronche, France; [11]Hématologie adulte, Hôpital Necker, Paris, France; [12]CHU de Dijon, Pathology, Dijon, France; [13]Institut Paoli Calmette, Marseille, France; [14]Hospices Civils de Lyon, Laboratoire d'hématologie, Hôpital Lyon Sud, Lyon, France; [15]Department of Hematology, Cell Therapy and Hematology, University Hospital Leipzig, Leipzig, Germany; [16]Lymphoma Service, Memorial Sloan Kettering Cancer Center, New York, United States of America; [17]IUCT Oncopole, Pathology, Toulouse, France; [18]Anatomopathologie - Centre Léon Bérard, Lyon, France; [19]Hospices Civils de Lyon, Service de Pathologie, Centre Hospitalier Lyon-Sud, Pierre-Bénite, France; [20]Hôpital Henri Mondor, Département de Pathologie, Créteil, France;[21]Pathologie CHUV, Institut universitaire de pathologie, Lausanne, France; [22]Equipe LIB, CIRI, Inserm U1111, CNRS UMR5308, Lyon, France;*

### Background

Mature T-cell lymphomas and leukemias (MTCL) are a heterogeneous group of aggressive malignancies with poor prognosis. Accurate diagnosis requires specialized pathology expertise, with up to 50% of cases being reclassified in some entities following expert review (Laurent, JCO 2017). Assay for Transposase-Accessible Chromatin using sequencing (ATAC-seq) is a powerful method for profiling open chromatin regions, proven to be highly discriminative for cell-of-origin identification and cancer clustering (Corces, Science 2018).

### Aims

This study aims to characterize the epigenetic landscape of MTCL using ATAC-seq, compare these profiles to normal T-cell subtypes, and develop a predictive model to assist in the differential diagnosis of these complex entities.

### Methods

A training cohort (n = 190 samples, encompassing various MTCL subtypes) underwent ATAC-seq, followed by epigenetic clustering using iterative non-negative matrix factorization (NMF), UMAP, and HDBSCAN, enabling identification of MTCL-specific epigenetic signatures. RNA-seq, WES and CNV profiling from ATAC-seq reads was used to further characterize a new epigenetic cluster of PTCL-NOS. MTCL signatures were compared to those of normal T and NK cells sorted from blood and lymph nodes of healthy donors. Single-cell ATAC-seq data from follicular helper T cells (TFH) were used for deconvolution of TFHL lymphomas. An iterative NMF coupled with LASSO regression was developed to predict MTCL subtypes. Model performances were validated on a multicenter cohort (n = 186 samples).

**Copyright Information:** (Online) ISSN: 2572-9241
©2026 The Author(s). HemaSphere is published by John Wiley & Sons Ltd on behalf of European Hematology Association. This is an open access Abstract Book distributed under the Attribution-NonCommercial-NoDerivs (CC BY-NC-ND), which allows third parties to download the articles and share them with others as long as they credit the author and the Abstract Book, but they cannot change the content in any way or use them commercially.
**Abstract Book Citations:** Authors, Title, HemaSphere, 2026;10:(S1):pages. The abstract book DOIs can be found on the "Author Information" page.
**Disclaimer:** Articles published in the journal HemaSphere exclusively reflect the opinions of the authors. The authors are responsible for all content in their abstracts including accuracy of the facts, statements, citing resources, etc.

**Results**

UMAP projection and Shared Nearest Neighbors (SNN) clustering of ATAC-seq data revealed epigenetic groups highly concordant with initial pathological diagnoses (Figure A). Anaplastic large cell lymphoma (ALCL) subtypes aligned with recently described type 1 and type 2 ALCL (Feldman, Blood 2025). Peripheral T-cell lymphoma, not otherwise specified (PTCL-NOS), were largely reclassified into specific entities, except for an isolated cluster of samples (cluster 5, see Figure A) characterized by a strong GATA3 signature. This subgroup exhibited a high rate of *TP53* mutations, *NCOR1* mutations (7/9), and frequent del17p, affecting both *TP53* and *NCOR1*. Cell-of-origin analysis showed proximity between T-prolymphocytic leukemia (T-PLL) and naïve T cells, cutaneous T-cell lymphoma (CTCL) and central memory T cells, and TFH lymphoma with normal TFH cells. Deconvolution of the TFH lymphoma microenvironment confirmed the favorable prognostic impact of B-cell-rich infiltrates (Figure B and C). Finally, an ATAC-seq method for FFPE samples was developed to expand the routine applicability of this technique. An epigenetic classifier was built, achieving a diagnostic accuracy of 96.8% in predicting MTCL entities after expert review (Figure D). Notably, ATAC-seq enabled a refined diagnosis of PTCL-NOS, with 22 of 35 epigenetic reclassifications judged compatible by pathology review (Figure E).

**Summary/Conclusion**

This study demonstrates the utility of ATAC-seq in deciphering the epigenetic landscape of MTCL and presents a novel diagnostic tool with robust performance and potential for clinical integration.

**Copyright Information:** (Online) ISSN: 2572-9241
©2026 The Author(s). HemaSphere is published by John Wiley & Sons Ltd on behalf of European Hematology Association. This is an open access Abstract Book distributed under the Attribution-NonCommercial-NoDerivs (CC BY-NC-ND), which allows third parties to download the articles and share them with others as long as they credit the author and the Abstract Book, but they cannot change the content in any way or use them commercially.
**Abstract Book Citations:** Authors, Title, HemaSphere, 2026;10:(S1):pages. The abstract book DOIs can be found on the "Author Information" page.
**Disclaimer:** Articles published in the journal HemaSphere exclusively reflect the opinions of the authors. The authors are responsible for all content in their abstracts including accuracy of the facts, statements, citing resources, etc.

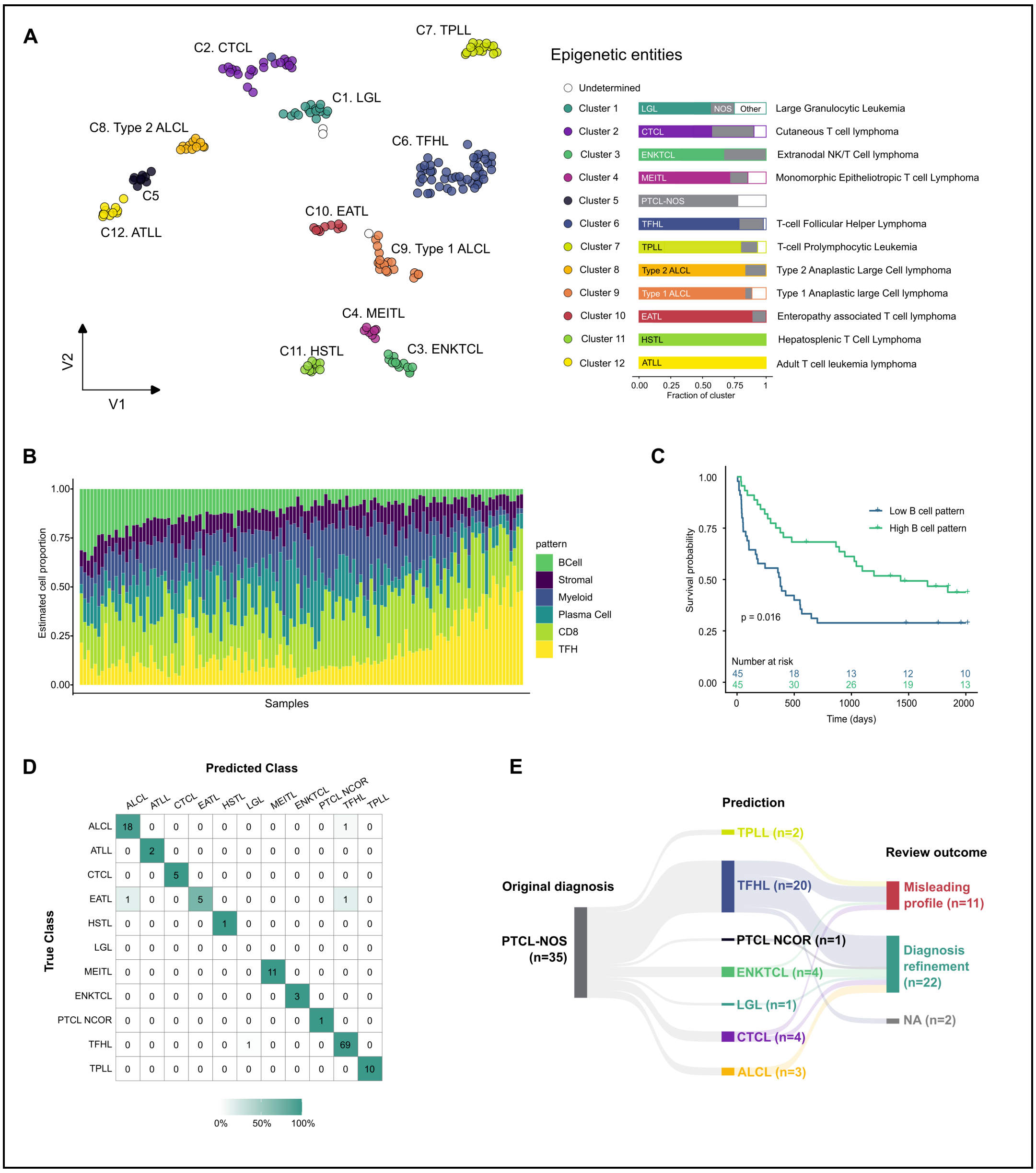

**Copyright Information:** (Online) ISSN: 2572-9241
©2026 The Author(s). HemaSphere is published by John Wiley & Sons Ltd on behalf of European Hematology Association. This is an open access Abstract Book distributed under the Attribution-NonCommercial-NoDerivs (CC BY-NC-ND), which allows third parties to download the articles and share them with others as long as they credit the author and the Abstract Book, but they cannot change the content in any way or use them commercially.
**Abstract Book Citations:** Authors, Title, HemaSphere, 2026;10:(S1):pages. The abstract book DOIs can be found on the "Author Information" page.
**Disclaimer:** Articles published in the journal HemaSphere exclusively reflect the opinions of the authors. The authors are responsible for all content in their abstracts including accuracy of the facts, statements, citing resources, etc.

### (S332) NANOPORE-BASED GENOMIC AND EPIGENOMIC PROFILING FOR PEDIATRIC HEMATOLOGIC MALIGNANCIES USING TARGETED ADAPTIVE SAMPLING LONG-READ SEQUENCING: INTERIM REPORT OF THE JCCG-WGS STUDY

**Topic:** 38. Novel technologies, techniques and digital analytical tools in hematology

Shota Kato*[1], Aiko Sato-Otsubo[1], Masahiro Sekiguchi[2], Miho Kato[3], Takao Deguchi[4], Daisuke Tomizawa[5], Shuhei Karakawa[6], Yuki Arakawa[7], Yuki Yuza[8], Toshihiro Fujiki[9], Kenichiro Watanabe[10], Hiroyuki Fujisaki[11], Masaharu Akiyama[12], Nao Yoshida[13], Reiji Fukano[14], Yuichi Shiraishi[15], Motohiro Kato[1]

*[1]The University of Tokyo, Department of Pediatrics, Graduate School of Medicine, Tokyo, Japan;[2]National Center for Child Health and Development, Division of Cancer Genomics, Children's Cancer Center, Tokyo, Japan; [3]National Center for Child Health and Development, Department of Childhood Cancer Data Management, Tokyo, Japan; [4]National Center for Child Health and Development, Division of Cancer Immunodiagnostics, Children's Cancer Center, Tokyo, Japan; [5]National Center for Child Health and Development, Division of Leukemia and Lymphoma, Children's Cancer Center, Tokyo, Japan; [6]Hiroshima University Hospital, Department of Pediatrics, Hiroshima, Japan; [7]Saitama Children's Medical Center, Department of Hematology/Oncology, Saitama, Japan; [8]Tokyo Metropolitan Children's Medical Center, Department of Hematology-Oncology, Fuchu, Japan; [9]Kanazawa University, Department of Pediatrics, Kanazawa, Japan; [10]Shizuoka Children's Hospital, Department of Hematology and Oncology, Shizuoka, Japan; [11]Osaka City General Hospital, Department of Pediatric Hematology and Oncology, Osaka, Japan; [12]The Jikei University School of Medicine, Department of Pediatrics, Tokyo, Japan; [13]Japanese Red Cross Aichi Medical Center Nagoya First Hospital, Department of Hematology and Oncology, Children's Medical Center, Nagoya, Japan; [14]School of Medicine, University of Occupational and Environmental Health, Japan, Department of Pediatrics, Kitakyushu, Japan; [15]National Cancer Center Research Institute, Division of Genome Analysis Platform Development, Tokyo, Japan;*

### Background

Short-read-based targeted capture sequencing has several limitations as a clinical sequencing approach, including prolonged turnaround time (TAT) and the potential to miss structural and copy number variations (SVs and CNVs). To overcome these challenges, we previously developed a genome profiling pipeline for pediatric leukemia using targeted adaptive sampling long-read sequencing (TAS-LRS), a nanopore-based targeted sequencing method. However, analyses have been limited to a small number of cases, and the capability to comprehensively detect the diverse genomic alterations observed in pediatric hematologic malignancies has not been sufficiently validated.

### Aims

We prospectively investigated whether our analytical platform retains feasibility (analysis success rate and TAT) and clinical utility (probability of potentially actionable findings [PAFs]) when applied to large numbers of children with hematologic malignancies in the JCCG-WGS study, a nationwide prospective observational study employing TAS-LRS-based genome profiling.

### Methods

Patients aged ≤40 who were diagnosed with hematologic malignancies were eligible. Tumor samples obtained at diagnosis or relapse were submitted and DNA-based libraries were prepared. TAS-LRS was performed using GridION sequencers with R10 flow cells. Target regions generally encompassed 592 genes associated with hematologic malignancies along with their flanking regions, with adjustments made as appropriate for individual cases. Data analysis was conducted in parallel with 60–72 hours of sequencing, and results were compiled into reports including small variants, SVs, CNVs, and MARLIN-based methylation classifications.

**Copyright Information:** (Online) ISSN: 2572-9241
©2026 The Author(s). HemaSphere is published by John Wiley & Sons Ltd on behalf of European Hematology Association. This is an open access Abstract Book distributed under the Attribution-NonCommercial-NoDerivs (CC BY-NC-ND), which allows third parties to download the articles and share them with others as long as they credit the author and the Abstract Book, but they cannot change the content in any way or use them commercially.
**Abstract Book Citations:** Authors, Title, HemaSphere, 2026;10:(S1):pages. The abstract book DOIs can be found on the "Author Information" page.
**Disclaimer:** Articles published in the journal HemaSphere exclusively reflect the opinions of the authors. The authors are responsible for all content in their abstracts including accuracy of the facts, statements, citing resources, etc.

### Results

Between August 2025 and January 2026, 47 patients were enrolled (2.0 registrations/week). Institutional diagnoses comprised 13 cases of acute myeloid leukemia (AML), 13 of B-cell acute lymphoblastic leukemia (B-ALL), 6 of T-ALL, 5 of non-Hodgkin lymphoma, 4 of myelodysplastic neoplasm, 2 of chronic myeloid leukemia, 3 of other leukemias, and 1 of histiocytosis. TAS-LRS and analyses were successfully completed in all cases. The median TAT was 6.0 days (4.0–10.0). The mean depth in target regions was 47.9× in median (15.8–74.0). PAFs were identified in 37 cases (79%) for diagnosis, 22 (47%) for prognosis, and 26 (55%) for therapeutic targets (Figure, left). In one case initially diagnosed as myeloid/lymphoid neoplasm with eosinophilia, detection of *CBFA2T3*::*GLIS2* led to a revised diagnosis of AML. In addition to canonical fusion genes, we identified SVs with breakpoints in noncoding regions that were presumed to cause enhancer hijacking, including *IGH*::*CEBPA* and *TLX3*::*BCL11B*. Using an additional in-house pipeline, *IGH*::*DUX4* was identified in a case with B-ALL. Subtype-defining CNVs, including iAMP21 and near-haploid karyotype, were successfully detected. Methylation-based classification of acute leukemia was concordant with genomic subtypes in 21 cases (66%), excluding 8 cases lacking corresponding categories in MARLIN, 3 cases with no detectable genomic subtypes, and 3 discordant cases with low blast percentage (Figure, right).

### Summary/Conclusion

Genome profiling using TAS-LRS proved feasible and clinically informative in a prospective multicenter study setting. The rapid detection of diagnostically significant genomic alterations, including SVs and CNVs, was valuable in guiding therapeutic decision-making by physicians. Future efforts will involve integration with clinical trials and expansion to larger-scale analyses.

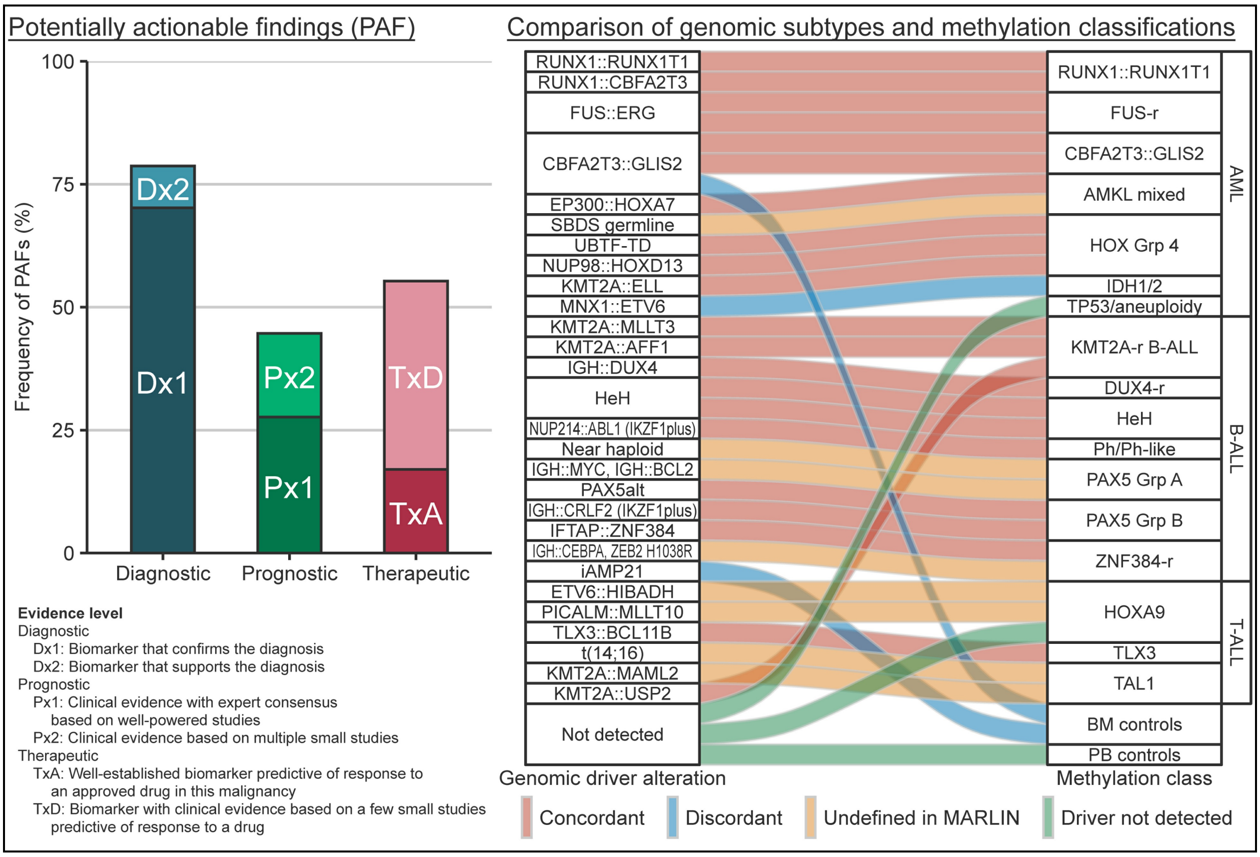

**Copyright Information:** (Online) ISSN: 2572-9241
©2026 The Author(s). HemaSphere is published by John Wiley & Sons Ltd on behalf of European Hematology Association. This is an open access Abstract Book distributed under the Attribution-NonCommercial-NoDerivs (CC BY-NC-ND), which allows third parties to download the articles and share them with others as long as they credit the author and the Abstract Book, but they cannot change the content in any way or use them commercially.
**Abstract Book Citations:** Authors, Title, HemaSphere, 2026;10:(S1):pages. The abstract book DOIs can be found on the “Author Information” page.
**Disclaimer:** Articles published in the journal HemaSphere exclusively reflect the opinions of the authors. The authors are responsible for all content in their abstracts including accuracy of the facts, statements, citing resources, etc.

### (S333) NANOLEUKEMIA - A HIERARCHICAL CLASSIFICATION FRAMEWORK FOR HEMATOPOIETIC NEOPLASMS INTEGRATING EPIGENETIC AND GENETIC INFORMATION FROM WHOLE GENOME NANOPORE SEQUENCING

**Topic:** 38. Novel technologies, techniques and digital analytical tools in hematology

Maximilian Schönung*[1, 2], Melissa Türe[1, 2, 3], Panna Lajer[1, 2, 3], Simon Renders[4, 5, 6, 7], Tobias Rausch[8, 9], Til Steinicke[10, 11, 12], Jessica Heilmann[13], Lena Katzenstein[1, 2], Etienne Sollier[13], Peter Horak[2, 6, 14], Tim Sauer[4], Jonathan Strefford[15], Martí Duran-Ferrer[16, 17], Christopher Oakes[18, 19, 20], José Ignacio Martín-Subero[16, 17, 21, 22], Christian Flotho[23, 24], Charlotte Niemeyer[23, 24], Hartmut Döhner[25], Volker Hovestadt[10, 11, 26], Stefan Fröhling[2, 6, 14, 27], Richard Schlenk[4, 28, 29], Carsten Müller-Tidow[4], Florian Heidel[30], Jan Korbel[8, 31, 32, 33], Clarissa Gerhäuser[13], Mark Hartmann[1, 2], Pavlo Lutsik[34], Michael Hundemer[4, 35], Miriam Erlacher[36], Lars Bullinger[37, 38], Christoph Plass[13], Daniel Lipka[1, 2, 6, 39]

*[1]German Cancer Research Center (DKFZ), Section of Translational Cancer Epigenomics, Division of Translational Medical Oncology, Heidelberg, Germany; [2]National Center for Tumor Diseases (NCT), Heidelberg, Germany; [3]Heidelberg University, Faculty of Biosciences, Heidelberg, Germany; [4]Heidelberg University Hospital, Department of Internal Medicine V, Hematology, Oncology and Rheumatology, Heidelberg, Germany; [5]Heidelberg Institute for Stem Cell Technology and Experimental Medicine (HI-STEM gGmbH), Heidelberg, Germany; [6]German Cancer Consortium (DKTK), DKFZ, Core Center Heidelberg, Heidelberg, Germany; [7]German Cancer Research Center (DKFZ), Division of Stem Cells and Cancer, Heidelberg, Germany; [8]European Molecular Biology Laboratory (EMBL), Genome Biology Unit, Heidelberg, Germany; [9]European Molecular Biology Laboratory (EMBL), Genomics Core Facility, Heidelberg, Germany; [10]Dana-Farber Cancer Institute, Department of Pediatric Oncology, Boston, United States of America; [11]Broad Institute of MIT and Harvard, Boston, United States of America; [12]University Hospital Münster, Department of Medicine A, Münster, Germany; [13]German Cancer Research Center (DKFZ), Division of Cancer Epigenomics, Heidelberg, Germany; [14]German Cancer Research Center (DKFZ), Division of Translational Medical Oncology, Heidelberg, Germany; [15]University of Southampton, Cancer Sciences, Faculty of Medicine, Southampton, United Kingdom; [16]Institut d'Investigacions Biomèdiques August Pi i Sunyer (IDIBAPS), Barcelona, Spain; [17]Centro de Investigación Biomédica en Red de Cáncer (CIBERONC), Madrid, Spain; [18]The Ohio State University, The Comprehensive Cancer Center, Columbus, United States of America; [19]The Ohio State University, Department of Biomedical Informatics, Columbus, United States of America; [20]The Ohio State University, Division of Hematology, Department of Internal Medicine, Columbus, United States of America; [21]Universitat de Barcelona, Departament de Fonaments Clinics, Facultat de Medicina, Barcelona, Spain; [22]Institució Catalana de Recerca i Estudis Avançats (ICREA), Barcelona, Spain; [23]University of Freiburg, Division of Pediatric Hematology and Oncology, Department of Pediatrics and Adolescent Medicine, Medical Center, Faculty of Medicine, Freiburg, Germany; [24]German Cancer Consortium (DKTK), Partner site Freiburg, a partnership between DKFZ and University Medical Center Freiburg, Heidelberg, Germany; [25]University Hospital of Ulm, Department of Internal Medicine III, Ulm, Germany; [26]Boston Children's Hospital, Division of Hematology/Oncology, Boston, United States of America; [27]Heidelberg University, Institute of Human Genetics, Heidelberg, Germany; [28]National Center for Tumor Diseases (NCT) & German Cancer Research Center (DKFZ), NCT Clinical Trial Center, Heidelberg, Germany; [29]National Center for Tumor Diseases (NCT), Department of Medical Oncology, Heidelberg, Germany; [30]Hannover Medical School, Department of Hematology, Hemostasis, Oncology and Stem Cell Transplantation, Hannover, Germany; [31]European Molecular Biology Laboratory (EMBL), Molecular Medicine Partnership Unit (MMPU), Heidelberg, Germany; [32]European Bioinformatics Institute (EMBL-EBI), Hinxton, United Kingdom; [33]German Cancer Research Center (DKFZ), Bridging Research Division on Mechanisms of Genomic Variation and Data Science, Heidelberg, Germany; [34]KU Leuven, Computational Cancer Biology and Epigenomics, Department of Oncology, Leuven, Belgium; [35]MVZ Haematologische Diagnostik, Heppenheim, Germany; [36]University Medical Center Ulm, Department of Pediatrics and Adolescent Medicine, Ulm, Germany; [37]Charité - Universitätsmedizin Berlin, Department of Hematology, Oncology, and Tumor Immunology, Berlin, Germany; [38]German Cancer Consortium (DKTK), Partner site Berlin, and German Cancer Research Center (DKFZ), Heidelberg, Germany; [39]Otto-von-Guericke-University Magdeburg, Faculty of Medicine, Magdeburg, Germany;*

**Copyright Information:** (Online) ISSN: 2572-9241
©2026 The Author(s). HemaSphere is published by John Wiley & Sons Ltd on behalf of European Hematology Association. This is an open access Abstract Book distributed under the Attribution-NonCommercial-NoDerivs (CC BY-NC-ND), which allows third parties to download the articles and share them with others as long as they credit the author and the Abstract Book, but they cannot change the content in any way or use them commercially.
**Abstract Book Citations:** Authors, Title, HemaSphere, 2026;10:(S1):pages. The abstract book DOIs can be found on the "Author Information" page.
**Disclaimer:** Articles published in the journal HemaSphere exclusively reflect the opinions of the authors. The authors are responsible for all content in their abstracts including accuracy of the facts, statements, citing resources, etc.

### Background

Diagnostic workup of hematopoietic neoplasms involves several assays with varying turnaround times to identify disease subtypes and select appropriate treatment regimens. DNA methylation-based classifiers have been published as novel robust diagnostic tools for the classification of different leukemia entities. However, currently established classifiers are disease entity specific and thus require prior disease entity diagnostics.

### Aims

We aimed to overcome the limitations of current diagnostic approaches and established *nanoLeukemia*, a hierarchical framework that combines epigenetic and genetic information. This framework leverages whole genome nanopore sequencing (WGNS) data to simultaneously assess i) hematopoietic neoplasm entities, ii) epigenetic disease subgroups (epitypes) within specified entities and iii) genetic aberrations within 3-4 days.

### Methods

We collected publicly available and in-house generated DNA methylation array data of 5,319 hematopoietic neoplasms and healthy controls followed by training and validation of an XGBoost model for prediction of hematopoietic disease entities. Previously established DNA methylation epitype definitions were adopted to further classify CLL, JMML and acute leukemias (AL). Applicability of these classifiers to WGNS data was confirmed using a technical control dataset followed by benchmarking against standard-of-care (SOC) diagnostics for AML.

### Results

The initial step of the *nanoLeukemia* framework relies on a DNA methylation classifier for prediction of hematopoietic disease entities from blood or bone marrow samples. This classifier allows entity-level prediction using stringent accuracy cutoffs and currently comprises 20 classes, covering healthy blood/bone marrow, as well as acute and chronic myeloid and lymphoid neoplasms. In a second step, we apply entity-specific classifiers for epitype identification in CLL, JMML, and AL covering a total of 44 prognostically or therapeutically relevant epitypes (accuracy range: 0.94-1.0). These classifiers were validated in independent cohorts comprising 424 CLL, 116 JMML and 477 AML patients. Technical controls (JMML, AML and healthy) confirmed cross-platform functionality of our classifiers between DNA methylation array and WGNS. In a third step, genetic data (copy number variations, structural variants and mutations) obtained from WGNS were integrated to identify genomic aberrations of clinical relevance. This hierarchical classification strategy was validated against SOC in a cohort of 50 AML samples. WGNS using one flow cell per patient achieved a median genome coverage of 34x (range:12-53) and demonstrated diagnostic performance comparable to SOC with identification of additional prognostically or therapeutically relevant aberrations.

### Summary/Conclusion

In the present study, we established a hierarchical framework that predicts hematopoietic neoplasm entities from DNA methylation data and subsequently uses appropriate disease-specific classifiers for epitype identification. WGNS enables to combine these classifiers with genomic data for rapid integrated molecular analysis of hematopoietic neoplasms in a single assay. Following appropriate prospective validation, this approach has the potential to fundamentally change molecular diagnostics in hematology.

**Copyright Information:** (Online) ISSN: 2572-9241
©2026 The Author(s). HemaSphere is published by John Wiley & Sons Ltd on behalf of European Hematology Association. This is an open access Abstract Book distributed under the Attribution-NonCommercial-NoDerivs (CC BY-NC-ND), which allows third parties to download the articles and share them with others as long as they credit the author and the Abstract Book, but they cannot change the content in any way or use them commercially.
**Abstract Book Citations:** Authors, Title, HemaSphere, 2026;10:(S1):pages. The abstract book DOIs can be found on the "Author Information" page.
**Disclaimer:** Articles published in the journal HemaSphere exclusively reflect the opinions of the authors. The authors are responsible for all content in their abstracts including accuracy of the facts, statements, citing resources, etc.

### (S334) A BIMODAL FRAMEWORK FOR RELIABLE AND ACCESSIBLE ACUTE LEUKEMIA MACHINE LEARNING CLASSIFICATION USING GENE EXPRESSION AND DNA METHYLATION DATA

**Topic:** 38. Novel technologies, techniques and digital analytical tools in hematology

Mariya Lysenkova Wiklander*[1, 2], Dave Zachariah[3], Emma Dizdarevic[1, 2], Olga Krali[1, 2], Linda Holmfeldt[4], Nina Hollfelder[2, 4], Ola Wallerman[2, 4], Tatjana Pandzic[4], Vasilios Zachariadis[5, 6], Ingegerd Öfverholm[7], Kajsa Paulsson[8], Gisela Barbany[7], Josefine Palle[9], Lucia Cavelier Franco[7], Jessica Nordlund[1, 2]

*[1]Department of Medical Sciences, Uppsala University, Uppsala, Sweden;[2]SciLifeLab, Uppsala University, Uppsala, Sweden; [3]Department of Information Technology, Uppsala University, Uppsala, Sweden;[4]Department of Immunology, Genetics, and Pathology, Uppsala University, Uppsala, Sweden; [5]Department of Oncology-Pathology, Karolinska Institutet, Stockholm, Sweden; [6]Paediatric Oncology, Astrid Lindgren Children's Hospital, Karolinska University Hospital, Stockholm, Sweden;[7]Department of Molecular Medicine and Surgery, Karolinska Institutet, Stockholm, Sweden; [8]Division of Clinical Genetics, Department of Laboratory Medicine, Lund University, Lund, Sweden; [9]Department of Woman's and Children's Health, Uppsala University, Uppsala, Sweden;*

### Background

In recent years, an abundance of machine learning (ML) models have been developed to predict molecular subtypes of various cancers, including leukemias. These models, while often showing impressive accuracy, suffer from several shortcomings, including opacity, a lack of uncertainty quantification and statistical guarantees, as well as a paucity of infrastructure for their translation to clinical use. In addition, these models are usually trained using a single data modality.

### Aims

Here, we present MLOmix, an end-to-end framework for training and deploying explainable machine learning models developed using RNA-sequencing gene expression (GEX) and array-based DNA methylation (DNAm) data. Further, we use this framework to implement PALLAS (Pan Acute Leukemia Learning and Subtyping), a multi-modal classifier used to predict subtypes of pediatric acute lymphoblastic leukemia (ALL) and acute myeloid leukemia (AML).

### Methods

The MLOmix framework includes two Nextflow pipelines, gexML and methylML, for the preprocessing and standardization of omics data; a ML training module; a conformal prediction layer to guarantee outputs at a user-specified error rate; and a web interface allowing users without technical expertise to perform inference on their datasets. To develop PALLAS, we applied the random forest algorithm to a generous dataset of over 8000 samples, including 5537 RNA-seq and 2479 DNAm samples.

### Results

From the overall dataset, 4885 labeled samples were used for model training and validation, including n=955 samples representing 11 AML subtypes, and n=3930 samples representing 19 ALL subtypes. 70% of this dataset was used for training, 15% for calibrating the conformal predictor, and 15% for testing. The resulting models achieve a weighted F1 score of up to 0.94, with class-specific F1 scores ranging from 0.67 for the classes AML-*PML::RARA* and ALL-*MEF2D*-r, both underrepresented in the DNAm data, and up to 0.99 for T-ALL in both data modalities.

**Copyright Information:** (Online) ISSN: 2572-9241
©2026 The Author(s). HemaSphere is published by John Wiley & Sons Ltd on behalf of European Hematology Association. This is an open access Abstract Book distributed under the Attribution-NonCommercial-NoDerivs (CC BY-NC-ND), which allows third parties to download the articles and share them with others as long as they credit the author and the Abstract Book, but they cannot change the content in any way or use them commercially.
**Abstract Book Citations:** Authors, Title, HemaSphere, 2026;10:(S1):pages. The abstract book DOIs can be found on the “Author Information” page.
**Disclaimer:** Articles published in the journal HemaSphere exclusively reflect the opinions of the authors. The authors are responsible for all content in their abstracts including accuracy of the facts, statements, citing resources, etc.

### Summary/Conclusion

We present PALLAS, a unique classifier that enables the prediction of leukemia subtypes using GEX and DNAm data, expanding the potential utility of -omics data in settings where one or both data modalities are generated. Further, we open-source our ML framework MLOmix, allowing the re-use of these software components for the training of future multimodal classifiers for hematology and beyond.

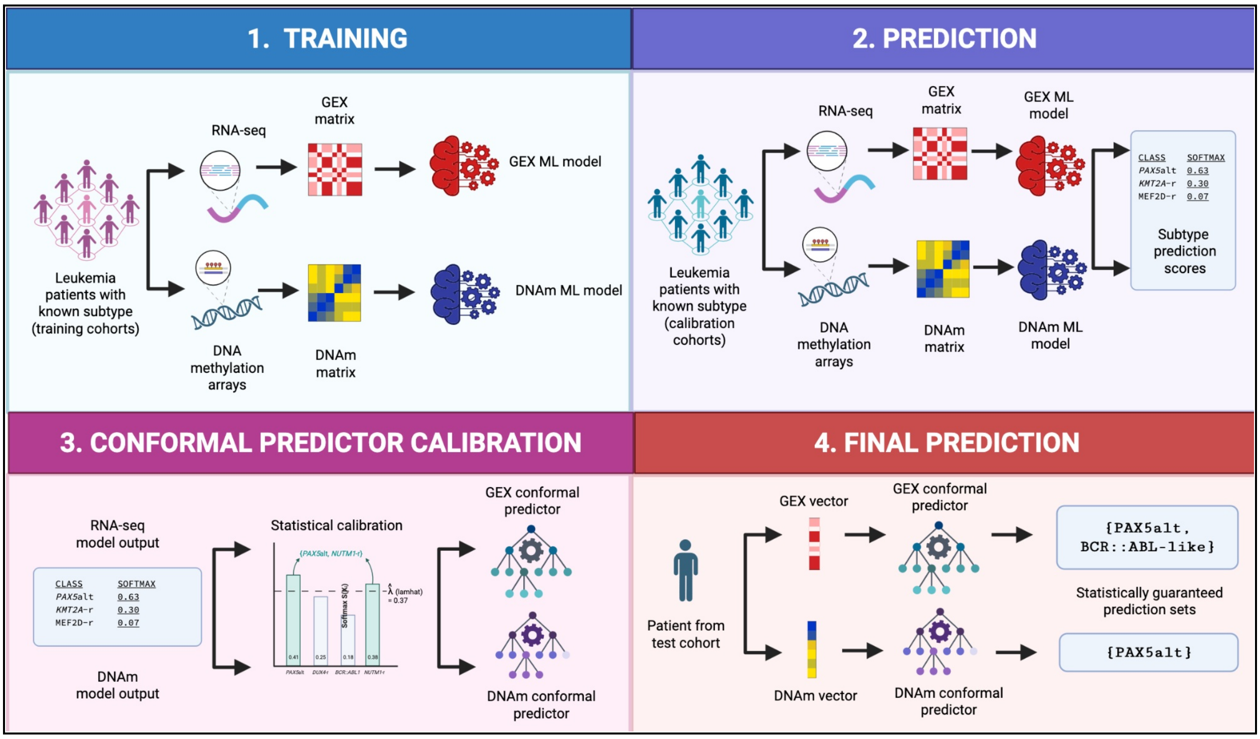

**Copyright Information:** (Online) ISSN: 2572-9241
©2026 The Author(s). HemaSphere is published by John Wiley & Sons Ltd on behalf of European Hematology Association. This is an open access Abstract Book distributed under the Attribution-NonCommercial-NoDerivs (CC BY-NC-ND), which allows third parties to download the articles and share them with others as long as they credit the author and the Abstract Book, but they cannot change the content in any way or use them commercially.
**Abstract Book Citations:** Authors, Title, HemaSphere, 2026;10:(S1):pages. The abstract book DOIs can be found on the "Author Information" page.
**Disclaimer:** Articles published in the journal HemaSphere exclusively reflect the opinions of the authors. The authors are responsible for all content in their abstracts including accuracy of the facts, statements, citing resources, etc.
